# The Search for the Optimal Methodology for Predicting Fluorinated Cathinone Drugs NMR Chemical Shifts

**DOI:** 10.3390/molecules30010054

**Published:** 2024-12-27

**Authors:** Natalina Makieieva, Teobald Kupka, Oimahmad Rahmonov

**Affiliations:** 1 Faculty of Chemistry and Pharmacy, University of Opole, 48, Oleska Str., 45-052 Opole, Poland; 2 Institute of Earth Sciences, Faculty of Natural Sciences, University of Silesia in Katowice, 60, Będzińska, 41-200 Sosnowiec, Poland; oimahmad.rahmonov@us.edu.pl

**Keywords:** cathinone, fluorinated cathinone derivatives, DFT, GIAO NMR

## Abstract

Cathinone and its synthetic derivatives belong to organic compounds with narcotic properties. Their structural diversity and massive illegal use create the need to develop new analytical methods for their identification in different matrices. NMR spectroscopy is one of the most versatile methods for identifying the structure of organic substances. However, its use could sometimes be very difficult and time-consuming due to the complexity of NMR spectra, as well as the technical limitations of measurements. In such cases, molecular modeling serves as a good supporting technique for interpreting ambiguous spectral data. Theoretical prediction of NMR spectra includes calculation of nuclear magnetic shieldings and sometimes also indirect spin–spin coupling constants (SSCC). The quality of theoretical prediction is strongly dependent on the choice of the theory level. In the current study, cathinone and its 12 fluorinated derivatives were selected for gauge-including atomic orbital (GIAO) NMR calculations using Hartree–Fock (HF) and 28 density functionals combined with 6-311++G** basis set to find the optimal level of theory for ^1^H, ^13^C, and ^19^F chemical shifts modeling. All calculations were performed in the gas phase, and solutions were modeled with a polarized-continuum model (PCM) and solvation model based on density (SMD). The results were critically compared with available experimental data.

## 1. Introduction

Cathinone has been known for centuries to be an abused drug originating from hot-climate plant material. This compound occurs naturally as a metabolite of the *Catha edulis* (Khat) plant and is very popular in North Africa and West Asia countries, such as Ethiopia, the Republic of Somaliland, Kuwait, and Qatar. Although, previously, this metabolite was used in ritual practices and the initiation of socio-political contacts, today, it is increasingly used as a recreational drug [1]. Cathinone and its synthetic derivatives are one of the most popular psychotropic substances in the world [1,2]. This is due to their relatively low cost, ease of synthesis, and isolation from plant material [3,4,5]. Depending on its structural modification, the psychotropic effect could be amphetamine- or cocaine-type [6,7]. However, it is worth noting that this group of drugs has a pharmaceutical potential. For example, bupropion is a registered antidepressant and a smoking-cessation drug [8,9]. The cytostatic potential of cathinone and its derivatives studied on cell lines of some cancer forms is promising [10,11,12,13]. However, the lack of detailed studies of their toxic effects and psychotropic properties significantly limits the research of cathinones in the field of new drug discovery [14,15]. New representatives of cathinones are constantly appearing in the world register of narcotic substances. However, the production of cathinone synthetic derivatives is so massive and rapidly developing that regulatory schedules are updated with a certain delay [16,17]. For this reason, it is difficult to accurately determine how extensive this drug group is today. It is even more difficult to accurately and quickly identify new cathinone synthetic derivatives, as well as known substances in complex biological matrices (e.g., in blood, urine, or hair) [18,19]. For this reason, structural and spectroscopic data of both known and new cathinone derivatives, as well as improved analytical protocols for medical and forensic science, are constantly appearing in the scientific literature [5,17,20,21,22,23]. High-performance liquid chromatography–mass spectrometry (HPLC-MS), gas chromatography–mass spectrometry (GC-MS), mass spectrometry–electron ionization (MS-EI), and Fourier-transform infrared spectroscopy (FTIR) are most often described as suitable instrumental tools for routine analysis of this class of drugs [5,17,24,25]. NMR spectroscopy is also used for cathinone identification but is rarely mentioned as a suitable tool for this narcotics group characterization [5,25,26]. This is due to the relatively low sensitivity of this analytical method, although it certainly allows for a comprehensive description of both the structure of the analyzed organic substance and studies of its interactions with the environment. Another limitation in the use of nuclear magnetic resonance spectroscopy is the structural diversity of cathinones. Due to the easy-to-perform modifications at both single and multiple positions of the benzene ring, alpha carbon, and amine group, cathinone could be transformed into larger structures [5]. This leads to complex NMR spectra that could be very difficult to interpret. In addition, due to the moderate psychotropic potential of some cathinones [4], they are often used in the form of mixtures with other derivatives and stronger drugs. This further complicates the interpretation of their NMR spectra. For this reason, NMR characterization of cathinone and its derivatives, along with improving sample preparation and measurement conditions, continues [26,27,28,29]. Molecular modeling could be used as a good tool to support analyses of cathinone and its synthetic derivatives’ experimental data. A good theoretical methodology, which could be developed with relatively small financial and time investments, could allow for a fairly quick and accurate determination of a number of structural and spectroscopic properties of the analyzed substances. Relatively few theoretical works that focus on predicting various parameters of cathinone and its derivatives are available in the literature. Due to the psychotropic and cytotoxic properties of cathinones, authors have primarily focused on simulating conformational transitions and their impact on the strength and nature of interactions with different molecular targets [30,31,32,33,34]. Another direction was the search for potential sensors and sorbents for screening and removal of these drugs from the human body and other matrices (e.g., from powders, tablets, and the environment) [35,36,37,38,39,40]. Attention was also paid to the structural and spectroscopic parameters of cathinone and its synthetic derivatives [30,41,42,43,44]. In particular, modeling of IR and NMR spectra and determining the most accurate methodology for this parameter prediction could be found. However, in the case of NMR, a small number of theoretical methods and solvent effects were considered. It was also noted that the level of theory was fairly simplified. For this reason, the question of the performance of theoretical literature protocols for predicting cathinone chemical shifts and indirect spin–spin coupling constants remains open.

**Figure 1:**
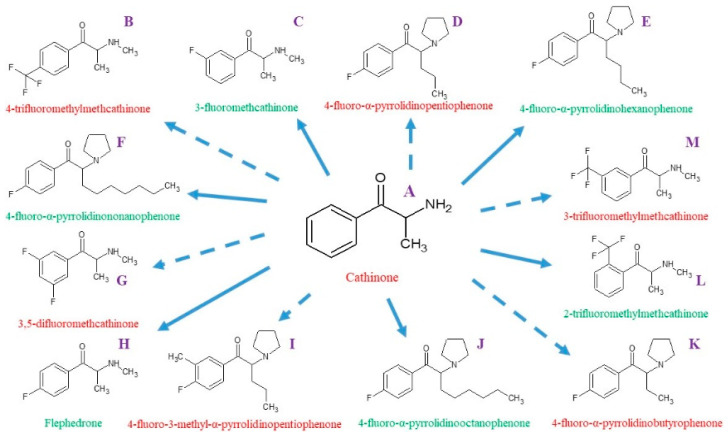
Structure of cathinone and its fluorinated derivatives modeled in the current study. Compounds are labeled by capital letters (**A**–**M**).

This work aims to search for an optimal methodology for predicting ^1^H, ^13^C, and ^19^F NMR parameters since these spectra are the most often measured for both cathinones and other organic compounds. To achieve this goal, theoretical prediction of chemical shifts was performed for cathinone and its 12 synthetic derivatives fluorinated in different positions (see Figure 1). The performance of the Hartree–Fock method and a number of density functionals in the gas phase and solvents with increasing polarity were analyzed. The obtained parameters were critically compared with available experimental data.

## 2. Results and Discussion

**Figure 2:**
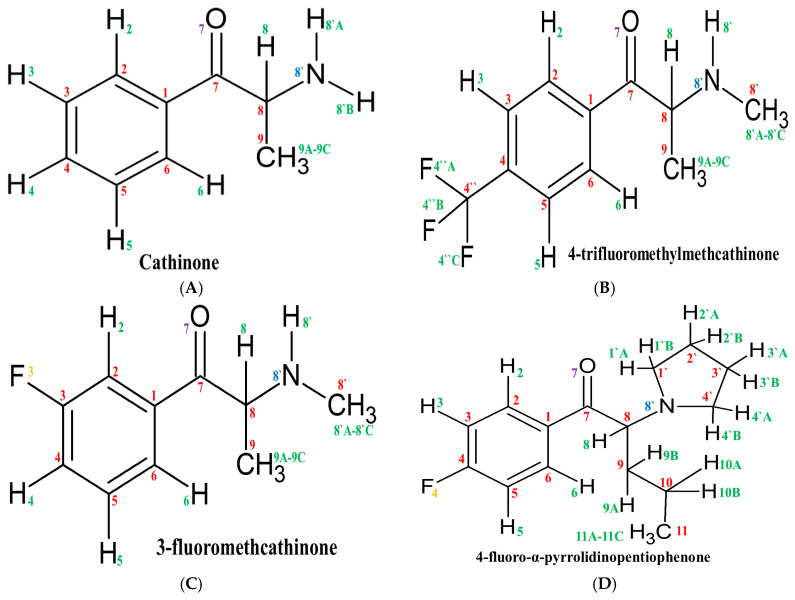

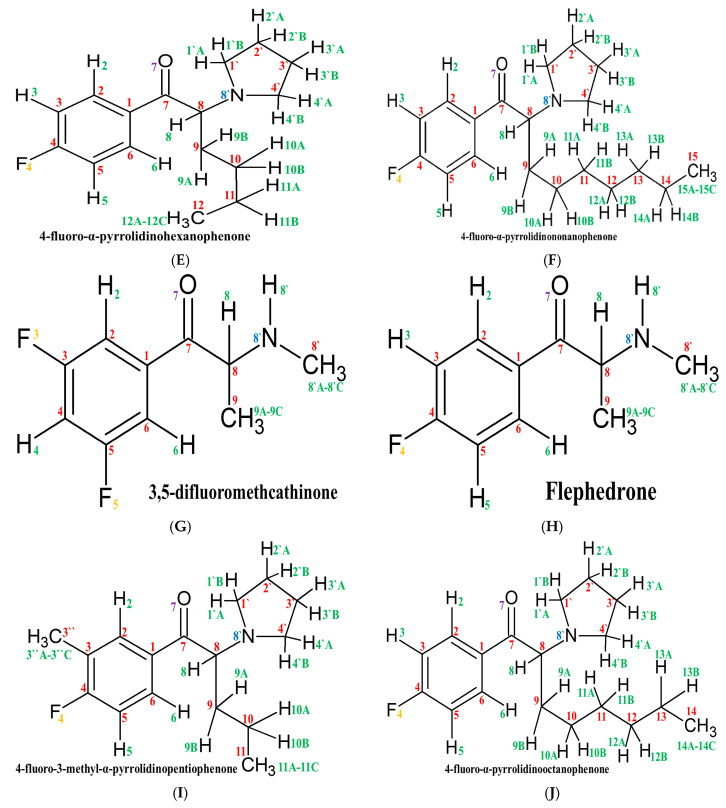

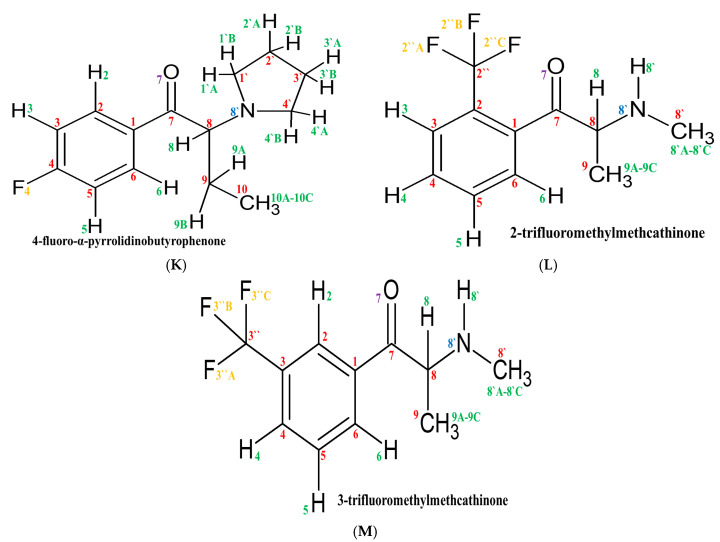
Cathinone and its fluorinated derivatives (**A**–**M**) with atoms numbered. Hydrogen is marked in green, carbon—in red, nitrogen—in blue, oxygen—in violet, and fluorine—in orange.

As a result of systematic theoretical calculations, a series of NMR data were obtained. To simplify their analysis, the atoms included in the structures of the studied drugs were numbered separately for each element. The numbering for cathinone and each fluorinated derivative is presented in Figure 2.

In order to avoid overloading the main text with data, the theoretical values of chemical shifts of cathinone and all its fluorinated derivatives have been moved to the Supplementary Material (see Tables S1–S17). The chemical shifts in the order of ^13^C, ^1^H, and ^19^F are tabularized separately for each analyzed drug and are presented in the alphabetical order of the used method of calculation (B3LYP, B3P86, B3PW91, B98, B971, B972, BHandH, BHandHLYP, BLYP, BMK, BP86, CAM-B3LYP, LC-BLYP, M06, M06-2X, mpW1LYP, mpW1PW91, O3LYP, OLYP, OPBE, OPW91, PBE, PBE0, TPSS, VSXC, wB97, wB97X, and X3LYP). For analysis of the influence of the configuration for each fluorinated molecule, the data for the *R/S*-configurations are presented. In order to consider the performance of the selected method, the results of calculations in vacuum are presented at the beginning of each table (see Tables S1–S17). Further, to consider the influence of the polarity of the environment on the accuracy of the obtained results, the data appear in the order of increasing solvent polarity (from chloroform to water). Both PCM [45] and SMD [46] models are used to mimic the solvent effect. To determine the total effect of the method used and the environment on the accuracy of the results, the ^1^H, ^13^C, and ^19^F chemical shift values were separately compared with available literature data (shown in bold in Tables S1–S17). Since some of the cathinones atoms have specific chemical neighborhoods, significant fluctuations between their experimental chemical shifts and, consequently, significantly different deviations of theoretical values could be expected. For this reason, the RMS value was chosen as the main statistical parameter to determine the accuracy of the analyzed methods. It allows averaging a significant divergence of errors and, based on its smallest value, determines which of the method performs the best.

For a clear presentation of the theoretical chemical shifts, only selected RMS values for all analyzed nuclei are presented in the text. However, a methods performance in predicting hydrogen-1, carbon-13, and fluorine-19 shifts and their corresponding trends will be presented in subsequent parts of the text.

### 2.1. Performance of HF and 28 Selected Density Functionals in ^1^H, ^13^C, and ^19^F Chemical Shifts Prediction

Analyzing the accuracy of results obtained with the HF approximation and 28 selected density functionals, it is worth noting their different performance for each element (see Table 1, Table 2 and Table 3). As can be seen, the absolute values of deviations vary significantly between chemical elements. This can be explained by considering the width of the measured spectral window for each nucleus. For example, ^1^H has the narrowest spectral range of chemical shifts (about 0–14 ppm). Therefore, a 5% deviation of a proton signal amounts to 0.7 ppm. In analogy, for carbon spectra (typical spectral window up to 200 ppm), the same deviation is 10 ppm. Therefore, in both cases, the chemical shift percentage error is the same, but its absolute value will differ. For this reason, the accuracy of each method in the current work was considered separately for the chemical shift of each element. It is also worth mentioning that in the case of magnetically equivalent atoms (some -CH_2_- groups) and atoms from rapidly rotating groups (-CH_3_ and -CF_3_), the theoretical values of chemical shifts were averaged.

To analyze in detail the performance of different methods for predicting ^1^H chemical shifts, it is worth paying attention to Table 1A,B, as well as Tables S1–S17 in the Supporting Information. It can be seen that the RMS values varied for the analyzed cathinones (see Table 1A,B), but these differences were not significant. For example, in the case of B3LYP, the RMS values in the gas phase (see Table 1A,B) varied from 0.241 to 0.556 ppm. This can be partly explained due to the discrepancies in the experimental data. As can be seen, some publications present low-resolution spectra and partly overlapped signals [25,47,48,49]. As a consequence, it was difficult to accurately assign some peak positions. For this reason, the theoretical shifts for the partly overlapped multiplets were averaged. It is also worth noting that the RMS values were different for the *R/S*-configurations of many fluorinated cathinones (see Table 1A,B). This can be explained by the energy preference of one configuration in the solution, and as a result, there is a higher similarity between the theoretical results and the experimental values. Another reason for the variations in the RMS values of each method was the different accuracy in predicting proton shifts in different chemical environments. These deviations will be described in more detail in Section 2.2.

Analyzing the synergistic effect of the method and solvent on the accuracy of proton shift prediction, it is worth paying attention to Table 1A,B and Tables S1–S17. In the Supporting Information, the methods that predicted ^1^H shifts with the highest accuracy, as well as their lowest RMS values, are marked in green. Similarly, the worst methods for predicting ^1^H shifts and their highest values for each drug are marked in orange. It is worth noting that the best methods were selected independently for each analyzed substance based on two parameters: the RMS values for all analyzed solvents were below the limiting value for a given substance, and the deviation of only one RMS value from the limit (this value was taken as a random superposition of errors of a given method). This approach was chosen due to the previously mentioned variations in experimental data and different accuracy of prediction for some atoms. As can be seen, the set of best methods varied for different cathinones. However, the most frequently best-performed density functionals were BLYP, BP86, PBE, and OLYP. Analyzing the influence of the environment on the accuracy of theoretical proton shifts, it is worth noting the absence of a direct correlation with solvent polarity. Although for many cathinones, the lowest RMS values obtained by different methods were observed in water using the PCM or SMD model (see Table S18), a number of the lowest values were noted for the most accurate methods in less polar environments. No direct influence of the choice of PCM or SMD model on the accuracy of the result was also observed. However, it is important to mention that the spread of RMS values for different solvents was small.

**Table 1:** (**A**) RMS values of ^1^H chemical shifts (in ppm) calculated in the gas phase with the studied methods for *S*-cathinone and *S*-configurations of its fluorinated derivatives. (**B**) RMS values of ^1^H chemical shifts (in ppm) calculated in the gas phase with the studied methods for *R*-configurations of cathinone fluorinated derivatives. RMS values of the best-performed methods are in bold, while RMS values of the worst method are in underlined italics.

**(A)**																	
**Method**	**Studied Compounds**																
	**A ^1^**	**B ^2^**	**C ^3^**	**D ^4^**	**E ^5^**	**E ^6^**	**F ^7^**	**G ^8^**	**H ^9^**	**H ^10^**	**H ^11^**	**I ^12^**	**J ^13^**	**K ^14^**	**K ^15^**	**L ^16^**	**M ^17^**
B3LYP	0.438	0.321	0.313	0.268	0.47	0.427	0.443	0.498	0.347	0.392	0.346	0.556	0.537	0.528	0.500	0.330	0.306
B3P86	0.457	0.304	0.302	0.268	0.476	0.431	0.447	0.477	0.333	0.381	0.337	0.565	0.544	0.536	0.508	0.300	0.282
B3PW91	0.456	0.309	0.294	0.276	0.484	0.439	0.458	0.483	0.337	0.385	0.336	0.567	0.550	0.544	0.517	0.321	0.287
B98	0.451	0.320	0.304	0.276	0.481	0.470	0.452	0.496	0.345	0.392	0.344	0.568	0.553	0.543	0.517	0.338	0.301
B971	0.452	0.317	0.301	0.276	0.482	0.438	0.452	0.495	0.343	0.390	0.341	0.569	0.554	0.545	0.518	0.337	0.300
B972	0.459	0.319	0.297	0.282	0.490	0.446	0.463	0.494	0.348	0.396	0.344	0.571	0.556	0.551	0.524	0.347	0.299
BHandH	0.587	0.417	0.433	0.370	0.558	0.521	0.533	0.497	0.44	0.503	0.463	0.649	0.654	0.641	0.616	0.354	0.379
BHandHLYP	0.561	0.429	0.429	0.396	0.584	0.545	0.567	0.535	0.456	0.515	0.462	0.660	0.676	0.662	0.639	0.437	0.410
BLYP	0.350	**0.285**	**0.261**	**0.229**	**0.406**	**0.362**	**0.361**	**0.515**	**0.477**	**0.525**	**0.474**	**0.502**	**0.448**	**0.449**	**0.417**	**0.295**	**0.275**
BMK	0.680	0.473	0.472	0.401	0.562	0.568	0.564	0.456	0.482	0.555	0.515	0.643	0.673	0.660	0.633	0.390	0.421
BP86	0.355	**0.250**	**0.232**	**0.220**	**0.408**	**0.363**	**0.359**	**0.502**	**0.282**	**0.302**	**0.272**	**0.512**	**0.451**	**0.451**	**0.421**	**0.255**	**0.232**
CAM-B3LYP	0.505	0.370	0.374	0.322	0.513	0.474	0.494	0.501	0.394	0.451	0.403	0.597	0.595	0.583	0.558	0.372	0.350
HF	0.675	0.594	0.566	0.611	*0.784*	*0.748*	*0.77*	*0.682*	0.632	0.691	0.624	*0.855*	*0.891*	*0.882*	*0.87*	*0.662*	0.602
LC-BLYP	0.605	0.451	0.467	0.400	0.569	0.539	0.561	0.506	0.472	0.539	0.494	0.651	0.668	0.654	0.633	0.418	0.419
M06	0.468	0.338	0.295	0.307	0.528	0.487	0.501	0.544	0.373	0.423	0.374	0.618	0.635	0.577	0.565	0.367	0.355
M06-2X	*0.842*	0.608	*0.606*	*0.490*	0.490	0.595	0.612	0.468	*0.588*	*0.666*	*0.636*	0.684	0.733	0.725	0.698	0.450	*0.541*
mpW1LYP	0.460	0.340	0.337	0.285	0.484	0.442	0.457	0.501	0.366	0.413	0.367	0.570	0.558	0.547	0.519	0.349	0.325
mpW1PW91	0.486	0.327	0.318	0.295	0.500	0.457	0.475	0.478	0.354	0.407	0.358	0.584	0.573	0.566	0.540	0.334	0.303
O3LYP	**0.418**	0.302	0.266	0.260	0.447	0.403	0.420	0.495	0.328	0.369	0.320	**0.527**	0.504	0.510	0.474	0.329	0.281
OLYP	**0.361**	**0.292**	**0.219**	0.251	**0.411**	**0.367**	**0.375**	0.511	**0.445**	**0.495**	**0.432**	**0.490**	**0.458**	**0.470**	**0.429**	0.307	**0.496**
OPBE	**0.372**	0.275	**0.174**	0.249	0.430	0.383	0.395	0.508	**0.281**	**0.309**	**0.255**	**0.503**	**0.477**	**0.489**	**0.450**	0.305	**0.252**
OPW91	**0.373**	0.275	**0.178**	0.249	0.429	0.383	0.396	0.507	**0.282**	**0.311**	**0.257**	**0.502**	**0.476**	**0.489**	**0.449**	0.305	**0.253**
PBE	0.367	**0.244**	**0.233**	**0.217**	**0.405**	**0.362**	**0.358**	**0.493**	**0.278**	**0.302**	**0.273**	**0.510**	**0.450**	**0.454**	**0.424**	**0.252**	**0.222**
PBE0	0.485	0.325	0.316	0.293	0.500	0.457	0.473	0.481	0.352	0.405	0.357	0.585	0.572	0.566	0.540	0.332	0.301
TPSS	0.393	0.300	**0.259**	**0.247**	0.456	0.405	0.408	**0.530**	0.327	**0.327**	**0.306**	0.548	0.512	**0.506**	0.478	**0.308**	**0.289**
VSXC	0.409	0.304	0.276	0.326	0.543	0.518	0.440	0.519	0.322	0.357	0.329	0.682	0.628	**0.589**	0.578	**0.213**	0.275
wB97	0.561	*0.408*	0.409	0.371	0.561	0.524	0.543	0.518	0.432	0.495	0.441	0.651	0.653	0.637	0.620	0.435	0.397
wB97X	0.541	0.391	0.387	0.349	0.545	0.507	0.525	0.512	0.415	0.476	0.422	0.630	0.629	0.618	0.598	0.412	0.374
X3LYP	0.447	0.326	0.321	0.272	0.474	0.431	0.446	0.498	0.353	0.398	0.353	0.560	0.543	0.533	0.505	0.333	0.310
**(B)**																	
**Method**	**Studied Compounds**																
	**B ^18^**		**C ^19^**	**D ^20^**	**E ^21^**	**E ^22^**	**F ^23^**	**G ^24^**	**H ^25^**	**H ^26^**	**H ^27^**	**I ^28^**	**J ^29^**	**K ^30^**	**K ^31^**	**L ^32^**	**M ^33^**
B3LYP	0.351		0.318	0.241	0.441	0.384	0.448	0.495	0.385	0.438	0.364	0.534	0.509	0.491	0.459	0.448	0.371
B3P86	0.353		0.333	0.243	0.450	0.393	0.453	0.487	0.392	0.449	0.380	0.547	0.517	0.501	0.473	0.423	0.367
B3PW91	0.359		0.330	0.250	0.457	0.400	0.463	0.497	0.397	0.452	0.380	0.547	0.523	0.509	0.479	0.439	0.377
B98	0.363		0.329	0.250	0.452	0.396	0.459	0.503	0.399	0.453	0.381	0.546	0.527	0.507	0.477	0.453	0.379
B971	0.364		0.330	0.249	0.453	0.396	0.459	0.505	0.399	0.454	0.381	0.547	0.527	0.509	0.478	0.454	0.380
B972	0.370		0.332	0.255	0.461	0.404	0.469	0.510	0.407	0.462	0.387	0.549	0.528	0.515	0.483	0.463	0.390
BHandH	0.494		0.490	0.356	0.541	0.496	0.541	0.555	0.524	0.591	0.531	0.641	0.633	0.616	0.594	0.513	0.491
BHandHLYP	0.492		0.472	0.378	0.561	0.512	0.573	0.576	0.525	0.588	0.517	0.644	0.656	0.636	0.608	0.573	0.505
BLYP	**0.276**		**0.207**	**0.196**	**0.372**	**0.312**	**0.365**	**0.477**	**0.297**	**0.328**	**0.253**	**0.477**	**0.410**	**0.401**	0.367	**0.383**	**0.302**
BMK	0.540		0.539	0.383	0.533	0.494	0.567	0.511	0.561	0.634	0.576	0.624	0.652	0.632	0.598	0.526	0.519
BP86	**0.253**		**0.211**	**0.188**	**0.377**	**0.317**	**0.364**	**0.471**	**0.294**	**0.331**	**0.262**	**0.491**	**0.414**	**0.405**	0.377	**0.344**	**0.279**
CAM-B3LYP	0.419		0.403	0.300	0.487	0.436	0.500	0.525	0.451	0.512	0.442	0.578	0.572	0.552	0.522	0.505	0.433
HF	0.669		0.630	0.598	*0.765*	*0.721*	*0.775*	*0.760*	0.713	0.772	0.695	*0.841*	*0.879*	*0.864*	*0.844*	*0.799*	0.705
LC-BLYP	0.514		0.514	0.384	0.547	0.508	0.566	0.562	0.544	0.613	0.550	0.636	0.650	0.631	0.603	0.563	0.517
M06	0.373		0.345	0.277	0.497	0.440	0.513	0.521	0.423	0.480	0.412	0.603	0.559	0.539	0.519	0.467	0.399
M06-2X	*0.668*		*0.669*	*0.473*	0.580	0.559	0.619	0.540	*0.678*	*0.757*	*0.711*	0.667	0.709	0.698	0.663	0.560	*0.648*
mpW1LYP	0.374		0.349	0.259	0.456	0.400	0.463	0.504	0.407	0.462	0.390	0.549	0.531	0.512	0.479	0.469	0.392
mpW1PW91	0.386		0.362	0.271	0.475	0.420	0.481	0.504	0.423	0.482	0.411	0.565	0.548	0.534	0.504	0.458	0.400
O3LYP	0.338		0.270	0.228	0.412	0.353	0.428	0.505	0.363	0.412	0.333	**0.503**	0.470	0.467	0.423	0.448	0.372
OLYP	**0.312**		**0.202**	0.217	**0.373**	**0.311**	**0.385**	0.508	**0.313**	**0.349**	**0.265**	**0.463**	**0.417**	**0.420**	0.373	0.414	**0.345**
OPBE	0.316		**0.214**	0.217	0.396	0.331	0.405	0.519	**0.331**	**0.369**	**0.287**	**0.478**	**0.438**	**0.441**	0.398	0.404	**0.348**
OPW91	0.316		**0.214**	0.217	0.395	0.331	0.405	0.517	**0.330**	**0.369**	**0.286**	**0.478**	**0.438**	**0.441**	0.398	0.404	**0.348**
PBE	**0.254**		**0.219**	**0.183**	**0.373**	**0.314**	**0.364**	**0.466**	**0.297**	**0.338**	**0.270**	**0.486**	**0.413**	**0.406**	0.377	**0.347**	**0.279**
PBE0	0.387		0.363	0.269	0.474	0.419	0.478	0.508	0.424	0.483	0.412	0.566	0.547	0.532	0.504	0.461	0.401
TPSS	**0.315**		**0.250**	**0.224**	0.434	0.372	0.413	**0.510**	0.345	**0.380**	**0.306**	0.532	0.485	**0.472**	0.448	**0.393**	**0.334**
VSXC	0.346		0.318	0.289	0.507	0.461	0.489	0.519	0.372	0.426	0.371	0.676	0.565	**0.498**	0.509	**0.224**	0.349
wB97	0.484		0.462	0.352	0.536	0.488	0.550	0.578	0.514	0.578	0.507	0.634	0.629	0.609	0.586	0.560	0.496
wB97X	0.458		0.436	0.328	0.519	0.470	0.532	0.561	0.488	0.551	0.479	0.611	0.606	0.589	0.564	0.536	0.471
X3LYP	0.359		0.332	0.245	0.445	0.389	0.452	0.497	0.392	0.447	0.374	0.539	0.515	0.497	0.465	0.453	0.378

^1^ Experimental data from [50]; ^2^ experimental data from [25]; ^3^ experimental data from [4]; ^4^ experimental data from [51]; ^5^ experimental data from [5]; ^6^ experimental data from [26]; ^7^ experimental data from [47]; ^8^ experimental data from [52]; ^9^ experimental data from [53]; ^10^ experimental data from [24]; ^11^ experimental data from [4]; ^12^ experimental data from [23]; ^13^ experimental data from [48]; ^14^ experimental data from [49] in CDCI_3_; ^15^ experimental data from [49] in D_2_O; ^16^ experimental data from [25]; ^17^ experimental data from [25]. ^18^ Experimental data from [25]; ^19^ experimental data from [4]; ^20^ experimental data from [51]; ^21^ experimental data from [5]; ^22^ experimental data from [26]; ^23^ experimental data from [47]; ^24^ experimental data from [52]; ^25^ experimental data from [53]; ^26^ experimental data from [24]; ^27^ experimental data from [4]; ^28^ experimental data from [23]; ^29^ experimental data from [48]; ^30^ experimental data from [49] in CDCI_3_; ^31^ experimental data from [49] in D_2_O; ^32^ experimental data from [25]; ^33^ experimental data from [25].

Considering the theoretical prediction of the carbon-13 chemical shift, it is worth paying attention to Table 2A,B, as well as Tables S1–S17 in the Supporting Information. Similarly to ^1^H shifts, the ^13^C RMS values differ between the cathinones (see Table 2A,B). For example, in the case of B3LYP in a vacuum (see Table 2A,B), this parameter ranged from 4.764 to 8.523 ppm. However, as mentioned earlier, the carbon-13 nucleus has a fairly wide spectral window (up to 200 ppm). As can be seen, the fluctuation of the RSM in this case is about 4 ppm, which is a fairly low value of the deviation for the ^13^C spectral window (about 2%). It is worth mentioning that the differences between the RMS values for *R/S*-configurations were also small (within 1 ppm; see Table 2A,B). The fluctuations in the ^13^C RMS values for different cathinones are caused primarily by the precision of the chemical shift prediction for atoms with different chemical environments. This effect will be described in Section 2.2 in more detail. Table S19 is systematized similarly, as for the ^1^H spectrum (see Table S18), a performance of analyzed methods in different solvents. It could be observed that the set of the best-performed ^13^C chemical shift prediction methods also varied for different cathinones. The best-performed functionals are OPBE, OPW91, and OLYP. Analyzing the influence of the method on the accuracy of the ^13^C shifts prediction, an HF approximation performance should be mentioned separately. Although this method demonstrated one of the worst predictions of the ^1^H spectra (see Table S18), in the case of carbon-13, it provided theoretical values with medium accuracy, and for one of the analyzed substances, it was among the best-performed (see Table S19). This confirms previously noticed for other models [54,55] reasonable performance of HF can predict different chemical shifts. However, it is worth using the HF method with caution and as a “quick source”, which allows only rough prediction of ^13^C signals in the different chemical environments. For a more accurate analysis, it is recommended to use HF, which is verified by more accurate density functionals. Considering the effect of the solvent on the accuracy of the results, it is worth noting that in the majority of cases, the smallest values of ^13^C RMS were obtained in the gas phase. Only a few results departed from the trend and were obtained in chloroform using PCM and SMD models (see Table S19). However, it is worth noting that the deviations were slightly different from the data in a vacuum. For this reason, finding a minimum of RMS in a solvent that was closest in polarity to the gas phase could be due to accidental error cancelation. The increase in the solvent polarity led to a decrease in the accuracy of theoretical ^13^C shifts. However, the unambiguous influence of polarity on method performance has not been noticed. It is also worth noting that modeling solvents with different polarities using the PCM model theoretically resulted in higher accuracy (see Table S19). Furthermore, it is worth mentioning that in experimental NMR measurements, solvent had an insignificant effect on the ^13^C shift values. For example, in the work [49], the biggest difference between the signals in the D_2_O and CDCl_3_ was about 6 ppm, and the average value was about 1.6 ppm. Based on the obtained results, it is recommended to optimize the structure of cathinones with subsequent ^13^C shift prediction in the gas phase. This approach will significantly reduce calculation time and provide carbon-13 spectra with the highest accuracy for the used method.

**Table 2:** (**A**) RMS values of ^13^C chemical shifts (in ppm) calculated in the gas phase with the studied methods for *S*-cathinone and *S*-configurations of its fluorinated derivatives. (**B**) RMS values of ^13^C chemical shifts (in ppm) calculated in the gas phase with the studied methods for *R*-configurations of cathinone fluorinated derivatives. RMS values of the best-performed methods are in bold, while RMS values of the worst method are in underlined italics.

**(A)**																
**Method**	**Studied Compounds**															
	**A ^1^**	**B ^2^**	**C ^3^**	**D ^4^**	**E ^5^**	**E ^6^**	**G ^7^**	**H ^8^**	**H ^9^**	**H ^10^**	**I ^11^**	**J ^12^**	**K ^13^**	**K ^14^**	**L ^15^**	**M ^16^**
B3LYP	7.758	7.408	6.984	5.228	4.939	5.083	7.737	6.774	5.889	6.662	6.876	6.874	5.505	5.103	8.348	7.218
B3P86	6.527	6.187	5.612	4.188	3.955	4.084	6.162	5.405	4.564	5.277	5.788	5.652	4.288	4.164	6.908	5.827
B3PW91	6.569	6.241	5.675	4.187	3.977	4.103	6.221	5.465	4.622	5.338	5.861	5.626	4.324	4.217	6.929	5.933
B98	6.491	6.077	5.531	4.342	4.029	4.152	6.080	5.284	4.323	5.161	6.099	6.181	4.429	4.152	6.829	5.734
B971	6.334	6.036	5.373	4.217	3.927	4.046	5.931	5.114	4.170	4.991	5.965	6.072	4.274	4.027	6.747	5.663
B972	4.283	**5.047**	**3.577**	2.948	3.114	**3.139**	3.892	3.280	2.684	3.137	**4.886**	**4.591**	2.637	3.258	4.972	**4.194**
BHandH	8.939	7.280	7.652	6.086	5.892	5.987	7.612	7.752	6.923	7.617	6.708	6.426	6.214	6.366	8.594	7.353
BHandHLYP	7.686	6.551	6.869	5.209	5.106	5.200	7.130	6.886	6.009	6.755	6.510	5.947	5.351	5.524	7.564	6.445
BLYP	7.787	9.372	7.228	5.908	5.513	5.671	8.297	6.770	5.874	6.729	7.623	8.042	6.217	5.435	10.059	9.143
BMK	20.917	*17.445*	*18.725*	*14.715*	13.846	*14.027*	*18.833*	*18.641*	*17.569*	*18.534*	*15.758*	*14.929*	*15.471*	*14.693*	*19.388*	*18.008*
BP86	6.182	7.584	5.294	4.337	3.961	4.108	6.107	4.823	4.075	4.722	6.055	6.441	4.430	3.884	7.913	7.138
CAM-B3LYP	9.298	8.132	8.410	6.187	5.881	6.025	9.013	8.327	7.425	8.210	7.492	7.182	6.564	6.274	9.311	8.152
HF	6.484	7.753	6.078	5.898	6.104	6.106	5.590	6.538	5.902	6.402	6.564	**5.191**	5.668	6.660	8.158	7.657
LC-BLYP	13.471	11.254	12.255	9.334	8.894	9.047	12.614	12.334	11.430	12.218	10.015	9.332	9.906	9.627	12.770	11.630
M06	6.888	5.917	5.984	4.728	4.419	4.508	6.346	6.077	5.330	5.929	5.829	5.658	4.731	4.743	6.703	5.566
M06-2X	*20.739*	16.802	18.112	14.073	*14.073*	13.471	17.914	18.165	17.145	18.054	14.876	13.850	14.933	14.308	18.884	17.448
mpW1LYP	7.804	7.190	7.004	5.242	5.006	5.138	7.706	6.828	5.937	6.714	6.834	6.877	5.487	5.160	8.193	7.007
mpW1PW91	6.366	5.844	5.447	4.058	3.928	4.032	5.890	5.283	4.453	5.151	5.647	5.364	4.118	4.186	6.532	5.462
O3LYP	**3.783**	5.466	3.123	**2.747**	**3.144**	**3.118**	3.364	**2.557**	**2.371**	**2.436**	**4.982**	**4.919**	**2.481**	**3.171**	4.933	4.474
OLYP	**3.779**	6.499	**3.087**	**3.128**	**3.529**	**3.469**	3.048	**1.615**	**1.955**	**1.712**	**5.238**	**5.465**	**2.906**	**3.476**	5.496	4.815
OPBE	**3.557**	**6.216**	**3.118**	**3.381**	**3.906**	**3.770**	**2.464**	**2.675**	**3.610**	**2.677**	**5.105**	**4.586**	**2.841**	**4.105**	**4.702**	**4.918**
OPW91	**3.519**	**6.179**	**3.027**	**3.257**	**3.779**	**3.650**	**2.409**	**2.534**	**3.434**	**2.528**	**5.054**	**4.577**	**2.734**	**3.967**	**4.725**	**4.901**
PBE	6.117	7.398	5.132	4.121	3.845	3.980	5.910	4.649	3.941	4.544	5.792	6.273	4.147	3.703	7.756	6.948
PBE0	6.170	5.685	5.210	3.925	3.838	3.933	5.611	5.044	4.240	4.909	5.451	5.246	3.919	4.063	6.358	5.308
TPSS	4.505	6.784	**3.498**	3.396	**3.386**	**3.397**	3.904	**2.976**	**2.976**	**2.882**	**5.218**	**5.454**	**3.075**	**3.238**	6.200	5.822
VSXC	5.690	7.617	5.270	5.953	5.928	5.880	5.204	4.992	4.759	4.924	7.372	8.752	5.242	4.895	7.289	6.707
wB97	6.504	6.081	5.882	4.487	4.413	4.505	6.302	5.844	4.987	5.707	5.682	5.162	4.504	4.784	6.799	5.842
wB97X	7.392	6.614	6.588	4.857	4.708	4.824	7.048	6.519	5.631	6.388	6.157	5.789	5.049	5.061	7.501	6.466
X3LYP	7.831	7.325	7.021	5.251	4.980	5.120	7.742	6.827	5.939	6.713	6.849	6.881	5.511	5.138	8.308	7.146
**(B)**																
**Method**	**Studied Compounds**															
	**B ^17^**		**C ^18^**	**D ^19^**	**E ^20^**	**E ^21^**	**G ^22^**	**H ^23^**	**H ^24^**	**H ^25^**	**I ^26^**	**J ^27^**	**K ^28^**	**K ^29^**	**L ^30^**	**M ^31^**
B3LYP	7.528		7.210	5.228	4.764	4.854	7.960	6.962	5.826	6.860	6.809	6.820	5.387	4.884	8.523	7.284
B3P86	6.262		5.873	4.188	3.743	3.807	6.436	5.655	4.513	5.539	5.712	5.591	4.142	3.905	7.074	5.888
B3PW91	6.312		5.907	4.187	3.765	3.827	6.465	5.671	4.527	5.555	5.786	5.563	4.179	3.961	7.083	5.935
B98	6.454		6.135	4.342	3.816	3.873	6.657	5.880	4.711	5.776	6.025	6.122	4.283	3.882	7.245	6.072
B971	6.401		5.995	4.217	3.708	3.759	6.522	5.731	4.573	5.628	5.889	6.012	4.122	3.749	7.166	6.004
B972	**5.205**		**4.132**	2.948	2.840	**2.770**	4.453	3.821	2.819	3.708	**4.795**	**4.514**	2.391	2.918	5.219	**4.358**
BHandH	6.763		7.292	6.086	5.764	5.819	7.305	7.349	6.213	7.212	6.649	6.376	6.121	6.211	8.433	6.856
BHandHLYP	6.309		6.745	5.209	4.947	4.990	7.061	6.698	5.526	6.570	6.443	5.889	5.237	5.335	7.525	6.165
BLYP	9.744		7.809	5.908	5.351	5.459	8.808	7.375	6.356	7.303	7.561	7.994	6.110	5.223	10.364	9.456
BMK	*17.484*		*18.809*	*14.715*	*13.785*	*13.946*	*19.003*	*18.728*	*17.568*	*18.624*	*15.731*	*14.903*	*15.429*	*14.618*	*19.585*	*18.039*
BP86	7.951		6.070	4.337	3.739	3.820	6.795	5.638	4.657	5.560	5.980	6.384	4.283	3.592	8.281	7.501
CAM-B3LYP	7.984		8.319	6.187	5.741	5.842	8.970	8.169	7.028	8.056	7.434	7.134	6.470	6.105	9.337	7.943
HF	7.377		5.758	5.898	5.983	5.942	5.398	6.062	5.121	5.925	6.503	**5.128**	5.568	6.516	7.993	7.227
LC-BLYP	10.798		11.825	9.334	8.812	8.938	12.250	11.820	10.735	11.705	9.975	9.298	9.849	9.527	12.613	11.122
M06	5.473		5.828	4.728	4.234	4.263	6.246	5.851	4.730	5.710	5.751	5.587	4.595	4.513	6.814	5.309
M06-2X	16.686		17.894	14.073	13.238	13.386	17.741	17.986	16.865	17.876	14.847	13.824	14.892	14.235	19.085	17.248
mpW1LYP	7.273		7.186	5.242	4.833	4.911	7.896	6.976	5.828	6.871	6.767	6.822	5.368	4.943	8.360	7.038
mpW1PW91	5.854		5.621	4.058	3.715	3.754	6.088	5.432	4.276	5.309	5.569	5.300	3.967	3.931	6.663	5.452
O3LYP	5.752		3.983	**2.747**	**2.866**	**2.738**	4.189	**3.459**	**2.775**	**3.380**	**4.890**	**4.844**	**2.208**	**2.807**	5.241	4.784
OLYP	6.851		**4.324**	**3.128**	**3.280**	**3.127**	4.299	**3.709**	**3.507**	**3.685**	**5.149**	**5.397**	**2.674**	**3.142**	5.904	5.844
OPBE	**6.444**		**4.011**	**3.381**	**3.694**	**3.473**	**3.581**	**3.620**	**3.982**	**3.619**	**5.018**	**4.510**	**2.613**	**3.839**	**4.898**	**5.191**
OPW91	**6.418**		**3.950**	**3.257**	**3.560**	**3.342**	**3.547**	**3.525**	**3.831**	**3.520**	**4.966**	**4.500**	**2.497**	**3.690**	**4.935**	**5.184**
PBE	7.696		5.812	4.121	3.616	3.682	6.511	5.375	4.398	5.293	5.713	6.215	3.989	3.395	8.088	7.240
PBE0	5.665		5.384	3.925	3.621	3.648	5.815	5.191	4.039	5.067	5.372	5.181	3.761	3.799	6.485	5.241
TPSS	7.179		**4.684**	3.396	**3.146**	**3.073**	4.984	**4.275**	**3.727**	**4.215**	**5.137**	**5.392**	**2.877**	**2.911**	6.733	6.339
VSXC	8.447		6.788	5.953	5.861	5.788	6.867	6.487	6.150	6.470	7.323	8.736	5.138	4.706	9.225	7.720
wB97	5.929		5.891	4.487	4.249	4.289	6.396	5.767	4.603	5.638	5.615	5.100	4.379	4.583	6.831	5.585
wB97X	6.504		6.602	4.857	4.544	4.608	7.134	6.449	5.287	6.326	6.091	5.731	4.932	4.862	7.578	6.252
X3LYP	7.417		7.215	5.251	4.806	4.894	7.942	6.989	5.847	6.885	6.782	6.827	5.393	4.921	8.479	7.188

^1^ Experimental data from [50]; ^2^ experimental data from [25]; ^3^ experimental data from [4]; ^4^ experimental data from [51]; ^5^ experimental data from [5]; ^6^ experimental data from [26]; ^7^ experimental data from [52]; ^8^ experimental data from [53]; ^9^ experimental data from [24]; ^10^experimental data from [4]; ^11^ experimental data from [23]; ^12^experimental data from [48]; ^13^ experimental data from [49] in CDCI_3_; ^14^ experimental data from [49] in D_2_O; ^15^ experimental data from [25]; ^16^ experimental data from [25]. ^17^ Experimental data from [25]; ^18^ experimental data from [4]; ^19^ experimental data from [51]; ^20^ experimental data from [5]; ^21^ experimental data from [26]; ^22^ experimental data from [52]; ^23^ experimental data from [53]; ^24^ experimental data from [24]; ^25^ experimental data from [4]; ^26^ experimental data from [23]; ^27^ experimental data from [48]; ^28^ experimental data from [49] in CDCI_3_; ^29^ experimental data from [49] in D_2_O; ^30^ experimental data from [25]; ^31^ experimental data from [25].

In the case of fluorinated cathinones, ^19^F NMR data for only three studied derivatives were available in the literature. However, an unambiguous trend in the most accurate prediction using BHandH and LC-BLYP density functionals was noted (see Table 3 and Table S20). The best reproduction of the experiment was observed in a vacuum, similar to ^13^C data. A small influence of *R/S*-configuration change on the deviation from experimental values was also noticed.

**Table 3:** Deviations of ^19^F NMR chemical shifts (in ppm) calculated in the gas phase with the studied methods for *R,S*-configurations of cathinone fluorinated derivatives. Deviations for the best-performed methods are in bold, while deviations for the worst method are in underlined italics.

Method	Studied Compounds					
	*R*			*S*		
	C ^1^	E ^2^	H ^3^	C ^1^	E ^2^	H ^3^
B3LYP	−16.919	−20.315	−22.487	−16.766	−20.315	−22.186
B3P86	−9.618	−12.890	−15.065	−9.673	−12.890	−14.908
B3PW91	−8.972	−12.296	−14.468	−9.004	−12.296	−14.297
B98	−11.985	−15.418	−17.503	−11.870	−15.418	−17.358
B971	−12.616	−16.040	−18.135	−12.518	−16.040	−18.005
B972	−6.449	−9.898	−12.039	−6.486	−9.898	−11.890
BHandH	**2.249**	**−0.911**	**−3.166**	**2.188**	**−0.911**	**−3.004**
BHandHLYP	**−0.842**	−4.536	−6.674	**−0.938**	−4.536	−6.392
BLYP	*−35.281*	*−38.070*	*−40.355*	*−35.121*	*−38.070*	*−39.088*
BMK	−9.804	−13.526	−15.731	−9.696	−13.526	−15.218
BP86	−26.807	−29.222	−31.434	−26.698	−29.222	−31.226
CAM-B3LYP	−7.152	−11.273	−13.449	−7.207	−11.273	−13.183
HF	14.499	12.023	9.899	14.276	12.023	10.273
LC-BLYP	**5.656**	**1.735**	**−0.492**	**5.606**	**1.735**	**−0.268**
M06	−10.749	−13.198	−15.886	−10.757	−13.198	−15.024
M06-2X	−6.008	−9.938	−12.387	−6.052	−9.938	−11.990
mpW1LYP	−15.508	−19.180	−21.434	−15.554	−19.180	−21.121
mpW1PW91	−5.826	−9.134	−11.396	−5.878	−9.134	−11.201
O3LYP	−11.240	−14.685	−16.692	−10.986	−14.685	−16.462
OLYP	−19.011	−22.394	−24.140	−18.787	−22.394	−23.011
OPBE	−8.383	−11.272	−13.064	−8.211	−11.272	−12.998
OPW91	−8.983	−11.889	−13.685	−8.810	−11.889	−13.601
PBE	−24.498	−26.844	−29.042	−24.388	−26.844	−28.892
PBE0	−5.790	−9.177	−11.335	−5.850	−9.177	−11.228
TPSS	−18.531	−20.444	−22.610	−18.328	−20.444	−22.525
VSXC	−19.051	−22.203	−23.336	−19.778	−22.203	−23.685
wB97	**0.620**	−3.508	−5.695	**0.554**	−3.508	−5.576
wB97X	−1.661	−5.843	−8.004	−1.720	−5.843	−7.912
X3LYP	−16.073	−19.670	−21.845	−16.105	−19.670	−21.561

^1^ Experimental data from [4]; ^2^ experimental data from [26]; ^3^ experimental data from [53].

When analyzing the performance of different methods in the prediction of chemical shifts, it is worth paying attention to Table 4. It contains all the best-performed methods for ^1^H, ^13^C, and ^19^F shift calculations. It is worth mentioning that in the current work, theoretical chemical shifts were compared with 17, 16, and 3 data sets for ^1^H, ^13^C, and ^19^F shifts, respectively. Table 4 indicates how often different methods were among the best performers for different nuclei. As can be observed, it was not possible to choose a method that would be optimal for the calculations of all analyzed nuclei chemical shifts. However, it is worth noting that the OLYP density functional was among the best-performed methods for the prediction of ^1^H and ^13^C shifts in the majority of studied cathinones. Therefore, we recommend it as the most optimal way for simultaneous prediction of carbon and proton data. It is apparent from the results mentioned above that BLYP and BP86 are suitable for ^1^H, and OPBE and OPW91 are well-performing for ^13^C. Since the unambiguous influence of the environment polarity on ^1^H chemical shift values was not noticed, and the RMS value deviations were low, it is recommended to predict both ^1^H and ^13^C nuclei in a vacuum to reduce calculation time. In the case of the ^19^F spectrum, calculations in the gas phase using BH and H or the LC-BLYP functional are recommended.

### 2.2. Performance of Optimal Methods in Prediction of Chemical Shifts

**Figure 3:**
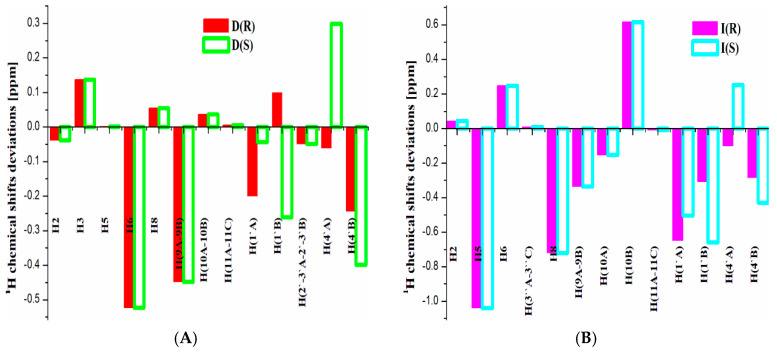
OLYP/6-311++G** predicted ^1^H shift deviations for (**A**) most accurately (structure D) and (**B**) worst (structure I) predicted cathinone derivatives in *R* and *S* configurations in the gas phase.

Analyzing the accuracy of ^1^H shift prediction for atoms in different chemical environments, it is worth noting an ambiguous trend (see Figure 3). Most often, the highest deviation from the experiment was obtained for a chemical shift of the H8 proton (up to 0.8 ppm using OLYP; see Tables S1–S17). It could be due to the proximity of protonated or deprotonated amine groups in different environments, leading to different electron density distributions. However, its signal appears in a specific spectral position (about 4.5–6 ppm), where other proton signals of cathinones are not observed. Therefore, in the routine analysis of experimental spectra, this deviation will not provide the incorrect H8 signal assignment. Other ^1^H chemical shifts were characterized by relatively small deviations. However, it is worth mentioning that in symmetric *p*-fluorinated cathinones (structures D, E, F, H, J, and K: see Tables S1–S17 and Figure 3), some differences were observed in the prediction of magnetically equivalent nuclei. This effect can be explained by the lack of rotation of the molecular fragments during calculations. To minimize this effect, it is recommended to average the theoretical shifts of magnetically equal nuclei. The difference in the position of the diasteretopic nuclei in the pyrrolidine ring is also noted for the *R/S*-configurations. As a result, this effect allows the use of theoretical ^1^H spectra for the determination of the dominant configuration of cathinones in the solution. Despite the fact that every ^1^H shift was predicted with a certain error, it is also apparent that the prediction of ^1^H NMR data is good enough to follow the experimental order of peaks in a spectrum (see Tables S1–S17).

**Figure 4:**
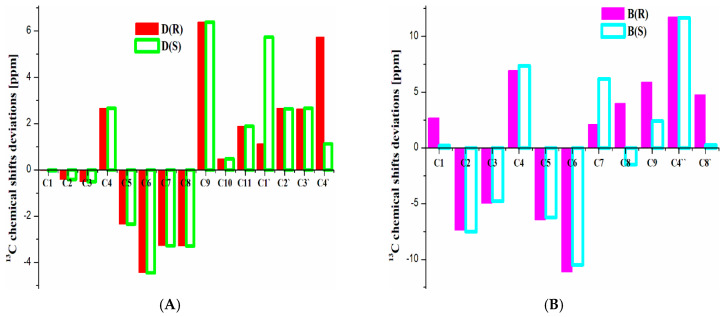
OLYP/6-311++G** predicted ^13^C shift deviations for (**A**) most accurately (structure D) and (**B**) worst (structure B) predicted cathinone derivatives in *R* and *S* configurations in the gas phase.

In the case of ^13^C spectra, it was more difficult to accurately predict the chemical shift of atoms with different chemical environments (see Figure 4 and Tables S1–S17). However, several trends for ^13^C data were found to be similar to ^1^H theoretical chemical shifts. In the case of *p*-fluorinated derivatives, small deviations between magnetically equivalent signal values were also noticed. Such shifts should also be averaged. In addition, the differences in the ^13^C shifts of the pyrrolidine ring were noticed, which serves as additional information to determine the dominant *R/S*-configuration of the cathinone derivative in solution. However, certain difficulties were observed in the prediction of the relative positions of nuclei in the ^13^C spectrum. In the case of *p*-fluorinated derivative K, if its theoretical values were compared with experimental data [49] in CDCI_3_, relative positions of C4, C5, and C3 atom signals reproduced the experiment, but in the case of D_2_O, the opposite trend was shown. In the case of another *p*-fluorinated derivative, D [51], the theoretical relative positions of signals were well reproduced in the experiment. Moreover, for *m*-fluorinated derivative G, the theoretical relative position of all signals on the ^13^C spectrum was also well reproduced in the experiment. Based on these results, it could be assumed that the problems in the experimental signals assignment may be responsible for such deviations. ^13^C NMR data for derivatives B, L, and M were predicted with the lowest accuracy (see Figure 4B). Each of these compounds has the –CF_3_ substituent in different positions of a benzene ring. As a result of calculations with each theoretical method, the highest deviations from the experimental values were obtained for ^13^C shifts of carbon in the -CF_3_ group and carbon *ipso*. As a result of calculations on model substances (CH_3_F, benzene, fluorobenzene, and trifluorotoluene), similar deviations were observed for trifluorotoluene (about 11 ppm for –CF_3_ and about 1 ppm for C *ipso*), while other models ^13^C shifts were predicted with high accuracy (see Table S21). Consequently, these deviations for the studied cathinones could be explained by the deficiencies of the used theoretical methods and the different influence of substituents with fluorine on the accuracy of the theoretical ^13^C shifts.

Currently, the only ^19^F NMR data available for a single fluorine atom in *ortho*- and *para*-position is for a carbonyl-substituted carbon. Theoretical data, presented in Table 3 and Table S20, varied for different methods and cathinone derivatives. However, observed deviations were not significant and could be explained by the accidental error compensation.

**Figure 5:**
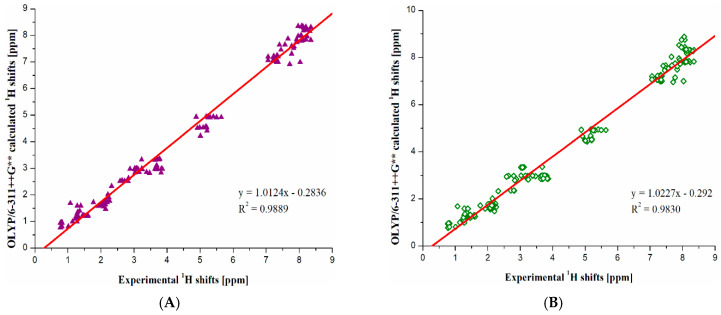
Correlation between theoretical and experimental ^1^H NMR chemical shifts for (**A**) *R*-configurations and (**B**) *S*-configurations of the studied cathinones. Parameters of linear correlations are also given.

**Figure 6:**
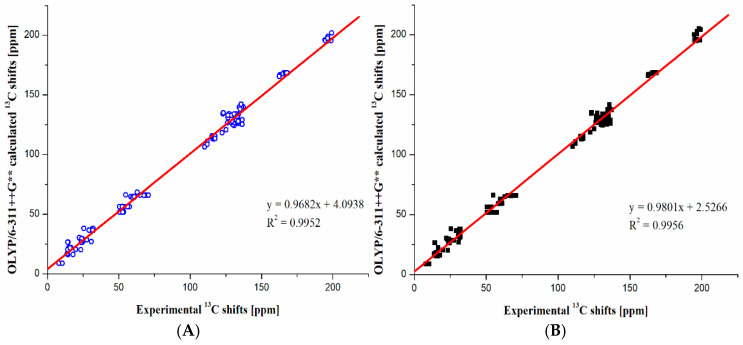
Correlation between theoretical and experimental ^13^C NMR chemical shifts for (**A**) *R*-configurations and (**B**) *S*-configurations of the studied cathinones. Parameters of linear correlations are also given.

It is pleasing to notice that all theoretical results calculated using the best-performed method (OLYP for ^1^H and ^13^C NMR, see Figure 5 and Figure 6) show very good agreement with experimental data for all 13 studied compounds. This is reflected by a very high R^2^ value and linear coefficient close to 1. In the case of ^1^H NMR, the R^2^ = 0.9889 and 0.9830, while linear coefficients of a fitting formula y = A*x + B, where A and B are fitting parameters, are 1.0124 and 1.0227 for *R*- and *S*-configurations, respectively. In the case of ^13^C NMR, the R^2^ = 0.9952 and 0.9956, while linear coefficients A are 0.9682 and 0.9801 for R- and S-configurations, respectively. It should be mentioned that the perfect agreement is for A = 1.0000. According to R^2^ values, the highest sensitivity of ^1^H signals to *R/S*-configuration change is additionally confirmed. On the other hand, the ^13^C nuclei are significantly less sensitive to configuration change. For this reason, the R^2^ values of ^13^C shifts are, in general, larger. Thus, the differences in R^2^ values between *R/S*-configurations are smaller than for ^1^H data (compare Figure 5 and Figure 6).

In the case of ^19^F data, experimental results for only three compounds are available. For this reason, it is hard to observe a clear statistical correlation between such a small amount of data. It should also be noticed that the difference between experimental results for *p*- and *m*-fluorinated cathinones is about 10 ppm. Therefore, the corresponding linear-like dependence of theoretical vs. experimental deviations is less perfect, and the statistical parameters of the linear fit are worse (see Figure S1 in Supporting Information).

## 3. Methodology

All calculations were performed using the Gaussian 16 program package [56,57]. A neutral form of cathinone and its 12 fluorinated derivatives were studied. In the case of cathinone, *S*-configuration was analyzed as a naturally occurring psychoactive form [58]. Due to limited data on the biological effects of *R*/*S*-configurations of fluorinated derivatives of cathinone, both drug forms were studied. In the first step, a full structure optimization of cathinone and its fluorinated derivatives was performed. Calculations were carried out with very tight convergence criteria and default grid. The HF method and 28 density functionals, including B3LYP [59,60,61], B3P86 [62], B3PW91 [63], B98 [64], B971 [65], B972 [66], BHandH [67], BHandHLYP [67], BLYP [68], BMK [69], BP86 [59], CAM-B3LYP [70], LC-BLYP [71], M06 [72], M06-2X [72], mpW1LYP [73], mpW1PW91 [73], O3LYP [74], OLYP [74], OPBE [75], OPW91 [76], PBE [77], PBE0 [78], TPSS [79], VSXC [80], wB97 [81], wB97X [81], and X3LYP [82], were chosen. These density functionals were selected according to literature data [83] reporting their good efficiency in NMR parameters prediction of some organic compounds. A middle-size Pople type basis set 6-311++G** was used to analyze the method performance. This basis set is suitable for calculations using both supercomputers and a standard desktop computer in an analytical laboratory. It is also important to mention that larger basis sets may result in a surprising worsening of the method performance [84]. All compounds were optimized in the gas phase, as well as in solvents of different polarity (chloroform, DMSO, methanol, ethanol, and water using PCM [45] and SMD [46] solvent models). A lack of imaginary frequencies confirmed the ground state of the optimized structures.

NMR spectra prediction was performed at the same level of theory. Isotropic nuclear magnetic shieldings were calculated using the GIAO approach [85,86]. Theoretical chemical shift *δ*_i_ (in ppm) was calculated according to Equation (1):

(1) $$\delta_{i}=\sigma_{ref}-\sigma_{i}$$

where *σ_ref_* and *σ_i_* are isotropic shieldings of the reference and studied compound nucleus, respectively. ^1^H, ^13^C, and ^19^F chemical shifts were referenced using TMS for carbon and proton signals and CFCI_3_ for ^19^F.

## 4. Conclusions

As a result of systematic theoretical studies, ^1^H, ^13^C, and ^19^F NMR spectral data of the *S*-cathinone and *R/S*-configurations of its 12 fluorinated derivatives were predicted. It was noted that a relatively small basis set 6-311++G** yields results with acceptable accuracy. Twenty-nine analyzed methods demonstrated different accuracies in the ^1^H, ^13^C, and ^19^F spectra calculations. A nice linear correlation between theoretical (DFT) predicted proton and carbon chemical shifts with available experimental data was observed. The chemical environment of atoms played a key role in the performance of chemical shift prediction. However, the use of the best-performed methods for different nuclei can provide an accurate prediction of signal positions and determination of the cathinone dominant configuration in solution. It was not possible to find the optimal method for all studied nuclei. However, it was observed that the OLYP density functional yields the most accurate data for ^1^H and ^13^C. ^19^F data of cathinones should be predicted using BH and H or LC-BLYP. It is also recommended to perform all calculations in the gas phase, since the highest accuracy of carbon-13 and fluor-19 data could be obtained. In the case of ^1^H shift calculations, no significant impact of solvent polarity was observed.

## Figures and Tables

**Table 4:** Performance of different methods for ^1^H, ^13^C, and ^19^F chemical shift prediction. Recommended methods are marked in bold.

Method	δ, ppm		Σ_δC+δH_
	^13^C	^1^H	
**O3LYP**	11	2	13
**OLYP**	**12**	**14**	**26**
OPBE	16	10	26
OPW91	16	10	26
B972	6	-	6
TPSS	9	9	18
HF	1	-	1
BLYP	-	16	16
BP86	-	16	16
PBE	-	16	16
VSXC	-	2	2
**Method**	**^19^F**		
**BHandH**	**3**		
BHandHLYP	1		
**LC-BLYP**	**3**		
wB97	1		

## Data Availability

Data are contained within the article and Supplementary Materials.

## Supplementary Materials

The following supporting information can be downloaded at: https://www.mdpi.com/article/10.3390/molecules30010054/s1. Table S1. Theoretical chemical shifts (in ppm) of S-cathinone calculated with HF and selected functionals combined with 6-311++G** basis set. Table S2. Theoretical chemical shifts (in ppm) of (R,S)-4-trifluoromethylmethcathinone calculated with HF and selected functionals combined with 6-311++G** basis set. Table S3. Theoretical chemical shifts (in ppm) of (R,S)-3-fluoromethcathinone calculated with HF and selected functionals combined with 6-311++G** basis set. Table S4. Theoretical chemical shifts (in ppm) of (R,S)-4-fluoro-α-pyrrolidinopentiophenone calculated with HF and selected functionals combined with 6-311++G** basis set. Table S5. Theoretical chemical shifts (in ppm) of (R,S)-4-fluoro-α-pyrrolidinohexanophenone calculated with HF and selected functionals combined with 6-311++G** basis set. Table S6. Theoretical chemical shifts (in ppm) of (R,S)-4-fluoro-α-pyrrolidinohexanophenone calculated with HF and selected functionals combined with 6-311++G** basis set. Table S7. Theoretical chemical shifts (in ppm) of (R,S)-4-fluoro-α-pyrrolidinononanophenone calculated with HF and selected functionals combined with 6-311++G** basis set. Table S8. Theoretical chemical shifts (in ppm) of (R,S)-3,5-difluoromethcathinone calculated with HF and selected functionals combined with 6-311++G** basis set. Table S9. Theoretical chemical shifts (in ppm) of (R,S)-flephedrone calculated with HF and selected functionals combined with 6-311++G** basis set. Table S10. Theoretical chemical shifts (in ppm) of (R,S)-flephedrone calculated with HF and selected functionals combined with 6-311++G** basis set. Table S11. Theoretical chemical shifts (in ppm) of (R,S)-flephedrone calculated with HF and selected functionals combined with 6-311++G** basis set. Table S12. Theoretical chemical shifts (in ppm) of (R,S)-4-fluoro-3-methyl-α-pyrrolidinopentiophenone calculated with HF and selected functionals combined with 6-311++G** basis set. Table S13. Theoretical chemical shifts (in ppm) of (R,S)-4-fluoro-α-pyrrolidinooctanophenone calculated with HF and selected functionals combined with 6-311++G** basis set. Table S14. Theoretical chemical shifts (in ppm) of (R,S)-4-fluoro-α-pyrrolidinobutyrophenone calculated with HF and selected functionals combined with 6-311++G** basis set. Table S15. Theoretical chemical shifts (in ppm) of (R,S)-4-fluoro-α-pyrrolidinobutyrophenone calculated with HF and selected functionals combined with 6-311++G** basis set. Table S16. Theoretical chemical shifts (in ppm) of (R,S)-2-trifluoromethylmethcathinone calculated with HF and selected functionals combined with 6-311++G** basis set. Table S17. Theoretical chemical shifts (in ppm) of (R,S)-3-trifluoromethylmethcathinone calculated with HF and selected functionals combined with 6-311++G** basis set. Table S18. RMS values of proton chemical shifts of S-cathinone and its selected fluorinated synthetic derivatives calculated with HF and selected functionals combined with 6-311++G** basis set. The most accurate methods and their smallest RMS values are in boald and marked in green, the worst methods with their highests RMS values are in italic font and marked in orange. Table S19. RMS values of carbon-13 chemical shifts of S-cathinone and its selected fluorinated synthetic derivatives calculated with HF and selected functionals combined with 6-311++G** basis set. The most accurate methods and their smallest RMS values in boald and marked in green, the the worst methods with its highests RMS values are in italic font and marked in orange. Table S20. Deviations of calculated fluorine-19 chemical shifts from experimental data of selected fluorinated synthetic derivatives of cathinone calculated with HF and selected functionals combined with 6-311++G** basis set. Smallest deviations of most accurate methods are in boald and marked in green, highests deviations of the worst methods are in italic font and marked in orange. Table S21. Theoretical chemical shifts [ppm] of some nuclei of model fluorinated compounds calculated in the gas phase using OLYP/6-311++G** level of the theory compared with available experimental data. Figure S1. Correlation between theoretical and experimental ^19^F NMR chemical shifts for (A) R-configurations and (B) S-configurations of the studied cathinones. Parameters of linear correlations are also given. Refs. [87,88,89] are cited in the Supplementary Materials.
