# Supplementary material for: The Search for the Optimal Methodology for Predicting Fluorinated Cathinone Drugs NMR Chemical Shifts
Source: Molecules. 2024 Dec 27;30(1):54. doi: 10.3390/molecules30010054 (PMC11721958; doi:10.3390/molecules30010054)
Supplement: Supplementary file 1 [file molecules-30-00054-s001.zip › molecules-3388179-supplementary.pdf]

SUPPORTING INFORMATION FOR

The search for the optimal methodology for predicting fluorinated cathinone drugs NMR chemical shifts

Natalina Makieieva*, Teobald Kupka* and Oimahmad Rahmonov

For better comparison of results, the data in Tables S1-S21 are given with four dessimal places.

Table S1. Theoretical chemical shifts (in ppm) of *S*-cathinone calculated with HF and selected functionals combined with 6-311++G** basis set[a]

Nuclei	B3LYP											B3P86											Exp. [b]
	Gas	$CHCl_3$ (PCM)	$CHCl_3$ (SMD)	DMSO (PCM)	DMSO (SMD)	CH_3OH (PCM)	CH_3OH (SMD)	C_2H_5OH (PCM)	C_2H_5OH (SMD)	H_2O (PCM)	H_2O (SMD)	Gas	$CHCl_3$ (PCM)	$CHCl_3$ (SMD)	DMSO (PCM)	DMSO (SMD)	CH_3OH (PCM)	CH_3OH (SMD)	C_2H_5OH (PCM)	C_2H_5OH (SMD)	H_2O (PCM)	H_2O (SMD)	
**C1**	140.8253	141.1187	142.1132	141.2558	142.6706	141.2401	142.4580	141.2248	142.4660	141.2699	142.4982	138.6291	138.8962	139.9644	139.0633	140.4390	139.0716	140.2763	139.0583	140.4139	139.0996	140.3400	**132.3**
**C2**	136.2269	135.9719	136.4747	135.7308	136.2765	135.7409	136.6568	135.7508	136.5509	135.7199	136.6695	135.2377	134.9625	135.5217	134.7575	135.3722	134.7904	135.6745	134.8016	135.6927	134.7703	135.7073	**129.3**
**C3**	135.1175	135.9997	136.5970	136.2776	137.0283	136.2581	137.1773	136.2382	137.1068	136.2944	137.2567	134.1100	135.0070	135.6838	135.3367	136.1793	135.3392	136.3177	135.3195	136.3582	135.3783	136.4260	**128.9**
**C4**	139.5081	141.4069	142.0273	142.1559	142.7598	142.1096	143.6598	142.0627	143.4039	142.1973	143.8635	138.5201	140.4040	141.0979	141.1919	141.9076	141.1684	142.7638	141.1223	142.6155	141.2580	142.9883	**135.2**
**C5**	133.7356	135.1468	135.8072	135.7648	136.6783	135.7244	136.7579	135.6834	136.6669	135.8014	136.8845	132.7560	134.1915	134.9326	134.8676	135.8685	134.8485	135.9395	134.8072	135.9564	134.9294	136.0934	**128.9**
**C6**	134.6077	136.1580	137.1964	136.9035	138.4376	136.8551	138.8833	136.8062	138.6601	136.9477	139.1613	133.5373	135.0402	136.1159	135.8046	137.3816	135.7798	137.7856	135.7329	137.6741	135.8718	138.0706	**129.3**
**C7**	213.1205	216.8948	218.5279	218.4139	219.1379	218.3225	224.2807	218.2301	223.2978	218.4987	224.9571	211.1221	214.6643	216.3245	216.1126	216.8356	216.0508	221.8637	215.9665	221.0143	216.2142	222.5295	**198.1**
**C8**	57.6817	58.2515	58.8301	58.4304	59.1286	58.4144	59.8120	58.3983	59.6436	58.4442	59.8637	56.1564	56.7270	57.3522	56.9392	57.6140	56.9473	58.3206	56.9335	58.2764	56.9754	58.4000	**51.9**
**C9**	23.8951	24.0979	24.0782	24.2701	24.3789	24.2537	24.2138	24.2373	24.1439	24.2841	24.1358	23.6267	23.8131	23.8150	24.0234	24.1504	24.0295	23.9608	24.0138	24.0072	24.0617	23.9181	**16.6**
**H2**	8.7076	8.6201	8.6182	8.5677	8.5770	8.5707	8.5616	8.5737	8.5635	8.5647	8.5679	8.7654	8.6782	8.6749	8.6267	8.6421	8.6311	8.6164	8.6336	8.6239	8.6238	8.6227	**7.89**
**H3**	7.6873	7.8135	7.8560	7.8587	7.9231	7.8559	7.9353	7.8531	7.9254	7.8611	7.9536	7.7335	7.8655	7.9137	7.9131	7.9842	7.9116	7.9961	7.9084	7.9921	7.9156	8.0160	**7.48**
**H4**	7.7138	7.8998	7.9473	7.9739	8.0378	7.9694	8.0767	7.9649	8.0597	7.9778	8.0998	7.7591	7.9492	8.0025	8.0250	8.0959	8.0219	8.1334	8.0170	8.1224	8.0290	8.1579	**7.67**
**H5**	7.5854	7.7439	7.7856	7.8123	7.8814	7.8081	7.8874	7.8038	7.8763	7.8161	7.9091	7.6285	7.7941	7.8413	7.8654	7.9418	7.8625	7.9464	7.8577	7.9411	7.8694	7.9696	**7.48**
**H6**	8.0664	8.2348	8.3286	8.3115	8.4634	8.3068	8.4902	8.3020	8.4737	8.3157	8.5281	8.1510	8.3149	8.4093	8.3896	8.5467	8.3864	8.5698	8.3815	8.5585	8.3937	8.6090	**7.89**
**H8**	4.3181	4.5117	4.5771	4.5955	4.6934	4.5903	4.7435	4.5850	4.7285	4.6002	4.7547	4.3554	4.5430	4.6098	4.6230	4.7219	4.6195	4.7699	4.6141	4.7604	4.6275	4.7810	**5.06**
**H(9A-9C)**	1.2628	1.2693	1.2570	1.2644	1.2462	1.2646	1.2837	1.2647	1.2759	1.2641	1.2941	1.2622	1.2714	1.2597	1.2669	1.2499	1.2686	1.2875	1.2684	1.2854	1.2666	1.2997	**1.46**
Nuclei	**B3PW91**											**B98**											
**C1**	138.8583	139.2634	140.2055	139.4648	140.8278	139.4536	140.6563	139.4345	140.6904	139.4785	140.7308	139.7533	140.0127	140.9971	140.1901	141.5967	140.1916	141.3447	140.1909	141.3776	140.1916	141.3965	**132.3**
**C2**	135.3457	135.1166	135.6034	134.9091	135.5260	134.9261	135.8212	134.9346	135.7428	134.8952	135.8542	135.0205	134.7603	135.2595	134.5642	135.1609	134.5909	135.4929	134.6153	135.4137	134.5410	135.5146	**129.3**
**C3**	134.2150	135.1321	135.7282	135.4488	136.2803	135.4360	136.4129	135.4143	136.3570	135.4627	136.5229	133.9800	134.8313	135.4365	135.1484	135.9584	135.1457	136.0817	135.1405	136.0327	135.1529	136.1718	**128.9**
**C4**	138.5536	140.4351	141.0538	141.1932	141.8977	141.1559	142.7401	141.1092	142.5010	141.2301	142.9633	138.2677	140.0924	140.7232	140.8570	141.5400	140.8291	142.3968	140.7984	142.1665	140.8851	142.6033	**135.2**
**C5**	132.8749	134.3258	134.9856	134.9794	135.9719	134.9459	136.0339	134.9034	135.9563	135.0130	136.1898	132.6440	134.0126	134.6748	134.6637	135.6283	134.6404	135.6832	134.6144	135.6148	134.6878	135.8202	**128.9**
**C6**	133.5564	135.0682	136.0929	135.8173	137.3888	135.7780	137.7789	135.7295	137.5762	135.8562	138.0721	133.5603	135.0172	136.0512	135.7683	137.3297	135.7388	137.7470	135.7063	137.5513	135.7986	138.0253	**129.3**
**C7**	211.2345	214.9126	216.4394	216.3942	217.0783	216.3133	222.1336	216.2231	221.1772	216.4728	222.8074	209.6080	213.2785	214.8703	214.7988	215.5126	214.7267	220.6274	214.6518	219.6493	214.8691	221.3005	**198.1**
**C8**	56.1416	56.7863	57.3308	57.0265	57.6794	57.0160	58.3723	56.9969	58.2284	57.0387	58.4702	57.8371	58.3960	58.9229	58.6261	59.2562	58.6266	59.9113	58.6248	59.7630	58.6280	59.9789	**51.9**
**C9**	23.2995	23.4921	23.4641	23.6856	23.8209	23.6773	23.6585	23.6604	23.6105	23.6955	23.6201	24.0738	24.2442	24.2280	24.4424	24.5710	24.4437	24.4148	24.4427	24.3678	24.4435	24.3504	**16.6**
**H2**	8.7455	8.6492	8.6508	8.5955	8.6125	8.5994	8.5911	8.6030	8.5944	8.5919	8.5945	8.7273	8.6387	8.6404	8.5880	8.6033	8.5913	8.5900	8.5947	8.5943	8.5852	8.5934	**7.89**
**H3**	7.7355	7.8649	7.9122	7.9130	7.9823	7.9109	7.9968	7.9083	7.9887	7.9152	8.0144	7.7139	7.8436	7.8923	7.8924	7.9627	7.8898	7.9763	7.8873	7.9686	7.8951	7.9929	**7.48**
**H4**	7.7604	7.9478	7.9996	8.0234	8.0923	8.0196	8.1319	8.0155	8.1169	8.0271	8.1540	7.7389	7.9264	7.9805	8.0030	8.0738	7.9988	8.1132	7.9946	8.0986	8.0072	8.1345	**7.67**
**H5**	7.6323	7.7943	7.8415	7.8657	7.9403	7.8621	7.9477	7.8581	7.9383	7.8693	7.9682	7.6153	7.7767	7.8254	7.8487	7.9240	7.8447	7.9316	7.8407	7.9228	7.8527	7.9516	**7.48**
**H6**	8.1353	8.2964	8.3929	8.3712	8.5269	8.3675	8.5517	8.3633	8.5366	8.3749	8.5883	8.0983	8.2621	8.3632	8.3395	8.5012	8.3352	8.5300	8.3309	8.5160	8.3439	8.5659	**7.89**
**H8**	4.3391	4.5245	4.5867	4.6037	4.7000	4.5996	4.7475	4.5950	4.7341	4.6078	4.7558	4.3121	4.4971	4.5653	4.5782	4.6807	4.5735	4.7301	4.5689	4.7174	4.5828	4.7367	**5.06**
**H(9A-9C)**	1.2393	1.2440	1.2348	1.2403	1.2214	1.2412	1.2605	1.2418	1.2547	1.2396	1.2707	1.2628	1.2679	1.2561	1.2646	1.2425	1.2651	1.2827	1.2656	1.2775	1.2644	1.2917	**1.46**
Nuclei	**B971**											**B972**											
**C1**	139.3668	139.6203	140.6194	139.8139	141.2045	139.8074	140.9658	139.8009	140.9977	139.8265	141.0245	136.2011	136.5636	137.4979	136.8490	138.1950	136.8262	137.9832	136.8026	138.0149	136.8679	138.0587	**132.3**
**C2**	134.7335	134.4769	134.9685	134.2892	134.8710	134.3083	135.1926	134.3267	135.1149	134.2773	135.2199	132.7378	132.4726	132.9764	132.3428	132.9077	132.3518	133.2020	132.3584	133.1234	132.3302	133.2372	**129.3**
**C3**	133.7868	134.6485	135.2523	134.9762	135.7812	134.9656	135.9011	134.9542	135.8516	134.9919	135.9975	131.4508	132.2816	132.8829	132.6572	133.4196	132.6372	133.5413	132.6146	133.4882	132.6718	133.6496	**128.9**
**C4**	138.0264	139.8611	140.4886	140.6350	141.3172	140.5992	142.1573	140.5623	141.9297	140.6747	142.3692	135.7289	137.4949	138.1201	138.2925	138.9202	138.2498	139.7447	138.2039	139.5130	138.3280	139.9635	**135.2**
**C5**	132.4519	133.8304	134.4901	134.4930	135.4509	134.4616	135.5030	134.4296	135.4341	134.5287	135.6460	130.1376	131.4779	132.1397	132.1733	133.0909	132.1338	133.1440	132.0918	133.0709	132.2059	133.2975	**128.9**
**C6**	133.3169	134.7711	135.7951	135.5240	137.0798	135.4865	137.4853	135.4485	137.2904	135.5653	137.7705	130.9745	132.3968	133.4204	133.1931	134.6581	133.1486	135.0359	133.1011	134.8414	133.2303	135.3301	**129.3**
**C7**	209.5647	213.1855	214.8007	214.7002	215.4329	214.6216	220.4742	214.5414	219.5180	214.7804	221.1535	206.8457	210.4219	211.9064	211.9682	212.6127	211.8770	217.5295	211.7841	216.5826	212.0507	218.1821	**198.1**
**C8**	57.7265	58.2750	58.7985	58.5120	59.1268	58.5048	59.7750	58.4973	59.6271	58.5247	59.8507	55.1581	55.7377	56.2702	56.0631	56.6448	56.0424	57.2738	56.0199	57.1336	56.0790	57.3700	**51.9**
**C9**	24.0602	24.2414	24.2110	24.4464	24.5613	24.4397	24.4057	24.4326	24.3568	24.4588	24.3561	22.9112	23.0714	23.0947	23.3318	23.4303	23.3169	23.2872	23.2996	23.2442	23.3414	23.2495	**16.6**
**H2**	8.7243	8.6383	8.6373	8.5886	8.6019	8.5916	8.5876	8.5947	8.5919	8.5861	8.5910	8.7315	8.6331	8.6379	8.5812	8.5939	8.5849	8.5751	8.5883	8.5786	8.5774	8.5789	**7.89**
**H3**	7.7162	7.8450	7.8936	7.8943	7.9640	7.8915	7.9769	7.8888	7.9693	7.8972	7.9936	7.7297	7.8563	7.9038	7.9072	7.9738	7.9045	7.9880	7.9015	7.9801	7.9096	8.0056	**7.48**
**H4**	7.7381	7.9244	7.9786	8.0014	8.0717	7.9970	8.1100	7.9927	8.0957	8.0058	8.1313	7.7592	7.9432	7.9952	8.0211	8.0874	8.0169	8.1269	8.0123	8.1121	8.0250	8.1489	**7.67**
**H5**	7.6183	7.7795	7.8279	7.8520	7.9266	7.8478	7.9339	7.8436	7.9250	7.8563	7.9540	7.6260	7.7866	7.8340	7.8603	7.9316	7.8562	7.9394	7.8519	7.9303	7.8641	7.9600	**7.48**
**H6**	8.1046	8.2659	8.3652	8.3423	8.5029	8.3379	8.5305	8.3334	8.5165	8.3469	8.5668	8.1126	8.2739	8.3712	8.3519	8.5021	8.3477	8.5266	8.3432	8.5118	8.3557	8.5628	**7.89**
**H8**	4.3073	4.4912	4.5602	4.5725	4.6750	4.5677	4.7227	4.5630	4.7105	4.5774	4.7293	4.3032	4.4875	4.5500	4.5696	4.6648	4.5649	4.7112	4.5600	4.6978	4.5739	4.7196	**5.06**
**H(9A-9C)**	1.2601	1.2651	1.2519	1.2624	1.2392	1.2627	1.2787	1.2629	1.2734	1.2626	1.2881	1.2330	1.2369	1.2275	1.2369	1.2122	1.2373	1.2487	1.2374	1.2433	1.2364	1.2585	**1.46**
Nuclei	**BHandH**											**BHandHLYP**											
**C1**	140.7900	140.8037	141.6669	140.8704	141.6145	140.8560	141.7805	140.8602	141.8228	140.8539	141.8556	141.1171	141.1608	142.1665	141.2867	142.6574	141.2763	142.2570	141.2692	142.3474	141.2967	142.3026	**132.3**
**C2**	139.8520	139.5078	140.0370	139.2844	140.0285	139.2876	140.2157	139.3094	140.1387	139.2511	140.2632	138.4651	138.1194	138.6237	137.9139	138.4568	137.9263	138.8215	137.9414	138.7531	137.9020	138.8546	**129.3**
**C3**	137.6264	138.4636	139.1356	138.7861	139.5302	138.7578	139.6936	138.7477	139.6604	138.7826	139.7988	135.8460	136.5613	137.1206	136.8464	137.5469	136.8305	137.6018	136.8170	137.5868	136.8609	137.6939	**128.9**
**C4**	143.0358	144.9714	145.7407	145.8009	146.5450	145.7432	147.4860	145.7036	147.2467	145.8248	147.7143	141.1052	142.8817	143.4877	143.6434	144.2454	143.6011	145.1539	143.5602	144.9192	143.6829	145.3761	**135.2**
**C5**	136.0333	137.4978	138.2288	138.2151	138.9592	138.1625	139.2297	138.1282	139.1671	138.2349	139.3922	134.3312	135.6287	136.2583	136.2769	137.1461	136.2387	137.1451	136.2027	137.1023	136.3127	137.2901	**128.9**
**C6**	138.1993	139.8168	141.0135	140.6705	141.4146	140.6070	142.8049	140.5619	142.6034	140.7011	143.1192	136.5140	138.0273	139.0442	138.8189	140.3431	138.7721	140.8083	138.7275	140.6056	138.8625	141.1129	**129.3**
**C7**	212.8064	216.4928	217.9637	218.0078	218.7519	217.9138	223.7319	217.8367	222.6782	218.0660	224.4378	210.6877	214.6185	216.1623	216.2870	216.7511	216.1923	222.3118	216.0988	221.2162	216.3765	223.0179	**198.1**
**C8**	52.2103	52.7223	53.2241	52.9270	53.6711	52.9038	54.1728	52.8991	54.0206	52.9189	54.2992	54.0698	54.5440	55.0296	54.7668	55.3089	54.7532	55.9335	54.7420	55.8045	54.7796	56.0002	**51.9**
**C9**	23.7954	23.9568	23.9549	24.1768	24.9209	24.1497	23.9612	24.1415	23.9699	24.1728	23.9342	23.0867	23.1824	23.1563	23.3736	23.4862	23.3604	23.2341	23.3498	23.2281	23.3858	23.1797	**16.6**
**H2**	9.0253	8.9355	8.9432	8.8872	8.8060	8.8908	8.8801	8.8937	8.8889	8.8849	8.8820	8.9165	8.8237	8.8210	8.7720	8.7873	8.7754	8.7696	8.7787	8.7731	8.7687	8.7741	**7.89**
**H3**	7.8340	7.9670	8.0146	8.0164	7.9352	8.0146	8.0995	8.0119	8.0921	8.0191	8.1175	7.7948	7.9211	7.9658	7.9703	8.0338	7.9678	8.0496	7.9652	8.0413	7.9726	8.0666	**7.48**
**H4**	7.8857	8.0796	8.1330	8.1582	8.0770	8.1547	8.2729	8.1504	8.2572	8.1625	8.2959	7.8613	8.0490	8.1007	8.1275	8.1932	8.1233	8.2394	8.1190	8.2226	8.1314	8.2617	**7.67**
**H5**	7.7305	7.9054	7.9544	7.9827	7.9015	7.9791	8.0671	7.9747	8.0578	7.9871	8.0894	7.6891	7.8523	7.8959	7.9266	7.9952	7.9226	8.0051	7.9184	7.9950	7.9304	8.0261	**7.48**
**H6**	8.3497	8.5217	8.6360	8.6080	8.5268	8.6036	8.8077	8.5983	8.7937	8.6132	8.8476	8.1969	8.3722	8.4722	8.4571	8.6192	8.4525	8.6514	8.4476	8.6335	8.4615	8.6887	**7.89**
**H8**	4.2510	4.4387	4.5024	4.5240	4.4428	4.5201	4.6762	4.5153	4.6604	4.5287	4.6812	4.1446	4.3394	4.4111	4.4282	4.5318	4.4232	4.5868	4.4182	4.5698	4.4330	4.5958	**5.06**
**H(9A-9C)**	1.3172	1.3331	1.3256	1.3340	1.2527	1.3347	1.3551	1.3345	1.3499	1.3344	1.3694	1.2723	1.2766	1.2573	1.2738	1.2475	1.2743	1.2833	1.2746	1.2768	1.2732	1.2919	**1.46**

[a] methyl protons shifts are averaged

[b] according to [1]

Table S1. Cont. I

Nuclei	BLYP											BMK											Exp.[b]
	Gas	$CHCl_3$ (PCM)	$CHCl_3$ (SMD)	DMSO (PCM)	DMSO (SMD)	CH_3OH (PCM)	CH_3OH (SMD)	C_2H_5OH (PCM)	C_2H_5OH (SMD)	H_2O (PCM)	H_2O (SMD)	Gas	$CHCl_3$ (PCM)	$CHCl_3$ (SMD)	DMSO (PCM)	DMSO (SMD)	CH_3OH (PCM)	CH_3OH (SMD)	C_2H_5OH (PCM)	C_2H_5OH (SMD)	H_2O (PCM)	H_2O (SMD)	
**C1**	140.0008	140.3683	141.3039	140.6035	142.0894	140.8626	141.8008	140.6593	142.0242	140.9858	141.9234	157.0784	157.0702	158.3945	157.2660	158.9781	157.2403	158.6759	157.4251	158.6446	157.4531	158.6634	**132.3**
**C2**	133.9448	133.6447	134.0766	133.4261	134.0154	133.7173	134.1921	133.5465	134.2950	133.7770	134.3021	151.4917	150.9931	151.6608	150.6842	151.5855	150.6859	151.8182	150.8993	151.6666	150.8441	151.7994	**129.3**
**C3**	133.9152	134.7866	135.3654	135.1017	135.9361	135.3628	135.9210	135.1615	136.0449	135.4807	136.0995	150.5844	151.3877	152.2060	151.6976	152.8034	151.6654	152.8665	151.8439	152.7272	151.8895	152.9296	**128.9**
**C4**	137.8141	139.6912	140.2799	140.4807	141.1420	140.7147	141.8197	140.4863	141.7704	140.8849	142.1274	155.2394	157.1059	157.9473	157.8918	158.8961	157.8320	159.7389	157.9830	159.3932	158.1095	159.9292	**135.2**
**C5**	132.6375	133.9841	134.6247	134.6206	135.6171	134.8614	135.5244	134.6403	135.6300	135.0184	135.7469	149.0282	150.4597	151.3652	151.1421	152.4616	151.0864	152.4024	151.2416	152.2617	151.3565	152.5332	**128.9**
**C6**	132.6868	134.1712	135.1418	134.9195	136.5241	135.1542	136.7155	134.9269	136.6950	135.3233	137.1029	149.9685	151.5472	152.6620	152.2428	154.1469	152.1815	154.5533	152.3316	154.2295	152.4625	154.7860	**129.3**
**C7**	214.5100	217.9083	219.5856	219.3667	220.3224	219.5574	224.8885	219.2853	224.2709	219.8129	225.6245	228.1863	232.2298	234.2164	233.9949	234.8284	233.8820	240.8416	233.9786	239.6087	234.2633	241.5538	**198.1**
**C8**	60.6156	61.2053	61.7518	61.3723	62.3232	61.6435	62.7931	61.4512	62.8592	61.7414	62.9468	63.2096	63.7660	64.2123	64.0095	64.5723	63.9803	65.2734	64.1620	64.9635	64.1995	65.2948	**51.9**
**C9**	24.4872	24.6944	24.6521	24.8814	25.1237	25.1480	24.8059	24.9529	24.9135	25.2553	24.8356	26.3023	26.3592	26.4049	26.5944	26.8249	26.5643	26.8104	26.7449	26.5908	26.7832	26.6566	**16.6**
**H2**	8.5158	8.4307	8.4298	8.3775	8.3831	8.4284	8.5412	8.3869	8.3753	8.4293	8.5613	9.0875	8.9952	8.9827	8.9475	8.9621	8.9469	8.9514	8.9464	8.9539	8.9385	8.9587	**7.89**
**H3**	7.5687	7.6870	7.7291	7.7299	7.7949	7.7749	7.9752	7.7275	7.8009	7.7873	8.0044	8.1001	8.2370	8.2860	8.2973	8.3648	8.2909	8.3847	8.2845	8.3761	8.2937	8.4017	**7.48**
**H4**	7.5692	7.7482	7.7946	7.8209	7.8839	7.8642	8.0893	7.8149	7.9084	7.8800	8.1232	8.1086	8.3007	8.3556	8.3882	8.4576	8.3803	8.5012	8.3723	8.4858	8.3861	8.5231	**7.67**
**H5**	7.4706	7.6184	7.6596	7.6830	7.7521	7.7268	7.9259	7.6779	7.7502	7.7418	7.9581	7.9923	8.1637	8.2143	8.2469	8.3232	8.2391	8.3246	8.2313	8.3180	8.2446	8.3496	**7.48**
**H6**	7.9240	8.0839	8.1763	8.1562	8.3012	8.1995	8.4960	8.1503	8.3144	8.2154	8.5461	8.5033	8.6774	8.7771	8.7609	8.9353	8.7525	8.9656	8.7440	8.9501	8.7593	8.9924	**7.89**
**H8**	4.4254	4.6097	4.6787	4.6930	4.7910	4.7353	5.0085	4.6852	4.8303	4.7530	5.0325	4.3182	4.4823	4.5714	4.5858	4.6946	4.5772	4.7413	4.5684	4.7248	4.5843	4.7419	**5.06**
**H(9A-9C)**	1.2563	1.2616	1.2535	1.2555	1.2444	1.3036	1.4520	1.2592	1.2795	1.3099	1.4757	1.2762	1.2947	1.2476	1.2943	1.2424	1.2912	1.2789	1.2881	1.2691	1.2877	1.2879	**1.46**
Nuclei	**BP86**											**CAM-B3LYP**											
**C1**	137.2123	137.7375	138.6925	137.9160	139.2776	137.9105	139.2400	137.9004	139.2466	137.9166	139.4585	142.5310	142.7350	143.7443	142.9133	144.3153	142.9034	144.0549	142.8946	144.0960	142.9250	144.1185	**132.3**
**C2**	132.5697	132.4233	132.8637	132.1798	132.7165	132.2026	133.0004	132.2205	132.9176	132.1534	133.1847	138.9249	138.6454	139.1151	138.4380	139.0090	138.4541	139.3610	138.4708	139.2770	138.4251	139.3951	**129.3**
**C3**	132.5824	133.6467	134.2352	133.9533	134.7484	133.9449	134.9143	133.9311	134.8451	133.9561	135.1788	137.2388	138.0368	138.5893	138.3191	139.0883	138.3072	139.1693	138.2957	139.1283	138.3323	139.2661	**128.9**
**C4**	136.4983	138.5341	139.1251	139.3003	139.9369	139.2658	140.7576	139.2253	140.5224	139.3277	141.1385	142.2050	143.8208	144.4098	144.5620	145.2380	144.5245	146.0548	144.4865	145.8266	144.5995	146.2719	**135.2**
**C5**	131.3428	132.8941	133.5383	133.5265	134.4773	133.4977	134.5597	133.4636	134.4730	133.5485	134.8656	135.8035	137.1812	137.8065	137.8273	138.7704	137.7931	138.7848	137.7590	138.7198	137.8616	138.9318	**128.9**
**C6**	131.2429	132.8146	133.7720	133.5283	135.0135	133.4938	135.3616	133.4538	135.1617	133.5557	135.8098	137.1487	138.6788	139.6785	139.4410	141.0372	139.3997	141.4276	139.3586	141.2255	139.4822	141.7265	**129.3**
**C7**	211.5059	214.8691	216.5117	216.1501	217.0579	216.0852	221.7054	216.0145	220.8962	216.2068	222.4997	215.0587	219.0132	220.6480	220.6603	221.3163	220.5702	226.6816	220.4798	225.6588	220.7474	227.4067	**198.1**
**C8**	58.7125	59.3741	59.9780	59.5452	60.2948	59.5419	61.0255	59.5336	60.8838	59.5437	61.2865	56.2265	56.8152	57.31600	57.0423	57.6401	57.0325	58.3340	57.0231	58.1853	57.0537	58.4162	**51.9**
**C9**	24.2157	24.5400	24.4392	24.7264	24.7771	24.7212	24.6223	24.7111	24.5453	24.7266	24.7498	23.6281	23.7215	23.6032	23.8957	23.9758	23.8868	23.6936	23.8785	23.6678	23.9066	23.6296	**16.6**
**H2**	8.5695	8.4885	8.4755	8.4399	8.4351	8.4438	8.4165	8.4474	8.4188	8.4357	8.4348	8.8348	8.7384	8.7323	8.6821	8.6980	8.6858	8.6717	8.6895	8.6772	8.6787	8.6750	**7.89**
**H3**	7.6104	7.7395	7.7761	7.7868	7.8455	7.7850	7.8572	7.7829	7.8487	7.7880	7.8881	7.7687	7.8920	7.9366	7.9384	8.0042	7.9360	8.0163	7.9337	8.0092	7.9406	8.0328	**7.48**
**H4**	7.6080	7.7961	7.8368	7.8719	7.9292	7.8685	7.9647	7.8647	7.9500	7.8747	8.0000	7.8136	7.9957	8.0466	8.0704	8.1377	8.0664	8.1762	8.0624	8.1619	8.0741	8.1976	**7.67**
**H5**	7.5096	7.6686	7.7040	7.7380	7.8011	7.7349	7.8051	7.7314	7.7954	7.7406	7.8390	7.6732	7.8322	7.8759	7.9031	7.9741	7.8992	7.9799	7.8953	7.9713	7.9067	8.0002	**7.48**
**H6**	8.0080	8.1658	8.2460	8.2372	8.3724	8.2340	8.3961	8.2304	8.3823	8.2399	8.4470	8.1724	8.3383	8.4361	8.4160	8.5796	8.4117	8.6008	8.4074	8.5864	8.4200	8.6367	**7.89**
**H8**	4.4728	4.6561	4.7089	4.7345	4.8187	4.7308	4.8656	4.7267	4.8549	4.7377	4.8889	4.2588	4.4530	4.5272	4.5386	4.6481	4.5337	4.6947	4.5290	4.6812	4.5431	4.7025	**5.06**
**H(9A-9C)**	1.2634	1.2703	1.2538	1.2664	1.2408	1.2676	1.2843	1.2686	1.2781	1.2649	1.3102	1.2887	1.2937	1.2782	1.2894	1.2699	1.2900	1.3053	1.2906	1.2999	1.2888	1.3141	**1.46**
Nuclei	**HF**											**LC-BLYP**											
**C1**	139.6607	139.6241	140.5073	139.7386	141.1358	139.7228	140.3960	139.7095	140.5014	139.7550	140.4472	146.4335	146.5179	147.6341	146.6803	148.0758	146.6491	147.8623	146.6450	147.9135	146.6925	147.9311	**132.3**
**C2**	139.7984	139.4288	139.9138	139.1784	139.7397	139.1944	140.1865	139.2106	140.0876	139.1628	140.2275	144.7320	144.3466	144.9226	144.1597	144.7668	144.1492	145.1320	144.1664	145.0434	144.1523	145.1667	**129.3**
**C3**	134.4250	134.9332	135.3717	135.1214	135.7122	135.1101	135.6168	135.0993	135.6227	135.1320	135.6838	142.1299	142.8151	143.4593	143.1146	143.8898	143.0766	143.9553	143.0658	143.9162	143.1331	144.0489	**128.9**
**C4**	141.5127	143.1664	143.7409	143.8266	144.3848	143.7923	145.4481	143.7575	145.1784	143.8574	145.6624	147.6194	149.3801	150.0864	150.1640	150.9007	150.0982	151.7095	150.0594	151.4692	150.2086	151.9257	**135.2**
**C5**	132.7479	133.8752	134.3900	134.4341	135.2114	134.4006	135.0696	134.3675	135.0473	134.4654	135.1978	140.5833	141.9218	142.6663	142.6176	143.6025	142.5552	143.5941	142.5199	143.5281	142.6593	143.7354	**128.9**
**C6**	137.3230	138.8285	139.8520	139.5756	141.0917	139.5349	141.6945	139.4938	141.4667	139.6122	141.9954	142.6364	144.1986	145.2422	145.0634	146.6612	144.9903	147.0472	144.9440	146.8187	145.1157	147.3843	**129.3**
**C7**	204.0791	208.4010	209.7219	210.2412	210.3651	210.1321	216.6742	210.0239	215.3200	210.3447	217.4306	220.5659	224.7425	226.5075	226.5077	227.0605	226.3879	232.8670	226.2944	231.7605	226.6021	233.6017	**198.1**
**C8**	49.2972	49.7513	50.10233	49.9877	50.4171	49.9708	50.9385	49.9550	50.7898	50.0040	50.9852	54.1861	54.8077	55.3009	55.1233	55.5711	55.0839	56.3632	55.0716	56.1971	55.1429	56.4986	**51.9**
**C9**	21.2779	21.2889	21.3256	21.4018	21.6008	21.3951	21.3176	21.3886	21.3283	21.4073	21.2542	23.0604	23.0011	22.8780	23.1812	23.2319	23.1468	22.8636	23.1401	22.8449	23.1968	22.8209	**16.6**
**H2**	9.0113	8.9084	8.9225	8.8476	8.8805	8.8521	8.8727	8.8562	8.8780	8.8433	8.8776	9.0197	8.9088	8.9008	8.8536	8.8714	8.8559	8.8279	8.8598	8.8344	8.8505	8.8307	**7.89**
**H3**	7.7722	7.9034	7.9510	7.9542	8.0226	7.9515	8.0327	7.9485	8.0256	7.9568	8.0503	7.8991	8.0182	8.0640	8.0644	8.1298	8.0611	8.1404	8.0592	8.1338	8.0666	8.1584	**7.48**
**H4**	7.9257	8.1238	8.1821	8.2047	8.2795	8.2003	8.3311	8.1957	8.3131	8.2088	8.3554	7.9699	8.1484	8.2018	8.2225	8.2920	8.2175	8.3290	8.2141	8.3149	8.2262	8.3519	**7.67**
**H5**	7.6612	7.8325	7.8798	7.9100	7.9832	7.9056	7.9890	7.9010	7.9801	7.9141	8.0111	7.8118	7.9715	8.0163	8.0447	8.1154	8.0397	8.1198	8.0362	8.1112	8.0485	8.1422	**7.48**
**H6**	8.1910	8.3879	8.5069	8.4811	8.6641	8.4762	8.7054	8.4708	8.6863	8.4858	8.7438	8.3497	8.5153	8.6125	8.5986	8.7648	8.5928	8.7774	8.5886	8.7621	8.6031	8.8160	**7.89**
**H8**	3.7932	3.9963	4.0749	4.0889	4.2068	4.0837	4.2644	4.0782	4.2449	4.0940	4.2755	4.2365	4.4266	4.5107	4.5102	4.6300	4.5045	4.6671	4.5005	4.6545	4.5144	4.6714	**5.06**
**H(9A-9C)**	1.2245	1.2212	1.2012	1.2143	1.1835	1.2151	1.2120	1.2156	1.2084	1.2135	1.2179	1.3196	1.3261	1.3013	1.3288	1.2974	1.3278	1.3291	1.3283	1.3226	1.3288	1.3428	**1.46**
Nuclei	**M06**											**M06-2X**											
**C1**	137.2959	137.3554	138.4171	137.4923	138.9580	137.4704	138.5981	137.4571	138.6818	137.5283	138.6701	155.6275	155.9009	156.9075	156.0514	157.4126	156.0648	157.2600	156.0794	157.3208	156.0318	157.3499	**132.3**
**C2**	136.5335	136.2154	136.8128	136.0226	136.7106	136.0245	137.0622	136.0341	136.9781	136.0356	137.1015	151.4273	151.0547	151.6292	150.7995	151.4974	150.8357	151.8029	150.8733	151.7298	150.7588	151.8539	**129.3**
**C3**	135.1423	135.9375	136.6414	136.2724	137.1957	136.2436	137.2891	136.2225	137.2479	136.3141	137.3932	151.0502	151.9890	152.7074	152.3393	153.3074	152.3410	153.4136	152.3437	153.3577	152.3311	153.5464	**128.9**
**C4**	140.0619	141.9249	142.6688	142.7534	143.5556	142.6967	144.4568	142.6470	144.2048	142.8215	144.6728	155.3691	157.4042	158.1928	158.2595	159.1718	158.2320	160.0440	158.2051	159.7819	158.2788	160.3034	**135.2**
**C5**	133.7830	135.1241	135.8828	135.8084	136.8851	135.7578	136.9213	135.7148	136.8526	135.8708	137.0784	149.2161	150.8315	151.6534	151.6083	152.7942	151.5836	152.8538	151.5599	152.7641	151.6253	153.0395	**128.9**
**C6**	134.9534	136.3969	137.5431	137.1869	138.8668	137.1302	139.3202	137.0809	139.1081	137.2551	139.5808	149.9291	151.6010	152.7472	152.4542	154.2425	152.4233	154.6584	152.3935	154.4281	152.4772	155.0182	**129.3**
**C7**	211.8406	215.2956	216.9132	216.7621	217.4900	216.6671	222.4224	216.5795	221.4975	216.8664	223.0986	229.7447	233.9967	235.6542	235.7261	236.2097	235.6524	242.3668	235.5784	241.1884	235.7887	243.2011	**198.1**
**C8**	52.8747	53.2467	53.8660	53.4339	54.1741	53.4124	54.7537	53.3989	54.6422	53.4689	54.8089	58.7148	59.4172	59.8416	59.6916	60.2084	59.6971	60.8882	59.7036	60.7442	59.6800	61.0113	**51.9**
**C9**	23.0447	23.1511	23.2516	23.3616	23.6421	23.3374	23.3825	23.3212	23.3548	23.3994	23.2646	23.9249	24.1047	24.1395	24.2659	24.4907	24.2748	24.2687	24.2853	24.2333	24.2513	24.2742	**16.6**
**H2**	8.7512	8.6609	8.6715	8.6034	8.6311	8.6070	8.6197	8.6111	8.6229	8.6006	8.6211	9.2802	9.1886	9.1985	9.1464	9.1715	9.1474	9.1485	9.1482	9.1502	9.1456	9.1507	**7.89**
**H3**	7.7290	7.8605	7.9182	7.9091	7.9847	7.9062	8.0087	7.9039	7.9997	7.9124	8.0239	8.3146	8.4542	8.5107	8.5107	8.5830	8.5064	8.6077	8.5018	8.5990	8.5149	8.6241	**7.48**
**H4**	7.7271	7.9142	7.9722	7.9893	8.0638	7.9849	8.1078	7.9811	8.0923	7.9940	8.1271	8.3275	8.5115	8.5712	8.5902	8.6676	8.5846	8.7022	8.5786	8.6880	8.5957	8.7226	**7.67**
**H5**	7.6099	7.7613	7.8077	7.8258	7.9064	7.8220	7.9111	7.8188	7.9019	7.8299	7.9330	8.2207	8.3887	8.4443	8.4680	8.5505	8.4621	8.5574	8.4560	8.5479	8.4736	8.5777	**7.48**
**H6**	8.2120	8.3595	8.4583	8.4280	8.5875	8.4239	8.6102	8.4204	8.5960	8.4325	8.6377	8.7846	8.9450	9.0544	9.0307	9.2019	9.0243	9.2249	9.0175	9.2078	9.0369	9.2584	**7.89**
**H8**	4.3173	4.4907	4.5604	4.5643	4.6615	4.5597	4.7141	4.5558	4.7015	4.5691	4.7190	4.3313	4.4998	4.5882	4.5830	4.6998	4.5770	4.7401	4.5707	4.7269	4.5887	4.7387	**5.06**
**H(9A-9C)**	1.2282	1.2336	1.2288	1.2254	1.2159	1.2260	1.2527	1.2271	1.2472	1.2254	1.2588	1.3024	1.3171	1.2932	1.3225	1.2915	1.3209	1.3196	1.3189	1.3130	1.3241	1.3313	**1.46**

[a] methyl protons shifts are averaged.

[b] according to [1]

Table S1. Cont. II

Nuclei	mpW1LYP											mpW1PW91											Exp. [b]
	Gas	$CHCl_3$ (PCM)	$CHCl_3$ (SMD)	DMSO (PCM)	DMSO (SMD)	CH_3OH (PCM)	CH_3OH (SMD)	C_2H_5OH (PCM)	C_2H_5OH (SMD)	H_2O (PCM)	H_2O (SMD)	Gas	$CHCl_3$ (PCM)	$CHCl_3$ (SMD)	DMSO (PCM)	DMSO (SMD)	CH_3OH (PCM)	CH_3OH (SMD)	C_2H_5OH (PCM)	C_2H_5OH (SMD)	H_2O (PCM)	H_2O (SMD)	
**C1**	141.0342	141.2018	142.2062	141.3909	142.7682	141.3738	142.5034	141.3586	142.5431	141.4060	142.5316	138.6123	138.9367	139.9004	139.1359	140.4726	139.1208	140.2891	139.1120	140.3310	139.1501	140.3635	**132.3**
**C2**	136.5712	136.2480	136.7437	136.0646	136.5628	136.0727	136.9360	136.0825	136.8566	136.0552	136.9356	135.5123	135.2465	135.7476	135.0433	135.6626	135.0551	135.9587	135.0729	135.8806	135.0313	135.9935	**129.3**
**C3**	135.2922	136.0945	136.6809	136.4259	137.1313	136.4047	137.2405	136.3849	137.2017	136.4441	137.3054	134.1948	135.0691	135.6794	135.3887	136.2267	135.3706	136.3463	135.3583	136.2928	135.4048	136.4542	**128.9**
**C4**	139.8121	141.6490	142.2651	142.4565	143.0279	142.4082	143.9036	142.3612	143.6753	142.4996	144.0935	138.6609	140.5055	141.1447	141.2669	141.9961	141.2241	142.8310	141.1865	142.5896	141.3062	143.0524	**135.2**
**C5**	133.8731	135.2167	135.8655	135.8953	136.7635	135.8526	136.8064	135.8113	136.7455	135.9336	136.9199	132.8276	134.2451	134.9216	134.9075	135.9091	134.8686	135.9608	134.8349	135.8839	134.9436	136.1148	**128.9**
**C6**	135.0215	136.5242	137.5638	137.3309	138.8439	137.2802	139.2725	137.2308	139.0769	137.3769	139.5398	133.7434	135.2291	136.2642	135.9808	137.5791	135.9363	137.9612	135.8972	137.7571	136.0221	138.2449	**129.3**
**C7**	212.7896	216.5521	218.2073	218.1660	218.8608	218.0707	224.0122	217.9763	223.0407	218.2542	224.6910	210.5451	214.1999	215.7470	215.7025	216.3588	215.6163	221.4381	215.5352	220.4681	215.7832	222.1179	**198.1**
**C8**	57.2880	57.7809	58.3419	58.0122	58.6472	57.9945	59.2999	57.9784	59.1614	58.0273	59.3411	55.3732	55.9792	56.5080	56.2213	56.8479	56.2066	57.5282	56.1975	57.3885	56.2346	57.6104	**51.9**
**C9**	23.9827	24.1263	24.0870	24.3578	24.4178	24.3393	24.2158	24.3223	24.1785	24.3737	24.1241	23.2444	23.4084	23.3817	23.6004	23.7479	23.5872	23.5654	23.5798	23.5213	23.6123	23.5222	**16.6**
**H2**	8.7489	8.6605	8.6613	8.6118	8.6199	8.6152	8.6059	8.6182	8.6110	8.6085	8.6078	8.8014	8.7046	8.7044	8.6495	8.6693	8.6532	8.6457	8.6570	8.6489	8.6462	8.6493	**7.89**
**H3**	7.7005	7.8243	7.8712	7.8742	7.9367	7.8718	7.9499	7.8690	7.9431	7.8764	7.9641	7.7652	7.8956	7.9431	7.9436	8.0138	7.9412	8.0281	7.9390	8.0201	7.9460	8.0459	**7.48**
**H4**	7.7326	7.9164	7.9688	7.9953	8.0581	7.9912	8.0985	7.9867	8.0844	7.9991	8.1177	7.7958	7.9838	8.0363	8.0592	8.1297	8.0553	8.1694	8.0514	8.1544	8.0631	8.1917	**7.67**
**H5**	7.5990	7.7542	7.7998	7.8269	7.8940	7.8230	7.9012	7.8187	7.8931	7.8304	7.9189	7.6621	7.8243	7.8714	7.8954	7.9713	7.8916	7.9784	7.8879	7.9690	7.8992	7.9993	**7.48**
**H6**	8.0848	8.2546	8.3549	8.3372	8.4915	8.3327	8.5200	8.3279	8.5061	8.3413	8.5539	8.1779	8.3413	8.4388	8.4164	8.5768	8.4124	8.6011	8.4085	8.5856	8.4204	8.6376	**7.89**
**H8**	4.2822	4.4764	4.5504	4.5671	4.6677	4.5621	4.7195	4.5567	4.7067	4.5717	4.7267	4.3100	4.4975	4.5629	4.5772	4.6769	4.5728	4.7245	4.5686	4.7105	4.5816	4.7329	**5.06**
**H(9A-9C)**	1.2707	1.2763	1.2671	1.2756	1.2551	1.2760	1.2935	1.2762	1.2889	1.2750	1.3001	1.2444	1.2499	1.2376	1.2455	1.2266	1.2462	1.2646	1.2470	1.2588	1.2450	1.2740	**1.46**
Nuclei	**O3LYP**											**OLYP**											
**C1**	135.1298	135.7941	136.8990	137.1851	138.6909	137.1408	138.8350	137.0946	139.0824	137.2250	139.0466	133.4154	135.1976	136.3781	136.5446	138.2528	136.4952	138.1930	136.4354	138.2937	136.5787	138.3629	**132.3**
**C2**	130.8375	130.5900	131.1366	130.5372	131.1953	130.5526	131.4195	130.5629	131.3300	130.5165	131.4237	128.3780	128.2293	128.8305	128.2004	128.8119	128.2119	128.9986	128.2176	128.9409	128.1803	129.0023	**129.3**
**C3**	130.1313	130.9364	131.5863	131.3459	132.1467	131.3330	132.2784	131.3149	132.2405	131.3521	132.3755	128.3085	129.1167	129.8028	129.5149	130.3482	129.5013	130.4791	129.4822	130.4315	129.5185	130.5601	**128.9**
**C4**	133.9955	135.6723	136.3030	136.2758	136.8721	136.2491	137.5391	136.2163	137.2948	136.2933	137.7039	131.7901	133.2935	133.9159	133.8829	134.4648	133.8558	135.1522	133.8236	134.9464	133.9000	135.3058	**135.2**
**C5**	128.8432	130.1185	130.8263	130.7387	131.6875	130.7109	131.7084	130.6778	131.6267	130.7582	131.8354	127.0665	128.2306	128.9497	128.8078	129.7167	128.7811	129.7754	128.7491	129.6966	128.8243	129.8930	**128.9**
**C6**	129.1493	130.5079	131.5112	130.9596	132.3989	130.9294	132.5915	130.8934	132.3475	130.9799	132.7887	126.8794	127.9107	128.8769	128.4412	129.7331	128.4102	129.9626	128.3755	129.7905	128.4624	130.1804	**129.3**
**C7**	206.1988	209.8236	211.4728	212.3073	213.0453	212.2010	218.0244	212.0922	217.3854	212.4047	218.7989	205.1073	209.3827	211.0521	211.7448	212.8612	211.6346	217.4570	211.5141	216.7439	211.8364	218.1218	**198.1**
**C8**	55.6096	56.2735	56.9423	56.9217	57.7363	56.8940	58.5171	56.8626	58.4216	56.9433	58.5731	56.3992	57.3951	58.2424	57.9871	58.5470	57.9641	59.2467	57.9346	59.1116	57.9993	59.3518	**51.9**
**C9**	22.6084	22.7550	22.8398	22.8855	23.0696	22.8803	22.9350	22.8699	22.8634	22.8836	22.8281	22.5893	22.6735	22.8199	22.8846	22.9567	22.8769	22.8869	22.8639	22.8335	22.8833	22.8002	**16.6**
**H2**	8.6475	8.5299	8.5250	8.3790	8.3915	8.3855	8.3516	8.3914	8.3309	8.3723	8.3440	8.5235	8.3157	8.3131	8.1774	8.1714	8.1835	8.1623	8.1900	8.1533	8.1715	8.1599	**7.89**
**H3**	7.7140	7.8322	7.8804	7.8784	7.9446	7.8762	7.9571	7.8737	7.9496	7.8803	7.9739	7.6611	7.7706	7.8193	7.8125	7.8790	7.8104	7.8925	7.8082	7.8851	7.8141	7.9075	**7.48**
**H4**	7.7270	7.9014	7.9531	7.9744	8.0369	7.9708	8.0711	7.9669	8.0575	7.9775	8.0923	7.6575	7.8248	7.8747	7.8914	7.9546	7.8880	7.9890	7.8844	7.9760	7.8942	8.0082	**7.67**
**H5**	7.6096	7.7574	7.8045	7.8197	7.8889	7.8164	7.8933	7.8126	7.8837	7.8228	7.9119	7.5557	7.6892	7.7360	7.7479	7.8167	7.7447	7.8243	7.7413	7.8157	7.7506	7.8413	**7.48**
**H6**	8.0423	8.1961	8.2875	8.2302	8.3668	8.2276	8.3710	8.2244	8.3499	8.2326	8.3991	7.9638	8.0720	8.1513	8.1099	8.2480	8.1071	8.2206	8.1044	8.2453	8.1123	8.2874	**7.89**
**H8**	4.3630	4.5397	4.6063	4.6154	4.6998	4.6123	4.7315	4.6087	4.7191	4.6182	4.7418	4.4310	4.5931	4.6442	4.6469	4.7477	4.6440	4.7809	4.6410	4.7660	4.6494	4.7914	**5.06**
**H(9A-9C)**	1.2058	1.2073	1.1967	1.1763	1.1583	1.1771	1.1871	1.1775	1.1742	1.1753	1.1854	1.1958	1.1699	1.1710	1.1489	1.1194	1.1497	1.1570	1.1505	1.1491	1.1478	1.1552	**1.46**
Nuclei	**OPBE**											**OPW91**											
**C1**	131.0265	133.0024	133.8530	134.0591	135.4874	134.0341	135.6518	133.9909	135.9319	134.0778	135.8999	131.2953	133.2412	134.1193	134.3212	135.7114	134.2861	135.8983	134.2333	136.1872	134.3361	136.1512	**132.3**
**C2**	127.0529	126.9788	127.5161	126.8443	127.5003	126.8685	127.6077	126.8747	127.5473	126.8167	127.6398	127.2720	127.1969	127.7305	127.0667	127.7100	127.0810	127.8212	127.0781	127.7622	127.0353	127.8551	**129.3**
**C3**	126.8965	127.8196	128.4631	128.1500	129.0240	128.1470	129.1639	128.1262	129.1127	128.1478	129.2854	127.0924	128.0159	128.6528	128.3507	129.2093	128.3378	129.3493	128.3078	129.2998	128.3446	129.4724	**128.9**
**C4**	130.3007	131.8372	132.4563	132.3525	133.0338	132.3356	133.6349	132.3008	133.3981	132.3632	133.8132	130.5086	132.0576	132.6642	132.5769	133.2485	132.5497	133.8486	132.5055	133.6127	132.5837	134.0287	**135.2**
**C5**	125.6936	126.9653	127.6571	127.4894	128.4656	127.4728	128.5115	127.4382	128.4104	127.4999	128.6611	125.8887	127.1645	127.8475	127.6918	128.6562	127.6650	128.6995	127.6215	128.5989	127.6984	128.8503	**128.9**
**C6**	125.2037	126.2920	127.2454	126.7787	128.1479	126.7584	128.3078	126.7200	128.0816	126.7932	128.5328	125.4325	126.5316	127.4728	127.0197	128.3837	126.9891	128.5412	126.9418	128.3114	127.0305	128.7673	**129.3**
**C7**	202.6468	206.9020	208.2465	208.9005	209.7081	208.8190	214.3683	208.7189	213.8875	208.9722	215.0932	202.9971	207.2491	208.6136	209.2756	210.0457	209.1835	214.7402	209.0731	214.2731	209.3437	215.4739	**198.1**
**C8**	54.3177	55.4974	56.0919	56.0480	56.5343	56.0424	57.3986	56.0186	57.2414	56.0484	57.5269	54.4940	55.6567	56.2715	56.2202	56.6862	56.2046	57.5646	56.1715	57.3905	56.2167	57.6928	**51.9**
**C9**	21.6593	21.8576	21.9356	22.0012	22.1197	22.0047	22.0287	21.9905	21.9367	21.9932	21.9700	21.7269	21.9284	22.0045	22.0764	22.1894	22.0697	22.0997	22.0464	22.0035	22.0645	22.0418	**16.6**
**H2**	8.5335	8.3176	8.3344	8.2006	8.2179	8.2058	8.1899	8.2104	8.1645	8.1949	8.1858	8.5364	8.3232	8.3369	8.2046	8.2234	8.2092	8.1931	8.2136	8.1669	8.1986	8.1884	**7.89**
**H3**	7.7195	7.8371	7.8841	7.8799	7.9484	7.8780	7.9614	7.8754	7.9526	7.8810	7.9794	7.7185	7.8358	7.8825	7.8791	7.9465	7.8766	7.9595	7.8738	7.9507	7.8798	7.9774	**7.48**
**H4**	7.7162	7.8884	7.9355	7.9538	8.0186	7.9507	8.0505	7.9468	8.0367	7.9560	8.0725	7.7159	7.8881	7.9349	7.9541	8.0179	7.9502	8.0499	7.9462	8.0360	7.9559	8.0717	**7.67**
**H5**	7.6107	7.7520	7.7984	7.8115	7.8833	7.8084	7.8878	7.8046	7.8779	7.8136	7.9071	7.6102	7.7516	7.7974	7.8115	7.8825	7.8077	7.8868	7.8037	7.8769	7.8132	7.9060	**7.48**
**H6**	8.0215	8.1222	8.2076	8.1606	8.3055	8.1578	8.3056	8.1543	8.2863	8.1625	8.3345	8.0213	8.1237	8.2072	8.1620	8.3067	8.1586	8.3060	8.1549	8.2870	8.1636	8.3347	**7.89**
**H8**	4.4592	4.6036	4.6495	4.6504	4.7507	4.6477	4.7755	4.6443	4.7631	4.6522	4.7884	4.4564	4.6030	4.6478	4.6505	4.7502	4.6471	4.7746	4.6435	4.7625	4.6520	4.7873	**5.06**
**H(9A-9C)**	1.1460	1.1223	1.1210	1.1055	1.0744	1.1069	1.1117	1.1074	1.0961	1.1037	1.1103	1.1488	1.1255	1.1239	1.1090	1.0778	1.1096	1.1147	1.1099	1.0985	1.1069	1.1131	**1.46**
Nuclei	**PBE**											**PBE0**											
**C1**	136.7513	137.2600	138.3042	137.4553	138.9532	137.4372	138.9785	137.4302	138.9681	137.4910	139.0764	138.2012	138.5127	139.5094	138.6963	140.0322	138.6779	139.9544	138.6618	139.9361	138.7170	139.9691	**132.3**
**C2**	132.5052	132.3390	132.8232	132.0845	132.7741	132.0967	133.0604	132.1196	132.9792	132.0911	133.1068	135.2288	134.9792	135.4801	134.7726	135.3757	134.7796	135.7523	134.7887	135.6181	134.7692	135.7329	**129.3**
**C3**	132.6372	133.6617	134.2990	133.9470	134.9104	133.9283	135.0679	133.9204	135.0016	133.9819	135.1931	134.0341	134.9259	135.5415	135.2413	136.0740	135.2185	136.2738	135.1973	136.1631	135.2659	136.3262	**128.9**
**C4**	136.5241	138.5195	139.1607	139.2613	140.0826	139.2167	140.8704	139.1824	140.6447	139.3206	141.1075	138.4771	140.3412	140.9834	141.1017	141.8289	141.0536	142.7285	141.0069	142.4336	141.1498	142.8949	**135.2**
**C5**	131.3872	132.9060	133.5967	133.5206	134.6386	133.4815	134.7143	133.4528	134.6301	133.5751	134.8802	132.6595	134.0946	134.7788	134.7574	135.7539	134.7131	135.8862	134.6704	135.7518	134.8024	135.9842	**128.9**
**C6**	131.2741	132.8032	133.8222	133.4888	135.1651	133.4447	135.4864	133.4110	135.2973	133.5482	135.7907	133.5401	135.0434	136.0586	135.7906	137.3685	135.7407	137.8195	135.6927	137.5601	135.8404	138.0490	**129.3**
**C7**	211.6949	214.9651	216.7136	216.2158	217.3470	216.1408	221.8839	216.0762	221.0911	216.3045	222.5420	210.2895	213.8793	215.4720	215.3444	216.0413	215.2559	221.1317	215.1685	220.1251	215.4304	221.7474	**198.1**
**C8**	58.1047	58.7464	59.4234	58.9045	59.7953	58.8904	60.5456	58.8873	60.3982	58.9359	60.6861	55.1079	55.7049	56.2148	55.9298	56.5205	55.9112	57.2875	55.8943	57.0906	55.9503	57.3145	**51.9**
**C9**	24.1834	24.4833	24.4146	24.6524	24.8524	24.6368	24.6942	24.6323	24.6253	24.6852	24.6857	23.2759	23.4659	23.4095	23.6560	23.7694	23.6375	23.6511	23.6211	23.5509	23.6767	23.5581	**16.6**
**H2**	8.5961	8.5158	8.5082	8.4631	8.4710	8.4666	8.4492	8.4706	8.4531	8.4605	8.4521	8.7988	8.7070	8.7031	8.6539	8.6690	8.6571	8.6484	8.6603	8.6491	8.6505	8.6496	**7.89**
**H3**	7.6377	7.7646	7.8111	7.8084	7.8799	7.8062	7.8916	7.8043	7.8840	7.8112	7.9086	7.7628	7.8923	7.9398	7.9398	8.0089	7.9372	8.0271	7.9345	8.0164	7.9419	8.0419	**7.48**
**H4**	7.6318	7.8172	7.8683	7.8895	7.9601	7.8856	7.9947	7.8821	7.9811	7.8939	8.0159	7.7895	7.9765	8.0297	8.0517	8.1214	8.0474	8.1645	8.0431	8.1471	8.0553	8.1839	**7.67**
**H5**	7.5411	7.6979	7.7432	7.7638	7.8399	7.7601	7.8440	7.7569	7.8353	7.7679	7.8634	7.6611	7.8246	7.8717	7.8961	7.9707	7.7920	7.9820	7.8878	7.9699	7.8996	8.0001	**7.48**
**H6**	8.0408	8.1972	8.2882	8.2644	8.4162	8.2607	8.4379	8.2575	8.4255	8.2686	8.4745	8.1829	8.3447	8.4401	8.4192	8.5776	8.4149	8.6050	8.4105	8.5869	8.4229	8.6391	**7.89**
**H8**	4.4695	4.6530	4.7200	4.7291	4.8300	4.7248	4.8763	4.7209	4.8663	4.7338	4.8854	4.3100	4.4948	4.5634	4.5735	4.6745	4.5689	4.7259	4.5642	4.7097	4.5775	4.7308	**5.06**
**H(9A-9C)**	1.2601	1.2686	1.2634	1.2624	1.2513	1.2631	1.2953	1.2642	1.2897	1.2625	1.3078	1.2475	1.2543	1.2384	1.2501	1.2269	1.2505	1.2691	1.2508	1.2605	1.2495	1.2761	**1.46**

[a] methyl protons shifts are averaged.
[b] according to [1]

Table S1. Cont. III

Nuclei	TPSS											VSXC											Exp. [b]
	Gas	$CHCl_3$ (PCM)	$CHCl_3$ (SMD)	DMSO (PCM)	DMSO (SMD)	CH_3OH (PCM)	CH_3OH (SMD)	C_2H_5OH (PCM)	C_2H_5OH (SMD)	H_2O (PCM)	H_2O (SMD)	Gas	$CHCl_3$ (PCM)	$CHCl_3$ (SMD)	DMSO (PCM)	DMSO (SMD)	CH_3OH (PCM)	CH_3OH (SMD)	C_2H_5OH (PCM)	C_2H_5OH (SMD)	H_2O (PCM)	H_2O (SMD)	
**C1**	133.8442	134.0013	135.0500	134.1846	135.5449	134.1779	135.4490	134.1829	135.2870	134.2018	135.4839	131.8630	131.8201	132.5994	131.8494	133.0134	131.8494	132.5912	131.8476	132.6286	131.8510	132.6464	**132.3**
**C2**	129.7258	129.4226	129.9822	129.2635	129.8220	129.2795	130.1188	129.3068	130.4474	129.2592	130.1285	128.2191	127.9849	128.4680	127.8363	128.3483	127.8481	128.6974	127.8579	128.6064	127.8268	128.7563	**129.3**
**C3**	129.0139	129.8376	130.5298	130.1956	131.0042	130.1821	131.1596	130.1799	131.3140	130.2187	131.2465	127.6097	128.5474	129.1598	128.9497	129.7362	128.9297	129.8926	128.9073	129.8152	128.9699	130.0119	**128.9**
**C4**	133.1384	134.9400	135.6481	135.7572	136.4277	135.7174	137.2675	135.6885	136.7813	135.8050	137.4731	131.6101	133.6166	134.2496	134.5120	135.1815	134.4637	136.0626	134.4128	135.8257	134.5589	136.3174	**135.2**
**C5**	127.8274	129.1132	129.8553	129.7810	130.7328	129.7481	130.8124	129.7262	130.8637	129.8226	130.9365	126.2933	127.6959	128.3507	128.4083	129.3093	128.3687	129.4309	128.3266	129.3303	128.4474	129.5846	**128.9**
**C6**	128.3912	129.7722	130.8179	130.5395	132.0240	130.4999	132.4127	130.4715	131.6034	130.5875	132.6809	128.2563	129.7579	130.6719	130.5283	131.9028	130.4849	132.2921	130.4394	132.1502	130.5710	132.6015	**129.3**
**C7**	206.9645	209.9849	211.6685	211.2862	212.1700	211.2187	216.7444	211.1615	211.6882	211.3603	217.3713	201.6393	204.3358	205.5812	205.4655	206.0483	205.4042	209.9511	205.3399	209.1835	205.5248	210.5908	**198.1**
**C8**	57.7397	58.1997	58.8346	58.4262	59.1327	58.4186	59.8509	58.4224	60.0947	58.4442	59.9257	61.1191	61.5554	61.9503	61.7409	62.3010	61.7330	62.5766	61.7230	62.5028	61.7500	62.6167	**51.9**
**C9**	24.4235	24.6244	24.6726	24.8798	25.0225	24.8686	24.9032	24.8692	26.7931	24.9012	24.8649	29.6427	30.3368	30.6408	30.7648	31.0815	30.7403	31.4702	30.7138	31.2833	30.7897	31.5540	**16.6**
**H2**	8.5827	8.5037	8.5081	8.4600	8.4712	8.4628	8.4482	8.4662	8.5759	8.4577	8.4476	8.6673	8.5980	8.5978	8.5599	8.5645	8.5623	8.5696	8.5648	8.5792	8.5576	8.5937	**7.89**
**H3**	7.5564	7.6830	7.7356	7.7341	7.8063	7.7314	7.8188	7.7293	7.8445	7.7370	7.8327	7.6100	7.7387	7.7798	7.7870	7.8500	7.7845	7.8657	7.7820	7.8605	7.7893	7.8910	**7.48**
**H4**	7.5750	7.7603	7.8180	7.8403	7.9119	7.8359	7.9501	7.8321	7.9466	7.8448	7.9686	7.6333	7.8181	7.8627	7.8943	7.9549	7.8901	7.9975	7.8860	7.9845	7.8982	8.0266	**7.67**
**H5**	7.4652	7.6220	7.6733	7.6951	7.7710	7.6910	7.7759	7.6875	7.8031	7.6993	7.7916	7.5455	7.7080	7.7530	7.7799	7.8482	7.7759	7.8668	7.7719	7.8576	7.7837	7.8942	**7.48**
**H6**	7.9314	8.0967	8.1960	8.1773	8.3328	8.1727	8.3612	8.1687	8.2807	8.1819	8.3967	8.1437	8.2938	8.3904	8.3715	8.5062	8.3668	8.5790	8.3623	8.5555	8.3759	8.6251	**7.89**
**H8**	4.3217	4.5075	4.5827	4.5961	4.7010	4.5910	4.7496	4.5865	4.7253	4.6012	4.7564	4.3672	4.5380	4.5649	4.6109	4.6585	4.6069	4.7531	4.6030	4.7434	4.6146	4.8027	**5.06**
**H(9A-9C)**	1.2656	1.2748	1.2692	1.2756	1.2597	1.2759	1.3039	1.2766	1.4402	1.2758	1.3120	1.4571	1.4976	1.4877	1.5067	1.4874	1.5064	1.5441	1.5062	1.5359	1.5070	1.5702	**1.46**

Nuclei	wB97											wB97X											
**C1**	138.9705	139.0197	140.0068	139.1625	140.4868	139.1535	140.1516	139.1436	140.2312	139.1714	140.2229	140.2155	140.3084	141.3213	140.4667	141.8248	140.4591	141.5429	140.4506	141.5522	140.4748	141.5816	**132.3**
**C2**	136.6575	136.2946	136.8147	136.0943	136.7084	136.1044	136.9927	136.1139	136.9108	136.0853	137.0272	137.2285	136.9071	137.4167	136.7108	137.3120	136.7236	137.6435	136.7355	137.5011	136.7000	137.6605	**129.3**
**C3**	134.5888	135.2845	135.8971	135.5728	136.3757	135.5553	136.4057	135.5371	136.3680	135.5895	136.4973	135.3963	136.1180	136.7238	136.4125	137.2120	136.3974	137.2926	136.3814	137.1922	136.4277	137.3720	**128.9**
**C4**	139.4400	141.1178	141.7949	141.8441	142.6123	141.8013	143.3418	141.7574	143.1161	141.8847	143.5445	140.0391	141.7475	142.4062	142.4828	143.2269	142.4422	144.0152	142.4003	143.7276	142.5223	144.2101	**135.2**
**C5**	133.1924	134.4554	135.1411	135.1011	136.0762	135.0613	136.0438	135.0208	135.9804	135.1389	136.1835	133.9880	135.2740	135.9624	135.9272	136.9054	135.8897	136.9280	135.8512	136.8025	135.9638	137.0543	**128.9**
**C6**	134.9408	136.3657	137.3591	137.1218	138.6749	137.0741	139.0346	137.0258	138.8231	137.1675	139.3267	135.4709	136.9142	137.8818	137.6807	139.2069	137.6354	139.6104	137.5894	139.3375	137.7249	139.8960	**129.3**
**C7**	209.7234	213.4973	215.0558	215.1119	215.5978	215.0217	220.8345	214.9294	219.8564	215.1965	221.5673	211.5808	215.3541	216.9529	216.9591	217.5146	216.8716	222.8061	216.7816	221.7555	217.0417	223.4911	**198.1**
**C8**	54.1575	54.7096	55.1072	54.9796	55.3878	54.9629	56.0014	54.9454	55.8774	54.9958	56.0883	55.0869	55.6353	56.0384	55.9282	56.3257	55.9134	57.0159	55.8973	56.8122	55.9432	57.1003	**51.9**
**C9**	23.0304	23.0822	23.0706	23.2375	23.4247	23.2247	23.1200	23.2115	23.0955	23.2504	23.0824	23.2890	23.3915	23.3054	23.5633	23.6703	23.5527	23.4270	23.5413	23.3322	23.5749	23.3699	**16.6**
**H2**	8.8754	8.7780	8.7788	8.7239	8.7523	8.7268	8.7205	8.7296	8.7227	8.7214	8.7220	8.8561	8.7629	8.7593	8.7093	8.7318	8.7124	8.7030	8.7155	8.7036	8.7066	8.7042	**7.89**
**H3**	7.8286	7.9509	7.9985	7.9956	8.0683	7.9931	8.0773	7.9905	8.0702	7.9980	8.0945	7.8057	7.9299	7.9757	7.9765	8.0468	7.9741	8.0584	7.9717	8.0484	7.9788	8.0736	**7.48**
**H4**	7.8892	8.0683	8.1232	8.1395	8.2158	8.1355	8.2505	8.1314	8.2363	8.1433	8.2724	7.8598	8.0416	8.0947	8.1150	8.1885	8.1111	8.2255	8.1071	8.2086	8.1188	8.2454	**7.67**
**H5**	7.7387	7.9005	7.9484	7.9711	8.0490	7.9670	8.0543	7.9627	8.0452	7.9750	8.0756	7.7192	7.8817	7.9296	7.9546	8.0313	7.9506	8.0382	7.9465	8.0264	7.9586	8.0580	**7.48**
**H6**	8.2757	8.4306	8.5306	8.5066	8.6767	8.5020	8.6891	8.4972	8.6724	8.5111	8.7252	8.2414	8.4011	8.4930	8.4761	8.6406	8.4718	8.6590	8.4674	8.6404	8.4805	8.6924	**7.89**
**H8**	4.1547	4.3318	4.4162	4.4109	4.5350	4.4064	4.5637	4.4018	4.5507	4.4152	4.5680	4.1869	4.3690	4.4494	4.4475	4.5678	4.4433	4.6053	4.4389	4.5906	4.4518	4.6075	**5.06**
**H(9A-9C)**	1.3053	1.3152	1.2873	1.3135	1.2822	1.3134	1.3112	1.3133	1.3047	1.3137	1.3214	1.2881	1.2965	1.2698	1.2976	1.2638	1.2977	1.2978	1.2977	1.2881	1.2977	1.3076	**1.46**

Nuclei	X3LYP											Exp. [b]
**C1**	140.8852	141.1026	142.1042	141.2902	142.6957	141.2794	142.4537	141.2661	142.4859	141.3047	142.4969	**132.3**
**C2**	136.4292	136.1319	136.6277	135.9444	136.4839	135.9591	136.8407	135.9709	136.7578	135.9342	136.8544	**129.3**
**C3**	135.2808	136.1196	136.7103	136.4511	137.2000	136.4361	137.3155	136.4180	137.2689	136.4686	137.3973	**128.9**
**C4**	139.7313	141.5961	142.2127	142.4020	143.0129	142.3602	143.8816	142.3148	143.6477	142.4447	144.0869	**135.2**
**C5**	133.8833	135.2574	135.9107	135.9339	136.8450	135.8976	136.8922	135.8581	136.8239	135.9717	137.0217	**128.9**
**C6**	134.8463	136.3615	137.3970	137.1614	138.7037	137.1175	139.1228	137.0699	138.9223	137.2068	139.4046	**129.3**
**C7**	213.1079	216.8393	218.4861	218.4173	219.1506	218.3302	224.2621	218.2396	223.2992	218.5029	224.9437	**198.1**
**C8**	57.4488	57.9664	58.5355	58.1925	58.8685	58.1814	59.5343	58.1673	59.3900	58.2070	59.5961	**51.9**
**C9**	23.9811	24.1449	24.1040	24.3733	24.4676	24.3613	24.2637	24.3462	24.2188	24.3886	24.1893	**16.6**
**H2**	8.7269	8.6390	8.6403	8.5912	8.6001	8.5945	8.5847	8.5973	8.5894	8.5880	8.5879	**7.89**
**H3**	7.6938	7.8179	7.8651	7.8679	7.9316	7.8654	7.9447	7.8624	7.9375	7.8702	7.9605	**7.48**
**H4**	7.7213	7.9055	7.9580	7.9845	8.0482	7.9803	8.0880	7.9756	8.0736	7.9884	8.1086	**7.67**
**H5**	7.5928	7.7494	7.7957	7.8227	7.8912	7.8187	7.8982	7.8142	7.8896	7.8264	7.9174	**7.48**
**H6**	8.0788	8.2461	8.3451	8.3276	8.4809	8.3232	8.5087	8.3182	8.4946	8.3318	8.5441	**7.89**
**H8**	4.3086	4.5009	4.5730	4.5903	4.6894	4.5853	4.7409	4.5798	4.7281	4.5950	4.7494	**5.06**
**H(9A-9C)**	1.2676	1.2729	1.2646	1.2724	1.2531	1.2728	1.2921	1.2727	1.2870	1.2720	1.3003	**1.46**

[a] methyl protons shifts are averaged

[b] according to [1]

Table S2. Theoretical chemical shifts (in ppm) of *(R,S)*-4-trifluoromethylmethcathinone calculated with HF and selected functionals combined with 6-311++G** basis set[a]

Nuclei	R											S											Exp. [b]
	B3LYP																						
	Gas	$CHCl_3$ (PCM)	$CHCl_3$ (SMD)	DMSO (PCM)	DMSO (SMD)	CH_3OH (PCM)	CH_3OH (SMD)	C_2H_5OH (PCM)	C_2H_5OH (SMD)	H_2O (PCM)	H_2O (SMD)	Gas	$CHCl_3$ (PCM)	$CHCl_3$ (SMD)	DMSO (PCM)	DMSO (SMD)	CH_3OH (PCM)	CH_3OH (SMD)	C_2H_5OH (PCM)	C_2H_5OH (SMD)	H_2O (PCM)	H_2O (SMD)	
**C1**	146.0958	146.2278	147.5491	147.4218	150.8969	147.4088	149.3403	147.4036	150.1234	147.4439	149.4787	143.7395	145.7960	147.3774	146.9694	148.8010	146.9053	149.1986	146.8200	149.2962	147.0435	149.3862	**137.3**
**C2**	136.8518	136.8850	137.3336	136.6336	136.9960	136.6497	137.5933	136.6663	137.3168	136.6170	137.2877	136.2761	136.6236	137.4407	136.5876	137.0038	136.6803	137.6513	136.6725	137.5525	136.5896	137.4512	**136.5**
**C3**	132.8796	133.6062	134.4412	133.8049	134.5903	133.7929	135.2838	133.7797	134.7280	133.8132	134.6689	132.9055	133.5539	135.2064	133.6654	134.4225	133.9503	135.0151	133.9312	134.9607	133.6718	134.6523	**130.8**
**C4**	142.3544	142.0621	142.1229	141.9755	141.5681	141.9652	142.4924	141.9541	142.2121	141.9828	142.6772	142.6200	142.0044	141.9670	141.8279	141.7753	141.8344	142.2976	141.8412	142.1390	141.8214	142.4337	**127.3**
**C5**	131.4105	132.7894	133.5620	133.4888	134.9076	133.4388	134.4074	133.3892	134.8464	133.5368	135.3018	131.7048	132.9037	132.9075	133.5558	135.0195	133.2113	134.4192	133.1803	134.3270	133.5927	135.0615	**130.8**
**C6**	133.4691	135.4594	136.3625	136.1167	137.0755	136.0603	137.4922	136.0018	137.2782	136.1648	138.1149	133.8854	135.8776	136.3245	136.7886	138.2473	136.6061	138.3767	136.5513	138.1476	136.8408	138.8067	**136.5**
**C7**	204.5842	209.4312	211.3479	213.3234	215.9221	213.1217	220.4917	212.9260	220.0350	213.5251	221.2874	209.0453	213.6104	215.6212	216.0451	217.3393	215.9749	222.2187	215.8303	221.2371	216.1784	222.8007	**196.6**
**C8**	65.8330	66.8466	67.5195	67.3198	68.5992	67.3093	68.8908	67.2977	68.9990	67.3265	69.5579	60.1665	62.3074	63.0745	63.3374	64.1080	63.2096	65.0826	63.1315	64.9303	63.4088	65.3533	**61**
**C9**	22.6569	23.0280	23.1991	23.0523	23.1669	23.0342	23.3493	23.0146	23.2029	23.0644	23.4581	19.4003	19.7115	19.8109	19.9242	20.0244	19.8800	20.2392	19.8634	20.1865	19.9367	20.5768	**15.8**
**C4``**	135.4005	136.6305	137.6446	137.0660	138.4974	137.0394	138.2736	137.0127	138.3038	137.0896	138.3640	135.3118	136.6140	137.4461	137.0437	138.4421	137.0188	138.3427	136.9898	138.3026	137.0685	138.3762	**123.2**
**C8`**	36.5261	37.3149	37.5781	37.7206	38.0154	37.6956	38.1934	37.6683	38.1390	37.7409	38.6069	32.0616	32.1937	32.3459	32.2980	32.5680	32.2988	32.5977	32.2892	32.5480	32.3053	32.6500	**31.8**
**H2**	8.6052	8.6146	8.5856	8.4711	8.3202	8.4741	8.4332	8.4764	8.3584	8.4672	8.4261	8.7001	8.5167	8.4998	8.4210	8.4000	8.4331	8.3692	8.4400	8.3608	8.4145	8.3618	**8.24**
**H3**	7.9718	8.0737	8.1407	8.0983	8.1938	8.0967	8.2022	8.0949	8.1979	8.0997	8.2362	7.9848	8.0780	8.1283	8.1112	8.2056	8.1027	8.2137	8.1011	8.2041	8.1126	8.2318	**7.92**
**H5**	7.8793	8.0287	8.0888	8.0863	8.1664	8.0819	8.1917	8.0773	8.1666	8.0902	8.2052	7.8774	8.0030	8.0728	8.0579	8.1628	8.0603	8.1579	8.0567	8.1467	8.0608	8.1834	**7.92**
**H6**	8.1154	8.3540	8.4200	8.4248	8.4907	8.4167	8.5647	8.4084	8.5175	8.4319	8.6196	7.9490	8.1142	8.1606	8.1938	8.3447	8.1855	8.3213	8.1818	8.3005	8.1973	8.3707	**8.24**
**H8**	4.4118	4.5372	4.5645	4.6145	4.6975	4.6051	4.7529	4.5960	4.7108	4.6240	4.7854	4.5748	4.7197	4.7805	4.7820	4.9106	4.7826	4.9151	4.7794	4.8977	4.7847	4.9481	**5.17**
**H(9A-9C)**	1.3239	1.3479	1.3163	1.2910	1.2079	1.2933	1.2482	1.2951	1.2224	1.2881	1.2659	1.4062	1.4238	1.3898	1.4403	1.4015	1.4393	1.4116	1.4383	1.4010	1.4412	1.4350	**1.59**
**H(8`A-8`C)**	2.5446	2.4696	2.4496	2.4722	2.4554	2.4730	2.4371	2.4740	2.4437	2.4714	2.4186	2.9695	2.7936	2.7376	2.6984	2.6624	2.7085	2.5658	2.7149	2.5721	2.6928	2.5380	**2.8**
Nuclei	**B3P86**																						
**C1**	143.5720	143.7526	145.1950	144.9447	146.9265	144.9906	146.4144	145.0104	146.3991	144.9405	146.7483	141.6240	143.3605	144.8846	144.5601	146.0812	144.5129	146.7846	144.4437	147.1002	144.6486	146.9466	**137.3**
**C2**	135.8551	135.9006	136.4528	135.8101	136.2449	135.8453	136.4673	135.8577	136.4585	135.8197	136.4479	135.3794	135.6635	136.5493	135.6941	136.1456	135.7119	136.8061	135.7083	136.8259	135.7215	136.6451	**136.5**
**C3**	131.8409	132.5775	133.4990	132.8437	133.8578	132.8532	133.9416	132.8401	133.9682	132.8781	134.0354	131.8583	132.5262	134.2026	132.7017	133.5319	132.7150	134.0763	132.7061	134.1347	132.7332	133.7972	**130.8**
**C4**	140.7715	140.5654	140.6963	140.5344	140.4704	140.5402	141.2965	140.5255	141.2687	140.5742	141.4100	140.9951	140.5161	140.5894	140.4288	140.4812	140.4529	140.9976	140.4553	140.9280	140.4499	141.1352	**127.3**
**C5**	130.4888	131.8254	132.5909	132.4914	133.7124	132.4665	133.8940	132.4203	133.9397	132.5597	134.0753	130.6891	131.8834	132.0165	132.5559	134.0699	132.5370	133.4953	132.4959	133.5095	132.6175	134.0913	**130.8**
**C6**	132.5391	134.4862	135.4902	135.2618	136.7598	135.2167	137.0919	135.1503	137.0432	135.3493	137.4008	132.9247	134.9130	135.4670	135.8477	137.5078	135.8142	137.5199	135.7576	137.3824	135.9222	137.9901	**136.5**
**C7**	202.7303	207.2866	209.1382	210.5354	211.8480	210.4208	217.0332	210.2835	215.9685	210.6864	218.0446	206.6078	211.0529	213.0049	213.4903	214.5194	213.3713	219.4874	213.2296	218.6808	213.6460	219.9852	**196.6**
**C8**	64.2834	65.3255	66.0463	65.9429	66.8854	65.9531	67.3948	65.9394	67.2557	65.9748	68.1720	58.8172	60.5420	61.2735	61.5378	62.2615	61.4952	63.2346	61.4312	63.2428	61.6220	63.5164	**61**
**C9**	22.4523	22.7713	23.0334	22.9264	23.2633	22.9308	23.1789	22.9135	23.2319	22.9666	23.3202	19.2595	19.6026	19.7315	19.8572	20.0569	19.8676	20.2141	19.8558	20.2862	19.8911	20.6180	**15.8**
**C4``**	132.3434	133.4327	134.4391	133.8453	135.2457	133.8445	135.0789	133.8215	135.1512	133.8895	135.1585	132.2800	133.4280	134.2350	133.8368	135.1804	133.8349	135.0820	133.8108	135.1608	133.8823	135.1227	**123.2**
**C8`**	35.9329	36.6681	36.9118	37.0081	37.3997	37.0081	37.6077	36.9863	37.6627	37.0524	38.0829	31.2144	31.4571	31.6577	31.6149	31.9357	31.6278	31.9411	31.6185	31.9663	31.6465	32.0080	**31.8**
**H2**	8.6821	8.6848	8.6524	8.5563	8.5205	8.5567	8.5299	8.5556	8.5431	8.5575	8.5179	8.7368	8.5872	8.5803	8.4935	8.4975	8.5006	8.4425	8.5060	8.4314	8.4881	8.4397	**8.24**
**H3**	8.0231	8.1280	8.1963	8.1538	8.2563	8.1532	8.2744	8.1508	8.2729	8.1556	8.2937	8.0339	8.1321	8.1879	8.1663	8.2642	8.1659	8.2748	8.1636	8.2710	8.1679	8.2911	**7.92**
**H5**	7.9280	8.0809	8.1428	8.1404	8.2366	8.1369	8.2466	8.1316	8.2427	8.1450	8.2723	7.9210	8.0563	8.1308	8.1122	8.2236	8.1102	8.2186	8.1064	8.2131	8.1153	8.2431	**7.92**
**H6**	8.2040	8.4370	8.4993	8.4887	8.6145	8.4808	8.6497	8.4713	8.6493	8.4977	8.6830	8.0127	8.1939	8.2472	8.2747	8.4444	8.2719	8.4062	8.2671	8.3859	8.2785	8.4597	**8.24**
**H8**	4.4450	4.5701	4.5932	4.6175	4.6853	4.6098	4.7297	4.6009	4.7249	4.6265	4.7616	4.6258	4.7697	4.8328	4.8341	4.9626	4.8319	4.9687	4.8279	4.9542	4.8373	5.0038	**5.17**
**H(9A-9C)**	1.3433	1.3656	1.3330	1.3264	1.2873	1.3278	1.2945	1.3276	1.2953	1.3263	1.3059	1.4207	1.4365	1.4015	1.4494	1.4212	1.4500	1.4180	1.4488	1.4111	1.4503	1.4473	**1.59**
**H(8`A-8`C)**	2.5241	2.4423	2.4317	2.4510	2.4262	2.4546	2.4102	2.4563	2.4110	2.4488	2.3981	2.9048	2.7459	2.6925	2.6514	2.6165	2.6576	2.5126	2.6621	2.5239	2.6469	2.4886	**2.8**
Nuclei	**B3PW91**																						
**C1**	143.9680	145.2861	145.3030	145.3252	147.6021	145.3151	147.8816	145.3012	148.1122	145.3430	147.8471	141.9101	143.7628	145.7481	145.1440	147.2658	145.1036	147.4325	145.0195	147.4892	145.1993	147.5528	**137.3**
**C2**	135.9436	136.0575	136.4438	135.8229	136.5065	135.8446	136.6622	135.8598	136.5815	135.8054	136.6329	135.4997	135.7925	136.6812	135.8193	136.3557	135.9145	136.8678	135.9002	136.8027	135.8177	136.7097	**136.5**
**C3**	131.9691	132.7466	133.5595	132.9686	134.1950	132.9630	134.0521	132.9487	134.0196	132.9752	134.1507	131.9865	132.6536	134.3275	132.8146	133.6765	133.0543	134.1660	133.0184	134.1271	132.8187	133.9040	**130.8**
**C4**	140.7482	140.4245	140.6506	140.5439	140.8105	140.5398	141.0441	140.5257	140.8604	140.5490	141.2257	140.9748	140.4917	140.4641	140.3675	140.3322	140.3821	140.8723	140.3857	140.7350	140.3606	141.0332	**127.3**
**C5**	130.6038	131.9409	132.7115	132.6639	134.3345	132.6241	134.0533	132.5742	133.9549	132.7039	134.2242	130.8127	132.0709	132.0575	132.7307	134.1535	132.4250	133.6524	132.4074	133.5700	132.7644	134.2519	**130.8**
**C6**	132.4390	134.1915	135.3335	135.1221	136.9534	135.0729	136.5202	135.0147	136.2969	135.1700	136.9657	132.8861	134.8788	135.2846	135.7770	137.2374	135.6212	137.3589	135.5782	137.1594	135.8226	137.8301	**136.5**
**C7**	202.9996	208.5381	209.4049	211.3573	212.8967	211.1744	218.8219	210.9795	217.9146	211.5349	219.4046	206.7552	211.3682	213.4772	213.8868	215.1902	213.8014	220.0464	213.6466	219.0113	214.0138	220.5442	**196.6**
**C8**	64.0681	65.3873	65.6926	65.6411	66.8256	65.6323	67.4513	65.6169	67.3379	65.6527	68.1075	58.7625	60.5900	61.3971	61.6676	62.5814	61.5804	63.4137	61.5102	63.2651	61.7238	63.6836	**61**
**C9**	22.1615	22.4308	22.6684	22.5791	23.1523	22.5677	22.8212	22.5480	22.7617	22.5911	22.9949	18.9729	19.3137	19.4310	19.5605	19.7581	19.5429	19.8692	19.5310	19.8398	19.5655	20.2492	**15.8**
**C4``**	132.5359	133.6842	134.5991	134.0738	135.7815	134.0572	135.2910	134.0317	135.2703	134.0917	135.3721	132.4868	133.6715	134.4351	134.0758	135.4233	134.0667	135.3131	134.0402	135.2933	134.0939	135.3553	**123.2**
**C8`**	35.8894	36.6726	36.8915	37.0969	37.7509	37.0774	37.5509	37.0465	37.4996	37.1156	37.9989	31.2628	31.5298	31.6068	31.6487	31.8819	31.6491	31.9461	31.6393	31.9031	31.6544	32.0201	**31.8**
**H2**	8.6365	8.5362	8.6282	8.5114	8.4952	8.5151	8.4216	8.5182	8.4029	8.5076	8.4374	8.7058	8.5482	8.5258	8.4427	8.4052	8.4530	8.3930	8.4594	8.3897	8.4377	8.3904	**8.24**
**H3**	8.0172	8.1150	8.1918	8.1483	8.2689	8.1473	8.2619	8.1458	8.2532	8.1494	8.2814	8.0305	8.1268	8.1807	8.1606	8.2559	8.1544	8.2688	8.1534	8.2613	8.1619	8.2844	**7.92**
**H5**	7.9208	8.0597	8.1377	8.1348	8.2465	8.1309	8.2353	8.1266	8.2249	8.1385	8.2621	7.9218	8.0540	8.1231	8.1109	8.2138	8.1114	8.2148	8.1081	8.2052	8.1136	8.2390	**7.92**
**H6**	8.1761	8.3482	8.4880	8.4820	8.6315	8.4751	8.5890	8.4674	8.5650	8.4882	8.6385	7.9955	8.1748	8.2163	8.2504	8.3924	8.2408	8.3765	8.2379	8.3583	8.2540	8.4298	**8.24**
**H8**	4.4236	4.4987	4.5816	4.6259	4.7307	4.6179	4.7435	4.6092	4.7237	4.6336	4.7789	4.5969	4.7399	4.8013	4.8026	4.9260	4.8017	4.9359	4.7985	4.9185	4.8056	4.9711	**5.17**
**H(9A-9C)**	1.3002	1.2899	1.2925	1.2687	1.2434	1.2713	1.2102	1.2736	1.1993	1.2663	1.2309	1.3803	1.3980	1.3654	1.4121	1.3646	1.4111	1.3828	1.4106	1.3759	1.4131	1.4087	**1.59**
**H(8`A-8`C)**	2.4962	2.4531	2.3965	2.4192	2.4050	2.4208	2.3919	2.4225	2.3951	2.4181	2.3742	2.8737	2.7158	2.6490	2.6190	2.5669	2.6257	2.4850	2.6309	2.4968	2.6145	2.4595	**2.8**
Nuclei	**B98**																						
**C1**	144.6817	144.7735	146.1060	146.0230	149.6033	146.0491	147.3430	146.0726	147.2536	146.0020	147.6851	142.6920	144.7148	146.4355	145.7596	147.5403	145.7132	147.9368	145.6463	148.0281	145.8144	148.0860	**137.3**
**C2**	135.6236	135.6313	136.1341	135.4960	135.9251	135.5317	136.1963	135.5637	136.1119	135.4642	136.1530	135.1384	135.4310	136.2726	135.4219	135.8823	135.5307	136.4802	135.5367	136.4127	135.4105	136.2879	**136.5**
**C3**	131.6876	132.3770	133.2050	132.6082	133.5093	132.6142	133.5992	132.6174	133.5457	132.6044	133.6544	131.6892	132.3271	134.0012	132.4781	133.2729	132.7411	133.8363	132.7295	133.8100	132.4740	133.5023	**130.8**
**C4**	141.6154	141.3650	141.4455	141.3519	141.0444	141.3518	142.0639	141.3495	141.9398	141.3547	142.1758	141.8162	141.2600	141.2835	141.1553	141.1883	141.1616	141.6483	141.1817	141.5148	141.1389	141.8200	**127.3**
**C5**	130.2933	131.5703	132.3049	132.2867	133.6565	132.2545	133.6606	132.2200	133.5991	132.3193	133.8638	130.4845	131.6904	131.6722	132.3505	133.8536	132.0417	133.2371	132.0316	133.1625	132.3744	133.8898	**130.8**
**C6**	132.4618	134.4024	135.3078	135.0616	136.0082	135.0166	136.9583	134.9685	136.8119	135.1059	137.2407	132.8518	134.7326	135.1926	135.6895	137.2053	135.5422	137.2615	135.5088	137.0579	135.7259	137.7069	**136.5**
**C7**	201.1408	205.8480	207.6423	209.5337	212.2712	209.3659	215.7906	209.1983	214.5722	209.6972	216.9081	205.0936	209.8308	211.8380	212.1990	213.4128	212.1126	218.4306	211.9741	217.4175	212.3241	218.9937	**196.6**
**C8**	65.7063	66.6959	67.3740	67.2385	68.5057	67.2520	68.7663	67.2626	68.5520	67.2290	69.4477	60.3977	62.2464	63.0208	63.2563	64.0443	63.1691	64.9719	63.1136	64.8262	63.3072	65.2120	**61**
**C9**	22.9529	23.2852	23.4567	23.3273	23.4646	23.3284	23.6497	23.3281	23.6182	23.3294	23.7704	19.8640	20.1672	20.2357	20.3492	20.5006	20.3405	20.6538	20.3434	20.6259	20.3453	20.9937	**15.8**
**C4``**	131.0991	132.1915	133.1530	132.6219	134.0294	132.6150	133.8547	132.6050	133.8345	132.6305	133.9201	131.0280	132.1772	133.0081	132.6015	133.9668	132.5942	133.8516	132.5833	133.8355	132.6114	133.8933	**123.2**
**C8`**	36.7268	37.4157	37.6378	37.8586	38.1851	37.8462	38.3927	37.8330	38.3535	37.8739	38.8230	32.0654	32.2381	32.3594	32.4189	32.7073	32.4289	32.7601	32.4320	32.7196	32.4158	32.8078	**31.8**
**H2**	8.6441	8.6517	8.6308	8.5158	8.3593	8.5168	8.5091	8.5178	8.5190	8.5148	8.4932	8.7072	8.5485	8.5236	8.4503	8.4357	8.4627	8.4090	8.4693	8.4055	8.4449	8.4001	**8.24**
**H3**	7.9998	8.1033	8.1757	8.1321	8.2308	8.1304	8.2526	8.1287	8.2470	8.1338	8.2697	8.0053	8.1066	8.1633	8.1390	8.2376	8.1316	8.2493	8.1304	8.2422	8.1408	8.2636	**7.92**
**H5**	7.9012	8.0534	8.1195	8.1160	8.2066	8.1113	8.2243	8.1068	8.2162	8.1206	8.2476	7.9013	8.0357	8.1096	8.0907	8.2008	8.0918	8.1947	8.0886	8.1858	8.0938	8.2207	**7.92**
**H6**	8.1436	8.3798	8.4472	8.4483	8.5180	8.4387	8.6085	8.4293	8.6011	8.4575	8.6467	7.9678	8.1483	8.1982	8.2259	8.3865	8.2182	8.3604	8.2151	8.3422	8.2296	8.4109	**8.24**
**H8**	4.3980	4.5187	4.5470	4.5861	4.6789	4.5767	4.6807	4.5674	4.6676	4.5951	4.7251	4.5626	4.7149	4.7791	4.7785	4.9063	4.7780	4.9132	4.7749	4.8986	4.7817	4.9452	**5.17**
**H(9A-9C)**	1.3259	1.3503	1.3226	1.2975	1.2035	1.2992	1.2865	1.3008	1.2849	1.2959	1.2883	1.4079	1.4295	1.3903	1.4348	1.3950	1.4357	1.4034	1.4358	1.3962	1.4355	1.4262	**1.59**
**H(8`A-8`C)**	2.5205	2.4463	2.4312	2.4500	2.4376	2.4522	2.4215	2.4545	2.4191	2.4483	2.4039	2.9109	2.7459	2.6877	2.6567	2.6175	2.6650	2.5240	2.6709	2.5362	2.6520	2.4964	**2.8**

[a] methyl protons shifts are averaged

[b] according to [2]

Table S2. Cont. I

Nuclei	R											S											Exp. [b]
	B971																						
	Gas	$CHCl_3$ (PCM)	$CHCl_3$ (SMD)	DMSO (PCM)	DMSO (SMD)	CH_3OH (PCM)	CH_3OH (SMD)	C_2H_5OH (PCM)	C_2H_5OH (SMD)	H_2O (PCM)	H_2O (SMD)	Gas	$CHCl_3$ (PCM)	$CHCl_3$ (SMD)	DMSO (PCM)	DMSO (SMD)	CH_3OH (PCM)	CH_3OH (SMD)	C_2H_5OH (PCM)	C_2H_5OH (SMD)	H_2O (PCM)	H_2O (SMD)	
**C1**	144.2075	144.3591	145.7057	145.6369	147.5389	145.6525	146.9067	145.6650	148.2176	145.6256	147.2579	142.2795	144.2596	145.9794	145.3233	147.0026	145.2669	147.4501	145.1910	147.5471	145.3890	147.6230	**137.3**
**C2**	135.3519	135.3692	135.8806	135.2827	135.7053	135.3069	135.9694	135.3296	135.9961	135.2643	135.9354	134.8588	135.1644	136.0033	135.1678	135.6057	135.2651	136.2203	135.1808	136.1514	135.1677	136.0256	**136.5**
**C3**	131.4605	132.1526	132.9895	132.4070	133.3759	132.4041	133.4077	132.4002	133.4151	132.4151	133.4842	131.4602	132.1101	133.7921	132.2743	133.0533	132.5150	133.6344	132.2705	133.6083	132.2817	133.2870	**130.8**
**C4**	141.2443	140.9936	141.0677	140.9785	140.8811	140.9707	141.6898	140.9638	141.3802	140.9931	141.7994	141.4387	140.8943	140.9208	140.7992	140.8381	140.7977	141.2840	140.8233	141.1513	140.7941	141.4602	**127.3**
**C5**	130.0816	131.3751	132.0828	132.0707	133.2556	132.0314	133.4231	131.9920	133.4091	132.1143	133.6009	130.2704	131.4714	131.4519	132.1340	133.6427	131.8341	132.9971	132.0717	132.9231	132.1689	133.6781	**130.8**
**C6**	132.2808	134.2107	135.1262	134.8825	136.3871	134.8299	136.7643	134.7775	135.9994	134.9387	137.0603	132.6477	134.5407	134.9971	135.5047	137.0565	135.3573	137.0861	135.4135	136.8802	135.5523	137.5353	**136.5**
**C7**	201.0262	205.7073	207.5076	209.2633	210.3800	209.0943	215.4862	208.9267	216.0420	209.4319	216.5760	204.9786	209.6701	211.6953	212.0392	213.2059	211.9402	218.1699	211.7602	217.1879	212.1755	218.7518	**196.6**
**C8**	65.6623	66.6670	67.3459	67.2469	68.2192	67.2521	68.6790	67.2540	68.6676	67.2449	69.3882	60.3706	62.1546	62.9077	63.1588	63.9241	63.0675	64.8290	63.0412	64.6869	63.2195	65.0725	**61**
**C9**	22.9552	23.2943	23.4615	23.3548	23.7034	23.3509	23.6587	23.3479	23.4768	23.3656	23.7827	19.8902	20.2028	20.2527	20.3861	20.5451	20.3727	20.6956	20.3825	20.6663	20.3927	21.0396	**15.8**
**C4``**	131.6640	132.7636	133.7194	133.2007	134.5849	133.1856	134.4193	133.1694	134.4087	133.2207	134.4927	131.5899	132.7409	133.5724	133.1730	134.5328	133.1581	134.4092	133.1396	134.3927	133.1941	134.4597	**123.2**
**C8`**	36.7351	37.4122	37.6112	37.8308	38.1771	37.8156	38.3455	37.8001	38.2754	37.8519	38.7827	32.0021	32.1890	32.2975	32.3668	32.6529	32.3705	32.6872	32.3642	32.6414	32.3738	32.7349	**31.8**
**H2**	8.6491	8.6497	8.6282	8.5164	8.4883	8.5169	8.5141	8.5176	8.4221	8.5164	8.4969	8.7055	8.5528	8.5254	8.4536	8.4437	8.4660	8.4133	8.4663	8.4090	8.4484	8.4025	**8.24**
**H3**	7.9994	8.1013	8.1731	8.1301	8.2318	8.1282	8.2495	8.1263	8.2380	8.1323	8.2660	8.0056	8.1052	8.1620	8.1377	8.2349	8.1309	8.2462	8.1342	8.2392	8.1398	8.2599	**7.92**
**H5**	7.9003	8.0509	8.1166	8.1137	8.2102	8.1087	8.2230	8.1039	8.2122	8.1187	8.2454	7.9011	8.0338	8.1080	8.0891	8.1986	8.0906	8.1928	8.0828	8.1841	8.0925	8.2176	**7.92**
**H6**	8.1485	8.3825	8.4473	8.4436	8.5681	8.4342	8.6089	8.4250	8.5633	8.4532	8.6430	7.9689	8.1514	8.2007	8.2287	8.3914	8.2215	8.3646	8.2213	8.3462	8.2326	8.4141	**8.24**
**H8**	4.3968	4.5154	4.5426	4.5752	4.6358	4.5654	4.6770	4.556	4.7126	4.5852	4.7102	4.5661	4.7165	4.7811	4.7819	4.9077	4.7813	4.9154	4.7752	4.9015	4.7855	4.9468	**5.17**
**H(9A-9C)**	1.3297	1.3507	1.3232	1.3023	1.2710	1.3037	1.2883	1.3052	1.2216	1.3012	1.2929	1.4115	1.4317	1.3906	1.4369	1.3996	1.4377	1.4056	1.4361	1.3982	1.4379	1.4284	**1.59**
**H(8`A-8`C)**	2.5158	2.4418	2.4283	2.4478	2.4235	2.4500	2.4132	2.4522	2.4135	2.4461	2.3968	2.9000	2.7383	2.6806	2.6506	2.6110	2.6584	2.5169	2.6611	2.5297	2.6463	2.4890	**2.8**
Nuclei	B972																						
**C1**	141.3855	142.3892	142.6458	144.0881	146.1903	144.0786	145.1463	144.0602	145.6156	144.0857	145.1997	139.2872	141.1596	143.1121	142.6886	144.9073	142.6729	144.8268	142.5969	144.8243	142.7317	144.9164	**137.3**
**C2**	133.3133	133.3116	133.6241	133.2395	133.6732	133.2458	133.8190	133.2490	133.7363	133.2290	133.8277	132.8890	133.1052	133.9686	133.1724	133.6384	133.2749	134.0936	133.2673	134.0359	133.1689	133.9361	**136.5**
**C3**	129.3416	130.0366	130.7900	130.3404	131.2218	130.3330	130.9522	130.3216	130.9365	130.3423	131.0885	129.3427	129.9137	131.6207	130.1127	130.9233	130.4156	131.3943	130.3947	131.3549	130.1163	131.1310	**130.8**
**C4**	138.0491	137.7117	137.9532	137.6000	137.4100	137.5910	138.2767	137.5815	138.0382	137.6058	138.4388	138.2617	137.7042	137.6890	137.6250	137.5017	137.6159	138.0254	137.6182	137.9058	137.6202	138.2148	**127.3**
**C5**	127.9135	129.1809	130.0590	129.8632	131.1212	129.8155	131.5741	129.7668	131.4328	129.9052	131.7024	128.1482	129.3461	129.2693	130.0892	131.3822	129.7056	130.8910	129.6712	130.8140	130.1251	131.5082	**130.8**
**C6**	129.7716	131.4533	132.5446	132.0478	133.2631	131.9830	133.8612	131.9177	133.5469	132.1071	134.2333	130.2562	132.0857	132.5268	132.9957	134.2870	132.8131	134.4372	132.7650	134.2634	133.0421	134.9004	**136.5**
**C7**	199.0631	204.4435	205.6649	208.3211	209.7781	208.1668	214.4876	208.0065	213.6878	208.4576	215.1195	202.6406	207.2108	209.1752	209.7820	211.0719	209.7166	215.7680	209.5671	214.7083	209.9037	216.2767	**196.6**
**C8**	62.7897	63.9111	64.1399	64.8318	65.6221	64.8026	66.0819	64.7684	66.1193	64.8523	66.7284	57.6874	59.4758	60.3520	60.6579	61.5790	60.5674	62.3296	60.4982	62.1533	60.7085	62.5668	**61**
**C9**	21.8674	22.0407	22.2262	22.2335	22.4502	22.2185	22.5155	22.2016	22.4381	22.2442	22.6756	18.7458	18.9793	19.1654	19.2809	19.4503	19.2506	19.5379	19.2381	19.4995	19.2853	19.8791	**15.8**
**C4``**	129.4413	130.5130	131.4344	130.9910	132.2841	130.966	132.0755	130.9390	132.0703	131.0098	132.1693	129.3960	130.5030	131.2850	130.9692	132.2407	130.9560	132.1348	130.9280	132.1129	130.9874	132.1811	**123.2**
**C8`**	35.6477	36.3949	36.6433	36.7863	37.0730	36.7590	37.1996	36.7303	37.1493	36.8084	37.6152	31.1859	31.3739	31.4675	31.5248	31.7044	31.5167	31.7547	31.5044	31.7281	31.5335	31.8377	**31.8**
**H2**	8.6067	8.5252	8.5911	8.3672	8.3300	8.3688	8.3874	8.3707	8.3491	8.3661	8.3995	8.6854	8.5157	8.4972	8.4018	8.3519	8.4108	8.3573	8.4164	8.3585	8.3980	8.3561	**8.24**
**H3**	8.0032	8.1022	8.1800	8.1296	8.2266	8.1278	8.2537	8.1258	8.2431	8.1312	8.2715	8.0166	8.1106	8.1666	8.1470	8.2384	8.1391	8.2514	8.1372	8.2444	8.1487	8.2674	**7.92**
**H5**	7.9050	8.045	8.1215	8.1076	8.2027	8.1035	8.2074	8.0992	8.1953	8.1113	8.2349	7.9065	8.0348	8.1013	8.0949	8.1935	8.0926	8.1931	8.0890	8.1839	8.0980	8.2209	**7.92**
**H6**	8.1439	8.3337	8.4722	8.3718	8.5033	8.3646	8.5646	8.3578	8.5167	8.3789	8.6071	7.9730	8.1461	8.1857	8.2199	8.3505	8.2067	8.3393	8.2029	8.3246	8.2243	8.3945	**8.24**
**H8**	4.3871	4.4813	4.5705	4.5491	4.6507	4.5431	4.6925	4.5372	4.6505	4.5552	4.7232	4.5562	4.6946	4.7520	4.7551	4.8727	4.7534	4.8817	4.7499	4.8662	4.7583	4.9184	**5.17**
**H(9A-9C)**	1.2754	1.2659	1.2574	1.2224	1.1629	1.2224	1.2011	1.2223	1.1893	1.2223	1.2197	1.3619	1.3777	1.3519	1.3944	1.3429	1.3918	1.3661	1.3905	1.3611	1.3959	1.3893	**1.59**
**H(8`A-8`C)**	2.4868	2.4414	2.3923	2.4466	2.4064	2.4474	2.3965	2.4480	2.4041	2.4455	2.3780	2.8679	2.7102	2.6407	2.6152	2.5576	2.6209	2.4852	2.6252	2.4982	2.6116	2.4598	**2.8**
Nuclei	BHandH																						
**C1**	145.7019	146.1416	147.4184	146.3886	148.1155	146.3524	147.3162	146.3373	147.6906	146.3969	147.6156	144.4298	145.6978	146.4805	146.3536	147.0977	146.3011	147.8321	146.2675	147.4549	146.3736	148.0981	**137.3**
**C2**	140.0914	140.0917	140.6887	139.9711	140.3671	139.9699	140.7074	139.9870	140.6668	139.9424	140.7223	139.8089	139.9586	140.5869	139.8854	140.6295	139.8787	140.9162	139.8905	140.8450	139.8621	140.7983	**136.5**
**C3**	135.1669	135.8248	136.7648	136.0532	136.9621	136.0308	137.0385	136.0266	137.0481	136.0443	137.1305	135.2159	135.8456	136.9134	136.0259	136.7700	136.0056	137.3482	136.0037	137.3164	136.0151	137.1778	**130.8**
**C4**	144.3785	144.1738	144.2765	144.3037	144.2332	144.2821	145.1007	144.2789	144.9043	144.2944	145.2415	144.5924	144.1848	144.3847	144.1940	144.9381	144.1799	144.8271	144.1849	144.7566	144.1773	144.9655	**127.3**
**C5**	133.5573	134.9149	135.5938	135.6558	136.9209	135.5972	136.9760	135.5575	136.8891	135.6814	137.1467	133.6557	134.9210	135.5246	135.6503	136.3944	135.5956	136.5549	135.5594	136.4748	135.6721	137.0131	**130.8**
**C6**	137.0738	139.0908	140.1712	140.1104	141.8547	140.0418	142.3276	139.9908	142.0316	140.1446	142.6903	137.3760	139.5800	140.6540	140.6656	141.4097	140.5955	142.6329	140.5427	142.5034	140.7010	143.0654	**136.5**
**C7**	204.0156	208.7373	210.4711	211.2602	212.2504	211.0847	217.1069	210.9305	216.2432	211.3998	218.0467	208.0179	212.2447	213.7490	214.5364	215.2805	214.3847	220.1811	214.2517	218.9100	214.6508	220.8594	**196.6**
**C8**	60.4414	61.6304	62.3870	62.1025	63.0298	62.0653	63.5103	62.0464	63.4181	62.1076	64.2768	54.5559	55.9472	56.4343	56.7013	57.4454	56.6451	58.1501	56.6069	57.8360	56.7247	58.4949	**61**
**C9**	22.0777	22.3139	22.6589	22.5481	23.0631	22.5254	22.9624	22.5211	22.8939	22.5399	23.1241	18.8080	19.1580	19.3069	19.3513	20.0954	19.3326	19.7545	19.3319	19.7168	19.3382	20.1267	**15.8**
**C4``**	121.6910	122.7021	123.6414	123.0950	124.3794	123.0623	124.2265	123.0477	124.2202	123.0957	124.3285	121.6363	122.6689	123.5830	123.0738	123.8179	123.0408	124.2025	123.0258	124.1798	123.0752	124.3124	**123.2**
**C8`**	33.7954	34.5397	34.8533	34.9118	35.2617	34.8763	35.4701	34.8593	35.4575	34.9158	35.9810	28.5695	28.9934	29.3575	29.2580	30.0021	29.2316	29.6683	29.2230	29.7441	29.2525	29.8112	**31.8**
**H2**	9.0057	8.9629	8.9413	8.9192	8.8889	8.9238	8.9061	8.9273	8.8857	8.9156	8.8945	9.0260	8.8929	8.9231	8.8116	8.7304	8.8171	8.7802	8.8218	8.8242	8.8074	8.7699	**8.24**
**H3**	8.1943	8.2934	8.3585	8.3254	8.4285	8.3245	8.4458	8.3229	8.4376	8.3271	8.4634	8.2008	8.2949	8.3656	8.3287	8.2475	8.3277	8.4401	8.3259	8.4335	8.3306	8.4581	**7.92**
**H5**	8.0848	8.2389	8.3013	8.3132	8.4069	8.3097	8.4200	8.3053	8.4074	8.3176	8.4457	8.0768	8.2189	8.2948	8.2855	8.2043	8.2825	8.3999	8.2786	8.3921	8.2894	8.4191	**7.92**
**H6**	8.3966	8.6212	8.6906	8.7261	8.8708	8.7209	8.9171	8.7147	8.8824	8.7319	8.9501	8.2073	8.4147	8.5275	8.5145	8.4333	8.5097	8.6930	8.5041	8.6950	8.5199	8.7394	**8.24**
**H8**	4.2120	4.3340	4.3352	4.4212	4.4816	4.4160	4.5274	4.4100	4.4996	4.4273	4.5511	4.4806	4.6494	4.7236	4.7331	4.6519	4.7289	4.8821	4.7238	4.8739	4.7382	4.9150	**5.17**
**H(9A-9C)**	1.4175	1.4308	1.4026	1.4227	1.3902	1.4249	1.4035	1.4261	1.3909	1.4214	1.4185	1.4962	1.5077	1.4956	1.5142	1.4330	1.5146	1.4951	1.5141	1.5055	1.5149	1.5141	**1.59**
**H(8`A-8`C)**	2.5808	2.4999	2.4871	2.4733	2.4554	2.4757	2.4253	2.4772	2.4358	2.4723	2.4148	2.9111	2.7427	2.7071	2.6666	2.5854	2.6714	2.5240	2.6754	2.5577	2.6634	2.4974	**2.8**
Nuclei	BHandHLYP																						
**C1**	146.3121	146.5525	147.8583	147.5581	149.6920	147.5636	148.8377	147.5762	148.8568	147.5629	149.1315	144.5727	146.2680	147.5969	147.0512	148.7082	147.0176	149.2967	146.9576	149.6938	147.1096	149.4106	**137.3**
**C2**	138.6016	138.5440	138.9688	138.4261	138.6517	138.4425	138.9710	138.4597	138.8978	138.4079	138.9527	138.2358	138.3538	139.0367	138.2429	138.5840	138.3388	139.1401	138.3425	139.0524	138.2358	139.0093	**136.5**
**C3**	134.0359	134.5814	135.4061	134.8426	135.7194	134.8359	135.7277	134.8308	135.7014	134.8477	135.8105	134.0221	134.5913	136.0934	134.7087	135.4762	134.9262	135.9861	134.9094	135.9923	134.7123	135.7178	**130.8**
**C4**	143.3617	143.0291	143.0714	142.9946	142.8299	142.9786	143.6807	142.9666	143.5511	143.0103	143.8002	143.6072	142.9649	142.9717	142.8616	142.8169	142.8712	143.3124	142.8798	143.1200	142.8558	143.4747	**127.3**
**C5**	132.4297	133.7007	134.4084	134.4248	135.6855	134.3750	135.7269	134.3301	135.6828	134.4760	135.9161	132.5700	133.7586	133.8833	134.4954	135.8406	134.2125	135.3448	134.1841	135.3066	134.5392	135.8925	**130.8**
**C6**	135.0266	136.9881	137.8564	137.7174	138.9615	137.6586	139.4531	137.5982	139.2987	137.7662	139.7559	135.4225	137.3313	137.8061	138.3083	139.7084	138.1463	139.7508	138.1015	139.4898	138.3567	140.2118	**136.5**
**C7**	202.2642	207.3002	209.0695	210.7544	212.0678	210.5655	217.6680	210.3940	216.3727	210.9595	218.7321	206.5494	211.1452	213.0417	213.5723	214.5673	213.4765	220.1641	213.3257	219.1680	213.7160	220.7486	**196.6**
**C8**	61.2143	62.1854	62.8092	62.7125	63.5015	62.7099	64.0602	62.7051	63.8792	62.7082	64.7152	55.94815	57.7045	58.2737	58.6126	59.2881	58.5194	60.2216	58.4596	60.2070	58.6723	60.4896	**61**
**C9**	21.9019	22.1062	22.2853	22.1853	22.3814	22.1790	22.2770	22.1722	22.2756	22.1860	22.4002	18.7855	18.9103	18.9455	19.0174	19.1544	18.9986	19.2691	18.9937	19.3062	19.0233	19.5690	**15.8**
**C4``**	123.9654	125.0152	125.9444	125.4484	126.7633	125.4255	126.5959	125.4050	126.5890	125.4698	126.6896	123.8961	124.9998	125.7652	125.4378	126.7161	125.4060	126.5830	125.3840	126.5836	125.4603	126.6635	**123.2**
**C8`**	34.7861	35.5022	35.7539	35.8991	36.2723	35.8756	36.4696	35.8554	36.4359	35.9233	36.8786	30.4947	30.6543	30.8775	30.8773	31.1566	30.8681	31.1523	30.8589	31.0884	30.8866	31.2586	**31.8**
**H2**	8.8567	8.8305	8.8027	8.7110	8.6532	8.7124	8.6791	8.7133	8.6834	8.7084	8.6655	8.9189	8.7319	8.7245	8.6412	8.6292	8.6511	8.5762	8.6575	8.5492	8.6348	8.5765	**8.24**
**H3**	8.1220	8.2200	8.2888	8.2508	8.3540	8.2492	8.3733	8.2475	8.3663	8.2524	8.3905	8.1292	8.2227	8.2764	8.2587	8.3548	8.2509	8.3688	8.2494	8.3604	8.2604	8.3892	**7.92**
**H5**	8.0237	8.1724	8.2333	8.2391	8.3279	8.2346	8.3400	8.2299	8.3304	8.2433	8.3642	8.0164	8.1491	8.2265	8.2122	8.3137	8.2165	8.3169	8.2125	8.3064	8.2152	8.3365	**7.92**
**H6**	8.2177	8.4560	8.5295	8.5355	8.6677	8.5257	8.7093	8.5161	8.6953	8.5451	8.7449	8.0753	8.2606	8.3234	8.3532	8.5113	8.3435	8.4914	8.3391	8.4606	8.3574	8.5415	**8.24**
**H8**	4.1304	4.2693	4.3064	4.3494	4.4440	4.3382	4.4773	4.3279	4.4595	4.3612	4.5127	4.3324	4.4933	4.5642	4.5666	4.6951	4.5658	4.7150	4.5619	4.6908	4.5700	4.7434	**5.17**
**H(9A-9C)**	1.3112	1.3272	1.2928	1.2892	1.2274	1.2915	1.2550	1.2928	1.2511	1.2856	1.2615	1.3879	1.3911	1.3550	1.4030	1.3627	1.4023	1.3722	1.4017	1.3572	1.4034	1.3945	**1.59**
**H(8`A-8`C)**	2.4762	2.4107	2.3942	2.4187	2.3854	2.4212	2.3850	2.4232	2.3815	2.4161	2.3726	2.8760	2.7046	2.6627	2.6264	2.5905	2.6340	2.4950	2.6392	2.4953	2.6217	2.4699	**2.8**

[a] methyl protons shifts are averaged

[b] according to [2]

Table S2. Cont. II

Nuclei	R											S											Exp. [b]
	BLYP																						
	Gas	$CHCl_3$ (PCM)	$CHCl_3$ (SMD)	DMSO (PCM)	DMSO (SMD)	CH_3OH (PCM)	CH_3OH (SMD)	C_2H_5OH (PCM)	C_2H_5OH (SMD)	H_2O (PCM)	H_2O (SMD)	Gas	$CHCl_3$ (PCM)	$CHCl_3$ (SMD)	DMSO (PCM)	DMSO (SMD)	CH_3OH (PCM)	CH_3OH (SMD)	C_2H_5OH (PCM)	C_2H_5OH (SMD)	H_2O (PCM)	H_2O (SMD)	
**C1**	145.5691	146.6600	146.5234	146.9367	150.1369	147.1375	148.8278	146.9070	149.3222	147.4258	148.8749	142.3549	144.8625	146.5583	146.0678	144.9821	146.2450	148.1781	145.9637	148.3966	146.5221	148.6549	**137.3**
**C2**	134.8518	134.8129	135.1590	134.5621	135.1393	134.8472	135.5103	134.6733	135.6288	134.9242	135.5643	134.0693	134.4932	135.3458	134.5644	134.2789	134.9297	135.6398	134.7370	135.7384	134.9340	135.4918	**136.5**
**C3**	131.4357	132.1595	132.9628	132.3380	133.2580	132.6124	133.7236	132.4254	133.8579	132.7081	133.9167	131.5325	132.1094	133.8333	132.2584	134.1568	132.8854	133.6138	132.6908	133.7752	132.6272	133.1362	**130.8**
**C4**	141.1003	140.5808	140.7358	140.5463	140.1739	140.8348	140.8203	140.6570	140.8153	140.8911	141.0651	141.4410	140.6641	140.5575	140.4714	141.0576	140.7536	140.7729	140.5828	140.8429	140.8233	140.9911	**127.3**
**C5**	130.0199	131.3508	132.2481	132.1327	133.5020	132.3692	132.8747	132.1393	132.9537	132.5242	133.0940	130.5351	131.6260	131.4128	132.2051	133.5053	132.1044	132.8535	131.8921	132.9563	132.5977	133.7610	**130.8**
**C6**	131.6964	133.3553	134.4573	134.2296	135.4201	134.4561	135.5383	134.2152	135.4683	134.6318	136.0571	132.1611	134.0348	134.4695	134.9687	136.5949	135.0698	136.4976	134.8343	136.4925	135.3806	137.0076	**136.5**
**C7**	205.7569	211.4352	212.4165	214.5902	216.9674	214.6727	221.0058	214.3064	220.4511	215.1569	221.6868	210.5147	214.9191	216.9136	217.1238	220.8124	217.3423	222.6570	217.0251	222.0130	217.6115	223.4084	**196.6**
**C8**	69.8760	71.0382	71.3826	71.3041	72.7011	71.5240	72.9912	71.2975	73.1469	71.7501	73.7169	63.7503	66.2861	67.0905	67.4097	69.7838	67.5360	69.1487	67.2701	69.1714	67.8525	69.5429	**61**
**C9**	23.4060	23.6195	23.8880	23.8593	24.0973	24.1246	24.1549	23.9272	24.2462	24.2331	24.4374	20.0665	20.5318	20.6228	20.7449	24.4284	20.9752	21.0816	20.7764	21.1883	21.1222	21.5539	**15.8**
**C4``**	145.4983	146.8369	147.8684	147.3069	148.9554	147.5568	148.4956	147.3451	148.6634	147.6977	148.6336	145.3895	146.7787	147.6522	147.278	148.7094	147.5324	148.5749	147.3205	148.7378	147.6660	148.6777	**123.2**
**C8`**	38.2168	38.9589	39.1759	39.3452	39.6889	39.5998	39.6660	39.3915	39.8168	39.7275	40.1541	33.7079	33.6516	33.7261	33.7350	39.6742	34.0300	33.8693	33.8437	34.0401	34.1004	33.9740	**31.8**
**H2**	8.3554	8.2819	8.3764	8.2361	8.1263	8.2907	8.3818	8.2505	8.1905	8.2806	8.4140	8.5076	8.3168	8.3026	8.2185	8.4154	8.2812	8.3608	8.2437	8.1909	8.2666	8.3486	**8.24**
**H3**	7.8140	7.9069	7.9837	7.9340	8.0257	7.9805	8.2049	7.9342	8.0294	7.9898	8.2364	7.8399	7.9321	7.9693	7.9560	8.0747	7.9947	8.2254	7.9484	8.0520	8.0123	8.2511	**7.92**
**H5**	7.7255	7.8572	7.9348	7.9219	8.0051	7.9664	8.1931	7.9180	8.0155	7.9796	8.2325	7.7377	7.8518	7.9232	7.9007	8.0759	7.9507	8.1703	7.9026	7.9954	7.9584	8.2073	**7.92**
**H6**	7.9764	8.1608	8.2937	8.2825	8.3485	8.3271	8.5690	8.2772	8.3701	8.3364	8.6281	7.8114	7.9514	7.9975	8.0197	8.4505	8.0613	8.3163	8.0133	8.1340	8.0776	8.3721	**8.24**
**H8**	4.6194	4.6944	4.7506	4.8193	4.8680	4.8646	5.0681	4.8159	4.8746	4.8737	5.1169	4.7461	4.8766	4.9368	4.9331	4.6286	4.9824	5.2265	4.9348	5.0468	4.9903	5.2715	**5.17**
**H(9A-9C)**	1.3404	1.3295	1.3398	1.2971	1.2421	1.3455	1.4513	1.3013	1.2742	1.3511	1.4842	1.4264	1.4565	1.4351	1.4715	1.1906	1.5191	1.6285	1.4732	1.4541	1.5276	1.6614	**1.59**
**H(8`A-8`C)**	2.6100	2.5494	2.4979	2.5243	2.5074	2.5719	2.6623	2.5274	2.5006	2.5804	2.6514	3.0578	2.8675	2.8083	2.7722	2.4494	2.8320	2.8056	2.7941	2.6486	2.8207	2.7846	**2.8**
Nuclei	BMK																						
**C1**	162.1489	162.5844	164.0500	162.8924	164.7003	162.8385	164.6408	163.0004	164.4061	163.1095	164.8594	160.7828	162.4101	163.3813	163.4015	165.2813	163.3225	165.7581	163.4575	165.9039	163.6403	165.9851	**137.3**
**C2**	152.3702	152.2294	152.7678	152.0151	152.5796	152.0178	152.8191	152.2305	152.6988	152.1734	152.7628	151.8865	151.8126	152.7096	151.5887	152.2296	151.5814	152.9402	151.7879	152.7907	151.7593	152.6803	**136.5**
**C3**	147.8971	148.4038	149.3231	148.5730	149.7972	148.5473	149.9162	148.7329	149.7496	148.7594	149.9594	148.0229	148.6432	150.4182	148.6456	149.7140	148.6269	150.2574	148.8214	150.1445	148.8257	149.8594	**130.8**
**C4**	159.0221	158.5621	158.7661	158.5116	158.7354	158.4984	159.4333	158.6965	159.2265	158.6858	159.5007	159.1598	158.5656	158.6943	158.3245	158.4583	158.3194	159.2047	158.5265	158.9527	158.4919	159.0710	**127.3**
**C5**	146.0877	147.5663	148.5303	148.2203	149.7341	148.1654	149.7982	148.3220	149.6452	148.3436	149.8943	146.3633	147.4663	147.8095	148.1460	149.9241	148.0908	149.3408	148.2454	149.1755	148.3594	149.8800	**130.8**
**C6**	149.0518	150.9702	152.2328	151.9767	154.0266	151.9029	154.1714	152.0399	153.9111	152.2074	154.4901	149.2994	151.3630	152.1414	152.4473	154.4305	152.3715	154.3431	152.5048	153.9915	152.6789	154.8513	**136.5**
**C7**	218.1751	223.5815	225.5580	226.4561	227.5627	226.2518	233.9701	226.2629	232.4177	226.8155	234.9721	222.5349	227.6432	229.3304	230.2334	231.3661	230.0458	237.6187	230.0707	236.4269	230.5746	238.2951	**196.6**
**C8**	71.0752	72.3742	73.2350	72.8705	73.9673	72.8224	74.7292	72.9859	74.4130	73.0782	75.4659	65.4191	66.9836	67.6768	67.9199	68.9170	67.8480	69.8369	67.9883	69.6498	68.1509	70.0932	**61**
**C9**	24.9257	25.6319	25.7358	25.7424	26.2413	25.7301	26.1534	25.9288	26.0751	25.9163	26.1278	22.1002	22.2767	22.5169	22.2221	22.6719	22.2080	22.7122	22.4067	22.6142	22.3983	22.9474	**15.8**
**C4``**	133.4438	134.4888	135.5976	134.8604	136.4792	134.8234	136.2765	134.9974	136.1624	135.0570	136.3210	133.5024	134.5926	135.5943	134.9543	136.6883	134.9171	136.3798	135.0911	136.2708	135.1513	136.5088	**123.2**
**C8`**	38.7377	39.4936	39.7790	39.7515	40.2471	39.7212	40.6929	39.9021	40.5525	39.9415	41.1304	33.7908	34.0582	34.3832	34.0967	34.5972	34.0830	34.5047	34.2798	34.2266	34.2705	34.5456	**31.8**
**H2**	9.0938	9.0679	9.0203	9.0076	8.9904	9.0107	8.9552	9.0134	8.9764	8.9946	8.9485	9.1036	8.9442	8.9687	8.8526	8.8601	8.8557	8.7947	8.8586	8.7737	8.8400	8.7949	**8.24**
**H3**	8.3639	8.4823	8.5545	8.5126	8.6249	8.5082	8.6455	8.5038	8.6435	8.5073	8.6603	8.4197	8.5279	8.5564	8.5588	8.6498	8.5542	8.6598	8.5496	8.6512	8.5536	8.6696	**7.92**
**H5**	8.2970	8.4347	8.5028	8.5103	8.6171	8.5025	8.6208	8.4947	8.6129	8.5082	8.6483	8.2686	8.4352	8.5235	8.4782	8.5585	8.4723	8.6358	8.4667	8.6315	8.4744	8.5928	**7.92**
**H6**	8.5066	8.7584	8.8206	8.8510	9.0074	8.8438	9.0154	8.8363	9.0131	8.8482	9.0489	8.3681	8.5747	8.6555	8.6234	8.7797	8.6150	8.8028	8.6070	8.7854	8.6219	8.8427	**8.24**
**H8**	4.3221	4.4690	4.4853	4.5567	4.6355	4.5479	4.6679	4.5392	4.6508	4.5556	4.6909	4.5449	4.7461	4.7948	4.8057	4.9302	4.7996	4.9372	4.7937	4.9324	4.8021	4.9774	**5.17**
**H(9A-9C)**	1.3300	1.3666	1.3216	1.3614	1.3173	1.3599	1.3135	1.3581	1.3190	1.3532	1.3277	1.3839	1.3991	1.3852	1.4013	1.3618	1.3973	1.3694	1.3935	1.3694	1.3957	1.4017	**1.59**
**H(8`A-8`C)**	2.4630	2.3876	2.3792	2.3941	2.3517	2.3895	2.3552	2.3851	2.3522	2.3891	2.3391	2.8009	2.6649	2.6226	2.5759	2.5284	2.5773	2.4350	2.5789	2.4500	2.5651	2.4140	**2.8**
Nuclei	BP86																						
**C1**	142.2646	143.7967	143.7179	143.7733	145.7769	143.7983	146.0836	143.8174	146.3697	143.7525	145.7449	139.6980	141.9170	143.7007	143.1322	144.9804	143.0756	145.3484	142.9928	145.3536	143.1845	145.7628	**137.3**
**C2**	133.4166	133.6634	134.0556	133.4064	133.7486	133.4468	134.1792	133.4774	134.1360	133.3638	134.2001	132.7981	133.3000	134.2243	133.3792	133.8505	133.4653	134.5457	133.3793	134.4621	133.3713	134.4826	**136.5**
**C3**	129.9889	130.8670	131.7557	131.1277	132.0521	131.1244	132.1394	131.1151	132.1198	131.1244	132.3793	130.0666	130.8531	132.5427	130.9799	131.7302	131.2312	132.3693	130.9784	132.3287	130.9727	132.0978	**130.8**
**C4**	139.0519	138.7900	138.9600	138.8747	138.7182	138.8678	139.3814	138.8577	139.1809	138.8752	139.7657	139.3303	138.9119	138.8678	138.7574	138.6913	138.7803	139.2332	138.7839	139.1107	138.7379	139.5171	**127.3**
**C5**	128.7014	130.1207	130.8447	130.7991	132.0480	130.7576	132.2340	130.7136	132.0726	130.8338	132.5371	129.0444	130.3500	130.2439	130.8983	132.4032	130.6124	131.7364	130.8457	131.6537	130.9162	132.6390	**130.8**
**C6**	130.4160	132.2124	133.3640	133.0818	134.5452	133.0422	134.5088	132.9948	134.2449	133.1140	135.4175	130.8009	132.8271	133.2622	133.7618	135.2945	133.6170	135.4010	133.6702	135.2012	133.7971	136.0261	**136.5**
**C7**	202.8078	208.1644	208.9623	210.7829	212.2899	210.5886	217.7502	210.4063	216.9637	210.9661	217.7314	206.9460	211.4050	213.3954	213.5283	214.8867	213.4545	219.0973	213.2805	218.2467	213.6352	219.7694	**196.6**
**C8**	67.8843	69.3335	69.6779	69.5409	70.4242	69.5564	71.1584	69.5625	71.0890	69.5238	72.0440	62.1863	64.1865	64.9372	65.24985	66.0652	65.1540	66.9231	65.1273	66.7453	65.2981	67.3638	**61**
**C9**	23.1337	23.5410	23.8162	23.6945	23.8770	23.6902	23.9562	23.6814	23.8516	23.6956	24.3512	19.9182	20.5341	20.5499	20.7715	20.8608	20.7498	21.1238	20.7608	21.0535	20.7678	21.7281	**15.8**
**C4``**	140.7811	142.0861	143.0219	142.4585	143.8858	142.4472	143.6876	142.4300	143.6671	142.4641	143.9114	140.7074	142.0443	142.8261	142.4413	143.8188	142.4363	143.7000	142.4092	143.6774	142.4486	143.8700	**123.2**
**C8`**	37.4868	38.2791	38.3911	38.6410	38.8798	38.6135	39.0726	38.5858	39.0160	38.6614	39.6960	32.54885	32.8078	32.8375	32.8627	33.0371	32.8736	33.0995	32.8666	33.0724	32.8547	33.2815	**31.8**
**H2**	8.4331	8.3442	8.4264	8.3297	8.2787	8.3317	8.2600	8.3330	8.2368	8.3267	8.3138	8.5262	8.3879	8.3556	8.2859	8.2521	8.2977	8.2452	8.2991	8.2436	8.2799	8.2446	**8.24**
**H3**	7.8575	7.9549	8.0224	7.9896	8.0803	7.9887	8.0902	7.9873	8.0801	7.9900	8.1270	7.8812	7.9805	8.0200	8.0076	8.0899	8.0039	8.1031	8.0061	8.0957	8.0078	8.1278	**7.92**
**H5**	7.7646	7.8998	7.9692	7.9747	8.0619	7.9707	8.0684	7.9664	8.0567	7.9780	8.1086	7.7716	7.8999	7.9566	7.9521	8.0500	7.9539	8.0466	7.9474	8.0375	7.9538	8.0845	**7.92**
**H6**	8.0653	8.2205	8.3463	8.3520	8.4613	8.3423	8.4633	8.3328	8.4265	8.3602	8.5319	7.8580	8.0294	8.0571	8.0981	8.2366	8.0917	8.2198	8.0922	8.2031	8.1006	8.2827	**8.24**
**H8**	4.6570	4.7066	4.7715	4.8202	4.8879	4.8100	4.9403	4.8004	4.9108	4.8291	4.9488	4.8048	4.9410	4.9862	4.9950	5.1087	4.9956	5.1146	4.9905	5.1012	4.9967	5.1640	**5.17**
**H(9A-9C)**	1.3727	1.3650	1.3267	1.3466	1.2958	1.3502	1.2795	1.3530	1.2690	1.3425	1.3488	1.4586	1.4897	1.4414	1.4989	1.4497	1.4992	1.4660	1.4986	1.4581	1.4988	1.5069	**1.59**
**H(8`A-8`C)**	2.5962	2.5441	2.4807	2.5028	2.4656	2.5068	2.4540	2.5104	2.4587	2.4991	2.4517	2.9894	2.8226	2.7466	2.7223	2.6666	2.7314	2.5717	2.7352	2.5855	2.7161	2.5566	**2.8**
Nuclei	CAM-B3LYP																						
**C1**	147.8331	148.0612	149.4087	149.2392	151.3209	149.2575	151.9757	149.2753	152.0486	149.2236	147.1352	145.9561	147.7934	149.4617	148.9977	150.7759	148.9300	151.3446	148.8625	151.5034	149.0634	151.4483	**137.3**
**C2**	139.2196	139.2384	139.6705	139.1519	139.4930	139.1691	139.8694	139.1859	139.7962	139.1372	139.8883	138.8272	139.0516	139.8200	138.9926	139.3951	138.9981	139.9546	139.0053	139.8833	138.9896	139.7938	**136.5**
**C3**	135.1057	135.7619	136.5895	136.0205	136.9854	136.0164	137.1114	136.0119	137.0757	136.0265	137.2358	135.1182	135.7316	137.3113	135.8616	136.6664	135.8587	137.1937	135.8568	137.1705	135.8666	136.9149	**130.8**
**C4**	144.5181	144.2000	144.2052	144.1235	143.9978	144.1154	144.5894	144.1087	144.4582	144.1343	145.3632	144.7712	144.1513	144.0841	144.0023	143.9868	144.0138	144.3978	144.0256	144.2495	143.9939	144.5629	**127.3**
**C5**	133.6120	134.9268	135.5905	135.6077	136.8241	135.5668	137.0138	135.5271	136.9540	135.6488	136.9963	133.7704	135.0168	135.0367	135.7448	137.1757	135.7078	136.6219	135.6700	136.5454	135.7810	137.1885	**130.8**
**C6**	135.8297	137.8172	138.7028	138.5346	139.9659	138.4736	139.6822	138.4129	139.4975	138.5938	141.6518	136.2466	138.2289	138.5649	139.1705	140.6843	139.1237	140.6353	139.0757	140.4194	139.2158	141.1226	**136.5**
**C7**	206.4300	211.4112	213.2687	214.9491	216.2792	214.7938	223.3939	214.6410	222.3086	215.1017	222.4203	210.6466	215.3777	217.4653	218.0053	219.1690	217.8558	224.5036	217.7056	223.4555	218.1473	225.0595	**196.6**
**C8**	64.0416	65.1055	65.7587	65.6972	66.6293	65.6893	67.3170	65.6799	67.1138	65.7048	64.9357	58.4519	60.2829	61.0035	61.3035	62.0728	61.2406	63.0518	61.1782	62.9527	61.3651	63.3456	**61**
**C9**	22.0749	22.3373	22.5065	22.4375	22.7481	22.4276	22.4563	22.4183	22.4207	22.4492	19.7576	19.0778	19.2606	19.2650	19.4223	19.5949	19.4204	19.6547	19.4187	19.6569	19.4260	20.0096	**15.8**
**C4``**	133.2084	134.3677	135.3027	134.8064	136.2040	134.7870	136.0491	134.7677	136.0203	134.8266	136.0181	133.1468	134.3508	135.1315	134.7907	136.1580	134.7701	136.0284	134.7496	136.0058	134.8120	136.1044	**123.2**
**C8`**	35.5599	36.3294	36.5371	36.7112	37.1211	36.6937	37.2322	36.6767	37.1742	36.7298	34.6845	30.9234	31.1677	31.2719	31.3452	31.6371	31.3411	31.6285	31.3371	31.5700	31.3512	31.7430	**31.8**
**H2**	8.7541	8.7416	8.7094	8.6099	8.5657	8.6103	8.4853	8.6108	8.4831	8.6095	8.7927	8.8263	8.6455	8.6136	8.5409	8.5265	8.5479	8.4657	8.5551	8.4567	8.5345	8.4655	**8.24**
**H3**	8.0855	8.1815	8.2482	8.2081	8.3088	8.2066	8.3223	8.2052	8.3163	8.2094	8.3547	8.0960	8.1845	8.2363	8.2195	8.3165	8.2179	8.3224	8.2164	8.3152	8.2209	8.3416	**7.92**
**H5**	7.9899	8.1376	8.1985	8.1994	8.2908	8.1949	8.3011	8.1906	8.2928	8.2036	8.3570	7.9879	8.1159	8.1919	8.1726	8.2760	8.1697	8.2766	8.1669	8.2677	8.1754	8.2950	**7.92**
**H6**	8.1943	8.4319	8.5003	8.4945	8.6252	8.4855	8.6137	8.4768	8.6024	8.5031	8.6860	8.0430	8.2250	8.2698	8.3093	8.4683	8.3054	8.4327	8.3016	8.4114	8.3128	8.4834	**8.24**
**H8**	4.2721	4.4091	4.4398	4.4757	4.5583	4.4663	4.6372	4.4575	4.6259	4.4851	4.7466	4.4692	4.6298	4.6984	4.7032	4.8365	4.6997	4.8419	4.6962	4.8222	4.7064	4.8725	**5.17**
**H(9A-9C)**	1.3399	1.3586	1.3274	1.3196	1.2759	1.3203	1.2273	1.3210	1.2204	1.3184	1.3342	1.4178	1.4252	1.3830	1.4396	1.4032	1.4390	1.4036	1.4386	1.3956	1.4401	1.4273	**1.59**
**H(8`A-8`C)**	2.5511	2.4741	2.4620	2.4842	2.4579	2.4863	2.4505	2.4884	2.4491	2.4821	2.6052	2.9352	2.7654	2.7099	2.6729	2.6362	2.6780	2.5375	2.6833	2.5448	2.6681	2.5119	**2.8**

[a] methyl protons shifts are averaged

[b] according to [2]

Table S2. Cont. III

Nuclei	R											S											Exp. [b]
	HF																						
	Gas	$CHCl_3$ (PCM)	$CHCl_3$ (SMD)	DMSO (PCM)	DMSO (SMD)	CH_3OH (PCM)	CH_3OH (SMD)	C_2H_5OH (PCM)	C_2H_5OH (SMD)	H_2O (PCM)	H_2O (SMD)	Gas	$CHCl_3$ (PCM)	$CHCl_3$ (SMD)	DMSO (PCM)	DMSO (SMD)	CH_3OH (PCM)	CH_3OH (SMD)	C_2H_5OH (PCM)	C_2H_5OH (SMD)	H_2O (PCM)	H_2O (SMD)	
**C1**	145.4746	146.5190	146.8081	148.1520	151.2446	148.1657	149.9183	148.1803	149.2436	148.1389	148.9112	143.6786	145.7401	147.3630	147.0208	149.5293	146.8994	149.2120	146.7746	149.3477	147.0185	149.3030	**137.3**
**C2**	138.9119	138.7196	139.0655	138.3417	138.5966	138.3530	138.9051	138.3649	139.0067	138.3299	139.1089	138.8085	138.6264	139.0274	138.3491	138.4108	138.3730	138.7497	138.3986	138.6921	138.2557	138.7611	**136.5**
**C3**	133.7396	134.1385	134.6703	134.3730	135.9521	134.3812	135.8562	134.3905	135.7851	134.3651	136.0240	133.6502	134.2448	135.4236	134.4395	135.0940	134.4163	135.4077	134.3939	135.4077	134.1925	135.4770	**130.8**
**C4**	142.9631	142.5000	142.7296	142.2208	141.7357	142.2070	142.7203	142.1933	142.8021	142.2332	143.1391	143.3113	142.4814	142.3291	142.2447	141.9375	142.2711	142.4787	142.2990	142.3183	142.2539	142.6305	**127.3**
**C5**	131.6479	133.0547	133.7854	133.9384	134.7111	133.8946	134.5910	133.8499	134.4060	133.9787	134.3032	131.5261	132.9037	133.2407	133.6819	135.2673	133.6346	134.8526	133.5850	134.8091	134.0013	134.9857	**130.8**
**C6**	134.6762	136.3087	137.3819	136.6846	137.0557	136.6094	137.7254	136.5345	137.8490	136.7549	138.4447	135.2713	136.7389	137.0688	137.4040	138.3842	137.3945	138.5285	137.3856	138.3316	137.5778	138.8358	**136.5**
**C7**	196.0598	202.2945	203.2985	206.6321	208.5341	206.4672	215.2152	206.3000	212.7757	206.7860	214.8933	200.0583	205.2035	206.9481	208.1492	209.0586	207.9511	215.2860	207.7494	213.8542	208.1908	216.0633	**196.6**
**C8**	54.9908	55.9707	56.2601	56.7627	57.7256	56.7493	58.2795	56.7370	57.7262	56.7751	58.3544	50.2906	52.0564	52.7875	52.9453	53.9336	52.8697	54.7243	52.7950	54.5739	53.0302	54.9440	**61**
**C9**	20.4042	20.3340	20.4915	20.3608	20.5307	20.3519	20.3589	20.3440	20.3836	20.3690	20.4682	17.6736	17.4625	17.5321	17.4770	17.7311	17.4769	17.6417	17.4788	17.6565	17.4987	17.8176	**15.8**
**C4``**	108.7244	109.6737	110.4984	110.0621	111.2233	110.0421	111.0795	110.0222	111.0150	110.0803	111.0350	108.6816	109.6465	110.3873	110.0293	111.2293	110.0076	111.1109	109.9863	111.0926	110.0610	111.2125	**123.2**
**C8`**	32.7295	33.4363	33.6903	33.7315	34.0331	33.7143	34.1806	33.6972	34.1361	33.7471	34.5396	29.0093	29.1671	29.3364	29.3551	29.5595	29.3505	29.5823	29.3454	29.5540	29.3565	29.6888	**31.8**
**H2**	8.9162	8.7901	8.8751	8.5939	8.4989	8.5946	8.5536	8.5951	8.6229	8.5932	8.6514	9.0182	8.7587	8.7178	8.6319	8.5604	8.6448	8.5441	8.6580	8.5400	8.6267	8.5518	**8.24**
**H3**	8.1230	8.2340	8.3167	8.2754	8.3768	8.2733	8.3920	8.2710	8.3826	8.2774	8.4025	8.1222	8.2263	8.2942	8.2701	8.3959	8.2676	8.4001	8.2649	8.3926	8.2868	8.4192	**7.92**
**H5**	8.0282	8.1810	8.2525	8.2516	8.3780	8.2475	8.3880	8.2432	8.3761	8.2555	8.4121	8.0301	8.1769	8.2554	8.2448	8.3374	8.2413	8.3450	8.2375	8.3357	8.2365	8.3702	**7.92**
**H6**	8.1362	8.3677	8.5034	8.4340	8.5255	8.4249	8.5758	8.4155	8.5966	8.4426	8.6649	8.0488	8.2411	8.3015	8.3304	8.4696	8.3282	8.4581	8.3256	8.4385	8.3439	8.5005	**8.24**
**H8**	3.6783	3.8283	3.9138	3.9154	4.0206	3.9071	4.0652	3.8983	4.0697	3.9233	4.1272	3.8749	4.0599	4.1336	4.1480	4.2705	4.1448	4.2835	4.1412	4.2628	4.1494	4.3130	**5.17**
**H(9A-9C)**	1.2042	1.1823	1.1829	1.1259	1.0558	1.1257	1.0896	1.1254	1.1005	1.1262	1.1176	1.2983	1.2622	1.2253	1.2714	1.2111	1.2725	1.2298	1.2739	1.2244	1.2736	1.2426	**1.59**
**H(8`A-8`C)**	2.3078	2.2838	2.2303	2.2973	2.2624	2.2980	2.2685	2.2986	2.2566	2.2968	2.2503	2.6969	2.5346	2.4798	2.4585	2.4026	2.4636	2.3393	2.4688	2.3469	2.4520	2.3189	**2.8**
Nuclei	LC-BLYP																						
**C1**	152.0509	152.2904	153.7367	153.3601	155.5681	153.3670	154.9437	153.3954	154.4339	153.3288	155.2680	150.5045	152.2031	153.7908	153.2534	155.8212	153.1622	156.0226	153.0608	156.1468	153.3303	156.1618	**137.3**
**C2**	144.7125	144.6312	145.1272	144.5499	144.8944	144.5365	145.2491	144.5496	145.1530	144.5447	145.2395	144.4802	144.5126	145.2941	144.4095	144.8488	144.5040	145.2713	144.3999	145.1959	144.4110	145.2848	**136.5**
**C3**	140.2145	140.7752	141.7113	141.0557	142.0498	141.0254	142.1229	141.0212	141.9892	141.0660	142.2143	140.2499	140.7596	142.3781	140.8962	141.8768	141.1074	142.3066	140.8601	142.2860	140.9090	142.3933	**130.8**
**C4**	149.2071	148.8173	148.8841	148.7901	148.6545	148.7528	149.4392	148.7442	149.3874	148.8102	149.5669	149.4608	148.7676	148.8184	148.6584	148.5160	148.6472	148.9934	148.6594	148.8377	148.6557	149.1307	**127.3**
**C5**	138.5876	139.8632	140.5990	140.6264	141.8705	140.5611	141.8918	140.5236	141.8563	140.6714	142.0907	138.6374	139.9495	140.1075	140.7741	142.1180	140.4431	141.7181	140.6653	141.6278	140.8194	141.8604	**130.8**
**C6**	141.0535	143.0445	144.0231	143.8824	145.2947	143.7857	145.6579	143.7183	145.6385	143.9589	145.9777	141.4719	143.4938	143.9739	144.4883	145.8383	144.2965	145.9051	144.3790	145.6901	144.5358	146.2724	**136.5**
**C7**	211.6896	216.8845	218.8796	220.4281	221.7615	220.2641	227.5203	220.1231	225.8335	220.5608	228.6578	215.6685	220.5967	222.6674	223.3869	224.7998	223.2342	230.4409	223.0210	229.2955	223.5507	231.1013	**196.6**
**C8**	61.8066	62.9317	63.6984	63.6108	64.5342	63.5680	65.1152	63.5513	64.7959	63.6317	65.8516	56.0740	57.7256	58.4200	58.7387	59.6991	58.6176	60.5826	58.5785	60.4712	58.8057	60.9407	**61**
**C9**	21.1661	21.2770	21.5524	21.4751	21.7877	21.4332	21.6156	21.4196	21.6205	21.4987	21.7354	18.4099	18.4575	18.5392	18.5549	18.8782	18.5245	18.8809	18.5270	18.8811	18.5621	19.2427	**15.8**
**C4``**	132.5172	133.5825	134.5684	134.0496	135.3951	134.0033	135.2576	133.9839	135.2137	134.0758	135.3557	132.4788	133.5691	134.4274	134.0437	135.3697	133.9815	135.2416	133.9748	135.2063	134.0714	135.3360	**123.2**
**C8`**	33.6857	34.4375	34.7733	34.9029	35.3193	34.8597	35.5481	34.8435	35.4453	34.9260	36.0715	28.9840	29.3256	29.5147	29.5678	29.7478	29.5398	29.8261	29.5348	29.7508	29.5815	29.9991	**31.8**
**H2**	8.9592	8.9238	8.8891	8.8020	8.7486	8.7999	8.7505	8.7999	8.8006	8.8037	8.7371	9.0153	8.8275	8.8032	8.7157	8.6572	8.7274	8.6098	8.7322	8.6016	8.7088	8.6148	**8.24**
**H3**	8.2581	8.3475	8.4141	8.3755	8.4752	8.3732	8.4903	8.3724	8.4872	8.3768	8.5094	8.2680	8.3511	8.4017	8.3845	8.4819	8.3733	8.4866	8.3809	8.4799	8.3859	8.5050	**7.92**
**H5**	8.1636	8.3101	8.3726	8.3761	8.4648	8.3705	8.4731	8.3664	8.4673	8.3806	8.4990	8.1630	8.2921	8.3734	8.3499	8.4461	8.3581	8.4547	8.3435	8.4460	8.3527	8.4792	**7.92**
**H6**	8.3451	8.5831	8.6564	8.6531	8.7830	8.6429	8.8104	8.6347	8.8269	8.6622	8.8478	8.2072	8.4014	8.4562	8.4867	8.6272	8.4762	8.6044	8.4795	8.5835	8.4901	8.6475	**8.24**
**H8**	4.1559	4.3070	4.3311	4.3813	4.4678	4.3707	4.5064	4.3622	4.5124	4.3908	4.5393	4.3917	4.5685	4.6440	4.6477	4.7849	4.6466	4.7928	4.6400	4.7697	4.6512	4.8243	**5.17**
**H(9A-9C)**	1.3604	1.3752	1.3412	1.3433	1.2926	1.3421	1.3021	1.3426	1.3118	1.3437	1.3127	1.4296	1.4287	1.3910	1.4345	1.3876	1.4341	1.3963	1.4339	1.3899	1.4351	1.4191	**1.59**
**H(8`A-8`C)**	2.5416	2.4622	2.4501	2.4737	2.4486	2.4749	2.4370	2.4774	2.4232	2.4715	2.4263	2.8817	2.7085	2.6604	2.6308	2.5674	2.6366	2.4814	2.6403	2.4851	2.6266	2.4583	**2.8**
Nuclei	M06																						
**C1**	142.5304	142.5214	143.9959	143.5563	145.9436	143.5938	145.2542	143.6307	145.5188	143.5248	145.5315	140.8593	142.5937	144.5792	144.0367	145.8867	143.8832	146.3241	143.8079	146.4972	144.1042	146.2943	**137.3**
**C2**	137.3881	137.3101	137.8151	137.1436	137.6404	137.1585	137.9385	137.1789	137.8754	137.1414	137.9348	137.0441	137.2283	137.7575	136.6347	137.2838	137.1287	138.3154	137.1180	138.2547	136.6642	137.7695	**136.5**
**C3**	133.9825	134.3636	135.3553	134.5515	135.7811	134.5292	135.8873	134.5159	135.8726	134.5880	135.9711	134.5832	134.8735	135.7514	132.8369	133.9725	135.0082	136.1351	135.0026	136.1164	132.8791	134.1329	**130.8**
**C4**	140.3342	140.0366	140.1713	140.0494	139.9833	140.0193	140.7589	139.9992	140.5818	140.0946	140.8758	140.4119	139.6902	139.8023	139.7549	139.8604	139.5337	140.1577	139.5367	140.0045	139.7794	140.5695	**127.3**
**C5**	130.4780	131.8510	133.0096	132.7277	134.2279	132.6652	134.2392	132.6119	134.1416	132.8026	134.4146	130.5129	131.8350	132.8544	134.3366	135.5591	132.5786	133.7689	132.5373	133.6953	134.3885	135.7114	**130.8**
**C6**	133.6983	135.5708	136.5678	136.3975	137.6546	136.3241	138.0807	136.2581	137.8349	136.4818	138.3952	133.7133	135.6859	136.7150	137.3373	138.9866	136.6725	138.6820	136.6123	138.4382	137.4152	139.6779	**136.5**
**C7**	203.3427	208.0439	210.0199	211.2717	212.6612	211.1231	217.6004	210.9825	216.6066	211.4269	218.4942	207.5557	211.9284	213.7581	214.1467	215.1908	214.0737	219.9951	213.9362	219.0198	214.2939	220.3471	**196.6**
**C8**	61.1980	62.2855	63.0763	62.9953	64.0262	62.9770	64.5450	62.9621	64.4476	63.0228	65.3182	54.5954	56.2359	57.0974	57.3333	58.2781	57.1446	59.0542	57.0811	58.9330	57.4050	59.4515	**61**
**C9**	21.6160	22.0167	22.4473	22.2890	22.6669	22.2353	22.7136	22.1942	22.5978	22.3590	22.8140	19.1901	19.2771	19.4926	19.5442	19.8770	19.4422	19.9670	19.4291	19.9423	19.4856	20.4634	**15.8**
**C4``**	125.7597	126.7920	127.9093	127.2691	128.8332	127.2418	128.6555	127.2213	128.6480	127.3085	128.7376	125.5460	126.7182	127.8794	127.7747	129.2811	127.1786	128.7081	127.1476	128.6869	127.8217	129.2054	**123.2**
**C8`**	37.0450	37.6940	37.8835	37.8772	38.3973	37.8448	38.6069	37.8202	38.5778	37.9226	39.0934	31.5291	31.7000	31.8863	31.8182	32.2538	31.8472	32.4883	31.8311	32.3995	31.8623	32.6089	**31.8**
**H2**	8.7069	8.6784	8.6928	8.6053	8.5454	8.6073	8.5479	8.6098	8.5343	8.6039	8.5372	8.7807	8.6012	8.5696	8.4457	8.4404	8.5036	8.4665	8.5093	8.4619	8.4427	8.4200	**8.24**
**H3**	8.0158	8.0988	8.2272	8.1377	8.2827	8.1368	8.2910	8.1364	8.2841	8.1391	8.3061	7.9920	8.0946	8.2076	8.2048	8.2973	8.1316	8.2916	8.1305	8.2844	8.2068	8.3249	**7.92**
**H5**	7.9494	8.0972	8.1803	8.1686	8.2684	8.1645	8.2725	8.1608	8.2623	8.1730	8.2960	7.9197	8.0617	8.1458	8.0756	8.2233	8.1348	8.2328	8.1318	8.2227	8.0796	8.2598	**7.92**
**H6**	8.2234	8.4837	8.5504	8.8512	8.6199	8.5047	8.6524	8.4954	8.6194	8.5265	8.6775	8.0120	8.1807	8.2580	8.2738	8.4318	8.2652	8.4178	8.2609	8.3913	8.2799	8.4899	**8.24**
**H8**	4.4104	4.5520	4.6020	4.6273	4.6777	4.6180	4.6910	4.6096	4.6575	4.6370	4.7194	4.5352	4.7033	4.7593	4.7525	4.8737	4.7515	4.8659	4.7494	4.8444	4.7564	4.9040	**5.17**
**H(9A-9C)**	1.3045	1.3201	1.3096	1.2974	1.2497	1.2984	1.2666	1.2998	1.2614	1.2969	1.2714	1.3986	1.4265	1.3984	1.4181	1.3894	1.4393	1.4093	1.4389	1.4028	1.4199	1.4150	**1.59**
**H(8`A-8`C)**	2.5039	2.4289	2.4185	2.4392	2.4200	2.4398	2.4123	2.4409	2.4141	2.4391	2.3982	2.9118	2.7517	2.6818	2.6405	2.5916	2.6543	2.5092	2.6599	2.5172	2.6379	2.4728	**2.8**
Nuclei	M06-2X																						
**C1**	160.7812	161.0469	162.5455	161.6717	163.2331	161.5936	163.0308	162.1862	162.9763	161.7362	163.3570	159.4780	161.2884	163.1285	162.8746	164.9031	162.8708	165.0996	162.8652	165.2939	162.8691	165.3736	**137.3**
**C2**	152.3716	152.4344	152.8979	152.2272	152.6934	152.2499	153.0785	152.3390	153.0188	152.1973	153.1001	152.1224	152.2293	153.1677	152.2015	152.6835	152.2268	153.2056	152.2531	153.1595	152.1711	153.0350	**136.5**
**C3**	148.0777	149.1088	149.5307	148.9264	149.9379	148.9295	150.0878	148.9831	150.0285	148.9164	150.1950	148.3996	148.8696	150.7241	148.9722	149.7604	148.9863	150.2854	149.0018	150.2481	148.9523	150.0513	**130.8**
**C4**	157.0834	156.7269	156.8402	156.7636	156.8158	156.7870	157.5708	156.7323	157.4212	156.7358	157.7244	157.2275	156.6756	156.6273	156.5684	156.5103	156.5882	157.1793	156.6098	157.0072	156.5437	157.2822	**127.3**
**C5**	146.2865	147.4241	148.6402	148.5356	149.9022	148.5156	149.9231	148.4517	149.8325	148.5489	150.1218	146.0862	147.5102	147.7038	148.2411	149.9800	148.2162	149.3829	148.1923	149.2666	148.2584	150.0473	**130.8**
**C6**	149.2115	151.2168	152.5585	152.3737	154.3147	152.3414	154.5563	152.1990	154.3608	152.3992	154.9380	149.3500	151.6799	152.2678	152.7029	154.6092	152.6622	154.6326	152.6226	154.3948	152.7349	155.1715	**136.5**
**C7**	219.8379	225.1136	227.1287	228.2419	229.1118	228.0337	235.1503	228.2818	233.7990	228.4332	236.2713	223.2667	228.5162	230.4809	231.5768	232.6758	231.4403	238.7422	231.3023	237.5446	231.6985	239.5725	**196.6**
**C8**	66.6877	67.8725	68.6463	68.4731	69.4288	68.4435	70.0657	68.5768	69.8849	68.4932	70.8457	60.9160	62.6250	63.4930	63.7820	64.7141	63.7606	65.5453	63.7390	65.4520	63.7952	65.9087	**61**
**C9**	22.7187	23.0614	23.2458	23.2276	23.6788	23.2358	23.5684	23.2017	23.5225	23.2166	23.7532	20.1129	20.1977	20.4014	20.3278	20.6954	20.3490	20.7794	20.3710	20.7796	20.3011	21.1513	**15.8**
**C4``**	128.9729	130.1874	131.0653	130.5257	131.9061	130.5242	131.7423	130.5161	131.7167	130.5206	131.8568	129.0341	130.2231	130.9361	130.7024	132.0283	130.6988	131.9000	130.6959	131.8685	130.6991	132.0710	**123.2**
**C8`**	36.1583	36.8117	37.0490	37.1950	37.5165	37.1919	37.7409	37.2318	37.6971	37.1907	38.2470	30.6328	31.0950	31.0595	31.1086	31.3260	31.1080	31.3212	31.1094	31.2246	31.1036	31.5266	**31.8**
**H2**	9.2752	9.2781	9.2443	9.2079	9.2051	9.2147	9.1892	9.1728	9.2001	9.2015	9.1779	9.3141	9.1632	9.1533	9.0260	9.0085	9.0261	8.9735	9.0261	8.9598	9.0263	8.9697	**8.24**
**H3**	8.6139	8.7129	8.7869	8.7471	8.8480	8.7444	8.8596	8.7387	8.8539	8.7497	8.8756	8.6388	8.7165	8.7880	8.7413	8.8344	8.7380	8.8518	8.7344	8.8449	8.7446	8.8630	**7.92**
**H5**	8.5057	8.6790	8.7320	8.7320	8.8378	8.7265	8.8434	8.7173	8.8343	8.7374	8.8672	8.5500	8.6753	8.7083	8.7291	8.8526	8.7238	8.8247	8.7182	8.8128	8.7342	8.8646	**7.92**
**H6**	8.7790	9.0052	9.1051	9.1177	9.2838	9.1126	9.3018	9.0803	9.2876	9.1227	9.3349	8.6506	8.8368	8.8660	8.9036	9.0820	8.8958	9.0507	8.8877	9.0256	8.9113	9.1112	**8.24**
**H8**	4.2826	4.4385	4.4624	4.5326	4.6216	4.5267	4.6533	4.4977	4.6336	4.5388	4.6780	4.5967	4.7305	4.7817	4.7824	4.9132	4.7762	4.9131	4.7698	4.8896	4.7884	4.9457	**5.17**
**H(9A-9C)**	1.3663	1.3956	1.3572	1.3769	1.3484	1.3786	1.3512	1.3596	1.3525	1.3755	1.3638	1.4344	1.4359	1.4105	1.4346	1.4038	1.4314	1.4037	1.4281	1.3976	1.4377	1.4301	**1.59**
**H(8`A-8`C)**	2.4903	2.4194	2.4092	2.4158	2.3835	2.4115	2.3883	2.4317	2.3857	2.4201	2.3743	2.7768	2.6286	2.5753	2.5515	2.5055	2.5519	2.4360	2.5522	2.4378	2.5513	2.4113	**2.8**

[a] methyl protons shifts are averaged
[b] according to [2]

Table S2. Cont. IV

Nuclei	R											S											Exp. [b]
	mpW1LYP																						
	Gas	$CHCl_3$ (PCM)	$CHCl_3$ (SMD)	DMSO (PCM)	DMSO (SMD)	CH_3OH (PCM)	CH_3OH (SMD)	C_2H_5OH (PCM)	C_2H_5OH (SMD)	H_2O (PCM)	H_2O (SMD)	Gas	$CHCl_3$ (PCM)	$CHCl_3$ (SMD)	DMSO (PCM)	DMSO (SMD)	CH_3OH (PCM)	CH_3OH (SMD)	C_2H_5OH (PCM)	C_2H_5OH (SMD)	H_2O (PCM)	H_2O (SMD)	
**C1**	146.3011	146.4224	147.7951	147.6820	151.2162	147.6958	149.5841	147.7119	148.8340	147.6710	149.5731	144.0082	145.9547	147.5148	147.1335	148.8614	147.0617	149.2721	146.9772	149.4582	147.2103	149.4472	**137.3**
**C2**	137.1269	137.0834	137.5504	136.9264	137.2035	136.9488	137.5009	136.9726	137.4156	136.9040	137.4474	136.5419	136.8143	137.6048	136.8271	137.1730	136.9147	137.8074	136.9065	137.7305	136.8297	137.5980	**136.5**
**C3**	133.0432	133.6785	134.5277	133.9494	134.7771	133.9381	134.6612	133.9284	134.7983	133.9577	134.7653	133.0462	133.6460	135.2918	133.8145	134.5268	134.0843	135.1048	134.0604	135.0811	133.8225	134.7247	**130.8**
**C4**	142.6891	142.3187	142.3565	142.2937	141.8367	142.2759	142.8151	142.2594	142.7725	142.3083	142.9353	142.9332	142.2438	142.2003	142.1354	142.0510	142.1359	142.5366	142.1420	142.3943	142.1308	142.6653	**127.3**
**C5**	131.5256	132.8272	133.5462	133.5849	134.9095	133.5293	135.2277	133.4752	134.8319	133.6353	135.2839	131.8034	132.9444	132.9679	133.6763	135.1049	133.3379	134.4725	133.3099	134.4118	133.7163	135.1008	**130.8**
**C6**	133.8544	135.8080	136.7082	136.4859	137.3352	136.4220	137.9905	136.3589	138.1817	136.5439	138.3712	134.2310	136.1799	136.6053	137.1577	138.5996	136.9769	138.6820	136.9226	138.4679	137.2117	139.1059	**136.5**
**C7**	204.1435	208.9718	210.8713	212.8431	215.5762	212.6436	220.2740	212.4482	217.9395	213.0320	220.9021	208.7698	213.2274	215.2815	215.7249	216.9554	215.6428	221.8605	215.4967	220.9247	215.8621	222.4402	**196.6**
**C8**	65.3405	66.3268	67.0108	66.8427	68.0810	66.8366	68.2723	66.8328	68.1115	66.8481	68.9293	59.7565	61.7690	62.4885	62.8117	63.5389	62.6898	64.4543	62.6146	64.3535	62.8823	64.7146	**61**
**C9**	22.6898	23.0014	23.1882	23.0516	23.1193	23.0335	23.2111	23.0178	23.2603	23.0672	23.3444	19.4329	19.6698	19.7280	19.8989	19.9691	19.8615	20.1344	19.8467	20.1180	19.9107	20.4549	**15.8**
**C4``**	133.5566	134.7311	135.7328	135.2303	136.6214	135.2016	136.3970	135.1742	136.4256	135.2554	136.4626	133.4548	134.6985	135.5224	135.1936	136.5468	135.1632	136.4269	135.1339	136.4140	135.2205	136.4564	**123.2**
**C8`**	36.3421	37.0508	37.2997	37.5198	37.8215	37.4840	37.9658	37.4509	37.9881	37.5518	38.3753	31.8478	31.9274	32.0924	32.1118	32.3621	32.1074	32.3640	32.0964	32.3328	32.1213	32.4094	**31.8**
**H2**	8.6559	8.6549	8.6278	8.5206	8.3551	8.5219	8.4612	8.5226	8.5169	8.5189	8.4695	8.7476	8.5595	8.5464	8.4689	8.4508	8.4808	8.4134	8.4878	8.4037	8.4620	8.4033	**8.24**
**H3**	7.9953	8.0939	8.1639	8.1243	8.2189	8.1227	8.2453	8.1206	8.2355	8.1256	8.2588	8.0058	8.0969	8.1508	8.1352	8.2304	8.1259	8.2369	8.1245	8.2303	8.1365	8.2532	**7.92**
**H5**	7.9041	8.0501	8.1136	8.1125	8.1920	8.1082	8.2021	8.1034	8.2032	8.1165	8.2269	7.8973	8.0223	8.0976	8.0831	8.1850	8.0860	8.1827	8.0823	8.1743	8.0857	8.2025	**7.92**
**H6**	8.1307	8.3666	8.4338	8.4377	8.5030	8.4278	8.6044	8.4175	8.5882	8.4466	8.6391	7.9691	8.1345	8.1869	8.2218	8.3751	8.2137	8.3504	8.2100	8.3302	8.2251	8.3959	**8.24**
**H8**	4.3551	4.4794	4.5126	4.5575	4.6447	4.5475	4.7145	4.5370	4.6436	4.5664	4.7365	4.5303	4.6786	4.7462	4.7476	4.8759	4.7482	4.8822	4.7450	4.8668	4.7502	4.9096	**5.17**
**H(9A-9C)**	1.3321	1.3540	1.3262	1.3040	1.2099	1.3061	1.2486	1.3077	1.2890	1.3019	1.2667	1.4215	1.4330	1.4003	1.4518	1.4141	1.4514	1.4203	1.4506	1.4112	1.4523	1.4402	**1.59**
**H(8`A-8`C)**	2.5558	2.4799	2.4688	2.4871	2.4716	2.4893	2.4473	2.4913	2.4542	2.4852	2.4278	2.9797	2.7985	2.7509	2.7098	2.6741	2.7196	2.5777	2.7260	2.5857	2.7040	2.5459	**2.8**
Nuclei	mpW1PW91																						
**C1**	143.6775	143.7517	145.0712	145.0383	147.0466	145.0375	147.4508	145.0422	147.5493	145.0398	146.9032	141.7344	143.5002	145.1332	144.8167	146.6312	144.7477	147.1214	144.6821	147.2314	144.8781	147.2352	**137.3**
**C2**	136.0542	136.0919	136.5570	135.9437	136.3200	135.9637	136.7277	135.9875	136.6637	135.9216	136.5838	135.6181	135.8562	136.7006	135.8799	136.3768	135.8791	136.9015	135.8845	136.8364	135.8801	136.7516	**136.5**
**C3**	131.9531	132.6848	133.5226	132.9283	133.8886	132.9196	134.0313	132.9157	134.0011	132.9344	134.0480	131.9585	132.6033	134.2436	132.7719	133.6291	132.7639	134.1271	132.7614	134.0917	132.7784	133.8757	**130.8**
**C4**	140.7326	140.5294	140.6108	140.5113	140.4711	140.4968	141.0862	140.4896	140.9295	140.5250	141.3562	140.9263	140.4248	140.4138	140.3268	140.3470	140.3314	140.8255	140.3419	140.6750	140.3225	140.9882	**127.3**
**C5**	130.5628	131.8435	132.5777	132.5599	133.8610	132.5118	133.9579	132.4705	133.8692	132.6067	134.1525	130.7440	131.9721	132.0038	132.6590	134.1172	132.6195	133.5873	132.5853	133.5021	132.6959	134.1599	**130.8**
**C6**	132.6202	134.5827	135.5253	135.2716	136.7450	135.2185	136.6493	135.1701	136.4412	135.3201	137.3994	133.0360	135.0018	135.4098	135.9117	137.4654	135.8608	137.4920	135.8146	137.2809	135.9591	137.9659	**136.5**
**C7**	202.2625	206.7780	208.5712	210.3873	211.7261	210.1980	218.0071	210.0218	216.9703	210.5764	217.9186	206.0032	210.5726	212.5449	213.0801	214.2283	212.9380	219.2496	212.7991	218.2288	213.2122	219.7443	**196.6**
**C8**	63.1677	64.1978	64.8164	64.7550	65.6257	64.7497	66.4081	64.7473	66.2313	64.7573	67.0008	57.9642	59.6631	60.4091	60.6813	61.4932	60.6183	62.4089	60.5602	62.2899	60.7395	62.6802	**61**
**C9**	22.0834	22.4018	22.5994	22.4665	22.7324	22.4602	22.6398	22.4596	22.5781	22.4708	22.8588	18.9157	19.2074	19.2625	19.4196	19.6241	19.4114	19.7309	19.4086	19.7091	19.4264	20.1124	**15.8**
**C4``**	130.1889	131.2662	132.1970	131.6747	133.0433	131.6527	132.8682	131.6363	132.8445	131.6945	132.9589	130.1341	131.2651	132.0161	131.6691	132.9895	131.6468	132.8908	131.6297	132.8706	131.6892	132.9353	**123.2**
**C8`**	35.5524	36.2834	36.4651	36.6650	37.0422	36.6365	37.2069	36.6156	37.1572	36.6948	37.6786	30.8808	31.1456	31.2562	31.2890	31.5810	31.2816	31.5853	31.2802	31.5300	31.2961	31.6619	**31.8**
**H2**	8.7045	8.7154	8.6873	8.5759	8.5386	8.5780	8.4913	8.5801	8.4848	8.5738	8.5357	8.7643	8.6062	8.5877	8.5029	8.4874	8.5092	8.4460	8.5159	8.4386	8.4976	8.4443	**8.24**
**H3**	8.0587	8.1621	8.2311	8.1896	8.2951	8.1881	8.3063	8.1867	8.2989	8.1912	8.3303	8.0691	8.1663	8.2190	8.1995	8.2980	8.1981	8.3080	8.1968	8.3004	8.2011	8.3249	**7.92**
**H5**	7.9630	8.1147	8.1778	8.1763	8.2724	8.1720	8.2797	8.1679	8.2701	8.1806	8.3060	7.9605	8.0949	8.1640	8.1533	8.2582	8.1504	8.2570	8.1477	8.2472	8.1562	8.2802	**7.92**
**H6**	8.2149	8.4502	8.5198	8.5109	8.6446	8.5024	8.6424	8.4942	8.6249	8.5195	8.7010	8.0374	8.2199	8.2609	8.2983	8.4549	8.2948	8.4233	8.2913	8.4024	8.3020	8.4774	**8.24**
**H8**	4.3734	4.5005	4.5323	4.5682	4.6533	4.5586	4.7151	4.5495	4.6974	4.5780	4.7166	4.5651	4.7101	4.7707	4.7748	4.9019	4.7718	4.9077	4.7688	4.8883	4.7779	4.9417	**5.17**
**H(9A-9C)**	1.3071	1.3334	1.2996	1.2790	1.2333	1.2818	1.2077	1.2844	1.2005	1.2762	1.2596	1.3921	1.4050	1.3646	1.4166	1.3764	1.4160	1.3836	1.4157	1.3759	1.4175	1.4105	**1.59**
**H(8`A-8`C)**	2.4970	2.4170	2.4027	2.4228	2.3920	2.4256	2.3878	2.4283	2.3876	2.4203	2.3751	2.8634	2.7056	2.6488	2.6124	2.5680	2.6174	2.4766	2.6225	2.4862	2.6082	2.4514	**2.8**
Nuclei	O3LYP																						
**C1**	141.1836	142.7400	144.7813	144.2184	146.2802	144.1968	145.4808	144.2640	145.5812	144.2797	145.6497	138.4959	141.4472	142.5593	142.6679	145.9405	142.7270	144.7837	142.6883	144.7861	142.6851	144.9676	**137.3**
**C2**	131.4397	131.4889	132.2644	131.8082	132.4821	131.8138	132.5709	131.7208	132.6034	131.7954	132.5734	131.1540	131.4322	132.2584	131.3754	131.9766	131.5291	132.3676	131.5305	132.3173	131.3604	132.3268	**136.5**
**C3**	127.8612	128.3961	129.3971	129.1794	130.7180	129.1803	130.3909	128.7868	130.7892	129.1598	130.5140	127.8400	128.5426	130.3396	128.4583	129.7865	128.8913	129.8068	128.8782	129.7650	128.4543	129.9043	**130.8**
**C4**	136.4132	135.8351	135.8460	135.7653	135.7374	135.7520	136.4750	135.7540	136.3443	135.7729	136.5459	136.7030	135.9339	136.0357	135.8282	135.4494	135.7963	136.1345	135.8003	136.0338	135.8155	136.2511	**127.3**
**C5**	126.3016	127.6143	128.3374	127.8450	128.5701	127.8035	129.0516	128.1224	128.5556	127.8864	129.1458	126.6645	127.6228	127.5737	128.6440	129.3611	128.1325	129.3505	128.1013	129.2848	128.6690	129.4945	**130.8**
**C6**	127.6592	129.2225	129.8546	129.9015	130.9261	129.8395	131.4597	129.9361	131.1426	129.9494	131.7250	128.3326	129.8832	130.5409	130.8174	131.5342	130.6026	132.2011	130.5537	132.0382	130.8568	132.4289	**136.5**
**C7**	199.3378	205.1848	207.8598	208.9941	210.4109	208.8298	215.0291	208.7695	214.2122	209.1941	215.6717	202.7281	207.7227	208.9774	210.0470	212.5723	210.0386	215.7368	209.9072	214.7938	210.1540	216.4382	**196.6**
**C8**	63.5509	64.9998	65.7170	65.6909	66.5746	65.6872	67.2126	65.6604	67.0719	65.6939	67.8575	58.5365	60.8631	61.7113	62.0574	63.1139	61.9868	63.8450	61.9390	63.6432	62.0893	64.0165	**61**
**C9**	21.5536	21.7932	21.9663	22.0544	22.2615	22.0402	22.4359	22.0265	22.3402	22.0696	22.5836	18.1798	18.5075	18.7628	18.7014	18.7680	18.6811	19.0418	18.6763	18.9601	18.6957	19.2949	**15.8**
**C4``**	131.5244	132.6294	133.6082	133.0271	134.3315	133.0045	134.2651	133.0784	134.1496	133.0490	134.3169	131.4658	132.6230	133.4807	133.1078	134.5097	133.1175	134.3494	133.0933	134.3222	133.1178	134.4127	**123.2**
**C8`**	35.8915	36.6320	36.9704	37.1226	37.4695	37.1121	37.5623	37.0969	37.5134	37.1135	37.9350	31.4810	31.5404	31.7444	31.7522	32.0722	31.7475	31.9705	31.7311	31.9701	31.7574	32.0236	**31.8**
**H2**	8.4038	8.2843	8.2386	8.1853	8.1710	8.1874	8.2206	8.1783	8.2093	8.1791	8.2247	8.5333	8.3098	8.3603	8.2130	8.1123	8.2214	8.2048	8.2238	8.2033	8.2108	8.2060	**8.24**
**H3**	7.9446	8.0359	8.1015	8.0532	8.1753	8.0498	8.1909	8.0622	8.1861	8.0574	8.2043	7.9626	8.0515	8.1131	8.0909	8.1821	8.0837	8.1932	8.0819	8.1874	8.0923	8.2114	**7.92**
**H5**	7.8530	7.9763	8.0267	8.0293	8.1306	8.0252	8.1438	8.0134	8.1375	8.0331	8.1626	7.8517	7.9709	8.0477	8.0283	8.1193	8.0243	8.1255	8.0208	8.1167	8.0313	8.1494	**7.92**
**H6**	8.0516	8.2251	8.2627	8.2720	8.3793	8.2645	8.4188	8.2678	8.3941	8.2782	8.4440	7.8868	8.0207	8.0821	8.0984	8.1882	8.0807	8.2094	8.0754	8.1959	8.1035	8.2483	**8.24**
**H8**	4.4518	4.5459	4.5421	4.5785	4.6535	4.5722	4.6637	4.5690	4.6572	4.5863	4.6845	4.5975	4.7281	4.7930	4.7909	4.9096	4.7872	4.9084	4.7828	4.8946	4.7947	4.9488	**5.17**
**H(9A-9C)**	1.2134	1.2014	1.1623	1.1786	1.1372	1.1794	1.1808	1.1769	1.1655	1.1764	1.1943	1.3130	1.3368	1.3465	1.3564	1.2868	1.3547	1.3468	1.3530	1.3430	1.3577	1.3596	**1.59**
**H(8`A-8`C)**	2.5244	2.4849	2.4681	2.4873	2.4562	2.4877	2.4515	2.4888	2.4546	2.4874	2.4316	2.9203	2.7421	2.6837	2.6580	2.6204	2.6641	2.5362	2.6674	2.5484	2.6547	2.5105	**2.8**
Nuclei	OLYP																						
**C1**	139.9602	142.1202	143.6629	142.8443	145.6409	142.8247	144.2168	142.8022	144.5979	142.8634	144.3219	137.5022	140.9341	142.4201	141.6150	144.5011	141.6107	143.4280	141.5952	143.4745	141.6043	143.5978	**137.3**
**C2**	129.0985	129.3943	129.9739	129.5799	129.9603	129.5878	130.2659	129.5894	130.1611	129.5609	130.2586	128.9770	129.3066	130.0189	129.3003	129.7427	129.3070	130.0958	129.3077	130.0390	129.2849	130.0356	**136.5**
**C3**	125.8183	126.4055	127.2472	127.1042	127.6922	127.1051	127.9839	127.0979	127.9494	127.0884	128.1240	126.0334	127.0496	128.5779	126.9285	127.7588	126.9213	127.7943	126.9084	127.7484	126.9263	127.8950	**130.8**
**C4**	134.2360	133.5218	133.6917	133.6468	133.2511	133.6439	134.1973	133.6367	133.9875	133.6421	134.3242	134.6346	133.6249	133.8060	133.5807	133.2260	133.5856	133.8951	133.5864	133.7881	133.5683	134.0106	**127.3**
**C5**	124.3149	125.5415	126.4757	125.8444	127.1774	125.8037	127.2441	125.7596	127.1354	125.8787	127.3730	124.5820	125.2320	125.5470	126.1307	127.2817	126.1017	127.3451	126.0673	127.2851	126.1494	127.4852	**130.8**
**C6**	125.3481	126.8775	127.7206	127.6594	128.8246	127.6058	129.2498	127.5467	129.0188	127.7001	129.5341	125.9976	127.2141	127.7780	128.2067	129.2784	128.1507	129.7985	128.0911	129.6341	128.2533	130.0248	**136.5**
**C7**	198.7061	205.0031	207.1463	207.8294	210.2832	207.6757	213.9628	207.5229	213.0854	207.9863	214.3909	202.7830	207.5159	209.6461	209.4435	211.3523	209.3617	214.4687	209.2687	213.6168	209.5061	215.2012	**196.6**
**C8**	64.9955	66.4933	67.2492	67.1658	68.3192	67.1364	68.6159	67.1031	68.7169	67.1901	69.2892	59.5164	62.4587	63.1159	63.4829	64.6565	63.4329	65.3533	63.3772	65.1734	63.5208	65.4493	**61**
**C9**	21.6882	21.9650	22.1779	22.2783	22.5195	22.2700	22.8023	22.2556	22.6416	22.2747	22.8138	18.1987	18.5447	18.7225	18.7771	18.9690	18.7657	19.2330	18.7499	19.1542	18.7801	19.4739	**15.8**
**C4``**	134.9145	136.0601	137.1334	136.5354	137.9918	136.5107	137.7975	136.4810	137.8046	136.5518	137.8754	134.8717	136.0092	137.0114	136.6086	138.0035	136.5871	137.8419	136.5598	137.8153	136.6195	137.8932	**123.2**
**C8`**	36.56895	37.3427	37.6131	37.6993	38.1818	37.6821	38.1865	37.6607	38.0949	37.7101	38.4667	32.0509	32.1657	32.5010	32.4185	32.6442	32.4111	32.5601	32.3981	32.5506	32.4164	32.6271	**31.8**
**H2**	8.2078	8.0715	8.0687	8.0362	7.9736	8.0380	8.0739	8.0395	8.0420	8.0331	8.0802	8.3185	8.0821	8.0927	8.0542	7.9924	8.0540	8.0721	8.0541	8.0658	8.0544	8.0741	**8.24**
**H3**	7.8553	7.9389	8.0214	7.9735	8.0756	7.9711	8.1007	7.9688	8.0917	7.9756	8.1183	7.8838	7.9638	8.0429	8.0096	8.0974	8.0079	8.1147	8.0062	8.1089	8.0108	8.1313	**7.92**
**H5**	7.7659	7.8612	7.9345	7.9375	8.0374	7.9337	8.0587	7.9298	8.0401	7.9408	8.0724	7.7686	7.8814	7.9500	7.9330	8.0250	7.9297	8.0352	7.9263	8.0263	7.9358	8.0582	**7.92**
**H6**	7.9547	8.0913	8.1664	8.1673	8.2311	8.1615	8.2965	8.1557	8.2613	8.1723	8.3396	7.7916	7.8770	7.9503	7.9601	8.0659	7.9547	8.0863	7.9494	8.0709	7.9650	8.1261	**8.24**
**H8**	4.5622	4.5888	4.6023	4.6465	4.6517	4.6426	4.7084	4.6387	4.6846	4.6494	4.7477	4.6763	4.7899	4.8584	4.8610	4.9696	4.8566	4.9758	4.8521	4.9592	4.8648	5.0184	**5.17**
**H(9A-9C)**	1.2077	1.1908	1.1787	1.1926	1.1536	1.1923	1.1937	1.1919	1.1875	1.1923	1.2043	1.2884	1.3277	1.3136	1.3578	1.3099	1.3553	1.3597	1.3528	1.3542	1.3599	1.3702	**1.59**
**H(8`A-8`C)**	2.5653	2.5334	2.5139	2.5254	2.5139	2.5265	2.5013	2.5274	2.5009	2.5239	2.4674	2.9743	2.7841	2.7578	2.7126	2.6563	2.7171	2.5832	2.7214	2.5934	2.7079	2.5580	**2.8**

[a] methyl protons shifts are averaged

[b] according to [2]

Table S2. Cont. V

Nuclei	R											S											Exp. [b]
	Gas	$CHCl_3$ (PCM)	$CHCl_3$ (SMD)	DMSO (PCM)	DMSO (SMD)	CH_3OH (PCM)	CH_3OH (SMD)	C_2H_5OH (PCM)	C_2H_5OH (SMD)	H_2O (PCM)	H_2O (SMD)	Gas	$CHCl_3$ (PCM)	$CHCl_3$ (SMD)	DMSO (PCM)	DMSO (SMD)	CH_3OH (PCM)	CH_3OH (SMD)	C_2H_5OH (PCM)	C_2H_5OH (SMD)	H_2O (PCM)	H_2O (SMD)	
	**OPBE**																						
**C1**	137.1705	139.3531	140.8748	140.1631	142.5639	140.1575	141.3810	140.0883	141.7297	140.1583	141.3492	134.7067	138.4686	139.9807	138.9365	142.0073	138.9275	140.8309	138.9022	141.0985	138.9391	141.1038	**137.3**
**C2**	127.7617	128.1200	128.6511	128.2188	128.7282	128.2367	128.9618	128.2383	128.7878	128.1968	129.1422	127.6513	128.0267	128.7495	128.0062	128.4535	128.0240	128.8323	128.0236	128.7275	127.9840	128.7912	**136.5**
**C3**	124.3807	125.1126	125.8983	125.7515	126.4788	125.7607	126.6838	125.7624	126.4959	125.7388	127.6591	124.5553	125.2614	127.0954	125.4862	126.3248	125.4873	126.3989	125.4706	126.3649	125.4812	126.5224	**130.8**
**C4**	131.9388	131.4820	131.5975	131.4504	131.3346	131.4539	132.1930	131.4423	131.9240	131.4422	132.3309	132.2005	131.4977	131.5545	131.4104	131.0407	131.4272	131.7672	131.4263	131.5951	131.3905	131.8913	**127.3**
**C5**	122.9812	124.2328	125.0742	124.4322	125.7634	124.4046	125.8959	124.3546	125.9314	124.4526	125.4455	123.2533	124.2950	124.2296	124.8077	125.9186	124.7943	126.0436	124.7630	125.9272	124.8168	126.2206	**130.8**
**C6**	123.8307	125.4372	126.2114	126.0669	127.3200	126.0241	127.7150	125.9679	127.5633	126.1029	127.7894	124.4203	125.7670	126.1452	126.6426	127.6163	126.6054	128.1281	126.5500	127.8906	126.6723	128.3575	**136.5**
**C7**	196.3849	202.5087	204.5290	205.1428	207.0013	205.0151	210.8618	204.8288	209.9772	205.2541	211.1105	199.1310	204.5085	206.3201	206.2041	208.2288	206.1145	211.4903	206.0087	210.7194	206.2851	212.1782	**196.6**
**C8**	62.4709	64.0302	64.6985	64.7424	65.6816	64.7349	66.1331	64.6974	66.1717	64.7445	66.8068	57.8242	60.1799	61.0203	61.1110	62.3348	61.0769	62.9027	61.0240	62.7872	61.1368	63.1085	**61**
**C9**	20.9292	21.2927	21.4056	21.5100	21.7296	21.5111	21.9116	21.4904	21.8147	21.5036	22.0506	17.5730	17.9159	18.0716	18.1348	18.2682	18.1374	18.5028	18.1220	18.4151	18.1267	18.8048	**15.8**
**C4``**	130.5332	131.6387	132.5622	131.9557	133.3215	131.9477	133.1551	131.9164	133.1932	131.9577	133.0760	130.5203	131.6580	132.4534	132.0497	133.3431	132.0441	133.1826	132.0205	133.1696	132.0504	133.2688	**123.2**
**C8`**	35.5134	36.3454	36.5158	36.5901	36.9518	36.5905	37.0128	36.5812	36.9266	36.5865	37.3636	30.8398	31.0922	31.1927	31.2282	31.3990	31.2324	31.3836	31.2195	31.3509	31.2200	31.4215	**31.8**
**H2**	8.2340	8.1148	8.1032	8.0690	8.0346	8.0708	8.1186	8.0754	8.0832	8.0668	8.1358	8.3339	8.1013	8.1121	8.0860	8.0136	8.0874	8.0969	8.0880	8.0770	8.0840	8.0950	**8.24**
**H3**	7.9047	7.9952	8.0729	8.0251	8.1370	8.0227	8.1625	8.0185	8.1460	8.0265	8.1810	7.9349	8.0361	8.0896	8.0694	8.1477	8.0682	8.1620	8.0661	8.1617	8.0699	8.1809	**7.92**
**H5**	7.8140	7.9193	7.9869	7.9902	8.0903	7.9866	8.1083	7.9824	8.0863	7.9928	8.1279	7.8184	7.9310	8.0002	7.9874	8.0758	7.9847	8.0903	7.9813	8.0770	7.9893	8.1156	**7.92**
**H6**	8.0050	8.1449	8.2105	8.2001	8.2963	8.1943	8.3508	8.1895	8.3179	8.2050	8.3699	7.8118	7.9244	7.9694	8.0103	8.1006	8.0054	8.1403	7.9997	8.1090	8.0143	8.1780	**8.24**
**H8**	4.5750	4.6169	4.6163	4.6535	4.6849	4.6492	4.7248	4.6435	4.6925	4.6569	4.7507	4.7071	4.8224	4.8825	4.8870	4.9836	4.8835	5.0029	4.8793	4.9746	4.8898	5.0476	**5.17**
**H(9A-9C)**	1.1645	1.1520	1.1319	1.1511	1.1078	1.1516	1.1496	1.1519	1.1411	1.1500	1.1631	1.2584	1.2784	1.2604	1.3115	1.2451	1.3101	1.3038	1.3080	1.2913	1.3119	1.3163	**1.59**
**H(8`A-8`C)**	2.4882	2.4560	2.4281	2.4490	2.4167	2.4506	2.4058	2.4512	2.4095	2.4467	2.3784	2.8231	2.6613	2.5976	2.5866	2.5173	2.5908	2.4581	2.5944	2.4688	2.5822	2.4318	**2.8**
Nuclei	**OPW91**																						
**C1**	137.4597	139.6571	141.1765	140.4597	142.8571	140.4439	141.6692	140.3742	142.0279	140.4515	141.8930	134.9688	138.7552	140.2125	139.1782	142.2827	139.1624	141.0945	139.1309	141.3464	139.1755	141.3625	**137.3**
**C2**	127.9797	128.3398	128.8650	128.4443	128.9343	128.4521	129.1728	128.4444	129.0010	128.4183	129.2461	127.8652	128.2436	128.9631	128.2357	128.6605	128.2429	129.0418	128.2332	128.9504	128.2106	129.0047	**136.5**
**C3**	124.6039	125.3315	126.1131	125.9782	126.6927	125.9772	126.8985	125.9676	126.7142	125.9618	127.1807	124.7724	125.4850	127.3195	125.7202	126.5414	125.7113	126.6139	125.6857	126.5796	125.7104	126.7402	**130.8**
**C4**	132.1909	131.7183	131.8284	131.6944	131.5594	131.6888	132.4210	131.6689	132.1531	131.6818	132.5623	132.4606	131.7467	131.8184	131.6700	131.2796	131.6759	132.0098	131.6652	131.8362	131.6463	132.1379	**127.3**
**C5**	123.1962	124.4561	125.2948	124.6617	125.9811	124.6247	126.1113	124.5671	126.1427	124.6781	125.1629	123.4743	124.5119	124.4563	125.0520	126.1344	125.0281	126.2586	124.9869	126.1569	125.0563	126.4370	**130.8**
**C6**	124.0524	125.6614	126.4310	126.3037	127.5409	126.2513	127.9385	126.1856	127.7829	126.3356	128.1599	124.6525	125.9973	126.3809	126.8753	127.8433	126.8264	128.3623	126.7596	128.1099	126.9030	128.5956	**136.5**
**C7**	196.7274	202.8832	204.9061	205.5281	207.3817	205.3903	211.2558	205.2026	210.3858	205.6361	211.9283	199.5026	204.9103	206.7634	206.6519	208.6291	206.5552	211.8811	206.4436	211.2017	206.7273	212.5756	**196.6**
**C8**	62.6493	64.2129	64.8810	64.9221	65.8580	64.9033	66.3094	64.8572	66.3503	64.9210	67.0023	57.9751	60.3483	61.1057	61.2786	62.4953	61.2347	63.0688	61.1727	62.9503	61.3009	63.2711	**61**
**C9**	20.9903	21.3587	21.4686	21.5829	21.7945	21.5740	21.9756	21.5548	21.8774	21.5726	22.0940	17.6485	17.9941	18.1352	18.2188	18.3457	18.2106	18.5830	18.1850	18.4931	18.2079	18.8831	**15.8**
**C4``**	131.0411	132.1581	133.0850	132.4847	133.8494	132.4664	133.6815	132.4269	133.7194	132.4833	133.7333	131.0250	132.1776	132.9708	132.5794	133.8715	132.5634	133.7093	132.5303	133.6927	132.5760	133.7972	**123.2**
**C8`**	35.5788	36.4142	36.5880	36.6695	37.0215	36.6598	37.0840	36.6392	37.0002	36.6620	37.4600	30.9369	31.1884	31.3183	31.3166	31.4965	31.3104	31.4770	31.2881	31.4411	31.3047	31.5220	**31.8**
**H2**	8.2376	8.1163	8.1047	8.0719	8.0358	8.0731	8.1200	8.0769	8.0843	8.0694	8.1283	8.3398	8.1037	8.1179	8.0926	8.0157	8.0928	8.0997	8.0927	8.0780	8.0907	8.0982	**8.24**
**H3**	7.9051	7.9951	8.0727	8.0259	8.1364	8.0228	8.1619	8.0188	8.1458	8.0269	8.1820	7.9350	8.0360	8.0904	8.0608	8.1473	8.0588	8.1617	8.0567	8.1527	8.0609	8.1805	**7.92**
**H5**	7.8149	7.9194	7.9869	7.9911	8.0901	7.9869	8.1081	7.9825	8.0864	7.9933	8.1347	7.8193	7.9314	8.0022	7.9941	8.0760	7.9907	8.0907	7.9870	8.0786	7.9956	8.1160	**7.92**
**H6**	8.0050	8.1444	8.2103	8.2020	8.2966	8.1956	8.3515	8.1905	8.3180	8.2065	8.3823	7.8141	7.9258	7.9756	8.0220	8.1020	8.0163	8.1416	8.0104	8.1176	8.0256	8.1797	**8.24**
**H8**	4.5746	4.6164	4.6158	4.6549	4.6853	4.6499	4.7254	4.6444	4.6935	4.6578	4.7597	4.7034	4.8187	4.8824	4.8952	4.9811	4.8910	5.0002	4.8866	4.9819	4.8976	5.0449	**5.17**
**H(9A-9C)**	1.1663	1.1536	1.1336	1.1534	1.1093	1.1531	1.1514	1.1530	1.1427	1.1521	1.1644	1.2598	1.2794	1.2615	1.3158	1.2465	1.3136	1.3056	1.3111	1.2943	1.3162	1.3181	**1.59**
**H(8`A-8`C)**	2.4892	2.4573	2.4298	2.4510	2.4185	2.4519	2.4075	2.4523	2.4114	2.4485	2.3848	2.8285	2.6667	2.6107	2.5893	2.5237	2.5929	2.4635	2.5963	2.4721	2.5846	2.4377	**2.8**
Nuclei	**PBE**																						
**C1**	141.8631	143.3996	144.4023	143.4628	145.4879	143.4561	145.4178	143.4610	145.1279	143.4874	145.5867	139.2708	141.5219	143.4102	142.7731	144.9146	142.7178	145.2630	142.6372	145.2443	142.8538	145.4659	**137.3**
**C2**	133.4186	133.6311	134.0721	133.4133	133.9799	133.4308	134.2150	133.4581	134.1386	133.4134	134.2237	132.7759	133.2763	134.2330	133.3136	133.9525	133.3935	134.3866	133.3136	134.5231	133.3361	134.3976	**136.5**
**C3**	129.9155	130.7528	131.7007	131.0070	132.1694	130.9925	132.2060	130.9890	132.1564	131.0382	132.3163	129.9508	130.7042	132.5348	130.8157	131.7360	131.0657	131.8477	130.8099	132.3223	130.8405	131.9693	**130.8**
**C4**	138.7860	138.5046	138.7194	138.5600	138.5740	138.5476	139.3123	138.5468	139.2036	138.5901	139.4665	139.0345	138.5902	138.6141	138.4166	138.5086	138.4027	139.0528	138.4375	138.8754	138.4305	139.1911	**127.3**
**C5**	128.6033	129.9779	130.7274	130.5997	131.9005	130.5547	132.1191	130.5201	132.0328	130.6600	132.3177	128.9825	130.2494	130.1344	130.7821	132.4342	130.4568	132.3849	130.7224	131.6757	130.8339	132.5415	**130.8**
**C6**	130.4794	132.2379	133.4645	133.0987	134.7677	133.0463	134.9776	133.0030	134.8855	133.1652	135.3860	130.8938	132.8695	133.3564	133.7717	135.4639	133.6211	135.7114	133.6776	135.3592	133.8387	136.0397	**136.5**
**C7**	202.7985	208.0403	208.9232	210.4607	212.0494	210.2892	216.8047	210.1344	215.8580	210.6467	217.5814	206.9804	211.3922	213.4617	213.4967	215.0791	213.4114	219.1548	213.2492	218.3341	213.6333	219.6605	**196.6**
**C8**	67.3143	68.7236	69.1465	69.9770	70.1078	68.9677	70.6698	68.9674	70.4564	69.0017	71.4631	61.7677	63.6266	64.4232	64.6359	65.6899	64.5404	66.4416	64.5187	66.2891	64.7123	66.7519	**61**
**C9**	23.0003	23.3850	23.7083	23.5481	23.9889	23.5356	23.9528	23.5348	23.9301	23.5790	24.1831	19.8407	20.3930	20.4366	20.5729	20.8750	20.5480	21.0490	20.5636	21.0175	20.5992	21.5615	**15.8**
**C4``**	140.2034	141.4730	142.4538	141.8314	143.4051	141.8084	143.2287	141.7959	143.2013	141.8707	143.3096	140.1262	141.4196	142.2762	141.7968	143.3315	141.7792	143.1634	141.7603	143.1858	141.8365	143.2506	**123.2**
**C8`**	37.1014	37.8421	37.9986	38.1066	38.5774	38.0827	38.7418	38.0703	38.7064	38.1476	39.2237	32.0004	32.2555	32.3336	32.2971	32.6181	32.2950	32.6818	32.2939	32.6459	32.3228	32.7231	**31.8**
**H2**	8.4576	8.36777	8.4586	8.3482	8.3212	8.3499	8.3239	8.3521	8.3407	8.3470	8.3269	8.5525	8.4135	8.3882	8.3033	8.2705	8.3149	8.2537	8.3155	8.2691	8.2991	8.2561	**8.24**
**H3**	7.8848	7.9803	8.0583	8.0121	8.1140	8.0105	8.1296	8.0094	8.1247	8.0141	8.1479	7.9054	8.0006	8.0563	8.0246	8.1188	8.0195	8.1278	8.0227	8.1255	8.0264	8.1434	**7.92**
**H5**	7.7893	7.9230	8.0029	7.9940	8.0932	7.9897	8.1043	7.9859	8.0962	7.9987	8.1286	7.8007	7.9300	7.9901	7.9773	8.0868	7.9748	8.0862	7.9724	8.0702	7.9806	8.1082	**7.92**
**H6**	8.0993	8.2516	8.3867	8.3644	8.4865	8.3568	8.5054	8.3498	8.5034	8.3723	8.5446	7.8842	8.0572	8.0908	8.1210	8.2687	8.1127	8.2628	8.1145	8.2356	8.1251	8.3036	**8.24**
**H8**	4.6410	4.6918	4.7669	4.7880	4.8517	4.7797	4.8908	4.7723	4.8667	4.7968	4.9231	4.8068	4.9481	5.0044	5.0002	5.1266	4.9997	5.1330	4.9949	5.1207	5.0035	5.1702	**5.17**
**H(9A-9C)**	1.3709	1.3634	1.3719	1.3478	1.3212	1.3499	1.3256	1.3521	1.3321	1.3463	1.3428	1.4738	1.4982	1.4579	1.5012	1.4642	1.5010	1.4814	1.5004	1.4734	1.5026	1.5081	**1.59**
**H(8`A-8`C)**	2.6087	2.5568	2.5036	2.5160	2.4922	2.5187	2.4795	2.5218	2.4777	2.5141	2.4579	2.9833	2.8192	2.7537	2.7177	2.6688	2.7251	2.5838	2.7291	2.5962	2.7134	2.5525	**2.8**
Nuclei	**PBE0**																						
**C1**	143.1162	143.2977	144.6404	144.4795	146.4414	144.4926	146.0592	144.5049	145.8510	144.4668	146.4097	141.2941	143.0511	144.9279	144.3102	145.9402	144.2352	146.6936	144.1611	146.7784	144.3820	146.7562	**137.3**
**C2**	135.7837	135.8343	136.3252	135.7455	136.1677	135.7541	136.4962	135.7638	136.3403	135.7395	136.4205	135.3588	135.6224	136.5017	135.6393	136.0947	135.6335	136.7346	135.6301	136.6126	135.6483	136.5231	**136.5**
**C3**	131.6955	132.4336	133.2786	132.6861	133.6635	132.6700	133.8245	132.6552	133.7000	132.7038	133.8625	131.6976	132.3612	134.0368	132.5253	133.3429	132.5124	133.9498	132.5011	133.8595	132.5405	133.6333	**130.8**
**C4**	140.4830	140.3056	140.3817	140.2842	140.2185	140.2648	141.0889	140.2484	140.9088	140.3066	141.1465	140.6735	140.2088	140.2282	140.1177	140.1591	140.1166	140.6777	140.1178	140.4656	140.1222	140.7939	**127.3**
**C5**	130.3075	131.6292	132.3294	132.2917	133.5018	132.2427	133.7261	132.1957	133.6056	132.3419	133.8592	130.4918	131.7197	131.7438	132.3989	133.8730	132.3544	133.3835	132.3116	133.2385	132.4437	133.9130	**130.8**
**C6**	132.4835	134.4432	135.3932	135.1955	136.6965	135.1265	137.0785	135.0599	136.9046	135.2644	137.3019	132.8916	134.8800	135.3198	135.7838	137.3947	135.7280	137.4465	135.6732	137.1697	135.8389	137.8655	**136.5**
**C7**	201.8665	206.4072	208.1892	209.6696	210.9568	209.5261	216.1894	209.3836	214.8901	209.8082	217.2456	205.6493	210.1743	212.2659	212.6673	213.7386	212.5198	218.8237	212.3722	217.7955	212.8082	219.2669	**196.6**
**C8**	63.0753	64.1043	64.7522	64.6963	65.6215	64.6796	66.2128	64.6630	65.9330	64.7132	66.9059	57.7716	59.4213	60.1473	60.3870	61.1355	60.3198	62.1586	60.2544	61.9990	60.4531	62.3768	**61**
**C9**	22.0745	22.3894	22.5854	22.5293	22.8504	22.5097	22.8273	22.4922	22.7550	22.5512	22.8925	18.9014	19.2082	19.3020	19.4057	19.5967	19.3927	19.7945	19.3813	19.7137	19.4206	20.1343	**15.8**
**C4``**	129.7474	130.8239	131.7444	131.2173	132.5619	131.1911	132.4830	131.1666	132.4014	131.2449	132.5118	129.6964	130.8170	131.5870	131.2078	132.5042	131.1808	132.4835	131.1554	132.4061	131.2360	132.4783	**123.2**
**C8`**	35.4674	36.1857	36.3689	36.5170	36.8975	36.4923	37.1587	36.4697	37.0542	36.5433	37.5564	30.6234	30.9137	31.0079	31.0525	31.3470	31.0410	31.3975	31.0315	31.2792	31.0670	31.4143	**31.8**
**H2**	8.7147	8.7173	8.6873	8.5885	8.5507	8.5882	8.5652	8.5880	8.5746	8.5887	8.5435	8.7671	8.6127	8.5951	8.5129	8.5049	8.5191	8.4552	8.5256	8.4426	8.5069	8.4483	**8.24**
**H3**	8.0553	8.1585	8.2263	8.1848	8.2844	8.1830	8.3080	8.1812	8.2993	8.1862	8.3227	8.0640	8.1588	8.2150	8.1916	8.2875	8.1900	8.3027	8.1883	8.2923	8.1929	8.3153	**7.92**
**H5**	7.9549	8.1064	8.1682	8.1671	8.2608	8.1625	8.2776	8.1578	8.2669	8.1713	8.2985	7.9550	8.0876	8.1567	8.1456	8.2534	8.1424	8.2505	8.1393	8.2379	8.1482	8.2733	**7.92**
**H6**	8.2264	8.4572	8.5229	8.5125	8.6393	8.5038	8.6750	8.4954	8.6680	8.5206	8.7022	8.0392	8.2228	8.2684	8.3016	8.4654	8.2978	8.4308	8.2938	8.4058	8.3049	8.4818	**8.24**
**H8**	4.3669	4.4939	4.5180	4.5458	4.6122	4.5371	4.6572	4.5289	4.6431	4.5540	4.6881	4.5682	4.7145	4.7804	4.7826	4.9085	4.7792	4.9194	4.7757	4.8970	4.7854	4.9494	**5.17**
**H(9A-9C)**	1.3197	1.3409	1.3074	1.3001	1.2578	1.3004	1.2731	1.3008	1.2705	1.2995	1.2751	1.4057	1.4160	1.3801	1.4268	1.3902	1.4261	1.3951	1.4257	1.3842	1.4273	1.4190	**1.59**
**H(8`A-8`C)**	2.5034	2.4217	2.4093	2.4310	2.4023	2.4333	2.3945	2.4355	2.3886	2.4285	2.3771	2.8635	2.7056	2.6441	2.6150	2.5714	2.6197	2.4795	2.6245	2.4852	2.6105	2.4508	**2.8**

[a] methyl protons shifts are averaged

[b] according to [2]

Table S2. Cont. VI

Nuclei	R											S											Exp. [b]
	TPSS																						
	Gas	$CHCl_3$ (PCM)	$CHCl_3$ (SMD)	DMSO (PCM)	DMSO (SMD)	CH_3OH (PCM)	CH_3OH (SMD)	C_2H_5OH (PCM)	C_2H_5OH (SMD)	H_2O (PCM)	H_2O (SMD)	Gas	$CHCl_3$ (PCM)	$CHCl_3$ (SMD)	DMSO (PCM)	DMSO (SMD)	CH_3OH (PCM)	CH_3OH (SMD)	C_2H_5OH (PCM)	C_2H_5OH (SMD)	H_2O (PCM)	H_2O (SMD)	
**C1**	138.4874	138.6401	140.0652	139.8115	141.6833	139.8518	140.8491	139.8948	140.4265	139.7736	141.1525	136.2205	138.0809	139.8089	139.1643	140.5853	139.0922	141.1900	139.0319	140.0996	139.2438	141.3795	**137.3**
**C2**	130.3555	130.3455	130.9630	130.3380	130.7904	130.3579	131.0082	130.3877	130.8814	130.3281	130.9656	129.7085	130.0618	130.9902	130.1591	130.5676	130.1582	131.2788	130.1690	131.3032	130.1707	131.0704	**136.5**
**C3**	126.7618	127.4477	128.3735	127.7101	128.7284	127.7024	128.8162	127.7054	128.1883	127.7275	128.8928	126.7542	127.3701	129.1561	127.6033	128.4227	127.5966	128.9720	127.6013	128.7547	127.6200	128.6506	**130.8**
**C4**	135.5994	135.2656	135.4261	135.2439	135.1511	135.2340	136.0274	135.2371	135.7502	135.2659	136.1231	135.8288	135.1965	135.3258	135.1133	135.1747	135.1233	135.6693	135.1449	136.2418	135.1145	135.8311	**127.3**
**C5**	125.6556	126.8716	127.6285	127.4992	128.6210	127.4593	128.8274	127.4318	127.9136	127.5486	128.9717	125.9826	127.0144	127.1185	127.6052	129.1010	127.5779	128.4905	127.5616	128.7153	127.6414	129.1349	**130.8**
**C6**	127.4328	129.1977	130.1831	129.9871	131.4172	129.9253	131.8798	129.8768	130.3684	130.0588	132.1768	127.7647	129.5289	130.1358	130.5039	132.1055	130.4562	132.1868	130.4187	131.5510	130.5585	132.6150	**136.5**
**C7**	198.5920	202.7828	204.5831	205.7343	207.1045	205.6224	211.3801	205.5167	206.3109	205.8452	212.3667	202.5104	206.5448	208.5961	208.6571	209.7881	208.5301	214.0975	208.4147	208.9620	208.7876	214.5995	**196.6**
**C8**	65.8155	66.7612	67.5340	67.3893	68.3695	67.3846	68.7561	67.3888	67.6305	67.4014	69.4876	60.8765	62.5968	63.3482	63.5790	64.3286	63.5196	65.1597	63.4720	65.1064	63.6461	65.3912	**61**
**C9**	23.6237	23.8996	24.1677	24.1003	24.4389	24.0875	24.5658	24.0866	24.1551	24.1238	24.6959	20.5688	21.0214	21.1212	21.2722	21.4782	21.2648	21.7195	21.2684	23.2193	21.2896	22.1093	**15.8**
**C4``**	137.7788	138.8875	139.9522	139.3580	140.7821	139.3391	140.6054	139.3315	139.8609	139.3859	140.6586	137.7230	138.8767	139.7854	139.3487	140.7126	139.3287	140.6145	139.3199	140.3854	139.3780	140.6202	**123.2**
**C8`**	37.5430	38.1648	38.4275	38.4800	38.8830	38.4626	39.0889	38.4571	38.5831	38.5075	39.5229	33.1386	33.1916	33.4054	33.3412	33.6585	33.3418	33.6704	33.3534	35.2137	33.3509	33.6757	**31.8**
**H2**	8.4831	8.4885	8.4688	8.3752	8.3439	8.3732	8.3901	8.3725	8.2124	8.3783	8.3726	8.5639	8.4194	8.4047	8.3249	8.3289	8.3317	8.2944	8.3392	8.4499	8.3189	8.2746	**8.24**
**H3**	7.8383	7.9432	8.0169	7.9713	8.0724	7.9691	8.0931	7.9676	7.8059	7.9738	8.1075	7.8492	7.9458	8.0094	7.9788	8.0799	7.9772	8.0911	7.9762	8.0896	7.9807	8.1012	**7.92**
**H5**	7.7486	7.8970	7.9648	7.9568	8.0557	7.9518	8.0732	7.9475	7.7857	7.9620	8.0916	7.7600	7.8918	7.9556	7.9473	8.0616	7.9444	8.0474	7.9419	8.0555	7.9505	8.0756	**7.92**
**H6**	7.9880	8.2148	8.2769	8.2724	8.3956	8.2628	8.4566	8.2542	8.0936	8.2825	8.4864	7.8034	7.9859	8.0380	8.0684	8.2400	8.0645	8.2144	8.0610	8.1738	8.0725	8.2631	**8.24**
**H8**	4.5160	4.6256	4.6486	4.6692	4.7290	4.6608	4.7842	4.6536	4.4786	4.6781	4.8076	4.6565	4.8048	4.8679	4.8726	4.9986	4.8689	5.0142	4.8657	5.0024	4.8764	5.0440	**5.17**
**H(9A-9C)**	1.3674	1.3877	1.3661	1.3612	1.3285	1.3608	1.3535	1.3611	1.1729	1.3623	1.3613	1.4597	1.4867	1.4510	1.4999	1.4790	1.4994	1.4828	1.4994	1.6613	1.5010	1.5074	**1.59**
**H(8`A-8`C)**	2.5573	2.4706	2.4641	2.4810	2.4598	2.4838	2.4370	2.4869	2.3032	2.4784	2.4181	2.9545	2.7853	2.7366	2.6999	2.6676	2.7052	2.5726	2.7113	2.8641	2.6954	2.5405	**2.8**
Nuclei	**VSXC**																						
**C1**	135.5320	136.1083	136.8637	136.2826	137.4077	136.2774	137.1255	136.2699	137.0028	136.2885	137.1077	135.7904	137.5107	138.1956	137.8330	139.2979	137.9754	139.3047	137.9630	139.6696	137.8585	139.4916	**137.3**
**C2**	129.3157	129.4736	129.9493	129.4494	129.8698	129.4551	129.8004	129.4584	130.1343	129.4454	130.2937	129.1972	129.3664	129.9198	128.9784	129.4637	129.3870	130.2399	129.3877	130.1862	128.9796	129.7951	**136.5**
**C3**	125.2117	125.7702	126.6038	125.9248	126.9657	125.9213	125.6262	125.9150	127.0602	125.9296	127.0892	126.2216	126.6147	127.5239	124.5753	125.5735	126.7396	127.8493	126.7316	127.7586	124.5965	125.7305	**130.8**
**C4**	135.3713	135.1595	135.2035	135.2960	135.2445	135.2872	135.9584	135.2767	135.8365	135.3064	136.1458	135.2871	134.8722	134.9284	135.3574	135.2907	134.9218	135.4560	134.9153	135.2984	135.3635	136.0927	**127.3**
**C5**	121.9823	123.5127	124.3640	124.3347	125.4634	124.2878	128.0607	124.2388	125.4072	124.3807	125.6829	121.5537	122.8415	123.6747	125.8259	126.9191	123.5476	124.7157	123.5007	124.5929	125.8586	126.9858	**130.8**
**C6**	127.7647	129.5771	130.5541	130.5765	132.0581	130.5182	132.5500	130.4575	132.2802	130.6335	132.8392	128.0349	130.1858	131.1601	131.3995	132.9642	131.1499	133.0889	131.0905	132.8624	131.4521	133.5690	**136.5**
**C7**	192.2113	196.2843	197.5772	198.3240	199.2710	198.2056	202.9130	198.0847	202.1073	198.4373	203.6553	194.1043	198.2719	199.6023	200.2182	201.2591	200.1943	204.9444	200.0763	204.2310	200.3315	205.5324	**196.6**
**C8**	71.3941	72.2540	72.8585	72.5622	73.4140	72.5483	73.7226	72.5321	73.6227	72.5767	74.3146	63.7285	64.8944	65.5445	65.5908	66.5560	65.4844	66.8116	65.4488	66.7643	65.6271	67.3515	**61**
**C9**	26.9127	27.8221	28.1721	28.4757	29.0419	28.4366	29.3216	28.3957	29.2968	28.5146	29.4164	28.2922	28.3244	28.2994	28.3788	28.5289	28.4052	28.4778	28.4007	28.4742	28.3840	28.7185	**15.8**
**C4``**	135.5317	136.7335	137.6571	137.2377	138.6073	137.2117	138.3820	137.1832	138.3972	137.2640	138.5237	135.7401	136.9252	137.8376	137.2008	138.5060	137.3761	138.5740	137.3505	138.5691	137.2248	138.4294	**123.2**
**C8`**	40.5707	40.9800	41.1265	41.1967	41.3611	41.1869	41.4015	41.1749	41.2908	41.2076	41.7454	35.5106	35.8394	36.1088	36.2260	36.5003	36.1191	36.4555	36.0951	36.2952	36.2453	36.8886	**31.8**
**H2**	8.7582	8.7052	8.7087	8.6706	8.6653	8.6728	8.6045	8.6752	8.6636	8.6684	8.6626	8.7140	8.5943	8.6061	8.4823	8.4696	8.5515	8.5281	8.5536	8.5220	8.4797	8.4460	**8.24**
**H3**	7.9427	8.0319	8.1219	8.0555	8.1795	8.0546	8.1119	8.0537	8.1835	8.0563	8.1992	7.9927	8.0899	8.1784	7.9874	8.0670	8.1147	8.2500	8.1138	8.2470	7.9891	8.0908	**7.92**
**H5**	7.7953	7.9388	7.9985	8.0111	8.1090	8.0069	8.1888	8.0027	8.1059	8.0151	8.1415	7.8402	7.9674	8.0357	8.0601	8.1881	8.0319	8.1306	8.0277	8.1157	8.0635	8.2126	**7.92**
**H6**	8.1822	8.3666	8.4536	8.4649	8.6205	8.4589	8.6981	8.4531	8.6426	8.4705	8.6960	7.8519	8.0504	8.1544	8.1973	8.3615	8.1500	8.3438	8.1435	8.3146	8.2031	8.4172	**8.24**
**H8**	4.4313	4.4994	4.5225	4.5633	4.6386	4.5590	4.6539	4.5548	4.6453	4.5674	4.6896	4.7033	4.8678	4.9239	4.9588	5.0770	4.9551	5.1062	4.9498	5.0899	4.9646	5.1360	**5.17**
**H(9A-9C)**	1.6030	1.6168	1.6077	1.6168	1.6004	1.6169	1.6244	1.6171	1.6161	1.6166	1.6302	1.7817	1.7716	1.7670	1.7683	1.7542	1.7863	1.7833	1.7851	1.7721	1.7694	1.7810	**1.59**
**H(8`A-8`C)**	2.7295	2.6787	2.6488	2.6592	2.6160	2.6602	2.5999	2.6614	2.6029	2.6582	2.5741	2.8584	2.7230	2.6927	2.6715	2.6207	2.6910	2.6028	2.6921	2.5929	2.6699	2.5358	**2.8**
Nuclei	**wB97**																						
**C1**	143.9410	144.0359	145.4039	144.9644	147.0502	145.0037	146.2819	145.0327	145.8015	144.9159	146.5497	142.7671	144.3174	145.9622	145.4512	147.5684	137.3985	147.5404	145.2738	147.6441	145.5026	147.7260	**137.3**
**C2**	136.6884	136.6565	137.1174	136.5369	136.9387	136.5406	137.2677	136.5439	137.2066	136.5339	137.2596	136.4760	136.5383	137.3048	136.4391	136.8418	129.3931	137.2952	136.4387	137.2511	136.4397	137.1025	**136.5**
**C3**	132.8553	133.3920	134.2739	133.6457	134.6750	133.6380	134.6782	133.6282	134.5420	133.6513	134.7684	132.8922	133.3673	135.0791	133.5222	134.3936	128.0421	134.8855	133.4979	134.8784	133.5325	134.5769	**130.8**
**C4**	141.4346	141.0833	141.1483	141.0269	140.9689	141.0102	141.6927	140.9950	141.6451	141.0450	141.8132	141.6206	141.0401	141.0423	140.9044	140.8447	130.0193	141.3475	140.9196	141.2085	140.9026	141.4248	**127.3**
**C5**	131.3432	132.5448	133.2637	133.2367	134.4359	133.1949	134.4607	133.1525	134.4295	133.2770	134.6278	131.3341	132.5796	132.6244	133.2789	134.6998	128.0424	134.1661	133.1945	134.0689	133.3183	134.7169	**130.8**
**C6**	133.6351	135.5124	136.4089	136.2730	137.6498	136.1982	138.0148	136.1261	138.0013	136.3488	138.3322	134.0044	135.9014	136.2252	136.7447	138.1142	129.3273	138.1065	136.6595	137.8836	136.7918	138.5940	**136.5**
**C7**	201.4912	206.1891	208.0118	209.3481	210.6130	209.2325	215.6752	209.1082	214.0834	209.4485	216.7392	204.6947	209.3963	211.3874	212.1390	213.3397	212.0885	218.5298	211.7964	217.4508	212.2863	219.1963	**196.6**
**C8**	61.5724	62.4646	63.1724	63.0682	63.9439	63.0540	64.3441	63.0374	64.1035	63.0797	64.9694	56.1153	57.5729	58.2689	58.5246	59.4252	50.0648	60.1639	58.3778	60.0598	58.5781	60.4430	**61**
**C9**	21.7679	21.8798	22.0516	22.0084	22.3096	21.9907	22.1547	21.9738	22.1655	22.0276	22.2617	19.3435	19.4051	19.5094	19.4841	19.7597	13.2611	19.7753	19.4630	19.7773	19.4894	20.1203	**15.8**
**C4``**	130.8539	131.8504	132.7873	132.2680	133.5885	132.2444	133.4289	132.2197	133.3886	132.2905	133.5129	130.7582	131.8047	132.5348	132.2228	133.5296	137.4366	133.4132	132.1711	133.3861	132.2466	133.4794	**123.2**
**C8`**	35.2078	35.7488	36.0278	36.1523	36.5421	36.1366	36.6761	36.1195	36.6219	36.1669	37.1152	30.4674	30.8523	30.9043	30.9239	31.1102	22.9410	31.0977	30.9223	31.0190	30.9349	31.2156	**31.8**
**H2**	8.8346	8.8195	8.7758	8.6849	8.6395	8.6820	8.6672	8.6800	8.7144	8.6890	8.6583	8.8685	8.7172	8.6770	8.5838	8.5466	7.6951	8.5093	8.6015	8.5033	8.5791	8.5095	**8.24**
**H3**	8.1537	8.2420	8.3086	8.2688	8.3737	8.2677	8.3885	8.2665	8.3819	8.2699	8.4066	8.1681	8.2591	8.2999	8.2873	8.3846	7.2220	8.3890	8.2842	8.3816	8.2890	8.4090	**7.92**
**H5**	8.0502	8.1961	8.2583	8.2574	8.3538	8.2528	8.3642	8.2481	8.3571	8.2619	8.3892	8.0530	8.1792	8.2620	8.2294	8.3280	7.2027	8.3537	8.2237	8.3453	8.2323	8.3536	**7.92**
**H6**	8.2797	8.5178	8.5808	8.5700	8.6914	8.5605	8.7237	8.5515	8.7404	8.5797	8.7629	8.1381	8.3239	8.3574	8.3934	8.5333	7.2635	8.5225	8.3864	8.5007	8.3979	8.5686	**8.24**
**H8**	4.1375	4.2899	4.3068	4.3473	4.4211	4.3379	4.4707	4.3289	4.4686	4.3566	4.5007	4.3612	4.5170	4.5838	4.5799	4.7095	3.6580	4.7190	4.5738	4.6969	4.5835	4.7461	**5.17**
**H(9A-9C)**	1.3569	1.3751	1.3377	1.3396	1.2951	1.3386	1.3076	1.3379	1.3211	1.3410	1.3171	1.4204	1.4276	1.3839	1.4227	1.3801	1.5219	1.3913	1.4217	1.3864	1.4240	1.4149	**1.59**
**H(8`A-8`C)**	2.4966	2.4144	2.4057	2.4316	2.4125	2.4342	2.3962	2.4364	2.3811	2.4289	2.3832	2.8146	2.6459	2.5928	2.5660	2.5155	1.7253	2.4374	2.5758	2.4440	2.5629	2.4184	**2.8**
Nuclei	**wB97X**																						
**C1**	145.1605	145.2874	146.6458	146.2848	148.3997	146.3204	147.6708	146.3479	147.1264	146.2434	147.9985	143.8236	145.5472	147.2426	146.6171	148.8015	146.5265	148.8718	146.4389	148.9067	146.6848	148.9849	**137.3**
**C2**	137.3527	137.3402	137.8108	137.2594	137.6597	137.2669	138.0192	137.2733	137.8915	137.2526	137.9936	137.1254	137.2618	138.0227	137.1496	137.5673	137.2629	138.0379	137.1566	137.9270	137.1482	137.8498	**136.5**
**C3**	133.4276	134.0238	134.8776	134.2799	135.2601	134.2719	135.3172	134.2621	135.1310	134.2870	135.3986	133.4658	133.9831	135.6436	134.1299	134.9988	134.3788	135.4826	134.1097	135.4033	134.1392	135.2111	**130.8**
**C4**	142.5545	142.2510	142.3196	142.2159	142.1420	142.2011	142.9238	142.1872	142.8146	142.2330	143.0259	142.7516	142.1904	142.2084	142.0818	142.0167	142.1031	142.5045	142.1003	142.3048	142.0766	142.6323	**127.3**
**C5**	131.9459	133.1669	133.8635	133.8409	135.0437	133.8000	135.1225	133.7590	135.0464	133.8817	135.2819	131.9543	133.1935	133.2524	133.9199	135.3126	133.6154	134.8390	133.8397	134.6845	133.9584	135.3864	**130.8**
**C6**	134.1955	136.0773	136.9789	136.8241	138.2192	136.7529	138.6486	136.6834	138.5467	136.8956	138.9343	134.6000	136.5057	136.8422	137.3690	138.7164	137.2164	138.7678	137.2920	138.4934	137.4094	139.2412	**136.5**
**C7**	203.2519	208.0315	209.8243	211.2359	212.5253	211.1137	217.7110	210.9856	216.0652	211.3471	218.8212	206.6854	211.4650	213.5088	214.1355	215.3861	213.9920	220.6343	213.7946	219.4764	214.2880	221.2353	**196.6**
**C8**	62.6717	63.5832	64.2841	64.1815	65.0810	64.1711	65.5597	64.1578	65.1930	64.1901	66.2196	57.1385	58.6964	59.4150	59.6548	60.5813	59.5586	61.3892	59.5134	61.2155	59.7154	61.6555	**61**
**C9**	21.9214	22.1150	22.2952	22.2375	22.5337	22.2185	22.4488	22.2000	22.3983	22.2583	22.5383	19.3739	19.5137	19.5802	19.5944	19.8483	19.5884	19.8823	19.5835	19.8211	19.5987	20.2115	**15.8**
**C4``**	130.8431	131.8615	132.8010	132.2791	133.6047	132.2571	133.4769	132.2343	133.3792	132.3004	133.5480	130.7294	131.7941	132.5116	132.2220	133.5289	132.1959	133.4436	132.1749	133.3557	132.2444	133.5004	**123.2**
**C8`**	35.4871	36.0765	36.3435	36.4510	36.8380	36.4354	37.0124	36.4189	36.8840	36.4666	37.4685	30.6384	30.9820	31.0644	31.1112	31.2987	31.1161	31.3580	31.1143	31.2203	31.1159	31.4703	**31.8**
**H2**	8.8025	8.7887	8.7493	8.6612	8.6140	8.6590	8.6421	8.6575	8.6888	8.6642	8.6248	8.8443	8.6875	8.6457	8.5670	8.5201	8.5812	8.4874	8.5855	8.4797	8.5604	8.4859	**8.24**
**H3**	8.1223	8.2163	8.2830	8.2437	8.3449	8.2423	8.3620	8.2409	8.3538	8.2452	8.3780	8.1326	8.2233	8.2703	8.2554	8.3515	8.2458	8.3578	8.2526	8.3479	8.2570	8.3762	**7.92**
**H5**	8.0155	8.1643	8.2254	8.2272	8.3216	8.2227	8.3340	8.2182	8.3251	8.2316	8.3568	8.0247	8.1525	8.2358	8.2048	8.3026	8.2165	8.3159	8.1997	8.3041	8.2075	8.3260	**7.92**
**H6**	8.2495	8.4895	8.5515	8.5406	8.6641	8.5313	8.7017	8.5225	8.7140	8.5502	8.7358	8.0998	8.2925	8.3290	8.3625	8.5004	8.3545	8.4880	8.3573	8.4645	8.3659	8.5363	**8.24**
**H8**	4.1938	4.3388	4.3570	4.3972	4.4693	4.3883	4.5119	4.3798	4.5145	4.4064	4.5381	4.3991	4.5576	4.6261	4.6256	4.7504	4.6262	4.7598	4.6204	4.7354	4.6286	4.7870	**5.17**
**H(9A-9C)**	1.3393	1.3564	1.3216	1.3226	1.2759	1.3221	1.2923	1.3218	1.2993	1.3234	1.2983	1.4088	1.4216	1.3744	1.4202	1.3715	1.4218	1.3834	1.4207	1.3753	1.4207	1.4035	**1.59**
**H(8`A-8`C)**	2.5051	2.4246	2.4155	2.4403	2.4174	2.4428	2.4029	2.4451	2.3851	2.4378	2.3878	2.8360	2.6696	2.6170	2.5932	2.5394	2.6002	2.4591	2.6032	2.4647	2.5894	2.4357	**2.8**

[a] methyl protons shifts are averaged

[b] according to [2]

Table S2. Cont. VII

Nuclei	R											S											Exp. [b]
	X3LYP																						
	Gas	$CHCl_3$ (PCM)	$CHCl_3$ (SMD)	DMSO (PCM)	DMSO (SMD)	CH_3OH (PCM)	CH_3OH (SMD)	C_2H_5OH (PCM)	C_2H_5OH (SMD)	H_2O (PCM)	H_2O (SMD)	Gas	$CHCl_3$ (PCM)	$CHCl_3$ (SMD)	DMSO (PCM)	DMSO (SMD)	CH_3OH (PCM)	CH_3OH (SMD)	C_2H_5OH (PCM)	C_2H_5OH (SMD)	H_2O (PCM)	H_2O (SMD)	
**C1**	146.1048	146.2369	147.6127	147.5146	151.0595	147.5332	148.8151	147.5504	149.9356	147.5056	149.1366	143.8300	145.7814	147.3530	146.9782	148.7186	146.9158	149.1537	146.8328	149.3218	147.0535	149.3399	**137.3**
**C2**	137.0373	137.0277	137.5002	136.8751	137.2093	136.9037	137.4856	136.9291	137.5213	136.8525	137.4389	136.4594	136.7592	137.5642	136.7741	137.1681	136.8676	137.8025	136.8601	137.7223	136.7767	137.6064	**136.5**
**C3**	133.0047	133.6828	134.5301	133.9479	134.8150	133.9424	134.8864	133.9338	134.8679	133.9566	134.9368	133.0208	133.6394	135.2851	133.8048	134.5558	134.0777	135.1303	134.0542	135.0993	133.8121	134.7673	**130.8**
**C4**	142.5362	142.2018	142.2431	142.1747	141.7671	142.1630	142.8068	142.1483	142.4849	142.1886	142.9070	142.7859	142.1419	142.1046	142.0293	141.9873	142.0372	142.4740	142.0451	142.3292	142.0244	142.6163	**127.3**
**C5**	131.5122	132.8420	133.5624	133.5930	134.9355	133.5433	134.9424	133.4904	134.9801	133.6434	135.1484	131.8062	132.9622	132.9776	133.6789	135.1498	133.3494	134.5121	133.3253	134.4438	133.7176	135.1577	**130.8**
**C6**	133.7279	135.7001	136.6102	136.3878	137.3022	136.3306	138.2601	136.2695	137.5618	136.4449	138.5382	134.1213	136.0951	136.5285	137.0664	138.5591	136.8965	138.6351	136.8452	138.4184	137.1194	139.0739	**136.5**
**C7**	204.4817	209.2686	211.1566	213.1239	215.8606	212.9312	219.4265	212.7378	219.7934	213.3125	220.5408	209.0137	213.4876	215.5325	215.9775	217.2252	215.8995	222.1035	215.7551	221.1703	216.1138	222.6794	**196.6**
**C8**	65.6184	66.6266	67.3076	67.1501	68.4234	67.1504	68.6635	67.1486	68.6197	67.1554	69.3636	59.9737	62.0021	62.7278	63.0537	63.8054	62.9386	64.7375	62.8647	64.6262	63.1237	65.0136	**61**
**C9**	22.6919	23.0326	23.2213	23.0869	23.1937	23.0748	23.3540	23.0607	23.2188	23.1026	23.4795	19.4270	19.7103	19.7755	19.9555	20.0612	19.9248	20.2351	19.9112	20.2107	19.9669	20.5801	**15.8**
**C4``**	134.5945	135.7800	136.7805	136.2700	137.7026	136.2481	137.5154	136.2227	137.4899	136.2941	137.5760	134.5034	135.7578	136.5792	136.2420	137.6319	136.2196	137.5040	136.1925	137.4868	136.2677	137.5454	**123.2**
**C8`**	36.4173	37.1488	37.3817	37.6027	37.9307	37.5728	38.1275	37.5408	38.0390	37.6343	38.5586	31.8764	31.9835	32.1379	32.1549	32.4329	32.1572	32.4332	32.1484	32.3981	32.1635	32.4900	**31.8**
**H2**	8.6344	8.6361	8.6084	8.5004	8.3388	8.5018	8.4863	8.5023	8.4090	8.4986	8.4717	8.7230	8.5409	8.5275	8.4504	8.4342	8.4626	8.3974	8.4693	8.3881	8.4438	8.3880	**8.24**
**H3**	7.9838	8.0829	8.1528	8.1130	8.2066	8.1113	8.2292	8.1091	8.2183	8.1144	8.2454	7.9957	8.0863	8.1412	8.1241	8.2188	8.1158	8.2269	8.1142	8.2199	8.1255	8.2432	**7.92**
**H5**	7.8904	8.0376	8.1010	8.0999	8.1817	8.0954	8.2002	8.0904	8.1874	8.1041	8.2229	7.8876	8.0119	8.0866	8.0724	8.1770	8.0747	8.1726	8.0707	8.1638	8.0752	8.1952	**7.92**
**H6**	8.1295	8.3641	8.4302	8.4344	8.4969	8.4246	8.5909	8.4140	8.5554	8.4433	8.6264	7.9604	8.1272	8.1788	8.2132	8.3675	8.2051	8.3427	8.2013	8.3225	8.2165	8.3898	**8.24**
**H8**	4.3939	4.5153	4.5467	4.5924	4.6784	4.5824	4.6910	4.5717	4.7180	4.6015	4.7345	4.5641	4.7106	4.7778	4.7787	4.9072	4.7792	4.9140	4.7757	4.8986	4.7815	4.9439	**5.17**
**H(9A-9C)**	1.3332	1.3553	1.3274	1.3051	1.2139	1.3072	1.2925	1.3086	1.2352	1.3030	1.2952	1.4181	1.4326	1.4000	1.4522	1.4153	1.4518	1.4221	1.4507	1.4129	1.4528	1.4438	**1.59**
**H(8`A-8`C)**	2.5536	2.4759	2.4648	2.4823	2.4686	2.4845	2.4511	2.4864	2.4494	2.4805	2.4328	2.9749	2.7962	2.7470	2.7065	2.6712	2.7163	2.5736	2.7224	2.5818	2.7010	2.5432	**2.8**

[a] methyl protons shifts are averaged

[b] according to [2]

Table S3. Theoretical chemical shifts (in ppm) of *(R,S)*-3-fluoromethcathinone calculated with HF and selected functionals combined with 6-311++G** basis set[a]

Nuclei	R											S											Exp. [b]
	B3LYP																						
	Gas	$CHCl_3$ (PCM)	$CHCl_3$ (SMD)	DMSO (PCM)	DMSO (SMD)	CH_3OH (PCM)	CH_3OH (SMD)	C_2H_5OH (PCM)	C_2H_5OH (SMD)	H_2O (PCM)	H_2O (SMD)	Gas	$CHCl_3$ (PCM)	$CHCl_3$ (SMD)	DMSO (PCM)	DMSO (SMD)	CH_3OH (PCM)	CH_3OH (SMD)	C_2H_5OH (PCM)	C_2H_5OH (SMD)	H_2O (PCM)	H_2O (SMD)	
**C1**	145.9125	146.4378	146.4912	146.3710	147.9538	146.3708	146.9562	146.3695	147.1512	146.3678	146.9965	144.5358	145.5782	146.1256	145.9704	147.3342	145.9419	147.2635	145.9147	147.1982	145.9972	147.3747	**134.46**
**C2**	122.9260	122.3003	122.7525	121.8359	122.3217	121.8605	122.7793	121.8852	122.7064	121.8107	122.7318	122.4060	122.0652	122.5169	121.7563	122.2625	121.7710	122.8409	121.7860	122.7190	121.7412	122.8223	**115.52**
**C3**	173.9878	174.3260	175.0099	174.3733	175.3361	174.3667	175.2414	174.3598	175.2239	174.3780	175.2902	174.1815	174.4177	175.1129	174.4036	175.3334	174.3972	175.2330	174.3906	175.2220	174.4083	175.2751	**162.83**
**C4**	125.5986	127.1790	127.8855	127.9137	128.3905	127.8643	129.5774	127.8149	129.2812	127.9588	129.7748	125.8762	127.0814	127.7667	127.5997	128.1588	127.5657	128.9174	127.5310	128.7210	127.6297	129.0948	**122.41**
**C5**	134.9964	136.9996	137.6291	137.9445	138.8915	137.8832	139.0197	137.8214	138.8955	138.0008	139.1862	135.1978	137.0408	137.7110	137.9261	138.8510	137.8705	138.8594	137.8142	138.7663	137.9770	139.0276	**131.43**
**C6**	128.9977	130.9805	132.4300	132.1085	133.6801	132.0337	134.3057	131.9596	133.9950	132.1790	134.7081	129.2939	131.5219	132.8127	132.6150	134.3602	132.5486	134.6859	132.4815	134.4831	132.6760	135.0257	**125.17**
**C7**	203.7081	209.3132	210.1408	211.9227	212.8230	211.7560	217.8151	211.5884	216.7697	212.0766	218.5808	208.7432	213.1237	214.5711	215.3499	216.1919	215.2135	220.9591	215.0765	219.9013	215.4780	221.5020	**196.61**
**C8**	65.7615	66.9869	67.4023	67.3686	68.1466	67.3491	68.5355	67.3279	68.3865	67.3842	69.2181	60.1689	62.1821	62.5942	63.0877	63.8787	63.0292	64.6529	62.9715	64.3784	63.1421	64.9851	**59.97**
**C9**	22.9207	23.1873	23.5297	23.4084	23.6757	23.3908	23.7957	23.3734	23.7131	23.4239	23.9365	19.5719	19.9819	20.0777	20.1398	20.4102	20.1253	20.5600	20.1110	20.4657	20.1521	20.9193	**15.26**
**C8`**	36.4904	37.3464	37.5061	37.6810	38.0283	37.6565	38.2354	37.6311	38.1780	37.7017	38.6712	32.0251	32.2650	32.6135	32.4119	32.7000	32.3979	32.7740	32.3836	32.7822	32.4230	32.8216	**31.09**
**H2**	8.2255	8.1131	8.2462	8.0580	8.09478	8.0611	8.1523	8.0643	8.1429	8.0550	8.1549	8.2965	8.1093	8.1864	8.0062	8.0578	8.0115	8.0358	8.0167	8.0461	8.0008	8.0364	**7.66**
**H4**	7.3391	7.5398	7.6288	7.6203	7.7378	7.6153	7.7867	7.6103	7.7670	7.6247	7.8114	7.3521	7.5331	7.6255	7.5964	7.7169	7.5921	7.7487	7.5878	7.7332	7.6001	7.7719	**7.33**
**H5**	7.4816	7.6920	7.7462	7.7871	7.8655	7.7812	7.8824	7.7752	7.8679	7.7925	7.9089	7.4810	7.6739	7.7262	7.7642	7.8413	7.7588	7.8462	7.7533	7.8345	7.7690	7.8708	**7.45**
**H6**	7.8000	7.9984	8.1341	8.1125	8.2458	8.1048	8.3087	8.0972	8.2827	8.1197	8.3510	7.6283	7.8139	7.9279	7.9126	8.0785	7.9068	8.0769	7.9010	8.0644	7.9177	8.1193	**7.57**
**H8**	4.3672	4.4656	4.5210	4.5488	4.6239	4.5421	4.6709	4.5354	4.6545	4.5552	4.6968	4.5097	4.6815	4.7418	4.7560	4.8756	4.7515	4.8938	4.7469	4.8801	4.7600	4.9230	**4.92**
**H(9A-9C)**	1.3188	1.3068	1.3225	1.2998	1.2629	1.3001	1.2906	1.3005	1.2800	1.2994	1.3118	1.3894	1.4246	1.4101	1.4498	1.4220	1.4482	1.4406	1.4464	1.4323	1.4513	1.4655	**1.44**
**H(8`A-8`C)**	2.521	2.47031	2.4174	2.4506	2.4187	2.4514	2.4028	2.4523	2.4032	2.4496	2.3861	2.9624	2.7956	2.7637	2.7148	2.6725	2.7189	2.5876	2.7229	2.6029	2.7107	2.5545	**2.64**
**F3**	-131.1490	-134.4920	-136.9050	-135.7630	-139.2430	-135.6910	-138.8130	-135.6180	-138.7680	-135.8310	-138.7900	-130.9960	-134.3940	-137.0130	-135.7940	-139.3040	-135.7270	-138.9380	-135.6590	-138.8820	-135.8570	-138.8960	**-114.23**
Nuclei	**B3P86**																						
**C1**	143.5882	143.8857	144.2601	143.9944	145.4860	144.0184	144.6761	144.0202	144.7972	144.0161	144.6223	142.5032	143.3389	143.9361	143.7927	145.0846	143.7838	145.0234	143.7552	144.9969	143.8483	145.1531	**134.46**
**C2**	122.0500	121.4140	121.9484	120.9786	121.5496	121.0264	121.9407	121.0521	121.9662	120.9775	121.8958	121.6059	121.2313	121.7336	120.9810	121.5467	121.0160	122.0953	121.0302	122.0647	120.9924	122.0967	**115.52**
**C3**	171.4766	171.7572	172.4534	171.8562	172.8205	171.8722	172.7552	171.8663	172.8432	171.8848	172.8132	171.5989	171.7923	172.5273	171.8492	172.7903	171.8669	172.6872	171.8624	172.7898	171.8762	172.7509	**162.83**
**C4**	124.7383	126.3386	127.1703	127.1465	127.7289	127.1190	128.8825	127.0698	128.7318	127.2163	129.0989	124.9692	126.2742	127.0621	126.8523	127.5412	126.8407	128.2740	126.8062	128.2031	126.9060	128.4801	**122.41**
**C5**	134.1190	136.0923	136.8360	137.1175	138.1511	137.0776	138.2932	137.0156	138.2758	137.1994	138.4696	134.2869	136.1384	136.9160	137.0534	138.0822	137.0204	138.0830	136.9648	138.1060	137.1283	138.2758	**131.43**
**C6**	127.7437	129.7963	131.1240	130.8021	132.4588	130.7549	132.9204	130.6858	132.8108	130.8906	133.4203	128.0255	130.2460	131.5499	131.2899	133.1034	131.2477	133.4157	131.1830	133.3543	131.3732	133.7598	**125.17**
**C7**	201.9099	206.8478	207.9904	209.5808	210.2942	209.4481	215.2960	209.2939	214.2210	209.7511	215.8620	206.3180	210.5062	211.9865	212.7171	213.5157	212.6075	218.1374	212.4759	217.1507	212.8653	218.6886	**196.61**
**C8**	64.1947	65.4630	65.8606	65.8682	66.6991	65.8672	67.0646	65.8435	66.9744	65.9125	67.8105	58.7228	60.4363	60.8267	61.3178	62.0716	61.2816	62.8455	61.2251	62.6601	61.3983	63.1673	**59.97**
**C9**	22.7475	23.0717	23.3242	23.2283	23.6105	23.2337	23.6256	23.2170	23.6953	23.2667	23.8703	19.4531	19.8849	19.9864	20.0966	20.4037	20.1003	20.5372	20.0831	20.5704	20.1379	20.9405	**15.26**
**C8`**	35.8781	36.6414	36.8568	37.0306	37.3127	37.0303	37.5686	37.0080	37.6041	37.0752	38.0187	31.2493	31.5208	31.9177	31.6781	32.0171	31.6880	32.0895	31.6758	32.2063	31.7119	32.1736	**31.09**
**H2**	8.2745	8.1793	8.2813	8.1109	8.1549	8.1156	8.1944	8.1185	8.2075	8.1077	8.2042	8.3112	8.1470	8.2209	8.0493	8.1095	8.0566	8.0849	8.0618	8.1072	8.0432	8.0875	**7.66**
**H4**	7.3557	7.5607	7.6556	7.6463	7.7696	7.6427	7.8178	7.6373	7.8047	7.6509	7.8419	7.3607	7.5493	7.6483	7.6231	7.7505	7.6202	7.7815	7.6155	7.7723	7.6270	7.8060	**7.33**
**H5**	7.5209	7.7338	7.7950	7.8329	7.9161	7.8283	7.9352	7.8219	7.9277	7.8384	7.9625	7.5202	7.7209	7.7789	7.8106	7.8950	7.8066	7.8993	7.8007	7.8943	7.8156	7.9253	**7.45**
**H6**	7.8758	8.0757	8.2047	8.1846	8.3140	8.1788	8.3726	8.1712	8.3643	8.1913	8.4241	7.6910	7.8848	8.0037	7.9751	8.1504	7.9710	8.1467	7.9649	8.1448	7.9801	8.1911	**7.57**
**H8**	4.4009	4.4924	4.5542	4.5758	4.6402	4.5712	4.6993	4.5650	4.6918	4.5814	4.7239	4.5720	4.7356	4.8033	4.8046	4.9268	4.8020	4.9412	4.7974	4.9366	4.8082	4.9727	**4.92**
**H(9A-9C)**	1.3389	1.3452	1.3394	1.3236	1.2953	1.3256	1.3051	1.3258	1.3092	1.3229	1.3403	1.4048	1.4431	1.4244	1.4604	1.4333	1.4604	1.4511	1.4585	1.4512	1.4617	1.4785	**1.44**
**H(8`A-8`C)**	2.4988	2.4477	2.3928	2.4185	2.3931	2.4210	2.3678	2.4216	2.3713	2.4175	2.3530	2.9054	2.7426	2.7200	2.6666	2.6266	2.6725	2.5336	2.6764	2.5595	2.6621	2.5055	**2.64**
**F3**	-123.8480	-126.8610	-129.2580	-127.9970	-131.2570	-127.9330	-130.7960	-127.8690	-130.7610	-128.0560	-130.7680	-123.9030	-126.9660	-129.4040	-128.0530	-131.3380	-127.9940	-130.9330	-127.9350	-130.8970	-128.1080	-130.8890	**-114.23**
Nuclei	**B3PW91**																						
**C1**	143.8800	144.4147	145.4722	144.3995	146.2931	144.4034	145.1105	144.3978	145.5447	144.3973	145.1306	142.7315	143.8074	144.2290	144.1743	145.4535	144.1473	145.5161	144.1157	145.4085	144.2081	145.6977	**134.46**
**C2**	122.1447	121.5756	122.0735	121.1515	122.0417	121.1830	122.1122	121.2053	122.0772	121.1240	122.0749	121.7362	121.4309	121.8580	121.1399	121.7072	121.1604	122.2245	121.1730	122.1307	121.1241	122.2513	**115.52**
**C3**	171.5748	171.8425	172.5139	171.9060	173.1870	171.9073	172.7796	171.8997	172.7815	171.9067	172.9125	171.6836	171.9096	172.5674	171.9623	172.8953	171.9643	172.7360	171.9574	172.7937	171.9621	172.8561	**162.83**
**C4**	124.7719	126.4292	127.0364	127.2002	128.0962	127.1580	128.8928	127.1074	128.5317	127.2421	129.0779	124.9875	126.2657	126.9872	126.8378	127.5108	126.8124	128.2019	126.7769	128.0256	126.8618	128.3806	**122.41**
**C5**	134.2338	136.2477	136.9376	137.2244	138.5703	137.1700	138.3662	137.1070	138.2394	137.2776	138.5637	134.3858	136.2471	136.9390	137.1454	138.1528	137.0978	138.1519	137.0409	138.0629	137.1916	138.3314	**131.43**
**C6**	127.6758	129.6648	130.6611	130.7720	132.6653	130.7089	132.8829	130.6376	132.5347	130.8346	133.2961	128.0141	130.1811	131.4346	131.2282	133.0315	131.1721	133.3189	131.1059	133.1252	131.2819	133.6088	**125.17**
**C7**	202.0718	207.4183	209.0569	209.9610	211.1159	209.8061	215.7742	209.6417	214.8888	210.1096	216.4884	206.4108	210.8306	212.1503	213.0297	213.7709	212.9022	218.6056	212.7666	217.4713	213.1539	219.1794	**196.61**
**C8**	63.9750	65.2393	65.7508	65.6534	66.7534	65.6399	66.8360	65.6163	66.8245	65.6670	67.5695	58.6675	60.5171	60.8101	61.3625	62.1253	61.3082	62.9717	61.2481	62.6815	61.4201	63.3178	**59.97**
**C9**	22.4502	22.7145	22.8333	22.9525	23.5657	22.9430	23.3193	22.9254	23.2343	22.9634	23.4981	19.1804	19.5801	19.6279	19.7626	20.0693	19.7521	20.1999	19.7340	20.1209	19.7756	20.5623	**15.26**
**C8`**	35.8422	36.7074	36.8658	37.0444	37.6935	37.0291	37.5500	37.0040	37.4984	37.0605	38.0013	31.2940	31.5836	31.9173	31.7595	32.0797	31.7558	32.1105	31.7423	32.1322	31.7634	32.1905	**31.09**
**H2**	8.2403	8.1215	8.1517	8.0665	8.1219	8.0706	8.1519	8.0744	8.1204	8.0629	8.1604	8.2833	8.0990	8.1864	8.0124	8.0735	8.0191	8.0316	8.0250	8.0587	8.0056	8.0376	**7.66**
**H4**	7.3561	7.5583	7.6532	7.6420	7.7789	7.6376	7.8139	7.6329	7.7939	7.6463	7.8374	7.3610	7.5461	7.6437	7.6203	7.7448	7.6167	7.7721	7.6128	7.7638	7.6238	7.7990	**7.33**
**H5**	7.5260	7.7362	7.7925	7.8341	7.9320	7.8288	7.9354	7.8231	7.9202	7.8392	7.9619	7.5242	7.7192	7.7774	7.8094	7.8917	7.8047	7.8997	7.7996	7.8879	7.8140	7.9205	**7.45**
**H6**	7.8568	8.0468	8.1242	8.1588	8.3049	8.1520	8.3452	8.1453	8.3011	8.1652	8.3959	7.6747	7.8589	7.9798	7.9551	8.1278	7.9504	8.1225	7.9450	8.1138	7.9594	8.1595	**7.57**
**H8**	4.3822	4.4758	4.5140	4.5605	4.6465	4.5544	4.6815	4.5483	4.6513	4.5667	4.7074	4.5456	4.7034	4.7694	4.7755	4.8955	4.7721	4.9137	4.7681	4.9035	4.7787	4.9401	**4.92**
**H(9A-9C)**	1.3011	1.2890	1.2558	1.2813	1.2589	1.2826	1.2670	1.2836	1.2547	1.2803	1.2891	1.3711	1.4019	1.3884	1.4242	1.3969	1.4232	1.4187	1.4220	1.4115	1.4251	1.4380	**1.44**
**H(8`A-8`C)**	2.4674	2.4163	2.3862	2.3971	2.3791	2.3987	2.3498	2.4000	2.3588	2.3959	2.3304	2.8744	2.7115	2.6894	2.6415	2.5988	2.6465	2.5082	2.6510	2.5310	2.6365	2.4757	**2.64**
**F3**	-123.2020	-126.4690	-128.8900	-127.6170	-129.7320	-127.5520	-130.4150	-127.4870	-130.3890	-127.6770	-130.1910	-123.2340	-126.3520	-128.8000	-127.4490	-130.7460	-127.3900	-130.5610	-127.3300	-130.3050	-127.5050	-130.2930	**-114.23**
Nuclei	**B98**																						
**C1**	144.6912	145.1473	145.2484	145.1160	146.6617	145.1308	145.7032	145.1429	145.8633	145.1039	145.7376	143.5207	144.4784	144.9860	144.8610	146.2082	144.8492	146.1736	144.8363	146.0946	144.8771	146.3230	**134.46**
**C2**	121.8392	121.1925	121.6495	120.7723	121.3222	120.8136	121.7340	120.8523	121.6761	120.7355	121.6994	121.3720	121.0411	121.4894	120.7551	121.3064	120.7868	121.8401	120.8168	121.7380	120.7278	121.8371	**115.52**
**C3**	171.3798	171.6392	172.2889	171.7095	172.6763	171.7205	172.5821	171.7290	172.5866	171.7014	172.6373	171.5646	171.6951	172.3690	171.7571	172.6945	171.7685	172.5796	171.7776	172.5921	171.7486	172.6330	**162.83**
**C4**	124.5512	126.0897	126.8377	126.8573	127.4027	126.8258	128.5411	126.7921	128.2839	126.8894	128.7456	124.7334	125.9597	126.6672	126.5323	127.1662	126.5153	127.8641	126.4952	127.7038	126.5497	128.0437	**122.41**
**C5**	133.9957	135.9330	136.5648	136.9065	137.8988	136.8625	138.0162	136.8159	137.9178	136.9503	138.1891	134.1576	135.9511	136.6379	136.8545	137.8258	136.8161	137.8101	136.7747	137.7441	136.8929	137.9858	**131.43**
**C6**	127.7600	129.6454	131.0479	130.7507	132.3478	130.6979	132.8888	130.6429	132.6290	130.8035	133.2983	128.0343	130.1734	131.4142	131.2223	132.9822	131.1747	133.2801	131.1239	133.1102	131.2694	133.6069	**125.17**
**C7**	200.3847	205.8210	206.6302	208.4239	209.2010	208.2766	214.2818	208.1271	213.1864	208.5658	215.0160	204.8815	209.2606	210.7400	211.5452	212.3559	211.4233	217.1974	211.2992	216.1094	211.6637	217.8036	**196.61**
**C8**	65.6444	66.8320	67.2503	67.2632	68.0685	67.2581	68.4263	67.2491	68.2684	67.2685	69.1043	60.3450	62.1437	62.4694	62.9801	63.7622	62.9409	64.5626	62.9005	64.2805	63.0214	64.8946	**59.97**
**C9**	23.1980	23.4234	23.7294	23.6797	24.0099	23.6790	24.0670	23.6758	24.0133	23.6828	24.2291	20.0207	20.3805	20.4020	20.5221	20.8130	20.5255	20.9448	20.5271	20.8729	20.5220	21.2988	**15.26**
**C8`**	36.6858	37.4599	37.6057	37.8314	38.1507	37.8240	38.3667	37.8133	38.3252	37.8400	38.8016	32.0806	32.3129	32.6647	32.5156	32.8281	32.5190	32.8955	32.5193	32.9209	32.5139	32.9413	**31.09**
**H2**	8.2422	8.1254	8.2502	8.0710	8.1142	8.0747	8.1700	8.0786	8.1688	8.0678	8.1716	8.2869	8.0982	8.1880	8.0133	8.0716	8.0189	8.0477	8.0244	8.0637	8.0080	8.0451	**7.66**
**H4**	7.3491	7.5528	7.6486	7.6371	7.7598	7.6324	7.8106	7.6277	7.7936	7.6418	7.8334	7.3601	7.5372	7.6372	7.6122	7.7387	7.6082	7.7714	7.6041	7.7584	7.6163	7.7929	**7.33**
**H5**	7.5101	7.7209	7.7802	7.8189	7.9020	7.8132	7.9211	7.8077	7.9093	7.8245	7.9448	7.5068	7.7030	7.7610	7.7958	7.8786	7.7905	7.8855	7.7852	7.8760	7.8010	7.9077	**7.45**
**H6**	7.8240	8.0213	8.1543	8.1379	8.2740	8.1307	8.3365	8.1236	8.3167	8.1451	8.3778	7.6446	7.8376	7.9585	7.9304	8.1040	7.9251	8.1030	7.9197	8.0932	7.9357	8.1447	**7.57**
**H8**	4.3568	4.4526	4.5088	4.5358	4.6085	4.5295	4.6622	4.5236	4.6511	4.5422	4.6846	4.5104	4.6782	4.7425	4.7482	4.8718	4.7442	4.8870	4.7403	4.8770	4.7521	4.9144	**4.92**
**H(9A-9C)**	1.3224	1.3104	1.3261	1.3036	1.2685	1.3046	1.2927	1.3056	1.2869	1.3030	1.3135	1.3935	1.4261	1.4092	1.4455	1.4167	1.4443	1.4350	1.4431	1.4301	1.4469	1.4568	**1.44**
**H(8`A-8`C)**	2.4937	2.4463	2.3982	2.4270	2.3963	2.4282	2.3810	2.4293	2.3827	2.4263	2.3622	2.9118	2.7469	2.7222	2.6733	2.6323	2.6777	2.5444	2.6821	2.5664	2.6694	2.5101	**2.64**
**F3**	-126.2150	-129.2130	-131.7250	-130.3470	-133.6920	-130.2830	-133.2670	-130.2190	-133.2300	-130.4070	-133.2220	-126.1000	-129.3280	-131.8480	-130.4130	-133.7870	-130.3550	-133.4210	-130.2950	-133.3780	-130.4680	-133.3550	**-114.23**

[a] methyl protons shifts are averaged

[b] according to [3]

Table S3. Cont. I

Nuclei	R											S											Exp. [b]
	B971																						
	Gas	$CHCl_3$ (PCM)	$CHCl_3$ (SMD)	DMSO (PCM)	DMSO (SMD)	CH_3OH (PCM)	CH_3OH (SMD)	C_2H_5OH (PCM)	C_2H_5OH (SMD)	H_2O (PCM)	H_2O (SMD)	Gas	$CHCl_3$ (PCM)	$CHCl_3$ (SMD)	DMSO (PCM)	DMSO (SMD)	CH_3OH (PCM)	CH_3OH (SMD)	C_2H_5OH (PCM)	C_2H_5OH (SMD)	H_2O (PCM)	H_2O (SMD)	
**C1**	144.2858	144.6963	144.8810	144.7090	146.1990	144.7156	145.3255	144.7228	145.4535	144.7095	145.3479	143.1278	144.0596	144.5992	144.4695	145.8067	144.4484	145.7610	144.4287	145.6754	144.4977	145.9166	**134.46**
**C2**	121.5536	120.8971	121.3534	120.4829	121.0157	120.5166	121.4207	120.5498	121.3599	120.4572	121.3902	121.0799	120.7403	121.1826	120.4666	120.9980	120.4902	121.5306	120.5135	121.4243	120.4508	121.5318	**115.52**
**C3**	171.3009	171.5668	172.2221	171.6510	172.6078	171.6538	172.5198	171.6562	172.5233	171.6544	172.5803	171.4842	171.6233	172.2984	171.6936	172.6252	171.6972	172.5067	171.7001	172.5183	171.6963	172.5664	**162.83**
**C4**	124.3159	125.8533	126.5929	126.6316	127.1722	126.5925	128.2854	126.5526	128.0375	126.6747	128.4970	124.4899	125.7349	126.4358	126.3143	126.9410	126.2895	127.6345	126.2635	127.4750	126.3429	127.8216	**122.41**
**C5**	133.7740	135.7159	136.3600	136.7103	137.6949	136.6577	137.8185	136.6047	137.7197	136.7662	137.9956	133.9388	135.7465	136.4351	136.6619	137.6329	136.6155	137.6106	136.5684	137.5459	136.7113	137.7923	**131.43**
**C6**	127.5496	129.4609	130.8010	130.5367	132.1567	130.4780	132.6331	130.4187	132.3883	130.5982	133.0588	127.7907	129.9596	131.1729	131.0198	132.7763	130.9641	133.0736	130.9075	132.9085	131.0779	133.4019	**125.17**
**C7**	200.2828	205.5725	206.5032	208.2255	208.9531	208.0715	214.0194	207.9184	212.9210	208.3790	214.7353	204.7631	209.0909	210.6142	211.3822	212.2103	211.2531	216.9467	211.1230	215.8795	211.5108	217.5640	**196.61**
**C8**	65.6155	66.8022	67.2103	67.2219	68.0383	67.2073	68.3845	67.1914	68.2192	67.2404	69.0669	60.3135	62.0473	62.3658	62.8947	63.6702	62.8471	64.4461	62.8002	64.1686	62.9474	64.7738	**59.97**
**C9**	23.2095	23.4487	23.7131	23.6905	24.0412	23.6825	24.0738	23.6737	24.0266	23.7042	24.2534	20.0362	20.4070	20.4206	20.5640	20.8483	20.5581	20.9884	20.5527	20.9160	20.5765	21.3570	**15.26**
**C8`**	36.6917	37.4457	37.5883	37.8149	38.0991	37.7979	38.3371	37.7806	38.2887	37.8365	38.7686	32.0232	32.2662	32.6085	32.4612	32.7611	32.4576	32.8140	32.4530	32.8408	32.4699	32.8668	**31.09**
**H2**	8.2393	8.1270	8.2441	8.0716	8.1154	8.0752	8.1650	8.0788	8.1660	8.0685	8.1683	8.2815	8.1000	8.1852	8.0140	8.0699	8.0195	8.0481	8.0249	8.0638	8.0089	8.0458	**7.66**
**H4**	7.3460	7.5474	7.6433	7.6329	7.7543	7.6280	7.8043	7.6231	7.7877	7.6379	7.8269	7.3560	7.5329	7.6330	7.6084	7.7339	7.6041	7.7661	7.5999	7.7532	7.6127	7.7878	**7.33**
**H5**	7.5117	7.7212	7.7814	7.8202	7.9023	7.8143	7.9213	7.8086	7.9099	7.8261	7.9449	7.5092	7.7054	7.7628	7.7981	7.8805	7.7927	7.8864	7.7873	7.8773	7.8036	7.9085	**7.45**
**H6**	7.8296	8.0263	8.1533	8.1409	8.2748	8.1335	8.3348	8.1263	8.3168	8.1482	8.3769	7.6455	7.8396	7.9610	7.9337	8.1063	7.9280	8.1077	7.9223	8.0977	7.9394	8.1491	**7.57**
**H8**	4.3551	4.4487	4.5048	4.5324	4.6023	4.5263	4.6565	4.5204	4.6464	4.5389	4.6784	4.5121	4.6785	4.7453	4.7492	4.8719	4.7450	4.8880	4.7408	4.8778	4.7536	4.9154	**4.92**
**H(9A-9C)**	1.3229	1.3162	1.3247	1.3073	1.2754	1.3081	1.2934	1.3089	1.2888	1.3068	1.3160	1.3965	1.4286	1.4098	1.4487	1.4194	1.4472	1.4375	1.4459	1.4329	1.4504	1.4594	**1.44**
**H(8`A-8`C)**	2.4912	2.4428	2.3934	2.4220	2.3921	2.4230	2.3743	2.4241	2.3755	2.4215	2.3550	2.9037	2.7397	2.7169	2.6672	2.6257	2.6714	2.5384	2.6755	2.5602	2.6637	2.5047	**2.64**
**F3**	-126.8460	-129.8690	-132.3840	-131.0090	-134.3690	-130.9450	-133.9360	-130.8800	-133.8980	-131.0690	-133.8950	-126.7480	-129.9850	-132.5090	-131.0820	-134.4640	-131.0220	-134.0970	-130.9630	-134.0520	-131.1370	-134.0340	**-114.23**
Nuclei	B972																						
**C1**	141.2342	141.6506	142.7266	141.7299	143.2843	141.7305	142.4664	141.7293	142.9618	141.7258	143.1773	140.0113	141.1468	141.5287	141.5569	142.8076	141.5189	142.8883	141.4848	142.9386	141.5980	143.0817	**134.46**
**C2**	119.7317	119.1483	119.6658	118.8149	119.3431	118.8372	119.7074	118.8570	119.6937	118.7896	119.7506	119.3405	119.0263	119.4669	118.8013	119.3217	118.8137	119.7986	118.8249	119.7255	118.7869	119.8144	**115.52**
**C3**	168.2732	168.4793	169.1572	168.6101	169.4995	168.6044	169.4066	168.5960	169.4009	168.6113	169.4863	168.3917	168.5517	169.2157	168.6764	169.5422	168.6705	169.3844	168.6622	169.3884	168.6775	169.4549	**162.83**
**C4**	122.1892	123.7396	124.3607	124.5654	125.0699	124.5163	126.1100	124.4648	125.7655	124.6072	126.1425	122.3897	123.5421	124.2685	124.1675	124.7609	124.1365	125.4016	124.1012	125.2031	124.1894	125.5840	**122.41**
**C5**	131.5363	133.4137	134.0989	134.4183	135.3588	134.3592	135.4500	134.2977	135.3373	134.4699	135.6259	131.1632	133.3991	134.0780	134.3355	135.2487	134.2822	135.2475	134.2258	135.1627	134.3810	135.4358	**131.43**
**C6**	125.0627	126.9763	127.9602	128.1319	129.6026	128.0595	130.1473	127.9845	129.7780	128.1963	130.3129	125.4310	127.4217	128.6994	128.5016	130.1890	128.4427	130.4260	128.3792	130.2192	128.5499	130.7362	**125.17**
**C7**	198.1130	203.2735	204.9522	205.9151	206.7453	205.7587	211.5986	205.5099	210.9110	206.0594	212.9086	202.2824	206.6676	207.8990	208.9064	209.5861	208.7689	214.4033	208.6311	213.3890	209.0354	214.9807	**196.61**
**C8**	62.7055	63.8602	64.3230	64.2776	64.9739	64.2619	65.4325	64.2431	65.4239	64.2879	66.3446	57.6239	59.4402	59.7721	60.3181	61.0364	60.2576	61.8700	60.1978	61.6540	60.3764	62.2175	**59.97**
**C9**	22.1305	22.3749	22.5096	22.6434	22.8754	22.6258	22.9796	22.6063	22.8864	22.6564	23.0535	18.9733	19.2591	19.3543	19.4810	19.7298	19.4663	19.8374	19.4498	19.7701	19.4914	20.1871	**15.26**
**C8`**	35.6102	36.4027	36.5773	36.8090	37.1085	36.7901	37.2019	36.7683	37.1923	36.8224	37.6237	31.1992	31.4270	31.7695	31.6774	31.9475	31.6669	31.9423	31.6525	31.9351	31.6807	32.0059	**31.09**
**H2**	8.2202	8.1039	8.1362	8.0508	8.0874	8.0538	8.1218	8.0565	8.0850	8.0477	8.0643	8.2713	8.0721	8.1659	7.9914	8.0486	7.9978	8.0032	8.0034	8.0079	7.9843	7.9995	**7.66**
**H4**	7.3567	7.5561	7.6513	7.6433	7.7610	7.6384	7.8102	7.6332	7.7911	7.6478	7.8290	7.3656	7.5462	7.6429	7.6230	7.7433	7.6189	7.7684	7.6145	7.7539	7.6266	7.7903	**7.33**
**H5**	7.5304	7.7369	7.7933	7.8364	7.9152	7.8306	7.9316	7.8247	7.9164	7.8417	7.9510	7.5255	7.7139	7.7729	7.8059	7.8843	7.8008	7.8925	7.7953	7.8819	7.8105	7.9154	**7.45**
**H6**	7.8294	8.0190	8.1022	8.1371	8.2676	8.1296	8.3137	8.1218	8.2736	8.1440	8.3117	7.6512	7.8295	7.9511	7.9302	8.0965	7.9251	8.0904	7.9194	8.0760	7.9343	8.1266	**7.57**
**H8**	4.3502	4.4406	4.4870	4.5320	4.6055	4.5254	4.6441	4.5186	4.6161	4.5382	4.6478	4.5078	4.6585	4.7265	4.7353	4.8523	4.7313	4.8712	4.7269	4.8595	4.7386	4.8963	**4.92**
**H(9A-9C)**	1.2782	1.2679	1.2367	1.2621	1.2209	1.2623	1.2488	1.2623	1.2313	1.2618	1.2497	1.3582	1.3850	1.3772	1.4110	1.3811	1.4097	1.4012	1.4081	1.3940	1.4120	1.4219	**1.44**
**H(8`A-8`C)**	2.4578	2.4146	2.3838	2.3997	2.3632	2.4005	2.3560	2.4012	2.3627	2.3989	2.3495	2.8672	2.7056	2.6834	2.6429	2.5976	2.6471	2.5119	2.6510	2.5283	2.6383	2.4759	**2.64**
**F3**	-120.6790	-123.8960	-126.2360	-125.0220	-128.1940	-124.9590	-127.7520	-124.8970	-127.7220	-125.0820	-127.7200	-120.7160	-123.7860	-126.1710	-124.8600	-128.0700	-124.8020	-127.9050	-124.7440	-127.8710	-124.9140	-127.8630	**-114.23**
Nuclei	BHandH																						
**C1**	145.6360	145.6202	146.2594	145.4915	146.8219	145.4920	145.8942	145.5103	146.0198	145.4598	146.0408	144.8250	145.0762	145.8833	145.6125	146.3566	145.5754	146.5035	145.5557	146.3936	145.6175	146.6148	**134.46**
**C2**	125.5346	124.8614	125.3852	124.4017	124.9632	124.4203	125.3070	124.4573	125.2714	124.3538	125.3096	125.1931	124.6852	125.2900	124.4661	125.2102	124.4704	125.5203	124.4930	125.4125	124.4318	125.5282	**115.52**
**C3**	171.8732	171.9700	172.6046	172.0080	172.8208	171.9951	172.7345	172.0008	172.7559	171.9901	172.8083	171.9185	171.9893	172.6152	172.0294	172.7735	172.0161	172.7471	172.0215	172.7571	172.0120	172.8168	**162.83**
**C4**	128.1593	129.8441	130.6558	130.7021	131.3619	130.6394	132.4962	130.5948	132.2208	130.7320	132.7262	128.3774	129.8761	130.6155	130.4811	131.2252	130.4340	132.0262	130.4051	131.8519	130.4957	132.2546	**122.41**
**C5**	137.7618	139.7698	140.4697	140.8119	141.7707	140.7383	141.8522	140.6830	141.7474	140.8514	142.0546	137.8857	139.7969	140.4930	140.7560	141.5001	140.6878	141.7509	140.6375	141.6660	140.7909	141.9471	**131.43**
**C6**	131.8650	134.1148	135.4404	135.2854	137.1236	135.2039	137.6614	135.1406	137.4270	135.3330	138.0745	132.0987	134.5952	135.9847	135.7379	136.4820	135.6609	138.0145	135.6017	137.8882	135.7808	138.3869	**125.17**
**C7**	203.6138	208.5172	209.8103	210.9896	211.6240	210.8308	216.6493	210.6894	215.4270	211.1096	217.5301	207.9046	212.0671	213.4264	214.2911	215.0352	214.1425	219.7537	214.0124	218.5758	214.4019	220.3276	**196.61**
**C8**	60.4282	61.6949	62.2727	62.1498	62.9860	62.0891	63.4098	62.0761	63.2734	62.1183	64.1380	54.5800	55.8057	56.5007	56.7153	57.4594	56.6558	58.0038	56.6138	57.8144	56.7411	58.3269	**59.97**
**C9**	22.3805	22.6640	22.8450	22.9402	23.3623	22.9132	23.3034	22.9043	23.2689	22.9357	23.4993	18.9705	19.4038	19.6250	19.6671	20.4112	19.6428	19.9886	19.6365	20.0067	19.6602	20.4147	**15.26**
**C8`**	33.7019	34.5319	34.7480	34.8536	35.2051	34.8254	35.4138	34.8155	35.3360	34.8501	35.9159	28.6013	29.0694	29.3172	29.2263	29.9704	29.2005	29.6834	29.1938	29.7130	29.2211	29.8358	**31.09**
**H2**	8.5722	8.4795	8.5451	8.4246	8.4730	8.4286	8.5049	8.4318	8.5079	8.4219	8.5011	8.5877	8.4565	8.4984	8.3373	8.2561	8.3446	8.3818	8.3512	8.4001	8.3316	8.3840	**7.66**
**H4**	7.4985	7.7095	7.8085	7.7982	7.9259	7.7940	7.9763	7.7889	7.9578	7.8032	8.0012	7.5018	7.7017	7.7993	7.7806	7.6994	7.7770	7.9489	7.7725	7.9342	7.7850	7.9735	**7.33**
**H5**	7.6246	7.8443	7.9007	7.9466	8.0283	7.9415	8.0466	7.9355	8.0338	7.9525	8.0731	7.6234	7.8356	7.8898	7.9314	7.8502	7.9267	8.0222	7.9211	8.0126	7.9368	8.0471	**7.45**
**H6**	8.0335	8.2570	8.3730	8.3788	8.5362	8.3720	8.6047	8.3644	8.5771	8.3864	8.6503	7.8509	8.0823	8.1934	8.1739	8.0927	8.1690	8.3690	8.1633	8.3621	8.1795	8.4148	**7.57**
**H8**	4.1658	4.2674	4.3192	4.3652	4.4301	4.3592	4.5088	4.3524	4.4784	4.3720	4.5395	4.4307	4.6046	4.6688	4.6828	4.6016	4.6792	4.8401	4.6747	4.8222	4.6872	4.8693	**4.92**
**H(9A-9C)**	1.4018	1.4107	1.3957	1.4074	1.3829	1.4085	1.3982	1.4087	1.3961	1.4074	1.4174	1.4787	1.5148	1.4955	1.5189	1.4377	1.5194	1.5006	1.5190	1.5024	1.5196	1.5250	**1.44**
**H(8`A-8`C)**	2.5560	2.4887	2.4337	2.4585	2.4255	2.4613	2.3897	2.4632	2.3949	2.4568	2.3736	2.9080	2.7589	2.7052	2.6642	2.5829	2.6700	2.5306	2.6752	2.5535	2.6597	2.5073	**2.64**
**F3**	-111.9810	-114.6650	-116.6860	-115.6670	-118.3730	-115.6120	-118.0960	-115.5550	-118.0360	-115.7180	-118.1120	-112.0420	-114.7710	-116.8330	-115.7370	-116.0740	-115.6860	-118.1890	-115.6330	-118.1500	-115.7860	-118.1880	**-114.23**
Nuclei	BHandHLYP																						
**C1**	145.9669	146.1759	146.2853	146.1113	147.5505	146.1200	146.5389	146.1313	146.5831	146.1038	146.5447	144.8879	145.5103	146.1158	146.0017	147.2410	145.9780	146.9278	145.9578	146.8892	146.0259	147.0243	**134.46**
**C2**	124.7515	124.0653	124.5461	123.6469	124.1741	123.6733	124.5921	123.7021	124.5316	123.6218	124.5706	124.3796	123.9052	124.4021	123.6341	124.1686	123.6521	124.6622	123.6725	124.5779	123.6171	124.6729	**115.52**
**C3**	171.7043	171.8078	172.3461	171.8437	172.6613	171.8422	172.5332	171.8432	172.5540	171.8447	172.5906	171.7888	171.8290	172.4188	171.8672	172.6522	171.8651	172.5289	171.8651	172.5561	171.8690	172.5864	**162.83**
**C4**	126.8528	128.3654	129.1479	129.1567	129.6354	129.1088	130.8346	129.0636	130.6113	129.2014	131.0509	127.1371	128.3244	129.0171	128.8473	129.4342	128.8162	130.1960	128.7867	130.0374	128.8755	130.4016	**122.41**
**C5**	136.1415	137.9845	138.5163	138.9472	139.8001	138.8900	139.8405	138.8351	139.7634	139.0011	140.0097	136.2629	137.9948	138.5973	138.8877	139.7198	138.8352	139.6727	138.7846	139.6227	138.9370	139.8503	**131.43**
**C6**	130.4116	132.4695	133.9130	133.6262	135.2232	133.5527	135.7764	133.4813	135.5814	133.6955	136.2221	130.7205	132.9798	134.2257	134.0284	135.8034	133.9634	136.1403	133.9004	135.9856	134.0893	136.5014	**125.17**
**C7**	201.8321	207.4006	208.2736	210.2494	210.7410	210.0807	216.5002	209.9158	215.1153	210.4091	217.2571	206.5177	210.9495	212.4315	213.3909	213.9546	213.2498	219.1800	213.1105	217.9581	213.5236	219.7995	**196.61**
**C8**	61.1863	62.2965	62.6692	62.6877	63.4497	62.6691	63.7555	62.6519	63.5794	62.7040	64.4069	55.9833	57.6105	58.0052	58.5002	59.2270	58.4476	59.8950	58.3984	59.6616	58.5510	60.2030	**59.97**
**C9**	22.1319	22.2439	22.5189	22.4257	22.7743	22.4127	22.6380	22.4021	22.6483	22.4376	22.8001	18.9225	19.1131	19.1758	19.2384	19.5320	19.2278	19.5100	19.2205	19.4920	19.2488	19.8200	**15.26**
**C8`**	34.7229	35.4951	35.6672	35.8743	36.1736	35.8529	36.3975	35.8339	36.3632	35.8942	36.8278	30.4753	30.7423	31.063	30.9170	31.2202	30.9040	31.3030	30.8934	31.3347	30.9286	31.4094	**31.09**
**H2**	8.4732	8.3531	8.4758	8.2941	8.3382	8.2976	8.3879	8.3009	8.4012	8.2908	8.3907	8.5140	8.3314	8.3985	8.2222	8.2799	8.2283	8.2660	8.2342	8.2819	8.2162	8.2679	**7.66**
**H4**	7.5023	7.7057	7.7988	7.7926	7.9095	7.7879	7.9669	7.7830	7.9483	7.7970	7.9897	7.5103	7.6957	7.7919	7.7705	7.8916	7.7665	7.9298	7.7623	7.9150	7.7741	7.9526	**7.33**
**H5**	7.6036	7.8161	7.8699	7.9168	7.9910	7.9111	8.0117	7.9054	7.9997	7.9221	8.0365	7.6006	7.8024	7.8543	7.8958	7.9708	7.8906	7.9797	7.8852	7.9693	7.9007	8.0032	**7.45**
**H6**	7.8862	8.1057	8.2423	8.2348	8.3728	8.2268	8.4463	8.2188	8.4310	8.2423	8.4902	7.7397	7.9492	8.0666	8.0466	8.2224	8.0407	8.2327	8.0347	8.2216	8.0519	8.2752	**7.57**
**H8**	4.0886	4.2077	4.2753	4.3124	4.3846	4.3052	4.4614	4.2981	4.4439	4.3194	4.4850	4.2755	4.4537	4.5253	4.5332	4.6547	4.5288	4.6820	4.5243	4.6690	4.5372	4.7086	**4.92**
**H(9A-9C)**	1.2996	1.2921	1.3009	1.2820	1.2498	1.2828	1.2651	1.2833	1.2665	1.2812	1.2869	1.3654	1.3916	1.3722	1.4072	1.3768	1.4061	1.3973	1.4048	1.3934	1.4082	1.4181	**1.44**
**H(8`A-8`C)**	2.4510	2.4053	2.3496	2.3879	2.3566	2.3890	2.3405	2.3899	2.3357	2.3869	2.3262	2.8661	2.7046	2.6760	2.6318	2.5854	2.6359	2.5109	2.6399	2.5281	2.6278	2.4822	**2.64**
**F3**	-115.0720	-118.0370	-120.2260	-119.1810	-122.0510	-119.1160	-121.7530	-119.0510	-121.7000	-119.2420	-121.7810	-115.1680	-118.1450	-120.3290	-119.2170	-122.1200	-119.1590	-121.8290	-119.1000	-121.7810	-119.2720	-121.8290	**-114.23**

[a] methyl protons shifts are averaged

[b] according to [3]

Table S3. Cont. II

Nuclei	R											S											Exp. [b]
	BLYP																						
	Gas	$CHCl_3$ (PCM)	$CHCl_3$ (SMD)	DMSO (PCM)	DMSO (SMD)	CH_3OH (PCM)	CH_3OH (SMD)	C_2H_5OH (PCM)	C_2H_5OH (SMD)	H_2O (PCM)	H_2O (SMD)	Gas	$CHCl_3$ (PCM)	$CHCl_3$ (SMD)	DMSO (PCM)	DMSO (SMD)	CH_3OH (PCM)	CH_3OH (SMD)	C_2H_5OH (PCM)	C_2H_5OH (SMD)	H_2O (PCM)	H_2O (SMD)	
**C1**	145.5450	146.0429	147.1140	146.1068	147.7986	146.3885	146.6492	146.2085	147.2347	146.4667	146.8219	143.7828	144.7209	145.5672	145.4278	146.9795	145.6805	146.8743	145.4719	147.0441	145.8161	147.0664	**134.46**
**C2**	120.8047	120.1141	120.5513	119.6783	120.1736	119.9844	120.4482	119.8286	120.6128	120.0147	120.5020	120.1479	119.6918	120.1601	119.4918	120.0265	119.7882	120.4772	119.6229	120.5599	119.8378	120.5410	**115.52**
**C3**	175.9013	176.3257	177.0927	176.4452	177.6220	176.7175	177.3132	176.5274	177.4898	176.8136	177.4629	176.1113	176.4275	177.1619	176.5133	177.6627	176.7855	177.3261	176.5958	177.5168	176.8816	177.4729	**162.83**
**C4**	124.0175	125.5362	126.0703	126.2893	126.7806	126.5226	127.7623	126.2931	127.6290	126.6945	128.0567	124.4345	125.5186	126.0303	125.9745	126.5488	126.2213	127.0869	126.0057	127.0861	126.3668	127.3739	**122.41**
**C5**	133.4939	135.4708	136.1370	136.4608	137.5177	136.6797	137.4897	136.4362	137.5416	136.8800	137.7612	133.8536	135.6443	136.2784	136.5056	137.5472	136.7303	137.3589	136.4925	137.4573	136.9191	137.6361	**131.43**
**C6**	127.3335	129.2443	130.2486	130.3468	132.0288	130.5519	132.4283	130.2954	132.2771	130.7791	132.9242	127.7381	129.9027	131.0864	130.8943	132.6839	131.1099	132.7663	130.8629	132.7695	131.3161	133.1971	**125.17**
**C7**	204.3147	209.5974	211.3643	212.2138	213.3986	212.3424	217.6300	212.0072	217.0965	212.7173	218.5008	209.9855	213.7889	215.2206	215.8739	217.0497	216.0246	221.1931	215.7137	220.4874	216.3575	221.7907	**196.61**
**C8**	69.7467	70.9133	71.4699	71.3079	72.2357	71.5712	72.4406	71.3721	72.5974	71.6848	73.2670	63.4344	65.8959	66.5807	67.0939	68.0176	67.3157	68.6302	67.0757	68.5879	67.5108	69.0388	**59.97**
**C9**	23.7062	24.0257	24.1727	24.2581	24.6168	24.5176	24.6672	24.3164	24.7533	24.6398	24.9144	20.0844	20.7739	20.9191	21.0394	21.3485	21.3048	21.4163	21.1084	21.5141	21.4143	21.9134	**15.26**
**C8`**	38.1900	38.9597	39.1326	39.3487	39.7506	39.6097	39.7477	39.4076	39.8899	39.7268	40.2871	33.7250	33.8484	34.0039	33.8977	34.2370	34.1650	34.0793	33.9700	34.2737	34.2710	34.2020	**31.09**
**H2**	8.0144	7.9189	7.9482	7.8624	7.8961	7.9128	8.1257	7.8706	7.9334	7.9147	8.1393	8.1164	7.9489	7.9914	7.8300	7.8766	7.8827	8.0190	7.8427	7.8585	7.8803	8.0324	**7.66**
**H4**	7.1890	7.3809	7.4696	7.4593	7.5757	7.5022	7.7919	7.4526	7.6069	7.5187	7.8276	7.2020	7.3728	7.4591	7.4377	7.5578	7.4812	7.7564	7.4322	7.5764	7.4965	7.7911	**7.33**
**H5**	7.3549	7.5559	7.6077	7.6483	7.7289	7.6903	7.9148	7.6398	7.7335	7.7087	7.9524	7.3623	7.5446	7.5939	7.6255	7.7039	7.6680	7.8758	7.6179	7.6995	7.6853	7.9117	**7.45**
**H6**	7.6850	7.8736	7.9488	7.9791	8.1082	8.0197	8.3379	7.9677	8.1352	8.0407	8.3884	7.5195	7.6882	7.7795	7.7578	7.9182	7.8000	8.0791	7.7495	7.9004	7.8181	8.1330	**7.57**
**H8**	4.5761	4.6500	4.6786	4.7145	4.7859	4.7570	4.9873	4.7070	4.8028	4.7746	5.0235	4.6740	4.8401	4.8901	4.9038	5.0249	4.9471	5.2085	4.8978	5.0292	4.9630	5.2513	**4.92**
**H(9A-9C)**	1.3402	1.3303	1.3028	1.3212	1.2904	1.3686	1.4914	1.3236	1.3125	1.3764	1.5231	1.4094	1.4647	1.4498	1.4862	1.4612	1.5321	1.6498	1.4855	1.4752	1.5429	1.6882	**1.44**
**H(8`A-8`C)**	2.5822	2.5238	2.4953	2.5006	2.4750	2.5491	2.6267	2.5052	2.4680	2.5548	2.6184	3.0640	2.8899	2.8367	2.7907	2.7527	2.8426	2.8332	2.8020	2.6829	2.8417	2.8107	**2.64**
**F3**	-149.5110	-153.2650	-156.3110	-154.7170	-158.8230	-154.4270	-157.2910	-154.5530	-158.2730	-154.7280	-157.7550	-149.3510	-153.2220	-156.3260	-154.6670	-158.7980	-154.3830	-157.3990	-154.5130	-158.3430	-154.6730	-157.8360	**-114.23**
Nuclei	BMK																						
**C1**	162.2400	162.1101	162.7462	162.1787	163.7432	162.1732	162.7329	162.3788	162.5499	162.3453	162.8250	161.3138	161.8189	162.6178	162.4354	163.8322	162.3810	163.7489	162.5443	163.5016	162.6573	163.8918	**134.46**
**C2**	136.7126	135.7407	136.3571	135.1216	135.9083	135.1421	136.2364	135.3741	136.0691	135.2636	136.1930	136.2628	135.4326	136.0286	135.0703	135.7132	135.0819	136.1680	135.3055	135.9639	135.2213	136.1478	**115.52**
**C3**	187.0301	187.0551	187.8408	187.0477	188.1962	187.0333	188.0420	187.2302	187.9469	187.2230	188.0648	187.2376	187.1687	187.9784	187.1390	188.2778	187.1240	188.1259	187.3205	188.0362	187.3149	188.1513	**162.83**
**C4**	139.5338	141.0867	141.9440	141.8030	142.6696	141.7419	143.7062	141.8918	143.3926	142.0227	143.8873	139.7892	141.0292	141.9252	141.5094	142.4656	141.4635	143.1447	141.6273	142.8800	141.7124	143.3050	**122.41**
**C5**	150.5620	152.5708	153.4546	153.6247	154.9031	153.5456	154.9676	153.6775	154.7813	153.8616	155.1400	150.7561	152.7680	153.5758	153.6700	154.9685	153.6018	154.8508	153.7437	154.7018	153.8949	155.0130	**131.43**
**C6**	143.3877	145.7280	147.0994	146.7575	148.8367	146.6701	149.2565	146.7941	148.9691	147.0018	149.6360	143.4992	146.0572	147.3665	147.2515	149.4923	147.1717	149.7664	147.3009	149.5231	147.4870	150.0856	**125.17**
**C7**	217.8060	223.4655	224.9383	226.4686	227.1939	226.2755	233.0517	226.2929	231.5604	226.8133	233.9788	222.3530	227.3456	229.0592	230.0127	231.0492	229.8346	236.7529	229.8700	235.3030	230.3458	237.5066	**196.61**
**C8**	71.2103	72.3800	73.0712	72.9676	74.0630	72.9310	74.4301	73.1053	74.0994	73.1633	75.0734	65.2856	66.6120	67.1563	67.8075	68.5986	67.7283	69.5166	67.8632	69.0652	68.0480	69.8209	**59.97**
**C9**	25.5376	25.8446	26.0318	25.9188	26.4894	25.8901	26.4426	26.0718	26.2908	26.1081	26.6342	22.3266	22.2570	22.4909	22.7080	22.9725	22.6732	23.0897	22.8502	22.9345	22.9034	23.4149	**15.26**
**C8`**	38.7669	39.4262	39.7117	39.8197	40.3008	39.7903	40.3722	39.9721	40.2640	40.0092	40.7922	33.7006	34.0446	34.4504	34.1208	34.6961	34.0997	34.6697	34.2891	34.6786	34.3014	34.7612	**31.09**
**H2**	8.5985	8.5179	8.5943	8.4278	8.4833	8.4281	8.5266	8.4285	8.5512	8.4179	8.5251	8.5848	8.4049	8.4853	8.3273	8.3863	8.3305	8.3735	8.3332	8.4009	8.3138	8.3733	**7.66**
**H4**	7.6493	7.8608	7.9448	7.9468	8.0661	7.9386	8.1039	7.9304	8.0859	7.9449	8.1322	7.6620	7.8408	7.9425	7.9230	8.0503	7.9150	8.0813	7.9070	8.0691	7.9209	8.1058	**7.33**
**H5**	7.8765	8.1019	8.1629	8.2055	8.2858	8.1960	8.3072	8.1865	8.2981	8.2048	8.3310	7.8678	8.0844	8.1336	8.1798	8.2616	8.1705	8.2654	8.1612	8.2551	8.1790	8.2876	**7.45**
**H6**	8.2070	8.4386	8.5498	8.5495	8.6971	8.5388	8.7420	8.5280	8.7421	8.5502	8.7907	8.0160	8.2064	8.3309	8.3395	8.5254	8.3290	8.5327	8.3183	8.5189	8.3395	8.5783	**7.57**
**H8**	4.2821	4.4049	4.4538	4.4832	4.5594	4.4734	4.6132	4.4638	4.6089	4.4829	4.6388	4.4973	4.6358	4.7186	4.7368	4.8600	4.7268	4.8923	4.7167	4.8668	4.7368	4.9222	**4.92**
**H(9A-9C)**	1.3069	1.3289	1.3147	1.3201	1.2859	1.3172	1.3085	1.3142	1.3133	1.3134	1.3258	1.3376	1.3959	1.3567	1.4192	1.3884	1.4140	1.4066	1.4088	1.3969	1.4144	1.4317	**1.44**
**H(8`A-8`C)**	2.4403	2.3909	2.3332	2.3812	2.3387	2.3786	2.3210	2.3761	2.3112	2.3741	2.3056	2.8178	2.6558	2.6295	2.5940	2.5372	2.5946	2.4500	2.5949	2.4707	2.5833	2.4236	**2.64**
**F3**	-124.0340	-127.1270	-129.6180	-128.2800	-131.6250	-128.2180	-131.2150	-128.1530	-131.1640	-128.3400	-131.1870	-123.9260	-127.2520	-129.7640	-128.3300	-131.6780	-128.2740	-131.2840	-128.2160	-131.2510	-128.3830	-131.2370	**-114.23**
Nuclei	BP86																						
**C1**	142.6033	143.2892	144.3217	143.3129	144.8294	143.3161	144.1093	143.3149	144.3473	143.3051	144.2880	141.2102	142.3913	142.8955	142.7789	144.0934	142.7583	144.2034	142.7343	144.0933	142.7962	144.5179	**134.46**
**C2**	119.5132	118.9948	119.4330	118.5273	118.9749	118.5643	119.3960	118.5964	119.3473	118.4873	119.5119	118.9530	118.7496	119.1040	118.4438	118.9042	118.4690	119.5071	118.4896	119.3803	118.4158	119.6576	**115.52**
**C3**	172.4608	172.9866	173.7055	173.0692	174.0928	173.0730	174.0023	173.0716	173.9922	173.0606	174.2325	172.6041	173.0252	173.7451	173.0803	174.0764	173.0859	173.9418	173.0864	173.9453	173.0699	174.1650	**162.83**
**C4**	122.8492	124.5769	125.1420	125.3271	125.8172	125.2897	126.9376	125.2471	126.6421	125.3577	127.3199	123.0920	124.4730	125.1351	125.0277	125.6030	125.0052	126.3091	124.9769	126.1381	125.0431	126.6652	**122.41**
**C5**	132.3039	134.4559	135.1483	135.4396	136.4619	135.3882	136.6274	135.3316	136.4931	135.4832	136.9837	132.5764	134.5444	135.2681	135.4289	136.4465	135.3852	136.4384	135.3359	136.3593	135.4648	136.7926	**131.43**
**C6**	125.6950	127.6383	128.6236	128.6503	130.2114	128.5969	130.6655	128.5381	130.3845	128.6961	131.2615	125.9992	128.2203	129.3919	129.2180	130.9403	129.1674	131.2237	129.1109	131.0571	129.2602	131.7128	**125.17**
**C7**	201.4869	206.6192	208.2390	209.0105	209.9463	208.8668	214.4244	208.7193	213.5707	209.1433	215.2402	206.3315	210.3628	211.8523	212.3568	213.2823	212.2454	217.4302	212.1289	216.5301	212.4577	218.1117	**196.61**
**C8**	67.7583	69.1024	69.6319	69.4925	70.2735	69.4833	70.6802	69.4672	70.5751	69.4949	71.6087	62.0427	64.0265	64.2886	64.8784	65.6473	64.8306	66.4624	64.7787	66.1861	64.9206	66.9619	**59.97**
**C9**	23.4587	23.8784	23.9802	24.1183	24.3813	24.1120	24.5183	24.1003	24.4332	24.1189	24.8862	20.1385	20.7994	20.7668	21.0088	21.2511	21.0015	21.5039	20.9895	21.4011	21.0115	22.1254	**15.26**
**C8`**	37.4532	38.3350	38.4359	38.6311	38.8361	38.6206	39.0764	38.6051	39.0143	38.6360	39.7128	32.5953	32.8935	33.1929	33.0035	33.2356	33.0063	33.2521	33.0032	33.2824	32.9950	33.4517	**31.09**
**H2**	8.0460	7.9530	7.9729	7.8987	7.9248	7.9033	7.9680	7.9075	7.9548	7.8941	7.9863	8.1062	7.9414	8.0081	7.8573	7.8978	7.8638	7.8711	7.8698	7.8847	7.8505	7.8826	**7.66**
**H4**	7.1940	7.3974	7.4813	7.4806	7.5914	7.4767	7.6388	7.4724	7.6207	7.4840	7.6760	7.1971	7.3813	7.4684	7.4550	7.5689	7.4518	7.5995	7.4482	7.5860	7.4577	7.6351	**7.33**
**H5**	7.3836	7.5932	7.6394	7.6900	7.7639	7.6851	7.7826	7.6799	7.7686	7.6943	7.8221	7.3932	7.5853	7.6333	7.6731	7.7466	7.6689	7.7505	7.6643	7.7411	7.6767	7.7875	**7.45**
**H6**	7.7551	7.9399	7.9988	8.0409	8.1524	8.0354	8.2056	8.0298	8.1770	8.0457	8.2633	7.5562	7.7292	7.8336	7.8195	7.9743	7.8153	7.9668	7.8106	7.9591	7.8230	8.0215	**7.57**
**H8**	4.6102	4.6886	4.7066	4.7545	4.8085	4.7500	4.8491	4.7453	4.8359	4.7587	4.8868	4.7499	4.9016	4.9510	4.9712	5.0773	4.9680	5.0911	4.9645	5.0812	4.9737	5.1350	**4.92**
**H(9A-9C)**	1.3746	1.3642	1.3222	1.3541	1.3091	1.3560	1.3291	1.3576	1.3202	1.3518	1.3676	1.4508	1.4923	1.4616	1.5156	1.4781	1.5149	1.4991	1.5140	1.4923	1.5158	1.5398	**1.44**
**H(8`A-8`C)**	2.5693	2.5039	2.4639	2.4798	2.4397	2.4819	2.4190	2.4838	2.4247	2.4774	2.4119	2.9949	2.8241	2.7917	2.7480	2.6939	2.7534	2.5979	2.7585	2.6174	2.7424	2.5769	**2.64**
**F3**	-141.0370	-144.3320	-147.1730	-145.5650	-149.3730	-145.4950	-148.8330	-145.4250	-148.8150	-145.6310	-148.7520	-140.9280	-144.3630	-147.1850	-145.5720	-149.4050	-145.5060	-148.9800	-145.4390	-148.9300	-145.6350	-148.8910	**-114.23**
Nuclei	CAM-B3LYP																						
**C1**	147.5356	147.8355	148.0266	147.8495	149.3617	147.8601	148.4435	147.8717	148.5998	147.8423	148.4144	146.3788	147.2835	147.7550	147.7021	148.9802	147.6764	148.8682	147.6547	148.7837	147.7335	149.0350	**134.46**
**C2**	125.3419	124.7113	125.1447	124.2638	124.8115	124.2955	125.2207	124.3274	125.1608	124.2361	125.1909	124.9277	124.5601	124.9831	124.2725	124.8107	124.2933	125.3385	124.3149	125.2347	124.2562	125.3523	**115.52**
**C3**	175.2350	175.4104	175.9761	175.4531	176.3474	175.4552	176.2406	175.4577	176.2474	175.4534	176.2969	175.3257	175.4458	176.0405	175.4756	176.3409	175.4775	176.2215	175.4799	176.2296	175.4758	176.2811	**162.83**
**C4**	127.7281	129.2396	129.9748	130.0025	130.5411	129.9608	131.6302	129.9196	131.3634	130.0440	131.8418	127.9995	129.1976	129.8916	129.7256	130.3720	129.7006	131.0401	129.6749	130.8844	129.7497	131.2249	**122.41**
**C5**	137.3892	139.3230	139.8977	140.3179	141.2561	140.2631	141.3314	140.2087	141.2302	140.3722	141.5022	137.5314	139.3463	139.9686	140.2385	141.1638	140.1900	141.1167	140.1415	141.0496	140.2861	141.2976	**131.43**
**C6**	131.2257	133.3580	134.7016	134.4471	136.1424	134.3790	136.5910	134.3108	136.3369	134.5135	137.0724	131.5278	133.8293	135.0818	134.9175	136.7818	134.8560	137.0470	134.7942	136.8772	134.9768	137.3859	**125.17**
**C7**	205.8158	211.3203	212.3152	214.2540	214.9544	214.0898	220.3783	213.9268	219.1675	214.4135	221.0775	210.5254	215.0693	216.5808	217.4571	218.2184	217.3208	223.2425	217.1839	222.0917	217.5892	223.8996	**196.61**
**C8**	63.9844	65.2551	65.6029	65.6396	66.4641	65.6224	66.8154	65.6052	66.6859	65.6570	67.5204	58.4440	60.2366	60.5256	61.1146	61.8719	61.0606	62.6313	61.0093	62.3592	61.1711	62.9887	**59.97**
**C9**	22.3327	22.5619	22.7611	22.7043	23.0742	22.6947	22.9898	22.6852	22.9701	22.7152	23.1866	19.2303	19.4890	19.4813	19.6125	19.9006	19.6045	19.9102	19.5981	19.8670	19.6232	20.2578	**15.26**
**C8`**	35.4863	36.2975	36.4201	36.6866	37.0001	36.6684	37.2088	36.6510	37.1430	36.7059	37.6692	30.9431	31.2277	31.5600	31.4215	31.7541	31.4134	31.8210	31.4050	31.8326	31.4304	31.9378	**31.09**
**H2**	8.3798	8.2664	8.3814	8.2018	8.2456	8.2053	8.2878	8.2089	8.2860	8.1983	8.2930	8.4262	8.2344	8.3139	8.1354	8.1963	8.1420	8.1687	8.1483	8.1867	8.1287	8.1656	**7.66**
**H4**	7.4469	7.6448	7.7395	7.7293	7.8500	7.7247	7.8996	7.7202	7.8822	7.7336	7.9211	7.4536	7.6353	7.7333	7.7082	7.8332	7.7043	7.8644	7.7005	7.8518	7.7117	7.8860	**7.33**
**H5**	7.5707	7.7782	7.8342	7.8767	7.9539	7.8711	7.9715	7.8656	7.9595	7.8819	7.9956	7.5711	7.7675	7.8212	7.8578	7.9361	7.8527	7.9405	7.8477	7.9316	7.8625	7.9629	**7.45**
**H6**	7.8683	8.0780	8.2089	8.2034	8.3425	8.1957	8.4017	8.1882	8.3774	8.2107	8.4461	7.7114	7.9097	8.0319	8.0073	8.1871	8.0019	8.1841	7.9963	8.1760	8.0122	8.2230	**7.57**
**H8**	4.2292	4.3359	4.4025	4.4390	4.5151	4.4322	4.5799	4.4257	4.5586	4.4456	4.6008	4.4120	4.5862	4.6603	4.6626	4.7906	4.6585	4.8082	4.6544	4.7987	4.6662	4.8353	**4.92**
**H(9A-9C)**	1.3314	1.3306	1.3316	1.3144	1.2856	1.3152	1.2974	1.3159	1.2948	1.3135	1.3232	1.3989	1.4252	1.4082	1.4443	1.4181	1.4431	1.4338	1.4420	1.4305	1.4452	1.4544	**1.44**
**H(8`A-8`C)**	2.5237	2.4766	2.4216	2.4512	2.4244	2.4524	2.4046	2.4537	2.4070	2.4499	2.3891	2.9298	2.7596	2.7397	2.6871	2.6470	2.6918	2.5606	2.6963	2.5813	2.6823	2.5287	**2.64**
**F3**	-121.3820	-124.6950	-127.0670	-125.9590	-129.1170	-125.8870	-128.7520	-125.8150	-128.6920	-126.0270	-128.7820	-121.4370	-124.7910	-127.1700	-126.0010	-129.1880	-125.9350	-128.8540	-125.8680	-128.7980	-126.0640	-128.8540	**-114.23**

[a] methyl protons shifts are averaged

[b] according to [3]

Table S3. Cont. III

Nuclei	R											S											Exp. [b]
	HF																						
	Gas	$CHCl_3$ (PCM)	$CHCl_3$ (SMD)	DMSO (PCM)	DMSO (SMD)	CH_3OH (PCM)	CH_3OH (SMD)	C_2H_5OH (PCM)	C_2H_5OH (SMD)	H_2O (PCM)	H_2O (SMD)	Gas	$CHCl_3$ (PCM)	$CHCl_3$ (SMD)	DMSO (PCM)	DMSO (SMD)	CH_3OH (PCM)	CH_3OH (SMD)	C_2H_5OH (PCM)	C_2H_5OH (SMD)	H_2O (PCM)	H_2O (SMD)	
**C1**	145.1677	145.2378	146.1793	145.1951	146.5230	145.2415	146.2831	145.2879	146.5411	145.1504	146.2684	143.8874	144.9107	145.1291	145.1548	146.6395	145.1154	146.3722	145.0877	146.8385	145.2125	146.5032	**134.46**
**C2**	124.8548	124.2329	124.7641	123.8094	124.3650	123.8340	124.8109	123.8592	124.7478	123.7855	124.8079	124.6664	124.1659	124.7260	123.7863	124.4122	123.8105	124.7666	123.8347	124.6550	123.7618	124.7910	**115.52**
**C3**	167.1337	167.0098	167.5260	166.9284	167.6437	166.9374	167.5171	166.9470	167.5577	166.9190	167.5617	167.0818	167.0306	167.4573	166.9955	167.6886	166.9946	167.5593	166.9954	167.6231	166.9981	167.6129	**162.83**
**C4**	126.3184	127.7879	128.3536	128.5420	129.0127	128.4876	129.9189	128.4333	129.6154	128.5927	130.1142	126.7038	127.6162	128.3776	128.1090	128.5953	128.0889	129.2704	128.0654	128.9167	128.1196	129.4438	**122.41**
**C5**	135.8334	137.4527	137.9371	138.2817	138.9895	138.2389	138.9602	138.1962	138.9178	138.3215	139.1093	135.8066	137.4361	137.7994	138.2373	138.9137	138.1866	138.8311	138.1375	138.8328	138.2876	138.9885	**131.43**
**C6**	129.7535	131.8567	132.8318	133.0111	134.5804	132.9177	134.7522	132.8251	134.4567	133.0994	135.1656	130.2746	132.2328	133.5988	133.2539	134.7807	133.2069	135.0040	133.1539	134.6024	133.2868	135.3099	**125.17**
**C7**	195.8399	202.0153	203.5372	205.3507	205.4212	205.1916	212.9887	205.0320	211.3990	205.5006	213.7654	200.1574	205.1172	206.2747	207.6847	208.1644	207.5170	214.5301	207.3560	213.2937	207.8540	215.2728	**196.61**
**C8**	55.0095	56.0239	56.4212	56.2553	56.9002	56.2429	57.6872	56.2310	57.5214	56.2665	58.2199	50.3637	52.1147	52.4148	52.8310	53.5355	52.7807	54.3163	52.7345	54.2258	52.8847	54.6077	**59.97**
**C9**	20.5409	20.5028	20.6164	20.5360	20.8734	20.5232	20.5925	20.5111	20.5996	20.5481	20.6991	17.7127	17.6210	17.6828	17.6093	17.8513	17.6102	17.7197	17.6114	17.7151	17.6077	17.9090	**15.26**
**C8`**	32.6536	33.4069	33.5694	33.6973	34.0330	33.6772	34.1688	33.6578	34.1278	33.7159	34.5276	29.0067	29.1901	29.5101	29.4080	29.6885	29.3969	29.7184	29.3850	29.6688	29.4150	29.8130	**31.09**
**H2**	8.5518	8.4155	8.4613	8.3404	8.4137	8.3406	8.3611	8.3408	8.3577	8.3402	8.3658	8.6211	8.3563	8.4700	8.2623	8.3167	8.2704	8.2791	8.2771	8.2537	8.2526	8.2794	**7.66**
**H4**	7.5717	7.7846	7.8839	7.8760	7.9999	7.8708	8.0500	7.8652	8.0291	7.8811	8.0741	7.5798	7.7683	7.8722	7.8468	7.9725	7.8427	8.0088	7.8383	7.9874	7.8505	8.0328	**7.33**
**H5**	7.6227	7.8424	7.8973	7.9449	8.0244	7.9392	8.0380	7.9332	8.0261	7.9504	8.0619	7.6213	7.8329	7.8846	7.9311	8.0078	7.9255	8.0137	7.9197	8.0030	7.9365	8.0378	**7.45**
**H6**	7.7959	8.0473	8.1542	8.1962	8.3642	8.1857	8.3722	8.1750	8.3462	8.2062	8.4149	7.7020	7.9162	8.0530	8.0283	8.2023	8.0229	8.2021	8.0167	8.1679	8.0325	8.2407	**7.57**
**H8**	3.6366	3.7867	3.8521	3.9217	4.0280	3.9130	4.0563	3.9041	4.0325	3.9301	4.0811	3.8329	4.0130	4.0991	4.1004	4.2326	4.0960	4.2631	4.0910	4.2381	4.1042	4.2842	**4.92**
**H(9A-9C)**	1.1917	1.1740	1.1473	1.1559	1.1242	1.1553	1.1164	1.1545	1.1112	1.1565	1.1329	1.2684	1.2605	1.2550	1.2769	1.2348	1.2767	1.2445	1.2760	1.2304	1.2765	1.2586	**1.44**
**H(8`A-8`C)**	2.2784	2.2543	2.2224	2.2433	2.2059	2.2439	2.2257	2.2445	2.2237	2.2427	2.2162	2.6838	2.5215	2.4984	2.4642	2.4215	2.4678	2.3589	2.4711	2.3640	2.4603	2.3320	**2.64**
**F3**	-99.7310	-102.3540	-104.0890	-103.3870	-105.7190	-103.3210	-105.4830	-103.2540	-105.3970	-103.4480	-105.5420	-99.9537	-102.4910	-104.3720	-103.4150	-105.7580	-103.3690	-105.5650	-103.3200	-105.4340	-103.4530	-105.5770	**-114.23**
Nuclei	**LC-BLYP**																						
**C1**	151.6406	151.6150	152.1182	151.6273	153.2014	151.6051	149.9239	151.6087	151.8050	151.6298	152.0605	150.6311	151.4407	151.8398	151.8586	153.0937	151.7947	152.9477	151.7655	152.9773	151.9101	153.1198	**134.46**
**C2**	130.5572	129.8547	130.4105	129.4435	130.0664	129.4484	123.3853	129.4803	130.3267	129.4213	130.4315	130.2390	129.7777	130.2908	129.5014	130.0877	129.4945	130.5914	129.5158	130.4994	129.4922	130.6146	**115.52**
**C3**	179.0494	178.9660	179.5524	178.9789	179.7589	178.9566	175.4331	178.9615	179.6318	178.9829	179.7007	179.0746	178.9989	179.5800	179.0214	179.7698	178.9977	179.6790	179.0016	179.6788	179.0269	179.7371	**162.83**
**C4**	132.7367	134.2318	135.0568	135.0341	135.6405	134.9645	128.8534	134.9220	136.5643	135.0830	136.9680	132.9846	134.1607	135.0182	134.7493	135.4701	134.6991	136.1429	134.6738	135.9371	134.7773	136.3295	**122.41**
**C5**	142.6444	144.5170	145.1617	145.5629	146.5042	145.4777	139.0341	145.4195	146.3819	145.6259	146.6952	142.7457	144.5442	145.2217	145.5022	146.4054	145.4248	146.3535	145.3743	146.2703	145.5578	146.5303	**131.43**
**C6**	136.1743	138.5344	139.9181	139.7387	141.4675	139.6445	132.9154	139.5784	141.9509	139.8114	142.5261	136.5101	138.9208	140.3700	140.1268	142.0728	140.0378	142.3382	139.9738	142.1186	140.1909	142.6918	**125.17**
**C7**	211.2213	216.7556	218.0599	219.7660	220.5761	219.5580	218.1908	219.3757	224.4550	219.9444	226.8586	215.6172	220.4133	221.9804	223.0103	223.7365	222.8297	229.1980	222.6786	227.9504	223.1673	229.8745	**196.61**
**C8**	61.7675	63.0661	63.6132	63.5795	64.4141	63.5336	75.5767	63.5142	64.6026	63.6053	65.5012	56.0690	57.7378	58.0853	58.6359	59.3376	58.5505	60.1132	58.4958	59.8892	58.7036	60.5024	**59.97**
**C9**	21.3939	21.6030	21.8003	21.7764	22.1042	21.7442	30.4102	21.7399	22.0256	21.7900	22.1817	18.5296	18.6772	18.7286	18.8273	19.0770	18.7934	19.0073	18.7875	18.9893	18.8429	19.3517	**15.26**
**C8`**	33.5846	34.3905	34.6247	34.8135	35.1791	34.7680	45.4877	34.7497	35.2897	34.8392	35.8447	29.0279	29.3230	29.7330	29.6296	29.9390	29.5919	30.0597	29.5801	30.0124	29.6464	30.2415	**31.09**
**H2**	8.5744	8.4657	8.5518	8.3981	8.4359	8.4016	7.9211	8.4068	8.5218	8.3939	8.4885	8.6079	8.4037	8.4921	8.3033	8.3675	8.3101	8.3382	8.3177	8.3467	8.2953	8.3367	**7.66**
**H4**	7.6035	7.7991	7.8984	7.8840	8.0081	7.8783	7.8461	7.8741	8.0402	7.8884	8.0801	7.6063	7.7883	7.8903	7.8634	7.9913	7.8586	8.0222	7.8552	8.0079	7.8670	8.0457	**7.33**
**H5**	7.7110	7.9193	7.9730	8.0188	8.0931	8.0122	7.9710	8.0071	8.0989	8.0241	8.1344	7.7124	7.9120	7.9653	8.0056	8.0806	7.9995	8.0843	7.9948	8.0746	8.0104	8.1083	**7.45**
**H6**	8.0036	8.2305	8.3501	8.3565	8.5000	8.3477	8.2629	8.3407	8.5698	8.3638	8.6162	7.8616	8.0684	8.2025	8.1729	8.3589	8.1667	8.3548	8.1617	8.3402	8.1775	8.3952	**7.57**
**H8**	4.1114	4.2259	4.2904	4.3352	4.4194	4.3266	4.8855	4.3197	4.4795	4.3425	4.5201	4.3377	4.5163	4.6030	4.5967	4.7328	4.5916	4.7521	4.5879	4.7384	4.6004	4.7808	**4.92**
**H(9A-9C)**	1.3478	1.3562	1.3463	1.3476	1.3085	1.3476	1.3914	1.3493	1.3424	1.3466	1.3563	1.4109	1.4272	1.4135	1.4452	1.4151	1.4434	1.4286	1.4431	1.4235	1.4457	1.4511	**1.44**
**H(8`A-8`C)**	2.5108	2.4643	2.4127	2.4417	2.4101	2.4421	2.6377	2.4441	2.3757	2.4404	2.3745	2.8768	2.7011	2.6883	2.6296	2.5949	2.6338	2.5084	2.6393	2.5263	2.6244	2.4822	**2.64**
**F3**	-108.5750	-111.9820	-114.1030	-113.2930	-116.0980	-113.2200	-117.5970	-113.1470	-115.7710	-113.3620	-115.9000	-108.6240	-112.0630	-114.1980	-113.3260	-116.1750	-113.2580	-115.8890	-113.1890	-115.8250	-113.3900	-115.9390	**-114.23**
Nuclei	**M06**																						
**C1**	142.6100	142.7300	143.0454	142.7340	143.7952	142.7250	143.3688	142.7244	143.0681	142.7578	143.3211	141.5753	142.5015	142.9778	143.0204	144.3722	142.9669	144.4920	142.9269	144.4076	143.0943	144.6737	**134.46**
**C2**	122.8621	122.1289	122.8254	121.7371	122.3743	121.7508	122.8697	121.7726	122.7712	121.7391	122.8266	122.1978	121.7023	122.2692	121.4963	122.1744	121.4943	122.8413	121.5020	122.7016	121.5149	122.8658	**115.52**
**C3**	170.5734	170.8459	171.4345	170.9665	171.9071	170.9501	171.8655	170.9415	171.7854	170.9966	171.9210	170.7644	170.8909	171.6339	170.9578	171.9704	170.9443	171.8259	170.9386	171.8434	170.9851	171.8826	**162.83**
**C4**	125.6640	127.3094	128.2074	128.1692	128.9348	128.1068	130.0787	128.0522	129.8388	128.2430	130.2864	125.9065	127.1879	128.0140	127.7923	128.5669	127.7505	129.2452	127.7148	129.0687	127.8445	129.4364	**122.41**
**C5**	135.6887	137.5510	138.2569	138.5484	139.6022	138.4783	139.6833	138.4160	139.5531	138.6293	139.8671	135.8725	137.5786	138.3359	138.4620	139.5146	138.4005	139.4387	138.3463	139.3793	138.5343	139.6213	**131.43**
**C6**	128.9117	130.9149	132.4880	132.0695	134.1091	131.9866	134.5013	131.9118	134.4441	132.1630	134.9347	129.3042	131.3920	132.8302	132.4756	134.4064	132.4023	134.6565	132.3351	134.4766	132.5576	134.9979	**125.17**
**C7**	202.5219	207.4680	208.5314	210.0985	210.4038	209.9294	215.8172	209.7687	214.2685	210.2746	216.4449	207.0367	211.4372	212.7257	213.5404	214.3027	213.4054	218.9142	213.2798	217.8567	213.6852	219.5410	**196.61**
**C8**	61.2220	62.4204	62.9714	62.8873	63.7173	62.8533	64.2539	62.8266	64.0542	62.9341	64.9711	55.0945	56.4420	56.9674	57.2880	58.1503	57.2180	58.9305	57.1583	58.6774	57.3730	59.3588	**59.97**
**C9**	22.1763	22.5044	22.8687	22.8545	23.3921	22.8224	23.4598	22.7979	23.4809	22.8990	23.6035	19.4991	19.6749	19.8107	19.8576	20.2441	19.8327	20.2193	19.8162	20.1885	19.8969	20.6729	**15.26**
**C8`**	36.7689	37.4563	37.7123	37.8164	38.2128	37.7839	38.4849	37.7598	38.3566	37.8624	38.9626	31.7478	32.1694	32.5224	32.5253	32.9929	32.4927	33.1917	32.4677	33.1587	32.5701	33.2539	**31.09**
**H2**	8.2548	8.1658	8.2720	8.1141	8.1983	8.1170	8.2281	8.1205	8.2426	8.1120	8.2273	8.2324	8.0482	8.1325	7.9578	8.0248	7.9635	8.0244	7.9696	8.0327	7.9523	8.0153	**7.66**
**H4**	7.2524	7.4645	7.5576	7.5559	7.6784	7.5505	7.7436	7.5457	7.7161	7.5615	7.7627	7.2868	7.4768	7.5766	7.5517	7.6834	7.5475	7.7165	7.5437	7.7023	7.5563	7.7398	**7.33**
**H5**	7.5526	7.7659	7.8199	7.8661	7.9476	7.8601	7.9704	7.8547	7.9557	7.8724	7.9978	7.5369	7.7301	7.7898	7.8103	7.8940	7.8057	7.8868	7.8017	7.8806	7.8150	7.9090	**7.45**
**H6**	7.8636	8.0641	8.2300	8.1778	8.3636	8.1701	8.4193	8.1630	8.4189	8.1859	8.4537	7.6558	7.8449	7.9575	7.9519	8.1258	7.9453	8.1377	7.9391	8.1202	7.9586	8.1847	**7.57**
**H8**	4.3341	4.4115	4.4919	4.4913	4.5727	4.4847	4.6419	4.4790	4.6192	4.4983	4.6596	4.4942	4.6900	4.7345	4.7695	4.8867	4.7649	4.8906	4.7609	4.8810	4.7742	4.9283	**4.92**
**H(9A-9C)**	1.3114	1.3092	1.3133	1.2953	1.2992	1.2962	1.2903	1.2977	1.3150	1.2950	1.3185	1.4056	1.4366	1.4199	1.4455	1.4222	1.4450	1.4101	1.4449	1.4105	1.4464	1.4388	**1.44**
**H(8`A-8`C)**	2.5014	2.4361	2.3931	2.4129	2.3778	2.4140	2.3705	2.4156	2.3656	2.4124	2.3546	2.9241	2.7396	2.7165	2.6531	2.6143	2.6586	2.5127	2.6645	2.5340	2.6482	2.4863	**2.64**
**F3**	-124.9790	-128.3630	-130.3560	-128.9050	-132.1690	-128.8450	-131.7870	-128.7830	-131.8180	-128.9630	-131.7620	-124.9870	-128.4560	-130.3320	-128.9730	-132.1950	-128.9160	-131.8410	-128.8590	-131.8080	-129.0260	-131.8080	**-114.23**
Nuclei	**M06-2X**																						
**C1**	160.2039	160.4075	160.8616	160.3809	161.8509	160.4014	160.8912	160.4238	160.8117	160.3557	161.0333	159.5562	160.4489	160.8060	160.9398	162.3797	160.9052	162.2314	160.8799	162.1593	160.9778	162.4971	**134.46**
**C2**	136.2986	135.4893	136.0083	134.9546	135.5838	135.0088	135.9044	135.0640	135.8241	134.8968	135.9063	136.0353	135.4050	135.8346	135.0522	135.6519	135.0881	136.1343	135.1272	136.0198	135.0143	136.1769	**115.52**
**C3**	184.7965	184.9329	185.5597	184.9658	185.8905	184.9847	185.8255	185.0046	185.8016	184.9419	185.9159	184.9731	185.0160	185.6807	185.0134	185.9144	185.0350	185.8171	185.0576	185.8216	184.9867	185.9028	**162.83**
**C4**	139.5616	141.2328	142.0505	142.0631	142.7998	142.0346	143.9051	142.0075	143.6702	142.0835	144.1570	139.6623	141.0405	141.9051	141.6654	142.4562	141.6559	143.2040	141.6456	143.0062	141.6654	143.4178	**122.41**
**C5**	150.6292	152.8608	153.6432	154.0076	155.1735	153.9591	155.3006	153.9119	155.1651	154.0469	155.5351	150.6966	152.7864	153.6075	153.8244	154.9891	153.7859	154.9758	153.7477	154.8831	153.8532	155.2040	**131.43**
**C6**	143.3981	145.7620	147.1273	146.9508	148.9090	146.9007	149.3533	146.8517	149.1444	146.9916	149.8293	143.7814	146.2730	147.7056	147.4606	149.4805	147.4161	149.7934	147.3704	149.5889	147.4939	150.1576	**125.17**
**C7**	219.0157	224.7861	226.0210	227.7348	228.4245	227.5720	234.2341	227.4109	232.7365	227.8838	235.2505	223.0297	228.1750	229.6967	230.8889	231.7104	230.7365	237.4898	230.5890	236.1098	231.0318	238.3293	**196.61**
**C8**	66.6741	67.9547	68.5793	68.4286	69.4017	68.4237	69.7847	68.4195	69.5857	68.4263	70.5200	61.0495	62.6358	63.0376	63.5084	64.3313	63.4653	65.0479	63.4268	64.7912	63.5472	65.4736	**59.97**
**C9**	23.0147	23.3948	23.5897	23.6329	24.0432	23.6410	24.0607	23.6499	24.0373	23.6187	24.2702	20.2632	20.6357	20.6578	20.7707	21.0327	20.7823	20.9843	20.7953	20.9724	20.7539	21.3638	**15.26**
**C8`**	35.8732	36.6055	36.7501	36.9007	37.2623	36.9035	37.4137	36.9075	37.3413	36.8920	37.9308	30.7163	31.2212	31.4977	31.4763	31.7634	31.4863	31.8713	31.4960	31.8361	31.4581	32.0763	**31.09**
**H2**	8.7559	8.6567	8.7466	8.6032	8.6533	8.6056	8.7013	8.6076	8.7188	8.6010	8.6968	8.7460	8.5978	8.6819	8.5161	8.5704	8.5213	8.5683	8.5257	8.5773	8.5107	8.5663	**7.66**
**H4**	7.8487	8.0556	8.1525	8.1474	8.2738	8.1409	8.3223	8.1340	8.3035	8.1537	8.3453	7.8550	8.0357	8.1431	8.1120	8.2410	8.1068	8.2708	8.1012	8.2583	8.1169	8.2914	**7.33**
**H5**	8.1179	8.3327	8.3961	8.4380	8.5234	8.4305	8.5437	8.4228	8.5328	8.4451	8.5678	8.1000	8.3064	8.3681	8.4047	8.4891	8.3979	8.4957	8.3908	8.4860	8.4110	8.5179	**7.45**
**H6**	8.4358	8.6417	8.7621	8.7595	8.9095	8.7511	8.9642	8.7424	8.9568	8.7675	9.0065	8.2680	8.4624	8.5993	8.5640	8.7471	8.5577	8.7545	8.5507	8.7417	8.5697	8.7892	**7.57**
**H8**	4.2595	4.3525	4.4097	4.4474	4.5186	4.4399	4.5950	4.4320	4.5876	4.4548	4.6191	4.4926	4.6553	4.7343	4.7346	4.8691	4.7291	4.8908	4.7231	4.8800	4.7399	4.9125	**4.92**
**H(9A-9C)**	1.3628	1.3640	1.3646	1.3614	1.3332	1.3605	1.3528	1.3593	1.3556	1.3623	1.3705	1.4127	1.4357	1.4257	1.4525	1.4183	1.4507	1.4382	1.4484	1.4333	1.4542	1.4540	**1.44**
**H(8`A-8`C)**	2.4796	2.4391	2.3858	2.4208	2.3884	2.4206	2.3634	2.4200	2.3556	2.4212	2.3454	2.7754	2.6266	2.6214	2.5637	2.5257	2.5670	2.4591	2.5697	2.4796	2.5603	2.4208	**2.64**
**F3**	-120.2380	-123.3530	-125.7360	-124.5130	-127.6330	-124.4490	-127.2790	-124.3840	-127.2500	-124.5730	-127.2790	-120.2820	-123.4440	-125.8070	-124.5250	-127.6640	-124.4700	-127.2530	-124.4110	-127.2290	-124.5770	-127.2190	**-114.23**

[a] methyl protons shifts are averaged

[b] according to [3]

Table S3. Cont. IV

Nuclei	R Gas	R $CHCl_3$ (PCM)	R $CHCl_3$ (SMD)	R DMSO (PCM)	R DMSO (SMD)	R CH_3OH (PCM)	R CH_3OH (SMD)	R C_2H_5OH (PCM)	R C_2H_5OH (SMD)	R H_2O (PCM)	R H_2O (SMD)	S Gas	S $CHCl_3$ (PCM)	S $CHCl_3$ (SMD)	S DMSO (PCM)	S DMSO (SMD)	S CH_3OH (PCM)	S CH_3OH (SMD)	S C_2H_5OH (PCM)	S C_2H_5OH (SMD)	S H_2O (PCM)	S H_2O (SMD)	Exp. [b]
												**mpW1LYP**											
**C1**	146.0269	146.5336	146.5552	146.4909	147.9806	146.4894	147.0113	146.4898	147.1371	146.4900	146.9834	144.6962	145.5706	146.1755	146.0537	147.3805	146.0231	147.2167	145.9948	147.1868	146.0820	147.3103	**134.46**
**C2**	123.1094	122.3966	122.8616	121.9837	122.4212	122.0066	122.8751	122.0310	122.8154	121.9599	122.8045	122.5930	122.1596	122.6132	121.9150	122.3785	121.9277	122.9347	121.9421	122.8375	121.9013	122.9038	**115.52**
**C3**	173.8719	174.1228	174.7674	174.2263	175.1374	174.2176	175.0172	174.2103	175.0277	174.2323	175.0914	174.0539	174.2203	174.8994	174.3104	175.1895	174.3022	175.0255	174.2954	175.0429	174.3164	175.0544	**162.83**
**C4**	125.7145	127.2554	127.9629	128.0518	128.4834	128.0006	129.6503	127.9511	129.4103	128.0984	129.8091	126.0216	127.1831	127.8524	127.7447	128.2606	127.7090	129.0144	127.6743	128.8429	127.7761	129.1810	**122.41**
**C5**	135.1659	137.1062	137.7034	138.1091	138.9981	138.0457	139.0952	137.9839	139.0057	138.1672	139.2444	135.3594	137.1444	137.7962	138.0654	138.9359	138.0077	138.9419	137.9513	138.8780	138.1180	139.0963	**131.43**
**C6**	129.3751	131.3203	132.7943	132.5147	134.0992	132.4387	134.6438	132.3645	134.3996	132.5852	135.0376	129.6470	131.8580	133.1308	132.9678	134.7027	132.8986	135.0596	132.8304	134.8827	133.0310	135.3911	**125.17**
**C7**	203.3925	208.9937	209.8106	211.6811	212.4428	211.5115	217.5884	211.3437	216.4668	211.8405	218.3507	208.5264	212.8448	214.3763	215.1372	215.9496	214.9971	220.6988	214.8582	219.6652	215.2681	221.2491	**196.61**
**C8**	65.2746	66.4394	66.8668	66.8744	67.6480	66.8517	67.9858	66.8290	67.8159	66.8928	68.6348	59.8158	61.6502	62.0736	62.6041	63.3735	62.5446	64.0667	62.4872	63.8345	62.6592	64.3785	**59.97**
**C9**	22.9532	23.1223	23.4677	23.3996	23.6906	23.3798	23.7037	23.3614	23.6649	23.4162	23.8427	19.5881	19.9102	19.9859	20.1059	20.3504	20.0902	20.4529	20.0764	20.3931	20.1192	20.7932	**15.26**
**C8`**	36.2978	37.0995	37.2411	37.4892	37.7732	37.4625	38.0041	37.4372	37.9639	37.5126	38.4061	31.7987	32.0119	32.3518	32.2157	32.4757	32.1994	32.5335	32.1844	32.5666	32.2289	32.5799	**31.09**
**H2**	8.2878	8.1579	8.2983	8.1091	8.1475	8.1125	8.2029	8.1156	8.2062	8.1057	8.2112	8.3471	8.1610	8.2358	8.0657	8.1145	8.0714	8.0904	8.0769	8.1028	8.0599	8.0877	**7.66**
**H4**	7.3683	7.5679	7.6614	7.6532	7.7695	7.6486	7.8204	7.6436	7.8045	7.6575	7.8409	7.3835	7.5624	7.6599	7.6364	7.7557	7.6325	7.7818	7.6283	7.7691	7.6399	7.8012	**7.33**
**H5**	7.5006	7.7079	7.7648	7.8075	7.8833	7.8020	7.9013	7.7960	7.8907	7.8127	7.9241	7.4958	7.6858	7.7418	7.7768	7.8520	7.7718	7.8615	7.7664	7.8528	7.7814	7.8821	**7.45**
**H6**	7.8174	8.0129	8.1541	8.1340	8.2670	8.1266	8.3320	8.1190	8.3158	8.1409	8.3796	7.6446	7.8383	7.9561	7.9352	8.1029	7.9296	8.1121	7.9237	8.1021	7.9402	8.1507	**7.57**
**H8**	4.3146	4.4120	4.4746	4.5020	4.5746	4.4955	4.6290	4.4889	4.6197	4.5083	4.6551	4.4685	4.6448	4.7128	4.7216	4.8412	4.7173	4.8617	4.7127	4.8508	4.7254	4.8862	**4.92**
**H(9A-9C)**	1.3264	1.3108	1.3319	1.3086	1.2748	1.3093	1.2969	1.3097	1.2929	1.3077	1.3153	1.4020	1.4346	1.4218	1.4584	1.4295	1.4571	1.4517	1.4555	1.4467	1.4595	1.4718	**1.44**
**H(8`A-8`C)**	2.5322	2.4814	2.4318	2.4652	2.4344	2.4663	2.4167	2.4673	2.4176	2.4640	2.3937	2.9695	2.8027	2.7753	2.7261	2.6815	2.7305	2.5991	2.7346	2.6164	2.7219	2.5627	**2.64**
**F3**	-129.7380	-133.2440	-135.6320	-134.5300	-137.9270	-134.4580	-137.5450	-134.3840	-137.4910	-134.5980	-137.3580	-129.7840	-133.1640	-135.7430	-134.4050	-137.8460	-134.3380	-137.7110	-134.269	-137.6470	-134.4680	-137.6800	**-114.23**
Nuclei												**mpW1PW91**											
**C1**	143.5699	144.0168	144.0785	143.9817	145.5150	143.9824	144.6596	143.9894	144.8877	143.9806	144.6840	142.4669	143.4285	143.8758	143.8157	145.0827	143.7825	145.0818	143.7593	144.9713	143.8532	145.2597	**134.46**
**C2**	122.1983	121.5726	122.0392	121.1463	121.7251	121.1727	122.1109	121.2048	122.0571	121.1207	122.0754	121.8044	121.4455	121.8869	121.1662	121.7402	121.1808	122.2592	121.2022	122.1497	121.1531	122.2704	**115.52**
**C3**	170.8862	171.1532	171.7537	171.2210	172.1680	171.2172	172.0847	171.2188	172.0873	171.2242	172.1525	171.0089	171.1828	171.8372	171.2329	172.1499	171.2302	172.0433	171.2331	172.0483	171.2346	172.1087	**162.83**
**C4**	124.7842	126.3705	127.1434	127.1543	127.7398	127.1073	128.8626	127.0658	128.5580	127.1984	129.0738	124.9842	126.2508	126.9888	126.8254	127.5208	126.7952	128.2051	126.7692	128.0411	126.8513	128.3964	**122.41**
**C5**	134.2524	136.2287	136.8633	137.2128	138.2370	137.1532	138.3503	137.0993	138.2355	137.2683	138.5358	134.3880	136.2192	136.9194	137.1225	138.1335	137.0700	138.1147	137.0223	138.0412	137.1711	138.3068	**131.43**
**C6**	127.7955	129.7468	131.1644	130.8537	132.4713	130.7861	132.9556	130.7240	132.6762	130.9174	133.3944	128.1038	130.2639	131.5313	131.3161	133.1320	131.2546	133.3953	131.1974	133.2343	131.3723	133.7132	**125.17**
**C7**	201.4506	206.7343	207.4632	209.2834	210.0345	209.1252	215.0943	208.9740	214.0069	209.4327	215.7743	205.7263	210.1038	211.4586	212.3148	213.0760	212.1820	217.8610	212.0560	216.7366	212.4407	218.4680	**196.61**
**C8**	63.0839	64.3145	64.6607	64.7258	65.5397	64.7053	65.9089	64.6893	65.8029	64.7436	66.6321	57.9004	59.6130	59.9266	60.4530	61.2024	60.3939	61.9921	60.3435	61.7102	60.5123	62.3270	**59.97**
**C9**	22.3568	22.5873	22.8840	22.8205	23.1655	22.8063	23.1630	22.7976	23.1112	22.8334	23.3562	19.1018	19.4568	19.5101	19.6448	19.9454	19.6287	20.0374	19.6194	19.9779	19.6602	20.4051	**15.26**
**C8`**	35.5026	36.3104	36.4144	36.6456	36.9609	36.6251	37.1698	36.6103	37.1030	36.6644	37.6249	30.9155	31.1907	31.5390	31.3758	31.7148	31.3656	31.7582	31.3603	31.7703	31.3833	31.8402	**31.09**
**H2**	8.2991	8.1886	8.3132	8.1328	8.1738	8.1368	8.2183	8.1408	8.2071	8.1293	8.2200	8.3365	8.1549	8.2387	8.0645	8.1272	8.0712	8.0989	8.0776	8.1171	8.0577	8.0961	**7.66**
**H4**	7.3921	7.5973	7.6904	7.6812	7.8038	7.6768	7.8544	7.6723	7.8359	7.6857	7.8772	7.3996	7.5855	7.6837	7.6595	7.7858	7.6557	7.8182	7.6520	7.8049	7.6631	7.8404	**7.33**
**H5**	7.5608	7.7733	7.8309	7.8711	7.9535	7.8656	7.9731	7.8603	7.9599	7.8764	7.9981	7.5546	7.7498	7.8077	7.8398	7.9228	7.8349	7.9286	7.8301	7.9193	7.8446	7.9520	**7.45**
**H6**	7.8927	8.0902	8.2272	8.2042	8.3364	8.1975	8.3948	8.1909	8.3649	8.2109	8.4384	7.7148	7.9057	8.0277	8.0006	8.1767	7.9958	8.1718	7.9908	8.1651	8.0050	8.2104	**7.57**
**H8**	4.3334	4.4323	4.4938	4.5187	4.5864	4.5127	4.6447	4.5070	4.6226	4.5248	4.6677	4.5200	4.6792	4.7487	4.7481	4.8711	4.7448	4.8866	4.7414	4.8784	4.7513	4.9140	**4.92**
**H(9A-9C)**	1.3079	1.2964	1.3106	1.2870	1.2528	1.2882	1.2700	1.2894	1.2638	1.2861	1.2945	1.3817	1.4112	1.3957	1.4304	1.4022	1.4295	1.4205	1.4286	1.4170	1.4313	1.4424	**1.44**
**H(8`A-8`C)**	2.4705	2.4159	2.3645	2.3947	2.3647	2.3961	2.3462	2.3976	2.3513	2.3935	2.3302	2.8641	2.7030	2.6811	2.6308	2.5901	2.6360	2.5014	2.6409	2.5234	2.6258	2.4687	**2.64**
**F3**	-120.0560	-123.0390	-125.3950	-124.1620	-127.3090	-124.1000	-126.8840	-124.0360	-126.8480	-124.2210	-126.8620	-120.1080	-123.1640	-125.5300	-124.2430	-127.4320	-124.1850	-127.0530	-124.1260	-127.0160	-124.2990	-127.0170	**-114.23**
Nuclei												**O3LYP**											
**C1**	140.8997	141.9466	143.2729	142.4032	144.4207	142.3049	143.1195	142.1745	143.5932	142.4183	143.4379	139.0203	140.4638	141.9430	141.6630	143.3736	141.6465	142.9837	141.5765	143.1903	141.6575	143.0888	**134.46**
**C2**	117.9082	117.5570	118.1520	117.3237	117.8850	117.3477	118.1887	117.3620	118.1720	117.2906	118.1760	117.4220	117.1821	117.8195	117.0863	117.6912	117.1065	118.0262	117.1162	117.9728	117.0591	118.0140	**115.52**
**C3**	167.5165	167.7112	168.4244	167.8550	168.8211	167.8565	168.7431	167.8540	168.7252	167.8481	168.8048	167.7102	167.8453	168.5409	167.9755	168.9022	167.9768	168.7816	167.9752	168.7821	167.9682	168.8406	**162.83**
**C4**	120.3932	121.7407	122.2980	122.4581	122.8238	122.4457	123.9552	122.4384	123.5727	122.4812	124.0535	120.6586	121.6250	122.0982	122.0374	122.5064	122.0159	123.1802	122.0013	122.9548	122.0548	123.3653	**122.41**
**C5**	129.9513	131.7207	132.4390	132.6715	133.6148	132.6285	133.7702	132.5829	133.6330	132.7094	133.9281	130.1391	131.7960	132.4601	132.6642	133.5740	132.6214	133.5937	132.5764	133.5006	132.6990	133.7718	**131.43**
**C6**	123.0408	124.8626	125.8820	125.9817	127.3998	125.9424	127.9714	125.9083	127.6222	126.0322	128.2235	123.6577	125.5240	126.5029	126.2800	127.8225	126.2291	128.1629	126.1919	127.9056	126.3276	128.4425	**125.17**
**C7**	197.6897	203.5719	205.5736	206.6169	207.9554	206.3492	212.3594	206.0691	211.4753	206.7853	213.1635	201.9831	206.3849	208.2014	209.1385	210.1475	209.0303	214.3678	208.8806	213.5525	209.2214	214.8852	**196.61**
**C8**	63.3589	64.5386	65.2615	65.1762	66.1014	65.1096	66.3981	65.0214	66.5018	65.1945	67.1484	58.1496	60.4441	61.3149	61.6476	62.5796	61.5886	63.3277	61.5062	63.1529	61.6929	63.5679	**59.97**
**C9**	21.8183	22.0587	22.2184	22.3011	22.5583	22.3024	22.7288	22.3027	22.6200	22.3067	22.8473	18.4006	18.7140	18.8718	18.9018	19.1361	18.8951	19.3083	18.8851	19.2278	18.9034	19.5833	**15.26**
**C8`**	35.8881	36.6338	36.9019	37.1057	37.4549	37.0775	37.6035	37.0483	37.5427	37.1164	37.9353	31.5880	31.7215	31.9149	31.9045	32.1649	31.9002	32.1712	31.8977	32.1533	31.9038	32.2022	**31.09**
**H2**	8.0517	7.8936	7.9199	7.8228	7.8430	7.8353	7.9300	7.8501	7.8958	7.8176	7.9122	8.1796	7.9467	7.9398	7.7986	7.8379	7.8028	7.8504	7.8113	7.8388	7.7959	7.8536	**7.66**
**H4**	7.3263	7.5151	7.6090	7.6021	7.7162	7.5976	7.7700	7.5933	7.7461	7.6064	7.7887	7.3402	7.4960	7.5832	7.5650	7.6786	7.5613	7.7108	7.5577	7.6958	7.5683	7.7331	**7.33**
**H5**	7.5191	7.6984	7.7476	7.7823	7.8549	7.7791	7.8758	7.7759	7.8601	7.7864	7.8969	7.5075	7.6828	7.7325	7.7644	7.8379	7.7598	7.8474	7.7552	7.8359	7.7687	7.8693	**7.45**
**H6**	7.7403	7.9188	8.0045	8.0357	8.1619	8.0319	8.2349	8.0308	8.1820	8.0423	8.2510	7.5926	7.7537	7.8265	7.8111	7.9580	7.8063	7.9681	7.8036	7.9466	7.8162	8.0054	**7.57**
**H8**	4.4153	4.4962	4.5220	4.5699	4.6391	4.5679	4.6787	4.5680	4.6410	4.5750	4.6963	4.5712	4.7077	4.7652	4.7684	4.8911	4.7649	4.9133	4.7620	4.8966	4.7718	4.9427	**4.92**
**H(9A-9C)**	1.2189	1.2025	1.1806	1.2003	1.1616	1.2026	1.2011	1.2046	1.1947	1.1995	1.2124	1.3233	1.3611	1.3327	1.3666	1.3326	1.3645	1.3666	1.3635	1.3547	1.3689	1.3845	**1.44**
**H(8`A-8`C)**	2.4971	2.4632	2.4406	2.4534	2.4264	2.4531	2.4158	2.4519	2.4227	2.4528	2.3977	2.9315	2.7673	2.7104	2.6886	2.6373	2.6937	2.5630	2.6994	2.5772	2.6839	2.5298	**2.64**
**F3**	-125.4700	-128.6940	-131.2040	-129.8780	-133.1980	-129.8160	-132.6800	-129.7490	-132.6960	-129.9370	-132.6490	-125.2160	-128.7070	-131.2940	-129.9000	-133.3250	-129.8380	-132.9370	-129.7710	-132.8980	-129.9580	-132.8750	**-114.23**
Nuclei												**OLYP**											
**C1**	139.7233	141.0789	141.6734	141.2583	143.3840	141.2435	142.5023	141.2258	142.5923	141.2664	142.5553	137.6104	139.4304	140.6371	140.4210	141.9170	140.4196	141.6925	140.4105	141.8243	140.4109	141.7913	**134.46**
**C2**	115.7645	115.4147	115.9344	115.1368	115.6978	115.1623	115.9635	115.1824	115.9629	115.1040	115.9375	115.2249	115.0273	115.6178	114.9025	115.4276	114.9246	115.7890	114.9412	115.7370	114.8729	115.7565	**115.52**
**C3**	166.6520	166.9005	167.6958	167.1236	168.0959	167.1200	168.0114	167.1108	168.0145	167.1179	168.0780	166.8854	167.1065	167.8640	167.2535	168.2360	167.2502	168.0977	167.2418	168.0981	167.2478	168.1533	**162.83**
**C4**	118.3116	119.5549	120.2884	120.3169	120.5159	120.2884	121.4317	120.2533	121.2532	120.3336	121.6372	118.6368	119.4496	119.9624	119.8574	120.3478	119.8289	120.9627	119.7957	120.7595	119.8761	121.1381	**122.41**
**C5**	127.8865	129.5987	130.4042	130.5497	131.4455	130.5032	131.6125	130.4505	131.5020	130.5844	131.7897	128.1770	129.7359	130.4447	130.5808	131.5174	130.5343	131.5406	130.4824	131.4396	130.6158	131.7131	**131.43**
**C6**	120.7979	122.6442	123.7878	123.8070	125.0823	123.7476	125.4938	123.6820	125.2820	123.8536	125.8314	121.5280	123.1048	124.2090	123.9342	125.5401	123.8771	125.7960	123.8149	125.5882	123.9796	126.0685	**125.17**
**C7**	196.6851	202.9787	204.1837	205.6074	206.7091	205.4542	210.7690	205.2967	210.0025	205.7460	211.4251	200.9576	205.3766	206.8862	207.7614	208.7224	207.6594	212.7123	207.5507	211.8864	207.8480	213.1717	**196.61**
**C8**	64.5964	65.8444	66.3688	66.3872	67.5527	66.3530	68.1551	66.3151	67.9491	66.4123	68.7145	59.1188	61.8546	62.7447	63.1433	63.9669	63.0966	64.7876	63.0433	64.6140	63.1775	65.0044	**59.97**
**C9**	21.9765	22.2709	22.4545	22.6141	22.8208	22.6015	22.9884	22.5835	22.9273	22.6178	23.1296	18.3905	18.8370	19.0529	19.1040	19.3001	19.0970	19.4960	19.0841	19.4420	19.1016	19.7940	**15.26**
**C8`**	36.5321	37.2950	37.6251	37.7477	38.0214	37.7321	38.0661	37.7104	38.0890	37.7531	38.4144	32.2384	32.3312	32.5297	32.4904	32.7767	32.4790	32.7546	32.4626	32.7295	32.4929	32.7534	**31.09**
**H2**	7.8759	7.7225	7.8156	7.6820	7.6960	7.6862	7.7447	7.6901	7.7479	7.6772	7.7508	8.0240	7.7734	7.8048	7.6596	7.7195	7.6617	7.7281	7.6639	7.7205	7.6574	7.7292	**7.66**
**H4**	7.2468	7.4439	7.5373	7.5281	7.6289	7.5239	7.6697	7.5196	7.6587	7.5316	7.6927	7.2578	7.4112	7.4984	7.4769	7.5908	7.4731	7.6214	7.4691	7.6068	7.4802	7.6423	**7.33**
**H5**	7.4574	7.6162	7.6798	7.7043	7.7760	7.6995	7.7946	7.6946	7.7831	7.7084	7.8168	7.4520	7.6143	7.6669	7.6933	7.7688	7.6885	7.7772	7.6837	7.7662	7.6973	7.7975	**7.45**
**H6**	7.6549	7.8627	7.9685	7.9801	8.0016	7.9749	8.0865	7.9693	8.0812	7.9844	8.1261	7.5289	7.6390	7.7270	7.7014	7.8599	7.6955	7.8629	7.6897	7.8444	7.7066	7.8986	**7.57**
**H8**	4.5165	4.6114	4.6292	4.6680	4.7021	4.6652	4.7091	4.6622	4.6990	4.6702	4.7402	4.6608	4.7777	4.8401	4.8411	4.9706	4.8368	4.9867	4.8324	4.9708	4.8448	5.0181	**4.92**
**H(9A-9C)**	1.2131	1.2018	1.1989	1.2076	1.1768	1.2078	1.2151	1.2077	1.2087	1.2071	1.2273	1.3168	1.3562	1.3465	1.3777	1.3507	1.3756	1.3820	1.3733	1.3739	1.3793	1.3991	**1.44**
**H(8`A-8`C)**	2.5388	2.5013	2.4785	2.4898	2.4672	2.4906	2.4613	2.4911	2.4605	2.4887	2.4354	2.9772	2.8106	2.7538	2.7281	2.6815	2.7321	2.6050	2.7360	2.6197	2.7241	2.5686	**2.64**
**F3**	-133.2410	-136.5690	-139.2300	-137.7280	-141.4070	-137.6610	-140.9120	-137.5930	-140.8340	-137.7910	-140.8320	-133.0170	-136.5700	-139.3360	-137.8530	-141.5280	-137.7890	-141.1350	-137.7240	-141.0970	-137.9130	-141.0540	**-114.23**

[a] methyl protons shifts are averaged

[b] according to [3]

Table S3. Cont. V

Nuclei	R											S											Exp. [b]
	OPBE																						
	Gas	$CHCl_3$ (PCM)	$CHCl_3$ (SMD)	DMSO (PCM)	DMSO (SMD)	CH_3OH (PCM)	CH_3OH (SMD)	C_2H_5OH (PCM)	C_2H_5OH (SMD)	H_2O (PCM)	H_2O (SMD)	Gas	$CHCl_3$ (PCM)	$CHCl_3$ (SMD)	DMSO (PCM)	DMSO (SMD)	CH_3OH (PCM)	CH_3OH (SMD)	C_2H_5OH (PCM)	C_2H_5OH (SMD)	H_2O (PCM)	H_2O (SMD)	
**C1**	137.0168	138.5097	138.9262	138.8224	140.7563	138.8125	139.9428	138.7824	140.1179	138.8238	140.0850	135.3672	137.6783	138.1895	138.0126	139.3776	138.0216	139.3757	138.0099	139.4882	137.9961	139.5225	**134.46**
**C2**	114.6170	114.3904	114.8828	114.0743	114.6778	114.1109	114.8941	114.1297	114.8951	114.0345	114.9023	114.2576	114.1953	114.6619	113.9396	114.5080	113.9726	114.8059	113.9873	114.7495	113.9033	114.8074	**115.52**
**C3**	163.0652	163.4107	164.1167	163.5270	164.4693	163.5371	164.4137	163.5296	164.3919	163.5127	164.5182	163.2349	163.5035	164.2073	163.6022	164.5519	163.6116	164.4283	163.6033	164.4261	163.5888	164.5180	**162.83**
**C4**	117.0875	118.4716	119.1644	119.0733	119.4321	119.0586	120.3014	119.0262	120.0439	119.0835	120.5267	117.2011	118.1217	118.7728	118.6300	119.2379	118.6126	119.7559	118.5778	119.5598	118.6426	119.9561	**122.41**
**C5**	126.6629	128.4455	129.2158	129.2889	130.2429	129.2557	130.4292	129.2044	130.3007	129.3158	130.6388	126.8326	128.4577	129.1821	129.2747	130.2740	129.2405	130.2868	129.1887	130.1857	129.3024	130.4917	**131.43**
**C6**	118.9473	120.7741	121.8309	121.7446	123.0406	121.7025	123.4150	121.6435	123.1562	121.7812	123.7562	119.5104	121.0091	122.2007	121.8840	123.4734	121.8430	123.6469	121.7851	123.4558	121.9191	123.9365	**125.17**
**C7**	194.4087	200.3927	201.3265	202.9629	204.0112	202.8239	207.9731	202.6644	207.0787	203.0868	208.6560	197.8357	202.7029	203.7837	204.6315	205.5188	204.5407	209.6365	204.4296	208.8338	204.7102	210.0688	**196.61**
**C8**	62.0246	63.4759	63.8328	64.0390	64.9607	64.01308	65.6396	63.9678	65.5766	64.0562	66.2790	57.4808	59.8474	60.2403	60.6966	61.3470	60.6657	62.3193	60.6159	62.1121	60.7199	62.5834	**59.97**
**C9**	21.2184	21.5692	21.6964	21.7937	22.0246	21.7954	22.1798	21.7804	22.0714	21.7887	22.3487	17.8387	18.2469	18.3199	18.4106	18.5662	18.4149	18.7700	18.4018	18.6893	18.4017	19.1117	**15.26**
**C8`**	35.4607	36.2977	36.4862	36.6343	36.8947	36.6333	36.9156	36.6134	36.9219	36.6293	37.3065	30.9597	31.1232	31.3803	31.3230	31.6166	31.3250	31.5559	31.3095	31.5441	31.3168	31.5647	**31.09**
**H2**	7.8619	7.7166	7.8155	7.6603	7.6905	7.6655	7.7354	7.6701	7.7254	7.6549	7.7410	7.9540	7.6922	7.7799	7.6410	7.7078	7.6436	7.7010	7.6456	7.6926	7.6381	7.7040	**7.66**
**H4**	7.2666	7.4701	7.5590	7.5531	7.6586	7.5493	7.6975	7.5448	7.6779	7.5560	7.7235	7.2631	7.4306	7.5193	7.5021	7.6172	7.4985	7.6457	7.4942	7.6312	7.5048	7.6689	**7.33**
**H5**	7.5078	7.6769	7.7375	7.7622	7.8354	7.7579	7.8542	7.7528	7.8399	7.7656	7.8788	7.4993	7.6672	7.7222	7.7493	7.8266	7.7449	7.8326	7.7399	7.8217	7.7526	7.8554	**7.45**
**H6**	7.6973	7.8916	7.9919	7.9940	8.0938	7.9897	8.1090	7.9849	8.0766	7.9974	8.1508	7.5284	7.6456	7.7588	7.7358	7.8955	7.7304	7.8875	7.7244	7.8700	7.7403	7.9247	**7.57**
**H8**	4.5310	4.6254	4.6380	4.6769	4.7172	4.6748	4.7188	4.6723	4.6855	4.6786	4.7541	4.6902	4.8053	4.8724	4.8739	5.0018	4.8702	5.0121	4.8658	4.9970	4.8767	5.0472	**4.92**
**H(9A-9C)**	1.1719	1.1616	1.1564	1.1635	1.1255	1.1641	1.1665	1.1640	1.1637	1.1622	1.1815	1.2856	1.3043	1.2938	1.3335	1.2959	1.3320	1.3270	1.3298	1.3170	1.3343	1.3487	**1.44**
**H(8`A-8`C)**	2.4599	2.4252	2.3906	2.4107	2.3774	2.4119	2.3736	2.4124	2.3768	2.4087	2.3501	2.8429	2.6777	2.6358	2.6088	2.5655	2.6129	2.4825	2.6164	2.4962	2.6043	2.4468	**2.64**
**F3**	-122.6130	-125.4550	-127.8350	-126.4160	-129.6860	-126.3610	-129.1560	-126.3060	-129.1800	-126.4670	-129.0510	-122.4410	-125.5720	-127.9890	-126.5560	-129.8500	-126.5050	-129.4250	-126.4540	-129.4040	-126.6020	-129.3270	**-114.23**
Nuclei	**OPW91**																						
**C1**	137.2927	138.7921	139.2035	139.0741	141.0334	139.0551	140.2122	139.0181	140.3789	139.0716	140.3514	135.6218	137.9465	138.4497	138.2826	139.6367	138.2817	139.6226	138.2612	139.7359	138.2620	139.7679	**134.46**
**C2**	114.8303	114.6035	115.0905	114.2896	114.8811	114.3160	115.0991	114.3257	115.0998	114.2457	115.1094	114.4634	114.4014	114.8641	114.1493	114.7047	114.1720	115.0072	114.1778	114.9516	114.1090	115.0108	**115.52**
**C3**	163.4635	163.8083	164.5122	163.9322	164.8624	163.9321	164.8065	163.9150	164.7866	163.9140	164.9121	163.6352	163.9044	164.6060	164.0086	164.9492	164.0077	164.8252	163.9903	164.8239	163.9912	164.9169	**162.83**
**C4**	117.2951	118.6815	119.3705	119.3009	119.6251	119.2756	120.5032	119.2333	120.2527	119.3071	120.7313	117.4203	118.3352	118.9825	118.8493	119.4412	118.8217	119.9684	118.7779	119.7726	118.8581	120.1723	**122.41**
**C5**	126.8583	128.6464	129.4103	129.5007	130.4367	129.4571	130.6217	129.3963	130.4954	129.5239	130.8334	127.0353	128.6631	129.3828	129.4872	130.4718	129.4429	130.4861	129.3816	130.3856	129.5111	130.6930	**131.43**
**C6**	119.1957	121.0351	122.0933	122.0275	123.2987	121.9747	123.6819	121.9057	123.4265	122.0604	124.0270	119.7712	121.2773	122.4677	122.1625	123.7425	122.1109	123.9225	122.0434	123.7298	122.1942	124.2154	**125.17**
**C7**	194.7470	200.7723	201.7073	203.3291	204.3636	203.1806	208.3618	203.0130	207.4704	203.4495	209.0423	198.2213	203.0945	204.1684	205.0327	205.9047	204.9322	210.0310	204.8120	209.2232	205.1075	210.4680	**196.61**
**C8**	62.2071	63.6563	64.0081	64.2018	65.1534	64.1663	65.8205	64.1134	65.7476	64.2157	66.4583	57.6233	60.0248	60.4177	60.8806	61.5261	60.8394	62.4923	60.7799	62.2839	60.9006	62.7553	**59.97**
**C9**	21.2792	21.6330	21.7606	21.8706	22.0843	21.8624	22.2432	21.8382	22.1362	21.8615	22.4153	17.9107	18.3290	18.4025	18.4985	18.6462	18.4926	18.8517	18.4702	18.7720	18.4862	19.1928	**15.26**
**C8`**	35.5266	36.3700	36.5580	36.7130	36.9604	36.7014	36.9890	36.6718	36.9980	36.7049	37.3801	31.0590	31.2167	31.4730	31.4226	31.7084	31.4144	31.6513	31.3896	31.6400	31.4123	31.6650	**31.09**
**H2**	7.8669	7.7201	7.8191	7.6674	7.6929	7.6718	7.7382	7.6761	7.7293	7.6616	7.7437	7.9620	7.6970	7.7851	7.6464	7.7121	7.6482	7.7060	7.6501	7.6978	7.6432	7.7090	**7.66**
**H4**	7.2686	7.4718	7.5607	7.5557	7.6587	7.5512	7.6985	7.5465	7.6795	7.5582	7.7242	7.2659	7.4323	7.5210	7.5044	7.6183	7.5002	7.6472	7.4957	7.6326	7.5067	7.6703	**7.33**
**H5**	7.5077	7.6765	7.7370	7.7628	7.8343	7.7578	7.8535	7.7525	7.8393	7.7659	7.8779	7.4994	7.6671	7.7220	7.7499	7.8261	7.7449	7.8324	7.7397	7.8215	7.7530	7.8550	**7.45**
**H6**	7.6978	7.8929	7.9939	7.9989	8.0926	7.9939	8.1100	7.9886	8.0796	8.0020	8.1514	7.5322	7.6480	7.7612	7.7390	7.8976	7.7329	7.8906	7.7267	7.8730	7.7432	7.9278	**7.57**
**H8**	4.5305	4.6248	4.6386	4.6792	4.7153	4.6764	4.7179	4.6736	4.6861	4.6804	4.7528	4.6867	4.8012	4.8683	4.8708	4.9972	4.8664	5.0086	4.8618	4.9934	4.8733	5.0435	**4.92**
**H(9A-9C)**	1.1734	1.1630	1.1578	1.1659	1.1278	1.1659	1.1683	1.1656	1.1652	1.1644	1.1833	1.2862	1.3054	1.2954	1.3354	1.2975	1.3331	1.3287	1.3307	1.3188	1.3359	1.3503	**1.44**
**H(8`A-8`C)**	2.4610	2.4265	2.3921	2.4124	2.3795	2.4130	2.3757	2.4132	2.3785	2.4103	2.3525	2.8480	2.6819	2.6403	2.6141	2.5699	2.6176	2.4874	2.6209	2.5013	2.6094	2.4523	**2.64**
**F3**	-123.2130	-126.1040	-128.4990	-127.0830	-130.3850	-127.0270	-129.8500	-126.9710	-129.8680	-127.1360	-129.7490	-123.0400	-126.2170	-128.6490	-127.2210	-130.5370	-127.1690	-130.1110	-127.1170	-130.0890	-127.2690	-130.0160	**-114.23**
Nuclei	**PBE**																						
**C1**	142.1816	142.8010	143.8198	142.8834	144.5207	142.8776	143.9094	142.8834	144.0984	142.9074	143.9127	140.7424	141.9216	142.4937	142.3319	143.7918	142.2972	143.9628	142.2757	143.8304	142.3862	144.1505	**134.46**
**C2**	119.2950	118.7283	119.2020	118.2285	118.8283	118.2562	119.2398	118.2946	119.1888	118.2203	119.2122	118.7147	118.4960	118.8862	118.1803	118.7819	118.1942	119.3915	118.2199	119.2629	118.1859	119.4026	**115.52**
**C3**	172.1464	172.6278	173.3848	172.7002	173.8707	172.6935	173.7994	172.6975	173.7890	172.7242	173.8899	172.3082	172.6943	173.4582	172.7298	173.8790	172.7254	173.7470	172.7317	173.7524	172.7516	173.8329	**162.83**
**C4**	122.6934	124.3511	124.9743	125.0895	125.7479	125.0427	126.8224	125.0067	126.5469	125.1513	127.0617	122.9119	124.2681	124.9736	124.7986	125.5432	124.7665	126.2227	124.7444	126.0613	124.8457	126.4363	**122.41**
**C5**	132.2849	134.3920	135.1418	135.3713	136.5603	135.3097	136.7276	135.2589	136.5974	135.4475	136.9403	132.5421	134.4790	135.2588	135.3430	136.5326	135.2895	136.5190	135.2460	136.4441	135.4107	136.7335	**131.43**
**C6**	125.6771	127.6409	128.6911	128.5688	130.3315	128.5053	130.7038	128.4524	130.4480	128.6465	131.1919	125.9574	128.1465	129.3617	129.1140	131.0219	129.0540	131.2761	129.0040	131.1183	129.1879	131.6187	**125.17**
**C7**	201.4517	206.3214	207.9921	208.8009	209.9020	208.6525	214.2617	208.5163	213.4149	208.9608	214.8731	206.3668	210.3182	211.9033	212.2804	213.4325	212.1598	217.4356	212.0495	216.5627	212.4127	217.9681	**196.61**
**C8**	67.1718	68.5140	69.1180	68.8732	69.8486	68.8510	70.2565	68.8390	70.1298	68.9111	71.0458	61.6156	63.4701	63.7948	64.2819	65.2151	64.2241	66.0027	64.17873	65.7294	64.3566	66.3598	**59.97**
**C9**	23.3212	23.7874	23.9124	23.9572	24.4157	23.9398	24.5298	23.9331	24.4525	23.9912	24.7904	20.0440	20.6474	20.6698	20.8257	21.2360	20.8085	21.4694	20.8028	21.3763	20.8607	21.9582	**15.26**
**C8`**	37.0709	37.8528	38.0432	38.1530	38.5274	38.1334	38.7413	38.1247	38.6798	38.1898	39.2159	32.0699	32.3535	32.7068	32.4432	32.8601	32.4358	32.8546	32.4386	32.8871	32.4667	32.9169	**31.09**
**H2**	8.0613	7.9709	8.0060	7.9085	7.9489	7.9124	7.9856	7.9168	7.9764	7.9054	7.9921	8.1252	7.9615	8.0342	7.8721	7.9248	7.8783	7.8974	7.8847	7.9129	7.8665	7.8947	**7.66**
**H4**	7.2105	7.4115	7.5061	7.4936	7.6170	7.4891	7.6646	7.4850	7.6478	7.4985	7.6872	7.2161	7.3988	7.4967	7.4693	7.5963	7.4656	7.6262	7.4622	7.6139	7.4735	7.6478	**7.33**
**H5**	7.4152	7.6204	7.6775	7.7144	7.8007	7.7091	7.8198	7.7042	7.8073	7.7202	7.8446	7.4218	7.6096	7.6685	7.6931	7.7793	7.6884	7.7834	7.6841	7.7750	7.6981	7.8059	**7.45**
**H6**	7.7882	7.9703	8.0440	8.0699	8.1952	8.0640	8.2411	8.0585	8.2170	8.0761	8.2863	7.5805	7.7575	7.8725	7.8436	8.0145	7.8389	8.0055	7.8346	7.9998	7.8485	8.0452	**7.57**
**H8**	4.5982	4.6739	4.6984	4.7382	4.8050	4.7335	4.8465	4.7295	4.8356	4.7435	4.8689	4.7599	4.9123	4.9777	4.9774	5.1006	4.9739	5.1117	4.9708	5.1039	4.9813	5.1413	**4.92**
**H(9A-9C)**	1.3755	1.3742	1.3462	1.3558	1.3275	1.3569	1.3428	1.3584	1.3355	1.3552	1.3707	1.4676	1.5049	1.4844	1.5225	1.4988	1.5215	1.5185	1.5210	1.5133	1.5241	1.5445	**1.44**
**H(8`A-8`C)**	2.5809	2.5203	2.4881	2.4883	2.4634	2.4898	2.4423	2.4919	2.4481	2.4875	2.4202	2.9909	2.8234	2.8026	2.7459	2.7059	2.7508	2.6109	2.7560	2.6322	2.7418	2.5746	**2.64**
**F3**	-138.7280	-142.0230	-144.8290	-143.2550	-147.0170	-143.1870	-146.4840	-143.1160	-146.4590	-143.3210	-146.4110	-138.6180	-142.0770	-144.8620	-143.3070	-147.0980	-143.2400	-146.6950	-143.1710	-146.6390	-143.3710	-146.6200	**-114.23**
Nuclei	**PBE0**																						
**C1**	143.1198	143.4402	143.7210	143.5364	145.0234	143.5338	144.2813	143.5337	144.2780	143.5419	144.2049	142.0685	142.9891	143.4758	143.4059	144.6630	143.3676	144.7287	143.3353	144.5476	143.4505	144.8343	**134.46**
**C2**	121.8507	121.2420	121.6941	120.8009	121.3653	120.8230	121.8101	120.8473	121.6886	120.7828	121.7174	121.4451	121.1106	121.5389	120.8391	121.3850	120.8485	121.9863	120.8609	121.8141	120.8343	121.9375	**115.52**
**C3**	170.5382	170.7930	171.4194	170.8673	171.7866	170.8584	171.8008	170.8514	171.7367	170.8786	171.8075	170.6486	170.8332	171.4838	170.8758	171.7737	170.8682	171.7516	170.8625	171.6988	170.8857	171.7619	**162.83**
**C4**	124.5260	126.1173	126.8865	126.9041	127.4816	126.8521	128.6626	126.8018	128.3526	126.9564	128.8241	124.7157	126.0193	126.7515	126.5880	127.2766	126.5523	128.0398	126.5170	127.8223	126.6232	128.1802	**122.41**
**C5**	134.0593	136.0273	136.6965	137.0363	138.0523	136.9715	138.2554	136.9083	138.0842	137.1008	138.3770	134.1915	136.0387	136.7471	136.9380	137.9472	136.8803	138.0112	136.8236	137.8814	136.9953	138.1465	**131.43**
**C6**	127.5835	129.6369	130.9138	130.6381	132.2739	130.5658	132.7945	130.4952	132.5243	130.7093	133.2195	127.8690	130.0621	131.3120	131.1004	132.9014	131.0341	133.2515	130.9684	133.0368	131.1650	133.5245	**125.17**
**C7**	201.0956	206.0865	207.1149	208.7983	209.5312	208.6395	214.5599	208.4828	213.3382	208.9524	215.1321	205.3965	209.6985	211.1075	211.8981	212.7064	211.7605	217.4540	211.6262	216.2948	212.0327	217.9863	**196.61**
**C8**	62.9977	64.2517	64.5885	64.6346	65.4442	64.6084	65.8894	64.5836	65.6717	64.6617	66.5520	57.6970	59.3578	59.6693	60.1920	60.9105	60.1298	61.7516	60.0719	61.4146	60.2570	62.0221	**59.97**
**C9**	22.3499	22.6729	22.8713	22.8296	23.1821	22.8100	23.2575	22.7919	23.1732	22.8511	23.4263	19.0803	19.4589	19.4902	19.6439	19.9135	19.6236	20.0860	19.6057	19.9729	19.6669	20.4182	**15.26**
**C8`**	35.4113	36.1702	36.3016	36.5288	36.8075	36.5039	37.0966	36.4810	36.9830	36.5557	37.4814	30.6707	30.9567	31.2958	31.1302	31.4530	31.1144	31.5755	31.0997	31.5332	31.1471	31.6022	**31.09**
**H2**	8.3010	8.2028	8.3059	8.1346	8.1748	8.1381	8.2203	8.1416	8.2233	8.1308	8.2234	8.3350	8.1625	8.2397	8.0678	8.1270	8.0742	8.1047	8.0803	8.1208	8.0609	8.1003	**7.66**
**H4**	7.3834	7.5862	7.6817	7.6721	7.7930	7.6673	7.8476	7.6625	7.8276	7.6764	7.8670	7.3888	7.5758	7.6742	7.6498	7.7752	7.6457	7.8116	7.6415	7.7958	7.6532	7.8309	**7.33**
**H5**	7.5550	7.7647	7.8243	7.8629	7.9435	7.8572	7.9680	7.8514	7.9533	7.8680	7.9899	7.5522	7.7484	7.8060	7.8379	7.9195	7.8327	7.9300	7.8275	7.9182	7.8424	7.9505	**7.45**
**H6**	7.8969	8.0970	8.2242	8.2086	8.3380	8.2015	8.3994	8.1944	8.3801	8.2149	8.4445	7.7110	7.9030	8.0242	7.9958	8.1712	7.9907	8.1717	7.9852	8.1628	8.0001	8.2087	**7.57**
**H8**	4.3272	4.4212	4.4835	4.5096	4.5742	4.5036	4.6369	4.4977	4.6185	4.5150	4.6586	4.5220	4.6815	4.7530	4.7491	4.8729	4.7454	4.8926	4.7416	4.8820	4.7521	4.9175	**4.92**
**H(9A-9C)**	1.3171	1.3205	1.3153	1.2999	1.2663	1.3007	1.2850	1.3015	1.2820	1.2988	1.3116	1.3929	1.4222	1.4036	1.4390	1.4077	1.4379	1.4305	1.4368	1.4245	1.4396	1.4507	**1.44**
**H(8`A-8`C)**	2.4766	2.4262	2.3704	2.3988	2.3690	2.4000	2.3524	2.4012	2.3501	2.3974	2.3322	2.8658	2.7045	2.6832	2.6303	2.5894	2.6351	2.5048	2.6398	2.5250	2.6251	2.4693	**2.64**
**F3**	-120.0200	-122.9510	-125.2790	-124.0450	-127.1820	-123.9840	-126.7530	-123.9230	-126.7200	-124.1020	-126.7350	-120.0800	-123.0690	-125.4230	-124.1240	-127.2910	-124.0670	-126.9120	-124.0090	-126.8770	-124.1770	-126.8760	**-114.23**

[a] methyl protons shifts are averaged

[b] according to [3]

Table S3. Cont. VI

Nuclei	R											S											Exp. [b]
	TPSS																						
	Gas	$CHCl_3$ (PCM)	$CHCl_3$ (SMD)	DMSO (PCM)	DMSO (SMD)	CH_3OH (PCM)	CH_3OH (SMD)	C_2H_5OH (PCM)	C_2H_5OH (SMD)	H_2O (PCM)	H_2O (SMD)	Gas	$CHCl_3$ (PCM)	$CHCl_3$ (SMD)	DMSO (PCM)	DMSO (SMD)	CH_3OH (PCM)	CH_3OH (SMD)	C_2H_5OH (PCM)	C_2H_5OH (SMD)	H_2O (PCM)	H_2O (SMD)	
**C1**	138.8059	139.0898	139.5919	139.2633	140.3810	139.2664	139.9486	139.2801	139.8093	139.2707	139.8094	137.5807	138.0367	139.0764	138.7925	140.1732	138.7671	140.0161	138.7539	139.8237	138.8281	140.0916	**134.46**
**C2**	117.0898	116.3926	116.9394	115.9959	116.4764	116.0269	116.9067	116.0692	116.5056	115.9775	116.8315	116.5199	116.0238	116.6111	115.8859	116.3977	115.9044	116.9415	115.9346	117.1755	115.8793	116.9047	**115.52**
**C3**	168.5071	168.7990	169.6005	168.9534	169.9650	168.9513	169.9108	168.9609	169.6089	168.9659	169.9557	168.6864	168.8675	169.6723	168.9756	169.9824	168.9754	169.8707	168.9866	169.8296	168.9862	169.9215	**162.83**
**C4**	120.1045	121.5549	122.3588	122.3538	122.9272	122.3127	124.0169	122.2828	122.7440	122.4037	124.2308	120.2817	121.5251	122.2233	122.0560	122.6607	122.0289	123.4415	122.0129	123.2082	122.0916	123.6365	**122.41**
**C5**	128.9155	130.7559	131.5414	131.7781	132.7862	131.7236	132.9510	131.6804	132.1792	131.8405	133.1007	129.1270	130.8671	131.6520	131.7543	132.7661	131.7072	132.7899	131.6711	132.7940	131.8090	132.9662	**131.43**
**C6**	122.9481	124.8260	126.0633	125.8027	127.5335	125.7443	127.8489	125.6976	126.1725	125.8688	128.3679	123.1349	125.3084	126.4565	126.2797	127.9647	126.2247	128.3259	126.1809	127.3450	126.3418	128.6562	**125.17**
**C7**	197.4897	201.9788	203.2574	204.5614	205.0734	204.4204	209.8003	204.2901	205.0794	204.7056	210.2600	201.9641	205.5398	207.3066	207.6437	208.6125	207.5293	212.6078	207.4257	207.9804	207.7624	213.0826	**196.61**
**C8**	65.6953	66.8552	67.3546	67.2991	68.1357	67.2804	68.5446	67.2724	67.5136	67.3268	69.2522	60.8248	62.3487	62.8990	63.3800	64.1895	63.3295	64.8370	63.2909	64.9267	63.4393	65.0940	**59.97**
**C9**	23.9076	24.2385	24.4947	24.4318	24.8941	24.4215	24.9472	24.4227	24.4925	24.4522	25.2171	20.7231	21.2301	21.3245	21.5131	21.8274	21.5005	22.0763	21.4997	23.4489	21.5363	22.4915	**15.26**
**C8`**	37.4869	38.1479	38.4201	38.5776	38.8327	38.5590	39.133	38.5518	38.6781	38.6061	39.5078	33.1142	33.3568	33.6821	33.3954	33.7048	33.3928	33.7628	33.4016	35.2615	33.4081	33.7999	**31.09**
**H2**	8.0852	7.9945	8.0901	7.9325	8.0086	7.9360	8.0214	7.9402	7.7784	7.9295	8.0315	8.1488	8.0103	8.0468	7.8888	7.9374	7.8946	7.9272	7.9010	8.0150	7.8835	7.9262	**7.66**
**H4**	7.1903	7.3893	7.4911	7.4780	7.6017	7.4732	7.6528	7.4688	7.3051	7.4830	7.6724	7.1988	7.3761	7.4792	7.4517	7.5797	7.4476	7.6142	7.4440	7.5614	7.4560	7.6328	**7.33**
**H5**	7.3552	7.5622	7.6283	7.6646	7.7514	7.6588	7.7711	7.6535	7.4905	7.6705	7.7919	7.3590	7.5515	7.6120	7.6409	7.7269	7.6357	7.7346	7.6310	7.7470	7.6462	7.7547	**7.45**
**H6**	7.6781	7.8717	7.9890	7.9830	8.1286	7.9762	8.1677	7.9700	7.8083	7.9899	8.2205	7.4937	7.7092	7.8091	7.7899	7.9591	7.7846	7.9671	7.7798	7.8947	7.7953	8.0078	**7.57**
**H8**	4.4745	4.5555	4.6040	4.6298	4.6976	4.6248	4.7397	4.6205	4.4454	4.6351	4.7627	4.6045	4.7682	4.8386	4.8432	4.9649	4.8391	4.9845	4.8356	4.9725	4.8473	5.0085	**4.92**
**H(9A-9C)**	1.3648	1.3728	1.3688	1.3606	1.3534	1.3613	1.3563	1.3626	1.1744	1.3603	1.3897	1.4408	1.4977	1.4703	1.5129	1.4866	1.5115	1.5132	1.5107	1.6725	1.5147	1.5371	**1.44**
**H(8`A-8`C)**	2.5368	2.4806	2.4340	2.4542	2.4272	2.4554	2.4076	2.4572	2.2734	2.4534	2.3836	2.9531	2.8059	2.7740	2.7196	2.6797	2.7240	2.5968	2.7290	2.8817	2.7158	2.5604	**2.64**
**F3**	-132.7610	-136.2000	-138.8700	-137.5020	-141.1170	-137.4290	-140.6060	-137.3560	-138.9140	-137.5710	-140.5450	-132.5580	-136.2530	-138.9790	-137.5790	-141.2260	-137.5090	-140.8040	-137.4380	-135.8180	-137.6450	-140.7380	**-114.23**
Nuclei	VSXC																						
**C1**	136.3307	136.2864	136.9028	136.1548	137.2539	136.1671	136.6742	136.1775	136.6870	136.1449	136.8382	136.2863	136.7108	137.6402	137.0135	138.2163	136.9798	137.8969	136.9488	137.8849	137.0523	138.0756	**134.46**
**C2**	114.3022	113.7629	114.1502	113.3732	113.7252	113.4016	114.1239	113.4278	114.0515	113.3479	114.1933	114.7622	114.1703	114.5041	113.8039	114.1621	113.8290	114.5435	113.8523	114.4710	113.7817	114.5265	**115.52**
**C3**	166.3069	166.7057	167.4257	166.8970	167.9094	166.8884	167.8189	166.8778	167.8042	166.9067	167.9013	165.6138	166.0033	166.7046	166.1989	167.1866	166.1900	167.0910	166.1789	167.0764	166.2090	167.1850	**162.83**
**C4**	118.1148	119.8138	120.4539	120.7250	121.3055	120.6716	122.3128	120.6162	122.0735	120.7772	122.5570	118.2844	119.8217	120.4022	120.5762	121.1664	120.5368	121.9950	120.4944	121.7780	120.6143	122.2183	**122.41**
**C5**	127.4791	129.5095	130.2304	130.5985	131.6175	130.5348	131.7214	130.4690	131.6228	130.6604	131.9236	127.4262	129.2999	129.9028	130.2602	131.2189	130.2088	131.2382	130.1541	131.1464	130.3096	131.4329	**131.43**
**C6**	123.0292	124.9919	126.0663	126.0647	127.6576	126.0015	128.1142	125.9362	127.8535	126.1261	128.4608	123.8719	126.0352	127.2339	127.1388	128.7805	127.0754	129.1759	127.0095	128.9842	127.2004	129.4534	**125.17**
**C7**	192.0244	196.0207	197.2558	198.1348	198.8965	198.0066	202.6570	197.8766	201.8931	198.2577	203.3608	192.8407	196.8027	198.2324	198.9390	199.8282	198.7978	203.5454	198.6585	202.6718	199.0789	204.3314	**196.61**
**C8**	71.7409	72.5868	73.1222	72.8179	73.6123	72.8129	73.8930	72.8049	73.8144	72.8231	74.5203	63.9006	64.9936	65.7007	65.6462	66.5943	65.6012	66.9548	65.5561	66.8125	65.6928	67.4158	**59.97**
**C9**	27.8470	28.7573	29.0954	29.5548	30.0731	29.4996	30.3676	29.4437	30.3289	29.6103	30.4871	28.7860	28.9261	29.0849	29.0553	29.2750	29.0454	29.2406	29.0344	29.2395	29.0675	29.4468	**15.26**
**C8`**	40.4195	40.7975	40.7781	40.9513	41.0897	40.9454	41.1784	40.9374	41.0912	40.9586	41.6289	35.4155	36.3399	36.5974	36.8641	37.2017	36.8313	37.4160	36.7971	37.3209	36.8972	37.8521	**31.09**
**H2**	8.2134	8.1231	8.1776	8.0764	8.1275	8.0791	8.1390	8.0819	8.1563	8.0737	8.1295	8.2284	8.1088	8.1211	8.0305	8.0750	8.0372	8.0690	8.0436	8.0792	8.0236	8.0591	**7.66**
**H4**	7.2051	7.3960	7.4902	7.4835	7.6012	7.4784	7.6457	7.4734	7.6338	7.4883	7.6673	7.2200	7.4073	7.5008	7.4880	7.6091	7.4834	7.6473	7.4789	7.6319	7.4923	7.6718	**7.33**
**H5**	7.4674	7.6754	7.7330	7.7775	7.8614	7.7716	7.8756	7.7656	7.8670	7.7832	7.9004	7.4446	7.6396	7.6945	7.7326	7.8143	7.7272	7.8241	7.7220	7.8131	7.7376	7.8462	**7.45**
**H6**	7.8523	8.0466	8.1462	8.1625	8.3141	8.1553	8.3579	8.1481	8.3448	8.1693	8.3919	7.5400	7.7386	7.8420	7.8464	8.0069	7.8401	8.0421	7.8339	8.0215	7.8523	8.0752	**7.57**
**H8**	4.3974	4.4948	4.5209	4.5791	4.6442	4.5733	4.6934	4.5676	4.6749	4.5847	4.7266	4.6276	4.7885	4.8608	4.8788	4.9951	4.8730	5.0355	4.8675	5.0171	4.8844	5.0639	**4.92**
**H(9A-9C)**	1.6028	1.6275	1.6151	1.6319	1.6077	1.6320	1.6341	1.6320	1.6330	1.6318	1.6445	1.7178	1.7227	1.7091	1.7330	1.7185	1.7323	1.7387	1.7317	1.7317	1.7338	1.7524	**1.44**
**H(8`A-8`C)**	2.7216	2.6729	2.6333	2.6461	2.6098	2.6478	2.5916	2.6497	2.5862	2.6444	2.5709	2.8282	2.7185	2.6641	2.6717	2.6195	2.6751	2.5848	2.6786	2.5962	2.6683	2.5437	**2.64**
**F3**	-133.2810	-136.7820	-139.4990	-138.0900	-141.7570	-138.0180	-141.2770	-137.9360	-141.2250	-138.1570	-141.2470	-134.0080	-137.6750	-140.4880	-139.0190	-142.7420	-138.9450	-142.2750	-138.8610	-142.2330	-139.0880	-142.2230	**-114.23**
Nuclei	wB97																						
**C1**	143.8884	143.7754	144.3011	143.7230	145.1388	143.7226	144.0079	143.7217	143.9586	143.7240	144.0913	143.0868	143.7292	144.1324	144.1072	145.3921	144.0526	145.2995	144.0106	145.4679	144.1802	145.5016	**134.46**
**C2**	123.3047	122.6459	123.1621	122.2304	122.8304	122.2552	123.1655	122.2792	123.0612	122.2075	123.1476	123.0749	122.6064	123.0585	122.3117	122.8698	122.3239	123.2791	122.3375	123.1970	122.3028	123.2931	**115.52**
**C3**	171.6610	171.6577	172.2588	171.6725	172.5210	171.6698	172.3794	171.6667	172.3812	171.6753	172.4364	171.6722	171.6725	172.2700	171.6959	172.5239	171.6917	172.3409	171.6874	172.3473	171.7010	172.4000	**162.83**
**C4**	125.5590	127.0034	127.7560	127.7463	128.3922	127.7004	129.3852	127.6537	129.1961	127.7901	129.5874	125.7034	126.8590	127.6546	127.4086	128.1276	127.3837	128.7101	127.3540	128.4843	127.4267	128.8729	**122.41**
**C5**	135.1939	136.9748	137.6055	137.9433	138.8839	137.8828	138.8851	137.8215	138.7827	138.0011	139.0641	135.2939	137.0103	137.6532	137.8973	138.8267	137.8450	138.7455	137.7911	138.6664	137.9460	138.9150	**131.43**
**C6**	128.7249	130.9092	132.1475	132.0136	133.7323	131.9461	134.2186	131.8779	134.0409	132.0781	134.6183	129.0338	131.2512	132.5567	132.3578	134.1699	132.2979	134.4117	132.2337	134.1653	132.4093	134.7131	**125.17**
**C7**	201.0619	206.0886	207.3362	208.7941	209.4820	208.6239	214.3985	208.4522	213.1530	208.9565	215.2764	204.5747	209.1359	210.5409	211.5926	212.3843	211.4335	217.5009	211.2786	216.4473	211.7522	218.2229	**196.61**
**C8**	61.5755	62.6237	63.1206	63.0390	63.8459	63.0179	64.1400	62.9952	63.9605	63.0589	64.7431	56.1541	57.5883	57.9147	58.3983	59.0916	58.3316	59.8257	58.2709	59.6675	58.4723	60.1584	**59.97**
**C9**	21.9065	22.1377	22.2844	22.2892	22.6479	22.2781	22.5363	22.2667	22.5441	22.3001	22.6948	19.4140	19.5878	19.6011	19.7258	19.9639	19.7112	19.9262	19.6974	19.9035	19.7417	20.2516	**15.26**
**C8`**	35.1209	35.7278	35.9043	36.0351	36.3575	36.0164	36.4784	35.9967	36.4261	36.0531	36.8959	30.5100	30.7786	31.1230	31.0115	31.3129	31.0012	31.3491	30.9876	31.2871	31.0171	31.5156	**31.09**
**H2**	8.4230	8.3351	8.4136	8.2701	8.3200	8.2745	8.3709	8.2788	8.3910	8.2659	8.3667	8.4514	8.2793	8.3597	8.1835	8.2441	8.1917	8.1994	8.1987	8.1955	8.1738	8.1974	**7.66**
**H4**	7.5006	7.6971	7.7936	7.7781	7.9051	7.7734	7.9507	7.7686	7.9350	7.7825	7.9739	7.4992	7.6802	7.7808	7.7528	7.8832	7.7489	7.9088	7.7448	7.8934	7.7563	7.9306	**7.33**
**H5**	7.6385	7.8475	7.9026	7.9438	8.0250	7.9382	8.0406	7.9324	8.0319	7.9492	8.0656	7.6372	7.8393	7.8930	7.9311	8.0127	7.9258	8.0181	7.9204	8.0072	7.9360	8.0412	**7.45**
**H6**	7.9237	8.1419	8.2495	8.2585	8.4057	8.2514	8.4696	8.2441	8.4635	8.2654	8.5119	7.7826	7.9791	8.1071	8.0800	8.2600	8.0753	8.2560	8.0698	8.2345	8.0835	8.2907	**7.57**
**H8**	4.0931	4.2075	4.2558	4.3046	4.3831	4.2977	4.4531	4.2907	4.4473	4.3114	4.4814	4.2823	4.4487	4.5285	4.5266	4.6574	4.5228	4.6820	4.5185	4.6664	4.5298	4.7045	**4.92**
**H(9A-9C)**	1.3404	1.3512	1.3385	1.3415	1.3122	1.3423	1.3335	1.3431	1.3361	1.3407	1.3531	1.3998	1.4195	1.4072	1.4335	1.4028	1.4334	1.4186	1.4329	1.4086	1.4333	1.4354	**1.44**
**H(8`A-8`C)**	2.4713	2.4291	2.3810	2.4046	2.3761	2.4057	2.3500	2.4067	2.3429	2.4037	2.3365	2.8007	2.6446	2.6318	2.5750	2.5446	2.5802	2.4603	2.5849	2.4743	2.5692	2.4288	**2.64**
**F3**	-113.6110	-117.0360	-119.2440	-118.3680	-121.2950	-118.2940	-120.9950	-118.2200	-120.9270	-118.4370	-121.0480	-113.6760	-117.1380	-119.3520	-118.4050	-121.3530	-118.3390	-121.1410	-118.2700	-121.0730	-118.4660	-121.1710	**-114.23**
Nuclei	wB97X																						
**C1**	145.0757	145.0547	145.5379	145.1077	146.5506	145.1027	145.4800	145.0974	145.2743	145.1125	145.4922	144.1861	144.9230	145.3717	145.3228	146.5831	145.2815	146.5366	145.2475	146.4295	145.3759	146.7158	**134.46**
**C2**	123.8370	123.1807	123.6696	122.7709	123.3544	122.7986	123.7407	122.8252	123.5677	122.7453	123.7022	123.5475	123.1357	123.5890	122.8537	123.4031	122.8700	123.8837	122.8867	123.7285	122.8412	123.8803	**115.52**
**C3**	172.4812	172.5210	173.1395	172.5546	173.4143	172.5537	173.3261	172.5520	173.2668	172.5567	173.3711	172.5182	172.5553	173.1668	172.5918	173.4298	172.5902	173.3425	172.5882	173.2834	172.5949	173.3887	**162.83**
**C4**	126.1181	127.5819	128.3415	128.3423	128.9544	128.2982	130.0033	128.2531	129.7630	128.3854	130.2091	126.2716	127.4409	128.2077	128.0042	128.7071	127.9775	129.3380	127.9475	129.1133	128.0270	129.4948	**122.41**
**C5**	135.8003	137.6105	138.2473	138.6080	139.5563	138.5481	139.6226	138.4872	139.4415	138.6660	139.7791	135.9075	137.6384	138.2976	138.5373	139.4744	138.4860	139.4392	138.4332	139.3088	138.5860	139.5983	**131.43**
**C6**	129.3481	131.4874	132.7164	132.5436	134.2351	132.4808	134.7542	132.4172	134.5627	132.6044	135.1839	129.6638	131.8726	133.1460	132.9668	134.7767	132.9080	135.0313	132.8458	134.7911	133.0196	135.3318	**125.17**
**C7**	202.7304	207.8177	209.0366	210.6501	211.3249	210.4728	216.3919	210.2950	214.9594	210.8197	217.1628	206.5991	211.0861	212.5382	213.5311	214.2935	213.3777	219.4465	213.2261	218.2232	213.6827	220.1295	**196.61**
**C8**	62.6761	63.7691	64.2047	64.1664	64.9656	64.1471	65.3262	64.1273	65.0736	64.1859	65.9302	57.1458	58.6871	58.9842	59.5211	60.2069	59.4622	60.9946	59.4068	60.6771	59.5843	61.3297	**59.97**
**C9**	22.1384	22.3762	22.5287	22.5236	22.8690	22.5163	22.8035	22.5078	22.7734	22.5318	22.9819	19.5078	19.6722	19.6759	19.8113	20.0432	19.8020	20.0370	19.7926	19.9392	19.8221	20.3619	**15.26**
**C8`**	35.3925	36.0358	36.1998	36.3571	36.6737	36.3381	36.8447	36.3191	36.7404	36.3767	37.2550	30.7290	30.9668	31.3358	31.2099	31.5193	31.1989	31.5582	31.1857	31.4807	31.2191	31.6731	**31.09**
**H2**	8.3948	8.3031	8.3888	8.2310	8.2787	8.2363	8.3308	8.2414	8.3566	8.2262	8.3323	8.4221	8.2480	8.3270	8.1543	8.2173	8.1617	8.1811	8.1683	8.1914	8.1465	8.1756	**7.66**
**H4**	7.4701	7.6675	7.7654	7.7523	7.8765	7.7477	7.9241	7.7429	7.9055	7.7569	7.9460	7.4739	7.6561	7.7551	7.7310	7.8593	7.7271	7.8910	7.7231	7.8749	7.7348	7.9115	**7.33**
**H5**	7.6120	7.8212	7.8772	7.9189	7.9989	7.9133	8.0174	7.9078	8.0058	7.9243	8.0403	7.6138	7.8130	7.8679	7.9054	7.9865	7.9003	7.9931	7.8951	7.9810	7.9104	8.0146	**7.45**
**H6**	7.9050	8.1194	8.2316	8.2387	8.3831	8.2312	8.4449	8.2237	8.4390	8.2460	8.4898	7.7597	7.9506	8.0758	8.0534	8.2329	8.0483	8.2323	8.0428	8.2195	8.0577	8.2671	**7.57**
**H8**	4.1493	4.2579	4.3151	4.3622	4.4378	4.3550	4.4993	4.3480	4.4881	4.3692	4.5274	4.3358	4.4931	4.5733	4.5704	4.7003	4.5664	4.7239	4.5622	4.7136	4.5739	4.7475	**4.92**
**H(9A-9C)**	1.3277	1.3372	1.3245	1.3221	1.2911	1.3238	1.3137	1.3253	1.3194	1.3206	1.3356	1.3927	1.4126	1.3942	1.4268	1.3969	1.4265	1.4126	1.4261	1.4068	1.4267	1.4304	**1.44**
**H(8`A-8`C)**	2.4780	2.4346	2.3835	2.4123	2.3823	2.4138	2.3583	2.4151	2.3485	2.4110	2.3414	2.8390	2.6697	2.6555	2.6009	2.5689	2.6059	2.4854	2.6104	2.5042	2.5958	2.4525	**2.64**
**F3**	-115.8910	-119.1010	-121.3360	-120.3230	-123.2980	-120.2550	-122.9770	-120.1860	-122.9300	-120.3860	-123.0060	-115.9500	-119.2000	-121.4440	-120.3750	-123.3770	-120.3130	-123.0450	-120.2480	-122.9930	-120.4340	-123.0530	**-114.23**

[a] methyl protons shifts are averaged

[b] according to [3]

Table S3. Cont. VII

Nuclei	R											S											Exp. [b]
	X3LYP																						
	Gas	$CHCl_3$ (PCM)	$CHCl_3$ (SMD)	DMSO (PCM)	DMSO (SMD)	CH_3OH (PCM)	CH_3OH (SMD)	C_2H_5OH (PCM)	C_2H_5OH (SMD)	H_2O (PCM)	H_2O (SMD)	Gas	$CHCl_3$ (PCM)	$CHCl_3$ (SMD)	DMSO (PCM)	DMSO (SMD)	CH_3OH (PCM)	CH_3OH (SMD)	C_2H_5OH (PCM)	C_2H_5OH (SMD)	H_2O (PCM)	H_2O (SMD)	
**C1**	145.8940	146.4306	146.4662	146.3956	147.9242	146.3998	146.9732	146.4011	147.0962	146.3953	146.9582	144.5867	145.5136	146.0930	145.9784	147.3288	145.9542	147.1933	145.9276	147.1459	146.0063	147.3097	**134.46**
**C2**	123.0375	122.3621	122.8136	121.9461	122.4226	121.9755	122.8617	122.0017	122.7982	121.9218	122.8035	122.5169	122.1240	122.5692	121.8724	122.3719	121.8915	122.9235	121.9077	122.8194	121.8583	122.9076	**115.52**
**C3**	173.9683	174.2327	174.8979	174.3326	175.2832	174.3305	175.1671	174.3254	175.1715	174.3384	175.2647	174.1357	174.3255	175.0102	174.4116	175.3309	174.4100	175.1574	174.4052	175.1689	174.4169	175.2023	**162.83**
**C4**	125.6972	127.2737	127.9767	128.0684	128.5422	128.0237	129.7045	127.9759	129.4570	128.1148	129.8775	126.0040	127.1940	127.8742	127.7610	128.3205	127.7315	129.0689	127.6986	128.8942	127.7919	129.2504	**122.41**
**C5**	135.1518	137.1236	137.7353	138.1279	139.0663	138.0707	139.1702	138.0103	139.0724	138.1856	139.3354	135.3487	137.1586	137.8258	138.0790	139.0006	138.0277	139.0063	137.9730	138.9364	138.1308	139.1774	**131.43**
**C6**	129.2079	131.1671	132.6227	132.3480	133.9589	132.2791	134.4980	132.2072	134.2474	132.4173	134.9089	129.4988	131.7189	132.9890	132.8206	134.5914	132.7581	134.9427	132.6921	134.7648	132.8828	135.2825	**125.17**
**C7**	203.6700	209.2273	210.0454	211.8819	212.6837	211.7197	217.7740	211.5557	216.6687	212.0391	218.5290	208.7411	213.0443	214.5616	215.3172	216.1501	215.1847	220.8595	215.0487	219.8258	215.4462	221.4208	**196.61**
**C8**	65.5438	66.7396	67.1559	67.1713	67.9742	67.1550	68.3138	67.1343	68.1395	67.1889	68.9925	59.9947	61.8769	62.2719	62.8214	63.6188	62.7678	64.3355	62.7118	64.0902	62.8764	64.6639	**59.97**
**C9**	22.9705	23.1692	23.5115	23.4457	23.7659	23.4323	23.7895	23.4156	23.7428	23.4619	23.9482	19.6027	19.9654	20.0344	20.1635	20.4457	20.1535	20.5622	20.1410	20.4948	20.1769	20.9242	**15.26**
**C8`**	36.3709	37.1931	37.3296	37.5740	37.8820	37.5541	38.1127	37.5309	38.0680	37.5966	38.5358	31.8426	32.0663	32.4101	32.2640	32.5525	32.2547	32.6038	32.2421	32.6340	32.2762	32.6621	**31.09**
**H2**	8.2605	8.1327	8.2705	8.0837	8.1222	8.0871	8.1762	8.0901	8.1785	8.0804	8.1857	8.3168	8.1335	8.2105	8.0407	8.0908	8.0464	8.0650	8.0516	8.0779	8.0351	8.0632	**7.66**
**H4**	7.3475	7.5472	7.6408	7.6329	7.7500	7.6282	7.8007	7.6230	7.7843	7.6372	7.8227	7.3620	7.5420	7.6399	7.6168	7.7370	7.6128	7.7633	7.6083	7.7502	7.6204	7.7841	**7.33**
**H5**	7.4902	7.6979	7.7560	7.7979	7.8752	7.7923	7.8936	7.7861	7.8826	7.8032	7.9174	7.4879	7.6791	7.7359	7.7705	7.8472	7.7655	7.8566	7.7598	7.8476	7.7753	7.8786	**7.45**
**H6**	7.8144	8.0087	8.1481	8.1283	8.2599	8.1210	8.3245	8.1133	8.3079	8.1353	8.3735	7.6367	7.8260	7.9445	7.9233	8.0913	7.9177	8.0987	7.9116	8.0889	7.9285	8.1386	**7.57**
**H8**	4.3520	4.4472	4.5076	4.5355	4.6068	4.5290	4.6609	4.5223	4.6516	4.5418	4.6880	4.5015	4.6738	4.7413	4.7512	4.8710	4.7469	4.8918	4.7421	4.8807	4.7552	4.9187	**4.92**
**H(9A-9C)**	1.3272	1.3126	1.3324	1.3104	1.2771	1.3112	1.2987	1.3113	1.2940	1.3097	1.3190	1.4001	1.4334	1.4207	1.4581	1.4303	1.4567	1.4530	1.4548	1.4478	1.4594	1.4750	**1.44**
**H(8`A-8`C)**	2.5285	2.4765	2.4277	2.4602	2.4300	2.4614	2.4121	2.4620	2.4130	2.4591	2.3905	2.9671	2.7987	2.7731	2.7234	2.6794	2.7278	2.5949	2.7317	2.6124	2.7193	2.5600	**2.64**
**F3**	-130.3030	-133.7990	-136.1940	-135.0750	-138.5060	-135.0030	-138.0860	-134.9300	-138.0360	-135.1430	-137.9040	-130.3350	-133.7010	-136.2980	-134.9270	-138.3970	-134.8610	-138.2210	-134.7930	-138.1630	-134.9900	-138.1850	**-114.23**

[a] methyl protons shifts are averaged

[b] according to [3]

Table S4. Theoretical chemical shifts (in ppm) of *(R,S)*-4-fluoro-α-pyrrolidinopentiophenone calculated with HF and selected functionals combined with 6-311++G** basis set[a]

Nuclei	R											S											Exp. [b]
	B3LYP																						
	Gas	$CHCl_3$ (PCM)	$CHCl_3$ (SMD)	DMSO (PCM)	DMSO (SMD)	CH_3OH (PCM)	CH_3OH (SMD)	C_2H_5OH (PCM)	C_2H_5OH (SMD)	H_2O (PCM)	H_2O (SMD)	Gas	$CHCl_3$ (PCM)	$CHCl_3$ (SMD)	DMSO (PCM)	DMSO (SMD)	CH_3OH (PCM)	CH_3OH (SMD)	C_2H_5OH (PCM)	C_2H_5OH (SMD)	H_2O (PCM)	H_2O (SMD)	
**C1**	139.6595	141.0130	142.0566	141.4838	143.2056	141.4487	142.6536	141.4144	142.6775	141.5164	143.0299	139.6595	141.0131	142.0568	141.4838	143.2056	141.4487	142.6536	141.4144	142.6773	141.5164	143.0300	**133.8**
**C2**	139.5607	139.5533	140.0992	139.3810	139.9034	139.3881	140.3839	139.3950	140.2814	139.3726	140.3845	139.5607	139.5533	140.0993	139.3811	139.9034	139.3881	140.3839	139.3950	140.2813	139.3725	140.3845	**132.1**
**C3**	121.8992	122.6659	123.2367	122.8572	123.5136	122.8442	123.8248	122.8308	123.7329	122.8674	123.8998	121.8992	122.6660	123.2367	122.8571	123.5136	122.8442	123.8248	122.8308	123.7328	122.8674	123.8997	**115.8**
**C4**	176.3634	177.2400	177.9170	177.5979	178.3179	177.5726	178.9122	177.5470	178.7385	177.6202	179.0146	176.3634	177.2401	177.9169	177.5978	178.3179	177.5726	178.9122	177.5470	178.7385	177.6202	179.0144	**165.8**
**C5**	119.9425	121.2697	121.8855	121.8481	122.6291	121.8091	122.8607	121.7699	122.7343	121.8835	122.9906	119.9425	121.2697	121.8855	121.8481	122.6291	121.8091	122.8607	121.7699	122.7343	121.8835	122.9905	**115.8**
**C6**	135.8367	137.5847	138.5465	138.6062	140.0186	138.5384	140.6828	138.4705	140.3917	138.6689	140.9248	135.8368	137.5847	138.5464	138.6062	140.0186	138.5384	140.6828	138.4705	140.3918	138.6689	140.9249	**132.1**
**C7**	203.8262	208.9948	210.3970	211.6481	212.3534	211.4769	217.6027	211.3067	216.4065	211.8097	218.7711	203.8262	208.9948	210.3967	211.6481	212.3534	211.4769	217.6027	211.3067	216.4064	211.8097	218.7711	**198.9**
**C8**	66.4966	67.7258	68.4480	68.3496	69.0459	68.3005	69.8267	68.2525	69.6471	68.3953	70.1540	66.4967	67.7257	68.4479	68.3496	69.0459	68.3005	69.8267	68.2525	69.6470	68.3953	70.1539	**69.3**
**C9**	38.7755	38.3733	38.4550	38.1579	38.3135	38.1667	38.5364	38.1754	38.4611	38.1474	38.5718	38.7755	38.3732	38.4549	38.1578	38.3135	38.1667	38.5364	38.1754	38.4610	38.1474	38.5718	**31.5**
**C10**	19.9937	20.4460	20.7085	20.6771	21.1223	20.6584	21.0452	20.6393	21.0064	20.6931	21.1559	19.9937	20.4461	20.7085	20.6771	21.1223	20.6584	21.0452	20.6393	21.0065	20.6931	21.1559	**19.9**
**C11**	16.0946	16.2853	16.4075	16.3075	16.5083	16.3018	16.4979	16.2960	16.4791	16.3112	16.6477	16.0946	16.2853	16.4075	16.3075	16.5083	16.3018	16.4979	16.2960	16.4791	16.3112	16.6476	**14.3**
**C1`**	52.3223	53.1970	53.5209	53.6907	54.1936	53.6541	54.2321	53.6172	54.1877	53.7237	54.6654	56.8876	57.9796	58.3853	58.4297	58.9956	58.3966	59.1331	58.3636	59.0349	58.4600	59.3734	**50.6**
**C2`**	26.1592	26.6379	26.9310	26.8078	27.2269	26.7905	27.2729	26.7736	27.2316	26.8230	27.1130	25.7213	25.9896	26.3448	26.0758	26.6580	26.0646	26.5476	26.0537	26.5196	26.0853	26.3447	**23.8**
**C3`**	25.7214	25.9896	26.3449	26.0759	26.6580	26.0646	26.5476	26.0537	26.5196	26.0853	26.3449	26.1592	26.6379	26.9310	26.8079	27.2269	26.7905	27.2729	26.7736	27.2316	26.8230	27.1131	**23.8**
**C4`**	56.8875	57.9797	58.3854	58.4296	58.9956	58.3966	59.1331	58.3636	59.0349	58.4601	59.3733	52.3224	53.1971	53.5209	53.6908	54.1936	53.6541	54.2321	53.6172	54.1877	53.7237	54.6654	**50.6**
**H2**	8.6658	8.5221	8.5209	8.4446	8.4398	8.4493	8.4470	8.4539	8.4473	8.4398	8.4321	8.6658	8.5221	8.5209	8.4446	8.4398	8.4493	8.4470	8.4539	8.4473	8.4398	8.4321	**8.35**
**H3**	7.2988	7.4402	7.5270	7.4790	7.5943	7.4769	7.6101	7.4747	7.6007	7.4807	7.6258	7.2988	7.4402	7.5271	7.4790	7.5943	7.4769	7.6101	7.4747	7.6007	7.4807	7.6258	**7.06**
**H5**	7.1478	7.3279	7.4131	7.4001	7.5227	7.3956	7.5364	7.3911	7.5218	7.4041	7.5568	7.1478	7.3279	7.4131	7.4001	7.5227	7.3956	7.5364	7.3911	7.5218	7.4041	7.5568	**7.06**
**H6**	7.9815	8.1560	8.2289	8.2444	8.3759	8.2389	8.4114	8.2333	8.3880	8.2495	8.4315	7.9815	8.1560	8.2289	8.2445	8.3759	8.2389	8.4114	8.2333	8.3880	8.2495	8.4315	**8.35**
**H8**	4.6382	4.7005	4.7167	4.7308	4.8264	4.7285	4.8453	4.7263	4.8230	4.7330	4.8374	4.6382	4.7005	4.7167	4.7308	4.8264	4.7285	4.8453	4.7263	4.8230	4.7330	4.8374	**4.88**
**H(9A-9B)**	1.6769	1.6946	1.6718	1.7219	1.7054	1.7196	1.7129	1.7173	1.7075	1.7237	1.7365	1.6769	1.6946	1.6718	1.7219	1.7054	1.7196	1.7129	1.7173	1.7075	1.7237	1.7365	**2.05**
**H(10A-10B)**	1.4170	1.3092	1.2585	1.2423	1.1832	1.2464	1.1948	1.2505	1.1943	1.2381	1.1836	1.4170	1.3092	1.2585	1.2423	1.1832	1.2464	1.1949	1.2505	1.1943	1.2381	1.1835	**1.36**
**H(11A-11C)**	0.7806	0.7859	0.7568	0.7886	0.7603	0.7881	0.7593	0.7875	0.7566	0.7889	0.7528	0.7806	0.7859	0.7568	0.7886	0.7603	0.7881	0.7593	0.7875	0.7566	0.7889	0.7528	**0.77**
**H(1`A)**	2.7396	2.7448	2.7135	2.7304	2.6812	2.7311	2.6921	2.7318	2.6904	2.7295	2.6710	2.8685	2.8717	2.8592	2.8509	2.8194	2.8517	2.8290	2.8526	2.8282	2.8499	2.7949	**3.03**
**H(1`B)**	3.1966	3.2378	3.2732	3.2692	3.3388	3.2666	3.3209	3.2641	3.3137	3.2715	3.3213	2.8682	2.7242	2.7036	2.6902	2.6871	2.6914	2.6646	2.6927	2.6635	2.6889	2.6644	**3.23**
**H(2`-3`A-2`-3`B)**	1.6690	1.7354	1.7006	1.7713	1.7310	1.7687	1.7485	1.7660	1.7408	1.7737	1.7768	1.6690	1.7354	1.7006	1.7713	1.7310	1.7687	1.7485	1.7660	1.7408	1.7738	1.7768	**1.77**
**H(4`A)**	2.8682	2.7242	2.7036	2.6902	2.6871	2.6914	2.6646	2.6927	2.6635	2.6889	2.6644	3.1966	3.2378	3.2732	3.2692	3.3388	3.2666	3.3209	3.2641	3.3137	3.2715	3.3213	**3.03**
**H(4`B)**	2.8685	2.8718	2.8592	2.8509	2.8194	2.8517	2.8290	2.8526	2.8282	2.8499	2.7949	2.7396	2.7448	2.7135	2.7304	2.6812	2.7311	2.6921	2.7318	2.6904	2.7295	2.6710	**3.23**
Nuclei	B3P86																						
**C1**	137.0834	137.9700	138.7039	138.2940	140.4959	138.2856	139.9806	138.2578	139.9799	138.3478	140.2201	137.0834	137.9700	138.7039	138.2939	140.4957	138.2855	139.9806	138.2579	139.9799	138.3479	140.2200	**133.8**
**C2**	138.6697	138.6011	139.2492	138.4593	139.0695	138.4902	139.4613	138.4992	139.5061	138.4735	139.4640	138.6697	138.6011	139.2492	138.4593	139.0695	138.4902	139.4613	138.4995	139.5060	138.4735	139.4640	**132.1**
**C3**	121.0740	121.8419	122.4817	122.0957	122.8695	122.1028	123.1861	122.0878	123.2309	122.1325	123.2858	121.0740	121.8419	122.4817	122.0956	122.8695	122.1029	123.1861	122.0880	123.2309	122.1324	123.2858	**115.8**
**C4**	173.8519	174.6554	175.3491	175.0610	175.7802	175.0567	176.4157	175.0301	176.4015	175.1095	176.5441	173.8519	174.6554	175.3491	175.0611	175.7802	175.0566	176.4157	175.0301	176.4015	175.1096	176.5440	**165.8**
**C5**	119.1494	120.5275	121.2124	121.2172	122.1039	121.1955	122.3368	121.1515	122.3319	121.2823	122.4971	119.1494	120.5275	121.2124	121.2175	122.1039	121.1955	122.3368	121.1515	122.3319	121.2824	122.4971	**115.8**
**C6**	134.8903	136.7414	137.9127	137.8080	139.0992	137.7648	139.7068	137.6992	139.5516	137.8923	140.0277	134.8903	136.7414	137.9127	137.8081	139.0992	137.7650	139.7068	137.6993	139.5517	137.8924	140.0277	**132.1**
**C7**	201.9935	206.5379	207.6449	208.9027	210.1328	208.7685	215.1507	208.6149	213.8462	209.0759	216.1905	201.9935	206.5379	207.6449	208.9027	210.1328	208.7685	215.1507	208.6149	213.8463	209.0758	216.1905	**198.9**
**C8**	64.8034	65.9736	66.6913	66.5410	67.5554	66.5226	68.2240	66.4836	67.9231	66.6033	68.6619	64.8034	65.9736	66.6913	66.5410	67.5554	66.5226	68.2240	66.4836	67.9231	66.6033	68.6619	**69.3**
**C9**	37.6384	37.4660	37.7532	37.3213	37.5375	37.3547	37.6239	37.3657	37.5293	37.3326	37.7648	37.6384	37.4660	37.7532	37.3212	37.5375	37.3548	37.6239	37.3658	37.5293	37.3326	37.7648	**31.5**
**C10**	18.7463	19.2110	19.5410	19.4685	19.9226	19.4732	19.8038	19.4560	19.8274	19.5080	20.0120	18.7463	19.2110	19.5410	19.4685	19.9226	19.4733	19.8038	19.4559	19.8274	19.5080	20.0119	**19.9**
**C11**	15.6799	15.8487	16.0027	15.8858	16.1260	15.9043	16.0704	15.9010	16.1497	15.9122	16.2415	15.6799	15.8487	16.0027	15.8858	16.1261	15.9044	16.0704	15.9010	16.1496	15.9122	16.2414	**14.3**
**C1`**	51.2447	52.0577	52.3607	52.5446	53.1062	52.5338	53.0540	52.5013	53.0966	52.5988	53.5834	55.5720	56.5444	56.9649	57.0256	57.5965	57.0137	57.7967	56.9807	57.8796	57.0811	58.0295	**50.6**
**C2`**	24.9427	25.3436	25.6830	25.5269	25.9615	25.5347	25.9987	25.5209	26.0503	25.5637	25.8565	24.3771	24.5999	24.9475	24.7179	25.2484	24.7307	25.1836	24.7216	25.2911	24.7497	25.0495	**23.8**
**C3`**	24.3771	24.5999	24.9475	24.7179	25.2484	24.7307	25.1836	24.7217	25.2911	24.7496	25.0495	24.9427	25.3436	25.6830	25.5270	25.9615	25.5347	25.9987	25.5209	26.0503	25.5638	25.8565	**23.8**
**C4`**	55.5720	56.5444	56.9649	57.0254	57.5964	57.0137	57.7967	56.9808	57.8797	57.0812	58.0295	51.2447	52.0577	52.3607	52.5446	53.1061	52.5338	53.0540	52.5013	53.0967	52.5987	53.5835	**50.6**
**H2**	8.7356	8.6315	8.6671	8.5696	8.5174	8.5756	8.5132	8.5795	8.5326	8.5649	8.5091	8.7356	8.6315	8.6671	8.5696	8.5174	8.5756	8.5132	8.5795	8.5326	8.5650	8.5091	**8.35**
**H3**	7.3191	7.4609	7.5518	7.5063	7.6326	7.5051	7.6514	7.5020	7.6478	7.5087	7.6717	7.3191	7.4609	7.5518	7.5063	7.6326	7.5051	7.6514	7.5020	7.6478	7.5086	7.6717	**7.06**
**H5**	7.1598	7.3540	7.4469	7.4346	7.5590	7.4312	7.5697	7.4260	7.5622	7.4390	7.5920	7.1598	7.3540	7.4469	7.4346	7.5590	7.4312	7.5697	7.4260	7.5622	7.4390	7.5920	**7.06**
**H6**	8.0640	8.2389	8.3289	8.3344	8.4363	8.3300	8.4815	8.3236	8.4785	8.3398	8.5089	8.0640	8.2389	8.3289	8.3344	8.4363	8.3300	8.4815	8.3236	8.4785	8.3398	8.5089	**8.35**
**H8**	4.6400	4.7026	4.7244	4.7366	4.8022	4.7356	4.8453	4.7328	4.8505	4.7388	4.8420	4.6400	4.7026	4.7244	4.7366	4.8022	4.7356	4.8453	4.7328	4.8505	4.7388	4.8420	**4.88**
**H(9A-9B)**	1.7416	1.7883	1.7937	1.8184	1.7905	1.8182	1.7669	1.8161	1.7538	1.8196	1.8042	1.7416	1.7883	1.7937	1.8183	1.7905	1.8182	1.7669	1.8161	1.7538	1.8196	1.8042	**2.05**
**H(10A-10B)**	1.4033	1.2887	1.2417	1.2217	1.1482	1.2273	1.1635	1.2309	1.1803	1.2176	1.1520	1.4033	1.2887	1.2417	1.2217	1.1482	1.2273	1.1635	1.2310	1.1803	1.2176	1.1520	**1.36**
**H(11A-11C)**	0.7436	0.7503	0.7264	0.7554	0.7181	0.7563	0.7178	0.7554	0.7285	0.7558	0.7112	0.7436	0.7503	0.7264	0.7554	0.7181	0.7563	0.7178	0.7554	0.7285	0.7558	0.7112	**0.77**
**H(1`A)**	2.6954	2.7070	2.6878	2.6933	2.6530	2.6955	2.6625	2.6959	2.6647	2.6923	2.6372	2.8227	2.8243	2.8115	2.8064	2.7807	2.8087	2.7945	2.8092	2.7940	2.8054	2.7646	**3.03**
**H(1`B)**	3.1996	3.2388	3.2707	3.2664	3.3284	3.2657	3.3054	3.2632	3.3068	3.2683	3.3052	2.9093	2.7544	2.7264	2.6975	2.7241	2.7016	2.6736	2.7040	2.6611	2.6950	2.6628	**3.23**
**H(2`-3`A-2`-3`B)**	1.6557	1.7151	1.6795	1.7495	1.7149	1.7483	1.7350	1.7453	1.7365	1.7520	1.7641	1.6557	1.7151	1.6795	1.7495	1.7149	1.7483	1.7350	1.7453	1.7365	1.7520	1.7640	**1.77**
**H(4`A)**	2.9093	2.7544	2.7264	2.6975	2.7241	2.7016	2.6736	2.7040	2.6611	2.6950	2.6629	3.1996	3.2388	3.2707	3.2664	3.3283	3.2656	3.3054	3.2632	3.3068	3.2683	3.3052	**3.03**
**H(4`B)**	2.8227	2.8243	2.8115	2.8064	2.7807	2.8087	2.7945	2.8092	2.7941	2.8054	2.7646	2.6954	2.7070	2.6878	2.6933	2.6530	2.6955	2.6625	2.6959	2.6647	2.6923	2.6372	**3.23**

[a] methyl protons shifts are averaged

[b] according to [4]

Table S4. Cont. I

Nuclei	R											S											Exp. [b]
	B3PW91																						
	Gas	$CHCl_3$ (PCM)	$CHCl_3$ (SMD)	DMSO (PCM)	DMSO (SMD)	CH_3OH (PCM)	CH_3OH (SMD)	C_2H_5OH (PCM)	C_2H_5OH (SMD)	H_2O (PCM)	H_2O (SMD)	Gas	$CHCl_3$ (PCM)	$CHCl_3$ (SMD)	DMSO (PCM)	DMSO (SMD)	CH_3OH (PCM)	CH_3OH (SMD)	C_2H_5OH (PCM)	C_2H_5OH (SMD)	H_2O (PCM)	H_2O (SMD)	
**C1**	137.3240	138.3570	138.9423	139.0780	141.3135	139.0503	140.5015	139.0142	140.3718	139.1064	140.8683	137.3240	138.3570	138.9423	139.0780	140.9945	139.0503	140.5015	139.0142	140.3718	139.1064	140.8684	**133.8**
**C2**	138.7406	138.7359	139.3047	138.5884	139.5155	138.6032	139.5708	138.6087	139.5248	138.5765	139.5857	138.7406	138.7359	139.3048	138.5884	139.1965	138.6032	139.5708	138.6087	139.5248	138.5765	139.5857	**132.1**
**C3**	121.1602	121.9507	122.5047	122.2008	123.2602	122.1943	123.2452	122.1786	123.1838	122.2088	123.3490	121.1602	121.9508	122.5047	122.2008	122.9412	122.1943	123.2452	122.1786	123.1838	122.2088	123.3490	**115.8**
**C4**	173.8875	174.7119	175.3233	175.0897	176.1045	175.0726	176.3829	175.0466	176.2739	175.1079	176.5084	173.8875	174.7119	175.3233	175.0897	175.7855	175.0726	176.3829	175.0466	176.2739	175.1080	176.5085	**165.8**
**C5**	119.2614	120.6353	121.2374	121.2949	122.4633	121.2608	122.3700	121.2179	122.2743	121.3290	122.5313	119.2614	120.6353	121.2374	121.2949	122.1443	121.2608	122.3700	121.2179	122.2743	121.3291	122.5314	**115.8**
**C6**	134.8881	136.7480	137.8575	137.6742	139.3812	137.6161	139.6614	137.5494	139.4318	137.7317	139.9273	134.8881	136.7480	137.8575	137.6742	139.0622	137.6161	139.6615	137.5494	139.4318	137.7318	139.9274	**132.1**
**C7**	202.0068	206.7256	207.7025	209.5098	210.7621	209.3506	215.5636	209.1833	214.1346	209.6636	216.7030	202.0068	206.7255	207.7027	209.5098	210.4431	209.3506	215.5636	209.1833	214.1346	209.6636	216.7029	**198.9**
**C8**	64.7706	66.0246	66.6937	66.7619	68.0060	66.7169	68.3767	66.6643	67.9973	66.8079	68.7804	64.7706	66.0245	66.6937	66.7619	67.6870	66.7169	68.3767	66.6643	67.9973	66.8079	68.7804	**69.3**
**C9**	37.1523	37.0117	37.2475	36.7905	37.3749	36.8050	37.1661	36.8112	37.0112	36.7793	37.2445	37.1523	37.0117	37.2476	36.7905	37.0559	36.8050	37.1661	36.8112	37.0112	36.7794	37.2446	**31.5**
**C10**	18.7078	19.2020	19.4695	19.4349	20.2138	19.4228	19.7891	19.4020	19.7285	19.4488	19.9748	18.7078	19.2020	19.4696	19.4349	19.8948	19.4228	19.7891	19.4020	19.7285	19.4488	19.9748	**19.9**
**C11**	15.7083	15.8983	15.9801	15.9249	16.4664	15.9275	16.0952	15.9214	16.0810	15.9249	16.2589	15.7083	15.8984	15.9801	15.9249	16.1474	15.9275	16.0952	15.9214	16.0810	15.9250	16.2588	**14.3**
**C1`**	51.1668	51.9977	52.2339	52.5010	53.3444	52.4744	52.9853	52.4389	52.9389	52.5279	53.4848	55.5096	56.5041	56.8262	56.9997	57.5394	56.9736	57.7030	56.9391	57.6758	57.0268	57.9585	**50.6**
**C2`**	25.1909	25.6108	25.8728	25.7945	26.5286	25.7859	26.2348	25.7688	26.1945	25.8052	26.0679	24.6529	24.8754	25.1417	24.9807	25.5012	24.9798	25.4208	24.9700	25.4250	24.9834	25.2497	**23.8**
**C3`**	24.6529	24.8754	25.1416	24.9807	25.8202	24.9798	25.4208	24.9700	25.4250	24.9835	25.2498	25.1909	25.6108	25.8728	25.7945	26.2096	25.7859	26.2348	25.7688	26.1945	25.8052	26.0679	**23.8**
**C4`**	55.5096	56.5041	56.8263	56.9997	57.8584	56.9736	57.7030	56.9391	57.6758	57.0267	57.9585	51.1668	51.9977	52.2338	52.5010	53.0254	52.4744	52.9853	52.4389	52.9389	52.5279	53.4847	**50.6**
**H2**	8.7063	8.5916	8.6349	8.4920	8.4832	8.4975	8.4709	8.5026	8.4896	8.4870	8.4576	8.7063	8.5916	8.6349	8.4920	8.4677	8.4975	8.4709	8.5026	8.4896	8.4870	8.4575	**8.35**
**H3**	7.3201	7.4605	7.5499	7.5052	7.6405	7.5036	7.6437	7.5016	7.6363	7.5069	7.6603	7.3201	7.4605	7.5499	7.5052	7.6250	7.5036	7.6437	7.5016	7.6363	7.5069	7.6603	**7.06**
**H5**	7.1620	7.3498	7.4420	7.4265	7.5662	7.4225	7.5662	7.4181	7.5549	7.4305	7.5848	7.1620	7.3498	7.4420	7.4265	7.5507	7.4225	7.5662	7.4181	7.5549	7.4305	7.5848	**7.06**
**H6**	8.0473	8.2195	8.3070	8.2987	8.4277	8.2939	8.4532	8.2889	8.4474	8.3033	8.4728	8.0473	8.2195	8.3070	8.2987	8.4122	8.2939	8.4532	8.2889	8.4474	8.3033	8.4728	**8.35**
**H8**	4.6321	4.6900	4.7084	4.7159	4.8047	4.7146	4.8315	4.7131	4.8294	4.7173	4.8251	4.6321	4.6900	4.7084	4.7159	4.7892	4.7146	4.8315	4.7131	4.8294	4.7173	4.8252	**4.88**
**H(9A-9B)**	1.6981	1.7410	1.7540	1.7511	1.7538	1.7496	1.7252	1.7479	1.7130	1.7528	1.7506	1.6981	1.7410	1.7540	1.7511	1.7383	1.7496	1.7252	1.7479	1.7130	1.7528	1.7506	**2.05**
**H(10A-10B)**	1.3764	1.2627	1.2145	1.1925	1.1401	1.1975	1.1436	1.2023	1.1537	1.1878	1.1315	1.3764	1.2627	1.2145	1.1925	1.1246	1.1975	1.1436	1.2023	1.1537	1.1878	1.1314	**1.36**
**H(11A-11C)**	0.7339	0.7383	0.7137	0.7390	0.7204	0.7393	0.7072	0.7392	0.7126	0.7390	0.6984	0.7339	0.7383	0.7136	0.7390	0.7049	0.7393	0.7072	0.7392	0.7126	0.7390	0.6984	**0.77**
**H(1`A)**	2.6891	2.6965	2.6752	2.6813	2.6535	2.6829	2.6507	2.6842	2.6495	2.6800	2.6248	2.8185	2.8168	2.8044	2.8024	2.7725	2.8038	2.7885	2.8049	2.7837	2.8012	2.7537	**3.03**
**H(1`B)**	3.1751	3.2151	3.2483	3.2426	3.3187	3.2409	3.2853	3.2390	3.2822	3.2444	3.2835	2.8650	2.7239	2.6983	2.6825	2.6990	2.6853	2.6603	2.6878	2.6451	2.6802	2.6502	**3.23**
**H(2`-3`A-2`-3`B)**	1.6449	1.7030	1.6659	1.7420	1.7171	1.7401	1.7233	1.7380	1.7203	1.7439	1.7513	1.6449	1.7030	1.6659	1.7420	1.7016	1.7401	1.7233	1.7380	1.7203	1.7439	1.7513	**1.77**
**H(4`A)**	2.8650	2.7239	2.6982	2.6825	2.7145	2.6853	2.6603	2.6878	2.6451	2.6802	2.6502	3.1751	3.2151	3.2483	3.2426	3.3032	3.2409	3.2853	3.2390	3.2822	3.2444	3.2835	**3.03**
**H(4`B)**	2.8185	2.8168	2.8045	2.8024	2.7880	2.8038	2.7885	2.8049	2.7837	2.8012	2.7537	2.6891	2.6965	2.6752	2.6813	2.6380	2.6829	2.6507	2.6842	2.6495	2.6800	2.6247	**3.23**
Nuclei	B98																						
**C1**	138.2224	139.1640	139.9051	139.8882	141.7003	139.8842	141.1990	139.8762	141.1387	139.8933	141.5892	138.2225	139.1640	139.9050	139.8882	141.7003	139.8843	141.1989	139.8762	141.1387	139.8934	141.5892	**133.8**
**C2**	138.3975	138.3805	138.9375	138.2296	138.7920	138.2538	139.2215	138.2758	139.1629	138.2089	139.2303	138.3977	138.3805	138.9374	138.2296	138.7920	138.2538	139.2215	138.2758	139.1630	138.2088	139.2303	**132.1**
**C3**	120.9098	121.6403	122.1892	121.9083	122.6382	121.9116	122.9215	121.9121	122.8614	121.9068	123.0044	120.9098	121.6403	122.1891	121.9083	122.6382	121.9116	122.9215	121.9121	122.8615	121.9069	123.0044	**115.8**
**C4**	173.6926	174.4602	175.0884	174.8453	175.5834	174.8371	176.1492	174.8264	176.0280	174.8553	176.2539	173.6926	174.4602	175.0884	174.8453	175.5834	174.8371	176.1492	174.8264	176.0279	174.8553	176.2539	**165.8**
**C5**	118.9981	120.3027	120.8910	120.9799	121.8149	120.9557	122.0144	120.9289	121.9207	121.0050	122.1558	118.9980	120.3027	120.8911	120.9799	121.8149	120.9557	122.0144	120.9289	121.9207	121.0051	122.1558	**115.8**
**C6**	134.8559	136.6645	137.7541	137.6013	139.0072	137.5486	139.5986	137.4941	139.3624	137.6546	139.8326	134.8558	136.6645	137.7543	137.6013	139.0072	137.5486	139.5985	137.4941	139.3624	137.6545	139.8326	**132.1**
**C7**	200.4682	205.2187	206.3918	208.0300	208.8092	207.8906	214.1407	207.7481	212.7881	208.1635	215.3302	200.4686	205.2187	206.3918	208.0300	208.8092	207.8906	214.1407	207.7481	212.7880	208.1636	215.3302	**198.9**
**C8**	66.3871	67.6168	68.4226	68.2708	69.0696	68.2442	69.8409	68.2155	69.5502	68.2988	70.2427	66.3871	67.6168	68.4226	68.2708	69.0696	68.2442	69.8409	68.2155	69.5501	68.2989	70.2427	**69.3**
**C9**	38.4616	38.2575	38.5178	38.0095	38.2028	38.0334	38.4017	38.0552	38.2887	37.9894	38.4483	38.4616	38.2575	38.5178	38.0095	38.2028	38.0334	38.4017	38.0552	38.2886	37.9894	38.4483	**31.5**
**C10**	20.1212	20.5386	20.8234	20.7589	21.1975	20.7577	21.1155	20.7539	21.0819	20.7625	21.2510	20.1211	20.5386	20.8234	20.7589	21.1975	20.7577	21.1154	20.7539	21.0820	20.7625	21.2510	**19.9**
**C11**	16.3161	16.4561	16.5672	16.5007	16.7076	16.5122	16.6770	16.5215	16.6738	16.4920	16.8309	16.3162	16.4561	16.5671	16.5007	16.7075	16.5122	16.6770	16.5215	16.6738	16.4920	16.8309	**14.3**
**C1`**	52.4322	53.2094	53.4644	53.7262	54.1926	53.7098	54.1985	53.6905	54.1769	53.7438	54.6700	56.9107	57.8097	58.1546	58.3077	58.9077	58.2931	59.0087	58.2762	58.9647	58.3242	59.2359	**50.6**
**C2`**	26.3567	26.7481	27.0235	26.9415	27.3702	26.9430	27.3853	26.9422	27.3586	26.9426	27.2367	25.8357	26.0296	26.3025	26.1673	26.7228	26.1753	26.6111	26.1807	26.6224	26.1617	26.4450	**23.8**
**C3`**	25.8357	26.0296	26.3025	26.1673	26.7228	26.1753	26.6111	26.1807	26.6224	26.1617	26.4450	26.3566	26.7481	27.0234	26.9415	27.3702	26.9430	27.3852	26.9422	27.3587	26.9427	27.2367	**23.8**
**C4`**	56.9107	57.8097	58.1547	58.3077	58.9077	58.2931	59.0087	58.2762	58.9647	58.3241	59.2359	52.4323	53.2094	53.4643	53.7262	54.1926	53.7098	54.1985	53.6905	54.1769	53.7438	54.6700	**50.6**
**H2**	8.6894	8.5909	8.6224	8.4935	8.4821	8.4973	8.4850	8.5013	8.4994	8.4903	8.4675	8.6894	8.5909	8.6224	8.4935	8.4821	8.4973	8.4850	8.5013	8.4994	8.4902	8.4675	**8.35**
**H3**	7.3105	7.4535	7.5447	7.5015	7.6240	7.4994	7.6413	7.4973	7.6346	7.5037	7.6546	7.3105	7.4535	7.5447	7.5015	7.6240	7.4994	7.6413	7.4973	7.6346	7.5037	7.6546	**7.06**
**H5**	7.1523	7.3441	7.4354	7.4263	7.5528	7.4218	7.5657	7.4173	7.5546	7.4309	7.5834	7.1523	7.3441	7.4354	7.4263	7.5528	7.4218	7.5657	7.4173	7.5546	7.4309	7.5834	**7.06**
**H6**	8.0177	8.1926	8.2749	8.2771	8.4094	8.2712	8.4410	8.2654	8.4303	8.2831	8.4600	8.0177	8.1926	8.2749	8.2771	8.4094	8.2712	8.4410	8.2654	8.4303	8.2831	8.4600	**8.35**
**H8**	4.5931	4.6518	4.6646	4.6876	4.7858	4.6853	4.8089	4.6831	4.8002	4.6902	4.7991	4.5931	4.6518	4.6646	4.6876	4.7858	4.6853	4.8089	4.6831	4.8002	4.6902	4.7991	**4.88**
**H(9A-9B)**	1.6901	1.7360	1.7485	1.7400	1.7203	1.7377	1.7240	1.7356	1.7164	1.7426	1.7462	1.6901	1.7360	1.7485	1.7400	1.7203	1.7377	1.7240	1.7356	1.7164	1.7426	1.7462	**2.05**
**H(10A-10B)**	1.4152	1.2960	1.2444	1.2276	1.1642	1.2323	1.1763	1.2369	1.1845	1.2235	1.1606	1.4152	1.2960	1.2444	1.2276	1.1642	1.2323	1.1762	1.2369	1.1845	1.2235	1.1606	**1.36**
**H(11A-11C)**	0.7611	0.7656	0.7386	0.7672	0.7371	0.7669	0.7360	0.7668	0.7397	0.7677	0.7254	0.7611	0.7656	0.7386	0.7672	0.7371	0.7669	0.7360	0.7668	0.7397	0.7677	0.7254	**0.77**
**H(1`A)**	2.7078	2.7124	2.6914	2.6987	2.6511	2.6997	2.6647	2.7008	2.6644	2.6979	2.6436	2.8359	2.8334	2.8251	2.8174	2.7884	2.8186	2.8028	2.8200	2.8005	2.8165	2.7709	**3.03**
**H(1`B)**	3.1881	3.2199	3.2554	3.2500	3.3125	3.2475	3.2980	3.2452	3.2948	3.2527	3.2997	2.8681	2.7298	2.7147	2.6956	2.6924	2.6978	2.6789	2.7002	2.6697	2.6937	2.6724	**3.23**
**H(2`-3`A-2`-3`B)**	1.6629	1.7224	1.6860	1.7638	1.7243	1.7616	1.7439	1.7594	1.7405	1.7663	1.7711	1.6629	1.7224	1.6861	1.7638	1.7243	1.7616	1.7439	1.7594	1.7405	1.7662	1.7711	**1.77**
**H(4`A)**	2.8681	2.7298	2.7147	2.6956	2.6924	2.6978	2.6788	2.7002	2.6697	2.6937	2.6724	3.1881	3.2199	3.2554	3.2500	3.3125	3.2475	3.2980	3.2452	3.2948	3.2527	3.2997	**3.03**
**H(4`B)**	2.8359	2.8334	2.8251	2.8174	2.7884	2.8186	2.8028	2.8200	2.8005	2.8165	2.7709	2.7078	2.7124	2.6914	2.6987	2.6511	2.6997	2.6647	2.7008	2.6644	2.6979	2.6436	**3.23**

[a] methyl protons shifts are averaged

[b] according to [4]

Table S4. Cont. II

Nuclei	R: Gas	R: $CHCl_3$ (PCM)	R: $CHCl_3$ (SMD)	R: DMSO (PCM)	R: DMSO (SMD)	R: CH_3OH (PCM)	R: CH_3OH (SMD)	R: C_2H_5OH (PCM)	R: C_2H_5OH (SMD)	R: H_2O (PCM)	R: H_2O (SMD)
	**B971**										
**C1**	137.7896	138.7258	139.4757	139.3145	141.3703	139.2969	140.8309	139.2753	140.7248	139.3352	141.1889
**C2**	138.1048	138.0869	138.6350	137.9511	138.5060	137.9671	138.9203	137.9828	138.8733	137.9418	138.9371
**C3**	120.7076	121.4428	121.9913	121.7128	122.4280	121.7077	122.7193	121.7016	122.6649	121.7232	122.8145
**C4**	173.5909	174.3695	174.9990	174.7694	175.4614	174.7530	176.0418	174.7363	175.9345	174.7910	176.1612
**C5**	118.7920	120.1069	120.6911	120.8026	121.6266	120.7691	121.8331	120.7350	121.7422	120.8401	121.9820
**C6**	134.5937	136.4061	137.4903	137.3977	138.7332	137.3391	139.3078	137.2814	139.0782	137.4601	139.5577
**C7**	200.3690	205.0557	206.2416	207.7038	208.8030	207.5540	213.9618	207.4011	212.5592	207.8508	215.1135
**C8**	66.2089	67.4207	68.2147	68.0185	69.0026	67.9848	69.6635	67.9509	69.3168	68.0569	70.0400
**C9**	38.4612	38.2837	38.5360	38.0822	38.3178	38.1001	38.4549	38.1185	38.3110	38.0718	38.5001
**C10**	20.1289	20.5580	20.8276	20.7907	21.2145	20.7818	21.1285	20.7727	21.0890	20.8054	21.2735
**C11**	16.2682	16.4143	16.5169	16.4684	16.6720	16.4723	16.6310	16.4757	16.6242	16.4709	16.7883
**C1`**	52.3575	53.1298	53.3760	53.6428	54.1294	53.6181	54.1018	53.5927	54.0746	53.6717	54.5739
**C2`**	26.1707	26.5679	26.8437	26.7698	27.1862	26.7633	27.2121	26.7565	27.1781	26.7821	27.0715
**C3`**	25.6512	25.8535	26.1266	25.9996	26.5125	25.9994	26.4279	25.9984	26.4461	26.0053	26.2931
**C4`**	56.8144	57.7041	58.0473	58.1960	58.7517	58.1727	58.8883	58.1489	58.8567	58.2244	59.1183
**H2**	8.6882	8.5915	8.6209	8.5103	8.4727	8.5144	8.4810	8.5188	8.5000	8.5071	8.4668
**H3**	7.3085	7.4501	7.5415	7.4990	7.6202	7.4966	7.6384	7.4942	7.6316	7.5016	7.6522
**H5**	7.1494	7.3412	7.4323	7.4259	7.5519	7.4211	7.5645	7.4163	7.5535	7.4308	7.5823
**H6**	8.0262	8.1982	8.2822	8.2875	8.3973	8.2816	8.4362	8.2759	8.4293	8.2936	8.4567
**H8**	4.5982	4.6556	4.6721	4.6947	4.7692	4.6923	4.8069	4.6900	4.8035	4.6974	4.7993
**H(9A-9B)**	1.6969	1.7418	1.7519	1.7550	1.7355	1.7530	1.7286	1.7513	1.7184	1.7575	1.7500
**H(10A-10B)**	1.4117	1.2931	1.2426	1.2275	1.1562	1.2319	1.1722	1.2362	1.1828	1.2238	1.1586
**H(11A-11C)**	0.7557	0.7592	0.7327	0.7628	0.7269	0.7624	0.7273	0.7620	0.7328	0.7637	0.7178
**H(1`A)**	2.6983	2.7040	2.6840	2.6920	2.6456	2.6929	2.6578	2.6938	2.6567	2.6916	2.6341
**H(1`B)**	3.1922	3.2226	3.2567	3.2538	3.3131	3.2513	3.2981	3.2489	3.2952	3.2567	3.2988
**H(2`-3`A-2`-3`B)**	1.6619	1.7208	1.6851	1.7611	1.7208	1.7585	1.7415	1.7560	1.7388	1.7639	1.7689
**H(4`A)**	2.8701	2.7333	2.7168	2.6954	2.7118	2.6975	2.6836	2.6997	2.6697	2.6938	2.6730
**H(4`B)**	2.8264	2.8244	2.8168	2.8089	2.7828	2.8100	2.7961	2.8111	2.7921	2.8083	2.7640
Nuclei	**B972**										
**C1**	134.7000	136.2436	137.1818	136.8329	138.4324	136.7985	137.8759	136.7628	137.7451	136.8625	138.2351
**C2**	136.0990	136.0628	136.6204	135.9754	136.5157	135.9836	136.9002	135.9887	136.8502	135.9635	136.9116
**C3**	118.5870	119.3172	119.9025	119.6168	120.2925	119.6024	120.5790	119.5852	120.5089	119.6260	120.6806
**C4**	170.5548	171.2810	171.9148	171.6922	172.3497	171.6680	172.9154	171.6416	172.7991	171.7106	173.0415
**C5**	116.7290	118.0016	118.6162	118.6724	119.4580	118.6330	119.6671	118.5914	119.5732	118.7054	119.8271
**C6**	132.3207	133.8605	134.8157	134.8931	136.2813	134.8294	136.8581	134.7633	136.6544	134.9490	137.1200
**C7**	197.9191	203.0510	204.3145	205.7374	206.2945	205.5739	211.3106	205.4087	209.9428	205.8898	212.4204
**C8**	63.4911	64.8297	65.5365	65.6161	66.2773	65.5680	66.9467	65.5180	66.6397	65.6578	67.2902
**C9**	36.4665	36.1386	36.2826	36.0589	36.2549	36.0665	36.4100	36.0714	36.3170	36.0469	36.4935
**C10**	18.8488	19.2698	19.5409	19.6061	20.0211	19.5873	19.9147	19.5659	19.8801	19.6194	20.0861
**C11**	15.8897	16.0176	16.1272	16.1216	16.2966	16.1158	16.2532	16.1079	16.2460	16.1230	16.4162
**C1`**	50.3452	51.1497	51.4234	51.6980	52.1403	51.6664	52.1193	51.6323	52.0919	51.7233	52.5644
**C2`**	25.1240	25.4950	25.7942	25.7589	26.1290	25.7426	26.1316	25.7238	26.1053	25.7704	25.9833
**C3`**	24.6170	24.8027	25.1397	24.9600	25.4610	24.9502	25.3578	24.9383	25.3545	24.9650	25.1771
**C4`**	54.6924	55.6376	56.0022	56.1680	56.6750	56.1343	56.7805	56.0986	56.7295	56.1964	57.0279
**H2**	8.6882	8.5150	8.5284	8.4403	8.4433	8.4451	8.4562	8.4495	8.4736	8.4356	8.4447
**H3**	7.3161	7.4512	7.5417	7.4954	7.6123	7.4933	7.6312	7.4909	7.6246	7.4972	7.6480
**H5**	7.1578	7.3389	7.4289	7.4180	7.5411	7.4135	7.5577	7.4087	7.5460	7.4222	7.5769
**H6**	8.0334	8.1775	8.2555	8.2618	8.3931	8.2564	8.4269	8.2508	8.4195	8.2668	8.4475
**H8**	4.6059	4.6489	4.6756	4.6813	4.7723	4.6784	4.8012	4.6755	4.7908	4.6840	4.7938
**H(9A-9B)**	1.6638	1.6691	1.6582	1.6982	1.6869	1.6966	1.6880	1.6946	1.6833	1.6995	1.7129
**H(10A-10B)**	1.3756	1.2612	1.2159	1.1986	1.1370	1.2026	1.1556	1.2063	1.1622	1.1947	1.1443
**H(11A-11C)**	0.7480	0.7466	0.7214	0.7531	0.7224	0.7525	0.7247	0.7518	0.7276	0.7536	0.7164
**H(1`A)**	2.6909	2.6924	2.6690	2.6803	2.6344	2.6813	2.6467	2.6821	2.6463	2.6792	2.6221
**H(1`B)**	3.1749	3.2131	3.2469	3.2467	3.3078	3.2444	3.2919	3.2419	3.2886	3.2488	3.2903
**H(2`-3`A-2`-3`B)**	1.6437	1.7062	1.6731	1.7448	1.7011	1.7423	1.7207	1.7397	1.7165	1.7470	1.7477
**H(4`A)**	2.8410	2.7272	2.7113	2.6915	2.6918	2.6936	2.6671	2.6955	2.6562	2.6895	2.6608
**H(4`B)**	2.8262	2.8279	2.8199	2.8132	2.7776	2.8142	2.7912	2.8150	2.7884	2.8122	2.7566

Nuclei	S: Gas	S: $CHCl_3$ (PCM)	S: $CHCl_3$ (SMD)	S: DMSO (PCM)	S: DMSO (SMD)	S: CH_3OH (PCM)	S: CH_3OH (SMD)	S: C_2H_5OH (PCM)	S: C_2H_5OH (SMD)	S: H_2O (PCM)	S: H_2O (SMD)	**Exp.** [b]
	**B971**											
**C1**	137.7897	138.7257	139.4757	139.3145	141.3704	139.2969	140.8309	139.2753	140.7248	139.3352	141.1889	**133.8**
**C2**	138.1048	138.0869	138.6351	137.9511	138.5061	137.9671	138.9203	137.9827	138.8733	137.9418	138.9372	**132.1**
**C3**	120.7077	121.4429	121.9912	121.7128	122.4279	121.7077	122.7193	121.7016	122.6649	121.7232	122.8144	**115.8**
**C4**	173.5907	174.3696	174.9991	174.7693	175.4613	174.7530	176.0419	174.7364	175.9345	174.7910	176.1611	**165.8**
**C5**	118.7921	120.1070	120.6912	120.8026	121.6267	120.7691	121.8330	120.7351	121.7422	120.8401	121.9820	**115.8**
**C6**	134.5937	136.4060	137.4904	137.3977	138.7332	137.3391	139.3077	137.2816	139.0782	137.4601	139.5577	**132.1**
**C7**	200.3691	205.0558	206.2416	207.7038	208.8030	207.5540	213.9616	207.4011	212.5592	207.8508	215.1135	**198.9**
**C8**	66.2089	67.4207	68.2147	68.0186	69.0027	67.9848	69.6636	67.9509	69.3168	68.0569	70.4000	**69.3**
**C9**	38.4612	38.2837	38.5360	38.0822	38.3179	38.1001	38.4549	38.1184	38.3110	38.0718	38.5001	**31.5**
**C10**	20.1289	20.5579	20.8276	20.7908	21.2145	20.7818	21.1285	20.7727	21.0890	20.8054	21.2735	**19.9**
**C11**	16.2682	16.4142	16.5169	16.4685	16.6719	16.4723	16.6310	16.4756	16.6242	16.4709	16.7882	**14.3**
**C1`**	56.8143	57.7040	58.0473	58.1961	58.7517	58.1727	58.8883	58.1489	58.8567	58.2244	59.1183	**50.6**
**C2`**	25.6513	25.8535	26.1266	25.9996	26.5124	25.9994	26.4278	25.9984	26.4461	26.0053	26.2931	**23.8**
**C3`**	26.1707	26.5680	26.8437	26.7698	27.1863	26.7633	27.2122	26.7565	27.1781	26.7821	27.0716	**23.8**
**C4`**	52.3575	53.1298	53.3759	53.6428	54.1294	53.6182	54.1016	53.5925	54.0746	53.6717	54.5739	**50.6**
**H2**	8.6882	8.5915	8.6209	8.5103	8.4727	8.5144	8.4811	8.5188	8.5000	8.5071	8.4668	**8.35**
**H3**	7.3085	7.4502	7.5415	7.4990	7.6202	7.4966	7.6384	7.4942	7.6316	7.5016	7.6522	**7.06**
**H5**	7.1494	7.3412	7.4323	7.4259	7.5519	7.4211	7.5645	7.4163	7.5535	7.4308	7.5823	**7.06**
**H6**	8.0262	8.1982	8.2822	8.2875	8.3973	8.2816	8.4362	8.2759	8.4293	8.2936	8.4567	**8.35**
**H8**	4.5982	4.6556	4.6721	4.6945	4.7692	4.6923	4.8069	4.6900	4.8035	4.6974	4.7993	**4.88**
**H(9A-9B)**	1.6969	1.7418	1.7519	1.7550	1.7355	1.7530	1.7286	1.7513	1.7184	1.7575	1.7500	**2.05**
**H(10A-10B)**	1.4117	1.2931	1.2426	1.2275	1.1562	1.2319	1.1722	1.2362	1.1828	1.2238	1.1586	**1.36**
**H(11A-11C)**	0.7557	0.7592	0.7327	0.7628	0.7269	0.7624	0.7273	0.7620	0.7328	0.7637	0.7178	**0.77**
**H(1`A)**	2.8264	2.8244	2.8168	2.8089	2.7828	2.8100	2.7961	2.8111	2.7921	2.8083	2.7640	**3.03**
**H(1`B)**	2.8701	2.7333	2.7168	2.6954	2.7118	2.6975	2.6836	2.6997	2.6697	2.6938	2.6730	**3.23**
**H(2`-3`A-2`-3`B)**	1.6619	1.7208	1.6851	1.7611	1.7208	1.7585	1.7415	1.7560	1.7388	1.7639	1.7689	**1.77**
**H(4`A)**	3.1922	3.2226	3.2567	3.2538	3.3131	3.2513	3.2981	3.2489	3.2952	3.2567	3.2988	**3.03**
**H(4`B)**	2.6983	2.7040	2.6840	2.6920	2.6456	2.6929	2.6578	2.6938	2.6567	2.6916	2.6341	**3.23**
Nuclei	**B972**											
**C1**	134.7000	136.2436	137.1818	136.8330	138.4324	136.7985	137.8759	136.7628	137.7451	136.8625	138.2351	**133.8**
**C2**	136.0991	136.0628	136.6204	135.9755	136.5157	135.9836	136.9002	135.9887	136.8502	135.9635	136.9116	**132.1**
**C3**	118.5870	119.3172	119.9024	119.6168	120.2924	119.6024	120.5789	119.5852	120.5089	119.6260	120.6806	**115.8**
**C4**	170.5549	171.2810	171.9147	171.6921	172.3496	171.6680	172.9153	171.6416	172.7991	171.7107	173.0415	**165.8**
**C5**	116.7289	118.0016	118.6162	118.6724	119.4580	118.6330	119.6671	118.5914	119.5732	118.7054	119.8271	**115.8**
**C6**	132.3206	133.8605	134.8157	134.8932	136.2813	134.8294	136.8581	134.7633	136.6544	134.9490	137.1200	**132.1**
**C7**	197.9188	203.0510	204.3144	205.7374	206.2946	205.5739	211.3106	205.4087	209.9428	205.8898	212.4204	**198.9**
**C8**	63.4911	64.8297	65.5366	65.6161	66.2773	65.5680	66.9467	65.5180	66.6397	65.6578	67.2902	**69.3**
**C9**	36.4665	36.1386	36.2827	36.0589	36.2549	36.0665	36.4101	36.0714	36.3170	36.0469	36.4935	**31.5**
**C10**	18.8488	19.2698	19.5408	19.6061	20.0211	19.5873	19.9146	19.5659	19.8801	19.6194	20.0861	**19.9**
**C11**	15.8897	16.0176	16.1271	16.1216	16.2966	16.1158	16.2531	16.1079	16.2460	16.1230	16.4162	**14.3**
**C1`**	54.6924	55.6376	56.0022	56.1680	56.6754	56.1343	56.7805	56.0986	56.7295	56.1964	57.0279	**50.6**
**C2`**	24.6170	24.8027	25.1396	24.9599	25.4611	24.9502	25.3578	24.9384	25.3545	24.9649	25.1771	**23.8**
**C3`**	25.1242	25.4950	25.7941	25.7589	26.1290	25.7426	26.1315	25.7238	26.1053	25.7704	25.9833	**23.8**
**C4`**	50.3452	51.1497	51.4235	51.6979	52.1403	51.6664	52.1192	51.6323	52.0919	51.7233	52.5644	**50.6**
**H2**	8.6882	8.5150	8.5284	8.4403	8.4433	8.4451	8.4562	8.4495	8.4736	8.4356	8.4447	**8.35**
**H3**	7.3161	7.4512	7.5417	7.4954	7.6123	7.4933	7.6312	7.4909	7.6246	7.4972	7.6480	**7.06**
**H5**	7.1578	7.3389	7.4289	7.4180	7.5411	7.4135	7.5577	7.4087	7.5460	7.4222	7.5769	**7.06**
**H6**	8.0334	8.1775	8.2555	8.2618	8.3931	8.2564	8.4269	8.2508	8.4195	8.2668	8.4475	**8.35**
**H8**	4.6059	4.6489	4.6756	4.6813	4.7723	4.6784	4.8012	4.6755	4.7908	4.6840	4.7938	**4.88**
**H(9A-9B)**	1.6638	1.6691	1.6582	1.6982	1.6870	1.6966	1.6880	1.6946	1.6833	1.6995	1.7129	**2.05**
**H(10A-10B)**	1.3756	1.2612	1.2158	1.1986	1.1370	1.2027	1.1555	1.2063	1.1622	1.1947	1.1443	**1.36**
**H(11A-11C)**	0.7480	0.7466	0.7214	0.7531	0.7224	0.7525	0.7247	0.7518	0.7276	0.7536	0.7164	**0.77**
**H(1`A)**	2.8262	2.8279	2.8199	2.8132	2.7776	2.8142	2.7913	2.8150	2.7885	2.8122	2.7566	**3.03**
**H(1`B)**	2.8410	2.7272	2.7113	2.6915	2.6918	2.6936	2.6671	2.6955	2.6562	2.6895	2.6608	**3.23**
**H(2`-3`A-2`-3`B)**	1.6438	1.7062	1.6730	1.7448	1.7011	1.7423	1.7207	1.7397	1.7165	1.7470	1.7477	**1.77**
**H(4`A)**	3.1749	3.2131	3.2469	3.2467	3.3078	3.2444	3.2919	3.2419	3.2886	3.2488	3.2903	**3.03**
**H(4`B)**	2.6909	2.6924	2.6690	2.6803	2.6344	2.6813	2.6467	2.6821	2.6463	2.6792	2.6221	**3.23**

[a] methyl protons shifts are averaged

[b] according to [4]

Table S4. Cont. III

Nuclei	R											S											Exp. [b]
	BHandH																						
	Gas	$CHCl_3$ (PCM)	$CHCl_3$ (SMD)	DMSO (PCM)	DMSO (SMD)	CH_3OH (PCM)	CH_3OH (SMD)	C_2H_5OH (PCM)	C_2H_5OH (SMD)	H_2O (PCM)	H_2O (SMD)	Gas	$CHCl_3$ (PCM)	$CHCl_3$ (SMD)	DMSO (PCM)	DMSO (SMD)	CH_3OH (PCM)	CH_3OH (SMD)	C_2H_5OH (PCM)	C_2H_5OH (SMD)	H_2O (PCM)	H_2O (SMD)	
**C1**	138.6239	139.3098	140.1633	139.5276	141.1787	139.5165	140.0987	139.5222	140.2563	139.5071	140.4432	138.6239	139.3098	140.1633	139.5276	140.2717	139.5165	140.0987	139.5222	140.2563	139.5071	140.4431	**133.8**
**C2**	143.5738	143.4947	144.0312	143.3147	143.8526	143.3175	144.3129	143.3387	144.2043	143.2814	144.3448	143.5738	143.4947	144.0312	143.3147	144.0588	143.3175	144.3129	143.3387	144.2043	143.2814	144.3448	**132.1**
**C3**	123.6964	124.4238	125.0999	124.6746	125.3915	124.6516	125.6126	124.6467	125.5819	124.6660	125.6923	123.6964	124.4238	125.0999	124.6746	125.4187	124.6516	125.6126	124.6467	125.5819	124.6660	125.6922	**115.8**
**C4**	174.7958	175.5262	176.1958	175.9464	176.5287	175.9082	177.2561	175.8886	177.0899	175.9528	177.3931	174.7958	175.5262	176.1958	175.9464	176.6905	175.9083	177.2561	175.8886	177.0899	175.9529	177.3932	**165.8**
**C5**	121.4818	122.8933	123.6425	123.6703	124.5646	123.6119	124.8041	123.5718	124.7042	123.6956	124.9837	121.4818	122.8933	123.6425	123.6703	124.4144	123.6119	124.8041	123.5718	124.7042	123.6956	124.9838	**115.8**
**C6**	139.8395	141.7777	142.9069	142.9335	144.5142	142.8465	145.2948	142.7787	145.0237	142.9871	145.6621	139.8395	141.7777	142.9069	142.9335	143.6776	142.8465	145.2948	142.7787	145.0237	142.9870	145.6622	**132.1**
**C7**	204.1536	208.7473	209.9381	211.1734	211.8512	211.0273	216.7295	210.8976	215.6262	211.2809	217.9024	204.1536	208.7473	209.9381	211.1734	211.9175	211.0273	216.7295	210.8976	215.6262	211.2809	217.9027	**198.9**
**C8**	60.1105	61.2911	61.8318	61.8324	62.6666	61.7978	63.0997	61.7803	62.9850	61.8343	63.6345	60.1105	61.2911	61.8318	61.8324	62.5765	61.7978	63.0997	61.7803	62.9850	61.8343	63.6346	**69.3**
**C9**	36.0832	35.8942	36.0047	35.7106	36.0824	35.7146	36.0744	35.7361	36.0332	35.6765	36.3132	36.0832	35.8942	36.0047	35.7106	36.4547	35.7146	36.0744	35.7361	36.0332	35.6764	36.3132	**31.5**
**C10**	16.5118	16.9597	17.2604	17.2441	17.7611	17.2147	17.6352	17.2037	17.6119	17.2423	17.8191	16.5118	16.9597	17.2604	17.2441	17.9882	17.2147	17.6352	17.2037	17.6119	17.2423	17.8192	**19.9**
**C11**	15.0257	15.1517	15.3021	15.1736	15.4059	15.1626	15.3623	15.1698	15.3719	15.1539	15.5551	15.0257	15.1517	15.3021	15.1736	15.9177	15.1626	15.3623	15.1698	15.3718	15.1540	15.5550	**14.3**
**C1`**	48.2361	49.0965	49.3704	49.5476	50.1639	49.5114	50.0607	49.4936	50.0482	49.5517	50.5118	52.1815	53.1322	53.5867	53.6620	54.4061	53.6183	54.3625	53.5933	54.3033	53.6736	54.6277	**50.6**
**C2`**	21.6424	22.0034	22.3249	22.2037	22.6190	22.1811	22.6630	22.1771	22.6622	22.1954	22.6800	21.0763	21.2802	21.6706	21.4316	22.1757	21.4090	21.8657	21.4052	21.8725	21.4234	21.7951	**23.8**
**C3`**	21.0763	21.2802	21.6706	21.4316	21.9126	21.4090	21.8657	21.4052	21.8725	21.4234	21.7950	21.6424	22.0034	22.3249	22.2037	22.9478	22.1811	22.6630	22.1771	22.6622	22.1954	22.6801	**23.8**
**C4`**	52.1815	53.1322	53.5867	53.6620	54.2099	53.6183	54.3625	53.5933	54.3033	53.6736	54.6277	48.2361	49.0965	49.3704	49.5476	50.2917	49.5114	50.0607	49.4936	50.0482	49.5517	50.5119	**50.6**
**H2**	9.0187	8.9184	8.9416	8.8630	8.8530	8.8660	8.9011	8.8684	8.8933	8.8611	8.8800	9.0187	8.9184	8.9416	8.8630	8.7818	8.8660	8.9011	8.8684	8.8933	8.8612	8.8800	**8.35**
**H3**	7.4583	7.5981	7.6918	7.6419	7.7660	7.6406	7.7830	7.6384	7.7755	7.6441	7.7978	7.4583	7.5981	7.6918	7.6419	7.5607	7.6406	7.7830	7.6384	7.7755	7.6441	7.7978	**7.06**
**H5**	7.2809	7.4790	7.5747	7.5691	7.7003	7.5646	7.7181	7.5593	7.7066	7.5743	7.7422	7.2809	7.4790	7.5747	7.5691	7.4879	7.5646	7.7181	7.5593	7.7066	7.5743	7.7422	**7.06**
**H6**	8.2276	8.4076	8.5091	8.5190	8.6554	8.5119	8.7355	8.5042	8.7089	8.5268	8.7614	8.2276	8.4076	8.5091	8.5190	8.4378	8.5119	8.7355	8.5042	8.7089	8.5268	8.7614	**8.35**
**H8**	4.2707	4.3319	4.4006	4.3985	4.4661	4.3936	4.5556	4.3880	4.5270	4.4043	4.5618	4.2707	4.3319	4.4006	4.3985	4.3173	4.3936	4.5556	4.3880	4.5270	4.4043	4.5618	**4.88**
**H(9A-9B)**	1.7526	1.8039	1.7713	1.8251	1.8368	1.8248	1.8202	1.8236	1.8142	1.8264	1.8579	1.7526	1.8039	1.7713	1.8251	1.7438	1.8248	1.8202	1.8236	1.8142	1.8264	1.8579	**2.05**
**H(10A-10B)**	1.3693	1.2395	1.2010	1.1704	1.0883	1.1748	1.1128	1.1785	1.1147	1.1672	1.0936	1.3693	1.2395	1.2010	1.1704	1.0891	1.1748	1.1128	1.1785	1.1147	1.1672	1.0936	**1.36**
**H(11A-11C)**	0.7598	0.7638	0.7408	0.7704	0.7291	0.7705	0.7393	0.7696	0.7386	0.7715	0.7301	0.7598	0.7638	0.7408	0.7704	0.6892	0.7705	0.7393	0.7696	0.7386	0.7715	0.7301	**0.77**
**H(1`A)**	2.5811	2.5765	2.5597	2.5625	2.5141	2.5642	2.5321	2.5650	2.5320	2.5619	2.5148	2.7241	2.7319	2.7078	2.7181	2.6783	2.7204	2.6863	2.7218	2.6889	2.7169	2.6658	**3.03**
**H(1`B)**	3.0809	3.1130	3.1395	3.1381	3.1895	3.1372	3.1758	3.1354	3.1708	3.1400	3.1806	2.8917	2.7175	2.6455	2.6342	2.6346	2.6408	2.5366	2.6465	2.5450	2.6289	2.5331	**3.23**
**H(2`-3`A-2`-3`B)**	1.6289	1.6841	1.6486	1.7194	1.6743	1.7179	1.6955	1.7156	1.6898	1.7218	1.7223	1.6289	1.6841	1.6486	1.7194	1.6381	1.7179	1.6955	1.7156	1.6898	1.7218	1.7223	**1.77**
**H(4`A)**	2.8917	2.7175	2.6455	2.6342	2.6346	2.6408	2.5366	2.6465	2.5450	2.6289	2.5331	3.0809	3.1130	3.1395	3.1381	3.1895	3.1372	3.1758	3.1354	3.1708	3.1400	3.1806	**3.03**
**H(4`B)**	2.7241	2.7319	2.7078	2.7181	2.6782	2.7204	2.6863	2.7218	2.6889	2.7169	2.6658	2.5811	2.5765	2.5597	2.5625	2.5141	2.5642	2.5321	2.5650	2.5320	2.5619	2.5148	**3.23**
Nuclei	BHandHLYP																						
**C1**	139.1995	139.9344	140.7124	140.2115	142.4073	140.1838	141.7706	140.1622	141.7596	140.2418	142.2408	139.1995	139.9344	140.7124	140.2116	142.4071	140.1838	141.7706	140.1622	141.7596	140.2418	142.2408	**133.8**
**C2**	142.1036	141.9531	142.4769	141.8156	142.2866	141.8255	142.7286	141.8377	142.6885	141.8061	142.7175	142.1036	141.9531	142.4769	141.8158	142.2866	141.8255	142.7286	141.8376	142.6885	141.8060	142.7175	**132.1**
**C3**	122.4961	123.1091	123.6312	123.3084	124.0035	123.2980	124.1932	123.2900	124.1687	123.3180	124.2908	122.4961	123.1091	123.6312	123.3085	124.0035	123.2980	124.1932	123.2900	124.1687	123.3180	124.2908	**115.8**
**C4**	174.5922	175.2566	175.8308	175.6409	176.2467	175.6172	176.8399	175.5962	176.7288	175.6628	176.9445	174.5922	175.2566	175.8308	175.6409	176.2467	175.6172	176.8399	175.5962	176.7288	175.6628	176.9445	**165.8**
**C5**	120.3085	121.5940	122.1677	122.2586	123.0862	122.2171	123.2268	122.1783	123.1489	122.2980	123.3912	120.3085	121.5940	122.1677	122.2586	123.0863	122.2171	123.2268	122.1783	123.1489	122.2981	123.3912	**115.8**
**C6**	138.1484	139.9784	141.0642	141.0829	142.1719	141.0196	142.8408	140.9569	142.5998	141.1408	143.0584	138.1484	139.9784	141.0642	141.0829	142.1720	141.0196	142.8408	140.9569	142.5997	141.1408	143.0584	**132.1**
**C7**	202.2649	207.2010	208.3379	209.8082	210.7606	209.6369	216.7690	209.4723	215.2774	209.9747	218.0633	202.2649	207.2010	208.3379	209.8079	210.7610	209.6369	216.7690	209.4723	215.2771	209.9744	218.0633	**198.9**
**C8**	61.8458	62.8304	63.5344	63.3115	63.9662	63.2792	64.8164	63.2505	64.5141	63.3431	65.2211	61.8458	62.8304	63.5344	63.3115	63.9662	63.2792	64.8164	63.2505	64.5142	63.3431	65.2211	**69.3**
**C9**	35.5682	35.2268	35.4409	35.0480	35.0587	35.0643	35.1701	35.0818	35.0932	35.0309	35.1967	35.5682	35.2268	35.4409	35.0480	35.0587	35.0643	35.1701	35.0817	35.0932	35.0307	35.1967	**31.5**
**C10**	18.3108	18.7220	18.9982	18.9799	19.3800	18.9644	19.2649	18.9513	19.2456	18.9941	19.3876	18.3108	18.7220	18.9982	18.9799	19.3800	18.9644	19.2650	18.9513	19.2456	18.9941	19.3876	**19.9**
**C11**	15.8988	16.0123	16.1497	16.0717	16.2865	16.0682	16.2351	16.0671	16.2477	16.0747	16.3848	15.8988	16.0123	16.1497	16.0717	16.2865	16.0682	16.2351	16.0670	16.2477	16.0746	16.3848	**14.3**
**C1`**	49.0619	49.8235	50.1189	50.3249	50.8251	50.2909	50.8340	50.2599	50.8225	50.3574	51.2993	53.3229	54.2124	54.5676	54.6856	55.2693	54.6539	55.3618	54.6254	55.3266	54.7162	55.6172	**50.6**
**C2`**	24.4347	24.7574	25.0607	24.9331	25.3432	24.9209	25.3584	24.9113	25.3395	24.9448	25.2085	23.8796	24.0140	24.3263	24.1401	24.7203	24.1309	24.5630	24.1244	24.5892	24.1486	24.4005	**23.8**
**C3`**	23.8796	24.0140	24.3263	24.1401	24.7203	24.1309	24.5630	24.1245	24.5892	24.1486	24.4005	24.4347	24.7574	25.0607	24.9331	25.3432	24.9209	25.3584	24.9113	25.3394	24.9449	25.2085	**23.8**
**C4`**	53.3229	54.2124	54.5676	54.6856	55.2692	54.6539	55.3618	54.6254	55.3266	54.7162	55.6172	49.0620	49.8235	50.1189	50.3249	50.8250	50.2909	50.8340	50.2599	50.8223	50.3574	51.2993	**50.6**
**H2**	8.9120	8.8065	8.8331	8.7485	8.6943	8.7536	8.6881	8.7581	8.7047	8.7432	8.6588	8.9120	8.8065	8.8331	8.7485	8.6943	8.7536	8.6881	8.7581	8.7047	8.7432	8.6588	**8.35**
**H3**	7.4376	7.5713	7.6590	7.6162	7.7362	7.6141	7.7545	7.6118	7.7469	7.6181	7.7692	7.4376	7.5713	7.6590	7.6162	7.7362	7.6141	7.7545	7.6118	7.7470	7.6181	7.7692	**7.06**
**H5**	7.2696	7.4607	7.5483	7.5438	7.6626	7.5393	7.6778	7.5346	7.6656	7.5480	7.6980	7.2696	7.4607	7.5483	7.5438	7.6626	7.5393	7.6778	7.5346	7.6656	7.5481	7.6980	**7.06**
**H6**	8.0935	8.2902	8.3789	8.4029	8.5310	8.3964	8.5708	8.3897	8.5558	8.4089	8.5892	8.0935	8.2902	8.3789	8.4029	8.5310	8.3964	8.5708	8.3897	8.5558	8.4089	8.5892	**8.35**
**H8**	4.2157	4.3089	4.3348	4.3694	4.4804	4.3657	4.5083	4.3619	4.4942	4.3727	4.4993	4.2157	4.3089	4.3348	4.3694	4.4804	4.3657	4.5083	4.3619	4.4942	4.3727	4.4993	**4.88**
**H(9A-9B)**	1.5615	1.6076	1.6122	1.6423	1.5807	1.6413	1.5917	1.6398	1.5810	1.6430	1.6082	1.5615	1.6076	1.6122	1.6423	1.5807	1.6413	1.5917	1.6397	1.5810	1.6430	1.6082	**2.05**
**H(10A-10B)**	1.3552	1.2326	1.1775	1.1688	1.1078	1.1731	1.1127	1.1772	1.1234	1.1646	1.1005	1.3552	1.2326	1.1775	1.1688	1.1078	1.1731	1.1127	1.1773	1.1234	1.1646	1.1005	**1.36**
**H(11A-11C)**	0.7791	0.7824	0.7529	0.7916	0.7546	0.7913	0.7544	0.7908	0.7581	0.7918	0.7460	0.7791	0.7824	0.7529	0.7916	0.7546	0.7913	0.7544	0.7908	0.7581	0.7918	0.7460	**0.77**
**H(1`A)**	2.5995	2.6032	2.5768	2.5918	2.5385	2.5929	2.5592	2.5940	2.5572	2.5906	2.5374	2.7413	2.7309	2.7180	2.7108	2.6715	2.7123	2.6963	2.7136	2.6903	2.7094	2.6678	**3.03**
**H(1`B)**	3.0093	3.0609	3.0950	3.0991	3.1584	3.0968	3.1477	3.0944	3.1427	3.1012	3.1491	2.7058	2.5710	2.5558	2.5295	2.5291	2.5317	2.5179	2.5339	2.5054	2.5275	2.5185	**3.23**
**H(2`-3`A-2`-3`B)**	1.5889	1.6489	1.6104	1.6887	1.6526	1.6860	1.6745	1.6833	1.6697	1.6913	1.7016	1.5889	1.6489	1.6104	1.6887	1.6526	1.6860	1.6745	1.6833	1.6697	1.6913	1.7016	**1.77**
**H(4`A)**	2.7058	2.5710	2.5558	2.5295	2.5291	2.5317	2.5179	2.5339	2.5054	2.5275	2.5185	3.0093	3.0609	3.0950	3.0991	3.1583	3.0968	3.1477	3.0944	3.1426	3.1012	3.1491	**3.03**
**H(4`B)**	2.7413	2.7309	2.7180	2.7108	2.6715	2.7123	2.6963	2.7136	2.6903	2.7094	2.6678	2.5995	2.6032	2.5768	2.5918	2.5385	2.5929	2.5592	2.5940	2.5572	2.5906	2.5374	**3.23**

[a] methyl protons shifts are averaged

[b] according to [4]

Table S4. Cont. IV

Nuclei	R											S											**Exp.[b]**
	**BLYP**																						
	Gas	$CHCl_3$ (PCM)	$CHCl_3$ (SMD)	DMSO (PCM)	DMSO (SMD)	CH_3OH (PCM)	CH_3OH (SMD)	C_2H_5OH (PCM)	C_2H_5OH (SMD)	H_2O (PCM)	H_2O (SMD)	Gas	$CHCl_3$ (PCM)	$CHCl_3$ (SMD)	DMSO (PCM)	DMSO (SMD)	CH_3OH (PCM)	CH_3OH (SMD)	C_2H_5OH (PCM)	C_2H_5OH (SMD)	H_2O (PCM)	H_2O (SMD)	
**C1**	139.4895	140.9405	141.8206	141.4766	143.2818	141.7006	142.4159	141.4678	142.7930	141.8949	142.8093	139.4895	140.9405	141.8206	141.4766	143.2818	141.7006	142.4159	141.4678	142.7930	141.8949	142.8094	**133.8**
**C2**	137.2308	137.2081	137.6806	137.0743	137.6665	137.3605	137.9679	137.1854	138.0635	137.4306	138.0853	137.2309	137.2081	137.6806	137.0743	137.6665	137.3603	137.9679	137.1854	138.0635	137.4306	138.0855	**132.1**
**C3**	120.6710	121.3939	121.9003	121.6308	122.3081	121.8979	122.4734	121.7025	122.5601	122.0037	122.6525	120.6710	121.3939	121.9003	121.6308	122.3081	121.8979	122.4734	121.7025	122.5601	122.0037	122.6525	**115.8**
**C4**	178.0035	178.9429	179.6390	179.3664	180.2338	179.6209	180.6154	179.4131	180.6222	179.7517	180.8326	178.0034	178.9429	179.6390	179.3664	180.2338	179.6209	180.6156	179.4131	180.6222	179.7517	180.8325	**165.8**
**C5**	118.8398	119.9843	120.5493	120.5733	121.3733	120.8158	121.4705	120.5966	121.5178	120.9702	121.6972	118.8397	119.9843	120.5493	120.5733	121.3733	120.8159	121.4705	120.5966	121.5178	120.9702	121.6971	**115.8**
**C6**	133.6979	135.3938	136.4105	136.4593	138.0034	136.6736	138.4632	136.4250	138.3361	136.8823	138.8461	133.6981	135.3938	136.4105	136.4593	138.0034	136.6735	138.4632	136.4250	138.3361	136.8823	138.8460	**132.1**
**C7**	204.1334	209.0563	210.3744	211.5288	212.6039	211.6372	216.8249	211.2867	216.1919	212.0555	217.9816	204.1331	209.0563	210.3744	211.5288	212.6039	211.6372	216.8248	211.2867	216.1919	212.0555	217.9816	**198.9**
**C8**	70.4568	71.7688	72.5312	72.3141	73.3438	72.5565	73.7788	72.3363	73.9759	72.7087	74.1628	70.4568	71.7688	72.5312	72.3141	73.3438	72.5565	73.7789	72.3363	73.9759	72.7087	74.1627	**69.3**
**C9**	41.7168	41.1992	41.3838	40.9811	41.3059	41.2750	41.4199	41.1056	41.5562	41.3276	41.5382	41.7169	41.1992	41.3838	40.9811	41.3059	41.2749	41.4200	41.1056	41.5562	41.3276	41.5381	**31.5**
**C10**	21.7869	22.2253	22.4724	22.4392	23.0090	22.7070	22.7239	22.5118	22.9092	22.8109	22.9318	21.7868	22.2253	22.4724	22.4392	23.0090	22.7070	22.7239	22.5118	22.9092	22.8109	22.9319	**19.9**
**C11**	16.4022	16.5324	16.6233	16.5596	16.8485	16.8378	16.6378	16.6535	16.8340	16.9219	16.8955	16.4022	16.5324	16.6233	16.5596	16.8485	16.8378	16.6376	16.6535	16.8340	16.9219	16.8955	**14.3**
**C1`**	55.2667	56.1003	56.3800	56.5748	57.2181	56.8253	57.0602	56.6135	57.2187	56.9632	57.5578	60.0092	61.1782	61.4954	61.6847	62.2799	61.9321	62.2641	61.7182	62.3281	62.0778	62.6144	**50.6**
**C2`**	27.7055	28.2445	28.4534	28.4560	28.9441	28.7232	28.8130	28.5280	28.9595	28.8289	28.7854	27.5071	27.7380	28.0254	27.8939	28.5101	28.1587	28.2147	27.9626	28.3738	28.2707	28.0968	**23.8**
**C3`**	27.5070	27.7380	28.0254	27.8939	28.5101	28.1587	28.2148	27.9626	28.3737	28.2707	28.0969	27.7054	28.2445	28.4534	28.4560	28.9441	28.7232	28.8130	28.5280	28.9595	28.8289	28.7855	**23.8**
**C4`**	60.0092	61.1782	61.4954	61.6847	62.2799	61.9321	62.2641	61.7182	62.3281	62.0778	62.6144	55.2668	56.1003	56.3800	56.5748	57.2181	56.8254	57.0603	56.6135	57.2187	56.9632	57.5579	**50.6**
**H2**	8.4452	8.2989	8.3074	8.2267	8.2222	8.2803	8.4224	8.2411	8.2440	8.2758	8.4302	8.4452	8.2989	8.3074	8.2267	8.2222	8.2803	8.4224	8.2411	8.2440	8.2758	8.4302	**8.35**
**H3**	7.1620	7.2929	7.3792	7.3253	7.4406	7.3718	7.6280	7.3255	7.4527	7.3814	7.6553	7.1620	7.2929	7.3792	7.3253	7.4406	7.3718	7.6280	7.3255	7.4527	7.3814	7.6554	**7.06**
**H5**	7.0215	7.1808	7.2686	7.2526	7.3763	7.2956	7.5610	7.2461	7.3794	7.3122	7.5916	7.0215	7.1808	7.2686	7.2526	7.3763	7.2956	7.5610	7.2461	7.3794	7.3122	7.5915	**7.06**
**H6**	7.8520	8.0113	8.0781	8.0933	8.2078	8.1366	8.4230	8.0872	8.2227	8.1523	8.4588	7.8520	8.0113	8.0781	8.0933	8.2078	8.1366	8.4230	8.0872	8.2227	8.1523	8.4588	**8.35**
**H8**	4.9708	4.9960	4.9983	5.0252	5.0872	5.0703	5.2706	5.0230	5.0792	5.0829	5.2755	4.9709	4.9960	4.9983	5.0252	5.0872	5.0703	5.2706	5.0230	5.0792	5.0829	5.2755	**4.88**
**H(9A-9B)**	1.7560	1.7720	1.7722	1.7894	1.8023	1.8367	1.9811	1.7910	1.8154	1.8445	2.0196	1.7560	1.7720	1.7722	1.7894	1.8023	1.8367	1.9811	1.7910	1.8154	1.8445	2.0196	**2.05**
**H(10A-10B)**	1.4824	1.3795	1.3272	1.3243	1.2591	1.3750	1.4434	1.3332	1.2740	1.3768	1.4433	1.4824	1.3795	1.3272	1.3243	1.2591	1.3749	1.4433	1.3332	1.2740	1.3768	1.4433	**1.36**
**H(11A-11C)**	0.7919	0.7932	0.7650	0.7981	0.7705	0.8451	0.9397	0.7996	0.7702	0.8538	0.9453	0.7919	0.7932	0.7650	0.7981	0.7705	0.8451	0.9397	0.7996	0.7702	0.8538	0.9453	**0.77**
**H(1`A)**	2.8436	2.8449	2.8146	2.8300	2.7849	2.8784	2.9602	2.8344	2.7940	2.8842	2.9459	2.9651	2.9764	2.9648	2.9564	2.9333	3.0052	3.1045	2.9614	2.9423	3.0104	3.0765	**3.03**
**H(1`B)**	3.3529	3.3857	3.4292	3.4166	3.4920	3.4617	3.6409	3.4143	3.4686	3.4742	3.6522	3.0272	2.8489	2.8478	2.8158	2.8289	2.8634	2.9652	2.8187	2.8098	2.8710	2.9711	**3.23**
**H(2`-3`A-2`-3`B)**	1.7331	1.7967	1.7585	1.8321	1.7907	1.8770	1.9768	1.8294	1.8035	1.8898	2.0158	1.7331	1.7967	1.7585	1.8321	1.7907	1.8769	1.9768	1.8294	1.8035	1.8898	2.0158	**1.77**
**H(4`A)**	3.0272	2.8489	2.8478	2.8158	2.8289	2.8634	2.9652	2.8187	2.8098	2.8710	2.9711	3.3529	3.3857	3.4292	3.4166	3.4920	3.4617	3.6409	3.4143	3.4686	3.4742	3.6522	**3.03**
**H(4`B)**	2.9651	2.9764	2.9648	2.9564	2.9333	3.0052	3.1045	2.9614	2.9423	3.0104	3.0765	2.8436	2.8449	2.8146	2.8300	2.7849	2.8784	2.9602	2.8344	2.7940	2.8842	2.9460	**3.23**
Nuclei	**BMK**																						
**C1**	154.6534	155.3770	156.2661	155.4845	157.3071	155.4687	156.4807	155.6633	156.4569	155.6605	156.6331	154.6534	155.3770	156.2661	155.4846	157.3071	155.4687	156.4808	155.6633	156.4571	155.6605	156.6332	**133.8**
**C2**	155.3955	155.2632	155.9723	155.0000	155.7846	155.0032	156.0295	155.2173	155.9035	155.1585	155.9990	155.3955	155.2632	155.9723	155.0000	155.7845	155.0032	156.0293	155.2173	155.9035	155.1585	155.9990	**132.1**
**C3**	135.7205	136.4086	137.1308	136.6235	137.5562	136.5972	137.7878	136.7821	137.6260	136.8093	137.8595	135.7205	136.4086	137.1307	136.6234	137.5561	136.5972	137.7877	136.7821	137.6260	136.8093	137.8595	**115.8**
**C4**	189.5429	190.1910	190.9701	190.5526	191.4611	190.5142	192.0687	190.6869	191.8115	190.7506	192.1784	189.5429	190.1910	190.9700	190.5526	191.4611	190.5142	192.0687	190.6869	191.8114	190.7506	192.1784	**165.8**
**C5**	133.4889	134.8487	135.6048	135.5347	136.6482	135.4775	136.8252	135.6314	136.6085	135.7504	136.9992	133.4889	134.8487	135.6048	135.5347	136.6481	135.4775	136.8251	135.6314	136.6083	135.7504	136.9992	**115.8**
**C6**	151.2332	153.2462	154.6976	154.4747	156.4940	154.3799	157.1556	154.4968	156.7451	154.7267	157.5988	151.2332	153.2462	154.6975	154.4747	156.4940	154.3799	157.1557	154.4968	156.7454	154.7267	157.5988	**132.1**
**C7**	218.2075	223.1111	224.4657	225.7578	226.4957	225.5858	232.4885	225.6243	230.9674	226.0818	233.6134	218.2075	223.1111	224.4657	225.7578	226.4957	225.5858	232.4889	225.6243	230.9674	226.0818	233.6134	**198.9**
**C8**	71.9552	73.1264	73.9248	73.6017	74.6842	73.5600	75.3098	73.7290	75.0506	73.8024	75.6986	71.9552	73.1264	73.9249	73.6017	74.6842	73.5600	75.3097	73.7290	75.0506	73.8024	75.6986	**69.3**
**C9**	40.3028	40.3891	40.8734	40.3098	41.1107	40.3034	41.1837	40.5077	40.9816	40.4769	41.3861	40.3028	40.3891	40.8733	40.3098	41.1107	40.3034	41.1838	40.5077	40.9817	40.4769	41.3861	**31.5**
**C10**	21.6716	21.8649	22.1771	22.0249	22.6919	21.9982	22.4908	22.1830	22.3842	22.2121	22.5292	21.6716	21.8649	22.1771	22.0249	22.6919	21.9982	22.4909	22.1830	22.3843	22.2121	22.5292	**19.9**
**C11**	17.3061	17.3558	17.5267	17.3626	17.7488	17.3459	17.6638	17.5405	17.5702	17.5402	17.8322	17.3061	17.3558	17.5267	17.3627	17.7488	17.3459	17.6637	17.5405	17.5701	17.5402	17.8322	**14.3**
**C1`**	56.1424	56.8316	57.0621	57.1837	57.7854	57.1449	57.6152	57.3174	57.5088	57.3823	57.8793	60.6063	61.1573	61.6448	61.5063	62.1935	61.4670	62.2916	61.6396	62.1398	61.7055	62.4755	**50.6**
**C2`**	27.9938	28.1017	28.4391	28.2214	28.7603	28.1972	28.7515	28.3843	28.6356	28.4061	28.6534	27.3938	27.4030	27.7553	27.4792	28.0904	27.4554	27.9646	27.6433	27.8760	27.6637	27.9093	**23.8**
**C3`**	27.3938	27.4030	27.7552	27.4792	28.0903	27.4554	27.9647	27.6433	27.8760	27.6637	27.9093	27.9938	28.1017	28.4390	28.2213	28.7602	28.1972	28.7515	28.3843	28.6357	28.4061	28.6534	**23.8**
**C4`**	60.6063	61.1573	61.6448	61.5064	62.1937	61.4670	62.2914	61.6396	62.1399	61.7055	62.4755	56.1424	56.8316	57.0621	57.1838	57.7853	57.1449	57.6152	57.3174	57.5088	57.3823	57.8793	**50.6**
**H2**	9.0281	9.0099	9.0311	8.9772	8.9787	8.9761	8.9822	8.9750	8.9942	8.9687	8.9805	9.0281	9.0099	9.0311	8.9772	8.9787	8.9761	8.9822	8.9750	8.9941	8.9687	8.9805	**8.35**
**H3**	7.6171	7.7670	7.8548	7.8236	7.9447	7.8175	7.9730	7.8114	7.9625	7.8197	7.9954	7.6171	7.7670	7.8548	7.8236	7.9447	7.8175	7.9730	7.8114	7.9625	7.8197	7.9954	**7.06**
**H5**	7.4753	7.6779	7.7714	7.7700	7.9009	7.7615	7.9155	7.7530	7.9049	7.7685	7.9448	7.4753	7.6779	7.7714	7.7700	7.9008	7.7615	7.9155	7.7530	7.9049	7.7685	7.9448	**7.06**
**H6**	8.4133	8.5826	8.6802	8.6993	8.8327	8.6883	8.8889	8.6774	8.8682	8.7002	8.9186	8.4133	8.5826	8.6801	8.6993	8.8327	8.6883	8.8889	8.6774	8.8682	8.7002	8.9186	**8.35**
**H8**	4.4185	4.4927	4.5162	4.5629	4.6190	4.5545	4.6848	4.5462	4.6656	4.5615	4.6923	4.4185	4.4927	4.5162	4.5629	4.6190	4.5545	4.6848	4.5462	4.6656	4.5615	4.6923	**4.88**
**H(9A-9B)**	1.5971	1.6659	1.6759	1.7171	1.7479	1.7108	1.7421	1.7046	1.7346	1.7135	1.7968	1.5971	1.6659	1.6759	1.7171	1.7479	1.7108	1.7421	1.7046	1.7347	1.7135	1.7968	**2.05**
**H(10A-10B)**	1.3590	1.2180	1.1675	1.1566	1.0821	1.1572	1.0949	1.1579	1.1006	1.1464	1.0896	1.3590	1.2180	1.1674	1.1566	1.0821	1.1572	1.0950	1.1579	1.1006	1.1464	1.0896	**1.36**
**H(11A-11C)**	0.7078	0.7018	0.6697	0.7167	0.6735	0.7124	0.6766	0.7082	0.6783	0.7113	0.6762	0.7078	0.7018	0.6697	0.7167	0.6735	0.7124	0.6766	0.7082	0.6783	0.7113	0.6762	**0.77**
**H(1`A)**	2.6301	2.6339	2.6096	2.6281	2.5782	2.6256	2.5917	2.6230	2.5932	2.6210	2.5734	2.7678	2.7734	2.7516	2.7599	2.7104	2.7579	2.7352	2.7559	2.7374	2.7522	2.7047	**3.03**
**H(1`B)**	3.0373	3.0825	3.1037	3.1266	3.1785	3.1207	3.1629	3.1147	3.1598	3.1227	3.1748	2.7095	2.6167	2.5903	2.5819	2.5835	2.5809	2.5512	2.5798	2.5522	2.5733	2.5453	**3.23**
**H(2`-3`A-2`-3`B)**	1.6204	1.6842	1.6398	1.7230	1.6644	1.7172	1.6860	1.7116	1.6825	1.7189	1.7056	1.6204	1.6842	1.6398	1.7230	1.6644	1.7172	1.6860	1.7116	1.6825	1.7189	1.7056	**1.77**
**H(4`A)**	2.7095	2.6167	2.5903	2.5819	2.5835	2.5809	2.5512	2.5798	2.5522	2.5733	2.5453	3.0373	3.0825	3.1037	3.1266	3.1785	3.1207	3.1629	3.1147	3.1598	3.1227	3.1748	**3.03**
**H(4`B)**	2.7678	2.7734	2.7516	2.7599	2.7104	2.7579	2.7353	2.7559	2.7374	2.7522	2.7047	2.6301	2.6339	2.6096	2.6281	2.5782	2.6256	2.5917	2.6230	2.5932	2.6210	2.5734	**3.23**

[a] methyl protons shifts are averaged

[b] according to [4]

Table S4. Cont. V

Nuclei	R											S											Exp. [b]
	BP86																						
	Gas	$CHCl_3$ (PCM)	$CHCl_3$ (SMD)	DMSO (PCM)	DMSO (SMD)	CH_3OH (PCM)	CH_3OH (SMD)	C_2H_5OH (PCM)	C_2H_5OH (SMD)	H_2O (PCM)	H_2O (SMD)	Gas	$CHCl_3$ (PCM)	$CHCl_3$ (SMD)	DMSO (PCM)	DMSO (SMD)	CH_3OH (PCM)	CH_3OH (SMD)	C_2H_5OH (PCM)	C_2H_5OH (SMD)	H_2O (PCM)	H_2O (SMD)	
**C1**	136.1786	137.4302	137.9541	138.0013	139.8617	137.9889	139.5122	137.9709	139.3793	138.0084	140.0217	136.1786	137.4302	137.9541	138.0013	139.8616	137.9889	139.5123	137.9709	139.3793	138.0085	140.0217	**133.8**
**C2**	135.9369	136.0084	136.5965	135.8244	136.3954	135.8439	136.7969	135.8582	136.7385	135.8011	136.9677	135.9369	136.0084	136.5965	135.8244	136.3953	135.8439	136.7969	135.8582	136.7385	135.8011	136.9676	**132.1**
**C3**	119.5269	120.4467	120.9587	120.6876	121.3566	120.6849	121.7048	120.6766	121.6379	120.6847	121.9711	119.5269	120.4467	120.9587	120.6876	121.3565	120.6849	121.7048	120.6766	121.6379	120.6848	121.9710	**115.8**
**C4**	174.5696	175.5902	176.2076	175.9912	176.7401	175.9767	177.2974	175.9567	177.1827	176.0000	177.5836	174.5696	175.5902	176.2076	175.9912	176.7401	175.9767	177.2974	175.9567	177.1827	176.0001	177.5836	**165.8**
**C5**	117.7171	119.1506	119.7207	119.7697	120.5353	119.7402	120.7953	119.7055	120.6965	119.7929	121.1037	117.7171	119.1506	119.7207	119.7697	120.5354	119.7402	120.7953	119.7055	120.6965	119.7929	121.1037	**115.8**
**C6**	132.3618	134.2536	135.3367	135.1946	136.5907	135.1390	137.1393	135.0789	136.9019	135.2426	137.5755	132.3618	134.2536	135.3367	135.1946	136.5909	135.1390	137.1393	135.0789	136.9019	135.2426	137.5755	**132.1**
**C7**	201.3079	205.8618	206.8684	208.2537	209.3563	208.1242	213.8087	207.9894	212.5909	208.3731	215.0760	201.3079	205.8618	206.8684	208.2537	209.3563	208.1242	213.8087	207.9894	212.5909	208.3732	215.0761	**198.9**
**C8**	68.1155	69.5553	70.3110	70.2031	71.1644	70.16753	71.9112	70.1279	71.5380	70.2338	72.4855	68.1155	69.5553	70.3110	70.2031	71.1644	70.1675	71.9112	70.1279	71.5380	70.2338	72.4856	**69.3**
**C9**	40.3458	40.2327	40.4918	39.9769	40.1549	39.9964	40.3724	40.0111	40.2032	39.9541	40.6324	40.3458	40.2327	40.4918	39.9769	40.1549	39.9964	40.3723	40.0111	40.2032	39.9540	40.6324	**31.5**
**C10**	20.2946	20.8624	21.1245	21.0746	21.4590	21.0670	21.3752	21.0543	21.3076	21.0770	21.7363	20.2946	20.8624	21.1245	21.0746	21.4590	21.0670	21.3752	21.0543	21.3076	21.0770	21.7363	**19.9**
**C11**	15.9373	16.2103	16.2545	16.2021	16.3361	16.2098	16.2955	16.2125	16.2756	16.1902	16.6361	15.9373	16.2100	16.2545	16.2021	16.3361	16.2098	16.2955	16.2125	16.2756	16.1901	16.6361	**14.3**
**C1`**	53.7507	54.6651	54.8659	55.1290	55.5805	55.1072	55.5538	55.0804	55.5136	55.1448	56.2217	58.2906	59.4655	59.7520	59.9494	60.4426	59.9283	60.6372	59.9023	60.6067	59.9648	61.0456	**50.6**
**C2`**	26.2286	26.8185	27.0524	27.0028	27.3646	26.9978	27.4093	26.9879	27.3628	27.0030	27.4314	25.8079	26.1594	26.3791	26.2600	26.7272	26.2630	26.6575	26.2604	26.6605	26.2520	26.6671	**23.8**
**C3`**	25.8079	26.1594	26.3791	26.2600	26.7272	26.2630	26.6576	26.2604	26.6605	26.2519	26.6670	26.2286	26.8185	27.0524	27.0027	27.3646	26.9978	27.4093	26.9879	27.3628	27.0030	27.4315	**23.8**
**C4`**	58.2906	59.4655	59.7520	59.9494	60.4426	59.9283	60.6372	59.9023	60.6067	59.9648	61.0455	53.7507	54.6651	54.8659	55.1290	55.5805	55.1072	55.5537	55.0804	55.5134	55.1448	56.2218	**50.6**
**H2**	8.5222	8.4073	8.4412	8.3257	8.2874	8.3303	8.2897	8.3345	8.3026	8.3211	8.2924	8.5222	8.4073	8.4412	8.3257	8.2874	8.3303	8.2897	8.3345	8.3026	8.3211	8.2924	**8.35**
**H3**	7.1721	7.3156	7.3926	7.3613	7.4701	7.3599	7.4869	7.3582	7.4789	7.3621	7.5176	7.1721	7.3156	7.3926	7.3613	7.4701	7.3599	7.4869	7.3582	7.4789	7.3621	7.5176	**7.06**
**H5**	7.0215	7.2046	7.2873	7.2785	7.3923	7.2749	7.4064	7.2710	7.3950	7.2815	7.4377	7.0215	7.2046	7.2873	7.2785	7.3923	7.2749	7.4064	7.2710	7.3950	7.2815	7.4377	**7.06**
**H6**	7.9345	8.0936	8.1585	8.1734	8.2683	8.1689	8.3003	8.1642	8.2934	8.1772	8.3329	7.9345	8.0936	8.1585	8.1734	8.2683	8.1689	8.3003	8.1642	8.2934	8.1772	8.3329	**8.35**
**H8**	4.9786	5.0072	4.9965	5.0223	5.0748	5.0222	5.0960	5.0218	5.0943	5.0220	5.1034	4.9786	5.0072	4.9965	5.0223	5.0748	5.0222	5.0960	5.0218	5.0943	5.0220	5.1034	**4.88**
**H(9A-9B)**	1.8488	1.8885	1.8978	1.9030	1.8797	1.9015	1.8704	1.8998	1.8573	1.9042	1.9128	1.8488	1.8885	1.8978	1.9030	1.8797	1.9015	1.8704	1.8998	1.8573	1.9042	1.9128	**2.05**
**H(10A-10B)**	1.4788	1.3707	1.3093	1.3055	1.2278	1.3108	1.2423	1.3156	1.2507	1.3001	1.2427	1.4788	1.3707	1.3093	1.3055	1.2278	1.3108	1.2423	1.3156	1.2507	1.3001	1.2427	**1.36**
**H(11A-11C)**	0.7611	0.7670	0.7306	0.7691	0.7268	0.7696	0.7253	0.7699	0.7295	0.7683	0.7306	0.7611	0.7670	0.7306	0.7691	0.7268	0.7696	0.7253	0.7699	0.7295	0.7683	0.7306	**0.77**
**H(1`A)**	2.7940	2.8077	2.7772	2.7956	2.7436	2.7973	2.7499	2.7988	2.7481	2.7935	2.7342	2.9160	2.9279	2.9077	2.9136	2.8800	2.9153	2.8907	2.9169	2.8878	2.9115	2.8660	**3.03**
**H(1`B)**	3.3743	3.4038	3.4287	3.4274	3.4813	3.4263	3.4567	3.4250	3.4547	3.4281	3.4648	3.0722	2.8996	2.8654	2.8446	2.8443	2.8486	2.8084	2.8523	2.7966	2.8406	2.8085	**3.23**
**H(2`-3`A-2`-3`B)**	1.7277	1.7852	1.7366	1.8209	1.7709	1.8195	1.7901	1.8179	1.7869	1.8218	1.8340	1.7277	1.7852	1.7366	1.8209	1.7709	1.8195	1.7901	1.8179	1.7869	1.8218	1.8340	**1.77**
**H(4`A)**	3.0722	2.8996	2.8654	2.8446	2.8443	2.8486	2.8083	2.8523	2.7966	2.8406	2.8085	3.3743	3.4038	3.4287	3.4274	3.4813	3.4263	3.4567	3.4249	3.4547	3.4281	3.4648	**3.03**
**H(4`B)**	2.9160	2.9279	2.9077	2.9135	2.8800	2.9153	2.8907	2.9169	2.8878	2.9115	2.8660	2.7940	2.8077	2.7772	2.7956	2.7436	2.7973	2.7499	2.7987	2.7481	2.7935	2.7342	**3.23**
Nuclei	CAM-B3LYP																						
**C1**	140.9266	141.9077	142.5773	142.5860	144.7771	142.5347	144.2091	142.4721	144.1884	142.6310	144.5927	140.9266	141.9077	142.5773	142.5860	144.7771	142.5347	144.2091	142.4721	144.1884	142.6311	144.5927	**133.8**
**C2**	142.4301	142.3674	142.8646	142.2220	142.7350	142.2354	143.1452	142.2494	143.0878	142.2115	143.1534	142.4302	142.3674	142.8647	142.2220	142.7350	142.2355	143.1452	142.2494	143.0878	142.2115	143.1534	**132.1**
**C3**	123.8296	124.5019	125.0076	124.7106	125.4095	124.7045	125.6498	124.6974	125.5900	124.7176	125.7386	123.8297	124.5019	125.0076	124.7106	125.4095	124.7045	125.6498	124.6974	125.5900	124.7176	125.7386	**115.8**
**C4**	177.7516	178.4670	179.0444	178.8161	179.4341	178.7999	180.0116	178.7844	179.8802	178.8335	180.1274	177.7516	178.4670	179.0445	178.8160	179.4341	178.7999	180.0116	178.7844	179.8802	178.8334	180.1274	**165.8**
**C5**	121.8071	123.1309	123.6904	123.7994	124.6335	123.7627	124.8019	123.7254	124.7039	123.8356	124.9632	121.8071	123.1309	123.6903	123.7994	124.6335	123.7627	124.8019	123.7254	124.7039	123.8356	124.9632	**115.8**
**C6**	138.5574	140.4016	141.5123	141.3473	142.6341	141.2957	143.1895	141.2504	142.9402	141.4012	143.4541	138.5573	140.4016	141.5124	141.3473	142.6341	141.2957	143.1895	141.2504	142.9402	141.4012	143.4541	**132.1**
**C7**	206.2278	211.3109	212.3833	214.3098	215.3800	214.1127	221.0719	213.9048	219.6673	214.4947	222.2973	206.2279	211.3109	212.3833	214.3098	215.3800	214.1126	221.0719	213.9048	219.6673	214.4946	222.2973	**198.9**
**C8**	64.5921	65.7432	66.3737	66.4216	67.3595	66.3724	68.0155	66.3227	67.7673	66.4708	68.4288	64.5920	65.7432	66.3736	66.4216	67.3595	66.3724	68.0155	66.3227	67.7673	66.4709	68.4288	**69.3**
**C9**	36.9478	36.6632	36.8498	36.3985	36.6277	36.4198	36.6461	36.4449	36.5829	36.3826	36.6973	36.9479	36.6632	36.8498	36.3985	36.6277	36.4198	36.6461	36.4449	36.5829	36.3826	36.6973	**31.5**
**C10**	18.7015	19.1547	19.3875	19.3798	19.8399	19.3691	19.7280	19.3600	19.6927	19.3928	19.8728	18.7015	19.1547	19.3877	19.3798	19.8399	19.3691	19.7280	19.3600	19.6927	19.3928	19.8728	**19.9**
**C11**	15.6830	15.8449	15.9294	15.8751	16.1201	15.8770	16.0640	15.8796	16.0619	15.8757	16.2245	15.6831	15.8449	15.9294	15.8751	16.1201	15.8770	16.0640	15.8796	16.0619	15.8757	16.2245	**14.3**
**C1`**	50.8424	51.6920	51.9176	52.2185	52.7669	52.1897	52.7350	52.1604	52.6956	52.2476	53.2350	55.1496	56.0994	56.4320	56.6051	57.1606	56.5753	57.2952	56.5453	57.2367	56.6353	57.5631	**50.6**
**C2`**	25.0081	25.3772	25.6412	25.5596	25.9829	25.5500	26.0119	25.5411	25.9771	25.5711	25.8584	24.4589	24.6447	24.9269	24.7551	25.2974	24.7521	25.2056	24.7489	25.2002	24.7595	25.0505	**23.8**
**C3`**	24.4590	24.6447	24.9269	24.7551	25.2974	24.7521	25.2056	24.7489	25.2002	24.7595	25.0505	25.0080	25.3772	25.6413	25.5596	25.9829	25.5500	26.0119	25.5411	25.9771	25.5711	25.8584	**23.8**
**C4`**	55.1496	56.0994	56.4319	56.6051	57.1606	56.5753	57.2952	56.5453	57.2367	56.6353	57.5631	50.8424	51.6920	51.9176	52.2185	52.7669	52.1897	52.7350	52.1604	52.6956	52.2475	53.2350	**50.6**
**H2**	8.8110	8.6933	8.7294	8.5958	8.5502	8.6033	8.5477	8.6122	8.5611	8.5891	8.5304	8.8110	8.6933	8.7294	8.5958	8.5502	8.6033	8.5477	8.6122	8.5611	8.5891	8.5304	**8.35**
**H3**	7.4020	7.5341	7.6236	7.5769	7.6964	7.5750	7.7136	7.5731	7.7065	7.5786	7.7287	7.4020	7.5341	7.6236	7.5769	7.6964	7.5750	7.7136	7.5731	7.7065	7.5787	7.7287	**7.06**
**H5**	7.2447	7.4318	7.5220	7.5115	7.6341	7.5072	7.6476	7.5030	7.6363	7.5156	7.6673	7.2447	7.4318	7.5220	7.5115	7.6341	7.5072	7.6476	7.5030	7.6363	7.5156	7.6673	**7.06**
**H6**	8.0643	8.2435	8.3388	8.3319	8.4473	8.3272	8.4891	8.3231	8.4735	8.3365	8.5103	8.0643	8.2435	8.3388	8.3319	8.4473	8.3272	8.4891	8.3231	8.4735	8.3365	8.5103	**8.35**
**H8**	4.4117	4.4875	4.5224	4.5293	4.6167	4.5270	4.6653	4.5250	4.6500	4.5314	4.6575	4.4117	4.4875	4.5223	4.5293	4.6167	4.5270	4.6653	4.5250	4.6500	4.5314	4.6575	**4.88**
**H(9A-9B)**	1.6498	1.6930	1.6991	1.7084	1.6888	1.7077	1.6717	1.7081	1.6678	1.7093	1.6955	1.6498	1.6930	1.6992	1.7084	1.6888	1.7077	1.6717	1.7081	1.6678	1.7093	1.6955	**2.05**
**H(10A-10B)**	1.3768	1.2583	1.2118	1.1889	1.1200	1.1937	1.1412	1.1987	1.1473	1.1841	1.1304	1.3768	1.2583	1.2118	1.1889	1.1200	1.1937	1.1412	1.1987	1.1473	1.1841	1.1304	**1.36**
**H(11A-11C)**	0.7876	0.7903	0.7657	0.7934	0.7565	0.7934	0.7583	0.7936	0.7608	0.7933	0.7499	0.7876	0.7903	0.7657	0.7934	0.7565	0.7934	0.7583	0.7936	0.7608	0.7933	0.7499	**0.77**
**H(1`A)**	2.7019	2.7060	2.6841	2.6921	2.6466	2.6933	2.6608	2.6946	2.6608	2.6910	2.6344	2.8328	2.8275	2.8147	2.8678	2.7833	2.8142	2.7969	2.8152	2.7949	2.8123	2.6344	**3.03**
**H(1`B)**	3.1157	3.1616	3.1954	3.1924	3.2559	3.1904	3.2396	3.1887	3.2361	3.1944	3.2401	2.8021	2.6732	2.6438	2.6969	2.6568	2.6379	2.6216	2.6393	2.6170	2.6352	3.2401	**3.23**
**H(2`-3`A-2`-3`B)**	1.6571	1.7174	1.6802	1.7585	1.7196	1.7559	1.7423	1.7532	1.7374	1.7609	1.7694	1.6571	1.7174	1.6802	1.7585	1.7196	1.7559	1.7423	1.7532	1.7374	1.7609	1.7694	**1.77**
**H(4`A)**	2.8021	2.6732	2.6438	2.6365	2.6568	2.6379	2.6216	2.6393	2.6170	2.6352	2.6152	3.1157	3.1616	3.1954	3.2485	3.2559	3.1904	3.2396	3.1887	3.2361	3.1944	2.6152	**3.03**
**H(4`B)**	2.8328	2.8275	2.8147	2.8132	2.7833	2.8142	2.7969	2.8152	2.7949	2.8123	2.7637	2.7018	2.7060	2.6841	2.7372	2.6466	2.6933	2.6608	2.6946	2.6608	2.6910	2.7637	**3.23**

[a] methyl protons shifts are averaged

[b] according to [4]

Table S4. Cont. VI

Nuclei	R											S											Exp. [b]
	HF																						
	Gas	$CHCl_3$ (PCM)	$CHCl_3$ (SMD)	DMSO (PCM)	DMSO (SMD)	CH_3OH (PCM)	CH_3OH (SMD)	C_2H_5OH (PCM)	C_2H_5OH (SMD)	H_2O (PCM)	H_2O (SMD)	Gas	$CHCl_3$ (PCM)	$CHCl_3$ (SMD)	DMSO (PCM)	DMSO (SMD)	CH_3OH (PCM)	CH_3OH (SMD)	C_2H_5OH (PCM)	C_2H_5OH (SMD)	H_2O (PCM)	H_2O (SMD)	
**C1**	136.4414	137.5014	138.4538	138.1924	139.8150	138.1346	138.8330	138.0750	138.9018	138.2468	139.1504	136.4413	137.5014	138.4538	138.1924	139.8150	138.1346	138.8330	138.0750	138.9018	138.2467	139.1504	**133.8**
**C2**	144.4173	144.1728	144.6114	143.8967	144.2996	143.9185	144.8354	143.9409	144.7479	143.8757	144.8310	144.4173	144.1728	144.6114	143.8966	144.2996	143.9185	144.8354	143.9410	144.7479	143.8756	144.8310	**132.1**
**C3**	120.4744	121.0412	121.5064	121.1750	121.7383	121.1678	121.7760	121.1606	121.7557	121.1812	121.8450	120.4744	121.0412	121.5064	121.1750	121.7383	121.1678	121.7758	121.1606	121.7557	121.1811	121.8450	**115.8**
**C4**	171.1128	171.6055	172.1010	171.8031	172.3125	171.7947	173.0265	171.7874	172.8557	171.8108	173.1462	171.1128	171.6055	172.1010	171.8031	172.3125	171.7947	173.0265	171.7874	172.8556	171.8109	173.1462	**165.8**
**C5**	117.8496	119.1488	119.7030	119.7919	120.5418	119.7519	120.5591	119.7121	120.4895	119.8285	120.7326	117.8497	119.1488	119.7030	119.7919	120.5418	119.7519	120.5591	119.7121	120.4895	119.8286	120.7326	**115.8**
**C6**	140.0136	141.4903	142.3589	142.3541	143.6752	142.3088	144.4959	142.2651	144.2248	142.3962	144.7347	140.0135	141.4903	142.3589	142.3541	143.6752	142.3088	144.4962	142.2651	144.2248	142.3963	144.7347	**132.1**
**C7**	196.4495	202.1767	203.4250	205.4842	205.5403	205.2643	212.4997	205.0429	210.7934	205.6921	213.6871	196.4499	202.1767	203.4250	205.4842	205.5403	205.2643	212.4997	205.0433	210.7933	205.6921	213.6871	**198.9**
**C8**	55.7145	56.6022	56.9914	57.2558	57.7427	57.2041	58.5143	57.1519	58.3197	57.3033	58.7602	55.7144	56.6022	56.9914	57.2558	57.7427	57.2041	58.5142	57.1519	58.3197	57.3033	58.7602	**69.3**
**C9**	31.1481	30.5519	30.6807	30.3496	30.5631	30.3610	30.5462	30.3731	30.5463	30.3374	30.5372	31.1481	30.5519	30.6807	30.3496	30.5631	30.3610	30.5462	30.3731	30.5463	30.3374	30.5372	**31.5**
**C10**	16.7291	17.0912	17.3419	17.3136	17.7681	17.3011	17.6649	17.2889	17.6541	17.3243	17.7400	16.7292	17.0912	17.3419	17.3136	17.7681	17.3011	17.6649	17.2889	17.6541	17.3243	17.7400	**19.9**
**C11**	15.7593	15.8051	15.9437	15.8378	16.0843	15.8359	16.0348	15.8345	16.0336	15.8389	16.1539	15.7593	15.8051	15.9437	15.8378	16.0843	15.8359	16.0347	15.8345	16.0337	15.8389	16.1539	**14.3**
**C1`**	44.6427	45.3307	45.6608	45.8037	46.3013	45.7713	46.3321	45.7393	46.3006	45.8335	46.6765	48.5403	49.4088	49.6458	49.8163	50.2686	49.7920	50.3671	49.7682	50.3017	49.8388	50.6134	**50.6**
**C2`**	22.8166	23.0703	23.2952	23.1618	23.5813	23.1542	23.5742	23.1472	23.5602	23.1685	23.4000	22.2001	22.2972	22.6394	22.3564	22.9166	22.3503	22.7594	22.3451	22.7610	22.3622	22.5826	**23.8**
**C3`**	22.2001	22.2972	22.6394	22.3564	22.9166	22.3503	22.7594	22.3451	22.7610	22.3623	22.5826	22.8167	23.0703	23.2952	23.1618	23.5813	23.1542	23.5741	23.1472	23.5603	23.1686	23.4000	**23.8**
**C4`**	48.5403	49.4088	49.6459	49.8163	50.2686	49.7920	50.3671	49.7682	50.3017	49.8388	50.6134	44.6427	45.3307	45.6608	45.8037	46.3013	45.7713	46.3320	45.7393	46.3005	45.8336	46.6765	**50.6**
**H2**	9.0786	8.9268	8.9429	8.8084	8.8374	8.8178	8.8458	8.8273	8.8538	8.7996	8.8299	9.0786	8.9268	8.9429	8.8084	8.8374	8.8178	8.8458	8.8273	8.8538	8.7996	8.8299	**8.35**
**H3**	7.4354	7.5749	7.6671	7.6176	7.7395	7.6155	7.7519	7.6132	7.7449	7.6195	7.7676	7.4354	7.5749	7.6671	7.6176	7.7395	7.6155	7.7519	7.6132	7.7449	7.6195	7.7676	**7.06**
**H5**	7.2468	7.4451	7.5351	7.5315	7.6597	7.5266	7.6705	7.5215	7.6586	7.5362	7.6954	7.2468	7.4451	7.5351	7.5315	7.6597	7.5266	7.6705	7.5215	7.6586	7.5362	7.6954	**7.06**
**H6**	8.1005	8.3206	8.4139	8.4235	8.5927	8.4182	8.6446	8.4128	8.6190	8.4287	8.6771	8.1005	8.3206	8.4139	8.4235	8.5927	8.4182	8.6446	8.4128	8.6190	8.4287	8.6771	**8.35**
**H8**	3.6317	3.7654	3.8157	3.8310	3.9676	3.8272	4.0131	3.8234	3.9840	3.8350	4.0129	3.6316	3.7654	3.8157	3.8310	3.9676	3.8272	4.0131	3.8234	3.9840	3.8350	4.0129	**4.88**
**H(9A-9B)**	1.3010	1.3077	1.2916	1.3310	1.3204	1.3300	1.3347	1.3288	1.3337	1.3319	1.3499	1.3011	1.3077	1.2916	1.3310	1.3204	1.3300	1.3347	1.3288	1.3337	1.3319	1.3499	**2.05**
**H(10A-10B)**	1.2641	1.1347	1.1014	1.0575	1.0030	1.0626	1.0110	1.0677	1.0121	1.0529	1.0039	1.2641	1.1347	1.1014	1.0575	1.0030	1.0626	1.0110	1.0677	1.0121	1.0529	1.0039	**1.36**
**H(11A-11C)**	0.7647	0.7639	0.7366	0.7661	0.7356	0.7661	0.7378	0.7660	0.7370	0.7664	0.7334	0.7647	0.7639	0.7366	0.7661	0.7356	0.7661	0.7378	0.7660	0.7369	0.7664	0.7334	**0.77**
**H(1`A)**	2.3641	2.3616	2.3365	2.3490	2.2936	2.3501	2.3189	2.3510	2.3168	2.3479	2.3034	2.5251	2.5114	2.4900	2.4891	3.2500	2.4901	2.4669	2.4909	2.4667	2.4882	2.4403	**3.03**
**H(1`B)**	2.7108	2.7752	2.8023	2.8177	2.8790	2.8150	2.8770	2.8121	2.8700	2.8205	2.8850	2.3756	2.2648	2.2718	2.2718	3.1235	2.2702	2.2526	2.2682	2.2516	2.2734	2.2573	**3.23**
**H(2`-3`A-2`-3`B)**	1.4360	1.5100	1.4737	1.5555	1.5080	1.5525	1.5289	1.5493	1.5214	1.5585	1.5547	1.4360	1.5100	1.4737	1.5555	1.5080	1.5525	1.5289	1.5493	1.5214	1.5585	1.5547	**1.77**
**H(4`A)**	2.3756	2.2648	2.2718	2.2718	2.2686	2.2702	2.2526	2.2682	2.2516	2.2734	2.2573	2.7108	2.7752	2.8023	2.8177	3.7226	2.8150	2.8770	2.8121	2.8699	2.8205	2.8850	**3.03**
**H(4`B)**	2.5251	2.5114	2.4900	2.4891	2.4394	2.4901	2.4669	2.4909	2.4667	2.4882	2.4403	2.3641	2.3616	2.3365	2.3490	3.1133	2.3501	2.3189	2.3510	2.3168	2.3479	2.3034	**3.23**
Nuclei	LC-BLYP																						
**C1**	144.3308	145.4927	145.9944	145.9621	147.5542	145.9028	147.5626	145.8745	147.6800	146.0056	147.9719	144.3308	145.4927	145.9944	145.9622	147.5543	145.9028	147.5626	145.8746	147.6800	146.0056	147.9719	**133.8**
**C2**	148.6152	148.4676	149.0028	148.3230	148.8404	148.3132	149.2150	148.3305	149.1034	148.3145	149.1965	148.6152	148.4676	149.0029	148.3231	148.8407	148.3134	149.2150	148.3305	149.1034	148.3144	149.1965	**132.1**
**C3**	128.3178	128.9172	129.5180	129.1317	129.8023	129.0994	130.0812	129.0944	130.0300	129.1448	130.1620	128.3178	128.9172	129.5179	129.1316	129.8022	129.0994	130.0812	129.0944	130.0300	129.1448	130.1620	**115.8**
**C4**	181.9593	182.4590	183.1240	182.8206	183.3714	182.7772	183.9613	182.7607	183.7961	182.8441	184.0537	181.9593	182.4589	183.1240	182.8206	183.3713	182.7771	183.9613	182.7607	183.7961	182.8441	184.0537	**165.8**
**C5**	126.1620	127.4795	128.1520	128.2178	129.0591	128.1494	129.3479	128.1085	129.2485	128.2663	129.5150	126.1620	127.4795	128.1521	128.2178	129.0590	128.1492	129.3479	128.1085	129.2485	128.2664	129.5150	**115.8**
**C6**	144.4002	146.1413	147.4579	147.2869	148.8967	147.1981	149.0477	147.1351	148.7769	147.3519	149.3759	144.4002	146.1413	147.4579	147.2869	148.8966	147.1981	149.0477	147.1351	148.7769	147.3519	149.3759	**132.1**
**C7**	211.8618	217.4555	218.3176	220.4747	220.8255	220.2563	227.5439	220.0688	226.2474	220.6703	228.8976	211.8618	217.4554	218.3177	220.4747	220.8259	220.2563	227.5439	220.0688	226.2474	220.6702	228.8976	**198.9**
**C8**	62.4870	63.7764	64.1570	64.4288	65.1947	64.3660	65.8352	64.3316	65.6756	64.4723	66.4294	62.4870	63.7763	64.1570	64.4288	65.1947	64.3660	65.8352	64.3316	65.6756	64.4724	66.4294	**69.3**
**C9**	34.7784	34.4106	34.6278	34.2412	34.6042	34.2354	34.3458	34.2557	34.3082	34.2282	34.4958	34.7784	34.4106	34.6278	34.2412	34.6042	34.2354	34.3458	34.2557	34.3082	34.2283	34.4958	**31.5**
**C10**	16.5821	16.9598	17.2502	17.2282	17.7410	17.1911	17.5454	17.1812	17.5103	17.2458	17.7072	16.5821	16.9599	17.2502	17.2282	17.7410	17.1911	17.5454	17.1812	17.5103	17.2458	17.7072	**19.9**
**C11**	14.7041	14.7757	14.9360	14.8361	15.0888	14.8116	15.0340	14.8143	15.0234	14.8420	15.1917	14.7041	14.7758	14.9361	14.8361	15.0890	14.8115	15.0340	14.8143	15.0234	14.8421	15.1917	**14.3**
**C1`**	48.8010	49.6372	49.8785	50.1433	50.7500	50.0896	50.7271	50.0633	50.6647	50.1774	51.1918	52.7051	53.6090	53.9949	54.1344	54.6609	54.0789	54.8308	54.0513	54.7470	54.1705	55.1182	**50.6**
**C2`**	23.0101	23.2872	23.5583	23.4621	23.8479	23.4292	23.8490	23.4240	23.8258	23.4762	23.7763	22.3692	22.4486	22.8208	22.5763	23.0537	22.5459	23.0058	22.5430	23.0003	22.5881	22.8821	**23.8**
**C3`**	22.3692	22.4485	22.8208	22.5763	23.0537	22.5459	23.0058	22.5430	23.0003	22.5881	22.8821	23.0101	23.2872	23.5583	23.4621	23.8479	23.4293	23.8490	23.4240	23.8258	23.4761	23.7763	**23.8**
**C4`**	52.7051	53.6090	53.9949	54.1345	54.6610	54.0790	54.8308	54.0513	54.7469	54.1704	55.1181	48.8010	49.6372	49.8785	50.1433	50.7501	50.0895	50.7271	50.0633	50.6647	50.1774	51.1918	**50.6**
**H2**	9.0106	8.8554	8.9239	8.7786	8.7921	8.7835	8.7327	8.7896	8.7301	8.7725	8.7058	9.0106	8.8554	8.9239	8.7786	8.7921	8.7835	8.7327	8.7896	8.7301	8.7725	8.7058	**8.35**
**H3**	7.5456	7.6744	7.7676	7.7156	7.8350	7.7128	7.8538	7.7115	7.8461	7.7173	7.8696	7.5456	7.6744	7.7676	7.7156	7.8350	7.7128	7.8538	7.7115	7.8461	7.7172	7.8696	**7.06**
**H5**	7.3903	7.5763	7.6702	7.6619	7.7870	7.6561	7.8016	7.6518	7.7910	7.6665	7.8251	7.3903	7.5763	7.6702	7.6619	7.7870	7.6561	7.8016	7.6518	7.7910	7.6665	7.8251	**7.06**
**H6**	8.2180	8.3832	8.5114	8.4895	8.6346	8.4823	8.6528	8.4765	8.6305	8.4952	8.6741	8.2180	8.3832	8.5114	8.4895	8.6346	8.4823	8.6528	8.4765	8.6305	8.4952	8.6741	**8.35**
**H8**	4.2014	4.2699	4.3515	4.3370	4.4203	4.3314	4.5040	4.3275	4.4802	4.3412	4.5025	4.2014	4.2699	4.3515	4.3370	4.4203	4.3314	4.5040	4.3275	4.4802	4.3412	4.5025	**4.88**
**H(9A-9B)**	1.6373	1.6762	1.6736	1.7038	1.7253	1.7020	1.6446	1.7016	1.6394	1.7042	1.6801	1.6373	1.6762	1.6736	1.7038	1.7253	1.7020	1.6446	1.7016	1.6394	1.7042	1.6801	**2.05**
**H(10A-10B)**	1.3285	1.1875	1.1641	1.1190	1.0512	1.1224	1.0751	1.1274	1.0779	1.1147	1.0602	1.3285	1.1875	1.1641	1.1190	1.0512	1.1224	1.0751	1.1274	1.0779	1.1147	1.0602	**1.36**
**H(11A-11C)**	0.7838	0.7781	0.7632	0.7853	0.7488	0.7840	0.7460	0.7842	0.7463	0.7855	0.7370	0.7838	0.7782	0.7632	0.7853	0.7488	0.7840	0.7460	0.7842	0.7463	0.7855	0.7370	**0.77**
**H(1`A)**	2.6651	2.6587	2.6432	2.6437	2.5949	2.6441	2.6105	2.6461	2.6116	2.6423	2.5894	2.7943	2.7946	2.7650	2.7789	2.7365	2.7790	2.7481	2.7808	2.7503	2.7776	2.7269	**3.03**
**H(1`B)**	3.0078	3.0469	3.0820	3.0813	3.1389	3.0782	3.1224	3.0768	3.1178	3.0832	3.1257	2.7241	2.6071	2.5460	2.5645	2.5576	2.5659	2.5280	2.5691	2.5289	2.5622	2.5276	**3.23**
**H(2`-3`A-2`-3`B)**	1.6440	1.7039	1.6686	1.7445	1.6993	1.7408	1.7320	1.7388	1.7255	1.7470	1.7576	1.6440	1.7040	1.6686	1.7445	1.6993	1.7408	1.7320	1.7388	1.7255	1.7470	1.7576	**1.77**
**H(4`A)**	2.7241	2.6071	2.5460	2.5645	2.5576	2.5659	2.5280	2.5691	2.5289	2.5622	2.5276	3.0078	3.0469	3.0820	3.0813	3.1389	3.0782	3.1224	3.0768	3.1178	3.0832	3.1257	**3.03**
**H(4`B)**	2.7943	2.7946	2.7650	2.7788	2.7365	2.7790	2.7481	2.7808	2.7503	2.7776	2.7269	2.6651	2.6587	2.6432	2.6437	2.5949	2.6441	2.6105	2.6461	2.6116	2.6423	2.5894	**3.23**

[a] methyl protons shifts are averaged

[b] according to [4]

Table S4. Cont. VII

Nuclei	R											S											Exp. [b]
	M06																						
	Gas	$CHCl_3$ (PCM)	$CHCl_3$ (SMD)	DMSO (PCM)	DMSO (SMD)	CH_3OH (PCM)	CH_3OH (SMD)	C_2H_5OH (PCM)	C_2H_5OH (SMD)	H_2O (PCM)	H_2O (SMD)	Gas	$CHCl_3$ (PCM)	$CHCl_3$ (SMD)	DMSO (PCM)	DMSO (SMD)	CH_3OH (PCM)	CH_3OH (SMD)	C_2H_5OH (PCM)	C_2H_5OH (SMD)	H_2O (PCM)	H_2O (SMD)	
**C1**	136.8587	138.6854	139.8459	139.3176	141.4728	139.2641	140.3172	139.2203	140.4496	139.3850	140.6478	136.8587	138.6852	139.8458	139.3176	141.4728	139.2641	140.3172	139.2203	140.4496	139.3850	140.6478	**133.8**
**C2**	140.5440	139.9917	140.5692	139.6770	140.2730	139.6854	140.8393	139.7016	140.7125	139.6834	140.7721	140.5440	139.9916	140.5692	139.6770	140.2730	139.6854	140.8393	139.7016	140.7125	139.6834	140.7721	**132.1**
**C3**	121.6896	122.3564	123.0312	122.5833	123.4038	122.5622	123.6315	122.5486	123.5686	122.6176	123.7108	121.6896	122.3564	123.0311	122.5833	123.4038	122.5622	123.6315	122.5486	123.5686	122.6176	123.7108	**115.8**
**C4**	173.3794	174.2348	174.9757	174.6746	175.3987	174.6411	175.9914	174.6147	175.8470	174.7204	176.0960	173.3794	174.2348	174.9756	174.6746	175.3987	174.6411	175.9914	174.6147	175.8470	174.7204	176.0960	**165.8**
**C5**	119.6442	120.8138	121.5066	121.5143	122.3936	121.4596	122.6609	121.4131	122.5634	121.5816	122.8584	119.6442	120.8140	121.5067	121.5143	122.3937	121.4596	122.6609	121.4131	122.5634	121.5816	122.8584	**115.8**
**C6**	136.6190	138.0201	139.1415	139.0588	140.5391	138.9844	141.2587	138.9172	141.0233	139.1439	141.5635	136.6190	138.0200	139.1415	139.0588	140.5392	138.9844	141.2587	138.9172	141.0233	139.1439	141.5635	**132.1**
**C7**	203.4072	209.3010	210.7623	212.2146	213.2021	212.0162	217.9477	211.8286	216.8130	212.4212	219.1293	203.4072	209.3011	210.7622	212.2146	213.2021	212.0162	217.9477	211.8286	216.8130	212.4212	219.1293	**198.9**
**C8**	61.9542	63.5485	64.3954	64.2641	65.3612	64.2028	65.7924	64.1511	65.6176	64.3389	66.2226	61.9542	63.5486	64.3954	64.2641	65.3612	64.2028	65.7924	64.1511	65.6176	64.3389	66.2226	**69.3**
**C9**	35.7877	35.3811	35.6369	35.2340	35.6454	35.2369	35.7936	35.2468	35.7234	35.2447	35.8369	35.7877	35.3812	35.6369	35.2340	35.6455	35.2369	35.7936	35.2468	35.7234	35.2447	35.8369	**31.5**
**C10**	16.7497	17.5868	18.0478	17.9439	18.6854	17.9123	18.5635	17.8885	18.5397	17.9884	18.6445	16.7498	17.5868	18.0478	17.9439	18.6854	17.9123	18.5635	17.8885	18.5397	17.9884	18.6445	**19.9**
**C11**	15.6901	15.6596	15.8843	15.6650	16.0771	15.6524	15.9933	15.6482	15.9812	15.6918	16.4023	15.6902	15.6596	15.8843	15.6650	16.0772	15.6524	15.9933	15.6482	15.9812	15.6918	16.4023	**14.3**
**C1`**	50.7374	51.5622	51.9496	52.0003	52.7195	51.9638	52.7186	51.9350	52.6681	52.0495	53.2009	55.1196	56.0449	56.4891	56.5711	57.1984	56.5293	57.4389	56.4952	57.3498	56.6253	57.8474	**50.6**
**C2`**	23.4218	23.9115	24.3247	24.0780	24.6400	24.0597	24.7117	24.0488	24.6915	24.1098	24.4498	22.2913	22.6693	23.1895	22.7996	23.4794	22.7807	23.3297	22.7700	23.3617	22.8323	22.8485	**23.8**
**C3`**	22.2912	22.6693	23.1895	22.7996	23.4794	22.7807	23.3297	22.7700	23.3617	22.8323	22.8485	23.4218	23.9115	24.3246	24.0780	24.6400	24.0597	24.7117	24.0488	24.6915	24.1098	24.4498	**23.8**
**C4`**	55.1195	56.0449	56.4891	56.5711	57.1984	56.5293	57.4389	56.4952	57.3498	56.6253	57.8474	50.7375	51.5622	51.9496	52.0003	52.7196	51.9638	52.7186	51.9350	52.6681	52.0495	53.2009	**50.6**
**H2**	8.7504	8.5336	8.5336	8.4112	8.3909	8.4199	8.4277	8.4289	8.4206	8.4030	8.3966	8.7504	8.5336	8.5337	8.4112	8.3908	8.4199	8.4277	8.4289	8.4206	8.4030	8.3966	**8.35**
**H3**	7.3165	7.4747	7.5640	7.4911	7.6085	7.4913	7.6125	7.4919	7.6043	7.4912	7.6167	7.3165	7.4747	7.5640	7.4911	7.6085	7.4913	7.6125	7.4919	7.6043	7.4912	7.6167	**7.06**
**H5**	7.1178	7.2708	7.3584	7.3474	7.4768	7.3422	7.4985	7.3378	7.4865	7.3529	7.5256	7.1178	7.2708	7.3585	7.3474	7.4768	7.3422	7.4985	7.3378	7.4865	7.3529	7.5256	**7.06**
**H6**	8.0202	8.1899	8.2795	8.3048	8.4307	8.2981	8.5111	8.2919	8.4906	8.3115	8.5520	8.0202	8.1899	8.2795	8.3048	8.4307	8.2981	8.5111	8.2919	8.4906	8.3115	8.5520	**8.35**
**H8**	4.4111	4.4204	4.4597	4.4735	4.5499	4.4693	4.6253	4.4657	4.6082	4.4782	4.6702	4.4111	4.4203	4.4597	4.4735	4.5499	4.4693	4.6253	4.4657	4.6082	4.4782	4.6702	**4.88**
**H(9A-9B)**	1.7754	1.7284	1.7011	1.7283	1.7293	1.7285	1.7393	1.7291	1.7280	1.7285	1.7670	1.7753	1.7284	1.7011	1.7283	1.7293	1.7285	1.7393	1.7291	1.7280	1.7285	1.7670	**2.05**
**H(10A-10B)**	1.3278	1.1916	1.1442	1.1282	1.0765	1.1321	1.0962	1.1365	1.0963	1.1251	1.0944	1.3278	1.1916	1.1442	1.1282	1.0765	1.1321	1.0962	1.1365	1.0963	1.1251	1.0944	**1.36**
**H(11A-11C)**	0.7472	0.7318	0.7068	0.7358	0.7093	0.7350	0.7053	0.7349	0.7057	0.7372	0.6895	0.7472	0.7318	0.7068	0.7358	0.7093	0.7350	0.7053	0.7349	0.7057	0.7372	0.6895	**0.77**
**H(1`A)**	2.6657	2.6307	2.6129	2.6014	2.5600	2.6032	2.5669	2.6055	2.5709	2.6003	2.5489	2.9001	2.8586	2.8607	2.8302	2.7981	2.8312	2.8173	2.8329	2.8204	2.8300	2.7824	**3.03**
**H(1`B)**	3.0859	3.0897	3.1239	3.1217	3.1803	3.1192	3.1769	3.1173	3.1736	3.1247	3.1811	2.7571	2.6509	2.6319	2.6254	2.6408	2.6256	2.6162	2.6266	2.6154	2.6259	2.6110	**3.23**
**H(2`-3`A-2`-3`B)**	1.6159	1.6849	1.6504	1.7202	1.6839	1.7178	1.7001	1.7160	1.6946	1.7231	1.7398	1.6159	1.6849	1.6504	1.7202	1.6839	1.7178	1.7001	1.7160	1.6946	1.7231	1.7398	**1.77**
**H(4`A)**	2.7572	2.6509	2.6319	2.6254	2.6408	2.6256	2.6162	2.6266	2.6154	2.6259	2.6110	3.0859	3.0897	3.1239	3.1217	3.1803	3.1192	3.1769	3.1173	3.1736	3.1247	3.1811	**3.03**
**H(4`B)**	2.9001	2.8586	2.8607	2.8302	2.7981	2.8312	2.8173	2.8329	2.8204	2.8300	2.7824	2.6657	2.6307	2.6129	2.6014	2.5600	2.6032	2.5669	2.6055	2.5709	2.6003	2.5489	**3.23**
Nuclei	M06-2X																						
**C1**	153.3291	154.7871	155.3185	155.1446	156.8382	155.1472	155.7261	155.1514	155.7127	155.1362	156.0491	153.3291	154.7871	155.3186	155.1447	156.8382	155.1473	155.7261	155.1514	155.7127	155.1362	156.0491	**133.8**
**C2**	155.0583	154.9839	155.5683	154.8182	155.5016	154.8518	155.9516	154.8863	155.8250	154.7801	155.9906	155.0582	154.9839	155.5684	154.8183	155.5016	154.8520	155.9516	154.8863	155.8250	154.7801	155.9905	**132.1**
**C3**	135.8412	136.7326	137.3391	136.9751	137.7562	136.9847	138.0048	136.9953	137.9310	136.9592	138.1039	135.8413	136.7326	137.3391	136.9750	137.7562	136.9849	138.0048	136.9953	137.9310	136.9592	138.1038	**115.8**
**C4**	187.2173	187.9919	188.6609	188.4098	189.0989	188.4045	189.7883	188.4003	189.6232	188.4086	189.9747	187.2174	187.9919	188.6609	188.4097	189.0989	188.4044	189.7883	188.4003	189.6232	188.4086	189.9747	**165.8**
**C5**	133.6413	135.1246	135.8209	135.8987	136.8542	135.8716	137.1085	135.8456	136.9862	135.9180	137.3251	133.6413	135.1246	135.8207	135.8985	136.8544	135.8714	137.1085	135.8456	136.9862	135.9180	137.3251	**115.8**
**C6**	151.4165	153.0719	154.3982	154.2636	156.0404	154.2072	156.6989	154.1526	156.4570	154.3108	157.1474	151.4165	153.0719	154.3980	154.2635	156.0404	154.2072	156.6989	154.1526	156.4570	154.3108	157.1476	**132.1**
**C7**	219.5714	225.6251	226.4951	228.5930	229.0932	228.4336	234.8478	228.2756	233.3454	228.7382	236.1827	219.5713	225.6251	226.4950	228.5930	229.0931	228.4336	234.8478	228.2756	233.3454	228.7382	236.1823	**198.9**
**C8**	67.3496	68.9257	69.3640	69.5721	70.5321	69.5564	70.9435	69.5415	70.6953	69.5807	71.5060	67.3495	68.9257	69.3641	69.5721	70.5320	69.5563	70.9435	69.5415	70.6953	69.5807	71.5060	**69.3**
**C9**	36.8567	36.8589	37.0158	36.7999	37.1970	36.8253	37.3284	36.8517	37.2330	36.7693	37.5513	36.8568	36.8589	37.0158	36.7999	37.1970	36.8253	37.3284	36.8517	37.2330	36.7693	37.5513	**31.5**
**C10**	18.0885	18.5555	18.7874	18.7570	19.2509	18.7656	19.0880	18.7751	19.0752	18.7427	19.1439	18.0887	18.5555	18.7874	18.7570	19.2510	18.7655	19.0880	18.7751	19.0752	18.7427	19.1439	**19.9**
**C11**	15.0629	15.2089	15.3040	15.2094	15.4713	15.2310	15.4101	15.2538	15.3993	15.1825	15.6383	15.0629	15.2089	15.3040	15.2094	15.4713	15.2311	15.4101	15.2538	15.3993	15.1825	15.6384	**14.3**
**C1`**	52.8605	53.6361	53.9038	54.0414	54.6565	54.0379	54.5960	54.0357	54.5181	54.0382	54.9503	57.0031	57.9878	58.3074	58.4452	59.0028	58.4380	59.0955	58.4322	59.0095	58.4458	59.3761	**50.6**
**C2`**	24.6809	25.0293	25.2326	25.2047	25.6210	25.2150	25.5892	25.2263	25.5423	25.1889	25.6187	23.8984	24.0888	24.4330	24.2294	24.7165	24.2408	24.6837	24.2534	24.6853	24.2124	24.7030	**23.8**
**C3`**	23.8984	24.0888	24.4330	24.2294	24.7165	24.2407	24.6837	24.2534	24.6853	24.2124	24.7030	24.6810	25.0293	25.2326	25.2047	25.6209	25.2149	25.5892	25.2263	25.5423	25.1889	25.6186	**23.8**
**C4`**	57.0032	57.9878	58.3074	58.4452	59.0027	58.4380	59.0955	58.4322	59.0095	58.4458	59.3761	52.8604	53.6361	53.9037	54.0412	54.6565	54.0378	54.5960	54.0357	54.5181	54.0382	54.9503	**50.6**
**H2**	9.2435	9.1320	9.1902	9.0953	9.1007	9.0961	9.1720	9.0966	9.1725	9.0947	9.1650	9.2435	9.1320	9.1902	9.0953	9.1007	9.0961	9.1720	9.0966	9.1725	9.0947	9.1650	**8.35**
**H3**	7.8772	7.9982	8.0934	8.0483	8.1676	8.0444	8.1900	8.0402	8.1807	8.0521	8.2066	7.8772	7.9982	8.0934	8.0483	8.1676	8.0444	8.1900	8.0402	8.1807	8.0521	8.2066	**7.06**
**H5**	7.6746	7.8772	7.9724	7.9688	8.0991	7.9622	8.1171	7.9553	8.1054	7.9752	8.1429	7.6746	7.8772	7.9724	7.9688	8.0991	7.9622	8.1171	7.9553	8.1054	7.9752	8.1429	**7.06**
**H6**	8.6459	8.7647	8.8936	8.8676	9.0102	8.8595	9.0759	8.8512	9.0622	8.8755	9.1051	8.6459	8.7647	8.8936	8.8676	9.0102	8.8595	9.0759	8.8512	9.0622	8.8755	9.1051	**8.35**
**H8**	4.3303	4.3457	4.4379	4.4105	4.4876	4.4042	4.5805	4.3977	4.5668	4.4167	4.5866	4.3303	4.3457	4.4379	4.4105	4.4876	4.4042	4.5805	4.3977	4.5668	4.4167	4.5866	**4.88**
**H(9A-9B)**	1.6913	1.7175	1.7109	1.7491	1.7610	1.7458	1.7590	1.7421	1.7535	1.7525	1.7975	1.6913	1.7175	1.7109	1.7491	1.7611	1.7458	1.7590	1.7421	1.7535	1.7525	1.7976	**2.05**
**H(10A-10B)**	1.3094	1.1605	1.1400	1.0918	1.0252	1.0948	1.0487	1.0974	1.0562	1.0891	1.0370	1.3094	1.1605	1.1401	1.0917	1.0252	1.0948	1.0487	1.0974	1.0562	1.0891	1.0370	**1.36**
**H(11A-11C)**	0.7411	0.7207	0.7099	0.7259	0.6867	0.7241	0.6966	0.7221	0.7011	0.7277	0.6871	0.7412	0.7207	0.7099	0.7259	0.6867	0.7241	0.6966	0.7221	0.7011	0.7277	0.6871	**0.77**
**H(1`A)**	2.5866	2.5812	2.5720	2.5744	2.5355	2.5741	2.5565	2.5727	2.5557	2.5752	2.5400	2.7747	2.8007	2.7681	2.7901	2.7549	2.7905	2.7525	2.7891	2.7495	2.7905	2.7250	**3.03**
**H(1`B)**	3.0713	3.0935	3.1264	3.1261	3.1796	3.1201	3.1738	3.1187	3.1697	3.1297	3.1840	2.7188	2.6476	2.5393	2.6074	2.5518	2.6103	2.5494	2.6089	2.5464	2.6068	2.5468	**3.23**
**H(2`-3`A-2`-3`B)**	1.6590	1.7094	1.6801	1.7477	1.7048	1.7439	1.7265	1.7398	1.7202	1.7515	1.7471	1.6590	1.7094	1.6801	1.7477	1.7048	1.7439	1.7265	1.7398	1.7202	1.7515	1.7471	**1.77**
**H(4`A)**	2.7188	2.6476	2.5910	2.6074	2.6000	2.6103	2.5494	2.6089	2.5464	2.6068	2.5468	3.0713	3.0935	3.0313	3.1261	3.1762	3.1201	3.1738	3.1187	3.1697	3.1297	3.1840	**3.03**
**H(4`B)**	2.7747	2.8007	2.7677	2.7901	2.7550	2.7905	2.7525	2.7891	2.7495	2.7905	2.7250	2.5866	2.5812	2.5203	2.5744	2.5589	2.5741	2.5565	2.5727	2.5557	2.5752	2.5400	**3.23**

[a] methyl protons shifts are averaged

[b] according to [4]

Table S4. Cont. VIII

Nuclei	R											S											Exp. [b]
	mpW1LYP																						
	Gas	$CHCl_3$ (PCM)	$CHCl_3$ (SMD)	DMSO (PCM)	DMSO (SMD)	CH_3OH (PCM)	CH_3OH (SMD)	C_2H_5OH (PCM)	C_2H_5OH (SMD)	H_2O (PCM)	H_2O (SMD)	Gas	$CHCl_3$ (PCM)	$CHCl_3$ (SMD)	DMSO (PCM)	DMSO (SMD)	CH_3OH (PCM)	CH_3OH (SMD)	C_2H_5OH (PCM)	C_2H_5OH (SMD)	H_2O (PCM)	H_2O (SMD)	
**C1**	139.8356	140.7551	141.5393	141.5708	143.3380	141.5390	142.7670	141.5085	142.8018	141.5990	143.1474	139.8356	140.7550	141.5393	141.5707	143.3380	141.5390	142.7670	141.5085	142.8018	141.5990	143.1475	**133.8**
**C2**	139.8936	139.8117	140.3785	139.6961	140.1507	139.7012	140.6144	139.7078	140.5425	139.6893	140.5949	139.8936	139.8117	140.3787	139.6963	140.1507	139.7013	140.6144	139.7078	140.5425	139.6894	140.5948	**132.1**
**C3**	121.9367	122.5923	123.1083	122.8622	123.4811	122.8480	123.7561	122.8350	123.6954	122.8735	123.8211	121.9367	122.5922	123.1082	122.8624	123.4811	122.8482	123.7561	122.8350	123.6954	122.8735	123.8210	**115.8**
**C4**	176.3104	177.0944	177.7085	177.5496	178.2255	177.5218	178.7909	177.4952	178.6500	177.5741	178.8796	176.3104	177.0944	177.7086	177.5495	178.2255	177.5218	178.7909	177.4952	178.6500	177.5740	178.8795	**165.8**
**C5**	119.9370	121.1966	121.7621	121.8575	122.5921	121.8166	122.7836	121.7768	122.6894	121.8945	122.9039	119.9370	121.1966	121.7622	121.8575	122.5921	121.8164	122.7836	121.7768	122.6894	121.8946	122.9039	**115.8**
**C6**	136.2352	138.0554	139.1590	139.0014	140.3442	138.9299	140.9774	138.8603	140.7198	139.0675	141.2030	136.2352	138.0555	139.1590	139.0014	140.3442	138.9298	140.9774	138.8603	140.7198	139.0675	141.2031	**132.1**
**C7**	203.5876	208.4777	209.6892	211.4737	212.2101	211.3031	217.5630	211.1336	216.3437	211.6342	218.7456	203.5876	208.4777	209.6895	211.4737	212.2101	211.3030	217.5630	211.1336	216.3437	211.6341	218.7456	**198.9**
**C8**	65.8261	66.9441	67.7688	67.5937	68.2546	67.5448	69.0166	67.4979	68.8463	67.6389	69.3302	65.8261	66.9442	67.7688	67.5938	68.2546	67.5448	69.0166	67.4979	68.8463	67.6388	69.3302	**69.3**
**C9**	38.6093	38.2599	38.5123	37.9672	38.0706	37.9745	38.2488	37.9834	38.2026	37.9582	38.2596	38.6093	38.2599	38.5124	37.9672	38.0706	37.9746	38.2488	37.9834	38.2026	37.9582	38.2596	**31.5**
**C10**	20.0344	20.4519	20.7617	20.6985	21.1134	20.6777	21.0113	20.6581	20.9981	20.7163	21.1007	20.0344	20.4520	20.7619	20.6985	21.1134	20.6776	21.0113	20.6581	20.9981	20.7164	21.1007	**19.9**
**C11**	16.2102	16.3479	16.4871	16.4264	16.6030	16.4187	16.5694	16.4126	16.5762	16.4317	16.7050	16.2102	16.3479	16.4872	16.4263	16.6030	16.4187	16.5694	16.4125	16.5762	16.4316	16.7051	**14.3**
**C1`**	51.9563	52.7773	53.0752	53.3398	53.8163	53.3010	53.8278	53.2634	53.8118	53.3745	54.2584	56.5332	57.4832	57.8466	58.0506	58.5951	58.0156	58.6967	57.9824	58.6302	58.0824	58.9219	**50.6**
**C2`**	25.9214	26.3457	26.6481	26.5892	26.9854	26.5692	27.0121	26.5510	26.9968	26.6066	26.8527	25.4974	25.6876	25.9879	25.8794	26.4450	25.8655	26.3109	25.8536	26.3122	25.8908	26.1183	**23.8**
**C3`**	25.4974	25.6876	25.9879	25.8794	26.4450	25.8655	26.3109	25.8536	26.3122	25.8908	26.1184	25.9214	26.3456	26.6482	26.5892	26.9854	26.5691	27.0121	26.5510	26.9968	26.6065	26.8526	**23.8**
**C4`**	56.5332	57.4831	57.8466	58.0507	58.5951	58.0156	58.6967	57.9824	58.6302	58.0823	58.9220	51.9563	52.7772	53.0753	53.3398	53.8163	53.3010	53.8278	53.2634	53.8118	53.3746	54.2585	**50.6**
**H2**	8.7102	8.6001	8.6319	8.4981	8.4848	8.5027	8.4902	8.5070	8.4957	8.4936	8.4690	8.7102	8.6001	8.6319	8.4981	8.4848	8.5027	8.4902	8.5070	8.4957	8.4936	8.4690	**8.35**
**H3**	7.3263	7.4607	7.5494	7.5074	7.6210	7.5055	7.6375	7.5033	7.6313	7.5089	7.6488	7.3263	7.4607	7.5494	7.5073	7.6210	7.5055	7.6375	7.5033	7.6313	7.5089	7.6488	**7.06**
**H5**	7.1691	7.3525	7.4414	7.4299	7.5506	7.4256	7.5648	7.4211	7.5535	7.4337	7.5817	7.1691	7.3525	7.4414	7.4298	7.5506	7.4256	7.5648	7.4211	7.5535	7.4337	7.5817	**7.06**
**H6**	7.9988	8.1752	8.2550	8.2640	8.3936	8.2583	8.4294	8.2524	8.4103	8.2692	8.4459	7.9988	8.1752	8.2550	8.2640	8.3936	8.2583	8.4294	8.2524	8.4103	8.2692	8.4459	**8.35**
**H8**	4.5608	4.6221	4.6358	4.6634	4.7632	4.6610	4.7838	4.6587	4.7653	4.6656	4.7714	4.5608	4.6221	4.6358	4.6634	4.7632	4.6611	4.7838	4.6587	4.7653	4.6656	4.7714	**4.88**
**H(9A-9B)**	1.6625	1.7071	1.7231	1.7096	1.6856	1.7075	1.6934	1.7051	1.6906	1.7115	1.7113	1.6625	1.7071	1.7231	1.7096	1.6856	1.7075	1.6934	1.7051	1.6906	1.7115	1.7114	**2.05**
**H(10A-10B)**	1.4121	1.2981	1.2449	1.2382	1.1784	1.2427	1.1905	1.2469	1.1939	1.2339	1.1760	1.4121	1.2981	1.2449	1.2382	1.1784	1.2427	1.1905	1.2469	1.1939	1.2339	1.1760	**1.36**
**H(11A-11C)**	0.7928	0.7967	0.7702	0.8027	0.7723	0.8024	0.7723	0.8019	0.7730	0.8029	0.7617	0.7928	0.7967	0.7702	0.8027	0.7723	0.8024	0.7723	0.8019	0.7730	0.8028	0.7618	**0.77**
**H(1`A)**	2.7236	2.7275	2.7055	2.7174	2.6660	2.7185	2.6794	2.7191	2.6803	2.7164	2.6542	2.8554	2.8541	2.8458	2.8386	2.8048	2.8398	2.8166	2.8406	2.8183	2.8374	2.7805	**3.03**
**H(1`B)**	3.1803	3.2244	3.2675	3.2573	3.3254	3.2550	3.3093	3.2524	3.3051	3.2594	3.3063	2.8521	2.7125	2.7047	2.6810	2.6748	2.6828	2.6568	2.6843	2.6569	2.6793	2.6525	**3.23**
**H(2`-3`A-2`-3`B)**	1.6666	1.7253	1.6891	1.7717	1.7304	1.7693	1.7496	1.7667	1.7449	1.7739	1.7740	1.6666	1.7253	1.6891	1.7717	1.7304	1.7693	1.7496	1.7667	1.7449	1.7739	1.7739	**1.77**
**H(4`A)**	2.8521	2.7125	2.7047	2.6810	2.6748	2.6828	2.6568	2.6843	2.6569	2.6794	2.6525	3.1803	3.2244	3.2675	3.2573	3.3254	3.2550	3.3093	3.2524	3.3051	3.2594	3.3063	**3.03**
**H(4`B)**	2.8554	2.8541	2.8458	2.8386	2.8048	2.8398	2.8166	2.8406	2.8183	2.8374	2.7805	2.7236	2.7275	2.7055	2.7174	2.6660	2.7185	2.6794	2.7191	2.6803	2.7164	2.6542	**3.23**
Nuclei	mpW1PW91																						
**C1**	136.9559	137.8933	138.5161	138.5364	140.5558	138.4926	140.0136	138.4501	140.0184	138.5751	140.3824	136.9559	137.8933	138.5161	138.5363	140.5558	138.4926	140.0136	138.4501	140.0183	138.5751	140.3824	**133.8**
**C2**	138.9131	138.8764	139.4426	138.7354	139.3309	138.7450	139.6903	138.7603	139.6319	138.7258	139.6920	138.9131	138.8764	139.4426	138.7355	139.3309	138.7450	139.6903	138.7603	139.6319	138.7259	139.6921	**132.1**
**C3**	121.0595	121.8043	122.3814	122.0536	122.8153	122.0413	123.1065	122.0342	123.0344	122.0641	123.2061	121.0595	121.8043	122.3814	122.0537	122.8153	122.0413	123.1065	122.0342	123.0344	122.0642	123.2061	**115.8**
**C4**	173.2952	174.0694	174.6948	174.4526	175.1461	174.4300	175.7536	174.4129	175.6073	174.4733	175.8718	173.2952	174.0694	174.6948	174.4526	175.1461	174.4300	175.7536	174.4129	175.6073	174.4733	175.8718	**165.8**
**C5**	119.1323	120.4902	121.1083	121.1722	122.0465	121.1316	122.2645	121.0960	122.1539	121.2098	122.4220	119.1323	120.4902	121.1083	121.1721	122.0465	121.1316	122.2645	121.0960	122.1540	121.2096	122.4220	**115.8**
**C6**	135.1018	136.9403	138.0642	137.8904	139.2324	137.8314	139.8237	137.7800	139.5613	137.9471	140.0936	135.1018	136.9403	138.0642	137.8903	139.2324	137.8314	139.8237	137.7800	139.5613	137.9468	140.0936	**132.1**
**C7**	201.4508	206.1158	207.1079	208.8566	209.8570	208.6808	215.0117	208.5075	213.7402	209.0216	216.1719	201.4508	206.1157	207.1079	208.8569	209.8570	208.6808	215.0117	208.5075	213.7402	209.0214	216.1719	**198.9**
**C8**	63.7173	64.9068	65.5328	65.6188	66.5348	65.5678	67.1954	65.5223	66.9610	65.6670	67.6444	63.7173	64.9068	65.5328	65.6188	66.5348	65.5678	67.1954	65.5223	66.9609	65.6670	67.6444	**69.3**
**C9**	36.5915	36.4355	36.6600	36.2407	36.4857	36.2519	36.5631	36.2705	36.4791	36.2304	36.6567	36.5915	36.4355	36.6600	36.2407	36.4857	36.2519	36.5631	36.2705	36.4791	36.2304	36.6568	**31.5**
**C10**	18.4168	18.8786	19.1477	19.1084	19.5681	19.0932	19.4539	19.0843	19.4181	19.1227	19.6406	18.4169	18.8786	19.1477	19.1085	19.5681	19.0932	19.4539	19.0843	19.4180	19.1227	19.6406	**19.9**
**C11**	15.7070	15.8645	15.9583	15.8942	16.1302	15.8921	16.0752	15.8960	16.0731	15.8958	16.2350	15.7070	15.8645	15.9583	15.8942	16.1302	15.8920	16.0752	15.8960	16.0730	15.8958	16.2349	**14.3**
**C1`**	50.5467	51.3539	51.5885	51.8531	52.3925	51.8221	52.3437	51.7965	52.3054	51.8818	52.8568	54.8323	55.7682	56.1209	56.2585	56.8194	56.2270	56.9768	56.2009	56.9196	56.2876	57.2205	**50.6**
**C2`**	24.7107	25.0958	25.3710	25.2826	25.7032	25.2695	25.7283	25.2621	25.6958	25.2947	25.5762	24.1643	24.3659	24.6526	24.4783	25.0112	24.4715	24.9328	24.4702	24.9254	24.4839	24.7891	**23.8**
**C3`**	24.1643	24.3659	24.6526	24.4783	25.0112	24.4715	24.9328	24.4702	24.9254	24.4838	24.7890	24.7107	25.0958	25.3710	25.2826	25.7032	25.2695	25.7283	25.2621	25.6957	25.2946	25.5763	**23.8**
**C4`**	54.8323	55.7682	56.1209	56.2585	56.8195	56.2270	56.9768	56.2009	56.9196	56.2877	57.2206	50.5467	51.3539	51.5885	51.8531	52.3925	51.8221	52.3437	51.7965	52.3054	51.8818	52.8568	**50.6**
**H2**	8.7653	8.6546	8.6949	8.5614	8.5274	8.5679	8.5318	8.5749	8.5400	8.5557	8.5160	8.7653	8.6546	8.6949	8.5614	8.5274	8.5679	8.5318	8.57486	8.5400	8.5557	8.5160	**8.35**
**H3**	7.3564	7.4950	7.5856	7.5399	7.6616	7.5381	7.6806	7.5362	7.6727	7.5419	7.6974	7.3564	7.4950	7.5856	7.5399	7.6616	7.5381	7.6806	7.5362	7.6727	7.5419	7.6974	**7.06**
**H5**	7.1918	7.3823	7.4740	7.4623	7.5883	7.4580	7.6039	7.4539	7.5917	7.4665	7.6232	7.1918	7.3823	7.4740	7.4623	7.5883	7.4580	7.6039	7.4539	7.5917	7.4665	7.6232	**7.06**
**H6**	8.0912	8.2617	8.3568	8.3413	8.4516	8.3370	8.4945	8.3330	8.4771	8.3458	8.5141	8.0912	8.2617	8.3568	8.3413	8.4516	8.3370	8.4945	8.3330	8.4771	8.3458	8.5141	**8.35**
**H8**	4.5583	4.6205	4.6484	4.6484	4.7240	4.6471	4.7702	4.6460	4.7549	4.6499	4.7632	4.5583	4.6205	4.6484	4.6484	4.7240	4.6471	4.7702	4.6460	4.7549	4.6499	4.7632	**4.88**
**H(9A-9B)**	1.6918	1.7351	1.7416	1.7487	1.7252	1.7479	1.7091	1.7475	1.7044	1.7501	1.7363	1.6918	1.7351	1.7416	1.7487	1.7252	1.7479	1.7091	1.7475	1.7044	1.7501	1.7363	**2.05**
**H(10A-10B)**	1.3646	1.2488	1.2035	1.1765	1.1081	1.1816	1.1271	1.1867	1.1326	1.1719	1.1140	1.3646	1.2488	1.2035	1.1765	1.1081	1.1816	1.1271	1.1867	1.1326	1.1719	1.1140	**1.36**
**H(11A-11C)**	0.7361	0.7392	0.7155	0.7396	0.7037	0.7398	0.7066	0.7402	0.7086	0.7396	0.6966	0.7361	0.7392	0.7155	0.7397	0.7037	0.7398	0.7066	0.7402	0.7085	0.7397	0.6966	**0.77**
**H(1`A)**	2.6642	2.6704	2.6505	2.6544	2.6120	2.6559	2.6257	2.6575	2.6257	2.6533	2.5991	2.7948	2.7932	2.7786	2.7793	2.7486	2.7804	2.7644	2.7817	2.7629	2.7784	2.7336	**3.03**
**H(1`B)**	3.1541	3.1947	3.2255	3.2216	3.2804	3.2200	3.2629	3.2185	3.2596	3.2236	3.2612	2.8533	2.7136	2.6823	2.6703	2.6865	2.6724	2.6440	2.6747	2.6423	2.6686	2.6336	**3.23**
**H(2`-3`A-2`-3`B)**	1.6396	1.6975	1.6605	1.7354	1.6969	1.7332	1.7190	1.7311	1.7142	1.7377	1.7463	1.6396	1.6975	1.6605	1.7354	1.6969	1.7332	1.7190	1.7311	1.7142	1.7377	1.7463	**1.77**
**H(4`A)**	2.8533	2.7136	2.6823	2.6703	2.6865	2.6724	2.6440	2.6747	2.6423	2.6686	2.6336	3.1541	3.1947	3.2255	3.2216	3.2804	3.2200	3.2629	3.2185	3.2596	3.2236	3.2612	**3.03**
**H(4`B)**	2.7948	2.7932	2.7786	2.7793	2.7486	2.7804	2.7644	2.7817	2.7629	2.7784	2.7336	2.6642	2.6704	2.6505	2.6544	2.6120	2.6559	2.6257	2.6575	2.6257	2.6532	2.5991	**3.23**

[a] methyl protons shifts are averaged

[b] according to [4]

Table S4. Cont. IX

Nuclei	R											S											Exp. [b]
	O3LYP																						
	Gas	$CHCl_3$ (PCM)	$CHCl_3$ (SMD)	DMSO (PCM)	DMSO (SMD)	CH_3OH (PCM)	CH_3OH (SMD)	C_2H_5OH (PCM)	C_2H_5OH (SMD)	H_2O (PCM)	H_2O (SMD)	Gas	$CHCl_3$ (PCM)	$CHCl_3$ (SMD)	DMSO (PCM)	DMSO (SMD)	CH_3OH (PCM)	CH_3OH (SMD)	C_2H_5OH (PCM)	C_2H_5OH (SMD)	H_2O (PCM)	H_2O (SMD)	
**C1**	134.6700	136.7350	138.0774	137.7862	139.9680	137.7525	138.6540	137.7169	138.9233	137.8160	138.9525	134.6701	136.7350	138.0774	137.7862	139.9680	137.7525	138.6540	137.7169	138.9233	137.8160	138.9524	**133.8**
**C2**	134.1521	134.1172	134.7329	134.1503	134.7502	134.1633	135.1355	134.1711	135.0388	134.1313	135.1471	134.1521	134.1172	134.7329	134.1503	134.7503	134.1633	135.1355	134.1711	135.0388	134.1313	135.1470	**132.1**
**C3**	117.0796	117.7861	118.4184	118.1613	118.8456	118.1571	119.0899	118.1476	119.0204	118.1584	119.1418	117.0795	117.7861	118.4185	118.1613	118.8456	118.1571	119.0899	118.1476	119.0204	118.1584	119.1418	**115.8**
**C4**	169.5295	170.1231	170.7516	170.4650	171.0573	170.4530	171.6773	170.4354	171.4956	170.4694	171.7619	169.5294	170.1231	170.7516	170.4650	171.0572	170.4530	171.6773	170.4354	171.4956	170.4694	171.7618	**165.8**
**C5**	115.2924	116.3484	116.9871	116.9459	117.6904	116.9169	117.9563	116.8827	117.8349	116.9666	118.0874	115.2923	116.3484	116.9870	116.9459	117.6904	116.9169	117.9563	116.8827	117.8349	116.9666	118.0873	**115.8**
**C6**	130.1286	131.5708	132.4547	132.5104	133.6620	132.4536	134.4006	132.3911	134.0803	132.5568	134.6403	130.1286	131.5708	132.4546	132.5104	133.6621	132.4536	134.4006	132.3911	134.0803	132.5568	134.6402	**132.1**
**C7**	197.0416	202.5614	204.1833	205.5703	206.7569	205.4087	210.8503	205.2457	209.9957	205.7217	211.8476	197.0419	202.5614	204.1833	205.5703	206.7567	205.4087	210.8503	205.2457	209.9957	205.7217	211.8473	**198.9**
**C8**	64.5007	66.1237	66.7896	66.9482	68.0400	66.9077	68.4198	66.8643	68.3756	66.9825	68.7838	64.5008	66.1237	66.7896	66.9482	68.0399	66.9077	68.4198	66.8643	68.3756	66.9825	68.7837	**69.3**
**C9**	36.8866	36.4970	36.7329	36.3964	36.6310	36.4109	36.9071	36.4198	36.8496	36.3759	36.9523	36.8866	36.4970	36.7330	36.3964	36.6311	36.4109	36.9071	36.4198	36.8496	36.3760	36.9523	**31.5**
**C10**	19.5667	20.0008	20.2903	20.3020	20.8140	20.2929	20.7362	20.2786	20.7115	20.3040	20.8623	19.5667	20.0008	20.2903	20.3020	20.8140	20.2929	20.7362	20.2786	20.7115	20.3039	20.8624	**19.9**
**C11**	16.0407	16.1490	16.3067	16.2326	16.4675	16.2358	16.4305	16.2338	16.4349	16.2231	16.5667	16.0407	16.1490	16.3067	16.2326	16.4676	16.2358	16.4305	16.2338	16.4349	16.2231	16.5666	**14.3**
**C1`**	50.7757	51.5877	51.9059	52.0796	52.6905	52.0588	52.6700	52.0330	52.6459	52.0927	53.0142	55.2927	56.2797	56.6547	56.8181	57.3377	56.7973	57.3943	56.7718	57.3132	56.8316	57.5927	**50.6**
**C2`**	25.8874	26.2979	26.5421	26.5335	26.9416	26.5279	26.9525	26.5174	26.9218	26.5325	26.8274	25.6559	25.8485	26.2667	26.0617	26.5537	26.0582	26.3970	26.0502	26.3838	26.0594	26.1411	**23.8**
**C3`**	25.6559	25.8485	26.2668	26.0617	26.5537	26.0582	26.3970	26.0502	26.3838	26.0594	26.1410	25.8873	26.2979	26.5421	26.5335	26.9417	26.5279	26.9525	26.5174	26.9218	26.5325	26.8275	**23.8**
**C4`**	55.2927	56.2797	56.6547	56.8181	57.3377	56.7973	57.3943	56.7718	57.3132	56.8316	57.5928	50.7758	51.5877	51.9060	52.0796	52.6904	52.0588	52.6700	52.0330	52.6459	52.0927	53.0141	**50.6**
**H2**	8.5055	8.3012	8.2945	8.2161	8.1877	8.2208	8.2753	8.2248	8.2525	8.2112	8.2672	8.5055	8.3012	8.2946	8.2161	8.1877	8.2208	8.2753	8.2248	8.2525	8.2112	8.2672	**8.35**
**H3**	7.2858	7.3932	7.4811	7.4335	7.5463	7.4318	7.5693	7.4296	7.5598	7.4350	7.5821	7.2857	7.3932	7.4811	7.4335	7.5463	7.4318	7.5693	7.4296	7.5598	7.4350	7.5821	**7.06**
**H5**	7.1355	7.2960	7.3891	7.3704	7.4914	7.3660	7.5096	7.3614	7.4960	7.3743	7.5295	7.1355	7.2960	7.3891	7.3704	7.4914	7.3660	7.5096	7.3614	7.4960	7.3743	7.5295	**7.06**
**H6**	7.9390	8.0509	8.1185	8.1320	8.2238	8.1268	8.2873	8.1211	8.2535	8.1366	8.3045	7.9390	8.0509	8.1185	8.1320	8.2238	8.1268	8.2873	8.1211	8.2535	8.1366	8.3045	**8.35**
**H8**	4.7534	4.7762	4.7904	4.8059	4.8401	4.8043	4.8723	4.8022	4.8493	4.8073	4.8600	4.7534	4.7762	4.7904	4.8059	4.8401	4.8043	4.8723	4.8022	4.8493	4.8073	4.8600	**4.88**
**H(9A-9B)**	1.5857	1.5960	1.5743	1.6144	1.6160	1.6127	1.6478	1.6105	1.6420	1.6158	1.6759	1.5857	1.5960	1.5743	1.6144	1.6160	1.6127	1.6478	1.6105	1.6420	1.6158	1.6759	**2.05**
**H(10A-10B)**	1.3660	1.2834	1.2609	1.2547	1.1867	1.2579	1.1996	1.2607	1.1992	1.2516	1.1809	1.3660	1.2834	1.2609	1.2547	1.1867	1.2579	1.1996	1.2607	1.1992	1.2516	1.1809	**1.36**
**H(11A-11C)**	0.7648	0.7704	0.7545	0.7870	0.7544	0.7864	0.7543	0.7855	0.7523	0.7876	0.7412	0.7648	0.7704	0.7545	0.7870	0.7544	0.7864	0.7543	0.7855	0.7523	0.7876	0.7411	**0.77**
**H(1`A)**	2.7701	2.7625	2.7405	2.7555	2.7038	2.7567	2.7128	2.7576	2.7141	2.7543	2.6909	2.9160	2.9130	2.8950	2.8860	2.8551	2.8877	2.8575	2.8891	2.8620	2.8842	2.8234	**3.03**
**H(1`B)**	3.2375	3.2724	3.3158	3.3162	3.3800	3.3137	3.3688	3.3111	3.3631	3.3184	3.3660	2.8835	2.7784	2.7910	2.7707	2.7967	2.7708	2.7626	2.7707	2.7732	2.7708	2.7597	**3.23**
**H(2`-3`A-2`-3`B)**	1.6811	1.7424	1.7163	1.7850	1.7421	1.7829	1.7553	1.7804	1.7499	1.7870	1.7766	1.6811	1.7424	1.7163	1.7850	1.7421	1.7829	1.7553	1.7804	1.7499	1.7870	1.7766	**1.77**
**H(4`A)**	2.8835	2.7784	2.7910	2.7707	2.7967	2.7708	2.7626	2.7707	2.7732	2.7708	2.7597	3.2376	3.2724	3.3157	3.3162	3.3800	3.3137	3.3688	3.3111	3.3631	3.3184	3.3660	**3.03**
**H(4`B)**	2.9160	2.9130	2.8950	2.8860	2.8551	2.8877	2.8575	2.8891	2.8620	2.8842	2.8234	2.7701	2.7625	2.7405	2.7555	2.7038	2.7567	2.7128	2.7576	2.7141	2.7543	2.6909	**3.23**
Nuclei	OLYP																						
**C1**	133.7662	135.8243	136.8482	136.5375	138.7381	136.4802	137.5934	136.4203	137.8888	136.5829	137.8613	133.7663	135.8243	136.8482	136.5375	138.7381	136.4802	137.5934	136.4203	137.8888	136.5830	137.8613	**133.8**
**C2**	131.6800	131.7598	132.3806	131.7534	132.3384	131.7657	132.7233	131.7722	132.6422	131.7318	132.7671	131.6801	131.7598	132.3808	131.7534	132.3384	131.7657	132.7233	131.7722	132.6422	131.7318	132.7671	**132.1**
**C3**	115.2861	116.0410	116.6769	116.3352	116.9878	116.3293	117.2608	116.3176	117.1923	116.3311	117.3062	115.2861	116.0410	116.6770	116.3352	116.9878	116.3293	117.2608	116.3176	117.1923	116.3311	117.3062	**115.8**
**C4**	168.4638	169.0396	169.7744	169.4326	170.0838	169.4209	170.6437	169.4032	170.4653	169.4352	170.7329	168.4638	169.0396	169.7744	169.4326	170.0838	169.4209	170.6437	169.4032	170.4653	169.4351	170.7329	**165.8**
**C5**	113.4566	114.4501	115.1055	115.0778	115.7698	115.0503	116.0665	115.0167	115.9370	115.0947	116.1973	113.4565	114.4501	115.1053	115.0778	115.7698	115.0503	116.0665	115.0167	115.9370	115.0948	116.1973	**115.8**
**C6**	127.6564	129.0488	130.0717	130.0799	131.2151	130.0293	131.8524	129.9726	131.5313	130.1199	132.0848	127.6565	129.0488	130.0714	130.0799	131.2151	130.0293	131.8524	129.9726	131.5313	130.1200	132.0848	**132.1**
**C7**	195.6304	200.9205	202.2732	203.5752	204.9425	203.3942	208.7242	203.2126	207.9733	203.7392	209.6516	195.6304	200.9205	202.2731	203.5752	204.9425	203.3942	208.7242	203.2126	207.9733	203.7392	209.6516	**198.9**
**C8**	66.0164	67.6742	68.2766	68.4831	69.5230	68.4284	69.9658	68.3702	69.9271	68.5261	70.2727	66.0164	67.6742	68.2768	68.4831	69.5230	68.4284	69.9658	68.3702	69.9271	68.5261	70.2727	**69.3**
**C9**	37.8775	37.5355	37.8329	37.4817	37.6919	37.4945	37.9963	37.5015	37.9257	37.4599	37.9956	37.8775	37.5355	37.8329	37.4817	37.6919	37.4945	37.9963	37.5015	37.9257	37.4599	37.9956	**31.5**
**C10**	20.3708	20.8487	21.2009	21.1635	21.6411	21.1551	21.5754	21.1408	21.5473	21.1623	21.6809	20.3708	20.8487	21.2007	21.1635	21.6411	21.1551	21.5754	21.1408	21.5473	21.1623	21.6809	**19.9**
**C11**	16.1842	16.2669	16.4521	16.3855	16.6001	16.3875	16.5670	16.3839	16.5716	16.3747	16.6889	16.1841	16.2669	16.4521	16.3855	16.6001	16.3875	16.5670	16.3839	16.5716	16.3747	16.6889	**14.3**
**C1`**	51.7190	52.5287	52.8737	53.0310	53.6180	53.0102	53.6219	52.9837	53.5953	53.0416	53.9470	56.3231	57.2890	57.6654	57.8153	58.3436	57.7917	58.4155	57.7629	58.3216	57.8291	58.6136	**50.6**
**C2`**	26.4617	26.9073	27.1798	27.1974	27.5680	27.1902	27.5895	27.1773	27.5416	27.1953	27.4841	26.4356	26.6445	27.0375	26.8194	27.3310	26.8124	27.1412	26.8007	27.1395	26.8176	26.8747	**23.8**
**C3`**	26.4356	26.6445	27.0373	26.8194	27.3310	26.8124	27.1412	26.8007	27.1395	26.8175	26.8747	26.4616	26.9073	27.1798	27.1974	27.5680	27.1902	27.5895	27.1773	27.5416	27.1953	27.4841	**23.8**
**C4`**	56.3231	57.2890	57.6655	57.8153	58.3436	57.7917	58.4155	57.7629	58.3216	57.8292	58.6136	51.7190	52.5287	52.8739	53.0310	53.6180	53.0102	53.6220	52.9837	53.5953	53.0416	53.9470	**50.6**
**H2**	8.3122	8.1471	8.1735	8.0875	8.0577	8.0938	8.1401	8.0997	8.1164	8.0809	8.1378	8.3122	8.1471	8.1735	8.0875	8.0577	8.0938	8.1401	8.0997	8.1164	8.0809	8.1378	**8.35**
**H3**	7.1968	7.3083	7.4006	7.3510	7.4622	7.3492	7.4865	7.3473	7.4763	7.3522	7.4980	7.1968	7.3083	7.4006	7.3510	7.4622	7.3492	7.4865	7.3473	7.4763	7.3522	7.4980	**7.06**
**H5**	7.0617	7.2114	7.3059	7.2908	7.4116	7.2863	7.4297	7.2817	7.4161	7.2947	7.4486	7.0616	7.2114	7.3058	7.2908	7.4116	7.2863	7.4297	7.2817	7.4161	7.2947	7.4486	**7.06**
**H6**	7.8271	7.9413	8.0216	8.0276	8.1151	8.0236	8.1721	8.0194	8.1371	8.0310	8.1906	7.8271	7.9413	8.0216	8.0276	8.1151	8.0236	8.1721	8.0194	8.1371	8.0310	8.1906	**8.35**
**H8**	4.9345	4.9386	4.9511	4.9514	4.9728	4.9514	4.9922	4.9512	4.9740	4.9510	4.9799	4.9345	4.9386	4.9511	4.9514	4.9728	4.9514	4.9922	4.9512	4.9740	4.9510	4.9799	**4.88**
**H(9A-9B)**	1.6018	1.6344	1.6307	1.6677	1.6629	1.6668	1.6963	1.6656	1.6899	1.6680	1.7229	1.6018	1.6344	1.6307	1.6677	1.6629	1.6668	1.6963	1.6656	1.6899	1.6680	1.7229	**2.05**
**H(10A-10B)**	1.3968	1.3365	1.3099	1.2954	1.2395	1.2980	1.2503	1.3005	1.2528	1.2925	1.2330	1.3968	1.3365	1.3099	1.2954	1.2395	1.2980	1.2503	1.3005	1.2528	1.2925	1.2330	**1.36**
**H(11A-11C)**	0.7752	0.7858	0.7691	0.7990	0.7713	0.7981	0.7697	0.7972	0.7690	0.7994	0.7572	0.7752	0.7858	0.7691	0.7990	0.7713	0.7981	0.7697	0.7972	0.7690	0.7994	0.7572	**0.77**
**H(1`A)**	2.8307	2.8265	2.8045	2.8170	2.7654	2.8180	2.7704	2.8190	2.7715	2.8155	2.7426	2.9862	2.9788	2.9601	2.9549	2.9231	2.9567	2.9221	2.9583	2.9272	2.9528	2.7426	**3.03**
**H(1`B)**	3.3288	3.3720	3.4187	3.4096	3.4766	3.4072	3.4606	3.4047	3.4562	3.4116	3.4558	2.9692	2.8714	2.8781	2.8638	2.8924	2.8627	2.8599	2.8618	2.8718	2.8645	3.4558	**3.23**
**H(2`-3`A-2`-3`B)**	1.7212	1.7789	1.7513	1.8155	1.7775	1.8131	1.7899	1.8107	1.7855	1.8174	1.8096	1.7212	1.7789	1.7513	1.8155	1.7775	1.8131	1.7899	1.8107	1.7855	1.8174	1.8096	**1.77**
**H(4`A)**	2.9691	2.8714	2.8782	2.8638	2.8924	2.8627	2.8599	2.8618	2.8718	2.8645	2.8506	3.3288	3.3720	3.4187	3.4096	3.4766	3.4072	3.4606	3.4047	3.4562	3.4116	2.8506	**3.03**
**H(4`B)**	2.9861	2.9788	2.9600	2.9549	2.9231	2.9567	2.9221	2.9583	2.9272	2.9528	2.8867	2.8307	2.8265	2.8045	2.8170	2.7654	2.8180	2.7704	2.8190	2.7715	2.8155	2.8867	**3.23**

[a] methyl protons shifts are averaged

[b] according to [4]

Table S4. Cont. X

Nuclei	R											S											Exp. [b]
	OPBE																						
	Gas	$CHCl_3$ (PCM)	$CHCl_3$ (SMD)	DMSO (PCM)	DMSO (SMD)	CH_3OH (PCM)	CH_3OH (SMD)	C_2H_5OH (PCM)	C_2H_5OH (SMD)	H_2O (PCM)	H_2O (SMD)	Gas	$CHCl_3$ (PCM)	$CHCl_3$ (SMD)	DMSO (PCM)	DMSO (SMD)	CH_3OH (PCM)	CH_3OH (SMD)	C_2H_5OH (PCM)	C_2H_5OH (SMD)	H_2O (PCM)	H_2O (SMD)	
**C1**	130.4493	132.8644	133.7230	133.3810	135.5557	133.3573	134.3189	133.3178	134.5747	133.4005	134.7226	130.4493	132.8644	133.7229	133.3810	135.5557	133.3573	134.3189	133.3178	134.5747	133.4005	134.7226	**133.8**
**C2**	130.3689	130.4777	131.1106	130.4003	131.0794	130.4227	131.4243	130.4267	131.3408	130.3743	131.4713	130.3689	130.4777	131.1104	130.4003	131.0794	130.4227	131.4243	130.4267	131.3408	130.3743	131.4713	**132.1**
**C3**	114.1253	115.0494	115.6469	115.3003	116.0208	115.3045	116.3076	115.2901	116.2355	115.2913	116.3908	114.1253	115.0494	115.6470	115.3003	116.0208	115.3044	116.3075	115.2901	116.2355	115.2913	116.3908	**115.8**
**C4**	164.8904	165.5133	166.1484	165.8236	166.4138	165.8225	167.0193	165.8034	166.8477	165.8192	167.1132	164.8904	165.5133	166.1485	165.8236	166.4138	165.8226	167.0193	165.8034	166.8478	165.8192	167.1132	**165.8**
**C5**	112.4138	113.5251	114.1304	114.1087	114.8563	114.0899	115.1793	114.0531	115.0484	114.1213	115.3343	112.4138	113.5251	114.1305	114.1087	114.8563	114.0899	115.1793	114.0531	115.0483	114.1213	115.3343	**115.8**
**C6**	126.2446	127.6532	128.5340	128.5693	129.6701	128.5305	130.3399	128.4733	130.0468	128.6008	130.5598	126.2446	127.6532	128.5339	128.5693	129.6701	128.5305	130.3399	128.4733	130.0467	128.6008	130.5598	**132.1**
**C7**	192.8452	198.2855	199.3972	200.6172	201.8936	200.4792	205.3787	200.3260	204.6650	200.7460	206.4215	192.8452	198.2855	199.3972	200.6172	201.8936	200.4790	205.3787	200.3260	204.6650	200.7460	206.4215	**198.9**
**C8**	63.3511	65.2455	65.8299	65.9430	67.0082	65.9067	67.4062	65.8548	67.3284	65.9747	67.8269	63.3511	65.2455	65.8299	65.9430	67.0082	65.9067	67.4062	65.8548	67.3284	65.9747	67.8269	**69.3**
**C9**	35.4839	35.3259	35.4925	35.2408	35.4394	35.2625	35.7811	35.2662	35.6909	35.2151	35.8596	35.4839	35.3259	35.4924	35.2408	35.4394	35.2625	35.7811	35.2662	35.6909	35.2151	35.8596	**31.5**
**C10**	18.3839	18.9262	19.1709	19.1777	19.5816	19.1792	19.5409	19.1625	19.5080	19.1714	19.7487	18.3839	18.9262	19.1709	19.1777	19.5816	19.1791	19.5409	19.1625	19.5081	19.1714	19.7487	**19.9**
**C11**	15.5088	15.6615	15.7302	15.6889	15.8422	15.7034	15.8187	15.7002	15.8190	15.6703	15.9747	15.5088	15.6615	15.7301	15.6889	15.8422	15.7035	15.8187	15.7002	15.8190	15.6703	15.9748	**14.3**
**C1\`**	49.8310	50.6853	50.9616	51.0854	51.5959	51.0768	51.5674	51.0503	51.5468	51.0887	51.9458	54.1846	55.2341	55.5306	55.6629	56.1364	55.6521	56.2029	55.6236	56.1348	55.6686	56.4163	**50.6**
**C2\`**	25.0749	25.5194	25.7331	25.6915	26.0326	25.6960	26.0213	25.6827	26.0054	25.6828	25.8849	24.8732	25.1362	25.3971	25.1852	25.6271	25.1955	25.4682	25.1883	25.4683	25.1710	25.1804	**23.8**
**C3\`**	24.8732	25.1363	25.3971	25.1852	25.6271	25.1955	25.4682	25.1883	25.4683	25.1710	25.1805	25.0749	25.5194	25.7331	25.6915	26.0326	25.6959	26.0213	25.6827	26.0054	25.6828	25.8850	**23.8**
**C4\`**	54.1846	55.2340	55.5307	55.6629	56.1364	55.6521	56.2030	55.6236	56.1348	55.6686	56.4160	49.8310	50.6853	50.9616	51.0854	51.5959	51.0769	51.5673	51.0503	51.5468	51.0887	51.9458	**50.6**
**H2**	8.3740	8.1768	8.2025	8.1261	8.0964	8.1307	8.1856	8.1345	8.1640	8.1207	8.1758	8.3740	8.1768	8.2025	8.1261	8.0964	8.1307	8.1856	8.1345	8.1640	8.1207	8.1758	**8.35**
**H3**	7.2246	7.3451	7.4322	7.3881	7.4977	7.3867	7.5248	7.3844	7.5145	7.3887	7.5383	7.2246	7.3451	7.4322	7.3881	7.4977	7.3867	7.5247	7.3844	7.5145	7.3887	7.5383	**7.06**
**H5**	7.0810	7.2395	7.3267	7.3187	7.4394	7.3146	7.4579	7.3098	7.4452	7.3218	7.4772	7.0810	7.2395	7.3267	7.3187	7.4394	7.3146	7.4579	7.3098	7.4452	7.3218	7.4772	**7.06**
**H6**	7.9066	8.0122	8.0790	8.0965	8.1759	8.0923	8.2338	8.0874	8.2046	8.0995	8.2407	7.9066	8.0122	8.0790	8.0965	8.1759	8.0924	8.2338	8.0874	8.2046	8.0995	8.2407	**8.35**
**H8**	4.9256	4.9388	4.9338	4.9538	4.9732	4.9534	4.9991	4.9523	4.9798	4.9533	4.9840	4.9256	4.9388	4.9339	4.9538	4.9732	4.9534	4.9991	4.9523	4.9798	4.9533	4.9840	**4.88**
**H(9A-9B)**	1.6214	1.6370	1.6357	1.6693	1.6561	1.6679	1.6926	1.6659	1.6826	1.6697	1.7222	1.6214	1.6370	1.6357	1.6693	1.6561	1.6680	1.6926	1.6659	1.6826	1.6697	1.7222	**2.05**
**H(10A-10B)**	1.3274	1.2657	1.2230	1.2199	1.1597	1.2236	1.1691	1.2265	1.1733	1.2157	1.1568	1.3274	1.2657	1.2230	1.2199	1.1597	1.2236	1.1691	1.2265	1.1733	1.2157	1.1568	**1.36**
**H(11A-11C)**	0.7068	0.7178	0.6922	0.7271	0.6937	0.7269	0.6920	0.7261	0.6924	0.7265	0.6799	0.7068	0.7178	0.6922	0.7271	0.6937	0.7270	0.6920	0.7261	0.6924	0.7265	0.6799	**0.77**
**H(1\`A)**	2.7577	2.7604	2.7294	2.7459	2.6937	2.7476	2.7010	2.7486	2.7028	2.7435	2.6776	2.9072	2.9050	2.8848	2.8821	2.8463	2.8840	2.8482	2.8852	2.8520	2.8795	2.8116	**3.03**
**H(1\`B)**	3.2916	3.3295	3.3588	3.3606	3.4135	3.3589	3.4015	3.3565	3.3967	3.3616	3.3958	2.9565	2.8569	2.8458	2.8304	2.8505	2.8317	2.8185	2.8324	2.8248	2.8286	2.8111	**3.23**
**H(2\`-3\`A-2\`-3\`B)**	1.6772	1.7373	1.7020	1.7688	1.7262	1.7674	1.7389	1.7652	1.7354	1.7696	1.7649	1.6772	1.7373	1.7020	1.7688	1.7262	1.7674	1.7389	1.7652	1.7354	1.7696	1.7649	**1.77**
**H(4\`A)**	2.9565	2.8569	2.8458	2.8304	2.8505	2.8317	2.8185	2.8324	2.8248	2.8286	2.8111	3.2916	3.3294	3.3588	3.3606	3.4135	3.3589	3.4015	3.3565	3.3967	3.3616	3.3958	**3.03**
**H(4\`B)**	2.9072	2.9050	2.8848	2.8821	2.8463	2.8840	2.8482	2.8852	2.8520	2.8795	2.8116	2.7577	2.7604	2.7294	2.7459	2.6937	2.7476	2.7010	2.7486	2.7028	2.7435	2.6776	**3.23**
Nuclei	OPW91																						
**C1**	130.7667	133.1792	134.0352	133.7004	135.8728	133.6658	134.6250	133.6167	134.8875	133.7173	135.0268	130.7667	133.1792	134.0351	133.7004	135.8728	133.6658	134.6250	133.6167	134.8875	133.7172	135.0268	**133.8**
**C2**	130.5896	130.7006	131.3253	130.6292	131.2902	130.6414	131.6411	130.6365	131.5552	130.5989	131.6892	130.5896	130.7006	131.3251	130.6292	131.2902	130.6413	131.6411	130.6365	131.5550	130.5989	131.6891	**132.1**
**C3**	114.3241	115.2447	115.8366	115.4992	116.2043	115.4934	116.4921	115.4699	116.4192	115.4860	116.5761	114.3241	115.2447	115.8367	115.4992	116.2043	115.4934	116.4921	115.4699	116.4191	115.4861	116.5761	**115.8**
**C4**	165.2950	165.9218	166.5553	166.2389	166.8184	166.2280	167.4274	166.1994	167.2536	166.2307	167.5234	165.2950	165.9219	166.5553	166.2389	166.8184	166.2280	167.4274	166.1994	167.2535	166.2307	167.5234	**165.8**
**C5**	112.6079	113.7187	114.3194	114.3065	115.0387	114.2778	115.3624	114.2319	115.2313	114.3152	115.5192	112.6079	113.7188	114.3195	114.3065	115.0387	114.2778	115.3624	114.2319	115.2314	114.3152	115.5192	**115.8**
**C6**	126.4628	127.8788	128.7584	128.8065	129.8918	128.7573	130.5695	128.6908	130.2737	128.8343	130.7939	126.4628	127.8789	128.7584	128.8065	129.8918	128.7573	130.5695	128.6908	130.2737	128.8343	130.7940	**132.1**
**C7**	193.2313	198.6873	199.8005	201.0323	202.3062	200.8826	205.8061	200.7188	205.0944	201.1593	206.8474	193.2313	198.6874	199.8004	201.0323	202.3062	200.8826	205.8061	200.7188	205.0944	201.1592	206.8475	**198.9**
**C8**	63.5767	65.4651	66.0452	66.1615	67.2228	66.1149	67.6145	66.0538	67.5445	66.1896	68.0379	63.5767	65.4651	66.0452	66.1615	67.2228	66.1149	67.6145	66.0538	67.5445	66.1896	68.0379	**69.3**
**C9**	35.6376	35.4754	35.6401	35.3932	35.5818	35.4050	35.9238	35.3997	35.8366	35.3636	36.0036	35.6376	35.4753	35.6401	35.3932	35.5818	35.4050	35.9238	35.3997	35.8366	35.3636	36.0037	**31.5**
**C10**	18.5106	19.0562	19.3007	19.3117	19.7102	19.3031	19.6681	19.2774	19.6364	19.3015	19.8723	18.5106	19.0561	19.3007	19.3117	19.7102	19.3031	19.6681	19.2774	19.6363	19.3015	19.8722	**19.9**
**C11**	15.5472	15.7047	15.7735	15.7387	15.8867	15.7430	15.8623	15.7305	15.8639	15.7162	16.0213	15.5472	15.7047	15.7736	15.7387	15.8867	15.7430	15.8623	15.7305	15.8639	15.7162	16.0213	**14.3**
**C1\`**	49.9712	50.8301	51.1068	51.2369	51.7437	51.2180	51.7174	51.1822	51.6970	51.2363	52.0939	54.3361	55.3900	55.6843	55.8260	56.2909	55.8053	56.3591	55.7678	56.2892	55.8279	56.5750	**50.6**
**C2\`**	25.1936	25.6429	25.8533	25.8205	26.1542	25.8150	26.1443	25.7928	26.1277	25.8077	26.0144	24.9944	25.2626	25.5214	25.3197	25.7547	25.3196	25.5948	25.3033	25.5941	25.3018	25.3107	**23.8**
**C3\`**	24.9944	25.2626	25.5214	25.3197	25.7547	25.3196	25.5948	25.3033	25.5942	25.3018	25.3106	25.1936	25.6429	25.8532	25.8205	26.1542	25.8150	26.1443	25.7928	26.1278	25.8078	26.0143	**23.8**
**C4\`**	54.3361	55.3900	55.6843	55.8260	56.2909	55.8053	56.3591	55.7678	56.2891	55.8280	56.5750	49.9712	50.8300	51.1069	51.2369	51.7437	51.2180	51.7174	51.1822	51.6970	51.2364	52.0939	**50.6**
**H2**	8.3747	8.1783	8.2038	8.1283	8.0967	8.1324	8.1870	8.1360	8.1645	8.1225	8.1772	8.3747	8.1783	8.2037	8.1283	8.0967	8.1324	8.1870	8.1360	8.1645	8.1225	8.1772	**8.35**
**H3**	7.2263	7.3462	7.4332	7.3898	7.4982	7.3876	7.5253	7.3852	7.5150	7.3900	7.5388	7.2263	7.3462	7.4332	7.3898	7.4982	7.3876	7.5253	7.3852	7.5150	7.3900	7.5388	**7.06**
**H5**	7.0832	7.2411	7.3283	7.3208	7.4403	7.3161	7.4590	7.3111	7.4460	7.3236	7.4782	7.0832	7.2411	7.3283	7.3208	7.4403	7.3160	7.4590	7.3111	7.4460	7.3236	7.4782	**7.06**
**H6**	7.9058	8.0115	8.0785	8.0971	8.1751	8.0923	8.2342	8.0871	8.2041	8.0997	8.2416	7.9058	8.0115	8.0785	8.0971	8.1751	8.0923	8.2342	8.0871	8.2041	8.0998	8.2416	**8.35**
**H8**	4.9246	4.9379	4.9328	4.9539	4.9719	4.9528	4.9984	4.9516	4.9786	4.9531	4.9834	4.9246	4.9379	4.9328	4.9539	4.9719	4.9528	4.9984	4.9516	4.9786	4.9531	4.9834	**4.88**
**H(9A-9B)**	1.6183	1.6350	1.6336	1.6680	1.6547	1.6661	1.6913	1.6638	1.6817	1.6682	1.7210	1.6183	1.6350	1.6336	1.6680	1.6547	1.6661	1.6913	1.6638	1.6817	1.6682	1.7210	**2.05**
**H(10A-10B)**	1.3299	1.2685	1.2258	1.2236	1.1623	1.2266	1.1720	1.2292	1.1758	1.2191	1.1591	1.3299	1.2685	1.2258	1.2236	1.1623	1.2266	1.1720	1.2292	1.1758	1.2191	1.1591	**1.36**
**H(11A-11C)**	0.7090	0.7203	0.6949	0.7305	0.6965	0.7297	0.6950	0.7286	0.6951	0.7297	0.6829	0.7090	0.7203	0.6949	0.7305	0.6965	0.7297	0.6950	0.7286	0.6951	0.7297	0.6829	**0.77**
**H(1\`A)**	2.7617	2.7640	2.7332	2.7501	2.6971	2.7512	2.7043	2.7520	2.7061	2.7474	2.6810	2.9112	2.9085	2.8882	2.8858	2.8496	2.8871	2.8514	2.8881	2.8553	2.8830	2.8151	**3.03**
**H(1\`B)**	3.2883	3.3267	3.3565	3.3589	3.4115	3.3565	3.3997	3.3539	3.3947	3.3596	3.3940	2.9516	2.8524	2.8425	2.8277	2.8479	2.8281	2.8154	2.8285	2.8224	2.8258	2.8088	**3.23**
**H(2\`-3\`A-2\`-3\`B)**	1.6773	1.7378	1.7028	1.7702	1.7270	1.7681	1.7397	1.7656	1.7359	1.7707	1.7652	1.6773	1.7378	1.7028	1.7702	1.7270	1.7681	1.7397	1.7656	1.7359	1.7708	1.7652	**1.77**
**H(4\`A)**	2.9516	2.8524	2.8425	2.8277	2.8479	2.8282	2.8154	2.8285	2.8224	2.8258	2.8088	3.2883	3.3267	3.3565	3.3589	3.4115	3.3565	3.3997	3.3539	3.3947	3.3596	3.3940	**3.03**
**H(4\`B)**	2.9112	2.9085	2.8882	2.8858	2.8496	2.8870	2.8514	2.8881	2.8553	2.8830	2.8151	2.7617	2.7640	2.7332	2.7501	2.6971	2.7512	2.7043	2.7520	2.7061	2.7474	2.6810	**3.23**

[a] methyl protons shifts are averaged

[b] according to [4]

Table S4. Cont. XI

Nuclei	R											S											Exp. [b]
	PBE																						
	Gas	$CHCl_3$ (PCM)	$CHCl_3$ (SMD)	DMSO (PCM)	DMSO (SMD)	CH_3OH (PCM)	CH_3OH (SMD)	C_2H_5OH (PCM)	C_2H_5OH (SMD)	H_2O (PCM)	H_2O (SMD)	Gas	$CHCl_3$ (PCM)	$CHCl_3$ (SMD)	DMSO (PCM)	DMSO (SMD)	CH_3OH (PCM)	CH_3OH (SMD)	C_2H_5OH (PCM)	C_2H_5OH (SMD)	H_2O (PCM)	H_2O (SMD)	
**C1**	135.7538	137.0503	137.5475	137.8060	139.7406	137.7637	139.4272	137.7334	139.3332	137.8648	139.8075	135.7538	137.0503	137.5475	137.8061	139.7406	137.7637	139.4272	137.7334	139.3332	137.8647	139.8074	**133.8**
**C2**	135.8065	135.8860	136.4810	135.7036	136.4289	135.7123	136.7962	135.7320	136.7400	135.7130	136.8172	135.8066	135.8860	136.4810	135.7036	136.4289	135.7123	136.7962	135.7320	136.7400	135.7130	136.8172	**132.1**
**C3**	119.3937	120.2555	120.8293	120.4598	121.2955	120.4475	121.6507	120.4456	121.5790	120.4890	121.7777	119.3937	120.2554	120.8293	120.4599	121.2955	120.4475	121.6507	120.4456	121.5790	120.4890	121.7776	**115.8**
**C4**	174.2136	175.1821	175.8656	175.5336	176.4397	175.5105	176.9941	175.4979	176.8739	175.5729	177.1398	174.2136	175.1821	175.8656	175.5336	176.4397	175.5106	176.9941	175.4979	176.8739	175.5730	177.1398	**165.8**
**C5**	117.5986	119.0081	119.6203	119.6045	120.5387	119.5663	120.7914	119.5387	120.6893	119.6582	120.9616	117.5986	119.0081	119.6203	119.6045	120.5387	119.5663	120.7914	119.5387	120.6893	119.6582	120.9616	**115.8**
**C6**	132.3081	134.1508	135.3129	135.0151	136.5859	134.9539	137.0949	134.9038	136.8563	135.0911	137.3931	132.3080	134.1508	135.3129	135.0151	136.5859	134.9538	137.0949	134.9038	136.8563	135.0911	137.3933	**132.1**
**C7**	201.2795	205.7851	206.7994	208.3176	209.5076	208.1594	213.8421	208.0133	212.7045	208.4866	214.9789	201.2795	205.7848	206.7994	208.3174	209.5076	208.1594	213.8421	208.0133	212.7045	208.4867	214.9789	**198.9**
**C8**	67.3124	68.7273	69.4684	69.4840	70.5173	69.4274	71.2296	69.3828	70.9057	69.5568	71.6596	67.3124	68.7273	69.4684	69.4840	70.5173	69.4273	71.2296	69.3828	70.9057	69.5569	71.6596	**69.3**
**C9**	40.1276	40.0069	40.3088	39.7443	40.0956	39.7515	40.2930	39.7702	40.1520	39.7559	40.4120	40.1277	40.0069	40.3088	39.7442	40.0956	39.7514	40.2930	39.7702	40.1520	39.7559	40.4120	**31.5**
**C10**	20.0763	20.6106	20.9112	20.7941	21.3510	20.7768	21.2710	20.7705	21.2080	20.8283	21.4983	20.0763	20.6106	20.9112	20.7940	21.3510	20.7768	21.2710	20.7707	21.2080	20.8283	21.4983	**19.9**
**C11**	15.8694	16.1085	16.1979	16.0767	16.3861	16.0740	16.3423	16.0824	16.3286	16.0976	16.5359	15.8693	16.1084	16.1979	16.0766	16.3861	16.0739	16.3423	16.0824	16.3286	16.0976	16.5359	**14.3**
**C1`**	53.2289	54.1288	54.3704	54.5888	55.2146	54.5567	55.1614	54.5356	55.1128	54.6370	55.7066	57.7454	58.8405	59.1962	59.2838	59.9592	59.2540	60.1471	59.2350	60.1124	59.3302	60.4098	**50.6**
**C2`**	25.6779	26.2489	26.5423	26.4277	26.9602	26.4116	27.0066	26.4066	26.9614	26.4608	26.9077	25.2774	25.6126	25.8947	25.6955	26.3439	25.6884	26.2808	25.6922	26.2785	25.7195	26.1972	**23.8**
**C3`**	25.2775	25.6125	25.8947	25.6954	26.3439	25.6885	26.2808	25.6921	26.2785	25.7195	26.1974	25.6779	26.2488	26.5423	26.4277	26.9602	26.4115	27.0066	26.4066	26.9614	26.4608	26.9077	**23.8**
**C4`**	57.7453	58.8405	59.1962	59.2838	59.9592	59.2540	60.1471	59.2351	60.1124	59.3302	60.4096	53.2289	54.1288	54.3704	54.5888	55.2146	54.5568	55.1614	54.5355	55.1128	54.6370	55.7066	**50.6**
**H2**	8.5413	8.4249	8.4705	8.3290	8.3128	8.3344	8.3182	8.3402	8.3289	8.3244	8.3070	8.5413	8.4249	8.4705	8.3290	8.3128	8.3344	8.3182	8.3403	8.3289	8.3244	8.3070	**8.35**
**H3**	7.1939	7.3320	7.4217	7.3724	7.4949	7.3707	7.5141	7.3693	7.5065	7.3747	7.5304	7.1939	7.3320	7.4217	7.3724	7.4949	7.3707	7.5141	7.3693	7.5065	7.3747	7.5304	**7.06**
**H5**	7.0374	7.2231	7.3131	7.2966	7.4239	7.2926	7.4373	7.2890	7.4263	7.3011	7.4549	7.0374	7.2231	7.3131	7.2966	7.4239	7.2926	7.4373	7.2890	7.4263	7.3011	7.4549	**7.06**
**H6**	7.9743	8.1212	8.2101	8.1810	8.2933	8.1773	8.3259	8.1741	8.3170	8.1851	8.3441	7.9743	8.1220	8.2101	8.1810	8.2933	8.1773	8.3259	8.1741	8.3170	8.1851	8.3441	**8.35**
**H8**	4.9863	5.0087	5.0207	5.0083	5.0807	5.0085	5.1095	5.0092	5.1076	5.0089	5.1020	4.9863	5.0087	5.0207	5.0084	5.0807	5.0085	5.1095	5.0092	5.1076	5.0089	5.1020	**4.88**
**H(9A-9B)**	1.8543	1.8898	1.9093	1.8989	1.8881	1.8970	1.8743	1.8957	1.8641	1.9014	1.9019	1.8543	1.8898	1.9093	1.8989	1.8881	1.8970	1.8743	1.8957	1.8641	1.9014	1.9019	**2.05**
**H(10A-10B)**	1.4519	1.3468	1.3025	1.2772	1.2192	1.2819	1.2355	1.2870	1.2429	1.2734	1.2243	1.4519	1.3468	1.3025	1.2772	1.2192	1.2819	1.2355	1.2870	1.2429	1.2734	1.2243	**1.36**
**H(11A-11C)**	0.7585	0.7622	0.7398	0.7594	0.7316	0.7595	0.7316	0.7601	0.7356	0.7599	0.7223	0.7584	0.7622	0.7398	0.7594	0.7316	0.7595	0.7316	0.7601	0.7356	0.7599	0.7223	**0.77**
**H(1`A)**	2.7941	2.8061	2.7875	2.7895	2.7526	2.7907	2.7598	2.7925	2.7597	2.7890	2.7278	2.9117	2.9223	2.9116	2.9098	2.8864	2.9106	2.8960	2.9121	2.8947	2.9096	2.8590	**3.03**
**H(1`B)**	3.3775	3.4057	3.4374	3.4252	3.4888	3.4236	3.4656	3.4226	3.4634	3.4274	3.4588	3.0568	2.9041	2.8696	2.8574	2.8623	2.8597	2.8258	2.8626	2.8184	2.8560	2.8095	**3.23**
**H(2`-3`A-2`-3`B)**	1.7348	1.7907	1.7541	1.8228	1.7870	1.8210	1.8072	1.8196	1.8042	1.8252	1.8373	1.7348	1.7907	1.7541	1.8228	1.7870	1.8210	1.8072	1.8197	1.8042	1.8252	1.8372	**1.77**
**H(4`A)**	3.0568	2.9041	2.8696	2.8574	2.8623	2.8597	2.8258	2.8627	2.8184	2.8560	2.8095	3.3775	3.4057	3.4374	3.4252	3.4888	3.4236	3.4656	3.4227	3.4634	3.4274	3.4587	**3.03**
**H(4`B)**	2.9117	2.9223	2.9116	2.9097	2.8864	2.9106	2.8960	2.9121	2.8947	2.9096	2.8590	2.7941	2.8061	2.7875	2.7895	2.7526	2.7907	2.7598	2.7925	2.7597	2.7890	2.7278	**3.23**
Nuclei	PBE0																						
**C1**	136.4686	137.4247	138.0416	137.8938	140.0424	137.8360	139.5797	137.7866	139.5513	137.9591	139.8415	136.4685	137.4247	138.0416	137.8937	140.0424	137.8360	139.5796	137.7866	139.5513	137.9591	139.8415	**133.8**
**C2**	138.6351	138.6103	139.1605	138.4797	139.0718	138.4833	139.5175	138.4891	139.3957	138.4794	139.4670	138.6353	138.6103	139.1605	138.4797	139.0718	138.4833	139.5175	138.4891	139.3957	138.4794	139.4670	**132.1**
**C3**	120.8260	121.5877	122.1674	121.8254	122.5810	121.8075	122.9492	121.7912	122.8216	121.8451	122.9893	120.8261	121.5877	122.1674	121.8254	122.5810	121.8075	122.9492	121.7912	122.8216	121.8451	122.9893	**115.8**
**C4**	172.9139	173.6981	174.3281	174.0689	174.7360	174.0417	175.4226	174.0161	175.2162	174.0971	175.4854	172.9140	173.6981	174.3280	174.0689	174.7360	174.0417	175.4226	174.0161	175.2162	174.0971	175.4854	**165.8**
**C5**	118.8731	120.2602	120.8776	120.9475	121.8402	120.8988	122.1400	120.8521	121.9716	120.9970	122.2425	118.8732	120.2602	120.8776	120.9477	121.8402	120.8988	122.1401	120.8521	121.9716	120.9970	122.2426	**115.8**
**C6**	134.8944	136.7405	137.8709	137.7454	139.0196	137.6852	139.6731	137.6246	139.3535	137.8034	139.9155	134.8944	136.7405	137.8709	137.7454	139.0196	137.6852	139.6731	137.6246	139.3535	137.8034	139.9155	**132.1**
**C7**	201.1327	205.7535	206.7477	208.2864	209.4138	208.1016	214.5384	207.9254	213.2700	208.4730	215.6100	201.1327	205.7535	206.7477	208.2866	209.4138	208.1016	214.5385	207.9254	213.2700	208.4730	215.6100	**198.9**
**C8**	63.3643	64.5588	65.1497	65.2013	66.1093	65.1470	66.8326	65.0978	66.5623	65.2595	67.2452	63.3643	64.5588	65.1497	65.2013	66.1093	65.1470	66.8326	65.0978	66.5623	65.2595	67.2452	**69.3**
**C9**	36.7029	36.5564	36.7690	36.3980	36.5819	36.4075	36.7407	36.4178	36.6034	36.3913	36.8157	36.7029	36.5564	36.7690	36.3981	36.5819	36.4075	36.7408	36.4178	36.6033	36.3913	36.8157	**31.5**
**C10**	18.3038	18.7809	19.0349	19.0204	19.4473	19.0017	19.4138	18.9846	19.3219	19.0409	19.5600	18.3039	18.7809	19.0349	19.0204	19.4473	19.0017	19.4138	18.9846	19.3219	19.0409	19.5600	**19.9**
**C11**	15.6012	15.7676	15.8515	15.7904	16.0036	15.7839	16.0273	15.7793	15.9694	15.7998	16.1344	15.6012	15.7676	15.8515	15.7904	16.0036	15.7839	16.0273	15.7793	15.9694	15.7998	16.1344	**14.3**
**C1`**	50.3555	51.1875	51.3998	51.6650	52.1959	51.6290	52.2187	51.5955	52.1195	51.7027	52.6818	54.6265	55.5628	55.9223	56.0328	56.5940	55.9957	56.8284	55.9612	56.7115	56.0716	57.0056	**50.6**
**C2`**	24.3377	24.7374	25.0025	24.9136	25.3137	24.8961	25.4208	24.8808	25.3336	24.9338	25.2433	23.7806	24.0065	24.2873	24.1073	24.6266	24.0951	24.6313	24.0848	24.5689	24.1218	24.4625	**23.8**
**C3`**	23.7805	24.0065	24.2874	24.1073	24.6265	24.0951	24.6313	24.0848	24.5689	24.1218	24.4625	24.3377	24.7374	25.0026	24.9137	25.3137	24.8961	25.4208	24.8808	25.3336	24.9338	25.2433	**23.8**
**C4`**	54.6265	55.5628	55.9220	56.0328	56.5940	55.9957	56.8284	55.9612	56.7115	56.0716	57.0056	50.3556	51.1875	51.3998	51.6650	52.1959	51.6290	52.2187	51.5955	52.1195	51.7027	52.6818	**50.6**
**H2**	8.7662	8.6573	8.6954	8.5831	8.5346	8.5899	8.5448	8.5961	8.5475	8.5757	8.5295	8.7662	8.6573	8.6954	8.5831	8.5346	8.5899	8.5448	8.5961	8.5475	8.5757	8.5295	**8.35**
**H3**	7.3496	7.4870	7.5780	7.5310	7.6523	7.5287	7.6774	7.5264	7.6664	7.5327	7.6913	7.3496	7.4870	7.5780	7.5310	7.6523	7.5287	7.6774	7.5264	7.6664	7.5327	7.6913	**7.06**
**H5**	7.1827	7.3750	7.4666	7.4573	7.5830	7.4525	7.6019	7.4477	7.5873	7.4615	7.6192	7.1826	7.3750	7.4666	7.4573	7.5830	7.4525	7.6019	7.4477	7.5873	7.4615	7.6193	**7.06**
**H6**	8.0968	8.2654	8.3625	8.3511	8.4526	8.3470	8.5011	8.3425	8.4796	8.3543	8.5197	8.0968	8.2654	8.3625	8.3511	8.4526	8.3470	8.5011	8.3425	8.4796	8.3543	8.5197	**8.35**
**H8**	4.5595	4.6199	4.6513	4.6501	4.7223	4.6486	4.7730	4.6468	4.7544	4.6509	4.7634	4.5595	4.6199	4.6513	4.6501	4.7223	4.6486	4.7730	4.6468	4.7544	4.6509	4.7634	**4.88**
**H(9A-9B)**	1.7092	1.7507	1.7536	1.7754	1.7379	1.7750	1.7243	1.7743	1.7170	1.7752	1.7538	1.7092	1.7507	1.7536	1.7754	1.7379	1.7750	1.7243	1.7743	1.7170	1.7752	1.7538	**2.05**
**H(10A-10B)**	1.3665	1.2504	1.2064	1.1804	1.1095	1.1851	1.1324	1.1898	1.1348	1.1754	1.1162	1.3665	1.2504	1.2064	1.1804	1.1095	1.1851	1.1324	1.1898	1.1348	1.1754	1.1162	**1.36**
**H(11A-11C)**	0.7350	0.7375	0.7143	0.7399	0.7000	0.7399	0.7067	0.7399	0.7057	0.7395	0.6936	0.7350	0.7375	0.7144	0.7399	0.7000	0.7399	0.7067	0.7399	0.7057	0.7395	0.6936	**0.77**
**H(1`A)**	2.6575	2.6629	2.6444	2.6475	2.6041	2.6487	2.6218	2.6498	2.6195	2.6460	2.5912	2.7854	2.7861	2.7694	2.7720	2.7399	2.7726	2.7591	2.7734	2.7558	2.7711	2.7274	**3.03**
**H(1`B)**	3.1658	3.2038	3.2332	3.2306	3.2859	3.2288	3.2718	3.2269	3.2660	3.2321	3.2667	2.8630	2.7224	2.6881	2.6752	2.6893	2.6767	2.6504	2.6785	2.6477	2.6738	2.6351	**3.23**
**H(2`-3`A-2`-3`B)**	1.6432	1.7002	1.6634	1.7350	1.6970	1.7325	1.7235	1.7300	1.7158	1.7372	1.7470	1.6432	1.7002	1.6634	1.7350	1.6970	1.7325	1.7235	1.7300	1.7158	1.7372	1.7470	**1.77**
**H(4`A)**	2.8630	2.7224	2.6881	2.6752	2.6893	2.6767	2.6504	2.6785	2.6477	2.6738	2.6351	3.1658	3.2038	3.2332	3.2306	3.2859	3.2288	3.2718	3.2269	3.2660	3.2321	3.2667	**3.03**
**H(4`B)**	2.7854	2.7861	2.7694	2.7720	2.7399	2.7726	2.7591	2.7734	2.7558	2.7711	2.7274	2.6575	2.6629	2.6444	2.6475	2.6041	2.6487	2.6218	2.6498	2.6195	2.6460	2.5912	**3.23**

[a] methyl protons shifts are averaged

[b] according to [4]

Table S4. Cont. XII

Nuclei	R											S											**Exp.** [b]
	TPSS																						
	Gas	$CHCl_3$ (PCM)	$CHCl_3$ (SMD)	DMSO (PCM)	DMSO (SMD)	CH_3OH (PCM)	CH_3OH (SMD)	C_2H_5OH (PCM)	C_2H_5OH (SMD)	H_2O (PCM)	H_2O (SMD)	Gas	$CHCl_3$ (PCM)	$CHCl_3$ (SMD)	DMSO (PCM)	DMSO (SMD)	CH_3OH (PCM)	CH_3OH (SMD)	C_2H_5OH (PCM)	C_2H_5OH (SMD)	H_2O (PCM)	H_2O (SMD)	
**C1**	132.4252	133.2606	134.0626	133.5282	135.4463	133.5171	135.0853	133.5182	134.0244	133.5502	135.4091	132.4252	133.2606	134.0627	133.5282	135.4462	133.5171	135.0853	133.5182	134.6273	133.5502	135.4091	**133.8**
**C2**	132.8716	132.8075	133.4662	132.7281	133.3610	132.7403	133.7796	132.7642	133.2674	132.7268	133.7640	132.8716	132.8075	133.4663	132.7281	133.3609	132.7403	133.7796	132.7642	133.8789	132.7268	133.7641	**132.1**
**C3**	116.5341	117.2014	117.8434	117.4791	118.2171	117.4709	118.5555	117.4740	117.9160	117.4971	118.6079	116.5341	117.2014	117.8434	117.4791	118.2170	117.4709	118.5555	117.4740	118.7034	117.4971	118.6079	**115.8**
**C4**	170.8720	171.6565	172.4080	172.1110	172.9485	172.0898	173.5447	172.0799	172.7403	172.1417	173.6048	170.8720	171.6565	172.4079	172.1110	172.9486	172.0898	173.5447	172.0799	172.8997	172.1417	173.6048	**165.8**
**C5**	114.7873	116.0079	116.6761	116.6918	117.5648	116.6561	117.8009	116.6318	117.0704	116.7361	117.9140	114.7873	116.0079	116.6761	116.6918	117.5649	116.6561	117.8009	116.6318	117.8675	116.7362	117.9141	**115.8**
**C6**	129.5145	131.2357	132.3650	132.3133	133.5944	132.2529	134.1706	132.2038	132.7047	132.3812	134.4509	129.5145	131.2357	132.3650	132.3133	133.5945	132.2529	134.1706	132.2038	133.3227	132.3812	134.4509	**132.1**
**C7**	197.3886	201.4786	202.7123	203.5325	204.4931	203.4121	208.9449	203.3036	204.0889	203.6581	210.1722	197.3885	201.4786	202.7123	203.5325	204.4930	203.4121	208.9449	203.3036	203.8893	203.6581	210.1722	**198.9**
**C8**	66.1175	67.2102	68.0808	67.7179	68.4103	67.6925	69.1666	67.6788	67.9216	67.7529	69.8127	66.1175	67.2102	68.0807	67.7179	68.4103	67.6925	69.1666	67.6788	69.2817	67.7529	69.8127	**69.3**
**C9**	38.9328	38.7405	39.1060	38.6825	38.7429	38.6953	39.0004	38.7189	38.8459	38.6805	39.2532	38.9328	38.7405	39.1059	38.6825	38.7429	38.6953	39.0004	38.7189	40.5390	38.6805	39.2532	**31.5**
**C10**	20.8961	21.2537	21.6284	21.5187	21.9347	21.5080	21.8224	21.5088	21.5669	21.5395	22.0027	20.8961	21.2537	21.6284	21.5187	21.9346	21.5080	21.8224	21.5088	23.4579	21.5396	22.0026	**19.9**
**C11**	16.7316	16.8366	17.0508	16.9122	17.1364	16.9137	17.1088	16.9269	16.9667	16.9213	17.2672	16.7316	16.8366	17.0508	16.9122	17.1364	16.9137	17.1088	16.9269	18.9104	16.9214	17.2672	**14.3**
**C1`**	53.0734	53.7846	54.1144	54.2751	54.7525	54.2483	54.7593	54.2333	54.4223	54.3119	55.2518	57.2647	58.1927	58.6434	58.6885	59.3602	58.6623	59.5451	58.6481	60.3187	58.7241	59.6616	**50.6**
**C2`**	26.3500	26.7405	27.1281	26.9606	27.3558	26.9524	27.4021	26.9558	27.0357	26.9793	27.2559	26.0130	26.2008	26.5733	26.3792	26.9618	26.3743	26.8919	26.3809	28.2935	26.3945	26.7492	**23.8**
**C3`**	26.0130	26.2008	26.5733	26.3792	26.9619	26.3743	26.8919	26.3809	26.4585	26.3945	26.7491	26.3500	26.7405	27.1281	26.9606	27.3561	26.9524	27.4021	26.9558	28.8641	26.9792	27.2559	**23.8**
**C4`**	57.2647	58.1927	58.6434	58.6885	59.3602	58.6623	59.5451	58.6481	58.8548	58.7242	59.6616	53.0734	53.7846	54.1144	54.2751	54.7524	54.2483	54.7593	54.2333	55.9370	54.3118	55.2518	**50.6**
**H2**	8.5444	8.4411	8.4735	8.3949	8.3711	8.3981	8.3698	8.4018	8.2418	8.3922	8.3436	8.5444	8.4411	8.4735	8.3949	8.3711	8.3981	8.3698	8.4018	8.5120	8.3922	8.3436	**8.35**
**H3**	7.1571	7.2918	7.3886	7.3399	7.4688	7.3375	7.4891	7.3357	7.1714	7.3425	7.5004	7.1571	7.2918	7.3886	7.3399	7.4688	7.3375	7.4891	7.3357	7.4539	7.3425	7.5004	**7.06**
**H5**	6.9980	7.1862	7.2820	7.2712	7.4032	7.2664	7.4155	7.2622	7.0977	7.2761	7.4309	6.9980	7.1862	7.2820	7.2712	7.4032	7.2664	7.4155	7.2622	7.3809	7.2761	7.4309	**7.06**
**H6**	7.8769	8.0448	8.1304	8.1465	8.2585	8.1404	8.2937	8.1348	7.9737	8.1527	8.3050	7.8769	8.0448	8.1304	8.1465	8.2585	8.1404	8.2937	8.1348	8.2470	8.1527	8.3050	**8.35**
**H8**	4.7660	4.8208	4.8400	4.8620	4.9500	4.8596	4.9722	4.8577	4.6835	4.8647	4.9519	4.7661	4.8208	4.8400	4.8620	4.9500	4.8596	4.9722	4.8577	4.9945	4.8647	4.9519	**4.88**
**H(9A-9B)**	1.7801	1.8251	1.8387	1.8626	1.8155	1.8604	1.8120	1.8588	1.6726	1.8649	1.8574	1.7801	1.8251	1.8387	1.8626	1.8156	1.8604	1.8120	1.8588	2.0180	1.8649	1.8574	**2.05**
**H(10A-10B)**	1.4435	1.3291	1.2828	1.2725	1.2165	1.2763	1.2267	1.2806	1.0921	1.2692	1.1965	1.4435	1.3291	1.2828	1.2725	1.2165	1.2763	1.2267	1.2806	1.4442	1.2692	1.1965	**1.36**
**H(11A-11C)**	0.7625	0.7665	0.7452	0.7778	0.7499	0.7771	0.7503	0.7771	0.5866	0.7787	0.7325	0.7625	0.7665	0.7452	0.7778	0.7499	0.7771	0.7503	0.7771	0.9445	0.7787	0.7325	**0.77**
**H(1`A)**	2.7065	2.7209	2.7113	2.7166	2.6760	2.7172	2.6875	2.7184	2.6931	2.7164	2.6608	2.8208	2.8333	2.8304	2.8224	2.8015	2.8234	2.8121	2.8250	3.0511	2.8218	2.6608	**3.03**
**H(1`B)**	3.2753	3.3107	3.3498	3.3428	3.4055	3.3408	3.3884	3.3394	3.3693	3.3451	3.3789	3.0362	2.8613	2.8506	2.8046	2.7992	2.8080	2.7659	2.8120	2.9382	2.8017	3.3789	**3.23**
**H(2`-3`A-2`-3`B)**	1.6803	1.7391	1.7096	1.7781	1.7481	1.7756	1.7695	1.7736	1.5871	1.7810	1.7915	1.6803	1.7391	1.7096	1.7781	1.7481	1.7756	1.7695	1.7736	1.5871	1.7810	1.7915	**1.77**
**H(4`A)**	3.0362	2.8613	2.8506	2.8046	2.7992	2.8080	2.7659	2.8120	2.9382	2.8018	2.7647	3.2754	3.3107	3.3498	3.3428	3.4055	3.3408	3.3884	3.3394	3.3693	3.3451	2.7648	**3.03**
**H(4`B)**	2.8208	2.8333	2.8304	2.8224	2.8015	2.8234	2.8121	2.8250	3.0511	2.8218	2.7898	2.7065	2.7209	2.7113	2.7166	2.6760	2.7172	2.6875	2.7184	2.6931	2.7164	2.7898	**3.23**
Nuclei	**VSXC**																						
**C1**	132.9799	133.5448	134.3194	133.7889	135.4306	133.7784	134.5787	133.7656	134.8603	133.8001	135.1087	132.9799	133.5448	134.3194	133.7889	135.4306	133.7784	134.5787	133.7656	134.8603	133.8001	135.1087	**133.8**
**C2**	130.3508	130.4696	131.0673	130.4284	130.9709	130.4361	131.5414	130.4416	131.4163	130.4222	131.6461	130.3508	130.4696	131.0673	130.4284	130.9709	130.4361	131.5414	130.4416	131.4163	130.4222	131.6461	**132.1**
**C3**	114.7609	115.8434	116.3799	116.2619	116.9330	116.2424	117.2859	116.2202	117.1824	116.2818	117.3883	114.7609	115.8434	116.3799	116.2619	116.9330	116.2424	117.2859	116.2202	117.1824	116.2818	117.3883	**115.8**
**C4**	168.2029	169.1716	169.8086	169.6749	170.3621	169.6471	170.9472	169.6172	170.7674	169.7030	171.0735	168.2029	169.1716	169.8086	169.6749	170.3621	169.6471	170.9472	169.6172	170.7674	169.7029	171.0735	**165.8**
**C5**	112.8866	114.3236	114.8634	115.1078	115.8096	115.0619	116.1278	115.0141	115.9813	115.1529	116.2640	112.8866	114.3236	114.8634	115.1078	115.8096	115.0619	116.1278	115.0141	115.9813	115.1530	116.2640	**115.8**
**C6**	128.8107	130.4481	131.4649	131.4583	132.7728	131.3958	133.3057	131.3316	133.0226	131.5198	133.4595	128.8107	130.4481	131.4649	131.4583	132.7728	131.3958	133.3057	131.3316	133.0226	131.5198	133.4595	**132.1**
**C7**	193.4717	198.3010	199.5199	200.7253	201.5793	200.5819	205.7683	200.4360	204.9833	200.8623	207.0019	193.4717	198.3010	199.5199	200.7253	201.5793	200.5819	205.7683	200.4360	204.9833	200.8623	207.0019	**198.9**
**C8**	71.7209	72.5535	73.1801	72.9376	73.8113	72.9181	74.1060	72.8964	74.0842	72.9575	74.6516	71.7209	72.5535	73.1801	72.9376	73.8113	72.9181	74.1060	72.8964	74.0842	72.9574	74.6516	**69.3**
**C9**	44.3091	44.8449	45.1242	45.1109	45.3318	45.0974	45.8396	45.0821	45.6858	45.1252	45.9770	44.3091	44.8449	45.1242	45.1109	45.3318	45.0974	45.8396	45.0821	45.6858	45.1252	45.9769	**31.5**
**C10**	25.3631	26.3064	26.4506	26.8102	27.2910	26.7810	27.3351	26.7499	27.2336	26.8398	27.4496	25.3631	26.3064	26.4506	26.8102	27.2910	26.7810	27.3351	26.7499	27.2336	26.8398	27.4496	**19.9**
**C11**	20.7378	20.7695	20.8784	20.7890	20.9061	20.7909	20.8367	20.7906	20.8679	20.7890	20.9332	20.7378	20.7695	20.8784	20.7890	20.9061	20.7909	20.8367	20.7906	20.8679	20.7890	20.9332	**14.3**
**C1`**	58.2226	58.8627	59.2141	59.2518	59.7613	59.2296	59.6047	59.2056	59.5721	59.2745	59.7499	61.2855	62.0083	62.3372	62.3910	62.9295	62.3699	62.9168	62.3471	62.8547	62.4125	63.2144	**50.6**
**C2`**	30.1698	30.5938	30.8097	30.8407	31.1892	30.8292	31.2109	30.8153	31.2002	30.8532	31.3605	28.0487	28.4859	28.7670	28.7405	29.1785	28.7278	29.1540	28.7129	29.1473	28.7542	29.2562	**23.8**
**C3`**	28.0487	28.4859	28.7670	28.7405	29.1785	28.7278	29.1540	28.7129	29.1473	28.7542	29.2562	30.1698	30.5938	30.8097	30.8407	31.1892	30.8292	31.2109	30.8153	31.2002	30.8532	31.3605	**23.8**
**C4`**	61.2855	62.0083	62.3372	62.3910	62.9295	62.3699	62.9168	62.3471	62.8547	62.4125	63.2144	58.2226	58.8627	59.2141	59.2518	59.7613	59.2296	59.6047	59.2056	59.5721	59.2745	59.7499	**50.6**
**H2**	8.5043	8.5259	8.5340	8.5083	8.4923	8.5098	8.5615	8.5113	8.5296	8.5069	8.5306	8.5043	8.5259	8.5340	8.5083	8.4923	8.5098	8.5615	8.5113	8.5296	8.5069	8.5306	**8.35**
**H3**	7.2041	7.3685	7.4558	7.4223	7.5339	7.4196	7.5584	7.4170	7.5441	7.4248	7.5718	7.2041	7.3685	7.4558	7.4223	7.5339	7.4196	7.5584	7.4170	7.5441	7.4248	7.5718	**7.06**
**H5**	7.0368	7.2361	7.3195	7.3244	7.4440	7.3193	7.4612	7.3143	7.4505	7.3292	7.4831	7.0368	7.2361	7.3195	7.3244	7.4440	7.3193	7.4612	7.3143	7.4505	7.3292	7.4831	**7.06**
**H6**	7.9402	8.0804	8.1629	8.1670	8.2799	8.1613	8.3258	8.1558	8.2996	8.1724	8.3474	7.9402	8.0804	8.1629	8.1670	8.2799	8.1613	8.3258	8.1558	8.2996	8.1724	8.3474	**8.35**
**H8**	4.3488	4.4446	4.4830	4.5059	4.5848	4.5020	4.6379	4.4982	4.6203	4.5097	4.6646	4.3488	4.4446	4.4830	4.5059	4.5848	4.5020	4.6379	4.4982	4.6203	4.5096	4.6646	**4.88**
**H(9A-9B)**	1.9468	1.9563	1.9635	1.9717	1.9486	1.9705	1.9534	1.9695	1.9460	1.9728	1.9245	1.9468	1.9563	1.9635	1.9717	1.9486	1.9705	1.9534	1.9695	1.9460	1.9728	1.9245	**2.05**
**H(10A-10B)**	1.6549	1.5515	1.5141	1.4862	1.4021	1.4904	1.3954	1.4949	1.4045	1.4821	1.3472	1.6549	1.5515	1.5141	1.4862	1.4021	1.4904	1.3954	1.4949	1.4045	1.4821	1.3472	**1.36**
**H(11A-11C)**	1.0078	1.0219	1.0067	1.0310	0.9982	1.0304	1.0023	1.0300	1.0022	1.0315	0.9938	1.0078	1.0219	1.0067	1.0310	0.9982	1.0304	1.0023	1.0300	1.0022	1.0315	0.9938	**0.77**
**H(1`A)**	2.5129	2.5608	2.5584	2.5744	2.5569	2.5738	2.5791	2.5733	2.5697	2.5749	2.5805	2.9472	2.9433	2.9265	2.9411	2.9221	2.9414	2.9226	2.9418	2.9277	2.9408	2.9271	**3.03**
**H(1`B)**	3.1586	3.1786	3.2131	3.1874	3.2636	3.1870	3.2590	3.1867	3.2459	3.1878	3.2656	2.8507	2.8087	2.8095	2.8060	2.8161	2.8059	2.8079	2.8060	2.8148	2.8060	2.8195	**3.23**
**H(2`-3`A-2`-3`B)**	1.9969	2.0391	2.0113	2.0663	2.0287	2.0645	2.0435	2.0630	2.0389	2.0679	2.0571	1.9969	2.0391	2.0113	2.0663	2.0287	2.0645	2.0435	2.0630	2.0389	2.0679	2.0571	**1.77**
**H(4`A)**	2.8507	2.8087	2.8095	2.8060	2.8161	2.8059	2.8079	2.8060	2.8148	2.8060	2.8195	3.1586	3.1786	3.2131	3.1874	3.2636	3.1870	3.2590	3.1867	3.2459	3.1878	3.2656	**3.03**
**H(4`B)**	2.9472	2.9433	2.9265	2.9411	2.9221	2.9414	2.9226	2.9418	2.9277	2.9408	2.9271	2.5129	2.5608	2.5584	2.5744	2.5569	2.5738	2.5791	2.5733	2.5697	2.5749	2.5805	**3.23**

[a] methyl protons shifts are averaged

[b] according to [4]

Table S4. Cont. XIII

Nuclei	R											S											Exp. [b]
	wB97																						
	Gas	$CHCl_3$ (PCM)	$CHCl_3$ (SMD)	DMSO (PCM)	DMSO (SMD)	CH_3OH (PCM)	CH_3OH (SMD)	C_2H_5OH (PCM)	C_2H_5OH (SMD)	H_2O (PCM)	H_2O (SMD)	Gas	$CHCl_3$ (PCM)	$CHCl_3$ (SMD)	DMSO (PCM)	DMSO (SMD)	CH_3OH (PCM)	CH_3OH (SMD)	C_2H_5OH (PCM)	C_2H_5OH (SMD)	H_2O (PCM)	H_2O (SMD)	
**C1**	136.5194	137.5694	138.0796	137.9903	139.5805	137.9535	139.3576	137.9197	139.4471	138.0303	139.7190	136.5194	137.5694	138.0796	137.9903	139.5805	137.9535	139.3576	137.9197	139.4471	138.0302	139.7190	**133.8**
**C2**	140.4269	140.3358	140.8691	140.1553	140.7191	140.1691	140.9927	140.1817	140.8869	140.1416	140.9891	140.4269	140.3358	140.8691	140.1553	140.7191	140.1691	140.9927	140.1817	140.8869	140.1415	140.9892	**132.1**
**C3**	121.5174	122.1379	122.6998	122.3565	123.0564	122.3436	123.3074	122.3300	123.2520	122.3693	123.3970	121.5174	122.1379	122.6998	122.3565	123.0564	122.3434	123.3074	122.3299	123.2520	122.3694	123.3970	**115.8**
**C4**	174.2894	174.8437	175.4986	175.1890	175.8128	175.1664	176.3604	175.1432	176.2124	175.2102	176.4604	174.2894	174.8437	175.4986	175.1888	175.8128	175.1664	176.3604	175.1432	176.2124	175.2102	176.4604	**165.8**
**C5**	119.5219	120.7990	121.3961	121.4978	122.3313	121.4508	122.6135	121.4043	122.5189	121.5434	122.7700	119.5219	120.7990	121.3960	121.4976	122.3313	121.4510	122.6135	121.4043	122.5189	121.5434	122.7701	**115.8**
**C6**	136.6630	138.2616	139.4472	139.3054	140.8374	139.2411	141.0639	139.1751	140.8231	139.3653	141.3891	136.6630	138.2616	139.4472	139.3053	140.8374	139.2411	141.0639	139.1751	140.8231	139.3654	141.3891	**132.1**
**C7**	201.4834	206.6139	207.4634	209.4417	209.8309	209.2532	215.9182	209.0689	214.6939	209.6265	217.1938	201.4834	206.6139	207.4634	209.4417	209.8309	209.2532	215.9182	209.0689	214.6939	209.6263	217.1939	**198.9**
**C8**	61.9076	63.1189	63.5285	63.7225	64.5287	63.6812	64.9748	63.6405	64.8162	63.7635	65.4950	61.9077	63.1189	63.5284	63.7226	64.5287	63.6812	64.9748	63.6405	64.8162	63.7633	65.4951	**69.3**
**C9**	34.9590	34.7431	34.9674	34.6100	34.9988	34.6200	34.7266	34.6286	34.6954	34.5998	34.9146	34.9590	34.7431	34.9674	34.6099	34.9988	34.6200	34.7266	34.6286	34.6954	34.5998	34.9145	**31.5**
**C10**	18.2182	18.6328	18.8582	18.8774	19.3824	18.8618	19.1645	18.8453	19.1393	18.8923	19.3335	18.2182	18.6328	18.8582	18.8774	19.3824	18.8618	19.1645	18.8453	19.1393	18.8923	19.3335	**19.9**
**C11**	15.6605	15.7400	15.8627	15.7845	16.0377	15.7803	15.9543	15.7755	15.9469	15.7889	16.0956	15.6605	15.7400	15.8627	15.7845	16.0377	15.7803	15.9543	15.7755	15.9469	15.7890	16.0956	**14.3**
**C1`**	49.2054	49.9067	50.0783	50.3643	50.9579	50.3319	50.8766	50.2996	50.8098	50.3962	51.3133	52.9405	53.7375	54.0643	54.1917	54.7052	54.1598	54.8156	54.1280	54.7336	54.2229	55.0766	**50.6**
**C2`**	23.7861	24.0935	24.3287	24.2781	24.6956	24.2639	24.6624	24.2495	24.6296	24.2923	24.6415	23.1695	23.2934	23.6310	23.4056	23.8877	23.3972	23.8486	23.3880	23.8438	23.4142	23.7404	**23.8**
**C3`**	23.1695	23.2934	23.6310	23.4057	23.8877	23.3971	23.8486	23.3880	23.8438	23.4141	23.7402	23.7861	24.0935	24.3286	24.2780	24.6956	24.2639	24.6624	24.2495	24.6296	24.2923	24.6415	**23.8**
**C4`**	52.9405	53.7375	54.0643	54.1917	54.7052	54.1598	54.8156	54.1280	54.7336	54.2230	55.0770	49.2054	49.9067	50.0784	50.3643	50.9579	50.3319	50.8766	50.2996	50.8098	50.3961	51.3132	**50.6**
**H2**	8.8681	8.7541	8.8099	8.6731	8.6884	8.6796	8.6378	8.6854	8.6360	8.6666	8.6181	8.8681	8.7541	8.8099	8.6731	8.6884	8.6796	8.6378	8.6854	8.6360	8.6666	8.6181	**8.35**
**H3**	7.4384	7.5765	7.6668	7.6169	7.7406	7.6149	7.7561	7.6128	7.7479	7.6187	7.7730	7.4384	7.5765	7.6668	7.6169	7.7406	7.6149	7.7561	7.6128	7.7479	7.6187	7.7730	**7.06**
**H5**	7.2883	7.4763	7.5666	7.5600	7.6882	7.5550	7.7092	7.5498	7.6977	7.5649	7.7331	7.2883	7.4763	7.5666	7.5600	7.6882	7.5550	7.7092	7.5498	7.6977	7.5649	7.7331	**7.06**
**H6**	8.1624	8.3146	8.4330	8.4072	8.5462	8.4017	8.5655	8.3959	8.5452	8.4123	8.5874	8.1624	8.3146	8.4330	8.4072	8.5462	8.4017	8.5655	8.3959	8.5452	8.4123	8.5874	**8.35**
**H8**	4.2134	4.2738	4.3408	4.3252	4.4025	4.3216	4.4817	4.3180	4.4615	4.3285	4.4800	4.2134	4.2738	4.3408	4.3252	4.4025	4.3216	4.4817	4.3180	4.4615	4.3285	4.4800	**4.88**
**H(9A-9B)**	1.6731	1.7129	1.7065	1.7374	1.7556	1.7364	1.6776	1.7352	1.6726	1.7380	1.7178	1.6731	1.7129	1.7065	1.7374	1.7556	1.7364	1.6776	1.7352	1.6726	1.7381	1.7179	**2.05**
**H(10A-10B)**	1.3691	1.2281	1.2066	1.1519	1.0911	1.1568	1.1179	1.1616	1.1213	1.1472	1.1010	1.3691	1.2281	1.2066	1.1520	1.0911	1.1568	1.1179	1.1616	1.1213	1.1472	1.1010	**1.36**
**H(11A-11C)**	0.7709	0.7638	0.7486	0.7647	0.7310	0.7646	0.7311	0.7644	0.7315	0.7648	0.7203	0.7709	0.7638	0.7486	0.7647	0.7310	0.7646	0.7311	0.7644	0.7315	0.7648	0.7203	**0.77**
**H(1`A)**	2.6214	2.6146	2.6021	2.6010	2.5630	2.6020	2.5787	2.6029	2.5789	2.6000	2.5570	2.7655	2.7690	2.7492	2.7548	2.7238	2.7556	2.7330	2.7563	2.7351	2.7541	2.7077	**3.03**
**H(1`B)**	3.0521	3.0845	3.1117	3.1168	3.1755	3.1146	3.1592	3.1122	3.1545	3.1191	3.1653	2.7316	2.6336	2.5807	2.5957	2.5927	2.5976	2.5646	2.5995	2.5663	2.5941	2.5585	**3.23**
**H(2`-3`A-2`-3`B)**	1.6363	1.6962	1.6604	1.7316	1.6915	1.7290	1.7197	1.7265	1.7131	1.7341	1.7428	1.6363	1.6962	1.6603	1.7316	1.6915	1.7290	1.7197	1.7265	1.7131	1.7341	1.7428	**1.77**
**H(4`A)**	2.7316	2.6336	2.5807	2.5957	2.5927	2.5976	2.5646	2.5995	2.5663	2.5941	2.5585	3.0521	3.0845	3.1117	3.1168	3.1755	3.1146	3.1592	3.1122	3.1545	3.1190	3.1653	**3.03**
**H(4`B)**	2.7655	2.7690	2.7492	2.7548	2.7238	2.7556	2.7330	2.7563	2.7351	2.7541	2.7077	2.6214	2.6146	2.6020	2.6010	2.5630	2.6020	2.5787	2.6029	2.5789	2.6000	2.5570	**3.23**
Nuclei	wB97X																						
**C1**	137.9766	139.0348	139.5615	139.7498	141.9038	139.6550	141.1181	139.5647	141.0649	139.8295	141.4398	137.9765	139.0348	139.5616	139.7498	141.9038	139.6550	141.1181	139.5647	141.0649	139.8293	141.4398	**133.8**
**C2**	140.8355	140.7633	141.2870	140.5879	141.0754	140.6068	141.5044	140.6243	141.3594	140.5719	141.4950	140.8354	140.7633	141.2869	140.5879	141.0754	140.6068	141.5044	140.6242	141.3594	140.5719	141.4950	**132.1**
**C3**	122.2364	122.8727	123.4342	123.1085	123.8518	123.0954	124.0788	123.0811	123.9538	123.1210	124.1559	122.2364	122.8727	123.4341	123.1085	123.8518	123.0954	124.0788	123.0813	123.9538	123.1209	124.1559	**115.8**
**C4**	174.9612	175.5579	176.2171	175.8788	176.4658	175.8659	177.0811	175.8512	176.8817	175.8930	177.1775	174.9614	175.5579	176.2170	175.8788	176.4658	175.8659	177.0811	175.8512	176.8817	175.8930	177.1775	**165.8**
**C5**	120.2272	121.5229	122.1239	122.2792	123.1963	122.2251	123.4025	122.1704	123.2419	122.3290	123.5499	120.2271	121.5229	122.1240	122.2792	123.1963	122.2251	123.4025	122.1702	123.2419	122.3290	123.5500	**115.8**
**C6**	137.0717	138.7005	139.8784	139.6360	140.9084	139.5986	141.4835	139.5588	141.2029	139.6782	141.7655	137.0718	138.7005	139.8784	139.6360	140.9084	139.5986	141.4835	139.5588	141.2029	139.6783	141.7655	**132.1**
**C7**	203.1739	208.3154	209.2003	211.3740	212.3025	211.1326	217.7715	210.8968	216.3850	211.5943	218.9603	203.1742	208.3154	209.2004	211.3740	212.3025	211.1326	217.7715	210.8967	216.3850	211.5941	218.9603	**198.9**
**C8**	63.1864	64.3968	64.8287	65.0941	65.9082	65.0391	66.3729	64.9859	66.1122	65.1463	66.8276	63.1863	64.3968	64.8287	65.0941	65.9082	65.0390	66.3729	64.9859	66.1122	65.1462	66.8276	**69.3**
**C9**	35.8375	35.5746	35.7926	35.3700	35.5904	35.3964	35.5769	35.4217	35.4781	35.3494	35.7030	35.8375	35.5746	35.7927	35.3700	35.5904	35.3964	35.5769	35.4216	35.4781	35.3496	35.7030	**31.5**
**C10**	18.3607	18.7927	19.0156	19.0362	19.5064	19.0257	19.3889	19.0143	19.2972	19.0487	19.5359	18.3606	18.7927	19.0155	19.0362	19.5064	19.0257	19.3889	19.0143	19.2972	19.0487	19.5359	**19.9**
**C11**	15.6750	15.7621	15.8796	15.8054	16.0395	15.8041	16.0035	15.8022	15.9383	15.8082	16.1261	15.6751	15.7621	15.8797	15.8054	16.0396	15.8041	16.0035	15.8022	15.9383	15.8082	16.1261	**14.3**
**C1`**	49.9646	50.6995	50.8826	51.1794	51.7532	51.1477	51.6978	51.1157	51.5654	51.2108	52.1324	53.9228	54.7550	55.0967	55.2576	55.7916	55.2231	55.9248	55.1882	55.7834	55.2903	56.1503	**50.6**
**C2`**	24.2807	24.5787	24.8251	24.7702	25.1521	24.7585	25.1748	24.7463	25.0805	24.7824	25.0787	23.6488	23.7758	24.0992	23.8901	24.3927	23.8822	24.3619	23.8736	24.2981	23.8987	24.2530	**23.8**
**C3`**	23.6488	23.7758	24.0992	23.8902	24.3927	23.8822	24.3619	23.8736	24.2981	23.8987	24.2530	24.2806	24.5787	24.8252	24.7702	25.1521	24.7585	25.1748	24.7462	25.0805	24.7824	25.0787	**23.8**
**C4`**	53.9229	54.7550	55.0967	55.2576	55.7916	55.2231	55.9248	55.1883	55.7834	55.2904	56.1503	49.9646	50.6995	50.8825	51.1794	51.7532	51.1477	51.6978	51.1159	51.5654	51.2108	52.1324	**50.6**
**H2**	8.8371	8.7166	8.7711	8.6179	8.5782	8.6296	8.6010	8.6406	8.6048	8.6081	8.5832	8.8371	8.7166	8.7711	8.6179	8.5782	8.6296	8.6010	8.6406	8.6048	8.6081	8.5832	**8.35**
**H3**	7.4169	7.5537	7.6446	7.5961	7.7176	7.5944	7.7371	7.5927	7.7257	7.5978	7.7513	7.4169	7.5537	7.6446	7.5960	7.7176	7.5944	7.7371	7.5927	7.7257	7.5978	7.7513	**7.06**
**H5**	7.2613	7.4493	7.5394	7.5408	7.6738	7.5348	7.6911	7.5289	7.6771	7.5463	7.7124	7.2613	7.4493	7.5395	7.5408	7.6738	7.5348	7.6911	7.5289	7.6771	7.5463	7.7124	**7.06**
**H6**	8.1406	8.2926	8.4087	8.3703	8.4830	8.3681	8.5304	8.3656	8.5119	8.3732	8.5526	8.1406	8.2926	8.4087	8.3703	8.4830	8.3681	8.5304	8.3656	8.5119	8.3732	8.5526	**8.35**
**H8**	4.3072	4.3630	4.4254	4.4076	4.4891	4.4052	4.5561	4.4026	4.5364	4.4101	4.5532	4.3072	4.3630	4.4254	4.4076	4.4891	4.4052	4.5561	4.4026	4.5364	4.4101	4.5532	**4.88**
**H(9A-9B)**	1.6674	1.7042	1.7012	1.7158	1.6854	1.7186	1.6644	1.7211	1.6590	1.7142	1.6960	1.6674	1.7042	1.7012	1.7158	1.6854	1.7186	1.6644	1.7211	1.6590	1.7143	1.6960	**2.05**
**H(10A-10B)**	1.3567	1.2232	1.1944	1.1482	1.0878	1.1535	1.1154	1.1580	1.1170	1.1434	1.0997	1.3566	1.2232	1.1944	1.1482	1.0878	1.1535	1.1154	1.1588	1.1171	1.1434	1.0997	**1.36**
**H(11A-11C)**	0.7629	0.7583	0.7406	0.7593	0.7211	0.7598	0.7285	0.7603	0.7273	0.7591	0.7172	0.7629	0.7583	0.7406	0.7594	0.7211	0.7598	0.7285	0.7603	0.7273	0.7591	0.7172	**0.77**
**H(1`A)**	2.6430	2.6373	2.6231	2.6242	2.5827	2.6256	2.5985	2.6269	2.5966	2.6230	2.5733	2.7821	2.7826	2.7613	2.7723	2.7398	2.7728	2.7497	2.7733	2.7474	2.7720	2.7216	**3.03**
**H(1`B)**	3.0733	3.1032	3.1352	3.1345	3.1889	3.1329	3.1763	3.1312	3.1699	3.1363	3.1779	2.7503	2.6425	2.5949	2.6144	2.6223	2.6149	2.5804	2.6156	2.5770	2.6140	2.5722	**3.23**
**H(2`-3`A-2`-3`B)**	1.6363	1.6972	1.6607	1.7350	1.6982	1.7323	1.7208	1.7296	1.7115	1.7376	1.7435	1.6363	1.6972	1.6607	1.7350	1.6982	1.7323	1.7208	1.7296	1.7115	1.7376	1.7435	**1.77**
**H(4`A)**	2.7503	2.6425	2.5949	2.6144	2.6223	2.6149	2.5804	2.6156	2.5770	2.6120	2.5722	3.0733	3.1032	3.1352	3.1345	3.1889	3.1329	3.1763	3.1312	3.1699	3.1363	3.1779	**3.03**
**H(4`B)**	2.7821	2.7826	2.7613	2.7723	2.7398	2.7728	2.7497	2.7733	2.7474	2.7720	2.7216	2.6430	2.6373	2.6231	2.6242	2.5827	2.6256	2.5985	2.6269	2.5966	2.6230	2.5733	**3.23**

[a] methyl protons shifts are averaged

[b] according to [4]

Table S4. Cont. XIV

Nuclei	R											S											Exp. [b]
	X3LYP																						
	Gas	$CHCl_3$ (PCM)	$CHCl_3$ (SMD)	DMSO (PCM)	DMSO (SMD)	CH_3OH (PCM)	CH_3OH (SMD)	C_2H_5OH (PCM)	C_2H_5OH (SMD)	H_2O (PCM)	H_2O (SMD)	Gas	$CHCl_3$ (PCM)	$CHCl_3$ (SMD)	DMSO (PCM)	DMSO (SMD)	CH_3OH (PCM)	CH_3OH (SMD)	C_2H_5OH (PCM)	C_2H_5OH (SMD)	H_2O (PCM)	H_2O (SMD)	
**C1**	139.6773	140.6339	141.3871	141.3959	143.2104	141.3744	142.6749	141.3492	142.6918	141.4196	143.0874	139.6773	140.6338	141.3870	141.3959	143.2103	141.3743	142.6749	141.3492	142.6917	141.4197	143.0874	**133.8**
**C2**	139.7585	139.7030	140.2707	139.5959	140.1045	139.6071	140.5552	139.6152	140.4809	139.5891	140.5496	139.7585	139.7031	140.2707	139.5959	140.1045	139.6070	140.5553	139.6152	140.4809	139.5892	140.5496	**132.1**
**C3**	121.9861	122.6802	123.2021	122.9487	123.6109	122.9409	123.8915	122.9293	123.8260	122.9597	123.9709	121.9861	122.6802	123.2020	122.9487	123.6108	122.9409	123.8915	122.9294	123.8260	122.9598	123.9709	**115.8**
**C4**	176.3489	177.1519	177.7719	177.5931	178.3081	177.5716	178.8712	177.5469	178.7271	177.6171	178.9724	176.3489	177.1519	177.7719	177.5931	178.3081	177.5717	178.8710	177.5469	178.7272	177.6171	178.9724	**165.8**
**C5**	120.0117	121.3023	121.8748	121.9683	122.7435	121.9330	122.9374	121.8946	122.8381	122.0053	123.0685	120.0117	121.3021	121.8748	121.9683	122.7435	121.9331	122.9374	121.8946	122.8382	122.0053	123.0685	**115.8**
**C6**	136.0777	137.9163	139.0245	138.8770	140.2567	138.8111	140.8782	138.7428	140.6178	138.9433	141.1139	136.0777	137.9163	139.0245	138.8770	140.2567	138.8111	140.8781	138.7429	140.6178	138.9433	141.1139	**132.1**
**C7**	203.8418	208.6857	209.8719	211.5711	212.3697	211.4134	217.6482	211.2522	216.4305	211.7242	218.8619	203.8418	208.6857	209.8719	211.5711	212.3697	211.4134	217.6482	211.2522	216.4305	211.7242	218.8619	**198.9**
**C8**	66.1642	67.3192	68.1329	67.9557	68.6724	67.9148	69.4452	67.8714	69.2516	67.9988	69.8035	66.1642	67.3193	68.1329	67.9557	68.6724	67.9147	69.4452	67.8713	69.2517	67.9988	69.8035	**69.3**
**C9**	38.7646	38.4469	38.7057	38.1661	38.2956	38.1796	38.4813	38.1901	38.4199	38.1570	38.5195	38.7646	38.4469	38.7058	38.1661	38.2956	38.1795	38.4814	38.1901	38.4199	38.1570	38.5195	**31.5**
**C10**	19.9254	20.3688	20.6718	20.6195	21.0584	20.6045	20.9520	20.5865	20.9311	20.6371	21.0703	19.9254	20.3688	20.6717	20.6195	21.0584	20.6046	20.9519	20.5866	20.9311	20.6371	21.0703	**19.9**
**C11**	16.1032	16.2609	16.3934	16.3309	16.5321	16.3298	16.4931	16.3257	16.4949	16.3356	16.6466	16.1032	16.2610	16.3934	16.3309	16.5321	16.3298	16.4930	16.3257	16.4950	16.3357	16.6466	**14.3**
**C1`**	52.1349	52.9699	53.2615	53.5201	54.0202	53.4881	54.0262	53.4530	54.0060	53.5539	54.4800	56.6972	57.6795	58.0349	58.2404	58.8200	58.2115	58.9232	58.1797	58.8550	58.2717	59.1638	**50.6**
**C2`**	25.9378	26.3725	26.6671	26.6054	27.0284	26.5922	27.0481	26.5763	27.0281	26.6219	26.8982	25.4958	25.6991	25.9933	25.8744	26.4624	25.8677	26.3257	25.8582	26.3244	25.8844	26.1388	**23.8**
**C3`**	25.4958	25.6990	25.9933	25.8744	26.4624	25.8675	26.3257	25.8581	26.3244	25.8845	26.1388	25.9378	26.3725	26.6670	26.6054	27.0284	26.5922	27.0481	26.5763	27.0282	26.6220	26.8982	**23.8**
**C4`**	56.6972	57.6795	58.0350	58.2404	58.8199	58.2115	58.9232	58.1798	58.8550	58.2717	59.1638	52.1349	52.9698	53.2616	53.5201	54.0201	53.4880	54.0262	53.4530	54.0060	53.5540	54.4800	**50.6**
**H2**	8.6885	8.5787	8.6123	8.4834	8.4677	8.4876	8.4706	8.4913	8.4769	8.4795	8.4498	8.6885	8.5787	8.6123	8.4834	8.4677	8.4876	8.4706	8.4913	8.4769	8.4795	8.4498	**8.35**
**H3**	7.3083	7.4443	7.5331	7.4918	7.6070	7.4899	7.6238	7.4873	7.6171	7.4935	7.6369	7.3083	7.4443	7.5331	7.4918	7.6070	7.4898	7.6238	7.4873	7.6171	7.4935	7.6369	**7.06**
**H5**	7.1554	7.3386	7.4286	7.4154	7.5358	7.4111	7.5494	7.4063	7.5380	7.4194	7.5670	7.1554	7.3386	7.4286	7.4154	7.5358	7.4111	7.5494	7.4063	7.5380	7.4194	7.5670	**7.06**
**H6**	7.9949	8.1713	8.2504	8.2624	8.3913	8.2566	8.4255	8.2504	8.4072	8.2679	8.4416	7.9949	8.1713	8.2504	8.2624	8.3913	8.2566	8.4255	8.2503	8.4072	8.2679	8.4416	**8.35**
**H8**	4.6134	4.6719	4.6840	4.7119	4.8091	4.7097	4.8283	4.7071	4.8111	4.7141	4.8165	4.6134	4.6719	4.6840	4.7119	4.8091	4.7097	4.8283	4.7071	4.8111	4.7141	4.8165	**4.88**
**H(9A-9B)**	1.6826	1.7269	1.7438	1.7330	1.7085	1.7308	1.7151	1.7280	1.7110	1.7352	1.7357	1.6826	1.7269	1.7438	1.7330	1.7085	1.7308	1.7151	1.7280	1.7110	1.7352	1.7357	**2.05**
**H(10A-10B)**	1.4152	1.3019	1.2490	1.2424	1.1819	1.2468	1.1937	1.2507	1.1977	1.2382	1.1796	1.4152	1.3019	1.2490	1.2424	1.1818	1.2468	1.1937	1.2507	1.1977	1.2382	1.1796	**1.36**
**H(11A-11C)**	0.7841	0.7886	0.7627	0.7950	0.7650	0.7947	0.7644	0.7939	0.7653	0.7954	0.7547	0.7841	0.7887	0.7627	0.7950	0.7650	0.7947	0.7644	0.7939	0.7654	0.7954	0.7547	**0.77**
**H(1`A)**	2.7328	2.7377	2.7159	2.7280	2.6777	2.7289	2.6901	2.7294	2.6907	2.7270	2.6660	2.8620	2.8614	2.8530	2.8464	2.8146	2.8476	2.8262	2.8483	2.8270	2.8453	2.7908	**3.03**
**H(1`B)**	3.1958	3.2381	3.2807	3.2709	3.3390	3.2686	3.3214	3.2659	3.3172	3.2731	3.3189	2.8696	2.7257	2.7170	2.6914	2.6864	2.6934	2.6675	2.6950	2.6661	2.6895	2.6649	**3.23**
**H(2`-3`A-2`-3`B)**	1.6696	1.7279	1.6920	1.7730	1.7330	1.7707	1.7519	1.7678	1.7473	1.7752	1.7781	1.6696	1.7279	1.6920	1.7730	1.7330	1.7707	1.7519	1.7678	1.7473	1.7752	1.7781	**1.77**
**H(4`A)**	2.8696	2.7256	2.7171	2.6914	2.6864	2.6934	2.6675	2.6950	2.6661	2.6895	2.6649	3.1958	3.2382	3.2807	3.2709	3.3390	3.2686	3.3214	3.2659	3.3172	3.2731	3.3189	**3.03**
**H(4`B)**	2.8620	2.8614	2.8530	2.8464	2.8146	2.8476	2.8262	2.8483	2.8270	2.8453	2.7908	2.7328	2.7377	2.7159	2.7280	2.6777	2.7289	2.6901	2.7294	2.6907	2.7270	2.6660	**3.23**

[a] methyl protons shifts are averaged

[b] according to [4]

Table S5. Theoretical chemical shifts (in ppm) of *(R,S)*-4-fluoro-α-pyrrolidinohexanophenone calculated with HF and selected functionals combined with 6-311++G** basis set[a]

Nuclei	R											S											Exp. [b]
	B3LYP																						
	Gas	$CHCl_3$ (PCM)	$CHCl_3$ (SMD)	DMSO (PCM)	DMSO (SMD)	CH_3OH (PCM)	CH_3OH (SMD)	C_2H_5OH (PCM)	C_2H_5OH (SMD)	H_2O (PCM)	H_2O (SMD)	Gas	$CHCl_3$ (PCM)	$CHCl_3$ (SMD)	DMSO (PCM)	DMSO (SMD)	CH_3OH (PCM)	CH_3OH (SMD)	C_2H_5OH (PCM)	C_2H_5OH (SMD)	H_2O (PCM)	H_2O (SMD)	
**C1**	139.6461	140.7864	141.4058	141.3136	142.6443	141.2719	143.6401	141.2321	143.8057	141.3540	142.2745	139.6461	140.7864	141.4058	141.3136	142.6441	141.2719	143.6401	141.2321	142.2849	141.3540	142.2745	**132.2**
**C2**	139.5237	139.5283	140.0799	139.4229	139.8550	139.4233	140.6856	139.4245	140.5631	139.4217	140.3689	139.5237	139.5283	140.0799	139.4229	139.8550	139.4235	140.6856	139.4245	140.3254	139.4215	140.3689	**133.3**
**C3**	122.0016	122.7109	123.2701	122.8762	123.4842	122.8651	123.8294	122.8532	123.7527	122.8848	123.8436	122.0016	122.7109	123.2701	122.8762	123.4843	122.8651	123.8293	122.8532	123.6960	122.8848	123.8436	**117.6**
**C4**	176.3776	177.2686	177.9231	177.6172	178.2715	177.5929	178.7148	177.5682	178.5305	177.6383	179.0153	176.3776	177.2686	177.9231	177.6172	178.2715	177.5929	178.7149	177.5682	178.7108	177.6383	179.0153	**168.3**
**C5**	119.9008	121.1508	121.7637	121.7354	122.5277	121.6950	122.8173	121.6548	122.6947	121.7720	122.9044	119.9008	121.1508	121.7637	121.7355	122.5277	121.6952	122.8174	121.6548	122.6376	121.7721	122.9044	**117.6**
**C6**	135.7284	137.7097	138.8506	138.7726	140.3125	138.7018	140.2739	138.6307	139.9664	138.8376	141.3542	135.7284	137.7097	138.8506	138.7726	140.3125	138.7019	140.2738	138.6307	140.6449	138.8377	141.3542	**133.3**
**C7**	203.8433	208.7706	209.8361	211.3608	211.9257	211.1952	218.3779	211.0306	217.2932	211.5170	218.0294	203.8433	208.7706	209.8361	211.3607	211.9257	211.1952	218.3780	211.0306	215.9741	211.5172	218.0294	**195.8**
**C8**	66.5250	67.8264	68.3186	68.2996	69.3467	68.2734	70.0962	68.2461	69.9127	68.3213	70.3036	66.5250	67.8264	68.3186	68.2996	69.3467	68.2734	70.0962	68.2461	69.8306	68.3213	70.3036	**70.4**
**C9**	37.2783	36.9954	37.1608	36.7912	37.1386	36.7999	36.9134	36.8081	36.8238	36.7803	37.4426	37.2783	36.9954	37.1608	36.7911	37.1386	36.7999	36.9134	36.8081	37.2550	36.7803	37.4426	**31.1**
**C10**	29.1554	29.7052	30.2009	29.9688	30.7342	29.9463	30.3781	29.9237	30.3314	29.9886	30.6796	29.1554	29.7052	30.2009	29.9690	30.7343	29.9462	30.3780	29.9236	30.5104	29.9887	30.6796	**26.9**
**C11**	29.2176	29.3890	29.6800	29.3092	29.7544	29.3137	29.7204	29.3173	29.7037	29.3025	29.8834	29.2176	29.3890	29.6801	29.3092	29.7544	29.3137	29.7204	29.3173	29.7502	29.3025	29.8834	**23.4**
**C12**	16.6743	16.8847	16.9795	16.9675	17.0657	16.9553	17.1035	16.9436	17.0812	16.9780	17.0345	16.6743	16.8847	16.9795	16.9675	17.0657	16.9553	17.1034	16.9436	17.0289	16.9780	17.0345	**13.8**
**C1`**	52.3408	53.1867	53.5262	53.7049	54.2316	53.6668	54.3062	53.6291	54.2417	53.7402	54.7652	56.8561	58.0291	58.4014	58.4592	58.9820	58.4317	59.1878	58.4037	59.0555	58.4833	59.3691	**53.3**
**C2`**	26.1882	26.7217	27.0852	26.8508	27.3658	26.8407	27.3737	26.8301	27.3214	26.8583	27.1841	25.8103	26.0266	26.4225	26.1449	26.6169	26.1303	26.7163	26.1161	26.5729	26.1578	26.3817	**24.2**
**C3`**	25.8103	26.0266	26.4225	26.1449	26.6168	26.1303	26.7163	26.1161	26.6985	26.1577	26.3817	26.1882	26.7217	27.0852	26.8508	27.3658	26.8408	27.3737	26.8301	27.3873	26.8582	27.1841	**24.1**
**C4`**	56.8561	58.0291	58.4014	58.4593	58.9820	58.4317	59.1878	58.4037	59.0876	58.4834	59.3691	52.3408	53.1867	53.5262	53.7050	54.2316	53.6670	54.3062	53.6290	54.1753	53.7402	54.7652	**56.3**
**H2**	8.6809	8.5543	8.6087	8.4781	8.4949	8.4829	8.4082	8.4875	8.3955	8.4732	8.5014	8.6809	8.5543	8.6087	8.4781	8.4949	8.4829	8.4082	8.4875	8.4977	8.4732	8.5014	**8.2**
**H3**	7.3091	7.4552	7.5406	7.4963	7.6103	7.4942	7.6075	7.4919	7.5968	7.4981	7.6474	7.3091	7.4552	7.5406	7.4963	7.6103	7.4942	7.6075	7.4919	7.6192	7.4981	7.6474	**7.36**
**H5**	7.1276	7.2806	7.3732	7.3462	7.4808	7.3421	7.5147	7.3379	7.5013	7.3498	7.5128	7.1276	7.2806	7.3732	7.3462	7.4808	7.3421	7.5147	7.3379	7.4705	7.3498	7.5128	**7.36**
**H6**	7.9680	8.1482	8.2416	8.2410	8.3613	8.2351	8.3599	8.2291	8.3335	8.2462	8.4392	7.9680	8.1482	8.2416	8.2410	8.3613	8.2351	8.3599	8.2291	8.3823	8.2462	8.4392	**8.2**
**H8**	4.6493	4.7076	4.7462	4.7448	4.7972	4.7412	4.8339	4.7377	4.8186	4.7480	4.8140	4.6493	4.7076	4.7462	4.7448	4.7972	4.7412	4.8339	4.7377	4.8162	4.7480	4.8140	**5.4**
**H(9A-9B)**	1.7031	1.7451	1.7428	1.7658	1.8039	1.7645	1.6855	1.7631	1.6726	1.7666	1.8372	1.7031	1.7451	1.7428	1.7658	1.8039	1.7645	1.6855	1.7631	1.7829	1.7666	1.8372	**2.08**
**H(10A)**	1.0022	0.8621	0.8587	0.8274	0.7397	0.8276	0.8447	0.8281	0.8427	0.8272	0.7965	1.0022	0.8621	0.8587	0.8274	0.7397	0.8276	0.8447	0.8281	0.7822	0.8272	0.7965	**1.14**
**H(10B)**	1.6642	1.5565	1.4947	1.4743	1.3918	1.4794	1.3895	1.4845	1.3962	1.4693	1.3592	1.6642	1.5565	1.4947	1.4743	1.3918	1.4794	1.3895	1.4845	1.3901	1.4693	1.3592	**1.28**
**H(11A-11B)**	1.0615	1.0779	1.0484	1.0922	1.0551	1.0910	1.0592	1.0898	1.0563	1.0931	1.0432	1.0615	1.0779	1.0484	1.0922	1.0551	1.0910	1.0592	1.0898	1.0490	1.0931	1.0432	**1.28**
**H(12A-12C)**	0.9351	0.9251	0.9017	0.9172	0.8775	0.9172	0.9015	0.9173	0.9005	0.9171	0.8785	0.9351	0.9251	0.9017	0.9172	0.8775	0.9172	0.9015	0.9173	0.8821	0.9171	0.8785	**0.82**
**H(1`A)**	2.7510	2.7458	2.7299	2.7316	2.6917	2.7321	2.7055	2.7326	2.7038	2.7310	2.6732	2.8761	2.8891	2.8657	2.8618	2.8352	2.8637	2.8372	2.8655	2.8463	2.8598	2.8089	**3.38**
**H(1`B)**	3.2052	3.2576	3.2845	3.2906	3.3582	3.2880	3.3290	3.2854	3.3214	3.2928	3.3427	2.8957	2.7389	2.7000	2.7100	2.7063	2.7096	2.6874	2.7096	2.6733	2.7104	2.6644	**3.71**
**H(2`A)**	1.6352	1.6825	1.6512	1.6984	1.6442	1.6972	1.6749	1.6960	1.6698	1.6994	1.6809	1.5136	1.5612	1.5313	1.5819	1.5258	1.5803	1.5795	1.5786	1.5539	1.5833	1.5775	**2.08**
**H(2`B)**	1.8433	1.8614	1.8185	1.9039	1.8569	1.8998	1.8670	1.8959	1.8604	1.9077	1.8864	1.6988	1.8177	1.7693	1.8877	1.8415	1.8827	1.8996	1.8777	1.8567	1.8924	1.9131	**2.24**
**H(3`A)**	1.5136	1.5612	1.5313	1.5819	1.5258	1.5803	1.5795	1.5786	1.5746	1.5833	1.5775	1.6352	1.6825	1.6512	1.6984	1.6442	1.6972	1.6749	1.6960	1.6638	1.6994	1.6809	**2.08**
**H(3`B)**	1.6988	1.8177	1.7693	1.8877	1.8415	1.8827	1.8996	1.8777	1.8902	1.8924	1.9131	1.8433	1.8614	1.8185	1.9039	1.8569	1.8998	1.8670	1.8959	1.8491	1.9077	1.8864	**2.16**
**H(4`A)**	2.8957	2.7389	2.7000	2.7100	2.7062	2.7097	2.6874	2.7096	2.6927	2.7104	2.6644	3.2052	3.2576	3.2845	3.2906	3.3582	3.2880	3.3290	3.2854	3.3378	3.2928	3.3427	**3.09**
**H(4`B)**	2.8761	2.8891	2.8657	2.8618	2.8352	2.8637	2.8372	2.8655	2.8376	2.8598	2.8089	2.7510	2.7458	2.7299	2.7316	2.6917	2.7321	2.7055	2.7326	2.7068	2.7309	2.6732	**3.68**
Nuclei	B3P86																						
**C1**	136.9940	137.7944	138.6085	138.2350	139.7431	138.2382	139.0549	138.2181	139.2253	138.2747	139.3602	136.9940	137.7944	138.6085	138.2350	139.7431	138.2382	139.0549	138.2181	139.2253	138.2747	139.3602	**132.2**
**C2**	138.4918	138.4799	139.0262	138.3378	138.8680	138.3690	139.3660	138.3780	139.3322	138.3524	139.3371	138.4918	138.4799	139.0263	138.3378	138.8680	138.3690	139.3660	138.3780	139.3322	138.3524	139.3370	**133.3**
**C3**	121.1824	121.9496	122.5869	122.1830	122.8942	122.1927	123.1872	122.1799	123.2106	122.2177	123.2677	121.1824	121.9496	122.5869	122.1830	122.8942	122.1927	123.1872	122.1799	123.2106	122.2176	123.2677	**117.6**
**C4**	173.8427	174.7165	175.3771	175.1356	175.7951	175.1315	176.4511	175.1055	176.3855	175.1833	176.5856	173.8427	174.7165	175.3770	175.1356	175.7951	175.1316	176.4509	175.1055	176.3855	175.1833	176.5856	**168.3**
**C5**	119.1719	120.4985	121.1966	121.1547	122.0481	121.1341	122.2667	121.0918	122.2598	121.2183	122.4439	119.1720	120.4985	121.1966	121.1548	122.0481	121.1341	122.2666	121.0918	122.2598	121.2183	122.4440	**117.6**
**C6**	134.7482	136.6843	137.8759	137.8221	139.4030	137.7676	140.1213	137.6922	139.9543	137.9183	140.4961	134.7482	136.6843	137.8759	137.8222	139.4030	137.7676	140.1212	137.6922	139.9543	137.9184	140.4961	**133.3**
**C7**	201.9446	206.3100	207.5577	208.7236	209.4429	208.6038	214.1587	208.4609	213.2041	208.8803	215.2563	201.9446	206.3100	207.5577	208.7236	209.4429	208.6038	214.1587	208.4609	213.2041	208.8803	215.2563	**195.8**
**C8**	64.8267	65.9499	66.7652	66.4439	67.5035	66.4394	68.0435	66.4121	68.0288	66.4911	68.4972	64.8267	65.9499	66.7652	66.4439	67.5035	66.4394	68.0435	66.4121	68.0288	66.4911	68.4972	**70.4**
**C9**	36.2486	36.0166	36.3080	35.8772	36.2423	35.9043	36.4484	35.9094	36.4633	35.8954	36.5768	36.2486	36.0166	36.3080	35.8771	36.2423	35.9042	36.4484	35.9094	36.4633	35.8953	36.5768	**31.1**
**C10**	27.9025	28.4047	28.9185	28.7349	29.4674	28.7300	29.2804	28.7039	29.3529	28.7840	29.4513	27.9025	28.4046	28.9185	28.7349	29.4674	28.7300	29.2804	28.7039	29.3529	28.7840	29.4513	**26.9**
**C11**	27.6635	27.8017	28.0996	27.7450	28.1783	27.7719	28.1762	27.7764	28.2670	27.7630	28.3209	27.6635	27.8016	28.0997	27.7450	28.1783	27.7718	28.1763	27.7764	28.2670	27.7629	28.3209	**23.4**
**C12**	16.2632	16.4367	16.5321	16.5461	16.6415	16.5582	16.6066	16.5487	16.6923	16.5790	16.6054	16.2632	16.4367	16.5320	16.5462	16.6415	16.5582	16.6066	16.5487	16.6923	16.5790	16.6054	**13.8**
**C1`**	51.2290	52.0562	52.3501	52.5739	53.1095	52.5603	53.1155	52.5254	53.1243	52.6314	53.6115	55.5727	56.7173	57.1113	57.1649	57.6791	57.1609	57.8424	57.1348	57.8756	57.2124	58.1048	**53.3**
**C2`**	24.9726	25.3900	25.7808	25.5444	26.0182	25.5568	26.0360	25.5472	26.1322	25.5763	25.8635	24.4400	24.6560	24.9992	24.8082	25.2508	24.8198	25.2291	24.8094	25.3062	24.8411	25.0933	**24.2**
**C3`**	24.4400	24.6560	24.9990	24.8081	25.2508	24.8198	25.2291	24.8094	25.3062	24.8410	25.0933	24.9726	25.3900	25.7807	25.5444	26.0182	25.5568	26.0361	25.5472	26.1322	25.5763	25.8636	**24.1**
**C4`**	55.5727	56.7174	57.1114	57.1649	57.6791	57.1610	57.8424	57.1348	57.8756	57.2124	58.1048	51.2290	52.0562	52.3501	52.5740	53.1095	52.5603	53.1154	52.5254	53.1243	52.6314	53.6115	**56.3**
**H2**	8.7290	8.6450	8.6624	8.5763	8.5750	8.5811	8.6077	8.5843	8.6051	8.5730	8.5861	8.7290	8.6450	8.6624	8.5763	8.5750	8.5811	8.6077	8.5842	8.6051	8.5730	8.5861	**8.2**
**H3**	7.3236	7.4766	7.5645	7.5245	7.6430	7.5235	7.6668	7.5205	7.6614	7.5267	7.6840	7.3236	7.4766	7.5645	7.5245	7.6430	7.5234	7.6668	7.5205	7.6614	7.5267	7.6839	**7.36**
**H5**	7.1584	7.3295	7.4289	7.3944	7.5356	7.3914	7.5373	7.3866	7.5325	7.3986	7.5649	7.1584	7.3295	7.4289	7.3944	7.5356	7.3914	7.5373	7.3866	7.5325	7.3985	7.5649	**7.36**
**H6**	8.0391	8.2172	8.3003	8.3183	8.4387	8.3130	8.5009	8.3060	8.4828	8.3245	8.5279	8.0391	8.2172	8.3003	8.3183	8.4387	8.3130	8.5009	8.3060	8.4828	8.3245	8.5279	**8.2**
**H8**	4.6504	4.7081	4.7313	4.7587	4.8048	4.7563	4.8490	4.7522	4.8350	4.7621	4.8465	4.6504	4.7081	4.7313	4.7587	4.8048	4.7563	4.8490	4.7522	4.8350	4.7621	4.8465	**5.4**
**H(9A-9B)**	1.7737	1.8107	1.8173	1.8361	1.8597	1.8350	1.8591	1.8321	1.8559	1.8386	1.8916	1.7737	1.8107	1.8173	1.8361	1.8597	1.8350	1.8591	1.8321	1.8559	1.8386	1.8916	**2.08**
**H(10A)**	0.9752	0.8444	0.8027	0.7996	0.7098	0.8026	0.7576	0.8038	0.7475	0.7979	0.7640	0.9752	0.8444	0.8027	0.7996	0.7098	0.8026	0.7576	0.8038	0.7475	0.7979	0.7640	**1.14**
**H(10B)**	1.6857	1.5846	1.5093	1.4961	1.4096	1.5034	1.4026	1.5090	1.4115	1.4903	1.3722	1.6857	1.5846	1.5093	1.4961	1.4096	1.5034	1.4026	1.5090	1.4115	1.4903	1.3722	**1.28**
**H(11A-11B)**	1.0296	1.0511	1.0141	1.0605	1.0240	1.0614	1.0238	1.0603	1.0254	1.0611	1.0130	1.0296	1.0511	1.0141	1.0605	1.0240	1.0614	1.0238	1.0603	1.0254	1.0611	1.0130	**1.28**
**H(12A-12C)**	0.8986	0.8890	0.8627	0.8785	0.8412	0.8804	0.8463	0.8804	0.8493	0.8780	0.8425	0.8986	0.8890	0.8627	0.8785	0.8412	0.8804	0.8463	0.8804	0.8493	0.8780	0.8425	**0.82**
**H(1`A)**	2.7041	2.6985	2.6865	2.6863	2.6464	2.6882	2.6613	2.6883	2.6657	2.6858	2.6366	2.8371	2.8452	2.8347	2.8253	2.7986	2.8283	2.8066	2.8295	2.8150	2.8235	2.7746	**3.38**
**H(1`B)**	3.2036	3.2482	3.2821	3.2812	3.3423	3.2798	3.3295	3.2766	3.3304	3.2838	3.3300	2.9405	2.7570	2.7404	2.7125	2.7146	2.7151	2.6633	2.7161	2.6706	2.7113	2.6518	**3.71**
**H(2`A)**	1.6259	1.6711	1.6444	1.6878	1.6399	1.6880	1.6624	1.6865	1.6634	1.6887	1.6744	1.4935	1.5391	1.5121	1.5598	1.5104	1.5599	1.5432	1.5581	1.5414	1.5609	1.5631	**2.08**
**H(2`B)**	1.8219	1.8427	1.7996	1.8852	1.8441	1.8829	1.8426	1.8789	1.8389	1.8888	1.8732	1.6960	1.8018	1.7559	1.8659	1.8263	1.8630	1.8475	1.8584	1.8422	1.8699	1.9035	**2.24**
**H(3`A)**	1.4935	1.5391	1.5121	1.5598	1.5104	1.5599	1.5432	1.5581	1.5414	1.5609	1.5631	1.6259	1.6711	1.6444	1.6878	1.6399	1.6880	1.6624	1.6865	1.6634	1.6887	1.6744	**2.08**
**H(3`B)**	1.6960	1.8018	1.7559	1.8659	1.8263	1.8630	1.8475	1.8584	1.8422	1.8699	1.9035	1.8219	1.8428	1.7996	1.8852	1.8441	1.8829	1.8426	1.8789	1.8389	1.8888	1.8732	**2.16**
**H(4`A)**	2.9405	2.7570	2.7404	2.7125	2.7146	2.7151	2.6633	2.7161	2.6706	2.7113	2.6518	3.2036	3.2482	3.2821	3.2812	3.3423	3.2797	3.3295	3.2766	3.3304	3.2838	3.3300	**3.09**
**H(4`B)**	2.8371	2.8452	2.8347	2.8253	2.7986	2.8283	2.8065	2.8295	2.8150	2.8235	2.7746	2.7041	2.6985	2.6865	2.6864	2.6464	2.6882	2.6612	2.6883	2.6657	2.6858	2.6366	**3.68**

[a] methyl protons shifts are averaged

[b] according to [5]

Table S5. Cont. I

Nuclei	R											S											Exp. [b]
	**B3PW91**																						
	Gas	$CHCl_3$ (PCM)	$CHCl_3$ (SMD)	DMSO (PCM)	DMSO (SMD)	CH_3OH (PCM)	CH_3OH (SMD)	C_2H_5OH (PCM)	C_2H_5OH (SMD)	H_2O (PCM)	H_2O (SMD)	Gas	$CHCl_3$ (PCM)	$CHCl_3$ (SMD)	DMSO (PCM)	DMSO (SMD)	CH_3OH (PCM)	CH_3OH (SMD)	C_2H_5OH (PCM)	C_2H_5OH (SMD)	H_2O (PCM)	H_2O (SMD)	
**C1**	137.2683	138.3110	139.0251	138.7632	140.5159	138.7452	139.6322	138.7174	139.6500	138.7819	139.7985	137.2684	138.3110	139.0253	138.7632	140.1969	138.7451	139.6321	138.7176	139.6500	138.7819	139.7985	**132.2**
**C2**	138.6410	138.6605	139.1565	138.5404	139.4008	138.5539	139.5783	138.5587	139.4533	138.5296	139.5357	138.6410	138.6605	139.1563	138.5404	139.0818	138.5541	139.5784	138.5588	139.4534	138.5296	139.5357	**133.3**
**C3**	121.2594	122.0369	122.5996	122.2518	123.2768	122.2465	123.2362	122.2321	123.1632	122.2587	123.3253	121.2594	122.0369	122.5996	122.2518	122.9578	122.2466	123.2362	122.2321	123.1632	122.2587	123.3253	**117.6**
**C4**	173.9083	174.7882	175.3849	175.1648	176.1478	175.1468	176.4418	175.1200	176.2919	175.1841	176.5909	173.9084	174.7882	175.3847	175.1648	175.8288	175.1467	176.4417	175.1199	176.2919	175.1841	176.5909	**168.3**
**C5**	119.2542	120.5663	121.1995	121.2079	122.4095	121.1729	122.3091	121.1291	122.2069	121.2432	122.4865	119.2542	120.5663	121.1995	121.2079	122.0905	121.1728	122.3091	121.1291	122.2069	121.2432	122.4865	**117.6**
**C6**	134.7732	136.7237	137.8161	137.8284	139.7082	137.7607	140.0363	137.6852	139.7917	137.8952	140.4527	134.7731	136.7237	137.8164	137.8284	139.3892	137.7607	140.0362	137.6852	139.7918	137.8952	140.4527	**133.3**
**C7**	201.9625	206.6067	207.7419	209.0508	209.9966	208.9099	214.5826	208.7599	213.4600	209.1857	215.5648	201.9625	206.6067	207.7420	209.0508	209.6776	208.9099	214.5827	208.7599	213.4600	209.1857	215.5648	**195.8**
**C8**	64.7955	66.0465	66.7422	66.5611	67.8989	66.5428	68.2177	66.5141	68.0546	66.5791	68.5848	64.7955	66.0465	66.7421	66.5611	67.5799	66.5427	68.2177	66.5142	68.0547	66.5791	68.5848	**70.4**
**C9**	35.8212	35.6167	35.7953	35.4785	36.1538	35.4920	36.0457	35.4963	35.9670	35.4673	36.1611	35.8211	35.6167	35.7953	35.4785	35.8348	35.4919	36.0457	35.4963	35.9670	35.4673	36.1611	**31.1**
**C10**	27.6048	28.1256	28.5599	28.4339	29.4841	28.4158	28.9652	28.3894	28.9490	28.4536	29.1548	27.6048	28.1256	28.5601	28.4339	29.1651	28.4157	28.9652	28.3894	28.9490	28.4536	29.1548	**26.9**
**C11**	27.6205	27.7953	28.0026	27.7348	28.4759	27.7465	28.1492	27.7489	28.1449	27.7251	28.2980	27.6204	27.7953	28.0026	27.7347	28.1569	27.7465	28.1494	27.7489	28.1449	27.7251	28.2980	**23.4**
**C12**	16.2867	16.4838	16.5092	16.5803	16.9882	16.5767	16.6234	16.5648	16.6184	16.5862	16.6291	16.2866	16.4838	16.5092	16.5803	16.6692	16.5768	16.6233	16.5649	16.6184	16.5862	16.6291	**13.8**
**C1`**	51.1535	52.0040	52.2161	52.5029	53.3437	52.4743	53.0216	52.4374	52.9376	52.5328	53.5322	55.5036	56.6599	56.9753	57.0912	57.5895	57.0723	57.7369	57.0442	57.6754	57.1109	57.9949	**53.3**
**C2`**	25.2271	25.6707	25.9838	25.8063	26.5923	25.8042	26.2842	25.7931	26.2820	25.8101	26.0751	24.7121	24.9298	25.2094	25.0583	25.5027	25.0537	25.4471	25.0406	25.4422	25.0649	25.2887	**24.2**
**C3`**	24.7122	24.9298	25.2095	25.0584	25.8217	25.0538	25.4471	25.0407	25.4422	25.0649	25.2887	25.2272	25.6707	25.9838	25.8063	26.2733	25.8042	26.2842	25.7932	26.2820	25.8101	26.0751	**24.1**
**C4`**	55.5037	56.6599	56.9752	57.0912	57.9085	57.0722	57.7369	57.0442	57.6754	57.1109	57.9949	51.1535	52.0040	52.2161	52.5029	53.0247	52.4742	53.0217	52.4375	52.9376	52.5328	53.5322	**56.3**
**H2**	8.7062	8.5966	8.6228	8.5283	8.5523	8.5326	8.5570	8.5368	8.5576	8.5243	8.5484	8.7062	8.5966	8.6228	8.5283	8.5368	8.5326	8.5570	8.5368	8.5576	8.5243	8.5484	**8.2**
**H3**	7.3295	7.4785	7.5672	7.5237	7.6568	7.5220	7.6646	7.5199	7.6557	7.5254	7.6799	7.3295	7.4785	7.5672	7.5237	7.6413	7.5220	7.6646	7.5199	7.6557	7.5254	7.6799	**7.36**
**H5**	7.1489	7.3105	7.4083	7.3811	7.5343	7.3773	7.5250	7.3732	7.5160	7.3848	7.5528	7.1489	7.3105	7.4082	7.3811	7.5188	7.3773	7.5250	7.3732	7.5160	7.3848	7.5528	**7.36**
**H6**	8.0298	8.2071	8.2903	8.3046	8.4406	8.2988	8.4763	8.2927	8.4580	8.3103	8.5083	8.0298	8.2071	8.2903	8.3046	8.4251	8.2988	8.4763	8.2927	8.4580	8.3103	8.5083	**8.2**
**H8**	4.6458	4.7027	4.7242	4.7494	4.8097	4.7462	4.8338	4.7427	4.8182	4.7527	4.8284	4.6458	4.7027	4.7242	4.7494	4.7942	4.7462	4.8338	4.7427	4.8182	4.7527	4.8284	**5.4**
**H(9A-9B)**	1.7271	1.7604	1.7647	1.7862	1.8281	1.7847	1.8116	1.7831	1.8058	1.7877	1.8467	1.7270	1.7604	1.7647	1.7862	1.8126	1.7847	1.8115	1.7831	1.8058	1.7877	1.8467	**2.08**
**H(10A)**	0.9509	0.8160	0.7754	0.7757	0.7042	0.7773	0.7328	0.7788	0.7236	0.7745	0.7414	0.9509	0.8160	0.7754	0.7757	0.6887	0.7773	0.7328	0.7788	0.7236	0.7745	0.7414	**1.14**
**H(10B)**	1.6448	1.5409	1.4720	1.4568	1.3876	1.4629	1.3662	1.4686	1.3723	1.4513	1.3371	1.6448	1.5409	1.4720	1.4568	1.3721	1.4629	1.3661	1.4686	1.3723	1.4513	1.3371	**1.28**
**H(11A-11B)**	1.0151	1.0327	0.9969	1.0448	1.0240	1.0446	1.0073	1.0441	1.0064	1.0451	0.9964	1.0151	1.0327	0.9969	1.0448	1.0085	1.0446	1.0074	1.0441	1.0064	1.0451	0.9964	**1.28**
**H(12A-12C)**	0.8895	0.8782	0.8516	0.8692	0.8460	0.8701	0.8372	0.8709	0.8369	0.8685	0.8312	0.8895	0.8782	0.8516	0.8692	0.8306	0.8701	0.8372	0.8709	0.8369	0.8685	0.8312	**0.82**
**H(1`A)**	2.6965	2.6898	2.6781	2.6782	2.6537	2.6793	2.6560	2.6801	2.6567	2.6772	2.6259	2.8310	2.8358	2.8235	2.8145	2.7855	2.8167	2.7978	2.8187	2.8011	2.8123	2.7614	**3.38**
**H(1`B)**	3.1800	3.2261	3.2593	3.2607	3.3370	3.2586	3.3106	3.2563	3.3077	3.2628	3.3078	2.8932	2.7251	2.7090	2.6878	2.6888	2.6892	2.6489	2.6905	2.6501	2.6868	2.6322	**3.71**
**H(2`A)**	1.6162	1.6597	1.6325	1.6764	1.6422	1.6759	1.6524	1.6751	1.6488	1.6770	1.6619	1.4873	1.5328	1.5055	1.5538	1.5024	1.5530	1.5382	1.5519	1.5319	1.5547	1.5550	**2.08**
**H(2`B)**	1.8041	1.8261	1.7829	1.8695	1.8412	1.8663	1.8265	1.8629	1.8198	1.8728	1.8551	1.6820	1.7905	1.7437	1.8569	1.8120	1.8529	1.8384	1.8488	1.8273	1.8608	1.8876	**2.24**
**H(3`A)**	1.4873	1.5328	1.5055	1.5538	1.5179	1.5530	1.5382	1.5520	1.5319	1.5547	1.5550	1.6162	1.6597	1.6325	1.6764	1.6267	1.6759	1.6524	1.6751	1.6488	1.6770	1.6619	**2.08**
**H(3`B)**	1.6820	1.7905	1.7437	1.8569	1.8275	1.8530	1.8384	1.8488	1.8273	1.8608	1.8876	1.8041	1.8261	1.7828	1.8695	1.8257	1.8663	1.8265	1.8629	1.8198	1.8728	1.8551	**2.16**
**H(4`A)**	2.8932	2.7251	2.7090	2.6878	2.7043	2.6892	2.6488	2.6905	2.6501	2.6868	2.6322	3.1800	3.2261	3.2593	3.2607	3.3215	3.2586	3.3107	3.2563	3.3077	3.2628	3.3078	**3.09**
**H(4`B)**	2.8310	2.8358	2.8235	2.8145	2.8010	2.8168	2.7978	2.8186	2.8010	2.8123	2.7614	2.6964	2.6898	2.6781	2.6782	2.6382	2.6793	2.6560	2.6801	2.6567	2.6772	2.6259	**3.68**
Nuclei	**B98**																						
**C1**	138.2717	139.2268	139.9128	139.6404	141.1346	139.6354	140.5187	139.6273	140.5521	139.6471	140.7148	138.2716	139.2268	139.9128	139.6403	141.1346	139.6354	140.5187	139.6273	140.5521	139.6471	140.7148	**132.2**
**C2**	138.3658	138.4130	138.9400	138.2863	138.8240	138.3104	139.3230	138.3318	139.2146	138.2658	139.2706	138.3659	138.4130	138.9400	138.2864	138.8240	138.3104	139.3230	138.3318	139.2146	138.2658	139.2706	**133.3**
**C3**	120.9842	121.7099	122.2670	121.9312	122.6018	121.9357	122.8713	121.9373	122.8035	121.9289	122.9366	120.9842	121.7099	122.2670	121.9312	122.6018	121.9357	122.8714	121.9373	122.8035	121.9289	122.9366	**117.6**
**C4**	173.7110	174.5345	175.1530	174.9251	175.5881	174.9164	176.1881	174.9052	176.0450	174.9356	176.3122	173.7110	174.5345	175.1529	174.9250	175.5881	174.9164	176.1881	174.9052	176.0450	174.9356	176.3121	**168.3**
**C5**	118.9649	120.2115	120.8309	120.8532	121.6929	120.8287	121.9059	120.8015	121.8085	120.8789	122.0628	118.9649	120.2115	120.8308	120.8531	121.6929	120.8287	121.9058	120.8015	121.8086	120.8789	122.0628	**117.6**
**C6**	134.7148	136.5886	137.6963	137.6945	139.2264	137.6373	139.8807	137.5782	139.6376	137.7516	140.2548	134.7148	136.5886	137.6962	137.6945	139.2264	137.6373	139.8805	137.5782	139.6376	137.7516	140.2547	**133.3**
**C7**	200.3965	205.1270	206.2286	207.6183	208.2481	207.4848	213.3382	207.3483	212.1534	207.7463	214.3728	200.3966	205.1270	206.2283	207.6181	208.2481	207.4848	213.3381	207.3483	212.1534	207.7463	214.3729	**195.8**
**C8**	66.4517	67.6676	68.3328	68.1491	69.2093	68.1435	69.8344	68.1334	69.6562	68.1551	70.2146	66.4518	67.6676	68.3327	68.1492	69.2093	68.1435	69.8344	68.1334	69.6562	68.1551	70.2145	**70.4**
**C9**	37.0176	36.7658	36.9738	36.6486	37.0069	36.6711	37.2536	36.6906	37.1768	36.6290	37.3166	37.0176	36.7658	36.9738	36.6487	37.0069	36.6711	37.2535	36.6906	37.1768	36.6290	37.3166	**31.1**
**C10**	29.0963	29.5633	30.0409	29.8649	30.6136	29.8578	30.4248	29.8488	30.4145	29.8742	30.5904	29.0963	29.5633	30.0409	29.8648	30.6136	29.8578	30.4248	29.8488	30.4145	29.8742	30.5904	**26.9**
**C11**	28.9406	29.0642	29.3038	29.0121	29.4482	29.0334	29.4500	29.0515	29.4499	28.9937	29.5766	28.9406	29.0642	29.3037	29.0122	29.4482	29.0334	29.4499	29.0515	29.4499	28.9937	29.5766	**23.4**
**C12**	16.8660	17.0178	17.0824	17.1275	17.2327	17.1333	17.1960	17.1370	17.1978	17.1247	17.1872	16.8660	17.0178	17.0824	17.1275	17.2327	17.1333	17.1960	17.1370	17.1978	17.1247	17.1871	**13.8**
**C1`**	52.4335	53.2373	53.4722	53.7447	54.2542	53.7251	54.2559	53.7037	54.1881	53.7667	54.7388	56.8881	57.9505	58.2822	58.3585	58.8726	58.3505	59.0149	58.3396	58.9555	58.3681	59.2730	**53.3**
**C2`**	26.4056	26.8263	27.1611	26.9646	27.4451	26.9719	27.4788	26.9766	27.4727	26.9593	27.2819	25.8949	26.0922	26.4234	26.2448	26.6989	26.2484	26.6621	26.2500	26.6634	26.2440	26.4925	**24.2**
**C3`**	25.8950	26.0922	26.4233	26.2448	26.6989	26.2484	26.6622	26.2500	26.6634	26.2440	26.4925	26.4056	26.8263	27.1611	26.9645	27.4451	26.9719	27.4788	26.9766	27.4727	26.9593	27.2819	**24.1**
**C4`**	56.8881	57.9505	58.2822	58.3585	58.8726	58.3505	59.0149	58.3396	58.9555	58.3681	59.2731	52.4335	53.2373	53.4723	53.7448	54.2542	53.7251	54.2559	53.7037	54.1881	53.7667	54.7388	**56.3**
**H2**	8.6982	8.5886	8.6267	8.5270	8.5364	8.5306	8.5595	8.5343	8.5625	8.5239	8.5476	8.6982	8.5886	8.6267	8.5270	8.5364	8.5306	8.5595	8.5343	8.5625	8.5239	8.5476	**8.2**
**H3**	7.3243	7.4676	7.5579	7.5131	7.6334	7.5110	7.6550	7.5089	7.6467	7.5153	7.6698	7.3243	7.4676	7.5579	7.5131	7.6334	7.5110	7.6550	7.5089	7.6467	7.5153	7.6698	**7.36**
**H5**	7.1440	7.3083	7.4061	7.3810	7.5164	7.3767	7.5244	7.3726	7.5150	7.3852	7.5500	7.1439	7.3083	7.4061	7.3810	7.5164	7.3767	7.5244	7.3726	7.5150	7.3852	7.5500	**7.36**
**H6**	7.9991	8.1757	8.2656	8.2788	8.3991	8.2722	8.4528	8.2658	8.4346	8.2852	8.4806	7.9991	8.1757	8.2656	8.2788	8.3991	8.2722	8.4528	8.2658	8.4346	8.2852	8.4806	**8.2**
**H8**	4.6061	4.6605	4.6938	4.7112	4.7597	4.7072	4.8012	4.7034	4.7869	4.7153	4.7909	4.6061	4.6605	4.6938	4.7112	4.7597	4.7072	4.8012	4.7034	4.7869	4.7153	4.7909	**5.4**
**H(9A-9B)**	1.7078	1.7440	1.7500	1.7713	1.8003	1.7695	1.8017	1.7677	1.7957	1.7733	1.8332	1.7078	1.7440	1.7500	1.7713	1.8003	1.7695	1.8016	1.7677	1.7957	1.7733	1.8332	**2.08**
**H(10A)**	1.0100	0.8556	0.8250	0.8148	0.7317	0.8159	0.7784	0.8174	0.7740	0.8141	0.7819	1.0100	0.8556	0.8250	0.8147	0.7317	0.8159	0.7784	0.8174	0.7740	0.8141	0.7819	**1.14**
**H(10B)**	1.6566	1.5552	1.4953	1.4723	1.3892	1.4780	1.3847	1.4837	1.3917	1.4673	1.3576	1.6566	1.5552	1.4953	1.4723	1.3892	1.4780	1.3847	1.4837	1.3917	1.4673	1.3576	**1.28**
**H(11A-11B)**	1.0408	1.0554	1.0229	1.0697	1.0330	1.0689	1.0321	1.0683	1.0319	1.0707	1.0208	1.0408	1.0554	1.0229	1.0697	1.0330	1.0689	1.0321	1.0683	1.0319	1.0707	1.0208	**1.28**
**H(12A-12C)**	0.9137	0.8998	0.8755	0.8914	0.8529	0.8920	0.8600	0.8926	0.8602	0.8913	0.8544	0.9137	0.8998	0.8755	0.8914	0.8529	0.8919	0.8600	0.8926	0.8602	0.8913	0.8544	**0.82**
**H(1`A)**	2.7147	2.7096	2.6996	2.6998	2.6606	2.7004	2.6788	2.7011	2.6801	2.6994	2.6440	2.8444	2.8541	2.8420	2.8323	2.8064	2.8344	2.8190	2.8364	2.8217	2.8306	2.7831	**3.38**
**H(1`B)**	3.1966	3.2429	3.2781	3.2791	3.3414	3.2766	3.3292	3.2742	3.3264	3.2818	3.3230	2.8921	2.7320	2.7168	2.7042	2.7055	2.7047	2.6717	2.7055	2.6724	2.7042	2.6580	**3.71**
**H(2`A)**	1.6333	1.6782	1.6498	1.6962	1.6453	1.6953	1.6711	1.6945	1.6675	1.6974	1.6823	1.5089	1.5555	1.5281	1.5780	1.5262	1.5768	1.5620	1.5757	1.5564	1.5793	1.5795	**2.08**
**H(2`B)**	1.8213	1.8381	1.7969	1.8847	1.8396	1.8809	1.8420	1.8773	1.8357	1.8888	1.8673	1.7044	1.8128	1.7649	1.8774	1.8326	1.8732	1.8583	1.8691	1.8480	1.8817	1.9094	**2.24**
**H(3`A)**	1.5089	1.5555	1.5282	1.5780	1.5262	1.5768	1.5620	1.5757	1.5564	1.5793	1.5794	1.6333	1.6782	1.6498	1.6962	1.6453	1.6953	1.6711	1.6945	1.6675	1.6974	1.6823	**2.08**
**H(3`B)**	1.7044	1.8128	1.7649	1.8774	1.8326	1.8732	1.8583	1.8691	1.8480	1.8817	1.9094	1.8213	1.8381	1.7969	1.8847	1.8396	1.8809	1.8420	1.8773	1.8357	1.8888	1.8674	**2.16**
**H(4`A)**	2.8922	2.7320	2.7168	2.7042	2.7055	2.7047	2.6717	2.7055	2.6724	2.7042	2.6580	3.1966	3.2429	3.2782	3.2791	3.3414	3.2766	3.3292	3.2742	3.3264	3.2818	3.3229	**3.09**
**H(4`B)**	2.8445	2.8541	2.8420	2.8323	2.8064	2.8344	2.8190	2.8364	2.8217	2.8306	2.7831	2.7147	2.7096	2.6996	2.6998	2.6606	2.7004	2.6788	2.7011	2.6801	2.6994	2.6440	**3.68**

[a] methyl protons shifts are averaged

[b] according to [5]

Table S5. Cont. II

Nuclei	R											S											Exp. [b]
	B971																						
	Gas	$CHCl_3$ (PCM)	$CHCl_3$ (SMD)	DMSO (PCM)	DMSO (SMD)	CH_3OH (PCM)	CH_3OH (SMD)	C_2H_5OH (PCM)	C_2H_5OH (SMD)	H_2O (PCM)	H_2O (SMD)	Gas	$CHCl_3$ (PCM)	$CHCl_3$ (SMD)	DMSO (PCM)	DMSO (SMD)	CH_3OH (PCM)	CH_3OH (SMD)	C_2H_5OH (PCM)	C_2H_5OH (SMD)	H_2O (PCM)	H_2O (SMD)	
**C1**	137.8422	138.7483	139.4242	139.1819	140.7120	139.1719	140.0612	139.1604	140.1037	139.1965	140.2855	137.8422	138.7484	139.4242	139.1821	140.7120	139.1719	140.0613	139.1604	140.1037	139.1965	140.2855	**132.2**
**C2**	138.0799	138.1325	138.6399	138.0013	138.5299	138.0181	139.0158	138.0344	138.9092	137.9914	138.9727	138.0799	138.1323	138.6399	138.0013	138.5299	138.0181	139.0159	138.0344	138.9092	137.9914	138.9727	**133.3**
**C3**	120.7944	121.5215	122.0745	121.7507	122.4133	121.7473	122.6794	121.7430	122.6120	121.7596	122.7491	120.7944	121.5213	122.0745	121.7507	122.4133	121.7473	122.6794	121.7430	122.6119	121.7596	122.7491	**117.6**
**C4**	173.5971	174.4353	175.0465	174.8429	175.4966	174.8257	176.0952	174.8081	175.9514	174.8650	176.2233	173.5971	174.4353	175.0464	174.8427	175.4966	174.8257	176.0951	174.8081	175.9514	174.8650	176.2233	**168.3**
**C5**	118.7655	120.0340	120.6434	120.6887	121.5173	120.6557	121.7292	120.6223	121.6310	120.7260	121.8918	118.7655	120.0341	120.6435	120.6887	121.5173	120.6557	121.7293	120.6223	121.6311	120.7260	121.8918	**117.6**
**C6**	134.4339	136.3104	137.4295	137.4298	138.9589	137.3639	139.6148	137.2980	139.3694	137.4992	139.9897	134.4339	136.3105	137.4296	137.4299	138.9589	137.3638	139.6149	137.2980	139.3693	137.4992	139.9897	**133.3**
**C7**	200.2729	204.9044	206.0061	207.3978	208.0832	207.2601	213.0559	207.1201	211.9008	207.5342	214.1161	200.2729	204.9043	206.0060	207.3982	208.0832	207.2600	213.0562	207.1201	211.9008	207.5342	214.1161	**195.8**
**C8**	66.2684	67.4430	68.1355	67.9281	69.0012	67.9149	69.5789	67.8993	69.4095	67.9447	69.9754	66.2684	67.4428	68.1355	67.9282	69.0012	67.9149	69.5789	67.8993	69.4094	67.9447	69.9754	**70.4**
**C9**	37.0068	36.7745	37.0004	36.6683	37.0130	36.6820	37.2582	36.6949	37.1794	36.6609	37.3381	37.0068	36.7745	37.0004	36.6683	37.0130	36.6820	37.2583	36.6949	37.1793	36.6609	37.3381	**31.1**
**C10**	29.1505	29.6168	30.0966	29.9276	30.6566	29.9120	30.4719	29.8963	30.4604	29.9491	30.6448	29.1505	29.6167	30.0966	29.9277	30.6566	29.9120	30.4718	29.8963	30.4603	29.9491	30.6448	**26.9**
**C11**	28.9245	29.0484	29.2800	29.0001	29.4237	29.0135	29.4199	29.0260	29.4191	28.9929	29.5535	28.9245	29.0484	29.2800	29.0002	29.4237	29.0136	29.4199	29.0260	29.4191	28.9929	29.5535	**23.4**
**C12**	16.8110	16.9684	17.0237	17.0860	17.1770	17.0840	17.1383	17.0818	17.1401	17.0943	17.1357	16.8110	16.9683	17.0236	17.0860	17.1770	17.0840	17.1384	17.0818	17.1402	17.0943	17.1357	**13.8**
**C1`**	52.3547	53.1528	53.3732	53.6664	54.1692	53.6388	54.1635	53.6114	54.0937	53.6995	54.6432	56.7953	57.8551	58.1732	58.2702	58.7717	58.2546	58.9084	58.2381	58.8505	58.2908	59.1676	**53.3**
**C2`**	26.2113	26.6404	26.9708	26.7933	27.2590	26.7924	27.2868	26.7908	27.2809	26.7996	27.1109	25.6996	25.9076	26.2264	26.0756	26.5176	26.0712	26.4850	26.0668	26.4842	26.0859	26.3418	**24.2**
**C3`**	25.6996	25.9075	26.2264	26.0756	26.5176	26.0712	26.4850	26.0668	26.4841	26.0859	26.3418	26.2113	26.6403	26.9708	26.7933	27.2590	26.7924	27.2869	26.7908	27.2809	26.7996	27.1109	**24.1**
**C4`**	56.7953	57.8551	58.1732	58.2702	58.7717	58.2546	58.9084	58.2381	58.8505	58.2908	59.1676	52.3547	53.1529	53.3732	53.6663	54.1692	53.6387	54.1636	53.6114	54.0938	53.6995	54.6432	**56.3**
**H2**	8.6975	8.5947	8.6294	8.5328	8.5358	8.5359	8.5631	8.5391	8.5649	8.5303	8.5487	8.6975	8.5947	8.6294	8.5328	8.5358	8.5359	8.5631	8.5392	8.5649	8.5303	8.5487	**8.2**
**H3**	7.3205	7.4628	7.5522	7.5095	7.6286	7.5072	7.6504	7.5050	7.6421	7.5121	7.6648	7.3205	7.4628	7.5522	7.5095	7.6286	7.5072	7.6504	7.5050	7.6421	7.5121	7.6648	**7.36**
**H5**	7.1452	7.3121	7.4102	7.3844	7.5179	7.3799	7.5258	7.3755	7.5164	7.3890	7.5509	7.1452	7.3121	7.4102	7.3844	7.5179	7.3799	7.5258	7.3755	7.5164	7.3890	7.5509	**7.36**
**H6**	8.0058	8.1773	8.2674	8.2802	8.3987	8.2735	8.4538	8.2669	8.4356	8.2870	8.4802	8.0058	8.1773	8.2674	8.2802	8.3987	8.2735	8.4538	8.2669	8.4356	8.2870	8.4802	**8.2**
**H8**	4.6088	4.6617	4.6949	4.7134	4.7602	4.7093	4.8019	4.7054	4.7882	4.7177	4.7919	4.6088	4.6617	4.6949	4.7134	4.7602	4.7093	4.8019	4.7054	4.7882	4.7177	4.7919	**5.4**
**H(9A-9B)**	1.7161	1.7513	1.7594	1.7791	1.8050	1.7769	1.8071	1.7747	1.8006	1.7817	1.8378	1.7161	1.7513	1.7594	1.7791	1.8050	1.7769	1.8071	1.7747	1.8006	1.7817	1.8378	**2.08**
**H(10A)**	1.0061	0.8545	0.8220	0.8136	0.7285	0.8147	0.7778	0.8160	0.7734	0.8131	0.7805	1.0061	0.8545	0.8220	0.8136	0.7285	0.8147	0.7778	0.8160	0.7734	0.8131	0.7805	**1.14**
**H(10B)**	1.6552	1.5553	1.4937	1.4723	1.3892	1.4778	1.3845	1.4833	1.3914	1.4676	1.3570	1.6552	1.5553	1.4937	1.4723	1.3892	1.4778	1.3845	1.4833	1.3914	1.4676	1.3570	**1.28**
**H(11A-11B)**	1.0394	1.0539	1.0204	1.0676	1.0299	1.0668	1.0293	1.0659	1.0291	1.0689	1.0176	1.0394	1.0540	1.0204	1.0676	1.0299	1.0668	1.0293	1.0659	1.0291	1.0689	1.0176	**1.28**
**H(12A-12C)**	0.9081	0.8931	0.8684	0.8850	0.8458	0.8853	0.8530	0.8857	0.8533	0.8851	0.8480	0.9081	0.8931	0.8684	0.8850	0.8458	0.8853	0.8531	0.8857	0.8533	0.8851	0.8480	**0.82**
**H(1`A)**	2.7043	2.6996	2.6902	2.6911	2.6510	2.6915	2.6684	2.6920	2.6698	2.6911	2.6344	2.8345	2.8453	2.8343	2.8262	2.8010	2.8280	2.8117	2.8297	2.8145	2.8248	2.7764	**3.38**
**H(1`B)**	3.1997	3.2445	3.2806	3.2807	3.3411	3.2780	3.3284	3.2754	3.3259	3.2838	3.3220	2.8940	2.7339	2.7203	2.7054	2.7063	2.7059	2.6706	2.7066	2.6719	2.7055	2.6571	**3.71**
**H(2`A)**	1.6287	1.6741	1.6457	1.6934	1.6426	1.6922	1.6678	1.6912	1.6642	1.6949	1.6794	1.5028	1.5487	1.5215	1.5727	1.5216	1.5713	1.5566	1.5700	1.5510	1.5744	1.5740	**2.08**
**H(2`B)**	1.8228	1.8394	1.7978	1.8856	1.8402	1.8817	1.8424	1.8780	1.8362	1.8899	1.8679	1.7084	1.8122	1.7635	1.8768	1.8335	1.8725	1.8576	1.8684	1.8477	1.8812	1.9088	**2.24**
**H(3`A)**	1.5028	1.5487	1.5215	1.5727	1.5216	1.5713	1.5566	1.5700	1.5510	1.5744	1.5740	1.6287	1.6741	1.6458	1.6934	1.6426	1.6922	1.6678	1.6912	1.6642	1.6949	1.6794	**2.08**
**H(3`B)**	1.7084	1.8122	1.7635	1.8768	1.8335	1.8725	1.8576	1.8684	1.8477	1.8812	1.9088	1.8228	1.8394	1.7978	1.8856	1.8402	1.8817	1.8424	1.8780	1.8362	1.8899	1.8679	**2.16**
**H(4`A)**	2.8940	2.7339	2.7202	2.7054	2.7063	2.7059	2.6706	2.7066	2.6719	2.7055	2.6571	3.1997	3.2445	3.2806	3.2807	3.3411	3.2780	3.3285	3.2754	3.3259	3.2838	3.3220	**3.09**
**H(4`B)**	2.8345	2.8453	2.8343	2.8262	2.8010	2.8280	2.8117	2.8297	2.8145	2.8248	2.7764	2.7043	2.6996	2.6902	2.6911	2.6510	2.6915	2.6684	2.6920	2.6698	2.6911	2.6344	**3.68**
Nuclei	B972																						
**C1**	134.6971	135.8753	136.6169	136.6760	139.5533	136.6202	138.6512	136.5674	138.8176	136.7300	138.9783	134.6971	135.8753	136.6169	136.6759	139.5532	136.6203	138.6511	136.5674	138.8176	136.7300	138.9784	**132.2**
**C2**	136.1423	136.0686	136.6110	136.0524	136.6697	136.0535	137.1061	136.0530	137.0015	136.0477	137.0908	136.1423	136.0686	136.6111	136.0522	136.6695	136.0535	137.1058	136.0530	137.0015	136.0477	137.0908	**133.3**
**C3**	118.6863	119.3715	119.9318	119.6489	120.3301	119.6370	120.5956	119.6220	120.5384	119.6557	120.6719	118.6863	119.3715	119.9318	119.6490	120.3300	119.6370	120.5956	119.6220	120.5384	119.6557	120.6718	**117.6**
**C4**	170.5935	171.3556	171.9651	171.7320	172.1420	171.7133	172.7626	171.6911	172.6048	171.7444	172.8736	170.5934	171.3556	171.9650	171.7319	172.1420	171.7133	172.7627	171.6911	172.6048	171.7444	172.8736	**168.3**
**C5**	116.6790	117.8768	118.5101	118.5696	119.3676	118.5275	119.6225	118.4831	119.5235	118.6058	119.7745	116.6790	117.8768	118.5101	118.5698	119.3676	118.5275	119.6226	118.4831	119.5236	118.6058	119.7746	**117.6**
**C6**	132.2209	134.0034	135.0675	135.0035	135.8136	134.9461	136.4690	134.8843	136.2020	135.0514	136.7295	132.2209	134.0034	135.0674	135.0036	135.8136	134.9461	136.4689	134.8843	136.2019	135.0514	136.7297	**133.3**
**C7**	197.8670	202.6198	203.7484	205.3519	207.1219	205.1793	211.9055	205.0088	210.8523	205.5163	213.0007	197.8670	202.6198	203.7482	205.3522	207.1219	205.1793	211.9055	205.0088	210.8523	205.5163	213.0007	**195.8**
**C8**	63.5056	64.8160	65.4994	65.4760	66.7506	65.4415	67.2458	65.4046	67.0801	65.5049	67.6571	63.5056	64.8160	65.4994	65.4761	66.7505	65.4415	67.2458	65.4046	67.0801	65.5049	67.6571	**70.4**
**C9**	35.1659	34.9077	35.1070	34.8130	34.8409	34.8226	35.0047	34.8285	34.9106	34.7984	35.0884	35.1659	34.9077	35.1070	34.8131	34.8409	34.8226	35.0047	34.8285	34.9107	34.7984	35.0884	**31.1**
**C10**	27.4347	27.9105	28.3498	28.2459	28.8451	28.2248	28.6389	28.2010	28.6128	28.2615	28.8073	27.4347	27.9105	28.3499	28.2459	28.8452	28.2247	28.6389	28.2010	28.6128	28.2615	28.8074	**26.9**
**C11**	27.6676	27.7939	28.0328	27.8076	28.1669	27.8114	28.1400	27.8123	28.1369	27.7989	28.2738	27.6676	27.7939	28.0327	27.8075	28.1669	27.8114	28.1400	27.8123	28.1370	27.7989	28.2739	**23.4**
**C12**	16.4210	16.5676	16.6312	16.7389	16.8296	16.7272	16.8016	16.7136	16.8013	16.7461	16.8064	16.4210	16.5676	16.6312	16.7389	16.8296	16.7272	16.8016	16.7136	16.8013	16.7461	16.8064	**13.8**
**C1`**	50.3513	51.1355	51.3757	51.6819	52.1647	51.6473	52.1659	51.6111	52.1124	51.7109	52.6598	54.6831	55.7368	56.0738	56.2267	56.7429	56.2006	56.8535	56.1716	56.7908	56.2470	57.1038	**53.3**
**C2`**	25.1575	25.5809	25.9072	25.7805	26.1941	25.7710	26.2013	25.7588	26.1797	25.7847	25.9870	24.6675	24.8374	25.1474	25.0423	25.5381	25.0287	25.4907	25.0134	25.4932	25.0513	25.2788	**24.2**
**C3`**	24.6675	24.8374	25.1475	25.0423	25.5380	25.0287	25.4907	25.0134	25.4932	25.0513	25.2788	25.1574	25.5809	25.9072	25.7806	26.1941	25.7710	26.2013	25.7588	26.1797	25.7847	25.9870	**24.1**
**C4`**	54.6830	55.7368	56.0739	56.2268	56.7429	56.2006	56.8535	56.1716	56.7908	56.2470	57.1039	50.3513	51.1355	51.3758	51.6818	52.1647	51.6473	52.1659	51.6111	52.1125	51.7109	52.6599	**56.3**
**H2**	8.7007	8.5592	8.5908	8.4663	8.3592	8.4728	8.4141	8.4787	8.4039	8.4595	8.3953	8.7007	8.5592	8.5908	8.4663	8.3592	8.4728	8.4141	8.4787	8.4039	8.4595	8.3953	**8.2**
**H3**	7.3297	7.4738	7.5631	7.5139	7.6078	7.5126	7.6304	7.5107	7.6217	7.5149	7.6457	7.3297	7.4738	7.5631	7.5139	7.6078	7.5126	7.6304	7.5107	7.6217	7.5149	7.6457	**7.36**
**H5**	7.1325	7.2902	7.3841	7.3696	7.5156	7.3646	7.5407	7.3595	7.5307	7.3743	7.5618	7.1325	7.2902	7.3841	7.3696	7.5156	7.3646	7.5407	7.3595	7.5307	7.3743	7.5618	**7.36**
**H6**	8.0154	8.1828	8.2635	8.2621	8.3160	8.2580	8.3752	8.2534	8.3539	8.2656	8.3870	8.0154	8.1828	8.2635	8.2622	8.3160	8.2580	8.3752	8.2534	8.3539	8.2656	8.3870	**8.2**
**H8**	4.6168	4.6642	4.6860	4.7066	4.7485	4.7037	4.7874	4.7006	4.7743	4.7091	4.7672	4.6168	4.6642	4.6860	4.7066	4.7485	4.7037	4.7874	4.7006	4.7743	4.7091	4.7672	**5.4**
**H(9A-9B)**	1.6879	1.7170	1.7219	1.7279	1.6637	1.7278	1.6700	1.7273	1.6567	1.7274	1.7027	1.6879	1.7170	1.7218	1.7279	1.6637	1.7278	1.6700	1.7273	1.6567	1.7274	1.7027	**2.08**
**H(10A)**	0.9637	0.8246	0.7814	0.7958	0.7462	0.7959	0.7971	0.7962	0.7959	0.7958	0.8043	0.9637	0.8246	0.7814	0.7958	0.7462	0.7959	0.7971	0.7962	0.7959	0.7958	0.8043	**1.14**
**H(10B)**	1.6217	1.5183	1.4590	1.4425	1.3585	1.4475	1.3581	1.4523	1.3656	1.4377	1.3287	1.6217	1.5183	1.4591	1.4425	1.3585	1.4475	1.3581	1.4523	1.3656	1.4377	1.3287	**1.28**
**H(11A-11B)**	1.0221	1.0367	1.0037	1.0526	1.0131	1.0518	1.0176	1.0508	1.0171	1.0532	1.0044	1.0221	1.0367	1.0037	1.0526	1.0131	1.0518	1.0176	1.0508	1.0171	1.0532	1.0045	**1.28**
**H(12A-12C)**	0.9038	0.8919	0.8662	0.8885	0.8592	0.8886	0.8679	0.8886	0.8693	0.8883	0.8616	0.9038	0.8919	0.8662	0.8885	0.8592	0.8886	0.8679	0.8886	0.8693	0.8883	0.8616	**0.82**
**H(1`A)**	2.6968	2.6878	2.6766	2.6791	2.6372	2.6798	2.6542	2.6802	2.6545	2.6784	2.6219	2.8356	2.8425	2.8290	2.8230	2.7927	2.8246	2.8005	2.8261	2.8024	2.8212	2.7688	**3.38**
**H(1`B)**	3.1801	3.2276	3.2615	3.2635	3.3127	3.2613	3.3038	3.2588	3.2991	3.2655	3.3001	2.8657	2.7208	2.7098	2.7022	2.7288	2.7020	2.6867	2.7019	2.6919	2.7027	2.6798	**3.71**
**H(2`A)**	1.6133	1.6575	1.6300	1.6779	1.6287	1.6768	1.6531	1.6756	1.6504	1.6788	1.6625	1.4889	1.5353	1.5078	1.5622	1.5233	1.5606	1.5567	1.5589	1.5539	1.5637	1.5734	**2.08**
**H(2`B)**	1.7948	1.8147	1.7739	1.8616	1.8208	1.8579	1.8223	1.8542	1.8177	1.8652	1.8493	1.6849	1.7954	1.7490	1.8700	1.8504	1.8649	1.8713	1.8598	1.8649	1.8749	1.9225	**2.24**
**H(3`A)**	1.4889	1.5353	1.5078	1.5622	1.5233	1.5606	1.5567	1.5589	1.5539	1.5637	1.5734	1.6133	1.6575	1.6300	1.6779	1.6287	1.6768	1.6531	1.6756	1.6504	1.6788	1.6625	**2.08**
**H(3`B)**	1.6849	1.7954	1.7490	1.8700	1.8504	1.8649	1.8713	1.8598	1.8649	1.8749	1.9225	1.7948	1.8147	1.7739	1.8616	1.8208	1.8579	1.8223	1.8542	1.8177	1.8652	1.8493	**2.16**
**H(4`A)**	2.8657	2.7208	2.7099	2.7023	2.7288	2.7020	2.6867	2.7019	2.6919	2.7027	2.6798	3.1801	3.2276	3.2614	3.2635	3.3127	3.2613	3.3038	3.2588	3.2992	3.2655	3.3001	**3.09**
**H(4`B)**	2.8356	2.8425	2.8290	2.8230	2.7927	2.8246	2.8005	2.8261	2.8024	2.8212	2.7688	2.6968	2.6878	2.6766	2.6791	2.6372	2.6798	2.6542	2.6802	2.6545	2.6784	2.6220	**3.68**

[a] methyl protons shifts are averaged
[b] according to [5]

Table S5. Cont. III

Nuclei	R											S											Exp. [b]
	BHandH																						
	Gas	$CHCl_3$ (PCM)	$CHCl_3$ (SMD)	DMSO (PCM)	DMSO (SMD)	CH_3OH (PCM)	CH_3OH (SMD)	C_2H_5OH (PCM)	C_2H_5OH (SMD)	H_2O (PCM)	H_2O (SMD)	Gas	$CHCl_3$ (PCM)	$CHCl_3$ (SMD)	DMSO (PCM)	DMSO (SMD)	CH_3OH (PCM)	CH_3OH (SMD)	C_2H_5OH (PCM)	C_2H_5OH (SMD)	H_2O (PCM)	H_2O (SMD)	
**C1**	138.5465	139.0589	140.0282	139.2348	140.8674	139.2148	140.1811	139.2131	140.2774	139.2234	140.5547	138.5464	139.0589	140.0282	139.2348	139.9789	139.2149	140.1811	139.2130	140.2774	139.2234	140.5546	**132.2**
**C2**	143.5117	143.4353	144.0030	143.2870	143.8194	143.2877	144.2640	143.3068	144.2012	143.2560	144.2993	143.5117	143.4353	144.0030	143.2870	144.0311	143.2877	144.2640	143.3066	144.2012	143.2560	144.2993	**133.3**
**C3**	123.6216	124.3782	125.0268	124.6233	125.3404	124.6010	125.5677	124.5967	125.5216	124.6141	125.6558	123.6216	124.3782	125.0269	124.6233	125.3674	124.6011	125.5677	124.5967	125.5216	124.6141	125.6558	**117.6**
**C4**	174.7888	175.5536	176.1854	175.9620	176.5660	175.9263	177.2388	175.9091	177.0774	175.9658	177.3779	174.7888	175.5536	176.1854	175.9620	176.7061	175.9263	177.2388	175.9092	177.0774	175.9658	177.3779	**168.3**
**C5**	121.5046	122.9341	123.6411	123.6822	124.5984	123.6257	124.8174	123.5876	124.7076	123.7060	124.9940	121.5047	122.9341	123.6411	123.6822	124.4263	123.6257	124.8174	123.5877	124.7076	123.7060	124.9940	**117.6**
**C6**	139.7122	141.6732	142.8202	142.8468	144.3976	142.7615	145.0656	142.6949	144.7943	142.8980	145.4379	139.7122	141.6732	142.8202	142.8468	143.5909	142.7615	145.0656	142.6950	144.7943	142.8980	145.4378	**133.3**
**C7**	204.0554	208.3972	209.8032	210.7478	211.4240	210.5925	216.7982	210.4551	215.6159	210.8653	217.9800	204.0553	208.3972	209.8031	210.7478	211.4919	210.5925	216.7982	210.4554	215.6159	210.8653	217.9800	**195.8**
**C8**	60.0731	61.1362	61.9412	61.6394	62.5544	61.5974	63.2661	61.5739	63.1273	61.6492	63.7607	60.0731	61.1362	61.9412	61.6394	62.3835	61.5974	63.2661	61.5738	63.1273	61.6492	63.7607	**70.4**
**C9**	34.1020	33.8856	34.1209	33.7869	34.0297	33.7837	34.1234	33.7986	34.0914	33.7597	34.2791	34.1020	33.8856	34.1209	33.7869	34.5310	33.7838	34.1234	33.7987	34.0914	33.7597	34.2791	**31.1**
**C10**	27.2135	27.7537	28.1167	28.1371	28.8125	28.0995	28.5687	28.0810	28.5478	28.1431	28.8216	27.2135	27.7537	28.1167	28.1371	28.8812	28.0995	28.5687	28.0810	28.5478	28.1431	28.8216	**26.9**
**C11**	26.1644	26.2342	26.5746	26.1987	26.6023	26.1915	26.5850	26.2026	26.6070	26.1752	26.7570	26.1643	26.2342	26.5745	26.1987	26.9428	26.1914	26.5850	26.2025	26.6070	26.1752	26.7571	**23.4**
**C12**	15.6496	15.8310	15.8835	15.9590	16.0362	15.9394	15.9509	15.9385	15.9568	15.9476	15.9762	15.6495	15.8311	15.8834	15.9590	16.7031	15.9394	15.9509	15.9385	15.9568	15.9476	15.9762	**13.8**
**C1`**	48.2959	49.0980	49.4034	49.5638	50.0949	49.5213	50.0509	49.4977	50.0387	49.5750	50.4802	52.2406	53.2551	53.7083	53.7330	54.4771	53.6924	54.4518	53.6704	54.3989	53.7416	54.6924	**53.3**
**C2`**	21.6367	22.0035	22.3605	22.1802	22.6341	22.1578	22.7044	22.1539	22.7025	22.1716	22.6865	21.0585	21.3095	21.6337	21.4659	22.2100	21.4453	21.8796	21.4431	21.8815	21.4554	21.8474	**24.2**
**C3`**	21.0585	21.3095	21.6337	21.4659	21.9420	21.4453	21.8796	21.4431	21.8815	21.4554	21.8473	21.6367	22.0035	22.3605	22.1802	22.9243	22.1578	22.7044	22.1539	22.7025	22.1716	22.6865	**24.1**
**C4`**	52.2406	53.2551	53.7084	53.7330	54.3137	53.6925	54.4518	53.6705	54.3989	53.7416	54.6924	48.2958	49.0980	49.4034	49.5638	50.3079	49.5213	50.0509	49.4977	50.0387	49.5750	50.4801	**56.3**
**H2**	9.0133	8.9379	8.9431	8.8888	8.8838	8.8928	8.8900	8.8959	8.8935	8.8861	8.8693	9.0133	8.9379	8.9431	8.8888	8.8076	8.8928	8.8900	8.8959	8.8935	8.8861	8.8693	**8.2**
**H3**	7.4474	7.5927	7.6854	7.6383	7.7626	7.6369	7.7844	7.6346	7.7756	7.6405	7.8000	7.4474	7.5927	7.6854	7.6383	7.5571	7.6369	7.7844	7.6346	7.7756	7.6405	7.8000	**7.36**
**H5**	7.2803	7.4792	7.5728	7.5667	7.6983	7.5625	7.7142	7.5574	7.7025	7.5718	7.7363	7.2803	7.4792	7.5728	7.5667	7.4855	7.5625	7.7142	7.5574	7.7025	7.5718	7.7363	**7.36**
**H6**	8.1894	8.3806	8.4651	8.4861	8.6214	8.4803	8.6749	8.4737	8.6488	8.4926	8.7015	8.1894	8.3806	8.4651	8.4861	8.4049	8.4803	8.6749	8.4737	8.6488	8.4926	8.7015	**8.2**
**H8**	4.2693	4.3483	4.3771	4.4032	4.4839	4.4004	4.5358	4.3968	4.5082	4.4069	4.5389	4.2693	4.3483	4.3771	4.4032	4.3220	4.4004	4.5358	4.3968	4.5082	4.4069	4.5389	**5.4**
**H(9A-9B)**	1.7884	1.8248	1.8176	1.8632	1.8617	1.8613	1.8484	1.8587	1.8470	1.8660	1.8841	1.7883	1.8248	1.8176	1.8632	1.7820	1.8613	1.8484	1.8587	1.8470	1.8660	1.8841	**2.08**
**H(10A)**	0.9699	0.8271	0.7545	0.7425	0.6584	0.7484	0.6674	0.7535	0.6673	0.7380	0.6698	0.9699	0.8271	0.7545	0.7425	0.6584	0.7484	0.6674	0.7535	0.6673	0.7380	0.6698	**1.14**
**H(10B)**	1.6604	1.5673	1.5079	1.4980	1.4014	1.5040	1.3938	1.5089	1.4008	1.4933	1.3675	1.6604	1.5673	1.5080	1.4980	1.4014	1.5040	1.3938	1.5089	1.4008	1.4933	1.3675	**1.28**
**H(11A-11B)**	0.9886	1.0092	0.9788	1.0287	0.9857	1.0282	0.9820	1.0270	0.9828	1.0301	0.9726	0.9886	1.0092	0.9788	1.0287	0.9474	1.0282	0.9820	1.0270	0.9828	1.0301	0.9726	**1.28**
**H(12A-12C)**	0.9400	0.9236	0.8941	0.9103	0.8692	0.9121	0.8711	0.9129	0.8722	0.9097	0.8665	0.9400	0.9236	0.8941	0.9103	0.8291	0.9121	0.8711	0.9129	0.8722	0.9097	0.8665	**0.82**
**H(1`A)**	2.5921	2.5890	2.5638	2.5786	2.5274	2.5803	2.5416	2.5812	2.5420	2.5781	2.5258	2.7460	2.7455	2.7319	2.7273	2.6904	2.7296	2.7107	2.7311	2.7126	2.7260	2.6863	**3.38**
**H(1`B)**	3.0932	3.1308	3.1577	3.1643	3.2166	3.1627	3.1977	3.1603	3.1933	3.1669	3.2011	2.9077	2.7015	2.6593	2.6386	2.6117	2.6430	2.5508	2.6465	2.5576	2.6356	2.5450	**3.71**
**H(2`A)**	1.5987	1.6473	1.6123	1.6655	1.6051	1.6653	1.6330	1.6643	1.6301	1.6668	1.6474	1.4624	1.5143	1.4813	1.5342	1.4765	1.5339	1.5179	1.5328	1.5130	1.5355	1.5390	**2.08**
**H(2`B)**	1.7665	1.7910	1.7431	1.8278	1.7968	1.8259	1.7866	1.8233	1.7805	1.8307	1.8094	1.6896	1.7754	1.7352	1.8298	1.7997	1.8267	1.8301	1.8229	1.8205	1.8338	1.8777	**2.24**
**H(3`A)**	1.4624	1.5143	1.4813	1.5342	1.4765	1.5339	1.5179	1.5328	1.5130	1.5355	1.5390	1.5987	1.6473	1.6123	1.6655	1.6051	1.6653	1.6330	1.6643	1.6301	1.6668	1.6474	**2.08**
**H(3`B)**	1.6896	1.7754	1.7352	1.8298	1.7997	1.8267	1.8301	1.8229	1.8205	1.8338	1.8777	1.7665	1.7910	1.7431	1.8278	1.7968	1.8259	1.7866	1.8232	1.7805	1.8307	1.8095	**2.16**
**H(4`A)**	2.9077	2.7015	2.6593	2.6386	2.6117	2.6430	2.5508	2.6465	2.5576	2.6356	2.5450	3.0932	3.1308	3.1577	3.1643	3.2166	3.1627	3.1977	3.1603	3.1933	3.1669	3.2011	**3.09**
**H(4`B)**	2.7460	2.7455	2.7320	2.7273	2.6904	2.7296	2.7107	2.7311	2.7126	2.7260	2.6863	2.5921	2.5890	2.5638	2.5786	2.5274	2.5803	2.5416	2.5812	2.5420	2.5781	2.5258	**3.68**
Nuclei	BHandHLYP																						
**C1**	139.1706	139.9116	140.6432	140.3296	141.7914	140.3148	140.9419	140.3012	141.1074	140.3424	141.2702	139.1706	139.9116	140.6432	140.3295	141.7914	140.3148	140.9419	140.3012	141.1074	140.3424	141.2702	**132.2**
**C2**	142.0275	141.9201	142.3974	141.7860	142.2101	141.7956	142.7562	141.8074	142.6462	141.7766	142.7360	142.0275	141.9201	142.3974	141.7860	142.2101	141.7956	142.7562	141.8074	142.6462	141.7767	142.7360	**133.3**
**C3**	122.5523	123.1684	123.6876	123.3576	123.9512	123.3499	124.1309	123.3441	124.1042	123.3640	124.2025	122.5523	123.1684	123.6877	123.3575	123.9512	123.3499	124.1309	123.3441	124.1042	123.3640	124.2025	**117.6**
**C4**	174.5864	175.2899	175.8454	175.6713	176.1994	175.6486	176.8750	175.6280	176.7138	175.6927	176.9999	174.5864	175.2899	175.8454	175.6714	176.1994	175.6486	176.8750	175.6280	176.7138	175.6927	176.9999	**168.3**
**C5**	120.3052	121.5543	122.1308	122.2063	122.9812	122.1668	123.1213	122.1296	123.0494	122.2436	123.2920	120.3052	121.5543	122.1307	122.2064	122.9812	122.1668	123.1213	122.1296	123.0494	122.2436	123.2920	**117.6**
**C6**	138.0548	139.9133	141.0549	141.0312	142.5246	140.9560	143.2797	140.8840	142.9881	141.1031	143.6154	138.0548	139.9133	141.0548	141.0312	142.5246	140.9560	143.2797	140.8840	142.9881	141.1031	143.6154	**133.3**
**C7**	202.3068	207.1882	208.3236	209.9129	210.2715	209.7555	215.9054	209.5991	214.6690	210.0607	217.0820	202.3068	207.1882	208.3236	209.9129	210.2715	209.7555	215.9054	209.5991	214.6690	210.0607	217.0820	**195.8**
**C8**	61.8804	62.9219	63.4905	63.3415	64.2714	63.3206	64.7791	63.3010	64.6980	63.3599	65.1757	61.8804	62.9219	63.4905	63.3415	64.2714	63.3206	64.7791	63.3010	64.6980	63.3599	65.1757	**70.4**
**C9**	34.1386	33.7725	34.0157	33.6193	33.9168	33.6264	34.0171	33.6361	33.9882	33.6121	34.0669	34.1386	33.7725	34.0157	33.6193	33.9168	33.6264	34.0171	33.6361	33.9882	33.6121	34.0669	**31.1**
**C10**	27.2497	27.6893	28.1703	28.0164	28.7326	27.9923	28.5308	27.9711	28.5292	28.0393	28.6777	27.2497	27.6893	28.1703	28.0164	28.7326	27.9922	28.5308	27.9711	28.5292	28.0393	28.6777	**26.9**
**C11**	27.2405	27.3246	27.6178	27.2971	27.7280	27.3011	27.7115	27.3070	27.7273	27.2928	27.8503	27.2405	27.3246	27.6179	27.2972	27.7280	27.3010	27.7115	27.3070	27.7273	27.2928	27.8503	**23.4**
**C12**	16.4477	16.5986	16.6969	16.7338	16.8490	16.7238	16.7955	16.7165	16.8029	16.7434	16.8068	16.4477	16.5986	16.6969	16.7339	16.8490	16.7239	16.7955	16.7165	16.8029	16.7434	16.8068	**13.8**
**C1`**	49.0825	49.8396	50.1387	50.3717	50.8895	50.3364	50.8814	50.3041	50.8419	50.4053	51.3647	53.3202	54.3295	54.6354	54.7498	55.2630	54.7265	55.3513	54.7053	55.3102	54.7713	55.6150	**53.3**
**C2`**	24.4610	24.8112	25.1567	24.9344	25.4163	24.9287	25.4177	24.9250	25.4350	24.9393	25.2394	23.9171	24.0510	24.4256	24.1914	24.6650	24.1829	24.5877	24.1770	24.5932	24.1990	24.4088	**24.2**
**C3`**	23.9171	24.0510	24.4257	24.1913	24.6650	24.1830	24.5877	24.1770	24.5932	24.1990	24.4088	24.4610	24.8112	25.1568	24.9345	25.4163	24.9287	25.4177	24.9250	25.4350	24.9393	25.2394	**24.1**
**C4`**	53.3202	54.3295	54.6354	54.7499	55.2630	54.7265	55.3513	54.7053	55.3102	54.7714	55.6150	49.0825	49.8396	50.1387	50.3716	50.8895	50.3365	50.8814	50.3041	50.8419	50.4053	51.3647	**56.3**
**H2**	8.9174	8.8134	8.8432	8.7435	8.7514	8.7471	8.7822	8.7508	8.7728	8.7400	8.7555	8.9174	8.8134	8.8432	8.7434	8.7514	8.7471	8.7822	8.7508	8.7728	8.7400	8.7555	**8.2**
**H3**	7.4415	7.5833	7.6682	7.6294	7.7421	7.6275	7.7648	7.6254	7.7556	7.6312	7.7794	7.4415	7.5833	7.6682	7.6294	7.7421	7.6275	7.7648	7.6254	7.7556	7.6312	7.7794	**7.36**
**H5**	7.2649	7.4404	7.5319	7.5137	7.6436	7.5094	7.6523	7.5050	7.6416	7.5178	7.6758	7.2649	7.4404	7.5319	7.5137	7.6436	7.5094	7.6523	7.5050	7.6416	7.5178	7.6758	**7.36**
**H6**	8.0825	8.2754	8.3711	8.3872	8.5176	8.3804	8.5843	8.3735	8.5554	8.3937	8.6106	8.0825	8.2754	8.3711	8.3872	8.5176	8.3804	8.5843	8.3735	8.5554	8.3937	8.6106	**8.2**
**H8**	4.2250	4.3093	4.3596	4.3771	4.4432	4.3727	4.5018	4.3684	4.4740	4.3812	4.4872	4.2250	4.3093	4.3596	4.3771	4.4432	4.3728	4.5018	4.3684	4.4740	4.3812	4.4872	**5.4**
**H(9A-9B)**	1.5828	1.6221	1.6279	1.6514	1.6739	1.6488	1.6780	1.6462	1.6704	1.6538	1.7051	1.5827	1.6221	1.6279	1.6514	1.6739	1.6488	1.6780	1.6462	1.6704	1.6538	1.7051	**2.08**
**H(10A)**	0.9846	0.8118	0.7997	0.7653	0.6810	0.7674	0.7233	0.7695	0.7123	0.7634	0.7225	0.9846	0.8118	0.7997	0.7653	0.6810	0.7674	0.7233	0.7695	0.7123	0.7634	0.7225	**1.14**
**H(10B)**	1.5615	1.4724	1.4065	1.3921	1.3035	1.3980	1.3085	1.4037	1.3114	1.3864	1.2811	1.5615	1.4724	1.4065	1.3921	1.3035	1.3980	1.3085	1.4037	1.3114	1.3864	1.2811	**1.28**
**H(11A-11B)**	0.9843	1.0042	0.9708	1.0203	0.9796	1.0195	0.9842	1.0186	0.9801	1.0210	0.9727	0.9843	1.0042	0.9708	1.0203	0.9796	1.0195	0.9842	1.0186	0.9801	1.0210	0.9727	**1.28**
**H(12A-12C)**	0.9401	0.9227	0.8984	0.9153	0.8731	0.9159	0.8823	0.9165	0.8805	0.9146	0.8760	0.9401	0.9227	0.8984	0.9153	0.8731	0.9159	0.8823	0.9165	0.8805	0.9146	0.8760	**0.82**
**H(1`A)**	2.6097	2.6012	2.5884	2.5909	2.5431	2.5917	2.5662	2.5924	2.5652	2.5900	2.5385	2.7507	2.7499	2.7315	2.7241	2.6891	2.7264	2.7061	2.7286	2.7091	2.7217	2.6739	**3.38**
**H(1`B)**	3.0196	3.0719	3.1020	3.1129	3.1735	3.1101	3.1671	3.1072	3.1606	3.1156	3.1693	2.7250	2.5707	2.5548	2.5480	2.5462	2.5484	2.5090	2.5489	2.5117	2.5477	2.5052	**3.71**
**H(2`A)**	1.5817	1.6240	1.5910	1.6417	1.5824	1.6409	1.6104	1.6401	1.6066	1.6424	1.6217	1.4608	1.5084	1.4765	1.5305	1.4719	1.5296	1.5091	1.5286	1.5038	1.5313	1.5265	**2.08**
**H(2`B)**	1.7190	1.7481	1.7091	1.8017	1.7564	1.7978	1.7602	1.7939	1.7507	1.8056	1.7841	1.5980	1.7096	1.6575	1.7817	1.7361	1.7774	1.7630	1.7731	1.7535	1.7857	1.8122	**2.24**
**H(3`A)**	1.4608	1.5084	1.4765	1.5305	1.4719	1.5296	1.5091	1.5286	1.5038	1.5313	1.5265	1.5817	1.6240	1.5910	1.6417	1.5824	1.6409	1.6104	1.6401	1.6066	1.6424	1.6217	**2.08**
**H(3`B)**	1.5980	1.7096	1.6575	1.7817	1.7361	1.7774	1.7630	1.7731	1.7535	1.7857	1.8122	1.7190	1.7481	1.7091	1.8017	1.7564	1.7978	1.7602	1.7939	1.7507	1.8056	1.7841	**2.16**
**H(4`A)**	2.7250	2.5707	2.5548	2.5480	2.5462	2.5484	2.5090	2.5489	2.5117	2.5477	2.5052	3.0196	3.0719	3.1020	3.1129	3.1735	3.1101	3.1671	3.1072	3.1606	3.1156	3.1693	**3.09**
**H(4`B)**	2.7507	2.7499	2.7315	2.7241	2.6891	2.7264	2.7061	2.7286	2.7091	2.7217	2.6739	2.6097	2.6012	2.5884	2.5908	2.5431	2.5917	2.5662	2.5924	2.5652	2.5900	2.5385	**3.68**

[a] methyl protons shifts are averaged

[b] according to [5]

Table S5. Cont. IV

Nuclei	R											S											Exp. [b]
	BLYP																						
	Gas	$CHCl_3$ (PCM)	$CHCl_3$ (SMD)	DMSO (PCM)	DMSO (SMD)	CH_3OH (PCM)	CH_3OH (SMD)	C_2H_5OH (PCM)	C_2H_5OH (SMD)	H_2O (PCM)	H_2O (SMD)	Gas	$CHCl_3$ (PCM)	$CHCl_3$ (SMD)	DMSO (PCM)	DMSO (SMD)	CH_3OH (PCM)	CH_3OH (SMD)	C_2H_5OH (PCM)	C_2H_5OH (SMD)	H_2O (PCM)	H_2O (SMD)	
**C1**	139.5343	140.9652	141.5943	141.7357	144.5172	141.9636	143.4256	141.7300	143.9164	142.1462	143.7361	139.5343	140.9652	141.5943	141.7357	144.5172	141.9636	143.4256	141.7300	143.9164	142.1461	143.7361	**132.2**
**C2**	137.2556	137.2722	137.8854	137.3003	138.1317	137.5735	138.4112	137.3854	138.4615	137.6688	138.5024	137.2556	137.2722	137.8854	137.3003	138.1317	137.5735	138.4112	137.3854	138.4615	137.6689	138.5024	**133.3**
**C3**	120.7921	121.4191	121.9349	121.5949	122.2771	121.8658	122.4301	121.6744	122.5403	121.9644	122.5819	120.7921	121.4191	121.9349	121.5949	122.2771	121.8658	122.4301	121.6744	122.5403	121.9643	122.5819	**117.6**
**C4**	178.0446	178.9278	179.6458	179.2623	179.9892	179.5224	180.4161	179.3202	180.4077	179.6422	180.6443	178.0446	178.9278	179.6458	179.2623	179.9892	179.5224	180.4161	179.3202	180.4077	179.6422	180.6443	**168.3**
**C5**	118.7346	119.8592	120.4200	120.4485	121.2822	120.6894	121.4129	120.4685	121.4684	120.8468	121.6561	118.7346	119.8592	120.4200	120.4485	121.2822	120.6894	121.4129	120.4685	121.4684	120.8467	121.6561	**117.6**
**C6**	133.6680	135.5732	136.6203	136.5089	137.5639	136.7306	138.0767	136.4903	137.9461	136.9249	138.5039	133.6680	135.5732	136.6203	136.5089	137.5639	136.7306	138.0767	136.4903	137.9461	136.9250	138.5039	**133.3**
**C7**	204.1746	208.9870	210.1011	211.5855	213.4733	211.7040	217.6195	211.3603	217.0831	212.0989	218.7030	204.1746	208.9870	210.1011	211.5855	213.4733	211.7040	217.6195	211.3603	217.0831	212.0991	218.7030	**195.8**
**C8**	70.4591	71.9072	72.3407	72.5539	73.6351	72.8057	73.9567	72.5927	74.1428	72.9387	74.2784	70.4591	71.9072	72.3407	72.5539	73.6351	72.8057	73.9567	72.5927	74.1428	72.9386	74.2784	**70.4**
**C9**	40.1256	39.7708	39.8045	39.5605	39.5512	39.8527	39.6759	39.6825	39.8226	39.9098	39.7834	40.1256	39.7708	39.8045	39.5605	39.5512	39.8527	39.6759	39.6825	39.8226	39.9098	39.7834	**31.1**
**C10**	30.9956	31.5038	31.9353	31.7631	32.4330	32.0244	32.0419	31.8239	32.2296	32.1419	32.2060	30.9956	31.5038	31.9353	31.7631	32.4330	32.0244	32.0419	31.8239	32.2296	32.1419	32.2060	**26.9**
**C11**	31.2026	31.3178	31.5898	31.2585	31.7489	31.5442	31.5381	31.3673	31.7410	31.6138	31.7406	31.2026	31.3178	31.5898	31.2585	31.7489	31.5442	31.5381	31.3673	31.7410	31.6138	31.7406	**23.4**
**C12**	17.0012	17.1470	17.1923	17.2337	17.4506	17.5040	17.2382	17.3127	17.4172	17.6046	17.3304	17.0012	17.1470	17.1923	17.2337	17.4506	17.5040	17.2382	17.3127	17.4172	17.6045	17.3304	**13.8**
**C1`**	55.2831	56.0370	56.4053	56.5833	57.2950	56.8244	57.1633	56.6042	57.2744	56.9826	57.7328	59.9830	61.1273	61.5437	61.6339	62.2990	61.8849	62.3006	61.6734	62.3750	62.0222	62.6696	**53.3**
**C2`**	27.7274	28.2834	28.6122	28.5322	29.0355	28.8001	28.9330	28.6054	29.0959	28.9041	28.8068	27.6644	27.8009	28.1574	27.9578	28.6760	28.2245	28.3891	28.0299	28.5704	28.3322	28.2523	**24.2**
**C3`**	27.6644	27.8009	28.1574	27.9578	28.6760	28.2246	28.3891	28.0299	28.5704	28.3322	28.2523	27.7274	28.2834	28.6122	28.5322	29.0355	28.8002	28.9330	28.6054	29.0959	28.9042	28.8068	**24.1**
**C4`**	59.9830	61.1273	61.5437	61.6339	62.2990	61.8849	62.3006	61.6734	62.3750	62.0222	62.6696	55.2831	56.0370	56.4053	56.5833	57.2950	56.8243	57.1633	56.6042	57.2744	56.9825	57.7328	**56.3**
**H2**	8.4743	8.3172	8.3808	8.2334	8.1741	8.2858	8.4013	8.2457	8.2052	8.2841	8.4152	8.4743	8.3172	8.3808	8.2334	8.1741	8.2858	8.4013	8.2457	8.2052	8.2841	8.4152	**8.2**
**H3**	7.1772	7.3089	7.3990	7.3393	7.4354	7.3857	7.6265	7.3394	7.4492	7.3955	7.6556	7.1772	7.3089	7.3990	7.3393	7.4354	7.3857	7.6265	7.3394	7.4492	7.3955	7.6556	**7.36**
**H5**	6.9737	7.1133	7.1996	7.1888	7.3510	7.2313	7.5411	7.1813	7.3621	7.2487	7.5789	6.9737	7.1133	7.1996	7.1888	7.3510	7.2313	7.5411	7.1813	7.3621	7.2487	7.5789	**7.36**
**H6**	7.8508	8.0008	8.0879	8.0664	8.1493	8.1100	8.3792	8.0612	8.1751	8.1252	8.4240	7.8508	8.0008	8.0879	8.0664	8.1493	8.1100	8.3792	8.0612	8.1751	8.1252	8.4240	**8.2**
**H8**	4.9846	5.0134	5.0311	5.0158	5.0719	5.0626	5.2534	5.0171	5.0718	5.0716	5.2461	4.9846	5.0134	5.0311	5.0158	5.0719	5.0626	5.2534	5.0171	5.0718	5.0716	5.2461	**5.4**
**H(9A-9B)**	1.7832	1.8231	1.8147	1.8352	1.7750	1.8823	1.9577	1.8368	1.7845	1.8907	1.9990	1.7832	1.8231	1.8147	1.8352	1.7750	1.8823	1.9577	1.8368	1.7845	1.8908	1.9990	**2.08**
**H(10A)**	1.0374	0.9218	0.9214	0.8984	0.8849	0.9453	1.0941	0.9001	0.9201	0.9545	1.1206	1.0374	0.9218	0.9214	0.8984	0.8849	0.9453	1.0941	0.9001	0.9201	0.9545	1.1206	**1.14**
**H(10B)**	1.7538	1.6293	1.5697	1.5413	1.4721	1.5943	1.6301	1.5547	1.4719	1.5913	1.6106	1.7538	1.6293	1.5697	1.5413	1.4721	1.5943	1.6301	1.5547	1.4719	1.5913	1.6106	**1.28**
**H(11A-11B)**	1.1322	1.1424	1.1178	1.1560	1.1325	1.2024	1.3013	1.1563	1.1316	1.2123	1.3029	1.1322	1.1424	1.1178	1.1560	1.1325	1.2024	1.3013	1.1563	1.1316	1.2123	1.3029	**1.28**
**H(12A-12C)**	0.9419	0.9312	0.9110	0.9251	0.9070	0.9727	1.0813	0.9279	0.9137	0.9802	1.0909	0.9419	0.9312	0.9110	0.9251	0.9070	0.9727	1.0813	0.9279	0.9137	0.9802	1.0909	**0.82**
**H(1`A)**	2.8633	2.8501	2.8310	2.8377	2.7979	2.8860	2.9760	2.8418	2.8118	2.8922	2.9544	2.9651	2.9898	2.9671	2.9680	2.9372	3.0180	3.1018	2.9754	2.9444	3.0205	3.0695	**3.38**
**H(1`B)**	3.3633	3.4089	3.4348	3.4379	3.4925	3.4835	3.6471	3.4365	3.4756	3.4949	3.6586	3.0499	2.8913	2.8353	2.8526	2.8547	2.9002	2.9868	2.8556	2.8331	2.9080	2.9912	**3.71**
**H(2`A)**	1.6735	1.7250	1.6968	1.7428	1.6993	1.7892	1.8918	1.7431	1.7216	1.7990	1.9188	1.5605	1.6040	1.5772	1.6253	1.5927	1.6713	1.7891	1.6248	1.6193	1.6819	1.8186	**2.08**
**H(2`B)**	1.9472	1.9524	1.9050	1.9814	1.9489	2.0255	2.1131	1.9774	1.9402	2.0400	2.1618	1.7709	1.8959	1.8552	1.9654	1.9523	2.0081	2.1360	1.9584	1.9618	2.0252	2.1995	**2.24**
**H(3`A)**	1.5605	1.6040	1.5772	1.6253	1.5927	1.6713	1.7891	1.6248	1.6193	1.6819	1.8186	1.6735	1.7250	1.6968	1.7428	1.6993	1.7892	1.8918	1.7431	1.7216	1.7990	1.9188	**2.08**
**H(3`B)**	1.7709	1.8959	1.8552	1.9654	1.9523	2.0081	2.1360	1.9584	1.9618	2.0252	2.1995	1.9472	1.9524	1.9050	1.9814	1.9489	2.0255	2.1131	1.9774	1.9402	2.0400	2.1618	**2.16**
**H(4`A)**	3.0499	2.8913	2.8353	2.8526	2.8547	2.9002	2.9868	2.8556	2.8331	2.9080	2.9912	3.3633	3.4089	3.4348	3.4379	3.4925	3.4835	3.6471	3.4365	3.4756	3.4949	3.6586	**3.09**
**H(4`B)**	2.9651	2.9898	2.9671	2.9680	2.9372	3.0180	3.1018	2.9754	2.9444	3.0205	3.0695	2.8633	2.8501	2.8310	2.8377	2.7979	2.8860	2.9760	2.8418	2.8118	2.8922	2.9544	**3.68**
Nuclei	BMK																						
**C1**	154.6743	155.0332	155.8462	155.1583	157.1596	155.1396	156.3883	155.3301	156.3397	155.3339	156.6046	154.6743	155.0332	155.8461	155.1582	157.1596	155.1397	156.3884	155.3301	156.3396	155.3339	156.6046	**132.2**
**C2**	154.6877	154.7844	155.5459	154.6549	155.3498	154.6489	155.8469	154.8543	155.6657	154.8228	155.7777	154.6877	154.7844	155.5461	154.6548	155.3498	154.6489	155.8467	154.8543	155.6656	154.8230	155.7777	**133.3**
**C3**	135.9138	136.6373	137.3465	136.8279	137.6933	136.8045	137.9405	136.9917	137.7955	137.0117	137.8683	135.9138	136.6373	137.3465	136.8279	137.6933	136.8046	137.9404	136.9917	137.7954	137.0118	137.8683	**117.6**
**C4**	189.4877	190.2116	190.9837	190.5712	191.4900	190.5337	192.1186	190.7070	191.8573	190.7682	192.2003	189.4877	190.2116	190.9838	190.5712	191.4900	190.5337	192.1187	190.7070	191.8575	190.7681	192.2003	**168.3**
**C5**	133.6513	134.9680	135.5869	135.6169	136.6787	135.5615	136.8546	135.7170	136.6382	135.8301	136.9525	133.6513	134.9680	135.5868	135.6171	136.6787	135.5614	136.8546	135.7170	136.6383	135.8300	136.9525	**117.6**
**C6**	151.3083	153.3283	154.3456	154.4536	156.4249	154.3671	157.0666	154.4912	156.6478	154.6954	157.4119	151.3083	153.3283	154.3454	154.4537	156.4249	154.3671	157.0667	154.4912	156.6476	154.6955	157.4119	**133.3**
**C7**	218.4275	222.9521	224.3909	225.3913	226.5095	225.2230	232.5082	225.2643	230.9435	225.7107	234.0514	218.4275	222.9521	224.3912	225.3913	226.5095	225.2230	232.5081	225.2643	230.9437	225.7103	234.0514	**195.8**
**C8**	71.7818	72.7560	73.5185	73.2212	74.7487	73.1831	75.2261	73.3534	74.8979	73.4147	76.0945	71.7818	72.7560	73.5184	73.2212	74.7487	73.1831	75.2261	73.3534	74.8979	73.4148	76.0945	**70.4**
**C9**	38.9270	38.8753	38.9914	38.7229	39.3377	38.7191	39.5697	38.9259	39.3287	38.8870	39.8348	38.9270	38.8753	38.9914	38.7229	39.3377	38.7192	39.5696	38.9259	39.3287	38.8870	39.8348	**31.1**
**C10**	31.5063	31.8431	32.2898	32.1042	32.9319	32.0706	32.5932	32.2494	32.5113	32.3000	32.8436	31.5063	31.8431	32.2898	32.1043	32.9319	32.0705	32.5931	32.2494	32.5113	32.3000	32.8436	**26.9**
**C11**	30.1638	30.0934	30.5280	30.0558	30.6708	30.0424	30.6321	30.2397	30.5415	30.2295	30.7089	30.1638	30.0934	30.5281	30.0558	30.6708	30.0423	30.6321	30.2397	30.5416	30.2295	30.7089	**23.4**
**C12**	17.9216	17.9663	18.1186	18.0457	18.3196	18.0223	18.2191	18.2104	18.1235	18.2298	18.1813	17.9215	17.9663	18.1186	18.0458	18.3196	18.0222	18.2190	18.2104	18.1236	18.2298	18.1812	**13.8**
**C1`**	56.2140	56.7042	57.2624	57.3354	57.9775	57.2761	57.8726	57.4271	57.7807	57.5515	57.9072	60.6747	61.2631	61.7498	61.5953	62.2577	61.5585	62.3486	61.7326	62.2082	61.7911	62.6655	**53.3**
**C2`**	28.1109	28.2884	28.6899	28.4501	29.0183	28.4238	28.9833	28.6085	28.8847	28.6357	28.9334	27.2299	27.4471	27.8451	27.6148	28.2163	27.5901	28.0996	27.7764	28.0059	27.7997	28.0827	**24.2**
**C3`**	27.2299	27.4471	27.8451	27.6148	28.2163	27.5901	28.0997	27.7764	28.0059	27.7998	28.0827	28.1109	28.2884	28.6900	28.4501	29.0184	28.4240	28.9833	28.6085	28.8847	28.6357	28.9334	**24.1**
**C4`**	60.6747	61.2631	61.7497	61.5952	62.2576	61.5585	62.3485	61.7326	62.2080	61.7910	62.6655	56.2140	56.7042	57.2624	57.3355	57.9775	57.2760	57.8726	57.4271	57.7808	57.5514	57.9072	**56.3**
**H2**	8.9918	8.9298	8.9868	8.9078	8.9029	8.9057	8.9653	8.9038	8.9648	8.9005	8.9628	8.9918	8.9298	8.9868	8.9078	8.9029	8.9057	8.9653	8.9038	8.9648	8.9005	8.9628	**8.2**
**H3**	7.6541	7.7829	7.8752	7.8302	7.9474	7.8246	7.9758	7.8190	7.9680	7.8259	7.9947	7.6541	7.7829	7.8752	7.8302	7.9474	7.8246	7.9758	7.8190	7.9680	7.8259	7.9947	**7.36**
**H5**	7.4938	7.6754	7.7641	7.7548	7.8712	7.7469	7.8878	7.7390	7.8788	7.7528	7.9076	7.4938	7.6754	7.7641	7.7548	7.8712	7.7469	7.8878	7.7390	7.8788	7.7528	7.9076	**7.36**
**H6**	8.3432	8.5078	8.6605	8.6235	8.7819	8.6124	8.8278	8.6015	8.8045	8.6250	8.8898	8.3432	8.5078	8.6605	8.6235	8.7819	8.6123	8.8278	8.6015	8.8044	8.6250	8.8898	**8.2**
**H8**	4.4144	4.4718	4.5598	4.5582	4.6028	4.5477	4.6667	4.5377	4.6543	4.5594	4.6485	4.4144	4.4718	4.5598	4.5582	4.6028	4.5477	4.6667	4.5377	4.6543	4.5594	4.6485	**5.4**
**H(9A-9B)**	1.6329	1.6976	1.6442	1.7193	1.7410	1.7145	1.7510	1.7099	1.7352	1.7143	1.8258	1.6329	1.6976	1.6442	1.7193	1.7410	1.7145	1.7510	1.7099	1.7352	1.7143	1.8258	**2.08**
**H(10A)**	1.0054	0.8156	0.8417	0.7556	0.6584	0.7548	0.7160	0.7541	0.7163	0.7469	0.7118	1.0054	0.8156	0.8417	0.7556	0.6584	0.7548	0.7160	0.7541	0.7163	0.7469	0.7118	**1.14**
**H(10B)**	1.5665	1.5142	1.4152	1.4197	1.3176	1.4233	1.3145	1.4271	1.3225	1.4069	1.3045	1.5665	1.5142	1.4152	1.4197	1.3176	1.4234	1.3145	1.4271	1.3225	1.4069	1.3045	**1.28**
**H(11A-11B)**	0.9568	0.9779	0.9496	0.9998	0.9492	0.9949	0.9534	0.9903	0.9549	0.9951	0.9505	0.9568	0.9779	0.9497	0.9998	0.9492	0.9949	0.9534	0.9903	0.9550	0.9951	0.9505	**1.28**
**H(12A-12C)**	0.8618	0.8474	0.8194	0.8457	0.7952	0.8425	0.7967	0.8395	0.8006	0.8395	0.8000	0.8617	0.8474	0.8194	0.8457	0.7952	0.8425	0.7967	0.8395	0.8007	0.8395	0.8000	**0.82**
**H(1`A)**	2.6238	2.6293	2.5970	2.6303	2.5835	2.6272	2.6067	2.6240	2.6082	2.6234	2.5463	2.7737	2.7853	2.7534	2.7712	2.7426	2.7699	2.7487	2.7683	2.7505	2.7626	2.7334	**3.38**
**H(1`B)**	3.0937	3.1310	3.1474	3.1725	3.2050	3.1661	3.1984	3.1595	3.1935	3.1689	3.1717	2.7218	2.5991	2.5747	2.5696	2.5484	2.5668	2.5121	2.5643	2.5131	2.5631	2.5093	**3.71**
**H(2`A)**	1.6066	1.6472	1.6208	1.6740	1.6181	1.6691	1.6399	1.6642	1.6380	1.6690	1.6497	1.4626	1.5173	1.4830	1.5420	1.4897	1.5377	1.5230	1.5334	1.5190	1.5364	1.5474	**2.08**
**H(2`B)**	1.7500	1.7801	1.7577	1.8454	1.7854	1.8368	1.7922	1.8285	1.7891	1.8442	1.7924	1.6778	1.7533	1.6954	1.8157	1.7687	1.8087	1.7888	1.8016	1.7827	1.8127	1.8332	**2.24**
**H(3`A)**	1.4626	1.5173	1.4830	1.5420	1.4897	1.5378	1.5230	1.5334	1.5190	1.5365	1.5474	1.6066	1.6472	1.6208	1.6740	1.6181	1.6691	1.6400	1.6642	1.6380	1.6690	1.6497	**2.08**
**H(3`B)**	1.6778	1.7533	1.6954	1.8157	1.7687	1.8087	1.7888	1.8016	1.7827	1.8127	1.8332	1.7500	1.7801	1.7578	1.8454	1.7854	1.8368	1.7922	1.8285	1.7891	1.8442	1.7924	**2.16**
**H(4`A)**	2.7218	2.5991	2.5747	2.5696	2.5484	2.5668	2.5120	2.5643	2.5131	2.5631	2.5093	3.0937	3.1310	3.1474	3.1725	3.2050	3.1661	3.1984	3.1595	3.1935	3.1689	3.1717	**3.09**
**H(4`B)**	2.7737	2.7853	2.7534	2.7712	2.7426	2.7699	2.7487	2.7683	2.7505	2.7626	2.7334	2.6238	2.6293	2.5970	2.6303	2.5835	2.6272	2.6068	2.6240	2.6082	2.6235	2.5463	**3.68**

[a] methyl protons shifts are averaged
[b] according to [5]

Table S5. Cont. V

Nuclei	R											S											Exp.[b]
	BP86																						
	Gas	$CHCl_3$ (PCM)	$CHCl_3$ (SMD)	DMSO (PCM)	DMSO (SMD)	CH_3OH (PCM)	CH_3OH (SMD)	C_2H_5OH (PCM)	C_2H_5OH (SMD)	H_2O (PCM)	H_2O (SMD)	Gas	$CHCl_3$ (PCM)	$CHCl_3$ (SMD)	DMSO (PCM)	DMSO (SMD)	CH_3OH (PCM)	CH_3OH (SMD)	C_2H_5OH (PCM)	C_2H_5OH (SMD)	H_2O (PCM)	H_2O (SMD)	
**C1**	136.1088	137.3107	138.0964	137.7831	139.1376	137.7682	138.6145	137.7476	138.6273	137.7918	138.9792	136.1088	137.3107	138.0965	137.7831	139.1376	137.7682	138.6145	137.7476	138.6273	137.7918	138.9792	**132.2**
**C2**	135.7786	135.9025	136.4035	135.7790	136.2500	135.7953	136.7578	135.8067	136.6297	135.7589	136.8526	135.7786	135.9025	136.4034	135.7790	136.2500	135.7953	136.7578	135.8067	136.6297	135.7589	136.8526	**133.3**
**C3**	119.6549	120.5419	121.0715	120.7433	121.3759	120.7419	121.7193	120.7350	121.6286	120.7394	121.9674	119.6549	120.5419	121.0715	120.7433	121.3759	120.7419	121.7193	120.7350	121.6286	120.7394	121.9674	**117.6**
**C4**	174.5771	175.6557	176.2903	176.0366	176.7190	176.0219	177.3180	176.0021	177.1695	176.0455	177.6153	174.5771	175.6557	176.2903	176.0366	176.7190	176.0219	177.3180	176.0021	177.1695	176.0455	177.6153	**168.3**
**C5**	117.7054	119.0766	119.6753	119.6781	120.4892	119.6486	120.7558	119.6139	120.6434	119.7013	121.0874	117.7054	119.0766	119.6753	119.6781	120.4892	119.6486	120.7558	119.6139	120.6434	119.7013	121.0874	**117.6**
**C6**	132.2467	134.2864	135.3229	135.3472	136.8781	135.2854	137.5200	135.2189	137.2791	135.4016	138.0862	132.2467	134.2864	135.3229	135.3472	136.8781	135.2854	137.5200	135.2189	137.2791	135.4016	138.0862	**133.3**
**C7**	201.2567	205.6591	206.8764	207.8846	208.7243	207.7624	212.8796	207.6342	211.9294	207.9947	214.0254	201.2567	205.6591	206.8764	207.8846	208.7243	207.7624	212.8796	207.6342	211.9294	207.9947	214.0254	**195.8**
**C8**	68.1361	69.5249	70.2639	70.0439	71.1738	70.0330	71.7242	70.0145	71.5644	70.0470	72.2963	68.1361	69.5249	70.2639	70.0439	71.1738	70.0330	71.7242	70.0145	71.5644	70.0470	72.2963	**70.4**
**C9**	38.8652	38.7336	38.8380	38.5423	38.8956	38.5618	39.1809	38.5758	39.0858	38.5184	39.4764	38.8652	38.7336	38.8380	38.5423	38.8956	38.5619	39.1809	38.5758	39.0858	38.5184	39.4764	**31.1**
**C10**	29.5240	30.1529	30.5449	30.4255	31.1246	30.4126	30.9274	30.3953	30.9087	30.4332	31.2594	29.5240	30.1529	30.5449	30.4255	31.1246	30.4126	30.9274	30.3953	30.9087	30.4332	31.2594	**26.9**
**C11**	29.4418	29.6972	29.8600	29.5824	29.9487	29.6022	29.9365	29.6159	29.9309	29.5580	30.2368	29.4418	29.6972	29.8601	29.5824	29.9487	29.6021	29.9365	29.6159	29.9309	29.5580	30.2368	**23.4**
**C12**	16.5296	16.7824	16.7604	16.8352	16.8320	16.8373	16.8060	16.8346	16.7990	16.8289	16.9542	16.5296	16.7824	16.7604	16.8352	16.8320	16.8373	16.8060	16.8346	16.7990	16.8290	16.9543	**13.8**
**C1`**	53.7220	54.6467	54.8434	55.1464	55.6019	55.1212	55.6068	55.0916	55.5138	55.1663	56.3013	58.2667	59.6104	59.9327	60.0596	60.4729	60.0450	60.6886	60.0246	60.6186	60.0678	61.1204	**53.3**
**C2`**	26.2439	26.8649	27.1493	27.0337	27.4594	27.0338	27.4989	27.0283	27.4820	27.0283	27.4483	25.9185	26.2129	26.4676	26.3545	26.7189	26.3512	26.7270	26.3431	26.7097	26.3531	26.7220	**24.2**
**C3`**	25.9185	26.2129	26.4677	26.3545	26.7189	26.3512	26.7270	26.3431	26.7097	26.3531	26.7220	26.2438	26.8649	27.1491	27.0337	27.4594	27.0338	27.4989	27.0283	27.4820	27.0283	27.4483	**24.1**
**C4`**	58.2667	59.6104	59.9326	60.0596	60.4729	60.0450	60.6886	60.0246	60.6186	60.0678	61.1203	53.7220	54.6467	54.8434	55.1464	55.6019	55.1212	55.6068	55.0916	55.5138	55.1663	56.3010	**56.3**
**H2**	8.5202	8.4179	8.4255	8.3541	8.3445	8.3584	8.3760	8.3625	8.3748	8.3498	8.3764	8.5202	8.4179	8.4255	8.3541	8.3445	8.3584	8.3761	8.3625	8.3748	8.3498	8.3764	**8.2**
**H3**	7.1794	7.3318	7.4109	7.3778	7.4831	7.3764	7.5086	7.3746	7.4988	7.3787	7.5375	7.1794	7.3318	7.4109	7.3778	7.4831	7.3764	7.5086	7.3746	7.4988	7.3787	7.5375	**7.36**
**H5**	7.0044	7.1582	7.2405	7.2245	7.3556	7.2214	7.3589	7.2179	7.3494	7.2272	7.4008	7.0044	7.1582	7.2405	7.2245	7.3556	7.2214	7.3589	7.2179	7.3494	7.2272	7.4008	**7.36**
**H6**	7.9135	8.0785	8.1409	8.1670	8.2621	8.1621	8.3188	8.1568	8.3018	8.1713	8.3592	7.9135	8.0785	8.1409	8.1670	8.2621	8.1621	8.3188	8.1568	8.3018	8.1713	8.3592	**8.2**
**H8**	4.9923	5.0231	5.0204	5.0488	5.0657	5.0471	5.0991	5.0452	5.0903	5.0501	5.0979	4.9923	5.0231	5.0204	5.0488	5.0657	5.0471	5.0991	5.0452	5.0903	5.0501	5.0979	**5.4**
**H(9A-9B)**	1.8866	1.9233	1.9092	1.9458	1.9709	1.9451	1.9663	1.9441	1.9592	1.9462	2.0157	1.8866	1.9233	1.9092	1.9458	1.9709	1.9451	1.9663	1.9441	1.9592	1.9462	2.0157	**2.08**
**H(10A)**	1.0160	0.9024	0.8508	0.8713	0.7697	0.8724	0.8288	0.8734	0.8187	0.8670	0.8515	1.0160	0.9024	0.8508	0.8713	0.7697	0.8724	0.8288	0.8734	0.8187	0.8700	0.8515	**1.14**
**H(10B)**	1.7876	1.6671	1.5902	1.5808	1.4877	1.5871	1.4750	1.5931	1.4819	1.5746	1.4536	1.7876	1.6671	1.5902	1.5808	1.4877	1.5871	1.4750	1.5931	1.4819	1.5746	1.4535	**1.28**
**H(11A-11B)**	1.1095	1.1243	1.0787	1.1354	1.0880	1.1356	1.0865	1.1355	1.0849	1.1348	1.0877	1.1095	1.1243	1.0788	1.1354	1.0880	1.1356	1.0865	1.1355	1.0849	1.1348	1.0877	**1.28**
**H(12A-12C)**	0.9116	0.9045	0.8683	0.8963	0.8471	0.8975	0.8543	0.8985	0.8534	0.8948	0.8614	0.9116	0.9045	0.8683	0.8963	0.8471	0.8975	0.8543	0.8985	0.8534	0.8948	0.8614	**0.82**
**H(1`A)**	2.8037	2.7998	2.7761	2.7875	2.7401	2.7890	2.7544	2.7902	2.7555	2.7857	2.7327	2.9270	2.9443	2.9250	2.9302	2.8972	2.9326	2.9032	2.9346	2.9064	2.9275	2.8769	**3.38**
**H(1`B)**	3.3751	3.4176	3.4367	3.4453	3.4990	3.4441	3.4838	3.4427	3.4815	3.4461	3.4880	3.1070	2.9109	2.8780	2.8605	2.8494	2.8627	2.8030	2.8649	2.8065	2.8583	2.7978	**3.71**
**H(2`A)**	1.6748	1.7240	1.6882	1.7424	1.6850	1.7422	1.7086	1.7417	1.7041	1.7422	1.7337	1.5435	1.5855	1.5510	1.6075	1.5463	1.6069	1.5802	1.6060	1.5736	1.6077	1.6113	**2.08**
**H(2`B)**	1.9335	1.9409	1.8850	1.9746	1.9168	1.9722	1.9183	1.9696	1.9114	1.9768	1.9649	1.7819	1.8931	1.8408	1.9568	1.9001	1.9536	1.9222	1.9501	1.9124	1.9596	1.9912	**2.24**
**H(3`A)**	1.5435	1.5855	1.5510	1.6075	1.5463	1.6069	1.5802	1.6060	1.5736	1.6077	1.6113	1.6748	1.7240	1.6882	1.7424	1.6850	1.7422	1.7086	1.7417	1.7041	1.7422	1.7337	**2.08**
**H(3`B)**	1.7819	1.8931	1.8408	1.9568	1.9001	1.9536	1.9222	1.9501	1.9124	1.9596	1.9912	1.9335	1.9409	1.8850	1.9746	1.9168	1.9722	1.9183	1.9696	1.9114	1.9768	1.9649	**2.16**
**H(4`A)**	3.1070	2.9109	2.8780	2.8605	2.8494	2.8627	2.8030	2.8649	2.8065	2.8583	2.7978	3.3751	3.4176	3.4367	3.4452	3.4990	3.4441	3.4838	3.4427	3.4815	3.4461	3.4880	**3.09**
**H(4`B)**	2.9270	2.9443	2.9250	2.9302	2.8972	2.9326	2.9032	2.9346	2.9064	2.9275	2.8769	2.8037	2.7998	2.7761	2.7875	2.7401	2.7890	2.7544	2.7902	2.7555	2.7857	2.7327	**3.68**
Nuclei	CAM-B3LYP																						
**C1**	140.9000	141.8642	142.5134	142.3393	143.9931	142.3245	143.2389	142.3087	143.3053	142.3544	143.4213	140.8999	141.8642	142.5135	142.3392	143.9931	142.3245	143.2389	142.3087	143.3053	142.3544	143.4212	**132.2**
**C2**	142.3592	142.3291	142.7581	142.1837	142.6753	142.1979	143.2028	142.2124	143.0855	142.1723	143.1775	142.3594	142.3291	142.7582	142.1837	142.6754	142.1979	143.2028	142.2124	143.0855	142.1723	143.1775	**133.3**
**C3**	123.8875	124.5615	125.0610	124.7361	125.3833	124.7328	125.5918	124.7293	125.5399	124.7413	125.6630	123.8876	124.5615	125.0610	124.7363	125.3832	124.7328	125.5918	124.7293	125.5399	124.7412	125.6631	**117.6**
**C4**	177.7476	178.5115	179.0552	178.8743	179.4733	178.8556	180.0785	178.8375	179.9280	178.8943	180.2203	177.7477	178.5115	179.0551	178.8744	179.4733	178.8556	180.0785	178.8375	179.9280	178.8944	180.2204	**168.3**
**C5**	121.8135	123.1003	123.6648	123.7417	124.5660	123.7060	124.7320	123.6710	124.6384	123.7775	124.8939	121.8135	123.1003	123.6648	123.7415	124.5660	123.7060	124.7320	123.6709	124.6384	123.7775	124.8939	**117.6**
**C6**	138.4687	140.3307	141.5035	141.4320	142.9843	141.3615	143.6195	141.2922	143.3572	141.5021	144.0436	138.4687	140.3307	141.5034	141.4319	142.9843	141.3615	143.6195	141.2922	143.3572	141.5021	144.0435	**133.3**
**C7**	206.2190	211.2347	212.3397	213.9879	214.6304	213.8311	220.0283	213.6740	218.8123	214.1375	221.0855	206.2190	211.2347	212.3397	213.9878	214.6304	213.8311	220.0283	213.6739	218.8123	214.1374	221.0856	**195.8**
**C8**	64.6063	65.7712	66.4410	66.2572	67.2927	66.2389	67.9060	66.2194	67.7480	66.2753	68.2828	64.6064	65.7712	66.4410	66.2573	67.2928	66.2389	67.9060	66.2194	67.7480	66.2753	68.2828	**70.4**
**C9**	35.3917	35.0454	35.2739	34.8733	35.1875	34.8869	35.3176	34.9005	35.2558	34.8622	35.4063	35.3917	35.0454	35.2740	34.8733	35.1874	34.8869	35.3176	34.9006	35.2558	34.8622	35.4064	**31.1**
**C10**	28.1866	28.6440	29.1029	28.9663	29.6973	28.9451	29.5046	28.9250	29.4847	28.9892	29.6822	28.1867	28.6440	29.1029	28.9664	29.6974	28.9451	29.5046	28.9249	29.4847	28.9892	29.6823	**26.9**
**C11**	27.8708	27.9976	28.2402	27.9403	28.3933	27.9501	28.3825	27.9597	28.3778	27.9329	28.5418	27.8708	27.9976	28.2403	27.9403	28.3933	27.9501	28.3825	27.9596	28.3778	27.9330	28.5418	**23.4**
**C12**	16.2567	16.4391	16.4726	16.5524	16.6588	16.5468	16.6036	16.5421	16.5992	16.5601	16.6163	16.2567	16.4391	16.4726	16.5523	16.6589	16.5468	16.6036	16.5420	16.5992	16.5601	16.6163	**13.8**
**C1`**	50.8435	51.6933	51.8918	52.2148	52.7698	52.1836	52.7649	52.1535	52.6947	52.2469	53.2563	55.1554	56.2469	56.5379	56.6717	57.1952	56.6514	57.3190	56.6314	57.2584	56.6928	57.5773	**53.3**
**C2`**	25.0342	25.4284	25.7358	25.5492	26.0245	25.5467	26.0407	25.5443	26.0345	25.5535	25.8833	24.4922	24.6878	24.9644	24.8119	25.2810	24.8071	25.1973	24.8028	25.1947	24.8187	25.0647	**24.2**
**C3`**	24.4923	24.6878	24.9643	24.8118	25.2810	24.8071	25.1973	24.8028	25.1947	24.8188	25.0648	25.0342	25.4285	25.7358	25.5492	26.0245	25.5467	26.0407	25.5444	26.0345	25.5534	25.8833	**24.1**
**C4`**	55.1553	56.2469	56.5378	56.6715	57.1952	56.6514	57.3190	56.6314	57.2584	56.6927	57.5773	50.8435	51.6933	51.8918	52.2147	52.7697	52.1836	52.7649	52.1534	52.6947	52.2470	53.2562	**56.3**
**H2**	8.8102	8.6986	8.7289	8.6236	8.6210	8.6275	8.6425	8.6316	8.6418	8.6199	8.6323	8.8102	8.6986	8.7289	8.6236	8.6210	8.6275	8.6425	8.6316	8.6418	8.6199	8.6322	**8.2**
**H3**	7.4041	7.5438	7.6298	7.5866	7.7036	7.5847	7.7229	7.5828	7.7152	7.5883	7.7368	7.4041	7.5438	7.6298	7.5866	7.7036	7.5847	7.7229	7.5828	7.7152	7.5883	7.7368	**7.36**
**H5**	7.2406	7.4128	7.5085	7.4855	7.6154	7.4812	7.6240	7.4771	7.6145	7.4895	7.6484	7.2405	7.4128	7.5085	7.4855	7.6154	7.4812	7.6240	7.4771	7.6145	7.4895	7.6484	**7.36**
**H6**	8.0497	8.2319	8.3255	8.3359	8.4632	8.3292	8.5140	8.3227	8.4924	8.3422	8.5479	8.0497	8.2319	8.3255	8.3359	8.4632	8.3292	8.5140	8.3227	8.4924	8.3422	8.5479	**8.2**
**H8**	4.4210	4.4951	4.5329	4.5563	4.6154	4.5520	4.6593	4.5478	4.6382	4.5605	4.6576	4.4210	4.4951	4.5329	4.5563	4.6153	4.5520	4.6593	4.5478	4.6382	4.5605	4.6576	**5.4**
**H(9A-9B)**	1.6746	1.7066	1.7208	1.7333	1.7558	1.7312	1.7557	1.7292	1.7500	1.7352	1.7902	1.6746	1.7066	1.7208	1.7333	1.7558	1.7312	1.7557	1.7292	1.7500	1.7352	1.7902	**2.08**
**H(10A)**	0.9793	0.8280	0.7905	0.7827	0.6958	0.7844	0.7355	0.7864	0.7297	0.7811	0.7458	0.9793	0.8280	0.7905	0.7827	0.6958	0.7844	0.7355	0.7864	0.7297	0.7811	0.7458	**1.14**
**H(10B)**	1.6254	1.5324	1.4640	1.4472	1.3642	1.4530	1.3590	1.4589	1.3655	1.4418	1.3321	1.6253	1.5324	1.4640	1.4472	1.3642	1.4530	1.3590	1.4589	1.3655	1.4418	1.3321	**1.28**
**H(11A-11B)**	1.0293	1.0490	1.0127	1.0612	1.0239	1.0605	1.0223	1.0600	1.0216	1.0618	1.0127	1.0293	1.0490	1.0127	1.0612	1.0239	1.0605	1.0223	1.0600	1.0216	1.0618	1.0127	**1.28**
**H(12A-12C)**	0.9496	0.9353	0.9077	0.9264	0.8870	0.9270	0.8928	0.9277	0.8929	0.9257	0.8870	0.9496	0.9353	0.9077	0.9264	0.8871	0.9270	0.8928	0.9277	0.8929	0.9257	0.8870	**0.82**
**H(1`A)**	2.7110	2.7033	2.6912	2.6925	2.6485	2.6932	2.6648	2.6941	2.6655	2.6917	2.6365	2.8448	2.8448	2.8307	2.8212	2.7916	2.8233	2.8027	2.8254	2.8061	2.8191	2.7687	**3.38**
**H(1`B)**	3.1229	3.1689	3.2079	3.2088	3.2727	3.2060	3.2605	3.2034	3.2561	3.2114	3.2635	2.8211	2.6606	2.6465	2.6360	2.6365	2.6367	2.5996	2.6376	2.6000	2.6354	2.5884	**3.71**
**H(2`A)**	1.6362	1.6816	1.6509	1.6997	1.6466	1.6989	1.6722	1.6981	1.6689	1.7005	1.6821	1.5140	1.5653	1.5337	1.5881	1.5366	1.5871	1.5718	1.5862	1.5662	1.5891	1.5878	**2.08**
**H(2`B)**	1.7982	1.8246	1.7824	1.8735	1.8313	1.8698	1.8300	1.8663	1.8234	1.8771	1.8557	1.6828	1.7942	1.7423	1.8642	1.8262	1.8599	1.8521	1.8557	1.8411	1.8683	1.8964	**2.24**
**H(3`A)**	1.5140	1.5653	1.5337	1.5881	1.5366	1.5871	1.5718	1.5862	1.5662	1.5891	1.5878	1.6362	1.6817	1.6509	1.6997	1.6466	1.6989	1.6722	1.6981	1.6689	1.7004	1.6821	**2.08**
**H(3`B)**	1.6828	1.7942	1.7423	1.8642	1.8262	1.8599	1.8521	1.8557	1.8411	1.8683	1.8965	1.7982	1.8246	1.7824	1.8735	1.8312	1.8698	1.8300	1.8663	1.8234	1.8771	1.8557	**2.16**
**H(4`A)**	2.8211	2.6606	2.6465	2.6360	2.6365	2.6367	2.5996	2.6376	2.6000	2.6354	2.5884	3.1229	3.1689	3.2079	3.2088	3.2727	3.2060	3.2605	3.2033	3.2561	3.2114	3.2635	**3.09**
**H(4`B)**	2.8448	2.8448	2.8307	2.8212	2.7916	2.8233	2.8027	2.8254	2.8061	2.8191	2.7687	2.7110	2.7033	2.6912	2.6925	2.6485	2.6932	2.6648	2.6941	2.6655	2.6917	2.6364	**3.68**

[a] methyl protons shifts are averaged
[b] according to [5]

Table S5. Cont. VI

Nuclei	R											S											Exp. [b]
	HF																						
	Gas	$CHCl_3$ (PCM)	$CHCl_3$ (SMD)	DMSO (PCM)	DMSO (SMD)	CH_3OH (PCM)	CH_3OH (SMD)	C_2H_5OH (PCM)	C_2H_5OH (SMD)	H_2O (PCM)	H_2O (SMD)	Gas	$CHCl_3$ (PCM)	$CHCl_3$ (SMD)	DMSO (PCM)	DMSO (SMD)	CH_3OH (PCM)	CH_3OH (SMD)	C_2H_5OH (PCM)	C_2H_5OH (SMD)	H_2O (PCM)	H_2O (SMD)	
**C1**	136.4614	137.5594	137.9396	138.2608	141.1588	138.1812	139.8214	138.1121	140.2588	138.3352	140.0156	136.4614	137.5594	137.9396	138.2608	141.1590	138.1812	139.8214	138.1123	140.2588	138.3352	140.0156	**132.2**
**C2**	144.3880	144.1261	144.6323	143.8817	144.0884	143.9028	144.6434	143.9234	144.4625	143.8613	144.6473	144.3881	144.1261	144.6323	143.8817	144.0886	143.9028	144.6434	143.9233	144.4625	143.8613	144.6474	**133.3**
**C3**	120.4753	121.0267	121.4081	121.1842	121.9458	121.1734	121.9413	121.1639	121.9814	121.1933	121.9865	120.4753	121.0267	121.4081	121.1842	121.9457	121.1734	121.9413	121.1637	121.9814	121.1933	121.9866	**117.6**
**C4**	171.1212	171.5718	172.1467	171.7971	172.0666	171.7897	172.8696	171.7808	172.6355	171.8033	173.0028	171.1213	171.5718	172.1467	171.7971	172.0666	171.7897	172.8696	171.7807	172.6354	171.8033	173.0028	**168.3**
**C5**	117.8593	119.1512	119.5795	119.8198	120.6684	119.7762	120.6727	119.7340	120.6376	119.8599	120.8287	117.8593	119.1512	119.5795	119.8198	120.6682	119.7762	120.6727	119.7339	120.6376	119.8599	120.8287	**117.6**
**C6**	139.9930	141.4756	142.7481	142.2938	142.9370	142.2616	143.9248	142.2248	143.4971	142.3244	144.2237	139.9930	141.4756	142.7481	142.2938	142.9368	142.2616	143.9248	142.2249	143.4971	142.3244	144.2236	**133.3**
**C7**	196.4929	202.2641	202.9719	205.5898	206.7292	205.3508	213.4358	205.1213	212.0643	205.8156	214.4845	196.4932	202.2641	202.9719	205.5898	206.7294	205.3508	213.4358	205.1213	212.0643	205.8152	214.4845	**195.8**
**C8**	55.7610	56.8718	57.1616	57.3487	58.2739	57.3120	58.7513	57.2794	58.6807	57.3824	58.9817	55.7610	56.8718	57.1617	57.3487	58.2738	57.3120	58.7513	57.2794	58.6807	57.3825	58.9816	**70.4**
**C9**	29.9395	29.4452	29.6637	29.2244	29.3513	29.2420	29.2641	29.2585	29.2555	29.2074	29.2254	29.9396	29.4452	29.6637	29.2244	29.3513	29.2420	29.2641	29.2587	29.2555	29.2074	29.2254	**31.1**
**C10**	24.5112	24.8855	25.3200	25.1270	25.7191	25.1133	25.5163	25.0997	25.4870	25.1397	25.6454	24.5113	24.8855	25.3200	25.1270	25.7191	25.1133	25.5163	25.0997	25.4869	25.1397	25.6454	**26.9**
**C11**	24.9934	25.0012	25.2811	24.9675	25.3997	24.9711	25.3586	24.9750	25.3631	24.9631	25.4875	24.9934	25.0012	25.2811	24.9675	25.3997	24.9711	25.3586	24.9751	25.3632	24.9630	25.4875	**23.4**
**C12**	16.1893	16.3050	16.4087	16.4083	16.5840	16.3998	16.5424	16.3920	16.5403	16.4161	16.5585	16.1893	16.3050	16.4087	16.4083	16.5840	16.3998	16.5424	16.3920	16.5403	16.4161	16.5585	**13.8**
**C1\`**	44.6495	45.3638	45.5995	45.8292	46.3745	45.7972	46.3770	45.7670	46.3398	45.8597	46.7427	48.5492	49.4089	49.6427	49.7948	50.2999	49.7716	50.3741	49.7486	50.3061	49.8158	50.6100	**53.3**
**C2\`**	22.8232	23.0884	23.3924	23.1170	23.5554	23.1184	23.5262	23.1198	23.5079	23.1145	23.3375	22.1997	22.2737	22.6410	22.3541	22.9562	22.3468	22.8286	22.3405	22.8432	22.3604	22.6316	**24.2**
**C3\`**	22.1997	22.2737	22.6410	22.3541	22.9561	22.3468	22.8286	22.3405	22.8431	22.3603	22.6316	22.8232	23.0884	23.3924	23.1170	23.5554	23.1184	23.5262	23.1197	23.5079	23.1145	23.3375	**24.1**
**C4\`**	48.5492	49.4089	49.6427	49.7948	50.3000	49.7716	50.3741	49.7487	50.3060	49.8158	50.6100	44.6494	45.3638	45.5995	45.8291	46.3746	45.7972	46.3770	45.7670	46.3397	45.8597	46.7426	**56.3**
**H2**	9.0711	8.9082	8.9926	8.7974	8.6994	8.8084	8.7504	8.8181	8.7213	8.7871	8.7457	9.0711	8.9082	8.9926	8.7974	8.6994	8.8084	8.7504	8.8181	8.7212	8.7871	8.7457	**8.2**
**H3**	7.4358	7.5753	7.6646	7.6196	7.7433	7.6175	7.7557	7.6152	7.7493	7.6217	7.7711	7.4358	7.5753	7.6646	7.6196	7.7433	7.6175	7.7557	7.6152	7.7493	7.6217	7.7711	**7.36**
**H5**	7.2401	7.4378	7.5258	7.5268	7.6588	7.5216	7.6718	7.5162	7.6614	7.5317	7.6960	7.2401	7.4378	7.5258	7.5268	7.6588	7.5216	7.6718	7.5162	7.6614	7.5317	7.6960	**7.36**
**H6**	8.0965	8.3044	8.4316	8.4110	8.5225	8.4061	8.5996	8.4004	8.5605	8.4158	8.6363	8.0965	8.3044	8.4316	8.4110	8.5225	8.4061	8.5996	8.4004	8.5606	8.4158	8.6363	**8.2**
**H8**	3.6381	3.7437	3.8354	3.8239	3.9329	3.8188	4.0011	3.8133	3.9706	3.8288	3.9979	3.6381	3.7437	3.8354	3.8239	3.9329	3.8188	4.0011	3.8133	3.9706	3.8288	3.9979	**5.4**
**H(9A-9B)**	1.3084	1.3301	1.3445	1.3363	1.2736	1.3379	1.2820	1.3390	1.2675	1.3349	1.3033	1.3084	1.3301	1.3445	1.3363	1.2736	1.3379	1.2820	1.3390	1.2675	1.3349	1.3033	**2.08**
**H(10A)**	0.9656	0.7280	0.7412	0.6826	0.6635	0.6833	0.6994	0.6843	0.7008	0.6822	0.7041	0.9656	0.7280	0.7412	0.6826	0.6635	0.6833	0.6994	0.6843	0.7008	0.6822	0.7041	**1.14**
**H(10B)**	1.3770	1.3012	1.2518	1.2206	1.1379	1.2267	1.1517	1.2323	1.1569	1.2149	1.1303	1.3771	1.3012	1.2518	1.2206	1.1379	1.2267	1.1517	1.2323	1.1568	1.2149	1.1303	**1.28**
**H(11A-11B)**	0.8644	0.8763	0.8546	0.8936	0.8559	0.8927	0.8656	0.8916	0.8636	0.8945	0.8622	0.8644	0.8763	0.8546	0.8936	0.8559	0.8927	0.8656	0.8917	0.8636	0.8944	0.8622	**1.28**
**H(12A-12C)**	0.9257	0.9009	0.8816	0.8957	0.8713	0.8956	0.8816	0.8955	0.8831	0.8958	0.8787	0.9257	0.9009	0.8817	0.8957	0.8713	0.8956	0.8816	0.8956	0.8831	0.8958	0.8787	**0.82**
**H(1\`A)**	2.3729	2.3640	2.3490	2.3541	2.3106	2.3546	2.3370	2.3550	2.3368	2.3537	2.3150	2.5341	2.5327	2.5042	2.4991	2.4550	2.5016	2.4708	2.5039	2.4741	2.4967	2.4443	**3.38**
**H(1\`B)**	2.7192	2.7822	2.8168	2.8250	2.8747	2.8223	2.8737	2.8195	2.8658	2.8276	2.8825	2.3793	2.2880	2.2538	2.2872	2.3250	2.2852	2.2832	2.2834	2.2964	2.2895	2.2833	**3.71**
**H(2\`A)**	1.4596	1.5065	1.4695	1.5227	1.4643	1.5218	1.4902	1.5207	1.4875	1.5236	1.5015	1.3531	1.4150	1.3758	1.4387	1.3948	1.4370	1.4298	1.4353	1.4285	1.4403	1.4450	**2.08**
**H(2\`B)**	1.5116	1.5543	1.5178	1.6170	1.5855	1.6122	1.5872	1.6073	1.5811	1.6217	1.6111	1.4135	1.5446	1.4828	1.6324	1.6198	1.6256	1.6502	1.6189	1.6431	1.6390	1.6949	**2.24**
**H(3\`A)**	1.3531	1.4150	1.3758	1.4387	1.3948	1.4370	1.4298	1.4352	1.4285	1.4403	1.4450	1.4596	1.5065	1.4695	1.5227	1.4643	1.5218	1.4902	1.5207	1.4875	1.5236	1.5015	**2.08**
**H(3\`B)**	1.4135	1.5446	1.4828	1.6324	1.6198	1.6256	1.6502	1.6189	1.6431	1.6390	1.6949	1.5116	1.5543	1.5178	1.6170	1.5855	1.6122	1.5872	1.6073	1.5811	1.6217	1.6111	**2.16**
**H(4\`A)**	2.3793	2.2880	2.2538	2.2872	2.3250	2.2852	2.2832	2.2834	2.2964	2.2895	2.2833	2.7192	2.7822	2.8168	2.8250	2.8747	2.8223	2.8737	2.8195	2.8658	2.8276	2.8825	**3.09**
**H(4\`B)**	2.5341	2.5327	2.5042	2.4991	2.4550	2.5016	2.4708	2.5039	2.4741	2.4967	2.4443	2.3728	2.3640	2.3490	2.3541	2.3106	2.3546	2.3370	2.3550	2.3368	2.3537	2.3150	**3.68**
Nuclei	LC-BLYP																						
**C1**	144.3028	145.1950	146.1042	145.8010	147.4677	145.7589	146.7872	145.7404	146.8037	145.8203	147.0726	144.3028	145.1949	146.1041	145.8010	147.4677	145.7589	146.7872	145.7405	146.8037	145.8203	147.0725	**132.2**
**C2**	148.6053	148.4336	148.9601	148.2830	148.7557	148.2707	149.2718	148.2857	149.1701	148.2777	149.2677	148.6053	148.4336	148.9602	148.2830	148.7557	148.2707	149.2718	148.2859	149.1701	148.2778	149.2676	**133.3**
**C3**	128.2541	128.9264	129.5380	129.1390	129.8089	129.1086	129.9774	129.1049	129.9340	129.1500	130.0396	128.2541	128.9264	129.5380	129.1390	129.8089	129.1086	129.9774	129.1048	129.9339	129.1500	130.0397	**117.6**
**C4**	181.9735	182.5089	183.1029	182.8610	183.3907	182.8163	184.0004	182.7995	183.8613	182.8865	184.1304	181.9735	182.5091	183.1027	182.8610	183.3907	182.8164	184.0004	182.7995	183.8613	182.8865	184.1304	**168.3**
**C5**	126.1791	127.4745	128.1385	128.1224	129.0703	128.1579	129.2286	128.1194	129.1271	128.2680	129.3934	126.1791	127.4745	128.1384	128.2234	129.0703	128.1579	129.2287	128.1195	129.1270	128.2679	129.3934	**117.6**
**C6**	144.2567	146.1931	147.4116	147.2757	148.9440	147.1795	149.5452	147.1123	149.2785	147.3512	149.9505	144.2567	146.1930	147.4116	147.2757	148.9440	147.1795	149.5451	147.1124	149.2785	147.3512	149.9505	**133.3**
**C7**	211.8303	217.0927	218.4451	220.2118	220.7174	220.0151	226.8416	219.8419	225.4392	220.3777	228.0439	211.8303	217.0927	218.4455	220.2118	220.7174	220.0150	226.8416	219.8416	225.4392	220.3777	228.0436	**195.8**
**C8**	62.4946	63.6122	64.3010	64.2400	65.1246	64.1886	65.9586	64.1624	65.7354	64.2696	66.4491	62.4946	63.6122	64.3010	64.2400	65.1246	64.1886	65.9586	64.1624	65.7354	64.2696	66.4492	**70.4**
**C9**	33.0906	32.7128	32.9583	32.5697	32.8735	32.5573	33.0162	32.5722	32.9124	32.5640	33.1062	33.0906	32.7128	32.9583	32.5697	32.8734	32.5573	33.0162	32.5722	32.9124	32.5640	33.1062	**31.1**
**C10**	26.7174	27.0997	27.5416	27.4779	28.1510	27.4294	27.9545	27.4087	27.9216	27.5068	28.1708	26.7174	27.0997	27.5417	27.4779	28.1510	27.4295	27.9545	27.4088	27.9216	27.5068	28.1708	**26.9**
**C11**	25.6629	25.6738	26.0213	25.6762	26.0987	25.6556	26.0916	25.6622	26.0813	25.6781	26.2660	25.6628	25.6738	26.0212	25.6762	26.0987	25.6557	26.0917	25.6622	26.0813	25.6781	26.2660	**23.4**
**C12**	15.2825	15.3973	15.4777	15.5469	15.6332	15.5145	15.5678	15.5098	15.5562	15.5604	15.5999	15.2825	15.3974	15.4776	15.5468	15.6333	15.5145	15.5678	15.5098	15.5562	15.5604	15.5999	**13.8**
**C1\`**	48.8633	49.5850	49.8719	50.1266	50.6951	50.0699	50.6789	50.0406	50.6292	50.1636	51.1331	52.7573	53.6750	54.0588	54.1648	54.6804	54.1139	54.7997	54.0904	54.7504	54.1956	55.0687	**53.3**
**C2\`**	23.0097	23.2733	23.5886	23.4460	23.8619	23.4152	23.9107	23.4114	23.8912	23.4575	23.8212	22.3648	22.4849	22.8031	22.6272	23.0962	22.5957	22.9834	22.5919	22.9779	22.6398	22.9003	**24.2**
**C3\`**	22.3648	22.4849	22.8032	22.6272	23.0962	22.5957	22.9834	22.5918	22.9779	22.6398	22.9003	23.0098	23.2733	23.5887	23.4460	23.8619	23.4152	23.9107	23.4114	23.8911	23.4575	23.8213	**24.1**
**C4\`**	52.7572	53.6750	54.0587	54.1648	54.6804	54.1139	54.7997	54.0903	54.7504	54.1956	55.0688	48.8633	49.5850	49.8719	50.1267	50.6951	50.0699	50.6789	50.0406	50.6292	50.1636	51.1331	**56.3**
**H2**	9.0056	8.8849	8.9030	8.7965	8.7977	8.7995	8.7926	8.8043	8.8043	8.7932	8.7781	9.0056	8.8849	8.9030	8.7965	8.7977	8.7994	8.7926	8.8043	8.8043	8.7932	8.7781	**8.2**
**H3**	7.5396	7.6763	7.7674	7.7172	7.8365	7.7144	7.8519	7.7131	7.8455	7.7189	7.8671	7.5396	7.6763	7.7674	7.7172	7.8365	7.7144	7.8519	7.7131	7.8455	7.7189	7.8671	**7.36**
**H5**	7.3877	7.5702	7.6618	7.6532	7.7802	7.6475	7.7925	7.6434	7.7820	7.6575	7.8161	7.3877	7.5702	7.6618	7.6532	7.7802	7.6475	7.7926	7.6434	7.7820	7.6575	7.8161	**7.36**
**H6**	8.1905	8.3935	8.4921	8.4932	8.6406	8.4854	8.6743	8.4792	8.6601	8.4999	8.7076	8.1905	8.3935	8.4921	8.4932	8.6406	8.4854	8.6743	8.4792	8.6601	8.4999	8.7076	**8.2**
**H8**	4.2049	4.2951	4.3484	4.3672	4.4527	4.3612	4.4800	4.3569	4.4681	4.3721	4.4886	4.2049	4.2951	4.3484	4.3672	4.4527	4.3612	4.4800	4.3569	4.4681	4.3722	4.4886	**5.4**
**H(9A-9B)**	1.6602	1.6944	1.6916	1.7190	1.7312	1.7160	1.7354	1.7147	1.7257	1.7209	1.7694	1.6602	1.6944	1.6916	1.7190	1.7312	1.7160	1.7354	1.7147	1.7257	1.7209	1.7694	**2.08**
**H(10A)**	0.9464	0.7709	0.7337	0.7059	0.6281	0.7076	0.6393	0.7111	0.6461	0.7035	0.6520	0.9464	0.7709	0.7337	0.7059	0.6281	0.7076	0.6393	0.7111	0.6461	0.7035	0.6520	**1.14**
**H(10B)**	1.5956	1.4998	1.4331	1.4116	1.3290	1.4162	1.3204	1.4225	1.3282	1.4064	1.2946	1.5956	1.4998	1.4331	1.4116	1.3290	1.4162	1.3204	1.4225	1.3282	1.4064	1.2946	**1.28**
**H(11A-11B)**	0.9922	1.0091	0.9759	1.0198	0.9828	1.0178	0.9739	1.0175	0.9768	1.0208	0.9667	0.9922	1.0091	0.9759	1.0198	0.9828	1.0178	0.9739	1.0175	0.9768	1.0208	0.9667	**1.28**
**H(12A-12C)**	0.9529	0.9320	0.9064	0.9207	0.8812	0.9204	0.8804	0.9217	0.8832	0.9200	0.8772	0.9529	0.9320	0.9064	0.9207	0.8812	0.9204	0.8803	0.9216	0.8832	0.9200	0.8772	**0.82**
**H(1\`A)**	2.6756	2.6656	2.6476	2.6566	2.6054	2.6565	2.6213	2.6579	2.6203	2.6558	2.6008	2.8095	2.7976	2.7819	2.7777	2.7409	2.7787	2.7655	2.7812	2.7634	2.7757	2.7396	**3.38**
**H(1\`B)**	3.0184	3.0586	3.0951	3.0995	3.1550	3.0957	3.1431	3.0935	3.1381	3.1022	3.1499	2.7332	2.5800	2.5558	2.5523	2.5380	2.5532	2.5182	2.5557	2.5071	2.5505	2.5099	**3.71**
**H(2\`A)**	1.6383	1.6830	1.6519	1.7025	1.6442	1.7006	1.6698	1.7003	1.6669	1.7033	1.6810	1.5189	1.5743	1.5442	1.5997	1.5465	1.5976	1.5824	1.5971	1.5790	1.6007	1.6004	**2.08**
**H(2\`B)**	1.7521	1.7824	1.7443	1.8315	1.7969	1.8269	1.7886	1.8240	1.7826	1.8350	1.8143	1.6609	1.7682	1.7236	1.8425	1.8099	1.8368	1.8308	1.8328	1.8249	1.8469	1.8762	**2.24**
**H(3\`A)**	1.5189	1.5743	1.5442	1.5997	1.5465	1.5976	1.5824	1.5970	1.5790	1.6007	1.6004	1.6383	1.6830	1.6519	1.7025	1.6442	1.7006	1.6698	1.7003	1.6669	1.7033	1.6810	**2.08**
**H(3\`B)**	1.6609	1.7682	1.7236	1.8425	1.8099	1.8368	1.8308	1.8328	1.8249	1.8469	1.8762	1.7521	1.7824	1.7443	1.8315	1.7969	1.8269	1.7886	1.8240	1.7826	1.8350	1.8143	**2.16**
**H(4\`A)**	2.7332	2.5800	2.5558	2.5523	2.5380	2.5532	2.5182	2.5557	2.5071	2.5505	2.5099	3.0184	3.0586	3.0951	3.0995	3.1550	3.0957	3.1431	3.0935	3.1381	3.1022	3.1499	**3.09**
**H(4\`B)**	2.8095	2.7976	2.7819	2.7777	2.7409	2.7787	2.7655	2.7812	2.7634	2.7757	2.7396	2.6756	2.6656	2.6476	2.6566	2.6054	2.6565	2.6213	2.6579	2.6203	2.6558	2.6008	**3.68**

[a] methyl protons shifts are averaged

[b] according to [5]

Table S5. Cont. VII

Nuclei	R											S											Exp. [b]
	M06																						
	Gas	$CHCl_3$ (PCM)	$CHCl_3$ (SMD)	DMSO (PCM)	DMSO (SMD)	CH_3OH (PCM)	CH_3OH (SMD)	C_2H_5OH (PCM)	C_2H_5OH (SMD)	H_2O (PCM)	H_2O (SMD)	Gas	$CHCl_3$ (PCM)	$CHCl_3$ (SMD)	DMSO (PCM)	DMSO (SMD)	CH_3OH (PCM)	CH_3OH (SMD)	C_2H_5OH (PCM)	C_2H_5OH (SMD)	H_2O (PCM)	H_2O (SMD)	
**C1**	136.6837	138.1845	138.7887	138.8803	142.1616	138.8390	141.0201	138.8028	141.1968	138.9324	141.3525	136.6837	138.1845	138.7887	138.8803	142.1616	138.8390	141.0201	138.8027	141.1968	138.9324	141.3525	**132.2**
**C2**	139.8288	139.8302	140.5330	139.7616	140.5668	139.7588	141.0927	139.7635	140.9947	139.7793	141.0467	139.8288	139.8302	140.5330	139.7616	140.5668	139.7588	141.0927	139.7635	140.9947	139.7793	141.0466	**133.3**
**C3**	121.7274	122.3706	123.0781	122.5656	123.3591	122.5459	123.5764	122.5339	123.5302	122.5989	123.6983	121.7273	122.3706	123.0781	122.5656	123.3592	122.5459	123.5764	122.5338	123.5302	122.5989	123.6984	**117.6**
**C4**	173.6534	174.3654	175.1102	174.6855	175.0219	174.6538	175.6222	174.6298	175.4678	174.7300	175.7595	173.6534	174.3654	175.1102	174.6855	175.0220	174.6538	175.6222	174.6297	175.4678	174.7300	175.7595	**168.3**
**C5**	119.5548	120.7943	121.4738	121.5477	122.5989	121.4922	122.8231	121.4445	122.7327	121.6146	122.9843	119.5548	120.7943	121.4739	121.5477	122.5989	121.4922	122.8231	121.4445	122.7327	121.6146	122.9844	**117.6**
**C6**	136.4800	138.1760	139.3711	139.1977	140.2318	139.1198	140.8785	139.0504	140.6173	139.2868	141.1362	136.4800	138.1760	139.3711	139.1977	140.2318	139.1198	140.8785	139.0504	140.6173	139.2868	141.1362	**133.3**
**C7**	203.9031	208.9344	210.1431	211.5776	213.5179	211.4065	218.3465	211.2396	217.2496	211.7527	219.5336	203.9031	208.9344	210.1431	211.5776	213.5178	211.4065	218.3465	211.2398	217.2496	211.7527	219.5336	**195.8**
**C8**	62.4081	63.5176	64.2402	64.1969	65.5191	64.1481	66.0334	64.1062	65.8734	64.2578	66.5123	62.4081	63.5176	64.2401	64.1969	65.5191	64.1481	66.0334	64.1062	65.8734	64.2578	66.5124	**70.4**
**C9**	33.8614	33.5608	34.1825	33.4541	33.5991	33.4442	33.8295	33.4403	33.7236	33.4773	33.9495	33.8614	33.5608	34.1825	33.4541	33.5991	33.4442	33.8295	33.4403	33.7236	33.4773	33.9496	**31.1**
**C10**	25.9231	26.6415	27.3493	27.1312	28.1401	27.0798	27.8705	27.0349	27.8296	27.1942	28.2499	25.9232	26.6415	27.3493	27.1312	28.1402	27.0798	27.8705	27.0350	27.8296	27.1942	28.2499	**26.9**
**C11**	26.4897	26.5713	27.0992	26.6108	27.1999	26.5919	27.1386	26.5795	27.1135	26.6426	27.3645	26.4897	26.5713	27.0993	26.6108	27.1998	26.5919	27.1386	26.5795	27.1135	26.6426	27.3645	**23.4**
**C12**	15.9103	16.1276	16.3119	16.3078	16.6622	16.2818	16.5809	16.2642	16.5652	16.3475	16.6634	15.9104	16.1276	16.3119	16.3078	16.6622	16.2818	16.5809	16.2642	16.5652	16.3475	16.6635	**13.8**
**C1`**	50.7988	51.7722	52.1796	52.3154	52.9675	52.2717	52.8620	52.2364	52.8195	52.3722	53.3347	55.0385	56.1158	56.5140	56.6961	57.3840	56.6486	57.5406	56.6088	57.4838	56.7556	57.9703	**53.3**
**C2`**	23.3610	23.8539	24.2807	24.1064	24.6826	24.0798	24.6277	24.0608	24.6002	24.1462	24.5473	22.1499	22.4551	22.8638	22.6320	23.3567	22.6134	23.1653	22.6032	23.1850	22.6645	22.8605	**24.2**
**C3`**	22.1499	22.4551	22.8637	22.6320	23.3567	22.6134	23.1653	22.6032	23.1850	22.6645	22.8605	23.3610	23.8539	24.2807	24.1064	24.6825	24.0798	24.6277	24.0607	24.6002	24.1462	24.5472	**24.1**
**C4`**	55.0385	56.1158	56.5140	56.6961	57.3841	56.6486	57.5406	56.6087	57.4838	56.7556	57.9703	50.7988	51.7722	52.1796	52.3154	52.9675	52.2717	52.8620	52.2365	52.8195	52.3722	53.3348	**56.3**
**H2**	8.7200	8.5356	8.6199	8.4194	8.3302	8.4246	8.3913	8.4306	8.3825	8.4151	8.3799	8.7200	8.5356	8.6199	8.4194	8.3302	8.4246	8.3913	8.4306	8.3825	8.4151	8.3799	**8.2**
**H3**	7.3688	7.5079	7.6104	7.5243	7.6008	7.5236	7.6221	7.5236	7.6136	7.5255	7.6318	7.3688	7.5079	7.6104	7.5243	7.6008	7.5236	7.6221	7.5236	7.6136	7.5255	7.6319	**7.36**
**H5**	7.0547	7.2196	7.3131	7.3125	7.4915	7.3067	7.5114	7.3012	7.5006	7.3185	7.5350	7.0547	7.2196	7.3131	7.3125	7.4915	7.3067	7.5114	7.3012	7.5006	7.3185	7.5350	**7.36**
**H6**	8.0770	8.2186	8.3077	8.3010	8.3629	8.2950	8.4115	8.2895	8.3902	8.3074	8.4458	8.0770	8.2186	8.3077	8.3010	8.3629	8.2950	8.4115	8.2895	8.3902	8.3074	8.4458	**8.2**
**H8**	4.4275	4.4268	4.4630	4.4703	4.5762	4.4663	4.6245	4.4627	4.6087	4.4746	4.6669	4.4275	4.4268	4.4630	4.4703	4.5762	4.4663	4.6245	4.4627	4.6087	4.4746	4.6669	**5.4**
**H(9A-9B)**	1.8331	1.7715	1.8082	1.7461	1.6728	1.7461	1.6811	1.7470	1.6718	1.7469	1.7105	1.8331	1.7715	1.8082	1.7461	1.6728	1.7461	1.6811	1.7470	1.6718	1.7469	1.7105	**2.08**
**H(10A)**	0.7728	0.6586	0.6596	0.6274	0.6472	0.6280	0.6691	0.6289	0.6703	0.6272	0.7166	0.7728	0.6586	0.6596	0.6274	0.6472	0.6280	0.6691	0.6289	0.6678	0.6272	0.7166	**1.14**
**H(10B)**	1.7663	1.6085	1.5217	1.4729	1.3898	1.4826	1.4047	1.4937	1.4059	1.4648	1.3494	1.7663	1.6085	1.5217	1.4729	1.3897	1.4826	1.4047	1.4937	1.4183	1.4648	1.3494	**1.28**
**H(11A-11B)**	1.0514	1.0480	1.0176	1.0528	1.0214	1.0520	1.0203	1.0520	1.0195	1.0542	1.0055	1.0514	1.0480	1.0176	1.0528	1.0214	1.0520	1.0203	1.0519	1.0195	1.0542	1.0055	**1.28**
**H(12A-12C)**	0.9078	0.8833	0.8498	0.8652	0.8460	0.8666	0.8533	0.8688	0.8571	0.8646	0.8353	0.9078	0.8833	0.8498	0.8652	0.8460	0.8666	0.8533	0.8688	0.8571	0.8646	0.8354	**0.82**
**H(1`A)**	2.6899	2.6721	2.6596	2.6399	2.6025	2.6424	2.6121	2.6455	2.6133	2.6382	2.5768	2.8894	2.8866	2.8724	2.8603	2.8238	2.8625	2.8387	2.8652	2.8412	2.8590	2.8125	**3.38**
**H(1`B)**	3.1176	3.1382	3.1900	3.1575	3.2127	3.1551	3.2023	3.1532	3.2035	3.1604	3.1870	2.7389	2.6337	2.6366	2.6095	2.6250	2.6103	2.6180	2.6118	2.6158	2.6093	2.6306	**3.71**
**H(2`A)**	1.5606	1.6136	1.5883	1.6319	1.5867	1.6306	1.6047	1.6300	1.6059	1.6337	1.6278	1.4496	1.4934	1.4610	1.5084	1.4777	1.5076	1.5096	1.5075	1.5081	1.5098	1.5275	**2.08**
**H(2`B)**	1.7496	1.7708	1.7382	1.8129	1.8060	1.8093	1.8058	1.8063	1.8070	1.8169	1.8371	1.7891	1.8317	1.7800	1.8708	1.8518	1.8678	1.8668	1.8656	1.8648	1.8744	1.9311	**2.24**
**H(3`A)**	1.4496	1.4934	1.4610	1.5084	1.4777	1.5076	1.5096	1.5075	1.5108	1.5098	1.5275	1.5606	1.6136	1.5883	1.6319	1.5867	1.6306	1.6047	1.6300	1.6007	1.6337	1.6278	**2.08**
**H(3`B)**	1.7891	1.8317	1.7799	1.8708	1.8518	1.8678	1.8668	1.8656	1.8680	1.8744	1.9311	1.7496	1.7708	1.7382	1.8129	1.8059	1.8093	1.8058	1.8063	1.7990	1.8169	1.8371	**2.16**
**H(4`A)**	2.7389	2.6337	2.6366	2.6095	2.6225	2.6103	2.6180	2.6118	2.6192	2.6093	2.6306	3.1176	3.1382	3.1900	3.1575	3.2127	3.1551	3.2023	3.1532	3.1996	3.1604	3.1870	**3.09**
**H(4`B)**	2.8894	2.8866	2.8724	2.8603	2.8238	2.8625	2.8387	2.8652	2.8399	2.8590	2.8125	2.6899	2.6721	2.6596	2.6399	2.6025	2.6424	2.6121	2.6455	2.6150	2.6382	2.5768	**3.68**
Nuclei	M06-2X																						
**C1**	153.3139	154.1147	155.0923	154.6258	156.4278	154.6074	156.0736	154.5917	155.8323	154.6374	156.7424	153.3140	154.1147	155.0922	154.6258	156.4279	154.6072	156.0736	154.5917	155.8323	154.6374	156.7424	**132.2**
**C2**	154.9208	154.8472	155.4019	154.6764	155.2728	154.7107	155.7196	154.7454	155.6269	154.6371	155.7829	154.9207	154.8472	155.4018	154.6764	155.2727	154.7105	155.7196	154.7454	155.6269	154.6371	155.7829	**133.3**
**C3**	136.1763	136.8491	137.4978	137.0428	137.8093	137.0546	138.0584	137.0674	138.0027	137.0251	138.1494	136.1763	136.8491	137.4977	137.0428	137.8093	137.0547	138.0584	137.0674	138.0027	137.0251	138.1494	**117.6**
**C4**	187.1500	187.9566	188.6296	188.3781	189.0881	188.3727	189.7626	188.3687	189.6217	188.3768	189.9010	187.1498	187.9566	188.6296	188.3781	189.0881	188.3728	189.7626	188.3687	189.6217	188.3768	189.9010	**168.3**
**C5**	133.6665	135.1820	135.9032	135.9787	136.9449	135.9514	137.1623	135.9252	137.0543	135.9981	137.3183	133.6665	135.1820	135.9032	135.9787	136.9449	135.9515	137.1623	135.9252	137.0543	135.9981	137.3183	**117.6**
**C6**	151.1929	153.3671	154.5822	154.5301	156.3787	154.4823	156.8323	154.4355	156.6334	154.5694	157.1596	151.1930	153.3671	154.5822	154.5301	156.3788	154.4824	156.8323	154.4355	156.6334	154.5694	157.1596	**133.3**
**C7**	219.5245	224.8907	226.3983	228.0101	228.7404	227.8294	235.4867	227.6520	233.7316	228.1759	237.1101	219.5241	224.8907	226.3983	228.0101	228.7407	227.8297	235.4867	227.6520	233.7316	228.1759	237.1101	**195.8**
**C8**	67.2610	68.6483	69.3496	69.3548	70.4448	69.3293	71.3018	69.3060	71.0041	69.3732	72.0281	67.2610	68.6483	69.3496	69.3548	70.4448	69.3292	71.3018	69.3060	71.0041	69.3732	72.0281	**70.4**
**C9**	35.4642	35.4239	35.5992	35.3385	35.6219	35.3672	35.7953	35.3969	35.7017	35.3048	36.0192	35.4642	35.4239	35.5992	35.3385	35.6220	35.3672	35.7953	35.3969	35.7017	35.3048	36.0192	**31.1**
**C10**	28.1749	28.5554	28.9127	28.9302	29.6142	28.9248	29.4107	28.9207	29.3703	28.9290	29.6021	28.1749	28.5554	28.9126	28.9302	29.6141	28.9248	29.4107	28.9207	29.3703	28.9290	29.6021	**26.9**
**C11**	27.0694	27.0411	27.3428	27.0466	27.4659	27.0675	27.4579	27.0895	27.4476	27.0203	27.6157	27.0693	27.0411	27.3428	27.0466	27.4660	27.0676	27.4579	27.0895	27.4476	27.0203	27.6156	**23.4**
**C12**	15.6785	15.8894	15.9172	16.0069	16.0766	16.0199	16.0059	16.0341	16.0007	15.9879	15.9410	15.6786	15.8894	15.9172	16.0069	16.0767	16.0199	16.0059	16.0341	16.0007	15.9879	15.9410	**13.8**
**C1`**	52.7471	53.4371	53.7246	53.9470	54.4968	53.9305	54.5272	53.9162	54.4023	53.9572	54.9826	57.2784	58.0992	58.4834	58.4784	59.0562	58.4723	59.1518	58.4681	59.1113	58.4785	59.4310	**53.3**
**C2`**	24.6913	25.0617	25.3331	25.2432	25.6602	25.2509	25.6528	25.2603	25.6474	25.2298	25.6465	23.7535	24.0580	24.3338	24.2747	24.7880	24.2798	24.7486	24.2866	24.7300	24.2642	24.8143	**24.2**
**C3`**	23.7535	24.0580	24.3338	24.2747	24.7881	24.2797	24.7486	24.2866	24.7300	24.2642	24.8143	24.6914	25.0617	25.3332	25.2432	25.6603	25.2509	25.6528	25.2603	25.6474	25.2298	25.6465	**24.1**
**C4`**	57.2790	58.0992	58.4835	58.4784	59.0562	58.4723	59.1518	58.4681	59.1113	58.4785	59.4310	52.7471	53.4371	53.7245	53.9470	54.4969	53.9305	54.5272	53.9162	54.4023	53.9572	54.9826	**56.3**
**H2**	9.2553	9.1886	9.2043	9.1236	9.1185	9.1277	9.1058	9.1311	9.1346	9.1198	9.0783	9.2553	9.1886	9.2043	9.1236	9.1185	9.1277	9.1058	9.1311	9.1346	9.1198	9.0783	**8.2**
**H3**	7.8496	7.9991	8.0968	8.0550	8.1780	8.0508	8.2036	8.0462	8.1958	8.0590	8.2179	7.8496	7.9991	8.0968	8.0550	8.1780	8.0508	8.2036	8.0462	8.1958	8.0590	8.2179	**7.36**
**H5**	7.6700	7.8593	7.9559	7.9464	8.0779	7.9403	8.0866	7.9337	8.0791	7.9524	8.1072	7.6700	7.8593	7.9559	7.9464	8.0779	7.9403	8.0866	7.9337	8.0791	7.9524	8.1072	**7.36**
**H6**	8.5783	8.7576	8.8602	8.8626	9.0133	8.8546	9.0581	8.8464	9.0490	8.8703	9.0681	8.5783	8.7576	8.8602	8.8626	9.0133	8.8546	9.0581	8.8464	9.0490	8.8703	9.0681	**8.2**
**H8**	4.2922	4.3772	4.4355	4.4447	4.5306	4.4393	4.5577	4.4336	4.5580	4.4499	4.5479	4.2922	4.3772	4.4355	4.4447	4.5306	4.4393	4.5577	4.4336	4.5580	4.4499	4.5479	**5.4**
**H(9A-9B)**	1.6952	1.7512	1.7338	1.7870	1.7864	1.7839	1.7723	1.7805	1.7723	1.7901	1.8164	1.6952	1.7512	1.7338	1.7870	1.7864	1.7839	1.7723	1.7805	1.7723	1.7901	1.8164	**2.08**
**H(10A)**	0.8886	0.7034	0.6742	0.6310	0.5530	0.6339	0.5704	0.6364	0.5766	0.6284	0.6028	0.8886	0.7034	0.6742	0.6310	0.5530	0.6339	0.5704	0.6364	0.5766	0.6284	0.6028	**1.14**
**H(10B)**	1.5834	1.5136	1.4450	1.4129	1.3259	1.4186	1.3112	1.4240	1.3267	1.4077	1.2702	1.5834	1.5136	1.4449	1.4129	1.3259	1.4186	1.3112	1.4240	1.3267	1.4077	1.2702	**1.28**
**H(11A-11B)**	0.9471	0.9670	0.9367	0.9816	0.9426	0.9795	0.9338	0.9771	0.9397	0.9837	0.9138	0.9471	0.9670	0.9367	0.9816	0.9426	0.9795	0.9338	0.9771	0.9397	0.9837	0.9138	**1.28**
**H(12A-12C)**	0.8951	0.8808	0.8572	0.8688	0.8253	0.8683	0.8226	0.8674	0.8290	0.8694	0.8070	0.8951	0.8808	0.8572	0.8688	0.8253	0.8683	0.8226	0.8674	0.8290	0.8694	0.8070	**0.82**
**H(1`A)**	2.6201	2.6148	2.6019	2.6064	2.5567	2.6061	2.5649	2.6055	2.5694	2.6068	2.5425	2.8027	2.8010	2.7892	2.7839	2.7542	2.7840	2.7717	2.7838	2.7696	2.7840	2.7411	**3.38**
**H(1`B)**	3.0833	3.1152	3.1403	3.1536	3.1988	3.1498	3.1827	3.1456	3.1831	3.1574	3.1910	2.7295	2.6123	2.5775	2.5866	2.5699	2.5867	2.5575	2.5864	2.5371	2.5867	2.5782	**3.71**
**H(2`A)**	1.6290	1.6809	1.6547	1.7095	1.6574	1.7067	1.6794	1.7035	1.6774	1.7123	1.6820	1.5087	1.5740	1.5518	1.6067	1.5563	1.6036	1.5922	1.6001	1.5906	1.6099	1.6099	**2.08**
**H(2`B)**	1.7699	1.7936	1.7566	1.8343	1.7953	1.8301	1.7868	1.8258	1.7846	1.8383	1.8070	1.7124	1.7982	1.7646	1.8623	1.8353	1.8562	1.8634	1.8499	1.8538	1.8684	1.8987	**2.24**
**H(3`A)**	1.5087	1.5740	1.5519	1.6067	1.5563	1.6035	1.5922	1.6001	1.5906	1.6099	1.6099	1.6290	1.6809	1.6547	1.7095	1.6573	1.7067	1.6794	1.7035	1.6774	1.7123	1.6820	**2.08**
**H(3`B)**	1.7124	1.7982	1.7646	1.8623	1.8354	1.8562	1.8634	1.8499	1.8538	1.8684	1.8987	1.7699	1.7936	1.7567	1.8343	1.7954	1.8301	1.7868	1.8258	1.7846	1.8383	1.8070	**2.16**
**H(4`A)**	2.7295	2.6123	2.5775	2.5866	2.5699	2.5867	2.5575	2.5864	2.5371	2.5867	2.5782	3.0833	3.1152	3.1402	3.1536	3.1988	3.1498	3.1827	3.1456	3.1831	3.1574	3.1910	**3.09**
**H(4`B)**	2.8027	2.8010	2.7892	2.7839	2.7542	2.7840	2.7717	2.7838	2.7696	2.7840	2.7411	2.6201	2.6148	2.6019	2.6064	2.5567	2.6061	2.5649	2.6055	2.5694	2.6068	2.5425	**3.68**

[a] methyl protons shifts are averaged

[b] according to [5]

Table S5. Cont. VIII

Nuclei	R											S											Exp. [b]
	mpW1LYP																						
	Gas	$CHCl_3$ (PCM)	$CHCl_3$ (SMD)	DMSO (PCM)	DMSO (SMD)	CH_3OH (PCM)	CH_3OH (SMD)	C_2H_5OH (PCM)	C_2H_5OH (SMD)	H_2O (PCM)	H_2O (SMD)	Gas	$CHCl_3$ (PCM)	$CHCl_3$ (SMD)	DMSO (PCM)	DMSO (SMD)	CH_3OH (PCM)	CH_3OH (SMD)	C_2H_5OH (PCM)	C_2H_5OH (SMD)	H_2O (PCM)	H_2O (SMD)	
**C1**	139.8378	140.8440	141.4180	141.3625	142.7491	141.3294	142.1529	141.2986	142.1820	141.3923	142.2792	139.8378	140.8440	141.4181	141.3622	142.7491	141.3294	142.1530	141.2986	142.1819	141.3923	142.2792	**132.2**
**C2**	139.8714	139.7984	140.3079	139.7197	140.1190	139.7218	140.6847	139.7258	140.5678	139.7161	140.5968	139.8714	139.7984	140.3079	139.7196	140.1190	139.7218	140.6848	139.7258	140.5678	139.7161	140.5970	**133.3**
**C3**	122.0463	122.6720	123.2099	122.8903	123.4471	122.8775	123.7073	122.8655	123.6488	122.9006	123.7499	122.0463	122.6720	123.2099	122.8905	123.4470	122.8775	123.7074	122.8655	123.6487	122.9006	123.7498	**117.6**
**C4**	176.3397	177.1720	177.7901	177.5913	178.1904	177.5635	178.7928	177.5372	178.6520	177.6158	178.9042	176.3397	177.1719	177.7901	177.5914	178.1902	177.5635	178.7927	177.5372	178.6517	177.6158	178.9042	**168.3**
**C5**	119.8982	121.0713	121.6786	121.7085	122.4580	121.6663	122.6459	121.6259	122.5595	121.7466	122.7780	119.8982	121.0713	121.6786	121.7085	122.4581	121.6663	122.6458	121.6259	122.5594	121.7465	122.7779	**117.6**
**C6**	136.0966	138.0128	139.1739	139.1635	140.6582	139.0867	141.3023	139.0117	141.0638	139.2346	141.6841	136.0966	138.0128	139.1739	139.1635	140.6585	139.0867	141.3022	139.0117	141.0638	139.2346	141.6841	**133.3**
**C7**	203.6129	208.5205	209.5677	211.1801	211.7568	211.0176	216.9290	210.8561	215.7371	211.3332	217.9149	203.6129	208.5205	209.5677	211.1801	211.7568	211.0176	216.9290	210.8561	215.7372	211.3332	217.9147	**195.8**
**C8**	65.8463	67.0437	67.6010	67.5551	68.5804	67.5298	69.1701	67.5042	69.0076	67.5753	69.4725	65.8463	67.0437	67.6010	67.5551	68.5805	67.5298	69.1701	67.5042	69.0077	67.5753	69.4725	**70.4**
**C9**	37.0169	36.6560	36.8848	36.5180	36.8153	36.5233	37.0170	36.5296	36.9632	36.5106	37.0610	37.0169	36.6560	36.8848	36.5180	36.8153	36.5233	37.0170	36.5296	36.9632	36.5106	37.0610	**31.1**
**C10**	29.3292	29.7978	30.3192	30.1290	30.8587	30.1027	30.6588	30.0784	30.6559	30.1521	30.7746	29.3292	29.7978	30.3193	30.1290	30.8587	30.1027	30.6588	30.0784	30.6559	30.1521	30.7746	**26.9**
**C11**	29.4068	29.5120	29.8154	29.4911	29.9151	29.4934	29.9122	29.4963	29.9182	29.4864	30.0182	29.4067	29.5120	29.8153	29.4911	29.9151	29.4934	29.9121	29.4963	29.9182	29.4865	30.0180	**23.4**
**C12**	16.7769	16.9386	17.0341	17.0893	17.1643	17.0748	17.1257	17.0621	17.1298	17.1017	17.1017	16.7769	16.9387	17.0340	17.0893	17.1642	17.0748	17.1257	17.0621	17.1298	17.1017	17.1017	**13.8**
**C1`**	51.9766	52.7794	53.1018	53.3629	53.8586	53.3224	53.8640	53.2840	53.8039	53.4001	54.3551	56.5078	57.6071	57.9635	58.0839	58.5838	58.0550	58.7006	58.0272	58.6452	58.1090	58.9220	**53.3**
**C2`**	25.9559	26.4339	26.8102	26.6257	27.1139	26.6133	27.1490	26.6020	27.1457	26.6349	26.9197	25.5722	25.7544	26.1426	25.9442	26.3949	25.9276	26.3477	25.9127	26.3492	25.9585	26.1388	**24.2**
**C3`**	25.5722	25.7545	26.1426	25.9444	26.3949	25.9276	26.3478	25.9127	26.3493	25.9585	26.1388	25.9559	26.4339	26.8101	26.6257	27.1139	26.6133	27.1490	26.6020	27.1458	26.6349	26.9196	**24.1**
**C4`**	56.5078	57.6072	57.9635	58.0838	58.5838	58.0550	58.7006	58.0272	58.6452	58.1090	58.9219	51.9767	52.7795	53.1019	53.3630	53.8586	53.3223	53.8640	53.2840	53.8039	53.4001	54.3551	**56.3**
**H2**	8.7240	8.6003	8.6562	8.5315	8.5403	8.5359	8.5588	8.5399	8.5634	8.5273	8.5464	8.7240	8.6003	8.6562	8.5315	8.5403	8.5359	8.5588	8.5399	8.5634	8.5273	8.5464	**8.2**
**H3**	7.3356	7.4800	7.5666	7.5264	7.6381	7.5244	7.6591	7.5221	7.6515	7.5280	7.6715	7.3356	7.4800	7.5666	7.5264	7.6381	7.5244	7.6591	7.5221	7.6515	7.5280	7.6715	**7.36**
**H5**	7.1538	7.3065	7.4076	7.3765	7.5081	7.3726	7.5127	7.3684	7.5049	7.3800	7.5376	7.1538	7.3065	7.4076	7.3765	7.5081	7.3726	7.5127	7.3684	7.5049	7.3800	7.5376	**7.36**
**H6**	7.9770	8.1646	8.2590	8.2681	8.3872	8.2620	8.4387	8.2555	8.4208	8.2738	8.4656	7.9770	8.1646	8.2590	8.2681	8.3872	8.2620	8.4387	8.2555	8.4208	8.2738	8.4656	**8.2**
**H8**	4.5678	4.6311	4.6745	4.6785	4.7338	4.6749	4.7727	4.6711	4.7587	4.6818	4.7496	4.5678	4.6311	4.6745	4.6785	4.7339	4.6749	4.7727	4.6711	4.7588	4.6818	4.7496	**5.4**
**H(9A-9B)**	1.6834	1.7242	1.7339	1.7539	1.7850	1.7522	1.7829	1.7502	1.7789	1.7553	1.8165	1.6834	1.7242	1.7339	1.7539	1.7850	1.7522	1.7829	1.7502	1.7789	1.7553	1.8165	**2.08**
**H(10A)**	1.0055	0.8560	0.8506	0.8221	0.7324	0.8230	0.7821	0.8238	0.7767	0.8214	0.7850	1.0055	0.8560	0.8507	0.8221	0.7324	0.8230	0.7820	0.8238	0.7767	0.8214	0.7850	**1.14**
**H(10B)**	1.6473	1.5445	1.4864	1.4679	1.3838	1.4733	1.3800	1.4783	1.3873	1.4627	1.3500	1.6473	1.5445	1.4864	1.4679	1.3838	1.4733	1.3800	1.4783	1.3873	1.4627	1.3501	**1.28**
**H(11A-11B)**	1.0567	1.0723	1.0451	1.0911	1.0518	1.0902	1.0512	1.0890	1.0511	1.0918	1.0381	1.0567	1.0723	1.0451	1.0910	1.0518	1.0902	1.0512	1.0890	1.0511	1.0918	1.0380	**1.28**
**H(12A-12C)**	0.9504	0.9373	0.9166	0.9332	0.8920	0.9336	0.8995	0.9338	0.9000	0.9328	0.8904	0.9504	0.9373	0.9166	0.9332	0.8920	0.9336	0.8995	0.9338	0.9000	0.9328	0.8904	**0.82**
**H(1`A)**	2.7337	2.7265	2.7160	2.7171	2.6748	2.7178	2.6932	2.7183	2.6947	2.7162	2.6554	2.8635	2.8719	2.8555	2.8496	2.8211	2.8518	2.8323	2.8535	2.8358	2.8473	2.7929	**3.38**
**H(1`B)**	3.1890	3.2388	3.2723	3.2779	3.3445	3.2754	3.3319	3.2727	3.3288	3.2802	3.3270	2.8791	2.7212	2.6927	2.6981	2.6939	2.6982	2.6620	2.6982	2.6627	2.6981	2.6492	**3.71**
**H(2`A)**	1.6302	1.6757	1.6471	1.6964	1.6398	1.6955	1.6665	1.6943	1.6635	1.6971	1.6755	1.5104	1.5573	1.5296	1.5826	1.5248	1.5814	1.5608	1.5798	1.5559	1.5836	1.5751	**2.08**
**H(2`B)**	1.8396	1.8556	1.8161	1.9031	1.8550	1.8993	1.8566	1.8955	1.8505	1.9066	1.8794	1.7018	1.8178	1.7710	1.8908	1.8450	1.8864	1.8696	1.8817	1.8602	1.8949	1.9125	**2.24**
**H(3`A)**	1.5104	1.5573	1.5296	1.5826	1.5248	1.5814	1.5609	1.5798	1.5559	1.5836	1.5751	1.6302	1.6757	1.6471	1.6964	1.6398	1.6955	1.6665	1.6943	1.6635	1.6971	1.6754	**2.08**
**H(3`B)**	1.7018	1.8178	1.7710	1.8908	1.8451	1.8864	1.8697	1.8817	1.8602	1.8949	1.9125	1.8396	1.8556	1.8161	1.9030	1.8550	1.8993	1.8566	1.8955	1.8506	1.9066	1.8794	**2.16**
**H(4`A)**	2.8791	2.7212	2.6928	2.6981	2.6939	2.6982	2.6620	2.6982	2.6627	2.6981	2.6492	3.1890	3.2388	3.2723	3.2779	3.3445	3.2754	3.3319	3.2727	3.3288	3.2802	3.3270	**3.09**
**H(4`B)**	2.8635	2.8719	2.8555	2.8496	2.8211	2.8518	2.8323	2.8535	2.8358	2.8473	2.7929	2.7337	2.7265	2.7160	2.7171	2.6748	2.7178	2.6932	2.7183	2.6947	2.7162	2.6554	**3.68**
Nuclei	mpW1PW91																						
**C1**	136.9119	137.8138	138.5064	138.2193	139.7229	138.2010	139.0452	138.1875	139.1057	138.2354	139.2865	136.9120	137.8138	138.5064	138.2193	139.7229	138.2010	139.0452	138.1875	139.1056	138.2354	139.2865	**132.2**
**C2**	138.8276	138.8043	139.2973	138.6548	139.2065	138.6657	139.6814	138.6819	139.5637	138.6442	139.6547	138.8278	138.8043	139.2971	138.6548	139.2065	138.6657	139.6814	138.6819	139.5637	138.6442	139.6547	**133.3**
**C3**	121.1664	121.9017	122.4756	122.1203	122.8340	122.1098	123.0950	122.1047	123.0259	122.1294	123.1776	121.1664	121.9017	122.4756	122.1203	122.8340	122.1098	123.0950	122.1047	123.0259	122.1294	123.1776	**117.6**
**C4**	173.3303	174.1682	174.7602	174.5577	175.2107	174.5337	175.8375	174.5156	175.6799	174.5797	175.9764	173.3302	174.1682	174.7601	174.5577	175.2107	174.5337	175.8374	174.5156	175.6800	174.5797	175.9764	**168.3**
**C5**	119.1385	120.4194	121.0633	121.0661	121.9587	121.0256	122.1586	120.9910	122.0561	121.1039	122.3339	119.1384	120.4194	121.0632	121.0661	121.9587	121.0256	122.1586	120.9910	122.0561	121.1039	122.3339	**117.6**
**C6**	134.9559	136.8628	138.0011	137.9900	139.5612	137.9156	140.2367	137.8478	139.9729	138.0608	140.6233	134.9558	136.8628	138.0011	137.9900	139.5612	137.9156	140.2368	137.8478	139.9729	138.0608	140.6233	**133.3**
**C7**	201.3961	205.9561	207.0830	208.3989	209.0671	208.2545	213.9532	208.1148	212.8505	208.5345	215.0027	201.3961	205.9561	207.0830	208.3989	209.0671	208.2545	213.9531	208.1148	212.8505	208.5345	215.0027	**195.8**
**C8**	63.7271	64.9001	65.6204	65.4113	66.4236	65.3869	67.0211	65.3670	66.8807	65.4325	67.4256	63.7271	64.9001	65.6204	65.4113	66.4236	65.3869	67.0211	65.3670	66.8807	65.4325	67.4256	**70.4**
**C9**	35.2055	34.9743	35.2048	34.8541	35.1994	34.8613	35.3954	34.8739	35.3130	34.8463	35.5099	35.2055	34.9743	35.2048	34.8541	35.1994	34.8613	35.3953	34.8739	35.3130	34.8463	35.5099	**31.1**
**C10**	27.3826	27.8477	28.3064	28.1713	28.8921	28.1466	28.7034	28.1281	28.6810	28.1948	28.8882	27.3826	27.8477	28.3064	28.1713	28.8921	28.1466	28.7033	28.1281	28.6810	28.1948	28.8882	**26.9**
**C11**	27.3749	27.5041	27.7467	27.4542	27.8881	27.4594	27.8795	27.4701	27.8744	27.4481	28.0331	27.3749	27.5041	27.7467	27.4542	27.8881	27.4594	27.8795	27.4701	27.8744	27.4481	28.0331	**23.4**
**C12**	16.2791	16.4535	16.4886	16.5599	16.6580	16.5511	16.6097	16.5484	16.6016	16.5678	16.6160	16.2792	16.4535	16.4886	16.5599	16.6580	16.5511	16.6097	16.5484	16.6016	16.5678	16.6159	**13.8**
**C1`**	50.5318	51.3520	51.5695	51.8485	52.3867	51.8154	52.3789	51.7886	52.2993	51.8801	52.8661	54.8407	55.9339	56.2529	56.3574	56.8717	56.3332	57.0066	56.3147	56.9463	56.3796	57.2577	**53.3**
**C2`**	24.7484	25.1441	25.4666	25.2813	25.7455	25.2736	25.7527	25.2717	25.7502	25.2877	25.5751	24.2083	24.4220	24.6984	24.5579	25.0088	24.5487	24.9507	24.5454	24.9402	24.5661	24.8202	**24.2**
**C3`**	24.2083	24.4220	24.6984	24.5579	25.0088	24.5487	24.9506	24.5454	24.9402	24.5661	24.8202	24.7485	25.1441	25.4666	25.2813	25.7455	25.2736	25.7527	25.2717	25.7502	25.2877	25.5751	**24.1**
**C4`**	54.8407	55.9339	56.2529	56.3574	56.8717	56.3332	57.0066	56.3147	56.9463	56.3796	57.2577	50.5318	51.3520	51.5695	51.8485	52.3867	51.8154	52.3788	51.7886	52.2993	51.8801	52.8661	**56.3**
**H2**	8.7635	8.6626	8.6890	8.5947	8.5982	8.5987	8.6271	8.6029	8.6234	8.5912	8.6110	8.7635	8.6626	8.6890	8.5947	8.5982	8.5987	8.6270	8.6029	8.6234	8.5912	8.6110	**8.2**
**H3**	7.3658	7.5155	7.6032	7.5610	7.6800	7.5592	7.7031	7.5573	7.6942	7.5630	7.7179	7.3658	7.5155	7.6032	7.5610	7.6800	7.5592	7.7031	7.5573	7.6942	7.5630	7.7179	**7.36**
**H5**	7.1836	7.3496	7.4493	7.4199	7.5570	7.4159	7.5642	7.4120	7.5548	7.4239	7.5904	7.1836	7.3496	7.4493	7.4199	7.5570	7.4159	7.5642	7.4120	7.5548	7.4239	7.5904	**7.36**
**H6**	8.0628	8.2466	8.3313	8.3493	8.4725	8.3429	8.5300	8.3368	8.5082	8.3556	8.5578	8.0628	8.2466	8.3313	8.3493	8.4725	8.3429	8.5300	8.3368	8.5082	8.3556	8.5578	**8.2**
**H8**	4.5666	4.6312	4.6552	4.6838	4.7335	4.6802	4.7762	4.6767	4.7569	4.6876	4.7740	4.5667	4.6312	4.6551	4.6838	4.7335	4.6802	4.7761	4.6767	4.7569	4.6876	4.7740	**5.4**
**H(9A-9B)**	1.7143	1.7479	1.7554	1.7759	1.7961	1.7741	1.7987	1.7723	1.7919	1.7780	1.8300	1.7142	1.7479	1.7554	1.7759	1.7961	1.7741	1.7987	1.7723	1.7919	1.7780	1.8300	**2.08**
**H(10A)**	0.9433	0.8037	0.7605	0.7576	0.6696	0.7596	0.7118	0.7618	0.7018	0.7561	0.7199	0.9433	0.8037	0.7605	0.7576	0.6696	0.7596	0.7118	0.7618	0.7018	0.7561	0.7199	**1.14**
**H(10B)**	1.6289	1.5322	1.4591	1.4457	1.3602	1.4519	1.3550	1.4581	1.3605	1.4403	1.3252	1.6289	1.5322	1.4591	1.4457	1.3602	1.4519	1.3550	1.4581	1.3605	1.4403	1.3252	**1.28**
**H(11A-11B)**	1.0002	1.0196	0.9828	1.0300	0.9938	1.0298	0.9939	1.0296	0.9925	1.0305	0.9821	1.0003	1.0196	0.9828	1.0300	0.9938	1.0298	0.9939	1.0296	0.9925	1.0305	0.9821	**1.28**
**H(12A-12C)**	0.8944	0.8820	0.8543	0.8714	0.8330	0.8723	0.8394	0.8732	0.8390	0.8708	0.8339	0.8944	0.8820	0.8543	0.8714	0.8330	0.8723	0.8393	0.8732	0.8390	0.8708	0.8339	**0.82**
**H(1`A)**	2.6698	2.6627	2.6505	2.6511	2.6099	2.6521	2.6276	2.6531	2.6278	2.6505	2.6009	2.8087	2.8120	2.7991	2.7904	2.7608	2.7925	2.7725	2.7948	2.7761	2.7885	2.7396	**3.38**
**H(1`B)**	3.1591	3.2023	3.2377	3.2375	3.2965	3.2352	3.2866	3.2330	3.2830	3.2401	3.2859	2.8814	2.7114	2.6952	2.6721	2.6718	2.6738	2.6281	2.6757	2.6303	2.6709	2.6132	**3.71**
**H(2`A)**	1.6097	1.6533	1.6251	1.6698	1.6202	1.6692	1.6456	1.6686	1.6423	1.6707	1.6554	1.4812	1.5282	1.4990	1.5488	1.4982	1.5480	1.5338	1.5472	1.5278	1.5499	1.5511	**2.08**
**H(2`B)**	1.7940	1.8165	1.7727	1.8597	1.8175	1.8564	1.8170	1.8534	1.8101	1.8632	1.8446	1.6821	1.7880	1.7383	1.8517	1.8105	1.8478	1.8354	1.8441	1.8250	1.8557	1.8844	**2.24**
**H(3`A)**	1.4812	1.5282	1.4991	1.5488	1.4982	1.5480	1.5338	1.5472	1.5278	1.5499	1.5511	1.6097	1.6533	1.6251	1.6698	1.6202	1.6692	1.6456	1.6686	1.6423	1.6707	1.6554	**2.08**
**H(3`B)**	1.6821	1.7880	1.7383	1.8517	1.8105	1.8478	1.8354	1.8441	1.8250	1.8557	1.8844	1.7940	1.8165	1.7727	1.8597	1.8175	1.8564	1.8170	1.8534	1.8101	1.8632	1.8446	**2.16**
**H(4`A)**	2.8814	2.7114	2.6952	2.6721	2.6718	2.6738	2.6281	2.6757	2.6303	2.6709	2.6132	3.1591	3.2023	3.2377	3.2375	3.2965	3.2352	3.2866	3.2330	3.2830	3.2401	3.2859	**3.09**
**H(4`B)**	2.8087	2.8120	2.7991	2.7904	2.7608	2.7925	2.7725	2.7948	2.7761	2.7885	2.7396	2.6698	2.6627	2.6505	2.6511	2.6099	2.6521	2.6276	2.6531	2.6278	2.6505	2.6009	**3.68**

[a] methyl protons shifts are averaged

[b] according to [5]

Table S5. Cont. IX

Nuclei	R											S											Exp. [b]
	O3LYP																						
	Gas	$CHCl_3$ (PCM)	$CHCl_3$ (SMD)	DMSO (PCM)	DMSO (SMD)	CH_3OH (PCM)	CH_3OH (SMD)	C_2H_5OH (PCM)	C_2H_5OH (SMD)	H_2O (PCM)	H_2O (SMD)	Gas	$CHCl_3$ (PCM)	$CHCl_3$ (SMD)	DMSO (PCM)	DMSO (SMD)	CH_3OH (PCM)	CH_3OH (SMD)	C_2H_5OH (PCM)	C_2H_5OH (SMD)	H_2O (PCM)	H_2O (SMD)	
**C1**	134.8316	136.7253	138.2232	137.7522	139.6525	137.7211	138.6578	137.6778	138.8802	137.7701	138.8881	134.8316	136.7253	138.2232	137.7522	139.6525	137.7212	138.6578	137.6778	138.8802	137.7701	138.8880	**132.2**
**C2**	134.2703	134.3034	134.9028	134.2696	134.8696	134.2834	135.2076	134.2931	135.1090	134.2502	135.1565	134.2703	134.3034	134.9028	134.2695	134.8695	134.2836	135.2076	134.2931	135.1090	134.2502	135.1565	**133.3**
**C3**	117.1530	117.7967	118.4174	118.0690	118.7418	118.0647	118.9947	118.0553	118.9369	118.0662	119.0549	117.1529	117.7967	118.4174	118.0688	118.7418	118.0647	118.9947	118.0553	118.9369	118.0662	119.0548	**117.6**
**C4**	169.5381	170.0292	170.6806	170.3884	171.1053	170.3736	171.6575	170.3540	171.5014	170.3967	171.7332	169.5383	170.0292	170.6806	170.3884	171.1053	170.3735	171.6575	170.3540	171.5014	170.3966	171.7334	**168.3**
**C5**	115.0903	116.2587	116.9125	116.9091	117.6769	116.8807	117.9293	116.8473	117.8196	116.9300	118.0603	115.0903	116.2587	116.9125	116.9091	117.6769	116.8806	117.9293	116.8473	117.8196	116.9299	118.0604	**117.6**
**C6**	130.2300	131.5111	132.3612	132.3562	133.7414	132.3011	134.3672	132.2439	134.1030	132.4041	134.6024	130.2299	131.5111	132.3612	132.3563	133.7414	132.3011	134.3672	132.2439	134.1030	132.4042	134.6023	**133.3**
**C7**	197.0256	202.4069	204.2489	205.5483	206.5094	205.3897	210.9074	205.2170	210.0109	205.6862	211.8448	197.0256	202.4069	204.2489	205.5484	206.5094	205.3897	210.9074	205.2170	210.0109	205.6859	211.8450	**195.8**
**C8**	64.5596	66.2848	66.9158	67.1212	67.8247	67.0947	68.4239	67.0586	68.3028	67.1363	68.7969	64.5596	66.2848	66.9158	67.1213	67.8247	67.0948	68.4239	67.0586	68.3028	67.1362	68.7968	**70.4**
**C9**	35.5442	35.1982	35.2977	35.1245	35.2805	35.1355	35.5634	35.1421	35.4857	35.1083	35.6327	35.5442	35.1982	35.2977	35.1245	35.2805	35.1354	35.5634	35.1421	35.4857	35.1083	35.6327	**31.1**
**C10**	27.9914	28.4344	28.8370	28.7329	29.4158	28.7216	29.2733	28.7061	29.2396	28.7375	29.4326	27.9915	28.4344	28.8370	28.7329	29.4156	28.7216	29.2733	28.7061	29.2396	28.7376	29.4325	**26.9**
**C11**	28.7716	28.8814	29.1958	28.9216	29.3506	28.9307	29.3249	28.9345	29.3252	28.9062	29.4166	28.7716	28.8814	29.1958	28.9217	29.3505	28.9308	29.3249	28.9345	29.3252	28.9062	29.4166	**23.4**
**C12**	16.6093	16.7634	16.8889	16.9451	17.0851	16.9427	17.0428	16.9355	17.0386	16.9411	17.0277	16.6092	16.7634	16.8889	16.9451	17.0851	16.9427	17.0428	16.9355	17.0386	16.9411	17.0277	**13.8**
**C1`**	50.8019	51.5543	51.9691	52.1253	52.7447	52.0980	52.7177	52.0662	52.6691	52.1450	53.0975	55.2428	56.2398	56.6369	56.7276	57.3004	56.7088	57.3728	56.6851	57.2958	56.7387	57.5907	**53.3**
**C2`**	25.9053	26.4060	26.7287	26.6449	27.0942	26.6421	27.1363	26.6341	27.1078	26.6410	26.9388	25.6921	25.8886	26.3279	26.0899	26.6685	26.0890	26.5111	26.0826	26.5199	26.0843	26.2550	**24.2**
**C3`**	25.6920	25.8886	26.3279	26.0899	26.6685	26.0889	26.5111	26.0826	26.5199	26.0842	26.2549	25.9053	26.4060	26.7287	26.6449	27.0942	26.6421	27.1363	26.6341	27.1078	26.6411	26.9389	**24.1**
**C4`**	55.2428	56.2398	56.6369	56.7276	57.3004	56.7089	57.3728	56.6851	57.2958	56.7388	57.5907	50.8019	51.5543	51.9691	52.1253	52.7448	52.0981	52.7178	52.0662	52.6691	52.1450	53.0974	**56.3**
**H2**	8.5172	8.3119	8.3022	8.2114	8.2142	8.2161	8.2765	8.2212	8.2596	8.2074	8.2657	8.5172	8.3119	8.3022	8.2114	8.2142	8.2161	8.2765	8.2212	8.2596	8.2074	8.2656	**8.2**
**H3**	7.3026	7.4020	7.4910	7.4435	7.5680	7.4415	7.5874	7.4391	7.5799	7.4452	7.5960	7.3026	7.4020	7.4910	7.4435	7.5680	7.4415	7.5874	7.4391	7.5799	7.4452	7.5960	**7.36**
**H5**	7.0527	7.2542	7.3671	7.3566	7.4848	7.3519	7.5014	7.3466	7.4911	7.3609	7.5199	7.0527	7.2542	7.3671	7.3566	7.4848	7.3518	7.5014	7.3466	7.4911	7.3609	7.5199	**7.36**
**H6**	7.9525	8.0429	8.1075	8.0893	8.2184	8.0851	8.2708	8.0810	8.2439	8.0936	8.2835	7.9525	8.0429	8.1075	8.0893	8.2184	8.0851	8.2708	8.0810	8.2439	8.0936	8.2835	**8.2**
**H8**	4.7795	4.7678	4.7948	4.7779	4.8539	4.7763	4.8754	4.7747	4.8616	4.7796	4.8585	4.7795	4.7678	4.7948	4.7779	4.8539	4.7763	4.8754	4.7747	4.8616	4.7796	4.8584	**5.4**
**H(9A-9B)**	1.6182	1.6249	1.5871	1.6386	1.6272	1.6368	1.6562	1.6349	1.6437	1.6405	1.6970	1.6182	1.6249	1.5871	1.6386	1.6272	1.6368	1.6562	1.6349	1.6437	1.6405	1.6970	**2.08**
**H(10A)**	0.9587	0.8526	0.8607	0.8353	0.8038	0.8362	0.8278	0.8369	0.8280	0.8343	0.8249	0.9587	0.8526	0.8607	0.8353	0.8038	0.8362	0.8278	0.8369	0.8280	0.8343	0.8249	**1.14**
**H(10B)**	1.5716	1.4752	1.4290	1.4076	1.3426	1.4126	1.3358	1.4173	1.3447	1.4028	1.3107	1.5716	1.4752	1.4290	1.4076	1.3426	1.4126	1.3358	1.4173	1.3447	1.4028	1.3107	**1.28**
**H(11A-11B)**	1.0598	1.0760	1.0550	1.0975	1.0725	1.0966	1.0683	1.0953	1.0683	1.0983	1.0500	1.0598	1.0760	1.0550	1.0975	1.0724	1.0966	1.0683	1.0953	1.0683	1.0983	1.0500	**1.28**
**H(12A-12C)**	0.9311	0.9235	0.9109	0.9235	0.8974	0.9240	0.8992	0.9241	0.9006	0.9229	0.8910	0.9311	0.9235	0.9109	0.9235	0.8974	0.9239	0.8992	0.9241	0.9006	0.9229	0.8910	**0.82**
**H(1`A)**	2.7818	2.7720	2.7498	2.7606	2.7136	2.7618	2.7281	2.7626	2.7293	2.7594	2.6945	2.9185	2.9246	2.8997	2.8958	2.8542	2.8987	2.8592	2.9012	2.8637	2.8929	2.8314	**3.38**
**H(1`B)**	3.2477	3.2925	3.3182	3.3299	3.3897	3.3277	3.3821	3.3252	3.3769	3.3319	3.3829	2.9024	2.7877	2.7774	2.7855	2.7869	2.7851	2.7591	2.7844	2.7667	2.7859	2.7593	**3.71**
**H(2`A)**	1.6302	1.6734	1.6511	1.6905	1.6416	1.6900	1.6653	1.6893	1.6625	1.6908	1.6723	1.5249	1.5696	1.5582	1.5949	1.5522	1.5941	1.5774	1.5929	1.5757	1.5954	1.5892	**2.08**
**H(2`B)**	1.8442	1.8517	1.8178	1.8948	1.8599	1.8917	1.8559	1.8884	1.8516	1.8978	1.8790	1.7452	1.8526	1.8263	1.9301	1.9019	1.9256	1.9145	1.9206	1.9081	1.9343	1.9549	**2.24**
**H(3`A)**	1.5249	1.5696	1.5582	1.5949	1.5522	1.5941	1.5774	1.5929	1.5757	1.5954	1.5892	1.6302	1.6734	1.6511	1.6905	1.6416	1.6900	1.6653	1.6893	1.6625	1.6908	1.6724	**2.08**
**H(3`B)**	1.7452	1.8526	1.8263	1.9302	1.9019	1.9256	1.9144	1.9206	1.9081	1.9343	1.9549	1.8442	1.8517	1.8178	1.8948	1.8599	1.8917	1.8559	1.8884	1.8516	1.8978	1.8790	**2.16**
**H(4`A)**	2.9024	2.7877	2.7774	2.7855	2.7869	2.7851	2.7591	2.7844	2.7670	2.7859	2.7593	3.2477	3.2925	3.3182	3.3299	3.3897	3.3277	3.3821	3.3252	3.3769	3.3319	3.3829	**3.09**
**H(4`B)**	2.9185	2.9246	2.8997	2.8958	2.8542	2.8987	2.8592	2.9013	2.8637	2.8929	2.8314	2.7818	2.7720	2.7498	2.7606	2.7136	2.7618	2.7281	2.7626	2.7293	2.7594	2.6945	**3.68**
Nuclei	OLYP																						
**C1**	133.9821	136.0335	137.1367	136.6843	138.8035	136.6625	137.7402	136.6347	138.0258	136.6956	137.8922	133.9821	136.0335	137.1367	136.6843	138.8035	136.6625	137.7402	136.6347	138.0258	136.6955	137.8922	**132.2**
**C2**	131.9334	131.9290	132.5576	131.8799	132.4225	131.8897	132.7752	131.8944	132.6437	131.8621	132.7099	131.9334	131.9291	132.5576	131.8799	132.4223	131.8897	132.7752	131.8944	132.6437	131.8621	132.7099	**133.3**
**C3**	115.3313	115.9906	116.6137	116.2608	116.8575	116.2547	117.1455	116.2428	117.0771	116.2573	117.2258	115.3313	115.9906	116.6137	116.2609	116.8575	116.2547	117.1455	116.2429	117.0771	116.2573	117.2258	**117.6**
**C4**	168.3478	168.9536	169.7150	169.3969	170.1310	169.3775	170.6738	169.3528	170.4989	169.4065	170.7682	168.3478	168.9536	169.7150	169.3969	170.1309	169.3775	170.6738	169.3529	170.4989	169.4065	170.7682	**168.3**
**C5**	113.3354	114.4044	115.0462	115.0266	115.7191	114.9969	116.0153	114.9618	115.8944	115.0458	116.1580	113.3354	114.4045	115.0462	115.0266	115.7191	114.9969	116.0153	114.9617	115.8944	115.0458	116.1580	**117.6**
**C6**	127.6467	128.8978	129.9328	129.9379	131.2665	129.8770	131.8460	129.8113	131.5682	129.9875	132.0896	127.6467	128.8977	129.9328	129.9380	131.2665	129.8770	131.8460	129.8113	131.5682	129.9876	132.0896	**133.3**
**C7**	195.5731	201.1277	202.5411	203.7564	205.1541	203.6174	209.0255	203.4714	208.3346	203.8785	209.8788	195.5731	201.1277	202.5411	203.7565	205.1539	203.6174	209.0255	203.4717	208.3346	203.8785	209.8788	**195.8**
**C8**	66.0732	67.9957	68.4903	68.7224	69.6196	68.7003	70.0870	68.6709	70.0972	68.7322	70.3344	66.0732	67.9958	68.4903	68.7226	69.6196	68.7004	70.0870	68.6709	70.0972	68.7322	70.3344	**70.4**
**C9**	36.5013	36.1561	36.3808	36.1692	36.2657	36.1751	36.6313	36.1753	36.5703	36.1547	36.6703	36.5013	36.1562	36.3808	36.1692	36.2656	36.1751	36.6313	36.1753	36.5703	36.1547	36.6703	**31.1**
**C10**	28.6233	29.0478	29.5021	29.3898	30.0161	29.3768	29.8944	29.3581	29.8818	29.3929	30.0503	28.6233	29.0477	29.5021	29.3898	30.0160	29.3768	29.8944	29.3580	29.8818	29.3930	30.0503	**26.9**
**C11**	29.6468	29.7271	30.0848	29.7850	30.1842	29.7907	30.1750	29.7910	30.1811	29.7704	30.2632	29.6468	29.7272	30.0848	29.7849	30.1842	29.7907	30.1749	29.7910	30.1811	29.7704	30.2632	**23.4**
**C12**	16.7637	16.9097	17.0328	17.0844	17.2029	17.0811	17.1660	17.0727	17.1626	17.0786	17.1506	16.7637	16.9097	17.0328	17.0843	17.2029	17.0811	17.1660	17.0727	17.1626	17.0786	17.1506	**13.8**
**C1`**	51.7337	52.4858	52.9328	53.0582	53.6707	53.0297	53.6672	52.9964	53.6005	53.0766	54.0194	56.2482	57.2354	57.6350	57.7173	58.2870	57.6968	58.3670	57.6709	58.2796	57.7275	58.6081	**53.3**
**C2`**	26.4609	26.9933	27.3434	27.2952	27.6992	27.2887	27.7603	27.2761	27.7417	27.2919	27.6012	26.4501	26.6417	27.0756	26.8535	27.4196	26.8487	27.2629	26.8387	27.2654	26.8497	27.0231	**24.2**
**C3`**	26.4501	26.6418	27.0756	26.8535	27.4195	26.8487	27.2629	26.8388	27.2654	26.8497	27.0231	26.4609	26.9933	27.3434	27.2952	27.6992	27.2887	27.7603	27.2761	27.7417	27.2919	27.6012	**24.1**
**C4`**	56.2482	57.2355	57.6350	57.7172	58.2870	57.6968	58.3669	57.6709	58.2796	57.7275	58.6081	51.7337	52.4859	52.9328	53.0581	53.6707	53.0297	53.6672	52.9964	53.6005	53.0766	54.0194	**56.3**
**H2**	8.3249	8.1241	8.1537	8.0646	8.0342	8.0676	8.1182	8.0705	8.0902	8.0614	8.1158	8.3249	8.1241	8.1537	8.0646	8.0341	8.0676	8.1182	8.0705	8.0902	8.0614	8.1158	**8.2**
**H3**	7.2045	7.3251	7.4204	7.3707	7.4933	7.3687	7.5141	7.3664	7.5040	7.3723	7.5211	7.2045	7.3251	7.4204	7.3707	7.4933	7.3687	7.5141	7.3664	7.5040	7.3723	7.5211	**7.36**
**H5**	6.9910	7.2033	7.3016	7.2888	7.4115	7.2844	7.4314	7.2798	7.4198	7.2926	7.4471	6.9910	7.2033	7.3016	7.2888	7.4115	7.2844	7.4314	7.2798	7.4198	7.2926	7.4471	**7.36**
**H6**	7.8487	7.9010	7.9896	7.9745	8.0789	7.9695	8.1350	7.9645	8.1011	7.9789	8.1569	7.8487	7.9010	7.9896	7.9745	8.0789	7.9695	8.1350	7.9645	8.1011	7.9789	8.1569	**8.2**
**H8**	4.9446	4.9128	4.9412	4.9248	4.9737	4.9235	4.9921	4.9221	4.9774	4.9258	4.9759	4.9446	4.9128	4.9412	4.9248	4.9737	4.9235	4.9921	4.9221	4.9774	4.9258	4.9759	**5.4**
**H(9A-9B)**	1.6234	1.6455	1.6374	1.6833	1.6640	1.6808	1.6972	1.6781	1.6877	1.6853	1.7322	1.6234	1.6455	1.6374	1.6833	1.6640	1.6808	1.6972	1.6781	1.6877	1.6853	1.7322	**2.08**
**H(10A)**	0.9953	0.8946	0.8938	0.8729	0.8450	0.8735	0.8734	0.8742	0.8679	0.8721	0.8733	0.9953	0.8946	0.8938	0.8729	0.8450	0.8735	0.8734	0.8742	0.8679	0.8721	0.8733	**1.14**
**H(10B)**	1.5882	1.4997	1.4577	1.4325	1.3742	1.4374	1.3635	1.4420	1.3716	1.4275	1.3377	1.5882	1.4997	1.4577	1.4325	1.3742	1.4374	1.3635	1.4420	1.3716	1.4275	1.3377	**1.28**
**H(11A-11B)**	1.1047	1.1212	1.1028	1.1423	1.1197	1.1410	1.1151	1.1396	1.1134	1.1432	1.0967	1.1047	1.1213	1.1028	1.1423	1.1197	1.1410	1.1151	1.1396	1.1134	1.1432	1.0967	**1.28**
**H(12A-12C)**	0.9447	0.9384	0.9250	0.9365	0.9136	0.9368	0.9157	0.9370	0.9158	0.9358	0.9079	0.9447	0.9384	0.9250	0.9365	0.9136	0.9368	0.9157	0.9370	0.9158	0.9358	0.9079	**0.82**
**H(1`A)**	2.8493	2.8305	2.8097	2.8183	2.7703	2.8195	2.7798	2.8205	2.7831	2.8167	2.7472	2.9785	2.9775	2.9559	2.9533	2.9142	2.9558	2.9129	2.9582	2.9219	2.9503	2.8855	**3.38**
**H(1`B)**	3.3360	3.3804	3.4119	3.4210	3.4791	3.4185	3.4699	3.4159	3.4664	3.4229	3.4658	2.9866	2.8809	2.8679	2.8706	2.8859	2.8704	2.8588	2.8703	2.8677	2.8705	2.8536	**3.71**
**H(2`A)**	1.6502	1.6910	1.6705	1.7077	1.6607	1.7071	1.6856	1.7064	1.6822	1.7079	1.6958	1.5551	1.5955	1.5818	1.6135	1.5777	1.6127	1.6012	1.6118	1.5983	1.6138	1.6130	**2.08**
**H(2`B)**	1.9000	1.8976	1.8633	1.9323	1.8944	1.9293	1.8932	1.9264	1.8871	1.9348	1.9145	1.7989	1.9092	1.8751	1.9769	1.9570	1.9728	1.9659	1.9687	1.9582	1.9804	2.0063	**2.24**
**H(3`A)**	1.5551	1.5955	1.5818	1.6135	1.5777	1.6127	1.6012	1.6118	1.5983	1.6138	1.6130	1.6502	1.6910	1.6705	1.7077	1.6607	1.7071	1.6856	1.7064	1.6822	1.7079	1.6958	**2.08**
**H(3`B)**	1.7989	1.9092	1.8751	1.9769	1.9570	1.9728	1.9659	1.9687	1.9582	1.9804	2.0063	1.9000	1.8976	1.8633	1.9322	1.8944	1.9293	1.8932	1.9264	1.8871	1.9348	1.9145	**2.16**
**H(4`A)**	2.9866	2.8809	2.8679	2.8706	2.8859	2.8705	2.8588	2.8703	2.8677	2.8705	2.8536	3.3360	3.3804	3.4119	3.4210	3.4791	3.4185	3.4699	3.4159	3.4664	3.4229	3.4658	**3.09**
**H(4`B)**	2.9785	2.9775	2.9559	2.9533	2.9142	2.9558	2.9129	2.9582	2.9219	2.9503	2.8855	2.8493	2.8305	2.8097	2.8182	2.7703	2.8195	2.7798	2.8205	2.7831	2.8167	2.7472	**3.68**

[a] methyl protons shifts are averaged

[b] according to [5]

Table S5. Cont. X

Nuclei	R											S											Exp. [b]
	OPBE																						
	Gas	$CHCl_3$ (PCM)	$CHCl_3$ (SMD)	DMSO (PCM)	DMSO (SMD)	CH_3OH (PCM)	CH_3OH (SMD)	C_2H_5OH (PCM)	C_2H_5OH (SMD)	H_2O (PCM)	H_2O (SMD)	Gas	$CHCl_3$ (PCM)	$CHCl_3$ (SMD)	DMSO (PCM)	DMSO (SMD)	CH_3OH (PCM)	CH_3OH (SMD)	C_2H_5OH (PCM)	C_2H_5OH (SMD)	H_2O (PCM)	H_2O (SMD)	
**C1**	130.6787	132.6297	133.6336	133.3807	135.3198	133.3723	134.3661	133.3448	134.5349	133.3821	134.5269	130.6787	132.6296	133.6335	133.3807	135.3197	133.3723	134.3660	133.3448	134.5349	133.3821	134.5268	**132.2**
**C2**	130.5026	130.6500	131.1455	130.5229	131.1203	130.5465	131.4274	130.5522	131.3339	130.4956	131.4311	130.5026	130.6499	131.1455	130.5229	131.1203	130.5465	131.4274	130.5522	131.3339	130.4956	131.4309	**133.3**
**C3**	114.1774	114.9620	115.5644	115.1917	115.9530	115.1953	116.1865	115.1808	116.1149	115.1830	116.2950	114.1774	114.9619	115.5645	115.1917	115.9531	115.1953	116.1864	115.1808	116.1149	115.1830	116.2949	**117.6**
**C4**	164.7994	165.4524	166.0718	165.7743	166.4439	165.7692	166.9958	165.7465	166.8494	165.7742	167.1261	164.7994	165.4524	166.0719	165.7743	166.4440	165.7692	166.9958	165.7465	166.8494	165.7742	167.1261	**168.3**
**C5**	112.2619	113.5119	114.1131	114.0723	114.8593	114.0525	115.1463	114.0152	115.0300	114.0864	115.3281	112.2618	113.5119	114.1130	114.0723	114.8593	114.0525	115.1464	114.0152	115.0300	114.0864	115.3282	**117.6**
**C6**	126.2248	127.5436	128.4947	128.4132	129.7170	128.3671	130.2985	128.3037	130.0776	128.4529	130.5759	126.2248	127.5437	128.4947	128.4132	129.7170	128.3671	130.2986	128.3037	130.0776	128.4529	130.5758	**133.3**
**C7**	192.8492	198.0469	199.3180	200.6479	201.7981	200.5296	205.5758	200.3919	204.7558	200.7535	206.3278	192.8491	198.0466	199.3180	200.6479	201.7981	200.5296	205.5759	200.3919	204.7558	200.7535	206.3275	**195.8**
**C8**	63.5007	65.3509	66.0163	66.0708	67.1112	66.0585	67.5850	66.0270	67.4654	66.0760	67.8053	63.5007	65.3510	66.0163	66.0708	67.1112	66.0585	67.5850	66.0270	67.4654	66.0760	67.8053	**70.4**
**C9**	34.3082	34.1893	34.3213	34.1125	34.3179	34.1310	34.6281	34.1317	34.5209	34.0902	34.6999	34.3083	34.1893	34.3213	34.1125	34.3179	34.1310	34.6281	34.1317	34.5209	34.0902	34.6998	**31.1**
**C10**	26.3655	26.8815	27.2838	27.1239	27.8025	27.1222	27.6443	27.1030	27.6205	27.1207	27.8389	26.3655	26.8814	27.2838	27.1239	27.8024	27.1222	27.6443	27.1030	27.6205	27.1207	27.8389	**26.9**
**C11**	27.3220	27.5273	27.7359	27.4928	27.8362	27.5128	27.8134	27.5147	27.8103	27.4686	27.9319	27.3219	27.5273	27.7360	27.4928	27.8361	27.5128	27.8134	27.5147	27.8103	27.4686	27.9319	**23.4**
**C12**	16.0776	16.2963	16.3097	16.3835	16.4232	16.3927	16.4102	16.3844	16.4094	16.3702	16.4127	16.0776	16.2963	16.3098	16.3835	16.4232	16.3927	16.4102	16.3844	16.4094	16.3702	16.4127	**13.8**
**C1`**	49.8315	50.6513	50.9131	51.1124	51.5794	51.0991	51.6068	51.0680	51.5522	51.1202	52.0407	54.1207	55.1917	55.5154	55.5904	56.1099	55.5833	56.1855	55.5579	56.1235	55.5921	56.4254	**53.3**
**C2`**	25.1000	25.6677	25.9114	25.8276	26.2209	25.8356	26.2347	25.8256	26.2242	25.8151	25.9714	24.9036	25.1884	25.4269	25.2649	25.6750	25.2773	25.5882	25.2721	25.5991	25.2486	25.3040	**24.2**
**C3`**	24.9037	25.1884	25.4268	25.2649	25.6751	25.2773	25.5881	25.2721	25.5991	25.2486	25.3041	25.1001	25.6677	25.9114	25.8276	26.2210	25.8356	26.2347	25.8256	26.2242	25.8151	25.9714	**24.1**
**C4`**	54.1207	55.1916	55.5153	55.5904	56.1100	55.5833	56.1855	55.5579	56.1235	55.5921	56.4254	49.8315	50.6514	50.9130	51.1124	51.5794	51.0991	51.6068	51.0680	51.5522	51.1201	52.0406	**56.3**
**H2**	8.3644	8.1928	8.2044	8.1131	8.0948	8.1166	8.1711	8.1195	8.1553	8.1092	8.1745	8.3644	8.1928	8.2044	8.1131	8.0948	8.1166	8.1711	8.1195	8.1553	8.1092	8.1745	**8.2**
**H3**	7.2317	7.3506	7.4367	7.3986	7.5153	7.3968	7.5392	7.3943	7.5305	7.3995	7.5502	7.2317	7.3506	7.4367	7.3986	7.5153	7.3968	7.5392	7.3943	7.5305	7.3995	7.5502	**7.36**
**H5**	7.0085	7.2266	7.3170	7.3144	7.4418	7.3103	7.4561	7.3054	7.4452	7.3175	7.4766	7.0085	7.2266	7.3170	7.3144	7.4418	7.3103	7.4561	7.3054	7.4452	7.3175	7.4766	**7.36**
**H6**	7.9097	7.9800	8.0576	8.0368	8.1441	8.0322	8.1953	8.0270	8.1733	8.0405	8.2139	7.9097	7.9800	8.0576	8.0368	8.1441	8.0322	8.1953	8.0270	8.1733	8.0405	8.2139	**8.2**
**H8**	4.9509	4.9173	4.9339	4.9242	4.9761	4.9229	4.9923	4.9211	4.9808	4.9248	4.9868	4.9509	4.9173	4.9339	4.9242	4.9761	4.9229	4.9923	4.9211	4.9808	4.9248	4.9868	**5.4**
**H(9A-9B)**	1.6537	1.6636	1.6523	1.6862	1.6774	1.6845	1.7050	1.6821	1.6910	1.6872	1.7441	1.6537	1.6636	1.6523	1.6862	1.6774	1.6845	1.7050	1.6821	1.6910	1.6872	1.7441	**2.08**
**H(10A)**	0.9080	0.8215	0.7760	0.7960	0.7277	0.7977	0.7746	0.7987	0.7708	0.7938	0.7812	0.9080	0.8214	0.7760	0.7960	0.7277	0.7977	0.7746	0.7987	0.7708	0.7938	0.7812	**1.14**
**H(10B)**	1.5468	1.4570	1.4015	1.3905	1.3184	1.3954	1.3106	1.3997	1.3176	1.3850	1.2885	1.5468	1.4570	1.4015	1.3905	1.3184	1.3954	1.3106	1.3997	1.3176	1.3850	1.2884	**1.28**
**H(11A-11B)**	1.0380	1.0583	1.0237	1.0740	1.0368	1.0738	1.0344	1.0727	1.0341	1.0737	1.0195	1.0380	1.0583	1.0237	1.0740	1.0368	1.0738	1.0344	1.0727	1.0341	1.0737	1.0195	**1.28**
**H(12A-12C)**	0.8763	0.8722	0.8453	0.8670	0.8301	0.8680	0.8355	0.8684	0.8363	0.8653	0.8297	0.8763	0.8722	0.8453	0.8670	0.8301	0.8680	0.8355	0.8684	0.8363	0.8653	0.8297	**0.82**
**H(1`A)**	2.7652	2.7626	2.7400	2.7489	2.7012	2.7503	2.7146	2.7511	2.7162	2.7467	2.6727	2.9190	2.9188	2.8953	2.8880	2.8426	2.8911	2.8529	2.8934	2.8571	2.8843	2.8212	**3.38**
**H(1`B)**	3.2993	3.3459	3.3765	3.3785	3.4327	3.3769	3.4258	3.3746	3.4221	3.3793	3.4161	2.9751	2.8559	2.8439	2.8403	2.8379	2.8409	2.8138	2.8411	2.8191	2.8390	2.8061	**3.71**
**H(2`A)**	1.6236	1.6655	1.6386	1.6769	1.6256	1.6769	1.6499	1.6762	1.6468	1.6761	1.6606	1.5167	1.5612	1.5363	1.5781	1.5236	1.5780	1.5576	1.5771	1.5551	1.5774	1.5747	**2.08**
**H(2`B)**	1.8275	1.8380	1.7934	1.8734	1.8231	1.8710	1.8287	1.8680	1.8227	1.8751	1.8597	1.7579	1.8554	1.8133	1.9228	1.8837	1.9191	1.9005	1.9147	1.8947	1.9257	1.9488	**2.24**
**H(3`A)**	1.5167	1.5612	1.5363	1.5781	1.5236	1.5780	1.5576	1.5771	1.5551	1.5774	1.5747	1.6236	1.6655	1.6386	1.6769	1.6256	1.6769	1.6499	1.6762	1.6468	1.6761	1.6606	**2.08**
**H(3`B)**	1.7579	1.8554	1.8133	1.9228	1.8837	1.9191	1.9005	1.9147	1.8947	1.9257	1.9488	1.8275	1.8380	1.7934	1.8734	1.8231	1.8710	1.8287	1.8680	1.8227	1.8751	1.8597	**2.16**
**H(4`A)**	2.9751	2.8559	2.8439	2.8403	2.8379	2.8409	2.8138	2.8411	2.8191	2.8390	2.8061	3.2993	3.3459	3.3765	3.3785	3.4327	3.3769	3.4258	3.3746	3.4221	3.3793	3.4161	**3.09**
**H(4`B)**	2.9190	2.9187	2.8953	2.8880	2.8426	2.8911	2.8529	2.8934	2.8571	2.8843	2.8212	2.7652	2.7625	2.7400	2.7489	2.7012	2.7503	2.7146	2.7511	2.7162	2.7467	2.6727	**3.68**
Nuclei	OPW91																						
**C1**	130.9989	132.9583	133.9539	133.7056	135.6329	133.6877	134.6754	133.6513	134.8514	133.7031	134.8410	130.9989	132.9582	133.9539	133.7056	135.6330	133.6877	134.6754	133.6513	134.8515	133.7031	134.8410	**132.2**
**C2**	130.7262	130.8703	131.3673	130.7503	131.3340	130.7635	131.6450	130.7599	131.5541	130.7194	131.6496	130.7261	130.8703	131.3673	130.7503	131.3339	130.7635	131.6450	130.7599	131.5542	130.7194	131.6496	**133.3**
**C3**	114.3764	115.1564	115.7533	115.3897	116.1354	115.3831	116.3698	115.3596	116.2991	115.3771	116.4793	114.3764	115.1565	115.7533	115.3897	116.1354	115.3831	116.3698	115.3596	116.2991	115.3771	116.4793	**117.6**
**C4**	165.2041	165.8588	166.4788	166.1876	166.8477	166.1725	167.4033	166.1404	167.2572	166.1838	167.5338	165.2041	165.8588	166.4788	166.1876	166.8476	166.1725	167.4033	166.1404	167.2572	166.1838	167.5338	**168.3**
**C5**	112.4572	113.7054	114.3021	114.2692	115.0406	114.2396	115.3290	114.1931	115.2123	114.2792	115.5116	112.4573	113.7054	114.3021	114.2692	115.0407	114.2396	115.3290	114.1931	115.2123	114.2792	115.5116	**117.6**
**C6**	126.4459	127.7681	128.7165	128.6476	129.9387	128.5911	130.5278	128.5186	130.3040	128.6837	130.8058	126.4460	127.7681	128.7164	128.6476	129.9387	128.5911	130.5278	128.5186	130.3040	128.6837	130.8058	**133.3**
**C7**	193.2350	198.4648	199.7275	201.0721	202.2060	200.9435	206.0081	200.7961	205.1882	201.1740	206.7657	193.2352	198.4648	199.7276	201.0721	202.2063	200.9435	206.0081	200.7961	205.1882	201.1740	206.7657	**195.8**
**C8**	63.7228	65.5786	66.2262	66.3004	67.3297	66.2783	67.7924	66.2379	67.6675	66.3014	68.0264	63.7228	65.5786	66.2262	66.3004	67.3296	66.2783	67.7924	66.2379	67.6675	66.3014	68.0264	**70.4**
**C9**	34.4593	34.3374	34.4633	34.2679	34.4630	34.2763	34.7706	34.2678	34.6619	34.2417	34.8495	34.4593	34.3374	34.4633	34.2679	34.4630	34.2763	34.7706	34.2678	34.6619	34.2417	34.8495	**31.1**
**C10**	26.4933	27.0116	27.4118	27.2602	27.9311	27.2485	27.7713	27.2200	27.7463	27.2531	27.9654	26.4932	27.0116	27.4118	27.2602	27.9312	27.2485	27.7713	27.2200	27.7464	27.2531	27.9654	**26.9**
**C11**	27.4281	27.6358	27.8457	27.6079	27.9463	27.6176	27.9231	27.6102	27.9202	27.5798	28.0426	27.4281	27.6358	27.8457	27.6079	27.9462	27.6176	27.9231	27.6102	27.9203	27.5798	28.0426	**23.4**
**C12**	16.1147	16.3379	16.3532	16.4317	16.4668	16.4307	16.4541	16.4131	16.4540	16.4146	16.4592	16.1147	16.3380	16.3532	16.4317	16.4669	16.4307	16.4541	16.4131	16.4540	16.4146	16.4592	**13.8**
**C1`**	49.9744	50.7955	51.0613	51.2629	51.7275	51.2393	51.7560	51.1989	51.7048	51.2672	52.1859	54.2713	55.3450	55.6693	55.7494	56.2629	55.7320	56.3386	55.6974	56.2757	55.7475	56.5821	**53.3**
**C2`**	25.2138	25.7880	26.0320	25.9569	26.3447	25.9547	26.3594	25.9356	26.3475	25.9405	26.1094	25.0229	25.3126	25.5528	25.3973	25.7998	25.3995	25.7152	25.3852	25.7270	25.3771	25.4366	**24.2**
**C3`**	25.0230	25.3125	25.5528	25.3973	25.7999	25.3995	25.7152	25.3852	25.7270	25.3771	25.4366	25.2138	25.7880	26.0320	25.9569	26.3447	25.9547	26.3594	25.9356	26.3475	25.9405	26.1094	**24.1**
**C4`**	54.2713	55.3450	55.6693	55.7494	56.2627	55.7320	56.3386	55.6974	56.2757	55.7475	56.5821	49.9744	50.7955	51.0613	51.2629	51.7274	51.2393	51.7560	51.1989	51.7048	51.2672	52.1859	**56.3**
**H2**	8.3657	8.1926	8.2058	8.1146	8.0958	8.1174	8.1723	8.1200	8.1563	8.1104	8.1754	8.3657	8.1926	8.2058	8.1146	8.0958	8.1174	8.1723	8.1200	8.1564	8.1104	8.1754	**8.2**
**H3**	7.2334	7.3518	7.4382	7.4004	7.5160	7.3980	7.5403	7.3952	7.5319	7.4010	7.5508	7.2334	7.3518	7.4382	7.4004	7.5160	7.3980	7.5403	7.3952	7.5319	7.4010	7.5508	**7.36**
**H5**	7.0105	7.2283	7.3185	7.3163	7.4423	7.3115	7.4569	7.3065	7.4459	7.3190	7.4773	7.0105	7.2283	7.3185	7.3163	7.4423	7.3115	7.4569	7.3065	7.4458	7.3190	7.4773	**7.36**
**H6**	7.9098	7.9795	8.0579	8.0377	8.1442	8.0324	8.1963	8.0270	8.1738	8.0412	8.2151	7.9098	7.9795	8.0579	8.0377	8.1442	8.0324	8.1963	8.0270	8.1738	8.0412	8.2151	**8.2**
**H8**	4.9491	4.9159	4.9327	4.9236	4.9747	4.9217	4.9913	4.9198	4.9796	4.9239	4.9859	4.9491	4.9159	4.9327	4.9236	4.9747	4.9217	4.9913	4.9198	4.9796	4.9239	4.9859	**5.4**
**H(9A-9B)**	1.6497	1.6605	1.6494	1.6849	1.6763	1.6824	1.7032	1.6798	1.6890	1.6857	1.7429	1.6497	1.6605	1.6494	1.6849	1.6763	1.6824	1.7032	1.6798	1.6890	1.6857	1.7429	**2.08**
**H(10A)**	0.9129	0.8260	0.7820	0.8013	0.7323	0.8023	0.7801	0.8031	0.7773	0.7988	0.7858	0.9129	0.8260	0.7820	0.8013	0.7323	0.8023	0.7801	0.8031	0.7773	0.7988	0.7858	**1.14**
**H(10B)**	1.5483	1.4585	1.4043	1.3926	1.3200	1.3969	1.3127	1.4010	1.3200	1.3868	1.2904	1.5483	1.4585	1.4043	1.3926	1.3200	1.3969	1.3127	1.4010	1.3200	1.3868	1.2904	**1.28**
**H(11A-11B)**	1.0395	1.0599	1.0261	1.0765	1.0389	1.0755	1.0369	1.0742	1.0367	1.0758	1.0217	1.0396	1.0599	1.0261	1.0765	1.0389	1.0755	1.0369	1.0742	1.0367	1.0758	1.0217	**1.28**
**H(12A-12C)**	0.8777	0.8739	0.8474	0.8694	0.8320	0.8698	0.8377	0.8698	0.8386	0.8674	0.8317	0.8777	0.8739	0.8474	0.8694	0.8321	0.8698	0.8377	0.8698	0.8386	0.8674	0.8317	**0.82**
**H(1`A)**	2.7694	2.7663	2.7435	2.7534	2.7049	2.7542	2.7183	2.7548	2.7196	2.7509	2.6771	2.9218	2.9220	2.8985	2.8920	2.8459	2.8944	2.8559	2.8966	2.8599	2.8879	2.8245	**3.38**
**H(1`B)**	3.2959	3.3429	3.3734	3.3768	3.4303	3.3745	3.4237	3.3719	3.4196	3.3773	3.4149	2.9704	2.8525	2.8394	2.8380	2.8351	2.8379	2.8108	2.8378	2.8160	2.8365	2.8041	**3.71**
**H(2`A)**	1.6240	1.6661	1.6394	1.6783	1.6263	1.6776	1.6507	1.6767	1.6476	1.6772	1.6608	1.5177	1.5624	1.5377	1.5798	1.5247	1.5790	1.5586	1.5779	1.5563	1.5788	1.5749	**2.08**
**H(2`B)**	1.8287	1.8391	1.7947	1.8752	1.8243	1.8721	1.8300	1.8689	1.8242	1.8766	1.8606	1.7568	1.8554	1.8138	1.9233	1.8837	1.9189	1.9008	1.9143	1.8951	1.9259	1.9480	**2.24**
**H(3`A)**	1.5177	1.5624	1.5377	1.5798	1.5247	1.5790	1.5586	1.5779	1.5563	1.5788	1.5749	1.6240	1.6661	1.6394	1.6783	1.6263	1.6776	1.6507	1.6767	1.6475	1.6772	1.6608	**2.08**
**H(3`B)**	1.7568	1.8554	1.8138	1.9233	1.8837	1.9189	1.9008	1.9143	1.8951	1.9259	1.9480	1.8287	1.8391	1.7947	1.8752	1.8243	1.8721	1.8300	1.8689	1.8242	1.8766	1.8606	**2.16**
**H(4`A)**	2.9704	2.8525	2.8394	2.8380	2.8351	2.8379	2.8108	2.8378	2.8160	2.8365	2.8041	3.2959	3.3429	3.3734	3.3768	3.4303	3.3745	3.4237	3.3719	3.4196	3.3773	3.4149	**3.09**
**H(4`B)**	2.9218	2.9219	2.8985	2.8920	2.8459	2.8944	2.8559	2.8966	2.8599	2.8879	2.8245	2.7694	2.7663	2.7435	2.7534	2.7049	2.7542	2.7183	2.7548	2.7196	2.7509	2.6771	**3.68**

[a] methyl protons shifts are averaged
[b] according to [5]

Table S5. Cont. XI

Nuclei	R											S											Exp. [b]
	PBE																						
	Gas	$CHCl_3$ (PCM)	$CHCl_3$ (SMD)	DMSO (PCM)	DMSO (SMD)	CH_3OH (PCM)	CH_3OH (SMD)	C_2H_5OH (PCM)	C_2H_5OH (SMD)	H_2O (PCM)	H_2O (SMD)	Gas	$CHCl_3$ (PCM)	$CHCl_3$ (SMD)	DMSO (PCM)	DMSO (SMD)	CH_3OH (PCM)	CH_3OH (SMD)	C_2H_5OH (PCM)	C_2H_5OH (SMD)	H_2O (PCM)	H_2O (SMD)	
**C1**	135.7483	136.9338	137.8215	137.3803	138.9543	137.3545	138.4508	137.3389	138.4347	137.4219	138.6539	135.7483	136.9338	137.8215	137.3803	138.9543	137.3545	138.4509	137.3389	138.4347	137.4219	138.6539	**132.2**
**C2**	135.8078	135.8743	136.4215	135.6787	136.3342	135.6882	136.8147	135.7088	136.6975	135.6874	136.7796	135.8078	135.8743	136.4215	135.6787	136.3342	135.6882	136.8147	135.7088	136.6975	135.6874	136.7796	**133.3**
**C3**	119.5174	120.3563	120.9316	120.5420	121.3293	120.5297	121.6675	120.5282	121.5814	120.5709	121.7766	119.5174	120.3563	120.9316	120.5420	121.3293	120.5297	121.6674	120.5282	121.5814	120.5709	121.7766	**117.6**
**C4**	174.2361	175.2702	175.9491	175.6389	176.4757	175.6130	177.0574	175.5980	176.9178	175.6810	177.2200	174.2361	175.2703	175.9491	175.6389	176.4758	175.6130	177.0574	175.5980	176.9178	175.6810	177.2200	**168.3**
**C5**	117.5658	118.8904	119.5358	119.4738	120.4435	119.4339	120.7042	119.4048	120.5965	119.5296	120.8999	117.5658	118.8902	119.5358	119.4738	120.4435	119.4339	120.7041	119.4048	120.5965	119.5296	120.8999	**117.6**
**C6**	132.1464	134.1194	135.2182	135.1667	136.8848	135.0939	137.4752	135.0329	137.2528	135.2539	137.9118	132.1464	134.1194	135.2182	135.1667	136.8848	135.0939	137.4754	135.0329	137.2528	135.2539	137.9118	**133.3**
**C7**	201.1356	205.4775	206.7985	207.6645	208.7676	207.5322	212.7870	207.4100	211.8422	207.8073	213.7751	201.1356	205.4775	206.7985	207.6645	208.7676	207.5322	212.7866	207.4100	211.8422	207.8073	213.7751	**195.8**
**C8**	67.3305	68.6977	69.4799	69.1926	70.5026	69.1716	71.0535	69.1589	70.8716	69.2282	71.4612	67.3305	68.6976	69.4799	69.1926	70.5026	69.1716	71.0534	69.1589	70.8716	69.2282	71.4612	**70.4**
**C9**	38.5481	38.3924	38.5854	38.2027	38.7100	38.2115	39.0042	38.2308	38.9069	38.2116	39.1515	38.5481	38.3925	38.5854	38.2027	38.7100	38.2115	39.0042	38.2308	38.9069	38.2116	39.1515	**31.1**
**C10**	29.5828	30.1383	30.5931	30.3952	31.2462	30.3715	31.0588	30.3592	31.0404	30.4359	31.2758	29.5828	30.1382	30.5931	30.3952	31.2462	30.3715	31.0588	30.3592	31.0404	30.4359	31.2759	**26.9**
**C11**	29.4545	29.6678	29.8873	29.5253	30.0700	29.5343	30.0539	29.5536	30.0482	29.5337	30.2277	29.4545	29.6678	29.8873	29.5253	30.0700	29.5343	30.0540	29.5536	30.0482	29.5337	30.2277	**23.4**
**C12**	16.4263	16.6550	16.6777	16.6936	16.8479	16.6853	16.8192	16.6882	16.8147	16.7199	16.8332	16.4263	16.6550	16.6777	16.6936	16.8479	16.6853	16.8192	16.6882	16.8147	16.7199	16.8332	**13.8**
**C1`**	53.1868	54.1192	54.3314	54.5767	55.2085	54.5426	55.1990	54.5200	55.1018	54.6279	55.7537	57.7309	59.0198	59.3593	59.4308	60.0063	59.4053	60.1988	59.3904	60.1340	59.4724	60.4800	**53.3**
**C2`**	25.7091	26.3084	26.6327	26.4566	27.0413	26.4455	27.0629	26.4450	27.0465	26.4846	26.9107	25.3534	25.6633	25.9658	25.7952	26.3210	25.7806	26.3173	25.7773	26.3051	25.8271	26.2369	**24.2**
**C3`**	25.3534	25.6633	25.9658	25.7952	26.3211	25.7806	26.3173	25.7773	26.3051	25.8271	26.2369	25.7091	26.3084	26.6327	26.4566	27.0413	26.4455	27.0629	26.4450	27.0465	26.4846	26.9108	**24.1**
**C4`**	57.7309	59.0198	59.3593	59.4308	60.0063	59.4053	60.1988	59.3904	60.1340	59.4724	60.4800	53.1868	54.1193	54.3314	54.5767	55.2086	54.5426	55.1991	54.5200	55.1018	54.6279	55.7537	**56.3**
**H2**	8.5422	8.4404	8.4543	8.3726	8.3723	8.3764	8.4041	8.3809	8.4061	8.3697	8.3938	8.5422	8.4404	8.4543	8.3726	8.3723	8.3764	8.4041	8.3809	8.4061	8.3697	8.3938	**8.2**
**H3**	7.2025	7.3512	7.4409	7.3933	7.5117	7.3913	7.5367	7.3898	7.5283	7.3957	7.5512	7.2025	7.3512	7.4409	7.3933	7.5117	7.3913	7.5367	7.3898	7.5283	7.3957	7.5512	**7.36**
**H5**	7.0213	7.1777	7.2686	7.2421	7.3835	7.2384	7.3885	7.2352	7.3797	7.2463	7.4165	7.0213	7.1777	7.2686	7.2421	7.3835	7.2384	7.3885	7.2352	7.3797	7.2463	7.4165	**7.36**
**H6**	7.9455	8.1049	8.1784	8.1913	8.3013	8.1856	8.3527	8.1805	8.3388	8.1973	8.3808	7.9455	8.1050	8.1784	8.1913	8.3014	8.1856	8.3527	8.1805	8.3388	8.1973	8.3808	**8.2**
**H8**	4.9981	5.0221	5.0362	5.0495	5.0820	5.0469	5.1116	5.0449	5.1050	5.0527	5.1021	4.9981	5.0221	5.0362	5.0495	5.0820	5.0469	5.1116	5.0449	5.1050	5.0527	5.1021	**5.4**
**H(9A-9B)**	1.8877	1.9213	1.9179	1.9417	1.9768	1.9405	1.9732	1.9398	1.9667	1.9435	2.0076	1.8877	1.9214	1.9179	1.9417	1.9768	1.9405	1.9733	1.9398	1.9667	1.9435	2.0076	**2.08**
**H(10A)**	0.9903	0.8747	0.8363	0.8393	0.7493	0.8399	0.8067	0.8412	0.7986	0.8395	0.8199	0.9903	0.8747	0.8363	0.8393	0.7493	0.8399	0.8068	0.8413	0.7986	0.8395	0.8199	**1.14**
**H(10B)**	1.7513	1.6439	1.5780	1.5587	1.4835	1.5641	1.4701	1.5700	1.4779	1.5544	1.4380	1.7513	1.6439	1.5780	1.5587	1.4835	1.5641	1.4701	1.5700	1.4779	1.5544	1.4380	**1.28**
**H(11A-11B)**	1.1003	1.1160	1.0803	1.1235	1.0909	1.1231	1.0888	1.1233	1.0885	1.1245	1.0766	1.1003	1.1160	1.0803	1.1235	1.0909	1.1231	1.0888	1.1233	1.0885	1.1245	1.0766	**1.28**
**H(12A-12C)**	0.9133	0.9052	0.8805	0.8941	0.8588	0.8947	0.8663	0.8959	0.8665	0.8942	0.8605	0.9133	0.9052	0.8805	0.8941	0.8588	0.8947	0.8663	0.8959	0.8665	0.8942	0.8605	**0.82**
**H(1`A)**	2.7998	2.7946	2.7843	2.7819	2.7480	2.7827	2.7606	2.7840	2.7625	2.7819	2.7267	2.9257	2.9415	2.9313	2.9245	2.9038	2.9262	2.9082	2.9283	2.9120	2.9235	2.8692	**3.38**
**H(1`B)**	3.3750	3.4144	3.4496	3.4414	3.5072	3.4395	3.4925	3.4380	3.4919	3.4439	3.4830	3.0909	2.9057	2.8913	2.8575	2.8578	2.8592	2.8162	2.8617	2.8198	2.8566	2.7942	**3.71**
**H(2`A)**	1.6652	1.7153	1.6902	1.7321	1.6894	1.7312	1.7138	1.7309	1.7096	1.7335	1.7252	1.5339	1.5767	1.5534	1.5973	1.5509	1.5960	1.5860	1.5953	1.5792	1.5991	1.6048	**2.08**
**H(2`B)**	1.9457	1.9507	1.9086	1.9809	1.9365	1.9781	1.9392	1.9759	1.9473	1.9845	1.9691	1.8145	1.9190	1.8748	1.9758	1.9350	1.9721	1.9575	1.9689	1.9473	1.9800	2.0114	**2.24**
**H(3`A)**	1.5339	1.5767	1.5534	1.5973	1.5509	1.5960	1.5860	1.5953	1.5792	1.5991	1.6048	1.6652	1.7153	1.6902	1.7321	1.6894	1.7312	1.7138	1.7309	1.7096	1.7335	1.7252	**2.08**
**H(3`B)**	1.8145	1.9190	1.8748	1.9758	1.9350	1.9721	1.9575	1.9689	1.9473	1.9800	2.0114	1.9457	1.9507	1.9086	1.9809	1.9365	1.9781	1.9392	1.9759	1.9340	1.9845	1.9691	**2.16**
**H(4`A)**	3.0909	2.9057	2.8913	2.8575	2.8578	2.8592	2.8162	2.8617	2.8198	2.8566	2.7942	3.3750	3.4144	3.4496	3.4414	3.5072	3.4395	3.4925	3.4380	3.4919	3.4439	3.4830	**3.09**
**H(4`B)**	2.9257	2.9415	2.9313	2.9245	2.9038	2.9262	2.9082	2.9283	2.9120	2.9235	2.8692	2.7998	2.7946	2.7843	2.7819	2.7480	2.7827	2.7606	2.7840	2.7625	2.7819	2.7267	**3.68**
Nuclei	PBE0																						
**C1**	136.4530	137.3089	138.0508	137.7354	139.2688	137.7148	138.6567	137.6949	138.6719	137.7566	138.8652	136.4530	137.3090	138.0509	137.7354	139.2688	137.7148	138.6567	137.6949	138.6719	137.7566	138.8651	**132.2**
**C2**	138.6013	138.6001	139.0847	138.4264	138.9615	138.4331	139.5038	138.4418	139.3326	138.4230	139.4291	138.6013	138.6001	139.0847	138.4264	138.9613	138.4331	139.5038	138.4418	139.3328	138.4230	139.4292	**133.3**
**C3**	120.9188	121.6798	122.2526	121.8997	122.5990	121.8840	122.9416	121.8699	122.8160	121.9174	122.9678	120.9188	121.6797	122.2525	121.8997	122.5988	121.8840	122.9416	121.8699	122.8159	121.9174	122.9677	**117.6**
**C4**	172.9248	173.7704	174.3607	174.1635	174.7944	174.1342	175.5017	174.1070	175.2844	174.1944	175.5847	172.9248	173.7702	174.3606	174.1635	174.7943	174.1342	175.5017	174.1070	175.2844	174.1944	175.5847	**168.3**
**C5**	118.8873	120.2205	120.8491	120.8784	121.7495	120.8319	122.0378	120.7874	121.8751	120.9256	122.1589	118.8873	120.2204	120.8491	120.8784	121.7495	120.8319	122.0378	120.7874	121.8751	120.9256	122.1590	**117.6**
**C6**	134.7534	136.6483	137.8002	137.7535	139.3337	137.6742	140.0781	137.5975	139.7552	137.8328	140.4055	134.7534	136.6484	137.8002	137.7535	139.3337	137.6742	140.0781	137.5975	139.7551	137.8328	140.4054	**133.3**
**C7**	201.0382	205.4916	206.6635	207.9368	208.6564	207.7907	213.5288	207.6448	212.4021	208.0776	214.5410	201.0382	205.4915	206.6632	207.9368	208.6564	207.7907	213.5288	207.6448	212.4025	208.0776	214.5410	**195.8**
**C8**	63.3741	64.5227	65.2451	65.0149	66.0343	64.9854	66.6890	64.9567	66.5032	65.0447	67.0482	63.3741	64.5227	65.2451	65.0149	66.0342	64.9854	66.6890	64.9567	66.5033	65.0447	67.0483	**70.4**
**C9**	35.2480	35.0267	35.2621	34.8927	35.2211	34.8944	35.4911	34.8977	35.3528	34.8940	35.5658	35.2480	35.0267	35.2622	34.8927	35.2211	34.8944	35.4911	34.8978	35.3528	34.8940	35.5657	**31.1**
**C10**	27.5160	27.9999	28.4469	28.3217	29.0203	28.2908	28.9160	28.2627	28.8347	28.3546	29.0522	27.5160	27.9999	28.4468	28.3217	29.0203	28.2908	28.9160	28.2627	28.8348	28.3546	29.0522	**26.9**
**C11**	27.3762	27.5042	27.7451	27.4397	27.8548	27.4401	27.9245	27.4421	27.8635	27.4419	28.0248	27.3761	27.5043	27.7451	27.4397	27.8548	27.4401	27.9245	27.4422	27.8636	27.4419	28.0247	**23.4**
**C12**	16.1669	16.3506	16.3714	16.4494	16.5174	16.4361	16.5489	16.4251	16.4828	16.4654	16.4981	16.1668	16.3507	16.3713	16.4494	16.5175	16.4361	16.5489	16.4250	16.4827	16.4654	16.4981	**13.8**
**C1`**	50.3442	51.1746	51.3791	51.6553	52.1863	51.6178	52.2537	51.5828	52.1219	51.6945	52.6719	54.6441	55.7362	56.0471	56.1526	56.6508	56.1232	56.8627	56.0957	56.7432	56.1835	57.0525	**53.3**
**C2`**	24.3672	24.7765	25.0807	24.9101	25.3550	24.8969	25.4419	24.8857	25.3814	24.9254	25.2400	23.8126	24.0541	24.3180	24.1905	24.6209	24.1765	24.6494	24.1645	24.5778	24.2070	24.4911	**24.2**
**C3`**	23.8126	24.0541	24.3180	24.1905	24.6209	24.1765	24.6494	24.1645	24.5779	24.2070	24.4910	24.3672	24.7765	25.0807	24.9101	25.3550	24.8969	25.4419	24.8857	25.3814	24.9254	25.2399	**24.1**
**C4`**	54.6441	55.7362	56.0470	56.1526	56.6509	56.1232	56.8627	56.0957	56.7433	56.1835	57.0525	50.3442	51.1747	51.3791	51.6553	52.1864	51.6178	52.2537	51.5828	52.1220	51.6945	52.6719	**56.3**
**H2**	8.7672	8.6747	8.6943	8.6043	8.6001	8.6077	8.6358	8.6113	8.6278	8.6008	8.6154	8.7672	8.6747	8.6943	8.6043	8.6001	8.6077	8.6358	8.6113	8.6278	8.6008	8.6154	**8.2**
**H3**	7.3555	7.5027	7.5909	7.5485	7.6660	7.5463	7.6937	7.5440	7.6820	7.5502	7.7053	7.3555	7.5027	7.5909	7.5485	7.6660	7.5463	7.6937	7.5440	7.6820	7.5502	7.7053	**7.36**
**H5**	7.1793	7.3531	7.4495	7.4232	7.5573	7.4188	7.5698	7.4143	7.5575	7.4270	7.5924	7.1793	7.3531	7.4495	7.4232	7.5573	7.4188	7.5698	7.4143	7.5575	7.4270	7.5924	**7.36**
**H6**	8.0691	8.2434	8.3303	8.3437	8.4667	8.3371	8.5292	8.3305	8.5036	8.3498	8.5531	8.0691	8.2434	8.3303	8.3437	8.4667	8.3371	8.5292	8.3305	8.5036	8.3498	8.5531	**8.2**
**H8**	4.5650	4.6235	4.6513	4.6787	4.7273	4.6747	4.7749	4.6709	4.7514	4.6822	4.7725	4.5650	4.6235	4.6513	4.6787	4.7273	4.6747	4.7749	4.6708	4.7514	4.6822	4.7725	**5.4**
**H(9A-9B)**	1.7345	1.7658	1.7709	1.7908	1.8071	1.7885	1.8131	1.7862	1.8041	1.7927	1.8421	1.7345	1.7658	1.7709	1.7908	1.8071	1.7885	1.8131	1.7862	1.8041	1.7927	1.8421	**2.08**
**H(10A)**	0.9439	0.8046	0.7615	0.7568	0.6649	0.7587	0.9607	0.7606	0.6992	0.7548	0.7188	0.9439	0.8046	0.7615	0.7568	0.6649	0.7587	0.7124	0.7606	0.6992	0.7548	0.7189	**1.14**
**H(10B)**	1.6350	1.5420	1.4670	1.4541	1.3671	1.4600	1.4933	1.4660	1.3682	1.4482	1.3328	1.6350	1.5420	1.4670	1.4541	1.3671	1.4600	1.3654	1.4660	1.3682	1.4482	1.3327	**1.28**
**H(11A-11B)**	1.0056	1.0247	0.9871	1.0334	0.9945	1.0329	0.9994	1.0324	0.9951	1.0336	0.9846	1.0056	1.0247	0.9871	1.0334	0.9945	1.0329	0.9994	1.0324	0.9951	1.0336	0.9846	**1.28**
**H(12A-12C)**	0.8954	0.8818	0.8542	0.8706	0.8300	0.8712	0.8408	0.8719	0.8377	0.8697	0.8328	0.8954	0.8818	0.8542	0.8706	0.8300	0.8712	0.8408	0.8719	0.8377	0.8697	0.8328	**0.82**
**H(1`A)**	2.6626	2.6552	2.6426	2.6448	2.6013	2.6453	2.8989	2.6459	2.6192	2.6440	2.5936	2.8001	2.8064	2.7926	2.7859	2.7548	2.7878	2.7693	2.7895	2.7706	2.7838	2.7342	**3.38**
**H(1`B)**	3.1711	3.2099	3.2462	3.2445	3.3001	3.2418	3.6043	3.2391	3.2874	3.2468	3.2911	2.8887	2.7177	2.7028	2.6786	2.6737	2.6802	2.6333	2.6819	2.6335	2.6768	2.6159	**3.71**
**H(2`A)**	1.6081	1.6522	1.6239	1.6693	1.6181	1.6684	1.8313	1.6674	1.6421	1.6699	1.6551	1.4766	1.5231	1.4941	1.5442	1.4926	1.5431	1.5327	1.5420	1.5241	1.5450	1.5471	**2.08**
**H(2`B)**	1.8001	1.8195	1.7770	1.8622	1.8181	1.8588	2.0106	1.8554	1.8120	1.8653	1.8463	1.6936	1.7941	1.7444	1.8558	1.8158	1.8518	1.8446	1.8478	1.8316	1.8594	1.8910	**2.24**
**H(3`A)**	1.4766	1.5231	1.4941	1.5442	1.4926	1.5431	1.7392	1.5419	1.5241	1.5450	1.5471	1.6081	1.6522	1.6239	1.6693	1.6181	1.6684	1.6479	1.6674	1.6421	1.6699	1.6551	**2.08**
**H(3`B)**	1.6936	1.7941	1.7444	1.8558	1.8158	1.8518	2.0814	1.8478	1.8316	1.8594	1.8910	1.8001	1.8195	1.7770	1.8622	1.8181	1.8588	1.8218	1.8554	1.8120	1.8653	1.8463	**2.16**
**H(4`A)**	2.8887	2.7177	2.7028	2.6786	2.6737	2.6802	2.9914	2.6818	2.6335	2.6768	2.6159	3.1711	3.2099	3.2462	3.2445	3.3001	3.2418	3.2940	3.2391	3.2874	3.2468	3.2911	**3.09**
**H(4`B)**	2.8001	2.8064	2.7926	2.7859	2.7548	2.7878	3.0365	2.7895	2.7706	2.7838	2.7342	2.6626	2.6552	2.6426	2.6448	2.6013	2.6453	2.6219	2.6459	2.6192	2.6440	2.5936	**3.68**

[a] methyl protons shifts are averaged

[b] according to [5]

Table S5. Cont. XII

Nuclei	R											S											Exp. [b]
	TPSS																						
	Gas	$CHCl_3$ (PCM)	$CHCl_3$ (SMD)	DMSO (PCM)	DMSO (SMD)	CH_3OH (PCM)	CH_3OH (SMD)	C_2H_5OH (PCM)	C_2H_5OH (SMD)	H_2O (PCM)	H_2O (SMD)	Gas	$CHCl_3$ (PCM)	$CHCl_3$ (SMD)	DMSO (PCM)	DMSO (SMD)	CH_3OH (PCM)	CH_3OH (SMD)	C_2H_5OH (PCM)	C_2H_5OH (SMD)	H_2O (PCM)	H_2O (SMD)	
**C1**	132.4356	133.1505	133.9345	133.5892	135.0046	133.5760	134.3940	133.5726	134.0790	133.6107	134.7819	132.4356	133.1505	133.9343	133.5892	135.0046	133.5760	134.3940	133.5726	134.6812	133.6107	134.7819	**132.2**
**C2**	132.8929	132.8526	133.4725	132.7671	133.3034	132.7794	133.7783	132.8032	133.3065	132.7661	133.7282	132.8929	132.8526	133.4725	132.7671	133.3034	132.7793	133.7783	132.8032	133.9176	132.7661	133.7282	**133.3**
**C3**	116.6681	117.3246	117.9791	117.5839	118.2462	117.5771	118.5755	117.5815	118.0239	117.6006	118.6358	116.6681	117.3246	117.9791	117.5839	118.2462	117.5771	118.5755	117.5815	118.8101	117.6006	118.6358	**117.6**
**C4**	170.8911	171.7397	172.4965	172.2075	172.9099	172.1863	173.5612	172.1764	172.8372	172.2385	173.6673	170.8911	171.7397	172.4965	172.2075	172.9099	172.1863	173.5612	172.1764	172.9955	172.2385	173.6673	**168.3**
**C5**	114.7722	115.9311	116.6167	116.5690	117.3927	116.5353	117.6393	116.5131	116.9513	116.6120	117.7796	114.7722	115.9311	116.6167	116.5690	117.3927	116.5353	117.6393	116.5131	117.7497	116.6120	117.7796	**117.6**
**C6**	129.3185	131.1397	132.2995	132.2804	133.7885	132.2131	134.5062	132.1577	132.6584	132.3555	134.8302	129.3185	131.1397	132.2995	132.2804	133.7885	132.2131	134.5062	132.1577	133.2770	132.3555	134.8302	**133.3**
**C7**	197.2729	201.1909	202.3750	203.3951	204.2125	203.2728	208.3467	203.1606	203.9454	203.5200	209.4808	197.2729	201.1909	202.3748	203.3951	204.2125	203.2728	208.3467	203.1606	203.7473	203.5200	209.4808	**195.8**
**C8**	66.1554	67.2219	67.8668	67.7200	68.8092	67.7027	69.2644	67.6953	67.9382	67.7454	69.7480	66.1554	67.2219	67.8668	67.7200	68.8092	67.7027	69.2644	67.6953	69.2980	67.7454	69.7480	**70.4**
**C9**	37.7531	37.5421	37.8061	37.4640	37.8295	37.4741	38.1342	37.4954	37.6175	37.4651	38.2427	37.7531	37.5421	37.8059	37.4640	37.8295	37.4740	38.1342	37.4954	39.3246	37.4651	38.2427	**31.1**
**C10**	29.1817	29.5893	30.1503	29.9117	30.6464	29.8939	30.4886	29.8881	29.9798	29.9393	30.6120	29.1817	29.5893	30.1502	29.9117	30.6464	29.8939	30.4886	29.8881	31.7744	29.9393	30.6120	**26.9**
**C11**	29.3660	29.4429	29.7794	29.4146	29.8735	29.4257	29.8732	29.4479	29.5378	29.4144	30.0060	29.3660	29.4429	29.7794	29.4146	29.8736	29.4258	29.8732	29.4479	31.3375	29.4144	30.0060	**23.4**
**C12**	17.2694	17.3881	17.5648	17.5303	17.6714	17.5262	17.6562	17.5339	17.5761	17.5450	17.6292	17.2694	17.3881	17.5648	17.5303	17.6714	17.5262	17.6562	17.5339	19.5128	17.5450	17.6292	**13.8**
**C1`**	53.0445	53.7558	54.1667	54.3103	54.8418	54.2794	54.8722	54.2605	54.4497	54.3511	55.3404	57.2249	58.3026	58.7280	58.7950	59.3005	58.7753	59.5194	58.7666	60.4363	58.8238	59.7915	**53.3**
**C2`**	26.3341	26.7286	27.1499	26.9392	27.4229	26.9349	27.4595	26.9415	27.0214	26.9534	27.2605	26.0765	26.1994	26.6761	26.4209	26.8925	26.4135	26.9030	26.4175	28.3298	26.4384	26.7317	**24.2**
**C3`**	26.0765	26.1994	26.6761	26.4209	26.8925	26.4136	26.9030	26.4175	26.4953	26.4384	26.7317	26.3341	26.7286	27.1499	26.9392	27.4229	26.9349	27.4595	26.9415	28.8499	26.9534	27.2605	**24.1**
**C4`**	57.2249	58.3026	58.7280	58.7950	59.3005	58.7753	59.5194	58.7666	58.9738	58.8238	59.7915	53.0445	53.7558	54.1668	54.3103	54.8419	54.2794	54.8722	54.2605	55.9640	54.3511	55.3404	**56.3**
**H2**	8.5524	8.4665	8.5107	8.4102	8.4107	8.4132	8.4523	8.4170	8.2571	8.4079	8.4133	8.5524	8.4665	8.5107	8.4102	8.4107	8.4132	8.4523	8.4170	8.5271	8.4079	8.4133	**8.2**
**H3**	7.1623	7.3069	7.4039	7.3576	7.4767	7.3553	7.5043	7.3535	7.1893	7.3601	7.5144	7.1623	7.3069	7.4039	7.3576	7.4767	7.3553	7.5043	7.3535	7.4716	7.3601	7.5144	**7.36**
**H5**	6.9935	7.1616	7.2589	7.2313	7.3713	7.2271	7.3755	7.2236	7.0589	7.2357	7.3952	6.9935	7.1616	7.2589	7.2313	7.3713	7.2271	7.3755	7.2236	7.3426	7.2357	7.3952	**7.36**
**H6**	7.8543	8.0239	8.1241	8.1246	8.2413	8.1183	8.3087	8.1126	7.9514	8.1310	8.3186	7.8543	8.0239	8.1241	8.1246	8.2413	8.1183	8.3087	8.1126	8.2250	8.1310	8.3186	**8.2**
**H8**	4.7647	4.8126	4.8538	4.8581	4.9078	4.8551	4.9504	4.8528	4.6786	4.8614	4.9315	4.7647	4.8126	4.8538	4.8581	4.9078	4.8551	4.9504	4.8528	4.9896	4.8614	4.9315	**5.4**
**H(9A-9B)**	1.8001	1.8427	1.8422	1.8732	1.9021	1.8708	1.9032	1.8691	1.6830	1.8759	1.9251	1.8001	1.8427	1.8422	1.8732	1.9021	1.8708	1.9032	1.8691	2.0283	1.8759	1.9251	**2.08**
**H(10A)**	1.0002	0.8769	0.8927	0.8464	0.7648	0.8472	0.8274	0.8487	0.6585	0.8461	0.8190	1.0002	0.8769	0.8927	0.8464	0.7648	0.8472	0.8274	0.8487	1.0155	0.8461	0.8190	**1.14**
**H(10B)**	1.7385	1.6228	1.5525	1.5379	1.4588	1.5440	1.4467	1.5505	1.3631	1.5325	1.4054	1.7385	1.6228	1.5525	1.5379	1.4588	1.5440	1.4467	1.5505	1.9732	1.5325	1.4054	**1.28**
**H(11A-11B)**	1.0583	1.0759	1.0492	1.0919	1.0592	1.0911	1.0615	1.0909	0.9017	1.0929	1.0424	1.0583	1.0759	1.0492	1.0919	1.0592	1.0911	1.0615	1.0909	1.2559	1.0929	1.0424	**1.28**
**H(12A-12C)**	0.9078	0.8948	0.8800	0.8900	0.8562	0.8904	0.8645	0.8914	0.7014	0.8900	0.8548	0.9078	0.8948	0.8800	0.8900	0.8562	0.8904	0.8645	0.8914	1.0579	0.8900	0.8548	**0.82**
**H(1`A)**	2.7122	2.7112	2.7093	2.7074	2.6743	2.7077	2.6920	2.7086	2.5258	2.7075	2.6628	2.8273	2.8487	2.8382	2.8410	2.8258	2.8424	2.8307	2.8444	3.0399	2.8398	2.7945	**3.38**
**H(1`B)**	3.2745	3.3206	3.3526	3.3541	3.4150	3.3518	3.4072	3.3500	3.1698	3.3568	3.3950	3.0710	2.8744	2.8447	2.8264	2.8319	2.8280	2.7779	2.8303	2.9220	2.8255	2.7603	**3.71**
**H(2`A)**	1.6509	1.6969	1.6781	1.7203	1.6753	1.7191	1.6999	1.7184	1.5317	1.7219	1.7093	1.5223	1.5627	1.5474	1.5881	1.5405	1.5866	1.5738	1.5858	1.9477	1.5899	1.5867	**2.08**
**H(2`B)**	1.8625	1.8784	1.8460	1.9212	1.8838	1.9176	1.8876	1.9149	1.7290	1.9252	1.9113	1.7015	1.8125	1.7743	1.8818	1.8445	1.8776	1.8662	1.8740	2.5013	1.8862	1.9287	**2.24**
**H(3`A)**	1.5223	1.5627	1.5474	1.5881	1.5405	1.5866	1.5738	1.5858	1.3985	1.5899	1.5867	1.6509	1.6969	1.6781	1.7203	1.6753	1.7191	1.6999	1.7184	2.0894	1.7219	1.7093	**2.08**
**H(3`B)**	1.7015	1.8125	1.7743	1.8818	1.8445	1.8776	1.8662	1.8740	1.6879	1.8862	1.9287	1.8625	1.8784	1.8460	1.9212	1.8838	1.9176	1.8876	1.9149	2.0859	1.9252	1.9113	**2.16**
**H(4`A)**	3.0710	2.8744	2.8447	2.8264	2.8318	2.8280	2.7779	2.8303	2.6480	2.8255	2.7603	3.2745	3.3206	3.3526	3.3541	3.4150	3.3518	3.4072	3.3500	3.4375	3.3568	3.3950	**3.09**
**H(4`B)**	2.8273	2.8487	2.8382	2.8410	2.8258	2.8424	2.8307	2.8444	2.6622	2.8398	2.7945	2.7122	2.7112	2.7093	2.7074	2.6743	2.7077	2.6920	2.7086	2.7416	2.7075	2.6628	**3.68**
Nuclei	VSXC																						
**C1**	133.6805	134.5405	135.1885	134.8465	136.5075	134.8331	135.4592	134.8176	135.4598	134.8603	135.9108	133.6804	134.5405	135.1885	134.8465	136.5076	134.8331	135.4593	134.8176	135.4596	134.8603	135.9108	**132.2**
**C2**	130.8569	131.2148	131.6668	131.2177	131.8000	131.2250	132.3394	131.2296	132.2150	131.2117	132.4206	130.8567	131.2148	131.6668	131.2177	131.8000	131.2250	132.3394	131.2296	132.2150	131.2117	132.4206	**133.3**
**C3**	114.9211	115.9128	116.4365	116.3181	116.9073	116.2991	117.2645	116.2774	117.2025	116.3375	117.3624	114.9211	115.9128	116.4365	116.3181	116.9072	116.2991	117.2645	116.2774	117.2024	116.3375	117.3624	**117.6**
**C4**	168.3003	169.1032	169.7919	169.5485	170.2398	169.5239	170.8109	169.4971	170.6743	169.5735	170.9192	168.3003	169.1032	169.7919	169.5485	170.2398	169.5239	170.8108	169.4971	170.6743	169.5735	170.9192	**168.3**
**C5**	112.7729	114.1638	114.7218	114.9628	115.5777	114.9149	115.9637	114.8652	115.8581	115.0102	116.1017	112.7730	114.1638	114.7218	114.9628	115.5778	114.9149	115.9637	114.8652	115.8581	115.0102	116.1017	**117.6**
**C6**	128.4606	129.9982	131.1249	130.9894	132.3974	130.9269	132.9648	130.8631	132.7529	131.0511	133.1730	128.4606	129.9982	131.1249	130.9894	132.3975	130.9269	132.9647	130.8631	132.7528	131.0511	133.1730	**133.3**
**C7**	193.6144	198.7380	199.9101	201.2950	202.2459	201.1444	206.3068	200.9911	205.2665	201.4392	207.5617	193.6144	198.7380	199.9101	201.2950	202.2458	201.1444	206.3068	200.9911	205.2666	201.4392	207.5617	**195.8**
**C8**	71.2958	72.3979	72.9108	72.9017	73.7341	72.8757	74.0459	72.8472	73.8255	72.9277	74.6281	71.2958	72.3979	72.9108	72.9018	73.7341	72.8757	74.0459	72.8472	73.8255	72.9277	74.6281	**70.4**
**C9**	41.7566	42.2479	42.5811	42.4292	42.7524	42.4240	43.2429	42.4162	43.1046	42.4354	43.3121	41.7566	42.2479	42.5811	42.4293	42.7525	42.4240	43.2429	42.4162	43.1046	42.4354	43.3121	**31.1**
**C10**	36.4853	37.4189	37.7243	38.0913	38.8133	38.0483	38.7335	38.0038	38.7135	38.1344	39.0207	36.4853	37.4189	37.7243	38.0913	38.8133	38.0483	38.7334	38.0038	38.7134	38.1344	39.0207	**26.9**
**C11**	35.7245	35.7671	36.0142	35.6725	36.0233	35.6843	35.9517	35.6935	35.9513	35.6625	35.9686	35.7245	35.7671	36.0142	35.6725	36.0233	35.6843	35.9516	35.6935	35.9513	35.6625	35.9686	**23.4**
**C12**	18.7135	18.9231	19.0967	19.1393	19.3695	19.1260	19.2353	19.1108	19.2302	19.1539	19.2772	18.7135	18.9231	19.0967	19.1393	19.3695	19.1260	19.2353	19.1108	19.2301	19.1539	19.2772	**13.8**
**C1`**	58.8714	59.4981	59.7451	59.8519	60.3179	59.8335	60.1483	59.8130	60.1663	59.8709	60.3243	60.9536	61.9037	62.1420	62.3605	62.9177	62.3370	62.8341	62.3113	62.7698	62.3842	63.2050	**53.3**
**C2`**	29.5342	30.0310	30.1911	30.2855	30.6314	30.2737	30.6152	30.2596	30.5864	30.2979	30.6852	28.4893	28.8029	29.0331	29.0094	29.3917	28.9983	29.3626	28.9854	29.3504	29.0216	29.4075	**24.2**
**C3`**	28.4892	28.8029	29.0331	29.0094	29.3918	28.9983	29.3625	28.9854	29.3504	29.0216	29.4075	29.5341	30.0310	30.1911	30.2855	30.6313	30.2737	30.6153	30.2596	30.5863	30.2979	30.6852	**24.1**
**C4`**	60.9537	61.9037	62.1420	62.3605	62.9176	62.3370	62.8341	62.3113	62.7699	62.3842	63.2050	58.8714	59.4981	59.7451	59.8519	60.3180	59.8335	60.1482	59.8130	60.1664	59.8710	60.3243	**56.3**
**H2**	8.5227	8.5081	8.5164	8.4735	8.4550	8.4764	8.5360	8.4794	8.5408	8.4706	8.5151	8.5227	8.5081	8.5164	8.4735	8.4550	8.4764	8.5360	8.4794	8.5408	8.4706	8.5151	**8.2**
**H3**	7.1829	7.3512	7.4296	7.4126	7.5247	7.4094	7.5501	7.4062	7.5412	7.4155	7.5801	7.1829	7.3512	7.4296	7.4126	7.5247	7.4094	7.5501	7.4062	7.5412	7.4155	7.5801	**7.36**
**H5**	7.0781	7.2694	7.3540	7.3564	7.4727	7.3513	7.4935	7.3463	7.4820	7.3612	7.5113	7.0781	7.2694	7.3540	7.3564	7.4727	7.3513	7.4935	7.3463	7.4820	7.3612	7.5113	**7.36**
**H6**	7.8503	8.0072	8.0875	8.1024	8.2216	8.0962	8.2794	8.0902	8.2620	8.1082	8.3027	7.8503	8.0072	8.0875	8.1024	8.2216	8.0962	8.2794	8.0902	8.2620	8.1082	8.3027	**8.2**
**H8**	4.2573	4.4037	4.4274	4.4919	4.5768	4.4861	4.6320	4.4805	4.6030	4.4974	4.6706	4.2573	4.4037	4.4274	4.4919	4.5768	4.4861	4.6320	4.4805	4.6030	4.4974	4.6706	**5.4**
**H(9A-9B)**	1.9641	1.9764	1.9714	1.9883	1.9833	1.9875	1.9808	1.9869	1.9785	1.9889	1.9808	1.9641	1.9764	1.9714	1.9883	1.9833	1.9875	1.9808	1.9869	1.9785	1.9889	1.9808	**2.08**
**H(10A)**	0.9306	0.9326	0.9128	0.9285	0.9053	0.9291	0.8940	0.9297	0.8921	0.9279	0.8858	0.9306	0.9326	0.9128	0.9285	0.9053	0.9291	0.8940	0.9297	0.8921	0.9279	0.8858	**1.14**
**H(10B)**	2.2764	2.1111	2.0177	1.9949	1.8578	2.0028	1.8386	2.0106	1.8498	1.9873	1.7818	2.2764	2.1111	2.0177	1.9949	1.8578	2.0028	1.8386	2.0106	1.8498	1.9873	1.7818	**1.28**
**H(11A-11B)**	1.3715	1.3906	1.3742	1.4051	1.3862	1.4042	1.3808	1.4034	1.3793	1.4059	1.3754	1.3715	1.3906	1.3742	1.4051	1.3862	1.4042	1.3808	1.4034	1.3793	1.4059	1.3754	**1.28**
**H(12A-12C)**	1.0159	1.0221	0.9916	1.0217	0.9772	1.0218	0.9913	1.0219	0.9921	1.0216	0.9943	1.0159	1.0221	0.9916	1.0217	0.9772	1.0218	0.9914	1.0219	0.9921	1.0216	0.9943	**0.82**
**H(1`A)**	2.5466	2.6087	2.6123	2.6247	2.6165	2.6242	2.6238	2.6237	2.6182	2.6251	2.6253	2.9068	2.9249	2.9160	2.9333	2.9175	2.9330	2.9201	2.9327	2.9165	2.9335	2.9377	**3.38**
**H(1`B)**	3.2222	3.2385	3.2758	3.2572	3.3242	3.2559	3.3194	3.2547	3.3141	3.2584	3.3295	2.7958	2.7872	2.7812	2.8016	2.8140	2.8006	2.8086	2.7996	2.7986	2.8025	2.8470	**3.71**
**H(2`A)**	1.9140	1.9742	1.9544	1.9977	1.9460	1.9965	1.9671	1.9954	1.9660	1.9988	1.9528	1.7327	1.8117	1.7846	1.8479	1.7903	1.8458	1.8302	1.8439	1.8243	1.8498	1.8487	**2.08**
**H(2`B)**	1.9226	1.9364	1.9229	1.9674	1.9573	1.9650	1.9672	1.9627	1.9625	1.9696	1.9907	2.3326	2.3738	2.3431	2.4091	2.3794	2.4065	2.3820	2.4042	2.3779	2.4115	2.4139	**2.24**
**H(3`A)**	1.7327	1.8117	1.7846	1.8479	1.7903	1.8458	1.8301	1.8439	1.8243	1.8498	1.8487	1.9140	1.9742	1.9544	1.9977	1.9460	1.9965	1.9671	1.9954	1.9660	1.9988	1.9528	**2.08**
**H(3`B)**	2.3325	2.3738	2.3431	2.4091	2.3793	2.4065	2.3820	2.4042	2.3779	2.4115	2.4139	1.9226	1.9364	1.9229	1.9674	1.9573	1.9650	1.9672	1.9627	1.9625	1.9697	1.9907	**2.16**
**H(4`A)**	2.7958	2.7872	2.7812	2.8016	2.8140	2.8006	2.8086	2.7996	2.7986	2.8025	2.8470	3.2222	3.2385	3.2758	3.2572	3.3242	3.2559	3.3194	3.2547	3.3141	3.2584	3.3295	**3.09**
**H(4`B)**	2.9068	2.9249	2.9160	2.9333	2.9174	2.9330	2.9201	2.9327	2.9165	2.9335	2.9377	2.5466	2.6087	2.6123	2.6247	2.6165	2.6242	2.6238	2.6237	2.6182	2.6251	2.6253	**3.68**

[a] methyl protons shifts are averaged

[b] according to [5]

Table S5. Cont. XIII

Nuclei	R											S											Exp. [b]
	wB97																						
	Gas	$CHCl_3$ (PCM)	$CHCl_3$ (SMD)	DMSO (PCM)	DMSO (SMD)	CH_3OH (PCM)	CH_3OH (SMD)	C_2H_5OH (PCM)	C_2H_5OH (SMD)	H_2O (PCM)	H_2O (SMD)	Gas	$CHCl_3$ (PCM)	$CHCl_3$ (SMD)	DMSO (PCM)	DMSO (SMD)	CH_3OH (PCM)	CH_3OH (SMD)	C_2H_5OH (PCM)	C_2H_5OH (SMD)	H_2O (PCM)	H_2O (SMD)	
**C1**	136.6036	137.2931	138.0720	137.6679	139.4784	137.6596	138.7685	137.6458	138.8630	137.6709	139.1689	136.6037	137.2932	138.0720	137.6679	139.4785	137.6598	138.7686	137.6458	138.8630	137.6709	139.1689	**132.2**
**C2**	140.3841	140.2277	140.7307	140.0851	140.5886	140.0918	141.0437	140.0984	140.9507	140.0798	141.0312	140.3840	140.2279	140.7307	140.0850	140.5889	140.0917	141.0438	140.0984	140.9506	140.0796	141.0312	**133.3**
**C3**	121.6019	122.1976	122.7769	122.3867	123.0830	122.3768	123.2214	122.3659	123.1771	122.3963	123.2651	121.6019	122.1976	122.7769	122.3867	123.0830	122.3768	123.2213	122.3659	123.1771	122.3961	123.2650	**117.6**
**C4**	174.2312	174.8327	175.4522	175.1881	175.7881	175.1626	176.3324	175.1372	176.1882	175.2135	176.4253	174.2312	174.8326	175.4522	175.1882	175.7881	175.1627	176.3322	175.1372	176.1882	175.2133	176.4254	**168.3**
**C5**	119.5886	120.8444	121.4620	121.5301	122.3881	121.4874	122.5264	121.4436	122.4326	121.5708	122.6707	119.5886	120.8442	121.4620	121.5301	122.3881	121.4873	122.5263	121.4436	122.4326	121.5708	122.6707	**117.6**
**C6**	136.6592	138.3616	139.5017	139.3874	140.9361	139.3158	141.4313	139.2458	141.1605	139.4573	141.7769	136.6593	138.3615	139.5017	139.3873	140.9360	139.3158	141.4313	139.2458	141.1605	139.4574	141.7769	**133.3**
**C7**	201.4930	206.2813	207.4612	209.0200	209.7424	208.8666	215.3336	208.7073	214.1389	209.1604	216.6162	201.4932	206.2813	207.4612	209.0200	209.7424	208.8665	215.3336	208.7073	214.1389	209.1603	216.6162	**195.8**
**C8**	61.8488	62.9799	63.5330	63.4907	64.4664	63.4706	65.0895	63.4470	64.9281	63.5072	65.6139	61.8488	62.9799	63.5330	63.4908	64.4665	63.4705	65.0895	63.4470	64.9281	63.5073	65.6139	**70.4**
**C9**	33.4710	33.2416	33.4528	33.1389	33.4830	33.1466	33.5747	33.1536	33.4993	33.1317	33.7715	33.4709	33.2417	33.4528	33.1390	33.4830	33.1467	33.5747	33.1536	33.4993	33.1318	33.7715	**31.1**
**C10**	27.2566	27.6512	28.0056	27.9994	28.6490	27.9736	28.4332	27.9477	28.3999	28.0248	28.6428	27.2566	27.6512	28.0056	27.9993	28.6490	27.9737	28.4333	27.9477	28.3999	28.0248	28.6428	**26.9**
**C11**	26.2445	26.3038	26.5962	26.2779	26.6993	26.2792	26.6545	26.2801	26.6480	26.2768	26.8119	26.2446	26.3039	26.5962	26.2778	26.6993	26.2793	26.6544	26.2801	26.6480	26.2768	26.8119	**23.4**
**C12**	16.0982	16.1941	16.2592	16.3226	16.4257	16.3108	16.3515	16.2989	16.3401	16.3345	16.3725	16.0982	16.1942	16.2592	16.3225	16.4258	16.3108	16.3514	16.2989	16.3401	16.3346	16.3725	**13.8**
**C1`**	49.2568	49.9126	50.1155	50.3454	50.8618	50.3138	50.7647	50.2823	50.7307	50.3765	51.1612	53.0280	53.8362	54.1787	54.2424	54.7458	54.2152	54.7838	54.1877	54.7368	54.2686	54.9485	**53.3**
**C2`**	23.8426	24.1307	24.3776	24.2804	24.7127	24.2708	24.6901	24.2604	24.6738	24.2897	24.5996	23.1244	23.2955	23.5764	23.4577	23.9434	23.4448	23.8672	23.4312	23.8527	23.4704	23.8171	**24.2**
**C3`**	23.1243	23.2956	23.5764	23.4576	23.9434	23.4448	23.8671	23.4312	23.8527	23.4704	23.8171	23.8426	24.1307	24.3776	24.2804	24.7128	24.2708	24.6899	24.2604	24.6738	24.2898	24.5997	**24.1**
**C4`**	53.0280	53.8363	54.1787	54.2423	54.7459	54.2152	54.7840	54.1877	54.7368	54.2686	54.9485	49.2568	49.9127	50.1155	50.3454	50.8617	50.3139	50.7647	50.2823	50.7307	50.3765	51.1611	**56.3**
**H2**	8.8611	8.7550	8.7824	8.6884	8.6845	8.6907	8.6892	8.6933	8.6908	8.6868	8.6627	8.8611	8.7551	8.7824	8.6884	8.6845	8.6907	8.6892	8.6933	8.6908	8.6868	8.6628	**8.2**
**H3**	7.4418	7.5755	7.6657	7.6151	7.7392	7.6130	7.7548	7.6108	7.7474	7.6170	7.7687	7.4418	7.5755	7.6657	7.6151	7.7392	7.6130	7.7548	7.6108	7.7474	7.6170	7.7687	**7.36**
**H5**	7.2881	7.4745	7.5659	7.5546	7.6853	7.5499	7.6986	7.5450	7.6874	7.5592	7.7204	7.2881	7.4745	7.5659	7.5546	7.6853	7.5499	7.6986	7.5450	7.6874	7.5592	7.7204	**7.36**
**H6**	8.1472	8.3095	8.4115	8.4085	8.5457	8.4013	8.5816	8.3942	8.5608	8.4158	8.6004	8.1472	8.3095	8.4115	8.4085	8.5457	8.4013	8.5816	8.3942	8.5608	8.4158	8.6004	**8.2**
**H8**	4.2162	4.2825	4.3391	4.3494	4.4217	4.3438	4.4547	4.3384	4.4370	4.3551	4.4428	4.2162	4.2825	4.3391	4.3494	4.4217	4.3438	4.4547	4.3384	4.4370	4.3551	4.4427	**5.4**
**H(9A-9B)**	1.6980	1.7347	1.7280	1.7573	1.7680	1.7556	1.7679	1.7541	1.7590	1.7591	1.8079	1.6980	1.7347	1.7280	1.7573	1.7680	1.7556	1.7679	1.7541	1.7590	1.7591	1.8079	**2.08**
**H(10A)**	1.0016	0.8194	0.8010	0.7626	0.6910	0.7642	0.7142	0.7660	0.7163	0.7614	0.7212	1.0016	0.8194	0.8010	0.7625	0.6910	0.7642	0.7142	0.7660	0.7163	0.7614	0.7213	**1.14**
**H(10B)**	1.6278	1.5347	1.4753	1.4482	1.3716	1.4533	1.3682	1.4583	1.3751	1.4435	1.3347	1.6278	1.5347	1.4753	1.4482	1.3716	1.4533	1.3682	1.4583	1.3751	1.4435	1.3347	**1.28**
**H(11A-11B)**	0.9970	1.0164	0.9858	1.0253	0.9913	1.0240	0.9842	1.0229	0.9848	1.0267	0.9701	0.9970	1.0165	0.9858	1.0253	0.9913	1.0240	0.9842	1.0229	0.9848	1.0267	0.9701	**1.28**
**H(12A-12C)**	0.9260	0.9059	0.8807	0.8925	0.8533	0.8928	0.8544	0.8932	0.8560	0.8923	0.8453	0.9260	0.9059	0.8807	0.8925	0.8533	0.8928	0.8544	0.8932	0.8560	0.8923	0.8453	**0.82**
**H(1`A)**	2.6270	2.6255	2.6087	2.6149	2.5688	2.6154	2.5814	2.6159	2.5810	2.6146	2.5637	2.7756	2.7821	2.7653	2.7661	2.7415	2.7679	2.7612	2.7694	2.7614	2.7644	2.7326	**3.38**
**H(1`B)**	3.0717	3.1071	3.1361	3.1419	3.1899	3.1392	3.1745	3.1364	3.1704	3.1448	3.1707	2.7440	2.6065	2.5843	2.5750	2.5724	2.5770	2.5537	2.5787	2.5505	2.5729	2.5550	**3.71**
**H(2`A)**	1.6256	1.6765	1.6449	1.6937	1.6440	1.6928	1.6664	1.6917	1.6634	1.6947	1.6777	1.4958	1.5538	1.5251	1.5761	1.5317	1.5750	1.5665	1.5737	1.5625	1.5771	1.5832	**2.08**
**H(2`B)**	1.7472	1.7645	1.7289	1.8078	1.7723	1.8040	1.7693	1.8003	1.7626	1.8116	1.7916	1.6773	1.7645	1.7144	1.8250	1.7982	1.8210	1.8169	1.8169	1.8105	1.8288	1.8581	**2.24**
**H(3`A)**	1.4957	1.5538	1.5251	1.5761	1.5317	1.5750	1.5665	1.5737	1.5625	1.5771	1.5832	1.6256	1.6765	1.6449	1.6937	1.6440	1.6928	1.6664	1.6917	1.6634	1.6947	1.6777	**2.08**
**H(3`B)**	1.6772	1.7645	1.7144	1.8250	1.7982	1.8210	1.8169	1.8169	1.8105	1.8288	1.8581	1.7472	1.7645	1.7289	1.8078	1.7723	1.8040	1.7693	1.8003	1.7626	1.8116	1.7916	**2.16**
**H(4`A)**	2.7440	2.6065	2.5843	2.5750	2.5724	2.5770	2.5537	2.5787	2.5505	2.5729	2.5550	3.0717	3.1071	3.1361	3.1419	3.1899	3.1392	3.1744	3.1364	3.1704	3.1448	3.1707	**3.09**
**H(4`B)**	2.7756	2.7821	2.7653	2.7661	2.7415	2.7679	2.7612	2.7694	2.7614	2.7644	2.7326	2.6270	2.6255	2.6087	2.6149	2.5688	2.6155	2.5814	2.6159	2.5810	2.6146	2.5637	**3.68**
Nuclei	wB97X																						
**C1**	138.1726	138.9019	139.6559	139.4374	141.1483	139.4362	140.2893	139.4298	140.5386	139.4351	140.5881	138.1726	138.9020	139.6559	139.4374	141.1483	139.4362	140.2892	139.4298	140.5386	139.4351	140.5881	**132.2**
**C2**	140.9056	140.7859	141.2893	140.6233	141.1540	140.6326	141.6628	140.6415	141.4810	140.6157	141.6112	140.9055	140.7861	141.2893	140.6233	141.1540	140.6326	141.6628	140.6415	141.4810	140.6157	141.6112	**133.3**
**C3**	122.3191	122.9195	123.4907	123.1123	123.8008	123.1045	123.9961	123.0956	123.8881	123.1203	124.0304	122.3191	122.9193	123.4908	123.1123	123.8008	123.1045	123.9961	123.0956	123.8881	123.1203	124.0304	**117.6**
**C4**	174.9105	175.5432	176.1569	175.8933	176.4990	175.8689	177.1226	175.8445	176.8649	175.9183	177.2107	174.9104	175.5433	176.1570	175.8933	176.4990	175.8689	177.1226	175.8446	176.8649	175.9183	177.2107	**168.3**
**C5**	120.2722	121.5580	122.1552	122.2785	123.1172	122.2378	123.3127	122.1963	123.1518	122.3181	123.4476	120.2722	121.5580	122.1551	122.2785	123.1172	122.2378	123.3127	122.1963	123.1518	122.3181	123.4476	**117.6**
**C6**	136.9982	138.7129	139.8801	139.7133	141.2649	139.6414	141.8678	139.5704	141.4715	139.7845	142.2578	136.9982	138.7132	139.8800	139.7133	141.2649	139.6414	141.8678	139.5703	141.4715	139.7845	142.2578	**133.3**
**C7**	203.1280	207.9657	209.1246	210.8225	211.4559	210.6803	216.7975	210.5330	215.8333	210.9532	218.0343	203.1280	207.9653	209.1247	210.8225	211.4559	210.6803	216.7976	210.5330	215.8333	210.9532	218.0343	**195.8**
**C8**	63.1594	64.2444	64.8483	64.8139	65.7904	64.8024	66.3526	64.7861	66.3173	64.8222	66.9049	63.1594	64.2444	64.8484	64.8139	65.7904	64.8024	66.3525	64.7861	66.3173	64.8222	66.9049	**70.4**
**C9**	34.2867	33.9981	34.2392	33.8733	34.2116	33.8838	34.2855	33.8926	34.2464	33.8639	34.5266	34.2868	33.9981	34.2391	33.8733	34.2116	33.8838	34.2855	33.8926	34.2464	33.8639	34.5266	**31.1**
**C10**	27.6239	28.0338	28.4350	28.3754	29.0463	28.3517	28.8760	28.3279	28.7824	28.3996	29.0595	27.6239	28.0339	28.4349	28.3754	29.0463	28.3517	28.8759	28.3278	28.7824	28.3996	29.0595	**26.9**
**C11**	26.7734	26.8395	27.1217	26.8101	27.2443	26.8150	27.2454	26.8190	27.1824	26.8061	27.3901	26.7733	26.8395	27.1217	26.8101	27.2443	26.8150	27.2453	26.8190	27.1824	26.8061	27.3901	**23.4**
**C12**	16.1134	16.2233	16.2803	16.3492	16.4455	16.3394	16.4133	16.3295	16.3307	16.3601	16.4150	16.1135	16.2233	16.2803	16.3492	16.4455	16.3394	16.4133	16.3295	16.3307	16.3601	16.4150	**13.8**
**C1`**	49.9786	50.6789	50.8740	51.1549	51.6976	51.1232	51.6534	51.0913	51.5542	51.1870	52.0498	54.0121	54.8870	55.2128	55.3169	55.7989	55.2921	55.9370	55.2667	55.7988	55.3418	56.0923	**53.3**
**C2`**	24.3147	24.5823	24.8496	24.7428	25.1842	24.7362	25.2016	24.7285	25.1548	24.7501	25.0887	23.6105	23.7548	24.0311	23.9209	24.4101	23.9100	24.3738	23.8985	24.2790	23.9323	24.2815	**24.2**
**C3`**	23.6105	23.7548	24.0310	23.9209	24.4101	23.9100	24.3738	23.8985	24.2790	23.9323	24.2815	24.3147	24.5823	24.8496	24.7428	25.1842	24.7362	25.2015	24.7285	25.1548	24.7501	25.0887	**24.1**
**C4`**	54.0122	54.8869	55.2127	55.3169	55.7989	55.2921	55.9370	55.2667	55.7988	55.3418	56.0923	49.9785	50.6788	50.8741	51.1549	51.6976	51.1232	51.6535	51.0913	51.5542	51.1870	52.0498	**56.3**
**H2**	8.8389	8.7344	8.7644	8.6467	8.6499	8.6486	8.6801	8.6509	8.6532	8.6456	8.6538	8.8389	8.7344	8.7644	8.6467	8.6499	8.6486	8.6801	8.6509	8.6532	8.6456	8.6538	**8.2**
**H3**	7.4189	7.5552	7.6448	7.5956	7.7180	7.5936	7.7363	7.5916	7.7257	7.5975	7.7486	7.4189	7.5552	7.6448	7.5956	7.7180	7.5936	7.7363	7.5916	7.7257	7.5975	7.7486	**7.36**
**H5**	7.2591	7.4494	7.5388	7.5328	7.6613	7.5281	7.6770	7.5234	7.6630	7.5374	7.6981	7.2591	7.4494	7.5388	7.5328	7.6613	7.5281	7.6770	7.5234	7.6630	7.5374	7.6981	**7.36**
**H6**	8.1258	8.2888	8.3898	8.3787	8.5117	8.3714	8.5602	8.3643	8.5198	8.3862	8.5805	8.1258	8.2888	8.3898	8.3787	8.5117	8.3714	8.5602	8.3643	8.5198	8.3862	8.5805	**8.2**
**H8**	4.3096	4.3744	4.4229	4.4341	4.4965	4.4284	4.5479	4.4231	4.5054	4.4401	4.5323	4.3096	4.3744	4.4229	4.4341	4.4965	4.4284	4.5479	4.4231	4.5054	4.4401	4.5323	**5.4**
**H(9A-9B)**	1.6862	1.7156	1.7186	1.7332	1.7474	1.7318	1.7435	1.7303	1.7407	1.7347	1.7933	1.6862	1.7156	1.7186	1.7332	1.7474	1.7318	1.7435	1.7303	1.7407	1.7347	1.7933	**2.08**
**H(10A)**	0.9741	0.8166	0.7900	0.7615	0.6871	0.7623	0.7271	0.7634	0.7083	0.7612	0.7224	0.9741	0.8166	0.7900	0.7615	0.6871	0.7623	0.7271	0.7634	0.7083	0.7612	0.7224	**1.14**
**H(10B)**	1.6245	1.5295	1.4648	1.4366	1.3566	1.4418	1.3555	1.4472	1.3556	1.4319	1.3216	1.6245	1.5295	1.4648	1.4366	1.3566	1.4418	1.3555	1.4472	1.3556	1.4319	1.3216	**1.28**
**H(11A-11B)**	0.9968	1.0179	0.9853	1.0245	0.9912	1.0235	0.9920	1.0226	0.9837	1.0257	0.9745	0.9968	1.0179	0.9853	1.0245	0.9912	1.0235	0.9920	1.0226	0.9837	1.0257	0.9745	**1.28**
**H(12A-12C)**	0.9175	0.9002	0.8726	0.8866	0.8472	0.8870	0.8543	0.8875	0.8473	0.8864	0.8426	0.9175	0.9002	0.8727	0.8866	0.8472	0.8870	0.8543	0.8875	0.8473	0.8864	0.8426	**0.82**
**H(1`A)**	2.6470	2.6454	2.6305	2.6365	2.5914	2.6370	2.6054	2.6376	2.6015	2.6363	2.5818	2.7957	2.7944	2.7786	2.7812	2.7529	2.7831	2.7665	2.7849	2.7724	2.7793	2.7420	**3.38**
**H(1`B)**	3.0808	3.1191	3.1539	3.1560	3.2078	3.1531	3.1986	3.1503	3.1886	3.1590	3.1950	2.7613	2.6199	2.6024	2.5918	2.5863	2.5940	2.5496	2.5960	2.5607	2.5899	2.5507	**3.71**
**H(2`A)**	1.6199	1.6689	1.6385	1.6874	1.6359	1.6867	1.6587	1.6859	1.6546	1.6884	1.6672	1.4939	1.5491	1.5195	1.5747	1.5281	1.5739	1.5629	1.5729	1.5563	1.5756	1.5757	**2.08**
**H(2`B)**	1.7534	1.7752	1.7364	1.8180	1.7785	1.8141	1.7797	1.8104	1.7648	1.8221	1.8024	1.6804	1.7692	1.7162	1.8373	1.8050	1.8335	1.8284	1.8297	1.8175	1.8411	1.8621	**2.24**
**H(3`A)**	1.4939	1.5491	1.5195	1.5747	1.5281	1.5739	1.5629	1.5729	1.5563	1.5756	1.5757	1.6199	1.6689	1.6386	1.6874	1.6359	1.6867	1.6587	1.6859	1.6546	1.6884	1.6672	**2.08**
**H(3`B)**	1.6805	1.7691	1.7163	1.8373	1.8050	1.8335	1.8283	1.8297	1.8175	1.8411	1.8621	1.7534	1.7752	1.7364	1.8180	1.7785	1.8141	1.7797	1.8104	1.7648	1.8221	1.8024	**2.16**
**H(4`A)**	2.7613	2.6199	2.6025	2.5918	2.5863	2.5940	2.5496	2.5960	2.5607	2.5899	2.5507	3.0809	3.1191	3.1539	3.1560	3.2078	3.1531	3.1986	3.1503	3.1886	3.1590	3.1950	**3.09**
**H(4`B)**	2.7957	2.7944	2.7786	2.7812	2.7529	2.7831	2.7665	2.7849	2.7724	2.7793	2.7420	2.6470	2.6454	2.6305	2.6365	2.5914	2.6370	2.6054	2.6376	2.6015	2.6363	2.5818	**3.68**

[a] methyl protons shifts are averaged

[b] according to [5]

Table S5. Cont. XIV

Nuclei	R											S											Exp. [b]
	X3LYP																						
	Gas	$CHCl_3$ (PCM)	$CHCl_3$ (SMD)	DMSO (PCM)	DMSO (SMD)	CH_3OH (PCM)	CH_3OH (SMD)	C_2H_5OH (PCM)	C_2H_5OH (SMD)	H_2O (PCM)	H_2O (SMD)	Gas	$CHCl_3$ (PCM)	$CHCl_3$ (SMD)	DMSO (PCM)	DMSO (SMD)	CH_3OH (PCM)	CH_3OH (SMD)	C_2H_5OH (PCM)	C_2H_5OH (SMD)	H_2O (PCM)	H_2O (SMD)	
**C1**	139.6738	140.7029	141.2879	141.2181	142.6377	141.1931	142.0187	141.1652	142.0627	141.2455	142.1890	139.6738	140.7029	141.2877	141.2181	142.6377	141.1931	142.0187	141.1652	142.0627	141.2455	142.1891	**132.2**
**C2**	139.7415	139.6983	140.2072	139.6129	140.0578	139.6218	140.6071	139.6280	140.4859	139.6083	140.5357	139.7415	139.6983	140.2071	139.6129	140.0578	139.6218	140.6071	139.6280	140.4859	139.6083	140.5359	**133.3**
**C3**	122.0899	122.7613	123.3039	122.9822	123.5845	122.9753	123.8523	122.9648	123.7861	122.9920	123.9125	122.0899	122.7613	123.3039	122.9822	123.5845	122.9753	123.8523	122.9648	123.7861	122.9920	123.9125	**117.6**
**C4**	176.3624	177.2172	177.8363	177.6336	178.2690	177.6121	178.8733	177.5874	178.7243	177.6576	178.9971	176.3624	177.2172	177.8362	177.6336	178.2690	177.6121	178.8733	177.5874	178.7243	177.6576	178.9971	**168.3**
**C5**	119.9750	121.1943	121.8036	121.8371	122.6276	121.8009	122.8281	121.7616	122.7328	121.8752	122.9752	119.9750	121.1943	121.8037	121.8372	122.6276	121.8009	122.8281	121.7616	122.7328	121.8752	122.9752	**117.6**
**C6**	135.9479	137.8862	139.0367	139.0291	140.5596	138.9585	141.2161	138.8853	140.9649	139.0998	141.6039	135.9479	137.8862	139.0366	139.0291	140.5596	138.9585	141.2161	138.8853	140.9649	139.0998	141.6039	**133.3**
**C7**	203.8442	208.6848	209.7423	211.2993	211.9258	211.1467	216.9743	210.9904	215.8222	211.4475	217.9867	203.8442	208.6848	209.7423	211.2993	211.9258	211.1467	216.9743	210.9904	215.8222	211.4475	217.9866	**195.8**
**C8**	66.1900	67.3996	67.9886	67.9123	68.9737	67.8933	69.5487	67.8696	69.3941	67.9319	69.8838	66.1900	67.3996	67.9886	67.9123	68.9737	67.8933	69.5487	67.8696	69.3941	67.9319	69.8838	**70.4**
**C9**	37.2125	36.8829	37.1023	36.7383	37.0705	36.7499	37.2814	36.7581	37.2176	36.7305	37.3484	37.2125	36.8829	37.1024	36.7383	37.0705	36.7499	37.2813	36.7581	37.2176	36.7305	37.3483	**31.1**
**C10**	29.2336	29.7336	30.2430	30.0625	30.8205	30.0426	30.6156	30.0200	30.6061	30.0851	30.7479	29.2336	29.7336	30.2429	30.0624	30.8205	30.0426	30.6156	30.0200	30.6061	30.0851	30.7479	**26.9**
**C11**	29.2440	29.3726	29.6606	29.3435	29.7886	29.3527	29.7810	29.3582	29.7823	29.3378	29.9009	29.2440	29.3726	29.6607	29.3436	29.7886	29.3527	29.7810	29.3582	29.7823	29.3378	29.9009	**23.4**
**C12**	16.6790	16.8526	16.9369	16.9924	17.0898	16.9846	17.0466	16.9744	17.0456	17.0037	17.0335	16.6790	16.8526	16.9370	16.9924	17.0898	16.9846	17.0465	16.9744	17.0456	17.0037	17.0336	**13.8**
**C1`**	52.1500	52.9655	53.2787	53.5431	54.0664	53.5092	54.0666	53.4728	53.9991	53.5797	54.5782	56.6692	57.8046	58.1645	58.2841	58.8069	58.2617	58.9279	58.2356	58.8673	58.3087	59.1667	**53.3**
**C2`**	25.9688	26.4573	26.8289	26.6445	27.1554	26.6386	27.1840	26.6293	27.1770	26.6532	26.9598	25.5752	25.7645	26.1378	25.9438	26.4160	25.9341	26.3685	25.9216	26.3639	25.9571	26.1690	**24.2**
**C3`**	25.5752	25.7645	26.1378	25.9439	26.4160	25.9341	26.3685	25.9216	26.3639	25.9571	26.1690	25.9688	26.4573	26.8288	26.6445	27.1554	26.6386	27.1839	26.6293	27.1770	26.6532	26.9597	**24.1**
**C4`**	56.6692	57.8046	58.1645	58.2842	58.8069	58.2616	58.9278	58.2356	58.8673	58.3087	59.1668	52.1500	52.9655	53.2788	53.5431	54.0664	53.5092	54.0666	53.4728	53.9991	53.5797	54.5781	**56.3**
**H2**	8.7017	8.5820	8.6334	8.5144	8.5224	8.5185	8.5444	8.5222	8.5458	8.5104	8.5311	8.7017	8.5820	8.6334	8.5144	8.5224	8.5185	8.5444	8.5222	8.5458	8.5104	8.5311	**8.2**
**H3**	7.3180	7.4626	7.5498	7.5093	7.6221	7.5074	7.6437	7.5048	7.6355	7.5111	7.6574	7.3180	7.4626	7.5498	7.5093	7.6221	7.5074	7.6437	7.5048	7.6355	7.5111	7.6574	**7.36**
**H5**	7.1388	7.2932	7.3946	7.3636	7.4967	7.3596	7.5018	7.3552	7.4932	7.3672	7.5277	7.1388	7.2932	7.3946	7.3636	7.4967	7.3596	7.5018	7.3552	7.4932	7.3672	7.5277	**7.36**
**H6**	7.9778	8.1585	8.2522	8.2595	8.3785	8.2534	8.4310	8.2468	8.4121	8.2652	8.4587	7.9778	8.1585	8.2522	8.2595	8.3785	8.2534	8.4310	8.2468	8.4121	8.2652	8.4587	**8.2**
**H8**	4.6221	4.6810	4.7192	4.7261	4.7782	4.7226	4.8173	4.7187	4.8029	4.7294	4.7963	4.6221	4.6810	4.7192	4.7261	4.7782	4.7226	4.8173	4.7187	4.8029	4.7294	4.7963	**5.4**
**H(9A-9B)**	1.7073	1.7466	1.7566	1.7758	1.8078	1.7740	1.8065	1.7718	1.8013	1.7774	1.8410	1.7073	1.7466	1.7566	1.7758	1.8078	1.7740	1.8065	1.7718	1.8013	1.7774	1.8410	**2.08**
**H(10A)**	1.0017	0.8584	0.8480	0.8249	0.7364	0.8258	0.7864	0.8263	0.7798	0.8243	0.7913	1.0017	0.8584	0.8480	0.8249	0.7364	0.8258	0.7864	0.8263	0.7798	0.8243	0.7913	**1.14**
**H(10B)**	1.6621	1.5559	1.4962	1.4782	1.3946	1.4836	1.3899	1.4885	1.3968	1.4731	1.3608	1.6621	1.5559	1.4962	1.4782	1.3946	1.4836	1.3899	1.4885	1.3968	1.4731	1.3608	**1.28**
**H(11A-11B)**	1.0602	1.0755	1.0472	1.0937	1.0553	1.0929	1.0546	1.0915	1.0540	1.0945	1.0426	1.0602	1.0755	1.0473	1.0937	1.0553	1.0929	1.0547	1.0915	1.0540	1.0945	1.0426	**1.28**
**H(12A-12C)**	0.9403	0.9279	0.9065	0.9238	0.8835	0.9241	0.8907	0.9241	0.8907	0.9234	0.8830	0.9403	0.9279	0.9065	0.9238	0.8835	0.9241	0.8906	0.9241	0.8907	0.9234	0.8830	**0.82**
**H(1`A)**	2.7437	2.7364	2.7259	2.7268	2.6858	2.7276	2.7033	2.7279	2.7047	2.7261	2.6669	2.8709	2.8795	2.8646	2.8584	2.8308	2.8605	2.8410	2.8620	2.8446	2.8562	2.8024	**3.38**
**H(1`B)**	3.2035	3.2527	3.2856	3.2909	3.3575	3.2884	3.3446	3.2855	3.3413	3.2932	3.3404	2.8974	2.7352	2.7069	2.7090	2.7058	2.7093	2.6710	2.7092	2.6722	2.7090	2.6597	**3.71**
**H(2`A)**	1.6334	1.6786	1.6508	1.6992	1.6443	1.6983	1.6703	1.6969	1.6671	1.7000	1.6804	1.5112	1.5567	1.5297	1.5819	1.5257	1.5806	1.5610	1.5789	1.5558	1.5830	1.5767	**2.08**
**H(2`B)**	1.8444	1.8602	1.8198	1.9068	1.8596	1.9031	1.8609	1.8991	1.8545	1.9104	1.8866	1.7037	1.8180	1.7727	1.8911	1.8462	1.8867	1.8698	1.8818	1.8605	1.8953	1.9159	**2.24**
**H(3`A)**	1.5112	1.5567	1.5297	1.5818	1.5257	1.5806	1.5610	1.5789	1.5558	1.5830	1.5767	1.6334	1.6786	1.6507	1.6992	1.6443	1.6983	1.6703	1.6969	1.6671	1.7000	1.6804	**2.08**
**H(3`B)**	1.7037	1.8180	1.7727	1.8911	1.8462	1.8867	1.8698	1.8818	1.8605	1.8953	1.9159	1.8444	1.8602	1.8198	1.9068	1.8596	1.9031	1.8609	1.8991	1.8545	1.9104	1.8866	**2.16**
**H(4`A)**	2.8974	2.7352	2.7069	2.7090	2.7058	2.7093	2.6710	2.7092	2.6722	2.7090	2.6597	3.2035	3.2527	3.2855	3.2909	3.3575	3.2884	3.3446	3.2855	3.3413	3.2932	3.3404	**3.09**
**H(4`B)**	2.8709	2.8795	2.8646	2.8584	2.8308	2.8605	2.8410	2.8620	2.8446	2.8562	2.8024	2.7437	2.7364	2.7259	2.7268	2.6858	2.7276	2.7033	2.7279	2.7047	2.7261	2.6669	**3.68**

[a] methyl protons shifts are averaged

[b] according to [5]

Table S6. Theoretical chemical shifts (in ppm) of *(R,S)*-4-fluoro-α-pyrrolidinohexanophenone calculated with HF and selected functionals combined with 6-311++G** basis set[a]

Nuclei	R											S											Exp. [b]
	**B3LYP**																						
	Gas	$CHCl_3$ (PCM)	$CHCl_3$ (SMD)	DMSO (PCM)	DMSO (SMD)	CH_3OH (PCM)	CH_3OH (SMD)	C_2H_5OH (PCM)	C_2H_5OH (SMD)	H_2O (PCM)	H_2O (SMD)	Gas	$CHCl_3$ (PCM)	$CHCl_3$ (SMD)	DMSO (PCM)	DMSO (SMD)	CH_3OH (PCM)	CH_3OH (SMD)	C_2H_5OH (PCM)	C_2H_5OH (SMD)	H_2O (PCM)	H_2O (SMD)	
**C1**	139.6461	140.7864	141.4058	141.3136	142.6443	141.2719	143.6401	141.2321	143.8057	141.3540	142.2745	139.6461	140.7864	141.4058	141.3136	142.6441	141.2719	143.6401	141.2321	142.2849	141.3540	142.2745	**132.8**
**C2**	139.5237	139.5283	140.0799	139.4229	139.8550	139.4233	140.6856	139.4245	140.5631	139.4217	140.3689	139.5237	139.5283	140.0799	139.4229	139.8550	139.4235	140.6856	139.4245	140.3254	139.4215	140.3689	**132.9**
**C3**	122.0016	122.7109	123.2701	122.8762	123.4842	122.8651	123.8294	122.8532	123.7527	122.8848	123.8436	122.0016	122.7109	123.2701	122.8762	123.4843	122.8651	123.8293	122.8532	123.6960	122.8848	123.8436	**117.2**
**C4**	176.3776	177.2686	177.9231	177.6172	178.2715	177.5929	178.7148	177.5682	178.5305	177.6383	179.0153	176.3776	177.2686	177.9231	177.6172	178.2715	177.5929	178.7149	177.5682	178.7108	177.6383	179.0153	**167.5**
**C5**	119.9008	121.1508	121.7637	121.7354	122.5277	121.6950	122.8173	121.6548	122.6947	121.7720	122.9044	119.9008	121.1508	121.7637	121.7355	122.5277	121.6952	122.8174	121.6548	122.6376	121.7721	122.9044	**117.2**
**C6**	135.7284	137.7097	138.8506	138.7726	140.3125	138.7018	140.2739	138.6307	139.9664	138.8376	141.3542	135.7284	137.7097	138.8506	138.7726	140.3125	138.7019	140.2738	138.6307	140.6449	138.8377	141.3542	**132.9**
**C7**	203.8433	208.7706	209.8361	211.3608	211.9257	211.1952	218.3779	211.0306	217.2932	211.5170	218.0294	203.8433	208.7706	209.8361	211.3607	211.9257	211.1952	218.3780	211.0306	215.9741	211.5172	218.0294	**195.8**
**C8**	66.5250	67.8264	68.3186	68.2996	69.3467	68.2734	70.0962	68.2461	69.9127	68.3213	70.3036	66.5250	67.8264	68.3186	68.2996	69.3467	68.2734	70.0962	68.2461	69.8306	68.3213	70.3036	**68.4**
**C9**	37.2783	36.9954	37.1608	36.7912	37.1386	36.7999	36.9134	36.8081	36.8238	36.7803	37.4426	37.2783	36.9954	37.1608	36.7911	37.1386	36.7999	36.9134	36.8081	37.2550	36.7803	37.4426	**30.5**
**C10**	29.1554	29.7052	30.2009	29.9688	30.7342	29.9463	30.3781	29.9237	30.3314	29.9886	30.6796	29.1554	29.7052	30.2009	29.9690	30.7343	29.9462	30.3780	29.9236	30.5104	29.9887	30.6796	**27.6**
**C11**	29.2176	29.3890	29.6800	29.3092	29.7544	29.3137	29.7204	29.3173	29.7037	29.3025	29.8834	29.2176	29.3890	29.6801	29.3092	29.7544	29.3137	29.7204	29.3173	29.7502	29.3025	29.8834	**23.1**
**C12**	16.6743	16.8847	16.9795	16.9675	17.0657	16.9553	17.1035	16.9436	17.0812	16.9780	17.0345	16.6743	16.8847	16.9795	16.9675	17.0657	16.9553	17.1034	16.9436	17.0289	16.9780	17.0345	**13.8**
**C1`**	52.3408	53.1867	53.5262	53.7049	54.2316	53.6668	54.3062	53.6291	54.2417	53.7402	54.7652	56.8561	58.0291	58.4014	58.4592	58.9820	58.4317	59.1878	58.4037	59.0555	58.4833	59.3691	**53.5**
**C2`**	26.1882	26.7217	27.0852	26.8508	27.3658	26.8407	27.3737	26.8301	27.3214	26.8583	27.1841	25.8103	26.0266	26.4225	26.1449	26.6169	26.1303	26.7163	26.1161	26.5729	26.1578	26.3817	**24.5**
**C3`**	25.8103	26.0266	26.4225	26.1449	26.6168	26.1303	26.7163	26.1161	26.6985	26.1577	26.3817	26.1882	26.7217	27.0852	26.8508	27.3658	26.8408	27.3737	26.8301	27.3873	26.8582	27.1841	**24.3**
**C4`**	56.8561	58.0291	58.4014	58.4593	58.9820	58.4317	59.1878	58.4037	59.0876	58.4834	59.3691	52.3408	53.1867	53.5262	53.7050	54.2316	53.6668	54.3062	53.6290	54.1753	53.7402	54.7652	**57.5**
**H2**	8.6809	8.5543	8.6087	8.4781	8.4949	8.4829	8.4082	8.4875	8.3955	8.4732	8.5014	8.6809	8.5543	8.6087	8.4781	8.4949	8.4829	8.4082	8.4875	8.4977	8.4732	8.5014	**8.11**
**H3**	7.3091	7.4552	7.5406	7.4963	7.6103	7.4942	7.6075	7.4919	7.5968	7.4981	7.6474	7.3091	7.4552	7.5406	7.4963	7.6103	7.4942	7.6075	7.4919	7.6192	7.4981	7.6474	**7.31**
**H5**	7.1276	7.2806	7.3732	7.3462	7.4808	7.3421	7.5147	7.3379	7.5013	7.3498	7.5128	7.1276	7.2806	7.3732	7.3462	7.4808	7.3421	7.5147	7.3379	7.4705	7.3498	7.5128	**7.31**
**H6**	7.9680	8.1482	8.2416	8.2410	8.3613	8.2351	8.3599	8.2291	8.3335	8.2462	8.4392	7.9680	8.1482	8.2416	8.2410	8.3613	8.2351	8.3599	8.2291	8.3823	8.2462	8.4392	**8.11**
**H8**	4.6493	4.7076	4.7462	4.7448	4.7972	4.7412	4.8339	4.7377	4.8186	4.7480	4.8140	4.6493	4.7076	4.7462	4.7448	4.7972	4.7412	4.8339	4.7377	4.8162	4.7480	4.8140	**5.18**
**H(9A-9B)**	1.7031	1.7451	1.7428	1.7658	1.8039	1.7645	1.6855	1.7631	1.6726	1.7666	1.8372	1.7031	1.7451	1.7428	1.7658	1.8039	1.7645	1.6855	1.7631	1.7829	1.7666	1.8372	**2.05**
**H(10A)**	1.0022	0.8621	0.8587	0.8274	0.7397	0.8276	0.8447	0.8281	0.8427	0.8272	0.7965	1.0022	0.8621	0.8587	0.8274	0.7397	0.8276	0.8447	0.8281	0.7822	0.8272	0.7965	**1.14**
**H(10B)**	1.6642	1.5565	1.4947	1.4743	1.3918	1.4794	1.3895	1.4845	1.3962	1.4693	1.3592	1.6642	1.5565	1.4947	1.4743	1.3918	1.4794	1.3895	1.4845	1.3901	1.4693	1.3592	**1.39**
**H(11A-11B)**	1.0615	1.0779	1.0484	1.0922	1.0551	1.0910	1.0592	1.0898	1.0563	1.0931	1.0432	1.0615	1.0779	1.0484	1.0922	1.0551	1.0910	1.0592	1.0898	1.0490	1.0931	1.0432	**1.22**
**H(12A-12C)**	0.9351	0.9251	0.9017	0.9172	0.8775	0.9172	0.9015	0.9173	0.9005	0.9171	0.8785	0.9351	0.9251	0.9017	0.9172	0.8775	0.9172	0.9015	0.9173	0.8821	0.9171	0.8785	**0.77**
**H(1`A)**	2.7510	2.7458	2.7299	2.7316	2.6917	2.7321	2.7055	2.7326	2.7038	2.7310	2.6732	2.8761	2.8891	2.8657	2.8618	2.8352	2.8637	2.8372	2.8655	2.8463	2.8598	2.8089	**3.16**
**H(1`B)**	3.2052	3.2576	3.2845	3.2906	3.3582	3.2880	3.3290	3.2854	3.3214	3.2928	3.3427	2.8957	2.7389	2.7000	2.7100	2.7063	2.7096	2.6874	2.7096	2.6733	2.7104	2.6644	**3.76**
**H(2`A-2`B)**	1.7393	1.7720	1.7348	1.8012	1.7505	1.7985	1.7710	1.7960	1.7651	1.8036	1.7836	1.6062	1.6895	1.6503	1.7348	1.6836	1.7315	1.7396	1.7282	1.7053	1.7379	1.7453	**2.13**
**H(3`A-3`B)**	1.6062	1.6895	1.6503	1.7348	1.6836	1.7315	1.7396	1.7282	1.7324	1.7379	1.7453	1.7393	1.7720	1.7348	1.8012	1.7505	1.7985	1.7710	1.7960	1.7564	1.8036	1.7836	**2.05**
**H(4`A)**	2.8957	2.7389	2.7000	2.7100	2.7062	2.7097	2.6874	2.7096	2.6927	2.7104	2.6644	3.2052	3.2576	3.2845	3.2906	3.3582	3.2880	3.3290	3.2854	3.3378	3.2928	3.3427	**3.09**
**H(4`B)**	2.8761	2.8891	2.8657	2.8618	2.8352	2.8637	2.8372	2.8655	2.8376	2.8598	2.8089	2.7510	2.7458	2.7299	2.7316	2.6917	2.7321	2.7055	2.7326	2.7068	2.7309	2.6732	**3.63**
**F4**	-124.8149	-126.3152	-129.0636	-126.5914	-130.9346	-126.5786	-129.2811	-126.5649	-129.6710	-126.6047	-128.4774	-124.8149	-126.3152	-129.0636	-126.5914	-130.9347	-126.5786	-129.2810	-126.5649	-129.1519	-126.6046	-128.4774	**-104.5**
Nuclei	**B3P86**																						
**C1**	136.9940	137.7944	138.6085	138.2350	139.7431	138.2382	139.0549	138.2181	139.2253	138.2747	139.3602	136.9940	137.7944	138.6085	138.2350	139.7431	138.2382	139.0549	138.2181	139.2253	138.2747	139.3602	**132.8**
**C2**	138.4918	138.4799	139.0262	138.3378	138.8680	138.3690	139.3660	138.3780	139.3322	138.3524	139.3371	138.4918	138.4799	139.0263	138.3378	138.8680	138.3690	139.3660	138.3780	139.3322	138.3524	139.3370	**132.9**
**C3**	121.1824	121.9496	122.5869	122.1830	122.8942	122.1927	123.1872	122.1799	123.2106	122.2177	123.2677	121.1824	121.9496	122.5869	122.1830	122.8942	122.1927	123.1872	122.1799	123.2106	122.2176	123.2677	**117.2**
**C4**	173.8427	174.7165	175.3771	175.1356	175.7951	175.1315	176.4511	175.1055	176.3855	175.1833	176.5856	173.8427	174.7165	175.3770	175.1356	175.7951	175.1316	176.4509	175.1055	176.3855	175.1833	176.5856	**167.5**
**C5**	119.1719	120.4985	121.1966	121.1547	122.0481	121.1341	122.2667	121.0918	122.2598	121.2183	122.4439	119.1720	120.4985	121.1966	121.1548	122.0481	121.1341	122.2666	121.0918	122.2598	121.2183	122.4440	**117.2**
**C6**	134.7482	136.6843	137.8759	137.8221	139.4030	137.7676	140.1213	137.6922	139.9543	137.9183	140.4961	134.7482	136.6843	137.8759	137.8222	139.4030	137.7676	140.1212	137.6922	139.9543	137.9184	140.4961	**132.9**
**C7**	201.9446	206.3100	207.5577	208.7236	209.4429	208.6038	214.1587	208.4609	213.2041	208.8803	215.2563	201.9446	206.3100	207.5577	208.7236	209.4429	208.6038	214.1587	208.4609	213.2041	208.8803	215.2563	**195.8**
**C8**	64.8267	65.9499	66.7652	66.4439	67.5035	66.4394	68.0435	66.4121	68.0288	66.4911	68.4972	64.8267	65.9499	66.7652	66.4439	67.5035	66.4394	68.0435	66.4121	68.0288	66.4911	68.4972	**68.4**
**C9**	36.2486	36.0166	36.3080	35.8772	36.2423	35.9043	36.4484	35.9094	36.4633	35.8954	36.5768	36.2486	36.0166	36.3080	35.8771	36.2423	35.9042	36.4484	35.9094	36.4633	35.8953	36.5768	**30.5**
**C10**	27.9025	28.4047	28.9185	28.7349	29.4674	28.7300	29.2804	28.7039	29.3529	28.7840	29.4513	27.9025	28.4046	28.9185	28.7349	29.4674	28.7300	29.2804	28.7039	29.3529	28.7840	29.4513	**27.6**
**C11**	27.6635	27.8017	28.0996	27.7450	28.1783	27.7719	28.1762	27.7764	28.2670	27.7630	28.3209	27.6635	27.8016	28.0997	27.7450	28.1783	27.7718	28.1763	27.7764	28.2670	27.7629	28.3209	**23.1**
**C12**	16.2632	16.4367	16.5321	16.5461	16.6415	16.5582	16.6066	16.5487	16.6923	16.5790	16.6054	16.2632	16.4367	16.5320	16.5462	16.6415	16.5582	16.6066	16.5487	16.6923	16.5790	16.6054	**13.8**
**C1`**	51.2290	52.0562	52.3501	52.5739	53.1095	52.5603	53.1155	52.5254	53.1243	52.6314	53.6115	55.5727	56.7173	57.1113	57.1649	57.6791	57.1609	57.8424	57.1348	57.8756	57.2124	58.1048	**53.5**
**C2`**	24.9726	25.3900	25.7808	25.5444	26.0182	25.5568	26.0360	25.5472	26.1322	25.5763	25.8635	24.4400	24.6560	24.9992	24.8082	25.2508	24.8198	25.2291	24.8094	25.3062	24.8411	25.0933	**24.5**
**C3`**	24.4400	24.6560	24.9990	24.8081	25.2508	24.8198	25.2291	24.8094	25.3062	24.8410	25.0933	24.9726	25.3900	25.7807	25.5444	26.0182	25.5568	26.0361	25.5472	26.1322	25.5763	25.8636	**24.3**
**C4`**	55.5727	56.7174	57.1114	57.1649	57.6791	57.1610	57.8424	57.1348	57.8756	57.2124	58.1048	51.2290	52.0562	52.3501	52.5740	53.1095	52.5603	53.1154	52.5254	53.1243	52.6314	53.6115	**57.5**
**H2**	8.7290	8.6450	8.6624	8.5763	8.5750	8.5811	8.6077	8.5842	8.6051	8.5730	8.5861	8.7290	8.6450	8.6624	8.5763	8.5750	8.5811	8.6077	8.5842	8.6051	8.5730	8.5861	**8.11**
**H3**	7.3236	7.4766	7.5645	7.5245	7.6430	7.5235	7.6668	7.5205	7.6614	7.5267	7.6840	7.3236	7.4766	7.5645	7.5245	7.6430	7.5234	7.6668	7.5205	7.6614	7.5267	7.6839	**7.31**
**H5**	7.1584	7.3295	7.4289	7.3944	7.5356	7.3914	7.5373	7.3866	7.5325	7.3986	7.5649	7.1584	7.3295	7.4289	7.3944	7.5356	7.3914	7.5373	7.3866	7.5325	7.3985	7.5649	**7.31**
**H6**	8.0391	8.2172	8.3003	8.3183	8.4387	8.3130	8.5009	8.3060	8.4828	8.3245	8.5279	8.0391	8.2172	8.3003	8.3183	8.4387	8.3130	8.5009	8.3060	8.4828	8.3245	8.5279	**8.11**
**H8**	4.6504	4.7081	4.7313	4.7587	4.8048	4.7563	4.8490	4.7522	4.8350	4.7621	4.8465	4.6504	4.7081	4.7313	4.7587	4.8048	4.7563	4.8490	4.7522	4.8350	4.7621	4.8465	**5.18**
**H(9A-9B)**	1.7737	1.8107	1.8173	1.8361	1.8597	1.8350	1.8591	1.8321	1.8559	1.8386	1.8916	1.7737	1.8107	1.8173	1.8361	1.8597	1.8350	1.8591	1.8321	1.8559	1.8386	1.8916	**2.05**
**H(10A)**	0.9752	0.8444	0.8027	0.7996	0.7098	0.8026	0.7576	0.8038	0.7475	0.7979	0.7640	0.9752	0.8444	0.8027	0.7996	0.7098	0.8026	0.7576	0.8038	0.7475	0.7979	0.7640	**1.14**
**H(10B)**	1.6857	1.5846	1.5093	1.4961	1.4096	1.5034	1.4026	1.5090	1.4115	1.4903	1.3722	1.6857	1.5846	1.5093	1.4961	1.4096	1.5034	1.4026	1.5090	1.4115	1.4903	1.3722	**1.39**
**H(11A-11B)**	1.0296	1.0511	1.0141	1.0605	1.0240	1.0614	1.0238	1.0603	1.0254	1.0611	1.0130	1.0296	1.0511	1.0141	1.0605	1.0240	1.0614	1.0238	1.0603	1.0254	1.0611	1.0130	**1.22**
**H(12A-12C)**	0.8986	0.8890	0.8627	0.8785	0.8412	0.8804	0.8463	0.8804	0.8493	0.8780	0.8425	0.8986	0.8890	0.8627	0.8785	0.8412	0.8804	0.8463	0.8804	0.8493	0.8780	0.8425	**0.77**
**H(1`A)**	2.7041	2.6985	2.6865	2.6863	2.6464	2.6882	2.6613	2.6883	2.6657	2.6858	2.6366	2.8371	2.8452	2.8347	2.8253	2.7986	2.8283	2.8066	2.8295	2.8150	2.8235	2.7746	**3.16**
**H(1`B)**	3.2036	3.2482	3.2821	3.2812	3.3423	3.2798	3.3295	3.2766	3.3304	3.2838	3.3300	2.9405	2.7570	2.7404	2.7125	2.7146	2.7151	2.6633	2.7161	2.6706	2.7113	2.6518	**3.76**
**H(2`A-2`B)**	1.7239	1.7569	1.7220	1.7865	1.7420	1.7855	1.7525	1.7827	1.7511	1.7888	1.7738	1.5947	1.6704	1.6340	1.7128	1.6684	1.7115	1.6954	1.7083	1.6918	1.7154	1.7333	**2.13**
**H(3`A-3`B)**	1.5947	1.6704	1.6340	1.7128	1.6684	1.7115	1.6954	1.7083	1.6918	1.7154	1.7333	1.7239	1.7569	1.7220	1.7865	1.7420	1.7855	1.7525	1.7827	1.7511	1.7888	1.7738	**2.05**
**H(4`A)**	2.9405	2.7570	2.7404	2.7125	2.7146	2.7151	2.6633	2.7161	2.6706	2.7113	2.6518	3.2036	3.2482	3.2821	3.2812	3.3423	3.2797	3.3295	3.2766	3.3304	3.2838	3.3300	**3.09**
**H(4`B)**	2.8371	2.8452	2.8347	2.8253	2.7986	2.8283	2.8065	2.8295	2.8150	2.8235	2.7746	2.7041	2.6985	2.6865	2.6864	2.6464	2.6882	2.6612	2.6883	2.6657	2.6858	2.6366	**3.63**
**F4**	-117.3895	-118.3317	-120.9580	-118.3711	-122.4835	-118.3770	-120.1128	-118.3824	-120.5248	-118.3637	-119.8815	-117.3895	-118.3317	-120.9580	-118.3712	-122.4835	-118.3770	-120.1130	-118.3824	-120.5248	-118.3636	-119.8817	**-104.5**

[a] methyl protons shifts are averaged

[b] according to [6]

Table S6. Cont. I

Nuclei	R											S											Exp. [b]
	B3PW91																						
	Gas	$CHCl_3$ (PCM)	$CHCl_3$ (SMD)	DMSO (PCM)	DMSO (SMD)	CH_3OH (PCM)	CH_3OH (SMD)	C_2H_5OH (PCM)	C_2H_5OH (SMD)	H_2O (PCM)	H_2O (SMD)	Gas	$CHCl_3$ (PCM)	$CHCl_3$ (SMD)	DMSO (PCM)	DMSO (SMD)	CH_3OH (PCM)	CH_3OH (SMD)	C_2H_5OH (PCM)	C_2H_5OH (SMD)	H_2O (PCM)	H_2O (SMD)	
**C1**	137.2683	138.3110	139.0251	138.7632	140.5159	138.7452	139.6322	138.7174	139.6500	138.7819	139.7985	137.2684	138.3110	139.0253	138.7632	140.1969	138.7451	139.6321	138.7176	139.6500	138.7819	139.7985	**132.8**
**C2**	138.6410	138.6605	139.1565	138.5404	139.4008	138.5539	139.5783	138.5587	139.4533	138.5296	139.5357	138.6410	138.6605	139.1563	138.5404	139.0818	138.5541	139.5784	138.5588	139.4534	138.5296	139.5357	**132.9**
**C3**	121.2594	122.0369	122.5996	122.2518	123.2768	122.2465	123.2362	122.2321	123.1632	122.2587	123.3253	121.2594	122.0369	122.5996	122.2518	122.9578	122.2466	123.2362	122.2321	123.1632	122.2587	123.3253	**117.2**
**C4**	173.9083	174.7882	175.3849	175.1648	176.1478	175.1468	176.4418	175.1200	176.2919	175.1841	176.5909	173.9084	174.7882	175.3847	175.1648	175.8288	175.1467	176.4417	175.1199	176.2919	175.1841	176.5909	**167.5**
**C5**	119.2542	120.5663	121.1995	121.2079	122.4095	121.1729	122.3091	121.1291	122.2069	121.2432	122.4865	119.2542	120.5663	121.1995	121.2079	122.0905	121.1728	122.3091	121.1291	122.2069	121.2432	122.4865	**117.2**
**C6**	134.7732	136.7237	137.8161	137.8284	139.7082	137.7607	140.0363	137.6852	139.7917	137.8952	140.4527	134.7731	136.7237	137.8164	137.8284	139.3892	137.7607	140.0362	137.6852	139.7918	137.8952	140.4527	**132.9**
**C7**	201.9625	206.6067	207.7419	209.0508	209.9966	208.9099	214.5826	208.7599	213.4600	209.1857	215.5648	201.9625	206.6067	207.7420	209.0508	209.6776	208.9099	214.5827	208.7599	213.4600	209.1857	215.5648	**195.8**
**C8**	64.7955	66.0465	66.7422	66.5611	67.8989	66.5428	68.2177	66.5141	68.0546	66.5791	68.5848	64.7955	66.0465	66.7421	66.5611	67.5799	66.5427	68.2177	66.5142	68.0547	66.5791	68.5848	**68.4**
**C9**	35.8212	35.6167	35.7953	35.4785	36.1538	35.4920	36.0457	35.4963	35.9670	35.4673	36.1611	35.8211	35.6167	35.7953	35.4785	35.8348	35.4919	36.0457	35.4963	35.9670	35.4673	36.1611	**30.5**
**C10**	27.6048	28.1256	28.5599	28.4339	29.4841	28.4158	28.9652	28.3894	28.9490	28.4536	29.1548	27.6048	28.1256	28.5601	28.4339	29.1651	28.4157	28.9652	28.3894	28.9490	28.4536	29.1548	**27.6**
**C11**	27.6205	27.7953	28.0026	27.7348	28.4759	27.7465	28.1492	27.7489	28.1449	27.7251	28.2980	27.6204	27.7953	28.0026	27.7347	28.1569	27.7465	28.1494	27.7489	28.1449	27.7251	28.2980	**23.1**
**C12**	16.2867	16.4838	16.5092	16.5803	16.9882	16.5767	16.6234	16.5648	16.6184	16.5862	16.6291	16.2866	16.4838	16.5092	16.5803	16.6692	16.5768	16.6233	16.5649	16.6184	16.5862	16.6291	**13.8**
**C1`**	51.1535	52.0040	52.2161	52.5029	53.3437	52.4743	53.0216	52.4374	52.9376	52.5328	53.5322	55.5036	56.6599	56.9753	57.0912	57.5895	57.0723	57.7369	57.0442	57.6754	57.1109	57.9949	**53.5**
**C2`**	25.2271	25.6707	25.9838	25.8063	26.5923	25.8042	26.2842	25.7931	26.2820	25.8101	26.0751	24.7121	24.9298	25.2094	25.0583	25.5027	25.0537	25.4471	25.0406	25.4422	25.0649	25.2887	**24.5**
**C3`**	24.7122	24.9298	25.2095	25.0584	25.8217	25.0538	25.4471	25.0407	25.4422	25.0649	25.2887	25.2272	25.6707	25.9838	25.8063	26.2733	25.8042	26.2842	25.7932	26.2820	25.8101	26.0751	**24.3**
**C4`**	55.5037	56.6599	56.9752	57.0912	57.9085	57.0722	57.7369	57.0442	57.6754	57.1109	57.9949	51.1535	52.0040	52.2161	52.5029	53.0247	52.4742	53.0217	52.4375	52.9376	52.5328	53.5322	**57.5**
**H2**	8.7062	8.5966	8.6228	8.5283	8.5523	8.5326	8.5570	8.5368	8.5576	8.5243	8.5484	8.7062	8.5966	8.6228	8.5283	8.5368	8.5326	8.5570	8.5368	8.5576	8.5243	8.5484	**8.11**
**H3**	7.3295	7.4785	7.5672	7.5237	7.6568	7.5220	7.6646	7.5199	7.6557	7.5254	7.6799	7.3295	7.4785	7.5672	7.5237	7.6413	7.5220	7.6646	7.5199	7.6557	7.5254	7.6799	**7.31**
**H5**	7.1489	7.3105	7.4083	7.3811	7.5343	7.3773	7.5250	7.3732	7.5160	7.3848	7.5528	7.1489	7.3105	7.4082	7.3811	7.5188	7.3773	7.5250	7.3732	7.5160	7.3848	7.5528	**7.31**
**H6**	8.0298	8.2071	8.2903	8.3046	8.4406	8.2988	8.4763	8.2927	8.4580	8.3103	8.5083	8.0298	8.2071	8.2903	8.3046	8.4251	8.2988	8.4763	8.2927	8.4580	8.3103	8.5083	**8.11**
**H8**	4.6458	4.7027	4.7242	4.7494	4.8097	4.7462	4.8338	4.7427	4.8182	4.7527	4.8284	4.6458	4.7027	4.7242	4.7494	4.7942	4.7462	4.8338	4.7427	4.8182	4.7527	4.8284	**5.18**
**H(9A-9B)**	1.7271	1.7604	1.7647	1.7862	1.8281	1.7847	1.8116	1.7831	1.8058	1.7877	1.8467	1.7270	1.7604	1.7647	1.7862	1.8126	1.7847	1.8115	1.7831	1.8058	1.7877	1.8467	**2.05**
**H(10A)**	0.9509	0.8160	0.7754	0.7757	0.7042	0.7773	0.7328	0.7788	0.7236	0.7745	0.7414	0.9509	0.8160	0.7754	0.7757	0.6887	0.7773	0.7328	0.7788	0.7236	0.7745	0.7414	**1.14**
**H(10B)**	1.6448	1.5409	1.4720	1.4568	1.3876	1.4629	1.3662	1.4686	1.3723	1.4513	1.3371	1.6448	1.5409	1.4720	1.4568	1.3721	1.4629	1.3661	1.4686	1.3723	1.4513	1.3371	**1.39**
**H(11A-11B)**	1.0151	1.0327	0.9969	1.0448	1.0240	1.0446	1.0073	1.0441	1.0064	1.0451	0.9964	1.0151	1.0327	0.9969	1.0448	1.0085	1.0446	1.0074	1.0441	1.0064	1.0451	0.9964	**1.22**
**H(12A-12C)**	0.8895	0.8782	0.8516	0.8692	0.8460	0.8701	0.8372	0.8709	0.8369	0.8685	0.8312	0.8895	0.8782	0.8516	0.8692	0.8306	0.8701	0.8372	0.8709	0.8369	0.8685	0.8312	**0.77**
**H(1`A)**	2.6965	2.6898	2.6781	2.6782	2.6537	2.6793	2.6560	2.6801	2.6567	2.6772	2.6259	2.8310	2.8358	2.8235	2.8145	2.7855	2.8167	2.7978	2.8187	2.8011	2.8123	2.7614	**3.16**
**H(1`B)**	3.1800	3.2261	3.2593	3.2607	3.3370	3.2586	3.3106	3.2563	3.3077	3.2628	3.3078	2.8932	2.7251	2.7090	2.6878	2.6888	2.6892	2.6489	2.6905	2.6501	2.6868	2.6322	**3.76**
**H(2`A-2`B)**	1.7102	1.7429	1.7077	1.7729	1.7417	1.7711	1.7394	1.7690	1.7343	1.7749	1.7585	1.5847	1.6616	1.6246	1.7053	1.6572	1.7029	1.6883	1.7004	1.6796	1.7077	1.7213	**2.13**
**H(3`A-3`B)**	1.5847	1.6616	1.6246	1.7053	1.6727	1.7030	1.6883	1.7004	1.6796	1.7077	1.7213	1.7102	1.7429	1.7077	1.7729	1.7262	1.7711	1.7394	1.7690	1.7343	1.7749	1.7585	**2.05**
**H(4`A)**	2.8932	2.7251	2.7090	2.6878	2.7043	2.6892	2.6488	2.6905	2.6501	2.6868	2.6322	3.1800	3.2261	3.2593	3.2607	3.3215	3.2586	3.3107	3.2563	3.3077	3.2628	3.3078	**3.09**
**H(4`B)**	2.8310	2.8358	2.8235	2.8145	2.8010	2.8168	2.7978	2.8186	2.8010	2.8123	2.7614	2.6964	2.6898	2.6781	2.6782	2.6382	2.6793	2.6560	2.6801	2.6567	2.6772	2.6259	**3.63**
**F4**	-116.7959	-117.9095	-120.5427	-118.0094	-120.9748	-118.0111	-119.8565	-118.0120	-120.2252	-118.0064	-119.5682	-116.7958	-117.9095	-120.5429	-118.0094	-122.1115	-118.0112	-119.8566	-118.0121	-120.2252	-118.0064	-119.5682	**-104.5**
Nuclei	B98																						
**C1**	138.2717	139.2268	139.9128	139.6404	141.1346	139.6354	140.5187	139.6273	140.5521	139.6471	140.7148	138.2716	139.2268	139.9128	139.6403	141.1346	139.6354	140.5187	139.6273	140.5521	139.6471	140.7148	**132.8**
**C2**	138.3658	138.4130	138.9400	138.2863	138.8240	138.3104	139.3230	138.3318	139.2146	138.2658	139.2706	138.3659	138.4130	138.9400	138.2864	138.8240	138.3104	139.3230	138.3318	139.2146	138.2658	139.2706	**132.9**
**C3**	120.9842	121.7099	122.2670	121.9312	122.6018	121.9357	122.8713	121.9373	122.8035	121.9289	122.9366	120.9842	121.7099	122.2670	121.9312	122.6018	121.9357	122.8714	121.9373	122.8035	121.9289	122.9366	**117.2**
**C4**	173.7110	174.5345	175.1530	174.9251	175.5881	174.9164	176.1881	174.9052	176.0450	174.9356	176.3122	173.7110	174.5345	175.1529	174.9250	175.5881	174.9164	176.1881	174.9052	176.0450	174.9356	176.3121	**167.5**
**C5**	118.9649	120.2115	120.8309	120.8532	121.6929	120.8287	121.9059	120.8015	121.8085	120.8789	122.0628	118.9649	120.2115	120.8308	120.8531	121.6929	120.8287	121.9058	120.8015	121.8086	120.8789	122.0628	**117.2**
**C6**	134.7148	136.5886	137.6963	137.6948	139.2264	137.6373	139.8807	137.5782	139.6376	137.7516	140.2548	134.7148	136.5886	137.6962	137.6945	139.2264	137.6373	139.8805	137.5782	139.6376	137.7516	140.2547	**132.9**
**C7**	200.3965	205.1270	206.2286	207.6183	208.2481	207.4848	213.3382	207.3483	212.1534	207.7463	214.3728	200.3966	205.1270	206.2283	207.6181	208.2481	207.4848	213.3381	207.3483	212.1534	207.7463	214.3729	**195.8**
**C8**	66.4517	67.6676	68.3328	68.1491	69.2093	68.1435	69.8344	68.1334	69.6562	68.1551	70.2146	66.4518	67.6676	68.3327	68.1492	69.2093	68.1435	69.8344	68.1334	69.6562	68.1551	70.2145	**68.4**
**C9**	37.0176	36.7658	36.9738	36.6486	37.0069	36.6711	37.2536	36.6906	37.1768	36.6290	37.3166	37.0176	36.7658	36.9738	36.6487	37.0069	36.6711	37.2535	36.6906	37.1768	36.6290	37.3166	**30.5**
**C10**	29.0963	29.5633	30.0409	29.8649	30.6136	29.8578	30.4248	29.8488	30.4145	29.8742	30.5904	29.0963	29.5633	30.0409	29.8648	30.6136	29.8578	30.4248	29.8488	30.4145	29.8742	30.5904	**27.6**
**C11**	28.9406	29.0642	29.3038	29.0121	29.4482	29.0334	29.4500	29.0515	29.4499	28.9937	29.5766	28.9406	29.0642	29.3037	29.0122	29.4482	29.0334	29.4499	29.0515	29.4499	28.9937	29.5766	**23.1**
**C12**	16.8660	17.0178	17.0824	17.1275	17.2327	17.1333	17.1960	17.1370	17.1978	17.1247	17.1872	16.8660	17.0178	17.0824	17.1275	17.2327	17.1333	17.1960	17.1370	17.1978	17.1247	17.1871	**13.8**
**C1`**	52.4335	53.2373	53.4722	53.7447	54.2542	53.7251	54.2559	53.7037	54.1881	53.7667	54.7388	56.8881	57.9505	58.2822	58.3585	58.8726	58.3505	59.0149	58.3396	58.9555	58.3681	59.2730	**53.5**
**C2`**	26.4056	26.8263	27.1611	26.9646	27.4451	26.9719	27.4788	26.9766	27.4727	26.9593	27.2819	25.8949	26.0922	26.4234	26.2448	26.6989	26.2484	26.6621	26.2500	26.6634	26.2440	26.4925	**24.5**
**C3`**	25.8950	26.0922	26.4233	26.2448	26.6989	26.2484	26.6622	26.2500	26.6634	26.2440	26.4925	26.4056	26.8263	27.1611	26.9645	27.4451	26.9719	27.4788	26.9766	27.4727	26.9593	27.2819	**24.3**
**C4`**	56.8881	57.9505	58.2822	58.3585	58.8726	58.3505	59.0149	58.3396	58.9555	58.3681	59.2731	52.4335	53.2373	53.4723	53.7448	54.2542	53.7251	54.2559	53.7037	54.1881	53.7667	54.7388	**57.5**
**H2**	8.6982	8.5886	8.6267	8.5270	8.5364	8.5306	8.5595	8.5343	8.5625	8.5239	8.5476	8.6982	8.5886	8.6267	8.5270	8.5364	8.5306	8.5595	8.5343	8.5625	8.5239	8.5476	**8.11**
**H3**	7.3243	7.4676	7.5579	7.5131	7.6334	7.5110	7.6550	7.5089	7.6467	7.5153	7.6698	7.3243	7.4676	7.5579	7.5131	7.6334	7.5110	7.6550	7.5089	7.6467	7.5153	7.6698	**7.31**
**H5**	7.1440	7.3083	7.4061	7.3810	7.5164	7.3767	7.5244	7.3726	7.5150	7.3852	7.5500	7.1439	7.3083	7.4061	7.3810	7.5164	7.3767	7.5244	7.3726	7.5150	7.3852	7.5500	**7.31**
**H6**	7.9991	8.1757	8.2656	8.2788	8.3991	8.2722	8.4528	8.2658	8.4346	8.2852	8.4806	7.9991	8.1757	8.2656	8.2788	8.3991	8.2722	8.4528	8.2658	8.4346	8.2852	8.4806	**8.11**
**H8**	4.6061	4.6605	4.6938	4.7112	4.7597	4.7072	4.8012	4.7034	4.7869	4.7153	4.7909	4.6061	4.6605	4.6938	4.7112	4.7597	4.7072	4.8012	4.7034	4.7869	4.7153	4.7909	**5.18**
**H(9A-9B)**	1.7078	1.7440	1.7500	1.7713	1.8003	1.7695	1.8017	1.7677	1.7957	1.7733	1.8332	1.7078	1.7440	1.7500	1.7713	1.8003	1.7695	1.8016	1.7677	1.7957	1.7733	1.8332	**2.05**
**H(10A)**	1.0100	0.8556	0.8250	0.8148	0.7317	0.8159	0.7784	0.8174	0.7740	0.8141	0.7819	1.0100	0.8556	0.8250	0.8147	0.7317	0.8159	0.7784	0.8174	0.7740	0.8141	0.7819	**1.14**
**H(10B)**	1.6566	1.5552	1.4953	1.4723	1.3892	1.4780	1.3847	1.4837	1.3917	1.4673	1.3576	1.6566	1.5552	1.4953	1.4723	1.3892	1.4780	1.3847	1.4837	1.3917	1.4673	1.3576	**1.39**
**H(11A-11B)**	1.0408	1.0554	1.0229	1.0697	1.0330	1.0689	1.0321	1.0683	1.0319	1.0707	1.0208	1.0408	1.0554	1.0229	1.0697	1.0330	1.0689	1.0321	1.0683	1.0319	1.0707	1.0208	**1.22**
**H(12A-12C)**	0.9137	0.8998	0.8755	0.8914	0.8528	0.8919	0.8600	0.8926	0.8602	0.8913	0.8544	0.9137	0.8998	0.8755	0.8914	0.8528	0.8919	0.8600	0.8926	0.8602	0.8913	0.8544	**0.77**
**H(1`A)**	2.7147	2.7096	2.6996	2.6998	2.6606	2.7004	2.6788	2.7011	2.6801	2.6994	2.6440	2.8444	2.8541	2.8420	2.8323	2.8064	2.8344	2.8190	2.8364	2.8217	2.8306	2.7831	**3.16**
**H(1`B)**	3.1966	3.2429	3.2781	3.2791	3.3414	3.2766	3.3292	3.2742	3.3264	3.2818	3.3230	2.8921	2.7320	2.7168	2.7042	2.7055	2.7047	2.6717	2.7055	2.6724	2.7042	2.6580	**3.76**
**H(2`A-2`B)**	1.7273	1.7581	1.7234	1.7904	1.7425	1.7881	1.7566	1.7859	1.7516	1.7931	1.7748	1.5089	1.5555	1.5281	1.5780	1.5262	1.5768	1.5620	1.5757	1.5564	1.5793	1.5795	**2.13**
**H(3`A-3`B)**	1.6066	1.6841	1.6466	1.7277	1.6794	1.7250	1.7102	1.7224	1.7022	1.7305	1.7444	1.6688	1.7455	1.7074	1.7868	1.7390	1.7842	1.7647	1.7818	1.7578	1.7895	1.7959	**2.05**
**H(4`A)**	2.8922	2.7320	2.7168	2.7042	2.7055	2.7047	2.6717	2.7055	2.6724	2.7042	2.6580	2.5089	2.5405	2.5376	2.5819	2.5905	2.5787	2.5856	2.5757	2.5811	2.5853	2.5952	**3.09**
**H(4`B)**	2.8444	2.8541	2.8420	2.8323	2.8064	2.8344	2.8190	2.8364	2.8217	2.8306	2.7831	2.7147	2.7096	2.6996	2.6998	2.6606	2.7004	2.6788	2.7011	2.6801	2.6994	2.6440	**3.63**
**F4**	-119.9176	-121.0442	-123.7574	-121.1149	-125.3354	-121.1187	-123.1429	-121.1210	-123.5029	-121.1108	-122.8648	-119.9177	-121.0442	-123.7575	-121.1149	-125.3354	-121.1187	-123.1431	-121.1210	-123.5029	-121.1108	-122.8649	**-104.5**

[a] methyl protons shifts are averaged
[b] according to [6]

Table S6. Cont. II

Nuclei	R											S											Exp. [b]
	B971																						
	Gas	$CHCl_3$ (PCM)	$CHCl_3$ (SMD)	DMSO (PCM)	DMSO (SMD)	CH_3OH (PCM)	CH_3OH (SMD)	C_2H_5OH (PCM)	C_2H_5OH (SMD)	H_2O (PCM)	H_2O (SMD)	Gas	$CHCl_3$ (PCM)	$CHCl_3$ (SMD)	DMSO (PCM)	DMSO (SMD)	CH_3OH (PCM)	CH_3OH (SMD)	C_2H_5OH (PCM)	C_2H_5OH (SMD)	H_2O (PCM)	H_2O (SMD)	
**C1**	137.8422	138.7483	139.4242	139.1819	140.7120	139.1719	140.0612	139.1604	140.1037	139.1965	140.2855	137.8422	138.7484	139.4242	139.1821	140.7120	139.1719	140.0613	139.1604	140.1037	139.1965	140.2855	**132.8**
**C2**	138.0799	138.1325	138.6399	138.0013	138.5299	138.0181	139.0158	138.0344	138.9092	137.9914	138.9727	138.0799	138.1323	138.6399	138.0013	138.5299	138.0181	139.0159	138.0344	138.9092	137.9914	138.9727	**132.9**
**C3**	120.7944	121.5215	122.0745	121.7507	122.4133	121.7473	122.6794	121.7430	122.6120	121.7596	122.7491	120.7944	121.5213	122.0745	121.7507	122.4133	121.7473	122.6794	121.7430	122.6119	121.7596	122.7491	**117.2**
**C4**	173.5971	174.4353	175.0465	174.8429	175.4966	174.8257	176.0952	174.8081	175.9514	174.8650	176.2233	173.5971	174.4353	175.0464	174.8427	175.4966	174.8257	176.0951	174.8081	175.9514	174.8650	176.2233	**167.5**
**C5**	118.7655	120.0340	120.6434	120.6887	121.5173	120.6557	121.7292	120.6223	121.6310	120.7260	121.8918	118.7655	120.0341	120.6435	120.6887	121.5173	120.6557	121.7293	120.6223	121.6311	120.7260	121.8918	**117.2**
**C6**	134.4339	136.3104	137.4295	137.4298	138.9589	137.3639	139.6148	137.2980	139.3694	137.4992	139.9897	134.4339	136.3105	137.4296	137.4299	138.9589	137.3638	139.6149	137.2980	139.3693	137.4992	139.9897	**132.9**
**C7**	200.2729	204.9044	206.0061	207.3978	208.0832	207.2601	213.0559	207.1201	211.9008	207.5342	214.1161	200.2729	204.9043	206.0060	207.3982	208.0832	207.2600	213.0562	207.1201	211.9008	207.5342	214.1161	**195.8**
**C8**	66.2684	67.4430	68.1355	67.9281	69.0012	67.9149	69.5789	67.8993	69.4095	67.9447	69.9754	66.2684	67.4428	68.1355	67.9282	69.0012	67.9149	69.5789	67.8993	69.4094	67.9447	69.9754	**68.4**
**C9**	37.0068	36.7745	37.0004	36.6683	37.0130	36.6820	37.2582	36.6949	37.1794	36.6609	37.3381	37.0068	36.7745	37.0004	36.6683	37.0130	36.6820	37.2583	36.6949	37.1793	36.6609	37.3381	**30.5**
**C10**	29.1505	29.6168	30.0966	29.9276	30.6566	29.9120	30.4719	29.8963	30.4604	29.9491	30.6448	29.1505	29.6167	30.0966	29.9277	30.6566	29.9120	30.4718	29.8963	30.4603	29.9491	30.6448	**27.6**
**C11**	28.9245	29.0484	29.2800	29.0001	29.4237	29.0135	29.4199	29.0260	29.4191	28.9929	29.5535	28.9245	29.0484	29.2800	29.0002	29.4237	29.0136	29.4199	29.0260	29.4191	28.9929	29.5535	**23.1**
**C12**	16.8110	16.9684	17.0237	17.0860	17.1770	17.0840	17.1383	17.0818	17.1401	17.0943	17.1357	16.8110	16.9683	17.0236	17.0860	17.1770	17.0840	17.1384	17.0818	17.1402	17.0943	17.1357	**13.8**
**C1`**	52.3547	53.1528	53.3732	53.6664	54.1692	53.6388	54.1635	53.6114	54.0937	53.6995	54.6432	56.7953	57.8551	58.1732	58.2702	58.7717	58.2546	58.9084	58.2381	58.8505	58.2908	59.1676	**53.5**
**C2`**	26.2113	26.6404	26.9708	26.7933	27.2590	26.7924	27.2868	26.7908	27.2809	26.7996	27.1109	25.6996	25.9076	26.2264	26.0756	26.5176	26.0712	26.4850	26.0668	26.4842	26.0859	26.3418	**24.5**
**C3`**	25.6996	25.9075	26.2264	26.0756	26.5176	26.0712	26.4850	26.0668	26.4841	26.0859	26.3418	26.2113	26.6403	26.9708	26.7933	27.2590	26.7924	27.2869	26.7908	27.2809	26.7996	27.1109	**24.3**
**C4`**	56.7953	57.8551	58.1732	58.2702	58.7717	58.2546	58.9084	58.2381	58.8505	58.2908	59.1676	52.3547	53.1529	53.3732	53.6663	54.1692	53.6387	54.1636	53.6114	54.0938	53.6995	54.6432	**57.5**
**H2**	8.6975	8.5947	8.6294	8.5328	8.5358	8.5359	8.5631	8.5392	8.5649	8.5303	8.5487	8.6975	8.5947	8.6294	8.5328	8.5358	8.5359	8.5631	8.5392	8.5649	8.5303	8.5487	**8.11**
**H3**	7.3205	7.4628	7.5522	7.5095	7.6286	7.5072	7.6504	7.5050	7.6421	7.5121	7.6648	7.3205	7.4628	7.5522	7.5095	7.6286	7.5072	7.6504	7.5050	7.6421	7.5121	7.6648	**7.31**
**H5**	7.1452	7.3121	7.4102	7.3844	7.5179	7.3799	7.5258	7.3755	7.5164	7.3890	7.5509	7.1452	7.3121	7.4102	7.3844	7.5179	7.3799	7.5258	7.3755	7.5164	7.3890	7.5509	**7.31**
**H6**	8.0058	8.1773	8.2674	8.2802	8.3987	8.2735	8.4538	8.2669	8.4356	8.2870	8.4802	8.0058	8.1773	8.2674	8.2802	8.3987	8.2735	8.4538	8.2669	8.4356	8.2870	8.4802	**8.11**
**H8**	4.6088	4.6617	4.6949	4.7134	4.7602	4.7093	4.8019	4.7054	4.7882	4.7177	4.7919	4.6088	4.6617	4.6949	4.7134	4.7602	4.7093	4.8019	4.7054	4.7882	4.7177	4.7919	**5.18**
**H(9A-9B)**	1.7161	1.7513	1.7594	1.7791	1.8050	1.7769	1.8071	1.7747	1.8006	1.7817	1.8378	1.7161	1.7513	1.7594	1.7791	1.8050	1.7769	1.8071	1.7747	1.8006	1.7817	1.8378	**2.05**
**H(10A)**	1.0061	0.8545	0.8220	0.8136	0.7285	0.8147	0.7778	0.8160	0.7734	0.8131	0.7805	1.0061	0.8545	0.8220	0.8136	0.7285	0.8147	0.7778	0.8160	0.7734	0.8131	0.7805	**1.14**
**H(10B)**	1.6552	1.5553	1.4937	1.4723	1.3892	1.4778	1.3845	1.4833	1.3914	1.4676	1.3570	1.6552	1.5553	1.4937	1.4723	1.3892	1.4778	1.3845	1.4833	1.3914	1.4676	1.3570	**1.39**
**H(11A-11B)**	1.0394	1.0539	1.0204	1.0676	1.0299	1.0668	1.0293	1.0659	1.0291	1.0689	1.0176	1.0394	1.0540	1.0204	1.0676	1.0299	1.0668	1.0293	1.0659	1.0291	1.0689	1.0176	**1.22**
**H(12A-12C)**	0.9080	0.8931	0.8684	0.8850	0.8458	0.8853	0.8530	0.8857	0.8533	0.8851	0.8480	0.9080	0.8930	0.8684	0.8850	0.8458	0.8853	0.8531	0.8857	0.8533	0.8851	0.8480	**0.77**
**H(1`A)**	2.7043	2.6996	2.6902	2.6911	2.6510	2.6915	2.6684	2.6920	2.6698	2.6911	2.6344	2.8345	2.8453	2.8343	2.8262	2.8010	2.8280	2.8117	2.8297	2.8145	2.8248	2.7764	**3.16**
**H(1`B)**	3.1997	3.2445	3.2806	3.2807	3.3411	3.2780	3.3284	3.2754	3.3259	3.2838	3.3220	2.8940	2.7339	2.7203	2.7054	2.7063	2.7059	2.6706	2.7066	2.6719	2.7055	2.6571	**3.76**
**H(2`A-2`B)**	1.7257	1.7567	1.7217	1.7895	1.7414	1.7870	1.7551	1.7846	1.7502	1.7924	1.7737	1.6056	1.6804	1.6425	1.7247	1.6775	1.7219	1.7071	1.7192	1.6994	1.7278	1.7414	**2.13**
**H(3`A-3`B)**	1.6056	1.6804	1.6425	1.7247	1.6775	1.7219	1.7071	1.7192	1.6994	1.7278	1.7414	1.7257	1.7567	1.7218	1.7895	1.7414	1.7870	1.7551	1.7846	1.7502	1.7924	1.7737	**2.05**
**H(4`A)**	2.8940	2.7339	2.7202	2.7054	2.7063	2.7059	2.6706	2.7066	2.6719	2.7055	2.6571	3.1997	3.2445	3.2806	3.2807	3.3411	3.2780	3.3285	3.2754	3.3259	3.2838	3.3220	**3.09**
**H(4`B)**	2.8345	2.8453	2.8343	2.8262	2.8010	2.8280	2.8117	2.8297	2.8145	2.8248	2.7764	2.7043	2.6996	2.6902	2.6911	2.6510	2.6915	2.6684	2.6920	2.6698	2.6911	2.6344	**3.63**
**F4**	-120.5398	-121.6484	-124.3709	-121.7179	-125.9690	-121.7225	-123.7522	-121.7262	-124.1160	-121.7121	-123.4809	-120.5398	-121.6485	-124.3711	-121.7180	-125.9690	-121.7225	-123.7523	-121.7262	-124.1161	-121.7121	-123.4809	**-104.5**
Nuclei	B972																						
**C1**	134.6971	135.8753	136.6169	136.6760	139.5533	136.6202	138.6512	136.5674	138.8176	136.7300	138.9783	134.6971	135.8753	136.6169	136.6759	139.5532	136.6203	138.6511	136.5674	138.8176	136.7300	138.9784	**132.8**
**C2**	136.1423	136.0686	136.6110	136.0524	136.6697	136.0535	137.1061	136.0530	137.0015	136.0477	137.0908	136.1423	136.0686	136.6111	136.0522	136.6695	136.0535	137.1058	136.0530	137.0015	136.0477	137.0908	**132.9**
**C3**	118.6863	119.3715	119.9318	119.6489	120.3301	119.6370	120.5956	119.6220	120.5384	119.6557	120.6719	118.6863	119.3715	119.9318	119.6490	120.3300	119.6370	120.5956	119.6220	120.5384	119.6557	120.6718	**117.2**
**C4**	170.5935	171.3556	171.9651	171.7320	172.1420	171.7133	172.7626	171.6911	172.6048	171.7444	172.8736	170.5934	171.3556	171.9650	171.7319	172.1420	171.7133	172.7627	171.6911	172.6048	171.7444	172.8736	**167.5**
**C5**	116.6790	117.8768	118.5101	118.5696	119.3676	118.5275	119.6225	118.4831	119.5235	118.6058	119.7745	116.6790	117.8768	118.5101	118.5698	119.3676	118.5275	119.6226	118.4831	119.5236	118.6058	119.7746	**117.2**
**C6**	132.2209	134.0034	135.0675	135.0035	135.8136	134.9461	136.4690	134.8843	136.2020	135.0514	136.7295	132.2209	134.0034	135.0674	135.0036	135.8136	134.9461	136.4689	134.8843	136.2019	135.0514	136.7297	**132.9**
**C7**	197.8670	202.6198	203.7484	205.3519	207.1219	205.1793	211.9055	205.0088	210.8523	205.5163	213.0007	197.8670	202.6198	203.7482	205.3522	207.1219	205.1793	211.9055	205.0088	210.8523	205.5163	213.0007	**195.8**
**C8**	63.5056	64.8160	65.4994	65.4760	66.7506	65.4415	67.2458	65.4046	67.0801	65.5049	67.6571	63.5056	64.8160	65.4994	65.4761	66.7505	65.4415	67.2458	65.4046	67.0801	65.5049	67.6571	**68.4**
**C9**	35.1659	34.9077	35.1070	34.8130	34.8409	34.8226	35.0047	34.8285	34.9106	34.7984	35.0884	35.1659	34.9077	35.1070	34.8131	34.8409	34.8226	35.0047	34.8285	34.9107	34.7984	35.0884	**30.5**
**C10**	27.4347	27.9105	28.3498	28.2459	28.8451	28.2248	28.6389	28.2010	28.6128	28.2615	28.8073	27.4347	27.9105	28.3499	28.2459	28.8452	28.2247	28.6389	28.2010	28.6128	28.2615	28.8074	**27.6**
**C11**	27.6676	27.7939	28.0328	27.8076	28.1669	27.8114	28.1400	27.8123	28.1369	27.7989	28.2738	27.6676	27.7939	28.0327	27.8075	28.1669	27.8114	28.1400	27.8123	28.1370	27.7989	28.2739	**23.1**
**C12**	16.4210	16.5676	16.6312	16.7389	16.8296	16.7272	16.8016	16.7136	16.8013	16.7461	16.8064	16.4210	16.5676	16.6312	16.7389	16.8296	16.7272	16.8016	16.7136	16.8013	16.7461	16.8064	**13.8**
**C1`**	50.3513	51.1355	51.3757	51.6819	52.1647	51.6473	52.1659	51.6111	52.1124	51.7109	52.6598	54.6831	55.7368	56.0738	56.2267	56.7429	56.2006	56.8535	56.1716	56.7908	56.2470	57.1038	**53.5**
**C2`**	25.1575	25.5809	25.9072	25.7805	26.1941	25.7710	26.2013	25.7588	26.1797	25.7847	25.9870	24.6675	24.8374	25.1474	25.0423	25.5381	25.0287	25.4907	25.0134	25.4932	25.0513	25.2788	**24.5**
**C3`**	24.6675	24.8374	25.1475	25.0423	25.5380	25.0287	25.4907	25.0134	25.4932	25.0513	25.2788	25.1574	25.5809	25.9072	25.7806	26.1941	25.7710	26.2013	25.7588	26.1797	25.7847	25.9870	**24.3**
**C4`**	54.6830	55.7368	56.0739	56.2268	56.7429	56.2006	56.8535	56.1716	56.7908	56.2470	57.1039	50.3513	51.1355	51.3758	51.6818	52.1647	51.6473	52.1659	51.6111	52.1125	51.7109	52.6599	**57.5**
**H2**	8.7007	8.5592	8.5908	8.4663	8.3592	8.4728	8.4141	8.4787	8.4039	8.4595	8.3953	8.7007	8.5592	8.5908	8.4663	8.3592	8.4728	8.4141	8.4787	8.4039	8.4595	8.3953	**8.11**
**H3**	7.3297	7.4738	7.5631	7.5139	7.6078	7.5126	7.6304	7.5107	7.6217	7.5149	7.6457	7.3297	7.4738	7.5631	7.5139	7.6078	7.5126	7.6304	7.5107	7.6217	7.5149	7.6457	**7.31**
**H5**	7.1325	7.2902	7.3841	7.3696	7.5156	7.3646	7.5407	7.3595	7.5307	7.3743	7.5618	7.1325	7.2902	7.3841	7.3696	7.5156	7.3646	7.5407	7.3595	7.5307	7.3743	7.5618	**7.31**
**H6**	8.0154	8.1828	8.2635	8.2621	8.3160	8.2580	8.3752	8.2534	8.3539	8.2656	8.3870	8.0154	8.1828	8.2635	8.2622	8.3160	8.2580	8.3752	8.2534	8.3539	8.2656	8.3870	**8.11**
**H8**	4.6168	4.6642	4.6860	4.7066	4.7485	4.7037	4.7874	4.7006	4.7743	4.7091	4.7672	4.6168	4.6642	4.6860	4.7066	4.7485	4.7037	4.7874	4.7006	4.7743	4.7091	4.7672	**5.18**
**H(9A-9B)**	1.6879	1.7170	1.7219	1.7279	1.6637	1.7278	1.6700	1.7273	1.6567	1.7274	1.7027	1.6879	1.7170	1.7218	1.7279	1.6637	1.7278	1.6700	1.7273	1.6567	1.7274	1.7027	**2.05**
**H(10A)**	0.9637	0.8246	0.7814	0.7958	0.7462	0.7959	0.7971	0.7962	0.7959	0.7958	0.8043	0.9637	0.8246	0.7814	0.7958	0.7462	0.7959	0.7971	0.7962	0.7959	0.7958	0.8043	**1.14**
**H(10B)**	1.6217	1.5183	1.4590	1.4425	1.3585	1.4475	1.3581	1.4523	1.3656	1.4377	1.3287	1.6217	1.5183	1.4591	1.4425	1.3585	1.4475	1.3581	1.4523	1.3656	1.4377	1.3287	**1.39**
**H(11A-11B)**	1.0221	1.0367	1.0037	1.0526	1.0131	1.0518	1.0176	1.0508	1.0171	1.0532	1.0044	1.0221	1.0367	1.0037	1.0526	1.0131	1.0518	1.0176	1.0508	1.0171	1.0532	1.0045	**1.22**
**H(12A-12C)**	0.9038	0.8919	0.8662	0.8885	0.8592	0.8886	0.8679	0.8886	0.8693	0.8883	0.8616	0.9038	0.8919	0.8662	0.8885	0.8592	0.8886	0.8679	0.8886	0.8693	0.8883	0.8616	**0.77**
**H(1`A)**	2.6968	2.6878	2.6766	2.6791	2.6372	2.6798	2.6542	2.6802	2.6545	2.6784	2.6219	2.8356	2.8425	2.8290	2.8230	2.7927	2.8246	2.8005	2.8261	2.8024	2.8212	2.7688	**3.16**
**H(1`B)**	3.1801	3.2276	3.2615	3.2635	3.3127	3.2613	3.3038	3.2588	3.2991	3.2655	3.3001	2.8657	2.7208	2.7098	2.7022	2.7288	2.7020	2.6867	2.7019	2.6919	2.7027	2.6798	**3.76**
**H(2`A-2`B)**	1.7040	1.7361	1.7020	1.7698	1.7248	1.7673	1.7377	1.7649	1.7341	1.7720	1.7559	1.5869	1.6654	1.6284	1.7161	1.6869	1.7127	1.7140	1.7093	1.7094	1.7193	1.7479	**2.13**
**H(3`A-3`B)**	1.5869	1.6654	1.6284	1.7161	1.6869	1.7127	1.7140	1.7093	1.7094	1.7193	1.7479	1.7040	1.7361	1.7020	1.7698	1.7248	1.7673	1.7377	1.7649	1.7341	1.7720	1.7559	**2.05**
**H(4`A)**	2.8657	2.7208	2.7099	2.7023	2.7288	2.7020	2.6867	2.7019	2.6919	2.7027	2.6798	3.1801	3.2276	3.2614	3.2635	3.3127	3.2613	3.3038	3.2588	3.2992	3.2655	3.3001	**3.09**
**H(4`B)**	2.8356	2.8425	2.8290	2.8230	2.7927	2.8246	2.8005	2.8261	2.8024	2.8212	2.7688	2.6968	2.6878	2.6766	2.6791	2.6372	2.6798	2.6542	2.6802	2.6545	2.6784	2.6220	**3.63**
**F4**	-114.3977	-115.6364	-118.2166	-115.8900	-120.4650	-115.8748	-118.2436	-115.8631	-118.6277	-115.9061	-118.1036	-114.3977	-115.6364	-118.2168	-115.8901	-120.4650	-115.8748	-118.2438	-115.8631	-118.6278	-115.9061	-118.1037	**-104.5**

[a] methyl protons shifts are averaged

[b] according to [6]

Table S6. Cont. III

Nuclei	R											S											Exp. [b]
	BHandH																						
	Gas	$CHCl_3$ (PCM)	$CHCl_3$ (SMD)	DMSO (PCM)	DMSO (SMD)	CH_3OH (PCM)	CH_3OH (SMD)	C_2H_5OH (PCM)	C_2H_5OH (SMD)	H_2O (PCM)	H_2O (SMD)	Gas	$CHCl_3$ (PCM)	$CHCl_3$ (SMD)	DMSO (PCM)	DMSO (SMD)	CH_3OH (PCM)	CH_3OH (SMD)	C_2H_5OH (PCM)	C_2H_5OH (SMD)	H_2O (PCM)	H_2O (SMD)	
**C1**	138.5465	139.0589	140.0282	139.2348	140.8674	139.2148	140.1811	139.2131	140.2774	139.2234	140.5547	138.5464	139.0589	140.0282	139.2348	139.9789	139.2149	140.1811	139.2130	140.2774	139.2234	140.5546	**132.8**
**C2**	143.5117	143.4353	144.0030	143.2870	143.8194	143.2877	144.2640	143.3068	144.2012	143.2560	144.2993	143.5117	143.4353	144.0030	143.2870	144.0311	143.2877	144.2640	143.3066	144.2012	143.2560	144.2993	**132.9**
**C3**	123.6216	124.3782	125.0268	124.6233	125.3404	124.6010	125.5677	124.5967	125.5216	124.6141	125.6558	123.6216	124.3782	125.0269	124.6233	125.3674	124.6011	125.5677	124.5967	125.5216	124.6141	125.6558	**117.2**
**C4**	174.7888	175.5536	176.1854	175.9620	176.5660	175.9263	177.2388	175.9091	177.0774	175.9658	177.3779	174.7888	175.5536	176.1854	175.9620	176.7061	175.9263	177.2388	175.9092	177.0774	175.9658	177.3779	**167.5**
**C5**	121.5046	122.9341	123.6411	123.6822	124.5984	123.6257	124.8174	123.5876	124.7076	123.7060	124.9940	121.5047	122.9341	123.6411	123.6822	124.4263	123.6257	124.8174	123.5877	124.7076	123.7060	124.9940	**117.2**
**C6**	139.7122	141.6732	142.8202	142.8468	144.3976	142.7615	145.0656	142.6949	144.7943	142.8980	145.4379	139.7122	141.6732	142.8202	142.8468	143.5909	142.7615	145.0656	142.6950	144.7943	142.8980	145.4378	**132.9**
**C7**	204.0554	208.3972	209.8032	210.7478	211.4240	210.5925	216.7982	210.4551	215.6159	210.8653	217.9800	204.0553	208.3972	209.8031	210.7478	211.4919	210.5925	216.7982	210.4554	215.6159	210.8653	217.9800	**195.8**
**C8**	60.0731	61.1362	61.9412	61.6394	62.5544	61.5974	63.2661	61.5739	63.1273	61.6492	63.7607	60.0731	61.1362	61.9412	61.6394	62.3835	61.5974	63.2661	61.5738	63.1273	61.6492	63.7607	**68.4**
**C9**	34.1020	33.8856	34.1209	33.7869	34.0297	33.7837	34.1234	33.7986	34.0914	33.7597	34.2791	34.1020	33.8856	34.1209	33.7869	34.5310	33.7838	34.1234	33.7987	34.0914	33.7597	34.2791	**30.5**
**C10**	27.2135	27.7537	28.1167	28.1371	28.8125	28.0995	28.5687	28.0810	28.5478	28.1431	28.8216	27.2135	27.7537	28.1167	28.1371	28.8812	28.0995	28.5687	28.0810	28.5478	28.1431	28.8216	**27.6**
**C11**	26.1644	26.2342	26.5746	26.1987	26.6023	26.1915	26.5850	26.2026	26.6070	26.1752	26.7570	26.1643	26.2342	26.5745	26.1987	26.9428	26.1914	26.5850	26.2025	26.6070	26.1752	26.7571	**23.1**
**C12**	15.6496	15.8310	15.8835	15.9590	16.0362	15.9394	15.9509	15.9385	15.9568	15.9476	15.9762	15.6495	15.8311	15.8834	15.9590	16.7031	15.9394	15.9509	15.9385	15.9568	15.9476	15.9762	**13.8**
**C1`**	48.2959	49.0980	49.4034	49.5638	50.0949	49.5213	50.0509	49.4977	50.0387	49.5750	50.4802	52.2406	53.2551	53.7083	53.7330	54.4771	53.6924	54.4518	53.6704	54.3989	53.7416	54.6924	**53.5**
**C2`**	21.6367	22.0035	22.3605	22.1802	22.6341	22.1578	22.7044	22.1539	22.7025	22.1716	22.6865	21.0585	21.3095	21.6337	21.4659	22.2100	21.4453	21.8796	21.4431	21.8815	21.4554	21.8474	**24.5**
**C3`**	21.0585	21.3095	21.6337	21.4659	21.9420	21.4453	21.8796	21.4431	21.8815	21.4554	21.8473	21.6367	22.0035	22.3605	22.1802	22.9243	22.1578	22.7044	22.1539	22.7025	22.1716	22.6865	**24.3**
**C4`**	52.2406	53.2551	53.7084	53.7330	54.3137	53.6925	54.4518	53.6705	54.3989	53.7416	54.6924	48.2958	49.0980	49.4034	49.5638	50.3079	49.5213	50.0509	49.4977	50.0387	49.5750	50.4801	**57.5**
**H2**	9.0133	8.9379	8.9431	8.8888	8.8838	8.8928	8.8900	8.8959	8.8935	8.8861	8.8693	9.0133	8.9379	8.9431	8.8888	8.8076	8.8928	8.8900	8.8959	8.8935	8.8861	8.8693	**8.11**
**H3**	7.4474	7.5927	7.6854	7.6383	7.7626	7.6369	7.7844	7.6346	7.7756	7.6405	7.8000	7.4474	7.5927	7.6854	7.6383	7.5571	7.6369	7.7844	7.6346	7.7756	7.6405	7.8000	**7.31**
**H5**	7.2803	7.4792	7.5728	7.5667	7.6983	7.5625	7.7142	7.5574	7.7025	7.5718	7.7363	7.2803	7.4792	7.5728	7.5667	7.4855	7.5625	7.7142	7.5574	7.7025	7.5718	7.7363	**7.31**
**H6**	8.1894	8.3806	8.4651	8.4861	8.6214	8.4803	8.6749	8.4737	8.6488	8.4926	8.7015	8.1894	8.3806	8.4651	8.4861	8.4049	8.4803	8.6749	8.4737	8.6488	8.4926	8.7015	**8.11**
**H8**	4.2693	4.3483	4.3771	4.4032	4.4839	4.4004	4.5358	4.3968	4.5082	4.4069	4.5389	4.2693	4.3483	4.3771	4.4032	4.3220	4.4004	4.5358	4.3968	4.5082	4.4069	4.5389	**5.18**
**H(9A-9B)**	1.7884	1.8248	1.8176	1.8632	1.8617	1.8613	1.8484	1.8587	1.8470	1.8660	1.8841	1.7883	1.8248	1.8176	1.8632	1.7820	1.8613	1.8484	1.8587	1.8470	1.8660	1.8841	**2.05**
**H(10A)**	0.9699	0.8271	0.7545	0.7425	0.6584	0.7484	0.6674	0.7535	0.6673	0.7379	0.6698	0.9699	0.8271	0.7545	0.7425	0.6584	0.7484	0.6674	0.7535	0.6673	0.7379	0.6698	**1.14**
**H(10B)**	1.6604	1.5673	1.5079	1.4980	1.4014	1.5040	1.3938	1.5089	1.4008	1.4933	1.3675	1.6604	1.5673	1.5080	1.4980	1.4014	1.5040	1.3938	1.5089	1.4008	1.4933	1.3675	**1.39**
**H(11A-11B)**	0.9886	1.0092	0.9788	1.0287	0.9857	1.0282	0.9820	1.0270	0.9828	1.0301	0.9726	0.9886	1.0092	0.9788	1.0287	0.9474	1.0282	0.9820	1.0270	0.9828	1.0301	0.9726	**1.22**
**H(12A-12C)**	0.9400	0.9236	0.8941	0.9103	0.8692	0.9121	0.8710	0.9129	0.8722	0.9096	0.8665	0.9400	0.9236	0.8941	0.9103	0.8291	0.9121	0.8710	0.9129	0.8722	0.9096	0.8665	**0.77**
**H(1`A)**	2.5921	2.5890	2.5638	2.5786	2.5274	2.5803	2.5416	2.5812	2.5420	2.5781	2.5258	2.7460	2.7455	2.7319	2.7273	2.6904	2.7296	2.7107	2.7311	2.7126	2.7260	2.6863	**3.16**
**H(1`B)**	3.0932	3.1308	3.1577	3.1643	3.2166	3.1627	3.1977	3.1603	3.1933	3.1669	3.2011	2.9077	2.7015	2.6593	2.6386	2.6117	2.6430	2.5508	2.6465	2.5576	2.6356	2.5450	**3.76**
**H(2`A-2`B)**	1.6826	1.7192	1.6777	1.7467	1.7009	1.7456	1.7098	1.7438	1.7053	1.7487	1.7284	1.5760	1.6449	1.6083	1.6820	1.6381	1.6803	1.6740	1.6779	1.6668	1.6846	1.7083	**2.13**
**H(3`A-3`B)**	1.5760	1.6449	1.6083	1.6820	1.6381	1.6803	1.6740	1.6779	1.6668	1.6846	1.7083	1.6826	1.7192	1.6777	1.7467	1.7009	1.7456	1.7098	1.7438	1.7053	1.7487	1.7284	**2.05**
**H(4`A)**	2.9077	2.7015	2.6593	2.6386	2.6117	2.6430	2.5508	2.6465	2.5576	2.6356	2.5450	3.0932	3.1308	3.1577	3.1643	3.2166	3.1627	3.1977	3.1603	3.1933	3.1669	3.2011	**3.09**
**H(4`B)**	2.7460	2.7455	2.7320	2.7273	2.6904	2.7296	2.7107	2.7311	2.7126	2.7260	2.6863	2.5921	2.5890	2.5638	2.5786	2.5274	2.5803	2.5416	2.5812	2.5420	2.5781	2.5258	**3.63**
**F4**	-105.4115	-106.0759	-108.2201	-105.9738	-109.3505	-105.9854	-107.2526	-105.9960	-107.6314	-105.9624	-107.1068	-105.4115	-106.0759	-108.2201	-105.9738	-106.3105	-105.9854	-107.2526	-105.9960	-107.6314	-105.9624	-107.1068	**-104.5**
Nuclei	BHandHLYP																						
**C1**	139.1706	139.9116	140.6432	140.3296	141.7914	140.3148	140.9419	140.3012	141.1074	140.3424	141.2702	139.1706	139.9116	140.6432	140.3295	141.7914	140.3148	140.9419	140.3012	141.1074	140.3424	141.2702	**132.8**
**C2**	142.0275	141.9201	142.3974	141.7860	142.2101	141.7956	142.7562	141.8074	142.6462	141.7766	142.7360	142.0275	141.9201	142.3974	141.7860	142.2101	141.7956	142.7562	141.8074	142.6462	141.7767	142.7360	**132.9**
**C3**	122.5523	123.1684	123.6876	123.3576	123.9512	123.3499	124.1309	123.3441	124.1042	123.3640	124.2025	122.5523	123.1684	123.6877	123.3575	123.9512	123.3499	124.1309	123.3441	124.1042	123.3640	124.2025	**117.2**
**C4**	174.5864	175.2899	175.8454	175.6713	176.1994	175.6486	176.8750	175.6280	176.7138	175.6927	176.9999	174.5864	175.2899	175.8454	175.6714	176.1994	175.6486	176.8750	175.6280	176.7138	175.6927	176.9999	**167.5**
**C5**	120.3052	121.5543	122.1308	122.2063	122.9812	122.1668	123.1213	122.1296	123.0494	122.2436	123.2920	120.3052	121.5543	122.1307	122.2064	122.9812	122.1668	123.1213	122.1296	123.0494	122.2436	123.2920	**117.2**
**C6**	138.0548	139.9133	141.0549	141.0312	142.5246	140.9560	143.2797	140.8840	142.9881	141.1031	143.6154	138.0548	139.9133	141.0548	141.0312	142.5246	140.9560	143.2797	140.8840	142.9881	141.1031	143.6154	**132.9**
**C7**	202.3068	207.1882	208.3236	209.9129	210.2715	209.7555	215.9054	209.5991	214.6690	210.0607	217.0820	202.3068	207.1882	208.3236	209.9129	210.2715	209.7555	215.9054	209.5991	214.6690	210.0607	217.0820	**195.8**
**C8**	61.8804	62.9219	63.4905	63.3415	64.2714	63.3206	64.7791	63.3010	64.6980	63.3599	65.1757	61.8804	62.9219	63.4905	63.3415	64.2714	63.3206	64.7791	63.3010	64.6980	63.3599	65.1757	**68.4**
**C9**	34.1386	33.7725	34.0157	33.6193	33.9168	33.6264	34.0171	33.6361	33.9882	33.6121	34.0669	34.1386	33.7725	34.0157	33.6193	33.9168	33.6264	34.0171	33.6361	33.9882	33.6121	34.0669	**30.5**
**C10**	27.2497	27.6893	28.1703	28.0164	28.7326	27.9923	28.5308	27.9711	28.5292	28.0393	28.6777	27.2497	27.6893	28.1703	28.0164	28.7326	27.9922	28.5308	27.9711	28.5292	28.0393	28.6777	**27.6**
**C11**	27.2405	27.3246	27.6178	27.2971	27.7280	27.3011	27.7115	27.3070	27.7273	27.2928	27.8503	27.2405	27.3246	27.6179	27.2972	27.7280	27.3010	27.7115	27.3070	27.7273	27.2928	27.8503	**23.1**
**C12**	16.4477	16.5986	16.6969	16.7338	16.8490	16.7238	16.7955	16.7165	16.8029	16.7434	16.8068	16.4477	16.5986	16.6969	16.7339	16.8490	16.7239	16.7955	16.7165	16.8029	16.7434	16.8068	**13.8**
**C1`**	49.0825	49.8396	50.1387	50.3717	50.8895	50.3364	50.8814	50.3041	50.8419	50.4053	51.3647	53.3202	54.3295	54.6354	54.7498	55.2630	54.7265	55.3513	54.7053	55.3102	54.7713	55.6150	**53.5**
**C2`**	24.4610	24.8112	25.1567	24.9344	25.4163	24.9287	25.4177	24.9250	25.4350	24.9393	25.2394	23.9171	24.0510	24.4256	24.1914	24.6650	24.1829	24.5877	24.1770	24.5932	24.1990	24.4088	**24.5**
**C3`**	23.9171	24.0510	24.4257	24.1913	24.6650	24.1830	24.5877	24.1770	24.5932	24.1990	24.4088	24.4610	24.8112	25.1568	24.9345	25.4163	24.9287	25.4177	24.9250	25.4350	24.9393	25.2394	**24.3**
**C4`**	53.3202	54.3295	54.6354	54.7499	55.2630	54.7265	55.3513	54.7053	55.3102	54.7714	55.6150	49.0825	49.8396	50.1387	50.3716	50.8895	50.3365	50.8814	50.3041	50.8419	50.4053	51.3647	**57.5**
**H2**	8.9174	8.8134	8.8432	8.7435	8.7514	8.7471	8.7822	8.7508	8.7728	8.7400	8.7555	8.9174	8.8134	8.8432	8.7434	8.7514	8.7471	8.7822	8.7508	8.7728	8.7400	8.7555	**8.11**
**H3**	7.4415	7.5833	7.6682	7.6294	7.7421	7.6275	7.7648	7.6254	7.7556	7.6312	7.7794	7.4415	7.5833	7.6682	7.6294	7.7421	7.6275	7.7648	7.6254	7.7556	7.6312	7.7794	**7.31**
**H5**	7.2649	7.4404	7.5319	7.5137	7.6436	7.5094	7.6523	7.5050	7.6416	7.5178	7.6758	7.2649	7.4404	7.5319	7.5137	7.6436	7.5094	7.6523	7.5050	7.6416	7.5178	7.6758	**7.31**
**H6**	8.0825	8.2754	8.3711	8.3872	8.5176	8.3804	8.5843	8.3735	8.5554	8.3937	8.6106	8.0825	8.2754	8.3711	8.3872	8.5176	8.3804	8.5843	8.3735	8.5554	8.3937	8.6106	**8.11**
**H8**	4.2250	4.3093	4.3596	4.3771	4.4432	4.3727	4.5018	4.3684	4.4740	4.3812	4.4872	4.2250	4.3093	4.3596	4.3771	4.4432	4.3728	4.5018	4.3684	4.4740	4.3812	4.4872	**5.18**
**H(9A-9B)**	1.5827	1.6221	1.6279	1.6514	1.6739	1.6488	1.6780	1.6462	1.6704	1.6538	1.7051	1.5827	1.6221	1.6279	1.6514	1.6739	1.6488	1.6780	1.6462	1.6704	1.6538	1.7051	**2.05**
**H(10A)**	0.9846	0.8118	0.7997	0.7653	0.6810	0.7674	0.7233	0.7695	0.7123	0.7634	0.7225	0.9846	0.8118	0.7997	0.7653	0.6810	0.7674	0.7233	0.7695	0.7123	0.7634	0.7225	**1.14**
**H(10B)**	1.5615	1.4724	1.4065	1.3921	1.3035	1.3980	1.3085	1.4037	1.3114	1.3864	1.2811	1.5615	1.4724	1.4065	1.3921	1.3035	1.3980	1.3085	1.4037	1.3114	1.3864	1.2811	**1.39**
**H(11A-11B)**	0.9843	1.0042	0.9708	1.0203	0.9796	1.0195	0.9842	1.0186	0.9801	1.0210	0.9727	0.9843	1.0042	0.9708	1.0203	0.9796	1.0195	0.9842	1.0186	0.9801	1.0210	0.9727	**1.22**
**H(12A-12C)**	0.9401	0.9227	0.8984	0.9153	0.8731	0.9159	0.8823	0.9165	0.8805	0.9146	0.8760	0.9401	0.9227	0.8984	0.9153	0.8731	0.9159	0.8823	0.9165	0.8805	0.9146	0.8760	**0.77**
**H(1`A)**	2.6097	2.6012	2.5884	2.5909	2.5431	2.5917	2.5662	2.5924	2.5652	2.5900	2.5385	2.7507	2.7499	2.7315	2.7241	2.6891	2.7264	2.7061	2.7286	2.7091	2.7217	2.6739	**3.16**
**H(1`B)**	3.0196	3.0719	3.1020	3.1129	3.1735	3.1101	3.1671	3.1072	3.1606	3.1156	3.1693	2.7250	2.5707	2.5548	2.5480	2.5462	2.5484	2.5090	2.5489	2.5117	2.5477	2.5052	**3.76**
**H(2`A-2`B)**	1.6503	1.6861	1.6501	1.7217	1.6694	1.7194	1.6853	1.7170	1.6787	1.7240	1.7029	1.5294	1.6090	1.5670	1.6561	1.6040	1.6535	1.6361	1.6508	1.6287	1.6585	1.6693	**2.13**
**H(3`A-3`B)**	1.5294	1.6090	1.5670	1.6561	1.6040	1.6535	1.6361	1.6508	1.6287	1.6585	1.6693	1.6503	1.6861	1.6501	1.7217	1.6694	1.7194	1.6853	1.7170	1.6787	1.7240	1.7029	**2.05**
**H(4`A)**	2.7250	2.5707	2.5548	2.5480	2.5462	2.5484	2.5090	2.5489	2.5117	2.5477	2.5052	3.0196	3.0719	3.1020	3.1129	3.1735	3.1101	3.1671	3.1072	3.1606	3.1156	3.1693	**3.09**
**H(4`B)**	2.7507	2.7499	2.7315	2.7241	2.6891	2.7264	2.7061	2.7286	2.7091	2.7217	2.6739	2.6097	2.6012	2.5884	2.5908	2.5431	2.5917	2.5662	2.5924	2.5652	2.5900	2.5385	**3.63**
**F4**	-109.0357	-110.2556	-112.5349	-110.4787	-114.0316	-110.4726	-112.0026	-110.4662	-112.3770	-110.4837	-111.8763	-109.0357	-110.2556	-112.5350	-110.4787	-114.0316	-110.4726	-112.0026	-110.4662	-112.3770	-110.4836	-111.8763	**-104.5**

[a] methyl protons shifts are averaged
[b] according to [6]

Table S6. Cont. IV

Nuclei	R											S											Exp. [b]
	BLYP																						
	Gas	$CHCl_3$ (PCM)	$CHCl_3$ (SMD)	DMSO (PCM)	DMSO (SMD)	CH_3OH (PCM)	CH_3OH (SMD)	C_2H_5OH (PCM)	C_2H_5OH (SMD)	H_2O (PCM)	H_2O (SMD)	Gas	$CHCl_3$ (PCM)	$CHCl_3$ (SMD)	DMSO (PCM)	DMSO (SMD)	CH_3OH (PCM)	CH_3OH (SMD)	C_2H_5OH (PCM)	C_2H_5OH (SMD)	H_2O (PCM)	H_2O (SMD)	
**C1**	139.5343	140.9652	141.5943	141.7357	144.5172	141.9636	143.4256	141.7300	143.9164	142.1462	143.7361	139.5343	140.9652	141.5943	141.7357	144.5172	141.9636	143.4256	141.7300	143.9164	142.1461	143.7361	**132.8**
**C2**	137.2556	137.2722	137.8854	137.3003	138.1317	137.5735	138.4112	137.3854	138.4615	137.6688	138.5024	137.2556	137.2722	137.8854	137.3003	138.1317	137.5735	138.4112	137.3854	138.4615	137.6689	138.5024	**132.9**
**C3**	120.7921	121.4191	121.9349	121.5949	122.2771	121.8658	122.4301	121.6744	122.5403	121.9644	122.5819	120.7921	121.4191	121.9349	121.5949	122.2771	121.8658	122.4301	121.6744	122.5403	121.9643	122.5819	**117.2**
**C4**	178.0446	178.9278	179.6458	179.2623	179.9892	179.5224	180.4161	179.3202	180.4077	179.6422	180.6443	178.0446	178.9278	179.6458	179.2623	179.9892	179.5224	180.4161	179.3202	180.4077	179.6422	180.6443	**167.5**
**C5**	118.7346	119.8592	120.4200	120.4485	121.2822	120.6894	121.4129	120.4685	121.4684	120.8468	121.6561	118.7346	119.8592	120.4200	120.4485	121.2822	120.6894	121.4129	120.4685	121.4684	120.8467	121.6561	**117.2**
**C6**	133.6680	135.5732	136.6203	136.5089	137.5639	136.7306	138.0767	136.4903	137.9461	136.9249	138.5039	133.6680	135.5732	136.6203	136.5089	137.5639	136.7306	138.0767	136.4903	137.9461	136.9250	138.5039	**132.9**
**C7**	204.1746	208.9870	210.1011	211.5855	213.4733	211.7040	217.6195	211.3603	217.0831	212.0989	218.7030	204.1746	208.9870	210.1011	211.5855	213.4733	211.7040	217.6195	211.3603	217.0831	212.0991	218.7030	**195.8**
**C8**	70.4591	71.9072	72.3407	72.5539	73.6351	72.8057	73.9567	72.5927	74.1428	72.9387	74.2784	70.4591	71.9072	72.3407	72.5539	73.6351	72.8057	73.9567	72.5927	74.1428	72.9386	74.2784	**68.4**
**C9**	40.1256	39.7708	39.8045	39.5605	39.5512	39.8527	39.6759	39.6825	39.8226	39.9098	39.7834	40.1256	39.7708	39.8045	39.5605	39.5512	39.8527	39.6759	39.6825	39.8226	39.9098	39.7834	**30.5**
**C10**	30.9956	31.5038	31.9353	31.7631	32.4330	32.0244	32.0419	31.8239	32.2296	32.1419	32.2060	30.9956	31.5038	31.9353	31.7631	32.4330	32.0244	32.0419	31.8239	32.2296	32.1419	32.2060	**27.6**
**C11**	31.2026	31.3178	31.5898	31.2585	31.7489	31.5442	31.5381	31.3673	31.7410	31.6138	31.7406	31.2026	31.3178	31.5898	31.2585	31.7489	31.5442	31.5381	31.3673	31.7410	31.6138	31.7406	**23.1**
**C12**	17.0012	17.1470	17.1923	17.2337	17.4506	17.5040	17.2382	17.3127	17.4172	17.6046	17.3304	17.0012	17.1470	17.1923	17.2337	17.4506	17.5040	17.2382	17.3127	17.4172	17.6045	17.3304	**13.8**
**C1`**	55.2831	56.0370	56.4053	56.5833	57.2950	56.8244	57.1633	56.6042	57.2744	56.9826	57.7328	59.9830	61.1273	61.5437	61.6339	62.2990	61.8849	62.3006	61.6734	62.3750	62.0222	62.6696	**53.5**
**C2`**	27.7274	28.2834	28.6122	28.5322	29.0355	28.8001	28.9330	28.6054	29.0959	28.9041	28.8068	27.6644	27.8009	28.1574	27.9578	28.6760	28.2245	28.3891	28.0299	28.5704	28.3322	28.2523	**24.5**
**C3`**	27.6644	27.8009	28.1574	27.9578	28.6760	28.2246	28.3891	28.0299	28.5704	28.3322	28.2523	27.7274	28.2834	28.6122	28.5322	29.0355	28.8002	28.9330	28.6054	29.0959	28.9042	28.8068	**24.3**
**C4`**	59.9830	61.1273	61.5437	61.6339	62.2990	61.8849	62.3006	61.6734	62.3750	62.0222	62.6696	55.2831	56.0370	56.4053	56.5833	57.2950	56.8243	57.1633	56.6042	57.2744	56.9825	57.7328	**57.5**
**H2**	8.4743	8.3172	8.3808	8.2334	8.1741	8.2858	8.4013	8.2457	8.2052	8.2841	8.4152	8.4743	8.3172	8.3808	8.2334	8.1741	8.2858	8.4013	8.2457	8.2052	8.2841	8.4152	**8.11**
**H3**	7.1772	7.3089	7.3990	7.3393	7.4354	7.3857	7.6265	7.3394	7.4492	7.3955	7.6556	7.1772	7.3089	7.3990	7.3393	7.4354	7.3857	7.6265	7.3394	7.4492	7.3955	7.6556	**7.31**
**H5**	6.9737	7.1133	7.1996	7.1888	7.3510	7.2313	7.5411	7.1813	7.3621	7.2487	7.5789	6.9737	7.1133	7.1996	7.1888	7.3510	7.2313	7.5411	7.1813	7.3621	7.2487	7.5789	**7.31**
**H6**	7.8508	8.0008	8.0879	8.0664	8.1493	8.1100	8.3792	8.0612	8.1751	8.1252	8.4240	7.8508	8.0008	8.0879	8.0664	8.1493	8.1100	8.3792	8.0612	8.1751	8.1252	8.4240	**8.11**
**H8**	4.9846	5.0134	5.0311	5.0158	5.0719	5.0626	5.2534	5.0171	5.0718	5.0716	5.2461	4.9846	5.0134	5.0311	5.0158	5.0719	5.0626	5.2534	5.0171	5.0718	5.0716	5.2461	**5.18**
**H(9A-9B)**	1.7832	1.8231	1.8147	1.8352	1.7750	1.8823	1.9577	1.8368	1.7845	1.8907	1.9990	1.7832	1.8231	1.8147	1.8352	1.7750	1.8823	1.9577	1.8368	1.7845	1.8908	1.9990	**2.05**
**H(10A)**	1.0374	0.9218	0.9214	0.8984	0.8849	0.9453	1.0941	0.9001	0.9201	0.9545	1.1206	1.0374	0.9218	0.9214	0.8984	0.8849	0.9453	1.0941	0.9001	0.9201	0.9545	1.1206	**1.14**
**H(10B)**	1.7538	1.6293	1.5697	1.5413	1.4721	1.5943	1.6301	1.5547	1.4719	1.5913	1.6106	1.7538	1.6293	1.5697	1.5413	1.4721	1.5943	1.6301	1.5547	1.4719	1.5913	1.6106	**1.39**
**H(11A-11B)**	1.1322	1.1424	1.1178	1.1560	1.1325	1.2024	1.3013	1.1563	1.1316	1.2123	1.3029	1.1322	1.1424	1.1178	1.1560	1.1325	1.2024	1.3013	1.1563	1.1316	1.2123	1.3029	**1.22**
**H(12A-12C)**	0.9419	0.9312	0.9110	0.9251	0.9070	0.9727	1.0813	0.9279	0.9137	0.9802	1.0909	0.9419	0.9312	0.9110	0.9251	0.9070	0.9727	1.0813	0.9279	0.9137	0.9802	1.0909	**0.77**
**H(1`A)**	2.8633	2.8501	2.8310	2.8377	2.7979	2.8860	2.9760	2.8418	2.8118	2.8922	2.9544	2.9651	2.9898	2.9671	2.9680	2.9372	3.0180	3.1018	2.9754	2.9444	3.0205	3.0695	**3.16**
**H(1`B)**	3.3633	3.4089	3.4348	3.4379	3.4925	3.4835	3.6471	3.4365	3.4756	3.4949	3.6586	3.0499	2.8913	2.8353	2.8526	2.8547	2.9002	2.9868	2.8556	2.8331	2.9080	2.9912	**3.76**
**H(2`A-2`B)**	1.8103	1.8387	1.8009	1.8621	1.8241	1.9073	2.0025	1.8602	1.8309	1.9195	2.0403	1.6657	1.7499	1.7162	1.7953	1.7725	1.8397	1.9626	1.7916	1.7906	1.8535	2.0090	**2.13**
**H(3`A-3`B)**	1.6657	1.7499	1.7162	1.7953	1.7725	1.8397	1.9626	1.7916	1.7906	1.8535	2.0090	1.8103	1.8387	1.8009	1.8621	1.8241	1.9073	2.0025	1.8602	1.8309	1.9195	2.0403	**2.05**
**H(4`A)**	3.0499	2.8913	2.8353	2.8526	2.8547	2.9002	2.9868	2.8556	2.8331	2.9080	2.9912	3.3633	3.4089	3.4348	3.4379	3.4925	3.4835	3.6471	3.4365	3.4756	3.4949	3.6586	**3.09**
**H(4`B)**	2.9651	2.9898	2.9671	2.9680	2.9372	3.0180	3.1018	2.9754	2.9444	3.0205	3.0695	2.8633	2.8501	2.8310	2.8377	2.7979	2.8860	2.9760	2.8418	2.8118	2.8922	2.9544	**3.63**
**F4**	-142.5703	-144.4267	-147.7209	-144.8583	-150.4862	-144.6289	-146.8437	-144.8151	-148.3973	-144.8127	-147.0218	-142.5703	-144.4267	-147.7209	-144.8583	-150.4862	-144.6289	-146.8437	-144.8151	-148.3973	-144.8126	-147.0218	**-104.5**
Nuclei	BMK																						
**C1**	154.6743	155.0332	155.8462	155.1583	157.1596	155.1396	156.3883	155.3301	156.3397	155.3339	156.6046	154.6743	155.0332	155.8461	155.1582	157.1596	155.1397	156.3884	155.3301	156.3396	155.3339	156.6046	**132.8**
**C2**	154.6877	154.7844	155.5459	154.6549	155.3498	154.6489	155.8469	154.8543	155.6657	154.8228	155.7777	154.6877	154.7844	155.5461	154.6548	155.3498	154.6489	155.8467	154.8543	155.6656	154.8230	155.7777	**132.9**
**C3**	135.9138	136.6373	137.3465	136.8279	137.6933	136.8045	137.9405	136.9917	137.7955	137.0117	137.8683	135.9138	136.6373	137.3465	136.8279	137.6933	136.8046	137.9404	136.9917	137.7954	137.0118	137.8683	**117.2**
**C4**	189.4877	190.2116	190.9837	190.5712	191.4900	190.5337	192.1186	190.7070	191.8573	190.7682	192.2003	189.4877	190.2116	190.9838	190.5712	191.4900	190.5337	192.1187	190.7070	191.8575	190.7681	192.2003	**167.5**
**C5**	133.6513	134.9680	135.5869	135.6169	136.6787	135.5615	136.8546	135.7170	136.6382	135.8301	136.9525	133.6513	134.9680	135.5868	135.6171	136.6787	135.5614	136.8546	135.7170	136.6383	135.8300	136.9525	**117.2**
**C6**	151.3083	153.3283	154.3456	154.4536	156.4249	154.3671	157.0666	154.4912	156.6478	154.6954	157.4119	151.3083	153.3283	154.3454	154.4537	156.4249	154.3671	157.0667	154.4912	156.6476	154.6955	157.4119	**132.9**
**C7**	218.4275	222.9521	224.3909	225.3913	226.5095	225.2230	232.5082	225.2643	230.9435	225.7107	234.0514	218.4275	222.9521	224.3912	225.3913	226.5095	225.2230	232.5081	225.2643	230.9437	225.7103	234.0514	**195.8**
**C8**	71.7818	72.7560	73.5185	73.2212	74.7487	73.1831	75.2261	73.3534	74.8979	73.4147	76.0495	71.7818	72.7560	73.5184	73.2212	74.7487	73.1831	75.2261	73.3534	74.8979	73.4148	76.0495	**68.4**
**C9**	38.9270	38.8753	38.9914	38.7229	39.3377	38.7191	39.5697	38.9259	39.3287	38.8870	39.8348	38.9270	38.8753	38.9914	38.7229	39.3377	38.7192	39.5696	38.9259	39.3287	38.8870	39.8348	**30.5**
**C10**	31.5063	31.8431	32.2898	32.1042	32.9319	32.0706	32.5932	32.2494	32.5113	32.3000	32.8436	31.5063	31.8431	32.2898	32.1043	32.9319	32.0705	32.5931	32.2494	32.5113	32.3000	32.8436	**27.6**
**C11**	30.1638	30.0934	30.5280	30.0558	30.6708	30.0424	30.6321	30.2397	30.5415	30.2295	30.7089	30.1638	30.0934	30.5281	30.0558	30.6708	30.0423	30.6321	30.2397	30.5416	30.2295	30.7089	**23.1**
**C12**	17.9216	17.9663	18.1186	18.0457	18.3196	18.0223	18.2191	18.2104	18.1235	18.2298	18.1813	17.9215	17.9663	18.1186	18.0458	18.3196	18.0222	18.2190	18.2104	18.1236	18.2298	18.1812	**13.8**
**C1`**	56.2140	56.7042	57.2624	57.3354	57.9775	57.2761	57.8726	57.4271	57.7807	57.5515	57.9072	60.6747	61.2631	61.7498	61.5953	62.2577	61.5585	62.3486	61.7326	62.2082	61.7911	62.6655	**53.5**
**C2`**	28.1109	28.2884	28.6899	28.4501	29.0183	28.4238	28.9833	28.6085	28.8847	28.6357	28.9334	27.2299	27.4471	27.8451	27.6148	28.2163	27.5901	28.0996	27.7764	28.0059	27.7997	28.0827	**24.5**
**C3`**	27.2299	27.4471	27.8451	27.6148	28.2163	27.5901	28.0997	27.7764	28.0059	27.7998	28.0827	28.1109	28.2884	28.6900	28.4501	29.0184	28.4240	28.9833	28.6085	28.8847	28.6357	28.9334	**24.3**
**C4`**	60.6747	61.2631	61.7497	61.5952	62.2576	61.5585	62.3485	61.7326	62.2080	61.7910	62.6655	56.2140	56.7042	57.2624	57.3355	57.9775	57.2760	57.8726	57.4271	57.7808	57.5514	57.9072	**57.5**
**H2**	8.9918	8.9298	8.9868	8.9078	8.9029	8.9057	8.9653	8.9038	8.9648	8.9005	8.9628	8.9918	8.9298	8.9868	8.9078	8.9029	8.9057	8.9653	8.9038	8.9648	8.9005	8.9628	**8.11**
**H3**	7.6541	7.7829	7.8752	7.8302	7.9474	7.8246	7.9758	7.8190	7.9680	7.8259	7.9947	7.6541	7.7829	7.8752	7.8302	7.9474	7.8246	7.9758	7.8190	7.9680	7.8259	7.9947	**7.31**
**H5**	7.4938	7.6754	7.7641	7.7548	7.8712	7.7469	7.8878	7.7390	7.8788	7.7528	7.9076	7.4938	7.6754	7.7641	7.7548	7.8712	7.7469	7.8878	7.7390	7.8788	7.7528	7.9076	**7.31**
**H6**	8.3432	8.5078	8.6605	8.6235	8.7819	8.6124	8.8278	8.6015	8.8045	8.6250	8.8898	8.3432	8.5078	8.6605	8.6235	8.7819	8.6123	8.8278	8.6015	8.8044	8.6250	8.8898	**8.11**
**H8**	4.4144	4.4718	4.5598	4.5582	4.6028	4.5477	4.6667	4.5377	4.6543	4.5594	4.6485	4.4144	4.4718	4.5598	4.5582	4.6028	4.5477	4.6667	4.5377	4.6543	4.5594	4.6485	**5.18**
**H(9A-9B)**	1.6329	1.6976	1.6442	1.7193	1.7410	1.7145	1.7510	1.7099	1.7352	1.7143	1.8258	1.6329	1.6976	1.6442	1.7193	1.7410	1.7145	1.7510	1.7099	1.7352	1.7143	1.8258	**2.05**
**H(10A)**	1.0054	0.8156	0.8417	0.7556	0.6584	0.7548	0.7160	0.7541	0.7163	0.7469	0.7118	1.0054	0.8156	0.8417	0.7556	0.6584	0.7548	0.7160	0.7541	0.7163	0.7469	0.7118	**1.14**
**H(10B)**	1.5665	1.5142	1.4152	1.4197	1.3176	1.4233	1.3145	1.4271	1.3225	1.4069	1.3045	1.5665	1.5142	1.4152	1.4197	1.3176	1.4234	1.3145	1.4271	1.3225	1.4069	1.3045	**1.39**
**H(11A-11B)**	0.9568	0.9779	0.9496	0.9998	0.9492	0.9949	0.9534	0.9903	0.9549	0.9951	0.9505	0.9568	0.9779	0.9497	0.9998	0.9492	0.9949	0.9534	0.9903	0.9550	0.9951	0.9505	**1.22**
**H(12A-12C)**	0.8618	0.8474	0.8194	0.8457	0.7952	0.8425	0.7967	0.8395	0.8006	0.8395	0.8000	0.8617	0.8474	0.8194	0.8457	0.7952	0.8425	0.7967	0.8395	0.8007	0.8395	0.8000	**0.77**
**H(1`A)**	2.6238	2.6293	2.5970	2.6303	2.5835	2.6272	2.6067	2.6240	2.6082	2.6234	2.5463	2.7737	2.7853	2.7534	2.7712	2.7426	2.7699	2.7487	2.7683	2.7505	2.7626	2.7334	**3.16**
**H(1`B)**	3.0937	3.1310	3.1474	3.1725	3.2050	3.1661	3.1984	3.1595	3.1935	3.1689	3.1717	2.7218	2.5991	2.5747	2.5696	2.5484	2.5668	2.5121	2.5643	2.5131	2.5631	2.5093	**3.76**
**H(2`A-2`B)**	1.6783	1.7136	1.6893	1.7597	1.7017	1.7529	1.7160	1.7464	1.7135	1.7566	1.7210	1.4626	1.5173	1.4830	1.5420	1.4897	1.5377	1.5230	1.5334	1.5190	1.5364	1.5474	**2.13**
**H(3`A-3`B)**	1.5702	1.6353	1.5892	1.6789	1.6292	1.6732	1.6559	1.6675	1.6508	1.6746	1.6903	1.6422	1.7002	1.6581	1.7449	1.6934	1.7389	1.7144	1.7329	1.7103	1.7409	1.7414	**2.05**
**H(4`A)**	2.7218	2.5991	2.5747	2.5696	2.5484	2.5668	2.5120	2.5643	2.5131	2.5631	2.5093	2.4219	2.4555	2.4526	2.5090	2.4952	2.5014	2.4953	2.4940	2.4913	2.5066	2.4820	**3.09**
**H(4`B)**	2.7737	2.7853	2.7534	2.7712	2.7426	2.7699	2.7487	2.7683	2.7505	2.7626	2.7334	2.6238	2.6293	2.5970	2.6303	2.5835	2.6272	2.6068	2.6240	2.6082	2.6235	2.5463	**3.63**
**F4**	-118.0260	-119.1096	-121.7794	-119.1175	-123.2437	-119.1250	-121.0201	-119.1311	-121.3999	-119.1100	-120.7181	-118.0260	-119.1096	-121.7793	-119.1175	-123.2437	-119.1250	-121.0200	-119.1311	-121.3999	-119.1101	-120.7181	**-104.5**

[a] methyl protons shifts are averaged

[b] according to [6]

Table S6. Cont. V

Nuclei	R											S											Exp. [b]
	BP86																						
	Gas	$CHCl_3$ (PCM)	$CHCl_3$ (SMD)	DMSO (PCM)	DMSO (SMD)	CH_3OH (PCM)	CH_3OH (SMD)	C_2H_5OH (PCM)	C_2H_5OH (SMD)	H_2O (PCM)	H_2O (SMD)	Gas	$CHCl_3$ (PCM)	$CHCl_3$ (SMD)	DMSO (PCM)	DMSO (SMD)	CH_3OH (PCM)	CH_3OH (SMD)	C_2H_5OH (PCM)	C_2H_5OH (SMD)	H_2O (PCM)	H_2O (SMD)	
**C1**	136.1088	137.3107	138.0964	137.7831	139.1376	137.7682	138.6145	137.7476	138.6273	137.7918	138.9792	136.1088	137.3107	138.0965	137.7831	139.1376	137.7682	138.6145	137.7476	138.6273	137.7918	138.9792	**132.8**
**C2**	135.7786	135.9025	136.4035	135.7790	136.2500	135.7953	136.7578	135.8067	136.6297	135.7589	136.8526	135.7786	135.9025	136.4034	135.7790	136.2500	135.7953	136.7578	135.8067	136.6297	135.7589	136.8526	**132.9**
**C3**	119.6549	120.5419	121.0715	120.7433	121.3759	120.7419	121.7193	120.7350	121.6286	120.7394	121.9674	119.6549	120.5419	121.0715	120.7433	121.3759	120.7419	121.7193	120.7350	121.6286	120.7394	121.9674	**117.2**
**C4**	174.5771	175.6557	176.2903	176.0366	176.7190	176.0219	177.3180	176.0021	177.1695	176.0455	177.6153	174.5771	175.6557	176.2903	176.0366	176.7190	176.0219	177.3180	176.0021	177.1695	176.0455	177.6153	**167.5**
**C5**	117.7054	119.0766	119.6753	119.6781	120.4892	119.6486	120.7558	119.6139	120.6434	119.7013	121.0874	117.7054	119.0766	119.6753	119.6781	120.4892	119.6486	120.7558	119.6139	120.6434	119.7013	121.0874	**117.2**
**C6**	132.2467	134.2864	135.3229	135.3472	136.8781	135.2854	137.5200	135.2189	137.2791	135.4016	138.0862	132.2467	134.2864	135.3229	135.3472	136.8781	135.2854	137.5200	135.2189	137.2791	135.4016	138.0862	**132.9**
**C7**	201.2567	205.6591	206.8764	207.8846	208.7243	207.7624	212.8796	207.6342	211.9294	207.9947	214.0254	201.2567	205.6591	206.8764	207.8846	208.7243	207.7624	212.8796	207.6342	211.9294	207.9947	214.0254	**195.8**
**C8**	68.1361	69.5249	70.2639	70.0439	71.1738	70.0330	71.7242	70.0145	71.5644	70.0470	72.2963	68.1361	69.5249	70.2639	70.0439	71.1738	70.0330	71.7242	70.0145	71.5644	70.0470	72.2963	**68.4**
**C9**	38.8652	38.7336	38.8380	38.5423	38.8956	38.5618	39.1809	38.5758	39.0858	38.5184	39.4764	38.8652	38.7336	38.8380	38.5423	38.8956	38.5619	39.1809	38.5758	39.0858	38.5184	39.4764	**30.5**
**C10**	29.5240	30.1529	30.5449	30.4255	31.1246	30.4126	30.9274	30.3953	30.9087	30.4332	31.2594	29.5240	30.1529	30.5449	30.4255	31.1246	30.4126	30.9274	30.3953	30.9087	30.4332	31.2594	**27.6**
**C11**	29.4418	29.6972	29.8600	29.5824	29.9487	29.6022	29.9365	29.6159	29.9309	29.5580	30.2368	29.4418	29.6972	29.8601	29.5824	29.9487	29.6021	29.9365	29.6159	29.9309	29.5580	30.2368	**23.1**
**C12**	16.5296	16.7824	16.7604	16.8352	16.8320	16.8373	16.8060	16.8346	16.7990	16.8289	16.9542	16.5296	16.7824	16.7604	16.8352	16.8320	16.8373	16.8060	16.8346	16.7990	16.8290	16.9543	**13.8**
**C1`**	53.7220	54.6467	54.8434	55.1464	55.6019	55.1212	55.6068	55.0916	55.5138	55.1663	56.3013	58.2667	59.6104	59.9327	60.0596	60.4729	60.0450	60.6886	60.0246	60.6186	60.0678	61.1204	**53.5**
**C2`**	26.2439	26.8649	27.1493	27.0337	27.4594	27.0338	27.4989	27.0283	27.4820	27.0283	27.4483	25.9185	26.2129	26.4676	26.3545	26.7189	26.3512	26.7270	26.3431	26.7097	26.3531	26.7220	**24.5**
**C3`**	25.9185	26.2129	26.4677	26.3545	26.7189	26.3512	26.7270	26.3431	26.7097	26.3531	26.7220	26.2439	26.8649	27.1491	27.0337	27.4594	27.0338	27.4989	27.0283	27.4820	27.0283	27.4483	**24.3**
**C4`**	58.2667	59.6104	59.9326	60.0596	60.4729	60.0450	60.6886	60.0246	60.6186	60.0678	61.1203	53.7220	54.6467	54.8434	55.1464	55.6019	55.1212	55.6068	55.0916	55.5138	55.1663	56.3012	**57.5**
**H2**	8.5202	8.4179	8.4255	8.3541	8.3445	8.3584	8.3761	8.3625	8.3748	8.3498	8.3764	8.5202	8.4179	8.4255	8.3541	8.3445	8.3584	8.3761	8.3625	8.3748	8.3498	8.3764	**8.11**
**H3**	7.1794	7.3318	7.4109	7.3778	7.4831	7.3764	7.5086	7.3746	7.4988	7.3787	7.5375	7.1794	7.3318	7.4109	7.3778	7.4831	7.3764	7.5086	7.3746	7.4988	7.3787	7.5375	**7.31**
**H5**	7.0044	7.1582	7.2405	7.2245	7.3556	7.2214	7.3589	7.2179	7.3494	7.2272	7.4008	7.0044	7.1582	7.2405	7.2245	7.3556	7.2214	7.3589	7.2179	7.3494	7.2272	7.4008	**7.31**
**H6**	7.9135	8.0785	8.1409	8.1670	8.2621	8.1621	8.3188	8.1568	8.3018	8.1713	8.3592	7.9135	8.0785	8.1409	8.1670	8.2621	8.1621	8.3188	8.1568	8.3018	8.1713	8.3592	**8.11**
**H8**	4.9923	5.0231	5.0204	5.0488	5.0657	5.0471	5.0991	5.0452	5.0903	5.0501	5.0979	4.9923	5.0231	5.0204	5.0488	5.0657	5.0471	5.0991	5.0452	5.0903	5.0501	5.0979	**5.18**
**H(9A-9B)**	1.8866	1.9233	1.9092	1.9458	1.9709	1.9451	1.9663	1.9441	1.9592	1.9462	2.0157	1.8866	1.9233	1.9092	1.9458	1.9709	1.9451	1.9663	1.9441	1.9592	1.9462	2.0157	**2.05**
**H(10A)**	1.0160	0.9024	0.8508	0.8713	0.7697	0.8724	0.8287	0.8734	0.8187	0.8700	0.8515	1.0160	0.9024	0.8508	0.8713	0.7697	0.8724	0.8287	0.8734	0.8187	0.8700	0.8515	**1.14**
**H(10B)**	1.7876	1.6671	1.5902	1.5808	1.4877	1.5871	1.4750	1.5931	1.4819	1.5746	1.4536	1.7876	1.6671	1.5902	1.5808	1.4877	1.5871	1.4750	1.5931	1.4819	1.5746	1.4535	**1.39**
**H(11A-11B)**	1.1095	1.1243	1.0787	1.1354	1.0880	1.1356	1.0865	1.1355	1.0849	1.1348	1.0877	1.1095	1.1243	1.0788	1.1354	1.0880	1.1356	1.0865	1.1355	1.0849	1.1348	1.0877	**1.22**
**H(12A-12C)**	0.9116	0.9045	0.8683	0.8963	0.8471	0.8975	0.8543	0.8985	0.8534	0.8948	0.8614	0.9116	0.9045	0.8683	0.8963	0.8471	0.8975	0.8543	0.8985	0.8534	0.8948	0.8614	**0.77**
**H(1`A)**	2.8037	2.7998	2.7761	2.7875	2.7401	2.7890	2.7544	2.7902	2.7555	2.7857	2.7327	2.9270	2.9443	2.9250	2.9302	2.8972	2.9326	2.9032	2.9346	2.9064	2.9275	2.8769	**3.16**
**H(1`B)**	3.3751	3.4176	3.4367	3.4453	3.4990	3.4441	3.4838	3.4427	3.4815	3.4461	3.4880	3.1070	2.9109	2.8780	2.8605	2.8494	2.8627	2.8030	2.8649	2.8065	2.8583	2.7978	**3.76**
**H(2`A-2`B)**	1.8041	1.8324	1.7866	1.8585	1.8009	1.8572	1.8134	1.8556	1.8078	1.8595	1.8493	1.6627	1.7393	1.6959	1.7821	1.7232	1.7802	1.7512	1.7780	1.7430	1.7836	1.8013	**2.13**
**H(3`A-3`B)**	1.6627	1.7393	1.6959	1.7821	1.7232	1.7802	1.7512	1.7780	1.7430	1.7836	1.8013	1.8041	1.8324	1.7866	1.8585	1.8009	1.8572	1.8134	1.8556	1.8078	1.8595	1.8493	**2.05**
**H(4`A)**	3.1070	2.9109	2.8780	2.8605	2.8494	2.8627	2.8030	2.8649	2.8065	2.8583	2.7978	3.3751	3.4176	3.4367	3.4453	3.4990	3.4441	3.4838	3.4427	3.4815	3.4461	3.4880	**3.09**
**H(4`B)**	2.9270	2.9443	2.9250	2.9302	2.8972	2.9326	2.9032	2.9346	2.9064	2.9275	2.8769	2.8037	2.7998	2.7761	2.7875	2.7401	2.7890	2.7544	2.7902	2.7555	2.7857	2.7327	**3.63**
**F4**	-133.7224	-134.7487	-137.9211	-134.7351	-139.6524	-134.7459	-136.9829	-134.7552	-137.4182	-134.7242	-136.5988	-133.7224	-134.7487	-137.9211	-134.7351	-139.6524	-134.7459	-136.9829	-134.7552	-137.4182	-134.7242	-136.5988	**-104.5**
Nuclei	**CAM-B3LYP**																						
**C1**	140.9000	141.8642	142.5134	142.3393	143.9931	142.3245	143.2389	142.3087	143.3053	142.3544	143.4213	140.8999	141.8642	142.5135	142.3392	143.9931	142.3245	143.2389	142.3087	143.3053	142.3544	143.4212	**132.8**
**C2**	142.3592	142.3291	142.7581	142.1837	142.6753	142.1979	143.2028	142.2124	143.0855	142.1723	143.1775	142.3594	142.3291	142.7582	142.1837	142.6754	142.1979	143.2028	142.2124	143.0855	142.1723	143.1775	**132.9**
**C3**	123.8875	124.5615	125.0610	124.7361	125.3833	124.7328	125.5918	124.7293	125.5399	124.7413	125.6630	123.8876	124.5615	125.0610	124.7363	125.3832	124.7328	125.5918	124.7293	125.5399	124.7412	125.6631	**117.2**
**C4**	177.7476	178.5115	179.0552	178.8743	179.4733	178.8556	180.0785	178.8375	179.9280	178.8943	180.2203	177.7477	178.5115	179.0551	178.8744	179.4733	178.8556	180.0785	178.8375	179.9280	178.8944	180.2204	**167.5**
**C5**	121.8135	123.1003	123.6648	123.7417	124.5660	123.7060	124.7320	123.6710	124.6384	123.7775	124.8939	121.8135	123.1003	123.6648	123.7415	124.5660	123.7060	124.7320	123.6709	124.6384	123.7775	124.8939	**117.2**
**C6**	138.4687	140.3307	141.5035	141.4320	142.9843	141.3615	143.6195	141.2922	143.3572	141.5021	144.0436	138.4687	140.3307	141.5034	141.4319	142.9843	141.3615	143.6195	141.2922	143.3572	141.5021	144.0435	**132.9**
**C7**	206.2190	211.2347	212.3397	213.9879	214.6304	213.8311	220.0283	213.6740	218.8123	214.1375	221.0855	206.2190	211.2347	212.3397	213.9878	214.6304	213.8311	220.0283	213.6739	218.8123	214.1374	221.0856	**195.8**
**C8**	64.6063	65.7712	66.4410	66.2572	67.2927	66.2389	67.9060	66.2194	67.7480	66.2753	68.2828	64.6064	65.7712	66.4410	66.2573	67.2928	66.2389	67.9060	66.2194	67.7480	66.2753	68.2828	**68.4**
**C9**	35.3917	35.0454	35.2739	34.8733	35.1875	34.8869	35.3176	34.9005	35.2558	34.8622	35.4063	35.3917	35.0454	35.2740	34.8733	35.1874	34.8869	35.3176	34.9006	35.2558	34.8622	35.4064	**30.5**
**C10**	28.1866	28.6440	29.1029	28.9663	29.6973	28.9451	29.5046	28.9250	29.4847	28.9892	29.6822	28.1867	28.6440	29.1029	28.9664	29.6974	28.9451	29.5046	28.9249	29.4847	28.9892	29.6823	**27.6**
**C11**	27.8708	27.9976	28.2402	27.9403	28.3933	27.9501	28.3825	27.9597	28.3778	27.9329	28.5418	27.8708	27.9976	28.2403	27.9403	28.3933	27.9501	28.3825	27.9596	28.3778	27.9330	28.5418	**23.1**
**C12**	16.2567	16.4391	16.4726	16.5524	16.6588	16.5468	16.6036	16.5421	16.5992	16.5601	16.6163	16.2567	16.4391	16.4726	16.5523	16.6589	16.5468	16.6036	16.5420	16.5992	16.5601	16.6163	**13.8**
**C1`**	50.8435	51.6933	51.8918	52.2148	52.7698	52.1836	52.7649	52.1535	52.6947	52.2469	53.2563	55.1554	56.2469	56.5379	56.6717	57.1952	56.6514	57.3190	56.6314	57.2584	56.6928	57.5773	**53.5**
**C2`**	25.0342	25.4284	25.7358	25.5492	26.0245	25.5467	26.0407	25.5443	26.0345	25.5535	25.8833	24.4922	24.6878	24.9644	24.8119	25.2810	24.8071	25.1973	24.8028	25.1947	24.8187	25.0647	**24.5**
**C3`**	24.4923	24.6878	24.9643	24.8118	25.2810	24.8071	25.1973	24.8028	25.1947	24.8188	25.0648	25.0342	25.4285	25.7358	25.5492	26.0245	25.5467	26.0407	25.5444	26.0345	25.5534	25.8833	**24.3**
**C4`**	55.1553	56.2469	56.5378	56.6716	57.1952	56.6514	57.3190	56.6314	57.2584	56.6927	57.5773	50.8435	51.6933	51.8918	52.2147	52.7697	52.1836	52.7649	52.1534	52.6947	52.2470	53.2562	**57.5**
**H2**	8.8102	8.6986	8.7289	8.6236	8.6210	8.6275	8.6425	8.6316	8.6418	8.6199	8.6323	8.8102	8.6986	8.7289	8.6236	8.6210	8.6275	8.6425	8.6316	8.6418	8.6199	8.6322	**8.11**
**H3**	7.4041	7.5438	7.6298	7.5866	7.7036	7.5847	7.7229	7.5828	7.7152	7.5883	7.7368	7.4041	7.5438	7.6298	7.5866	7.7036	7.5847	7.7229	7.5828	7.7152	7.5883	7.7368	**7.31**
**H5**	7.2406	7.4128	7.5085	7.4855	7.6154	7.4812	7.6240	7.4771	7.6145	7.4895	7.6484	7.2405	7.4128	7.5085	7.4855	7.6154	7.4812	7.6240	7.4771	7.6145	7.4895	7.6484	**7.31**
**H6**	8.0497	8.2319	8.3255	8.3359	8.4632	8.3292	8.5140	8.3227	8.4924	8.3422	8.5479	8.0497	8.2319	8.3255	8.3359	8.4632	8.3292	8.5140	8.3227	8.4924	8.3422	8.5479	**8.11**
**H8**	4.4210	4.4951	4.5329	4.5563	4.6154	4.5520	4.6593	4.5478	4.6382	4.5605	4.6576	4.4210	4.4951	4.5329	4.5563	4.6153	4.5520	4.6593	4.5478	4.6382	4.5605	4.6576	**5.18**
**H(9A-9B)**	1.6746	1.7066	1.7208	1.7333	1.7558	1.7312	1.7557	1.7292	1.7500	1.7352	1.7902	1.6746	1.7066	1.7208	1.7333	1.7558	1.7312	1.7557	1.7292	1.7500	1.7352	1.7902	**2.05**
**H(10A)**	0.9793	0.8280	0.7905	0.7827	0.6958	0.7844	0.7355	0.7864	0.7297	0.7811	0.7458	0.9793	0.8280	0.7905	0.7827	0.6958	0.7844	0.7355	0.7864	0.7297	0.7811	0.7458	**1.14**
**H(10B)**	1.6254	1.5324	1.4640	1.4472	1.3642	1.4530	1.3590	1.4589	1.3655	1.4418	1.3321	1.6253	1.5324	1.4640	1.4472	1.3642	1.4530	1.3590	1.4589	1.3655	1.4418	1.3321	**1.39**
**H(11A-11B)**	1.0293	1.0490	1.0127	1.0612	1.0239	1.0605	1.0223	1.0600	1.0216	1.0618	1.0127	1.0293	1.0490	1.0127	1.0612	1.0239	1.0605	1.0223	1.0600	1.0216	1.0618	1.0127	**1.22**
**H(12A-12C)**	0.9496	0.9353	0.9077	0.9264	0.8870	0.9270	0.8927	0.9277	0.8929	0.9257	0.8870	0.9496	0.9353	0.9077	0.9264	0.8871	0.9270	0.8927	0.9277	0.8929	0.9257	0.8870	**0.77**
**H(1`A)**	2.7110	2.7033	2.6912	2.6925	2.6485	2.6932	2.6648	2.6941	2.6655	2.6917	2.6365	2.8448	2.8448	2.8307	2.8212	2.7916	2.8233	2.8027	2.8254	2.8061	2.8191	2.7687	**3.16**
**H(1`B)**	3.1229	3.1689	3.2079	3.2088	3.2727	3.2060	3.2605	3.2034	3.2561	3.2114	3.2635	2.8211	2.6606	2.6465	2.6360	2.6365	2.6367	2.5996	2.6376	2.6000	2.6354	2.5884	**3.76**
**H(2`A-2`B)**	1.7172	1.7531	1.7166	1.7866	1.7389	1.7843	1.7511	1.7822	1.7461	1.7888	1.7689	1.5984	1.6798	1.6380	1.7262	1.6814	1.7235	1.7120	1.7209	1.7036	1.7287	1.7421	**2.13**
**H(3`A-3`B)**	1.5984	1.6798	1.6380	1.7262	1.6814	1.7235	1.7120	1.7209	1.7036	1.7287	1.7422	1.7172	1.7532	1.7166	1.7866	1.7389	1.7843	1.7511	1.7822	1.7461	1.7887	1.7689	**2.05**
**H(4`A)**	2.8211	2.6606	2.6465	2.6360	2.6365	2.6367	2.5996	2.6376	2.6000	2.6354	2.5884	3.1229	3.1689	3.2079	3.2088	3.2727	3.2060	3.2605	3.2033	3.2561	3.2114	3.2635	**3.09**
**H(4`B)**	2.8448	2.8448	2.8307	2.8212	2.7916	2.8233	2.8027	2.8254	2.8061	2.8191	2.7687	2.7110	2.7033	2.6912	2.6925	2.6485	2.6932	2.6648	2.6941	2.6655	2.6917	2.6364	**3.63**
**F4**	-115.7732	-117.3132	-119.8083	-117.6410	-121.5488	-117.6299	-119.5812	-117.6175	-119.8864	-117.6514	-119.3760	-115.7731	-117.3132	-119.8084	-117.6410	-121.5487	-117.6299	-119.5812	-117.6176	-119.8864	-117.6513	-119.3760	**-104.5**

[a] methyl protons shifts are averaged

[b] according to [6]

Table S6. Cont. VI

Nuclei	R											S											Exp. [b]
	HF																						
	Gas	$CHCl_3$ (PCM)	$CHCl_3$ (SMD)	DMSO (PCM)	DMSO (SMD)	CH_3OH (PCM)	CH_3OH (SMD)	C_2H_5OH (PCM)	C_2H_5OH (SMD)	H_2O (PCM)	H_2O (SMD)	Gas	$CHCl_3$ (PCM)	$CHCl_3$ (SMD)	DMSO (PCM)	DMSO (SMD)	CH_3OH (PCM)	CH_3OH (SMD)	C_2H_5OH (PCM)	C_2H_5OH (SMD)	H_2O (PCM)	H_2O (SMD)	
**C1**	136.4614	137.5594	137.9396	138.2608	141.1588	138.1812	139.8214	138.1121	140.2588	138.3352	140.0156	136.4614	137.5594	137.9396	138.2608	141.1590	138.1812	139.8214	138.1123	140.2588	138.3352	140.0156	**132.8**
**C2**	144.3880	144.1261	144.6323	143.8817	144.0884	143.9028	144.6434	143.9234	144.4625	143.8613	144.6473	144.3881	144.1261	144.6323	143.8817	144.0886	143.9028	144.6434	143.9233	144.4625	143.8613	144.6474	**132.9**
**C3**	120.4753	121.0267	121.4081	121.1842	121.9458	121.1734	121.9413	121.1639	121.9814	121.1933	121.9865	120.4753	121.0267	121.4081	121.1842	121.9457	121.1734	121.9413	121.1637	121.9814	121.1933	121.9866	**117.2**
**C4**	171.1212	171.5718	172.1467	171.7971	172.0666	171.7897	172.8696	171.7808	172.6355	171.8033	173.0028	171.1213	171.5718	172.1467	171.7971	172.0666	171.7897	172.8696	171.7807	172.6354	171.8033	173.0028	**167.5**
**C5**	117.8593	119.1512	119.5795	119.8198	120.6684	119.7762	120.6727	119.7340	120.6376	119.8599	120.8287	117.8593	119.1512	119.5795	119.8198	120.6682	119.7762	120.6727	119.7339	120.6376	119.8599	120.8287	**117.2**
**C6**	139.9930	141.4756	142.7481	142.2938	142.9370	142.2616	143.9248	142.2248	143.4971	142.3244	144.2237	139.9930	141.4756	142.7481	142.2938	142.9368	142.2616	143.9248	142.2249	143.4971	142.3244	144.2236	**132.9**
**C7**	196.4929	202.2641	202.9719	205.5898	206.7292	205.3508	213.4358	205.1213	212.0643	205.8156	214.4845	196.4932	202.2641	202.9719	205.5898	206.7294	205.3508	213.4358	205.1213	212.0643	205.8152	214.4845	**195.8**
**C8**	55.7610	56.8718	57.1616	57.3487	58.2739	57.3120	58.7513	57.2794	58.6807	57.3824	58.9817	55.7610	56.8718	57.1617	57.3487	58.2738	57.3120	58.7513	57.2794	58.6807	57.3825	58.9816	**68.4**
**C9**	29.9395	29.4452	29.6637	29.2244	29.3513	29.2420	29.2641	29.2585	29.2555	29.2074	29.2254	29.9396	29.4452	29.6637	29.2244	29.3513	29.2420	29.2641	29.2587	29.2555	29.2074	29.2254	**30.5**
**C10**	24.5112	24.8855	25.3200	25.1270	25.7191	25.1133	25.5163	25.0997	25.4870	25.1397	25.6454	24.5113	24.8855	25.3200	25.1270	25.7191	25.1133	25.5163	25.0997	25.4869	25.1397	25.6454	**27.6**
**C11**	24.9934	25.0012	25.2811	24.9675	25.3997	24.9711	25.3586	24.9750	25.3631	24.9631	25.4875	24.9934	25.0012	25.2811	24.9675	25.3997	24.9711	25.3586	24.9751	25.3632	24.9630	25.4875	**23.1**
**C12**	16.1893	16.3050	16.4087	16.4083	16.5840	16.3998	16.5424	16.3920	16.5403	16.4161	16.5585	16.1893	16.3050	16.4087	16.4083	16.5840	16.3998	16.5424	16.3920	16.5403	16.4161	16.5585	**13.8**
**C1`**	44.6495	45.3638	45.5995	45.8292	46.3745	45.7972	46.3770	45.7670	46.3398	45.8597	46.7427	48.5492	49.4089	49.6427	49.7948	50.2999	49.7716	50.3741	49.7486	50.3061	49.8158	50.6100	**53.5**
**C2`**	22.8232	23.0884	23.3924	23.1170	23.5554	23.1184	23.5262	23.1198	23.5079	23.1145	23.3375	22.1997	22.2737	22.6410	22.3541	22.9562	22.3468	22.8286	22.3405	22.8432	22.3604	22.6316	**24.5**
**C3`**	22.1997	22.2737	22.6410	22.3541	22.9561	22.3468	22.8286	22.3405	22.8431	22.3603	22.6316	22.8232	23.0884	23.3924	23.1170	23.5554	23.1184	23.5262	23.1197	23.5079	23.1145	23.3375	**24.3**
**C4`**	48.5492	49.4089	49.6427	49.7948	50.3000	49.7716	50.3741	49.7487	50.3060	49.8158	50.6100	44.6494	45.3638	45.5995	45.8291	46.3746	45.7972	46.3770	45.7670	46.3397	45.8597	46.7426	**57.5**
**H2**	9.0711	8.9082	8.9926	8.7974	8.6994	8.8084	8.7504	8.8181	8.7213	8.7871	8.7457	9.0711	8.9082	8.9926	8.7974	8.6994	8.8084	8.7504	8.8181	8.7212	8.7871	8.7457	**8.11**
**H3**	7.4358	7.5753	7.6646	7.6196	7.7433	7.6175	7.7557	7.6152	7.7493	7.6217	7.7711	7.4358	7.5753	7.6646	7.6196	7.7433	7.6175	7.7557	7.6152	7.7493	7.6217	7.7711	**7.31**
**H5**	7.2401	7.4378	7.5258	7.5268	7.6588	7.5216	7.6718	7.5162	7.6614	7.5317	7.6960	7.2401	7.4378	7.5258	7.5268	7.6588	7.5216	7.6718	7.5162	7.6614	7.5317	7.6960	**7.31**
**H6**	8.0965	8.3044	8.4316	8.4110	8.5225	8.4061	8.5996	8.4004	8.5605	8.4158	8.6363	8.0965	8.3044	8.4316	8.4110	8.5225	8.4061	8.5996	8.4004	8.5606	8.4158	8.6363	**8.11**
**H8**	3.6381	3.7437	3.8354	3.8239	3.9329	3.8188	4.0011	3.8133	3.9706	3.8288	3.9979	3.6381	3.7437	3.8354	3.8239	3.9329	3.8188	4.0011	3.8133	3.9706	3.8288	3.9979	**5.18**
**H(9A-9B)**	1.3084	1.3301	1.3445	1.3363	1.2736	1.3379	1.2820	1.3390	1.2675	1.3349	1.3033	1.3084	1.3301	1.3445	1.3363	1.2736	1.3379	1.2820	1.3390	1.2675	1.3349	1.3033	**2.05**
**H(10A)**	0.9656	0.7280	0.7412	0.6826	0.6635	0.6833	0.6994	0.6843	0.7008	0.6822	0.7041	0.9656	0.7280	0.7412	0.6826	0.6635	0.6833	0.6994	0.6843	0.7008	0.6822	0.7041	**1.14**
**H(10B)**	1.3770	1.3012	1.2518	1.2206	1.1379	1.2267	1.1517	1.2323	1.1569	1.2149	1.1303	1.3771	1.3012	1.2518	1.2206	1.1379	1.2267	1.1517	1.2323	1.1568	1.2149	1.1303	**1.39**
**H(11A-11B)**	0.8644	0.8763	0.8546	0.8936	0.8559	0.8927	0.8656	0.8916	0.8636	0.8945	0.8622	0.8644	0.8763	0.8546	0.8936	0.8559	0.8927	0.8656	0.8917	0.8636	0.8944	0.8622	**1.22**
**H(12A-12C)**	0.9257	0.9009	0.8816	0.8957	0.8713	0.8956	0.8816	0.8955	0.8831	0.8958	0.8787	0.9257	0.9009	0.8817	0.8957	0.8713	0.8956	0.8816	0.8955	0.8831	0.8958	0.8787	**0.77**
**H(1`A)**	2.3729	2.3640	2.3490	2.3541	2.3106	2.3546	2.3370	2.3550	2.3368	2.3537	2.3150	2.5341	2.5327	2.5042	2.4991	2.4550	2.5016	2.4708	2.5039	2.4741	2.4967	2.4443	**3.16**
**H(1`B)**	2.7192	2.7822	2.8168	2.8250	2.8747	2.8223	2.8737	2.8195	2.8658	2.8276	2.8825	2.3793	2.2880	2.2538	2.2872	2.3250	2.2852	2.2832	2.2834	2.2964	2.2895	2.2833	**3.76**
**H(2`A-2`B)**	1.4856	1.5304	1.4937	1.5699	1.5249	1.5670	1.5387	1.5640	1.5343	1.5727	1.5563	1.3833	1.4798	1.4293	1.5356	1.5073	1.5313	1.5400	1.5271	1.5358	1.5397	1.5700	**2.13**
**H(3`A-3`B)**	1.3833	1.4798	1.4293	1.5356	1.5073	1.5313	1.5400	1.5270	1.5358	1.5397	1.5700	1.4856	1.5304	1.4937	1.5699	1.5249	1.5670	1.5387	1.5640	1.5343	1.5727	1.5563	**2.05**
**H(4`A)**	2.3793	2.2880	2.2538	2.2872	2.3250	2.2852	2.2832	2.2834	2.2964	2.2895	2.2833	2.7192	2.7822	2.8168	2.8250	2.8747	2.8223	2.8737	2.8195	2.8658	2.8276	2.8825	**3.09**
**H(4`B)**	2.5341	2.5327	2.5042	2.4991	2.4550	2.5016	2.4708	2.5039	2.4741	2.4967	2.4443	2.3728	2.3640	2.3490	2.3541	2.3106	2.3546	2.3370	2.3550	2.3368	2.3537	2.3150	**3.63**
**F4**	-92.4769	-93.7757	-95.4120	-94.1485	-97.5580	-94.1133	-95.6118	-94.0822	-96.0599	-94.1816	-95.4835	-92.4768	-93.7757	-95.4120	-94.1485	-97.5580	-94.1133	-95.6118	-94.0823	-96.0600	-94.1816	-95.4835	**-104.5**
Nuclei	LC-BLYP																						
**C1**	144.3028	145.1950	146.1042	145.8010	147.4677	145.7589	146.7872	145.7404	146.8037	145.8203	147.0726	144.3028	145.1949	146.1041	145.8010	147.4677	145.7589	146.7872	145.7405	146.8037	145.8203	147.0725	**132.8**
**C2**	148.6053	148.4336	148.9601	148.2830	148.7557	148.2707	149.2718	148.2857	149.1701	148.2777	149.2677	148.6053	148.4336	148.9602	148.2830	148.7557	148.2707	149.2718	148.2859	149.1701	148.2778	149.2676	**132.9**
**C3**	128.2541	128.9264	129.5380	129.1390	129.8089	129.1086	129.9774	129.1049	129.9340	129.1500	130.0396	128.2541	128.9264	129.5380	129.1390	129.8089	129.1086	129.9774	129.1048	129.9339	129.1500	130.0397	**117.2**
**C4**	181.9735	182.5089	183.1029	182.8610	183.3907	182.8163	184.0004	182.7995	183.8613	182.8865	184.1304	181.9735	182.5091	183.1027	182.8610	183.3907	182.8164	184.0004	182.7995	183.8613	182.8865	184.1304	**167.5**
**C5**	126.1791	127.4745	128.1385	128.2234	129.0703	128.1579	129.2286	128.1194	129.1271	128.2680	129.3934	126.1791	127.4745	128.1384	128.2234	129.0703	128.1579	129.2287	128.1195	129.1270	128.2679	129.3934	**117.2**
**C6**	144.2567	146.1931	147.4116	147.2757	148.9440	147.1795	149.5452	147.1123	149.2785	147.3512	149.9505	144.2567	146.1930	147.4116	147.2757	148.9440	147.1795	149.5451	147.1124	149.2785	147.3512	149.9505	**132.9**
**C7**	211.8303	217.0927	218.4451	220.2118	220.7174	220.0151	226.8416	219.8419	225.4392	220.3777	228.0439	211.8303	217.0927	218.4455	220.2118	220.7174	220.0150	226.8416	219.8416	225.4392	220.3777	228.0436	**195.8**
**C8**	62.4946	63.6122	64.3010	64.2400	65.1246	64.1886	65.9586	64.1624	65.7354	64.2696	66.4491	62.4946	63.6122	64.3010	64.2400	65.1246	64.1886	65.9586	64.1624	65.7354	64.2696	66.4492	**68.4**
**C9**	33.0906	32.7128	32.9583	32.5697	32.8735	32.5573	33.0162	32.5722	32.9124	32.5640	33.1062	33.0906	32.7128	32.9583	32.5697	32.8734	32.5573	33.0162	32.5722	32.9124	32.5640	33.1062	**30.5**
**C10**	26.7174	27.0997	27.5416	27.4779	28.1510	27.4294	27.9545	27.4087	27.9216	27.5068	28.1708	26.7174	27.0997	27.5417	27.4779	28.1510	27.4295	27.9545	27.4088	27.9216	27.5068	28.1708	**27.6**
**C11**	25.6629	25.6738	26.0213	25.6762	26.0987	25.6556	26.0916	25.6622	26.0813	25.6781	26.2660	25.6628	25.6738	26.0212	25.6762	26.0987	25.6557	26.0917	25.6622	26.0813	25.6781	26.2660	**23.1**
**C12**	15.2825	15.3973	15.4777	15.5469	15.6332	15.5145	15.5678	15.5098	15.5562	15.5604	15.5999	15.2825	15.3974	15.4776	15.5468	15.6333	15.5145	15.5678	15.5098	15.5562	15.5604	15.5999	**13.8**
**C1`**	48.8633	49.5850	49.8719	50.1266	50.6951	50.0699	50.6789	50.0406	50.6292	50.1636	51.1331	52.7573	53.6750	54.0588	54.1648	54.6804	54.1139	54.7997	54.0904	54.7504	54.1956	55.0687	**53.5**
**C2`**	23.0097	23.2733	23.5886	23.4460	23.8619	23.4152	23.9107	23.4114	23.8912	23.4575	23.8212	22.3648	22.4849	22.8031	22.6272	23.0962	22.5957	22.9834	22.5919	22.9779	22.6398	22.9003	**24.5**
**C3`**	22.3648	22.4849	22.8032	22.6272	23.0962	22.5957	22.9834	22.5918	22.9779	22.6398	22.9003	23.0098	23.2733	23.5887	23.4460	23.8619	23.4152	23.9107	23.4114	23.8911	23.4575	23.8213	**24.3**
**C4`**	52.7572	53.6750	54.0587	54.1648	54.6804	54.1139	54.7997	54.0903	54.7504	54.1956	55.0688	48.8633	49.5850	49.8719	50.1267	50.6951	50.0699	50.6789	50.0406	50.6292	50.1636	51.1331	**57.5**
**H2**	9.0056	8.8849	8.9030	8.7965	8.7977	8.7995	8.7926	8.8043	8.8043	8.7932	8.7781	9.0056	8.8849	8.9030	8.7965	8.7977	8.7994	8.7926	8.8043	8.8043	8.7932	8.7781	**8.11**
**H3**	7.5396	7.6763	7.7674	7.7172	7.8365	7.7144	7.8519	7.7131	7.8455	7.7189	7.8671	7.5396	7.6763	7.7674	7.7172	7.8365	7.7144	7.8519	7.7131	7.8455	7.7189	7.8671	**7.31**
**H5**	7.3877	7.5702	7.6618	7.6532	7.7802	7.6475	7.7925	7.6434	7.7820	7.6575	7.8161	7.3877	7.5702	7.6618	7.6532	7.7802	7.6475	7.7926	7.6434	7.7820	7.6575	7.8161	**7.31**
**H6**	8.1905	8.3935	8.4921	8.4932	8.6406	8.4854	8.6743	8.4792	8.6601	8.4999	8.7076	8.1905	8.3935	8.4921	8.4932	8.6406	8.4854	8.6743	8.4792	8.6601	8.4999	8.7076	**8.11**
**H8**	4.2049	4.2951	4.3484	4.3672	4.4527	4.3612	4.4800	4.3569	4.4681	4.3721	4.4886	4.2049	4.2951	4.3484	4.3672	4.4527	4.3612	4.4800	4.3569	4.4681	4.3722	4.4886	**5.18**
**H(9A-9B)**	1.6602	1.6944	1.6916	1.7190	1.7312	1.7160	1.7354	1.7147	1.7257	1.7209	1.7694	1.6602	1.6944	1.6916	1.7190	1.7312	1.7160	1.7354	1.7147	1.7257	1.7209	1.7694	**2.05**
**H(10A)**	0.9464	0.7709	0.7337	0.7059	0.6281	0.7076	0.6393	0.7111	0.6461	0.7035	0.6520	0.9464	0.7709	0.7337	0.7059	0.6281	0.7076	0.6393	0.7111	0.6461	0.7035	0.6520	**1.14**
**H(10B)**	1.5956	1.4998	1.4331	1.4116	1.3290	1.4162	1.3204	1.4225	1.3282	1.4064	1.2946	1.5956	1.4998	1.4331	1.4116	1.3290	1.4162	1.3204	1.4225	1.3282	1.4064	1.2946	**1.39**
**H(11A-11B)**	0.9922	1.0091	0.9759	1.0198	0.9828	1.0178	0.9739	1.0175	0.9768	1.0208	0.9667	0.9922	1.0091	0.9759	1.0198	0.9828	1.0178	0.9739	1.0175	0.9768	1.0208	0.9667	**1.22**
**H(12A-12C)**	0.9529	0.9320	0.9064	0.9207	0.8812	0.9204	0.8804	0.9217	0.8832	0.9200	0.8772	0.9529	0.9320	0.9064	0.9207	0.8812	0.9204	0.8803	0.9216	0.8832	0.9200	0.8772	**0.77**
**H(1`A)**	2.6756	2.6656	2.6476	2.6566	2.6054	2.6565	2.6213	2.6579	2.6203	2.6558	2.6008	2.8095	2.7976	2.7819	2.7777	2.7409	2.7787	2.7655	2.7812	2.7634	2.7757	2.7396	**3.16**
**H(1`B)**	3.0184	3.0586	3.0951	3.0995	3.1550	3.0957	3.1431	3.0935	3.1381	3.1022	3.1499	2.7332	2.5800	2.5558	2.5523	2.5380	2.5532	2.5182	2.5557	2.5071	2.5505	2.5099	**3.76**
**H(2`A-2`B)**	1.6952	1.7327	1.6981	1.7670	1.7206	1.7638	1.7292	1.7622	1.7248	1.7691	1.7476	1.5899	1.6712	1.6339	1.7211	1.6782	1.7172	1.7066	1.7150	1.7020	1.7238	1.7383	**2.13**
**H(3`A-3`B)**	1.5899	1.6712	1.6339	1.7211	1.6782	1.7172	1.7066	1.7149	1.7020	1.7238	1.7383	1.6952	1.7327	1.6981	1.7670	1.7206	1.7638	1.7292	1.7622	1.7248	1.7691	1.7476	**2.05**
**H(4`A)**	2.7332	2.5800	2.5558	2.5523	2.5380	2.5532	2.5182	2.5557	2.5071	2.5505	2.5099	3.0184	3.0586	3.0951	3.0995	3.1550	3.0957	3.1431	3.0935	3.1381	3.1022	3.1499	**3.09**
**H(4`B)**	2.8095	2.7976	2.7819	2.7777	2.7409	2.7787	2.7655	2.7812	2.7634	2.7757	2.7396	2.6756	2.6656	2.6476	2.6566	2.6054	2.6565	2.6213	2.6579	2.6203	2.6558	2.6008	**3.63**
**F4**	-102.7646	-104.5332	-106.7278	-104.9953	-108.4054	-104.9782	-106.6670	-104.9590	-106.8896	-105.0099	-106.5740	-102.7646	-104.5332	-106.7278	-104.9953	-108.4055	-104.9782	-106.6671	-104.9589	-106.8897	-105.0099	-106.5740	**-104.5**

[a] methyl protons shifts are averaged

[b] according to [6]

Table S6. Cont. VII

Nuclei	R											S											Exp. [b]
	M06																						
	Gas	$CHCl_3$ (PCM)	$CHCl_3$ (SMD)	DMSO (PCM)	DMSO (SMD)	CH_3OH (PCM)	CH_3OH (SMD)	C_2H_5OH (PCM)	C_2H_5OH (SMD)	H_2O (PCM)	H_2O (SMD)	Gas	$CHCl_3$ (PCM)	$CHCl_3$ (SMD)	DMSO (PCM)	DMSO (SMD)	CH_3OH (PCM)	CH_3OH (SMD)	C_2H_5OH (PCM)	C_2H_5OH (SMD)	H_2O (PCM)	H_2O (SMD)	
**C1**	136.6837	138.1845	138.7887	138.8803	142.1616	138.8390	141.0201	138.8028	141.1968	138.9324	141.3525	136.6837	138.1845	138.7887	138.8803	142.1616	138.8390	141.0201	138.8027	141.1968	138.9324	141.3525	**132.8**
**C2**	139.8288	139.8302	140.5330	139.7616	140.5668	139.7588	141.0927	139.7635	140.9947	139.7793	141.0467	139.8288	139.8302	140.5330	139.7616	140.5668	139.7588	141.0927	139.7635	140.9947	139.7793	141.0466	**132.9**
**C3**	121.7274	122.3706	123.0781	122.5656	123.3591	122.5459	123.5764	122.5339	123.5302	122.5989	123.6983	121.7273	122.3706	123.0781	122.5656	123.3592	122.5459	123.5764	122.5338	123.5302	122.5989	123.6984	**117.2**
**C4**	173.6534	174.3654	175.1102	174.6855	175.0219	174.6538	175.6222	174.6298	175.4678	174.7300	175.7595	173.6534	174.3654	175.1102	174.6855	175.0220	174.6538	175.6222	174.6297	175.4678	174.7300	175.7595	**167.5**
**C5**	119.5548	120.7943	121.4738	121.5477	122.5989	121.4922	122.8231	121.4445	122.7327	121.6146	122.9843	119.5548	120.7943	121.4739	121.5477	122.5989	121.4922	122.8231	121.4445	122.7327	121.6146	122.9844	**117.2**
**C6**	136.4800	138.1760	139.3711	139.1977	140.2318	139.1198	140.8785	139.0504	140.6173	139.2868	141.1362	136.4800	138.1760	139.3711	139.1977	140.2318	139.1198	140.8785	139.0504	140.6173	139.2868	141.1362	**132.9**
**C7**	203.9031	208.9344	210.1431	211.5776	213.5179	211.4065	218.3465	211.2396	217.2496	211.7527	219.5336	203.9031	208.9344	210.1431	211.5776	213.5178	211.4065	218.3465	211.2398	217.2496	211.7527	219.5336	**195.8**
**C8**	62.4081	63.5176	64.2402	64.1969	65.5191	64.1481	66.0334	64.1062	65.8734	64.2578	66.5123	62.4081	63.5176	64.2401	64.1969	65.5191	64.1481	66.0334	64.1062	65.8734	64.2578	66.5124	**68.4**
**C9**	33.8614	33.5608	34.1825	33.4541	33.5991	33.4442	33.8295	33.4403	33.7236	33.4773	33.9495	33.8614	33.5608	34.1825	33.4541	33.5991	33.4442	33.8295	33.4403	33.7236	33.4773	33.9496	**30.5**
**C10**	25.9231	26.6415	27.3493	27.1312	28.1401	27.0798	27.8705	27.0349	27.8296	27.1942	28.2499	25.9232	26.6415	27.3493	27.1312	28.1402	27.0798	27.8705	27.0350	27.8296	27.1942	28.2499	**27.6**
**C11**	26.4897	26.5713	27.0992	26.6108	27.1999	26.5919	27.1386	26.5795	27.1135	26.6426	27.3645	26.4897	26.5713	27.0993	26.6108	27.1998	26.5919	27.1386	26.5795	27.1135	26.6426	27.3645	**23.1**
**C12**	15.9103	16.1276	16.3119	16.3078	16.6622	16.2818	16.5809	16.2642	16.5652	16.3475	16.6634	15.9104	16.1276	16.3119	16.3078	16.6622	16.2818	16.5809	16.2642	16.5652	16.3475	16.6635	**13.8**
**C1`**	50.7988	51.7722	52.1796	52.3154	52.9675	52.2717	52.8620	52.2364	52.8195	52.3722	53.3347	55.0385	56.1158	56.5140	56.6961	57.3840	56.6486	57.5406	56.6088	57.4838	56.7556	57.9703	**53.5**
**C2`**	23.3610	23.8539	24.2807	24.1064	24.6826	24.0798	24.6277	24.0608	24.6002	24.1462	24.5473	22.1499	22.4551	22.8638	22.6320	23.3567	22.6134	23.1653	22.6032	23.1850	22.6645	22.8605	**24.5**
**C3`**	22.1499	22.4551	22.8637	22.6320	23.3567	22.6134	23.1653	22.6032	23.1850	22.6645	22.8605	23.3610	23.8539	24.2807	24.1064	24.6825	24.0798	24.6277	24.0607	24.6002	24.1462	24.5472	**24.3**
**C4`**	55.0385	56.1158	56.5140	56.6961	57.3841	56.6486	57.5406	56.6087	57.4838	56.7556	57.9703	50.7988	51.7722	52.1796	52.3154	52.9675	52.2717	52.8620	52.2365	52.8195	52.3722	53.3348	**57.5**
**H2**	8.7200	8.5356	8.6199	8.4194	8.3302	8.4246	8.3913	8.4306	8.3825	8.4151	8.3799	8.7200	8.5356	8.6199	8.4194	8.3302	8.4246	8.3913	8.4306	8.3825	8.4151	8.3799	**8.11**
**H3**	7.3688	7.5079	7.6104	7.5243	7.6008	7.5236	7.6221	7.5236	7.6136	7.5255	7.6318	7.3688	7.5079	7.6104	7.5243	7.6008	7.5236	7.6221	7.5236	7.6136	7.5255	7.6319	**7.31**
**H5**	7.0547	7.2196	7.3131	7.3125	7.4915	7.3067	7.5114	7.3012	7.5006	7.3185	7.5350	7.0547	7.2196	7.3131	7.3125	7.4915	7.3067	7.5114	7.3012	7.5006	7.3185	7.5350	**7.31**
**H6**	8.0770	8.2186	8.3077	8.3010	8.3629	8.2950	8.4115	8.2895	8.3902	8.3074	8.4458	8.0770	8.2186	8.3077	8.3010	8.3629	8.2950	8.4115	8.2895	8.3902	8.3074	8.4458	**8.11**
**H8**	4.4275	4.4268	4.4630	4.4703	4.5762	4.4663	4.6245	4.4627	4.6087	4.4746	4.6669	4.4275	4.4268	4.4630	4.4703	4.5762	4.4663	4.6245	4.4627	4.6087	4.4746	4.6669	**5.18**
**H(9A-9B)**	1.8331	1.7715	1.8082	1.7461	1.6728	1.7461	1.6811	1.7470	1.6718	1.7469	1.7105	1.8331	1.7715	1.8082	1.7461	1.6728	1.7461	1.6811	1.7470	1.6718	1.7469	1.7105	**2.05**
**H(10A)**	0.7728	0.6586	0.6596	0.6274	0.6472	0.6280	0.6691	0.6289	0.6703	0.6272	0.7166	0.7728	0.6586	0.6596	0.6274	0.6472	0.6280	0.6691	0.6289	0.6678	0.6272	0.7166	**1.14**
**H(10B)**	1.7663	1.6085	1.5217	1.4729	1.3898	1.4826	1.4047	1.4937	1.4059	1.4648	1.3494	1.7663	1.6085	1.5217	1.4729	1.3897	1.4826	1.4047	1.4937	1.4183	1.4648	1.3494	**1.39**
**H(11A-11B)**	1.0514	1.0480	1.0176	1.0528	1.0214	1.0520	1.0203	1.0520	1.0195	1.0542	1.0055	1.0514	1.0480	1.0176	1.0528	1.0214	1.0520	1.0203	1.0519	1.0195	1.0542	1.0055	**1.22**
**H(12A-12C)**	0.9078	0.8833	0.8498	0.8652	0.8460	0.8666	0.8533	0.8688	0.8571	0.8646	0.8353	0.9078	0.8833	0.8498	0.8652	0.8460	0.8666	0.8533	0.8688	0.8571	0.8646	0.8354	**0.77**
**H(1`A)**	2.6899	2.6721	2.6596	2.6399	2.6025	2.6424	2.6121	2.6455	2.6133	2.6382	2.5768	2.8894	2.8866	2.8724	2.8603	2.8238	2.8625	2.8387	2.8652	2.8412	2.8590	2.8125	**3.16**
**H(1`B)**	3.1176	3.1382	3.1900	3.1575	3.2127	3.1551	3.2023	3.1532	3.2035	3.1604	3.1870	2.7389	2.6337	2.6366	2.6095	2.6225	2.6103	2.6180	2.6118	2.6158	2.6093	2.6306	**3.76**
**H(2`A-2`B)**	1.6551	1.6922	1.6633	1.7224	1.6963	1.7200	1.7052	1.7181	1.7064	1.7253	1.7325	1.6193	1.6626	1.6205	1.6896	1.6647	1.6877	1.6882	1.6865	1.6864	1.6921	1.7293	**2.13**
**H(3`A-3`B)**	1.6193	1.6626	1.6205	1.6896	1.6647	1.6877	1.6882	1.6865	1.6894	1.6921	1.7293	1.6551	1.6922	1.6633	1.7224	1.6963	1.7200	1.7052	1.7181	1.6998	1.7253	1.7325	**2.05**
**H(4`A)**	2.7389	2.6337	2.6366	2.6095	2.6225	2.6103	2.6180	2.6118	2.6192	2.6093	2.6306	3.1176	3.1382	3.1900	3.1575	3.2127	3.1551	3.2023	3.1532	3.1996	3.1604	3.1870	**3.09**
**H(4`B)**	2.8894	2.8866	2.8724	2.8603	2.8238	2.8625	2.8387	2.8652	2.8399	2.8590	2.8125	2.6899	2.6721	2.6596	2.6399	2.6025	2.6424	2.6121	2.6455	2.6150	2.6382	2.5768	**3.63**
**F4**	-117.6983	-119.2994	-121.3029	-118.9567	-123.8039	-118.9528	-121.7203	-118.9471	-122.0604	-118.9592	-121.5181	-117.6983	-119.2994	-121.3029	-118.9567	-123.8038	-118.9528	-121.7203	-118.9472	-122.0604	-118.9592	-121.5182	**-104.5**
Nuclei	M06-2X																						
**C1**	153.3139	154.1147	155.0923	154.6258	156.4278	154.6074	156.0736	154.5917	155.8323	154.6374	156.7424	153.3140	154.1147	155.0922	154.6258	156.4279	154.6072	156.0736	154.5917	155.8323	154.6374	156.7424	**132.8**
**C2**	154.9208	154.8472	155.4019	154.6764	155.2728	154.7107	155.7196	154.7454	155.6269	154.6371	155.7829	154.9207	154.8472	155.4018	154.6764	155.2727	154.7105	155.7196	154.7454	155.6269	154.6371	155.7829	**132.9**
**C3**	136.1763	136.8491	137.4978	137.0428	137.8093	137.0546	138.0584	137.0674	138.0027	137.0251	138.1494	136.1763	136.8491	137.4977	137.0428	137.8093	137.0547	138.0584	137.0674	138.0027	137.0251	138.1494	**117.2**
**C4**	187.1500	187.9566	188.6296	188.3781	189.0881	188.3727	189.7626	188.3687	189.6217	188.3768	189.9010	187.1498	187.9566	188.6296	188.3781	189.0881	188.3728	189.7626	188.3687	189.6217	188.3768	189.9010	**167.5**
**C5**	133.6665	135.1820	135.9032	135.9787	136.9449	135.9514	137.1623	135.9252	137.0543	135.9981	137.3183	133.6665	135.1820	135.9032	135.9787	136.9449	135.9515	137.1623	135.9252	137.0543	135.9981	137.3183	**117.2**
**C6**	151.1929	153.3671	154.5822	154.5301	155.3787	154.4823	156.8323	154.4355	156.6334	154.5694	157.1596	151.1930	153.3671	154.5822	154.5301	155.3788	154.4824	156.8323	154.4355	156.6334	154.5694	157.1596	**132.9**
**C7**	219.5245	224.8907	226.3983	228.0101	228.7404	227.8294	235.4867	227.6520	233.7316	228.1759	237.1101	219.5241	224.8907	226.3983	228.0101	228.7407	227.8297	235.4867	227.6520	233.7316	228.1759	237.1101	**195.8**
**C8**	67.2610	68.6483	69.3496	69.3548	70.4448	69.3293	71.3018	69.3060	71.0041	69.3732	72.0281	67.2610	68.6483	69.3496	69.3548	70.4448	69.3292	71.3018	69.3060	71.0041	69.3732	72.0281	**68.4**
**C9**	35.4642	35.4239	35.5992	35.3385	35.6219	35.3672	35.7953	35.3969	35.7017	35.3048	36.0192	35.4642	35.4239	35.5992	35.3385	35.6220	35.3672	35.7953	35.3969	35.7017	35.3048	36.0192	**30.5**
**C10**	28.1749	28.5554	28.9127	28.9302	29.6142	28.9248	29.4107	28.9207	29.3703	28.9290	29.6021	28.1749	28.5554	28.9126	28.9302	29.6141	28.9248	29.4107	28.9207	29.3703	28.9290	29.6021	**27.6**
**C11**	27.0694	27.0411	27.3428	27.0466	27.4659	27.0675	27.4579	27.0895	27.4476	27.0203	27.6157	27.0693	27.0411	27.3428	27.0466	27.4660	27.0676	27.4579	27.0895	27.4476	27.0203	27.6156	**23.1**
**C12**	15.6785	15.8894	15.9172	16.0069	16.0766	16.0199	16.0059	16.0341	16.0007	15.9879	15.9410	15.6786	15.8894	15.9172	16.0069	16.0767	16.0199	16.0059	16.0341	16.0007	15.9879	15.9410	**13.8**
**C1`**	52.7471	53.4371	53.7246	53.9470	54.4968	53.9305	54.5272	53.9162	54.4023	53.9572	54.9826	57.2784	58.0992	58.4834	58.4784	59.0562	58.4723	59.1518	58.4681	59.1113	58.4785	59.4310	**53.5**
**C2`**	24.6913	25.0617	25.3331	25.2432	25.6602	25.2509	25.6528	25.2603	25.6474	25.2298	25.6465	23.7535	24.0580	24.3338	24.2747	24.7880	24.2798	24.7486	24.2866	24.7300	24.2642	24.8143	**24.5**
**C3`**	23.7535	24.0580	24.3338	24.2747	24.7881	24.2797	24.7486	24.2866	24.7300	24.2642	24.8143	24.6914	25.0617	25.3332	25.2432	25.6603	25.2509	25.6528	25.2603	25.6474	25.2298	25.6465	**24.3**
**C4`**	57.2784	58.0992	58.4835	58.4784	59.0562	58.4723	59.1518	58.4681	59.1113	58.4785	59.4310	52.7471	53.4371	53.7245	53.9470	54.4969	53.9305	54.5272	53.9162	54.4023	53.9572	54.9826	**57.5**
**H2**	9.2553	9.1886	9.2043	9.1236	9.1185	9.1277	9.1058	9.1311	9.1346	9.1198	9.0783	9.2553	9.1886	9.2043	9.1236	9.1185	9.1277	9.1058	9.1311	9.1346	9.1198	9.0783	**8.11**
**H3**	7.8496	7.9991	8.0968	8.0550	8.1780	8.0508	8.2036	8.0462	8.1958	8.0590	8.2179	7.8496	7.9991	8.0968	8.0550	8.1780	8.0508	8.2036	8.0462	8.1958	8.0590	8.2179	**7.31**
**H5**	7.6700	7.8593	7.9559	7.9464	8.0779	7.9403	8.0866	7.9337	8.0791	7.9524	8.1072	7.6700	7.8593	7.9559	7.9464	8.0779	7.9403	8.0866	7.9337	8.0791	7.9524	8.1072	**7.31**
**H6**	8.5783	8.7576	8.8602	8.8626	9.0133	8.8546	9.0581	8.8464	9.0490	8.8703	9.0681	8.5783	8.7576	8.8602	8.8626	9.0133	8.8546	9.0581	8.8464	9.0490	8.8703	9.0681	**8.11**
**H8**	4.2922	4.3772	4.4355	4.4447	4.5306	4.4393	4.5577	4.4336	4.5580	4.4499	4.4579	4.2922	4.3772	4.4355	4.4447	4.5306	4.4393	4.5577	4.4336	4.5580	4.4499	4.4579	**5.18**
**H(9A-9B)**	1.6952	1.7512	1.7338	1.7870	1.7864	1.7839	1.7723	1.7805	1.7723	1.7901	1.8164	1.6952	1.7512	1.7338	1.7870	1.7864	1.7839	1.7723	1.7805	1.7723	1.7901	1.8164	**2.05**
**H(10A)**	0.8886	0.7034	0.6742	0.6310	0.5530	0.6339	0.5704	0.6364	0.5766	0.6284	0.6028	0.8886	0.7034	0.6742	0.6310	0.5530	0.6339	0.5704	0.6364	0.5766	0.6284	0.6028	**1.14**
**H(10B)**	1.5834	1.5136	1.4450	1.4129	1.3259	1.4186	1.3112	1.4240	1.3267	1.4077	1.2702	1.5834	1.5136	1.4449	1.4129	1.3259	1.4186	1.3112	1.4240	1.3267	1.4077	1.2702	**1.39**
**H(11A-11B)**	0.9471	0.9670	0.9367	0.9816	0.9426	0.9795	0.9338	0.9771	0.9397	0.9837	0.9138	0.9471	0.9670	0.9367	0.9816	0.9426	0.9795	0.9338	0.9771	0.9397	0.9837	0.9138	**1.22**
**H(12A-12C)**	0.8951	0.8808	0.8572	0.8688	0.8253	0.8683	0.8226	0.8674	0.8290	0.8694	0.8069	0.8951	0.8808	0.8572	0.8688	0.8253	0.8683	0.8226	0.8674	0.8290	0.8694	0.8069	**0.77**
**H(1`A)**	2.6201	2.6148	2.6019	2.6064	2.5567	2.6061	2.5649	2.6055	2.5694	2.6068	2.5425	2.8027	2.8010	2.7892	2.7839	2.7542	2.7840	2.7717	2.7838	2.7696	2.7840	2.7411	**3.16**
**H(1`B)**	3.0833	3.1152	3.1403	3.1536	3.1988	3.1498	3.1827	3.1456	3.1831	3.1574	3.1910	2.7295	2.6123	2.5775	2.5866	2.5699	2.5867	2.5575	2.5864	2.5371	2.5867	2.5782	**3.76**
**H(2`A-2`B)**	1.6995	1.7373	1.7056	1.7719	1.7264	1.7684	1.7331	1.7647	1.7310	1.7753	1.7445	1.6106	1.6861	1.6582	1.7345	1.6958	1.7299	1.7278	1.7250	1.7222	1.7391	1.7543	**2.13**
**H(3`A-3`B)**	1.6106	1.6861	1.6582	1.7345	1.6959	1.7299	1.7278	1.7250	1.7222	1.7391	1.7543	1.6995	1.7373	1.7057	1.7719	1.7264	1.7684	1.7331	1.7647	1.7310	1.7753	1.7445	**2.05**
**H(4`A)**	2.7295	2.6123	2.5775	2.5866	2.5699	2.5867	2.5575	2.5864	2.5371	2.5867	2.5782	3.0833	3.1152	3.1402	3.1536	3.1988	3.1498	3.1827	3.1456	3.1831	3.1574	3.1910	**3.09**
**H(4`B)**	2.8027	2.8010	2.7892	2.7839	2.7542	2.7840	2.7717	2.7838	2.7696	2.7840	2.7411	2.6201	2.6148	2.6019	2.6064	2.5567	2.6061	2.5649	2.6055	2.5694	2.6068	2.5425	**3.63**
**F4**	-114.4379	-115.5001	-117.9210	-115.5535	-119.2724	-115.5557	-117.3185	-115.5562	-117.5468	-115.5516	-117.2639	-114.4381	-115.5001	-117.9210	-115.5535	-119.2724	-115.5556	-117.3185	-115.5562	-117.5468	-115.5516	-117.2639	**-104.5**

[a] methyl protons shifts are averaged

[b] according to [6]

Table S6. Cont. VIII

Nuclei	R											S											Exp. [b]
	mpW1LYP																						
	Gas	$CHCl_3$ (PCM)	$CHCl_3$ (SMD)	DMSO (PCM)	DMSO (SMD)	CH_3OH (PCM)	CH_3OH (SMD)	C_2H_5OH (PCM)	C_2H_5OH (SMD)	H_2O (PCM)	H_2O (SMD)	Gas	$CHCl_3$ (PCM)	$CHCl_3$ (SMD)	DMSO (PCM)	DMSO (SMD)	CH_3OH (PCM)	CH_3OH (SMD)	C_2H_5OH (PCM)	C_2H_5OH (SMD)	H_2O (PCM)	H_2O (SMD)	
**C1**	139.8378	140.8440	141.4180	141.3625	142.7491	141.3294	142.1529	141.2986	142.1820	141.3923	142.2792	139.8378	140.8440	141.4181	141.3622	142.7491	141.3294	142.1530	141.2986	142.1819	141.3923	142.2792	**132.8**
**C2**	139.8714	139.7984	140.3079	139.7197	140.1190	139.7218	140.6847	139.7258	140.5678	139.7161	140.5968	139.8714	139.7984	140.3079	139.7196	140.1190	139.7218	140.6848	139.7258	140.5678	139.7161	140.5970	**132.9**
**C3**	122.0463	122.6720	123.2099	122.8903	123.4471	122.8775	123.7073	122.8655	123.6488	122.9006	123.7499	122.0463	122.6720	123.2099	122.8905	123.4470	122.8775	123.7074	122.8655	123.6487	122.9006	123.7498	**117.2**
**C4**	176.3397	177.1720	177.7901	177.5913	178.1904	177.5635	178.7928	177.5372	178.6520	177.6158	178.9042	176.3397	177.1719	177.7901	177.5914	178.1902	177.5635	178.7927	177.5372	178.6517	177.6158	178.9042	**167.5**
**C5**	119.8982	121.0713	121.6786	121.7085	122.4580	121.6663	122.6459	121.6259	122.5595	121.7466	122.7780	119.8982	121.0713	121.6786	121.7085	122.4581	121.6663	122.6458	121.6259	122.5594	121.7465	122.7779	**117.2**
**C6**	136.0966	138.0128	139.1739	139.1635	140.6582	139.0867	141.3023	139.0117	141.0638	139.2346	141.6841	136.0966	138.0128	139.1739	139.1635	140.6585	139.0867	141.3022	139.0117	141.0638	139.2346	141.6841	**132.9**
**C7**	203.6129	208.5205	209.5677	211.1801	211.7568	211.0176	216.9290	210.8561	215.7371	211.3332	217.9149	203.6129	208.5205	209.5677	211.1801	211.7568	211.0176	216.9290	210.8561	215.7372	211.3332	217.9147	**195.8**
**C8**	65.8463	67.0437	67.6010	67.5551	68.5804	67.5298	69.1701	67.5042	69.0076	67.5753	69.4725	65.8463	67.0437	67.6010	67.5551	68.5805	67.5298	69.1701	67.5042	69.0077	67.5753	69.4725	**68.4**
**C9**	37.0169	36.6560	36.8848	36.5180	36.8153	36.5233	37.0170	36.5296	36.9632	36.5106	37.0610	37.0169	36.6560	36.8848	36.5180	36.8153	36.5233	37.0170	36.5296	36.9632	36.5106	37.0610	**30.5**
**C10**	29.3292	29.7978	30.3192	30.1290	30.8587	30.1027	30.6588	30.0784	30.6559	30.1521	30.7746	29.3292	29.7978	30.3193	30.1290	30.8587	30.1027	30.6588	30.0784	30.6559	30.1521	30.7746	**27.6**
**C11**	29.4068	29.5120	29.8154	29.4911	29.9151	29.4934	29.9122	29.4963	29.9182	29.4864	30.0182	29.4067	29.5120	29.8153	29.4911	29.9151	29.4934	29.9121	29.4963	29.9182	29.4865	30.0180	**23.1**
**C12**	16.7769	16.9386	17.0341	17.0893	17.1643	17.0748	17.1257	17.0621	17.1298	17.1017	17.1017	16.7769	16.9387	17.0340	17.0893	17.1642	17.0748	17.1257	17.0621	17.1298	17.1017	17.1017	**13.8**
**C1`**	51.9766	52.7794	53.1018	53.3629	53.8586	53.3224	53.8640	53.2840	53.8039	53.4001	54.3551	56.5078	57.6071	57.9635	58.0839	58.5838	58.0550	58.7006	58.0272	58.6452	58.1090	58.9220	**53.5**
**C2`**	25.9559	26.4339	26.8102	26.6257	27.1139	26.6133	27.1490	26.6020	27.1457	26.6349	26.9197	25.5722	25.7544	26.1426	25.9442	26.3949	25.9276	26.3477	25.9127	26.3492	25.9585	26.1388	**24.5**
**C3`**	25.5722	25.7545	26.1426	25.9444	26.3949	25.9276	26.3478	25.9127	26.3493	25.9585	26.1388	25.9559	26.4339	26.8101	26.6257	27.1139	26.6133	27.1490	26.6020	27.1458	26.6349	26.9196	**24.3**
**C4`**	56.5078	57.6072	57.9635	58.0838	58.5838	58.0550	58.7006	58.0272	58.6452	58.1090	58.9219	51.9767	52.7795	53.1019	53.3630	53.8586	53.3223	53.8640	53.2840	53.8039	53.4001	54.3551	**57.5**
**H2**	8.7240	8.6003	8.6562	8.5315	8.5403	8.5359	8.5588	8.5399	8.5634	8.5273	8.5464	8.7240	8.6003	8.6562	8.5315	8.5403	8.5359	8.5588	8.5399	8.5634	8.5273	8.5464	**8.11**
**H3**	7.3356	7.4800	7.5666	7.5264	7.6381	7.5244	7.6591	7.5221	7.6515	7.5280	7.6715	7.3356	7.4800	7.5666	7.5264	7.6381	7.5244	7.6591	7.5221	7.6515	7.5280	7.6715	**7.31**
**H5**	7.1538	7.3065	7.4076	7.3765	7.5081	7.3726	7.5127	7.3684	7.5049	7.3800	7.5376	7.1538	7.3065	7.4076	7.3765	7.5081	7.3726	7.5127	7.3684	7.5049	7.3800	7.5376	**7.31**
**H6**	7.9770	8.1646	8.2590	8.2681	8.3872	8.2620	8.4387	8.2555	8.4208	8.2738	8.4656	7.9770	8.1646	8.2590	8.2681	8.3872	8.2620	8.4387	8.2555	8.4208	8.2738	8.4656	**8.11**
**H8**	4.5678	4.6311	4.6745	4.6785	4.7338	4.6749	4.7727	4.6711	4.7587	4.6818	4.7496	4.5678	4.6311	4.6745	4.6785	4.7339	4.6749	4.7727	4.6711	4.7588	4.6818	4.7496	**5.18**
**H(9A-9B)**	1.6834	1.7242	1.7339	1.7539	1.7850	1.7522	1.7829	1.7502	1.7789	1.7553	1.8165	1.6834	1.7242	1.7339	1.7539	1.7850	1.7522	1.7829	1.7502	1.7789	1.7553	1.8165	**2.05**
**H(10A)**	1.0055	0.8560	0.8506	0.8221	0.7324	0.8230	0.7821	0.8238	0.7767	0.8214	0.7850	1.0055	0.8560	0.8507	0.8221	0.7324	0.8230	0.7820	0.8238	0.7767	0.8214	0.7850	**1.14**
**H(10B)**	1.6473	1.5445	1.4864	1.4679	1.3838	1.4733	1.3800	1.4783	1.3873	1.4627	1.3500	1.6473	1.5445	1.4864	1.4679	1.3838	1.4733	1.3800	1.4783	1.3873	1.4627	1.3501	**1.39**
**H(11A-11B)**	1.0567	1.0723	1.0451	1.0911	1.0518	1.0902	1.0512	1.0890	1.0511	1.0918	1.0381	1.0567	1.0723	1.0451	1.0910	1.0518	1.0902	1.0512	1.0890	1.0511	1.0918	1.0380	**1.22**
**H(12A-12C)**	0.9504	0.9373	0.9166	0.9332	0.8920	0.9336	0.8995	0.9338	0.9000	0.9328	0.8904	0.9504	0.9373	0.9166	0.9332	0.8920	0.9336	0.8995	0.9338	0.9000	0.9328	0.8904	**0.77**
**H(1`A)**	2.7337	2.7265	2.7160	2.7171	2.6748	2.7178	2.6932	2.7183	2.6947	2.7162	2.6554	2.8635	2.8719	2.8555	2.8496	2.8211	2.8518	2.8323	2.8535	2.8358	2.8473	2.7929	**3.16**
**H(1`B)**	3.1890	3.2388	3.2723	3.2779	3.3445	3.2754	3.3319	3.2727	3.3288	3.2802	3.3270	2.8791	2.7212	2.6927	2.6981	2.6939	2.6982	2.6620	2.6982	2.6627	2.6981	2.6492	**3.76**
**H(2`A-2`B)**	1.7349	1.7657	1.7316	1.7997	1.7474	1.7974	1.7616	1.7949	1.7570	1.8019	1.7774	1.6061	1.6876	1.6503	1.7367	1.6849	1.7339	1.7152	1.7308	1.7080	1.7393	1.7438	**2.13**
**H(3`A-3`B)**	1.6061	1.6876	1.6503	1.7367	1.6850	1.7339	1.7153	1.7308	1.7080	1.7393	1.7438	1.7349	1.7657	1.7316	1.7997	1.7474	1.7974	1.7616	1.7949	1.7570	1.8019	1.7774	**2.05**
**H(4`A)**	2.8791	2.7212	2.6928	2.6981	2.6939	2.6982	2.6620	2.6982	2.6627	2.6981	2.6492	3.1890	3.2388	3.2723	3.2779	3.3445	3.2754	3.3319	3.2727	3.3288	3.2802	3.3270	**3.09**
**H(4`B)**	2.8635	2.8719	2.8555	2.8496	2.8211	2.8518	2.8323	2.8535	2.8358	2.8473	2.7929	2.7337	2.7265	2.7160	2.7171	2.6748	2.7178	2.6932	2.7183	2.6947	2.7162	2.6554	**3.63**
**F4**	-123.6800	-125.1567	-127.8792	-125.4417	-129.7061	-125.4315	-127.5801	-125.4197	-127.9117	-125.4509	-127.3327	-123.6800	-125.1568	-127.8792	-125.4416	-129.7063	-125.4315	-127.5801	-125.4197	-127.9118	-125.4509	-127.3325	**-104.5**
Nuclei	mpW1PW91																						
**C1**	136.9119	137.8138	138.5064	138.2193	139.7229	138.2010	139.0452	138.1875	139.1057	138.2354	139.2865	136.9120	137.8138	138.5064	138.2193	139.7229	138.2010	139.0452	138.1875	139.1056	138.2354	139.2865	**132.8**
**C2**	138.8276	138.8043	139.2973	138.6548	139.2065	138.6657	139.6814	138.6819	139.5637	138.6442	139.6547	138.8278	138.8043	139.2971	138.6548	139.2065	138.6657	139.6814	138.6819	139.5637	138.6442	139.6547	**132.9**
**C3**	121.1664	121.9017	122.4756	122.1203	122.8340	122.1098	123.0950	122.1047	123.0259	122.1294	123.1776	121.1664	121.9017	122.4756	122.1203	122.8340	122.1098	123.0950	122.1047	123.0259	122.1294	123.1776	**117.2**
**C4**	173.3303	174.1682	174.7602	174.5577	175.2107	174.5337	175.8375	174.5156	175.6799	174.5797	175.9764	173.3302	174.1682	174.7601	174.5577	175.2107	174.5337	175.8374	174.5156	175.6800	174.5797	175.9764	**167.5**
**C5**	119.1385	120.4194	121.0633	121.0661	121.9587	121.0256	122.1586	120.9910	122.0561	121.1039	122.3339	119.1384	120.4194	121.0632	121.0661	121.9587	121.0256	122.1586	120.9910	122.0561	121.1039	122.3339	**117.2**
**C6**	134.9559	136.8628	138.0011	137.9900	139.5612	137.9156	140.2367	137.8478	139.9729	138.0608	140.6233	134.9558	136.8628	138.0011	137.9900	139.5612	137.9156	140.2368	137.8478	139.9729	138.0608	140.6233	**132.9**
**C7**	201.3961	205.9561	207.0830	208.3989	209.0671	208.2545	213.9532	208.1148	212.8505	208.5345	215.0027	201.3961	205.9561	207.0830	208.3989	209.0671	208.2545	213.9531	208.1148	212.8505	208.5345	215.0027	**195.8**
**C8**	63.7271	64.9001	65.6204	65.4113	66.4236	65.3869	67.0211	65.3670	66.8807	65.4325	67.4256	63.7271	64.9001	65.6204	65.4113	66.4236	65.3869	67.0211	65.3670	66.8807	65.4325	67.4256	**68.4**
**C9**	35.2055	34.9743	35.2048	34.8541	35.1994	34.8613	35.3954	34.8739	35.3130	34.8463	35.5099	35.2055	34.9743	35.2048	34.8541	35.1994	34.8613	35.3953	34.8739	35.3130	34.8463	35.5099	**30.5**
**C10**	27.3826	27.8477	28.3064	28.1713	28.8921	28.1466	28.7034	28.1281	28.6810	28.1948	28.8882	27.3826	27.8477	28.3064	28.1713	28.8921	28.1466	28.7033	28.1281	28.6810	28.1948	28.8882	**27.6**
**C11**	27.3749	27.5041	27.7467	27.4542	27.8881	27.4594	27.8795	27.4701	27.8744	27.4481	28.0331	27.3749	27.5041	27.7467	27.4542	27.8881	27.4594	27.8795	27.4701	27.8744	27.4481	28.0331	**23.1**
**C12**	16.2791	16.4535	16.4886	16.5599	16.6580	16.5511	16.6097	16.5484	16.6016	16.5678	16.6160	16.2792	16.4535	16.4886	16.5599	16.6580	16.5511	16.6097	16.5484	16.6016	16.5678	16.6159	**13.8**
**C1`**	50.5318	51.3520	51.5695	51.8485	52.3867	51.8154	52.3789	51.7886	52.2993	51.8801	52.8661	54.8407	55.9339	56.2529	56.3574	56.8717	56.3332	57.0066	56.3147	56.9463	56.3796	57.2577	**53.5**
**C2`**	24.7484	25.1441	25.4666	25.2813	25.7455	25.2736	25.7527	25.2717	25.7502	25.2877	25.5751	24.2083	24.4220	24.6984	24.5579	25.0088	24.5487	24.9507	24.5454	24.9402	24.5661	24.8202	**24.5**
**C3`**	24.2083	24.4220	24.6984	24.5579	25.0088	24.5487	24.9506	24.5454	24.9402	24.5661	24.8202	24.7485	25.1441	25.4666	25.2813	25.7455	25.2736	25.7527	25.2717	25.7502	25.2877	25.5751	**24.3**
**C4`**	54.8407	55.9339	56.2529	56.3574	56.8717	56.3332	57.0066	56.3147	56.9463	56.3796	57.2577	50.5318	51.3520	51.5695	51.8485	52.3867	51.8154	52.3788	51.7886	52.2993	51.8801	52.8661	**57.5**
**H2**	8.7635	8.6626	8.6890	8.5947	8.5982	8.5987	8.6271	8.6029	8.6234	8.5912	8.6110	8.7635	8.6626	8.6890	8.5947	8.5982	8.5987	8.6270	8.6029	8.6234	8.5912	8.6110	**8.11**
**H3**	7.3658	7.5155	7.6032	7.5610	7.6800	7.5592	7.7031	7.5573	7.6942	7.5630	7.7179	7.3658	7.5155	7.6032	7.5610	7.6800	7.5592	7.7031	7.5573	7.6942	7.5630	7.7179	**7.31**
**H5**	7.1836	7.3496	7.4493	7.4199	7.5570	7.4159	7.5642	7.4120	7.5548	7.4239	7.5904	7.1836	7.3496	7.4493	7.4199	7.5570	7.4159	7.5642	7.4120	7.5548	7.4239	7.5904	**7.31**
**H6**	8.0628	8.2466	8.3313	8.3493	8.4725	8.3429	8.5300	8.3368	8.5082	8.3556	8.5578	8.0628	8.2466	8.3313	8.3493	8.4725	8.3429	8.5300	8.3368	8.5082	8.3556	8.5578	**8.11**
**H8**	4.5666	4.6312	4.6552	4.6838	4.7335	4.6802	4.7762	4.6767	4.7569	4.6876	4.7740	4.5667	4.6312	4.6551	4.6838	4.7335	4.6802	4.7761	4.6767	4.7569	4.6876	4.7740	**5.18**
**H(9A-9B)**	1.7143	1.7479	1.7554	1.7759	1.7961	1.7741	1.7987	1.7723	1.7919	1.7780	1.8300	1.7142	1.7479	1.7554	1.7759	1.7961	1.7741	1.7987	1.7723	1.7919	1.7780	1.8300	**2.05**
**H(10A)**	0.9433	0.8037	0.7605	0.7576	0.6696	0.7596	0.7118	0.7618	0.7018	0.7561	0.7199	0.9433	0.8037	0.7605	0.7576	0.6696	0.7596	0.7118	0.7618	0.7018	0.7561	0.7199	**1.14**
**H(10B)**	1.6289	1.5322	1.4591	1.4457	1.3602	1.4519	1.3550	1.4581	1.3605	1.4403	1.3252	1.6289	1.5322	1.4591	1.4457	1.3602	1.4519	1.3550	1.4581	1.3605	1.4403	1.3252	**1.39**
**H(11A-11B)**	1.0002	1.0196	0.9828	1.0300	0.9938	1.0298	0.9939	1.0296	0.9925	1.0305	0.9821	1.0003	1.0196	0.9828	1.0300	0.9938	1.0298	0.9939	1.0296	0.9925	1.0305	0.9821	**1.22**
**H(12A-12C)**	0.8944	0.8820	0.8543	0.8714	0.8330	0.8723	0.8394	0.8732	0.8390	0.8708	0.8339	0.8944	0.8820	0.8543	0.8714	0.8330	0.8723	0.8393	0.8732	0.8390	0.8708	0.8339	**0.77**
**H(1`A)**	2.6698	2.6627	2.6505	2.6511	2.6099	2.6521	2.6276	2.6531	2.6278	2.6505	2.6009	2.8087	2.8120	2.7991	2.7904	2.7608	2.7925	2.7725	2.7948	2.7761	2.7885	2.7396	**3.16**
**H(1`B)**	3.1591	3.2023	3.2377	3.2375	3.2965	3.2352	3.2866	3.2330	3.2830	3.2401	3.2859	2.8814	2.7114	2.6952	2.6721	2.6718	2.6738	2.6281	2.6757	2.6303	2.6709	2.6132	**3.76**
**H(2`A-2`B)**	1.7019	1.7349	1.6989	1.7648	1.7189	1.7628	1.7313	1.7610	1.7262	1.7670	1.7500	1.5817	1.6581	1.6186	1.7003	1.6544	1.6979	1.6846	1.6956	1.6764	1.7028	1.7178	**2.13**
**H(3`A-3`B)**	1.5817	1.6581	1.6187	1.7003	1.6544	1.6979	1.6846	1.6956	1.6764	1.7028	1.7178	1.7019	1.7349	1.6989	1.7648	1.7189	1.7628	1.7313	1.7610	1.7262	1.7670	1.7500	**2.05**
**H(4`A)**	2.8814	2.7114	2.6952	2.6721	2.6718	2.6738	2.6281	2.6757	2.6303	2.6709	2.6132	3.1591	3.2023	3.2377	3.2375	3.2965	3.2352	3.2866	3.2330	3.2830	3.2401	3.2859	**3.09**
**H(4`B)**	2.8087	2.8120	2.7991	2.7904	2.7608	2.7925	2.7725	2.7948	2.7761	2.7885	2.7396	2.6698	2.6627	2.6505	2.6511	2.6099	2.6521	2.6276	2.6531	2.6278	2.6505	2.6009	**3.63**
**F4**	-113.6339	-114.6702	-117.2070	-114.7395	-118.7138	-114.7443	-116.4756	-114.7472	-116.8590	-114.7353	-116.2551	-113.6339	-114.6702	-117.2070	-114.7395	-118.7138	-114.7443	-116.4758	-114.7472	-116.8590	-114.7353	-116.2551	**-104.5**

[a] methyl protons shifts are averaged

[b] according to [6]

Table S6. Cont. IX

Nuclei	R											S											Exp. [b]
	O3LYP																						
	Gas	$CHCl_3$ (PCM)	$CHCl_3$ (SMD)	DMSO (PCM)	DMSO (SMD)	CH_3OH (PCM)	CH_3OH (SMD)	C_2H_5OH (PCM)	C_2H_5OH (SMD)	H_2O (PCM)	H_2O (SMD)	Gas	$CHCl_3$ (PCM)	$CHCl_3$ (SMD)	DMSO (PCM)	DMSO (SMD)	CH_3OH (PCM)	CH_3OH (SMD)	C_2H_5OH (PCM)	C_2H_5OH (SMD)	H_2O (PCM)	H_2O (SMD)	
**C1**	134.8316	136.7253	138.2232	137.7522	139.6525	137.7211	138.6578	137.6778	138.8802	137.7701	138.8881	134.8316	136.7253	138.2232	137.7522	139.6525	137.7212	138.6578	137.6778	138.8802	137.7701	138.8880	**132.8**
**C2**	134.2703	134.3034	134.9028	134.2696	134.8696	134.2834	135.2076	134.2931	135.1090	134.2502	135.1565	134.2703	134.3034	134.9028	134.2695	134.8695	134.2836	135.2076	134.2931	135.1090	134.2502	135.1565	**132.9**
**C3**	117.1530	117.7967	118.4174	118.0690	118.7418	118.0647	118.9947	118.0553	118.9369	118.0662	119.0549	117.1529	117.7967	118.4174	118.0688	118.7418	118.0647	118.9947	118.0553	118.9369	118.0662	119.0548	**117.2**
**C4**	169.5381	170.0292	170.6806	170.3884	171.1053	170.3736	171.6575	170.3540	171.5014	170.3967	171.7332	169.5383	170.0292	170.6806	170.3884	171.1053	170.3735	171.6575	170.3540	171.5014	170.3966	171.7334	**167.5**
**C5**	115.0903	116.2587	116.9125	116.9091	117.6769	116.8807	117.9293	116.8473	117.8196	116.9300	118.0603	115.0903	116.2587	116.9125	116.9091	117.6769	116.8806	117.9293	116.8473	117.8196	116.9299	118.0604	**117.2**
**C6**	130.2300	131.5111	132.3612	132.3562	133.7414	132.3011	134.3672	132.2439	134.1030	132.4041	134.6024	130.2299	131.5111	132.3612	132.3563	133.7414	132.3011	134.3672	132.2439	134.1030	132.4042	134.6023	**132.9**
**C7**	197.0256	202.4069	204.2489	205.5483	206.5094	205.3897	210.9074	205.2170	210.0109	205.6862	211.8448	197.0256	202.4069	204.2489	205.5484	206.5094	205.3897	210.9074	205.2170	210.0109	205.6859	211.8450	**195.8**
**C8**	64.5596	66.2848	66.9158	67.1212	67.8247	67.0947	68.4239	67.0586	68.3028	67.1363	68.7969	64.5596	66.2848	66.9158	67.1213	67.8247	67.0948	68.4239	67.0586	68.3028	67.1362	68.7968	**68.4**
**C9**	35.5442	35.1982	35.2977	35.1245	35.2805	35.1355	35.5634	35.1421	35.4857	35.1083	35.6327	35.5442	35.1982	35.2977	35.1245	35.2805	35.1354	35.5634	35.1421	35.4857	35.1083	35.6327	**30.5**
**C10**	27.9914	28.4344	28.8370	28.7329	29.4158	28.7216	29.2733	28.7061	29.2396	28.7375	29.4326	27.9915	28.4344	28.8370	28.7329	29.4156	28.7216	29.2733	28.7061	29.2396	28.7376	29.4325	**27.6**
**C11**	28.7716	28.8814	29.1958	28.9216	29.3506	28.9307	29.3249	28.9345	29.3252	28.9062	29.4166	28.7716	28.8814	29.1958	28.9217	29.3505	28.9308	29.3249	28.9345	29.3252	28.9062	29.4166	**23.1**
**C12**	16.6093	16.7634	16.8889	16.9451	17.0851	16.9427	17.0428	16.9355	17.0386	16.9411	17.0277	16.6092	16.7634	16.8889	16.9451	17.0851	16.9427	17.0428	16.9355	17.0386	16.9411	17.0277	**13.8**
**C1`**	50.8019	51.5543	51.9691	52.1253	52.7447	52.0980	52.7177	52.0662	52.6691	52.1450	53.0975	55.2428	56.2398	56.6369	56.7276	57.3004	56.7088	57.3728	56.6851	57.2958	56.7387	57.5907	**53.5**
**C2`**	25.9053	26.4060	26.7287	26.6449	27.0942	26.6421	27.1363	26.6341	27.1078	26.6410	26.9388	25.6921	25.8886	26.3279	26.0899	26.6685	26.0890	26.5111	26.0826	26.5199	26.0843	26.2550	**24.5**
**C3`**	25.6920	25.8886	26.3279	26.0899	26.6685	26.0889	26.5111	26.0826	26.5199	26.0842	26.2549	25.9053	26.4060	26.7287	26.6449	27.0942	26.6421	27.1363	26.6341	27.1078	26.6411	26.9389	**24.3**
**C4`**	55.2428	56.2398	56.6369	56.7276	57.3004	56.7089	57.3728	56.6851	57.2958	56.7388	57.5907	50.8019	51.5543	51.9691	52.1253	52.7448	52.0981	52.7178	52.0662	52.6691	52.1450	53.0974	**57.5**
**H2**	8.5172	8.3119	8.3022	8.2114	8.2142	8.2161	8.2765	8.2212	8.2596	8.2074	8.2657	8.5172	8.3119	8.3022	8.2114	8.2142	8.2161	8.2765	8.2212	8.2596	8.2074	8.2656	**8.11**
**H3**	7.3026	7.4020	7.4910	7.4435	7.5680	7.4415	7.5874	7.4391	7.5799	7.4452	7.5960	7.3026	7.4020	7.4910	7.4435	7.5680	7.4415	7.5874	7.4391	7.5799	7.4452	7.5960	**7.31**
**H5**	7.0527	7.2542	7.3671	7.3566	7.4848	7.3519	7.5014	7.3466	7.4911	7.3609	7.5199	7.0527	7.2542	7.3671	7.3566	7.4848	7.3518	7.5014	7.3466	7.4911	7.3609	7.5199	**7.31**
**H6**	7.9525	8.0429	8.1075	8.0893	8.2184	8.0851	8.2708	8.0810	8.2439	8.0936	8.2835	7.9525	8.0429	8.1075	8.0893	8.2184	8.0851	8.2708	8.0810	8.2439	8.0936	8.2835	**8.11**
**H8**	4.7795	4.7678	4.7948	4.7779	4.8539	4.7763	4.8754	4.7747	4.8616	4.7796	4.8585	4.7795	4.7678	4.7948	4.7779	4.8539	4.7763	4.8754	4.7747	4.8616	4.7796	4.8584	**5.18**
**H(9A-9B)**	1.6182	1.6249	1.5871	1.6386	1.6272	1.6368	1.6562	1.6349	1.6437	1.6405	1.6970	1.6182	1.6249	1.5871	1.6386	1.6272	1.6368	1.6562	1.6349	1.6437	1.6405	1.6970	**2.05**
**H(10A)**	0.9587	0.8526	0.8607	0.8353	0.8038	0.8362	0.8278	0.8369	0.8280	0.8343	0.8249	0.9587	0.8526	0.8607	0.8353	0.8038	0.8362	0.8278	0.8369	0.8280	0.8343	0.8249	**1.14**
**H(10B)**	1.5716	1.4752	1.4290	1.4076	1.3426	1.4126	1.3358	1.4173	1.3447	1.4028	1.3107	1.5716	1.4752	1.4290	1.4076	1.3426	1.4126	1.3358	1.4173	1.3447	1.4028	1.3107	**1.39**
**H(11A-11B)**	1.0598	1.0760	1.0550	1.0975	1.0725	1.0966	1.0683	1.0953	1.0683	1.0983	1.0500	1.0598	1.0760	1.0550	1.0975	1.0724	1.0966	1.0683	1.0953	1.0683	1.0983	1.0500	**1.22**
**H(12A-12C)**	0.9311	0.9235	0.9109	0.9235	0.8974	0.9240	0.8992	0.9241	0.9006	0.9229	0.8910	0.9311	0.9235	0.9109	0.9235	0.8974	0.9239	0.8992	0.9241	0.9006	0.9229	0.8910	**0.77**
**H(1`A)**	2.7818	2.7720	2.7498	2.7606	2.7136	2.7618	2.7281	2.7626	2.7293	2.7594	2.6945	2.9185	2.9246	2.8997	2.8958	2.8542	2.8987	2.8592	2.9012	2.8637	2.8929	2.8314	**3.16**
**H(1`B)**	3.2477	3.2925	3.3182	3.3299	3.3897	3.3277	3.3821	3.3252	3.3769	3.3319	3.3829	2.9024	2.7877	2.7774	2.7855	2.7869	2.7851	2.7591	2.7844	2.7670	2.7859	2.7593	**3.76**
**H(2`A-2`B)**	1.7372	1.7625	1.7344	1.7927	1.7507	1.7909	1.7606	1.7888	1.7570	1.7943	1.7756	1.6350	1.7111	1.6922	1.7625	1.7270	1.7599	1.7459	1.7567	1.7419	1.7649	1.7720	**2.13**
**H(3`A-3`B)**	1.6350	1.7111	1.6922	1.7626	1.7270	1.7599	1.7459	1.7567	1.7419	1.7649	1.7720	1.7372	1.7625	1.7344	1.7927	1.7507	1.7909	1.7606	1.7888	1.7570	1.7943	1.7757	**2.05**
**H(4`A)**	2.9024	2.7877	2.7774	2.7855	2.7869	2.7851	2.7591	2.7844	2.7670	2.7859	2.7593	3.2477	3.2925	3.3182	3.3299	3.3897	3.3277	3.3821	3.3252	3.3769	3.3319	3.3829	**3.09**
**H(4`B)**	2.9185	2.9246	2.8997	2.8958	2.8542	2.8987	2.8592	2.9012	2.8637	2.8929	2.8314	2.7818	2.7720	2.7498	2.7606	2.7136	2.7618	2.7281	2.7626	2.7293	2.7594	2.6945	**3.63**
**F4**	-119.1850	-121.2457	-124.2185	-121.8398	-126.1256	-121.8215	-123.9355	-121.7987	-124.3373	-121.8539	-123.7649	-119.1848	-121.2457	-124.2185	-121.8396	-126.1256	-121.8216	-123.9355	-121.7987	-124.3373	-121.8539	-123.7646	**-104.5**
Nuclei	OLYP																						
**C1**	133.9821	136.0335	137.1367	136.6843	138.8035	136.6625	137.7402	136.6347	138.0258	136.6956	137.8922	133.9821	136.0335	137.1367	136.6843	138.8035	136.6625	137.7402	136.6347	138.0258	136.6955	137.8922	**132.8**
**C2**	131.9334	131.9290	132.5576	131.8799	132.4225	131.8897	132.7752	131.8944	132.6437	131.8621	132.7099	131.9334	131.9291	132.5576	131.8799	132.4223	131.8897	132.7752	131.8944	132.6437	131.8621	132.7099	**132.9**
**C3**	115.3313	115.9906	116.6137	116.2608	116.8575	116.2547	117.1455	116.2428	117.0771	116.2573	117.2258	115.3313	115.9906	116.6137	116.2609	116.8575	116.2547	117.1455	116.2429	117.0771	116.2573	117.2258	**117.2**
**C4**	168.3478	168.9536	169.7150	169.3969	170.1310	169.3775	170.6738	169.3528	170.4989	169.4065	170.7682	168.3478	168.9536	169.7150	169.3969	170.1309	169.3775	170.6738	169.3529	170.4989	169.4065	170.7682	**167.5**
**C5**	113.3354	114.4044	115.0462	115.0266	115.7191	114.9969	116.0153	114.9618	115.8944	115.0458	116.1580	113.3354	114.4045	115.0462	115.0266	115.7191	114.9969	116.0153	114.9617	115.8944	115.0458	116.1580	**117.2**
**C6**	127.6467	128.8978	129.9328	129.9379	131.2665	129.8770	131.8460	129.8113	131.5682	129.9875	132.0896	127.6467	128.8977	129.9328	129.9380	131.2665	129.8770	131.8460	129.8113	131.5682	129.9876	132.0896	**132.9**
**C7**	195.5731	201.1277	202.5411	203.7564	205.1541	203.6174	209.0255	203.4714	208.3346	203.8785	209.8788	195.5731	201.1277	202.5411	203.7565	205.1539	203.6174	209.0255	203.4717	208.3346	203.8785	209.8788	**195.8**
**C8**	66.0732	67.9957	68.4903	68.7224	69.6196	68.7003	70.0870	68.6709	70.0972	68.7322	70.3344	66.0732	67.9958	68.4903	68.7226	69.6196	68.7004	70.0870	68.6709	70.0972	68.7322	70.3344	**68.4**
**C9**	36.5013	36.1561	36.3808	36.1692	36.2657	36.1751	36.6313	36.1753	36.5703	36.1547	36.6703	36.5013	36.1562	36.3808	36.1692	36.2656	36.1751	36.6313	36.1753	36.5703	36.1547	36.6703	**30.5**
**C10**	28.6233	29.0478	29.5021	29.3898	30.0161	29.3768	29.8944	29.3581	29.8818	29.3929	30.0503	28.6233	29.0477	29.5021	29.3898	30.0160	29.3768	29.8944	29.3580	29.8818	29.3929	30.0503	**27.6**
**C11**	29.6468	29.7271	30.0848	29.7850	30.1842	29.7907	30.1750	29.7910	30.1811	29.7704	30.2632	29.6468	29.7272	30.0848	29.7849	30.1842	29.7907	30.1749	29.7910	30.1811	29.7704	30.2632	**23.1**
**C12**	16.7637	16.9097	17.0328	17.0844	17.2029	17.0811	17.1660	17.0727	17.1626	17.0786	17.1506	16.7637	16.9097	17.0328	17.0843	17.2029	17.0811	17.1660	17.0727	17.1626	17.0786	17.1506	**13.8**
**C1`**	51.7337	52.4858	52.9328	53.0582	53.6707	53.0297	53.6672	52.9964	53.6005	53.0766	54.0194	56.2482	57.2354	57.6350	57.7173	58.2870	57.6968	58.3670	57.6709	58.2796	57.7275	58.6081	**53.5**
**C2`**	26.4609	26.9933	27.3434	27.2952	27.6992	27.2887	27.7603	27.2761	27.7417	27.2919	27.6012	26.4501	26.6417	27.0756	26.8535	27.4196	26.8487	27.2629	26.8387	27.2654	26.8497	27.0231	**24.5**
**C3`**	26.4501	26.6418	27.0756	26.8535	27.4195	26.8487	27.2629	26.8388	27.2654	26.8497	27.0231	26.4609	26.9933	27.3434	27.2952	27.6992	27.2887	27.7603	27.2761	27.7417	27.2919	27.6012	**24.3**
**C4`**	56.2482	57.2355	57.6350	57.7172	58.2870	57.6968	58.3669	57.6709	58.2796	57.7275	58.6081	51.7337	52.4859	52.9328	53.0581	53.6707	53.0297	53.6672	52.9964	53.6005	53.0766	54.0194	**57.5**
**H2**	8.3249	8.1241	8.1537	8.0646	8.0342	8.0676	8.1182	8.0705	8.0902	8.0614	8.1158	8.3249	8.1241	8.1537	8.0646	8.0341	8.0676	8.1182	8.0705	8.0902	8.0614	8.1158	**8.11**
**H3**	7.2045	7.3251	7.4204	7.3707	7.4933	7.3687	7.5141	7.3664	7.5040	7.3723	7.5211	7.2045	7.3251	7.4204	7.3707	7.4933	7.3687	7.5141	7.3664	7.5040	7.3723	7.5211	**7.31**
**H5**	6.9910	7.2033	7.3016	7.2888	7.4115	7.2844	7.4314	7.2798	7.4198	7.2926	7.4471	6.9910	7.2033	7.3016	7.2888	7.4115	7.2844	7.4314	7.2798	7.4198	7.2926	7.4471	**7.31**
**H6**	7.8487	7.9010	7.9896	7.9745	8.0789	7.9695	8.1350	7.9645	8.1011	7.9789	8.1569	7.8487	7.9010	7.9896	7.9745	8.0789	7.9695	8.1350	7.9645	8.1011	7.9789	8.1569	**8.11**
**H8**	4.9446	4.9128	4.9412	4.9248	4.9737	4.9235	4.9921	4.9221	4.9774	4.9258	4.9759	4.9446	4.9128	4.9412	4.9248	4.9737	4.9235	4.9921	4.9221	4.9774	4.9258	4.9759	**5.18**
**H(9A-9B)**	1.6234	1.6455	1.6374	1.6833	1.6640	1.6808	1.6972	1.6781	1.6877	1.6853	1.7322	1.6234	1.6455	1.6374	1.6833	1.6640	1.6808	1.6972	1.6781	1.6877	1.6853	1.7322	**2.05**
**H(10A)**	0.9953	0.8946	0.8938	0.8729	0.8450	0.8735	0.8733	0.8742	0.8679	0.8721	0.8733	0.9953	0.8946	0.8938	0.8729	0.8450	0.8735	0.8733	0.8742	0.8679	0.8721	0.8733	**1.14**
**H(10B)**	1.5882	1.4997	1.4577	1.4325	1.3742	1.4374	1.3635	1.4420	1.3716	1.4275	1.3377	1.5882	1.4997	1.4577	1.4325	1.3742	1.4374	1.3635	1.4420	1.3716	1.4275	1.3377	**1.39**
**H(11A-11B)**	1.1047	1.1212	1.1028	1.1423	1.1197	1.1410	1.1151	1.1396	1.1134	1.1432	1.0967	1.1047	1.1213	1.1028	1.1423	1.1197	1.1410	1.1151	1.1396	1.1134	1.1432	1.0967	**1.22**
**H(12A-12C)**	0.9447	0.9384	0.9250	0.9365	0.9136	0.9368	0.9156	0.9370	0.9158	0.9358	0.9079	0.9447	0.9384	0.9250	0.9365	0.9136	0.9368	0.9156	0.9370	0.9158	0.9358	0.9079	**0.77**
**H(1`A)**	2.8493	2.8305	2.8097	2.8183	2.7703	2.8195	2.7798	2.8205	2.7831	2.8167	2.7472	2.9785	2.9775	2.9559	2.9533	2.9142	2.9558	2.9129	2.9582	2.9219	2.9503	2.8855	**3.16**
**H(1`B)**	3.3360	3.3804	3.4119	3.4210	3.4791	3.4185	3.4699	3.4159	3.4664	3.4229	3.4658	2.9866	2.8809	2.8679	2.8706	2.8859	2.8704	2.8588	2.8703	2.8677	2.8705	2.8536	**3.76**
**H(2`A-2`B)**	1.7751	1.7943	1.7669	1.8200	1.7776	1.8182	1.7894	1.8164	1.7847	1.8214	1.8051	1.6770	1.7523	1.7284	1.7952	1.7674	1.7928	1.7835	1.7902	1.7783	1.7971	1.8096	**2.13**
**H(3`A-3`B)**	1.6770	1.7523	1.7284	1.7952	1.7674	1.7928	1.7835	1.7902	1.7783	1.7971	1.8096	1.7751	1.7943	1.7669	1.8200	1.7776	1.8182	1.7894	1.8164	1.7847	1.8214	1.8051	**2.05**
**H(4`A)**	2.9866	2.8809	2.8679	2.8706	2.8859	2.8705	2.8588	2.8703	2.8677	2.8705	2.8536	3.3360	3.3804	3.4119	3.4210	3.4791	3.4185	3.4699	3.4159	3.4664	3.4229	3.4658	**3.09**
**H(4`B)**	2.9785	2.9775	2.9559	2.9533	2.9142	2.9558	2.9129	2.9582	2.9219	2.9503	2.8855	2.8493	2.8305	2.8097	2.8182	2.7703	2.8195	2.7798	2.8205	2.7831	2.8167	2.7472	**3.63**
**F4**	-126.8941	-129.2746	-132.2452	-129.6606	-134.4154	-129.6500	-132.0547	-129.6387	-132.5484	-129.6690	-131.7814	-126.8941	-129.2745	-132.2452	-129.6605	-134.4155	-129.6500	-132.0547	-129.6385	-132.5484	-129.6690	-131.7814	**-104.5**

[a] methyl protons shifts are averaged
[b] according to [6]

Table S6. Cont. X

Nuclei	R											S											Exp. [b]
	OPBE																						
	Gas	$CHCl_3$ (PCM)	$CHCl_3$ (SMD)	DMSO (PCM)	DMSO (SMD)	CH_3OH (PCM)	CH_3OH (SMD)	C_2H_5OH (PCM)	C_2H_5OH (SMD)	H_2O (PCM)	H_2O (SMD)	Gas	$CHCl_3$ (PCM)	$CHCl_3$ (SMD)	DMSO (PCM)	DMSO (SMD)	CH_3OH (PCM)	CH_3OH (SMD)	C_2H_5OH (PCM)	C_2H_5OH (SMD)	H_2O (PCM)	H_2O (SMD)	
**C1**	130.6787	132.6297	133.6336	133.3807	135.3198	133.3723	134.3661	133.3448	134.5349	133.3821	134.5269	130.6787	132.6296	133.6335	133.3807	135.3197	133.3723	134.3660	133.3448	134.5349	133.3821	134.5268	**132.8**
**C2**	130.5026	130.6500	131.1455	130.5229	131.1203	130.5465	131.4274	130.5522	131.3339	130.4956	131.4311	130.5026	130.6499	131.1455	130.5229	131.1203	130.5465	131.4274	130.5522	131.3339	130.4956	131.4309	**132.9**
**C3**	114.1774	114.9620	115.5644	115.1917	115.9530	115.1953	116.1865	115.1808	116.1149	115.1830	116.2950	114.1774	114.9619	115.5645	115.1917	115.9531	115.1953	116.1864	115.1808	116.1149	115.1830	116.2949	**117.2**
**C4**	164.7994	165.4524	166.0718	165.7743	166.4439	165.7692	166.9958	165.7465	166.8494	165.7742	167.1261	164.7994	165.4524	166.0719	165.7743	166.4440	165.7692	166.9958	165.7465	166.8494	165.7742	167.1261	**167.5**
**C5**	112.2619	113.5119	114.1131	114.0723	114.8593	114.0525	115.1463	114.0152	115.0300	114.0864	115.3281	112.2618	113.5119	114.1130	114.0723	114.8593	114.0525	115.1464	114.0152	115.0300	114.0864	115.3282	**117.2**
**C6**	126.2248	127.5436	128.4947	128.4132	129.7170	128.3671	130.2985	128.3037	130.0776	128.4529	130.5759	126.2248	127.5437	128.4947	128.4132	129.7170	128.3671	130.2986	128.3037	130.0776	128.4529	130.5758	**132.9**
**C7**	192.8492	198.0469	199.3180	200.6479	201.7981	200.5296	205.5758	200.3919	204.7558	200.7535	206.3278	192.8491	198.0466	199.3180	200.6479	201.7981	200.5296	205.5759	200.3919	204.7558	200.7535	206.3275	**195.8**
**C8**	63.5007	65.3509	66.0163	66.0708	67.1112	66.0585	67.5850	66.0270	67.4654	66.0760	67.8053	63.5007	65.3510	66.0163	66.0708	67.1112	66.0585	67.5850	66.0270	67.4654	66.0760	67.8053	**68.4**
**C9**	34.3082	34.1893	34.3213	34.1125	34.3179	34.1310	34.6281	34.1317	34.5209	34.0902	34.6999	34.3083	34.1893	34.3213	34.1125	34.3179	34.1310	34.6281	34.1317	34.5209	34.0902	34.6998	**30.5**
**C10**	26.3655	26.8815	27.2838	27.1239	27.8025	27.1222	27.6443	27.1030	27.6205	27.1207	27.8389	26.3655	26.8814	27.2838	27.1239	27.8024	27.1222	27.6443	27.1030	27.6205	27.1207	27.8389	**27.6**
**C11**	27.3220	27.5273	27.7359	27.4928	27.8362	27.5128	27.8134	27.5147	27.8103	27.4686	27.9319	27.3219	27.5273	27.7360	27.4928	27.8361	27.5128	27.8134	27.5147	27.8103	27.4686	27.9319	**23.1**
**C12**	16.0776	16.2963	16.3097	16.3835	16.4232	16.3927	16.4102	16.3844	16.4094	16.3702	16.4127	16.0776	16.2963	16.3098	16.3835	16.4232	16.3927	16.4102	16.3844	16.4094	16.3702	16.4127	**13.8**
**C1`**	49.8315	50.6513	50.9131	51.1124	51.5794	51.0991	51.6068	51.0680	51.5522	51.1202	52.0407	54.1207	55.1917	55.5154	55.5904	56.1099	55.5833	56.1855	55.5579	56.1235	55.5921	56.4254	**53.5**
**C2`**	25.1000	25.6677	25.9114	25.8276	26.2209	25.8356	26.2347	25.8256	26.2242	25.8151	25.9714	24.9036	25.1884	25.4269	25.2649	25.6750	25.2773	25.5882	25.2721	25.5991	25.2486	25.3040	**24.5**
**C3`**	24.9037	25.1884	25.4268	25.2649	25.6751	25.2773	25.5881	25.2721	25.5991	25.2486	25.3041	25.1001	25.6677	25.9114	25.8276	26.2210	25.8356	26.2347	25.8256	26.2242	25.8151	25.9714	**24.3**
**C4`**	54.1207	55.1916	55.5153	55.5904	56.1100	55.5833	56.1855	55.5579	56.1235	55.5921	56.4254	49.8315	50.6514	50.9130	51.1124	51.5794	51.0991	51.6068	51.0680	51.5522	51.1201	52.0406	**57.5**
**H2**	8.3644	8.1928	8.2044	8.1131	8.0948	8.1166	8.1711	8.1195	8.1553	8.1092	8.1745	8.3644	8.1928	8.2044	8.1131	8.0948	8.1166	8.1711	8.1195	8.1553	8.1092	8.1745	**8.11**
**H3**	7.2317	7.3506	7.4367	7.3986	7.5153	7.3968	7.5392	7.3943	7.5305	7.3995	7.5502	7.2317	7.3506	7.4367	7.3986	7.5153	7.3968	7.5392	7.3943	7.5305	7.3995	7.5502	**7.31**
**H5**	7.0085	7.2266	7.3170	7.3144	7.4418	7.3103	7.4561	7.3054	7.4452	7.3175	7.4766	7.0085	7.2266	7.3170	7.3144	7.4418	7.3103	7.4561	7.3054	7.4452	7.3175	7.4766	**7.31**
**H6**	7.9097	7.9800	8.0576	8.0368	8.1441	8.0322	8.1953	8.0270	8.1733	8.0405	8.2139	7.9097	7.9800	8.0576	8.0368	8.1441	8.0322	8.1953	8.0270	8.1733	8.0405	8.2139	**8.11**
**H8**	4.9509	4.9173	4.9339	4.9242	4.9761	4.9229	4.9923	4.9211	4.9808	4.9248	4.9868	4.9509	4.9173	4.9339	4.9242	4.9761	4.9229	4.9923	4.9211	4.9808	4.9248	4.9868	**5.18**
**H(9A-9B)**	1.6537	1.6636	1.6523	1.6862	1.6774	1.6845	1.7050	1.6821	1.6910	1.6872	1.7441	1.6537	1.6636	1.6523	1.6862	1.6774	1.6845	1.7050	1.6821	1.6910	1.6872	1.7441	**2.05**
**H(10A)**	0.9080	0.8215	0.7760	0.7960	0.7277	0.7977	0.7746	0.7987	0.7708	0.7938	0.7812	0.9080	0.8214	0.7760	0.7960	0.7277	0.7977	0.7746	0.7987	0.7708	0.7938	0.7812	**1.14**
**H(10B)**	1.5468	1.4570	1.4015	1.3905	1.3184	1.3954	1.3106	1.3997	1.3176	1.3850	1.2885	1.5468	1.4570	1.4015	1.3905	1.3184	1.3954	1.3106	1.3997	1.3176	1.3850	1.2884	**1.39**
**H(11A-11B)**	1.0380	1.0583	1.0237	1.0740	1.0368	1.0738	1.0344	1.0727	1.0341	1.0737	1.0195	1.0380	1.0583	1.0237	1.0740	1.0368	1.0738	1.0344	1.0727	1.0341	1.0737	1.0195	**1.22**
**H(12A-12C)**	0.8763	0.8722	0.8453	0.8670	0.8301	0.8680	0.8355	0.8684	0.8363	0.8653	0.8297	0.8763	0.8722	0.8453	0.8670	0.8301	0.8680	0.8355	0.8684	0.8363	0.8653	0.8297	**0.77**
**H(1`A)**	2.7652	2.7626	2.7400	2.7489	2.7012	2.7503	2.7146	2.7511	2.7162	2.7467	2.6727	2.9190	2.9188	2.8953	2.8880	2.8426	2.8911	2.8529	2.8934	2.8571	2.8843	2.8212	**3.16**
**H(1`B)**	3.2993	3.3459	3.3765	3.3785	3.4327	3.3769	3.4258	3.3746	3.4221	3.3793	3.4161	2.9751	2.8559	2.8439	2.8403	2.8379	2.8409	2.8138	2.8411	2.8191	2.8390	2.8061	**3.76**
**H(2`A-2`B)**	1.7256	1.7518	1.7160	1.7751	1.7243	1.7740	1.7393	1.7721	1.7348	1.7756	1.7601	1.6373	1.7083	1.6748	1.7504	1.7036	1.7486	1.7291	1.7459	1.7249	1.7515	1.7617	**2.13**
**H(3`A-3`B)**	1.6373	1.7083	1.6748	1.7504	1.7036	1.7486	1.7291	1.7459	1.7249	1.7515	1.7617	1.7256	1.7518	1.7160	1.7751	1.7243	1.7740	1.7393	1.7721	1.7348	1.7756	1.7601	**2.05**
**H(4`A)**	2.9751	2.8559	2.8439	2.8403	2.8379	2.8409	2.8138	2.8411	2.8191	2.8390	2.8061	3.2993	3.3459	3.3765	3.3785	3.4327	3.3769	3.4258	3.3746	3.4221	3.3793	3.4161	**3.09**
**H(4`B)**	2.9190	2.9187	2.8953	2.8880	2.8426	2.8911	2.8529	2.8934	2.8571	2.8843	2.8212	2.7652	2.7625	2.7400	2.7489	2.7012	2.7503	2.7146	2.7511	2.7162	2.7467	2.6727	**3.63**
**F4**	-115.7719	-117.4650	-120.2202	-117.7317	-122.1750	-117.7322	-119.6997	-117.7324	-120.1322	-117.7293	-119.3803	-115.7717	-117.4650	-120.2200	-117.7317	-122.1749	-117.7322	-119.6998	-117.7324	-120.1322	-117.7293	-119.3804	**-104.5**
Nuclei	OPW91																						
**C1**	130.9989	132.9583	133.9539	133.7056	135.6329	133.6877	134.6754	133.6513	134.8514	133.7031	134.8410	130.9989	132.9582	133.9539	133.7056	135.6330	133.6877	134.6754	133.6513	134.8515	133.7031	134.8410	**132.8**
**C2**	130.7262	130.8703	131.3673	130.7503	131.3340	130.7635	131.6450	130.7599	131.5541	130.7194	131.6496	130.7261	130.8703	131.3673	130.7503	131.3339	130.7635	131.6450	130.7599	131.5542	130.7194	131.6496	**132.9**
**C3**	114.3764	115.1564	115.7533	115.3897	116.1354	115.3831	116.3698	115.3596	116.2991	115.3771	116.4793	114.3764	115.1565	115.7533	115.3897	116.1354	115.3831	116.3698	115.3596	116.2991	115.3771	116.4793	**117.2**
**C4**	165.2041	165.8588	166.4788	166.1876	166.8477	166.1725	167.4033	166.1404	167.2572	166.1838	167.5338	165.2041	165.8588	166.4788	166.1876	166.8476	166.1725	167.4033	166.1404	167.2572	166.1838	167.5338	**167.5**
**C5**	112.4572	113.7054	114.3021	114.2692	115.0406	114.2396	115.3290	114.1931	115.2123	114.2792	115.5116	112.4573	113.7054	114.3021	114.2692	115.0407	114.2396	115.3290	114.1931	115.2123	114.2792	115.5116	**117.2**
**C6**	126.4459	127.7681	128.7165	128.6476	129.9387	128.5911	130.5278	128.5186	130.3040	128.6837	130.8058	126.4460	127.7681	128.7164	128.6476	129.9387	128.5911	130.5278	128.5186	130.3040	128.6837	130.8058	**132.9**
**C7**	193.2350	198.4648	199.7275	201.0721	202.2060	200.9435	206.0081	200.7961	205.1882	201.1740	206.7657	193.2352	198.4648	199.7276	201.0721	202.2063	200.9435	206.0081	200.7961	205.1882	201.1740	206.7657	**195.8**
**C8**	63.7228	65.5786	66.2262	66.3004	67.3297	66.2783	67.7924	66.2379	67.6675	66.3014	68.0264	63.7228	65.5786	66.2262	66.3004	67.3296	66.2783	67.7924	66.2379	67.6675	66.3014	68.0264	**68.4**
**C9**	34.4593	34.3374	34.4633	34.2679	34.4630	34.2763	34.7706	34.2678	34.6619	34.2417	34.8495	34.4593	34.3374	34.4633	34.2679	34.4630	34.2763	34.7706	34.2678	34.6619	34.2417	34.8495	**30.5**
**C10**	26.4933	27.0116	27.4118	27.2602	27.9311	27.2485	27.7713	27.2200	27.7463	27.2531	27.9654	26.4932	27.0116	27.4118	27.2602	27.9312	27.2485	27.7713	27.2200	27.7464	27.2531	27.9654	**27.6**
**C11**	27.4281	27.6358	27.8457	27.6079	27.9463	27.6176	27.9231	27.6102	27.9202	27.5798	28.0426	27.4281	27.6358	27.8457	27.6079	27.9462	27.6176	27.9231	27.6102	27.9203	27.5798	28.0426	**23.1**
**C12**	16.1147	16.3379	16.3532	16.4317	16.4668	16.4307	16.4541	16.4131	16.4540	16.4146	16.4592	16.1147	16.3380	16.3532	16.4317	16.4669	16.4307	16.4541	16.4131	16.4540	16.4146	16.4592	**13.8**
**C1`**	49.9744	50.7955	51.0613	51.2629	51.7275	51.2393	51.7560	51.1989	51.7048	51.2672	52.1859	54.2713	55.3450	55.6693	55.7494	56.2629	55.7320	56.3386	55.6974	56.2757	55.7475	56.5821	**53.5**
**C2`**	25.2138	25.7880	26.0320	25.9569	26.3447	25.9547	26.3594	25.9356	26.3475	25.9405	26.1094	25.0229	25.3126	25.5528	25.3973	25.7998	25.3995	25.7152	25.3852	25.7270	25.3771	25.4366	**24.5**
**C3`**	25.0230	25.3125	25.5528	25.3973	25.7999	25.3995	25.7152	25.3852	25.7270	25.3771	25.4366	25.2138	25.7880	26.0320	25.9569	26.3447	25.9547	26.3594	25.9356	26.3475	25.9405	26.1094	**24.3**
**C4`**	54.2713	55.3450	55.6693	55.7494	56.2627	55.7320	56.3386	55.6974	56.2757	55.7475	56.5821	49.9744	50.7955	51.0613	51.2629	51.7274	51.2393	51.7560	51.1989	51.7048	51.2672	52.1859	**57.5**
**H2**	8.3657	8.1926	8.2058	8.1146	8.0958	8.1174	8.1723	8.1200	8.1563	8.1104	8.1754	8.3657	8.1926	8.2058	8.1146	8.0958	8.1174	8.1723	8.1200	8.1564	8.1104	8.1754	**8.11**
**H3**	7.2334	7.3518	7.4382	7.4004	7.5160	7.3980	7.5403	7.3952	7.5319	7.4010	7.5508	7.2334	7.3518	7.4382	7.4004	7.5160	7.3980	7.5403	7.3952	7.5319	7.4010	7.5508	**7.31**
**H5**	7.0105	7.2283	7.3185	7.3163	7.4423	7.3115	7.4569	7.3065	7.4459	7.3190	7.4773	7.0105	7.2283	7.3185	7.3163	7.4423	7.3115	7.4569	7.3065	7.4458	7.3190	7.4773	**7.31**
**H6**	7.9098	7.9795	8.0579	8.0377	8.1442	8.0324	8.1963	8.0270	8.1738	8.0412	8.2151	7.9098	7.9795	8.0579	8.0377	8.1442	8.0324	8.1963	8.0270	8.1738	8.0412	8.2151	**8.11**
**H8**	4.9491	4.9159	4.9327	4.9236	4.9747	4.9217	4.9913	4.9198	4.9796	4.9239	4.9859	4.9491	4.9159	4.9327	4.9236	4.9747	4.9217	4.9913	4.9198	4.9796	4.9239	4.9859	**5.18**
**H(9A-9B)**	1.6497	1.6605	1.6494	1.6849	1.6763	1.6824	1.7032	1.6798	1.6890	1.6857	1.7429	1.6497	1.6605	1.6494	1.6849	1.6763	1.6824	1.7032	1.6798	1.6890	1.6857	1.7429	**2.05**
**H(10A)**	0.9129	0.8260	0.7820	0.8013	0.7323	0.8023	0.7801	0.8031	0.7773	0.7988	0.7858	0.9129	0.8260	0.7820	0.8013	0.7323	0.8023	0.7801	0.8031	0.7773	0.7988	0.7858	**1.14**
**H(10B)**	1.5483	1.4585	1.4043	1.3926	1.3200	1.3969	1.3127	1.4010	1.3200	1.3868	1.2904	1.5483	1.4585	1.4043	1.3926	1.3200	1.3969	1.3127	1.4010	1.3200	1.3868	1.2904	**1.39**
**H(11A-11B)**	1.0395	1.0599	1.0261	1.0765	1.0389	1.0755	1.0369	1.0742	1.0367	1.0758	1.0217	1.0396	1.0599	1.0261	1.0765	1.0389	1.0755	1.0369	1.0742	1.0367	1.0758	1.0217	**1.22**
**H(12A-12C)**	0.8777	0.8739	0.8474	0.8694	0.8320	0.8698	0.8377	0.8698	0.8386	0.8674	0.8317	0.8777	0.8739	0.8474	0.8694	0.8321	0.8698	0.8377	0.8698	0.8386	0.8674	0.8317	**0.77**
**H(1`A)**	2.7694	2.7663	2.7435	2.7534	2.7049	2.7542	2.7183	2.7548	2.7196	2.7509	2.6771	2.9218	2.9220	2.8985	2.8920	2.8459	2.8944	2.8559	2.8966	2.8599	2.8879	2.8245	**3.16**
**H(1`B)**	3.2959	3.3429	3.3734	3.3768	3.4303	3.3745	3.4237	3.3719	3.4196	3.3773	3.4149	2.9704	2.8525	2.8394	2.8380	2.8351	2.8379	2.8108	2.8378	2.8160	2.8365	2.8041	**3.76**
**H(2`A-2`B)**	1.7263	1.7526	1.7171	1.7767	1.7253	1.7749	1.7403	1.7728	1.7359	1.7769	1.7607	1.6372	1.7089	1.6758	1.7515	1.7042	1.7490	1.7297	1.7461	1.7257	1.7524	1.7615	**2.13**
**H(3`A-3`B)**	1.6372	1.7089	1.6758	1.7515	1.7042	1.7490	1.7297	1.7461	1.7257	1.7524	1.7615	1.7263	1.7526	1.7171	1.7767	1.7253	1.7749	1.7403	1.7728	1.7359	1.7769	1.7607	**2.05**
**H(4`A)**	2.9704	2.8525	2.8394	2.8380	2.8351	2.8379	2.8108	2.8378	2.8160	2.8365	2.8041	3.2959	3.3429	3.3734	3.3768	3.4303	3.3745	3.4237	3.3719	3.4196	3.3773	3.4149	**3.09**
**H(4`B)**	2.9218	2.9219	2.8985	2.8920	2.8459	2.8944	2.8559	2.8966	2.8599	2.8879	2.8245	2.7694	2.7663	2.7435	2.7534	2.7049	2.7542	2.7183	2.7548	2.7196	2.7509	2.6771	**3.63**
**F4**	-116.3894	-118.1382	-120.9029	-118.4211	-122.8862	-118.4208	-120.4042	-118.4200	-120.8382	-118.4199	-120.0932	-116.3894	-118.1382	-120.9028	-118.4211	-122.8863	-118.4208	-120.4042	-118.4200	-120.8382	-118.4199	-120.0932	**-104.5**

[a] methyl protons shifts are averaged

[b] according to [6]

Table S6. Cont. XI

Nuclei	R											S											Exp. [b]
	PBE																						
	Gas	$CHCl_3$ (PCM)	$CHCl_3$ (SMD)	DMSO (PCM)	DMSO (SMD)	CH_3OH (PCM)	CH_3OH (SMD)	C_2H_5OH (PCM)	C_2H_5OH (SMD)	H_2O (PCM)	H_2O (SMD)	Gas	$CHCl_3$ (PCM)	$CHCl_3$ (SMD)	DMSO (PCM)	DMSO (SMD)	CH_3OH (PCM)	CH_3OH (SMD)	C_2H_5OH (PCM)	C_2H_5OH (SMD)	H_2O (PCM)	H_2O (SMD)	
**C1**	135.7483	136.9338	137.8215	137.3803	138.9543	137.3545	138.4508	137.3389	138.4347	137.4219	138.6539	135.7483	136.9338	137.8215	137.3803	138.9543	137.3545	138.4509	137.3389	138.4347	137.4219	138.6539	**132.8**
**C2**	135.8078	135.8743	136.4215	135.6787	136.3342	135.6882	136.8147	135.7088	136.6975	135.6874	136.7796	135.8078	135.8743	136.4215	135.6787	136.3342	135.6882	136.8147	135.7088	136.6975	135.6874	136.7796	**132.9**
**C3**	119.5174	120.3563	120.9316	120.5420	121.3293	120.5297	121.6675	120.5282	121.5814	120.5709	121.7766	119.5174	120.3563	120.9316	120.5420	121.3293	120.5297	121.6674	120.5282	121.5814	120.5709	121.7766	**117.2**
**C4**	174.2361	175.2702	175.9491	175.6389	176.4757	175.6130	177.0574	175.5980	176.9178	175.6810	177.2200	174.2361	175.2703	175.9491	175.6389	176.4758	175.6130	177.0574	175.5980	176.9178	175.6810	177.2200	**167.5**
**C5**	117.5658	118.8904	119.5358	119.4738	120.4435	119.4339	120.7042	119.4048	120.5965	119.5296	120.8999	117.5658	118.8902	119.5358	119.4738	120.4435	119.4339	120.7041	119.4048	120.5965	119.5296	120.8999	**117.2**
**C6**	132.1464	134.1194	135.2182	135.1667	136.8848	135.0939	137.4752	135.0329	137.2528	135.2539	137.9118	132.1464	134.1194	135.2182	135.1667	136.8848	135.0939	137.4754	135.0329	137.2528	135.2539	137.9118	**132.9**
**C7**	201.1356	205.4775	206.7985	207.6645	208.7676	207.5322	212.7870	207.4100	211.8422	207.8073	213.7751	201.1356	205.4775	206.7985	207.6645	208.7676	207.5322	212.7866	207.4100	211.8422	207.8073	213.7751	**195.8**
**C8**	67.3305	68.6977	69.4799	69.1926	70.5026	69.1716	71.0535	69.1589	70.8716	69.2282	71.4612	67.3305	68.6976	69.4799	69.1926	70.5026	69.1716	71.0534	69.1589	70.8716	69.2282	71.4612	**68.4**
**C9**	38.5481	38.3924	38.5854	38.2027	38.7100	38.2115	39.0042	38.2308	38.9069	38.2116	39.1515	38.5481	38.3925	38.5854	38.2027	38.7100	38.2115	39.0042	38.2308	38.9069	38.2116	39.1515	**30.5**
**C10**	29.5828	30.1383	30.5931	30.3952	31.2462	30.3715	31.0588	30.3592	31.0404	30.4359	31.2758	29.5828	30.1382	30.5931	30.3952	31.2462	30.3715	31.0588	30.3592	31.0404	30.4359	31.2759	**27.6**
**C11**	29.4545	29.6678	29.8873	29.5253	30.0700	29.5343	30.0539	29.5536	30.0482	29.5337	30.2277	29.4545	29.6678	29.8873	29.5253	30.0700	29.5343	30.0540	29.5536	30.0482	29.5337	30.2277	**23.1**
**C12**	16.4263	16.6550	16.6777	16.6936	16.8479	16.6853	16.8192	16.6882	16.8147	16.7199	16.8332	16.4263	16.6550	16.6777	16.6936	16.8479	16.6853	16.8192	16.6882	16.8147	16.7199	16.8332	**13.8**
**C1`**	53.1868	54.1192	54.3314	54.5767	55.2085	54.5426	55.1990	54.5200	55.1018	54.6279	55.7537	57.7309	59.0198	59.3593	59.4308	60.0063	59.4053	60.1988	59.3904	60.1340	59.4724	60.4800	**53.5**
**C2`**	25.7091	26.3084	26.6327	26.4566	27.0413	26.4455	27.0629	26.4450	27.0465	26.4846	26.9107	25.3534	25.6633	25.9658	25.7952	26.3210	25.7806	26.3173	25.7773	26.3051	25.8271	26.2369	**24.5**
**C3`**	25.3534	25.6633	25.9658	25.7952	26.3211	25.7806	26.3173	25.7773	26.3051	25.8271	26.2369	25.7091	26.3084	26.6327	26.4566	27.0413	26.4455	27.0629	26.4450	27.0465	26.4846	26.9108	**24.3**
**C4`**	57.7309	59.0198	59.3593	59.4308	60.0063	59.4053	60.1988	59.3904	60.1340	59.4724	60.4800	53.1868	54.1193	54.3314	54.5767	55.2086	54.5426	55.1991	54.5200	55.1018	54.6279	55.7537	**57.5**
**H2**	8.5422	8.4404	8.4543	8.3726	8.3723	8.3764	8.4041	8.3809	8.4061	8.3697	8.3938	8.5422	8.4404	8.4543	8.3726	8.3723	8.3764	8.4041	8.3809	8.4061	8.3697	8.3938	**8.11**
**H3**	7.2025	7.3512	7.4409	7.3933	7.5117	7.3913	7.5367	7.3898	7.5283	7.3957	7.5512	7.2025	7.3512	7.4409	7.3933	7.5117	7.3913	7.5367	7.3898	7.5283	7.3957	7.5512	**7.31**
**H5**	7.0213	7.1777	7.2686	7.2421	7.3835	7.2384	7.3885	7.2352	7.3797	7.2463	7.4165	7.0213	7.1777	7.2686	7.2421	7.3835	7.2384	7.3885	7.2352	7.3797	7.2463	7.4165	**7.31**
**H6**	7.9455	8.1049	8.1784	8.1913	8.3013	8.1856	8.3527	8.1805	8.3388	8.1973	8.3808	7.9455	8.1050	8.1784	8.1913	8.3014	8.1856	8.3527	8.1805	8.3388	8.1973	8.3808	**8.11**
**H8**	4.9981	5.0221	5.0362	5.0495	5.0820	5.0469	5.1116	5.0449	5.1050	5.0527	5.1021	4.9981	5.0221	5.0362	5.0495	5.0820	5.0469	5.1116	5.0449	5.1050	5.0527	5.1021	**5.18**
**H(9A-9B)**	1.8877	1.9213	1.9179	1.9417	1.9768	1.9405	1.9732	1.9398	1.9667	1.9435	2.0076	1.8877	1.9214	1.9179	1.9417	1.9768	1.9405	1.9733	1.9398	1.9667	1.9435	2.0076	**2.05**
**H(10A)**	0.9903	0.8747	0.8363	0.8393	0.7493	0.8399	0.8067	0.8412	0.7986	0.8395	0.8199	0.9903	0.8747	0.8363	0.8393	0.7493	0.8399	0.8068	0.8413	0.7986	0.8395	0.8199	**1.14**
**H(10B)**	1.7513	1.6439	1.5780	1.5587	1.4835	1.5641	1.4701	1.5700	1.4779	1.5544	1.4380	1.7513	1.6439	1.5780	1.5587	1.4835	1.5641	1.4701	1.5700	1.4779	1.5544	1.4380	**1.39**
**H(11A-11B)**	1.1003	1.1160	1.0803	1.1235	1.0909	1.1231	1.0888	1.1233	1.0885	1.1245	1.0766	1.1003	1.1160	1.0803	1.1235	1.0909	1.1231	1.0888	1.1233	1.0885	1.1245	1.0766	**1.22**
**H(12A-12C)**	0.9133	0.9052	0.8805	0.8941	0.8588	0.8947	0.8663	0.8959	0.8665	0.8942	0.8605	0.9133	0.9052	0.8805	0.8941	0.8588	0.8947	0.8663	0.8959	0.8665	0.8942	0.8605	**0.77**
**H(1`A)**	2.7998	2.7946	2.7843	2.7819	2.7480	2.7827	2.7606	2.7840	2.7625	2.7819	2.7267	2.9257	2.9415	2.9313	2.9245	2.9038	2.9262	2.9082	2.9283	2.9120	2.9235	2.8692	**3.16**
**H(1`B)**	3.3750	3.4144	3.4496	3.4414	3.5072	3.4395	3.4925	3.4380	3.4919	3.4439	3.4830	3.0909	2.9057	2.8913	2.8575	2.8578	2.8592	2.8162	2.8617	2.8198	2.8566	2.7942	**3.76**
**H(2`A-2`B)**	1.8055	1.8330	1.7994	1.8565	1.8130	1.8547	1.8265	1.8534	1.8218	1.8590	1.8472	1.6742	1.7478	1.7141	1.7866	1.7430	1.7841	1.7717	1.7821	1.7633	1.7896	1.8081	**2.13**
**H(3`A-3`B)**	1.6742	1.7478	1.7141	1.7866	1.7430	1.7841	1.7717	1.7821	1.7633	1.7896	1.8081	1.8055	1.8330	1.7994	1.8565	1.8130	1.8547	1.8265	1.8534	1.8218	1.8590	1.8472	**2.05**
**H(4`A)**	3.0909	2.9057	2.8913	2.8575	2.8578	2.8592	2.8162	2.8617	2.8198	2.8566	2.7942	3.3750	3.4144	3.4496	3.4414	3.5072	3.4395	3.4925	3.4380	3.4919	3.4439	3.4830	**3.09**
**H(4`B)**	2.9257	2.9415	2.9313	2.9245	2.9038	2.9262	2.9082	2.9283	2.9120	2.9235	2.8692	2.7998	2.7946	2.7843	2.7819	2.7480	2.7827	2.7606	2.7840	2.7625	2.7819	2.7267	**3.63**
**F4**	-131.3443	-132.3866	-135.5345	-132.3677	-137.2648	-132.3799	-134.6625	-132.3899	-135.0650	-132.3559	-134.2918	-131.3443	-132.3863	-135.5345	-132.3677	-137.2648	-132.3799	-134.6625	-132.3899	-135.0650	-132.3559	-134.2918	**-104.5**
Nuclei	PBE0																						
**C1**	136.4530	137.3089	138.0508	137.7354	139.2688	137.7148	138.6567	137.6949	138.6719	137.7566	138.8652	136.4530	137.3090	138.0509	137.7354	139.2688	137.7148	138.6567	137.6949	138.6719	137.7566	138.8651	**132.8**
**C2**	138.6013	138.6001	139.0847	138.4264	138.9615	138.4331	139.5038	138.4418	139.3326	138.4230	139.4291	138.6013	138.6001	139.0847	138.4264	138.9613	138.4331	139.5038	138.4418	139.3328	138.4230	139.4292	**132.9**
**C3**	120.9188	121.6798	122.2526	121.8997	122.5990	121.8840	122.9416	121.8699	122.8160	121.9174	122.9678	120.9188	121.6797	122.2525	121.8997	122.5988	121.8840	122.9416	121.8699	122.8159	121.9174	122.9677	**117.2**
**C4**	172.9248	173.7704	174.3607	174.1635	174.7944	174.1342	175.5017	174.1070	175.2844	174.1944	175.5847	172.9248	173.7702	174.3606	174.1635	174.7943	174.1342	175.5017	174.1070	175.2844	174.1944	175.5847	**167.5**
**C5**	118.8873	120.2205	120.8491	120.8784	121.7495	120.8319	122.0378	120.7874	121.8751	120.9256	122.1589	118.8873	120.2204	120.8491	120.8784	121.7495	120.8319	122.0378	120.7874	121.8751	120.9256	122.1590	**117.2**
**C6**	134.7534	136.6483	137.8002	137.7535	139.3337	137.6742	140.0781	137.5975	139.7552	137.8328	140.4055	134.7534	136.6484	137.8002	137.7535	139.3337	137.6742	140.0781	137.5975	139.7551	137.8328	140.4054	**132.9**
**C7**	201.0382	205.4916	206.6635	207.9368	208.6564	207.7907	213.5288	207.6448	212.4021	208.0776	214.5410	201.0382	205.4915	206.6632	207.9368	208.6564	207.7907	213.5288	207.6448	212.4025	208.0776	214.5410	**195.8**
**C8**	63.3741	64.5227	65.2451	65.0149	66.0343	64.9854	66.6890	64.9567	66.5032	65.0447	67.0482	63.3741	64.5227	65.2451	65.0149	66.0342	64.9854	66.6890	64.9567	66.5033	65.0447	67.0483	**68.4**
**C9**	35.2480	35.0267	35.2621	34.8927	35.2211	34.8944	35.4911	34.8977	35.3528	34.8940	35.5658	35.2480	35.0267	35.2622	34.8927	35.2211	34.8944	35.4911	34.8978	35.3528	34.8940	35.5657	**30.5**
**C10**	27.5160	27.9999	28.4469	28.3217	29.0203	28.2908	28.9160	28.2627	28.8347	28.3546	29.0522	27.5160	27.9999	28.4468	28.3217	29.0203	28.2908	28.9160	28.2627	28.8348	28.3546	29.0522	**27.6**
**C11**	27.3762	27.5042	27.7451	27.4397	27.8548	27.4401	27.9245	27.4421	27.8635	27.4419	28.0248	27.3761	27.5043	27.7451	27.4397	27.8548	27.4401	27.9245	27.4422	27.8636	27.4419	28.0247	**23.1**
**C12**	16.1669	16.3506	16.3714	16.4494	16.5174	16.4361	16.5489	16.4251	16.4828	16.4654	16.4981	16.1668	16.3507	16.3713	16.4494	16.5175	16.4361	16.5489	16.4250	16.4827	16.4654	16.4981	**13.8**
**C1`**	50.3442	51.1746	51.3791	51.6553	52.1863	51.6178	52.2537	51.5828	52.1219	51.6945	52.6719	54.6441	55.7362	56.0471	56.1526	56.6508	56.1232	56.8627	56.0957	56.7432	56.1835	57.0525	**53.5**
**C2`**	24.3672	24.7765	25.0807	24.9101	25.3550	24.8969	25.4419	24.8857	25.3814	24.9254	25.2400	23.8126	24.0541	24.3180	24.1905	24.6209	24.1765	24.6494	24.1645	24.5778	24.2070	24.4911	**24.5**
**C3`**	23.8126	24.0541	24.3180	24.1905	24.6209	24.1765	24.6494	24.1645	24.5779	24.2070	24.4910	24.3672	24.7765	25.0807	24.9101	25.3550	24.8969	25.4419	24.8857	25.3814	24.9254	25.2399	**24.3**
**C4`**	54.6441	55.7362	56.0470	56.1526	56.6509	56.1232	56.8627	56.0957	56.7433	56.1835	57.0525	50.3442	51.1747	51.3791	51.6553	52.1864	51.6178	52.2537	51.5828	52.1220	51.6945	52.6719	**57.5**
**H2**	8.7672	8.6747	8.6943	8.6043	8.6001	8.6077	8.6358	8.6113	8.6278	8.6008	8.6154	8.7672	8.6747	8.6943	8.6043	8.6001	8.6077	8.6358	8.6113	8.6278	8.6008	8.6154	**8.11**
**H3**	7.3555	7.5027	7.5909	7.5485	7.6660	7.5463	7.6937	7.5440	7.6820	7.5502	7.7053	7.3555	7.5027	7.5909	7.5485	7.6660	7.5463	7.6937	7.5440	7.6820	7.5502	7.7053	**7.31**
**H5**	7.1793	7.3531	7.4495	7.4232	7.5573	7.4188	7.5698	7.4143	7.5575	7.4270	7.5924	7.1793	7.3531	7.4495	7.4232	7.5573	7.4188	7.5698	7.4143	7.5575	7.4270	7.5924	**7.31**
**H6**	8.0691	8.2434	8.3303	8.3437	8.4667	8.3371	8.5292	8.3305	8.5036	8.3498	8.5531	8.0691	8.2434	8.3303	8.3437	8.4667	8.3371	8.5292	8.3305	8.5036	8.3498	8.5531	**8.11**
**H8**	4.5650	4.6235	4.6513	4.6787	4.7273	4.6747	4.7749	4.6709	4.7514	4.6822	4.7725	4.5650	4.6235	4.6513	4.6787	4.7273	4.6747	4.7749	4.6708	4.7514	4.6822	4.7725	**5.18**
**H(9A-9B)**	1.7345	1.7658	1.7709	1.7908	1.8071	1.7885	1.8131	1.7862	1.8041	1.7927	1.8421	1.7345	1.7658	1.7709	1.7908	1.8071	1.7885	1.8131	1.7862	1.8041	1.7927	1.8421	**2.05**
**H(10A)**	0.9439	0.8046	0.7615	0.7568	0.6649	0.7587	0.9607	0.7606	0.6992	0.7548	0.7188	0.9439	0.8046	0.7615	0.7568	0.6649	0.7587	0.7124	0.7606	0.6992	0.7548	0.7189	**1.14**
**H(10B)**	1.6350	1.5420	1.4670	1.4541	1.3671	1.4600	1.4933	1.4660	1.3682	1.4482	1.3328	1.6350	1.5420	1.4670	1.4541	1.3671	1.4600	1.3654	1.4660	1.3682	1.4482	1.3327	**1.39**
**H(11A-11B)**	1.0056	1.0247	0.9871	1.0334	0.9945	1.0329	0.9994	1.0324	0.9951	1.0336	0.9846	1.0056	1.0247	0.9871	1.0334	0.9945	1.0329	0.9994	1.0324	0.9951	1.0336	0.9846	**1.22**
**H(12A-12C)**	0.8954	0.8818	0.8542	0.8706	0.8300	0.8712	0.8408	0.8719	0.8377	0.8697	0.8328	0.8954	0.8818	0.8542	0.8706	0.8300	0.8712	0.8408	0.8719	0.8377	0.8697	0.8328	**0.77**
**H(1`A)**	2.6626	2.6552	2.6426	2.6448	2.6013	2.6453	2.8989	2.6459	2.6192	2.6440	2.5936	2.8001	2.8064	2.7926	2.7859	2.7548	2.7878	2.7693	2.7895	2.7706	2.7838	2.7342	**3.16**
**H(1`B)**	3.1711	3.2099	3.2462	3.2445	3.3001	3.2418	3.6043	3.2391	3.2874	3.2468	3.2911	2.8887	2.7177	2.7028	2.6786	2.6737	2.6802	2.6333	2.6819	2.6335	2.6768	2.6159	**3.76**
**H(2`A-2`B)**	1.7041	1.7358	1.7004	1.7657	1.7181	1.7636	1.9209	1.7614	1.7270	1.7676	1.7507	1.5851	1.6586	1.6192	1.7000	1.6542	1.6974	1.6886	1.6949	1.6778	1.7022	1.7191	**2.13**
**H(3`A-3`B)**	1.5851	1.6586	1.6192	1.7000	1.6542	1.6974	1.9103	1.6948	1.6778	1.7022	1.7191	1.7041	1.7358	1.7004	1.7657	1.7181	1.7636	1.7348	1.7614	1.7270	1.7676	1.7507	**2.05**
**H(4`A)**	2.8887	2.7177	2.7028	2.6786	2.6737	2.6802	2.9914	2.6818	2.6335	2.6768	2.6159	3.1711	3.2099	3.2462	3.2445	3.3001	3.2418	3.2940	3.2391	3.2874	3.2468	3.2911	**3.09**
**H(4`B)**	2.8001	2.8064	2.7926	2.7859	2.7548	2.7878	3.0365	2.7895	2.7706	2.7838	2.7342	2.6626	2.6552	2.6426	2.6448	2.6013	2.6453	2.6219	2.6459	2.6192	2.6440	2.5936	**3.63**
**F4**	-113.6775	-114.6292	-117.1471	-114.6715	-118.6310	-114.6782	-116.3951	-114.6840	-116.7842	-114.6635	-116.1705	-113.6775	-114.6293	-117.1472	-114.6715	-118.6310	-114.6782	-116.3951	-114.6840	-116.7840	-114.6635	-116.1704	**-104.5**

[a] methyl protons shifts are averaged

[b] according to [6]

Table S6. Cont. XII

Nuclei	R											S											Exp. [b]
	TPSS																						
	Gas	$CHCl_3$ (PCM)	$CHCl_3$ (SMD)	DMSO (PCM)	DMSO (SMD)	CH_3OH (PCM)	CH_3OH (SMD)	C_2H_5OH (PCM)	C_2H_5OH (SMD)	H_2O (PCM)	H_2O (SMD)	Gas	$CHCl_3$ (PCM)	$CHCl_3$ (SMD)	DMSO (PCM)	DMSO (SMD)	CH_3OH (PCM)	CH_3OH (SMD)	C_2H_5OH (PCM)	C_2H_5OH (SMD)	H_2O (PCM)	H_2O (SMD)	
**C1**	132.4356	133.1505	133.9345	133.5892	135.0046	133.5760	134.3940	133.5726	134.0790	133.6107	134.7819	132.4356	133.1505	133.9343	133.5892	135.0046	133.5760	134.3940	133.5726	134.6812	133.6107	134.7819	**132.8**
**C2**	132.8929	132.8526	133.4725	132.7671	133.3034	132.7794	133.7783	132.8032	133.3065	132.7661	133.7282	132.8929	132.8526	133.4725	132.7671	133.3034	132.7793	133.7783	132.8032	133.9176	132.7661	133.7282	**132.9**
**C3**	116.6681	117.3246	117.9791	117.5839	118.2462	117.5771	118.5755	117.5815	118.0239	117.6006	118.6358	116.6681	117.3246	117.9791	117.5839	118.2462	117.5771	118.5755	117.5815	118.8101	117.6006	118.6358	**117.2**
**C4**	170.8911	171.7397	172.4965	172.2075	172.9099	172.1863	173.5612	172.1764	172.8372	172.2385	173.6673	170.8911	171.7397	172.4965	172.2075	172.9099	172.1863	173.5612	172.1764	172.9955	172.2385	173.6673	**167.5**
**C5**	114.7722	115.9311	116.6167	116.5690	117.3927	116.5353	117.6393	116.5131	116.9513	116.6120	117.7796	114.7722	115.9311	116.6167	116.5690	117.3927	116.5353	117.6393	116.5131	117.7497	116.6120	117.7796	**117.2**
**C6**	129.3185	131.1397	132.2995	132.2804	133.7885	132.2131	134.5062	132.1577	132.6584	132.3555	134.8302	129.3185	131.1397	132.2995	132.2804	133.7885	132.2131	134.5062	132.1577	133.2770	132.3555	134.8302	**132.9**
**C7**	197.2729	201.1909	202.3750	203.3951	204.2125	203.2728	208.3467	203.1606	203.9454	203.5200	209.4808	197.2729	201.1909	202.3748	203.3951	204.2125	203.2728	208.3467	203.1606	203.7473	203.5200	209.4808	**195.8**
**C8**	66.1554	67.2219	67.8668	67.7200	68.8092	67.7027	69.2644	67.6953	67.9382	67.7454	69.7480	66.1554	67.2219	67.8668	67.7200	68.8092	67.7027	69.2644	67.6953	69.2980	67.7454	69.7480	**68.4**
**C9**	37.7531	37.5421	37.8061	37.4640	37.8295	37.4741	38.1342	37.4954	37.6175	37.4651	38.2427	37.7531	37.5421	37.8059	37.4640	37.8295	37.4740	38.1342	37.4954	39.3246	37.4651	38.2427	**30.5**
**C10**	29.1817	29.5893	30.1503	29.9117	30.6464	29.8939	30.4886	29.8881	29.9798	29.9393	30.6120	29.1817	29.5893	30.1502	29.9117	30.6464	29.8939	30.4886	29.8881	31.7744	29.9393	30.6120	**27.6**
**C11**	29.3660	29.4429	29.7794	29.4146	29.8735	29.4257	29.8732	29.4479	29.5378	29.4144	30.0060	29.3660	29.4429	29.7794	29.4146	29.8736	29.4258	29.8732	29.4479	31.3375	29.4144	30.0060	**23.1**
**C12**	17.2694	17.3881	17.5648	17.5303	17.6714	17.5262	17.6562	17.5339	17.5761	17.5450	17.6292	17.2694	17.3881	17.5648	17.5303	17.6714	17.5262	17.6562	17.5339	19.5128	17.5450	17.6292	**13.8**
**C1`**	53.0445	53.7558	54.1667	54.3103	54.8418	54.2794	54.8722	54.2605	54.4497	54.3511	55.3404	57.2249	58.3026	58.7280	58.7950	59.3005	58.7753	59.5194	58.7666	60.4363	58.8238	59.7915	**53.5**
**C2`**	26.3341	26.7286	27.1499	26.9392	27.4229	26.9349	27.4595	26.9415	27.0214	26.9534	27.2605	26.0765	26.1994	26.6761	26.4209	26.8925	26.4135	26.9030	26.4175	28.3298	26.4384	26.7317	**24.5**
**C3`**	26.0765	26.1994	26.6761	26.4209	26.8925	26.4136	26.9030	26.4175	26.4953	26.4384	26.7317	26.3341	26.7286	27.1499	26.9392	27.4229	26.9349	27.4595	26.9415	28.8499	26.9534	27.2605	**24.3**
**C4`**	57.2249	58.3026	58.7280	58.7950	59.3005	58.7753	59.5194	58.7666	58.9738	58.8238	59.7915	53.0445	53.7558	54.1668	54.3103	54.8419	54.2794	54.8722	54.2605	55.9640	54.3511	55.3404	**57.5**
**H2**	8.5524	8.4665	8.5107	8.4102	8.4107	8.4132	8.4523	8.4170	8.2571	8.4079	8.4133	8.5524	8.4665	8.5107	8.4102	8.4107	8.4132	8.4523	8.4170	8.5271	8.4079	8.4133	**8.11**
**H3**	7.1623	7.3069	7.4039	7.3576	7.4767	7.3553	7.5043	7.3535	7.1893	7.3601	7.5144	7.1623	7.3069	7.4039	7.3576	7.4767	7.3553	7.5043	7.3535	7.4716	7.3601	7.5144	**7.31**
**H5**	6.9935	7.1616	7.2589	7.2313	7.3713	7.2271	7.3755	7.2236	7.0589	7.2357	7.3952	6.9935	7.1616	7.2589	7.2313	7.3713	7.2271	7.3755	7.2236	7.3426	7.2357	7.3952	**7.31**
**H6**	7.8543	8.0239	8.1241	8.1246	8.2413	8.1183	8.3087	8.1126	7.9514	8.1310	8.3186	7.8543	8.0239	8.1241	8.1246	8.2413	8.1183	8.3087	8.1126	8.2250	8.1310	8.3186	**8.11**
**H8**	4.7647	4.8126	4.8538	4.8581	4.9078	4.8551	4.9504	4.8528	4.6786	4.8614	4.9315	4.7647	4.8126	4.8538	4.8581	4.9078	4.8551	4.9504	4.8528	4.9896	4.8614	4.9315	**5.18**
**H(9A-9B)**	1.8001	1.8427	1.8422	1.8732	1.9021	1.8708	1.9032	1.8691	1.6830	1.8759	1.9251	1.8001	1.8427	1.8422	1.8732	1.9021	1.8708	1.9032	1.8691	2.0283	1.8759	1.9251	**2.05**
**H(10A)**	1.0002	0.8769	0.8927	0.8464	0.7648	0.8472	0.8274	0.8487	0.6585	0.8461	0.8190	1.0002	0.8769	0.8927	0.8464	0.7648	0.8472	0.8274	0.8487	1.0155	0.8461	0.8190	**1.14**
**H(10B)**	1.7385	1.6228	1.5525	1.5379	1.4588	1.5440	1.4467	1.5505	1.3631	1.5325	1.4054	1.7385	1.6228	1.5525	1.5379	1.4588	1.5440	1.4467	1.5505	1.9732	1.5325	1.4054	**1.39**
**H(11A-11B)**	1.0583	1.0759	1.0492	1.0919	1.0592	1.0911	1.0615	1.0909	0.9017	1.0929	1.0424	1.0583	1.0759	1.0492	1.0919	1.0592	1.0911	1.0615	1.0909	1.2559	1.0929	1.0424	**1.22**
**H(12A-12C)**	0.9078	0.8948	0.8800	0.8900	0.8562	0.8904	0.8645	0.8914	0.7014	0.8900	0.8548	0.9078	0.8948	0.8800	0.8900	0.8562	0.8904	0.8645	0.8914	1.0579	0.8900	0.8548	**0.77**
**H(1`A)**	2.7122	2.7112	2.7093	2.7074	2.6743	2.7077	2.6920	2.7086	2.5258	2.7075	2.6628	2.8273	2.8487	2.8382	2.8410	2.8258	2.8424	2.8307	2.8444	3.0399	2.8398	2.7945	**3.16**
**H(1`B)**	3.2745	3.3206	3.3526	3.3541	3.4150	3.3518	3.4072	3.3500	3.1698	3.3568	3.3950	3.0710	2.8744	2.8447	2.8264	2.8319	2.8280	2.7779	2.8303	2.9220	2.8255	2.7603	**3.76**
**H(2`A-2`B)**	1.7567	1.7877	1.7621	1.8208	1.7796	1.8184	1.7937	1.8167	1.6303	1.8235	1.8103	1.6119	1.6876	1.6609	1.7350	1.6925	1.7321	1.7200	1.7299	2.2245	1.7380	1.7577	**2.13**
**H(3`A-3`B)**	1.6119	1.6876	1.6609	1.7350	1.6925	1.7321	1.7200	1.7299	1.5432	1.7380	1.7577	1.7567	1.7877	1.7621	1.8208	1.7796	1.8184	1.7937	1.8167	2.0876	1.8235	1.8103	**2.05**
**H(4`A)**	3.0710	2.8744	2.8447	2.8264	2.8318	2.8280	2.7779	2.8303	2.6480	2.8255	2.7603	3.2745	3.3206	3.3526	3.3541	3.4150	3.3518	3.4072	3.3500	3.4375	3.3568	3.3950	**3.09**
**H(4`B)**	2.8273	2.8487	2.8382	2.8410	2.8258	2.8424	2.8307	2.8444	2.6622	2.8398	2.7945	2.7122	2.7112	2.7093	2.7074	2.6743	2.7077	2.6920	2.7086	2.7416	2.7075	2.6628	**3.63**
**F4**	-124.9440	-126.1785	-129.1128	-126.2557	-130.9715	-126.2608	-128.2470	-126.2651	-127.7783	-126.2491	-128.0382	-124.9440	-126.1785	-129.1129	-126.2557	-130.9715	-126.2608	-128.2470	-126.2651	-124.7287	-126.2491	-128.0382	**-104.5**
Nuclei	VSXC																						
**C1**	133.6805	134.5405	135.1885	134.8465	136.5075	134.8331	135.4592	134.8176	135.4598	134.8603	135.9108	133.6804	134.5405	135.1885	134.8465	136.5076	134.8331	135.4593	134.8176	135.4596	134.8603	135.9108	**132.8**
**C2**	130.8568	131.2148	131.6668	131.2177	131.8000	131.2250	132.3394	131.2296	132.2150	131.2117	132.4206	130.8567	131.2148	131.6668	131.2177	131.8000	131.2250	132.3394	131.2296	132.2150	131.2117	132.4206	**132.9**
**C3**	114.9211	115.9128	116.4365	116.3181	116.9073	116.2991	117.2645	116.2774	117.2025	116.3375	117.3624	114.9211	115.9128	116.4365	116.3181	116.9072	116.2991	117.2645	116.2774	117.2024	116.3375	117.3624	**117.2**
**C4**	168.3003	169.1032	169.7919	169.5485	170.2398	169.5239	170.8109	169.4971	170.6743	169.5735	170.9192	168.3003	169.1032	169.7919	169.5485	170.2398	169.5239	170.8108	169.4971	170.6743	169.5735	170.9192	**167.5**
**C5**	112.7729	114.1638	114.7218	114.9628	115.5777	114.9149	115.9637	114.8652	115.8581	115.0102	116.1017	112.7730	114.1638	114.7218	114.9628	115.5778	114.9149	115.9637	114.8652	115.8581	115.0102	116.1017	**117.2**
**C6**	128.4606	129.9982	131.1249	130.9894	132.3974	130.9269	132.9648	130.8631	132.7529	131.0511	133.1730	128.4606	129.9982	131.1249	130.9894	132.3975	130.9269	132.9647	130.8631	132.7528	131.0511	133.1730	**132.9**
**C7**	193.6144	198.7380	199.9101	201.2950	202.2459	201.1444	206.3068	200.9911	205.2665	201.4392	207.5617	193.6144	198.7380	199.9101	201.2950	202.2458	201.1444	206.3068	200.9911	205.2666	201.4392	207.5617	**195.8**
**C8**	71.2958	72.3979	72.9108	72.9017	73.7341	72.8757	74.0459	72.8472	73.8255	72.9277	74.6281	71.2958	72.3979	72.9108	72.9018	73.7341	72.8757	74.0459	72.8472	73.8255	72.9277	74.6281	**68.4**
**C9**	41.7566	42.2479	42.5811	42.4292	42.7524	42.4240	43.2429	42.4162	43.1046	42.4354	43.3121	41.7566	42.2479	42.5811	42.4293	42.7525	42.4240	43.2429	42.4162	43.1046	42.4354	43.3121	**30.5**
**C10**	36.4853	37.4189	37.7243	38.0913	38.8133	38.0483	38.7335	38.0038	38.7135	38.1344	39.0207	36.4853	37.4189	37.7243	38.0913	38.8133	38.0483	38.7334	38.0038	38.7134	38.1344	39.0207	**27.6**
**C11**	35.7245	35.7671	36.0142	35.6725	36.0233	35.6843	35.9517	35.6935	35.9513	35.6625	35.9686	35.7245	35.7671	36.0142	35.6725	36.0233	35.6843	35.9516	35.6935	35.9513	35.6625	35.9686	**23.1**
**C12**	18.7135	18.9231	19.0967	19.1393	19.3695	19.1260	19.2353	19.1108	19.2302	19.1539	19.2772	18.7135	18.9231	19.0967	19.1393	19.3695	19.1260	19.2353	19.1108	19.2301	19.1539	19.2772	**13.8**
**C1`**	58.8714	59.4981	59.7451	59.8519	60.3179	59.8335	60.1483	59.8130	60.1663	59.8709	60.3243	60.9536	61.9037	62.1420	62.3605	62.9177	62.3370	62.8341	62.3113	62.7698	62.3842	63.2050	**53.5**
**C2`**	29.5342	30.0310	30.1911	30.2855	30.6314	30.2737	30.6152	30.2596	30.5864	30.2979	30.6852	28.4893	28.8029	29.0331	29.0094	29.3917	28.9983	29.3626	28.9854	29.3504	29.0216	29.4075	**24.5**
**C3`**	28.4892	28.8029	29.0331	29.0094	29.3918	28.9983	29.3625	28.9854	29.3504	29.0216	29.4075	29.5341	30.0310	30.1911	30.2855	30.6313	30.2737	30.6153	30.2596	30.5863	30.2979	30.6852	**24.3**
**C4`**	60.9537	61.9037	62.1420	62.3605	62.9176	62.3370	62.8341	62.3113	62.7699	62.3842	63.2050	58.8714	59.4981	59.7451	59.8519	60.3180	59.8335	60.1482	59.8130	60.1664	59.8710	60.3243	**57.5**
**H2**	8.5227	8.5081	8.5164	8.4735	8.4550	8.4764	8.5360	8.4794	8.5408	8.4706	8.5151	8.5227	8.5081	8.5164	8.4735	8.4550	8.4764	8.5360	8.4794	8.5408	8.4706	8.5151	**8.11**
**H3**	7.1829	7.3512	7.4296	7.4126	7.5247	7.4094	7.5501	7.4062	7.5412	7.4155	7.5801	7.1829	7.3512	7.4296	7.4126	7.5247	7.4094	7.5501	7.4062	7.5412	7.4155	7.5801	**7.31**
**H5**	7.0781	7.2694	7.3540	7.3564	7.4727	7.3513	7.4935	7.3463	7.4820	7.3612	7.5113	7.0781	7.2694	7.3540	7.3564	7.4727	7.3513	7.4935	7.3463	7.4820	7.3612	7.5113	**7.31**
**H6**	7.8503	8.0072	8.0875	8.1024	8.2216	8.0962	8.2794	8.0902	8.2620	8.1082	8.3027	7.8503	8.0072	8.0875	8.1024	8.2216	8.0962	8.2794	8.0902	8.2620	8.1082	8.3027	**8.11**
**H8**	4.2573	4.4037	4.4274	4.4919	4.5768	4.4861	4.6320	4.4805	4.6030	4.4974	4.6706	4.2573	4.4037	4.4274	4.4919	4.5768	4.4861	4.6320	4.4805	4.6030	4.4974	4.6706	**5.18**
**H(9A-9B)**	1.9641	1.9764	1.9714	1.9883	1.9833	1.9875	1.9808	1.9869	1.9785	1.9889	1.9808	1.9641	1.9764	1.9714	1.9883	1.9833	1.9875	1.9808	1.9869	1.9785	1.9889	1.9808	**2.05**
**H(10A)**	0.9306	0.9326	0.9128	0.9285	0.9053	0.9291	0.8940	0.9297	0.8921	0.9279	0.8858	0.9306	0.9326	0.9128	0.9285	0.9053	0.9291	0.8940	0.9297	0.8921	0.9279	0.8858	**1.14**
**H(10B)**	2.2764	2.1111	2.0177	1.9949	1.8578	2.0028	1.8386	2.0106	1.8498	1.9873	1.7818	2.2764	2.1111	2.0177	1.9949	1.8578	2.0028	1.8386	2.0106	1.8498	1.9873	1.7818	**1.39**
**H(11A-11B)**	1.3715	1.3906	1.3742	1.4051	1.3862	1.4042	1.3808	1.4034	1.3793	1.4059	1.3754	1.3715	1.3906	1.3742	1.4051	1.3862	1.4042	1.3808	1.4034	1.3793	1.4059	1.3754	**1.22**
**H(12A-12C)**	1.0159	1.0221	0.9916	1.0217	0.9772	1.0218	0.9913	1.0219	0.9921	1.0216	0.9943	1.0159	1.0221	0.9916	1.0217	0.9772	1.0218	0.9914	1.0219	0.9921	1.0216	0.9943	**0.77**
**H(1`A)**	2.5466	2.6087	2.6123	2.6247	2.6165	2.6242	2.6238	2.6237	2.6182	2.6251	2.6253	2.9068	2.9249	2.9160	2.9333	2.9175	2.9330	2.9201	2.9327	2.9165	2.9335	2.9377	**3.16**
**H(1`B)**	3.2222	3.2385	3.2758	3.2572	3.3242	3.2559	3.3194	3.2547	3.3141	3.2584	3.3295	2.7958	2.7872	2.7812	2.8016	2.8140	2.8006	2.8086	2.7996	2.7986	2.8025	2.8470	**3.76**
**H(2`A-2`B)**	1.9183	1.9553	1.9386	1.9826	1.9516	1.9807	1.9672	1.9790	1.9642	1.9842	1.9718	2.0326	2.0928	2.0638	2.1285	2.0848	2.1261	2.1061	2.1240	2.1011	2.1307	2.1313	**2.13**
**H(3`A-3`B)**	2.0326	2.0928	2.0638	2.1285	2.0848	2.1261	2.1061	2.1240	2.1011	2.1307	2.1313	1.9183	1.9553	1.9386	1.9826	1.9516	1.9807	1.9672	1.9790	1.9642	1.9843	1.9718	**2.05**
**H(4`A)**	2.7958	2.7872	2.7812	2.8016	2.8140	2.8006	2.8086	2.7996	2.7986	2.8025	2.8470	3.2222	3.2385	3.2758	3.2572	3.3242	3.2559	3.3194	3.2547	3.3141	3.2584	3.3295	**3.09**
**H(4`B)**	2.9068	2.9249	2.9160	2.9333	2.9174	2.9330	2.9201	2.9327	2.9165	2.9335	2.9377	2.5466	2.6087	2.6123	2.6247	2.6165	2.6242	2.6238	2.6237	2.6182	2.6251	2.6253	**3.63**
**F4**	-126.7028	-127.8970	-130.8935	-127.7985	-132.7002	-127.8157	-129.8887	-127.8226	-130.2655	-127.7806	-129.6275	-126.7030	-127.8970	-130.8935	-127.7985	-132.7001	-127.8157	-129.8888	-127.8226	-130.2654	-127.7806	-129.6275	**-104.5**

[a] methyl protons shifts are averaged
[b] according to [6]

Table S6. Cont. XIII

Nuclei	R											S											Exp. [b]
	wB97																						
	Gas	$CHCl_3$ (PCM)	$CHCl_3$ (SMD)	DMSO (PCM)	DMSO (SMD)	CH_3OH (PCM)	CH_3OH (SMD)	C_2H_5OH (PCM)	C_2H_5OH (SMD)	H_2O (PCM)	H_2O (SMD)	Gas	$CHCl_3$ (PCM)	$CHCl_3$ (SMD)	DMSO (PCM)	DMSO (SMD)	CH_3OH (PCM)	CH_3OH (SMD)	C_2H_5OH (PCM)	C_2H_5OH (SMD)	H_2O (PCM)	H_2O (SMD)	
**C1**	136.6036	137.2931	138.0720	137.6679	139.4784	137.6596	138.7685	137.6458	138.8630	137.6709	139.1689	136.6037	137.2932	138.0720	137.6679	139.4785	137.6598	138.7686	137.6458	138.8630	137.6709	139.1689	**132.8**
**C2**	140.3841	140.2277	140.7307	140.0851	140.5886	140.0918	141.0437	140.0984	140.9507	140.0798	141.0312	140.3840	140.2279	140.7307	140.0850	140.5889	140.0917	141.0438	140.0984	140.9506	140.0796	141.0312	**132.9**
**C3**	121.6019	122.1976	122.7769	122.3867	123.0830	122.3768	123.2214	122.3659	123.1771	122.3963	123.2651	121.6019	122.1976	122.7769	122.3867	123.0830	122.3768	123.2213	122.3659	123.1771	122.3961	123.2650	**117.2**
**C4**	174.2312	174.8327	175.4522	175.1881	175.7881	175.1626	176.3324	175.1372	176.1882	175.2135	176.4253	174.2312	174.8326	175.4522	175.1882	175.7881	175.1627	176.3322	175.1372	176.1882	175.2133	176.4254	**167.5**
**C5**	119.5886	120.8444	121.4620	121.5301	122.3881	121.4874	122.5264	121.4436	122.4326	121.5708	122.6707	119.5886	120.8442	121.4620	121.5301	122.3881	121.4873	122.5263	121.4436	122.4326	121.5708	122.6707	**117.2**
**C6**	136.6592	138.3616	139.5017	139.3874	140.9361	139.3158	141.4313	139.2458	141.1605	139.4573	141.7769	136.6593	138.3615	139.5017	139.3873	140.9360	139.3158	141.4313	139.2458	141.1605	139.4574	141.7769	**132.9**
**C7**	201.4930	206.2813	207.4612	209.0200	209.7424	208.8666	215.3336	208.7073	214.1389	209.1604	216.6162	201.4932	206.2813	207.4612	209.0200	209.7424	208.8665	215.3336	208.7073	214.1389	209.1603	216.6162	**195.8**
**C8**	61.8488	62.9799	63.5330	63.4907	64.4664	63.4706	65.0895	63.4470	64.9281	63.5072	65.6139	61.8488	62.9799	63.5330	63.4908	64.4665	63.4705	65.0895	63.4470	64.9281	63.5073	65.6139	**68.4**
**C9**	33.4710	33.2416	33.4528	33.1389	33.4830	33.1466	33.5747	33.1536	33.4993	33.1317	33.7715	33.4709	33.2417	33.4528	33.1390	33.4830	33.1467	33.5747	33.1536	33.4993	33.1318	33.7715	**30.5**
**C10**	27.2566	27.6512	28.0056	27.9994	28.6490	27.9736	28.4332	27.9477	28.3999	28.0248	28.6428	27.2566	27.6512	28.0056	27.9993	28.6490	27.9737	28.4333	27.9477	28.3999	28.0248	28.6428	**27.6**
**C11**	26.2445	26.3038	26.5962	26.2779	26.6993	26.2792	26.6545	26.2801	26.6480	26.2768	26.8119	26.2446	26.3039	26.5962	26.2778	26.6993	26.2793	26.6544	26.2801	26.6480	26.2768	26.8119	**23.1**
**C12**	16.0982	16.1941	16.2592	16.3226	16.4257	16.3108	16.3515	16.2989	16.3401	16.3345	16.3725	16.0982	16.1942	16.2592	16.3225	16.4258	16.3108	16.3514	16.2989	16.3401	16.3346	16.3725	**13.8**
**C1`**	49.2568	49.9126	50.1155	50.3454	50.8618	50.3138	50.7647	50.2823	50.7307	50.3765	51.1612	53.0280	53.8362	54.1787	54.2424	54.7458	54.2152	54.7838	54.1877	54.7368	54.2686	54.9485	**53.5**
**C2`**	23.8426	24.1307	24.3776	24.2804	24.7127	24.2708	24.6901	24.2604	24.6738	24.2897	24.5996	23.1244	23.2955	23.5764	23.4577	23.9434	23.4448	23.8672	23.4312	23.8527	23.4704	23.8171	**24.5**
**C3`**	23.1243	23.2956	23.5764	23.4576	23.9434	23.4448	23.8671	23.4312	23.8527	23.4704	23.8171	23.8426	24.1307	24.3776	24.2804	24.7128	24.2708	24.6899	24.2604	24.6738	24.2898	24.5997	**24.3**
**C4`**	53.0280	53.8363	54.1787	54.2423	54.7459	54.2152	54.7840	54.1877	54.7368	54.2686	54.9485	49.2568	49.9127	50.1155	50.3454	50.8617	50.3139	50.7647	50.2823	50.7307	50.3765	51.1611	**57.5**
**H2**	8.8611	8.7550	8.7824	8.6884	8.6845	8.6907	8.6892	8.6933	8.6908	8.6868	8.6627	8.8611	8.7551	8.7824	8.6884	8.6845	8.6907	8.6892	8.6933	8.6908	8.6868	8.6628	**8.11**
**H3**	7.4418	7.5755	7.6657	7.6151	7.7392	7.6130	7.7548	7.6108	7.7474	7.6170	7.7687	7.4418	7.5755	7.6657	7.6151	7.7392	7.6130	7.7548	7.6108	7.7474	7.6170	7.7687	**7.31**
**H5**	7.2881	7.4745	7.5659	7.5546	7.6853	7.5499	7.6986	7.5450	7.6874	7.5592	7.7204	7.2881	7.4745	7.5659	7.5546	7.6853	7.5499	7.6986	7.5450	7.6874	7.5592	7.7204	**7.31**
**H6**	8.1472	8.3095	8.4115	8.4085	8.5457	8.4013	8.5816	8.3942	8.5608	8.4158	8.6004	8.1472	8.3095	8.4115	8.4085	8.5457	8.4013	8.5816	8.3942	8.5608	8.4158	8.6004	**8.11**
**H8**	4.2162	4.2825	4.3391	4.3494	4.4217	4.3438	4.4547	4.3384	4.4370	4.3551	4.4428	4.2162	4.2825	4.3391	4.3494	4.4217	4.3438	4.4547	4.3384	4.4370	4.3551	4.4427	**5.18**
**H(9A-9B)**	1.6980	1.7347	1.7280	1.7573	1.7680	1.7556	1.7679	1.7541	1.7590	1.7591	1.8079	1.6980	1.7347	1.7280	1.7573	1.7680	1.7556	1.7679	1.7541	1.7590	1.7591	1.8079	**2.05**
**H(10A)**	1.0016	0.8194	0.8010	0.7626	0.6910	0.7642	0.7142	0.7660	0.7163	0.7614	0.7212	1.0016	0.8194	0.8010	0.7625	0.6910	0.7642	0.7142	0.7660	0.7163	0.7614	0.7213	**1.14**
**H(10B)**	1.6278	1.5347	1.4753	1.4482	1.3716	1.4533	1.3682	1.4583	1.3751	1.4435	1.3347	1.6278	1.5347	1.4753	1.4482	1.3716	1.4533	1.3682	1.4583	1.3751	1.4435	1.3347	**1.39**
**H(11A-11B)**	0.9970	1.0164	0.9858	1.0253	0.9913	1.0240	0.9842	1.0229	0.9848	1.0267	0.9701	0.9970	1.0165	0.9858	1.0253	0.9913	1.0240	0.9842	1.0229	0.9848	1.0267	0.9701	**1.22**
**H(12A-12C)**	0.9260	0.9059	0.8807	0.8925	0.8533	0.8928	0.8544	0.8932	0.8560	0.8923	0.8453	0.9260	0.9059	0.8807	0.8925	0.8533	0.8928	0.8544	0.8932	0.8560	0.8923	0.8453	**0.77**
**H(1`A)**	2.6270	2.6255	2.6087	2.6149	2.5688	2.6154	2.5814	2.6159	2.5810	2.6146	2.5637	2.7756	2.7821	2.7653	2.7661	2.7415	2.7679	2.7612	2.7694	2.7614	2.7644	2.7326	**3.16**
**H(1`B)**	3.0717	3.1071	3.1361	3.1419	3.1899	3.1392	3.1745	3.1364	3.1704	3.1448	3.1707	2.7440	2.6065	2.5843	2.5750	2.5724	2.5770	2.5537	2.5787	2.5505	2.5729	2.5550	**3.76**
**H(2`A-2`B)**	1.6864	1.7205	1.6869	1.7508	1.7082	1.7484	1.7178	1.7460	1.7130	1.7532	1.7346	1.5865	1.6591	1.6198	1.7006	1.6650	1.6980	1.6917	1.6953	1.6865	1.7030	1.7206	**2.13**
**H(3`A-3`B)**	1.5864	1.6591	1.6198	1.7006	1.6650	1.6980	1.6917	1.6953	1.6865	1.7030	1.7206	1.6864	1.7205	1.6869	1.7508	1.7082	1.7484	1.7178	1.7460	1.7130	1.7532	1.7346	**2.05**
**H(4`A)**	2.7440	2.6065	2.5843	2.5750	2.5724	2.5770	2.5537	2.5787	2.5505	2.5729	2.5550	3.0717	3.1071	3.1361	3.1419	3.1899	3.1392	3.1744	3.1364	3.1704	3.1448	3.1707	**3.09**
**H(4`B)**	2.7756	2.7821	2.7653	2.7661	2.7415	2.7679	2.7612	2.7694	2.7614	2.7644	2.7326	2.6270	2.6255	2.6087	2.6149	2.5688	2.6155	2.5814	2.6159	2.5810	2.6146	2.5637	**3.63**
**F4**	-108.0081	-109.6688	-111.9384	-110.0751	-113.6828	-110.0627	-111.9416	-110.0477	-112.1966	-110.0845	-111.9270	-108.0083	-109.6688	-111.9384	-110.0751	-113.6827	-110.0627	-111.9418	-110.0477	-112.1966	-110.0845	-111.9269	**-104.5**
Nuclei	wB97X																						
**C1**	138.1726	138.9019	139.6559	139.4374	141.1483	139.4362	140.2893	139.4298	140.5386	139.4351	140.5881	138.1726	138.9020	139.6559	139.4374	141.1483	139.4362	140.2892	139.4298	140.5386	139.4351	140.5881	**132.8**
**C2**	140.9056	140.7859	141.2893	140.6233	141.1540	140.6326	141.6628	140.6415	141.4810	140.6157	141.6112	140.9055	140.7861	141.2893	140.6233	141.1540	140.6326	141.6628	140.6415	141.4810	140.6157	141.6112	**132.9**
**C3**	122.3191	122.9195	123.4907	123.1123	123.8008	123.1045	123.9961	123.0956	123.8881	123.1203	124.0304	122.3191	122.9193	123.4908	123.1123	123.8008	123.1045	123.9961	123.0956	123.8881	123.1203	124.0304	**117.2**
**C4**	174.9105	175.5432	176.1569	175.8933	176.4990	175.8689	177.1226	175.8445	176.8649	175.9183	177.2107	174.9104	175.5433	176.1570	175.8933	176.4990	175.8689	177.1226	175.8446	176.8649	175.9183	177.2107	**167.5**
**C5**	120.2722	121.5580	122.1552	122.2785	123.1172	122.2378	123.3127	122.1963	123.1518	122.3181	123.4476	120.2722	121.5580	122.1551	122.2785	123.1172	122.2378	123.3127	122.1963	123.1518	122.3181	123.4476	**117.2**
**C6**	136.9982	138.7129	139.8801	139.7133	141.2649	139.6414	141.8678	139.5704	141.4715	139.7845	142.2578	136.9982	138.7132	139.8800	139.7133	141.2649	139.6414	141.8678	139.5703	141.4715	139.7845	142.2578	**132.9**
**C7**	203.1280	207.9657	209.1246	210.8225	211.4559	210.6803	217.6975	210.5330	215.8333	210.9532	218.0343	203.1280	207.9653	209.1247	210.8225	211.4559	210.6803	217.6976	210.5330	215.8333	210.9532	218.0343	**195.8**
**C8**	63.1594	64.2444	64.8483	64.8139	65.7904	64.8024	66.3526	64.7861	66.3173	64.8222	66.9049	63.1594	64.2444	64.8484	64.8139	65.7904	64.8024	66.3525	64.7861	66.3173	64.8222	66.9049	**68.4**
**C9**	34.2867	33.9981	34.2392	33.8733	34.2116	33.8838	34.2855	33.8926	34.2464	33.8639	34.5266	34.2868	33.9981	34.2391	33.8733	34.2116	33.8838	34.2855	33.8926	34.2464	33.8639	34.5266	**30.5**
**C10**	27.6239	28.0338	28.4350	28.3754	29.0463	28.3517	28.8760	28.3279	28.7824	28.3996	29.0595	27.6239	28.0339	28.4349	28.3754	29.0463	28.3517	28.8759	28.3278	28.7824	28.3996	29.0595	**27.6**
**C11**	26.7734	26.8395	27.1217	26.8101	27.2443	26.8150	27.2454	26.8190	27.1824	26.8061	27.3901	26.7733	26.8395	27.1217	26.8101	27.2443	26.8150	27.2453	26.8190	27.1824	26.8061	27.3901	**23.1**
**C12**	16.1134	16.2233	16.2803	16.3492	16.4455	16.3394	16.4133	16.3295	16.3307	16.3601	16.4150	16.1135	16.2233	16.2803	16.3492	16.4455	16.3394	16.4133	16.3295	16.3307	16.3601	16.4150	**13.8**
**C1`**	49.9786	50.6789	50.8740	51.1549	51.6976	51.1232	51.6534	51.0913	51.5542	51.1870	52.0498	54.0121	54.8870	55.2128	55.3169	55.7989	55.2921	55.9370	55.2667	55.7988	55.3418	56.0923	**53.5**
**C2`**	24.3147	24.5823	24.8496	24.7428	25.1842	24.7362	25.2016	24.7285	25.1548	24.7501	25.0887	23.6105	23.7548	24.0311	23.9209	24.4101	23.9100	24.3738	23.8985	24.2790	23.9323	24.2815	**24.5**
**C3`**	23.6105	23.7548	24.0310	23.9209	24.4101	23.9100	24.3738	23.8985	24.2790	23.9323	24.2815	24.3147	24.5823	24.8496	24.7428	25.1842	24.7362	25.2015	24.7285	25.1548	24.7501	25.0887	**24.3**
**C4`**	54.0122	54.8869	55.2127	55.3169	55.7989	55.2921	55.9370	55.2667	55.7988	55.3418	56.0923	49.9785	50.6788	50.8741	51.1549	51.6976	51.1232	51.6535	51.0913	51.5542	51.1870	52.0498	**57.5**
**H2**	8.8389	8.7344	8.7644	8.6467	8.6499	8.6486	8.6801	8.6509	8.6532	8.6456	8.6538	8.8389	8.7344	8.7644	8.6467	8.6499	8.6486	8.6801	8.6509	8.6532	8.6456	8.6538	**8.11**
**H3**	7.4189	7.5552	7.6448	7.5956	7.7180	7.5936	7.7363	7.5916	7.7258	7.5975	7.7486	7.4189	7.5552	7.6448	7.5956	7.7180	7.5936	7.7363	7.5916	7.7257	7.5975	7.7486	**7.31**
**H5**	7.2591	7.4494	7.5388	7.5328	7.6613	7.5281	7.6770	7.5234	7.6630	7.5374	7.6981	7.2591	7.4494	7.5388	7.5328	7.6613	7.5281	7.6770	7.5234	7.6630	7.5374	7.6981	**7.31**
**H6**	8.1258	8.2888	8.3898	8.3787	8.5117	8.3714	8.5602	8.3643	8.5198	8.3862	8.5805	8.1258	8.2888	8.3898	8.3787	8.5117	8.3714	8.5602	8.3643	8.5198	8.3862	8.5805	**8.11**
**H8**	4.3096	4.3744	4.4229	4.4341	4.4965	4.4284	4.5479	4.4231	4.5054	4.4401	4.5323	4.3096	4.3744	4.4229	4.4341	4.4965	4.4284	4.5479	4.4231	4.5054	4.4401	4.5323	**5.18**
**H(9A-9B)**	1.6862	1.7156	1.7186	1.7332	1.7474	1.7318	1.7435	1.7303	1.7407	1.7347	1.7933	1.6862	1.7156	1.7186	1.7332	1.7474	1.7318	1.7435	1.7303	1.7407	1.7347	1.7933	**2.05**
**H(10A)**	0.9741	0.8166	0.7900	0.7615	0.6871	0.7623	0.7271	0.7634	0.7083	0.7612	0.7224	0.9741	0.8166	0.7900	0.7615	0.6871	0.7623	0.7271	0.7634	0.7083	0.7612	0.7224	**1.14**
**H(10B)**	1.6245	1.5295	1.4648	1.4366	1.3566	1.4418	1.3555	1.4472	1.3556	1.4319	1.3216	1.6245	1.5295	1.4648	1.4366	1.3566	1.4418	1.3555	1.4472	1.3556	1.4319	1.3216	**1.39**
**H(11A-11B)**	0.9968	1.0179	0.9853	1.0245	0.9912	1.0235	0.9920	1.0226	0.9837	1.0257	0.9745	0.9968	1.0179	0.9853	1.0245	0.9912	1.0235	0.9920	1.0226	0.9837	1.0257	0.9745	**1.22**
**H(12A-12C)**	0.9175	0.9002	0.8726	0.8866	0.8472	0.8870	0.8543	0.8875	0.8473	0.8864	0.8426	0.9175	0.9002	0.8727	0.8866	0.8472	0.8870	0.8543	0.8875	0.8473	0.8864	0.8426	**0.77**
**H(1`A)**	2.6470	2.6454	2.6305	2.6365	2.5914	2.6370	2.6054	2.6376	2.6015	2.6363	2.5818	2.7957	2.7944	2.7786	2.7812	2.7529	2.7831	2.7665	2.7849	2.7724	2.7793	2.7420	**3.16**
**H(1`B)**	3.0808	3.1191	3.1539	3.1560	3.2078	3.1531	3.1986	3.1503	3.1886	3.1590	3.1950	2.7613	2.6199	2.6024	2.5918	2.5863	2.5940	2.5496	2.5960	2.5607	2.5899	2.5507	**3.76**
**H(2`A-2`B)**	1.6866	1.7220	1.6875	1.7527	1.7072	1.7504	1.7192	1.7481	1.7097	1.7552	1.7348	1.5871	1.6591	1.6179	1.7060	1.6665	1.7037	1.6956	1.7013	1.6869	1.7083	1.7189	**2.13**
**H(3`A-3`B)**	1.5872	1.6591	1.6179	1.7060	1.6665	1.7037	1.6956	1.7013	1.6869	1.7083	1.7189	1.6866	1.7220	1.6875	1.7527	1.7072	1.7504	1.7192	1.7481	1.7097	1.7552	1.7348	**2.05**
**H(4`A)**	2.7613	2.6199	2.6025	2.5918	2.5863	2.5940	2.5496	2.5960	2.5607	2.5899	2.5507	3.0809	3.1191	3.1539	3.1560	3.2078	3.1531	3.1986	3.1503	3.1886	3.1590	3.1950	**3.09**
**H(4`B)**	2.7957	2.7944	2.7786	2.7812	2.7529	2.7831	2.7665	2.7849	2.7724	2.7793	2.7420	2.6470	2.6454	2.6305	2.6365	2.5914	2.6370	2.6054	2.6376	2.6015	2.6363	2.5818	**3.63**
**F4**	-110.3425	-111.8026	-114.1109	-112.1812	-115.8382	-112.1764	-113.9559	-112.1690	-114.3523	-112.1830	-113.9045	-110.3428	-111.8023	-114.1108	-112.1812	-115.8382	-112.1764	-113.9558	-112.1689	-114.3524	-112.1830	-113.9045	**-104.5**

[a] methyl protons shifts are averaged

[b] according to [6]

Table S6. Cont. XIV

Nuclei	R											S											Exp. [b]
	X3LYP																						
	Gas	$CHCl_3$ (PCM)	$CHCl_3$ (SMD)	DMSO (PCM)	DMSO (SMD)	CH_3OH (PCM)	CH_3OH (SMD)	C_2H_5OH (PCM)	C_2H_5OH (SMD)	H_2O (PCM)	H_2O (SMD)	Gas	$CHCl_3$ (PCM)	$CHCl_3$ (SMD)	DMSO (PCM)	DMSO (SMD)	CH_3OH (PCM)	CH_3OH (SMD)	C_2H_5OH (PCM)	C_2H_5OH (SMD)	H_2O (PCM)	H_2O (SMD)	
**C1**	139.6738	140.7029	141.2879	141.2181	142.6377	141.1931	142.0187	141.1652	142.0627	141.2455	142.1890	139.6738	140.7029	141.2877	141.2181	142.6377	141.1931	142.0187	141.1652	142.0627	141.2455	142.1891	**132.8**
**C2**	139.7415	139.6983	140.2072	139.6129	140.0578	139.6218	140.6071	139.6280	140.4859	139.6083	140.5357	139.7415	139.6983	140.2071	139.6129	140.0578	139.6218	140.6071	139.6280	140.4859	139.6083	140.5359	**132.9**
**C3**	122.0899	122.7613	123.3039	122.9822	123.5845	122.9753	123.8523	122.9648	123.7861	122.9920	123.9125	122.0899	122.7613	123.3039	122.9822	123.5845	122.9753	123.8523	122.9648	123.7861	122.9920	123.9125	**117.2**
**C4**	176.3624	177.2172	177.8363	177.6336	178.2690	177.6121	178.8733	177.5874	178.7243	177.6576	178.9971	176.3624	177.2172	177.8362	177.6336	178.2690	177.6121	178.8733	177.5874	178.7243	177.6576	178.9971	**167.5**
**C5**	119.9750	121.1943	121.8036	121.8371	122.6276	121.8009	122.8281	121.7616	122.7328	121.8752	122.9752	119.9750	121.1943	121.8037	121.8372	122.6276	121.8009	122.8281	121.7616	122.7328	121.8752	122.9752	**117.2**
**C6**	135.9479	137.8862	139.0367	139.0291	140.5596	138.9585	141.2161	138.8853	140.9649	139.0998	141.6039	135.9479	137.8862	139.0366	139.0291	140.5596	138.9585	141.2161	138.8853	140.9649	139.0998	141.6039	**132.9**
**C7**	203.8442	208.6848	209.7423	211.2993	211.9258	211.1467	216.9743	210.9904	215.8222	211.4475	217.9867	203.8442	208.6848	209.7423	211.2993	211.9258	211.1467	216.9743	210.9904	215.8222	211.4475	217.9866	**195.8**
**C8**	66.1900	67.3996	67.9886	67.9123	68.9737	67.8933	69.5487	67.8696	69.3941	67.9319	69.8838	66.1900	67.3996	67.9886	67.9123	68.9737	67.8933	69.5487	67.8696	69.3941	67.9319	69.8838	**68.4**
**C9**	37.2125	36.8829	37.1023	36.7383	37.0705	36.7499	37.2814	36.7581	37.2176	36.7305	37.3484	37.2125	36.8829	37.1024	36.7383	37.0705	36.7499	37.2813	36.7581	37.2176	36.7305	37.3483	**30.5**
**C10**	29.2336	29.7336	30.2430	30.0625	30.8205	30.0426	30.6156	30.0200	30.6061	30.0851	30.7479	29.2336	29.7336	30.2429	30.0624	30.8205	30.0426	30.6156	30.0200	30.6061	30.0851	30.7479	**27.6**
**C11**	29.2440	29.3726	29.6606	29.3435	29.7886	29.3527	29.7810	29.3582	29.7823	29.3378	29.9009	29.2440	29.3726	29.6607	29.3436	29.7886	29.3527	29.7810	29.3582	29.7823	29.3378	29.9009	**23.1**
**C12**	16.6790	16.8526	16.9369	16.9924	17.0898	16.9846	17.0466	16.9744	17.0456	17.0037	17.0335	16.6790	16.8526	16.9370	16.9924	17.0898	16.9846	17.0465	16.9744	17.0456	17.0037	17.0336	**13.8**
**C1`**	52.1500	52.9655	53.2787	53.5431	54.0664	53.5092	54.0666	53.4728	53.9991	53.5797	54.5782	56.6692	57.8046	58.1645	58.2841	58.8069	58.2617	58.9279	58.2356	58.8673	58.3087	59.1667	**53.5**
**C2`**	25.9688	26.4573	26.8289	26.6445	27.1554	26.6386	27.1840	26.6293	27.1770	26.6532	26.9598	25.5752	25.7645	26.1378	25.9438	26.4160	25.9341	26.3685	25.9216	26.3639	25.9571	26.1690	**24.5**
**C3`**	25.5752	25.7645	26.1378	25.9439	26.4160	25.9341	26.3685	25.9216	26.3639	25.9571	26.1690	25.9688	26.4573	26.8288	26.6445	27.1554	26.6386	27.1839	26.6293	27.1770	26.6532	26.9597	**24.3**
**C4`**	56.6692	57.8046	58.1645	58.2842	58.8069	58.2616	58.9278	58.2356	58.8673	58.3087	59.1668	52.1500	52.9655	53.2788	53.5431	54.0664	53.5092	54.0666	53.4728	53.9991	53.5797	54.5781	**57.5**
**H2**	8.7017	8.5820	8.6334	8.5144	8.5224	8.5185	8.5444	8.5222	8.5458	8.5104	8.5311	8.7017	8.5820	8.6334	8.5144	8.5224	8.5185	8.5444	8.5222	8.5458	8.5104	8.5311	**8.11**
**H3**	7.3180	7.4626	7.5498	7.5093	7.6221	7.5074	7.6437	7.5048	7.6355	7.5111	7.6574	7.3180	7.4626	7.5498	7.5093	7.6221	7.5074	7.6437	7.5048	7.6355	7.5111	7.6574	**7.31**
**H5**	7.1388	7.2932	7.3946	7.3636	7.4967	7.3596	7.5018	7.3552	7.4932	7.3672	7.5277	7.1388	7.2932	7.3946	7.3636	7.4967	7.3596	7.5018	7.3552	7.4932	7.3672	7.5277	**7.31**
**H6**	7.9778	8.1585	8.2522	8.2595	8.3785	8.2534	8.4310	8.2468	8.4121	8.2652	8.4587	7.9778	8.1585	8.2522	8.2595	8.3785	8.2534	8.4310	8.2468	8.4121	8.2652	8.4587	**8.11**
**H8**	4.6221	4.6810	4.7192	4.7261	4.7782	4.7226	4.8173	4.7187	4.8029	4.7294	4.7963	4.6221	4.6810	4.7192	4.7261	4.7782	4.7226	4.8173	4.7187	4.8029	4.7294	4.7963	**5.18**
**H(9A-9B)**	1.7073	1.7466	1.7566	1.7758	1.8078	1.7740	1.8065	1.7718	1.8013	1.7774	1.8410	1.7073	1.7466	1.7566	1.7758	1.8078	1.7740	1.8065	1.7718	1.8013	1.7774	1.8410	**2.05**
**H(10A)**	1.0017	0.8584	0.8480	0.8249	0.7364	0.8258	0.7864	0.8263	0.7798	0.8243	0.7913	1.0017	0.8584	0.8480	0.8249	0.7364	0.8258	0.7864	0.8263	0.7798	0.8243	0.7913	**1.14**
**H(10B)**	1.6621	1.5559	1.4962	1.4782	1.3946	1.4836	1.3899	1.4885	1.3968	1.4731	1.3608	1.6621	1.5559	1.4962	1.4782	1.3946	1.4836	1.3899	1.4885	1.3968	1.4731	1.3608	**1.39**
**H(11A-11B)**	1.0602	1.0755	1.0472	1.0937	1.0553	1.0929	1.0546	1.0915	1.0540	1.0945	1.0426	1.0602	1.0755	1.0473	1.0937	1.0553	1.0929	1.0547	1.0915	1.0540	1.0945	1.0426	**1.22**
**H(12A-12C)**	0.9403	0.9279	0.9065	0.9238	0.8835	0.9241	0.8907	0.9241	0.8907	0.9234	0.8830	0.9403	0.9279	0.9065	0.9238	0.8835	0.9241	0.8906	0.9241	0.8907	0.9234	0.8830	**0.77**
**H(1`A)**	2.7437	2.7364	2.7259	2.7268	2.6858	2.7276	2.7033	2.7279	2.7047	2.7261	2.6669	2.8709	2.8795	2.8646	2.8584	2.8308	2.8605	2.8410	2.8620	2.8446	2.8562	2.8024	**3.16**
**H(1`B)**	3.2035	3.2527	3.2856	3.2909	3.3575	3.2884	3.3446	3.2855	3.3413	3.2932	3.3404	2.8974	2.7352	2.7069	2.7090	2.7058	2.7093	2.6710	2.7092	2.6722	2.7090	2.6597	**3.76**
**H(2`A-2`B)**	1.7389	1.7694	1.7353	1.8030	1.7519	1.8007	1.7656	1.7980	1.7608	1.8052	1.7835	1.6075	1.6874	1.6512	1.7365	1.6859	1.7336	1.7154	1.7303	1.7082	1.7392	1.7463	**2.13**
**H(3`A-3`B)**	1.6075	1.6874	1.6512	1.7364	1.6859	1.7336	1.7154	1.7303	1.7082	1.7392	1.7463	1.7389	1.7694	1.7352	1.8030	1.7519	1.8007	1.7656	1.7980	1.7608	1.8052	1.7835	**2.05**
**H(4`A)**	2.8974	2.7352	2.7069	2.7090	2.7058	2.7093	2.6710	2.7092	2.6722	2.7090	2.6597	3.2035	3.2527	3.2855	3.2909	3.3575	3.2884	3.3446	3.2855	3.3413	3.2932	3.3404	**3.09**
**H(4`B)**	2.8709	2.8795	2.8646	2.8584	2.8308	2.8605	2.8410	2.8620	2.8446	2.8562	2.8024	2.7437	2.7364	2.7259	2.7268	2.6858	2.7276	2.7033	2.7279	2.7047	2.7261	2.6669	**3.63**
**F4**	-124.1700	-125.6079	-128.3558	-125.8662	-130.1796	-125.8578	-127.9823	-125.8489	-128.3359	-125.8737	-127.7278	-124.1700	-125.6079	-128.3558	-125.8663	-130.1796	-125.8578	-127.9823	-125.8489	-128.3359	-125.8737	-127.7277	**-104.5**

[a] methyl protons shifts are averaged

[b] according to [6]

Table S7. Theoretical chemical shifts (in ppm) of *(R,S)*-4-fluoro-α-pyrrolidinononanophenone calculated with HF and selected functionals combined with 6-311++G** basis set[a]

Nuclei	R											S											Exp. [b]
	B3LYP																						
	Gas	$CHCl_3$ (PCM)	$CHCl_3$ (SMD)	DMSO (PCM)	DMSO (SMD)	CH_3OH (PCM)	CH_3OH (SMD)	C_2H_5OH (PCM)	C_2H_5OH (SMD)	H_2O (PCM)	H_2O (SMD)	Gas	$CHCl_3$ (PCM)	$CHCl_3$ (SMD)	DMSO (PCM)	DMSO (SMD)	CH_3OH (PCM)	CH_3OH (SMD)	C_2H_5OH (PCM)	C_2H_5OH (SMD)	H_2O (PCM)	H_2O (SMD)	
**H2**	8.6693	8.5547	8.5907	8.4593	8.4987	8.4663	8.4890	8.4733	8.4013	8.4526	8.5172	8.6693	8.5547	8.5907	8.4593	8.4987	8.4663	8.4074	8.4733	8.4013	8.4526	8.5172	**8.07**
**H3**	7.3116	7.4512	7.5342	7.4886	7.6059	7.4867	7.5964	7.4848	7.5971	7.4902	7.6449	7.3116	7.4512	7.5342	7.4886	7.6059	7.4867	7.6071	7.4848	7.5971	7.4902	7.6449	**7.24**
**H5**	7.1309	7.3065	7.3948	7.3863	7.5022	7.3812	7.5008	7.3761	7.5337	7.3907	7.5420	7.1309	7.3065	7.3948	7.3863	7.5022	7.3812	7.5468	7.3761	7.5337	7.3907	7.5420	**7.24**
**H6**	7.9655	8.1627	8.2357	8.2433	8.3768	8.2391	8.3986	8.2347	8.3278	8.2472	8.4483	7.9655	8.1627	8.2357	8.2433	8.3768	8.2391	8.3515	8.2347	8.3278	8.2472	8.4483	**8.07**
**H8**	4.6433	4.7289	4.7500	4.7575	4.8095	4.7566	4.9005	4.7554	4.8158	4.7579	4.8360	4.6433	4.7289	4.7500	4.7575	4.8095	4.7566	4.8330	4.7554	4.8157	4.7579	4.8360	**5.24**
**H(9A-9B)**	1.7222	1.7545	1.7554	1.7680	1.8251	1.7663	1.7917	1.7651	1.6955	1.7701	1.8393	1.7222	1.7545	1.7554	1.7680	1.8251	1.7663	1.7066	1.7651	1.6955	1.7701	1.8393	**2.135**
**H(10A-10B)**	1.3431	1.2354	1.1938	1.1670	1.0950	1.1714	1.1011	1.1757	1.1250	1.1626	1.1135	1.3432	1.2354	1.1938	1.1670	1.0950	1.1714	1.1248	1.1757	1.1250	1.1626	1.1135	**1.275**
**H(11A-11B)**	0.9890	1.0109	0.9726	1.0284	0.9917	1.0266	0.9794	1.0250	0.9728	1.0300	0.9829	0.9890	1.0109	0.9726	1.0284	0.9917	1.0266	0.9762	1.0250	0.9728	1.0300	0.9829	**1.28**
**H(12A-12B)**	1.2375	1.2278	1.1976	1.2122	1.1670	1.2130	1.1658	1.2138	1.1829	1.2111	1.1744	1.2375	1.2278	1.1976	1.2122	1.1670	1.2130	1.1840	1.2138	1.1829	1.2111	1.1744	**1.285**
**H(13A-13B)**	1.1508	1.1568	1.1229	1.1633	1.1273	1.1624	1.1149	1.1615	1.1200	1.1641	1.1228	1.1508	1.1568	1.1229	1.1634	1.1273	1.1624	1.1226	1.1615	1.1200	1.1641	1.1228	**1.29**
**H(14A-14B)**	1.3097	1.3103	1.2856	1.3059	1.2726	1.3059	1.2612	1.3059	1.2795	1.3057	1.2750	1.3097	1.3103	1.2856	1.3059	1.2726	1.3059	1.2813	1.3059	1.2795	1.3057	1.2750	**1.295**
**H(15A-15C)**	0.9551	0.9535	0.9276	0.9506	0.9207	0.9505	0.8987	0.9503	0.9200	0.9505	0.9190	0.9551	0.9535	0.9276	0.9506	0.9207	0.9505	0.9219	0.9503	0.9200	0.9505	0.9190	**0.82**
**H(1`A)**	2.7487	2.7479	2.7208	2.7338	2.6854	2.7347	2.7043	2.7354	2.6956	2.7328	2.6736	2.8743	2.8645	2.8562	2.8422	2.8141	2.8430	2.8174	2.8438	2.8192	2.8414	2.7793	**3.82**
**H(1`B)**	3.2194	3.2608	3.2819	3.2881	3.3399	3.2861	3.3328	3.2841	3.3184	3.2898	3.3406	2.8699	2.7074	2.6744	2.6831	2.6934	2.6832	2.6813	2.6834	2.6802	2.6831	2.6443	**3.78**
**H(2`A-2`B)**	1.7379	1.7827	1.7394	1.8102	1.7582	1.8082	1.7578	1.8062	1.7637	1.8118	1.7850	1.5835	1.6758	1.6369	1.7260	1.6678	1.7224	1.7153	1.7187	1.7075	1.7292	1.7255	**2.26**
**H(3`A-3`B)**	1.5835	1.6758	1.6369	1.7260	1.6678	1.7224	1.6799	1.7187	1.7075	1.7292	1.7255	1.7380	1.7827	1.7394	1.8102	1.7582	1.8082	1.7705	1.8062	1.7637	1.8118	1.7850	**2.01**
**H(4`A)**	2.8699	2.7074	2.6744	2.6831	2.6934	2.6832	2.6795	2.6834	2.6802	2.6831	2.6443	3.2194	3.2608	3.2819	3.2881	3.3399	3.2861	3.3253	3.2841	3.3184	3.2898	3.3406	**3.68**
**H(4`B)**	2.8743	2.8645	2.8562	2.8422	2.8141	2.8430	2.8187	2.8438	2.8192	2.8414	2.7793	2.7486	2.7479	2.7208	2.7338	2.6854	2.7347	2.6976	2.7354	2.6956	2.7328	2.6736	**2.88**
Nuclei	B3P86																						
**H2**	8.7403	8.6754	8.6602	8.5732	8.5747	8.5834	8.5932	8.5916	8.6190	8.5641	8.5845	8.7403	8.6754	8.6602	8.5732	8.5747	8.5834	8.6167	8.5916	8.6190	8.5641	8.5845	**8.07**
**H3**	7.3317	7.4774	7.5723	7.5245	7.6458	7.5238	7.6354	7.5211	7.6610	7.5262	7.6837	7.3317	7.4774	7.5723	7.5245	7.6458	7.5238	7.6654	7.5211	7.6610	7.5262	7.6837	**7.24**
**H5**	7.1472	7.3358	7.4228	7.4044	7.5340	7.4017	7.5241	7.3971	7.5461	7.4081	7.5714	7.1472	7.3358	7.4228	7.4044	7.5340	7.4017	7.5542	7.3971	7.5461	7.4081	7.5714	**7.24**
**H6**	8.0225	8.2297	8.2939	8.3160	8.4305	8.3123	8.4715	8.3064	8.4849	8.3201	8.5353	8.0225	8.2297	8.2939	8.3160	8.4304	8.3123	8.5049	8.3064	8.4849	8.3201	8.5353	**8.07**
**H8**	4.6484	4.7296	4.7658	4.7652	4.8174	4.7659	4.9040	4.7639	4.8681	4.7649	4.8720	4.6484	4.7296	4.7658	4.7652	4.8174	4.7659	4.8794	4.7639	4.8681	4.7649	4.8720	**5.24**
**H(9A-9B)**	1.7933	1.8417	1.8102	1.8531	1.8752	1.8523	1.8728	1.8504	1.8610	1.8559	1.8873	1.7933	1.8416	1.8102	1.8531	1.8752	1.8523	1.8659	1.8504	1.8610	1.8559	1.8873	**2.135**
**H(10A-10B)**	1.3555	1.2402	1.2054	1.1692	1.1077	1.1752	1.1032	1.1792	1.1267	1.1648	1.1100	1.3555	1.2402	1.2054	1.1691	1.1077	1.1752	1.1197	1.1792	1.1267	1.1648	1.1100	**1.275**
**H(11A-11B)**	0.9713	0.9976	0.9548	1.0141	0.9765	1.0138	0.9661	1.0118	0.9711	1.0157	0.9646	0.9713	0.9976	0.9548	1.0141	0.9765	1.0138	0.9683	1.0118	0.9711	1.0157	0.9646	**1.28**
**H(12A-12B)**	1.2227	1.2083	1.1845	1.1914	1.1539	1.1939	1.1453	1.1944	1.1635	1.1903	1.1572	1.2227	1.2083	1.1845	1.1914	1.1539	1.1939	1.1583	1.1944	1.1634	1.1903	1.1572	**1.285**
**H(13A-13B)**	1.1281	1.1319	1.1013	1.1382	1.1048	1.1388	1.0891	1.1376	1.1018	1.1391	1.0987	1.1281	1.1319	1.1013	1.1383	1.1048	1.1388	1.0979	1.1376	1.1018	1.1391	1.0987	**1.29**
**H(14A-14B)**	1.2803	1.2766	1.2573	1.2717	1.2431	1.2733	1.2266	1.2729	1.2484	1.2716	1.2438	1.2803	1.2766	1.2573	1.2717	1.2431	1.2733	1.2440	1.2729	1.2485	1.2716	1.2438	**1.295**
**H(15A-15C)**	0.9213	0.9193	0.8972	0.9160	0.8890	0.9174	0.8642	0.9170	0.8914	0.9159	0.8861	0.9213	0.9193	0.8972	0.9160	0.8890	0.9174	0.8868	0.9170	0.8914	0.9159	0.8861	**0.82**
**H(1`A)**	2.7048	2.7089	2.6899	2.6926	2.6501	2.6953	2.6647	2.6961	2.6668	2.6911	2.6359	2.8302	2.8173	2.8105	2.8030	2.7716	2.8046	2.7750	2.8046	2.7819	2.8030	2.7462	**3.82**
**H(1`B)**	3.2227	3.2611	3.2768	3.2837	3.3293	3.2835	3.3203	3.2815	3.3230	3.2850	3.3277	2.9188	2.7123	2.7132	2.6794	2.7077	2.6795	2.6380	2.6787	2.6483	2.6817	2.6399	**3.78**
**H(2`A-2`B)**	1.7225	1.7609	1.7300	1.7869	1.7457	1.7864	1.7378	1.7839	1.7560	1.7888	1.7760	1.5695	1.6493	1.6195	1.6967	1.6507	1.6949	1.6753	1.6911	1.6739	1.6995	1.7185	**2.26**
**H(3`A-3`B)**	1.5695	1.6493	1.6195	1.6967	1.6507	1.6949	1.6528	1.6912	1.6739	1.6995	1.7185	1.7225	1.7609	1.7300	1.7869	1.7457	1.7864	1.7566	1.7840	1.7560	1.7888	1.7760	**2.01**
**H(4`A)**	2.9188	2.7123	2.7132	2.6794	2.7077	2.6795	2.6746	2.6787	2.6483	2.6817	2.6399	3.2227	3.2610	3.2768	3.2837	3.3293	3.2835	3.3234	3.2815	3.3230	3.2850	3.3277	**3.68**
**H(4`B)**	2.8302	2.8172	2.8105	2.8030	2.7716	2.8046	2.7840	2.8045	2.7819	2.8030	2.7462	2.7048	2.7089	2.6899	2.6926	2.6500	2.6953	2.6621	2.6961	2.6668	2.6911	2.6359	**2.88**
Nuclei	B3PW91																						
**H2**	8.7093	8.5947	8.6296	8.5029	8.5475	8.5105	8.5319	8.5174	8.5774	8.4956	8.5148	8.7093	8.5947	8.6296	8.5029	8.5320	8.5105	8.5584	8.5174	8.5774	8.4956	8.5148	**8.07**
**H3**	7.3315	7.4773	7.5665	7.5151	7.6534	7.5140	7.6281	7.5125	7.6516	7.5163	7.6727	7.3315	7.4773	7.5665	7.5151	7.6379	7.5140	7.6590	7.5125	7.6516	7.5163	7.6727	**7.24**
**H5**	7.1504	7.3277	7.4240	7.4068	7.5461	7.4027	7.5275	7.3983	7.5420	7.4106	7.5642	7.1504	7.3277	7.4240	7.4068	7.5306	7.4027	7.5506	7.3983	7.5420	7.4106	7.5642	**7.24**
**H6**	8.0045	8.2018	8.2745	8.3017	8.4629	8.2970	8.4558	8.2917	8.4654	8.3057	8.5091	8.0045	8.2018	8.2745	8.3017	8.4474	8.2970	8.4871	8.2917	8.4653	8.3058	8.5091	**8.07**
**H8**	4.6390	4.7201	4.7465	4.7519	4.8277	4.7516	4.8910	4.7506	4.8440	4.7519	4.8363	4.6390	4.7201	4.7465	4.7519	4.8122	4.7516	4.8522	4.7506	4.8440	4.7519	4.8363	**5.24**
**H(9A-9B)**	1.7418	1.7634	1.7672	1.7873	1.8487	1.7851	1.8084	1.7830	1.8158	1.7900	1.8213	1.7418	1.7633	1.7672	1.7873	1.8332	1.7851	1.8183	1.7830	1.8158	1.7900	1.8213	**2.135**
**H(10A-10B)**	1.3208	1.2036	1.1695	1.1336	1.0802	1.1386	1.0704	1.1433	1.0892	1.1290	1.0672	1.3208	1.2036	1.1695	1.1336	1.0647	1.1386	1.0819	1.1433	1.0892	1.1290	1.0672	**1.275**
**H(11A-11B)**	0.9479	0.9654	0.9293	0.9852	0.9675	0.9840	0.9384	0.9827	0.9432	0.9866	0.9301	0.9479	0.9654	0.9293	0.9852	0.9520	0.9841	0.9404	0.9827	0.9432	0.9867	0.9301	**1.28**
**H(12A-12B)**	1.1983	1.1829	1.1570	1.1665	1.1403	1.1681	1.1216	1.1695	1.1340	1.1651	1.1269	1.1983	1.1829	1.1570	1.1665	1.1248	1.1681	1.1311	1.1695	1.1340	1.1651	1.1269	**1.285**
**H(13A-13B)**	1.1073	1.1086	1.0783	1.1155	1.0979	1.1154	1.0676	1.1149	1.0768	1.1160	1.0721	1.1073	1.1086	1.0783	1.1155	1.0824	1.1154	1.0752	1.1149	1.0768	1.1160	1.0721	**1.29**
**H(14A-14B)**	1.2651	1.2621	1.2399	1.2576	1.2412	1.2584	1.2136	1.2588	1.2297	1.2570	1.2259	1.2651	1.2621	1.2399	1.2576	1.2257	1.2584	1.2288	1.2588	1.2297	1.2570	1.2259	**1.295**
**H(15A-15C)**	0.9104	0.9067	0.8840	0.9044	0.8911	0.9050	0.8538	0.9053	0.8766	0.9040	0.8719	0.9104	0.9067	0.8839	0.9044	0.8756	0.9050	0.8753	0.9053	0.8766	0.9040	0.8719	**0.82**
**H(1`A)**	2.6998	2.6997	2.6782	2.6831	2.6510	2.6848	2.6566	2.6863	2.6540	2.6815	2.6212	2.8232	2.8093	2.8031	2.7940	2.7612	2.7951	2.7703	2.7959	2.7696	2.7934	2.7387	**3.82**
**H(1`B)**	3.1960	3.2324	3.2531	3.2600	3.3199	3.2586	3.3003	3.2569	3.2992	3.2615	3.3021	2.8718	2.6962	2.6803	2.6637	2.6791	2.6640	2.6301	2.6646	2.6271	2.6643	2.6335	**3.78**
**H(2`A-2`B)**	1.7098	1.7508	1.7149	1.7758	1.7511	1.7746	1.7272	1.7731	1.7400	1.7772	1.7605	1.5602	1.6462	1.6068	1.6919	1.6423	1.6895	1.6688	1.6869	1.6602	1.6943	1.7139	**2.26**
**H(3`A-3`B)**	1.5602	1.6462	1.6068	1.6919	1.6578	1.6895	1.6488	1.6869	1.6602	1.6943	1.7139	1.7098	1.7508	1.7149	1.7758	1.7356	1.7746	1.7441	1.7731	1.7400	1.7772	1.7605	**2.01**
**H(4`A)**	2.8718	2.6962	2.6803	2.6637	2.6946	2.6640	2.6598	2.6646	2.6270	2.6643	2.6335	3.1960	3.2324	3.2531	3.2600	3.3044	3.2586	3.3037	3.2569	3.2992	3.2615	3.3021	**3.68**
**H(4`B)**	2.8232	2.8093	2.8031	2.7941	2.7767	2.7951	2.7747	2.7959	2.7696	2.7933	2.7386	2.6998	2.6997	2.6781	2.6831	2.6355	2.6848	2.6530	2.6863	2.6540	2.6815	2.6212	**2.88**
Nuclei	B98																						
**H2**	8.6854	8.5945	8.6132	8.5090	8.5292	8.5158	8.5704	8.5223	8.4385	8.5022	8.5395	8.6854	8.5945	8.6132	8.5090	8.5292	8.5158	8.4417	8.5223	8.4385	8.5022	8.5395	**8.07**
**H3**	7.3204	7.4616	7.5520	7.5049	7.6257	7.5030	7.6469	7.5011	7.6254	7.5068	7.6634	7.3204	7.4616	7.5520	7.5049	7.6257	7.5030	7.6334	7.5012	7.6254	7.5068	7.6634	**7.24**
**H5**	7.1529	7.3313	7.4256	7.4141	7.5381	7.4094	7.5577	7.4048	7.5623	7.4187	7.5722	7.1529	7.3313	7.4256	7.4141	7.5380	7.4094	7.5729	7.4048	7.5623	7.4187	7.5722	**7.24**
**H6**	8.0107	8.2017	8.2813	8.2935	8.4286	8.2885	8.4883	8.2834	8.3708	8.2982	8.5149	8.0107	8.2017	8.2813	8.2935	8.4286	8.2885	8.3904	8.2834	8.3708	8.2982	8.5149	**8.07**
**H8**	4.6099	4.6916	4.7237	4.7319	4.7887	4.7305	4.8405	4.7288	4.7931	4.7332	4.8346	4.6099	4.6916	4.7237	4.7319	4.7887	4.7305	4.8066	4.7288	4.7931	4.7332	4.8346	**5.24**
**H(9A-9B)**	1.7236	1.7570	1.7496	1.7733	1.8072	1.7717	1.8083	1.7702	1.6894	1.7754	1.8246	1.7235	1.7570	1.7496	1.7733	1.8072	1.7717	1.6990	1.7702	1.6894	1.7754	1.8246	**2.135**
**H(10A-10B)**	1.3506	1.2333	1.1950	1.1590	1.0899	1.1643	1.1084	1.1696	1.1203	1.1541	1.0977	1.3506	1.2333	1.1950	1.1590	1.0899	1.1643	1.1168	1.1696	1.1203	1.1541	1.0977	**1.275**
**H(11A-11B)**	0.9772	0.9946	0.9578	1.0135	0.9777	1.0120	0.9737	1.0106	0.9615	1.0154	0.9684	0.9772	0.9946	0.9578	1.0135	0.9777	1.0120	0.9620	1.0106	0.9615	1.0154	0.9684	**1.28**
**H(12A-12B)**	1.2204	1.2038	1.1781	1.1869	1.1455	1.1883	1.1542	1.1897	1.1659	1.1859	1.1526	1.2204	1.2038	1.1781	1.1869	1.1455	1.1883	1.1643	1.1897	1.1659	1.1859	1.1526	**1.285**
**H(13A-13B)**	1.1314	1.1330	1.1028	1.1401	1.1049	1.1396	1.1025	1.1392	1.1022	1.1409	1.1015	1.1314	1.1330	1.1028	1.1401	1.1049	1.1396	1.1020	1.1392	1.1022	1.1409	1.1015	**1.29**
**H(14A-14B)**	1.2851	1.2810	1.2600	1.2762	1.2448	1.2767	1.2490	1.2773	1.2562	1.2761	1.2466	1.2851	1.2810	1.2600	1.2762	1.2448	1.2767	1.2552	1.2773	1.2562	1.2761	1.2466	**1.295**
**H(15A-15C)**	0.9303	0.9255	0.9035	0.9235	0.8946	0.9237	0.8955	0.9240	0.8985	0.9235	0.8925	0.9303	0.9255	0.9035	0.9235	0.8946	0.9237	0.8977	0.9240	0.8985	0.9235	0.8925	**0.82**
**H(1`A)**	2.7132	2.7100	2.6906	2.6975	2.6553	2.6984	2.6699	2.6994	2.6714	2.6969	2.6463	2.8410	2.8275	2.8256	2.8114	2.7814	2.8123	2.7888	2.8133	2.7915	2.8110	2.7560	**3.82**
**H(1`B)**	3.2060	3.2428	3.2681	3.2664	3.3137	3.2654	3.3135	3.2644	3.3034	3.2675	3.3177	2.8666	2.6969	2.6851	2.6720	2.6908	2.6721	2.6802	2.6728	2.6821	2.6728	2.6373	**3.78**
**H(2`A-2`B)**	1.7256	1.7702	1.7345	1.8002	1.7523	1.7985	1.7678	1.7969	1.7606	1.8020	1.7847	1.5864	1.6710	1.6361	1.7198	1.6689	1.7167	1.7138	1.7138	1.7091	1.7230	1.7300	**2.26**
**H(3`A-3`B)**	1.5864	1.6710	1.6361	1.7198	1.6689	1.7167	1.6943	1.7138	1.7091	1.7230	1.7300	1.7256	1.7702	1.7345	1.8002	1.7523	1.7985	1.7646	1.7969	1.7606	1.8020	1.7847	**2.01**
**H(4`A)**	2.8666	2.6969	2.6851	2.6720	2.6908	2.6721	2.6390	2.6728	2.6821	2.6728	2.6373	3.2059	3.2428	3.2681	3.2664	3.3137	3.2654	3.3075	3.2644	3.3034	3.2675	3.3177	**3.68**
**H(4`B)**	2.8410	2.8275	2.8256	2.8114	2.7814	2.8123	2.7860	2.8133	2.7915	2.8110	2.7560	2.7132	2.7100	2.6906	2.6975	2.6553	2.6984	2.6707	2.6994	2.6714	2.6969	2.6463	**2.88**

[a] methyl protons shifts are averaged
[b] according to [7]

Table S7. Cont. I

Nuclei	R											S											Exp. [b]
	B971																						
	Gas	$CHCl_3$ (PCM)	$CHCl_3$ (SMD)	DMSO (PCM)	DMSO (SMD)	CH_3OH (PCM)	CH_3OH (SMD)	C_2H_5OH (PCM)	C_2H_5OH (SMD)	H_2O (PCM)	H_2O (SMD)	Gas	$CHCl_3$ (PCM)	$CHCl_3$ (SMD)	DMSO (PCM)	DMSO (SMD)	CH_3OH (PCM)	CH_3OH (SMD)	C_2H_5OH (PCM)	C_2H_5OH (SMD)	H_2O (PCM)	H_2O (SMD)	
**H2**	8.6852	8.6078	8.6116	8.5185	8.5287	8.5250	8.5705	8.5310	8.4361	8.5120	8.5410	8.6852	8.6078	8.6116	8.5185	8.5287	8.5249	8.4392	8.5310	8.4361	8.5120	8.5410	**8.07**
**H3**	7.3178	7.4574	7.5488	7.5026	7.6222	7.5005	7.6432	7.4984	7.6220	7.5049	7.6593	7.3178	7.4574	7.5488	7.5026	7.6222	7.5005	7.6300	7.4984	7.6220	7.5049	7.6592	**7.24**
**H5**	7.1534	7.3313	7.4252	7.4137	7.5380	7.4088	7.5567	7.4040	7.5597	7.4186	7.5715	7.1534	7.3313	7.4252	7.4137	7.5380	7.4088	7.5700	7.4040	7.5596	7.4186	7.5715	**7.24**
**H6**	8.0174	8.2095	8.2837	8.2988	8.4300	8.2934	8.4882	8.2879	8.3690	8.3040	8.5132	8.0174	8.2095	8.2837	8.2988	8.4300	8.2934	8.3881	8.2879	8.3690	8.3040	8.5132	**8.07**
**H8**	4.6121	4.6898	4.7260	4.7349	4.7908	4.7330	4.8426	4.7309	4.7969	4.7365	4.8374	4.6121	4.6898	4.7260	4.7348	4.7908	4.7330	4.8099	4.7309	4.7969	4.7365	4.8374	**5.24**
**H(9A-9B)**	1.7314	1.7750	1.7545	1.7849	1.8122	1.7829	1.8125	1.7811	1.6934	1.7874	1.8300	1.7314	1.7749	1.7545	1.7849	1.8122	1.7829	1.7029	1.7810	1.6934	1.7874	1.8300	**2.135**
**H(10A-10B)**	1.3490	1.2309	1.1938	1.1592	1.0905	1.1643	1.1088	1.1692	1.1193	1.1548	1.0994	1.3490	1.2309	1.1938	1.1592	1.0905	1.1643	1.1157	1.1692	1.1193	1.1548	1.0994	**1.275**
**H(11A-11B)**	0.9780	0.9957	0.9567	1.0136	0.9764	1.0118	0.9721	1.0102	0.9587	1.0158	0.9673	0.9780	0.9957	0.9567	1.0136	0.9764	1.0118	0.9591	1.0102	0.9587	1.0158	0.9673	**1.28**
**H(12A-12B)**	1.2202	1.2018	1.1771	1.1860	1.1448	1.1873	1.1531	1.1885	1.1645	1.1853	1.1521	1.2202	1.2018	1.1771	1.1860	1.1448	1.1873	1.1628	1.1884	1.1645	1.1853	1.1521	**1.285**
**H(13A-13B)**	1.1304	1.1311	1.1014	1.1391	1.1035	1.1384	1.1009	1.1377	1.1004	1.1403	1.1002	1.1304	1.1311	1.1014	1.1391	1.1035	1.1384	1.1001	1.1377	1.1004	1.1403	1.1002	**1.29**
**H(14A-14B)**	1.2837	1.2785	1.2584	1.2749	1.2432	1.2752	1.2473	1.2755	1.2542	1.2751	1.2449	1.2837	1.2785	1.2584	1.2749	1.2432	1.2752	1.2532	1.2755	1.2542	1.2751	1.2449	**1.295**
**H(15A-15C)**	0.9254	0.9202	0.8986	0.9191	0.8896	0.9191	0.8904	0.9191	0.8932	0.9195	0.8876	0.9254	0.9201	0.8986	0.9191	0.8897	0.9191	0.8924	0.9191	0.8932	0.9195	0.8876	**0.82**
**H(1\`A)**	2.7029	2.6995	2.6816	2.6881	2.6466	2.6887	2.6597	2.6895	2.6602	2.6879	2.6349	2.8303	2.8189	2.8165	2.8046	2.7740	2.8052	2.7827	2.8060	2.7852	2.8045	2.7485	**3.82**
**H(1\`B)**	3.2102	3.2465	3.2707	3.2695	3.3157	3.2684	3.3141	3.2672	3.3043	3.2708	3.3175	2.8720	2.6942	2.6887	2.6702	2.6903	2.6701	2.6811	2.6706	2.6831	2.6714	2.6365	**3.78**
**H(2\`A-2\`B)**	1.7231	1.7664	1.7330	1.7983	1.7513	1.7964	1.7661	1.7945	1.7583	1.8005	1.7830	1.5856	1.6670	1.6352	1.7174	1.6677	1.7143	1.7136	1.7112	1.7089	1.7208	1.7283	**2.26**
**H(3\`A-3\`B)**	1.5856	1.6670	1.6352	1.7174	1.6677	1.7142	1.6930	1.7112	1.7089	1.7208	1.7284	1.7231	1.7664	1.7330	1.7983	1.7513	1.7964	1.7622	1.7945	1.7583	1.8005	1.7830	**2.01**
**H(4\`A)**	2.8720	2.6942	2.6887	2.6702	2.6903	2.6701	2.6378	2.6706	2.6831	2.6714	2.6365	3.2102	3.2465	3.2707	3.2695	3.3157	3.2684	3.3082	3.2672	3.3044	3.2708	3.3175	**3.68**
**H(4\`B)**	2.8303	2.8189	2.8165	2.8046	2.7740	2.8052	2.7782	2.8060	2.7852	2.8045	2.7485	2.7029	2.6995	2.6816	2.6881	2.6466	2.6887	2.6594	2.6895	2.6602	2.6879	2.6349	**2.88**
Nuclei	**B972**																						
**H2**	8.6887	8.5379	8.6200	8.4376	8.4966	8.4437	8.4298	8.4495	8.4265	8.4315	8.4204	8.6887	8.5379	8.6200	8.4376	8.4966	8.4437	8.4298	8.4495	8.4265	8.4315	8.4204	**8.07**
**H3**	7.3253	7.4602	7.5558	7.4952	7.6251	7.4934	7.6266	7.4914	7.6190	7.4968	7.6412	7.3253	7.4602	7.5558	7.4952	7.6251	7.4934	7.6266	7.4914	7.6190	7.4968	7.6412	**7.24**
**H5**	7.1513	7.3315	7.4222	7.4177	7.5230	7.4129	7.5667	7.4078	7.5559	7.4221	7.5806	7.1513	7.3315	7.4222	7.4177	7.5230	7.4129	7.5667	7.4078	7.5559	7.4221	7.5806	**7.24**
**H6**	7.9809	8.1701	8.2489	8.2449	8.3897	8.2407	8.3824	8.2361	8.3607	8.2487	8.4095	7.9809	8.1701	8.2489	8.2450	8.3897	8.2407	8.3824	8.2361	8.3607	8.2487	8.4095	**8.07**
**H8**	4.6069	4.6785	4.7065	4.7030	4.7545	4.7017	4.7979	4.7001	4.7822	4.7040	4.7920	4.6069	4.6785	4.7065	4.7030	4.7545	4.7017	4.7979	4.7001	4.7822	4.7040	4.7920	**5.24**
**H(9A-9B)**	1.6994	1.7027	1.7372	1.7113	1.7930	1.7092	1.6834	1.7072	1.6746	1.7135	1.7038	1.6994	1.7027	1.7372	1.7113	1.7930	1.7092	1.6834	1.7072	1.6747	1.7135	1.7038	**2.135**
**H(10A-10B)**	1.3142	1.1970	1.1667	1.1347	1.0648	1.1386	1.0894	1.1424	1.0928	1.1307	1.0889	1.3142	1.1970	1.1667	1.1347	1.0648	1.1386	1.0893	1.1424	1.0928	1.1307	1.0889	**1.275**
**H(11A-11B)**	0.9493	0.9627	0.9332	0.9844	0.9480	0.9826	0.9388	0.9807	0.9380	0.9858	0.9390	0.9493	0.9627	0.9332	0.9844	0.9480	0.9826	0.9388	0.9807	0.9379	0.9858	0.9390	**1.28**
**H(12A-12B)**	1.2000	1.1847	1.1585	1.1744	1.1254	1.1752	1.1453	1.1759	1.1469	1.1733	1.1476	1.2000	1.1847	1.1585	1.1744	1.1254	1.1752	1.1453	1.1759	1.1469	1.1733	1.1476	**1.285**
**H(13A-13B)**	1.1099	1.1101	1.0815	1.1208	1.0830	1.1199	1.0827	1.1189	1.0826	1.1215	1.0851	1.1099	1.1101	1.0815	1.1208	1.0830	1.1199	1.0827	1.1189	1.0826	1.1215	1.0851	**1.29**
**H(14A-14B)**	1.2721	1.2702	1.2473	1.2704	1.2335	1.2705	1.2438	1.2704	1.2446	1.2701	1.2432	1.2721	1.2702	1.2473	1.2704	1.2335	1.2705	1.2438	1.2704	1.2446	1.2701	1.2432	**1.295**
**H(15A-15C)**	0.9220	0.9174	0.8953	0.9192	0.8872	0.9192	0.8901	0.9189	0.8906	0.9192	0.8892	0.9220	0.9174	0.8953	0.9192	0.8872	0.9192	0.8901	0.9189	0.8906	0.9192	0.8891	**0.82**
**H(1\`A)**	2.7009	2.6940	2.6729	2.6791	2.6330	2.6803	2.6446	2.6813	2.6445	2.6778	2.6161	2.8292	2.8167	2.8096	2.8028	2.7727	2.8036	2.7761	2.8042	2.7785	2.8020	2.7405	**3.82**
**H(1\`B)**	3.1955	3.2319	3.2600	3.2641	3.3159	3.2618	3.3000	3.2593	3.2953	3.3662	3.3005	2.8475	2.7035	2.6734	2.6832	2.6943	2.6837	2.6698	2.6843	2.6719	2.6831	2.6607	**3.78**
**H(2\`A-2\`B)**	1.7038	1.7444	1.7062	1.7718	1.7225	1.7700	1.7382	1.7682	1.7341	1.7733	1.7555	1.5630	1.6516	1.6076	1.7033	1.6425	1.7002	1.6903	1.6969	1.6850	1.7061	1.7309	**2.26**
**H(3\`A-3\`B)**	1.5630	1.6516	1.6076	1.7033	1.6425	1.7002	1.6903	1.6969	1.6850	1.7061	1.7309	1.7038	1.7444	1.7062	1.7718	1.7225	1.7700	1.7382	1.7682	1.7341	1.7733	1.7555	**2.01**
**H(4\`A)**	2.8475	2.7035	2.6734	2.6832	2.6943	2.6837	2.6698	2.6843	2.6719	2.6831	2.6607	3.1955	3.2319	3.2600	3.2641	3.3159	3.2618	3.3000	3.2593	3.2953	3.2662	3.3005	**3.68**
**H(4\`B)**	2.8292	2.8167	2.8096	2.8028	2.7727	2.8036	2.7761	2.8042	2.7785	2.8020	2.7405	2.7009	2.6940	2.6728	2.6791	2.6330	2.6803	2.6446	2.6813	2.6445	2.6778	2.6162	**2.88**
Nuclei	**BHandH**																						
**H2**	9.0170	8.9051	8.9385	8.8508	8.8461	8.8543	8.8769	8.8573	8.8930	8.8488	8.8828	9.0170	8.9051	8.9385	8.8508	8.7696	8.8543	8.8976	8.8573	8.8930	8.8488	8.8828	**8.07**
**H3**	7.4567	7.6000	7.6942	7.6445	7.7691	7.6431	7.7516	7.6408	7.7802	7.6469	7.8030	7.4567	7.6000	7.6942	7.6445	7.5633	7.6431	7.7884	7.6408	7.7802	7.6469	7.8030	**7.24**
**H5**	7.2801	7.4726	7.5649	7.5606	7.6921	7.5563	7.6814	7.5511	7.6974	7.5657	7.7331	7.2801	7.4726	7.5649	7.5606	7.4794	7.5563	7.7096	7.5511	7.6974	7.5657	7.7331	**7.24**
**H6**	8.1878	8.3660	8.4746	8.4772	8.6311	8.4704	8.6569	8.4630	8.6783	8.4848	8.7278	8.1878	8.3660	8.4746	8.4772	8.3960	8.4704	8.7047	8.4630	8.6783	8.4848	8.7278	**8.07**
**H8**	4.2844	4.3094	4.4069	4.3786	4.4963	4.3734	4.5524	4.3679	4.5574	4.3849	4.5830	4.2844	4.3094	4.4069	4.3786	4.2974	4.3734	4.5805	4.3679	4.5574	4.3849	4.5830	**5.24**
**H(9A-9B)**	1.8028	1.8603	1.8244	1.8782	1.8611	1.8781	1.8839	1.8773	1.8377	1.8792	1.8759	1.8029	1.8603	1.8244	1.8782	1.7969	1.8781	1.8434	1.8773	1.8377	1.8792	1.8760	**2.135**
**H(10A-10B)**	1.3477	1.2142	1.1778	1.1397	1.0616	1.1450	1.0642	1.1496	1.0823	1.1357	1.0635	1.3477	1.2142	1.1778	1.1397	1.0585	1.1450	1.0794	1.1496	1.0823	1.1358	1.0635	**1.275**
**H(11A-11B)**	0.9522	0.9783	0.9304	0.9933	0.9406	0.9931	0.9365	0.9921	0.9372	0.9946	0.9392	0.9522	0.9783	0.9304	0.9933	0.9120	0.9931	0.9391	0.9921	0.9372	0.9946	0.9392	**1.28**
**H(12A-12B)**	1.2257	1.2025	1.1740	1.1807	1.1354	1.1830	1.1278	1.1844	1.1448	1.1795	1.1429	1.2257	1.2025	1.1741	1.1807	1.0995	1.1830	1.1440	1.1844	1.1447	1.1795	1.1429	**1.285**
**H(13A-13B)**	1.1158	1.1190	1.0803	1.1239	1.0799	1.1243	1.0676	1.1239	1.0772	1.1246	1.0790	1.1158	1.1190	1.0803	1.1239	1.0427	1.1243	1.0777	1.1239	1.0772	1.1246	1.0790	**1.29**
**H(14A-14B)**	1.2680	1.2599	1.2323	1.2505	1.2138	1.2520	1.1977	1.2526	1.2177	1.2501	1.2159	1.2680	1.2599	1.2323	1.2505	1.1693	1.2519	1.2172	1.2526	1.2177	1.2501	1.2159	**1.295**
**H(15A-15C)**	0.9621	0.9564	0.9305	0.9540	0.9210	0.9549	0.8964	0.9550	0.9221	0.9541	0.9207	0.9621	0.9564	0.9305	0.9540	0.8727	0.9549	0.9215	0.9549	0.9221	0.9541	0.9207	**0.82**
**H(1\`A)**	2.5936	2.5836	2.5694	2.5710	2.5281	2.5725	2.5421	2.5732	2.5456	2.5706	2.5204	2.7283	2.7398	2.7023	2.7213	2.6711	2.7239	2.6817	2.7256	2.6846	2.7197	2.6522	**3.82**
**H(1\`B)**	3.1160	3.1325	3.1676	3.1602	3.2130	3.1587	3.1941	3.1566	3.2046	3.1629	3.2121	2.8959	2.7170	2.6626	2.6386	2.6231	2.6444	2.5402	2.6492	2.5510	2.6340	2.5341	**3.78**
**H(2\`A-2\`B)**	1.6779	1.7129	1.6806	1.7437	1.7028	1.7423	1.6923	1.7402	1.7106	1.7461	1.7347	1.5667	1.6372	1.5957	1.6777	1.5965	1.6759	1.6604	1.6734	1.6540	1.6804	1.6970	**2.26**
**H(3\`A-3\`B)**	1.5667	1.6372	1.5957	1.6777	1.6303	1.6759	1.6296	1.6734	1.6540	1.6804	1.6970	1.6779	1.7129	1.6806	1.7437	1.6625	1.7424	1.7155	1.7402	1.7106	1.7461	1.7347	**2.01**
**H(4\`A)**	2.8959	2.7170	2.6626	2.6386	2.6231	2.6444	2.5946	2.6493	2.5510	2.6340	2.5342	3.1160	3.1325	3.1676	3.1602	3.2130	3.1587	3.2089	3.1566	3.2046	3.1628	3.2121	**3.68**
**H(4\`B)**	2.7283	2.7398	2.7023	2.7213	2.6711	2.7240	2.6935	2.7256	2.6846	2.7197	2.6522	2.5936	2.5836	2.5694	2.5710	2.5281	2.5725	2.5454	2.5733	2.5455	2.5706	2.5204	**2.88**
Nuclei	**BHandHLYP**																						
**H2**	8.9150	8.8436	8.8258	8.7456	8.7390	8.7530	8.7662	8.7602	8.7959	8.7381	8.7752	8.9150	8.8436	8.8258	8.7456	8.7390	8.7530	8.8002	8.7602	8.7959	8.7381	8.7752	**8.07**
**H3**	7.4400	7.5788	7.6683	7.6260	7.7400	7.6241	7.7309	7.6220	7.7510	7.6277	7.7763	7.4400	7.5788	7.6683	7.6260	7.7400	7.6241	7.7597	7.6220	7.7510	7.6277	7.7763	**7.24**
**H5**	7.2661	7.4512	7.5377	7.5306	7.6504	7.5264	7.6464	7.5219	7.6606	7.5345	7.6916	7.2661	7.4512	7.5377	7.5306	7.6504	7.5264	7.6732	7.5219	7.6606	7.5345	7.6916	**7.24**
**H6**	8.0607	8.2729	8.3529	8.3735	8.5027	8.3677	8.5458	8.3617	8.5589	8.3786	8.6143	8.0607	8.2729	8.3529	8.3735	8.5027	8.3677	8.5866	8.3617	8.5589	8.3786	8.6143	**8.07**
**H8**	4.2225	4.3192	4.3753	4.3833	4.4522	4.3806	4.5570	4.3775	4.5065	4.3854	4.5171	4.2225	4.3192	4.3754	4.3833	4.4522	4.3806	4.5259	4.3775	4.5065	4.3854	4.5170	**5.24**
**H(9A-9B)**	1.5911	1.6478	1.6127	1.6576	1.6756	1.6552	1.6784	1.6532	1.6700	1.6602	1.7027	1.5911	1.6478	1.6127	1.6576	1.6756	1.6552	1.6822	1.6532	1.6700	1.6602	1.7027	**2.135**
**H(10A-10B)**	1.2887	1.1642	1.1247	1.0942	1.0167	1.0997	1.0229	1.1049	1.0456	1.0889	1.0320	1.2887	1.1642	1.1247	1.0942	1.0167	1.0997	1.0434	1.1049	1.0456	1.0889	1.0320	**1.275**
**H(11A-11B)**	0.9093	0.9316	0.8869	0.9506	0.9053	0.9493	0.9009	0.9479	0.9065	0.9520	0.9055	0.9093	0.9316	0.8869	0.9507	0.9053	0.9493	0.9096	0.9479	0.9066	0.9520	0.9055	**1.28**
**H(12A-12B)**	1.1678	1.1474	1.1206	1.1333	1.0872	1.1348	1.0861	1.1360	1.0992	1.1318	1.0967	1.1678	1.1474	1.1206	1.1333	1.0872	1.1348	1.0989	1.1360	1.0992	1.1318	1.0967	**1.285**
**H(13A-13B)**	1.0767	1.0777	1.0456	1.0881	1.0488	1.0876	1.0391	1.0869	1.0482	1.0887	1.0495	1.0767	1.0777	1.0456	1.0881	1.0488	1.0876	1.0496	1.0869	1.0482	1.0887	1.0495	**1.29**
**H(14A-14B)**	1.2389	1.2324	1.2099	1.2306	1.1956	1.2311	1.1853	1.2314	1.2013	1.2301	1.2004	1.2389	1.2324	1.2099	1.2306	1.1956	1.2311	1.2017	1.2314	1.2013	1.2301	1.2004	**1.295**
**H(15A-15C)**	0.9538	0.9474	0.9238	0.9484	0.9161	0.9486	0.8962	0.9486	0.9188	0.9482	0.9174	0.9538	0.9474	0.9238	0.9484	0.9161	0.9486	0.9192	0.9486	0.9188	0.9482	0.9174	**0.82**
**H(1\`A)**	2.6102	2.6077	2.5823	2.5955	2.5429	2.5968	2.5666	2.5980	2.5639	2.5941	2.5424	2.7444	2.7240	2.7194	2.7068	2.6699	2.7075	2.6791	2.7081	2.6814	2.7064	2.6529	**3.82**
**H(1\`B)**	3.0301	3.0830	3.1019	3.1164	3.1619	3.1144	3.1614	3.1122	3.1587	3.1183	3.1711	2.7085	2.5378	2.5326	2.5189	2.5482	2.5181	2.4839	2.5178	2.4875	2.5204	2.4861	**3.78**
**H(2\`A-2\`B)**	1.6501	1.6897	1.6532	1.7229	1.6721	1.7212	1.6718	1.7192	1.6829	1.7246	1.7050	1.5130	1.5887	1.5598	1.6429	1.5910	1.6399	1.6176	1.6365	1.6114	1.6458	1.6490	**2.26**
**H(3\`A-3\`B)**	1.5130	1.5887	1.5598	1.6430	1.5910	1.6399	1.5978	1.6365	1.6114	1.6458	1.6490	1.6501	1.6897	1.6532	1.7229	1.6721	1.7212	1.6886	1.7192	1.6829	1.7246	1.7050	**2.01**
**H(4\`A)**	2.7085	2.5378	2.5326	2.5189	2.5482	2.5181	2.5197	2.5178	2.4875	2.5204	2.4861	3.0301	3.0830	3.1019	3.1164	3.1619	3.1144	3.1648	3.1122	3.1587	3.1183	3.1711	**3.68**
**H(4\`B)**	2.7444	2.7240	2.7194	2.7068	2.6699	2.7075	2.6844	2.7081	2.6814	2.7064	2.6529	2.6102	2.6076	2.5823	2.5955	2.5429	2.5968	2.5652	2.5980	2.5639	2.5941	2.5424	**2.88**

[a] methyl protons shifts are averaged

[b] according to [7]

Table S7. Cont. II

Nuclei	R											S											Exp. [b]
	BLYP																						
	Gas	$CHCl_3$ (PCM)	$CHCl_3$ (SMD)	DMSO (PCM)	DMSO (SMD)	CH_3OH (PCM)	CH_3OH (SMD)	C_2H_5OH (PCM)	C_2H_5OH (SMD)	H_2O (PCM)	H_2O (SMD)	Gas	$CHCl_3$ (PCM)	$CHCl_3$ (SMD)	DMSO (PCM)	DMSO (SMD)	CH_3OH (PCM)	CH_3OH (SMD)	C_2H_5OH (PCM)	C_2H_5OH (SMD)	H_2O (PCM)	H_2O (SMD)	
**H2**	8.4535	8.3099	8.3833	8.2361	8.2020	8.2891	8.4374	8.2494	8.2316	8.2857	8.4096	8.4535	8.3099	8.3833	8.2361	8.2020	8.2891	8.4070	8.2494	8.2316	8.2857	8.4096	**8.07**
**H3**	7.1756	7.3027	7.3883	7.3357	7.4452	7.3817	7.6153	7.3351	7.4602	7.3923	7.6595	7.1756	7.3027	7.3883	7.3357	7.4452	7.3817	7.6349	7.3351	7.4602	7.3923	7.6595	**7.24**
**H5**	6.9972	7.1760	7.2493	7.2583	7.3909	7.3008	7.5465	7.2508	7.3965	7.3183	7.5953	6.9972	7.1760	7.2493	7.2583	7.3909	7.3008	7.5751	7.2508	7.3965	7.3183	7.5954	**7.24**
**H6**	7.8298	7.9878	8.0732	8.0594	8.1592	8.1031	8.3714	8.0542	8.1777	8.1180	8.4080	7.8298	7.9878	8.0732	8.0594	8.1592	8.1031	8.3682	8.0542	8.1777	8.1180	8.4081	**8.07**
**H8**	4.9684	5.0143	5.0187	5.0206	5.0867	5.0682	5.3137	5.0231	5.0770	5.0754	5.2626	4.9684	5.0143	5.0187	5.0206	5.0867	5.0682	5.2614	5.0231	5.0770	5.0754	5.2626	**5.24**
**H(9A-9B)**	1.8142	1.8165	1.8632	1.8292	1.7940	1.8756	2.0185	1.8296	1.8152	1.8854	2.0229	1.8142	1.8165	1.8632	1.8292	1.7940	1.8756	1.9886	1.8296	1.8152	1.8854	2.0229	**2.135**
**H(10A-10B)**	1.4126	1.3080	1.2577	1.2451	1.1827	1.2967	1.3551	1.2558	1.2017	1.2962	1.3775	1.4126	1.3080	1.2577	1.2451	1.1827	1.2967	1.3678	1.2558	1.2017	1.2962	1.3775	**1.275**
**H(11A-11B)**	1.0598	1.0728	1.0408	1.0906	1.0452	1.1365	1.2140	1.0900	1.0463	1.1474	1.2261	1.0598	1.0728	1.0408	1.0906	1.0452	1.1365	1.2142	1.0900	1.0463	1.1474	1.2261	**1.28**
**H(12A-12B)**	1.2980	1.2896	1.2562	1.2758	1.2410	1.3241	1.4039	1.2800	1.2496	1.3302	1.4297	1.2980	1.2896	1.2562	1.2758	1.2410	1.3241	1.4158	1.2800	1.2496	1.3302	1.4297	**1.285**
**H(13A-13B)**	1.2137	1.2165	1.1832	1.2230	1.1880	1.2697	1.3480	1.2240	1.1882	1.2789	1.3694	1.2137	1.2165	1.1832	1.2230	1.1880	1.2697	1.3555	1.2240	1.1882	1.2789	1.3694	**1.29**
**H(14A-14B)**	1.3717	1.3721	1.3463	1.3683	1.3423	1.4160	1.4972	1.3710	1.3470	1.4233	1.5254	1.3717	1.3721	1.3463	1.3683	1.3423	1.4159	1.5136	1.3710	1.3470	1.4233	1.5254	**1.295**
**H(15A-15C)**	0.9642	0.9602	0.9334	0.9573	0.9316	1.0048	1.0783	0.9598	0.9326	1.0123	1.1103	0.9642	0.9602	0.9334	0.9573	0.9316	1.0048	1.0994	0.9598	0.9326	1.0123	1.1103	**0.82**
**H(1`A)**	2.8596	2.8522	2.8271	2.8391	2.7974	2.8874	2.9814	2.8432	2.8051	2.8934	2.9497	2.9705	2.9701	2.9630	2.9434	2.9187	2.9927	3.0862	2.9494	2.9252	2.9970	3.0573	**3.82**
**H(1`B)**	3.3821	3.4122	3.4355	3.4344	3.4933	3.4804	3.6513	3.4337	3.4718	3.4910	3.6540	3.0238	2.8539	2.8033	2.8196	2.8379	2.8683	2.9769	2.8245	2.8108	2.8738	2.9857	**3.78**
**H(2`A-2`B)**	1.8078	1.8484	1.8015	1.8724	1.8323	1.9184	1.9918	1.8717	1.8358	1.9291	2.0394	1.6432	1.7393	1.6954	1.7862	1.7519	1.8303	1.9442	1.7821	1.7725	1.8446	1.9937	**2.26**
**H(3`A-3`B)**	1.6432	1.7393	1.6954	1.7861	1.7519	1.8303	1.9131	1.7821	1.7725	1.8446	1.9937	1.8078	1.8484	1.8015	1.8724	1.8323	1.9184	2.0079	1.8717	1.8358	1.9291	2.0395	**2.01**
**H(4`A)**	3.0238	2.8539	2.8033	2.8196	2.8379	2.8683	2.9907	2.8245	2.8108	2.8738	2.9858	3.3821	3.4122	3.4355	3.4344	3.4933	3.4804	3.6433	3.4337	3.4718	3.4910	3.6540	**3.68**
**H(4`B)**	2.9705	2.9701	2.9630	2.9435	2.9187	2.9927	3.0900	2.9494	2.9252	2.9970	3.0574	2.8596	2.8522	2.8271	2.8391	2.7974	2.8874	2.9712	2.8432	2.8051	2.8934	2.9497	**2.88**
Nuclei	BMK																						
**H2**	9.0627	9.0228	9.0111	8.9585	8.9678	8.9579	9.0493	8.9569	9.0453	8.9490	9.0386	9.0627	9.0228	9.0111	8.9585	8.9678	8.9579	9.0493	8.9569	9.0453	8.9490	9.0386	**8.07**
**H3**	7.6124	7.7732	7.8608	7.8262	7.9489	7.8202	7.9736	7.8141	7.9661	7.8224	7.9999	7.6124	7.7732	7.8608	7.8262	7.9489	7.8202	7.9736	7.8141	7.9661	7.8224	7.9999	**7.24**
**H5**	7.4921	7.6732	7.7490	7.7424	7.8695	7.7345	7.8914	7.7266	7.8802	7.7405	7.9089	7.4921	7.6732	7.7490	7.7424	7.8695	7.7345	7.8914	7.7266	7.8802	7.7404	7.9089	**7.24**
**H6**	8.4292	8.6281	8.6811	8.7248	8.8657	8.7127	8.8858	8.7014	8.8628	8.7280	8.9015	8.4292	8.6281	8.6811	8.7248	8.8657	8.7127	8.8858	8.7014	8.8628	8.7280	8.9015	**8.07**
**H8**	4.4581	4.4657	4.5471	4.5017	4.6695	4.4909	4.6627	4.4817	4.6492	4.5054	4.6577	4.4581	4.4656	4.5471	4.5017	4.6695	4.4909	4.6627	4.4817	4.6492	4.5053	4.6577	**5.24**
**H(9A-9B)**	1.6621	1.7742	1.6807	1.8006	1.7342	1.7978	1.8198	1.7935	1.8050	1.7906	1.8791	1.6621	1.7742	1.6807	1.8006	1.7342	1.7978	1.8197	1.7935	1.8050	1.7906	1.8791	**2.135**
**H(10A-10B)**	1.3436	1.1984	1.1153	1.0959	0.9961	1.0999	1.0490	1.1030	1.0512	1.0811	1.0583	1.3436	1.1984	1.1153	1.0960	0.9961	1.0999	1.0490	1.1030	1.0512	1.0811	1.0583	**1.275**
**H(11A-11B)**	0.9400	0.9525	0.8875	0.9640	0.9155	0.9584	0.9126	0.9532	0.9126	0.9605	0.9106	0.9401	0.9525	0.8875	0.9640	0.9155	0.9584	0.9126	0.9532	0.9126	0.9605	0.9106	**1.28**
**H(12A-12B)**	1.1929	1.1688	1.1216	1.1452	1.0958	1.1429	1.1039	1.1407	1.1066	1.1380	1.1009	1.1929	1.1688	1.1216	1.1452	1.0958	1.1429	1.1039	1.1407	1.1066	1.1380	1.1009	**1.285**
**H(13A-13B)**	1.0816	1.0826	1.0376	1.0872	1.0427	1.0832	1.0447	1.0792	1.0457	1.0815	1.0483	1.0816	1.0826	1.0376	1.0872	1.0427	1.0832	1.0447	1.0792	1.0457	1.0815	1.0483	**1.29**
**H(14A-14B)**	1.2204	1.2100	1.1762	1.2020	1.1608	1.1992	1.1674	1.1963	1.1693	1.1951	1.1682	1.2203	1.2100	1.1762	1.2020	1.1608	1.1992	1.1674	1.1963	1.1693	1.1951	1.1682	**1.295**
**H(15A-15C)**	0.8777	0.8689	0.8386	0.8681	0.8316	0.8648	0.8349	0.8615	0.8364	0.8616	0.8381	0.8777	0.8689	0.8386	0.8681	0.8316	0.8648	0.8349	0.8615	0.8364	0.8616	0.8381	**0.82**
**H(1`A)**	2.6154	2.6064	2.5872	2.5983	2.5634	2.5944	2.5546	2.5910	2.5576	2.5932	2.5132	2.7379	2.7451	2.7372	2.7380	2.6936	2.7367	2.6943	2.7353	2.7007	2.7299	2.6676	**3.82**
**H(1`B)**	3.0946	3.1480	3.1367	3.1610	3.1704	3.1595	3.1981	3.1566	3.1951	3.1504	3.1955	2.6890	2.4769	2.5208	2.4811	2.5279	2.4758	2.4416	2.4716	2.4507	2.4785	2.4512	**3.78**
**H(2`A-2`B)**	1.6966	1.7123	1.6876	1.7418	1.7076	1.7356	1.6990	1.7297	1.6977	1.7389	1.7027	1.5428	1.6152	1.5833	1.6615	1.6157	1.6550	1.6272	1.6489	1.6240	1.6588	1.6562	**2.26**
**H(3`A-3`B)**	1.5428	1.6152	1.5833	1.6615	1.6157	1.6550	1.6271	1.6489	1.6240	1.6588	1.6562	1.6966	1.7123	1.6876	1.7418	1.7076	1.7356	1.6990	1.7297	1.6977	1.7389	1.7027	**2.01**
**H(4`A)**	2.6890	2.4769	2.5208	2.4811	2.5279	2.4758	2.4416	2.4716	2.4507	2.4785	2.4512	3.0946	3.1480	3.1367	3.1610	3.1704	3.1595	3.1981	3.1566	3.1951	3.1504	3.1955	**3.68**
**H(4`B)**	2.7379	2.7451	2.7372	2.7380	2.6936	2.7367	2.6943	2.7353	2.7007	2.7299	2.6676	2.6154	2.6064	2.5872	2.5983	2.5634	2.5944	2.5546	2.5910	2.5576	2.5932	2.5132	**2.88**
Nuclei	BP86																						
**H2**	8.5211	8.4160	8.4356	8.3431	8.3460	8.3496	8.3612	8.3555	8.3850	8.3361	8.3758	8.5211	8.4160	8.4356	8.3431	8.3460	8.3496	8.3734	8.3555	8.3850	8.3361	8.3758	**8.07**
**H3**	7.1929	7.3340	7.4134	7.3753	7.4851	7.3743	7.4787	7.3728	7.4971	7.3760	7.5362	7.1929	7.3340	7.4134	7.3753	7.4852	7.3743	7.5058	7.3728	7.4971	7.3760	7.5362	**7.24**
**H5**	6.9950	7.1707	7.2555	7.2507	7.3666	7.2469	7.3611	7.2428	7.3740	7.2538	7.4177	6.9950	7.1707	7.2555	7.2506	7.3666	7.2469	7.3841	7.2428	7.3740	7.2538	7.4177	**7.24**
**H6**	7.9235	8.1146	8.1659	8.1933	8.2926	8.1904	8.3271	8.1868	8.3382	8.1953	8.4034	7.9235	8.1146	8.1659	8.1933	8.2926	8.1904	8.3574	8.1868	8.3382	8.1953	8.4034	**8.07**
**H8**	4.9854	5.0537	5.0470	5.0682	5.0819	5.0697	5.1716	5.0705	5.1218	5.0659	5.1501	4.9854	5.0537	5.0470	5.0682	5.0819	5.0697	5.1324	5.0705	5.1218	5.0659	5.1501	**5.24**
**H(9A-9B)**	1.9156	1.9378	1.9296	1.9623	1.9953	1.9602	1.9733	1.9582	1.9753	1.9643	2.0210	1.9156	1.9379	1.9296	1.9623	1.9953	1.9602	1.9806	1.9582	1.9753	1.9643	2.0210	**2.135**
**H(10A-10B)**	1.4202	1.3133	1.2640	1.2494	1.1743	1.2545	1.1770	1.2593	1.1895	1.2443	1.1977	1.4202	1.3133	1.2640	1.2494	1.1743	1.2545	1.1859	1.2593	1.1895	1.2443	1.1977	**1.275**
**H(11A-11B)**	1.0518	1.0710	1.0227	1.0903	1.0424	1.0895	1.0327	1.0885	1.0341	1.0908	1.0471	1.0518	1.0710	1.0227	1.0903	1.0424	1.0895	1.0334	1.0885	1.0341	1.0909	1.0471	**1.28**
**H(12A-12B)**	1.2900	1.2812	1.2428	1.2659	1.2133	1.2680	1.2104	1.2696	1.2207	1.2636	1.2340	1.2900	1.2812	1.2428	1.2659	1.2133	1.2680	1.2193	1.2696	1.2207	1.2636	1.2340	**1.285**
**H(13A-13B)**	1.2001	1.2041	1.1613	1.2108	1.1644	1.2110	1.1522	1.2109	1.1590	1.2103	1.1737	1.2001	1.2041	1.1613	1.2108	1.1644	1.2110	1.1587	1.2109	1.1590	1.2103	1.1737	**1.29**
**H(14A-14B)**	1.3511	1.3509	1.3184	1.3462	1.3041	1.3474	1.2915	1.3483	1.3071	1.3447	1.3190	1.3511	1.3509	1.3184	1.3462	1.3041	1.3474	1.3069	1.3483	1.3071	1.3447	1.3190	**1.295**
**H(15A-15C)**	0.9368	0.9356	0.9010	0.9325	0.8926	0.9336	0.8710	0.9343	0.8923	0.9312	0.9037	0.9368	0.9356	0.9010	0.9325	0.8926	0.9336	0.8919	0.9343	0.8923	0.9312	0.9037	**0.82**
**H(1`A)**	2.8051	2.8086	2.7800	2.7948	2.7420	2.7967	2.7582	2.7983	2.7511	2.7925	2.7334	2.9245	2.9231	2.9062	2.9072	2.8712	2.9089	2.8718	2.9104	2.8727	2.9053	2.8464	**3.82**
**H(1`B)**	3.3976	3.4262	3.4319	3.4461	3.4796	3.4459	3.4732	3.4453	3.4677	3.4459	3.4874	3.0775	2.8757	2.8390	2.8259	2.8309	2.8282	2.7764	2.8308	2.7751	2.8240	2.7809	**3.78**
**H(2`A-2`B)**	1.8009	1.8424	1.7943	1.8664	1.8096	1.8657	1.8055	1.8648	1.8178	1.8667	1.8549	1.6352	1.7278	1.6788	1.7716	1.7091	1.7698	1.7378	1.7676	1.7302	1.7728	1.7894	**2.26**
**H(3`A-3`B)**	1.6352	1.7278	1.6788	1.7716	1.7091	1.7698	1.7168	1.7676	1.7302	1.7728	1.7895	1.8009	1.8424	1.7943	1.8664	1.8096	1.8657	1.8232	1.8648	1.8178	1.8667	1.8549	**2.01**
**H(4`A)**	3.0775	2.8757	2.8390	2.8259	2.8309	2.8282	2.8063	2.8307	2.7751	2.8240	2.7810	3.3976	3.4262	3.4319	3.4461	3.4796	3.4459	3.4727	3.4452	3.4677	3.4459	3.4873	**3.68**
**H(4`B)**	2.9245	2.9231	2.9062	2.9072	2.8712	2.9089	2.8772	2.9103	2.8727	2.9053	2.8464	2.8051	2.8086	2.7800	2.7948	2.7420	2.7967	2.7504	2.7982	2.7511	2.7925	2.7334	**2.88**
Nuclei	CAM-B3LYP																						
**H2**	8.8137	8.7427	8.7185	8.6046	8.6226	8.6133	8.6259	8.6217	8.6669	8.5956	8.5831	8.8137	8.7427	8.7185	8.6046	8.6226	8.6133	8.6539	8.6217	8.6669	8.5956	8.5831	**8.07**
**H3**	7.4056	7.5424	7.6331	7.5855	7.7038	7.5840	7.6921	7.5824	7.7150	7.5868	7.7349	7.4056	7.5424	7.6331	7.5855	7.7038	7.5839	7.7225	7.5824	7.7150	7.5868	7.7349	**7.24**
**H5**	7.2427	7.4267	7.5144	7.4995	7.6257	7.4954	7.6153	7.4914	7.6329	7.5033	7.6554	7.2427	7.4267	7.5144	7.4995	7.6258	7.4954	7.6417	7.4914	7.6329	7.5033	7.6554	**7.24**
**H6**	8.0301	8.2398	8.3133	8.3260	8.4567	8.3209	8.4890	8.3154	8.5026	8.3304	8.5351	8.0301	8.2398	8.3133	8.3260	8.4568	8.3209	8.5206	8.3154	8.5026	8.3304	8.5351	**8.07**
**H8**	4.4185	4.5093	4.5609	4.5563	4.6265	4.5552	4.7167	4.5537	4.6736	4.5567	4.6645	4.4185	4.5093	4.5609	4.5563	4.6265	4.5552	4.6844	4.5537	4.6736	4.5567	4.6645	**5.24**
**H(9A-9B)**	1.6860	1.7382	1.7028	1.7332	1.7652	1.7310	1.7525	1.7292	1.7521	1.7356	1.7549	1.6860	1.7382	1.7028	1.7332	1.7651	1.7310	1.7567	1.7292	1.7521	1.7356	1.7549	**2.135**
**H(10A-10B)**	1.3235	1.2065	1.1686	1.1281	1.0653	1.1334	1.0616	1.1388	1.0865	1.1229	1.0600	1.3235	1.2065	1.1686	1.1281	1.0653	1.1334	1.0797	1.1388	1.0865	1.1229	1.0600	**1.275**
**H(11A-11B)**	0.9652	0.9883	0.9435	0.9989	0.9632	0.9975	0.9493	0.9964	0.9570	1.0001	0.9406	0.9652	0.9883	0.9436	0.9989	0.9632	0.9975	0.9549	0.9964	0.9570	1.0001	0.9406	**1.28**
**H(12A-12B)**	1.2202	1.2025	1.1771	1.1852	1.1451	1.1867	1.1385	1.1882	1.1533	1.1837	1.1452	1.2202	1.2024	1.1771	1.1853	1.1451	1.1867	1.1507	1.1882	1.1533	1.1837	1.1452	**1.285**
**H(13A-13B)**	1.1315	1.1337	1.1023	1.1400	1.1063	1.1394	1.0915	1.1391	1.1023	1.1404	1.0967	1.1315	1.1337	1.1023	1.1400	1.1063	1.1394	1.1010	1.1391	1.1023	1.1404	1.0967	**1.29**
**H(14A-14B)**	1.2864	1.2817	1.2602	1.2787	1.2470	1.2792	1.2339	1.2797	1.2508	1.2782	1.2469	1.2864	1.2817	1.2602	1.2787	1.2470	1.2792	1.2499	1.2797	1.2508	1.2782	1.2469	**1.295**
**H(15A-15C)**	0.9690	0.9644	0.9415	0.9633	0.9347	0.9635	0.9118	0.9638	0.9354	0.9630	0.9308	0.9690	0.9644	0.9414	0.9633	0.9347	0.9635	0.9344	0.9638	0.9354	0.9630	0.9308	**0.82**
**H(1`A)**	2.7122	2.7112	2.6890	2.6973	2.6501	2.6987	2.6685	2.7002	2.6667	2.6957	2.6354	2.8374	2.8193	2.8158	2.8689	2.7726	2.8086	2.7811	2.8094	2.7817	2.8077	2.6354	**3.82**
**H(1`B)**	3.1354	3.1829	3.2019	3.2098	3.2596	3.2081	3.2536	3.2065	3.2535	3.2113	3.2592	2.8053	2.6264	2.6258	2.6789	2.6368	2.6172	2.5885	2.6172	2.5863	2.6193	3.2592	**3.78**
**H(2`A-2`B)**	1.7172	1.7561	1.7220	1.7856	1.7408	1.7840	1.7358	1.7825	1.7498	1.7871	1.7711	1.5823	1.6597	1.6318	1.7170	1.6665	1.7139	1.6941	1.7110	1.6865	1.7198	1.7378	**2.26**
**H(3`A-3`B)**	1.5823	1.6597	1.6318	1.7170	1.6666	1.7139	1.6727	1.7110	1.6865	1.7198	1.7378	1.7172	1.7561	1.7220	1.7418	1.7408	1.7840	1.7541	1.7825	1.7498	1.7871	1.7711	**2.01**
**H(4`A)**	2.8053	2.6264	2.6258	2.6178	2.6368	2.6172	2.6177	2.6172	2.5863	2.6193	2.6007	3.1354	3.1829	3.2018	3.2549	3.2596	3.2081	3.2581	3.2065	3.2535	3.2113	2.6007	**3.68**
**H(4`B)**	2.8374	2.8193	2.8158	2.8081	2.7726	2.8086	2.7865	2.8094	2.7817	2.8077	2.7551	2.7122	2.7112	2.6890	2.7421	2.6501	2.6987	2.6658	2.7002	2.6667	2.6957	2.7551	**2.88**

[a] methyl protons shifts are averaged
[b] according to [7]

Table S7. Cont. III

Nuclei	R											S											Exp. [b]
	HF																						
	Gas	$CHCl_3$ (PCM)	$CHCl_3$ (SMD)	DMSO (PCM)	DMSO (SMD)	CH_3OH (PCM)	CH_3OH (SMD)	C_2H_5OH (PCM)	C_2H_5OH (SMD)	H_2O (PCM)	H_2O (SMD)	Gas	$CHCl_3$ (PCM)	$CHCl_3$ (SMD)	DMSO (PCM)	DMSO (SMD)	CH_3OH (PCM)	CH_3OH (SMD)	C_2H_5OH (PCM)	C_2H_5OH (SMD)	H_2O (PCM)	H_2O (SMD)	
**H2**	9.0669	8.9556	8.9901	8.7631	8.7246	8.7728	8.7533	8.7820	8.7511	8.7528	8.7429	9.0669	8.9556	8.9901	8.7631	8.7246	8.7728	8.7533	8.7820	8.7511	8.7528	8.7430	**8.07**
**H3**	7.4294	7.5691	7.6582	7.6152	7.7384	7.6132	7.7511	7.6108	7.7442	7.6172	7.7667	7.4294	7.5691	7.6582	7.6152	7.7384	7.6132	7.7511	7.6108	7.7442	7.6172	7.7667	**7.24**
**H5**	7.2468	7.4436	7.5321	7.5293	7.6578	7.5244	7.6694	7.5193	7.6586	7.5340	7.6936	7.2468	7.4436	7.5321	7.5293	7.6578	7.5244	7.6694	7.5193	7.6586	7.5340	7.6936	**7.24**
**H6**	8.0744	8.3036	8.4070	8.3982	8.5365	8.3926	8.5911	8.3866	8.5665	8.4031	8.6246	8.0744	8.3036	8.4070	8.3982	8.5365	8.3926	8.5911	8.3866	8.5665	8.4031	8.6246	**8.07**
**H8**	3.6336	3.7565	3.8251	3.8267	3.9402	3.8226	3.9863	3.8182	3.9645	3.8305	3.9882	3.6336	3.7565	3.8251	3.8267	3.9402	3.8226	3.9863	3.8182	3.9645	3.8305	3.9882	**5.24**
**H(9A-9B)**	1.3084	1.3386	1.3327	1.3126	1.2781	1.3118	1.3017	1.3109	1.2912	1.3131	1.3209	1.3084	1.3386	1.3327	1.3126	1.2781	1.3118	1.3017	1.3109	1.2912	1.3131	1.3209	**2.135**
**H(10A-10B)**	1.1757	1.0355	1.0026	0.9528	0.8871	0.9579	0.8942	0.9628	0.8992	0.9481	0.8886	1.1757	1.0355	1.0026	0.9528	0.8871	0.9579	0.8942	0.9628	0.8992	0.9481	0.8886	**1.275**
**H(11A-11B)**	0.7727	0.7822	0.7491	0.7923	0.7424	0.7906	0.7424	0.7890	0.7420	0.7939	0.7448	0.7727	0.7822	0.7491	0.7923	0.7424	0.7906	0.7424	0.7890	0.7420	0.7939	0.7449	**1.28**
**H(12A-12B)**	1.0258	1.0005	0.9756	0.9869	0.9484	0.9879	0.9519	0.9888	0.9539	0.9861	0.9534	1.0258	1.0005	0.9756	0.9870	0.9484	0.9879	0.9518	0.9888	0.9539	0.9861	0.9534	**1.285**
**H(13A-13B)**	0.9397	0.9382	0.9089	0.9459	0.9080	0.9451	0.9055	0.9442	0.9057	0.9467	0.9091	0.9397	0.9381	0.9089	0.9459	0.9080	0.9451	0.9055	0.9442	0.9057	0.9467	0.9091	**1.29**
**H(14A-14B)**	1.1123	1.1065	1.0835	1.1061	1.0752	1.1063	1.0765	1.1063	1.0774	1.1060	1.0771	1.1123	1.1065	1.0835	1.1061	1.0752	1.1063	1.0765	1.1063	1.0774	1.1060	1.0771	**1.295**
**H(15A-15C)**	0.9246	0.9168	0.8945	0.9193	0.8903	0.9192	0.8899	0.9188	0.8904	0.9196	0.8908	0.9246	0.9168	0.8945	0.9193	0.8903	0.9192	0.8899	0.9188	0.8904	0.9196	0.8908	**0.82**
**H(1`A)**	2.3785	2.3730	2.3471	2.3565	2.3023	2.3578	2.3282	2.3589	2.3267	2.3554	2.3100	2.5255	2.5057	2.4921	2.4867	3.2393	2.4877	2.4710	2.4886	2.4713	2.4859	2.4432	**3.82**
**H(1`B)**	2.7269	2.7948	2.8266	2.8273	2.8806	2.8249	2.8793	2.8224	2.8729	2.8295	2.8867	2.3711	2.2566	2.2390	2.2718	3.1035	2.2707	2.2847	2.2697	2.2843	2.2734	2.2859	**3.78**
**H(2`A-2`B)**	1.4839	1.5326	1.4910	1.5677	1.5188	1.5657	1.5290	1.5636	1.5244	1.5697	1.5471	1.3771	1.4631	1.4254	1.5342	1.4936	1.5302	1.5186	1.5261	1.5127	1.5383	1.5479	**2.26**
**H(3`A-3`B)**	1.3771	1.4631	1.4254	1.5342	1.4936	1.5302	1.5186	1.5261	1.5127	1.5383	1.5479	1.4839	1.5326	1.4910	1.5677	1.5188	1.5658	1.5289	1.5636	1.5244	1.5697	1.5471	**2.01**
**H(4`A)**	2.3711	2.2566	2.2390	2.2718	2.3030	2.2707	2.2847	2.2697	2.2843	2.2734	2.2859	2.7269	2.7948	2.8267	2.8273	3.7263	2.8249	2.8793	2.8224	2.8729	2.8295	2.8867	**3.68**
**H(4`B)**	2.5255	2.5057	2.4920	2.4867	2.4422	2.4877	2.4710	2.4886	2.4713	2.4858	2.4432	2.3785	2.3730	2.3471	2.3565	3.1168	2.3579	2.3282	2.3589	2.3267	2.3554	2.3100	**2.88**
Nuclei	**LC-BLYP**																						
**H2**	9.0090	8.9172	8.8774	8.7674	8.7637	8.7674	8.7446	8.8105	8.7309	8.7642	8.7211	9.0090	8.9172	8.8774	8.7674	8.7637	8.7672	8.7446	8.8105	8.7309	8.7642	8.7211	**8.07**
**H3**	7.5442	7.6758	7.7680	7.7162	7.8367	7.7133	7.8535	7.7128	7.8459	7.7180	7.8685	7.5442	7.6758	7.7680	7.7163	7.8367	7.7133	7.8535	7.7128	7.8459	7.7180	7.8685	**7.24**
**H5**	7.3920	7.5824	7.6728	7.6621	7.7888	7.6563	7.7984	7.6543	7.7872	7.6664	7.8208	7.3920	7.5824	7.6728	7.6621	7.7888	7.6563	7.7984	7.6544	7.7872	7.6664	7.8208	**7.24**
**H6**	8.1874	8.3994	8.4752	8.4661	8.6185	8.4552	8.6584	8.4705	8.6298	8.4737	8.6873	8.1875	8.3993	8.4752	8.4660	8.6185	8.4552	8.6583	8.4705	8.6298	8.4737	8.6873	**8.07**
**H8**	4.2077	4.3175	4.3817	4.3246	4.4577	4.3126	4.5153	4.3399	4.4897	4.3333	4.5219	4.2077	4.3175	4.3817	4.3246	4.4577	4.3125	4.5153	4.3399	4.4897	4.3333	4.5219	**5.24**
**H(9A-9B)**	1.6659	1.6997	1.6590	1.7261	1.7126	1.7266	1.6785	1.7206	1.6702	1.7247	1.7099	1.6659	1.6997	1.6590	1.7261	1.7126	1.7266	1.6785	1.7206	1.6702	1.7247	1.7099	**2.135**
**H(10A-10B)**	1.2866	1.1578	1.1207	1.0620	1.0043	1.0654	1.0050	1.0764	1.0076	1.0578	0.9989	1.2865	1.1578	1.1206	1.0620	1.0043	1.0654	1.0050	1.0764	1.0076	1.0578	0.9989	**1.275**
**H(11A-11B)**	0.9378	0.9566	0.9095	0.9662	0.9234	0.9644	0.9016	0.9649	0.8999	0.9668	0.9023	0.9378	0.9566	0.9095	0.9661	0.9234	0.9644	0.9016	0.9649	0.8999	0.9668	0.9023	**1.28**
**H(12A-12B)**	1.1999	1.1746	1.1506	1.1485	1.1119	1.1487	1.1132	1.1527	1.1141	1.1474	1.1144	1.1999	1.1746	1.1506	1.1486	1.1119	1.1487	1.1132	1.1527	1.1141	1.1475	1.1144	**1.285**
**H(13A-13B)**	1.1103	1.1093	1.0778	1.1134	1.0777	1.1121	1.0675	1.1125	1.0671	1.1137	1.0699	1.1103	1.1093	1.0778	1.1134	1.0777	1.1121	1.0674	1.1125	1.0670	1.1137	1.0699	**1.29**
**H(14A-14B)**	1.2590	1.2495	1.2279	1.2428	1.2107	1.2423	1.2123	1.2434	1.2129	1.2423	1.2128	1.2590	1.2494	1.2279	1.2428	1.2108	1.2422	1.2123	1.2434	1.2129	1.2423	1.2128	**1.295**
**H(15A-15C)**	0.9733	0.9650	0.9432	0.9632	0.9343	0.9622	0.9324	0.9634	0.9327	0.9632	0.9327	0.9732	0.9650	0.9432	0.9632	0.9344	0.9622	0.9324	0.9633	0.9327	0.9631	0.9327	**0.82**
**H(1`A)**	2.6766	2.6717	2.6489	2.6511	2.6063	2.6507	2.6202	2.6556	2.6204	2.5622	2.5969	2.7970	2.7801	2.7657	2.7792	2.7297	2.7811	2.7498	2.7746	2.7535	2.7768	2.7222	**3.82**
**H(1`B)**	3.0280	3.0713	3.0899	3.0911	3.1423	3.0863	3.1407	3.0926	3.1345	2.7776	3.1488	2.7236	2.5359	2.5536	2.5538	2.5546	2.5606	2.5267	2.5313	2.5351	2.5491	2.5240	**3.78**
**H(2`A-2`B)**	1.6938	1.7312	1.7027	1.7597	1.7219	1.7563	1.7326	1.7563	1.7275	1.7620	1.7524	1.5820	1.6600	1.6326	1.7150	1.6703	1.7107	1.7064	1.7072	1.7008	1.7182	1.7430	**2.26**
**H(3`A-3`B)**	1.5820	1.6600	1.6326	1.7150	1.6703	1.7107	1.7064	1.7072	1.7008	1.7182	1.7430	1.6938	1.7312	1.7027	1.7597	1.7219	1.7563	1.7326	1.7564	1.7275	1.7620	1.7524	**2.01**
**H(4`A)**	2.7236	2.5359	2.5536	2.5538	2.5545	2.5606	2.5267	2.5313	2.5351	2.5491	2.5240	3.0280	3.0713	3.0899	3.0911	3.1423	3.0863	3.1408	3.0926	3.1345	3.0942	3.1488	**3.68**
**H(4`B)**	2.7970	2.7801	2.7657	2.7792	2.7297	2.7811	2.7498	2.7746	2.7535	2.7768	2.7222	2.6766	2.6717	2.6489	2.6511	2.6063	2.6507	2.6202	2.6557	2.6204	2.6502	2.5969	**2.88**
Nuclei	**M06**																						
**H2**	8.7928	8.4981	8.5707	8.4194	8.4125	8.4243	8.4639	8.4296	8.4620	8.4154	8.4538	8.7928	8.4981	8.5707	8.4194	8.4125	8.4243	8.4635	8.4296	8.4620	8.4154	8.4538	**8.07**
**H3**	7.3654	7.4406	7.5625	7.4698	7.5889	7.4682	7.5910	7.4671	7.6073	7.4719	7.6465	7.3654	7.4406	7.5625	7.4698	7.5889	7.4682	7.6142	7.4671	7.6073	7.4719	7.6465	**7.24**
**H5**	6.9860	7.2831	7.3165	7.3832	7.5260	7.3775	7.5112	7.3723	7.5229	7.3890	7.5911	6.9860	7.2831	7.3165	7.3832	7.5260	7.3775	7.5414	7.3723	7.5229	7.3890	7.5911	**7.24**
**H6**	7.9129	8.1474	8.1943	8.2181	8.3472	8.2135	8.3817	8.2095	8.3682	8.2229	8.4170	7.9129	8.1474	8.1943	8.2181	8.3472	8.2135	8.3910	8.2095	8.3682	8.2229	8.4170	**8.07**
**H8**	4.3875	4.4787	4.4779	4.5111	4.5782	4.5089	4.6607	4.5071	4.5985	4.5136	4.6913	4.3875	4.4787	4.4779	4.5111	4.5782	4.5089	4.6204	4.5071	4.5985	4.5136	4.6913	**5.24**
**H(9A-9B)**	1.8387	1.7688	1.7995	1.7696	1.7495	1.7683	1.7853	1.7676	1.7550	1.7717	1.7108	1.8387	1.7688	1.7995	1.7696	1.7495	1.7683	1.7507	1.7676	1.7550	1.7717	1.7108	**2.135**
**H(10A-10B)**	1.3386	1.1655	1.1535	1.1144	1.0456	1.1173	1.0660	1.1207	1.0603	1.1123	1.0297	1.3386	1.1655	1.1536	1.1144	1.0456	1.1173	1.0537	1.1207	1.0603	1.1123	1.0297	**1.275**
**H(11A-11B)**	1.0466	1.0091	1.0034	1.0230	0.9823	1.0215	0.9775	1.0205	0.9836	1.0252	0.9346	1.0466	1.0091	1.0034	1.0230	0.9823	1.0215	0.9812	1.0205	0.9836	1.0252	0.9347	**1.28**
**H(12A-12B)**	1.1887	1.1855	1.1623	1.1773	1.1430	1.1778	1.1367	1.1787	1.1489	1.1775	1.1440	1.1887	1.1855	1.1624	1.1773	1.1430	1.1778	1.1482	1.1788	1.1489	1.1775	1.1440	**1.285**
**H(13A-13B)**	1.1510	1.1397	1.1200	1.1472	1.1089	1.1463	1.1035	1.1460	1.1090	1.1486	1.1008	1.1510	1.1397	1.1200	1.1472	1.1089	1.1463	1.1080	1.1460	1.1090	1.1486	1.1008	**1.29**
**H(14A-14B)**	1.2292	1.2248	1.2093	1.2150	1.1934	1.2154	1.1749	1.2163	1.1967	1.2152	1.1959	1.2293	1.2248	1.2093	1.2150	1.1934	1.2154	1.1954	1.2163	1.1967	1.2152	1.1959	**1.295**
**H(15A-15C)**	0.9148	0.9145	0.8994	0.9138	0.8937	0.9135	0.8693	0.9136	0.8957	0.9148	0.9001	0.9148	0.9145	0.8994	0.9138	0.8937	0.9135	0.8952	0.9136	0.8957	0.9148	0.9001	**0.82**
**H(1`A)**	2.7167	2.6896	2.6661	2.6699	2.6343	2.6711	2.6475	2.6729	2.6449	2.6692	2.6479	2.8881	2.8154	2.8217	2.7853	2.7501	2.7870	2.7779	2.7894	2.7801	2.7842	2.7525	**3.82**
**H(1`B)**	3.0905	3.1403	3.1518	3.1683	3.2274	3.1658	3.2112	3.1639	3.2028	3.1712	3.2009	2.7251	2.6174	2.6394	2.6205	2.6119	2.6193	2.6250	2.6188	2.6288	2.6225	2.5793	**3.78**
**H(2`A-2`B)**	1.6210	1.7047	1.6519	1.7362	1.7015	1.7338	1.6961	1.7321	1.7050	1.7389	1.7622	1.5772	1.6691	1.6316	1.6941	1.6542	1.6924	1.6693	1.6912	1.6642	1.6962	1.7078	**2.26**
**H(3`A-3`B)**	1.5772	1.6691	1.6316	1.6941	1.6543	1.6924	1.6532	1.6912	1.6642	1.6962	1.7078	1.6210	1.7047	1.6519	1.7361	1.7015	1.7338	1.7111	1.7321	1.7050	1.7389	1.7622	**2.01**
**H(4`A)**	2.7251	2.6174	2.6394	2.6205	2.6119	2.6193	2.6270	2.6188	2.6288	2.6225	2.5793	3.0905	3.1403	3.1518	3.1683	3.2274	3.1658	3.2059	3.1639	3.2028	3.1712	3.2009	**3.68**
**H(4`B)**	2.8881	2.8154	2.8217	2.7853	2.7501	2.7870	2.7642	2.7894	2.7801	2.7842	2.7525	2.7167	2.6896	2.6661	2.6699	2.6343	2.6711	2.6457	2.6729	2.6449	2.6692	2.6479	**2.88**
Nuclei	**M06-2X**																						
**H2**	9.2688	9.2022	9.2172	9.0689	9.1055	9.0698	9.1149	9.0705	9.0488	9.0684	9.0786	9.2688	9.2022	9.2172	9.0689	9.1055	9.0698	9.0583	9.0705	9.0488	9.0684	9.0786	**8.07**
**H3**	7.8638	7.9995	8.0888	8.0689	8.1821	8.0652	8.1797	8.0611	8.1877	8.0725	8.2067	7.8638	7.9995	8.0888	8.0689	8.1821	8.0652	8.1961	8.0611	8.1877	8.0725	8.2067	**7.24**
**H5**	7.6717	7.8528	7.9530	7.9157	8.0613	7.9095	8.0370	7.9030	8.0561	7.9216	8.0935	7.6717	7.8528	7.9530	7.9157	8.0613	7.9095	8.0681	7.9030	8.0561	7.9216	8.0935	**7.24**
**H6**	8.6124	8.8188	8.8989	8.8904	9.0545	8.8808	9.0649	8.8712	9.0535	8.9000	9.1025	8.6124	8.8188	8.8989	8.8904	9.0545	8.8808	9.0792	8.8712	9.0535	8.9000	9.1025	**8.07**
**H8**	4.3271	4.4158	4.4543	4.4033	4.5256	4.3941	4.5771	4.3853	4.6108	4.4129	4.6134	4.3271	4.4158	4.4543	4.4032	4.5256	4.3941	4.6342	4.3853	4.6108	4.4129	4.6134	**5.24**
**H(9A-9B)**	1.6984	1.7184	1.7356	1.7776	1.7778	1.7746	1.7854	1.7711	1.6992	1.7805	1.7994	1.6984	1.7184	1.7356	1.7776	1.7778	1.7746	1.7084	1.7711	1.6992	1.7805	1.7994	**2.135**
**H(10A-10B)**	1.2515	1.1192	1.0834	1.0230	0.9580	1.0266	0.9551	1.0298	0.9629	1.0198	0.9420	1.2515	1.1192	1.0834	1.0230	0.9580	1.0266	0.9586	1.0298	0.9629	1.0198	0.9420	**1.275**
**H(11A-11B)**	0.8898	0.9068	0.8693	0.9307	0.8776	0.9276	0.8778	0.9243	0.8547	0.9338	0.8533	0.8898	0.9068	0.8693	0.9307	0.8776	0.9276	0.8549	0.9243	0.8547	0.9338	0.8533	**1.28**
**H(12A-12B)**	1.1622	1.1268	1.1062	1.0940	1.0555	1.0940	1.0457	1.0936	1.0687	1.0943	1.0549	1.1622	1.1268	1.1062	1.0940	1.0555	1.0940	1.0672	1.0936	1.0687	1.0943	1.0549	**1.285**
**H(13A-13B)**	1.0631	1.0675	1.0305	1.0758	1.0370	1.0739	1.0244	1.0716	1.0312	1.0779	1.0207	1.0630	1.0675	1.0305	1.0759	1.0370	1.0739	1.0302	1.0716	1.0312	1.0779	1.0207	**1.29**
**H(14A-14B)**	1.2185	1.2100	1.1842	1.2033	1.1719	1.2026	1.1560	1.2015	1.1776	1.2042	1.1658	1.2185	1.2100	1.1843	1.2033	1.1719	1.2026	1.1761	1.2015	1.1776	1.2042	1.1658	**1.295**
**H(15A-15C)**	0.9426	0.9362	0.9102	0.9177	0.9033	0.9167	0.8643	0.9154	0.9057	0.9188	0.8933	0.9426	0.9362	0.9102	0.9177	0.9033	0.9167	0.9047	0.9154	0.9057	0.9188	0.8933	**0.82**
**H(1`A)**	2.6291	2.6208	2.6156	2.5808	2.5564	2.5793	2.5634	2.5779	2.5812	2.5823	2.5617	2.7832	2.7594	2.7291	2.7620	2.7345	2.7649	2.7321	2.7635	2.7351	2.7609	2.7081	**3.82**
**H(1`B)**	3.0890	3.1187	3.1545	3.1443	3.2041	3.1365	3.1858	3.1351	3.1888	3.1488	3.2018	2.7102	2.5544	2.5468	2.5983	2.5686	2.6036	2.5662	2.6022	2.5716	2.5960	2.5628	**3.78**
**H(2`A-2`B)**	1.6998	1.7388	1.7080	1.7684	1.7294	1.7646	1.7227	1.7607	1.7393	1.7722	1.7567	1.5990	1.6808	1.6378	1.7377	1.6807	1.7331	1.7376	1.7283	1.7313	1.7423	1.7569	**2.26**
**H(3`A-3`B)**	1.5990	1.6808	1.6378	1.7377	1.6807	1.7331	1.6948	1.7283	1.7313	1.7423	1.7569	1.6998	1.7388	1.7080	1.7684	1.7294	1.7646	1.7446	1.7607	1.7393	1.7722	1.7567	**2.01**
**H(4`A)**	2.7102	2.5544	2.5752	2.5983	2.5739	2.6036	2.5693	2.6022	2.5716	2.5960	2.5628	3.0890	3.1187	3.0592	3.1443	3.1979	3.1365	3.1955	3.1351	3.1888	3.1488	3.2018	**3.68**
**H(4`B)**	2.7832	2.7595	2.7552	2.7620	2.7174	2.7649	2.7380	2.7635	2.7351	2.7609	2.7081	2.6291	2.6208	2.5735	2.5808	2.5846	2.5793	2.5822	2.5779	2.5812	2.5823	2.5617	**2.88**

[a] methyl protons shifts are averaged
[b] according to [7]

Table S7. Cont. IV

Nuclei	R											S											Exp. [b]
	mpW1LYP																						
	Gas	$CHCl_3$ (PCM)	$CHCl_3$ (SMD)	DMSO (PCM)	DMSO (SMD)	CH_3OH (PCM)	CH_3OH (SMD)	C_2H_5OH (PCM)	C_2H_5OH (SMD)	H_2O (PCM)	H_2O (SMD)	Gas	$CHCl_3$ (PCM)	$CHCl_3$ (SMD)	DMSO (PCM)	DMSO (SMD)	CH_3OH (PCM)	CH_3OH (SMD)	C_2H_5OH (PCM)	C_2H_5OH (SMD)	H_2O (PCM)	H_2O (SMD)	
**H2**	8.7132	8.6044	8.6317	8.5148	8.5386	8.5221	8.5395	8.5286	8.4416	8.5072	8.5605	8.7132	8.6044	8.6317	8.5148	8.5386	8.5221	8.4445	8.5286	8.4416	8.5072	8.5605	**8.07**
**H3**	7.3358	7.4726	7.5601	7.5162	7.6306	7.5144	7.6218	7.5123	7.6266	7.5176	7.6660	7.3358	7.4726	7.5601	7.5161	7.6306	7.5144	7.6335	7.5123	7.6266	7.5176	7.6660	**7.24**
**H5**	7.1570	7.3289	7.4220	7.4117	7.5266	7.4072	7.5253	7.4023	7.5671	7.4158	7.5630	7.1570	7.3289	7.4220	7.4117	7.5266	7.4072	7.5769	7.4023	7.5671	7.4159	7.5630	**7.24**
**H6**	7.9788	8.1794	8.2570	8.2701	8.4015	8.2657	8.4281	8.2607	8.3427	8.2738	8.4692	7.9787	8.1794	8.2570	8.2701	8.4015	8.2657	8.3636	8.2607	8.3427	8.2738	8.4692	**8.07**
**H8**	4.5689	4.6562	4.6892	4.6947	4.7499	4.6938	4.8442	4.6923	4.7552	4.6950	4.7758	4.5689	4.6562	4.6892	4.6947	4.7499	4.6938	4.7696	4.6923	4.7552	4.6950	4.7758	**5.24**
**H(9A-9B)**	1.7079	1.7412	1.7392	1.7633	1.8086	1.7615	1.7837	1.7596	1.6745	1.7651	1.8186	1.7079	1.7412	1.7392	1.7633	1.8086	1.7615	1.6829	1.7596	1.6745	1.7651	1.8186	**2.135**
**H(10A-10B)**	1.3383	1.2285	1.1912	1.1619	1.0885	1.1669	1.0951	1.1716	1.1228	1.1570	1.1058	1.3383	1.2285	1.1912	1.1619	1.0885	1.1669	1.1194	1.1716	1.1228	1.1570	1.1058	**1.275**
**H(11A-11B)**	0.9852	1.0054	0.9704	1.0267	0.9875	1.0254	0.9768	1.0238	0.9713	1.0280	0.9766	0.9852	1.0054	0.9704	1.0267	0.9875	1.0254	0.9717	1.0238	0.9713	1.0280	0.9766	**1.28**
**H(12A-12B)**	1.2362	1.2234	1.1981	1.2108	1.1649	1.2122	1.1641	1.2132	1.1858	1.2095	1.1704	1.2362	1.2234	1.1982	1.2108	1.1649	1.2122	1.1840	1.2132	1.1858	1.2095	1.1704	**1.285**
**H(13A-13B)**	1.1485	1.1528	1.1235	1.1639	1.1264	1.1632	1.1153	1.1624	1.1235	1.1644	1.1201	1.1485	1.1528	1.1235	1.1639	1.1264	1.1632	1.1231	1.1624	1.1235	1.1644	1.1201	**1.29**
**H(14A-14B)**	1.3106	1.3088	1.2887	1.3083	1.2741	1.3088	1.2634	1.3089	1.2855	1.3079	1.2742	1.3106	1.3088	1.2887	1.3084	1.2741	1.3088	1.2843	1.3089	1.2855	1.3079	1.2742	**1.295**
**H(15A-15C)**	0.9698	0.9659	0.9445	0.9673	0.9358	0.9675	0.9150	0.9674	0.9396	0.9671	0.9320	0.9698	0.9659	0.9445	0.9673	0.9358	0.9675	0.9386	0.9674	0.9396	0.9671	0.9321	**0.82**
**H(1`A)**	2.7315	2.7295	2.7063	2.7196	2.6695	2.7208	2.6895	2.7216	2.6841	2.7183	2.6574	2.8607	2.8475	2.8460	2.8321	2.8015	2.8329	2.8065	2.8335	2.8107	2.8314	2.7667	**3.82**
**H(1`B)**	3.2045	3.2461	3.2724	3.2786	3.3290	3.2769	3.3237	3.2749	3.3102	3.2800	3.3283	2.8543	2.6888	2.6649	2.6700	2.6829	2.6701	2.6742	2.6703	2.6760	2.6703	2.6307	**3.78**
**H(2`A-2`B)**	1.7343	1.7767	1.7381	1.8083	1.7550	1.8067	1.7555	1.8048	1.7653	1.8097	1.7796	1.5860	1.6743	1.6419	1.7285	1.6712	1.7252	1.7197	1.7218	1.7151	1.7316	1.7246	**2.26**
**H(3`A-3`B)**	1.5860	1.6743	1.6419	1.7285	1.6712	1.7252	1.6822	1.7218	1.7151	1.7315	1.7246	1.7343	1.7767	1.7381	1.8083	1.7550	1.8067	1.7692	1.8048	1.7653	1.8097	1.7796	**2.01**
**H(4`A)**	2.8543	2.6888	2.6649	2.6700	2.6829	2.6701	2.6676	2.6703	2.6760	2.6703	2.6307	3.2045	3.2461	3.2724	3.2786	3.3290	3.2769	3.3142	3.2749	3.3101	3.2801	3.3283	**3.68**
**H(4`B)**	2.8607	2.8475	2.8460	2.8321	2.8015	2.8329	2.8082	2.8335	2.8107	2.8314	2.7667	2.7315	2.7295	2.7063	2.7196	2.6695	2.7208	2.6832	2.7216	2.6841	2.7183	2.6574	**2.88**
Nuclei	mpW1PW91																						
**H2**	8.7696	8.7001	8.6845	8.5654	8.5923	8.5734	8.5921	8.5809	8.6337	8.5576	8.5671	8.7696	8.7001	8.6845	8.5654	8.5923	8.5734	8.6194	8.5809	8.6337	8.5576	8.5671	**8.07**
**H3**	7.3657	7.5081	7.6005	7.5492	7.6728	7.5478	7.6614	7.5465	7.6850	7.5506	7.7083	7.3657	7.5081	7.6005	7.5492	7.6728	7.5478	7.6927	7.5465	7.6850	7.5506	7.7083	**7.24**
**H5**	7.1837	7.3637	7.4562	7.4371	7.5635	7.4329	7.5576	7.4287	7.5737	7.4412	7.5970	7.1837	7.3637	7.4562	7.4371	7.5635	7.4329	7.5827	7.4287	7.5737	7.4412	7.5970	**7.24**
**H6**	8.0406	8.2555	8.3153	8.3466	8.4607	8.3417	8.5044	8.3364	8.5126	8.3508	8.5559	8.0406	8.2555	8.3153	8.3466	8.4607	8.3417	8.5364	8.3364	8.5126	8.3508	8.5559	**8.07**
**H8**	4.5694	4.6538	4.6890	4.6897	4.7465	4.6894	4.8344	4.6888	4.7931	4.6894	4.7815	4.5694	4.6538	4.6890	4.6897	4.7465	4.6894	4.8037	4.6888	4.7931	4.6894	4.7815	**5.24**
**H(9A-9B)**	1.7346	1.7854	1.7537	1.7828	1.8157	1.7805	1.8030	1.7786	1.8015	1.7856	1.8016	1.7346	1.7854	1.7537	1.7828	1.8157	1.7805	1.8054	1.7786	1.8015	1.7856	1.8016	**2.135**
**H(10A-10B)**	1.3123	1.1970	1.1608	1.1205	1.0617	1.1256	1.0567	1.1307	1.0785	1.1161	1.0500	1.3123	1.1970	1.1608	1.1205	1.0617	1.1256	1.0712	1.1307	1.0785	1.1161	1.0500	**1.275**
**H(11A-11B)**	0.9365	0.9609	0.9176	0.9720	0.9379	0.9708	0.9250	0.9696	0.9306	0.9736	0.9133	0.9365	0.9609	0.9175	0.9720	0.9379	0.9708	0.9279	0.9696	0.9306	0.9736	0.9133	**1.28**
**H(12A-12B)**	1.1889	1.1716	1.1470	1.1534	1.1151	1.1551	1.1081	1.1567	1.1229	1.1521	1.1141	1.1889	1.1716	1.1470	1.1534	1.1151	1.1550	1.1200	1.1567	1.1229	1.1521	1.1142	**1.285**
**H(13A-13B)**	1.0963	1.0988	1.0675	1.1038	1.0704	1.1035	1.0557	1.1033	1.0659	1.1045	1.0598	1.0963	1.0988	1.0675	1.1038	1.0704	1.1035	1.0644	1.1033	1.0659	1.1045	1.0598	**1.29**
**H(14A-14B)**	1.2564	1.2514	1.2304	1.2465	1.2160	1.2472	1.2022	1.2480	1.2196	1.2462	1.2150	1.2564	1.2514	1.2304	1.2465	1.2160	1.2472	1.2186	1.2480	1.2196	1.2462	1.2150	**1.295**
**H(15A-15C)**	0.9153	0.9114	0.8884	0.9082	0.8801	0.9087	0.8573	0.9092	0.8808	0.9079	0.8754	0.9153	0.9114	0.8884	0.9082	0.8801	0.9087	0.8796	0.9092	0.8807	0.9079	0.8754	**0.82**
**H(1`A)**	2.6736	2.6743	2.6528	2.6560	2.6120	2.6579	2.6291	2.6598	2.6283	2.6543	2.5953	2.7998	2.7854	2.7780	2.7732	2.7382	2.7741	2.7484	2.7752	2.7481	2.7729	2.7184	**3.82**
**H(1`B)**	3.1778	3.2179	3.2347	3.2407	3.2872	3.2394	3.2798	3.2381	3.2798	3.2421	3.2811	2.8613	2.6680	2.6711	2.6489	2.6689	2.6487	2.6131	2.6493	2.6123	2.6503	2.6182	**3.78**
**H(2`A-2`B)**	1.7019	1.7403	1.7077	1.7675	1.7244	1.7661	1.7193	1.7648	1.7333	1.7692	1.7523	1.5594	1.6374	1.6057	1.6902	1.6381	1.6877	1.6683	1.6853	1.6601	1.6928	1.7143	**2.26**
**H(3`A-3`B)**	1.5594	1.6374	1.6056	1.6902	1.6381	1.6877	1.6470	1.6853	1.6601	1.6928	1.7143	1.7019	1.7403	1.7077	1.7675	1.7244	1.7661	1.7374	1.7648	1.7333	1.7692	1.7523	**2.01**
**H(4`A)**	2.8613	2.6680	2.6711	2.6489	2.6689	2.6487	2.6448	2.6493	2.6123	2.6503	2.6182	3.1778	3.2180	3.2347	3.2407	3.2872	3.2394	3.2840	3.2381	3.2797	3.2421	3.2811	**3.68**
**H(4`B)**	2.7998	2.7854	2.7779	2.7732	2.7382	2.7741	2.7542	2.7752	2.7481	2.7729	2.7184	2.6736	2.6743	2.6528	2.6560	2.6120	2.6579	2.6273	2.6598	2.6283	2.6543	2.5953	**2.88**
Nuclei	O3LYP																						
**H2**	8.5108	8.3361	8.3595	8.2440	8.2909	8.2584	8.3327	8.2630	8.3236	8.2348	8.3200	8.5108	8.3361	8.3595	8.2440	8.2909	8.2584	8.3327	8.2631	8.3236	8.2348	8.3200	**8.07**
**H3**	7.2901	7.3999	7.4958	7.4500	7.5610	7.4478	7.5814	7.4413	7.5741	7.4515	7.5921	7.2901	7.3998	7.4958	7.4500	7.5610	7.4478	7.5814	7.4413	7.5741	7.4515	7.5921	**7.24**
**H5**	7.1348	7.3190	7.4090	7.3885	7.5118	7.3860	7.5264	7.3846	7.5163	7.3911	7.5353	7.1348	7.3190	7.4090	7.3885	7.5118	7.3860	7.5265	7.3847	7.5163	7.3911	7.5353	**7.24**
**H6**	7.8977	8.0346	8.1139	8.1024	8.2544	8.1012	8.2954	8.1047	8.2732	8.1051	8.3171	7.8977	8.0346	8.1139	8.1024	8.2544	8.1012	8.2954	8.1046	8.2732	8.1051	8.3171	**8.07**
**H8**	4.7575	4.7780	4.7992	4.7896	4.8754	4.7911	4.8898	4.7935	4.8736	4.7890	4.8741	4.7575	4.7780	4.7992	4.7896	4.8754	4.7911	4.8898	4.7935	4.8736	4.7890	4.8741	**5.24**
**H(9A-9B)**	1.6371	1.6211	1.6327	1.6403	1.6588	1.6418	1.6900	1.6455	1.6821	1.6412	1.7050	1.6370	1.6211	1.6327	1.6403	1.6588	1.6418	1.6900	1.6455	1.6821	1.6412	1.7050	**2.135**
**H(10A-10B)**	1.2764	1.1837	1.1462	1.1350	1.0686	1.1374	1.0851	1.1414	1.0873	1.1317	1.0870	1.2764	1.1838	1.1462	1.1350	1.0686	1.1374	1.0851	1.1415	1.0873	1.1317	1.0870	**1.275**
**H(11A-11B)**	0.9651	0.9764	0.9462	1.0039	0.9600	1.0018	0.9590	0.9989	0.9581	1.0054	0.9580	0.9651	0.9764	0.9462	1.0038	0.9600	1.0018	0.9590	0.9990	0.9581	1.0054	0.9580	**1.28**
**H(12A-12B)**	1.2153	1.2073	1.1814	1.2043	1.1587	1.2042	1.1633	1.2026	1.1645	1.2036	1.1663	1.2153	1.2073	1.1814	1.2043	1.1587	1.2042	1.1633	1.2027	1.1645	1.2036	1.1663	**1.285**
**H(13A-13B)**	1.1340	1.1352	1.1076	1.1511	1.1115	1.1500	1.1096	1.1472	1.1095	1.1517	1.1112	1.1340	1.1352	1.1076	1.1511	1.1115	1.1500	1.1096	1.1472	1.1095	1.1517	1.1112	**1.29**
**H(14A-14B)**	1.3176	1.3196	1.2996	1.3233	1.2898	1.3230	1.2919	1.3219	1.2925	1.3230	1.2913	1.3176	1.3196	1.2996	1.3233	1.2898	1.3230	1.2919	1.3219	1.2925	1.3230	1.2913	**1.295**
**H(15A-15C)**	0.9493	0.9446	0.9232	0.9489	0.9186	0.9487	0.9178	0.9476	0.9181	0.9487	0.9157	0.9493	0.9447	0.9232	0.9489	0.9186	0.9487	0.9178	0.9476	0.9181	0.9487	0.9157	**0.82**
**H(1`A)**	2.7774	2.7637	2.7396	2.7555	2.7041	2.7568	2.7147	2.7547	2.7154	2.7544	2.6826	2.9128	2.8942	2.8872	2.8696	2.8347	2.8723	2.8401	2.8738	2.8441	2.8669	2.8018	**3.82**
**H(1`B)**	3.2635	3.2960	3.3296	3.3276	3.3894	3.3259	3.3790	3.3257	3.3746	3.3294	3.3772	2.8798	2.7677	2.7543	2.7567	2.7532	2.7561	2.7362	2.7551	2.7389	2.7567	2.7349	**3.78**
**H(2`A-2`B)**	1.7323	1.7680	1.7331	1.7990	1.7516	1.7980	1.7625	1.7931	1.7583	1.8000	1.7794	1.6117	1.6893	1.6613	1.7464	1.6931	1.7421	1.7145	1.7328	1.7097	1.7496	1.7527	**2.26**
**H(3`A-3`B)**	1.6117	1.6893	1.6613	1.7464	1.6931	1.7421	1.7145	1.7328	1.7097	1.7496	1.7527	1.7323	1.7680	1.7331	1.7990	1.7516	1.7980	1.7625	1.7932	1.7583	1.8000	1.7794	**2.01**
**H(4`A)**	2.8798	2.7677	2.7543	2.7567	2.7532	2.7561	2.7362	2.7548	2.7389	2.7567	2.7349	3.2635	3.2960	3.3296	3.3276	3.3894	3.3259	3.3790	3.3256	3.3746	3.3294	3.3771	**3.68**
**H(4`B)**	2.9128	2.8942	2.8872	2.8696	2.8347	2.8723	2.8401	2.8743	2.8441	2.8669	2.8018	2.7774	2.7637	2.7396	2.7555	2.7041	2.7568	2.7147	2.7550	2.7154	2.7544	2.6826	**2.88**
Nuclei	OLYP																						
**H2**	8.3248	8.1648	8.2538	8.0803	8.0015	8.0861	8.1354	8.0916	8.0872	8.0741	8.2124	8.3247	8.1648	8.2538	8.0803	8.0015	8.0861	8.0988	8.0916	8.0872	8.0741	8.2124	**8.07**
**H3**	7.2142	7.3287	7.4254	7.3644	7.4628	7.3630	7.4767	7.3615	7.4811	7.3651	7.5155	7.2142	7.3287	7.4254	7.3644	7.4628	7.3630	7.4870	7.3615	7.4811	7.3651	7.5155	**7.24**
**H5**	7.0994	7.2395	7.3358	7.3006	7.4044	7.2973	7.4189	7.2939	7.4181	7.3033	7.4518	7.0995	7.2395	7.3358	7.3006	7.4044	7.2973	7.4298	7.2939	7.4181	7.3033	7.4518	**7.24**
**H6**	7.7912	7.9146	8.0145	7.9898	8.0895	7.9855	8.1272	7.9810	8.1012	7.9933	8.2037	7.7912	7.9146	8.0145	7.9898	8.0895	7.9855	8.1269	7.9810	8.1012	7.9933	8.2037	**8.07**
**H8**	4.9255	4.9263	4.9478	4.9302	4.9556	4.9304	5.0427	4.9304	4.9578	4.9293	5.0069	4.9256	4.9263	4.9478	4.9302	4.9556	4.9304	4.9756	4.9304	4.9578	4.9293	5.0069	**5.24**
**H(9A-9B)**	1.6378	1.6482	1.6929	1.6836	1.6528	1.6811	1.7021	1.6786	1.6857	1.6856	1.7602	1.6378	1.6482	1.6929	1.6836	1.6528	1.6811	1.6907	1.6786	1.6857	1.6856	1.7602	**2.135**
**H(10A-10B)**	1.2986	1.2200	1.1776	1.1729	1.1263	1.1764	1.1192	1.1797	1.1387	1.1691	1.1219	1.2986	1.2200	1.1776	1.1729	1.1263	1.1764	1.1374	1.1797	1.1387	1.1691	1.1219	**1.275**
**H(11A-11B)**	0.9956	1.0127	0.9857	1.0388	1.0080	1.0371	0.9919	1.0354	1.0026	1.0400	0.9927	0.9956	1.0127	0.9857	1.0388	1.0080	1.0371	1.0022	1.0354	1.0026	1.0400	0.9927	**1.28**
**H(12A-12B)**	1.2490	1.2466	1.2155	1.2396	1.2113	1.2406	1.1975	1.2413	1.2137	1.2384	1.1999	1.2490	1.2466	1.2155	1.2396	1.2113	1.2406	1.2127	1.2413	1.2137	1.2384	1.1999	**1.285**
**H(13A-13B)**	1.1676	1.1730	1.1449	1.1854	1.1577	1.1848	1.1407	1.1840	1.1534	1.1857	1.1449	1.1676	1.1730	1.1449	1.1854	1.1577	1.1848	1.1531	1.1840	1.1534	1.1857	1.1449	**1.29**
**H(14A-14B)**	1.3596	1.3646	1.3413	1.3660	1.3422	1.3663	1.3251	1.3664	1.3428	1.3653	1.3324	1.3596	1.3646	1.3413	1.3660	1.3422	1.3663	1.3422	1.3664	1.3428	1.3653	1.3324	**1.295**
**H(15A-15C)**	0.9612	0.9582	0.9357	0.9607	0.9364	0.9607	0.9121	0.9607	0.9353	0.9601	0.9259	0.9612	0.9582	0.9357	0.9607	0.9364	0.9607	0.9351	0.9607	0.9353	0.9601	0.9259	**0.82**
**H(1`A)**	2.8378	2.8260	2.8047	2.8160	2.7664	2.8169	2.7846	2.8177	2.7763	2.8147	2.7410	2.9774	2.9588	2.9520	2.9351	2.9032	2.9372	2.8993	2.9391	2.9044	2.9324	2.7410	**3.82**
**H(1`B)**	3.3555	3.3841	3.4251	3.4191	3.4774	3.4169	3.4686	3.4147	3.4614	3.4207	3.4677	2.9631	2.8588	2.8417	2.8491	2.8809	2.8491	2.8582	2.8492	2.8578	2.8488	3.4677	**3.78**
**H(2`A-2`B)**	1.7671	1.8012	1.7690	1.8260	1.7833	1.8247	1.7762	1.8233	1.7903	1.8269	1.8151	1.6586	1.7375	1.7010	1.7856	1.7718	1.7826	1.7804	1.7795	1.7751	1.7880	1.7849	**2.26**
**H(3`A-3`B)**	1.6586	1.7375	1.7010	1.7856	1.7718	1.7826	1.7419	1.7795	1.7751	1.7880	1.7849	1.7671	1.8012	1.7690	1.8260	1.7833	1.8247	1.7950	1.8233	1.7903	1.8269	1.8151	**2.01**
**H(4`A)**	2.9631	2.8588	2.8417	2.8491	2.8809	2.8491	2.8573	2.8492	2.8578	2.8488	2.8263	3.3555	3.3841	3.4251	3.4191	3.4774	3.4169	3.4642	3.4147	3.4614	3.4207	2.8263	**3.68**
**H(4`B)**	2.9774	2.9588	2.9520	2.9351	2.9032	2.9372	2.9052	2.9391	2.9044	2.9324	2.8589	2.8378	2.8260	2.8047	2.8160	2.7664	2.8169	2.7745	2.8177	2.7762	2.8147	2.8589	**2.88**

[a] methyl protons shifts are averaged
[b] according to [7]

Table S7. Cont. V

Nuclei	R											S											Exp. [b]
	OPBE																						
	Gas	$CHCl_3$ (PCM)	$CHCl_3$ (SMD)	DMSO (PCM)	DMSO (SMD)	CH_3OH (PCM)	CH_3OH (SMD)	C_2H_5OH (PCM)	C_2H_5OH (SMD)	H_2O (PCM)	H_2O (SMD)	Gas	$CHCl_3$ (PCM)	$CHCl_3$ (SMD)	DMSO (PCM)	DMSO (SMD)	CH_3OH (PCM)	CH_3OH (SMD)	C_2H_5OH (PCM)	C_2H_5OH (SMD)	H_2O (PCM)	H_2O (SMD)	
**H2**	8.3493	8.2392	8.2718	8.1296	8.2064	8.1369	8.1837	8.1437	8.2434	8.1222	8.2247	8.3493	8.2392	8.2718	8.1296	8.2064	8.1369	8.2501	8.1437	8.2434	8.1221	8.2247	**8.07**
**H3**	7.2200	7.3458	7.4385	7.3939	7.5142	7.3929	7.5066	7.3911	7.5205	7.3941	7.5396	7.2200	7.3458	7.4385	7.3939	7.5142	7.3929	7.5275	7.3911	7.5205	7.3941	7.5396	**7.24**
**H5**	7.0981	7.2640	7.3492	7.3273	7.4517	7.3243	7.4461	7.3206	7.4585	7.3294	7.4808	7.0981	7.2640	7.3492	7.3273	7.4517	7.3243	7.4685	7.3206	7.4585	7.3294	7.4808	**7.24**
**H6**	7.8722	8.0145	8.0860	8.0625	8.2039	8.0600	8.1936	8.0566	8.2311	8.0641	8.2568	7.8722	8.0145	8.0860	8.0625	8.2039	8.0599	8.2512	8.0566	8.2311	8.0641	8.2568	**8.07**
**H8**	4.9240	4.9392	4.9500	4.9341	5.0091	4.9354	5.0372	4.9359	5.0098	4.9321	5.0098	4.9240	4.9392	4.9500	4.9341	5.0091	4.9354	5.0241	4.9359	5.0097	4.9321	5.0098	**5.24**
**H(9A-9B)**	1.6489	1.6720	1.6887	1.6820	1.6995	1.6801	1.6958	1.6778	1.7303	1.6833	1.7437	1.6489	1.6720	1.6887	1.6820	1.6995	1.6801	1.7362	1.6778	1.7303	1.6833	1.7437	**2.135**
**H(10A-10B)**	1.2436	1.1591	1.1199	1.1143	1.0434	1.1182	1.0550	1.1212	1.0658	1.1099	1.0670	1.2436	1.1591	1.1199	1.1143	1.0434	1.1182	1.0628	1.1212	1.0658	1.1098	1.0670	**1.275**
**H(11A-11B)**	0.9363	0.9558	0.9228	0.9816	0.9369	0.9805	0.9297	0.9787	0.9321	0.9819	0.9300	0.9363	0.9558	0.9228	0.9816	0.9369	0.9805	0.9322	0.9787	0.9321	0.9819	0.9300	**1.28**
**H(12A-12B)**	1.1882	1.1824	1.1506	1.1782	1.1284	1.1796	1.1296	1.1801	1.1335	1.1761	1.1363	1.1882	1.1823	1.1506	1.1782	1.1284	1.1796	1.1322	1.1801	1.1335	1.1761	1.1363	**1.285**
**H(13A-13B)**	1.1023	1.1082	1.0754	1.1204	1.0779	1.1204	1.0684	1.1196	1.0740	1.1198	1.0746	1.1023	1.1082	1.0754	1.1204	1.0779	1.1204	1.0738	1.1196	1.0740	1.1198	1.0746	**1.29**
**H(14A-14B)**	1.2966	1.2986	1.2725	1.2993	1.2608	1.3001	1.2515	1.3001	1.2628	1.2977	1.2615	1.2966	1.2986	1.2725	1.2993	1.2608	1.3001	1.2621	1.3001	1.2628	1.2977	1.2615	**1.295**
**H(15A-15C)**	0.8937	0.8918	0.8646	0.8928	0.8584	0.8935	0.8385	0.8934	0.8568	0.8914	0.8546	0.8937	0.8918	0.8646	0.8928	0.8585	0.8935	0.8564	0.8934	0.8568	0.8914	0.8546	**0.82**
**H(1\`A)**	2.7613	2.7514	2.7263	2.7415	2.6913	2.7426	2.7077	2.7431	2.6992	2.7396	2.6618	2.9015	2.8885	2.8777	2.8595	2.8206	2.8623	2.8281	2.8642	2.8304	2.8561	2.7954	**3.82**
**H(1\`B)**	3.3195	3.3467	3.3748	3.3738	3.4263	3.3724	3.4124	3.3702	3.4131	3.3745	3.4125	2.9527	2.8309	2.8138	2.8147	2.8015	2.8153	2.7845	2.8154	2.7866	2.8135	2.7913	**3.78**
**H(2\`A-2\`B)**	1.7220	1.7581	1.7210	1.7831	1.7379	1.7824	1.7296	1.7809	1.7436	1.7831	1.7663	1.6145	1.6835	1.6484	1.7372	1.6799	1.7345	1.6995	1.7310	1.6937	1.7390	1.7470	**2.26**
**H(3\`A-3\`B)**	1.6145	1.6835	1.6484	1.7372	1.6799	1.7345	1.6902	1.7310	1.6937	1.7390	1.7470	1.7220	1.7581	1.7210	1.7831	1.7379	1.7824	1.7469	1.7809	1.7436	1.7831	1.7663	**2.01**
**H(4\`A)**	2.9527	2.8309	2.8138	2.8147	2.8015	2.8153	2.8102	2.8154	2.7866	2.8135	2.7913	3.3195	3.3467	3.3748	3.3738	3.4263	3.3724	3.4169	3.3702	3.4130	3.3745	3.4125	**3.68**
**H(4\`B)**	2.9015	2.8885	2.8777	2.8595	2.8206	2.8623	2.8268	2.8642	2.8304	2.8561	2.7954	2.7613	2.7514	2.7263	2.7414	2.6913	2.7426	2.6981	2.7431	2.6992	2.7396	2.6618	**2.88**
Nuclei	OPW91																						
**H2**	8.3512	8.2401	8.2730	8.1313	8.0596	8.1378	8.1844	8.1442	8.2442	8.1235	8.2281	8.3512	8.2401	8.2730	8.1313	8.0596	8.1378	8.2518	8.1442	8.2442	8.1235	8.2281	**8.07**
**H3**	7.2224	7.3473	7.4399	7.3959	7.4884	7.3942	7.5076	7.3922	7.5214	7.3958	7.5404	7.2224	7.3473	7.4399	7.3959	7.4883	7.3942	7.5285	7.3922	7.5214	7.3958	7.5404	**7.24**
**H5**	7.1011	7.2662	7.3514	7.3297	7.4376	7.3260	7.4475	7.3221	7.4602	7.3314	7.4821	7.1011	7.2662	7.3514	7.3297	7.4377	7.3260	7.4703	7.3221	7.4602	7.3314	7.4821	**7.24**
**H6**	7.8704	8.0137	8.0851	8.0634	8.1417	8.0600	8.1939	8.0564	8.2309	8.0647	8.2591	7.8704	8.0137	8.0851	8.0634	8.1417	8.0600	8.2515	8.0564	8.2309	8.0647	8.2592	**8.07**
**H8**	4.9226	4.9378	4.9488	4.9339	4.9572	4.9344	5.0369	4.9346	5.0088	4.9316	5.0093	4.9226	4.9378	4.9488	4.9339	4.9572	4.9344	5.0235	4.9346	5.0088	4.9316	5.0093	**5.24**
**H(9A-9B)**	1.6463	1.6700	1.6867	1.6811	1.6483	1.6785	1.6945	1.6758	1.7291	1.6821	1.7437	1.6463	1.6700	1.6867	1.6811	1.6484	1.6785	1.7353	1.6758	1.7291	1.6821	1.7437	**2.135**
**H(10A-10B)**	1.2459	1.1611	1.1220	1.1172	1.0498	1.1204	1.0574	1.1233	1.0679	1.1124	1.0693	1.2459	1.1611	1.1220	1.1172	1.0498	1.1204	1.0651	1.1232	1.0679	1.1124	1.0693	**1.275**
**H(11A-11B)**	0.9378	0.9575	0.9248	0.9841	0.9384	0.9824	0.9318	0.9804	0.9342	0.9842	0.9326	0.9378	0.9575	0.9248	0.9841	0.9384	0.9824	0.9344	0.9804	0.9342	0.9842	0.9326	**1.28**
**H(12A-12B)**	1.1896	1.1838	1.1522	1.1805	1.1400	1.1812	1.1315	1.1815	1.1352	1.1782	1.1380	1.1896	1.1838	1.1522	1.1805	1.1400	1.1812	1.1339	1.1815	1.1352	1.1782	1.1380	**1.285**
**H(13A-13B)**	1.1039	1.1099	1.0773	1.1230	1.0830	1.1223	1.0707	1.1213	1.0761	1.1222	1.0769	1.1039	1.1099	1.0773	1.1231	1.0830	1.1223	1.0760	1.1213	1.0761	1.1222	1.0769	**1.29**
**H(14A-14B)**	1.2971	1.2993	1.2734	1.3008	1.2681	1.3010	1.2527	1.3008	1.2638	1.2990	1.2626	1.2971	1.2993	1.2734	1.3008	1.2681	1.3009	1.2632	1.3008	1.2638	1.2990	1.2626	**1.295**
**H(15A-15C)**	0.8951	0.8933	0.8664	0.8951	0.8635	0.8952	0.8404	0.8948	0.8586	0.8935	0.8565	0.8951	0.8933	0.8664	0.8951	0.8635	0.8951	0.8584	0.8948	0.8586	0.8935	0.8565	**0.82**
**H(1\`A)**	2.7654	2.7551	2.7299	2.7457	2.6947	2.7462	2.7115	2.7466	2.7028	2.7435	2.6658	2.9049	2.8918	2.8810	2.8636	2.8325	2.8657	2.8312	2.8674	2.8336	2.8598	2.7980	**3.82**
**H(1\`B)**	3.3164	3.3442	3.3724	3.3721	3.4175	3.3700	3.4105	3.3676	3.4109	3.3725	3.4107	2.9478	2.8271	2.8104	2.8121	2.8346	2.8120	2.7814	2.8119	2.7837	2.8106	2.7879	**3.78**
**H(2\`A-2\`B)**	1.7220	1.7583	1.7215	1.7843	1.7323	1.7828	1.7304	1.7811	1.7441	1.7841	1.7672	1.6145	1.6839	1.6490	1.7384	1.7103	1.7351	1.6998	1.7315	1.6941	1.7399	1.7461	**2.26**
**H(3\`A-3\`B)**	1.6145	1.6839	1.6490	1.7384	1.7103	1.7351	1.6909	1.7314	1.6941	1.7399	1.7461	1.7220	1.7583	1.7215	1.7843	1.7323	1.7828	1.7476	1.7811	1.7441	1.7841	1.7672	**2.01**
**H(4\`A)**	2.9478	2.8271	2.8104	2.8121	2.8346	2.8120	2.8076	2.8119	2.7837	2.8106	2.7879	3.3164	3.3442	3.3724	3.3721	3.4174	3.3700	3.4148	3.3676	3.4109	3.3725	3.4107	**3.68**
**H(4\`B)**	2.9049	2.8918	2.8810	2.8636	2.8325	2.8657	2.8303	2.8674	2.8336	2.8598	2.7980	2.7654	2.7551	2.7299	2.7457	2.6947	2.7462	2.7018	2.7466	2.7028	2.7435	2.6658	**2.88**
Nuclei	PBE																						
**H2**	8.5376	8.4278	8.4550	8.3490	8.3529	8.3550	8.3808	8.3612	8.3954	8.3435	8.3588	8.5376	8.4278	8.4550	8.3490	8.3529	8.3550	8.3913	8.3612	8.3954	8.3435	8.3588	**8.07**
**H3**	7.2081	7.3473	7.4380	7.3857	7.5089	7.3840	7.5023	7.3828	7.5222	7.3879	7.5433	7.2081	7.3473	7.4380	7.3857	7.5089	7.3840	7.5303	7.3828	7.5222	7.3879	7.5433	**7.24**
**H5**	7.0242	7.1954	7.2915	7.2732	7.3971	7.2688	7.3961	7.2649	7.4067	7.2779	7.4283	7.0242	7.1954	7.2915	7.2732	7.3971	7.2688	7.4167	7.2649	7.4067	7.2779	7.4283	**7.24**
**H6**	7.9566	8.1463	8.2083	8.2153	8.3301	8.2122	8.3630	8.2091	8.3747	8.2185	8.3849	7.9566	8.1464	8.2083	8.2153	8.3301	8.2122	8.3930	8.2091	8.3747	8.2186	8.3849	**8.07**
**H8**	4.9984	5.0614	5.0715	5.0695	5.1008	5.0707	5.1869	5.0719	5.1406	5.0683	5.1182	4.9984	5.0614	5.0715	5.0695	5.1008	5.0707	5.1510	5.0719	5.1406	5.0683	5.1182	**5.24**
**H(9A-9B)**	1.9217	1.9412	1.9445	1.9561	2.0018	1.9536	1.9803	1.9521	1.9828	1.9596	1.9803	1.9217	1.9412	1.9445	1.9561	2.0018	1.9536	1.9857	1.9521	1.9828	1.9596	1.9803	**2.135**
**H(10A-10B)**	1.3972	1.2918	1.2578	1.2275	1.1703	1.2317	1.1719	1.2364	1.1848	1.2242	1.1751	1.3972	1.2918	1.2578	1.2275	1.1703	1.2317	1.1808	1.2364	1.1848	1.2242	1.1751	**1.275**
**H(11A-11B)**	1.0474	1.0631	1.0274	1.0788	1.0455	1.0774	1.0352	1.0766	1.0367	1.0809	1.0228	1.0474	1.0631	1.0274	1.0788	1.0455	1.0774	1.0356	1.0766	1.0367	1.0809	1.0227	**1.28**
**H(12A-12B)**	1.2874	1.2759	1.2506	1.2583	1.2203	1.2597	1.2171	1.2617	1.2287	1.2576	1.2244	1.2874	1.2759	1.2506	1.2583	1.2203	1.2597	1.2270	1.2617	1.2287	1.2576	1.2244	**1.285**
**H(13A-13B)**	1.1952	1.1986	1.1680	1.2031	1.1707	1.2026	1.1589	1.2028	1.1666	1.2042	1.1611	1.1952	1.1987	1.1680	1.2031	1.1707	1.2026	1.1659	1.2028	1.1666	1.2042	1.1611	**1.29**
**H(14A-14B)**	1.3524	1.3519	1.3307	1.3450	1.3166	1.3456	1.3046	1.3466	1.3208	1.3451	1.3164	1.3524	1.3519	1.3307	1.3450	1.3166	1.3456	1.3200	1.3466	1.3208	1.3451	1.3164	**1.295**
**H(15A-15C)**	0.9401	0.9382	0.9153	0.9328	0.9065	0.9333	0.8855	0.9342	0.9075	0.9331	0.9028	0.9401	0.9382	0.9153	0.9328	0.9065	0.9333	0.9067	0.9342	0.9075	0.9331	0.9028	**0.82**
**H(1\`A)**	2.8023	2.8053	2.7873	2.7873	2.7471	2.7887	2.7633	2.7907	2.7585	2.7866	2.7199	2.9200	2.9195	2.9111	2.9036	2.8805	2.9046	2.8809	2.9061	2.8833	2.9034	2.8420	**3.82**
**H(1\`B)**	3.4002	3.4272	3.4462	3.4428	3.4914	3.4419	3.4838	3.4415	3.4808	3.4441	3.4777	3.0638	2.8750	2.8551	2.8274	2.8470	2.8285	2.7937	2.8307	2.7956	2.8277	2.7952	**3.78**
**H(2\`A-2\`B)**	1.8038	1.8449	1.8095	1.8654	1.8238	1.8642	1.8189	1.8635	1.8337	1.8673	1.8500	1.6481	1.7395	1.7013	1.7787	1.7332	1.7766	1.7612	1.7748	1.7551	1.7814	1.8066	**2.26**
**H(3\`A-3\`B)**	1.6481	1.7395	1.7013	1.7787	1.7332	1.7766	1.7379	1.7748	1.7551	1.7814	1.8066	1.8038	1.8449	1.8095	1.8654	1.8238	1.8642	1.8380	1.8635	1.8337	1.8673	1.8500	**2.01**
**H(4\`A)**	3.0638	2.8750	2.8551	2.8274	2.8470	2.8285	2.8221	2.8307	2.7956	2.8277	2.7952	3.4002	3.4272	3.4462	3.4428	3.4914	3.4419	3.4848	3.4415	3.4808	3.4441	3.4777	**3.68**
**H(4\`B)**	2.9200	2.9195	2.9111	2.9036	2.8805	2.9046	2.8870	2.9061	2.8833	2.9034	2.8420	2.8023	2.8053	2.7873	2.7873	2.7471	2.7887	2.7570	2.7907	2.7585	2.7866	2.7199	**2.88**
Nuclei	PBE0																						
**H2**	8.7681	8.6983	8.6797	8.5826	8.5885	8.5922	8.6077	8.6023	8.6333	8.5729	8.5672	8.7681	8.6983	8.6797	8.5826	8.5885	8.5922	8.6260	8.6023	8.6333	8.5729	8.5672	**8.07**
**H3**	7.3567	7.4986	7.5920	7.5425	7.6631	7.5408	7.6566	7.5389	7.6771	7.5437	7.7002	7.3567	7.4986	7.5920	7.5425	7.6631	7.5408	7.6879	7.5389	7.6771	7.5437	7.7002	**7.24**
**H5**	7.1816	7.3657	7.4557	7.4371	7.5642	7.4329	7.5602	7.4286	7.5753	7.4408	7.5952	7.1816	7.3657	7.4557	7.4371	7.5642	7.4328	7.5874	7.4286	7.5753	7.4408	7.5952	**7.24**
**H6**	8.0476	8.2582	8.3194	8.3440	8.4588	8.3395	8.5044	8.3347	8.5103	8.3473	8.5492	8.0476	8.2582	8.3194	8.3440	8.4588	8.3395	8.5351	8.3347	8.5103	8.3473	8.5492	**8.07**
**H8**	4.5684	4.6522	4.6899	4.6823	4.7431	4.6823	4.8286	4.6816	4.7923	4.6811	4.7822	4.5684	4.6522	4.6899	4.6823	4.7431	4.6823	4.8062	4.6816	4.7923	4.6811	4.7822	**5.24**
**H(9A-9B)**	1.7516	1.7990	1.7632	1.8043	1.8225	1.8030	1.8243	1.8028	1.8084	1.8062	1.8103	1.7516	1.7990	1.7632	1.8043	1.8225	1.8030	1.8158	1.8028	1.8084	1.8062	1.8103	**2.135**
**H(10A-10B)**	1.3175	1.2021	1.1663	1.1256	1.0654	1.1304	1.0642	1.1353	1.0841	1.1208	1.0561	1.3175	1.2021	1.1663	1.1256	1.0654	1.1305	1.0803	1.1353	1.0841	1.1208	1.0561	**1.275**
**H(11A-11B)**	0.9459	0.9690	0.9245	0.9804	0.9421	0.9792	0.9349	0.9781	0.9366	0.9815	0.9191	0.9459	0.9690	0.9246	0.9804	0.9421	0.9792	0.9372	0.9781	0.9366	0.9815	0.9191	**1.28**
**H(12A-12B)**	1.1988	1.1808	1.1562	1.1611	1.1218	1.1625	1.1180	1.1638	1.1316	1.1595	1.1225	1.1988	1.1808	1.1562	1.1611	1.1218	1.1625	1.1317	1.1637	1.1316	1.1595	1.1225	**1.285**
**H(13A-13B)**	1.1041	1.1059	1.0743	1.1103	1.0748	1.1097	1.0643	1.1092	1.0723	1.1107	1.0659	1.1041	1.1059	1.0743	1.1103	1.0748	1.1098	1.0737	1.1092	1.0723	1.1107	1.0659	**1.29**
**H(14A-14B)**	1.2633	1.2579	1.2366	1.2520	1.2200	1.2523	1.2099	1.2526	1.2256	1.2514	1.2206	1.2633	1.2579	1.2366	1.2520	1.2200	1.2523	1.2273	1.2526	1.2256	1.2514	1.2206	**1.295**
**H(15A-15C)**	0.9168	0.9124	0.8892	0.9087	0.8787	0.9089	0.8602	0.9091	0.8814	0.9083	0.8759	0.9168	0.9124	0.8892	0.9087	0.8787	0.9089	0.8830	0.9092	0.8814	0.9083	0.8759	**0.82**
**H(1\`A)**	2.6659	2.6665	2.6456	2.6482	2.6026	2.6500	2.6231	2.6515	2.6203	2.6461	2.5870	2.7915	2.7807	2.7695	2.7694	2.7313	2.7697	2.7450	2.7702	2.7422	2.7689	2.7127	**3.82**
**H(1\`B)**	3.1885	3.2257	3.2425	3.2467	3.2909	3.2455	3.2870	3.2442	3.2848	3.2474	3.2855	2.8709	2.6728	2.6797	2.6515	2.6707	2.6500	2.6189	2.6492	2.6167	2.6535	2.6221	**3.78**
**H(2\`A-2\`B)**	1.7045	1.7405	1.7093	1.7670	1.7237	1.7651	1.7212	1.7633	1.7342	1.7684	1.7528	1.5640	1.6413	1.6095	1.6899	1.6396	1.6869	1.6731	1.6836	1.6628	1.6924	1.7181	**2.26**
**H(3\`A-3\`B)**	1.5640	1.6412	1.6095	1.6899	1.6396	1.6869	1.6494	1.6836	1.6628	1.6924	1.7181	1.7045	1.7405	1.7093	1.7670	1.7237	1.7651	1.7412	1.7633	1.7342	1.7685	1.7528	**2.01**
**H(4\`A)**	2.8709	2.6728	2.6797	2.6514	2.6707	2.6500	2.6493	2.6492	2.6167	2.6535	2.6221	3.1885	3.2257	3.2425	3.2467	3.2909	3.2454	3.2915	3.2442	3.2848	3.2474	3.2855	**3.68**
**H(4\`B)**	2.7915	2.7807	2.7695	2.7694	2.7313	2.7697	2.7528	2.7702	2.7423	2.7689	2.7127	2.6659	2.6665	2.6456	2.6482	2.6026	2.6499	2.6220	2.6516	2.6203	2.6461	2.5870	**2.88**

[a] methyl protons shifts are averaged
[b] according to [7]

Table S7. Cont. VI

Nuclei	R											S											Exp. [b]
	TPSS																						
	Gas	$CHCl_3$ (PCM)	$CHCl_3$ (SMD)	DMSO (PCM)	DMSO (SMD)	CH_3OH (PCM)	CH_3OH (SMD)	C_2H_5OH (PCM)	C_2H_5OH (SMD)	H_2O (PCM)	H_2O (SMD)	Gas	$CHCl_3$ (PCM)	$CHCl_3$ (SMD)	DMSO (PCM)	DMSO (SMD)	CH_3OH (PCM)	CH_3OH (SMD)	C_2H_5OH (PCM)	C_2H_5OH (SMD)	H_2O (PCM)	H_2O (SMD)	
**H2**	8.5344	8.4605	8.4553	8.3885	8.3733	8.3942	8.4120	8.4002	8.2402	8.3828	8.3958	8.5344	8.4604	8.4553	8.3885	8.3733	8.3942	8.4231	8.4002	8.5104	8.3828	8.3958	**8.07**
**H3**	7.1653	7.3018	7.4016	7.3518	7.4739	7.3496	7.4691	7.3478	7.1836	7.3542	7.5105	7.1653	7.3018	7.4016	7.3518	7.4739	7.3496	7.4970	7.3478	7.4659	7.3542	7.5105	**7.24**
**H5**	6.9980	7.1744	7.2660	7.2508	7.3782	7.2462	7.3768	7.2421	7.0775	7.2556	7.4107	6.9980	7.1744	7.2660	7.2508	7.3782	7.2462	7.4001	7.2421	7.3610	7.2556	7.4107	**7.24**
**H6**	7.8602	8.0740	8.1335	8.1578	8.2718	8.1533	8.3177	8.1492	7.9882	8.1617	8.3602	7.8602	8.0740	8.1335	8.1578	8.2718	8.1533	8.3430	8.1492	8.2613	8.1617	8.3602	**8.07**
**H8**	4.7765	4.8553	4.8874	4.8990	4.9457	4.8983	5.0348	4.8975	4.7235	4.8988	4.9980	4.7765	4.8553	4.8874	4.8990	4.9457	4.8983	5.0032	4.8975	5.0340	4.8989	4.9980	**5.24**
**H(9A-9B)**	1.8183	1.8707	1.8513	1.8946	1.9166	1.8918	1.9239	1.8901	1.7040	1.8982	1.9375	1.8183	1.8707	1.8513	1.8946	1.9166	1.8918	1.9151	1.8901	2.0491	1.8982	1.9375	**2.135**
**H(10A-10B)**	1.3748	1.2619	1.2343	1.2042	1.1424	1.2083	1.1457	1.2131	1.0243	1.2006	1.1477	1.3748	1.2618	1.2343	1.2042	1.1424	1.2083	1.1568	1.2131	1.3772	1.2006	1.1477	**1.275**
**H(11A-11B)**	0.9969	1.0174	0.9836	1.0416	1.0066	1.0396	1.0017	1.0383	0.8488	1.0443	1.0000	0.9969	1.0173	0.9836	1.0416	1.0066	1.0396	1.0040	1.0383	1.2037	1.0443	1.0000	**1.28**
**H(12A-12B)**	1.2302	1.2141	1.1970	1.2044	1.1681	1.2053	1.1655	1.2069	1.0181	1.2037	1.1736	1.2302	1.2141	1.1970	1.2044	1.1681	1.2053	1.1769	1.2069	1.3711	1.2037	1.1736	**1.285**
**H(13A-13B)**	1.1404	1.1423	1.1191	1.1555	1.1238	1.1545	1.1137	1.1542	0.9652	1.1569	1.1193	1.1404	1.1423	1.1191	1.1555	1.1238	1.1545	1.1219	1.1542	1.3187	1.1569	1.1193	**1.29**
**H(14A-14B)**	1.2898	1.2864	1.2731	1.2876	1.2611	1.2878	1.2496	1.2885	1.1000	1.2878	1.2596	1.2898	1.2865	1.2731	1.2876	1.2611	1.2878	1.2652	1.2885	1.4520	1.2878	1.2596	**1.295**
**H(15A-15C)**	0.9257	0.9212	0.9047	0.9235	0.8975	0.9236	0.8774	0.9242	0.7342	0.9238	0.8927	0.9257	0.9212	0.9047	0.9235	0.8975	0.9236	0.8986	0.9242	1.0904	0.9238	0.8927	**0.82**
**H(1`A)**	2.7127	2.7178	2.7102	2.7102	2.6750	2.7111	2.6914	2.7125	2.6337	2.7096	2.6589	2.8260	2.8289	2.8263	2.8210	2.8000	2.8216	2.8003	2.8229	3.0758	2.8209	2.6589	**3.82**
**H(1`B)**	3.2909	3.3309	3.3545	3.3572	3.4044	3.3561	3.4007	3.3553	3.5656	3.3583	3.3932	3.0450	2.8239	2.8203	2.7747	2.8087	2.7755	2.7363	2.7776	2.7646	2.7759	3.3932	**3.78**
**H(2`A-2`B)**	1.7502	1.7958	1.7709	1.8300	1.7917	1.8280	1.7869	1.8265	1.6402	1.8324	1.8112	1.5908	1.6769	1.6541	1.7289	1.6875	1.7261	1.7151	1.7238	1.8840	1.7317	1.7516	**2.26**
**H(3`A-3`B)**	1.5908	1.6769	1.6541	1.7289	1.6875	1.7261	1.6900	1.7238	1.5370	1.7317	1.7516	1.7502	1.7959	1.7709	1.8300	1.7916	1.8280	1.8040	1.8265	1.9860	1.8324	1.8112	**2.01**
**H(4`A)**	3.0450	2.8239	2.8203	2.7747	2.8087	2.7755	2.7742	2.7776	2.7646	2.7758	2.7312	3.2909	3.3309	3.3545	3.3572	3.4044	3.3561	3.3969	3.3553	3.5656	3.3583	2.7312	**3.68**
**H(4`B)**	2.8260	2.8289	2.8263	2.8210	2.8000	2.8216	2.8091	2.8229	3.0758	2.8209	2.7679	2.7127	2.7178	2.7102	2.7102	2.6750	2.7111	2.6868	2.7125	2.6337	2.7096	2.7679	**2.88**
Nuclei	**VSXC**																						
**H2**	8.3988	8.3636	8.3860	8.3429	8.3459	8.3453	8.3826	8.3479	8.3995	8.3406	8.3943	8.3988	8.3636	8.3860	8.3429	8.3459	8.3453	8.4051	8.3479	8.3995	8.3406	8.3943	**8.07**
**H3**	7.2438	7.3807	7.4860	7.4142	7.5275	7.4117	7.5214	7.4094	7.5472	7.4165	7.5710	7.2437	7.3807	7.4860	7.4142	7.5275	7.4117	7.5553	7.4094	7.5472	7.4165	7.5710	**7.24**
**H5**	7.0522	7.2438	7.3088	7.3334	7.4452	7.3283	7.4462	7.3233	7.4560	7.3381	7.4816	7.0522	7.2438	7.3088	7.3334	7.4452	7.3283	7.4663	7.3233	7.4560	7.3381	7.4816	**7.24**
**H6**	8.0720	8.1953	8.2679	8.2596	8.3808	8.2563	8.4200	8.2532	8.3883	8.2629	8.4136	8.0720	8.1953	8.2679	8.2596	8.3808	8.2563	8.4001	8.2532	8.3883	8.2629	8.4136	**8.07**
**H8**	4.4372	4.5131	4.5324	4.5732	4.6373	4.5701	4.7152	4.5671	4.6645	4.5760	4.7037	4.4372	4.5131	4.5324	4.5732	4.6373	4.5701	4.6832	4.5671	4.6645	4.5760	4.7037	**5.24**
**H(9A-9B)**	2.0361	2.0457	2.1007	2.0505	2.0349	2.0484	2.0533	2.0466	2.0673	2.0528	2.0674	2.0361	2.0457	2.1007	2.0505	2.0349	2.0484	2.0773	2.0466	2.0673	2.0528	2.0674	**2.135**
**H(10A-10B)**	1.7784	1.6868	1.6612	1.6079	1.5394	1.6121	1.5186	1.6165	1.5390	1.6038	1.4950	1.7784	1.6868	1.6612	1.6079	1.5394	1.6121	1.5240	1.6165	1.5390	1.6038	1.4950	**1.275**
**H(11A-11B)**	1.4970	1.5023	1.4845	1.4801	1.4645	1.4815	1.4369	1.4830	1.4439	1.4788	1.4318	1.4970	1.5022	1.4845	1.4801	1.4645	1.4815	1.4325	1.4830	1.4439	1.4788	1.4318	**1.28**
**H(12A-12B)**	1.4833	1.4795	1.4536	1.4763	1.4203	1.4762	1.4270	1.4764	1.4444	1.4763	1.4483	1.4833	1.4795	1.4536	1.4763	1.4203	1.4762	1.4518	1.4764	1.4444	1.4763	1.4483	**1.285**
**H(13A-13B)**	1.4353	1.4394	1.4135	1.4443	1.4036	1.4436	1.3921	1.4432	1.4036	1.4448	1.4057	1.4353	1.4394	1.4135	1.4443	1.4036	1.4436	1.4042	1.4432	1.4036	1.4448	1.4057	**1.29**
**H(14A-14B)**	1.4717	1.4714	1.4430	1.4674	1.4283	1.4676	1.4186	1.4679	1.4322	1.4671	1.4322	1.4717	1.4713	1.4430	1.4674	1.4283	1.4676	1.4324	1.4680	1.4322	1.4671	1.4322	**1.295**
**H(15A-15C)**	1.0851	1.0821	1.0599	1.0800	1.0488	1.0800	1.0268	1.0802	1.0499	1.0799	1.0500	1.0851	1.0821	1.0599	1.0800	1.0488	1.0800	1.0487	1.0802	1.0499	1.0799	1.0500	**0.82**
**H(1`A)**	2.4739	2.5242	2.5459	2.5195	2.5150	2.5193	2.5065	2.5193	2.5103	2.5197	2.5107	2.9591	2.9754	2.9681	2.9692	2.9491	2.9699	2.9467	2.9707	2.9524	2.9686	2.9616	**3.82**
**H(1`B)**	3.3549	3.3780	3.4140	3.3893	3.4396	3.3883	3.4303	3.3875	3.4422	3.3902	3.4489	2.7107	2.6435	2.6243	2.6511	2.6400	2.6503	2.6487	2.6497	2.6412	2.6518	2.6682	**3.78**
**H(2`A-2`B)**	1.9376	1.9694	1.9490	1.9633	1.9355	1.9631	1.9217	1.9632	1.9355	1.9635	1.9424	2.0155	2.0509	2.0052	2.1050	2.0552	2.1022	2.0689	2.0994	2.0642	2.1076	2.0886	**2.26**
**H(3`A-3`B)**	2.0155	2.0509	2.0052	2.1050	2.0552	2.1022	2.0601	2.0994	2.0642	2.1076	2.0886	1.9376	1.9694	1.9490	1.9633	1.9355	1.9631	1.9323	1.9632	1.9355	1.9635	1.9424	**2.01**
**H(4`A)**	2.7107	2.6435	2.6243	2.6511	2.6400	2.6503	2.6260	2.6497	2.6412	2.6518	2.6682	3.3549	3.3780	3.4140	3.3893	3.4396	3.3883	3.4427	3.3875	3.4422	3.3902	3.4489	**3.68**
**H(4`B)**	2.9591	2.9754	2.9681	2.9692	2.9491	2.9699	2.9544	2.9707	2.9524	2.9686	2.9616	2.4739	2.5242	2.5459	2.5195	2.5150	2.5193	2.5061	2.5193	2.5103	2.5197	2.5107	**2.88**
Nuclei	**wB97**																						
**H2**	8.8640	8.7938	8.7724	8.6950	8.6604	8.7064	8.7098	8.7158	8.6221	8.6822	8.6045	8.8640	8.7938	8.7724	8.6950	8.6604	8.7064	8.6297	8.7158	8.6221	8.6822	8.6045	**8.07**
**H3**	7.4417	7.5762	7.6681	7.6190	7.7439	7.6167	7.7286	7.6142	7.7489	7.6212	7.7739	7.4416	7.5762	7.6681	7.6190	7.7439	7.6167	7.7566	7.6142	7.7489	7.6212	7.7739	**7.24**
**H5**	7.2983	7.4946	7.5901	7.5772	7.7114	7.5718	7.6975	7.5663	7.7118	7.5824	7.7409	7.2983	7.4946	7.5901	7.5772	7.7114	7.5718	7.7227	7.5663	7.7117	7.5824	7.7409	**7.24**
**H6**	8.1494	8.3316	8.3946	8.4104	8.5338	8.4085	8.5645	8.4056	8.5406	8.4113	8.5970	8.1494	8.3316	8.3946	8.4104	8.5337	8.4085	8.5654	8.4056	8.5406	8.4113	8.5970	**8.07**
**H8**	4.2038	4.2950	4.3535	4.3298	4.4371	4.3286	4.4900	4.3268	4.4695	4.3305	4.5006	4.2038	4.2949	4.3535	4.3298	4.4371	4.3286	4.4908	4.3268	4.4695	4.3305	4.5006	**5.24**
**H(9A-9B)**	1.7093	1.7342	1.6955	1.7645	1.7470	1.7629	1.7759	1.7612	1.6995	1.7657	1.7494	1.7093	1.7342	1.6955	1.7645	1.7470	1.7629	1.7066	1.7612	1.6995	1.7657	1.7493	**2.135**
**H(10A-10B)**	1.3372	1.2063	1.1725	1.1059	1.0455	1.1122	1.0403	1.1182	1.0640	1.0998	1.0543	1.3372	1.2064	1.1725	1.1059	1.0455	1.1123	1.0605	1.1182	1.0640	1.0998	1.0543	**1.275**
**H(11A-11B)**	0.9598	0.9683	0.9240	0.9796	0.9393	0.9787	0.9300	0.9778	0.9124	0.9805	0.9139	0.9598	0.9683	0.9240	0.9796	0.9393	0.9787	0.9137	0.9778	0.9124	0.9805	0.9139	**1.28**
**H(12A-12B)**	1.2118	1.1858	1.1641	1.1590	1.1261	1.1607	1.1137	1.1623	1.1290	1.1575	1.1259	1.2118	1.1858	1.1641	1.1590	1.1261	1.1606	1.1277	1.1623	1.1290	1.1575	1.1259	**1.285**
**H(13A-13B)**	1.1189	1.1154	1.0843	1.1183	1.0864	1.1178	1.0705	1.1174	1.0744	1.1187	1.0755	1.1189	1.1154	1.0843	1.1182	1.0864	1.1178	1.0748	1.1174	1.0745	1.1187	1.0755	**1.29**
**H(14A-14B)**	1.2522	1.2402	1.2213	1.2310	1.2052	1.2315	1.1878	1.2320	1.2067	1.2306	1.2060	1.2522	1.2402	1.2213	1.2310	1.2052	1.2315	1.2064	1.2319	1.2067	1.2306	1.2059	**1.295**
**H(15A-15C)**	0.9462	0.9415	0.9170	0.9372	0.9108	0.9373	0.8882	0.9374	0.9079	0.9371	0.9053	0.9462	0.9415	0.9170	0.9371	0.9108	0.9373	0.9078	0.9375	0.9079	0.9371	0.9053	**0.82**
**H(1`A)**	2.6200	2.6194	2.5974	2.6038	2.5658	2.6048	2.5825	2.6057	2.5759	2.6028	2.5496	2.7670	2.7640	2.7475	2.7515	2.7169	2.7516	2.7449	2.7518	2.7464	2.7518	2.7175	**3.82**
**H(1`B)**	3.0872	3.1201	3.1388	3.1409	3.1867	3.1396	3.1822	3.1380	3.1747	3.1420	3.1805	2.7305	2.5597	2.5819	2.5594	2.5927	2.5558	2.5743	2.5533	2.5790	2.5641	2.5750	**3.78**
**H(2`A-2`B)**	1.6835	1.7199	1.6886	1.7501	1.7148	1.7478	1.7063	1.7457	1.7198	1.7522	1.7457	1.5756	1.6498	1.6199	1.6950	1.6556	1.6915	1.6897	1.6881	1.6839	1.6986	1.7204	**2.26**
**H(3`A-3`B)**	1.5756	1.6498	1.6199	1.6950	1.6556	1.6915	1.6564	1.6881	1.6839	1.6986	1.7204	1.6835	1.7199	1.6886	1.7501	1.7147	1.7478	1.7253	1.7457	1.7198	1.7522	1.7456	**2.01**
**H(4`A)**	2.7305	2.5597	2.5819	2.5594	2.5927	2.5558	2.5617	2.5533	2.5790	2.5641	2.5750	3.0872	3.1200	3.1388	3.1409	3.1867	3.1396	3.1795	3.1380	3.1747	3.1420	3.1805	**3.68**
**H(4`B)**	2.7670	2.7640	2.7475	2.7515	2.7169	2.7516	2.7351	2.7518	2.7464	2.7518	2.7175	2.6199	2.6194	2.5974	2.6037	2.5658	2.6048	2.5766	2.6057	2.5759	2.6028	2.5496	**2.88**
Nuclei	**wB97X**																						
**H2**	8.8359	8.7674	8.7372	8.6493	8.6282	8.6649	8.6656	8.6764	8.5997	8.6342	8.5816	8.8359	8.7675	8.7372	8.6493	8.6282	8.6649	8.6122	8.6764	8.5996	8.6342	8.5816	**8.07**
**H3**	7.4161	7.5506	7.6418	7.5968	7.7182	7.5947	7.7064	7.5923	7.7239	7.5987	7.7502	7.4161	7.5506	7.6419	7.5968	7.7182	7.5947	7.7346	7.5923	7.7239	7.5987	7.7502	**7.24**
**H5**	7.2774	7.4724	7.5691	7.5618	7.6917	7.5567	7.6810	7.5516	7.6895	7.5665	7.7159	7.2774	7.4724	7.5691	7.5618	7.6917	7.5567	7.7033	7.5516	7.6895	7.5665	7.7159	**7.24**
**H6**	8.1156	8.3072	8.3717	8.3609	8.4893	8.3624	8.5165	8.3623	8.5000	8.3606	8.5574	8.1156	8.3072	8.3717	8.3609	8.4893	8.3625	8.5277	8.3623	8.5000	8.3605	8.5574	**8.07**
**H8**	4.3016	4.3950	4.4437	4.4142	4.5020	4.4138	4.5734	4.4135	4.5351	4.4150	4.5618	4.3016	4.3950	4.4437	4.4142	4.5020	4.4138	4.5588	4.4135	4.5351	4.4150	4.5618	**5.24**
**H(9A-9B)**	1.6953	1.7237	1.6844	1.7316	1.7266	1.7350	1.7408	1.7361	1.6842	1.7290	1.7289	1.6953	1.7237	1.6844	1.7316	1.7266	1.7350	1.6945	1.7361	1.6842	1.7290	1.7289	**2.135**
**H(10A-10B)**	1.3226	1.1982	1.1647	1.1086	1.0488	1.1151	1.0445	1.1214	1.0566	1.1029	1.0469	1.3226	1.1982	1.1647	1.1086	1.0488	1.1151	1.0563	1.1214	1.0566	1.1029	1.0469	**1.275**
**H(11A-11B)**	0.9574	0.9695	0.9246	0.9722	0.9341	0.9726	0.9218	0.9727	0.9126	0.9724	0.9161	0.9574	0.9695	0.9246	0.9722	0.9341	0.9726	0.9172	0.9727	0.9126	0.9724	0.9161	**1.28**
**H(12A-12B)**	1.2030	1.1788	1.1568	1.1540	1.1191	1.1557	1.1084	1.1574	1.1218	1.1528	1.1226	1.2031	1.1789	1.1568	1.1540	1.1191	1.1557	1.1236	1.1574	1.1218	1.1528	1.1226	**1.285**
**H(13A-13B)**	1.1145	1.1132	1.0807	1.1146	1.0810	1.1145	1.0664	1.1144	1.0700	1.1150	1.0716	1.1145	1.1132	1.0807	1.1146	1.0810	1.1145	1.0733	1.1144	1.0700	1.1150	1.0716	**1.29**
**H(14A-14B)**	1.2493	1.2390	1.2197	1.2311	1.2031	1.2315	1.1873	1.2320	1.2047	1.2309	1.2053	1.2493	1.2390	1.2197	1.2311	1.2031	1.2315	1.2072	1.2321	1.2047	1.2309	1.2052	**1.295**
**H(15A-15C)**	0.9401	0.9367	0.9111	0.9336	0.9049	0.9338	0.8836	0.9341	0.9011	0.9335	0.8986	0.9402	0.9367	0.9111	0.9336	0.9049	0.9338	0.9037	0.9341	0.9011	0.9335	0.8986	**0.82**
**H(1`A)**	2.6475	2.6473	2.6235	2.6282	2.5867	2.6300	2.6039	2.6317	2.6000	2.6268	2.5774	2.7857	2.7730	2.7577	2.7669	2.7299	2.7665	2.7549	2.7666	2.7542	2.7672	2.7273	**3.82**
**H(1`B)**	3.0968	3.1328	3.1511	3.1509	3.1938	3.1507	3.1906	3.1501	3.1857	3.1513	3.1923	2.7492	2.5754	2.5888	2.5733	2.6017	2.5684	2.5796	2.5654	2.5823	2.5780	2.5774	**3.78**
**H(2`A-2`B)**	1.6843	1.7247	1.6922	1.7506	1.7118	1.7491	1.7042	1.7476	1.7178	1.7524	1.7451	1.5751	1.6502	1.6210	1.7006	1.6570	1.6965	1.6917	1.6929	1.6836	1.7045	1.7192	**2.26**
**H(3`A-3`B)**	1.5751	1.6502	1.6210	1.7006	1.6570	1.6965	1.6602	1.6929	1.6836	1.7045	1.7192	1.6842	1.7247	1.6922	1.7506	1.7118	1.7491	1.7259	1.7476	1.7178	1.7524	1.7451	**2.01**
**H(4`A)**	2.7492	2.5754	2.5888	2.5733	2.6017	2.5684	2.5750	2.5654	2.5823	2.5780	2.5774	3.0968	3.1328	3.1511	3.1509	3.1938	3.1507	3.1933	3.1501	3.1857	3.1513	3.1923	**3.68**
**H(4`B)**	2.7857	2.7730	2.7578	2.7669	2.7299	2.7666	2.7491	2.7666	2.7543	2.7672	2.7273	2.6475	2.6472	2.6235	2.6282	2.5868	2.6300	2.6032	2.6317	2.6000	2.6268	2.5774	**2.88**

[a] methyl protons shifts are averaged

[b] according to [7]

Table S7. Cont. VII

Nuclei	R											S											Exp. [b]
	X3LYP																						
	Gas	$CHCI_3$ (PCM)	$CHCI_3$ (SMD)	DMSO (PCM)	DMSO (SMD)	CH_3OH (PCM)	CH_3OH (SMD)	C_2H_5OH (PCM)	C_2H_5OH (SMD)	H_2O (PCM)	H_2O (SMD)	Gas	$CHCI_3$ (PCM)	$CHCI_3$ (SMD)	DMSO (PCM)	DMSO (SMD)	CH_3OH (PCM)	CH_3OH (SMD)	C_2H_5OH (PCM)	C_2H_5OH (SMD)	H_2O (PCM)	H_2O (SMD)	
**H2**	8.6923	8.5851	8.6120	8.4998	8.5204	8.5067	8.5248	8.5126	8.4234	8.4928	8.5420	8.6923	8.5851	8.6120	8.4998	8.5204	8.5067	8.4265	8.5126	8.4234	8.4928	8.5420	**8.07**
**H3**	7.3199	7.4577	7.5459	7.5018	7.6170	7.5000	7.6088	7.4977	7.6123	7.5034	7.6546	7.3199	7.4577	7.5459	7.5018	7.6170	7.5000	7.6198	7.4977	7.6124	7.5034	7.6546	**7.24**
**H5**	7.1428	7.3159	7.4091	7.3975	7.5161	7.3930	7.5127	7.3880	7.5496	7.4016	7.5534	7.1428	7.3159	7.4091	7.3975	7.5161	7.3930	7.5599	7.3880	7.5496	7.4016	7.5534	**7.24**
**H6**	7.9762	8.1754	8.2527	8.2676	8.3950	8.2630	8.4240	8.2577	8.3426	8.2715	8.4642	7.9762	8.1754	8.2527	8.2676	8.3950	8.2630	8.3637	8.2577	8.3426	8.2715	8.4642	**8.07**
**H8**	4.6192	4.7050	4.7349	4.7424	4.7934	4.7415	4.8878	4.7398	4.8024	4.7427	4.8221	4.6192	4.7050	4.7349	4.7424	4.7934	4.7415	4.8169	4.7398	4.8024	4.7427	4.8221	**5.24**
**H(9A-9B)**	1.7276	1.7609	1.7597	1.7860	1.8299	1.7838	1.8069	1.7813	1.6968	1.7884	1.8419	1.7276	1.7609	1.7597	1.7860	1.8299	1.7838	1.7055	1.7813	1.6968	1.7884	1.8419	**2.135**
**H(10A-10B)**	1.3436	1.2341	1.1966	1.1686	1.0964	1.1736	1.1028	1.1778	1.1301	1.1640	1.1146	1.3436	1.2341	1.1966	1.1686	1.0964	1.1736	1.1270	1.1778	1.1301	1.1640	1.1146	**1.275**
**H(11A-11B)**	0.9902	1.0107	0.9751	1.0328	0.9936	1.0313	0.9836	1.0294	0.9776	1.0343	0.9838	0.9902	1.0107	0.9751	1.0328	0.9936	1.0313	0.9783	1.0294	0.9776	1.0343	0.9838	**1.28**
**H(12A-12B)**	1.2402	1.2279	1.2024	1.2155	1.1705	1.2167	1.1693	1.2175	1.1906	1.2142	1.1771	1.2402	1.2279	1.2024	1.2155	1.1705	1.2167	1.1890	1.2175	1.1906	1.2142	1.1771	**1.285**
**H(13A-13B)**	1.1521	1.1562	1.1267	1.1672	1.1302	1.1666	1.1189	1.1655	1.1267	1.1678	1.1249	1.1521	1.1563	1.1267	1.1672	1.1302	1.1666	1.1266	1.1655	1.1267	1.1678	1.1249	**1.29**
**H(14A-14B)**	1.3115	1.3097	1.2896	1.3093	1.2758	1.3097	1.2648	1.3095	1.2866	1.3090	1.2770	1.3115	1.3097	1.2896	1.3093	1.2758	1.3097	1.2858	1.3095	1.2867	1.3090	1.2770	**1.295**
**H(15A-15C)**	0.9605	0.9568	0.9354	0.9584	0.9276	0.9585	0.9066	0.9582	0.9308	0.9582	0.9248	0.9605	0.9568	0.9355	0.9584	0.9276	0.9585	0.9300	0.9582	0.9308	0.9582	0.9248	**0.82**
**H(1\`A)**	2.7415	2.7395	2.7170	2.7296	2.6807	2.7308	2.7002	2.7314	2.6945	2.7285	2.6685	2.8686	2.8553	2.8539	2.8395	2.8103	2.8403	2.8152	2.8407	2.8191	2.8389	2.7756	**3.82**
**H(1\`B)**	3.2180	3.2580	3.2831	3.2902	3.3399	3.2885	3.3345	3.2863	3.3214	3.2918	3.3402	2.8731	2.7021	2.6790	2.6801	2.6954	2.6804	2.6835	2.6806	2.6852	2.6804	2.6419	**3.78**
**H(2\`A-2\`B)**	1.7378	1.7799	1.7420	1.8113	1.7596	1.8096	1.7593	1.8075	1.7689	1.8129	1.7855	1.5853	1.6737	1.6412	1.7272	1.6716	1.7240	1.7208	1.7203	1.7158	1.7302	1.7275	**2.26**
**H(3\`A-3\`B)**	1.5853	1.6737	1.6412	1.7272	1.6716	1.7240	1.6816	1.7203	1.7158	1.7302	1.7275	1.7378	1.7799	1.7420	1.8113	1.7596	1.8096	1.7730	1.8075	1.7689	1.8129	1.7855	**2.01**
**H(4\`A)**	2.8731	2.7021	2.6790	2.6801	2.6954	2.6804	2.6773	2.6806	2.6852	2.6804	2.6419	3.2180	3.2580	3.2831	3.2902	3.3399	3.2885	3.3258	3.2863	3.3214	3.2918	3.3402	**3.68**
**H(4\`B)**	2.8686	2.8553	2.8539	2.8395	2.8103	2.8403	2.8165	2.8407	2.8191	2.8389	2.7755	2.7415	2.7395	2.7170	2.7296	2.6807	2.7308	2.6939	2.7314	2.6946	2.7285	2.6685	**2.88**

[a] methyl protons shifts are averaged

[b] according to [7]

Table S8. Theoretical chemical shifts (in ppm) of *(R,S)*-3,5-difluoromethcathinone calculated with HF and selected functionals combined with 6-311++G** basis set[a]

Nuclei	R											S											Exp. [b]
	**B3LYP**																						
	Gas	$CHCl_3$ (PCM)	$CHCl_3$ (SMD)	DMSO (PCM)	DMSO (SMD)	CH_3OH (PCM)	CH_3OH (SMD)	C_2H_5OH (PCM)	C_2H_5OH (SMD)	H_2O (PCM)	H_2O (SMD)	Gas	$CHCl_3$ (PCM)	$CHCl_3$ (SMD)	DMSO (PCM)	DMSO (SMD)	CH_3OH (PCM)	CH_3OH (SMD)	C_2H_5OH (PCM)	C_2H_5OH (SMD)	H_2O (PCM)	H_2O (SMD)	
**C1**	146.4677	146.9292	148.0988	147.6979	148.4736	147.6833	147.7806	147.6707	148.2614	147.7123	147.9596	145.1719	145.8243	146.9929	146.9594	148.1381	146.9613	148.5756	146.9602	148.3632	146.9530	148.5403	**136.1**
**C2**	118.2357	118.4415	119.2285	118.4740	119.0625	118.4789	119.5910	118.4837	119.5263	118.4676	119.6157	117.9173	118.1733	118.8888	118.3268	118.9778	118.3295	119.6312	118.3314	119.4749	118.3219	119.6136	**112.1**
**C3**	173.8901	174.1596	174.6988	174.1841	174.9250	174.1785	174.8915	174.1727	174.8780	174.1880	174.9444	173.8909	174.1647	174.7247	174.2315	174.9695	174.2248	174.9659	174.2180	174.9403	174.2361	175.0141	**162.6**
**C4**	113.3666	114.9502	115.5066	115.4999	116.1902	115.4557	117.1667	115.4109	116.8136	115.5401	117.3254	113.7037	115.0369	115.5638	115.3656	116.0247	115.3261	116.5833	115.2867	116.4317	115.4020	116.7917	**110.1**
**C5**	173.0985	173.7005	174.3084	173.9312	174.8307	173.9123	174.7786	173.8929	174.7050	173.9472	174.8439	173.3682	173.8541	174.3768	173.9601	174.7817	173.9440	174.6338	173.9278	174.6196	173.9740	174.7006	**162.5**
**C6**	116.2639	118.0937	119.0762	118.7843	120.6195	118.7221	121.0739	118.6593	120.7098	118.8407	121.3768	116.6165	118.6555	119.8320	119.3449	121.1227	119.2776	121.1635	119.2116	121.0566	119.4084	121.5448	**112.1**
**C7**	203.0400	208.1918	209.9625	211.4121	211.6927	211.2368	216.6248	211.0647	215.8909	211.5796	217.4578	207.8788	211.9529	213.2793	214.1274	214.9452	214.0189	220.0550	213.9076	218.8762	214.2254	220.4478	**194.5**
**C8**	65.7863	66.8618	67.6156	67.4306	68.1115	67.4068	68.7102	67.3819	68.6981	67.4507	69.4071	60.2985	61.8411	62.7739	63.1168	63.8414	63.0696	64.7521	63.0220	64.4420	63.1596	64.9809	**58.4**
**C9**	22.7168	22.9952	23.0739	23.0415	23.4528	23.0238	23.5512	23.0064	23.4437	23.0574	23.6823	19.3599	19.6571	19.9384	19.8480	20.1286	19.8299	20.1631	19.8126	20.0976	19.8645	20.5201	**15**
**C8`**	36.4011	37.2578	37.5459	37.6238	37.9821	37.6012	38.1346	37.5775	38.0714	37.6428	38.5413	31.9457	32.4408	32.5072	32.3536	32.6983	32.3345	32.7300	32.3154	32.7241	32.3704	32.8014	**30.6**
**H2**	7.9509	7.9079	7.9469	7.7980	7.9264	7.8011	7.9600	7.8041	7.9178	7.7946	7.9552	8.0159	7.9363	7.9537	7.7952	7.8738	7.7959	7.8128	7.7969	7.8288	7.7946	7.8284	**7.77**
**H4**	7.0566	7.2663	7.3996	7.3392	7.5222	7.3346	7.5629	7.3300	7.5412	7.3433	7.5859	7.0719	7.2685	7.3989	7.3290	7.5116	7.3250	7.5370	7.3209	7.5223	7.3326	7.5607	**7.71**
**H6**	7.4558	7.6869	7.7969	7.7595	7.9898	7.7522	8.0226	7.7448	7.9800	7.7662	8.0546	7.2829	7.4978	7.6339	7.5509	7.7952	7.5444	7.7655	7.5381	7.7586	7.5568	7.8162	**7.77**
**H8**	4.2675	4.3715	4.3854	4.4256	4.5217	4.4195	4.5442	4.4136	4.5179	4.4315	4.5673	4.3981	4.5613	4.6292	4.6357	4.7688	4.6310	4.7873	4.6263	4.7701	4.6399	4.8193	**5.21**
**H(9A-9C)**	1.3037	1.3032	1.2744	1.2674	1.2627	1.2682	1.2876	1.2688	1.2697	1.2664	1.3035	1.3822	1.4261	1.4103	1.4292	1.4110	1.4265	1.4041	1.4238	1.4015	1.4318	1.4343	**1.43**
**H(8`A-8`C)**	2.5005	2.4483	2.4213	2.4402	2.3951	2.4411	2.3865	2.4421	2.3958	2.4391	2.3719	2.9283	2.8026	2.7259	2.6969	2.6502	2.6998	2.5619	2.7027	2.5795	2.6942	2.5324	**2.58**
Nuclei	**B3P86**																						
**C1**	144.1660	144.4797	145.4429	144.8774	146.0553	144.8655	145.5049	144.8368	146.1053	144.9425	145.6123	143.1406	143.5737	144.8111	144.6471	145.7610	144.6639	145.8547	144.6578	145.8265	144.6747	145.9738	**136.1**
**C2**	117.1888	117.2869	118.0833	117.2508	117.9809	117.2729	118.5007	117.2740	118.5577	117.2762	118.5105	116.9546	117.1028	117.8952	117.2670	117.9437	117.2929	118.4927	117.2964	118.4581	117.2862	118.5197	**112.1**
**C3**	171.4614	171.7172	172.3130	171.7935	172.5569	171.8106	172.5150	171.8057	172.6070	171.8208	172.5919	171.4545	171.6953	172.3058	171.7939	172.5506	171.8099	172.5417	171.8041	172.6302	171.8227	172.6086	**162.6**
**C4**	112.6935	114.2631	114.9216	114.9543	115.6565	114.9348	116.6256	114.8930	116.3813	115.0160	116.8114	112.9577	114.3484	114.9712	114.7667	115.5303	114.7487	116.1670	114.7089	116.0943	114.8276	116.3590	**110.1**
**C5**	170.6396	171.2286	171.8606	171.5322	172.4184	171.5341	172.3812	171.5142	172.4223	171.5746	172.4697	170.8641	171.3394	171.9146	171.4849	172.3365	171.4886	172.1824	171.4706	172.2769	171.5255	172.2618	**162.5**
**C6**	115.1391	116.9725	118.0918	117.7875	119.5088	117.7611	119.8391	117.7107	119.6009	117.8524	120.2013	115.4610	117.4757	118.6570	118.2342	120.1056	118.1968	120.3513	118.1375	120.3252	118.3135	120.6914	**112.1**
**C7**	201.2424	205.9139	207.3479	208.5755	209.2297	208.4230	214.1956	208.2553	213.6177	208.7764	214.8811	205.4731	209.3552	210.7967	211.5125	212.2399	211.4135	216.8930	211.2924	215.8719	211.6489	217.3854	**194.5**
**C8**	64.2040	65.3664	66.1786	65.8597	66.6954	65.8541	67.1728	65.8252	67.2596	65.9068	67.9407	58.8166	60.1415	61.0329	61.3340	62.1120	61.3184	62.9127	61.2806	62.7586	61.3920	63.2267	**58.4**
**C9**	22.5143	22.8390	23.0810	22.9979	23.3784	23.0117	23.3286	23.0021	23.3230	23.0258	23.5336	19.2207	19.5615	19.8115	19.8253	20.1843	19.8395	20.2690	19.8308	20.3207	19.8571	20.6964	**15**
**C8`**	35.7833	36.5853	36.8683	36.9408	37.3138	36.9391	37.5076	36.9156	37.5815	36.9872	37.9639	31.1841	31.6716	31.7883	31.6040	31.9477	31.6036	32.0120	31.5817	32.0897	31.6487	32.0763	**30.6**
**H2**	7.9786	7.9412	7.9991	7.8711	7.9591	7.8776	7.9830	7.8818	7.9472	7.8653	7.9834	8.0190	7.9594	7.9790	7.8261	7.9101	7.8289	7.8714	7.8300	7.8893	7.8247	7.8757	**7.77**
**H4**	7.0496	7.2640	7.4025	7.3415	7.5279	7.3384	7.5689	7.3335	7.5532	7.3456	7.5929	7.0605	7.2639	7.4003	7.3291	7.5160	7.3262	7.5433	7.3215	7.5335	7.3329	7.5672	**7.71**
**H6**	7.4934	7.7155	7.8209	7.7953	8.0030	7.7918	8.0369	7.7864	8.0008	7.7997	8.0728	7.3080	7.5286	7.6680	7.5849	7.8409	7.5797	7.8294	7.5726	7.8251	7.5912	7.8753	**7.77**
**H8**	4.2911	4.3817	4.3832	4.4432	4.5173	4.4400	4.5561	4.4353	4.5396	4.4479	4.5755	4.4482	4.6057	4.6829	4.6830	4.8179	4.6795	4.8361	4.6742	4.8242	4.6875	4.8679	**5.21**
**H(9A-9C)**	1.3189	1.3286	1.3159	1.3066	1.2900	1.3098	1.2964	1.3108	1.2843	1.3042	1.3215	1.3944	1.4437	1.4215	1.4457	1.4327	1.4453	1.4410	1.4433	1.4431	1.4472	1.4705	**1.43**
**H(8`A-8`C)**	2.4791	2.4261	2.4038	2.4104	2.3732	2.4125	2.3542	2.4127	2.3706	2.4097	2.3422	2.8724	2.7513	2.6774	2.6438	2.6000	2.6476	2.5044	2.6496	2.5301	2.6416	2.4762	**2.58**
Nuclei	**B3PW91**																						
**C1**	144.4592	145.2078	145.9935	145.7239	147.0015	145.7191	145.9285	145.7072	146.3789	145.7326	146.3444	143.3662	143.8506	145.0132	145.0815	146.1621	145.0843	146.8130	145.0770	146.6448	145.0799	146.7877	**136.1**
**C2**	117.3307	117.5489	118.2475	117.5206	118.5354	117.5346	118.6517	117.5398	118.6043	117.5093	118.6949	117.0832	117.2591	117.9688	117.4343	118.1073	117.4441	118.6963	117.4446	118.5750	117.4271	118.7057	**112.1**
**C3**	171.5132	171.7956	172.3082	171.8477	172.9313	171.8494	172.5768	171.8421	172.5747	171.8482	172.6619	171.5364	171.8090	172.3416	171.9001	172.6567	171.9014	172.6539	171.8939	172.6430	171.9009	172.7272	**162.6**
**C4**	112.7108	114.2346	114.8779	114.8862	115.9273	114.8488	116.5867	114.8024	116.2602	114.9234	116.6946	112.9678	114.3785	114.9162	114.7374	115.5101	114.7065	115.9680	114.6669	115.8284	114.7684	116.2085	**110.1**
**C5**	170.7078	171.2992	171.8919	171.5720	172.7862	171.5603	172.4196	171.5395	172.3632	171.5850	172.4914	170.9345	171.4513	171.9527	171.5863	172.4462	171.5778	172.2691	171.5608	172.2700	171.5962	172.3650	**162.5**
**C6**	115.0871	116.8012	117.8678	117.5993	119.6959	117.5463	119.8014	117.4838	119.4710	117.6512	120.0586	115.4555	117.4808	118.5970	118.1662	119.9965	118.1103	119.9481	118.0458	119.8519	118.2219	120.3434	**112.1**
**C7**	201.4194	206.5504	208.0253	209.5367	210.1574	209.3785	214.7275	209.2146	213.9946	209.6915	215.6703	205.5779	209.6147	210.9505	211.8651	212.6209	211.7645	217.8155	211.6534	216.6474	211.9608	218.2041	**194.5**
**C8**	63.9919	65.2281	65.7920	65.7140	66.8128	65.6970	66.9647	65.6706	66.9471	65.7314	67.7902	58.7798	60.1105	60.9641	61.4005	62.0754	61.3592	63.0761	61.3092	62.7828	61.4417	63.3176	**58.4**
**C9**	22.2342	22.5003	22.5539	22.5857	23.3101	22.5760	23.0658	22.5577	22.9749	22.5973	23.2204	18.9613	19.2770	19.4721	19.5102	19.7921	19.4993	19.7986	19.4797	19.7485	19.5228	20.1855	**15**
**C8`**	35.7629	36.6018	36.8457	36.9870	37.6410	36.9718	37.4614	36.9478	37.4343	37.0029	37.8867	31.2201	31.7770	31.8162	31.6900	32.0674	31.6802	32.0347	31.6617	32.0352	31.7016	32.1307	**30.6**
**H2**	7.9465	7.8719	7.9441	7.7902	7.9161	7.7938	7.9414	7.7970	7.9062	7.7867	7.9115	7.9918	7.9331	7.9518	7.7857	7.8746	7.7877	7.7926	7.7895	7.8085	7.7840	7.8089	**7.77**
**H4**	7.0444	7.2542	7.3936	7.3330	7.5309	7.3290	7.5605	7.3247	7.5413	7.3370	7.5799	7.0574	7.2582	7.3929	7.3231	7.5082	7.3196	7.5345	7.3159	7.5214	7.3264	7.5573	**7.71**
**H6**	7.4717	7.6694	7.8034	7.7640	7.9880	7.7574	8.0207	7.7505	7.9831	7.7703	8.0372	7.2894	7.5101	7.6493	7.5623	7.8144	7.5567	7.7733	7.5508	7.7681	7.5679	7.8238	**7.77**
**H8**	4.2708	4.3565	4.3850	4.4296	4.5207	4.4242	4.5487	4.4186	4.5249	4.4352	4.5617	4.4224	4.5811	4.6535	4.6574	4.7923	4.6535	4.8078	4.6492	4.7939	4.6612	4.8399	**5.21**
**H(9A-9C)**	1.2804	1.2719	1.2534	1.2437	1.2527	1.2451	1.2572	1.2462	1.2421	1.2423	1.2686	1.3599	1.4063	1.3877	1.4070	1.3888	1.4051	1.3782	1.4029	1.3767	1.4091	1.4090	**1.43**
**H(8`A-8`C)**	2.4504	2.4051	2.3680	2.3871	2.3633	2.3888	2.3347	2.3902	2.3449	2.3856	2.3256	2.8413	2.7264	2.6509	2.6181	2.5764	2.6219	2.4793	2.6255	2.4999	2.6147	2.4510	**2.58**
Nuclei	**B98**																						
**C1**	145.2431	145.5980	146.7950	146.3977	147.2239	146.4086	146.5088	146.4181	147.0144	146.3917	146.6730	144.1542	144.6542	145.8254	145.8226	146.9890	145.8402	147.4686	145.8538	147.3092	145.8062	147.4307	**136.1**
**C2**	116.9915	117.1179	117.9091	117.1859	117.8073	117.2097	118.2952	117.2307	118.2607	117.1658	118.3202	116.7168	116.8872	117.5941	117.0412	117.7268	117.0618	118.3354	117.0796	118.2141	117.0232	118.3310	**112.1**
**C3**	171.3612	171.6009	172.1331	171.6672	172.4253	171.6789	172.3790	171.6878	172.3846	171.6586	172.4389	171.3851	171.6106	172.1649	171.7162	172.4760	171.7272	172.4651	171.7354	172.4603	171.7082	172.5217	**162.6**
**C4**	112.4441	113.9792	114.5487	114.5843	115.3250	114.5562	116.2656	114.5254	115.9370	114.6128	116.4350	112.6999	114.0387	114.5880	114.4269	115.1536	114.4048	115.6603	114.3803	115.5245	114.4504	115.8801	**110.1**
**C5**	170.6107	171.1556	171.7356	171.4091	172.3104	171.4076	172.2359	171.4036	172.1912	171.4127	172.3156	170.8199	171.2728	171.7973	171.4455	172.2870	171.4471	172.1421	171.4462	172.1445	171.4464	172.2188	**162.5**
**C6**	115.1035	116.8800	117.8041	117.5537	119.3779	117.5086	119.7883	117.4605	119.4619	117.5980	120.1077	115.4563	117.3985	118.5192	118.0815	119.8712	118.0331	119.8560	117.9829	119.7633	118.1306	120.2467	**112.1**
**C7**	199.6940	204.6260	206.4252	207.8938	208.1597	207.7477	213.1306	207.6003	212.4048	208.0376	213.9202	203.9754	208.0267	209.4013	210.3396	211.1517	210.2463	216.3829	210.1489	215.2059	210.4288	216.7846	**194.5**
**C8**	65.6127	66.6919	67.4152	67.2716	67.9782	67.2638	68.5429	67.2525	68.5500	67.2808	69.2417	60.4790	61.8152	62.6691	63.0463	63.7590	63.0172	64.6701	62.9853	64.3898	63.0757	64.8913	**58.4**
**C9**	22.9777	23.2841	23.3017	23.3293	23.7525	23.3292	23.8171	23.3269	23.7341	23.3316	23.9675	19.7846	20.0685	20.2914	20.2802	20.5401	20.2810	20.5315	20.2795	20.4854	20.2816	20.8848	**15**
**C8`**	36.5987	37.3601	37.6605	37.7622	38.1220	37.7564	38.2519	37.7478	38.2238	37.7697	38.6612	32.0510	32.4888	32.5522	32.4601	32.8095	32.4597	32.8534	32.4570	32.8663	32.4630	32.9323	**30.6**
**H2**	7.9554	7.9125	7.9530	7.8065	7.9316	7.8092	7.9690	7.8119	7.9301	7.8040	7.9629	8.0028	7.9321	7.9532	7.7901	7.8731	7.7915	7.8110	7.7931	7.8271	7.7892	7.8264	**7.77**
**H4**	7.0511	7.2646	7.4038	7.3423	7.5295	7.3379	7.5718	7.3335	7.5526	7.3467	7.5935	7.0634	7.2646	7.4028	7.3312	7.5185	7.3274	7.5453	7.3236	7.5327	7.3351	7.5674	**7.71**
**H6**	7.4491	7.6755	7.7918	7.7533	7.9836	7.7462	8.0188	7.7392	7.9806	7.7603	8.0497	7.2724	7.4928	7.6349	7.5483	7.7990	7.5422	7.7725	7.5363	7.7658	7.5544	7.8214	**7.77**
**H8**	4.2495	4.3497	4.3666	4.4112	4.5056	4.4055	4.5336	4.4001	4.5108	4.4172	4.5540	4.3937	4.5607	4.6374	4.6394	4.7756	4.6351	4.7917	4.6308	4.7776	4.6438	4.8206	**5.21**
**H(9A-9C)**	1.3064	1.3078	1.2759	1.2671	1.2609	1.2678	1.2863	1.2685	1.2718	1.2665	1.3019	1.3874	1.4304	1.4109	1.4276	1.4088	1.4253	1.3976	1.4230	1.3968	1.4303	1.4263	**1.43**
**H(8`A-8`C)**	2.4759	2.4262	2.4014	2.4176	2.3734	2.4189	2.3654	2.4204	2.3768	2.4165	2.3488	2.8759	2.7574	2.6855	2.6540	2.6107	2.6571	2.5201	2.6605	2.5391	2.6513	2.4889	**2.58**

[a] methyl protons shifts are averaged
[b] according to [8]

Table S8. Cont. I

Nuclei	R											S											Exp. [b]
	**B971**																						
	Gas	$CHCl_3$ (PCM)	$CHCl_3$ (SMD)	DMSO (PCM)	DMSO (SMD)	CH_3OH (PCM)	CH_3OH (SMD)	C_2H_5OH (PCM)	C_2H_5OH (SMD)	H_2O (PCM)	H_2O (SMD)	Gas	$CHCl_3$ (PCM)	$CHCl_3$ (SMD)	DMSO (PCM)	DMSO (SMD)	CH_3OH (PCM)	CH_3OH (SMD)	C_2H_5OH (PCM)	C_2H_5OH (SMD)	H_2O (PCM)	H_2O (SMD)	
**C1**	144.7908	145.1093	146.3417	145.9190	146.7460	145.9246	146.1038	145.9281	146.5947	145.9188	146.2517	143.7231	144.2297	145.3982	145.3717	146.5184	145.3816	146.9613	145.3899	146.6704	145.3670	146.9021	**136.1**
**C2**	116.7182	116.8200	117.6086	116.8908	117.4976	116.9070	117.9891	116.9219	117.9558	116.8814	118.0112	116.4392	116.6069	117.2999	116.7533	117.4193	116.7665	118.0110	116.7787	117.8707	116.7465	118.0089	**112.1**
**C3**	171.2626	171.5182	172.0500	171.5966	172.3488	171.6000	172.3020	171.6029	172.3033	171.5993	172.3670	171.2881	171.5208	172.0750	171.6321	172.3860	171.6350	172.3728	171.6373	172.3660	171.6354	172.4332	**162.6**
**C4**	112.2224	113.7518	114.3155	114.3799	115.1007	114.3436	116.0188	114.3069	115.6983	114.4203	116.1951	112.4728	113.8137	114.3596	114.2176	114.9344	114.1873	115.4543	114.1563	115.3506	114.2526	115.6843	**110.1**
**C5**	170.4955	171.0524	171.6296	171.3271	172.2176	171.3169	172.1396	171.3062	172.0959	171.3428	172.2276	170.6965	171.1633	171.6896	171.3509	172.1897	171.3436	172.0494	171.3361	172.0515	171.3639	172.1303	**162.5**
**C6**	114.8922	116.6615	117.5659	117.3309	119.1494	117.2771	119.5178	117.2234	119.2033	117.3882	119.8532	115.2149	117.1499	118.2656	117.8768	119.6626	117.8203	119.6607	117.7636	119.6298	117.9365	120.0793	**112.1**
**C7**	199.5894	204.4154	206.2513	207.6529	207.9480	207.5030	212.8864	207.3513	212.1825	207.8003	213.6578	203.8543	207.8620	209.2559	210.1489	210.9593	210.0463	216.1016	209.9424	214.8453	210.2518	216.4761	**194.5**
**C8**	65.5762	66.6481	67.3645	67.1942	67.9166	67.1783	68.4640	67.1609	68.4666	67.2145	69.1705	60.4393	61.7639	62.5905	62.9625	63.6824	62.9274	64.5187	62.8912	64.2085	63.0012	64.7482	**58.4**
**C9**	22.9853	23.2974	23.3010	23.3515	23.7733	23.3449	23.8047	23.3383	23.7195	23.3645	23.9697	19.8190	20.1285	20.3131	20.3366	20.5942	20.3311	20.5734	20.3247	20.5386	20.3479	20.9544	**15**
**C8`**	36.6012	37.3492	37.6400	37.7561	38.0902	37.7416	38.2237	37.7265	38.1980	37.7754	38.6405	31.9953	32.4301	32.4867	32.3980	32.7264	32.3887	32.7809	32.3794	32.8055	32.4132	32.8620	**30.6**
**H2**	7.9539	7.9146	7.9531	7.8123	7.9347	7.8145	7.9671	7.8168	7.9300	7.8107	7.9621	7.9991	7.9313	7.9504	7.7906	7.8731	7.7917	7.8134	7.7931	7.8414	7.7901	7.8310	**7.77**
**H4**	7.0456	7.2571	7.3968	7.3361	7.5220	7.3315	7.5629	7.3269	7.5442	7.3407	7.5844	7.0581	7.2584	7.3958	7.3249	7.5109	7.3209	7.5379	7.3169	7.5260	7.3291	7.5599	**7.71**
**H6**	7.4531	7.6745	7.7878	7.7523	7.9803	7.7450	8.0129	7.7380	7.9760	7.7596	8.0444	7.2715	7.4906	7.6316	7.5491	7.8001	7.5425	7.7770	7.5361	7.7763	7.5558	7.8280	**7.77**
**H8**	4.2479	4.3439	4.3620	4.4093	4.5006	4.4038	4.5285	4.3985	4.5075	4.4150	4.5475	4.3980	4.5632	4.6392	4.6411	4.7758	4.6366	4.7954	4.6321	4.7814	4.6459	4.8239	**5.21**
**H(9A-9C)**	1.3073	1.3119	1.2785	1.2700	1.2649	1.2704	1.2855	1.2709	1.2716	1.2701	1.3028	1.3907	1.4321	1.4117	1.4321	1.4137	1.4296	1.4018	1.4272	1.4073	1.4349	1.4330	**1.43**
**H(8`A-8`C)**	2.4734	2.4226	2.3973	2.4131	2.3686	2.4142	2.3595	2.4154	2.3710	2.4124	2.3429	2.8697	2.7511	2.6789	2.6470	2.6044	2.6497	2.5157	2.6526	2.5369	2.6450	2.4840	**2.58**
Nuclei	**B972**																						
**C1**	141.8439	142.8654	143.1956	143.0833	144.1751	143.0638	143.9325	143.0439	143.6594	143.1008	144.1551	140.6755	141.1808	142.3711	142.5940	143.5923	142.5645	144.3190	142.5363	144.7296	142.6256	144.3994	**136.1**
**C2**	114.9470	115.1874	115.8039	115.1402	115.8049	115.1464	116.2043	115.1500	116.1383	115.1305	116.2403	114.6936	114.8345	115.5573	115.0816	115.7095	115.0796	116.2393	115.0757	116.1568	115.0802	116.2656	**112.1**
**C3**	168.2354	168.4487	168.9790	168.5864	169.2875	168.5793	169.2747	168.5699	169.2536	168.5889	169.3570	168.2697	168.4906	169.0340	168.6546	169.3476	168.6474	169.3309	168.6378	169.3265	168.6576	169.4098	**162.6**
**C4**	110.2761	111.6462	112.3831	112.4020	113.0320	112.3592	113.7895	112.3135	113.6999	112.4377	113.9584	110.5291	111.8474	112.3834	112.2213	112.9342	112.1896	113.3018	112.1543	112.9880	112.2447	113.5098	**110.1**
**C5**	167.4761	167.9585	168.5759	168.2716	169.1049	168.2552	168.9765	168.2362	168.9946	168.2821	169.0703	167.7028	168.1692	168.6883	168.3809	169.1709	168.3660	168.9739	168.3480	168.9279	168.3903	169.0750	**162.5**
**C6**	112.6701	114.1657	115.3979	115.1558	116.7553	115.0985	116.9985	115.0384	116.9119	115.2047	117.2955	113.0675	114.9703	116.0492	115.6347	117.3656	115.5827	117.2613	115.5267	116.9661	115.6770	117.5938	**112.1**
**C7**	197.4999	202.8944	203.8939	205.5273	205.9613	205.3584	211.1350	205.1890	209.8629	205.6872	211.9595	201.4696	205.4837	206.8409	207.8610	208.5232	207.7389	213.6605	207.6153	212.8596	207.9752	214.1411	**194.5**
**C8**	62.7349	63.9221	64.3656	64.4061	65.1057	64.3834	65.8092	64.3576	65.5035	64.4226	66.4974	57.7428	58.9731	59.8460	60.3582	60.9534	60.3032	62.0355	60.2478	62.0271	60.4098	62.2889	**58.4**
**C9**	21.9310	22.0409	22.2645	22.3171	22.6537	22.2988	22.6494	22.2782	22.6406	22.3302	22.8106	18.7661	18.9404	19.1733	19.2028	19.4546	19.1883	19.4725	19.1717	19.4320	19.2125	19.8083	**15**
**C8`**	35.5496	36.3363	36.5462	36.7280	37.0369	36.7090	37.1429	36.6870	37.1188	36.7414	37.5449	31.1103	31.6217	31.6970	31.6065	31.9556	31.5929	31.8398	31.5762	31.7355	31.6135	31.9205	**30.6**
**H2**	7.9209	7.8097	7.9281	7.7663	7.8571	7.7699	7.8452	7.7730	7.8812	7.7626	7.8379	7.9746	7.9072	7.9237	7.7502	7.8423	7.7540	7.7507	7.7572	7.7175	7.7457	7.7588	**7.77**
**H4**	7.0439	7.2494	7.3897	7.3322	7.5095	7.3276	7.5474	7.3228	7.5364	7.3363	7.5692	7.0587	7.2567	7.3900	7.3226	7.5036	7.3188	7.5267	7.3147	7.5080	7.3260	7.5491	**7.71**
**H6**	7.4491	7.6326	7.7901	7.7452	7.9493	7.7385	7.9561	7.7316	7.9657	7.7513	7.9864	7.2702	7.4888	7.6217	7.5390	7.7831	7.5341	7.7375	7.5287	7.7044	7.5432	7.7808	**7.77**
**H8**	4.2386	4.3183	4.3640	4.4014	4.4813	4.3949	4.4923	4.3885	4.4986	4.4076	4.5152	4.3860	4.5444	4.6100	4.6159	4.7492	4.6117	4.7586	4.6071	4.7357	4.6198	4.7886	**5.21**
**H(9A-9C)**	1.2591	1.2359	1.2374	1.2289	1.2091	1.2300	1.2221	1.2309	1.2235	1.2276	1.2364	1.3458	1.3850	1.3673	1.3874	1.3669	1.3857	1.3581	1.3839	1.3441	1.3886	1.3823	**1.43**
**H(8`A-8`C)**	2.4434	2.4060	2.3644	2.3902	2.3509	2.3910	2.3540	2.3916	2.3470	2.3894	2.3383	2.8346	2.7236	2.6516	2.6205	2.5775	2.6242	2.4780	2.6276	2.4784	2.6165	2.4472	**2.58**
Nuclei	**BHandH**																						
**C1**	146.7064	146.8721	147.2928	146.8977	148.0630	146.8813	146.9542	146.8840	147.0552	146.8842	147.1233	145.9642	146.2100	147.4073	146.9364	147.6805	146.9319	147.9625	146.9439	148.1084	146.9088	148.1326	**136.1**
**C2**	119.9100	119.9902	120.6706	119.8826	120.6318	119.8801	121.0134	119.8961	120.9247	119.8547	121.0659	119.6804	119.8548	120.6824	119.9626	120.7067	119.9596	121.2208	119.9742	121.1200	119.9351	121.2628	**112.1**
**C3**	172.2567	172.3929	172.8671	172.4554	173.0529	172.4410	173.0058	172.4449	173.0124	172.4392	173.0805	172.2586	172.3753	172.9182	172.4783	173.2224	172.4641	173.0850	172.4684	173.1016	172.4615	173.1634	**162.6**
**C4**	114.9759	116.6253	117.4413	117.4281	118.1524	117.3708	119.2122	117.3318	118.9792	117.4522	119.4304	115.1761	116.7063	117.4027	117.2929	118.0370	117.2369	118.7856	117.1995	118.5670	117.3164	118.9966	**110.1**
**C5**	171.2234	171.7595	172.3061	172.0950	172.7920	172.0630	172.7565	172.0494	172.7490	172.0957	172.8671	171.3805	171.8197	172.3516	172.0549	172.7990	172.0260	172.6367	172.0159	172.6366	172.0526	172.7433	**162.5**
**C6**	118.0817	120.0847	121.5048	121.0732	122.9831	121.0051	123.5080	120.9549	123.3004	121.1074	123.8619	118.3488	120.5725	121.7374	121.5071	122.2512	121.4335	123.7351	121.3783	123.5374	121.5472	124.0530	**112.1**
**C7**	202.7549	207.4807	208.5761	209.8995	210.5153	209.7392	215.1259	209.5972	214.0518	210.0219	215.9636	206.9694	210.8941	212.5542	213.0323	213.7764	212.8968	218.5207	212.7790	217.5757	213.1303	219.1415	**194.5**
**C8**	60.4789	61.6908	62.3385	62.1804	63.0803	62.1429	63.5213	62.1233	63.3957	62.1857	64.2664	54.6338	55.8086	56.5061	56.6895	57.4336	56.6501	58.0980	56.6282	57.9527	56.6959	58.4204	**58.4**
**C9**	22.0369	22.3206	22.6715	22.5066	23.0416	22.4845	22.9913	22.4810	22.9733	22.4980	23.1296	18.6708	18.9972	19.1888	19.2223	19.9664	19.2063	19.6216	19.2083	19.5967	19.2076	20.0390	**15**
**C8`**	33.6317	34.3952	34.6480	34.8072	35.1457	34.7713	35.3476	34.7537	35.3224	34.8108	35.8572	28.4775	29.0384	29.2076	29.1366	29.8807	29.0972	29.5714	29.0769	29.5099	29.1448	29.6949	**30.6**
**H2**	8.2482	8.1975	8.2915	8.1550	8.2278	8.1590	8.2797	8.1621	8.2848	8.1522	8.2777	8.2682	8.2009	8.2173	8.0945	8.0133	8.0967	8.1337	8.0983	8.1284	8.0935	8.1332	**7.77**
**H4**	7.1939	7.4165	7.5628	7.5004	7.6920	7.4966	7.7381	7.4920	7.7211	7.5049	7.7628	7.2009	7.4137	7.5595	7.4886	7.4074	7.4851	7.7155	7.4806	7.7001	7.4930	7.7396	**7.71**
**H6**	7.6590	7.8848	8.0543	7.9916	8.2135	7.9862	8.2796	7.9800	8.2645	7.9977	8.3180	7.4772	7.7169	7.8518	7.7932	7.7120	7.7870	8.0555	7.7800	8.0344	7.8002	8.0990	**7.77**
**H8**	4.0534	4.1514	4.1898	4.2256	4.2878	4.2212	4.3484	4.2162	4.3361	4.2309	4.3713	4.3154	4.4796	4.5678	4.5630	4.4818	4.5586	4.7339	4.5534	4.7180	4.5683	4.7644	**5.21**
**H(9A-9C)**	1.3835	1.3943	1.3965	1.3879	1.3775	1.3896	1.4024	1.3904	1.4000	1.3873	1.4191	1.4655	1.5053	1.4605	1.5036	1.4224	1.5031	1.4851	1.5017	1.4773	1.5052	1.5066	**1.43**
**H(8`A-8`C)**	2.5330	2.4697	2.4177	2.4412	2.4156	2.4434	2.3734	2.4449	2.3715	2.4401	2.3582	2.8714	2.7371	2.6691	2.6377	2.5565	2.6409	2.4994	2.6433	2.5150	2.6360	2.4756	**2.58**
Nuclei	**BHandHLYP**																						
**C1**	147.0971	147.2298	147.5497	147.9509	148.7783	147.9374	147.8318	147.6746	148.4558	147.9583	147.9533	146.0335	146.3909	147.4660	147.4217	148.4707	147.4315	148.7285	147.4424	148.5924	147.4106	148.7594	**136.1**
**C2**	119.5227	119.6680	120.3645	119.7178	120.3946	119.7218	120.8936	119.6850	120.8465	119.7126	120.9314	119.2874	119.5095	120.2517	119.6599	120.3547	119.6635	120.8804	119.6691	120.7781	119.6560	120.9149	**112.1**
**C3**	172.0357	172.0965	172.5046	172.1449	172.7200	172.1441	172.6262	172.1415	172.6767	172.1453	172.6940	172.0160	172.0722	172.5389	172.1617	172.7395	172.1606	172.7333	172.1617	172.7419	172.1625	172.7915	**162.6**
**C4**	114.2460	115.7960	116.5442	116.4361	117.1432	116.3942	118.1497	116.3910	117.7942	116.4760	118.3346	114.5222	115.8680	116.4472	116.2796	117.0009	116.2395	117.5558	116.2020	117.4273	116.3174	117.7716	**110.1**
**C5**	171.1374	171.5666	172.0569	171.8620	172.5659	171.8447	172.4706	171.8198	172.4596	171.8780	172.5571	171.2862	171.6155	172.0672	171.7872	172.4725	171.7739	172.3182	171.7633	172.3367	171.7998	172.4002	**162.5**
**C6**	117.3272	119.2703	120.5898	119.9846	121.8171	119.9249	122.2865	119.9810	121.9118	120.0429	122.6318	117.6757	119.7621	120.9231	120.5248	122.3432	120.4555	122.3658	120.3887	122.2931	120.5904	122.7478	**112.1**
**C7**	201.0448	206.1473	207.1844	209.5334	209.7078	209.3518	215.1195	208.8831	214.3153	209.7002	215.9252	205.5713	209.7144	211.0502	212.0244	212.6582	211.9099	218.2110	211.7969	216.9179	212.1326	218.7267	**194.5**
**C8**	61.2366	62.2261	62.8487	62.7281	63.4265	62.7023	63.8920	62.6593	63.9175	62.7503	64.5689	56.0892	57.3845	58.1792	58.5311	59.1898	58.4930	59.9860	58.4565	59.7307	58.5660	60.2602	**58.4**
**C9**	21.8618	22.0458	22.3029	22.0300	22.4413	22.0232	22.3578	22.0945	22.3095	22.0386	22.5029	18.6877	18.7881	18.9723	18.9484	19.1985	18.9403	19.1018	18.9340	19.0958	18.9554	19.4121	**15**
**C8`**	34.6600	35.4024	35.6884	35.8401	36.1743	35.8143	36.3317	35.7719	36.3122	35.8629	36.7499	30.4097	30.8430	30.9567	30.8479	31.2009	30.8274	31.2358	30.8099	31.2481	30.8677	31.3427	**30.6**
**H2**	8.1616	8.1166	8.2260	8.0090	8.1249	8.0131	8.1718	8.0399	8.1232	8.0055	8.1680	8.2094	8.1272	8.1512	7.9911	8.0749	7.9919	8.0109	7.9928	8.0299	7.9904	8.0210	**7.77**
**H4**	7.2191	7.4319	7.5736	7.5113	7.6944	7.5070	7.7432	7.5029	7.7211	7.5152	7.7653	7.2298	7.4303	7.5656	7.4969	7.6803	7.4930	7.7122	7.4889	7.6979	7.5005	7.7347	**7.71**
**H6**	7.5433	7.7826	7.9395	7.8725	8.0978	7.8654	8.1474	7.8646	8.1024	7.8793	8.1810	7.4025	7.6306	7.7763	7.6978	7.9515	7.6910	7.9310	7.6841	7.9240	7.7044	7.9778	**7.77**
**H8**	3.9839	4.1060	4.1399	4.1860	4.2819	4.1802	4.3259	4.1728	4.2989	4.1917	4.3440	4.1684	4.3384	4.4123	4.4170	4.5523	4.4122	4.5784	4.4074	4.5609	4.4215	4.6056	**5.21**
**H(9A-9C)**	1.2824	1.2858	1.2917	1.2441	1.2347	1.2456	1.2585	1.2613	1.2390	1.2430	1.2754	1.3564	1.3944	1.3718	1.3949	1.3723	1.3927	1.3657	1.3903	1.3658	1.3970	1.3897	**1.43**
**H(8`A-8`C)**	2.4352	2.3896	2.3432	2.3810	2.3361	2.3820	2.3282	2.3819	2.3409	2.3800	2.3164	2.8350	2.7097	2.6385	2.6113	2.5658	2.6140	2.4840	2.6167	2.5034	2.6089	2.4560	**2.58**

[a] methyl protons shifts are averaged

[b] according to [8]

Table S8. Cont. II

Nuclei	R											S											Exp. [b]
	BLYP																						
	Gas	$CHCl_3$ (PCM)	$CHCl_3$ (SMD)	DMSO (PCM)	DMSO (SMD)	CH_3OH (PCM)	CH_3OH (SMD)	C_2H_5OH (PCM)	C_2H_5OH (SMD)	H_2O (PCM)	H_2O (SMD)	Gas	$CHCl_3$ (PCM)	$CHCl_3$ (SMD)	DMSO (PCM)	DMSO (SMD)	CH_3OH (PCM)	CH_3OH (SMD)	C_2H_5OH (PCM)	C_2H_5OH (SMD)	H_2O (PCM)	H_2O (SMD)	
**C1**	145.7481	146.8706	147.3418	147.0888	147.9633	147.3352	147.2820	147.1276	147.7789	147.4947	147.5994	144.0788	144.8979	146.2644	146.1471	147.7877	146.4160	147.8386	146.2215	147.8771	146.5178	147.9227	**136.1**
**C2**	116.4574	116.7505	117.2905	116.6437	117.2313	116.9289	117.5743	116.7529	117.7128	117.0012	117.7019	115.9818	116.2054	116.9121	116.4173	117.1907	116.6996	117.6546	116.5197	117.7062	116.7762	117.7244	**112.1**
**C3**	175.3583	175.6542	176.2566	175.7443	176.7032	176.0181	176.4713	175.8298	176.6457	176.1111	176.6223	175.3884	175.7148	176.3291	175.8216	176.7719	176.0936	176.5261	175.9035	176.7097	176.1904	176.6780	**162.6**
**C4**	112.0104	113.3624	113.9696	114.0411	114.7335	114.2805	115.4764	114.0571	115.3784	114.4393	115.7255	112.4719	113.6058	114.0369	113.9156	114.5243	114.1606	114.9126	113.9436	114.9654	114.3100	115.2086	**110.1**
**C5**	174.6266	175.2069	175.9037	175.4811	176.6205	175.7455	176.3533	175.5468	176.4868	175.8551	176.5106	175.0554	175.5021	176.0823	175.6082	176.6294	175.8717	176.2473	175.6732	176.4509	175.9849	176.4108	**162.5**
**C6**	114.7550	116.2176	117.3879	117.1399	119.0051	117.3672	119.2124	117.1298	119.1127	117.5462	119.6123	115.1558	117.0394	118.0233	117.7093	119.3599	117.9323	119.3263	117.6933	119.3849	118.1247	119.7777	**112.1**
**C7**	203.7110	209.1298	210.3612	211.7992	212.3368	211.8863	216.6956	211.5228	216.1393	212.3623	217.6394	209.0453	212.6434	214.2845	214.7480	216.1069	214.9130	220.3152	214.6147	219.5577	215.2168	220.7642	**194.5**
**C8**	69.7611	71.0057	71.5907	71.4206	72.2656	71.6796	72.6969	71.4758	72.8563	71.8008	73.5243	63.7226	65.7810	66.6301	67.1532	68.0268	67.3851	68.7420	67.1549	68.6250	67.5601	69.0623	**58.4**
**C9**	23.5482	23.7279	23.9360	23.9672	24.4435	24.2302	24.4467	24.0298	24.5339	24.3418	24.6971	20.0053	20.5233	20.7078	20.7866	21.0712	21.0526	21.0558	20.8572	21.1539	21.1613	21.5792	**15**
**C8\`**	38.1020	38.9152	39.1223	39.2641	39.7206	39.5239	39.6339	39.3218	39.7846	39.6453	40.1395	33.5181	33.8937	33.9448	33.8367	34.2271	34.0980	34.0487	33.8976	34.2564	34.2155	34.1800	**30.6**
**H2**	7.7531	7.6581	7.7617	7.6106	7.7383	7.6627	7.9305	7.6216	7.7414	7.6604	7.9364	7.8508	7.7603	7.7641	7.6200	7.6750	7.6694	7.8109	7.6264	7.6568	7.6735	7.8356	**7.77**
**H4**	6.9112	7.1081	7.2442	7.1837	7.3660	7.2268	7.5731	7.1774	7.3888	7.2431	7.6067	6.9350	7.1191	7.2495	7.1764	7.3586	7.2202	7.5510	7.1714	7.3725	7.2351	7.5854	**7.71**
**H6**	7.3448	7.5275	7.6783	7.6355	7.8663	7.6765	8.0600	7.6248	7.8603	7.6967	8.1010	7.1617	7.3501	7.4740	7.3959	7.6214	7.4379	7.7712	7.3873	7.5975	7.4563	7.8297	**7.77**
**H8**	4.4798	4.5331	4.5649	4.5980	4.6856	4.6398	4.8698	4.5893	4.6804	4.6591	4.9043	4.5619	4.7117	4.7798	4.7860	4.9186	4.8294	5.1022	4.7801	4.9212	4.8452	5.1465	**5.21**
**H(9A-9C)**	1.3278	1.3077	1.3070	1.2962	1.2951	1.3455	1.4884	1.3018	1.3117	1.3491	1.5150	1.4064	1.4581	1.4321	1.4644	1.4361	1.5100	1.6147	1.4633	1.4427	1.5213	1.6575	**1.43**
**H(8\`A-8\`C)**	2.5630	2.5162	2.4786	2.4920	2.4524	2.5408	2.6127	2.4971	2.4539	2.5459	2.6061	3.0206	2.8759	2.8075	2.7694	2.7262	2.8200	2.8068	2.7782	2.6641	2.8216	2.7859	**2.58**
Nuclei	BMK																						
**C1**	162.7315	162.6727	163.4322	162.8365	164.3766	162.8053	163.3756	162.9856	163.0232	163.0268	163.6289	161.8248	162.1219	163.4819	163.3052	164.6111	163.2831	164.5881	163.4722	164.3198	163.4875	164.6967	**136.1**
**C2**	130.9957	130.8889	131.6701	130.6982	131.6464	130.6949	132.0539	130.9028	131.8371	130.8633	132.0636	130.8393	130.6982	131.4966	130.6425	131.5797	130.6357	132.1162	130.8401	131.8633	130.8104	132.0880	**112.1**
**C3**	187.2124	187.2749	187.9692	187.2900	188.2319	187.2740	188.1455	187.4691	188.0715	187.4668	188.1443	187.2458	187.2726	187.8942	187.2019	188.1463	187.1854	188.0705	187.3805	187.9725	187.3790	188.1003	**162.6**
**C4**	126.1275	127.6104	128.4605	128.2640	129.1944	128.2109	130.1213	128.3686	129.8697	128.4757	130.2526	126.3061	127.5785	128.2780	127.9476	128.9377	127.8961	129.5448	128.0559	129.3015	128.1578	129.6499	**110.1**
**C5**	186.2743	186.7477	187.4664	186.9863	188.0708	186.9555	187.9481	187.1363	187.8597	187.1772	188.0091	186.2210	186.5854	187.2395	186.8041	187.7969	186.7739	187.6214	186.9550	187.5197	186.9945	187.6170	**162.5**
**C6**	129.0780	131.0787	132.3496	131.9671	133.9931	131.8970	134.3492	132.0379	134.0524	132.1953	134.6750	129.2190	131.2344	132.7079	132.1544	134.3901	132.0817	134.6006	132.2204	134.4055	132.3848	134.8976	**112.1**
**C7**	216.7020	221.9096	223.5339	224.8278	225.7871	224.6304	231.3580	224.6448	229.8414	225.1759	232.3444	221.0110	225.6175	227.2602	228.3265	229.1189	228.1640	234.7506	228.2115	233.2768	228.6421	235.1988	**194.5**
**C8**	71.1897	72.2890	72.9929	72.8023	73.8837	72.7579	74.3671	72.9259	74.0597	73.0068	75.0631	65.2505	66.5369	67.7039	67.9007	68.8828	67.8484	69.7076	68.0074	69.3153	68.1119	70.0736	**58.4**
**C9**	25.2414	25.5009	25.8219	25.6540	26.2407	25.6246	26.2211	25.8058	26.0413	25.8433	26.3165	21.9800	21.9981	22.5167	22.3986	22.9018	22.3830	22.7306	22.5785	22.7129	22.5750	23.2128	**15**
**C8\`**	38.8135	39.5255	39.7964	39.9061	40.4240	39.8777	40.5014	40.0610	40.3642	40.0952	40.9021	33.6850	34.1404	34.3964	34.3149	34.7561	34.2765	34.7112	34.4489	34.6334	34.5129	34.8165	**30.6**
**H2**	8.2738	8.2592	8.3378	8.2067	8.2816	8.2073	8.3336	8.2078	8.3546	8.1967	8.3272	8.3152	8.2376	8.2501	8.1116	8.1851	8.1102	8.1497	8.1088	8.1712	8.1032	8.1653	**7.77**
**H4**	7.3357	7.5509	7.6898	7.6330	7.8183	7.6254	7.8583	7.6178	7.8443	7.6305	7.8840	7.3302	7.5358	7.6733	7.6128	7.7954	7.6054	7.8212	7.5980	7.8111	7.6102	7.8394	**7.71**
**H6**	7.7226	7.9733	8.1216	8.0758	8.3049	8.0671	8.3433	8.0584	8.3341	8.0746	8.3824	7.5361	7.7448	7.9351	7.8786	8.1407	7.8688	8.1259	7.8589	8.1358	7.8784	8.1323	**7.77**
**H8**	4.1465	4.2490	4.3025	4.3121	4.4089	4.3042	4.4414	4.2963	4.4517	4.3100	4.4603	4.3736	4.5403	4.6411	4.6470	4.7854	4.6391	4.7838	4.6312	4.7905	4.6450	4.7720	**5.21**
**H(9A-9C)**	1.2990	1.3318	1.3084	1.3230	1.2855	1.3203	1.3117	1.3177	1.3166	1.3161	1.3255	1.3588	1.3981	1.3775	1.4016	1.3840	1.3974	1.4004	1.3933	1.4002	1.3961	1.4166	**1.43**
**H(8\`A-8\`C)**	2.4247	2.3701	2.3247	2.3683	2.3202	2.3649	2.3052	2.3616	2.2913	2.3620	2.2928	2.7914	2.6684	2.5947	2.5685	2.5254	2.5674	2.4314	2.5663	2.4700	2.5602	2.3779	**2.58**
Nuclei	BP86																						
**C1**	142.7538	143.4513	144.5775	144.1907	145.0356	144.1952	144.5412	144.1946	144.9194	144.1825	144.8732	141.4617	142.1656	143.3316	143.3152	144.4516	143.3270	144.8687	143.3320	144.4752	143.2976	144.9621	**136.1**
**C2**	114.9274	115.1445	115.8552	115.1479	115.6876	115.1686	116.1738	115.1842	116.1359	115.1227	116.3537	114.5927	114.8280	115.4815	114.9951	115.5581	115.0133	116.2025	115.0258	116.0166	114.9724	116.3334	**112.1**
**C3**	172.0493	172.5220	173.0745	172.5605	173.3928	172.5656	173.3561	172.5656	173.3351	172.5504	173.5903	172.0582	172.5221	173.0948	172.5909	173.4087	172.5955	173.3829	172.5948	173.3705	172.5815	173.6099	**162.6**
**C4**	110.9860	112.6318	113.1453	113.2279	113.8562	113.1935	114.7930	113.1540	114.4847	113.2553	115.1289	111.3231	112.7636	113.2292	113.0964	113.7399	113.0694	114.3116	113.0376	114.2126	113.1172	114.7015	**110.1**
**C5**	171.2599	172.0320	172.6542	172.2838	173.2497	172.2751	173.2009	172.2610	173.1351	172.2874	173.4429	171.5908	172.2342	172.7533	172.3093	173.2108	172.3048	173.0358	172.2953	173.0464	172.3088	173.2758	**162.5**
**C6**	113.1764	114.9405	115.8289	115.5842	117.3085	115.5368	117.6679	115.4840	117.3720	115.6244	118.1684	113.5099	115.5241	116.6130	116.2054	117.9367	116.1546	118.0325	116.0990	118.0123	116.2490	118.6108	**112.1**
**C7**	200.8740	205.6351	207.3718	208.5818	208.9794	208.4408	213.3927	208.2958	212.7636	208.7118	214.2723	205.4772	209.2841	210.5621	211.2242	212.0901	211.1381	216.5337	211.0452	215.3777	211.3010	216.9615	**194.5**
**C8**	67.7193	68.9807	69.6770	69.4781	70.2197	69.4688	70.7818	69.4533	70.7615	69.4808	71.7372	62.1228	63.6359	64.5221	64.9077	65.6311	64.8730	66.4515	64.8324	66.1235	64.9349	66.8802	**58.4**
**C9**	23.2849	23.7487	23.7013	23.7870	24.1448	23.7815	24.2820	23.7712	24.1688	23.7878	24.6409	19.9380	20.4850	20.7072	20.7855	21.0266	20.7754	21.1255	20.7599	21.0785	20.7900	21.8011	**15**
**C8\`**	37.3382	38.2084	38.4190	38.5638	38.8090	38.5521	38.9623	38.5346	38.9321	38.5694	39.5682	32.5225	33.0970	33.0398	32.9133	33.1895	32.9082	33.2254	32.8983	33.2582	32.9138	33.4315	**30.6**
**H2**	7.7724	7.7317	7.7645	7.6369	7.7426	7.6403	7.7694	7.6433	7.7390	7.6332	7.7790	7.8354	7.7769	7.7834	7.6413	7.7099	7.6432	7.6606	7.6450	7.6943	7.6393	7.6912	**7.77**
**H4**	6.8897	7.1017	7.2283	7.1789	7.3524	7.1752	7.3924	7.1712	7.3734	7.1819	7.4276	6.9069	7.1061	7.2290	7.1689	7.3440	7.1659	7.3699	7.1625	7.3576	7.1714	7.4058	**7.71**
**H6**	7.3735	7.5819	7.6754	7.6507	7.8531	7.6444	7.8854	7.6378	7.8495	7.6563	7.9286	7.1622	7.3706	7.4969	7.4202	7.6575	7.4148	7.6321	7.4092	7.6377	7.4250	7.7005	**7.77**
**H8**	4.5068	4.5848	4.5783	4.6315	4.6945	4.6277	4.7184	4.6238	4.6969	4.6350	4.7524	4.6215	4.7696	4.8319	4.8493	4.9678	4.8460	4.9887	4.8425	4.9722	4.8520	5.0351	**5.21**
**H(9A-9C)**	1.3547	1.3540	1.3166	1.3196	1.3050	1.3212	1.3244	1.3224	1.3114	1.3176	1.3578	1.4375	1.4867	1.4630	1.4945	1.4687	1.4928	1.4670	1.4910	1.4739	1.4957	1.5196	**1.43**
**H(8\`A-8\`C)**	2.5474	2.4876	2.4474	2.4694	2.4179	2.4719	2.4031	2.4741	2.4129	2.4668	2.3990	2.9606	2.8361	2.7475	2.7252	2.6672	2.7293	2.5745	2.7333	2.5980	2.7211	2.5556	**2.58**
Nuclei	CAM-B3LYP																						
**C1**	148.4624	148.6841	149.5577	149.3093	150.3034	149.2366	149.5630	149.1940	150.1230	149.4908	149.7081	147.3433	147.7962	148.9027	148.9377	149.9895	148.9426	150.6402	148.9472	150.3560	148.9346	150.6085	**136.1**
**C2**	120.3697	120.5288	121.2664	120.5450	121.2654	120.5429	121.7675	120.5459	121.7171	120.5678	121.7990	120.0897	120.3536	121.0773	120.5409	121.2386	120.5481	121.8243	120.5558	121.6912	120.5360	121.8482	**112.1**
**C3**	175.3492	175.4926	175.9358	175.5369	176.1861	175.5384	176.1153	175.5405	176.1333	175.5379	176.1841	175.3442	175.4726	175.9346	175.5461	176.1974	175.5484	176.2002	175.5514	176.1881	175.5461	176.2573	**162.6**
**C4**	115.1853	116.7203	117.3027	117.3420	118.0574	117.3114	118.9781	117.2787	118.6454	117.3619	119.1496	115.4504	116.8025	117.3467	117.1829	117.9449	117.1505	118.3719	117.1183	118.2754	117.2157	118.6005	**110.1**
**C5**	174.4445	174.9458	175.4191	175.2281	176.0054	175.2117	175.9367	175.1974	175.9002	175.2501	176.0203	174.6122	175.0145	175.4513	175.1606	175.9162	175.1514	175.7560	175.1425	175.7605	175.1717	175.8379	**162.5**
**C6**	118.2349	120.2035	121.2809	120.9421	122.7939	120.9171	123.1401	120.8794	122.7903	120.9217	123.4948	118.5853	120.7015	121.8580	121.4563	123.3720	121.3956	123.2336	121.3353	123.1908	121.5160	123.6524	**112.1**
**C7**	204.9813	210.0516	211.5270	213.2288	213.7015	212.9808	218.9767	212.7744	218.2644	213.6165	219.7880	209.5376	213.7156	215.0999	216.0406	216.7885	215.9258	222.3449	215.8104	221.0486	216.1513	222.7788	**194.5**
**C8**	64.0306	65.1714	65.8913	65.6727	66.4716	65.6559	66.9160	65.6362	66.9212	65.6891	67.6537	58.5409	59.9286	60.7324	61.1462	61.8646	61.1083	62.7669	61.0705	62.4393	61.1839	63.0463	**58.4**
**C9**	22.0699	22.3268	22.4756	22.3917	22.7878	22.4051	22.6785	22.4083	22.6005	22.3384	22.8481	19.0012	19.1778	19.3019	19.3324	19.6057	19.3296	19.4870	19.3270	19.4631	19.3369	19.8518	**15**
**C8\`**	35.4169	36.2356	36.4687	36.6181	36.9937	36.5948	37.1573	36.5744	37.1322	36.6513	37.6150	30.8785	31.3840	31.4431	31.3509	31.7242	31.3347	31.7233	31.3192	31.7367	31.3691	31.8575	**30.6**
**H2**	8.0798	8.0390	8.0961	7.9447	8.0515	7.9541	8.0852	7.9610	8.0417	7.9264	8.0787	8.1333	8.0587	8.0806	7.9149	8.0062	7.9164	7.9131	7.9182	7.9416	7.9134	7.9290	**7.77**
**H4**	7.1631	7.3729	7.5130	7.4499	7.6374	7.4457	7.6793	7.4416	7.6601	7.4539	7.7001	7.1734	7.3716	7.5092	7.4358	7.6246	7.4321	7.6486	7.4284	7.6373	7.4393	7.6708	**7.71**
**H6**	7.5236	7.7575	7.8789	7.8388	8.0671	7.8338	8.1068	7.8287	8.0661	7.8423	8.1386	7.3719	7.5974	7.7446	7.6580	7.9202	7.6515	7.8749	7.6451	7.8763	7.6641	7.9251	**7.77**
**H8**	4.1228	4.2306	4.2498	4.3082	4.4012	4.3011	4.4473	4.2954	4.4231	4.3181	4.4637	4.3004	4.4681	4.5457	4.5464	4.6862	4.5418	4.7032	4.5373	4.6904	4.5507	4.7319	**5.21**
**H(9A-9C)**	1.3143	1.3232	1.3076	1.2895	1.2794	1.2947	1.2903	1.2979	1.2735	1.2776	1.3088	1.3899	1.4312	1.4104	1.4309	1.4163	1.4289	1.3952	1.4270	1.3990	1.4327	1.4240	**1.43**
**H(8\`A-8\`C)**	2.5060	2.4548	2.4325	2.4448	2.4053	2.4460	2.3904	2.4471	2.4032	2.4427	2.3783	2.8981	2.7725	2.6987	2.6658	2.6251	2.6687	2.5302	2.6719	2.5552	2.6629	2.5016	**2.58**

[a] methyl protons shifts are averaged

[b] according to [8]

Table S8. Cont. III

Nuclei	R											S											Exp. [b]
	**HF**																						
	Gas	$CHCl_3$ (PCM)	$CHCl_3$ (SMD)	DMSO (PCM)	DMSO (SMD)	CH_3OH (PCM)	CH_3OH (SMD)	C_2H_5OH (PCM)	C_2H_5OH (SMD)	H_2O (PCM)	H_2O (SMD)	Gas	$CHCl_3$ (PCM)	$CHCl_3$ (SMD)	DMSO (PCM)	DMSO (SMD)	CH_3OH (PCM)	CH_3OH (SMD)	C_2H_5OH (PCM)	C_2H_5OH (SMD)	H_2O (PCM)	H_2O (SMD)	
**C1**	147.7121	148.1462	148.1492	149.1412	149.0104	149.0759	149.1926	148.9499	149.6027	149.1850	149.3437	146.2560	146.4572	148.9780	149.1791	150.1652	149.1278	149.7021	149.0849	150.0837	149.2368	149.9227	**136.1**
**C2**	118.3809	118.6300	119.3684	118.5413	119.3777	118.5527	119.7793	118.5693	119.6702	118.5314	119.8114	118.2524	118.5590	119.3010	118.4610	119.3758	118.4680	119.7023	118.4743	119.5755	118.4525	119.7337	**112.1**
**C3**	168.2802	168.1502	168.3945	168.1534	168.4870	168.1530	168.4813	168.1468	168.5414	168.1515	168.5428	168.1871	168.0226	168.5400	168.2712	168.6623	168.2682	168.5933	168.2665	168.6476	168.2748	168.6620	**162.6**
**C4**	112.6244	114.0962	114.9583	114.5391	115.5871	114.5192	116.2181	114.5177	115.8932	114.5620	116.3700	112.9802	114.3797	114.5193	114.1872	114.9663	114.1677	115.5798	114.1457	115.2770	114.2015	115.7131	**110.1**
**C5**	167.2757	167.5153	167.8482	167.7332	168.2463	167.7202	168.1164	167.7050	168.1379	167.7436	168.2049	167.2550	167.4221	167.8461	167.6776	168.1690	167.6656	167.9812	167.6546	168.0061	167.6890	168.0711	**162.5**
**C6**	115.6813	117.5848	118.9797	118.2479	120.4077	118.2059	120.3553	118.1894	120.0329	118.2948	120.6350	116.2984	118.4587	118.7549	118.2796	120.0365	118.2485	120.2939	118.2135	119.9941	118.3031	120.5345	**112.1**
**C7**	195.0864	201.1974	202.1057	205.1486	204.3050	204.9023	211.8991	204.6015	210.6015	205.3645	212.8018	199.0531	203.6043	205.9457	207.2282	207.6496	207.0438	213.5072	206.8640	212.2390	207.4078	214.2805	**194.5**
**C8**	55.1031	56.1200	56.4604	56.7648	57.0692	56.7164	57.8833	56.6498	57.7961	56.8040	58.4563	50.5284	51.7328	52.8831	53.2938	53.8474	53.2468	54.6595	53.2029	54.5809	53.3414	54.9739	**58.4**
**C9**	20.2409	20.1666	20.3679	20.1759	20.6108	20.1760	20.3164	20.1834	20.3224	20.1785	20.3997	17.4792	17.3170	17.3609	17.2432	17.5132	17.2524	17.4287	17.2617	17.4510	17.2335	17.6278	**15**
**C8`**	32.6404	33.3530	33.5761	33.6875	34.0417	33.6635	34.1472	33.6358	34.1005	33.7070	34.5026	28.8853	29.2953	29.2756	29.2181	29.5394	29.2016	29.5492	29.1854	29.4937	29.2326	29.6273	**30.6**
**H2**	8.1484	8.0454	8.1890	7.9006	8.1149	7.9103	8.0336	7.9258	8.0133	7.8932	8.0280	8.2369	8.1426	8.0199	7.8305	7.9414	7.8371	7.9338	7.8428	7.9118	7.8235	7.9287	**7.77**
**H4**	7.2655	7.4862	7.6373	7.5616	7.7619	7.5579	7.7990	7.5545	7.7775	7.5652	7.8213	7.2777	7.4892	7.6158	7.5371	7.7287	7.5336	7.7595	7.5297	7.7393	7.5404	7.7810	**7.71**
**H6**	7.4308	7.6757	7.8611	7.7642	8.0522	7.7593	8.0401	7.7569	8.0074	7.7697	8.0738	7.3542	7.6087	7.6943	7.6137	7.8665	7.6093	7.8640	7.6044	7.8341	7.6174	7.8978	**7.77**
**H8**	3.5241	3.6685	3.7491	3.7576	3.9052	3.7528	3.9202	3.7502	3.8944	3.7630	3.9383	3.7343	3.9245	3.9760	3.9636	4.1151	3.9591	4.1356	3.9542	4.1100	3.9676	4.1523	**5.21**
**H(9A-9C)**	1.1624	1.1432	1.1461	1.1019	1.1090	1.1042	1.0923	1.1080	1.0841	1.1003	1.1037	1.2474	1.2758	1.1946	1.2212	1.1918	1.2213	1.2139	1.2210	1.2036	1.2209	1.2251	**1.43**
**H(8`A-8`C)**	2.2753	2.2539	2.2078	2.2558	2.1999	2.2553	2.2229	2.2539	2.2248	2.2562	2.2153	2.6485	2.5366	2.4501	2.4179	2.3834	2.4206	2.3149	2.4231	2.3181	2.4152	2.2848	**2.58**
Nuclei	**LC-BLYP**																						
**C1**	153.0659	152.9932	153.9478	153.2994	154.4610	153.2671	153.6006	153.2592	154.4471	153.3100	153.6985	152.0909	152.3376	153.5064	153.5214	154.4478	153.4803	155.3938	153.4680	154.4876	153.5446	155.3545	**136.1**
**C2**	125.1117	125.2547	126.1171	125.2424	126.0930	125.2238	126.5824	125.2320	126.5273	125.2427	126.6298	124.8423	125.1096	125.9858	125.3336	126.1148	125.3094	126.6441	125.3129	126.5069	125.3393	126.6964	**112.1**
**C3**	179.4625	179.3659	179.8001	179.3978	179.9041	179.3751	179.8505	179.3795	179.8994	179.4019	179.9048	179.4596	179.3560	179.8147	179.4436	179.9341	179.4199	180.0140	179.4236	179.9283	179.4489	180.0556	**162.6**
**C4**	119.5922	121.1275	121.8706	121.8511	122.6302	121.7861	123.5400	121.7485	123.1661	121.8957	123.7389	119.7976	121.1785	121.8792	121.6443	122.5102	121.5858	122.8575	121.5539	122.8882	121.6819	123.0895	**110.1**
**C5**	178.4282	178.7477	179.2235	179.0477	179.6740	179.0069	179.6143	178.9933	179.6084	179.0693	179.6988	178.5367	178.7820	179.2208	178.9749	179.5914	178.9375	179.4631	178.9275	179.4263	178.9932	179.5311	**162.5**
**C6**	122.6656	124.8212	126.0160	125.7973	127.6946	125.7145	128.0371	125.6606	127.5187	125.8591	128.4352	123.0495	125.2950	126.5670	126.1254	128.2068	126.0458	127.9644	125.9928	128.2584	126.1828	128.4251	**112.1**
**C7**	210.1898	215.3210	216.9461	218.2917	218.9503	218.0958	224.3773	217.9244	223.8948	218.4584	225.2030	214.4768	218.7950	220.4137	221.4119	222.1020	221.2386	228.1827	221.0926	226.1958	221.5591	228.6369	**194.5**
**C8**	61.8392	63.0647	63.7969	63.6198	64.5104	63.5665	64.9590	63.5405	64.8427	63.6531	65.6885	56.1591	57.4696	58.2939	58.6291	59.3491	58.5642	60.3658	58.5274	59.9644	58.6739	60.7091	**58.4**
**C9**	21.0891	21.2058	21.4708	21.4307	21.8569	21.3946	21.6607	21.3855	21.4620	21.4473	21.8487	18.2806	18.3535	18.4911	18.4804	18.7770	18.4595	18.6189	18.4658	18.7216	18.4825	19.0174	**15**
**C8`**	33.5289	34.3585	34.6715	34.7962	35.2003	34.7498	35.3864	34.7313	35.3205	34.8234	35.8886	28.9379	29.4680	29.6380	29.5517	29.9040	29.5046	29.8876	29.4849	29.9104	29.5789	30.0694	**30.6**
**H2**	8.2569	8.2183	8.2766	8.1508	8.2350	8.1534	8.2673	8.1580	8.2074	8.1476	8.2684	8.2975	8.2232	8.2466	8.0788	8.1757	8.0813	8.0491	8.0853	8.1311	8.0754	8.0660	**7.77**
**H4**	7.3112	7.5223	7.6691	7.6022	7.7950	7.5970	7.8358	7.5933	7.8167	7.6061	7.8596	7.3178	7.5189	7.6634	7.5867	7.7812	7.5819	7.8014	7.5785	7.7933	7.5903	7.8256	**7.71**
**H6**	7.6561	7.9003	8.0344	8.0002	8.2277	7.9930	8.2620	7.9876	8.2108	8.0061	8.3036	7.5204	7.7594	7.9103	7.8231	8.0993	7.8161	8.0347	7.8106	8.0708	7.8287	8.0877	**7.77**
**H8**	3.9976	4.1071	4.1478	4.1990	4.2868	4.1918	4.3340	4.1861	4.3243	4.2049	4.3563	4.2244	4.3979	4.4877	4.4818	4.6295	4.4756	4.6345	4.4709	4.6231	4.4866	4.6627	**5.21**
**H(9A-9C)**	1.3288	1.3469	1.3244	1.3317	1.3134	1.3316	1.3208	1.3332	1.2881	1.3309	1.3443	1.4005	1.4383	1.4092	1.4335	1.4191	1.4317	1.3878	1.4315	1.4220	1.4340	1.4200	**1.43**
**H(8`A-8`C)**	2.4935	2.4373	2.4110	2.4286	2.3914	2.4284	2.3746	2.4297	2.3874	2.4278	2.3644	2.8450	2.7166	2.6464	2.6119	2.5768	2.6138	2.4655	2.6173	2.4997	2.6090	2.4403	**2.58**
Nuclei	**M06**																						
**C1**	143.6087	143.8341	144.1560	144.1466	145.4179	144.0943	144.6247	144.0567	145.2393	144.2187	144.7782	142.5659	142.8141	144.0659	144.3025	145.2584	144.2758	146.2582	144.2587	146.0881	144.3454	146.1763	**136.1**
**C2**	117.4969	117.6906	118.4694	117.6902	118.5084	117.6767	119.0643	117.6730	119.0111	117.7191	119.0861	117.3658	117.4199	118.3149	117.9277	118.6176	117.9154	119.5130	117.9116	119.3533	117.9549	119.4995	**112.1**
**C3**	171.0655	171.1576	171.6496	171.2572	172.0725	171.2405	171.9445	171.2313	172.0207	171.2873	172.0360	171.0875	171.2518	171.8988	171.4330	172.2147	171.4186	172.2217	171.4124	172.2255	171.4612	172.2612	**162.6**
**C4**	112.9084	114.5496	115.4222	115.3123	116.1173	115.2612	117.1351	115.2171	116.7511	115.3749	117.3127	112.9794	114.3851	115.0462	114.7549	115.6813	114.7084	116.0940	114.6689	115.9579	114.8129	116.3443	**110.1**
**C5**	170.4005	170.8302	171.5303	171.1355	172.0720	171.1040	172.0447	171.0813	171.9917	171.1814	172.0878	170.6007	170.8887	171.4197	170.9603	171.8434	170.9364	171.6630	170.9201	171.6715	170.9975	171.7353	**162.5**
**C6**	115.4653	117.3110	118.7542	118.2416	120.0756	118.1830	120.5423	118.1300	120.1478	118.3092	120.8598	116.0240	118.1108	119.3577	118.7529	120.8129	118.6840	120.6537	118.6219	120.5518	118.8320	121.0971	**112.1**
**C7**	201.9616	206.6108	207.7146	209.1984	209.8619	209.0015	214.6669	208.8227	214.1517	209.4126	215.4547	206.3552	210.2643	211.5991	212.2398	212.9368	212.1160	218.1946	211.9993	217.0620	212.3726	218.5362	**194.5**
**C8**	61.6157	62.7862	63.4448	63.1823	64.2103	63.1600	64.6298	63.1425	64.5045	63.2151	65.3923	54.7279	55.9736	56.9908	57.5120	58.3429	57.4505	59.2905	57.3979	58.9831	57.5862	59.6495	**58.4**
**C9**	22.0089	22.3738	22.7469	22.5355	23.0391	22.5242	23.0830	22.5175	22.8484	22.5571	23.1318	19.0663	19.1434	19.4193	19.5446	20.0187	19.5245	19.7995	19.5119	19.7729	19.5782	20.3394	**15**
**C8`**	36.6161	37.4384	37.6918	37.9406	38.3867	37.8952	38.6169	37.8602	38.6376	38.0014	39.1063	31.7384	32.2819	32.4719	32.2631	32.7052	32.2332	32.9206	32.2113	32.8556	32.3066	33.0530	**30.6**
**H2**	7.9698	7.9068	8.0191	7.8235	7.9100	7.8307	7.9337	7.8378	7.8931	7.8167	7.9258	8.0414	7.9552	8.0009	7.8337	7.9444	7.8368	7.8456	7.8403	7.8625	7.8311	7.8545	**7.77**
**H4**	6.9321	7.1543	7.3096	7.2320	7.4279	7.2277	7.4765	7.2239	7.4533	7.2368	7.4990	6.8809	7.0866	7.2281	7.1530	7.3512	7.1488	7.3800	7.1451	7.3652	7.1575	7.4064	**7.71**
**H6**	7.4835	7.7066	7.8772	7.8004	8.0089	7.7948	8.0705	7.7901	8.0210	7.8066	8.0863	7.3031	7.5132	7.6395	7.4952	7.7803	7.4896	7.6863	7.4846	7.6846	7.5008	7.7551	**7.77**
**H8**	4.2773	4.3549	4.3740	4.4090	4.4769	4.4049	4.5198	4.4017	4.4988	4.4137	4.5316	4.3646	4.5231	4.5871	4.5656	4.7090	4.5613	4.6783	4.5576	4.6733	4.5702	4.7342	**5.21**
**H(9A-9C)**	1.3212	1.3089	1.3280	1.2793	1.2697	1.2828	1.2807	1.2863	1.2576	1.2759	1.2984	1.3636	1.4097	1.3879	1.3910	1.3879	1.3895	1.3416	1.3885	1.3449	1.3929	1.3814	**1.43**
**H(8`A-8`C)**	2.4391	2.4091	2.3648	2.3976	2.3659	2.3979	2.3504	2.3988	2.3614	2.3977	2.3353	2.8564	2.7442	2.6579	2.5989	2.5664	2.6026	2.4555	2.6069	2.4814	2.5958	2.4296	**2.58**
Nuclei	**M06-2X**																						
**C1**	160.6475	160.9144	161.4815	161.0437	162.3465	161.0480	161.3271	161.0554	161.2325	161.0351	161.6236	160.0712	160.3972	161.6524	161.6045	162.7588	161.6050	162.9854	161.6083	162.7446	161.5997	163.2824	**136.1**
**C2**	130.9181	130.9182	131.5635	130.7532	131.4908	130.7852	131.8866	130.8187	131.7769	130.7164	131.9578	130.9607	130.9962	131.7658	131.0960	131.8330	131.1223	132.3766	131.1498	132.2266	131.0652	132.4467	**112.1**
**C3**	184.9329	185.0581	185.5654	185.0935	185.7983	185.1120	185.7543	185.1319	185.7356	185.0696	185.8423	184.9790	185.0980	185.6365	185.1575	185.8625	185.1752	185.8343	185.1940	185.8270	185.1345	185.9251	**162.6**
**C4**	125.6445	127.2128	127.9809	127.9383	128.7259	127.9178	129.7092	127.8979	129.5080	127.9512	129.9048	125.6848	127.1393	127.7953	127.6456	128.4840	127.6290	129.1001	127.6132	128.9477	127.6544	129.2943	**110.1**
**C5**	183.7556	184.2929	184.8831	184.6107	185.4542	184.6110	185.4231	184.6127	185.3957	184.6043	185.5193	183.8092	184.3112	184.8262	184.4932	185.3302	184.4954	185.1394	184.4991	185.1480	184.4848	185.2539	**162.5**
**C6**	129.0692	131.0857	132.4045	132.0845	134.0470	132.0477	134.4655	132.0110	134.2846	132.1126	134.8331	129.3298	131.4863	132.7479	132.3374	134.4293	132.3041	134.6571	132.2714	134.5460	132.3614	134.9566	**112.1**
**C7**	217.9685	223.3424	224.6748	226.1066	226.8183	225.9537	232.2546	225.8038	230.9246	226.2471	233.3156	221.9367	226.6882	228.3374	229.4086	230.1664	229.2713	235.8710	229.1354	234.4798	229.5329	236.7275	**194.5**
**C8**	66.7616	68.0409	68.6275	68.5924	69.5352	68.5805	69.9392	68.5696	69.7162	68.5968	70.7665	60.9624	62.2785	63.1475	63.4975	64.3331	63.4745	65.2376	63.4535	64.9234	63.5138	65.6052	**58.4**
**C9**	22.7521	23.1173	23.3932	23.4185	23.9222	23.4153	23.9593	23.4145	23.8682	23.4162	24.1436	19.9903	20.1564	20.3508	20.4075	20.7366	20.4268	20.7761	20.4472	20.7172	20.3830	21.1365	**15**
**C8`**	36.0489	36.6336	36.8478	36.9812	37.3318	36.9812	37.4719	36.9822	37.4087	36.9742	37.9825	30.6120	31.2137	31.4167	31.4523	31.7885	31.4526	31.8112	31.4542	31.7986	31.4457	32.0585	**30.6**
**H2**	8.5068	8.4519	8.5399	8.4036	8.4745	8.4062	8.5326	8.4083	8.5494	8.4011	8.5167	8.5232	8.4741	8.5005	8.3533	8.4358	8.3551	8.3895	8.3565	8.4116	8.3516	8.3854	**7.77**
**H4**	7.4581	7.6639	7.8093	7.7447	7.9311	7.7390	7.9740	7.7330	7.9597	7.7502	7.9947	7.4492	7.6452	7.7866	7.7203	7.9047	7.7148	7.9364	7.7089	7.9219	7.7256	7.9562	**7.71**
**H6**	8.0004	8.2271	8.3948	8.3308	8.5611	8.3239	8.6143	8.3166	8.6101	8.3374	8.6392	7.8271	8.0529	8.1960	8.1185	8.3804	8.1113	8.3619	8.1036	8.3608	8.1254	8.3921	**7.77**
**H8**	4.1763	4.2775	4.3389	4.3510	4.4417	4.3446	4.4925	4.3380	4.4918	4.3573	4.4967	4.4074	4.5573	4.6485	4.6471	4.7853	4.6404	4.7794	4.6334	4.7762	4.6535	4.7970	**5.21**
**H(9A-9C)**	1.3553	1.3677	1.3612	1.3661	1.3464	1.3655	1.3698	1.3645	1.3722	1.3666	1.3833	1.4090	1.4501	1.4248	1.4498	1.4343	1.4476	1.4389	1.4449	1.4425	1.4520	1.4478	**1.43**
**H(8`A-8`C)**	2.4514	2.4056	2.3644	2.3975	2.3613	2.3963	2.3388	2.3949	2.3302	2.3989	2.3264	2.7544	2.6581	2.5921	2.5605	2.5220	2.5613	2.4334	2.5618	2.4571	2.5598	2.3975	**2.58**

[a] methyl protons shifts are averaged
[b] according to [8]

Table S8. Cont. IV

Nuclei	R											S											**Exp.** [b]
	**mpW1LYP**																						
	Gas	$CHCl_3$ (PCM)	$CHCl_3$ (SMD)	DMSO (PCM)	DMSO (SMD)	CH_3OH (PCM)	CH_3OH (SMD)	C_2H_5OH (PCM)	C_2H_5OH (SMD)	H_2O (PCM)	H_2O (SMD)	Gas	$CHCl_3$ (PCM)	$CHCl_3$ (SMD)	DMSO (PCM)	DMSO (SMD)	CH_3OH (PCM)	CH_3OH (SMD)	C_2H_5OH (PCM)	C_2H_5OH (SMD)	H_2O (PCM)	H_2O (SMD)	
**C1**	146.6954	147.0297	148.2564	147.8907	148.6400	147.8778	147.9006	147.8678	148.4150	147.9029	148.0401	145.3975	145.9526	147.0982	147.1090	148.2406	147.1142	148.6029	147.1179	148.3530	147.0991	148.5512	**136.1**
**C2**	118.3114	118.4569	119.2628	118.5775	119.1262	118.5797	119.6482	118.5838	119.6100	118.5732	119.6569	118.0065	118.2052	118.9127	118.4124	119.0242	118.4141	119.6563	118.4164	119.5123	118.4082	119.6296	**112.1**
**C3**	173.8150	174.0060	174.5295	174.0927	174.7686	174.0856	174.6998	174.0799	174.7223	174.0975	174.7407	173.8076	174.0114	174.5579	174.1436	174.8245	174.1355	174.7995	174.1288	174.8010	174.1493	174.8317	**162.6**
**C4**	113.3275	114.8491	115.4058	115.4788	116.1242	115.4321	117.0762	115.3865	116.7496	115.5212	117.2251	113.6575	114.9373	115.4666	115.3459	115.9657	115.3033	116.5200	115.2623	116.4116	115.3849	116.7151	**110.1**
**C5**	173.0482	173.5647	174.1519	173.8579	174.6917	173.8369	174.6095	173.8172	174.5693	173.8756	174.6638	173.3048	173.7268	174.2432	173.9135	174.6800	173.8957	174.5185	173.8795	174.5322	173.9285	174.5686	**162.5**
**C6**	116.4785	118.2937	119.2255	118.9964	120.8217	118.9309	121.2423	118.8665	120.9070	119.0559	121.5448	116.7801	118.7954	119.9818	119.5693	121.3296	119.4964	121.3555	119.4261	121.3103	119.6378	121.7355	**112.1**
**C7**	202.6954	207.7338	209.6104	211.1097	211.3784	210.9360	216.3296	210.7657	215.6070	211.2754	217.1413	207.6460	211.6587	212.9987	213.8871	214.6863	213.7779	219.7701	213.6685	218.5665	213.9862	220.1650	**194.5**
**C8**	65.3085	66.3279	67.0698	66.9121	67.5805	66.8855	68.1375	66.8589	68.1550	66.9344	68.8103	59.9284	61.3760	62.2698	62.6392	63.3412	62.5920	64.1517	62.5455	63.8601	62.6809	64.3777	**58.4**
**C9**	22.7183	22.9747	22.9907	23.0217	23.4088	23.0033	23.4478	22.9868	23.3719	23.0377	23.5694	19.3696	19.5970	19.8456	19.8268	20.0576	19.8078	20.0460	19.7902	20.0217	19.8429	20.3845	**15**
**C8`**	36.2154	36.9814	37.3077	37.4364	37.7591	37.4100	37.8959	37.3843	37.8664	37.4585	38.2890	31.7290	32.1618	32.2327	32.1434	32.4620	32.1208	32.4852	32.1003	32.5146	32.1636	32.5516	**30.6**
**H2**	7.9970	7.9549	7.9939	7.8470	7.9734	7.8502	8.0094	7.8530	7.9702	7.8437	8.0023	8.0627	7.9784	8.0021	7.8446	7.9222	7.8453	7.8654	7.8459	7.8909	7.8442	7.8776	**7.77**
**H4**	7.0944	7.3016	7.4406	7.3797	7.5621	7.3755	7.6039	7.3709	7.5853	7.3836	7.6231	7.1094	7.3039	7.4400	7.3700	7.5518	7.3663	7.5797	7.3622	7.5681	7.3734	7.5992	**7.71**
**H6**	7.4794	7.7086	7.8257	7.7887	8.0197	7.7816	8.0548	7.7742	8.0150	7.7954	8.0834	7.3093	7.5248	7.6697	7.5860	7.8327	7.5796	7.8067	7.5729	7.8065	7.5921	7.8541	**7.77**
**H8**	4.2159	4.3197	4.3397	4.3826	4.4807	4.3770	4.5051	4.3714	4.4817	4.3881	4.5231	4.3616	4.5250	4.5988	4.6027	4.7354	4.5982	4.7549	4.5934	4.7415	4.6069	4.7822	**5.21**
**H(9A-9C)**	1.3129	1.3128	1.2823	1.2746	1.2695	1.2755	1.2944	1.2760	1.2797	1.2735	1.3073	1.3946	1.4360	1.4230	1.4411	1.4216	1.4385	1.4142	1.4357	1.4183	1.4436	1.4403	**1.43**
**H(8`A-8`C)**	2.5118	2.4603	2.4356	2.4540	2.4078	2.4553	2.3995	2.4562	2.4124	2.4528	2.3810	2.9355	2.8059	2.7355	2.7062	2.6583	2.7091	2.5732	2.7120	2.5952	2.7034	2.5396	**2.58**
Nuclei	**mpW1PW91**																						
**C1**	144.2225	144.6598	145.7040	145.3799	146.2379	145.3716	145.5329	145.3679	146.0569	145.3858	145.7331	143.1728	143.5731	144.7415	144.7586	145.7980	144.7551	146.4282	144.7571	146.1584	144.7610	146.3758	**136.1**
**C2**	117.2526	117.3898	118.1545	117.4212	118.0982	117.4293	118.5695	117.4425	118.5255	117.4126	118.5909	117.0273	117.1670	117.8923	117.3338	118.0065	117.3382	118.5880	117.3481	118.4544	117.3290	118.5954	**112.1**
**C3**	170.9185	171.1504	171.6651	171.2107	171.9534	171.2079	171.9032	171.2107	171.9086	171.2129	171.9840	170.9451	171.1682	171.7046	171.2683	172.0025	171.2643	172.0079	171.2663	171.9935	171.2712	172.0717	**162.6**
**C4**	112.5855	114.1255	114.7398	114.7594	115.5112	114.7164	116.4703	114.6788	116.1288	114.7997	116.6327	112.8243	114.2167	114.7815	114.6091	115.3972	114.5723	115.8720	114.5411	115.7564	114.6430	116.1076	**110.1**
**C5**	170.1326	170.6821	171.2539	170.9502	171.8287	170.9328	171.7689	170.9210	171.7130	170.9659	171.8579	170.3544	170.8292	171.3428	170.9936	171.8314	170.9794	171.6724	170.9707	171.6763	171.0067	171.7541	**162.5**
**C6**	115.0757	116.8642	117.8399	117.5632	119.4041	117.5045	119.7868	117.4528	119.4366	117.6183	120.1171	115.4038	117.4235	118.5471	118.1370	120.0137	118.0767	119.9540	118.0217	119.8987	118.1937	120.3774	**112.1**
**C7**	200.7895	205.6480	207.3324	208.7822	209.0294	208.6217	213.9502	208.4636	213.2810	208.9327	214.7277	204.8778	208.8681	210.2474	211.1288	211.8437	211.0165	217.0660	210.9092	215.8143	211.2338	217.4156	**194.5**
**C8**	63.1000	64.2403	64.8967	64.7653	65.5504	64.7429	66.0201	64.7247	66.0144	64.7852	66.7798	58.0020	59.2660	60.0876	60.4729	61.1841	60.4308	62.0933	60.3940	61.7725	60.5122	62.3622	**58.4**
**C9**	22.1261	22.4109	22.4089	22.4368	22.8830	22.4248	22.8945	22.4206	22.8007	22.4484	23.0762	18.8754	19.1563	19.3318	19.3779	19.6906	19.3670	19.6451	19.3613	19.6060	19.3874	20.0629	**15**
**C8`**	35.4186	36.2057	36.4938	36.6074	36.9572	36.5854	37.0943	36.5678	37.0740	36.6266	37.5278	30.8382	31.3589	31.4328	31.3034	31.6769	31.2857	31.6655	31.2743	31.6728	31.3199	31.7523	**30.6**
**H2**	7.9984	7.9462	7.9926	7.8437	7.9619	7.8472	7.9988	7.8508	7.9566	7.8408	7.9884	8.0381	7.9804	7.9995	7.8367	7.9277	7.8386	7.8462	7.8407	7.8716	7.8351	7.8636	**7.77**
**H4**	7.0832	7.2948	7.4334	7.3724	7.5572	7.3683	7.6011	7.3642	7.5818	7.3766	7.6225	7.0956	7.2971	7.4332	7.3637	7.5496	7.3601	7.5778	7.3566	7.5651	7.3673	7.6001	**7.71**
**H6**	7.5080	7.7233	7.8376	7.7993	8.0168	7.7927	8.0585	7.7861	8.0187	7.8060	8.0873	7.3277	7.5509	7.6920	7.6059	7.8635	7.5999	7.8225	7.5941	7.8231	7.6118	7.8746	**7.77**
**H8**	4.2244	4.3218	4.3363	4.3859	4.4639	4.3805	4.5066	4.3754	4.4841	4.3914	4.5242	4.3982	4.5566	4.6310	4.6308	4.7673	4.6266	4.7817	4.6225	4.7690	4.6350	4.8120	**5.21**
**H(9A-9C)**	1.2876	1.2869	1.2584	1.2481	1.2462	1.2494	1.2633	1.2508	1.2451	1.2472	1.2817	1.3692	1.4158	1.3930	1.4134	1.3981	1.4117	1.3832	1.4100	1.3862	1.4152	1.4170	**1.43**
**H(8`A-8`C)**	2.4511	2.4020	2.3692	2.3869	2.3468	2.3885	2.3327	2.3902	2.3449	2.3856	2.3205	2.8305	2.7152	2.6404	2.6069	2.5659	2.6103	2.4699	2.6139	2.4942	2.6039	2.4399	**2.58**
Nuclei	**O3LYP**																						
**C1**	141.3626	142.5917	143.4253	143.2785	144.7702	143.2292	143.9688	143.1840	144.3758	143.3425	144.1238	139.5491	141.4273	142.7236	142.9561	144.1120	142.9115	144.0561	142.8662	144.3164	142.9969	144.2411	**136.1**
**C2**	113.3703	113.4602	114.1215	113.4378	114.1196	113.4506	114.4476	113.4585	114.4048	113.4207	114.4618	112.9638	113.3134	114.0646	113.4415	114.1732	113.4479	114.5354	113.4494	114.4548	113.4293	114.5486	**112.1**
**C3**	167.2430	167.5085	168.1326	167.6741	168.4865	167.6758	168.4599	167.6720	168.4406	167.6643	168.5310	167.3751	167.6171	168.2128	167.7928	168.5569	167.7911	168.5084	167.7847	168.4988	167.7878	168.5684	**162.6**
**C4**	108.3682	109.6094	110.1761	110.2097	110.7348	110.1846	111.5917	110.1542	111.3103	110.2257	111.7229	108.7484	109.6303	110.1384	109.8983	110.5667	109.8821	111.0426	109.8595	110.7912	109.9051	111.1742	**110.1**
**C5**	166.5608	166.8955	167.5845	167.1445	168.0148	167.1419	167.9649	167.1328	167.9086	167.1370	168.0393	166.9785	167.3075	167.9004	167.4880	168.3477	167.4816	168.1602	167.4698	168.1189	167.4871	168.2288	**162.5**
**C6**	110.7147	112.1256	113.2291	113.0516	114.4820	113.0033	114.8817	112.9494	114.6110	113.0893	115.1454	111.3304	112.8001	113.7613	113.3356	114.9980	113.3060	115.1716	113.2692	114.9381	113.3550	115.4111	**112.1**
**C7**	197.1096	202.9227	204.0759	205.9307	206.6632	205.7257	211.1972	205.5335	210.6445	206.1620	211.8380	201.1308	205.4540	207.2431	208.2396	209.0233	208.0862	213.3073	207.9318	212.4937	208.3824	213.8264	**194.5**
**C8**	63.4071	64.6633	65.4427	65.3447	66.1262	65.3194	66.6835	65.2877	66.6497	65.3608	67.3719	58.3781	60.5921	61.4222	61.9639	62.6179	61.9184	63.5423	61.8694	63.4816	62.0026	63.8364	**58.4**
**C9**	21.6163	21.8223	22.0722	22.1483	22.4499	22.1292	22.5737	22.1078	22.4968	22.1649	22.6690	18.2684	18.4982	18.6825	18.6658	18.9344	18.6724	19.0724	18.6734	19.0207	18.6526	19.3603	**15**
**C8`**	35.8449	36.6061	36.8159	37.0179	37.3811	37.0044	37.4439	36.9872	37.4422	37.0283	37.8042	31.3601	31.6168	31.8255	31.7541	32.1167	31.7420	32.0608	31.7245	32.0052	31.7589	32.0695	**30.6**
**H2**	7.7654	7.6576	7.7241	7.5827	7.6548	7.5893	7.7013	7.5949	7.6739	7.5741	7.6983	7.8819	7.6803	7.7054	7.5307	7.6316	7.5358	7.6236	7.5402	7.6028	7.5253	7.6210	**7.77**
**H4**	6.9999	7.2006	7.3323	7.2754	7.4478	7.2716	7.4875	7.2675	7.4708	7.2788	7.5074	7.0293	7.2055	7.3385	7.2666	7.4450	7.2634	7.4681	7.2597	7.4517	7.2695	7.4881	**7.71**
**H6**	7.3873	7.5699	7.6916	7.6502	7.8505	7.6454	7.8778	7.6403	7.8485	7.6547	7.9048	7.2170	7.3700	7.4951	7.4119	7.6454	7.4082	7.6324	7.4040	7.6060	7.4150	7.6663	**7.77**
**H8**	4.3029	4.3767	4.3942	4.4296	4.5168	4.4250	4.5324	4.4205	4.5098	4.4343	4.5527	4.4484	4.5879	4.6569	4.6523	4.7905	4.6483	4.8028	4.6440	4.7818	4.6558	4.8290	**5.21**
**H(9A-9C)**	1.2073	1.1916	1.1914	1.1840	1.1583	1.1856	1.1894	1.1866	1.1765	1.1814	1.2046	1.3141	1.3340	1.3130	1.3401	1.3206	1.3398	1.3446	1.3391	1.3349	1.3401	1.3621	**1.43**
**H(8`A-8`C)**	2.4839	2.4429	2.4280	2.4396	2.4097	2.4402	2.4035	2.4405	2.4078	2.4390	2.3838	2.8879	2.7280	2.6798	2.6412	2.6063	2.6447	2.5273	2.6477	2.5313	2.6379	2.4875	**2.58**
Nuclei	**OLYP**																						
**C1**	139.9317	141.4893	141.9757	142.3264	144.1779	141.9699	143.5152	141.8960	143.4707	142.3356	143.5090	137.8422	139.8035	140.8771	141.3357	142.4088	141.2953	142.5404	141.2530	142.6580	141.3692	142.7229	**136.1**
**C2**	111.2607	111.3302	111.9291	111.3563	112.0438	111.3476	112.3810	111.3472	112.3202	111.3404	112.3789	110.8998	111.2102	111.9201	111.3416	112.0082	111.3460	112.3951	111.3452	112.3215	111.3287	112.4020	**112.1**
**C3**	166.1497	166.4561	167.1627	166.6043	167.4439	166.6241	167.4295	166.6204	167.4082	166.5963	167.5092	166.3507	166.6408	167.2835	166.8169	167.6269	166.8143	167.5598	166.8059	167.5509	166.8102	167.6178	**162.6**
**C4**	106.4331	107.5541	108.2744	108.1019	108.4532	108.1704	109.1728	108.1487	109.0950	108.1216	109.3670	106.9212	107.6884	108.1950	107.8978	108.5118	107.8833	108.9122	107.8620	108.7216	107.9008	109.0409	**110.1**
**C5**	165.4456	165.7469	166.5158	166.0783	166.9903	166.0754	166.9235	166.0589	166.9417	166.0844	166.9844	166.0689	166.3827	167.0307	166.5237	167.4338	166.5176	167.2233	166.5056	167.1985	166.5198	167.2896	**162.5**
**C6**	108.5633	109.9551	111.0626	110.9096	112.1659	110.9017	112.4208	110.8482	112.3470	110.9540	112.7072	109.3378	110.6674	111.7118	111.1800	112.8401	111.1455	112.9211	111.1038	112.7493	111.2019	113.1364	**112.1**
**C7**	196.1447	202.1235	203.2664	205.4845	206.6248	204.8831	210.6583	204.6537	209.9919	205.6478	211.1253	200.0067	204.2912	205.6616	206.9151	207.4985	206.7907	211.6259	206.6619	210.8097	207.0263	212.1594	**194.5**
**C8**	64.6454	66.0080	66.4984	66.7441	67.6551	66.5864	68.4561	66.5415	68.1288	66.7621	69.0325	59.2123	61.7856	62.8049	63.3246	64.0800	63.2618	65.0788	63.1959	64.9446	63.3777	65.3227	**58.4**
**C9**	21.7818	22.1684	22.4047	22.4255	22.6858	22.4461	22.8620	22.4340	22.8390	22.4269	23.0031	18.2976	18.6452	18.9362	18.8551	19.1674	18.8500	19.3449	18.8395	19.2711	18.8509	19.6177	**15**
**C8`**	36.4717	37.2166	37.5427	37.7192	38.0812	37.6914	38.0701	37.6695	38.1060	37.7376	38.4066	32.0326	32.2148	32.4179	32.3497	32.6591	32.3414	32.5761	32.3270	32.5628	32.3478	32.5677	**30.6**
**H2**	7.5999	7.4786	7.5958	7.3931	7.4452	7.4296	7.4930	7.4377	7.4967	7.3894	7.5064	7.7420	7.5478	7.6011	7.4092	7.5167	7.4133	7.5020	7.4171	7.4929	7.4045	7.4989	**7.77**
**H4**	6.9196	7.1122	7.2510	7.1848	7.3514	7.1837	7.3835	7.1800	7.3757	7.1881	7.4054	6.9544	7.1227	7.2540	7.1796	7.3553	7.1765	7.3758	7.1732	7.3616	7.1820	7.3947	**7.71**
**H6**	7.3116	7.4687	7.6349	7.5494	7.7186	7.5623	7.7153	7.5591	7.7268	7.5546	7.7549	7.1445	7.2752	7.4043	7.3090	7.5407	7.3055	7.5166	7.3016	7.4983	7.3117	7.5496	**7.77**
**H8**	4.4155	4.4608	4.4984	4.5091	4.5700	4.5087	4.5678	4.5049	4.5728	4.5124	4.5944	4.5330	4.6738	4.7440	4.7345	4.8709	4.7309	4.8761	4.7270	4.8588	4.7374	4.9028	**5.21**
**H(9A-9C)**	1.2034	1.1869	1.1887	1.1784	1.1488	1.1851	1.1898	1.1861	1.1785	1.1773	1.2046	1.3048	1.3421	1.3385	1.3528	1.3432	1.3515	1.3635	1.3501	1.3565	1.3535	1.3790	**1.43**
**H(8`A-8`C)**	2.5203	2.4877	2.4625	2.4859	2.4600	2.4826	2.4515	2.4824	2.4579	2.4854	2.4231	2.9420	2.7818	2.7233	2.6889	2.6446	2.6935	2.5668	2.6978	2.5744	2.6840	2.5264	**2.58**

[a] methyl protons shifts are averaged

[b] according to [8]

Table S8. Cont. V

Nuclei	R											S											Exp. [b]
	OPBE																						
	Gas	$CHCl_3$ (PCM)	$CHCl_3$ (SMD)	DMSO (PCM)	DMSO (SMD)	CH_3OH (PCM)	CH_3OH (SMD)	C_2H_5OH (PCM)	C_2H_5OH (SMD)	H_2O (PCM)	H_2O (SMD)	Gas	$CHCl_3$ (PCM)	$CHCl_3$ (SMD)	DMSO (PCM)	DMSO (SMD)	CH_3OH (PCM)	CH_3OH (SMD)	C_2H_5OH (PCM)	C_2H_5OH (SMD)	H_2O (PCM)	H_2O (SMD)	
**C1**	137.1812	138.8723	139.1163	139.4007	141.3382	139.3392	140.7373	139.2702	140.7294	139.4461	140.7684	135.6936	137.9869	138.2517	138.8857	140.7933	138.7908	140.2411	138.6942	140.0456	138.9967	140.3037	**136.1**
**C2**	109.8919	110.0173	110.5680	109.9350	110.6768	109.9539	110.9628	109.9563	110.9088	109.9135	110.9840	109.7048	110.0227	110.6205	109.9990	110.7310	110.0165	111.0026	110.0158	110.9537	109.9766	111.0267	**112.1**
**C3**	162.7071	163.0900	163.7248	163.1824	163.9798	163.1966	163.9918	163.1921	163.9661	163.1640	164.1116	162.8916	163.2572	163.8507	163.3748	164.1396	163.3860	164.1179	163.3786	164.1185	163.3582	164.2212	**162.6**
**C4**	105.4060	106.6202	107.3523	107.1368	107.5273	107.1357	108.2322	107.1143	108.1145	107.1363	108.4534	105.7018	106.5047	107.2449	106.8213	107.1853	106.8426	107.8126	106.8384	107.7685	106.7844	108.0344	**110.1**
**C5**	161.9164	162.3320	163.0243	162.5860	163.4548	162.5877	163.4218	162.5718	163.4143	162.5808	163.5195	162.4480	162.8275	163.5172	162.9936	163.7483	163.0087	163.6737	163.0030	163.6944	162.9696	163.7994	**162.5**
**C6**	106.9347	108.4280	109.4508	109.2584	110.4890	109.2292	110.7407	109.1815	110.6431	109.2853	111.0318	107.5274	108.8555	110.0114	109.3994	110.7165	109.4062	111.0252	109.3887	110.9879	109.3779	111.3368	**112.1**
**C7**	193.8597	199.7724	200.5894	202.4636	203.6628	202.2557	207.7374	202.0459	206.9446	202.6493	208.1667	197.0287	201.4856	202.6204	203.8990	204.9440	203.7358	208.7355	203.5619	207.7152	204.0614	209.1422	**194.5**
**C8**	62.0499	63.5352	63.8315	64.1699	65.0526	64.1327	65.8606	64.0792	65.5834	64.1917	66.5203	57.5673	59.9740	60.1727	60.9227	61.9934	60.8433	62.6928	60.7594	62.3345	61.0141	62.9129	**58.4**
**C9**	21.0213	21.4673	21.6288	21.6535	21.9018	21.6620	22.0532	21.6529	22.0202	21.6449	22.2126	17.7135	18.1133	18.1861	18.1690	18.4254	18.1815	18.6235	18.1769	18.5344	18.1540	18.9474	**15**
**C8`**	35.4110	36.2146	36.4350	36.5937	36.9190	36.5825	36.8950	36.5544	36.9150	36.5974	37.2735	30.7794	30.9977	31.3304	31.1931	31.3099	31.2089	31.3431	31.2022	31.4174	31.1660	31.3730	**30.6**
**H2**	7.5766	7.4529	7.5784	7.3907	7.4291	7.3995	7.4747	7.4067	7.4740	7.3824	7.4893	7.6608	7.4523	7.5762	7.3715	7.4058	7.3820	7.4462	7.3903	7.4647	7.3585	7.4563	**7.77**
**H4**	6.9048	7.1049	7.2391	7.1769	7.3396	7.1740	7.3728	7.1703	7.3619	7.1790	7.3974	6.9316	7.1080	7.2431	7.1705	7.3316	7.1686	7.3613	7.1657	7.3523	7.1710	7.3852	**7.71**
**H6**	7.3078	7.4687	7.6341	7.5464	7.7024	7.5450	7.6982	7.5424	7.7009	7.5477	7.7400	7.1032	7.2382	7.4012	7.2978	7.4777	7.2985	7.4906	7.2974	7.4933	7.2947	7.5355	**7.77**
**H8**	4.4207	4.4774	4.5101	4.5129	4.5671	4.5118	4.5675	4.5102	4.5668	4.5146	4.5972	4.5673	4.6985	4.7702	4.7580	4.8639	4.7571	4.8853	4.7547	4.8808	4.7571	4.9206	**5.21**
**H(9A-9C)**	1.1579	1.1423	1.1394	1.1402	1.1007	1.1419	1.1426	1.1425	1.1316	1.1379	1.1616	1.2676	1.2969	1.2853	1.3068	1.2710	1.3074	1.3042	1.3072	1.3029	1.3053	1.3266	**1.43**
**H(8`A-8`C)**	2.4393	2.4071	2.3704	2.3982	2.3658	2.3985	2.3593	2.3985	2.3633	2.3972	2.3334	2.7958	2.6308	2.6094	2.5655	2.4923	2.5721	2.4383	2.5771	2.4578	2.5571	2.4038	**2.58**
Nuclei	OPW91																						
**C1**	137.4649	139.1592	139.4169	139.6610	141.6192	139.5905	141.0231	139.5198	141.0076	139.7106	141.0485	135.9557	138.2763	138.5406	139.1355	141.0648	139.0425	140.4970	138.9459	140.3175	139.2166	140.5653	**136.1**
**C2**	110.1375	110.2661	110.8141	110.1874	110.9185	110.1968	111.2077	110.1903	111.1546	110.1619	111.2312	109.9425	110.2676	110.8643	110.2492	110.9718	110.2566	111.2474	110.2466	111.1970	110.2234	111.2745	**112.1**
**C3**	163.0964	163.4809	164.1118	163.5792	164.3651	163.5832	164.3755	163.5690	164.3505	163.5567	164.4971	163.2811	163.6458	164.2359	163.7698	164.5237	163.7705	164.5015	163.7535	164.5018	163.7505	164.6057	**162.6**
**C4**	105.6185	106.8346	107.5563	107.3683	107.7346	107.3572	108.4397	107.3242	108.3272	107.3613	108.6653	105.9244	106.7214	107.4557	107.0607	107.3992	107.0668	108.0369	107.0497	107.9846	107.0309	108.2592	**110.1**
**C5**	162.3102	162.7241	163.4119	162.9843	163.8421	162.9762	163.8068	162.9507	163.8018	162.9744	163.9064	162.8434	163.2203	163.9061	163.3985	164.1385	163.4014	164.0653	163.3852	164.0839	163.3751	164.1918	**162.5**
**C6**	107.1867	108.6822	109.7033	109.5296	110.7452	109.4903	110.9969	109.4319	110.9022	109.5506	111.2930	107.7879	109.1154	110.2699	109.6850	110.9795	109.6769	111.2976	109.6464	111.2534	109.6699	111.6092	**112.1**
**C7**	194.1982	200.1322	200.9603	202.8148	204.0355	202.5992	208.1332	202.3891	207.3379	203.0053	208.5588	197.4052	201.8757	203.0092	204.2746	205.3210	204.1075	209.1191	203.9293	208.1088	204.4200	209.5337	**194.5**
**C8**	62.2341	63.7170	64.0244	64.3411	65.2301	64.2932	66.0436	64.2338	65.7594	64.3640	66.7003	57.7227	60.1597	60.3639	61.0879	62.1725	61.0070	62.8615	60.9199	62.5151	61.1566	63.0850	**58.4**
**C9**	21.0828	21.5343	21.6908	21.7299	21.9658	21.7286	22.1168	21.7091	22.0865	21.7154	22.2778	17.7891	18.1949	18.2691	18.2576	18.5064	18.2600	18.7061	18.2461	18.6163	18.2380	19.0298	**15**
**C8`**	35.4773	36.2829	36.5031	36.6722	36.9885	36.6515	36.9687	36.6153	36.9900	36.6728	37.3482	30.8712	31.0887	31.4196	31.3002	31.4020	31.3026	31.4406	31.2844	31.5120	31.2757	31.4754	**30.6**
**H2**	7.5829	7.4592	7.5830	7.4002	7.4347	7.4084	7.4796	7.4147	7.4797	7.3908	7.4947	7.6697	7.4578	7.5813	7.3815	7.4120	7.3902	7.4536	7.3975	7.4706	7.3705	7.4633	**7.77**
**H4**	6.9089	7.1088	7.2427	7.1818	7.3430	7.1783	7.3763	7.1742	7.3656	7.1835	7.4008	6.9362	7.1118	7.2468	7.1755	7.3350	7.1728	7.3654	7.1695	7.3559	7.1762	7.3889	**7.71**
**H6**	7.3110	7.4719	7.6361	7.5537	7.7066	7.5516	7.7021	7.5483	7.7059	7.5541	7.7441	7.1085	7.2422	7.4048	7.3053	7.4824	7.3046	7.4965	7.3027	7.4979	7.3035	7.5410	**7.77**
**H8**	4.4204	4.4775	4.5098	4.5151	4.5678	4.5134	4.5680	4.5114	4.5679	4.5160	4.5975	4.5628	4.6945	4.7660	4.7566	4.8607	4.7544	4.8833	4.7514	4.8776	4.7562	4.9181	**5.21**
**H(9A-9C)**	1.1599	1.1444	1.1417	1.1432	1.1027	1.1441	1.1446	1.1444	1.1334	1.1406	1.1635	1.2686	1.2979	1.2867	1.3091	1.2728	1.3090	1.3062	1.3084	1.3045	1.3076	1.3284	**1.43**
**H(8`A-8`C)**	2.4408	2.4087	2.3725	2.4002	2.3676	2.3999	2.3614	2.3997	2.3653	2.3990	2.3356	2.8007	2.6350	2.6134	2.5722	2.4972	2.5776	2.4439	2.5820	2.4625	2.5648	2.4093	**2.58**
Nuclei	PBE																						
**C1**	142.3264	142.9692	144.1140	143.6534	144.6389	143.5845	144.3049	143.5172	144.6953	143.6967	144.4630	140.9980	141.6745	142.9296	142.8397	144.0948	142.8359	144.5159	142.8421	144.1071	142.8602	144.4215	**136.1**
**C2**	114.6465	114.8007	115.5427	114.7739	115.4551	114.7664	115.9778	114.7677	115.9491	114.7876	116.0037	114.3100	114.4990	115.1982	114.6369	115.3349	114.6447	115.9596	114.6625	115.7841	114.6471	115.9521	**112.1**
**C3**	171.7230	172.1720	172.7788	172.2080	173.1901	172.2058	173.1540	172.2141	173.1313	172.2296	173.2513	171.7500	172.1886	172.8133	172.2495	173.2218	172.2434	173.2035	172.2478	173.1868	172.2732	173.2844	**162.6**
**C4**	110.6135	112.2095	112.7580	112.7919	113.5797	112.7526	114.4831	112.7264	114.1839	112.8507	114.6795	110.9480	112.3532	112.8695	112.6836	113.4832	112.6466	114.0561	112.6206	113.9495	112.7363	114.2975	**110.1**
**C5**	170.9566	171.6896	172.3417	171.9184	173.0405	171.9015	172.9803	171.8955	172.9167	171.9540	173.0912	171.2912	171.9293	172.5189	172.0257	173.0859	172.0095	172.9227	172.0045	172.9243	172.0590	173.0127	**162.5**
**C6**	112.9770	114.7134	115.6669	115.3335	117.2369	115.3017	117.5110	115.2840	117.2177	115.3969	117.8935	113.2854	115.2657	116.3852	115.9336	117.8610	115.8750	117.9666	115.8270	117.9573	116.0070	118.4525	**112.1**
**C7**	200.8509	205.4530	207.1570	208.2153	208.8747	207.9959	213.2527	207.7855	212.6962	208.3981	213.9443	205.5002	209.2264	210.6267	211.1437	212.1756	211.0402	216.4495	210.9467	215.2852	211.2597	216.6164	**194.5**
**C8**	67.1268	68.3681	69.1368	68.8469	69.7914	68.8299	70.3156	68.8198	70.2917	68.8812	71.1404	61.6919	63.1272	64.0110	64.2948	65.2230	64.2534	65.9732	64.2220	65.7025	64.3512	66.3289	**58.4**
**C9**	23.1425	23.6066	23.6477	23.6635	24.1923	23.6677	24.2576	23.6820	24.1399	23.6884	24.5020	19.8413	20.3637	20.5834	20.6126	21.0496	20.5977	21.1236	20.5930	21.1134	20.6442	21.7535	**15**
**C8`**	36.9383	37.7451	37.9826	38.0604	38.4970	38.0341	38.6438	38.0196	38.6344	38.1013	39.1128	31.9936	32.5377	32.5508	32.3542	32.7959	32.3362	32.8235	32.3296	32.8398	32.3895	32.8605	**30.6**
**H2**	7.7868	7.7481	7.7927	7.6571	7.7742	7.6648	7.7943	7.6738	7.7638	7.6537	7.7916	7.8541	7.7964	7.8112	7.6571	7.7401	7.6587	7.6940	7.6609	7.7282	7.6562	7.7124	**7.77**
**H4**	6.8986	7.1083	7.2450	7.1820	7.3688	7.1778	7.4079	7.1742	7.3902	7.1866	7.4289	6.9185	7.1160	7.2504	7.1780	7.3648	7.1744	7.3924	7.1712	7.3796	7.1820	7.4126	**7.71**
**H6**	7.4038	7.6094	7.7089	7.6683	7.8894	7.6634	7.9151	7.6602	7.8817	7.6749	7.9457	7.1780	7.3881	7.5272	7.4387	7.6931	7.4327	7.6723	7.4272	7.6774	7.4451	7.7276	**7.77**
**H8**	4.4981	4.5739	4.5775	4.6157	4.6915	4.6104	4.7198	4.6062	4.7001	4.6208	4.7375	4.6331	4.7814	4.8582	4.8598	4.9935	4.8557	5.0146	4.8522	4.9971	4.8642	5.0431	**5.21**
**H(9A-9C)**	1.3572	1.3602	1.3373	1.3264	1.3268	1.3308	1.3382	1.3360	1.3247	1.3249	1.3608	1.4537	1.5017	1.4843	1.5026	1.4938	1.5009	1.4929	1.4996	1.5029	1.5049	1.5373	**1.43**
**H(8`A-8`C)**	2.5581	2.4988	2.4735	2.4798	2.4405	2.4818	2.4244	2.4838	2.4349	2.4785	2.4056	2.9585	2.8351	2.7604	2.7228	2.6787	2.7261	2.5878	2.7299	2.6101	2.7205	2.5499	**2.58**
Nuclei	PBE0																						
**C1**	143.7461	144.0537	144.9521	144.4974	145.5986	144.4554	145.1130	144.4229	145.5819	144.5521	145.1926	142.7533	143.1733	144.3512	144.3031	145.3449	144.2894	145.8180	144.2776	145.4321	144.3186	145.7442	**136.1**
**C2**	116.8542	116.9461	117.6760	116.9089	117.6070	116.9051	118.1795	116.9043	118.0904	116.9176	118.1421	116.6200	116.7717	117.4939	116.9207	117.5687	116.9203	118.1939	116.9217	117.9969	116.9242	118.1537	**112.1**
**C3**	170.5711	170.8237	171.3384	170.8919	171.6113	170.8833	171.6526	170.8766	171.5934	170.9029	171.6736	170.5958	170.8311	171.3650	170.9264	171.6399	170.9171	171.7266	170.9096	171.6474	170.9382	171.7307	**162.6**
**C4**	112.2591	113.8270	114.4269	114.4955	115.1959	114.4510	116.2103	114.4078	115.8161	114.5397	116.3379	112.4907	113.8980	114.4624	114.3076	115.0705	114.2658	115.6922	114.2254	115.5153	114.3503	115.8544	**110.1**
**C5**	169.7460	170.3268	170.8778	170.6171	171.4603	170.5937	171.4896	170.5723	171.3802	170.6425	171.5286	169.9477	170.4466	170.9573	170.6144	171.4293	170.5933	171.3601	170.5739	171.2951	170.6379	171.3812	**162.5**
**C6**	114.7836	116.6396	117.6901	117.4177	119.1506	117.3682	119.5336	117.3176	119.1379	117.4626	119.8318	115.1057	117.1331	118.2471	117.8622	119.7407	117.8009	119.8831	117.7415	119.8177	117.9233	120.2527	**112.1**
**C7**	200.4246	205.1043	206.5017	207.8220	208.4140	207.6373	213.4387	207.4637	212.7269	208.0149	214.1148	204.5369	208.4835	209.8905	210.7092	211.4193	210.5820	216.4328	210.4558	215.0489	210.8322	216.7267	**194.5**
**C8**	63.0036	64.1501	64.8786	64.6307	65.4465	64.6012	65.9772	64.5720	65.9054	64.6600	66.6666	57.7852	59.0529	59.8501	60.1927	60.9323	60.1504	61.8163	60.1099	61.4928	60.2359	62.0696	**58.4**
**C9**	22.1140	22.4409	22.6095	22.5719	22.9486	22.5614	22.9482	22.5508	22.7920	22.5812	23.0886	18.8504	19.1591	19.3141	19.3668	19.6962	19.3562	19.7491	19.3475	19.7050	19.3798	20.1570	**15**
**C8`**	35.3215	36.1066	36.3265	36.4449	36.7998	36.4180	37.0460	36.3936	36.9698	36.4744	37.4328	30.5943	31.1062	31.1761	31.0537	31.3870	31.0284	31.4879	31.0054	31.4180	31.0811	31.4971	**30.6**
**H2**	8.0001	7.9597	8.0168	7.8853	7.9746	7.8909	8.0071	7.8959	7.9631	7.8786	8.0008	8.0375	7.9791	7.9965	7.8393	7.9246	7.8413	7.8671	7.8434	7.8949	7.8370	7.8783	**7.77**
**H4**	7.0682	7.2802	7.4180	7.3579	7.5417	7.3536	7.5886	7.3492	7.5665	7.3616	7.6076	7.0802	7.2817	7.4180	7.3486	7.5328	7.3446	7.5662	7.3406	7.5500	7.3520	7.5849	**7.71**
**H6**	7.5106	7.7314	7.8402	7.8086	8.0186	7.8040	8.0571	7.7992	8.0142	7.8124	8.0884	7.3219	7.5445	7.6856	7.6025	7.8594	7.5963	7.8387	7.5900	7.8383	7.6082	7.8863	**7.77**
**H8**	4.2176	4.3106	4.3166	4.3741	4.4479	4.3693	4.4955	4.3648	4.4723	4.3787	4.5115	4.4008	4.5577	4.6351	4.6346	4.7687	4.6299	4.7928	4.6251	4.7730	4.6388	4.8164	**5.21**
**H(9A-9C)**	1.2972	1.3059	1.2906	1.2811	1.2640	1.2834	1.2767	1.2852	1.2565	1.2777	1.2948	1.3816	1.4270	1.4015	1.4245	1.4088	1.4232	1.4084	1.4219	1.4146	1.4256	1.4404	**1.43**
**H(8`A-8`C)**	2.4583	2.4055	2.3811	2.3913	2.3498	2.3921	2.3382	2.3930	2.3480	2.3902	2.3206	2.8339	2.7160	2.6412	2.6083	2.5641	2.6110	2.4752	2.6138	2.4949	2.6055	2.4399	**2.58**

[a] methyl protons shifts are averaged
[b] according to [8]

Table S8. Cont. VI

Nuclei	R											S											Exp. [b]
	TPSS																						
	Gas	$CHCl_3$ (PCM)	$CHCl_3$ (SMD)	DMSO (PCM)	DMSO (SMD)	CH_3OH (PCM)	CH_3OH (SMD)	C_2H_5OH (PCM)	C_2H_5OH (SMD)	H_2O (PCM)	H_2O (SMD)	Gas	$CHCl_3$ (PCM)	$CHCl_3$ (SMD)	DMSO (PCM)	DMSO (SMD)	CH_3OH (PCM)	CH_3OH (SMD)	C_2H_5OH (PCM)	C_2H_5OH (SMD)	H_2O (PCM)	H_2O (SMD)	
**C1**	139.0320	139.1927	139.8805	139.6963	140.8651	139.6715	140.4903	139.6590	140.1897	139.7346	140.5585	137.8969	138.3691	139.6062	139.3674	140.5425	139.3728	140.3747	139.3877	140.4527	139.3712	140.4449	**136.1**
**C2**	112.6267	112.6374	113.3677	112.6440	113.3116	112.6494	113.8609	112.6669	113.0897	112.6505	113.8475	112.3073	112.4047	113.1583	112.5649	113.1915	112.5767	113.7134	112.5991	113.8650	112.5637	113.7020	**112.1**
**C3**	168.1648	168.4103	169.0601	168.5412	169.3567	168.5411	169.3212	168.5523	169.1986	168.5515	169.3772	168.2185	168.4575	169.1344	168.5952	169.4188	168.5935	169.3950	168.6030	169.4489	168.6074	169.4494	**162.6**
**C4**	108.6424	110.0741	110.8358	110.7551	111.4035	110.7224	112.3338	110.7010	111.1159	110.7968	112.5058	108.9467	110.1588	110.7639	110.5940	111.2688	110.5601	111.9747	110.5379	111.8193	110.6371	112.1574	**110.1**
**C5**	167.4574	167.9837	168.7324	168.2968	169.2479	168.2844	169.1829	168.2835	168.9287	168.3189	169.2587	167.7620	168.2007	168.8555	168.3932	169.3017	168.3806	169.1518	168.3796	169.2272	168.4164	169.2226	**162.5**
**C6**	110.8894	112.5810	113.7273	113.3672	114.9798	113.3251	115.2832	113.2936	113.7189	113.4157	115.6298	111.0950	112.9078	114.0786	113.7000	115.4240	113.6475	115.8192	113.6066	114.8650	113.7609	116.1284	**112.1**
**C7**	196.8898	201.0326	202.3886	203.5739	204.3978	203.4243	208.8853	203.2879	204.0732	203.7317	209.5248	201.1453	204.6201	206.0260	206.5468	207.4085	206.4406	211.3099	206.3453	206.9081	206.6577	211.7826	**194.5**
**C8**	65.6505	66.7365	67.3988	67.2040	68.0191	67.1783	68.5460	67.1629	67.4037	67.2378	69.2740	60.9437	62.2356	63.1513	63.3814	64.1964	63.3533	64.9001	63.3354	64.9708	63.4172	65.1130	**58.4**
**C9**	23.7373	24.0413	24.3455	24.2739	24.6158	24.2687	24.6603	24.2744	24.3436	24.2875	24.8535	20.5507	20.9426	21.2845	21.2925	21.6427	21.2874	21.9056	21.2934	23.2441	21.3074	22.3209	**15**
**C8`**	37.3639	38.0816	38.4074	38.4438	38.8144	38.4249	39.0149	38.4179	38.5437	38.4734	39.4169	33.0644	33.3942	33.5030	33.3087	33.6361	33.2916	33.6935	33.2868	35.1476	33.3365	33.7248	**30.6**
**H2**	7.8252	7.7977	7.8940	7.7309	7.8235	7.7355	7.8412	7.7406	7.5780	7.7267	7.8368	7.8721	7.8044	7.8260	7.6834	7.7618	7.6844	7.7525	7.6862	7.8018	7.6831	7.7531	**7.77**
**H4**	6.9040	7.1119	7.2605	7.1929	7.3821	7.1886	7.4232	7.1849	7.0200	7.1973	7.4416	6.9252	7.1216	7.2632	7.1918	7.3797	7.1876	7.4108	7.1841	7.3034	7.1961	7.4288	**7.71**
**H6**	7.3174	7.5411	7.6864	7.6209	7.8341	7.6166	7.8578	7.6129	7.4497	7.6254	7.8906	7.1229	7.3367	7.4845	7.4089	7.6600	7.4021	7.6705	7.3959	7.5136	7.4159	7.7128	**7.77**
**H8**	4.3763	4.4463	4.4832	4.5118	4.5949	4.5086	4.6190	4.5061	4.3305	4.5155	4.6337	4.4877	4.6419	4.7253	4.7309	4.8643	4.7260	4.8878	4.7218	4.8596	4.7359	4.9156	**5.21**
**H(9A-9C)**	1.3498	1.3703	1.3660	1.3492	1.3314	1.3504	1.3433	1.3521	1.1638	1.3480	1.3628	1.4356	1.4849	1.4734	1.4977	1.4863	1.4959	1.5128	1.4947	1.6566	1.4998	1.5367	**1.43**
**H(8`A-8`C)**	2.5139	2.4524	2.4122	2.4440	2.4057	2.4445	2.3918	2.4455	2.2616	2.4439	2.3718	2.9239	2.7964	2.7296	2.6961	2.6526	2.6981	2.5640	2.7009	2.8538	2.6948	2.5317	**2.58**
Nuclei	VSXC																						
**C1**	136.4899	136.5707	137.2694	136.5436	137.6629	136.5500	137.1133	136.5540	137.1949	136.5390	137.2307	136.4584	136.7193	137.6773	137.6452	139.2991	137.6318	138.6256	137.6170	138.6294	137.6600	138.7786	**136.1**
**C2**	110.7860	110.9165	111.4359	110.8176	111.3120	110.8320	111.8107	110.8430	111.7327	110.8046	111.8623	110.2901	110.5045	111.0804	110.3228	110.8104	110.3311	111.2166	110.3371	111.1379	110.3161	111.2502	**112.1**
**C3**	165.9127	166.2688	166.8285	166.4713	167.2394	166.4599	167.1798	166.4472	167.1710	166.4841	167.2522	166.6137	166.9403	167.5028	167.1734	167.9889	167.1650	167.9516	167.1544	167.9288	167.1828	168.0245	**162.6**
**C4**	106.3237	107.9853	108.6190	108.8608	109.5102	108.8097	110.4017	108.7567	110.1607	108.9109	110.6389	106.3246	107.8270	108.4136	108.4550	108.9912	108.4154	109.8011	108.3733	109.5933	108.4938	110.0055	**110.1**
**C5**	165.2128	165.8995	166.5559	166.3090	167.2505	166.2853	167.1638	166.2598	167.1371	166.3333	167.2734	165.8733	166.4278	167.0232	166.8227	167.6567	166.8064	167.5709	166.7880	167.5430	166.8397	167.6586	**162.5**
**C6**	110.4069	112.1813	113.2342	113.1372	114.7166	113.0823	115.1142	113.0250	114.8896	113.1903	115.4863	110.1809	112.0242	113.1047	112.9314	114.4672	112.8798	114.8442	112.8259	114.6752	112.9818	115.1008	**112.1**
**C7**	191.1783	195.0592	196.3405	197.0699	198.0032	196.9526	201.5361	196.8329	200.7591	197.1822	202.2738	192.7128	196.2309	197.5011	198.0639	199.4455	197.9479	202.8024	197.8297	201.9453	198.1760	203.5027	**194.5**
**C8**	71.4856	72.2249	72.7672	72.5613	73.2980	72.5432	73.5879	72.5231	73.5167	72.5800	74.1214	63.5660	64.6615	65.3151	65.6520	66.7735	65.6137	66.9966	65.5735	66.8262	65.6903	67.4190	**58.4**
**C9**	27.3817	28.2799	28.6395	28.9932	29.5289	28.9381	29.6567	28.8839	29.5426	29.0505	29.7187	27.8953	27.9558	28.0274	28.5419	28.7874	28.5400	28.6536	28.5364	28.6330	28.5454	28.8949	**15**
**C8`**	40.5326	40.9917	41.0961	41.2845	41.4705	41.2682	41.5452	41.2499	41.5351	41.3016	41.9592	35.6840	36.4721	36.4542	36.8912	37.2295	36.8664	37.3102	36.8396	37.2360	36.9162	37.6915	**30.6**
**H2**	7.9505	7.9214	7.9800	7.8838	7.9436	7.8869	7.9781	7.8900	7.9781	7.8806	7.9767	7.9894	7.9329	7.9810	7.8988	7.8971	7.9024	7.9258	7.9060	7.9343	7.8953	7.9209	**7.77**
**H4**	6.9210	7.1330	7.2738	7.2145	7.4005	7.2102	7.4386	7.2058	7.4219	7.2185	7.4660	6.9619	7.1593	7.2992	7.2489	7.4289	7.2448	7.4639	7.2407	7.4473	7.2527	7.4842	**7.71**
**H6**	7.4961	7.6999	7.8382	7.8141	8.0312	7.8064	8.0578	7.7989	8.0390	7.8216	8.0949	7.1529	7.3593	7.4825	7.3795	7.6045	7.3738	7.6326	7.3680	7.6129	7.3849	7.6674	**7.77**
**H8**	4.2838	4.3651	4.3977	4.4448	4.5240	4.4385	4.5551	4.4325	4.5353	4.4511	4.5771	4.5819	4.7449	4.8133	4.7860	4.9358	4.7803	4.9634	4.7747	4.9426	4.7914	4.9967	**5.21**
**H(9A-9C)**	1.5988	1.6163	1.6026	1.6251	1.6006	1.6243	1.6225	1.6237	1.6177	1.6260	1.6324	1.8175	1.8343	1.8045	1.7137	1.6896	1.7132	1.7080	1.7128	1.7028	1.7142	1.7182	**1.43**
**H(8`A-8`C)**	2.6256	2.5899	2.5626	2.5759	2.5414	2.5766	2.5379	2.5776	2.5393	2.5752	2.5209	2.8621	2.7762	2.7019	2.6401	2.5700	2.6422	2.5503	2.6445	2.5608	2.6381	2.5171	**2.58**
Nuclei	wB97																						
**C1**	145.0826	144.9223	145.9029	145.0505	146.2222	145.0328	145.3538	145.0135	145.8907	145.0655	145.4755	144.2803	144.4261	145.5347	145.5296	146.4749	145.5118	146.6544	145.4943	146.3506	145.5484	146.8025	**136.1**
**C2**	118.1726	118.2742	119.0406	118.2213	119.0099	118.2243	119.4293	118.2267	119.3604	118.2189	119.4690	117.9485	118.1550	118.9170	118.2967	119.0518	118.2952	119.4923	118.2931	119.3739	118.2986	119.5328	**112.1**
**C3**	172.0070	171.9914	172.4482	172.0128	172.6148	172.0097	172.5295	172.0060	172.5450	172.0161	172.5793	172.0384	171.9949	172.4605	172.0596	172.6431	172.0558	172.6090	172.0517	172.5861	172.0637	172.6669	**162.6**
**C4**	113.2888	114.7490	115.4215	115.4241	116.2153	115.3840	117.0173	115.3432	116.7046	115.4627	117.1969	113.4176	114.7417	115.3931	115.1702	116.0260	115.1343	116.4766	115.0972	116.3765	115.2043	116.6526	**110.1**
**C5**	171.0046	171.3571	171.8358	171.6189	172.3403	171.5997	172.2476	171.5803	172.2210	171.6378	172.3346	171.0775	171.3627	171.8136	171.5531	172.2290	171.5365	172.0609	171.5193	172.0466	171.5695	172.1434	**162.5**
**C6**	115.9785	117.9769	119.0565	118.9213	120.7117	118.8665	121.0521	118.8112	120.6774	118.9744	121.3902	116.3838	118.4291	119.6122	119.1708	121.1674	119.1165	121.2452	119.0611	121.2338	119.2221	121.5429	**112.1**
**C7**	200.1093	204.7281	206.3248	207.3562	208.0548	207.1858	212.9349	207.0137	212.2226	207.5165	213.7911	203.5137	207.6133	209.1336	210.1467	210.7829	210.0042	215.8404	209.8607	214.5107	210.2822	216.5410	**194.5**
**C8**	61.6202	62.5762	63.2299	63.0115	63.8145	62.9864	64.2175	62.9609	64.1337	63.0364	64.8408	56.2260	57.3419	58.1165	58.4065	59.1322	58.3670	59.8998	58.3272	59.6263	58.4454	60.1581	**58.4**
**C9**	21.6048	21.7553	21.9731	21.9694	22.3893	21.9515	22.2642	21.9325	22.1502	21.9863	22.3841	19.2381	19.2990	19.4243	19.3675	19.7073	19.3674	19.6431	19.3663	19.6338	19.3674	19.9386	**15**
**C8`**	35.0716	35.7062	35.9678	36.0640	36.4100	36.0444	36.5589	36.0243	36.4966	36.0835	36.9939	30.3904	30.8863	31.0502	30.9975	31.3322	30.9739	31.2648	30.9497	31.2906	31.0203	31.4255	**30.6**
**H2**	8.1073	8.0899	8.1395	8.0383	8.1248	8.0423	8.1608	8.0464	8.1212	8.0347	8.1593	8.1326	8.0849	8.1020	7.9343	8.0324	7.9375	7.9647	7.9404	7.9979	7.9313	7.9669	**7.77**
**H4**	7.1872	7.3977	7.5404	7.4745	7.6675	7.4702	7.7068	7.4658	7.6881	7.4785	7.7293	7.1904	7.3904	7.5305	7.4542	7.6469	7.4504	7.6702	7.4464	7.6582	7.4579	7.6921	**7.71**
**H6**	7.5486	7.7850	7.9139	7.8803	8.1113	7.8749	8.1459	7.8695	8.1044	7.8855	8.1828	7.4139	7.6493	7.7974	7.7138	7.9807	7.7080	7.9500	7.7020	7.9567	7.7192	7.9905	**7.77**
**H8**	3.9702	4.0846	4.1184	4.1610	4.2571	4.1551	4.2909	4.1492	4.2740	4.1667	4.3126	4.1904	4.3475	4.4334	4.4273	4.5640	4.4225	4.5663	4.4175	4.5563	4.4318	4.5933	**5.21**
**H(9A-9C)**	1.3175	1.3402	1.3126	1.3274	1.3084	1.3286	1.3219	1.3300	1.3042	1.3263	1.3411	1.3947	1.4344	1.4039	1.4225	1.4124	1.4219	1.4134	1.4212	1.4202	1.4231	1.4308	**1.43**
**H(8`A-8`C)**	2.4496	2.4001	2.3793	2.3914	2.3555	2.3914	2.3419	2.3914	2.3537	2.3913	2.3288	2.7846	2.6734	2.6007	2.5656	2.5306	2.5682	2.4343	2.5709	2.4590	2.5630	2.4091	**2.58**
Nuclei	wB97X																						
**C1**	146.1533	146.0408	147.0630	146.3235	147.4985	146.3094	146.7087	146.2899	147.4259	146.3337	146.7758	145.2819	145.4934	146.6234	146.6407	147.5883	146.6277	148.5151	146.6145	147.5264	146.6549	148.3190	**136.1**
**C2**	118.8280	118.9086	119.6873	118.8638	119.6383	118.8692	120.1058	118.8732	119.9772	118.8594	120.1250	118.5660	118.7553	119.5141	118.8998	119.6210	118.9016	120.1059	118.9027	119.9349	118.8994	120.1334	**112.1**
**C3**	172.7139	172.7559	173.2264	172.8015	173.4277	172.7999	173.3966	172.7976	173.3654	172.8038	173.4317	172.7736	172.7904	173.2737	172.8788	173.4875	172.8770	173.5371	172.8747	173.4168	172.8819	173.5665	**162.6**
**C4**	113.7735	115.2536	115.8992	115.9264	116.6794	115.8879	117.5491	115.8493	117.1411	115.9643	117.7262	113.9279	115.2663	115.8959	115.7017	116.5290	115.6675	116.8714	115.6320	116.8556	115.7349	117.1119	**110.1**
**C5**	171.7216	172.1256	172.6258	172.4202	173.1643	172.4021	173.1174	172.3834	173.0385	172.4384	173.1939	171.8205	172.1586	172.6298	172.3663	173.0788	172.3517	172.9999	172.3363	172.8833	172.3814	173.0482	**162.5**
**C6**	116.5474	118.5245	119.5713	119.4110	121.1714	119.3587	121.5130	119.3074	120.9845	119.4636	121.8886	116.9492	118.9693	120.1294	119.7106	121.6734	119.6566	121.3860	119.6015	121.6875	119.7628	121.8870	**112.1**
**C7**	201.8675	206.5338	208.1370	209.3110	209.9762	209.1429	215.0236	208.9703	214.5731	209.4676	215.7754	205.5387	209.6469	211.1262	212.1533	212.7801	212.0169	218.6313	211.8787	216.5475	212.2840	218.9106	**194.5**
**C8**	62.6875	63.6992	64.3519	64.1313	64.9471	64.1039	65.3578	64.0753	65.2340	64.1577	66.0205	57.2642	58.4161	59.2042	59.5452	60.2474	59.5068	61.1728	59.4682	60.7630	59.5834	61.4158	**58.4**
**C9**	21.8596	22.0221	22.2439	22.2484	22.6326	22.2383	22.5022	22.2274	22.2411	22.2596	22.6780	19.3448	19.3922	19.5193	19.5010	19.8116	19.5023	19.6051	19.5028	19.7154	19.5009	20.0120	**15**
**C8`**	35.3135	36.0071	36.2338	36.3520	36.7174	36.3308	36.8859	36.3086	36.7846	36.3729	37.3249	30.6160	31.0923	31.2302	31.1627	31.5000	31.1419	31.4760	31.1203	31.4397	31.1835	31.5930	**30.6**
**H2**	8.0877	8.0686	8.1159	8.0053	8.0919	8.0093	8.1262	8.0138	8.0641	8.0019	8.1269	8.1197	8.0643	8.0830	7.9130	8.0118	7.9160	7.8863	7.9189	7.9697	7.9102	7.9141	**7.77**
**H4**	7.1555	7.3665	7.5075	7.4458	7.6352	7.4416	7.6762	7.4374	7.6552	7.4498	7.6974	7.1630	7.3625	7.5012	7.4292	7.6189	7.4255	7.6413	7.4216	7.6291	7.4329	7.6628	**7.71**
**H6**	7.5293	7.7636	7.8866	7.8553	8.0797	7.8498	8.1141	7.8445	8.0651	7.8609	8.1517	7.3888	7.6136	7.7621	7.6811	7.9460	7.6753	7.8840	7.6694	7.9178	7.6868	7.9366	**7.77**
**H8**	4.0316	4.1351	4.1676	4.2171	4.3034	4.2119	4.3468	4.2067	4.3449	4.2222	4.3621	4.2337	4.3870	4.4706	4.4696	4.6064	4.4651	4.6162	4.4604	4.5973	4.4741	4.6360	**5.21**
**H(9A-9C)**	1.3041	1.3261	1.2975	1.3075	1.2880	1.3088	1.2990	1.3102	1.2636	1.3066	1.3214	1.3829	1.4235	1.3954	1.4156	1.4040	1.4147	1.3676	1.4140	1.4060	1.4164	1.4032	**1.43**
**H(8`A-8`C)**	2.4586	2.4067	2.3861	2.4003	2.3618	2.4010	2.3484	2.4015	2.3566	2.3997	2.3332	2.8075	2.6909	2.6191	2.5864	2.5511	2.5893	2.4477	2.5920	2.4749	2.5837	2.4178	**2.58**

[a] methyl protons shifts are averaged

[b] according to [8]

Table S8. Cont. VII

Nuclei	R											S											Exp. [b]
	X3LYP																						
	Gas	$CHCl_3$ (PCM)	$CHCl_3$ (SMD)	DMSO (PCM)	DMSO (SMD)	CH_3OH (PCM)	CH_3OH (SMD)	C_2H_5OH (PCM)	C_2H_5OH (SMD)	H_2O (PCM)	H_2O (SMD)	Gas	$CHCl_3$ (PCM)	$CHCl_3$ (SMD)	DMSO (PCM)	DMSO (SMD)	CH_3OH (PCM)	CH_3OH (SMD)	C_2H_5OH (PCM)	C_2H_5OH (SMD)	H_2O (PCM)	H_2O (SMD)	
**C1**	146.5131	146.8886	148.1114	147.7279	148.5350	147.7222	147.8140	147.7144	148.3180	147.7403	147.9695	145.2469	145.8238	146.9766	146.9756	148.1417	146.9865	148.4999	146.9916	148.2323	146.9661	148.4624	**136.1**
**C2**	118.2943	118.4446	119.2449	118.5465	119.1345	118.5562	119.6437	118.5626	119.6028	118.5413	119.6659	117.9804	118.1916	118.8999	118.3918	119.0352	118.4002	119.6544	118.4049	119.5052	118.3866	119.6400	**112.1**
**C3**	173.8687	174.0969	174.6254	174.1783	174.9047	174.1774	174.8405	174.1732	174.8510	174.1828	174.8963	173.8692	174.0976	174.6471	174.2208	174.9462	174.2190	174.9203	174.2140	174.9156	174.2260	174.9690	**162.6**
**C4**	113.4075	114.9495	115.5069	115.5806	116.2576	115.5400	117.2070	115.4961	116.8765	115.6221	117.3707	113.7360	115.0424	115.5709	115.4450	116.1044	115.4087	116.6601	115.3698	116.5481	115.4831	116.8709	**110.1**
**C5**	173.0715	173.6274	174.2204	173.9225	174.7983	173.9075	174.7187	173.8893	174.6709	173.9401	174.7883	173.3300	173.7791	174.2988	173.9554	174.7688	173.9436	174.6024	173.9292	174.6114	173.9703	174.6701	**162.5**
**C6**	116.3968	118.2160	119.1487	118.9222	120.7628	118.8635	121.1769	118.8015	120.8400	118.9807	121.4949	116.7224	118.7434	119.9230	119.5108	121.3023	119.4453	121.3395	119.3777	121.2933	119.5777	121.7341	**112.1**
**C7**	202.9876	208.0050	209.8673	211.3345	211.6414	211.1695	216.5488	211.0033	215.8373	211.4987	217.3552	207.8705	211.8760	213.2107	214.0825	214.9054	213.9813	219.9420	213.8748	218.7319	214.1806	220.3322	**194.5**
**C8**	65.5754	66.6287	67.3695	67.2047	67.9103	67.1847	68.4674	67.1606	68.4791	67.2266	69.1711	60.1100	61.5763	62.4770	62.8571	63.5886	62.8163	64.4103	62.7715	64.1073	62.8984	64.6548	**58.4**
**C9**	22.7411	23.0238	23.0347	23.0706	23.4886	23.0585	23.5335	23.0440	23.4491	23.0858	23.6756	19.3798	19.6455	19.8964	19.8878	20.1551	19.8747	20.1559	19.8583	20.1226	19.9038	20.5216	**15**
**C8`**	36.2822	37.0691	37.3895	37.5204	37.8660	37.5004	38.0025	37.4765	37.9679	37.5421	38.4156	31.7762	32.2300	32.2895	32.1927	32.5392	32.1770	32.5622	32.1588	32.5884	32.2120	32.6385	**30.6**
**H2**	7.9718	7.9297	7.9690	7.8247	7.9488	7.8278	7.9838	7.8303	7.9445	7.8215	7.9780	8.0353	7.9546	7.9777	7.8211	7.8992	7.8218	7.8426	7.8222	7.8688	7.8207	7.8562	**7.77**
**H4**	7.0673	7.2752	7.4142	7.3538	7.5367	7.3495	7.5783	7.3447	7.5593	7.3578	7.5988	7.0821	7.2773	7.4133	7.3435	7.5260	7.3397	7.5537	7.3354	7.5418	7.3470	7.5748	**7.71**
**H6**	7.4687	7.6954	7.8099	7.7748	8.0026	7.7677	8.0371	7.7601	7.9973	7.7814	8.0669	7.2926	7.5069	7.6512	7.5681	7.8151	7.5616	7.7900	7.5548	7.7900	7.5743	7.8393	**7.77**
**H8**	4.2514	4.3527	4.3701	4.4145	4.5091	4.4091	4.5334	4.4033	4.5102	4.4200	4.5527	4.3907	4.5522	4.6269	4.6326	4.7652	4.6282	4.7866	4.6232	4.7722	4.6368	4.8159	**5.21**
**H(9A-9C)**	1.3127	1.3128	1.2832	1.2763	1.2711	1.2771	1.2954	1.2773	1.2805	1.2753	1.3103	1.3927	1.4352	1.4227	1.4420	1.4235	1.4394	1.4173	1.4363	1.4214	1.4445	1.4458	**1.43**
**H(8`A-8`C)**	2.5092	2.4567	2.4317	2.4494	2.4045	2.4506	2.3955	2.4513	2.4080	2.4482	2.3785	2.9338	2.8045	2.7332	2.7037	2.6564	2.7067	2.5696	2.7094	2.5917	2.7010	2.5374	**2.58**

[a] methyl protons shifts are averaged

[b] according to [8]

Table S9. Theoretical chemical shifts (in ppm) of *(R,S)*-flephedrone calculated with HF and selected functionals combined with 6-311++G** basis set[a]

Nuclei	R											S											**Exp.** [b]
	**B3LYP**																						
	Gas	$CHCl_3$ (PCM)	$CHCl_3$ (SMD)	DMSO (PCM)	DMSO (SMD)	CH_3OH (PCM)	CH_3OH (SMD)	C_2H_5OH (PCM)	C_2H_5OH (SMD)	H_2O (PCM)	H_2O (SMD)	Gas	$CHCl_3$ (PCM)	$CHCl_3$ (SMD)	DMSO (PCM)	DMSO (SMD)	CH_3OH (PCM)	CH_3OH (SMD)	C_2H_5OH (PCM)	C_2H_5OH (SMD)	H_2O (PCM)	H_2O (SMD)	
**C1**	139.3516	139.8992	140.9781	140.1863	142.0735	140.1611	141.5120	140.1358	141.5012	140.2082	141.7819	137.8738	139.3186	139.9816	139.9577	141.5554	139.9093	141.4183	139.8637	141.2810	140.0057	141.4292	**129**
**C2**	139.7181	139.6929	140.2565	139.4633	139.9708	139.4799	140.4199	139.4943	140.2834	139.4440	140.3925	139.0099	139.2247	139.7699	139.1806	139.8028	139.1780	140.3740	139.1755	140.2362	139.1821	140.3704	**132**
**C3**	121.9147	122.6511	123.2036	122.8744	123.4797	122.8576	123.7880	122.8405	123.7263	122.8887	123.8478	122.0546	122.6999	123.1853	122.8846	123.4913	122.8701	123.7328	122.8554	123.6526	122.8966	123.7867	**116**
**C4**	176.1996	177.1548	177.8016	177.6113	178.2556	177.5774	178.9142	177.5440	178.7922	177.6426	179.0429	176.5723	177.1853	177.8667	177.4190	178.1469	177.4023	178.5696	177.3850	178.4546	177.4322	178.7245	**167**
**C5**	119.7381	121.1100	121.7159	121.7876	122.5392	121.7417	122.8148	121.6959	122.7064	121.8299	122.9605	119.9339	121.1749	121.7562	121.7216	122.4902	121.6855	122.6663	121.6489	122.5622	121.7540	122.7986	**116**
**C6**	135.8982	137.9272	138.9534	138.9372	140.4067	138.8712	141.0202	138.8048	140.7434	138.9983	141.3636	136.2778	138.3528	139.6434	139.3323	140.9568	139.2716	141.4709	139.2095	141.2669	139.3872	141.8771	**132**
**C7**	202.8768	208.0691	209.7045	210.9565	211.8939	210.7561	217.3250	210.5621	216.3636	211.1520	218.3042	208.1087	212.3579	213.4837	214.5278	215.3474	214.3946	220.1153	214.2618	218.9963	214.6532	220.5944	**196**
**C8**	65.2601	66.4393	67.0844	66.6651	67.6873	66.6448	68.1168	66.6260	67.8105	66.6832	68.8556	59.4742	61.6544	62.2820	62.6180	63.4923	62.5505	64.2487	62.4848	63.9791	62.6825	64.4890	**60**
**C9**	22.9937	23.3369	23.4371	23.5103	23.7870	23.5040	23.8454	23.4947	23.7278	23.5117	23.9610	19.5432	20.0549	20.2357	20.2537	20.4684	20.2366	20.6702	20.2197	20.5736	20.2683	21.0311	**15**
**C8`**	36.5076	37.3387	37.5483	37.6307	37.9535	37.5944	38.1848	37.5637	38.1616	37.6692	38.6153	32.1915	32.3577	32.6229	32.4742	32.7092	32.4611	32.8316	32.4474	32.8288	32.4845	32.9175	**31**
**H2**	8.6288	8.5708	8.5739	8.5193	8.4862	8.5229	8.5099	8.5264	8.5151	8.5156	8.4958	8.6701	8.4976	8.5384	8.4115	8.4156	8.4168	8.3988	8.4218	8.4120	8.4060	8.4143	**8.07**
**H3**	7.3119	7.4474	7.5313	7.4874	7.6000	7.4852	7.6152	7.4829	7.6051	7.4891	7.6305	7.2978	7.4323	7.5140	7.4748	7.5907	7.4723	7.6048	7.4697	7.5943	7.4770	7.6211	**7.31**
**H5**	7.1614	7.3510	7.4352	7.4344	7.5536	7.4291	7.5704	7.4238	7.5589	7.4393	7.5939	7.1540	7.3281	7.4120	7.4019	7.5234	7.3974	7.5322	7.3928	7.5197	7.4059	7.5549	**7.31**
**H6**	8.0695	8.2736	8.3471	8.3829	8.4942	8.3751	8.5470	8.3677	8.5388	8.3904	8.5777	7.8720	8.0370	8.1480	8.1191	8.2781	8.1143	8.2908	8.1093	8.2781	8.1233	8.3419	**8.07**
**H8**	4.3623	4.4552	4.4783	4.5600	4.6044	4.5533	4.6526	4.5464	4.6504	4.5660	4.6741	4.4999	4.6578	4.7050	4.7188	4.8309	4.7153	4.8477	4.7118	4.8326	4.7217	4.8820	**5.09**
**H(9A-9C)**	1.3154	1.3242	1.3019	1.3025	1.2691	1.3041	1.2806	1.3055	1.2685	1.3007	1.2933	1.3888	1.4276	1.4302	1.4455	1.4215	1.4441	1.4397	1.4427	1.4360	1.4466	1.4683	**1.6**
**H(8`A-8`C)**	2.5071	2.4491	2.4216	2.4281	2.4102	2.4290	2.3990	2.4298	2.3931	2.4269	2.3861	2.9770	2.8044	2.7564	2.7204	2.6721	2.7257	2.5872	2.7307	2.6042	2.7151	2.5595	**2.81**
**F4**	-124.5867	-125.7404	-128.6276	-125.7622	-130.2938	-125.7680	-128.0611	-125.7715	-128.3171	-125.7554	-127.8310	-124.2856	-126.1284	-128.8884	-126.5189	-130.7445	-126.4983	-129.0169	-126.4776	-129.2395	-126.5402	-128.6511	**-102.1**
Nuclei	**B3P86**																						
**C1**	136.8453	137.2808	138.3582	137.5043	139.4519	137.5074	138.8537	137.4898	139.1351	137.5460	139.1283	135.6233	136.9063	137.5597	137.4853	138.9540	137.4549	138.8749	137.4076	138.7415	137.5634	138.9677	**129**
**C2**	138.7919	138.7416	139.3998	138.5747	139.1636	138.6075	139.5971	138.6182	139.5645	138.5873	139.5816	138.1598	138.3322	138.9376	138.3113	138.9809	138.3306	139.5077	138.3287	139.4734	138.3375	139.5260	**132**
**C3**	121.1158	121.8832	122.5099	122.1552	122.8745	122.1613	123.1693	122.1453	123.2080	122.1932	123.2475	121.2077	121.9036	122.4956	122.1573	122.8618	122.1638	123.0956	122.1483	123.1217	122.1950	123.1750	**116**
**C4**	173.6985	174.6092	175.2829	175.0662	175.7535	175.0598	176.4063	175.0315	176.3576	175.1159	176.5491	173.9907	174.6001	175.3439	174.8861	175.6462	174.8935	176.0480	174.8781	176.0672	174.9218	176.2106	**167**
**C5**	118.9511	120.3570	121.0389	121.0865	121.9545	121.0629	122.2291	121.0175	122.2236	121.1526	122.3983	119.0968	120.3750	121.0516	121.0069	121.8780	120.9911	122.0417	120.9530	122.0531	121.0653	122.1965	**116**
**C6**	134.9391	136.9571	138.0470	138.0317	139.4694	137.9871	140.0383	137.9202	139.8454	138.1179	140.4254	135.2870	137.3568	138.6630	138.3561	140.0839	138.3212	140.5742	138.2627	140.5163	138.4312	140.9668	**132**
**C7**	200.9937	205.8309	207.3690	208.3757	209.4937	208.2408	214.5962	208.0849	213.8337	208.5478	215.4998	205.5040	209.6504	210.9178	211.8502	212.6202	211.7370	217.2310	211.6033	216.1910	212.0035	217.7281	**196**
**C8**	63.6796	64.8464	65.6285	65.2068	66.1771	65.2071	66.5812	65.1854	66.4957	65.2505	67.3827	58.1084	59.9428	60.4781	60.8007	61.6333	60.7568	62.3872	60.6942	62.1777	60.8896	62.6693	**60**
**C9**	22.8065	23.0897	23.3077	23.3623	23.6359	23.3647	23.6593	23.3452	23.6707	23.4035	23.8216	19.4663	20.0187	20.1559	20.2089	20.4941	20.2153	20.6857	20.2003	20.7001	20.2472	21.1119	**15**
**C8`**	35.9286	36.7158	37.0084	37.0027	37.3475	37.0082	37.5596	36.9918	37.6189	37.0414	38.0311	31.3622	31.5731	31.9325	31.7646	32.0364	31.7748	32.1533	31.7622	32.2999	31.7969	32.2578	**31**
**H2**	8.6937	8.6392	8.6481	8.5947	8.5586	8.5992	8.5883	8.6018	8.5814	8.5915	8.5744	8.7028	8.5480	8.5959	8.4713	8.4868	8.4787	8.4611	8.4838	8.4913	8.4649	8.4716	**8.07**
**H3**	7.3323	7.4749	7.5633	7.5186	7.6361	7.5176	7.6523	7.5147	7.6476	7.5207	7.6690	7.3188	7.4600	7.5477	7.5057	7.6274	7.5044	7.6414	7.5013	7.6367	7.5080	7.6590	**7.31**
**H5**	7.1767	7.3724	7.4616	7.4573	7.5836	7.4536	7.6013	7.4480	7.5944	7.4620	7.6265	7.1655	7.3489	7.4382	7.4270	7.5553	7.4237	7.5640	7.4186	7.5573	7.4312	7.5880	**7.31**
**H6**	8.1523	8.3571	8.4270	8.4597	8.5681	8.4550	8.6211	8.4484	8.6073	8.4654	8.6536	7.9340	8.1076	8.2276	8.1948	8.3652	8.1917	8.3755	8.1865	8.3750	8.1986	8.4257	**8.07**
**H8**	4.3930	4.4836	4.4927	4.5762	4.6265	4.5711	4.6819	4.5642	4.6796	4.5824	4.7038	4.5540	4.6976	4.7569	4.7623	4.8783	4.7605	4.8928	4.7566	4.8883	4.7650	4.9288	**5.09**
**H(9A-9C)**	1.3335	1.3438	1.3286	1.3321	1.2867	1.3343	1.2965	1.3346	1.2886	1.3313	1.3143	1.4092	1.4428	1.4421	1.4554	1.4353	1.4561	1.4505	1.4549	1.4566	1.4559	1.4796	**1.6**
**H(8`A-8`C)**	2.4876	2.4212	2.3951	2.4005	2.3811	2.4023	2.3602	2.4024	2.3671	2.4001	2.3503	2.9176	2.7485	2.7146	2.6728	2.6287	2.6800	2.5349	2.6851	2.5651	2.6670	2.5072	**2.81**
**F4**	-117.1651	-117.8836	-120.5922	-117.7226	-121.9927	-117.7352	-119.6541	-117.7485	-120.0548	-117.7094	-119.4165	-117.0081	-118.3431	-120.8782	-118.5063	-122.4355	-118.4942	-120.6607	-118.4844	-120.8650	-118.5187	-120.3178	**-102.1**
Nuclei	**B3PW91**																						
**C1**	137.0994	137.5782	138.7234	137.8703	140.2084	137.8485	139.3905	137.8187	139.3095	137.8939	139.6388	135.8270	137.2800	137.7781	137.8580	139.3488	137.8089	139.3951	137.7573	139.1374	137.9141	139.4828	**129**
**C2**	138.8604	138.8385	139.3967	138.6503	139.5459	138.6706	139.6266	138.6815	139.5161	138.6320	139.6248	138.2843	138.4871	139.0226	138.4452	139.1209	138.4505	139.6147	138.4477	139.5068	138.4432	139.6376	**132**
**C3**	121.2185	122.0069	122.5742	122.2708	123.2963	122.2610	123.2495	122.2421	123.2072	122.2821	123.3409	121.3071	122.0247	122.5329	122.2639	122.9651	122.2553	123.1906	122.2380	123.1214	122.2739	123.2752	**116**
**C4**	173.7409	174.6858	175.2871	175.1369	176.1105	175.1138	176.3924	175.0819	176.3209	175.1612	176.5562	174.0199	174.6397	175.3121	174.9084	175.6627	174.9018	176.0329	174.8849	175.9623	174.9153	176.2007	**167**
**C5**	119.0599	120.4861	121.1030	121.2065	122.3683	121.1668	122.2995	121.1183	122.2138	121.2461	122.4777	119.2098	120.5084	121.1087	121.1148	121.9791	121.0849	122.1342	121.0458	122.0478	121.1449	122.2957	**116**
**C6**	134.8881	136.9286	137.8816	137.9622	139.7104	137.9061	139.9572	137.8415	139.7281	138.0174	140.3326	135.2770	137.3211	138.5843	138.3076	139.9907	138.2583	140.3962	138.1982	140.2701	138.3538	140.7941	**132**
**C7**	201.1247	206.1066	207.7353	208.8082	210.2420	208.6327	215.1374	208.4510	214.2009	208.9795	216.0833	205.6165	209.9651	211.0502	212.1644	212.9527	212.0348	217.7726	211.8977	216.5723	212.2928	218.2699	**196**
**C8**	63.4567	64.6178	65.2257	64.9342	66.2583	64.9202	66.4879	64.8987	66.1020	64.9510	67.2318	58.0320	59.9926	60.4452	60.8767	61.6836	60.8128	62.5182	60.7445	62.2018	60.9444	62.8000	**60**
**C9**	22.5160	22.8553	22.9353	23.0789	23.6687	23.0753	23.3953	23.0617	23.2786	23.0832	23.5418	19.1832	19.7035	19.7883	19.8973	20.1279	19.8867	20.3038	19.8683	20.2280	19.9102	20.7016	**15**
**C8`**	35.8834	36.7085	36.8598	36.9828	37.6339	36.9657	37.5129	36.9420	37.5231	37.0038	37.9753	31.4292	31.6510	31.9335	31.8297	32.1026	31.8260	32.1558	31.8123	32.2185	31.8333	32.2536	**31**
**H2**	8.6633	8.6108	8.6056	8.5605	8.5329	8.5652	8.5339	8.5693	8.5526	8.5562	8.5242	8.6835	8.5130	8.5718	8.4375	8.4524	8.4445	8.4188	8.4505	8.4482	8.4303	8.4283	**8.07**
**H3**	7.3301	7.4703	7.5577	7.5143	7.6458	7.5126	7.6483	7.5105	7.6406	7.5160	7.6634	7.3189	7.4577	7.5443	7.5040	7.6238	7.5020	7.6404	7.4996	7.6317	7.5060	7.6558	**7.31**
**H5**	7.1771	7.3713	7.4596	7.4576	7.5958	7.4530	7.5980	7.4480	7.5902	7.4622	7.6214	7.1690	7.3494	7.4382	7.4277	7.5538	7.4237	7.5651	7.4194	7.5542	7.4316	7.5870	**7.31**
**H6**	8.1341	8.3392	8.4057	8.4426	8.5635	8.4366	8.5897	8.4305	8.5951	8.4485	8.6240	7.9194	8.0892	8.2103	8.1769	8.3421	8.1732	8.3457	8.1688	8.3441	8.1801	8.3934	**8.07**
**H8**	4.3741	4.4705	4.4922	4.5694	4.6265	4.5633	4.6458	4.5568	4.6625	4.5752	4.6720	4.5270	4.6702	4.7258	4.7346	4.8501	4.7322	4.8642	4.7292	4.8566	4.7367	4.8976	**5.09**
**H(9A-9C)**	1.2980	1.3052	1.2789	1.2881	1.2601	1.2903	1.2615	1.2922	1.2492	1.2862	1.2747	1.3734	1.4034	1.4070	1.4206	1.3953	1.4203	1.4103	1.4196	1.4138	1.4210	1.4367	**1.6**
**H(8`A-8`C)**	2.4555	2.3914	2.3685	2.3743	2.3708	2.3754	2.3485	2.3764	2.3407	2.3732	2.3328	2.8889	2.7195	2.6844	2.6446	2.6004	2.6510	2.5113	2.6569	2.5341	2.6383	2.4811	**2.81**
**F4**	-116.5680	-117.3601	-120.1088	-117.2553	-120.4514	-117.2656	-119.3899	-117.2753	-119.5897	-117.2446	-119.1158	-116.3972	-117.8565	-120.3794	-118.0672	-122.0110	-118.0509	-120.3403	-118.0375	-120.5104	-118.0852	-119.9935	**-102.1**
Nuclei	**B98**																						
**C1**	137.8984	138.3641	139.2698	138.5615	140.4825	138.5589	139.6880	138.5551	139.9714	138.5675	140.0693	136.6672	137.9623	138.5980	138.5797	140.1392	138.5482	140.0261	138.5178	139.8988	138.6168	140.0992	**129**
**C2**	138.5254	138.4721	139.0519	138.3240	138.8948	138.3503	139.3548	138.3736	139.2402	138.3008	139.3473	137.9447	138.0861	138.6429	138.0628	138.7300	138.0773	139.2568	138.0902	139.1509	138.0518	139.2683	**132**
**C3**	120.9751	121.6883	122.2401	121.9412	122.6168	121.9423	122.8875	121.9406	122.8295	121.9422	122.9429	121.0500	121.6961	122.2005	121.9352	122.6051	121.9366	122.8272	121.9354	122.7659	121.9361	122.8940	**116**
**C4**	173.5548	174.4326	175.0664	174.8850	175.5697	174.8724	176.2406	174.8571	176.0696	174.8994	176.3345	173.8262	174.4272	175.1004	174.7024	175.4540	174.7034	175.8475	174.7008	175.7561	174.7028	176.0007	**167**
**C5**	118.7946	120.1408	120.7556	120.8501	121.6768	120.8218	121.9460	120.7906	121.8434	120.8797	122.0849	118.9548	120.1808	120.7785	120.7832	121.6150	120.7632	121.7668	120.7403	121.6817	120.8040	121.9090	**116**
**C6**	134.9458	136.9033	137.9770	137.9779	139.4196	137.9297	140.0378	137.8779	139.7250	138.0256	140.3768	135.2851	137.2480	138.5012	138.2327	139.8890	138.1917	140.3423	138.1470	140.1749	138.2722	140.7184	**132**
**C7**	199.5271	204.5615	206.0295	207.1995	208.2386	207.0479	213.5136	206.8943	212.6014	207.3482	214.4291	204.1599	208.4637	209.6427	210.7144	211.5176	210.5930	216.3722	210.4697	215.2290	210.8323	216.8957	**196**
**C8**	65.0962	66.2575	66.8997	66.5776	67.5549	66.5763	67.8574	66.5723	67.7776	66.5806	68.7079	59.7427	61.6256	62.1747	62.5454	63.3980	62.4979	64.1348	62.4503	63.8905	62.5954	64.3856	**60**
**C9**	23.2897	23.5779	23.6929	23.8388	24.0941	23.8397	24.1215	23.8371	24.0489	23.8399	24.2881	20.0303	20.4587	20.5633	20.6605	20.8945	20.6623	21.0556	20.6621	20.9805	20.6615	21.4185	**15**
**C8`**	36.7092	37.4629	37.6740	37.7309	38.1053	37.7287	38.3351	37.7243	38.2965	37.7357	38.7417	32.2204	32.4032	32.6737	32.5780	32.8420	32.5807	32.9732	32.5806	33.0077	32.5770	33.0565	**31**
**H2**	8.6652	8.6018	8.6259	8.5647	8.5366	8.5677	8.5885	8.5705	8.5669	8.5621	8.5601	8.6821	8.5181	8.5678	8.4391	8.4515	8.4446	8.4365	8.4498	8.4543	8.4338	8.4469	**8.07**
**H3**	7.3240	7.4617	7.5525	7.5069	7.6251	7.5048	7.6424	7.5026	7.6335	7.5092	7.6562	7.3088	7.4478	7.5364	7.4945	7.6157	7.4921	7.6318	7.4897	7.6238	7.4970	7.6471	**7.31**
**H5**	7.1716	7.3643	7.4556	7.4510	7.5764	7.4460	7.5983	7.4411	7.5859	7.4560	7.6179	7.1649	7.3464	7.4371	7.4256	7.5523	7.4212	7.5629	7.4169	7.5523	7.4299	7.5837	**7.31**
**H6**	8.0929	8.2967	8.3821	8.4075	8.5267	8.4008	8.6016	8.3943	8.5719	8.4141	8.6200	7.8961	8.0681	8.1863	8.1586	8.3256	8.1538	8.3364	8.1488	8.3271	8.1631	8.3835	**8.07**
**H8**	4.3470	4.4366	4.4606	4.5354	4.5891	4.5287	4.6555	4.5220	4.6334	4.5423	4.6625	4.4951	4.6493	4.7037	4.7151	4.8293	4.7118	4.8454	4.7085	4.8323	4.7182	4.8756	**5.09**
**H(9A-9C)**	1.3189	1.3268	1.3143	1.3162	1.2740	1.3175	1.2894	1.3187	1.2788	1.3151	1.3029	1.3937	1.4261	1.4275	1.4419	1.4166	1.4413	1.4301	1.4407	1.4303	1.4425	1.4532	**1.6**
**H(8`A-8`C)**	2.4802	2.4260	2.3972	2.4073	2.3842	2.4082	2.3651	2.4093	2.3720	2.4067	2.3565	2.9208	2.7533	2.7171	2.6803	2.6349	2.6856	2.5498	2.6907	2.5670	2.6754	2.5194	**2.81**
**F4**	-119.6030	-120.4442	-123.1898	-120.3267	-124.6696	-120.3380	-122.3243	-120.3491	-122.7908	-120.3158	-122.1756	-119.4577	-120.8773	-123.5272	-121.1015	-125.1436	-121.0896	-123.4668	-121.0780	-123.6940	-121.1150	-123.1078	**-102.1**

[a] methyl protons shifts are averaged

[b] according to [9]

Table S9. Cont. I

Nuclei	R											S											Exp. [b]
	B971																						
	Gas	$CHCl_3$ (PCM)	$CHCl_3$ (SMD)	DMSO (PCM)	DMSO (SMD)	CH_3OH (PCM)	CH_3OH (SMD)	C_2H_5OH (PCM)	C_2H_5OH (SMD)	H_2O (PCM)	H_2O (SMD)	Gas	$CHCl_3$ (PCM)	$CHCl_3$ (SMD)	DMSO (PCM)	DMSO (SMD)	CH_3OH (PCM)	CH_3OH (SMD)	C_2H_5OH (PCM)	C_2H_5OH (SMD)	H_2O (PCM)	H_2O (SMD)	
**C1**	137.5124	137.9668	138.8036	138.1617	140.0037	138.1528	139.1730	138.1442	139.5653	138.1768	139.6476	136.2962	137.5659	138.2070	138.1911	139.7146	138.1505	139.5849	138.1134	139.4699	138.2405	139.6733	**129**
**C2**	138.2273	138.1790	138.7520	138.0411	138.6151	138.0588	139.0874	138.0759	138.9703	138.0298	139.0743	137.6507	137.7943	138.3494	137.7788	138.4352	137.7852	138.9549	137.7921	138.8493	137.7793	138.9688	**132**
**C3**	120.7723	121.4948	122.0366	121.7575	122.4239	121.7508	122.6953	121.7432	122.6290	121.7695	122.7532	120.8439	121.4989	122.0013	121.7481	122.4060	121.7414	122.6248	121.7343	122.5637	121.7602	122.6979	**116**
**C4**	173.4520	174.3378	174.9730	174.7959	175.4831	174.7758	176.1541	174.7552	175.9535	174.8209	176.2409	173.7091	174.3304	175.0011	174.6204	175.3683	174.6132	175.7597	174.6046	175.6659	174.6323	175.9170	**167**
**C5**	118.5912	119.9438	120.5513	120.6619	121.4796	120.6255	121.7575	120.5889	121.6419	120.7023	121.8945	118.7577	119.9871	120.5816	120.5974	121.4204	120.5694	121.5676	120.5405	121.4822	120.6294	121.7173	**116**
**C6**	134.7050	136.6708	137.7813	137.7564	139.2253	137.6992	139.8111	137.6410	139.4847	137.8168	140.1424	135.0285	137.0109	138.2571	138.0065	139.6769	137.9579	140.1333	137.9074	139.9591	138.0571	140.5087	**132**
**C7**	199.4123	204.3866	205.8037	206.9774	207.9980	206.8235	213.1394	206.6697	212.2541	207.1300	214.1117	204.0187	208.2806	209.4897	210.5322	211.3305	210.4028	216.0716	210.2738	214.9613	210.6620	216.6144	**196**
**C8**	65.0628	66.2181	66.8745	66.5493	67.4912	66.5398	67.7569	66.5294	67.7680	66.5644	68.6439	59.7053	61.5405	62.0853	62.4460	63.2954	62.3913	63.9952	62.3385	63.7609	62.5067	64.2505	**60**
**C9**	23.2952	23.5805	23.7317	23.8547	24.1127	23.8461	24.1179	23.8365	24.0691	23.8689	24.2944	20.0444	20.4947	20.5876	20.7006	20.9390	20.6951	21.1020	20.6896	21.0266	20.7123	21.4780	**15**
**C8`**	36.7170	37.4689	37.6835	37.7337	38.0702	37.7257	38.3145	37.7173	38.2603	37.7475	38.7194	32.1504	32.3460	32.6128	32.5305	32.7822	32.5259	32.9103	32.5202	32.9431	32.5401	32.9962	**31**
**H2**	8.6629	8.6020	8.6311	8.5654	8.5437	8.5681	8.6026	8.5708	8.5678	8.5633	8.5647	8.6781	8.5197	8.5677	8.4422	8.4548	8.4475	8.4418	8.4527	8.4583	8.4370	8.4509	**8.07**
**H3**	7.3215	7.4590	7.5493	7.5044	7.6225	7.5021	7.6392	7.4997	7.6299	7.5070	7.6527	7.3076	7.4456	7.5341	7.4931	7.6131	7.4905	7.6286	7.4879	7.6208	7.4959	7.6437	**7.31**
**H5**	7.1696	7.3613	7.4527	7.4481	7.5728	7.4430	7.5957	7.4379	7.5815	7.4533	7.6142	7.1650	7.3449	7.4351	7.4237	7.5495	7.4192	7.5594	7.4147	7.5489	7.4283	7.5805	**7.31**
**H6**	8.0974	8.2998	8.3897	8.4087	8.5283	8.4021	8.6053	8.3956	8.5653	8.4153	8.6195	7.8972	8.0707	8.1892	8.1631	8.3306	8.1581	8.3421	8.1530	8.3320	8.1679	8.3885	**8.07**
**H8**	4.3445	4.4318	4.4558	4.5292	4.5852	4.5223	4.6544	4.5157	4.6229	4.5361	4.6585	4.4978	4.6489	4.7036	4.7168	4.8297	4.7133	4.8452	4.7097	4.8315	4.7203	4.8758	**5.09**
**H(9A-9C)**	1.3191	1.3280	1.3197	1.3196	1.2780	1.3205	1.2937	1.3214	1.2832	1.3191	1.3053	1.3960	1.4276	1.4283	1.4433	1.4195	1.4425	1.4331	1.4418	1.4326	1.4442	1.4560	**1.6**
**H(8`A-8`C)**	2.4778	2.4208	2.3902	2.4035	2.3793	2.4041	2.3533	2.4048	2.3662	2.4033	2.3481	2.9110	2.7452	2.7096	2.6744	2.6284	2.6794	2.5438	2.6843	2.5610	2.6698	2.5128	**2.81**
**F4**	-120.2351	-121.0807	-123.8156	-120.9622	-125.2909	-120.9730	-122.9021	-120.9844	-123.4531	-120.9506	-122.8061	-120.1050	-121.5166	-124.1797	-121.7361	-125.7875	-121.7233	-124.0920	-121.7123	-124.3270	-121.7490	-123.7384	**-102.1**
Nuclei	B972																						
**C1**	134.4302	134.9580	135.9573	135.2517	137.1882	135.2339	136.9092	135.2172	137.2291	135.2642	136.9986	133.0960	134.5569	135.0556	135.2214	136.6788	135.1632	136.7562	135.1090	136.5422	135.2805	136.8545	**129**
**C2**	136.2385	136.1466	136.7094	135.9825	136.5017	135.9974	136.8781	136.0094	136.7762	135.9648	136.9004	135.6839	135.8309	136.3876	135.8469	136.4661	135.8466	136.9148	135.8443	136.8312	135.8431	136.9435	**132**
**C3**	118.6420	119.3619	119.9304	119.6913	120.3271	119.6734	120.6091	119.6533	120.5621	119.7042	120.6885	118.7138	119.3622	119.8746	119.6667	120.3028	119.6505	120.5119	119.6318	120.4478	119.6777	120.5982	**116**
**C4**	170.4080	171.2888	171.9012	171.8240	172.3935	171.7908	172.9800	171.7559	172.8201	171.8517	173.1417	170.6771	171.2152	171.8947	171.5417	172.2306	171.5278	172.5783	171.5099	172.5046	171.5489	172.7454	**167**
**C5**	116.5455	117.8870	118.4963	118.6438	119.4131	118.5990	119.6446	118.5521	119.5386	118.6819	119.8150	116.6811	117.8807	118.4867	118.5356	119.3203	118.4989	119.4656	118.4592	119.3807	118.5657	119.6278	**116**
**C6**	132.2984	134.1683	135.1909	135.2810	136.6319	135.2152	137.0709	135.1451	136.7199	135.3380	137.5017	132.7192	134.6132	135.8785	135.6223	137.1863	135.5695	137.5344	135.5120	137.4030	135.6656	137.9182	**132**
**C7**	197.1472	202.1820	203.6394	205.0747	205.9500	204.8855	211.4809	204.7016	210.7109	205.2515	212.1626	201.5167	205.7889	206.8296	208.0334	208.7686	207.8942	213.5796	207.7543	212.4081	208.1644	214.0847	**196**
**C8**	62.2129	63.1640	63.7653	63.5253	64.4519	63.5132	65.0374	63.4956	64.9409	63.5298	65.7131	56.9800	58.9172	59.3838	59.8622	60.6095	59.7928	61.4484	59.7250	61.1550	59.9290	61.7202	**60**
**C9**	22.2017	22.5014	22.6308	22.6974	22.9802	22.6894	22.9728	22.6772	22.8720	22.7010	23.1432	18.9615	19.3718	19.4861	19.6193	19.7902	19.6032	19.9459	19.5854	19.8717	19.6315	20.3049	**15**
**C8`**	35.6349	36.3454	36.5312	36.8429	37.0375	36.8098	37.2438	36.7749	37.2215	36.8693	37.6484	31.3495	31.4924	31.7924	31.7285	31.9630	31.7188	31.9659	31.7053	32.0216	31.7314	32.0500	**31**
**H2**	8.6537	8.5910	8.6014	8.5457	8.5058	8.5490	8.4992	8.5518	8.4749	8.5424	8.5039	8.6825	8.4992	8.5620	8.4234	8.4354	8.4301	8.3959	8.4360	8.4226	8.4161	8.4046	**8.07**
**H3**	7.3227	7.4597	7.5484	7.5068	7.6209	7.5045	7.6375	7.5020	7.6284	7.5088	7.6533	7.3120	7.4483	7.5346	7.4976	7.6140	7.4951	7.6296	7.4923	7.6214	7.4999	7.6455	**7.31**
**H5**	7.1739	7.3666	7.4552	7.4573	7.5754	7.4520	7.5903	7.4465	7.5774	7.4620	7.6138	7.1665	7.3420	7.4301	7.4225	7.5446	7.4180	7.5559	7.4132	7.5449	7.4267	7.5779	**7.31**
**H6**	8.1158	8.3161	8.3945	8.4358	8.5373	8.4282	8.5663	8.4204	8.5382	8.4429	8.6045	7.9070	8.0727	8.1924	8.1605	8.3194	8.1565	8.3186	8.1518	8.3151	8.1637	8.3642	**8.07**
**H8**	4.3399	4.4508	4.4730	4.5472	4.5919	4.5405	4.6226	4.5339	4.6079	4.5536	4.6488	4.4883	4.6298	4.6848	4.6954	4.8083	4.6924	4.8202	4.6889	4.8127	4.6977	4.8518	**5.09**
**H(9A-9C)**	1.2779	1.2737	1.2581	1.2603	1.2187	1.2618	1.2249	1.2628	1.2101	1.2586	1.2452	1.3585	1.3871	1.3915	1.4080	1.3784	1.4071	1.3926	1.4058	1.3948	1.4087	1.4173	**1.6**
**H(8`A-8`C)**	2.4444	2.3879	2.3637	2.3740	2.3543	2.3748	2.3536	2.3753	2.3559	2.3732	2.3366	2.8829	2.7146	2.6793	2.6431	2.5976	2.6486	2.5129	2.6536	2.5335	2.6373	2.4818	**2.81**
**F4**	-114.1394	-114.9916	-117.6165	-114.8823	-119.1303	-114.8982	-117.0933	-114.9144	-117.5459	-114.8661	-116.7899	-113.9897	-115.4937	-117.9379	-115.7417	-119.5861	-115.7233	-118.0262	-115.7090	-118.1925	-115.7622	-117.6994	**-102.1**
Nuclei	BHandH																						
**C1**	138.2231	138.5552	139.4722	138.6613	140.1957	138.6447	139.2014	138.6466	139.2910	138.6472	139.3414	137.3260	138.1227	139.0514	138.6686	139.4127	138.6143	139.9113	138.5817	139.6959	138.6934	140.0677	**129**
**C2**	143.5201	143.3955	144.0049	143.2268	143.8191	143.2277	144.3374	143.2468	144.2139	143.1959	144.3719	143.1561	143.1628	143.7749	143.0888	143.8329	143.0819	144.2258	143.0940	144.0867	143.0659	144.2500	**132**
**C3**	123.7766	124.4896	125.0912	124.7508	125.4195	124.7262	125.6101	124.7198	125.5593	124.7437	125.6833	123.8317	124.5005	125.1213	124.7551	125.4992	124.7302	125.6175	124.7238	125.5792	124.7487	125.7026	**116**
**C4**	174.6910	175.5022	176.1372	175.9474	176.5413	175.9096	177.2138	175.8903	177.0668	175.9534	177.3817	174.9086	175.5511	176.2159	175.8630	176.6071	175.8357	176.9768	175.8261	176.9107	175.8585	177.1498	**167**
**C5**	121.3254	122.7876	123.4824	123.5575	124.4251	123.5004	124.6212	123.4617	124.5293	123.5816	124.7930	121.4351	122.8143	123.5066	123.5206	124.2647	123.4688	124.5249	123.4348	124.4609	123.5397	124.7001	**116**
**C6**	139.9383	142.0591	143.3061	143.2147	144.9304	143.1340	145.6585	143.0715	145.4144	143.2611	146.1074	140.2023	142.4966	143.7026	143.6179	144.3620	143.5430	145.9734	143.4853	145.7743	143.6576	146.3435	**132**
**C7**	202.9300	207.7859	209.1882	210.2924	210.9441	210.1306	215.8168	209.9874	214.6983	210.4156	216.6335	207.3621	211.4788	213.0278	213.6769	214.4210	213.5328	219.1023	213.4077	218.0494	213.7841	219.7557	**196**
**C8**	59.9494	61.2143	61.9067	61.6436	62.6312	61.6098	63.0978	61.5942	62.9356	61.6453	63.8442	54.0631	55.4214	55.9382	56.1974	56.9415	56.1327	57.6583	56.0882	57.3239	56.2306	57.9608	**60**
**C9**	22.4684	22.7173	23.0362	22.9961	23.4920	22.9658	23.4131	22.9537	23.4079	22.9951	23.6098	19.0747	19.5603	19.6330	19.8271	20.5712	19.7976	20.2453	19.7870	20.1575	19.8256	20.6529	**15**
**C8`**	33.7500	34.5612	34.8118	34.8998	35.2711	34.8687	35.5037	34.8562	35.4510	34.8996	35.9918	28.6454	29.0946	29.4036	29.3316	30.0757	29.3102	29.7782	29.3056	29.7931	29.3204	29.9429	**31**
**H2**	8.9875	8.9200	8.9348	8.8802	8.8770	8.8834	8.9222	8.8858	8.9249	8.8781	8.9222	8.9840	8.8580	8.8706	8.7732	8.6920	8.7803	8.7507	8.7862	8.7811	8.7672	8.7533	**8.07**
**H3**	7.4553	7.5997	7.6892	7.6466	7.7674	7.6451	7.7842	7.6427	7.7763	7.6491	7.8000	7.4516	7.5922	7.6830	7.6387	7.5575	7.6372	7.7745	7.6347	7.7677	7.6412	7.7910	**7.31**
**H5**	7.3003	7.5022	7.5933	7.5917	7.7206	7.5873	7.7378	7.5822	7.7268	7.5967	7.7629	7.2950	7.4886	7.5794	7.5727	7.4915	7.5687	7.7122	7.5639	7.7021	7.5774	7.7361	**7.31**
**H6**	8.3317	8.5445	8.6361	8.6585	8.7941	8.6524	8.8645	8.6454	8.8495	8.6654	8.9071	8.1293	8.3337	8.4417	8.4287	8.3475	8.4246	8.6263	8.4194	8.6221	8.4334	8.6718	**8.07**
**H8**	4.1505	4.2528	4.2749	4.3463	4.3866	4.3407	4.4438	4.3343	4.4339	4.3528	4.4693	4.4049	4.5696	4.6414	4.6481	4.5669	4.6447	4.7936	4.6404	4.7922	4.6522	4.8291	**5.09**
**H(9A-9C)**	1.3947	1.4083	1.3944	1.4043	1.3835	1.4056	1.4087	1.4060	1.4066	1.4041	1.4308	1.4777	1.5048	1.4783	1.5114	1.4301	1.5120	1.4963	1.5117	1.4978	1.5117	1.5179	**1.6**
**H(8`A-8`C)**	2.5482	2.4741	2.4402	2.4443	2.4243	2.4467	2.3801	2.4483	2.3790	2.4430	2.3667	2.9170	2.7545	2.7130	2.6709	2.5897	2.6772	2.5211	2.6825	2.5633	2.6659	2.4973	**2.81**
**F4**	-105.2660	-105.8310	-107.9761	-105.6345	-109.0248	-105.6521	-106.8723	-105.6688	-107.2259	-105.6171	-106.6144	-105.1037	-106.0291	-108.1331	-106.1131	-106.4498	-106.1094	-107.7443	-106.1061	-107.9144	-106.1172	-107.4800	**-102.1**
Nuclei	BHandHLYP																						
**C1**	138.8640	139.2644	139.9330	139.4014	141.4115	139.3890	140.3239	139.3799	140.8073	139.4140	140.7808	137.7359	138.7863	139.5663	139.4872	141.0279	139.4436	140.6370	139.4057	140.4914	139.5319	140.6845	**129**
**C2**	142.1451	142.0033	142.5518	141.8479	142.3133	141.8593	142.8334	141.8728	142.6946	141.8367	142.8238	141.6846	141.6776	142.2130	141.6154	142.1662	141.6189	142.6674	141.6254	142.5678	141.6119	142.7010	**132**
**C3**	122.5311	123.1246	123.6134	123.3344	123.9644	123.3231	124.1502	123.3143	124.1344	123.3446	124.2197	122.6170	123.1643	123.6329	123.3811	123.9687	123.3691	124.1171	123.3594	124.0780	123.3922	124.1810	**116**
**C4**	174.4859	175.2386	175.8387	175.6759	176.2231	175.6490	176.9757	175.6248	176.7716	175.7009	177.0601	174.7783	175.2638	175.8588	175.5025	176.1211	175.4893	176.5698	175.4775	176.5010	175.5141	176.7410	**167**
**C5**	120.1712	121.4535	122.0189	122.1435	122.9398	122.1013	123.1049	122.0616	123.0479	122.1835	123.2751	120.2936	121.5123	122.0694	122.1337	122.8970	122.0972	123.0006	122.0628	122.9395	122.1680	123.1441	**116**
**C6**	138.1510	140.1493	141.3422	141.2896	142.5504	141.2223	143.2999	141.1564	142.9195	141.3529	143.5841	138.4674	140.5298	141.7475	141.5098	143.1000	141.4526	143.6578	141.3968	143.5207	141.5620	144.0715	**132**
**C7**	201.2543	206.5546	207.7752	209.3182	210.2498	209.1454	216.0604	208.9760	215.0849	209.4828	217.0651	206.1611	210.4578	211.6283	212.8433	213.4066	212.7018	218.6263	212.5625	217.3826	212.9773	219.2046	**196**
**C8**	60.7965	61.8811	62.5000	62.2233	63.0588	62.2069	63.2656	62.1924	63.2636	62.2383	64.1041	55.4804	57.1821	57.8021	58.1054	58.8956	58.0473	59.5516	57.9937	59.2868	58.1628	59.8130	**60**
**C9**	22.2333	22.3886	22.6124	22.6282	22.8233	22.6139	22.7047	22.6016	22.6825	22.6409	22.8547	18.9648	19.2708	19.4004	19.4144	19.6557	19.4060	19.6995	19.4005	19.6674	19.4225	20.0180	**15**
**C8`**	34.7377	35.5130	35.7819	35.8289	36.1801	35.8108	36.3989	35.7951	36.3597	35.8459	36.8091	30.5960	30.8142	31.0655	30.9812	31.2247	30.9680	31.3407	30.9569	31.3887	30.9927	31.4699	**31**
**H2**	8.8761	8.8039	8.8520	8.7659	8.7241	8.7690	8.7906	8.7718	8.7505	8.7629	8.7519	8.8981	8.7359	8.7665	8.6405	8.6466	8.6464	8.6344	8.6519	8.6571	8.6345	8.6458	**8.07**
**H3**	7.4433	7.5783	7.6654	7.6233	7.7361	7.6213	7.7566	7.6190	7.7465	7.6252	7.7698	7.4323	7.5668	7.6511	7.6133	7.7280	7.6111	7.7441	7.6087	7.7353	7.6153	7.7592	**7.31**
**H5**	7.2878	7.4785	7.5663	7.5650	7.6852	7.5602	7.7061	7.5552	7.6928	7.5695	7.7273	7.2798	7.4603	7.5446	7.5400	7.6603	7.5356	7.6720	7.5311	7.6611	7.5440	7.6938	**7.31**
**H6**	8.1765	8.3928	8.4992	8.5149	8.6356	8.5078	8.7226	8.5005	8.6822	8.5217	8.7376	8.0113	8.2014	8.3159	8.2915	8.4587	8.2866	8.4826	8.2813	8.4746	8.2959	8.5310	**8.07**
**H8**	4.0776	4.1885	4.2247	4.3024	4.3719	4.2945	4.4593	4.2868	4.4300	4.3099	4.4624	4.2587	4.4280	4.4860	4.5022	4.6169	4.4983	4.6464	4.4943	4.6344	4.5056	4.6772	**5.09**
**H(9A-9C)**	1.2993	1.3062	1.3063	1.3002	1.2417	1.3014	1.2609	1.3023	1.2448	1.2990	1.2689	1.3682	1.3960	1.3920	1.4076	1.3798	1.4070	1.4019	1.4061	1.4026	1.4081	1.4244	**1.6**
**H(8`A-8`C)**	2.4396	2.3870	2.3452	2.3723	2.3466	2.3731	2.3248	2.3737	2.3360	2.3717	2.3239	2.8786	2.7124	2.6679	2.6359	2.5874	2.6411	2.5108	2.6458	2.5345	2.6307	2.4845	**2.81**
**F4**	-108.7738	-109.8192	-112.0485	-109.8454	-113.5515	-109.8482	-111.3452	-109.8508	-111.8572	-109.8427	-111.3217	-108.4921	-110.0709	-112.3417	-110.4795	-113.9112	-110.4590	-112.3617	-110.4396	-112.5235	-110.5005	-112.0844	**-102.1**

[a] methyl protons shifts are averaged
[b] according to [9]

Table S9. Cont. II

Nuclei	R											S											Exp. [b]
	BLYP																						
	Gas	$CHCl_3$ (PCM)	$CHCl_3$ (SMD)	DMSO (PCM)	DMSO (SMD)	CH_3OH (PCM)	CH_3OH (SMD)	C_2H_5OH (PCM)	C_2H_5OH (SMD)	H_2O (PCM)	H_2O (SMD)	Gas	$CHCl_3$ (PCM)	$CHCl_3$ (SMD)	DMSO (PCM)	DMSO (SMD)	CH_3OH (PCM)	CH_3OH (SMD)	C_2H_5OH (PCM)	C_2H_5OH (SMD)	H_2O (PCM)	H_2O (SMD)	
**C1**	139.2740	140.0627	140.7302	140.3557	142.0482	140.6261	141.6136	140.4250	141.4809	140.7208	141.8598	138.3037	138.8878	139.5939	139.5904	141.4131	139.8236	141.0985	139.5956	141.3416	139.9982	141.2014	**129**
**C2**	137.3888	137.3557	137.9052	137.1077	137.7439	137.4006	137.9857	137.2334	138.0955	137.4576	138.0801	136.8040	136.8446	137.3898	136.8417	137.6112	137.1207	137.9926	136.9376	138.0835	137.2041	138.0703	**132**
**C3**	120.6544	121.3842	121.8707	121.6716	122.2855	121.9330	122.4590	121.7316	122.5866	122.0499	122.6336	119.6392	121.4514	121.9240	121.6569	122.3097	121.9238	122.3772	121.7284	122.4989	122.0306	122.5353	**116**
**C4**	177.6519	178.7185	179.3932	179.3068	180.0988	179.5462	180.5234	179.3230	180.6223	179.7057	180.7915	178.1086	178.8813	179.6485	179.1527	180.0400	179.4160	180.2300	179.2172	180.2861	179.5290	180.4844	**167**
**C5**	118.5470	119.8601	120.3998	120.5456	121.3222	120.7815	121.4404	120.5550	121.5434	120.9483	121.6902	118.6599	120.0133	120.5942	120.5540	121.3508	120.8003	121.3513	120.5845	121.4329	120.9468	121.5891	**116**
**C6**	133.7911	135.6052	136.7931	136.6678	138.3637	136.8753	138.6685	136.6243	138.6992	137.1000	139.1691	138.8182	136.2738	137.4058	137.2526	138.9036	137.4733	139.2393	137.2316	139.1572	137.6692	139.7543	**132**
**C7**	203.2767	208.6048	209.8305	211.6985	212.3595	211.7964	217.3300	211.4147	216.3806	212.2229	218.3121	205.2971	212.7975	214.3032	214.9277	216.0897	215.0775	220.0988	214.7652	219.4181	215.4121	220.5992	**196**
**C8**	69.1241	70.1253	70.7992	70.3573	71.5764	70.6395	71.9231	70.4601	71.8008	70.7169	72.6971	68.1488	65.4631	65.8331	66.4810	67.5528	66.6957	68.0924	66.4492	68.1157	66.9054	68.4506	**60**
**C9**	23.7215	24.0520	24.2062	24.1836	24.6500	24.4455	24.6108	24.2503	24.7226	24.5640	24.8492	16.4537	20.8061	20.7678	21.0333	21.2955	21.2977	21.4360	21.1003	21.5017	21.4093	21.9663	**15**
**C8`**	38.2242	38.9477	39.0730	39.4073	39.6721	39.6658	39.7283	39.4601	39.9360	39.7878	40.2492	32.8912	33.8280	34.1359	33.9651	34.2232	34.2348	34.1613	34.0423	34.3099	34.3359	34.3012	**31**
**H2**	8.3954	8.3311	8.3657	8.2850	8.2783	8.3345	8.4554	8.2924	8.3194	8.3386	8.4701	8.3743	8.3060	8.3480	8.2230	8.2163	8.2757	8.3816	8.2357	8.2114	8.2732	8.4083	**8.07**
**H3**	7.1679	7.2953	7.3831	7.3318	7.4491	7.3773	7.6306	7.3305	7.4581	7.3888	7.6578	7.0966	7.2855	7.3681	7.3259	7.4422	7.3712	7.6247	7.3239	7.4500	7.3832	7.6520	**7.31**
**H5**	7.0230	7.2052	7.2897	7.2883	7.4065	7.3310	7.5902	7.2811	7.4161	7.3480	7.6256	6.9899	7.1903	7.2755	7.2597	7.3808	7.3031	7.5580	7.2540	7.3806	7.3187	7.5918	**7.31**
**H6**	7.9452	8.1295	8.2114	8.2406	8.3476	8.2808	8.5506	8.2286	8.3909	8.3029	8.6025	8.9754	7.8764	7.9806	7.9535	8.0977	7.9966	8.2837	7.9470	8.0952	8.0128	8.3454	**8.07**
**H8**	4.5730	4.6582	4.6658	4.7406	4.7734	4.7808	4.9699	4.7284	4.7971	4.8027	5.0172	4.4807	4.8131	4.8614	4.8724	4.9776	4.9165	5.1608	4.8680	4.9755	4.9306	5.2060	**5.09**
**H(9A-9C)**	1.3344	1.3224	1.3240	1.2985	1.2925	1.3477	1.4693	1.3049	1.3035	1.3524	1.4944	1.2856	1.4612	1.4487	1.4808	1.4580	1.5270	1.6506	1.4807	1.4754	1.5371	1.6944	**1.6**
**H(8`A-8`C)**	2.5702	2.5055	2.4761	2.4845	2.4652	2.5330	2.6299	2.4890	2.4571	2.5387	2.6207	2.5654	2.8839	2.8478	2.7998	2.7482	2.8522	2.8332	2.8121	2.6782	2.8501	2.8119	**2.81**
**F4**	-142.4546	-143.7783	-147.0938	-143.7711	-149.1057	-143.5779	-145.7067	-143.7967	-146.8744	-143.6896	-145.8209	-141.1875	-144.2745	-147.5672	-144.6769	-149.7768	-144.4505	-146.7327	-144.6396	-148.1049	-144.6295	-146.7999	**-102.1**
Nuclei	BMK																						
**C1**	154.5489	154.7956	155.8147	155.0346	156.7869	155.0048	155.7582	155.1850	155.9437	155.2232	155.7278	153.3708	154.7062	155.5737	155.3493	157.1804	155.2822	157.0114	155.4300	156.7717	155.5792	157.0888	**129**
**C2**	155.5489	155.3349	156.1444	155.0820	155.8798	155.0811	156.4462	155.2910	156.2529	155.2438	156.4023	154.7572	154.6732	155.3927	154.5734	155.4851	154.5591	155.9928	154.7574	155.7913	154.7494	155.9653	**132**
**C3**	135.9186	136.5742	137.2866	136.7717	137.6790	136.7457	137.8453	136.9307	137.6978	136.9577	137.8467	136.0540	136.5812	137.2516	136.7609	137.6638	136.7339	137.8432	136.9186	137.6908	136.9479	137.8865	**116**
**C4**	189.4005	190.1835	191.0076	190.6219	191.5290	190.5810	192.1342	190.7513	191.8731	190.8217	192.2418	189.7985	190.2293	191.0560	190.4438	191.4104	190.4180	191.7002	190.6026	191.5626	190.6291	191.8308	**167**
**C5**	133.4295	134.7613	135.4788	135.4278	136.5512	135.3675	136.6886	135.5196	136.4925	135.6472	136.8148	133.4535	134.7683	135.5390	135.3777	136.4835	135.3275	136.5822	135.4877	136.3606	135.5868	136.7328	**116**
**C6**	151.4094	153.6757	155.2637	155.0157	156.8896	154.9195	157.7543	155.0316	157.2746	155.2662	158.1427	152.1159	153.9639	155.4209	155.0094	157.0550	154.9327	157.4837	155.0660	157.2182	155.2429	157.8187	**132**
**C7**	216.9478	222.5474	224.1482	225.4916	226.2743	225.2972	232.0453	225.3132	230.7791	225.8361	232.8163	221.5389	226.5692	228.0915	229.2000	230.0942	229.0219	235.6763	229.0559	234.3215	229.5316	236.4133	**196**
**C8**	70.5725	71.6400	72.4428	72.1543	73.3210	72.1213	73.7576	72.2984	73.5299	72.3466	74.4771	64.4902	66.3795	67.0821	67.2150	68.2924	67.1424	69.1388	67.2827	68.7153	67.4478	69.3740	**60**
**C9**	25.4816	25.8566	26.3477	26.2252	26.6325	26.1938	26.7756	26.3697	26.5780	26.4143	26.8310	22.1260	22.5585	22.7087	22.6468	23.0607	22.6251	23.2222	22.8150	23.0572	22.8297	23.5137	**15**
**C8`**	38.7886	39.4905	39.7965	39.7916	40.2856	39.7653	40.4650	39.9500	40.3114	39.9781	40.8997	33.9443	34.1818	34.5674	34.3474	34.7515	34.3245	34.7335	34.5118	34.8069	34.5294	34.8356	**31**
**H2**	9.0522	9.0233	9.0737	8.9738	8.9666	8.9734	9.0551	8.9730	9.0302	8.9645	9.0669	9.0430	8.8827	8.9188	8.8117	8.8174	8.8133	8.8099	8.8146	8.8396	8.8005	8.8138	**8.07**
**H3**	7.6503	7.8088	7.9094	7.8680	7.9716	7.8622	8.0008	7.8561	7.9908	7.8636	8.0111	7.6582	7.8152	7.8999	7.8689	7.9851	7.8629	7.9933	7.8568	7.9965	7.8650	8.0096	**7.31**
**H5**	7.5098	7.7043	7.7841	7.7896	7.9181	7.7809	7.9299	7.7723	7.9192	7.7883	7.9561	7.4861	7.6973	7.7852	7.7793	7.9021	7.7717	7.9135	7.7640	7.8990	7.7768	7.9393	**7.31**
**H6**	8.4072	8.6270	8.7828	8.7961	8.9052	8.7837	9.0195	8.7706	8.9763	8.7979	9.0628	8.3050	8.4960	8.6092	8.6017	8.7715	8.5925	8.7332	8.5831	8.7698	8.6007	8.7910	**8.07**
**H8**	4.2317	4.3488	4.4104	4.4856	4.5292	4.4731	4.5903	4.4604	4.5756	4.4876	4.6005	4.4400	4.6482	4.6995	4.7284	4.8382	4.7206	4.8188	4.7127	4.8373	4.7262	4.8615	**5.09**
**H(9A-9C)**	1.3048	1.3335	1.3264	1.3372	1.2998	1.3347	1.3417	1.3320	1.3281	1.3300	1.3703	1.3776	1.3895	1.3774	1.4034	1.3701	1.3999	1.3895	1.3963	1.3784	1.3971	1.4125	**1.6**
**H(8`A-8`C)**	2.4334	2.3750	2.3238	2.3567	2.3235	2.3539	2.2956	2.3513	2.3080	2.3498	2.2771	2.8035	2.6688	2.6299	2.6096	2.5602	2.6105	2.4481	2.6113	2.4916	2.5990	2.4288	**2.81**
**F4**	-117.8305	-118.8370	-121.4737	-118.7660	-122.8911	-118.7769	-120.6770	-118.7873	-121.0954	-118.7564	-120.3901	-117.3179	-118.7955	-121.4641	-119.0073	-122.9757	-118.9968	-121.4127	-118.9864	-121.5751	-119.0189	-121.0973	**-102.1**
Nuclei	BP86																						
**C1**	135.9939	136.6320	137.6362	136.9544	138.7298	136.9390	138.2948	136.9186	138.3141	136.9642	138.7051	134.4352	135.9816	136.4890	136.5815	138.0085	136.5423	138.0378	136.5022	137.9223	136.6187	138.2985	**129**
**C2**	136.0461	136.1412	136.6915	135.9389	136.4876	135.9629	136.9072	135.9812	136.7985	135.9104	137.0603	135.2799	135.6510	136.1578	135.6672	136.3174	135.6758	136.8585	135.6802	136.7480	135.6552	137.0159	**132**
**C3**	119.5556	120.4857	121.0080	120.7308	121.3490	120.7247	121.6928	120.7132	121.6404	120.7314	121.9360	119.6977	120.5178	120.9979	120.7317	121.3594	120.7284	121.6195	120.7195	121.5439	120.7299	121.8637	**116**
**C4**	174.3602	175.5049	176.1304	175.8536	176.5630	175.8334	177.1710	175.8083	177.0813	175.8678	177.4848	174.7072	175.5024	176.1986	175.7445	176.5291	175.7420	176.8841	175.7330	176.7851	175.7408	177.2044	**167**
**C5**	117.4531	118.9871	119.5529	119.6506	120.4030	119.6163	120.7206	119.5768	120.6236	119.6782	121.0496	117.6959	119.0396	119.6143	119.6262	120.4183	119.6030	120.5906	119.5742	120.4936	119.6424	120.9111	**116**
**C6**	132.4823	134.5222	135.5397	135.4991	136.9975	135.4473	137.5315	135.3905	137.2521	135.5440	138.0880	132.8422	134.9807	136.2191	135.9532	137.6251	135.9065	138.0996	135.8536	137.8931	135.9910	138.6398	**132**
**C7**	200.3568	205.1844	206.6915	207.6362	208.7456	207.4854	213.3518	207.3301	212.6663	207.7757	214.3906	205.2587	209.2590	210.3906	211.2385	212.1476	211.1283	216.2414	211.0136	215.3050	211.3390	216.8705	**196**
**C8**	67.0914	68.3350	69.0224	68.6336	69.6697	68.6283	70.1232	68.6177	69.8169	68.6343	71.1003	61.2199	63.3467	63.8475	64.2565	65.1036	64.1999	65.8529	64.1410	65.6179	64.3090	66.2851	**60**
**C9**	23.4788	23.9387	24.0159	24.1770	24.4194	24.1716	24.5594	24.1605	24.4086	24.1770	24.8922	20.0447	20.8111	20.8964	21.0566	21.2467	21.0482	21.5506	21.0352	21.4325	21.0604	22.1852	**15**
**C8`**	37.5056	38.3780	38.4809	38.5899	38.8300	38.5853	39.0439	38.5763	39.0514	38.5903	39.6736	32.7385	32.9732	33.1909	33.0751	33.2543	33.0771	33.3634	33.0731	33.3752	33.0673	33.5888	**31**
**H2**	8.4690	8.4251	8.4168	8.3721	8.3303	8.3765	8.3610	8.3805	8.3653	8.3675	8.3657	8.5023	8.3508	8.3888	8.2771	8.2780	8.2836	8.2597	8.2894	8.2717	8.2701	8.2813	**8.07**
**H3**	7.1833	7.3254	7.4023	7.3644	7.4695	7.3630	7.4872	7.3613	7.4789	7.3653	7.5157	7.1759	7.3137	7.3896	7.3551	7.4646	7.3535	7.4797	7.3516	7.4705	7.3561	7.5088	**7.31**
**H5**	7.0316	7.2248	7.3017	7.3045	7.4170	7.3004	7.4360	7.2960	7.4267	7.3081	7.4730	7.0289	7.2047	7.2838	7.2819	7.3979	7.2785	7.4064	7.2747	7.3955	7.2847	7.4416	**7.31**
**H6**	8.0317	8.2270	8.2775	8.3141	8.4038	8.3094	8.4531	8.3044	8.4474	8.3183	8.4976	7.7817	7.9419	8.0474	8.0258	8.1760	8.0224	8.1879	8.0185	8.1750	8.0284	8.2496	**8.07**
**H8**	4.6085	4.6845	4.6748	4.7603	4.7803	4.7553	4.8233	4.7502	4.8256	4.7646	4.8587	4.7327	4.8659	4.9018	4.9243	5.0225	4.9224	5.0356	4.9199	5.0205	4.9256	5.0853	**5.09**
**H(9A-9C)**	1.3656	1.3684	1.3441	1.3548	1.3143	1.3570	1.3284	1.3589	1.3132	1.3524	1.3589	1.4453	1.4875	1.4805	1.5086	1.4790	1.5084	1.4981	1.5079	1.4947	1.5083	1.5410	**1.6**
**H(8`A-8`C)**	2.5546	2.4795	2.4461	2.4610	2.4324	2.4627	2.4144	2.4642	2.4111	2.4590	2.4105	3.0093	2.8351	2.7840	2.7540	2.6945	2.7609	2.6012	2.7671	2.6178	2.7468	2.5828	**2.81**
**F4**	-133.5336	-134.1591	-137.3936	-133.9827	-139.0671	-134.0019	-136.4323	-134.0203	-136.7655	-133.9638	-136.0779	-133.3260	-134.6989	-137.7252	-134.9784	-139.6686	-134.9729	-137.5976	-134.9689	-137.9175	-134.9853	-137.1579	**-102.1**
Nuclei	CAM-B3LYP																						
**C1**	140.5940	141.0501	141.9502	141.2482	143.3376	141.2328	142.6219	141.2195	142.7966	141.2664	142.8916	139.3811	140.7764	141.3414	141.3955	142.9372	141.3447	142.8551	141.3003	142.5657	141.4530	142.9212	**129**
**C2**	142.4322	142.3676	142.8920	142.2031	142.7302	142.2185	143.2393	142.2342	143.1183	142.1902	143.2313	141.9632	142.0722	142.5741	142.0137	142.6143	142.0196	143.1220	142.0264	143.0134	142.0107	143.1501	**132**
**C3**	123.8770	124.5297	125.0152	124.7351	125.3944	124.7281	125.5969	124.7212	125.5656	124.7437	125.6728	123.9590	124.5672	125.0168	124.7695	125.4058	124.7619	125.5862	124.7548	125.5220	124.7791	125.6510	**116**
**C4**	177.6432	178.4616	179.0236	178.8866	179.5004	178.8652	180.0958	178.8438	179.9671	178.9091	180.2452	177.9270	178.4619	179.0815	178.7166	179.4216	178.7091	179.7910	178.7009	179.7287	178.7245	179.9540	**167**
**C5**	121.6531	122.9882	123.5423	123.6761	124.5002	123.6378	124.6833	123.5998	124.6086	123.7146	124.8524	121.7948	123.0474	123.5984	123.6473	124.4684	123.6163	124.5780	123.5853	124.4990	123.6786	124.7226	**116**
**C6**	138.6325	140.6802	141.7768	141.7799	143.1835	141.7181	143.7450	141.6562	143.4517	141.8399	144.1171	138.9686	141.0487	142.3055	142.0534	143.7538	142.0017	144.1534	141.9484	144.0548	142.1023	144.5739	**132**
**C7**	205.1841	210.4669	211.8770	213.2659	214.3729	213.0946	219.8334	212.9249	218.8841	213.4315	220.8758	210.1044	214.5528	215.7343	216.9360	217.6675	216.7972	222.7736	216.6596	221.5003	217.0720	223.3268	**196**
**C8**	63.5728	64.7701	65.4574	65.1172	66.1087	65.1025	66.5638	65.0883	66.3449	65.1333	67.2834	57.9234	59.8230	60.3104	60.7065	61.5242	60.6433	62.2922	60.5845	61.9726	60.7729	62.5903	**60**
**C9**	22.4381	22.6575	22.8386	22.9008	23.1565	22.8881	23.1031	22.8752	23.0434	22.9144	23.2395	19.2771	19.6649	19.7105	19.7757	20.0125	19.7699	20.0783	19.7654	20.0320	19.7840	20.4496	**15**
**C8`**	35.5076	36.3386	36.5579	36.6501	37.0360	36.6375	37.2208	36.6255	37.1657	36.6643	37.6889	31.0486	31.2767	31.5537	31.4840	31.7595	31.4764	31.8336	31.4681	31.8936	31.4915	31.9753	**31**
**H2**	8.7689	8.7049	8.7244	8.6617	8.6168	8.6654	8.6438	8.6690	8.6389	8.6580	8.6285	8.7980	8.6167	8.6695	8.5340	8.5473	8.5409	8.5118	8.5472	8.5462	8.5267	8.5219	**8.07**
**H3**	7.4063	7.5412	7.6281	7.5846	7.6999	7.5826	7.7160	7.5807	7.7091	7.5865	7.7301	7.3996	7.5311	7.6171	7.5747	7.6929	7.5726	7.7060	7.5705	7.6986	7.5766	7.7206	**7.31**
**H5**	7.2620	7.4507	7.5382	7.5345	7.6567	7.5299	7.6715	7.5253	7.6620	7.5389	7.6941	7.2579	7.4349	7.5221	7.5119	7.6365	7.5077	7.6445	7.5036	7.6350	7.5158	7.6658	**7.31**
**H6**	8.1470	8.3590	8.4476	8.4737	8.5894	8.4673	8.6366	8.4608	8.6223	8.4796	8.6702	7.9705	8.1468	8.2705	8.2373	8.4092	8.2331	8.4121	8.2286	8.4132	8.2409	8.4600	**8.07**
**H8**	4.2201	4.3249	4.3484	4.4341	4.4931	4.4267	4.4588	4.4195	4.5400	4.4412	4.5650	4.3966	4.5587	4.6191	4.6315	4.7523	4.6282	4.7687	4.6248	4.7611	4.6343	4.8001	**5.09**
**H(9A-9C)**	1.3297	1.3390	1.3314	1.3291	1.2821	1.3304	1.2971	1.3316	1.2847	1.3277	1.3077	1.4016	1.4258	1.4289	1.4408	1.4198	1.4402	1.4318	1.4396	1.4375	1.4411	1.4563	**1.6**
**H(8`A-8`C)**	2.5132	2.4524	2.4238	2.4345	2.4170	2.4350	2.4044	2.4357	2.4044	2.4340	2.3914	2.9425	2.7653	2.7314	2.6913	2.6489	2.6974	2.5595	2.7032	2.5861	2.6851	2.5306	**2.81**
**F4**	-115.5485	-116.8719	-119.4187	-116.9933	-121.0302	-116.9892	-119.0882	-116.9849	-119.3688	-116.9983	-118.9035	-115.2826	-117.1929	-119.6136	-117.6345	-121.3475	-117.6057	-119.9108	-117.5789	-120.0081	-117.6651	-119.6376	**-102.1**

[a] methyl protons shifts are averaged
[b] according to [9]

Table S9. Cont. III

Nuclei	R											S											Exp. [b]
	HF																						
	Gas	$CHCl_3$ (PCM)	$CHCl_3$ (SMD)	DMSO (PCM)	DMSO (SMD)	CH_3OH (PCM)	CH_3OH (SMD)	C_2H_5OH (PCM)	C_2H_5OH (SMD)	H_2O (PCM)	H_2O (SMD)	Gas	$CHCl_3$ (PCM)	$CHCl_3$ (SMD)	DMSO (PCM)	DMSO (SMD)	CH_3OH (PCM)	CH_3OH (SMD)	C_2H_5OH (PCM)	C_2H_5OH (SMD)	H_2O (PCM)	H_2O (SMD)	
**C1**	136.0859	136.4286	136.9983	136.5642	138.6394	136.5601	136.8559	136.5607	137.5918	136.5707	137.8340	135.1106	136.0099	136.9165	136.8715	138.6196	136.8298	138.1526	136.7906	138.0357	136.9136	138.1693	**129**
**C2**	144.2959	144.1109	144.6184	143.9366	144.2969	143.9493	145.0625	143.9624	144.8336	143.9247	144.9263	144.1862	143.9606	144.4423	143.6963	144.1843	143.7105	144.6238	143.7251	144.5752	143.6812	144.6859	**132**
**C3**	120.5425	120.9815	121.3393	121.0997	121.6987	121.0951	121.5914	121.0919	121.6723	121.1042	121.7618	120.5755	121.0153	121.3955	121.1911	121.7425	121.1823	121.7739	121.1741	121.7432	121.1992	121.8101	**116**
**C4**	171.0685	171.6891	172.2443	172.0947	172.4686	172.0696	173.4111	172.0452	173.0954	172.1179	173.3624	171.3313	171.6695	172.1524	171.7723	172.2704	171.7643	172.7640	171.7564	172.6801	171.7787	172.9382	**167**
**C5**	117.7778	118.9699	119.4399	119.5840	120.3789	119.5486	120.2895	119.5143	120.3140	119.6174	120.5553	117.8113	119.0523	119.5559	119.6851	120.4237	119.6494	120.4603	119.6142	120.3849	119.7186	120.5859	**116**
**C6**	139.8710	141.8434	143.0414	142.9329	144.0689	142.8614	145.3610	142.7878	144.7144	142.9974	145.2266	140.2153	142.1535	143.2403	142.9363	144.2865	142.8868	144.8278	142.8360	144.6896	142.9813	145.2440	**132**
**C7**	195.4655	201.2748	202.3678	204.5817	204.9342	204.3815	211.4166	204.1898	210.2401	204.7750	212.8652	200.3146	204.7891	205.8595	207.3684	207.7885	207.2162	214.0876	207.0655	212.5722	207.5129	214.7501	**196**
**C8**	54.6914	55.6162	56.0452	55.7978	56.6132	55.7884	56.8235	55.7770	56.8761	55.8047	57.6680	49.9453	51.5670	52.1910	52.5059	53.2429	52.4542	53.9705	52.4041	53.6974	52.5554	54.1978	**60**
**C9**	20.7069	20.7584	20.8662	20.7421	21.0481	20.7452	20.9072	20.7468	20.8745	20.7388	20.9507	17.7976	17.8056	17.9115	17.8577	18.0665	17.8560	17.9858	17.8550	17.9557	17.8592	18.1883	**15**
**C8`**	32.6429	33.3486	33.5660	33.7469	34.0103	33.7215	34.1987	33.6970	34.1551	33.7689	34.5312	29.0930	29.3330	29.5248	29.4519	29.7044	29.4395	29.7559	29.4273	29.7928	29.4622	29.8640	**31**
**H2**	9.0358	8.9576	9.0240	8.9122	8.8795	8.9150	8.9903	8.9172	8.9268	8.9093	8.8935	9.0687	8.8942	8.9168	8.7657	8.7690	8.7719	8.7430	8.7777	8.7685	8.7596	8.7567	**8.07**
**H3**	7.4286	7.5694	7.6559	7.6163	7.7372	7.6140	7.7469	7.6115	7.7419	7.6185	7.7658	7.4270	7.5652	7.6521	7.6122	7.7319	7.6098	7.7417	7.6072	7.7343	7.6145	7.7576	**7.31**
**H5**	7.2620	7.4585	7.5485	7.5471	7.6720	7.5420	7.6831	7.5367	7.6720	7.5520	7.7063	7.2588	7.4515	7.5381	7.5363	7.6607	7.5316	7.6678	7.5266	7.6572	7.5408	7.6908	**7.31**
**H6**	8.1772	8.4177	8.5445	8.5717	8.7053	8.5623	8.8158	8.5526	8.7578	8.5806	8.8070	8.0620	8.2816	8.3955	8.3692	8.5389	8.3639	8.5572	8.3581	8.5485	8.3743	8.6055	**8.07**
**H8**	3.6262	3.7734	3.8337	3.9160	4.0002	3.9073	4.0866	3.8987	4.0448	3.9245	4.0816	3.7998	3.9985	4.0614	4.0795	4.2064	4.0749	4.2416	4.0700	4.2283	4.0838	4.2685	**5.09**
**H(9A-9C)**	1.2094	1.2101	1.2114	1.1856	1.1335	1.1867	1.1817	1.1873	1.1548	1.1845	1.1607	1.2797	1.2815	1.2708	1.2849	1.2453	1.2842	1.2618	1.2832	1.2631	1.2857	1.2803	**1.6**
**H(8`A-8`C)**	2.2583	2.2315	2.1858	2.2178	2.1970	2.2185	2.1768	2.2190	2.1940	2.2172	2.1932	2.6962	2.5446	2.4939	2.4664	2.4243	2.4706	2.3592	2.4746	2.3767	2.4625	2.3361	**2.81**
**F4**	-92.2012	-93.1244	-94.8492	-93.1160	-96.2760	-93.1234	-93.9449	-93.1302	-94.6053	-93.1088	-94.2664	-91.8275	-93.2856	-95.1728	-93.8334	-96.7123	-93.8098	-95.3711	-93.7864	-95.5009	-93.8563	-95.1316	**-102.1**
Nuclei	**LC-BLYP**																						
**C1**	143.9454	144.3001	145.4097	144.5079	146.3392	144.4682	145.1219	144.4578	146.1915	144.5303	146.2836	142.9429	144.2114	144.8868	144.9354	146.3990	144.8458	146.3781	144.7913	145.9849	145.0111	146.5188	**129**
**C2**	148.5022	148.3123	148.9120	148.1799	148.7307	148.1681	149.3075	148.1834	149.1118	148.1736	149.2571	148.2367	148.1595	148.7302	148.0898	148.6521	148.0728	149.1473	148.0835	149.0482	148.0884	149.1787	**132**
**C3**	128.3981	128.9432	129.5272	129.1700	129.8249	129.1365	129.9435	129.1302	129.9864	129.1845	130.1049	128.4577	128.9850	129.5383	129.2181	129.8639	129.1821	130.0193	129.1738	129.9481	129.2354	130.0806	**116**
**C4**	181.8964	182.5361	183.1128	182.9647	183.4846	182.9173	184.1724	182.8968	183.9350	182.9925	184.2385	182.1104	182.5115	183.1648	182.7723	183.4027	182.7394	183.7928	182.7322	183.7403	182.7849	183.9455	**167**
**C5**	126.0329	127.3491	128.0096	128.0921	128.9347	128.0253	129.0515	127.9857	129.0367	128.1384	129.2952	126.1441	127.4212	128.0695	128.1002	128.9526	128.0385	129.0416	128.0038	128.9555	128.1414	129.1882	**116**
**C6**	144.4457	146.5586	147.7101	147.7244	149.3132	147.6325	150.0897	147.5676	149.3684	147.7936	150.0269	144.7647	146.8890	148.2216	147.9599	149.7654	147.8834	150.1624	147.8313	150.0717	148.0120	150.5715	**132**
**C7**	210.7961	216.2518	217.8303	219.2261	219.8730	219.0191	225.3038	218.8395	225.0653	219.4065	227.1999	215.4470	220.1457	221.5590	222.7455	223.3196	222.5572	228.8329	222.3988	227.4675	222.9103	229.4875	**196**
**C8**	61.3984	62.6319	63.4214	63.1097	64.1261	63.0645	64.5480	63.0462	64.4091	63.1347	65.2976	55.6431	57.3143	57.8214	58.2576	59.0058	58.1666	59.8648	58.1066	59.4794	58.3300	60.2244	**60**
**C9**	21.5377	21.6825	21.9377	21.8979	22.3180	21.8578	22.1872	21.8462	22.0012	21.9197	22.1735	18.6324	18.8728	18.9378	19.0093	19.2711	18.9777	19.3164	18.9740	19.2407	19.0228	19.6888	**15**
**C8`**	33.6021	34.3992	34.7145	34.8359	35.1835	34.7918	35.4065	34.7739	35.3393	34.8597	35.8933	29.0895	29.3897	29.7641	29.6827	29.9584	29.6509	30.0425	29.6443	30.1159	29.6934	30.2365	**31**
**H2**	8.9711	8.8976	8.9038	8.8550	8.8357	8.8575	8.9003	8.8613	8.8099	8.8515	8.8027	8.9912	8.8085	8.8563	8.7154	8.7346	8.7227	8.6856	8.7307	8.7297	8.7068	8.6896	**8.07**
**H3**	7.5427	7.6754	7.7638	7.7201	7.8369	7.7170	7.8517	7.7154	7.8451	7.7221	7.8687	7.5437	7.6708	7.7599	7.7138	7.8323	7.7108	7.8425	7.7093	7.8366	7.7157	7.8586	**7.31**
**H5**	7.4058	7.5954	7.6843	7.6812	7.8049	7.6755	7.8189	7.6712	7.8049	7.6857	7.8395	7.4060	7.5871	7.6767	7.6670	7.7928	7.6617	7.7982	7.6579	7.7897	7.6710	7.8216	**7.31**
**H6**	8.3027	8.5213	8.6081	8.6422	8.7669	8.6342	8.8393	8.6277	8.7770	8.6487	8.8307	8.1441	8.3320	8.4605	8.4236	8.6087	8.4188	8.6040	8.4150	8.6111	8.4268	8.6501	**8.07**
**H8**	4.0994	4.2241	4.2473	4.3375	4.3854	4.3282	4.4499	4.3208	4.4440	4.3453	4.4811	4.3161	4.4914	4.5661	4.5712	4.6984	4.5663	4.7098	4.5628	4.7083	4.5747	4.7424	**5.09**
**H(9A-9C)**	1.3477	1.3582	1.3449	1.3500	1.3227	1.3501	1.3530	1.3517	1.2974	1.3487	1.3187	1.4131	1.4278	1.4251	1.4407	1.4202	1.4395	1.4310	1.4398	1.4374	1.4406	1.4542	**1.6**
**H(8`A-8`C)**	2.5010	2.4391	2.4146	2.4167	2.4026	2.4164	2.3702	2.4179	2.3908	2.4162	2.3818	2.8887	2.7145	2.6860	2.6365	2.5978	2.6411	2.5031	2.6470	2.5362	2.6309	2.4788	**2.81**
**F4**	-102.5915	-104.1036	-106.3783	-104.3438	-107.8530	-104.3346	-105.8842	-104.3254	-106.4778	-104.3524	-106.0793	-102.3680	-104.3796	-106.4884	-104.9374	-108.1733	-104.9008	-106.9030	-104.8663	-106.9326	-104.9747	-106.7271	**-102.1**
Nuclei	**M06**																						
**C1**	135.4072	135.7741	137.0115	135.8816	138.0210	135.8649	136.8091	135.8563	137.3380	135.9123	137.0556	134.1674	135.5202	136.1558	136.2300	137.8135	136.1538	137.9154	136.0917	137.5984	136.3238	137.9805	**129**
**C2**	140.6153	140.3648	140.9748	140.1830	140.7472	140.1863	141.1872	140.1968	141.0177	140.1945	141.1811	139.8441	139.8784	140.4612	139.8960	140.5961	139.8814	141.2124	139.8755	141.0600	139.9255	141.2192	**132**
**C3**	121.6176	122.2938	122.9735	122.5729	123.4101	122.5477	123.6473	122.5304	123.5932	122.6111	123.7286	121.6738	122.2553	122.8671	122.5213	123.3123	122.4950	123.5158	122.4767	123.4472	122.5609	123.5784	**116**
**C4**	173.1165	173.9963	174.7342	174.4927	175.3210	174.4521	176.0927	174.4196	175.9087	174.5457	176.2448	173.8447	174.2720	175.0646	174.5038	175.3524	174.4860	175.6260	174.4744	175.6126	174.5338	175.7886	**167**
**C5**	119.7718	121.0559	121.7752	121.7509	122.7315	121.6989	122.7996	121.6545	122.7382	121.8148	122.9828	119.7041	120.9774	121.6509	121.6232	122.5692	121.5755	122.6711	121.5353	122.6020	121.6826	122.8037	**116**
**C6**	136.8085	138.7038	139.7562	139.8590	141.2826	139.7763	142.3109	139.7016	141.8293	139.9522	142.5930	137.1469	139.1678	140.5409	140.2006	141.9881	140.1354	142.4239	140.0763	142.2946	140.2746	142.8394	**132**
**C7**	201.8270	206.8005	208.5395	209.3151	210.6085	209.1531	215.3851	208.9988	214.6233	209.4836	216.2344	206.3715	210.6515	211.8254	212.8411	213.6353	212.6921	218.3336	212.5525	217.1976	212.9995	218.7520	**196**
**C8**	60.7304	61.8816	62.6495	62.3326	63.4023	62.2980	63.5929	62.2707	63.6589	62.3799	64.4145	54.1844	55.7499	56.3619	56.6790	57.5433	56.6009	58.4113	56.5332	58.0797	56.7709	58.7141	**60**
**C9**	22.1450	22.4238	22.6903	22.7400	23.1676	22.7095	23.3573	22.6872	23.2995	22.7835	23.4614	19.4572	19.6907	19.8485	19.8638	20.2293	19.8402	20.3336	19.8248	20.2582	19.9018	20.8431	**15**
**C8`**	36.9348	37.6867	37.9199	37.9636	38.3405	37.9372	38.4473	37.9185	38.4095	38.0033	38.9535	31.7679	31.9520	32.3660	32.1758	32.5544	32.1571	32.7194	32.1443	32.7693	32.2063	32.7972	**31**
**H2**	8.7387	8.6519	8.6561	8.6165	8.5956	8.6186	8.6674	8.6211	8.6218	8.6152	8.6525	8.7596	8.5499	8.5974	8.4553	8.4747	8.4624	8.4395	8.4698	8.4663	8.4487	8.4486	**8.07**
**H3**	7.3369	7.4633	7.5604	7.5046	7.6321	7.5024	7.6528	7.5006	7.6377	7.5073	7.6581	7.2874	7.4302	7.5150	7.4793	7.6060	7.4760	7.6356	7.4734	7.6197	7.4830	7.6504	**7.31**
**H5**	7.1579	7.3516	7.4440	7.4353	7.5710	7.4303	7.5755	7.4258	7.5657	7.4405	7.6026	7.1555	7.3358	7.4320	7.4048	7.5377	7.4011	7.5349	7.3979	7.5315	7.4086	7.5553	**7.31**
**H6**	8.2198	8.4571	8.5350	8.5706	8.6748	8.5635	8.7980	8.5570	8.7357	8.5779	8.8111	7.9934	8.1490	8.2703	8.2290	8.4027	8.2251	8.4051	8.2215	8.4052	8.2330	8.4572	**8.07**
**H8**	4.3442	4.4692	4.4989	4.5607	4.6243	4.5542	4.7164	4.5485	4.6836	4.5675	4.7345	4.4477	4.6028	4.6519	4.6647	4.7811	4.6617	4.7907	4.6590	4.7893	4.6679	4.8239	**5.09**
**H(9A-9C)**	1.3018	1.3045	1.2808	1.2978	1.2531	1.2981	1.2815	1.2991	1.2659	1.2981	1.2924	1.3858	1.4182	1.4256	1.4306	1.4133	1.4304	1.4165	1.4305	1.4256	1.4312	1.4437	**1.6**
**H(8`A-8`C)**	2.4763	2.4121	2.3914	2.3956	2.3829	2.3959	2.3669	2.3969	2.3791	2.3959	2.3540	2.9033	2.7432	2.7104	2.6626	2.6248	2.6682	2.5329	2.6741	2.5623	2.6576	2.5047	**2.81**
**F4**	-117.9861	-119.2076	-121.2891	-118.5282	-122.5446	-118.5433	-120.1915	-118.5580	-120.6873	-118.5136	-119.9422	-117.1236	-118.6403	-120.5867	-118.2581	-122.0157	-118.2436	-120.5752	-118.2316	-120.6654	-118.2748	-120.2123	**-102.1**
Nuclei	**M06-2X**																						
**C1**	153.0917	153.5294	154.5657	153.6798	155.3824	153.6910	154.3824	153.7038	154.6410	153.6636	154.5610	152.3025	153.6253	154.2248	154.4172	155.8548	154.3561	155.9192	154.3050	155.6636	154.4801	156.1304	**129**
**C2**	155.1746	155.0543	155.6859	154.8589	155.5556	154.8934	156.0475	154.9292	155.9461	154.8195	156.0984	154.7655	154.7734	155.3565	154.6837	155.4125	154.7088	155.8874	154.7355	155.7791	154.6544	155.9277	**132**
**C3**	136.1590	136.8604	137.4721	137.0980	137.8395	137.1065	138.0767	137.1160	138.0006	137.0834	138.1754	136.1934	136.8293	137.4400	137.0599	137.8148	137.0677	137.9965	137.0767	137.9426	137.0459	138.0999	**116**
**C4**	186.9866	187.8245	188.4710	188.2660	188.9823	188.2597	189.6225	188.2546	189.4454	188.2657	189.8145	187.4071	187.9865	188.7105	188.2373	189.0085	188.2488	189.3855	188.2602	189.3135	188.2178	189.5659	**167**
**C5**	133.5146	135.0251	135.7169	135.8167	136.7899	135.7892	136.9947	135.7628	136.8748	135.8366	137.2061	133.4360	134.8666	135.5636	135.5696	136.5427	135.5491	136.6670	135.5297	136.5791	135.5821	136.8705	**116**
**C6**	151.2911	153.5510	154.7995	154.7508	156.6302	154.6981	157.2971	154.6466	156.9660	154.7945	157.7502	151.6315	153.9149	155.3078	154.9928	157.0373	154.9595	157.4443	154.9245	157.2709	155.0146	157.8386	**132**
**C7**	218.4206	224.0871	225.6863	227.0228	227.7392	226.8634	233.4654	226.7049	232.2929	227.1679	234.4902	222.3731	227.5635	229.0579	230.3177	230.9202	230.1563	236.7866	230.0003	235.3937	230.4701	237.6501	**196**
**C8**	66.1021	67.3894	68.1145	67.8657	68.9271	67.8608	69.3536	67.8567	69.2455	67.8636	70.1061	60.3620	62.0622	62.4883	63.0156	63.8536	62.9637	64.7319	62.9168	64.3967	63.0637	65.0835	**60**
**C9**	23.2058	23.4985	23.7070	23.7340	24.1504	23.7389	24.1959	23.7459	24.1017	23.7236	24.3456	20.3942	20.6118	20.6433	20.7319	21.0349	20.7420	21.1263	20.7541	21.0549	20.7172	21.5210	**15**
**C8`**	36.0510	36.7555	36.9669	37.1150	37.4247	37.1125	37.6459	37.1106	37.5970	37.1109	38.1632	30.7152	31.1896	31.5368	31.3585	31.6695	31.3831	31.7058	31.4062	31.7955	31.3257	31.8659	**31**
**H2**	9.2412	9.1940	9.2146	9.1667	9.1653	9.1672	9.2317	9.1673	9.2138	9.1663	9.2327	9.2302	9.0921	9.1468	9.0068	9.0354	9.0138	9.0044	9.0198	9.0317	8.9993	9.0042	**8.07**
**H3**	7.8930	8.0315	8.1240	8.0797	8.2018	8.0760	8.2142	8.0720	8.2077	8.0833	8.2275	7.8444	7.9817	8.0724	8.0317	8.1517	8.0277	8.1674	8.0235	8.1599	8.0355	8.1819	**7.31**
**H5**	7.7188	7.9115	8.0040	8.0015	8.1305	7.9950	8.1471	7.9882	8.1342	8.0078	8.1715	7.7205	7.8974	7.9934	7.9760	8.1058	7.9705	8.1095	7.9646	8.1016	7.9813	8.1306	**7.31**
**H6**	8.7195	8.9331	9.0357	9.0522	9.2020	9.0436	9.2622	9.0346	9.2341	9.0605	9.2978	8.5491	8.7357	8.8647	8.8195	9.0069	8.8151	9.0001	8.8099	9.0002	8.8232	9.0400	**8.07**
**H8**	4.2292	4.3451	4.3884	4.4506	4.5168	4.4418	4.5788	4.4328	4.5575	4.4593	4.6093	4.4880	4.6312	4.7027	4.6959	4.8114	4.6915	4.8114	4.6865	4.8097	4.7000	4.8405	**5.09**
**H(9A-9C)**	1.3567	1.3681	1.3531	1.3672	1.3372	1.3663	1.3712	1.3650	1.3600	1.3682	1.3871	1.4261	1.4313	1.4276	1.4367	1.4173	1.4356	1.4268	1.4342	1.4284	1.4376	1.4441	**1.6**
**H(8`A-8`C)**	2.4619	2.4039	2.3762	2.3853	2.3641	2.3849	2.3357	2.3842	2.3446	2.3860	2.3207	2.7873	2.6416	2.6191	2.5775	2.5343	2.5805	2.4563	2.5830	2.4800	2.5745	2.4273	**2.81**
**F4**	-114.4865	-115.5461	-118.0466	-115.5383	-119.2719	-115.5463	-117.2772	-115.5522	-117.6551	-115.5307	-117.0120	-114.0898	-115.5429	-117.8876	-115.8591	-119.4131	-115.8335	-118.0296	-115.8097	-118.1523	-115.8875	-117.7694	**-102.1**

[a] methyl protons shifts are averaged

[b] according to [9]

Table S9. Cont. IV

Nuclei	R											S											Exp. [b]
	mpW1LYP																						
	Gas	$CHCl_3$ (PCM)	$CHCl_3$ (SMD)	DMSO (PCM)	DMSO (SMD)	CH_3OH (PCM)	CH_3OH (SMD)	C_2H_5OH (PCM)	C_2H_5OH (SMD)	H_2O (PCM)	H_2O (SMD)	Gas	$CHCl_3$ (PCM)	$CHCl_3$ (SMD)	DMSO (PCM)	DMSO (SMD)	CH_3OH (PCM)	CH_3OH (SMD)	C_2H_5OH (PCM)	C_2H_5OH (SMD)	H_2O (PCM)	H_2O (SMD)	
**C1**	139.4828	139.9525	140.8951	140.2162	142.1269	140.1880	141.3847	140.1639	141.5911	140.2438	141.6737	138.0239	139.3201	140.0314	140.0519	141.6015	140.0019	141.3526	139.9557	141.2617	140.1006	141.3699	**129**
**C2**	140.0654	139.9633	140.5214	139.8120	140.2594	139.8221	140.7067	139.8327	140.5833	139.8000	140.6754	139.3485	139.4849	140.0212	139.4970	140.0614	139.4929	140.6129	139.4906	140.4986	139.4995	140.6006	**132**
**C3**	121.9628	122.6121	123.1375	122.8712	123.4386	122.8537	123.7099	122.8374	123.6709	122.8862	123.7449	122.0772	122.6487	123.1165	122.8876	123.4426	122.8714	123.6473	122.8567	123.5970	122.9013	123.6861	**116**
**C4**	176.1473	177.0246	177.6646	177.5228	178.1467	177.4890	178.8123	177.4567	178.6743	177.5533	178.9071	176.5361	177.1020	177.7627	177.3919	178.0767	177.3729	178.4887	177.3548	178.3978	177.4074	178.6295	**167**
**C5**	119.7588	121.0521	121.6454	121.7767	122.5021	121.7290	122.7447	121.6831	122.6591	121.8202	122.8719	119.9121	121.1021	121.6630	121.7119	122.4355	121.6731	122.5793	121.6353	122.5049	121.7464	122.6956	**116**
**C6**	136.3411	138.3279	139.4122	139.4387	140.8254	139.3726	141.4341	139.3066	141.1391	139.4983	141.7648	136.6810	138.7304	140.0172	139.7711	141.3801	139.7081	141.9011	139.6456	141.7184	139.8277	142.2839	**132**
**C7**	202.6211	207.7782	209.3145	210.5766	211.6308	210.3901	217.0461	210.2089	216.1478	210.7558	217.9713	207.9684	212.1537	213.3072	214.4009	215.1623	214.2635	219.8843	214.1282	218.8057	214.5296	220.3958	**196**
**C8**	64.8087	65.9413	66.6307	66.2710	67.2010	66.2555	67.4970	66.2398	67.3414	66.2823	68.2675	59.1606	61.1502	61.8125	62.1638	63.0102	62.0972	63.6763	62.0334	63.4471	62.2269	63.9055	**60**
**C9**	23.0194	23.2782	23.4066	23.5626	23.7640	23.5450	23.7639	23.5280	23.6835	23.5763	23.8872	19.5716	19.9981	20.1757	20.2377	20.4306	20.2213	20.5847	20.2064	20.5225	20.2514	20.9210	**15**
**C8`**	36.3159	37.0893	37.3474	37.4110	37.7421	37.3893	37.9553	37.3691	37.9323	37.4299	38.3577	31.9503	32.0958	32.3503	32.2714	32.4808	32.2555	32.5948	32.2408	32.6120	32.2842	32.6749	**31**
**H2**	8.6760	8.6134	8.6345	8.5731	8.5352	8.5769	8.5767	8.5801	8.5650	8.5692	8.5537	8.7155	8.5439	8.5843	8.4575	8.4611	8.4631	8.4509	8.4681	8.4656	8.4518	8.4608	**8.07**
**H3**	7.3397	7.4720	7.5610	7.5164	7.6269	7.5145	7.6429	7.5123	7.6350	7.5181	7.6539	7.3222	7.4540	7.5397	7.5009	7.6151	7.4987	7.6299	7.4961	7.6226	7.5029	7.6422	**7.31**
**H5**	7.1905	7.3768	7.4660	7.4635	7.5825	7.4586	7.6016	7.4535	7.5914	7.4679	7.6207	7.1791	7.3532	7.4411	7.4319	7.5518	7.4276	7.5619	7.4231	7.5524	7.4357	7.5804	**7.31**
**H6**	8.0834	8.2895	8.3770	8.4032	8.5186	8.3966	8.5837	8.3898	8.5634	8.4092	8.6049	7.8914	8.0614	8.1768	8.1478	8.3090	8.1432	8.3269	8.1382	8.3161	8.1519	8.3729	**8.07**
**H8**	4.3063	4.3981	4.4252	4.5055	4.5605	4.4978	4.6229	4.4902	4.6105	4.5128	4.6327	4.4564	4.6192	4.6720	4.6856	4.7973	4.6823	4.8170	4.6786	4.8046	4.6884	4.8463	**5.09**
**H(9A-9C)**	1.3216	1.3308	1.3202	1.3215	1.2774	1.3232	1.2903	1.3245	1.2801	1.3196	1.3009	1.4008	1.4361	1.4422	1.4560	1.4325	1.4551	1.4529	1.4538	1.4517	1.4567	1.4756	**1.6**
**H(8`A-8`C)**	2.5203	2.4606	2.4330	2.4451	2.4238	2.4459	2.4070	2.4465	2.4101	2.4443	2.3934	2.9829	2.8104	2.7656	2.7300	2.6802	2.7354	2.5986	2.7403	2.6192	2.7247	2.5674	**2.81**
**F4**	-123.5335	-124.7384	-127.5322	-124.8079	-129.1894	-124.8085	-126.9584	-124.8080	-127.3233	-124.8073	-126.7826	-123.2212	-124.9948	-127.7245	-125.4158	-129.5143	-125.3951	-127.8153	-125.3738	-128.0377	-125.4361	-127.4735	**-102.1**
Nuclei	mpW1PW91																						
**C1**	136.7091	137.1261	138.2904	137.3527	139.3925	137.3342	138.8484	137.3222	138.8794	137.3708	139.0868	135.4965	136.8443	137.3776	137.4319	138.8932	137.3747	138.9047	137.3297	138.6184	137.4927	138.9966	**129**
**C2**	139.0510	138.9813	139.5567	138.8023	139.3841	138.8154	139.7972	138.8338	139.6690	138.7889	139.7849	138.4920	138.6376	139.1844	138.5926	139.2587	138.5931	139.7425	138.5998	139.6330	138.5920	139.7648	**132**
**C3**	121.1434	121.8839	122.4572	122.1398	122.8577	122.1259	123.1111	122.1175	123.0669	122.1523	123.1972	121.1969	121.8835	122.4042	122.1282	122.8348	122.1142	123.0503	122.1059	122.9791	122.1411	123.1290	**116**
**C4**	173.1450	174.0361	174.6361	174.4798	175.1382	174.4530	175.7414	174.4320	175.6481	174.5044	175.9056	173.4356	174.0253	174.6981	174.2966	175.0454	174.2852	175.4189	174.2781	175.3524	174.3057	175.5825	**167**
**C5**	118.9666	120.3546	120.9855	121.0745	121.9413	121.0305	122.1805	120.9922	122.0946	121.1155	122.3576	119.0635	120.3480	120.9568	120.9724	121.8462	120.9359	121.9978	120.9051	121.9093	121.0057	122.1526	**116**
**C6**	135.1188	137.1393	138.1020	138.2106	139.6150	138.1465	140.1616	138.0877	139.9062	138.2707	140.5379	135.4925	137.5198	138.7879	138.5089	140.2202	138.4556	140.6265	138.4058	140.5120	138.5558	141.0214	**132**
**C7**	200.5499	205.4732	207.1087	208.0696	209.2352	207.9079	214.4321	207.7523	213.5361	208.2238	215.3863	204.9687	209.2878	210.4288	211.5065	212.2688	211.3693	217.0803	211.2400	215.8598	211.6406	217.5855	**196**
**C8**	62.5871	63.7277	64.3921	64.0779	65.0594	64.0579	65.5692	64.0437	65.2428	64.0959	66.2934	57.3088	59.1083	59.5712	59.9715	60.7733	59.9036	61.5602	59.8450	61.2368	60.0391	61.8431	**60**
**C9**	22.4303	22.7074	22.8165	22.9714	23.2346	22.9561	23.2515	22.9463	23.1372	22.9849	23.3928	19.1269	19.5971	19.6794	19.7761	20.0297	19.7623	20.1776	19.7543	20.1086	19.7888	20.5773	**15**
**C8`**	35.5389	36.3291	36.5286	36.6000	36.9580	36.5839	37.1500	36.5735	37.1332	36.6149	37.6110	31.0253	31.2540	31.5589	31.4502	31.7343	31.4412	31.7914	31.4363	31.8597	31.4556	31.8914	**31**
**H2**	8.7270	8.6729	8.6666	8.6268	8.5829	8.6306	8.6029	8.6343	8.6110	8.6233	8.5930	8.7399	8.5718	8.6280	8.4933	8.5106	8.5006	8.4771	8.5073	8.5099	8.4860	8.4856	**8.07**
**H3**	7.3666	7.5071	7.5941	7.5506	7.6672	7.5488	7.6842	7.5469	7.6761	7.5526	7.6994	7.3521	7.4918	7.5782	7.5379	7.6587	7.5357	7.6748	7.5336	7.6662	7.5401	7.6902	**7.31**
**H5**	7.2156	7.4099	7.4980	7.4952	7.6199	7.4906	7.6379	7.4860	7.6293	7.4999	7.6618	7.2035	7.3869	7.4755	7.4658	7.5930	7.4616	7.6047	7.4575	7.5936	7.4700	7.6265	**7.31**
**H6**	8.1720	8.3810	8.4466	8.4858	8.5927	8.4800	8.6356	8.4742	8.6335	8.4916	8.6714	7.9622	8.1369	8.2584	8.2244	8.3946	8.2208	8.3985	8.2168	8.3988	8.2277	8.4460	**8.07**
**H8**	4.3244	4.4232	4.4417	4.5212	4.5686	4.5149	4.6073	4.5088	4.6209	4.5275	4.6357	4.4958	4.6417	4.6998	4.7058	4.8233	4.7033	4.8370	4.7007	4.8311	4.7081	4.8693	**5.09**
**H(9A-9C)**	1.3031	1.3123	1.2861	1.3005	1.2512	1.3018	1.2673	1.3032	1.2532	1.2994	1.2798	1.3839	1.4107	1.4119	1.4245	1.4007	1.4244	1.4147	1.4241	1.4196	1.4248	1.4408	**1.6**
**H(8`A-8`C)**	2.4582	2.3926	2.3693	2.3752	2.3559	2.3760	2.3459	2.3770	2.3405	2.3747	2.3305	2.8765	2.7109	2.6756	2.6345	2.5913	2.6410	2.5022	2.6472	2.5276	2.6285	2.4719	**2.81**
**F4**	-113.4956	-114.2818	-116.9359	-114.1722	-118.3272	-114.1834	-116.1885	-114.1934	-116.4533	-114.1628	-115.9268	-113.3010	-114.6367	-117.0827	-114.8267	-118.6086	-114.8120	-116.9763	-114.7988	-117.1332	-114.8441	-116.6468	**-102.1**
Nuclei	O3LYP																						
**C1**	133.9706	135.2869	135.1280	135.8992	137.5413	135.8935	136.8634	135.8840	136.9936	135.9000	136.9568	131.9841	134.0269	134.9667	134.8776	136.4625	134.8098	136.5165	134.7451	136.4801	134.9454	136.6774	**129**
**C2**	134.2294	134.0386	134.6059	134.0020	134.6215	134.0143	135.0928	134.0931	135.0297	133.9844	135.0347	133.6011	133.8315	134.4861	133.8598	134.5658	133.8686	134.9908	133.8725	134.9155	133.8449	134.9967	**132**
**C3**	117.1429	117.8472	118.4244	118.1586	118.8498	118.1512	119.1048	118.1420	119.0383	118.1592	119.1705	117.2423	117.8329	118.4008	118.1462	118.7940	118.1374	118.9776	118.1236	118.9199	118.1480	119.0503	**116**
**C4**	169.3331	170.2178	170.9930	170.6800	171.4504	170.6575	171.9898	170.6246	171.8402	170.6953	172.1262	169.6935	170.1374	170.8394	170.4612	171.2113	170.4568	171.5048	170.4458	171.4066	170.4567	171.6400	**167**
**C5**	115.1244	116.3261	117.0316	116.9791	117.7550	116.9488	117.9702	116.9062	117.8594	117.0010	118.1340	115.2829	116.3481	116.9834	116.9736	117.7506	116.9474	117.8633	116.9154	117.7787	116.9914	118.0028	**116**
**C6**	130.1343	131.8403	133.2468	132.9143	134.4244	132.8471	134.9826	132.7768	134.7083	132.9717	135.3818	130.8195	132.4740	133.5676	133.4119	134.9398	133.3757	135.3121	133.3309	135.1128	133.4364	135.6140	**132**
**C7**	196.4324	202.4039	202.9208	205.2799	206.3184	205.1284	211.0569	204.9884	210.1352	205.4208	211.6531	200.9322	205.3707	206.9113	207.7346	208.6887	207.5898	212.9882	207.4429	212.0350	207.8698	213.5094	**196**
**C8**	62.7269	63.8638	64.1933	64.6392	65.2111	64.6247	65.7186	64.6144	65.6398	64.6448	66.4308	57.3727	59.9629	60.5156	61.0054	61.8178	60.9369	62.7983	60.8688	62.5812	61.0698	63.0624	**60**
**C9**	21.9077	22.1497	22.4381	22.4448	22.6922	22.4333	22.8050	22.4257	22.7348	22.4497	22.9545	18.3907	18.7982	18.9135	19.0083	19.2082	19.0039	19.4486	18.9953	19.3637	19.0069	19.7532	**15**
**C8`**	35.8615	36.6415	36.9984	37.0897	37.4384	37.0783	37.5559	37.0769	37.5415	37.0931	37.8889	31.6791	31.7885	32.1081	32.0205	32.3175	32.0201	32.2296	32.0132	32.2550	32.0126	32.2365	**31**
**H2**	8.5171	8.3815	8.5110	8.3059	8.3131	8.3086	8.3569	8.3167	8.3480	8.3030	8.3618	8.6115	8.3742	8.4056	8.2874	8.3033	8.2956	8.2770	8.3028	8.2844	8.2784	8.2790	**8.07**
**H3**	7.2896	7.4098	7.5061	7.4543	7.5768	7.4521	7.6030	7.4468	7.5953	7.4562	7.6133	7.2766	7.4071	7.4943	7.4552	7.5707	7.4530	7.5845	7.4505	7.5766	7.4572	7.5999	**7.31**
**H5**	7.1552	7.3290	7.4298	7.3986	7.5094	7.3950	7.5202	7.3889	7.5085	7.4017	7.5463	7.1395	7.3009	7.3898	7.3774	7.4980	7.3735	7.5074	7.3692	7.4968	7.3810	7.5278	**7.31**
**H6**	8.0527	8.2175	8.3672	8.3017	8.4640	8.2946	8.5029	8.2853	8.4809	8.3083	8.5390	7.8574	7.9837	8.0812	8.0642	8.2140	8.0612	8.2127	8.0575	8.2001	8.0664	8.2508	**8.07**
**H8**	4.4166	4.5098	4.5410	4.5642	4.6714	4.5583	4.6852	4.5532	4.6658	4.5699	4.7170	4.5458	4.6793	4.7411	4.7470	4.8642	4.7444	4.8665	4.7410	4.8556	4.7490	4.8955	**5.09**
**H(9A-9C)**	1.2321	1.2056	1.2312	1.2013	1.1647	1.2018	1.1980	1.2027	1.1895	1.2008	1.2166	1.3321	1.3605	1.3485	1.3797	1.3521	1.3795	1.3771	1.3787	1.3725	1.3796	1.3957	**1.6**
**H(8`A-8`C)**	2.4772	2.4452	2.4019	2.4401	2.4019	2.4414	2.3990	2.4451	2.4021	2.4388	2.3752	2.9410	2.7694	2.7347	2.6983	2.6563	2.7041	2.5681	2.7092	2.5820	2.6922	2.5313	**2.81**
**F4**	-118.7922	-120.1360	-122.4287	-120.4098	-124.6326	-120.4123	-122.4604	-120.5582	-122.8366	-120.4073	-122.1699	-118.5618	-120.4429	-123.1971	-120.8378	-124.9656	-120.8070	-123.4420	-120.7796	-123.6814	-120.8716	-123.1412	**-102.1**
Nuclei	OLYP																						
**C1**	132.8188	134.1516	135.6656	135.1449	137.5235	135.1515	135.6652	135.1532	135.8434	135.1337	135.7556	130.8981	132.7261	133.7888	133.6768	135.1782	133.6081	135.1883	133.5382	135.1931	133.7379	135.3980	**129**
**C2**	131.6623	131.6975	132.2794	131.7465	132.3472	131.7531	132.6913	131.7546	132.6220	131.7302	132.7039	131.2536	131.3661	132.0518	131.4162	132.0990	131.4235	132.5894	131.4250	132.5059	131.4005	132.5752	**132**
**C3**	115.3417	116.0422	116.6555	116.3161	116.9608	116.3056	117.2576	116.2899	117.1915	116.3169	117.3369	114.4114	116.0541	116.6230	116.3750	116.9979	116.3650	117.1604	116.3492	117.1047	116.3752	117.2329	**116**
**C4**	168.2126	169.2294	169.9205	169.5710	170.1814	169.5487	170.9191	169.5215	170.7744	169.5840	171.0771	168.4577	169.1151	169.8267	169.4427	170.2413	169.4360	170.4899	169.4224	170.3927	169.4386	170.6134	**167**
**C5**	113.3427	114.4633	115.0535	115.0655	115.7600	115.0339	116.1215	114.9960	116.0017	115.0864	116.2699	113.4100	114.5734	115.1849	115.1704	115.9016	115.1450	116.0297	115.1131	115.9473	115.1848	116.1602	**116**
**C6**	127.6886	129.4580	130.3992	130.3959	131.5991	130.3259	132.5021	130.2522	132.2325	130.4552	132.8719	132.4882	129.9940	131.1063	130.8821	132.4316	130.8446	132.8458	130.8002	132.6433	130.9066	133.1053	**132**
**C7**	195.3251	201.0301	202.9899	204.5243	206.1991	204.3850	209.0998	204.2443	208.2692	204.6535	209.6663	194.9333	204.0205	205.4470	206.3531	207.3591	206.2114	211.0678	206.0639	210.2799	206.4776	211.6073	**196**
**C8**	63.8385	65.2339	66.0677	65.6811	66.8369	65.6883	66.9203	65.6854	66.8721	65.6665	67.5606	62.8816	61.2250	62.0414	62.3546	63.1411	62.2822	64.2114	62.2081	64.0080	62.4201	64.4616	**60**
**C9**	22.0417	22.3211	22.5251	22.7061	22.8886	22.6962	23.1868	22.6811	23.0909	22.7070	23.3016	15.7972	18.8615	19.1193	19.1190	19.3070	19.1099	19.6432	19.0962	19.5371	19.1202	19.9486	**15**
**C8`**	36.5297	37.3375	37.5614	37.8176	38.0745	37.8036	38.1673	37.7827	38.1190	37.8211	38.5013	31.4966	32.4077	32.6511	32.6378	32.9272	32.6373	32.7834	32.6299	32.8079	32.6276	32.7561	**31**
**H2**	8.3522	8.2235	8.1969	8.1272	8.0714	8.1268	8.2234	8.1264	8.2066	8.1267	8.2299	8.4369	8.2352	8.2566	8.1468	8.1702	8.1543	8.1611	8.1613	8.1618	8.1388	8.1574	**8.07**
**H3**	7.2072	7.3275	7.4178	7.4008	7.5127	7.3974	7.5330	7.3939	7.5211	7.4035	7.5445	7.1593	7.3325	7.4196	7.3792	7.4934	7.3770	7.5033	7.3746	7.4956	7.3809	7.5175	**7.31**
**H5**	7.0851	7.2353	7.3143	7.2988	7.4157	7.2941	7.4375	7.2893	7.4251	7.3029	7.4576	7.0403	7.2266	7.3145	7.2993	7.4188	7.2955	7.4288	7.2915	7.4188	7.3025	7.4474	**7.31**
**H6**	7.9775	8.1327	8.1956	8.2132	8.2977	8.2041	8.4009	8.1954	8.3698	8.2217	8.4374	8.7484	7.8865	7.9743	7.9576	8.1089	7.9547	8.1075	7.9514	8.0939	7.9595	8.1417	**8.07**
**H8**	4.5323	4.5911	4.6096	4.6267	4.6824	4.6200	4.7353	4.6144	4.7106	4.6332	4.7707	4.4883	4.7554	4.8166	4.8221	4.9436	4.8190	4.9419	4.8155	4.9305	4.8243	4.9712	**5.09**
**H(9A-9C)**	1.2237	1.2082	1.1959	1.1823	1.1420	1.1826	1.2055	1.1826	1.1992	1.1815	1.2218	1.2466	1.3653	1.3601	1.3854	1.3618	1.3848	1.3978	1.3840	1.3909	1.3856	1.4134	**1.6**
**H(8`A-8`C)**	2.5179	2.4873	2.4697	2.4886	2.4649	2.4895	2.4510	2.4902	2.4526	2.4873	2.4211	2.3523	2.8150	2.7687	2.7435	2.7024	2.7489	2.6101	2.7538	2.6211	2.7376	2.5695	**2.81**
**F4**	-126.2399	-127.8764	-131.1829	-128.1328	-133.2759	-128.1639	-130.3061	-128.1920	-130.7905	-128.1043	-129.9584	-125.1106	-127.9468	-131.0095	-128.4021	-132.8593	-128.3693	-131.3222	-128.3392	-131.5975	-128.4363	-131.0041	**-102.1**

[a] methyl protons shifts are averaged
[b] according to [9]

Table S9. Cont. V

Nuclei	R											S											Exp. [b]
	OPBE																						
	Gas	$CHCl_3$ (PCM)	$CHCl_3$ (SMD)	DMSO (PCM)	DMSO (SMD)	CH_3OH (PCM)	CH_3OH (SMD)	C_2H_5OH (PCM)	C_2H_5OH (SMD)	H_2O (PCM)	H_2O (SMD)	Gas	$CHCl_3$ (PCM)	$CHCl_3$ (SMD)	DMSO (PCM)	DMSO (SMD)	CH_3OH (PCM)	CH_3OH (SMD)	C_2H_5OH (PCM)	C_2H_5OH (SMD)	H_2O (PCM)	H_2O (SMD)	
**C1**	129.7640	131.1387	132.6552	132.1246	134.4950	132.1378	132.7494	132.1341	132.8307	132.0968	132.7529	127.9531	129.7572	131.3960	130.9073	132.9633	130.8138	132.2249	130.6965	132.6314	130.9636	132.5011	**129**
**C2**	130.3586	130.4886	130.9968	130.4429	131.0543	130.4635	131.3872	130.4661	131.3319	130.3820	131.3838	129.9865	130.2359	130.9157	130.1823	130.9380	130.1990	131.3075	130.1994	131.2030	130.1628	131.3207	**132**
**C3**	114.2213	115.0550	115.6259	115.3016	116.0144	115.3021	116.3293	115.2846	116.2561	115.3063	116.4542	114.3041	115.0698	115.5900	115.3365	116.0072	115.3394	116.2309	115.3240	116.1537	115.3295	116.3409	**116**
**C4**	164.6226	165.6789	166.2199	165.9412	166.5402	165.9340	167.2498	165.9090	167.1104	166.0101	167.4931	164.9027	165.5555	166.0292	165.7368	166.3569	165.7530	166.8192	165.7519	166.6262	165.7218	166.9648	**167**
**C5**	112.2687	113.5344	114.0760	114.0744	114.8381	114.0527	115.1922	114.0131	115.0739	114.1130	115.4008	112.4202	113.6143	114.1128	114.1369	114.8497	114.1271	115.0869	114.0994	114.9493	114.1433	115.2524	**116**
**C6**	126.1608	127.9997	128.8093	128.8180	129.9411	128.7696	130.8401	128.7038	130.6005	128.8489	131.2575	126.8203	128.5704	129.4079	129.2674	130.6027	129.2570	131.2141	129.2292	130.8690	129.2803	131.4889	**132**
**C7**	192.8626	198.3520	200.1588	201.6561	203.2107	201.5462	206.1754	201.4188	205.3233	201.7358	206.5747	196.3497	200.6868	202.1872	203.0429	204.1492	202.9127	207.7285	202.7568	207.1557	203.1474	208.2542	**196**
**C8**	61.2284	62.7133	63.5636	63.1881	64.3519	63.1850	64.4468	63.1637	64.3074	63.1720	65.1108	56.6311	58.7512	59.9248	59.9517	61.0884	59.8539	61.5973	59.7391	61.6414	60.0230	61.9029	**60**
**C9**	21.2805	21.6678	21.7922	21.9610	22.1440	21.9624	22.3933	21.9464	22.3075	21.9463	22.5303	17.8291	18.2838	18.5005	18.5007	18.7647	18.4933	18.9349	18.4692	18.8763	18.4996	19.2950	**15**
**C8`**	35.4845	36.3569	36.4549	36.7323	36.9249	36.7278	37.0000	36.7052	36.9687	36.7163	37.3820	31.0988	31.3654	31.3318	31.4174	31.5251	31.4408	31.6115	31.4469	31.5375	31.3965	31.5941	**31**
**H2**	8.3870	8.2642	8.2215	8.1564	8.0952	8.1575	8.2408	8.1577	8.2328	8.1562	8.2594	8.4575	8.2769	8.2227	8.1564	8.1258	8.1678	8.1855	8.1792	8.1490	8.1477	8.1810	**8.07**
**H3**	7.2282	7.3602	7.4433	7.4277	7.5438	7.4252	7.5609	7.4218	7.5508	7.4327	7.5779	7.2304	7.3678	7.4497	7.4149	7.5264	7.4130	7.5438	7.4104	7.5341	7.4158	7.5608	**7.31**
**H5**	7.1021	7.2653	7.3426	7.3260	7.4471	7.3219	7.4668	7.3170	7.4549	7.3330	7.4935	7.0869	7.2574	7.3371	7.3302	7.4448	7.3268	7.4602	7.3227	7.4473	7.3327	7.4817	**7.31**
**H6**	8.0380	8.1929	8.2297	8.2560	8.3210	8.2498	8.4322	8.2430	8.4104	8.2608	8.4821	7.8021	7.9511	7.9981	8.0067	8.1209	8.0068	8.1616	8.0062	8.1245	8.0072	8.1968	**8.07**
**H8**	4.5419	4.6115	4.6071	4.6376	4.6660	4.6345	4.7386	4.6308	4.7174	4.6371	4.7816	4.6657	4.7892	4.8168	4.8395	4.9337	4.8401	4.9559	4.8394	4.9298	4.8389	4.9886	**5.09**
**H(9A-9C)**	1.1827	1.1710	1.1506	1.1424	1.0922	1.1427	1.1573	1.1423	1.1499	1.1403	1.1786	1.2938	1.3294	1.3061	1.3393	1.2986	1.3395	1.3428	1.3392	1.3289	1.3389	1.3621	**1.6**
**H(8`A-8`C)**	2.4371	2.4087	2.3861	2.4045	2.3742	2.4058	2.3612	2.4063	2.3625	2.4000	2.3311	2.8529	2.7064	2.6163	2.6177	2.5447	2.6266	2.4933	2.6347	2.4903	2.6099	2.4549	**2.81**
**F4**	-115.1642	-116.2433	-119.3426	-116.3765	-121.1136	-116.4067	-118.1552	-116.4377	-118.5815	-116.2483	-117.6067	-115.0982	-116.3610	-119.4888	-116.7532	-121.2078	-116.7051	-119.0436	-116.6554	-119.6253	-116.7819	-118.7471	**-102.1**
Nuclei	OPW91																						
**C1**	130.0734	131.4711	132.9769	132.4406	134.8087	132.4451	133.0129	132.4321	133.1402	132.4191	133.0519	128.2394	130.0724	131.7005	131.1724	133.2463	131.0761	132.5216	130.9590	132.9078	131.2337	132.7953	**129**
**C2**	130.5806	130.7074	131.2089	130.6288	131.2637	130.6390	131.5719	130.6356	131.5478	130.6007	131.6048	130.1962	130.4533	131.1259	130.4042	131.1441	130.4116	131.5201	130.4034	131.4168	130.3802	131.5359	**132**
**C3**	114.4183	115.2508	115.8151	115.5092	116.1968	115.4994	116.5211	115.4732	116.4393	115.4999	116.6391	114.5029	115.2650	115.7792	115.5361	116.1909	115.5285	116.4136	115.5035	116.3378	115.5247	116.5251	**116**
**C4**	165.0246	166.0821	166.6217	166.4195	166.9443	166.4003	167.7080	166.3664	167.5188	166.4192	167.9027	165.3169	165.9654	166.4385	166.1639	166.7698	166.1681	167.2288	166.1553	167.0408	166.1434	167.3773	**167**
**C5**	112.4634	113.7286	114.2665	114.2944	115.0217	114.2618	115.3914	114.2128	115.2585	114.3067	115.5871	112.6210	113.8117	114.3056	114.3432	115.0398	114.3225	115.2744	114.2843	115.1397	114.3450	115.4419	**116**
**C6**	126.3826	128.2204	129.0301	129.0438	130.1652	128.9844	131.0735	128.9103	130.8310	129.0819	131.4938	127.0499	128.7969	129.6351	129.5203	130.8358	129.4965	131.4451	129.4563	131.1082	129.5270	131.7238	**132**
**C7**	193.2195	198.7503	200.5473	202.0469	203.6032	201.9273	206.5400	201.7965	205.7249	202.1429	206.9853	196.7445	201.1005	202.5918	203.4416	204.5479	203.3022	208.1416	203.1393	207.5501	203.5465	208.6706	**196**
**C8**	61.4164	62.9130	63.7519	63.3730	64.5345	63.3605	64.5943	63.3262	64.4929	63.3665	65.2823	56.7828	58.9381	60.1067	60.1087	61.2534	60.0077	61.7812	59.8918	61.8124	60.1818	62.0859	**60**
**C9**	21.3412	21.7318	21.8564	22.0225	22.2056	22.0139	22.4453	21.9905	22.3695	22.0127	22.5934	17.9020	18.3623	18.5810	18.5780	18.8386	18.5624	19.0151	18.5310	18.9558	18.5743	19.3737	**15**
**C8`**	35.5474	36.4260	36.5197	36.7969	36.9960	36.7816	37.0617	36.7522	37.0400	36.7928	37.4582	31.1915	31.4588	31.4241	31.5288	31.6223	31.5399	31.7046	31.5341	31.6351	31.5020	31.6928	**31**
**H2**	8.3887	8.2639	8.2222	8.1590	8.0961	8.1592	8.2467	8.1596	8.2338	8.1570	8.2615	8.4616	8.2780	8.2245	8.1636	8.1292	8.1736	8.1874	8.1840	8.1527	8.1537	8.1831	**8.07**
**H3**	7.2300	7.3612	7.4442	7.4326	7.5443	7.4293	7.5651	7.4261	7.5509	7.4339	7.5785	7.2318	7.3686	7.4505	7.4163	7.5267	7.4137	7.5442	7.4109	7.5345	7.4169	7.5610	**7.31**
**H5**	7.1042	7.2672	7.3446	7.3319	7.4481	7.3269	7.4705	7.3219	7.4560	7.3348	7.4945	7.0894	7.2592	7.3388	7.3327	7.4461	7.3286	7.4614	7.3242	7.4485	7.3348	7.4827	**7.31**
**H6**	8.0365	8.1901	8.2278	8.2553	8.3202	8.2483	8.4351	8.2419	8.4096	8.2601	8.4824	7.8035	7.9505	7.9980	8.0099	8.1218	8.0088	8.1616	8.0074	8.1258	8.0096	8.1969	**8.07**
**H8**	4.5413	4.6099	4.6067	4.6350	4.6660	4.6313	4.7388	4.6276	4.7179	4.6370	4.7820	4.6622	4.7853	4.8138	4.8390	4.9316	4.8382	4.9531	4.8368	4.9279	4.8379	4.9856	**5.09**
**H(9A-9C)**	1.1844	1.1724	1.1522	1.1437	1.0939	1.1434	1.1586	1.1424	1.1520	1.1425	1.1806	1.2943	1.3301	1.3074	1.3418	1.3002	1.3413	1.3445	1.3406	1.3308	1.3411	1.3636	**1.6**
**H(8`A-8`C)**	2.4383	2.4106	2.3882	2.4045	2.3762	2.4051	2.3601	2.4055	2.3644	2.4024	2.3329	2.8575	2.7104	2.6209	2.6250	2.5505	2.6328	2.4979	2.6400	2.4956	2.6165	2.4597	**2.81**
**F4**	-115.7848	-116.9206	-120.0301	-116.9646	-121.8199	-116.9928	-118.7228	-117.0127	-119.2902	-116.9380	-118.3062	-115.7012	-117.0262	-120.1651	-117.4050	-121.8965	-117.3613	-119.7414	-117.3169	-120.3065	-117.4400	-119.4445	**-102.1**
Nuclei	PBE																						
**C1**	135.5369	136.0918	137.1653	136.3677	138.3715	136.3432	137.9723	136.3302	138.0000	136.4097	138.2666	133.9549	135.5103	136.0530	136.0986	137.6401	136.0426	137.7558	136.0033	137.5912	136.1760	137.8979	**129**
**C2**	135.9657	136.0179	136.6216	135.7847	136.4967	135.7983	136.9066	135.8222	136.8094	135.7890	136.9073	135.2241	135.5670	136.1193	135.5057	136.3042	135.5028	136.8654	135.5121	136.7580	135.5269	136.8854	**132**
**C3**	119.4465	120.3299	120.8882	120.5608	121.3376	120.5455	121.6570	120.5407	121.5966	120.5927	121.7624	119.5345	120.3283	120.8574	120.5295	121.3174	120.5157	121.5667	120.5123	121.4938	120.5600	121.6702	**116**
**C4**	173.9823	175.0866	175.7504	175.5136	176.3815	175.4853	176.9469	175.4679	176.8322	175.5580	177.1272	174.3115	175.1027	175.8451	175.3569	176.3062	175.3425	176.6457	175.3376	176.5574	175.3868	176.8245	**167**
**C5**	117.3377	118.8348	119.4453	119.5117	120.4263	119.4676	120.7320	119.4345	120.6335	119.5712	120.9231	117.5420	118.8521	119.4821	119.4030	120.3643	119.3686	120.5554	119.3443	120.4643	119.4527	120.7344	**116**
**C6**	132.4775	134.5082	135.5902	135.4775	137.1352	135.4145	137.6377	135.3620	137.3771	135.5552	138.0465	132.8577	134.9572	136.2454	135.8915	137.7657	135.8360	138.2041	135.7900	138.0185	135.9600	138.5976	**132**
**C7**	200.2846	204.9361	206.5139	207.3187	208.7358	207.1695	213.0970	207.0319	212.3370	207.4784	214.0538	205.2335	209.1883	210.4404	211.1454	212.2526	211.0237	216.2129	210.9132	215.2932	211.2804	216.6930	**196**
**C8**	66.5088	67.7352	68.5365	68.0186	69.2245	67.9992	69.7030	67.9905	69.4643	68.0544	70.5121	60.8379	62.8084	63.3380	63.6487	64.6563	63.5816	65.3895	63.5289	65.1438	63.7343	65.6928	**60**
**C9**	23.3476	23.7914	23.9606	24.0315	24.4395	24.0128	24.5728	24.0048	24.4653	24.0668	24.7581	19.9739	20.6830	20.7908	20.8644	21.2357	20.8478	21.5194	20.8429	21.4105	20.8983	22.0261	**15**
**C8`**	37.1172	37.9328	38.1214	38.1135	38.5395	38.1010	38.7049	38.0990	38.6815	38.1431	39.1975	32.1770	32.4188	32.7113	32.5246	32.8859	32.5166	32.9628	32.5184	32.9920	32.5484	33.0440	**31**
**H2**	8.4940	8.4541	8.4552	8.4051	8.3713	8.4089	8.3990	8.4131	8.4018	8.4020	8.3885	8.5295	8.3765	8.4273	8.2992	8.3159	8.3056	8.2934	8.3120	8.3107	8.2932	8.2994	**8.07**
**H3**	7.2033	7.3447	7.4313	7.3857	7.5032	7.3838	7.5208	7.3823	7.5132	7.3881	7.5355	7.1915	7.3278	7.4143	7.3701	7.4921	7.3680	7.5036	7.3664	7.4955	7.3727	7.5186	**7.31**
**H5**	7.0554	7.2476	7.3344	7.3285	7.4528	7.3239	7.4719	7.3198	7.4630	7.3335	7.4950	7.0514	7.2254	7.3152	7.2983	7.4275	7.2942	7.4386	7.2906	7.4284	7.3027	7.4597	**7.31**
**H6**	8.0650	8.2613	8.3213	8.3484	8.4499	8.3435	8.4938	8.3391	8.4841	8.3536	8.5258	7.8075	7.9693	8.0875	8.0493	8.2174	8.0456	8.2265	8.0420	8.2172	8.0532	8.2729	**8.07**
**H8**	4.5942	4.6682	4.6640	4.7434	4.7783	4.7384	4.8154	4.7339	4.8140	4.7488	4.8411	4.7365	4.8721	4.9225	4.9285	5.0427	4.9260	5.0540	4.9238	5.0416	4.9312	5.0891	**5.09**
**H(9A-9C)**	1.3652	1.3737	1.3620	1.3582	1.3268	1.3594	1.3445	1.3610	1.3344	1.3577	1.3585	1.4620	1.4973	1.5000	1.5101	1.4967	1.5097	1.5158	1.5096	1.5145	1.5110	1.5442	**1.6**
**H(8`A-8`C)**	2.5658	2.4898	2.4677	2.4656	2.4518	2.4665	2.4330	2.4680	2.4322	2.4654	2.4135	3.0023	2.8324	2.7952	2.7516	2.7069	2.7580	2.6137	2.7646	2.6329	2.7457	2.5792	**2.81**
**F4**	-131.1424	-131.7563	-134.9700	-131.5194	-136.5599	-131.5379	-134.0199	-131.5553	-134.3640	-131.5024	-133.6741	-130.9917	-132.3049	-135.2933	-132.4127	-137.0364	-132.4049	-135.1822	-132.3985	-135.4687	-132.4236	-134.7644	**-102.1**
Nuclei	PBE0																						
**C1**	136.2247	136.6526	137.6494	136.8626	138.8179	136.8401	138.3389	136.8206	138.4202	136.8878	138.5165	135.0705	136.3670	136.9426	136.9688	138.3856	136.9063	138.4604	136.8512	138.1253	137.0380	138.5078	**129**
**C2**	138.7455	138.6972	139.2899	138.5224	139.1126	138.5294	139.6279	138.5381	139.4524	138.5187	139.5604	138.2240	138.3806	138.9274	138.3295	138.9750	138.3256	139.5396	138.3238	139.3720	138.3367	139.5045	**132**
**C3**	120.8925	121.6540	122.2093	121.9097	122.6105	121.8907	122.9447	121.8736	122.8377	121.9304	122.9696	120.9444	121.6525	122.1782	121.8956	122.5853	121.8764	122.8788	121.8592	122.7522	121.9168	122.9010	**116**
**C4**	172.7695	173.6677	174.2592	174.1060	174.7504	174.0748	175.4368	174.0455	175.2494	174.1384	175.5356	173.0167	173.6449	174.3079	173.9164	174.6482	173.8991	175.1081	173.8827	174.9805	173.9346	175.2141	**167**
**C5**	118.6951	120.1057	120.7177	120.8251	121.6797	120.7761	122.0034	120.7290	121.8560	120.8746	122.1212	118.8136	120.1112	120.7251	120.7385	121.6008	120.6967	121.8294	120.6564	121.6863	120.7809	121.9289	**116**
**C6**	134.9467	136.9717	138.0243	138.0376	139.4645	137.9675	140.0789	137.8990	139.7408	138.1064	140.4199	135.3034	137.3666	138.6127	138.3429	140.0727	138.2853	140.5728	138.2274	140.3897	138.3981	140.9095	**132**
**C7**	200.1830	205.0445	206.4951	207.5876	208.7013	207.4269	213.8365	207.2685	212.8952	207.7437	214.6933	204.6033	208.8622	210.0809	211.0822	211.8366	210.9380	216.6033	210.7982	215.3664	211.2259	217.0626	**196**
**C8**	62.4961	63.6480	64.3663	63.9871	64.9554	63.9622	65.4882	63.9393	65.2096	64.0139	66.1783	57.1221	58.8511	59.3074	59.6939	60.4822	59.6234	61.3132	59.5575	60.9413	59.7669	61.5421	**60**
**C9**	22.4232	22.7099	22.8836	22.9713	23.2313	22.9485	23.3154	22.9273	23.1815	22.9957	23.4231	19.1174	19.6122	19.6632	19.7712	20.0251	19.7540	20.2525	19.7389	20.1245	19.7907	20.6140	**15**
**C8`**	35.4509	36.2307	36.4509	36.4979	36.8360	36.4785	37.0927	36.4609	36.9944	36.5195	37.4940	30.7624	31.0177	31.3187	31.2114	31.4738	31.1980	31.6175	31.1846	31.6291	31.2246	31.6596	**31**
**H2**	8.7291	8.6745	8.6839	8.6307	8.5912	8.6340	8.6207	8.6372	8.6136	8.6271	8.6069	8.7394	8.5811	8.6311	8.4997	8.5161	8.5067	8.4889	8.5132	8.5182	8.4920	8.4934	**8.07**
**H3**	7.3570	7.4984	7.5860	7.5429	7.6585	7.5407	7.6805	7.5383	7.6697	7.5447	7.6928	7.3444	7.4827	7.5699	7.5283	7.6473	7.5259	7.6672	7.5234	7.6563	7.5302	7.6796	**7.31**
**H5**	7.2043	7.3984	7.4865	7.4833	7.6061	7.4784	7.6287	7.4735	7.6162	7.4877	7.6494	7.1957	7.3777	7.4663	7.4563	7.5822	7.4518	7.5978	7.4472	7.5841	7.4603	7.6170	**7.31**
**H6**	8.1786	8.3833	8.4552	8.4878	8.5945	8.4818	8.6451	8.4757	8.6289	8.4931	8.6774	7.9635	8.1395	8.2598	8.2257	8.3971	8.2217	8.4076	8.2174	8.4040	8.2288	8.4520	**8.07**
**H8**	4.3167	4.4108	4.4199	4.5072	4.5534	4.5008	4.6039	4.4943	4.6020	4.5131	4.6265	4.4991	4.6448	4.7045	4.7095	4.8253	4.7065	4.8443	4.7033	4.8349	4.7119	4.8737	**5.09**
**H(9A-9C)**	1.3125	1.3212	1.3067	1.3099	1.2608	1.3109	1.2798	1.3118	1.2649	1.3088	1.2919	1.3963	1.4224	1.4195	1.4330	1.4094	1.4328	1.4284	1.4324	1.4298	1.4329	1.4515	**1.6**
**H(8`A-8`C)**	2.4651	2.3989	2.3727	2.3791	2.3574	2.3796	2.3472	2.3802	2.3450	2.3785	2.3301	2.8767	2.7128	2.6773	2.6349	2.5897	2.6409	2.5049	2.6468	2.5284	2.6289	2.4711	**2.81**
**F4**	-113.4353	-114.1433	-116.7497	-113.9892	-118.1064	-114.0013	-115.9498	-114.0142	-116.2896	-113.9770	-115.6973	-113.3277	-114.5966	-117.0322	-114.7673	-118.5219	-114.7530	-116.8851	-114.7415	-117.0489	-114.7824	-116.5622	**-102.1**

[a] methyl protons shifts are averaged

[b] according to [9]

Table S9. Cont. VI

Nuclei	R											S											Exp. [b]
	TPSS																						
	Gas	$CHCl_3$ (PCM)	$CHCl_3$ (SMD)	DMSO (PCM)	DMSO (SMD)	CH_3OH (PCM)	CH_3OH (SMD)	C_2H_5OH (PCM)	C_2H_5OH (SMD)	H_2O (PCM)	H_2O (SMD)	Gas	$CHCl_3$ (PCM)	$CHCl_3$ (SMD)	DMSO (PCM)	DMSO (SMD)	CH_3OH (PCM)	CH_3OH (SMD)	C_2H_5OH (PCM)	C_2H_5OH (SMD)	H_2O (PCM)	H_2O (SMD)	
**C1**	132.3080	132.7323	133.5947	132.9917	134.6209	132.9794	133.9174	132.9795	133.4835	133.0145	134.1627	130.9473	132.0464	132.8321	132.7165	134.1527	132.6760	133.8514	132.6492	133.7648	132.7685	133.9904	**129**
**C2**	133.0997	133.0157	133.6822	132.9050	133.5045	132.9199	133.9437	132.9461	133.4500	132.9010	133.9167	132.3746	132.5433	133.1843	132.5871	133.2524	132.5896	133.7520	132.6038	133.7197	132.5960	133.7438	**132**
**C3**	116.6148	117.2984	117.9237	117.5959	118.2555	117.5862	118.5980	117.5876	118.0301	117.6152	118.6571	116.6745	117.2824	117.8745	117.5564	118.2099	117.5476	118.4658	117.5497	118.7785	117.5754	118.5352	**116**
**C4**	170.6841	171.5811	172.3374	172.0858	172.8432	172.0623	173.5294	172.0498	172.7101	172.1186	173.6596	170.9980	171.6247	172.4068	171.9513	172.7573	171.9401	173.2076	171.9397	172.7606	171.9719	173.3439	**167**
**C5**	114.5838	115.8370	116.5194	116.5569	117.3608	116.5197	117.6964	116.4938	116.9319	116.6029	117.8309	114.7542	115.8493	116.5202	116.4562	117.2683	116.4268	117.4654	116.4086	117.6460	116.4943	117.6013	**116**
**C6**	129.6014	131.4435	132.6218	132.4972	133.9868	132.4389	134.5452	132.3920	132.8937	132.5630	134.9193	129.9037	131.8399	133.1059	132.8246	134.4809	132.7742	135.0675	132.7346	133.8495	132.8818	135.3960	**132**
**C7**	196.4615	200.8812	202.3135	203.2587	204.2943	203.1209	208.8163	202.9947	203.7788	203.4005	209.6447	201.0290	204.6160	205.9348	206.6362	207.5175	206.5206	211.4415	206.4167	206.9790	206.7570	211.9588	**196**
**C8**	65.1158	66.2120	66.9967	66.5823	67.5160	66.5694	67.7566	66.5676	66.8060	66.6049	68.5367	60.1278	61.8831	62.5632	62.8019	63.6923	62.7474	64.2607	62.7059	64.3460	62.8662	64.4976	**60**
**C9**	23.9382	24.2126	24.5053	24.5049	24.8006	24.4912	24.8817	24.4888	24.5589	24.5284	25.0320	20.6506	21.2665	21.4915	21.5390	21.8543	21.5290	22.1455	21.5306	23.4796	21.5593	22.5571	**15**
**C8`**	37.5256	38.2274	38.5539	38.5318	38.8578	38.5228	39.1217	38.5251	38.6513	38.5508	39.5382	33.2385	33.3352	33.6679	33.4955	33.7511	33.4908	33.9306	33.4973	35.3565	33.5099	33.9584	**31**
**H2**	8.5053	8.4506	8.4858	8.4146	8.4078	8.4174	8.4634	8.4207	8.2608	8.4123	8.4454	8.5333	8.3896	8.4302	8.3110	8.3267	8.3165	8.3314	8.3222	8.4330	8.3060	8.3282	**8.07**
**H3**	7.1662	7.3060	7.4014	7.3539	7.4751	7.3516	7.4934	7.3498	7.1856	7.3564	7.5050	7.1476	7.2856	7.3782	7.3357	7.4586	7.3331	7.4748	7.3311	7.4493	7.3384	7.4865	**7.31**
**H5**	7.0222	7.2106	7.3064	7.2978	7.4257	7.2928	7.4508	7.2885	7.1240	7.3028	7.4683	7.0178	7.1891	7.2832	7.2681	7.3973	7.2637	7.4087	7.2598	7.3786	7.2727	7.4263	**7.31**
**H6**	7.9512	8.1457	8.2359	8.2495	8.3724	8.2435	8.4400	8.2381	8.0774	8.2555	8.4692	7.7288	7.9081	8.0237	7.9982	8.1671	7.9933	8.1988	7.9889	8.1022	8.0030	8.2393	**8.07**
**H8**	4.4709	4.5419	4.5602	4.6283	4.6855	4.6222	4.7477	4.6168	4.4417	4.6345	4.7669	4.5859	4.7329	4.7881	4.8006	4.9128	4.7971	4.9372	4.7941	4.9314	4.8041	4.9650	**5.09**
**H(9A-9C)**	1.3567	1.3679	1.3676	1.3629	1.3311	1.3636	1.3485	1.3649	1.1767	1.3626	1.3622	1.4392	1.4842	1.4875	1.5034	1.4900	1.5025	1.5193	1.5021	1.6640	1.5046	1.5408	**1.6**
**H(8`A-8`C)**	2.5215	2.4527	2.4256	2.4341	2.4118	2.4348	2.3810	2.4361	2.2522	2.4338	2.3643	2.9653	2.7995	2.7626	2.7257	2.6817	2.7310	2.6015	2.7368	2.8894	2.7208	2.5649	**2.81**
**F4**	-124.7102	-125.6695	-128.6489	-125.5433	-130.3088	-125.5555	-127.4659	-125.5685	-127.0790	-125.5312	-127.1658	-124.6252	-126.1659	-129.1136	-126.4163	-130.9115	-126.4042	-128.7170	-126.3932	-124.8559	-126.4283	-128.3327	**-102.1**
Nuclei	VSXC																						
**C1**	130.0829	130.4045	131.1400	130.4934	131.6615	130.4929	131.1365	130.4902	131.2057	130.4951	131.2579	129.9454	130.6445	131.8488	131.2031	132.5520	131.1505	132.4267	131.1004	132.2788	131.2589	132.5715	**129**
**C2**	131.4719	131.5765	132.0332	131.5476	131.9755	131.5536	132.4149	131.5574	132.3103	131.5434	132.5007	131.5035	131.5629	132.0835	131.4748	131.9974	131.4854	132.4275	131.4940	132.3584	131.4659	132.4408	**132**
**C3**	115.0253	115.9281	116.4781	116.3182	116.9617	116.2986	117.2532	116.2767	117.1666	116.3381	117.3602	115.1018	115.8625	116.3437	116.1552	116.7869	116.1441	116.9700	116.1304	116.9294	116.1665	117.0707	**116**
**C4**	168.4085	169.4140	170.1094	169.9432	170.7388	169.9139	171.2602	169.8824	171.1101	169.9727	171.4432	168.2644	169.1345	169.7602	169.5550	170.3438	169.5352	170.7332	169.5129	170.6261	169.5745	170.8988	**167**
**C5**	112.9381	114.3706	114.9696	115.1434	115.9400	115.0989	116.2089	115.0522	116.0882	115.1874	116.3893	112.8384	114.1341	114.6817	114.8040	115.5837	114.7687	115.7340	114.7306	115.6555	114.8386	115.8930	**116**
**C6**	129.7735	131.5896	132.6289	132.6050	134.1264	132.5458	134.6888	132.4845	134.4417	132.6624	135.0466	129.7921	131.8114	132.8618	132.8875	134.4094	132.8259	134.9250	132.7615	134.6909	132.9470	135.2245	**132**
**C7**	190.4964	194.5948	195.8890	196.6716	197.5040	196.5484	201.3041	196.4236	200.4899	196.7897	202.0685	192.4201	196.4292	197.9570	198.6054	199.5513	198.4654	203.3086	198.3255	202.3644	198.7417	203.9258	**196**
**C8**	71.0293	71.8644	72.4464	72.1610	72.9614	72.1470	73.3160	72.1307	73.2068	72.1762	73.9137	63.2954	64.4627	65.0975	65.1337	65.9999	65.0894	66.5041	65.0447	66.3060	65.1788	66.9366	**60**
**C9**	27.6811	28.6013	28.9238	29.2582	29.7109	29.2182	30.0806	29.1763	29.9393	29.2980	30.1347	28.6031	28.7198	28.7057	28.8776	29.0104	28.8658	28.9877	28.8527	28.9354	28.8913	29.2920	**15**
**C8`**	40.4536	40.9423	41.0239	41.1700	41.3482	41.1601	41.3711	41.1480	41.3482	41.1808	41.7912	35.6942	36.3333	36.3426	36.5410	36.8365	36.5434	36.8289	36.5411	36.8765	36.5384	37.0830	**31**
**H2**	8.7013	8.6318	8.6383	8.5965	8.5956	8.5987	8.6008	8.6009	8.6015	8.5945	8.6002	8.6698	8.5714	8.5462	8.4980	8.4914	8.5044	8.4746	8.5105	8.4951	8.4915	8.4705	**8.07**
**H3**	7.2076	7.3421	7.4286	7.3891	7.5038	7.3867	7.5214	7.3843	7.5128	7.3914	7.5352	7.2377	7.3761	7.4621	7.4257	7.5415	7.4230	7.5574	7.4205	7.5480	7.4281	7.5730	**7.31**
**H5**	7.1085	7.2957	7.3836	7.3798	7.5056	7.3750	7.5203	7.3702	7.5095	7.3844	7.5443	7.1230	7.2984	7.3840	7.3775	7.4993	7.3729	7.5080	7.3684	7.4968	7.3817	7.5256	**7.31**
**H6**	8.1799	8.3473	8.4451	8.4415	8.5921	8.4354	8.6432	8.4296	8.6230	8.4472	8.6738	7.8041	7.9933	8.0857	8.0898	8.2480	8.0846	8.2771	8.0794	8.2615	8.0945	8.3072	**8.07**
**H8**	4.3876	4.4583	4.4912	4.5227	4.5993	4.5183	4.6338	4.5141	4.6138	4.5269	4.6595	4.6175	4.7683	4.8270	4.8563	4.9599	4.8507	5.0035	4.8453	4.9843	4.8616	5.0218	**5.09**
**H(9A-9C)**	1.5961	1.6138	1.6048	1.6171	1.6025	1.6170	1.6242	1.6171	1.6201	1.6172	1.6378	1.7731	1.7717	1.7506	1.7772	1.7569	1.7769	1.7723	1.7766	1.7683	1.7776	1.7889	**1.6**
**H(8`A-8`C)**	2.6858	2.6354	2.6002	2.6143	2.5757	2.6156	2.5544	2.6170	2.5572	2.6129	2.5289	2.8671	2.7641	2.7059	2.7132	2.6678	2.7172	2.6228	2.7210	2.6423	2.7093	2.5927	**2.81**
**F4**	-125.4361	-126.1598	-129.1323	-125.9174	-130.4800	-125.9405	-127.8419	-125.9540	-128.3085	-125.8941	-127.4076	-125.7847	-126.8972	-129.9759	-126.9215	-131.4340	-126.9246	-129.2349	-126.9188	-129.5967	-126.9184	-128.8423	**-102.1**
Nuclei	wB97																						
**C1**	136.3743	136.5643	137.5508	136.6805	138.3186	136.6682	137.1758	136.6563	137.6400	136.6935	137.3306	135.5496	136.5810	137.2430	137.2393	138.6376	137.1701	138.5360	137.1091	138.2897	137.3170	138.7211	**129**
**C2**	140.2891	140.1704	140.7301	140.0418	140.6111	140.0495	141.1373	140.0566	141.0098	140.0348	141.1780	140.0931	140.0448	140.6006	139.9574	140.5644	139.9621	140.9964	139.9662	140.9089	139.9528	141.0199	**132**
**C3**	121.6439	122.2148	122.7714	122.4318	123.1041	122.4188	123.2000	122.4051	123.1728	122.4445	123.2668	121.6716	122.2096	122.7417	122.4350	123.1020	122.4187	123.2279	122.4023	123.1663	122.4515	123.2949	**116**
**C4**	174.2577	174.9242	175.5185	175.3214	175.9512	175.2956	176.5447	175.2691	176.3511	175.3461	176.6908	174.3838	174.8494	175.5012	175.0939	175.7994	175.0823	176.1349	175.0683	176.0692	175.1036	176.2686	**167**
**C5**	119.4831	120.7345	121.3443	121.4210	122.2577	121.3777	122.3482	121.3337	122.2709	121.4624	122.5094	119.5892	120.7784	121.3962	121.3974	122.2486	121.3581	122.3057	121.3179	122.2298	121.4354	122.4485	**116**
**C6**	136.8221	138.8055	139.9029	139.8974	141.4938	139.8285	142.2089	139.7589	141.7730	139.9631	142.5634	137.0806	139.0577	140.2748	140.0174	141.7669	139.9692	142.1277	139.9168	141.9973	140.0596	142.4749	**132**
**C7**	200.5554	205.5196	206.9736	208.1930	208.8203	208.0253	213.7590	207.8570	212.9387	208.3529	214.6659	204.2854	208.7429	210.1036	211.2165	211.7853	211.0496	216.9049	210.8871	215.6715	211.3809	217.6015	**196**
**C8**	61.2286	62.1722	62.8269	62.5542	63.4515	62.5338	63.8040	62.5119	63.7336	62.5735	64.4107	55.7397	57.1823	57.6139	58.0157	58.7514	57.9530	59.4666	57.8934	59.1612	58.0807	59.7826	**60**
**C9**	22.0700	22.2380	22.4228	22.4373	22.8248	22.4194	22.7743	22.4016	22.6602	22.4557	22.8715	19.5465	19.7727	19.7821	19.8738	20.1420	19.8648	20.1501	19.8559	20.0913	19.8837	20.5181	**15**
**C8`**	35.1652	35.7649	35.9908	36.0743	36.3679	36.0567	36.5472	36.0377	36.5002	36.0907	36.9598	30.5263	30.8779	31.2072	31.0800	31.3808	31.0803	31.3789	31.0759	31.4642	31.0751	31.4895	**31**
**H2**	8.8384	8.7915	8.8061	8.7534	8.7565	8.7561	8.8198	8.7586	8.7788	8.7508	8.8177	8.844383	8.699283	8.746017	8.604942	8.634042	8.613558	8.589467	8.621308	8.622242	8.595417	8.591583	**8.07**
**H3**	7.4415	7.5770	7.6646	7.6196	7.7405	7.6173	7.7555	7.6149	7.7454	7.6217	7.7699	7.4525	7.5863	7.6739	7.6277	7.7500	7.6258	7.7576	7.6236	7.7514	7.6295	7.7730	**7.31**
**H5**	7.3028	7.4922	7.5805	7.5754	7.7036	7.5705	7.7161	7.5655	7.7041	7.5800	7.7398	7.3123	7.4932	7.5821	7.5675	7.6967	7.5632	7.7017	7.5588	7.6926	7.5714	7.7244	**7.31**
**H6**	8.2363	8.4473	8.5401	8.5626	8.6990	8.5558	8.7730	8.5487	8.7240	8.5691	8.8069	8.0771	8.2684	8.3886	8.3453	8.5299	8.3432	8.5210	8.3402	8.5241	8.3465	8.5617	**8.07**
**H8**	4.0794	4.2013	4.2289	4.3015	4.3612	4.2941	4.4167	4.2867	4.3964	4.3088	4.4426	4.2843	4.4417	4.5098	4.5068	4.6254	4.5039	4.6339	4.5005	4.6294	4.5091	4.6610	**5.09**
**H(9A-9C)**	1.3373	1.3502	1.3355	1.3434	1.3206	1.3441	1.3538	1.3446	1.3316	1.3428	1.3678	1.4052	1.4218	1.4124	1.4283	1.4126	1.4285	1.4264	1.4284	1.4276	1.4279	1.4449	**1.6**
**H(8`A-8`C)**	2.4600	2.4029	2.3721	2.3774	2.3594	2.3781	2.3295	2.3790	2.3441	2.3770	2.3180	2.8246	2.6620	2.6344	2.5859	2.5485	2.5907	2.4603	2.5952	2.4869	2.5812	2.4374	**2.81**
**F4**	-107.7949	-109.3040	-111.6571	-109.5462	-113.1227	-109.5381	-111.1741	-109.5298	-111.6029	-109.5542	-110.9940	-107.6757	-109.6708	-111.8955	-110.2470	-113.6052	-110.2079	-112.3236	-110.1720	-112.3996	-110.2883	-112.1601	**-102.1**
Nuclei	wB97X																						
**C1**	137.8029	138.0540	139.0211	138.1992	139.8961	138.1870	138.7316	138.1755	139.5920	138.2141	139.6814	136.9016	138.0110	138.6568	138.6712	140.0912	138.6060	140.0704	138.5482	139.7173	138.7443	140.1871	**129**
**C2**	140.7055	140.6032	141.1665	140.4847	141.0596	140.4945	141.6070	140.5037	141.4061	140.4762	141.5856	140.5526	140.5103	141.0764	140.4186	141.0232	140.4259	141.4852	140.4318	141.3445	140.4125	141.4959	**132**
**C3**	122.3475	122.9417	123.4842	123.1709	123.8319	123.1596	123.9911	123.1475	123.9336	123.1825	124.0993	122.3753	122.9427	123.4616	123.1718	123.8332	123.1584	124.0179	123.1444	123.8893	123.1862	124.0701	**116**
**C4**	174.9165	175.6237	176.2197	176.0405	176.6624	176.0165	177.3046	175.9916	177.0258	176.0640	177.3657	175.0408	175.5562	176.2009	175.8192	176.5285	175.8083	176.9078	175.7954	176.7811	175.8288	177.0410	**167**
**C5**	120.1726	121.4443	122.0434	122.1523	122.9687	122.1105	123.1411	122.0679	123.0163	122.1931	123.3151	120.2954	121.5031	122.1147	122.1275	122.9742	122.0916	123.0943	122.0548	122.9461	122.1629	123.2277	**116**
**C6**	137.1768	139.1619	140.2691	140.2457	141.8067	140.1813	142.5581	140.1156	141.8911	140.3073	142.5689	137.4753	139.4432	140.6731	140.4268	142.1500	140.3797	142.5033	140.3282	142.3574	140.4684	142.8687	**132**
**C7**	202.1768	207.1982	208.6196	209.9149	210.5679	209.7483	215.5392	209.5818	215.1279	210.0748	217.1331	206.1849	210.6989	211.9868	213.1284	213.7439	212.9665	218.9548	212.8080	217.5873	213.2885	219.5702	**196**
**C8**	62.2638	63.2785	63.9563	63.6403	64.5673	63.6254	64.9558	63.6092	64.7914	63.6554	65.6105	56.7269	58.2485	58.7010	59.1431	59.8693	59.0782	60.6326	59.0159	60.2657	59.2102	60.9139	**60**
**C9**	22.2545	22.4780	22.6788	22.6724	23.0493	22.6560	23.0235	22.6394	22.7994	22.6898	23.0038	19.5952	19.8548	19.8730	19.9815	20.2271	19.9737	20.2590	19.9655	20.1660	19.9902	20.6043	**15**
**C8`**	35.4261	36.0803	36.3153	36.4017	36.7065	36.3882	36.9314	36.3733	36.7947	36.4152	37.3166	30.7265	31.0292	31.3557	31.2646	31.5524	31.2617	31.6100	31.2549	31.6389	31.2646	31.7241	**31**
**H2**	8.8045	8.7552	8.7713	8.7178	8.7132	8.7209	8.7796	8.7237	8.6982	8.7149	8.6978	8.8202	8.6677	8.7159	8.5736	8.5991	8.5821	8.5540	8.5897	8.5893	8.5645	8.5574	**8.07**
**H3**	7.4131	7.5511	7.6385	7.5956	7.7147	7.5935	7.7298	7.5914	7.7207	7.5978	7.7443	7.4226	7.5575	7.6444	7.6018	7.7223	7.5997	7.7328	7.5974	7.7240	7.6039	7.7460	**7.31**
**H5**	7.2774	7.4679	7.5557	7.5537	7.6786	7.5490	7.6961	7.5442	7.6777	7.5583	7.7129	7.2894	7.4695	7.5577	7.5457	7.6732	7.5418	7.6791	7.5378	7.6674	7.5496	7.7001	**7.31**
**H6**	8.2021	8.4135	8.5069	8.5295	8.6573	8.5233	8.7307	8.5170	8.6684	8.5355	8.7254	8.0367	8.2260	8.3460	8.3104	8.4911	8.3074	8.4771	8.3036	8.4843	8.3126	8.5189	**8.07**
**H8**	4.1390	4.2501	4.2742	4.3513	4.4040	4.3440	4.4602	4.3368	4.4505	4.3585	4.4898	4.3255	4.4860	4.5491	4.5527	4.6724	4.5495	4.6828	4.5459	4.6757	4.5555	4.7093	**5.09**
**H(9A-9C)**	1.3232	1.3360	1.3236	1.3281	1.3024	1.3291	1.3388	1.3299	1.2880	1.3271	1.3094	1.3934	1.4128	1.4075	1.4242	1.4050	1.4244	1.4147	1.4244	1.4169	1.4237	1.4340	**1.6**
**H(8`A-8`C)**	2.4671	2.4104	2.3794	2.3869	2.3692	2.3875	2.3398	2.3882	2.3568	2.3865	2.3429	2.8470	2.6868	2.6544	2.6087	2.5717	2.6137	2.4834	2.6186	2.5074	2.6037	2.4553	**2.81**
**F4**	-110.1038	-111.3687	-113.7560	-111.4876	-115.1632	-111.4851	-113.1412	-111.4826	-113.7242	-111.4904	-113.2461	-110.0121	-111.7897	-114.0512	-112.2600	-115.6866	-112.2271	-114.3918	-112.1969	-114.4583	-112.2953	-114.1696	**-102.1**

[a] methyl protons shifts are averaged
[b] according to [9]

Table S9. Cont. VII

Nuclei	R										
	X3LYP										
	Gas	$CHCl_3$ (PCM)	$CHCl_3$ (SMD)	DMSO (PCM)	DMSO (SMD)	CH_3OH (PCM)	CH_3OH (SMD)	C_2H_5OH (PCM)	C_2H_5OH (SMD)	H_2O (PCM)	H_2O (SMD)
**C1**	139.3564	139.8444	140.8482	140.1134	142.0566	140.0909	141.3658	140.0679	141.5227	140.1411	141.6467
**C2**	139.9182	139.8474	140.4108	139.6953	140.1921	139.7116	140.6326	139.7238	140.5055	139.6831	140.6144
**C3**	122.0092	122.6988	123.2345	122.9633	123.5748	122.9518	123.8529	122.9370	123.8097	122.9783	123.9078
**C4**	176.1825	177.0882	177.7250	177.5818	178.2391	177.5544	178.8901	177.5242	178.7610	177.6114	179.0087
**C5**	119.8141	121.1439	121.7439	121.8710	122.6356	121.8294	122.8875	121.7849	122.7972	121.9143	123.0321
**C6**	136.1581	138.1629	139.2158	139.2637	140.6897	139.2042	141.2883	139.1404	140.9981	139.3229	141.6419
**C7**	202.8571	207.9768	209.5518	210.7426	211.8371	210.5640	217.1792	210.3863	216.3022	210.9197	218.1127
**C8**	65.0592	66.2153	66.9018	66.5407	67.5099	66.5316	67.8451	66.5179	67.6413	66.5516	68.6175
**C9**	23.0316	23.3183	23.4327	23.5997	23.8343	23.5889	23.8506	23.5740	23.7548	23.6127	23.9870
**C8`**	36.3944	37.1898	37.4326	37.5008	37.8523	37.4859	38.0604	37.4681	38.0401	37.5189	38.4847
**H2**	8.6524	8.5918	8.6063	8.5511	8.5135	8.5549	8.5499	8.5579	8.5426	8.5473	8.5316
**H3**	7.3213	7.4551	7.5439	7.5005	7.6121	7.4985	7.6290	7.4960	7.6209	7.5023	7.6416
**H5**	7.1716	7.3592	7.4485	7.4464	7.5662	7.4416	7.5856	7.4362	7.5755	7.4510	7.6064
**H6**	8.0809	8.2845	8.3663	8.3962	8.5098	8.3898	8.5716	8.3828	8.5557	8.4024	8.5971
**H8**	4.3443	4.4331	4.4575	4.5383	4.5907	4.5308	4.6494	4.5230	4.6418	4.5456	4.6641
**H(9A-9C)**	1.3227	1.3314	1.3179	1.3217	1.2790	1.3235	1.2915	1.3245	1.2806	1.3199	1.3039
**H(8`A-8`C)**	2.5170	2.4557	2.4302	2.4400	2.4194	2.4409	2.4037	2.4412	2.4048	2.4393	2.3899
**F4**	-123.9446	-125.0794	-127.9228	-125.1110	-129.5643	-125.1134	-127.2940	-125.1155	-127.6376	-125.1089	-127.0904

Nuclei	S											Exp. [b]
	X3LYP											
	Gas	$CHCl_3$ (PCM)	$CHCl_3$ (SMD)	DMSO (PCM)	DMSO (SMD)	CH_3OH (PCM)	CH_3OH (SMD)	C_2H_5OH (PCM)	C_2H_5OH (SMD)	H_2O (PCM)	H_2O (SMD)	
**C1**	137.9117	139.2562	139.9366	139.9579	141.5273	139.9149	141.3110	139.8710	141.2013	140.0058	141.3487	**129**
**C2**	139.2169	139.3848	139.9238	139.3913	140.0004	139.3937	140.5425	139.3932	140.4245	139.3932	140.5430	**132**
**C3**	122.1353	122.7433	123.2197	122.9818	123.5816	122.9719	123.7914	122.9587	123.7342	122.9949	123.8481	**116**
**C4**	176.5499	177.1393	177.8065	177.4289	178.1522	177.4164	178.5567	177.4002	178.4628	177.4439	178.7115	**167**
**C5**	119.9992	121.2093	121.7793	121.8153	122.5836	121.7831	122.7292	121.7474	122.6490	121.8494	122.8641	**116**
**C6**	136.5146	138.5767	139.8645	139.6151	141.2627	139.5588	141.7748	139.4982	141.5895	139.6709	142.1688	**132**
**C7**	208.1208	212.3136	213.4589	214.5395	215.3412	214.4092	220.0232	214.2763	218.9420	214.6672	220.5368	**196**
**C8**	59.3212	61.3577	61.9949	62.3629	63.2348	62.3022	63.9246	62.2400	63.6812	62.4263	64.1714	**60**
**C9**	19.5775	20.0461	20.2181	20.2904	20.5151	20.2799	20.6827	20.2663	20.6108	20.3042	21.0438	**15**
**C8`**	31.9991	32.1508	32.4082	32.3228	32.5612	32.3139	32.6685	32.3014	32.6855	32.3346	32.7610	**31**
**H2**	8.6901	8.5204	8.5632	8.4372	8.4423	8.4428	8.4297	8.4475	8.4451	8.4317	8.4405	**8.07**
**H3**	7.3067	7.4391	7.5255	7.4863	7.6016	7.4841	7.6164	7.4812	7.6087	7.4884	7.6303	**7.31**
**H5**	7.1634	7.3365	7.4251	7.4152	7.5364	7.4110	7.5463	7.4062	7.5365	7.4192	7.5665	**7.31**
**H6**	7.8813	8.0490	8.1656	8.1364	8.2978	8.1318	8.3147	8.1265	8.3041	8.1406	8.3622	**8.07**
**H8**	4.4903	4.6483	4.7010	4.7147	4.8269	4.7114	4.8459	4.7075	4.8333	4.7177	4.8776	**5.09**
**H(9A-9C)**	1.3991	1.4351	1.4417	1.4560	1.4332	1.4550	1.4533	1.4534	1.4521	1.4569	1.4778	**1.6**
**H(8`A-8`C)**	2.9813	2.8071	2.7639	2.7278	2.6788	2.7332	2.5951	2.7379	2.6158	2.7225	2.5651	**2.81**
**F4**	-123.6606	-125.4316	-128.1731	-125.8293	-129.9814	-125.8093	-128.2500	-125.7897	-128.4739	-125.8494	-127.9006	**-102.1**

[a] methyl protons shifts are averaged

[b] according to [9]

Table S10. Theoretical chemical shifts (in ppm) of *(R,S)*-flephedrone calculated with HF and selected functionals combined with 6-311++G** basis set[a]

Nuclei	R											S											Exp. [b]
	B3LYP																						
	Gas	$CHCl_3$ (PCM)	$CHCl_3$ (SMD)	DMSO (PCM)	DMSO (SMD)	CH_3OH (PCM)	CH_3OH (SMD)	C_2H_5OH (PCM)	C_2H_5OH (SMD)	H_2O (PCM)	H_2O (SMD)	Gas	$CHCl_3$ (PCM)	$CHCl_3$ (SMD)	DMSO (PCM)	DMSO (SMD)	CH_3OH (PCM)	CH_3OH (SMD)	C_2H_5OH (PCM)	C_2H_5OH (SMD)	H_2O (PCM)	H_2O (SMD)	
**C1**	139.3516	139.8992	140.9781	140.1863	142.0735	140.1611	141.5120	140.1358	141.5012	140.2082	141.7819	137.8738	139.3186	139.9816	139.9577	141.5554	139.9093	141.4183	139.8637	141.2810	140.0057	141.4292	**130.99**
**C2**	139.7181	139.6929	140.2565	139.4633	139.9708	139.4799	140.4199	139.4943	140.2834	139.4440	140.3925	139.0099	139.2247	139.7699	139.1806	139.8028	139.1780	140.3740	139.1755	140.2362	139.1821	140.3704	**133.34**
**C3**	121.9147	122.6511	123.2036	122.8744	123.4797	122.8576	123.7880	122.8405	123.7263	122.8887	123.8478	122.0546	122.6999	123.1853	122.8846	123.4913	122.8701	123.7328	122.8554	123.6526	122.8966	123.7867	**117.55**
**C4**	176.1996	177.1548	177.8016	177.6113	178.2556	177.5774	178.9142	177.5440	178.7922	177.6426	179.0429	176.5723	177.1853	177.8667	177.4190	178.1469	177.4023	178.5696	177.3850	178.4546	177.4322	178.7245	**168.13**
**C5**	119.7381	121.1100	121.7159	121.7876	122.5392	121.7417	122.8148	121.6959	122.7064	121.8299	122.9605	119.9339	121.1749	121.7562	121.7216	122.4902	121.6855	122.6663	121.6489	122.5622	121.7540	122.7986	**117.55**
**C6**	135.8982	137.9272	138.9534	138.9372	140.4067	138.8712	141.0202	138.8048	140.7434	138.9983	141.3636	136.2778	138.3528	139.6434	139.3323	140.9568	139.2716	141.4709	139.2095	141.2669	139.3872	141.8771	**133.34**
**C7**	202.8768	208.0691	209.7045	210.9565	211.8939	210.7561	217.3250	210.5621	216.3636	211.1520	218.3042	208.1087	212.3579	213.4837	214.5278	215.3474	214.3946	220.1153	214.2618	218.9963	214.6532	220.5944	**195.87**
**C8**	65.2601	66.4393	67.0844	66.6651	67.6873	66.6448	68.1168	66.6260	67.8105	66.6832	68.8556	59.4742	61.6544	62.2820	62.6180	63.4923	62.5505	64.2487	62.4848	63.9791	62.6825	64.4890	**60.75**
**C9**	22.9937	23.3369	23.4371	23.5103	23.7870	23.5040	23.8454	23.4947	23.7278	23.5117	23.9610	19.5432	20.0549	20.2357	20.2537	20.4684	20.2366	20.6702	20.2197	20.5736	20.2683	21.0311	**16.39**
**C8`**	36.5076	37.3387	37.5483	37.6307	37.9535	37.5944	38.1848	37.5637	38.1616	37.6692	38.6153	32.1915	32.3577	32.6229	32.4742	32.7092	32.4611	32.8316	32.4474	32.8288	32.4845	32.9175	**32**
**H2**	8.6288	8.5708	8.5739	8.5193	8.4862	8.5229	8.5099	8.5264	8.5151	8.5156	8.4958	8.6701	8.4976	8.5384	8.4115	8.4156	8.4168	8.3988	8.4218	8.4120	8.4060	8.4143	**7.971**
**H3**	7.3119	7.4474	7.5313	7.4874	7.6000	7.4852	7.6152	7.4829	7.6051	7.4891	7.6305	7.2978	7.4323	7.5140	7.4748	7.5907	7.4723	7.6048	7.4697	7.5943	7.4770	7.6211	**7.22**
**H5**	7.1614	7.3510	7.4352	7.4344	7.5536	7.4291	7.5704	7.4238	7.5589	7.4393	7.5939	7.1540	7.3281	7.4120	7.4019	7.5234	7.3974	7.5322	7.3928	7.5197	7.4059	7.5549	**7.22**
**H6**	8.0695	8.2736	8.3471	8.3829	8.4942	8.3751	8.5470	8.3677	8.5388	8.3904	8.5777	7.8720	8.0370	8.1480	8.1191	8.2781	8.1143	8.2908	8.1093	8.2781	8.1233	8.3419	**7.971**
**H8**	4.3623	4.4552	4.4783	4.5600	4.6044	4.5533	4.6526	4.5464	4.6504	4.5660	4.6741	4.4999	4.6578	4.7050	4.7188	4.8309	4.7153	4.8477	4.7118	4.8326	4.7217	4.8820	**5.2**
**H(9A-9C)**	1.3154	1.3242	1.3019	1.3025	1.2691	1.3041	1.2806	1.3055	1.2685	1.3007	1.2933	1.3888	1.4276	1.4302	1.4455	1.4215	1.4441	1.4397	1.4427	1.4360	1.4466	1.4683	**1.611**
**H(8`A-8`C)**	2.5071	2.4491	2.4216	2.4281	2.4102	2.4290	2.3990	2.4298	2.3931	2.4269	2.3861	2.9770	2.8044	2.7564	2.7204	2.6721	2.7257	2.5872	2.7307	2.6042	2.7151	2.5595	**2.82**
Nuclei	**B3P86**																						
**C1**	136.8453	137.2808	138.3582	137.5043	139.4519	137.5074	138.8537	137.4898	139.1351	137.5460	139.1283	135.6233	136.9063	137.5597	137.4853	138.9540	137.4549	138.8749	137.4076	138.7415	137.5634	138.9677	**130.99**
**C2**	138.7919	138.7416	139.3998	138.5747	139.1636	138.6075	139.5971	138.6182	139.5645	138.5873	139.5816	138.1598	138.3322	138.9376	138.3113	138.9809	138.3306	139.5077	138.3287	139.4734	138.3375	139.5260	**133.34**
**C3**	121.1158	121.8832	122.5099	122.1552	122.8745	122.1613	123.1693	122.1453	123.2080	122.1932	123.2475	121.2077	121.9036	122.4956	122.1573	122.8618	122.1638	123.0956	122.1483	123.1217	122.1950	123.1750	**117.55**
**C4**	173.6985	174.6092	175.2829	175.0662	175.7535	175.0598	176.4063	175.0315	176.3576	175.1159	176.5491	173.9907	174.6001	175.3439	174.8861	175.6462	174.8935	176.0480	174.8781	176.0672	174.9218	176.2106	**168.13**
**C5**	118.9511	120.3570	121.0389	121.0865	121.9545	121.0629	122.2291	121.0175	122.2236	121.1526	122.3983	119.0968	120.3750	121.0516	121.0069	121.8780	120.9911	122.0417	120.9530	122.0531	121.0653	122.1965	**117.55**
**C6**	134.9391	136.9571	138.0470	138.0317	139.4694	137.9871	140.0383	137.9202	139.8454	138.1179	140.4254	135.2870	137.3568	138.6630	138.3561	140.0839	138.3212	140.5742	138.2627	140.5163	138.4312	140.9668	**133.34**
**C7**	200.9937	205.8309	207.3690	208.3757	209.4937	208.2408	214.5962	208.0849	213.8337	208.5478	215.4998	205.5040	209.6504	210.9178	211.8502	212.6202	211.7370	217.2310	211.6033	216.1910	212.0035	217.7281	**195.87**
**C8**	63.6796	64.8464	65.6285	65.2068	66.1771	65.2071	66.5812	65.1854	66.4957	65.2505	67.3827	58.1084	59.9428	60.4781	60.8007	61.6333	60.7568	62.3872	60.6942	62.1777	60.8896	62.6693	**60.75**
**C9**	22.8065	23.0897	23.3077	23.3623	23.6359	23.3647	23.6593	23.3452	23.6707	23.4035	23.8216	19.4663	20.0187	20.1559	20.2089	20.4941	20.2153	20.6857	20.2003	20.7001	20.2472	21.1119	**16.39**
**C8`**	35.9286	36.7158	37.0084	37.0027	37.3475	37.0082	37.5596	36.9918	37.6189	37.0414	38.0311	31.3622	31.5731	31.9325	31.7646	32.0364	31.7748	32.1533	31.7622	32.2999	31.7969	32.2578	**32**
**H2**	8.6937	8.6392	8.6481	8.5947	8.5586	8.5992	8.5883	8.6018	8.5814	8.5915	8.5744	8.7028	8.5480	8.5959	8.4713	8.4868	8.4787	8.4611	8.4838	8.4913	8.4649	8.4716	**7.971**
**H3**	7.3323	7.4749	7.5633	7.5186	7.6361	7.5176	7.6523	7.5147	7.6476	7.5207	7.6690	7.3188	7.4600	7.5477	7.5057	7.6274	7.5044	7.6414	7.5013	7.6367	7.5080	7.6590	**7.22**
**H5**	7.1767	7.3724	7.4616	7.4573	7.5836	7.4536	7.6013	7.4480	7.5944	7.4620	7.6265	7.1655	7.3489	7.4382	7.4270	7.5553	7.4237	7.5640	7.4186	7.5573	7.4312	7.5880	**7.22**
**H6**	8.1523	8.3571	8.4270	8.4597	8.5681	8.4550	8.6211	8.4484	8.6073	8.4654	8.6536	7.9340	8.1076	8.2276	8.1948	8.3652	8.1917	8.3755	8.1865	8.3750	8.1986	8.4257	**7.971**
**H8**	4.3930	4.4836	4.4927	4.5762	4.6265	4.5711	4.6819	4.5642	4.6796	4.5824	4.7038	4.5540	4.6976	4.7569	4.7623	4.8783	4.7605	4.8928	4.7566	4.8883	4.7650	4.9288	**5.2**
**H(9A-9C)**	1.3335	1.3438	1.3286	1.3321	1.2867	1.3343	1.2965	1.3346	1.2886	1.3313	1.3143	1.4092	1.4428	1.4421	1.4554	1.4353	1.4561	1.4505	1.4549	1.4566	1.4559	1.4796	**1.611**
**H(8`A-8`C)**	2.4876	2.4212	2.3951	2.4005	2.3811	2.4023	2.3602	2.4024	2.3671	2.4001	2.3503	2.9176	2.7485	2.7146	2.6728	2.6287	2.6800	2.5349	2.6851	2.5651	2.6670	2.5072	**2.82**
Nuclei	**B3PW91**																						
**C1**	137.0994	137.5782	138.7234	137.8703	140.2084	137.8485	139.3905	137.8187	139.3095	137.8939	139.6388	135.8270	137.2800	137.7781	137.8580	139.3488	137.8089	139.3951	137.7573	139.1374	137.9141	139.4828	**130.99**
**C2**	138.8604	138.8385	139.3967	138.6503	139.5459	138.6706	139.6266	138.6815	139.5161	138.6320	139.6248	138.2843	138.4871	139.0226	138.4452	139.1209	138.4505	139.6147	138.4477	139.5068	138.4432	139.6376	**133.34**
**C3**	121.2185	122.0069	122.5742	122.2708	123.2963	122.2610	123.2495	122.2421	123.2072	122.2821	123.3409	121.3071	122.0247	122.5329	122.2639	122.9651	122.2553	123.1906	122.2380	123.1214	122.2739	123.2752	**117.55**
**C4**	173.7409	174.6858	175.2871	175.1369	176.1105	175.1138	176.3924	175.0819	176.3209	175.1612	176.5562	174.0199	174.6397	175.3121	174.9084	175.6627	174.9018	176.0329	174.8849	175.9623	174.9153	176.2007	**168.13**
**C5**	119.0599	120.4861	121.1030	121.2065	122.3683	121.1668	122.2995	121.1183	122.2138	121.2461	122.4777	119.2098	120.5084	121.1087	121.1148	121.9791	121.0849	122.1342	121.0458	122.0478	121.1449	122.2957	**117.55**
**C6**	134.8881	136.9286	137.8816	137.9622	139.7104	137.9061	139.9572	137.8415	139.7281	138.0174	140.3326	135.2770	137.3211	138.5843	138.3076	139.9907	138.2583	140.3962	138.1982	140.2701	138.3538	140.7941	**133.34**
**C7**	201.1247	206.1066	207.7353	208.8082	210.2420	208.6327	215.1374	208.4510	214.2009	208.9795	216.0833	205.6165	209.9651	211.0502	212.1644	212.9527	212.0348	217.7726	211.8977	216.5723	212.2928	218.2699	**195.87**
**C8**	63.4567	64.6178	65.2257	64.9342	66.2583	64.9202	66.4879	64.8987	66.1020	64.9510	67.2318	58.0320	59.9926	60.4452	60.8767	61.6836	60.8128	62.5182	60.7445	62.2018	60.9444	62.8000	**60.75**
**C9**	22.5160	22.8553	22.9353	23.0789	23.6687	23.0753	23.3953	23.0617	23.2786	23.0832	23.5418	19.1832	19.7035	19.7883	19.8973	20.1279	19.8867	20.3038	19.8683	20.2280	19.9102	20.7016	**16.39**
**C8`**	35.8834	36.7085	36.8598	36.9828	37.6339	36.9657	37.5129	36.9420	37.5231	37.0038	37.9753	31.4292	31.6510	31.9335	31.8297	32.1026	31.8260	32.1558	31.8123	32.2185	31.8333	32.2536	**32**
**H2**	8.6633	8.6108	8.6056	8.5605	8.5329	8.5652	8.5339	8.5693	8.5526	8.5562	8.5242	8.6835	8.5130	8.5718	8.4375	8.4524	8.4445	8.4188	8.4505	8.4482	8.4303	8.4283	**7.971**
**H3**	7.3301	7.4703	7.5577	7.5143	7.6458	7.5126	7.6483	7.5105	7.6406	7.5160	7.6634	7.3189	7.4577	7.5443	7.5040	7.6238	7.5020	7.6404	7.4996	7.6317	7.5060	7.6558	**7.22**
**H5**	7.1771	7.3713	7.4596	7.4576	7.5958	7.4530	7.5980	7.4480	7.5902	7.4622	7.6214	7.1690	7.3494	7.4382	7.4277	7.5538	7.4237	7.5651	7.4194	7.5542	7.4316	7.5870	**7.22**
**H6**	8.1341	8.3392	8.4057	8.4426	8.5635	8.4366	8.5897	8.4305	8.5951	8.4485	8.6240	7.9194	8.0892	8.2103	8.1769	8.3421	8.1732	8.3457	8.1688	8.3441	8.1801	8.3934	**7.971**
**H8**	4.3741	4.4705	4.4922	4.5694	4.6265	4.5633	4.6458	4.5568	4.6625	4.5752	4.6720	4.5270	4.6702	4.7258	4.7346	4.8501	4.7322	4.8642	4.7292	4.8566	4.7367	4.8976	**5.2**
**H(9A-9C)**	1.2980	1.3052	1.2789	1.2881	1.2601	1.2903	1.2615	1.2922	1.2492	1.2862	1.2747	1.3734	1.4034	1.4070	1.4206	1.3953	1.4203	1.4103	1.4196	1.4138	1.4210	1.4367	**1.611**
**H(8`A-8`C)**	2.4555	2.3914	2.3685	2.3743	2.3708	2.3754	2.3485	2.3764	2.3407	2.3732	2.3328	2.8889	2.7195	2.6844	2.6446	2.6004	2.6510	2.5113	2.6569	2.5341	2.6383	2.4811	**2.82**
Nuclei	**B98**																						
**C1**	137.8984	138.3641	139.2698	138.5615	140.4825	138.5589	139.6880	138.5551	139.9714	138.5675	140.0693	136.6672	137.9623	138.5980	138.5797	140.1392	138.5482	140.0261	138.5178	139.8988	138.6168	140.0992	**130.99**
**C2**	138.5254	138.4721	139.0519	138.3240	138.8948	138.3503	139.3548	138.3736	139.2402	138.3008	139.3473	137.9447	138.0861	138.6429	138.0628	138.7300	138.0773	139.2568	138.0902	139.1509	138.0518	139.2683	**133.34**
**C3**	120.9751	121.6883	122.2401	121.9412	122.6168	121.9423	122.8875	121.9406	122.8295	121.9422	122.9429	121.0500	121.6961	122.2005	121.9352	122.6051	121.9366	122.8272	121.9354	122.7659	121.9361	122.8940	**117.55**
**C4**	173.5548	174.4326	175.0664	174.8850	175.5697	174.8724	176.2406	174.8571	176.0696	174.8994	176.3345	173.8262	174.4272	175.1004	174.7024	175.4540	174.7034	175.8475	174.7008	175.7561	174.7028	176.0007	**168.13**
**C5**	118.7946	120.1408	120.7556	120.8501	121.6768	120.8218	121.9460	120.7906	121.8434	120.8797	122.0849	118.9548	120.1808	120.7785	120.7832	121.6150	120.7632	121.7668	120.7403	121.6817	120.8040	121.9090	**117.55**
**C6**	134.9458	136.9033	137.9770	137.9779	139.4196	137.9297	140.0378	137.8779	139.7250	138.0256	140.3768	135.2851	137.2480	138.5012	138.2327	139.8890	138.1917	140.3423	138.1470	140.1749	138.2722	140.7184	**133.34**
**C7**	199.5271	204.5615	206.0295	207.1995	208.2386	207.0479	213.5136	206.8943	212.6014	207.3482	214.4291	204.1599	208.4637	209.6427	210.7144	211.5176	210.5930	216.3722	210.4697	215.2290	210.8323	216.8957	**195.87**
**C8**	65.0962	66.2575	66.8997	66.5776	67.5549	66.5763	67.8574	66.5723	67.7776	66.5806	68.7079	59.7427	61.6256	62.1747	62.5454	63.3980	62.4979	64.1348	62.4503	63.8905	62.5954	64.3856	**60.75**
**C9**	23.2897	23.5779	23.6929	23.8388	24.0941	23.8397	24.1215	23.8371	24.0489	23.8399	24.2881	20.0303	20.4587	20.5633	20.6605	20.8945	20.6623	21.0556	20.6621	20.9805	20.6615	21.4185	**16.39**
**C8`**	36.7092	37.4629	37.6740	37.7309	38.1053	37.7287	38.3351	37.7243	38.2965	37.7357	38.7417	32.2204	32.4032	32.6737	32.5780	32.8420	32.5807	32.9732	32.5806	33.0077	32.5770	33.0565	**32**
**H2**	8.6652	8.6018	8.6259	8.5647	8.5366	8.5677	8.5885	8.5705	8.5669	8.5621	8.5601	8.6821	8.5181	8.5678	8.4391	8.4515	8.4446	8.4365	8.4498	8.4543	8.4338	8.4469	**7.971**
**H3**	7.3240	7.4617	7.5525	7.5069	7.6251	7.5048	7.6424	7.5026	7.6335	7.5092	7.6562	7.3088	7.4478	7.5364	7.4945	7.6157	7.4921	7.6318	7.4897	7.6238	7.4970	7.6471	**7.22**
**H5**	7.1716	7.3643	7.4556	7.4510	7.5764	7.4460	7.5983	7.4411	7.5859	7.4560	7.6179	7.1649	7.3464	7.4371	7.4256	7.5523	7.4212	7.5629	7.4169	7.5523	7.4299	7.5837	**7.22**
**H6**	8.0929	8.2967	8.3821	8.4075	8.5267	8.4008	8.6016	8.3943	8.5719	8.4141	8.6200	7.8961	8.0681	8.1863	8.1586	8.3256	8.1538	8.3364	8.1488	8.3271	8.1631	8.3835	**7.971**
**H8**	4.3470	4.4366	4.4606	4.5354	4.5891	4.5287	4.6555	4.5220	4.6334	4.5423	4.6625	4.4951	4.6493	4.7037	4.7151	4.8293	4.7118	4.8454	4.7085	4.8323	4.7182	4.8756	**5.2**
**H(9A-9C)**	1.3189	1.3268	1.3143	1.3162	1.2740	1.3175	1.2894	1.3187	1.2788	1.3151	1.3029	1.3937	1.4261	1.4275	1.4419	1.4166	1.4413	1.4301	1.4407	1.4303	1.4425	1.4532	**1.611**
**H(8`A-8`C)**	2.4802	2.4260	2.3972	2.4073	2.3842	2.4082	2.3651	2.4093	2.3720	2.4067	2.3565	2.9208	2.7533	2.7171	2.6803	2.6349	2.6856	2.5498	2.6907	2.5670	2.6754	2.5194	**2.82**

[a] methyl protons shifts are averaged

[b] according to [10]

Table S10. Cont. I

Nuclei	R											S											Exp. [b]
	B971																						
	Gas	$CHCl_3$ (PCM)	$CHCl_3$ (SMD)	DMSO (PCM)	DMSO (SMD)	CH_3OH (PCM)	CH_3OH (SMD)	C_2H_5OH (PCM)	C_2H_5OH (SMD)	H_2O (PCM)	H_2O (SMD)	Gas	$CHCl_3$ (PCM)	$CHCl_3$ (SMD)	DMSO (PCM)	DMSO (SMD)	CH_3OH (PCM)	CH_3OH (SMD)	C_2H_5OH (PCM)	C_2H_5OH (SMD)	H_2O (PCM)	H_2O (SMD)	
**C1**	137.5124	137.9668	138.8036	138.1617	140.0037	138.1528	139.1730	138.1442	139.5653	138.1768	139.6476	136.2962	137.5659	138.2070	138.1911	139.7146	138.1505	139.5849	138.1134	139.4699	138.2405	139.6733	**130.99**
**C2**	138.2273	138.1790	138.7520	138.0411	138.6151	138.0588	139.0874	138.0759	138.9703	138.0298	139.0743	137.6507	137.7943	138.3494	137.7788	138.4352	137.7852	138.9549	137.7921	138.8493	137.7793	138.9688	**133.34**
**C3**	120.7723	121.4948	122.0366	121.7575	122.4239	121.7508	122.6953	121.7432	122.6290	121.7695	122.7532	120.8439	121.4989	122.0013	121.7481	122.4060	121.7414	122.6248	121.7343	122.5637	121.7602	122.6979	**117.55**
**C4**	173.4520	174.3378	174.9730	174.7959	175.4831	174.7758	176.1541	174.7552	175.9535	174.8209	176.2409	173.7091	174.3304	175.0011	174.6204	175.3683	174.6132	175.7597	174.6046	175.6659	174.6323	175.9170	**168.13**
**C5**	118.5912	119.9438	120.5513	120.6619	121.4796	120.6255	121.7575	120.5889	121.6419	120.7023	121.8945	118.7577	119.9871	120.5816	120.5974	121.4204	120.5694	121.5676	120.5405	121.4822	120.6294	121.7173	**117.55**
**C6**	134.7050	136.6708	137.7813	137.7564	139.2253	137.6992	139.8111	137.6410	139.4847	137.8168	140.1424	135.0285	137.0109	138.2571	138.0065	139.6769	137.9579	140.1333	137.9074	139.9591	138.0571	140.5087	**133.34**
**C7**	199.4123	204.3866	205.8037	206.9774	207.9980	206.8235	213.1394	206.6697	212.2541	207.1300	214.1117	204.0187	208.2806	209.4897	210.5322	211.3305	210.4028	216.0716	210.2738	214.9613	210.6620	216.6144	**195.87**
**C8**	65.0628	66.2181	66.8745	66.5493	67.4912	66.5398	67.7569	66.5294	67.7680	66.5644	68.6439	59.7053	61.5405	62.0853	62.4460	63.2954	62.3913	63.9952	62.3385	63.7609	62.5067	64.2505	**60.75**
**C9**	23.2952	23.5805	23.7317	23.8547	24.1127	23.8461	24.1179	23.8365	24.0691	23.8689	24.2944	20.0444	20.4947	20.5876	20.7006	20.9390	20.6951	21.1020	20.6896	21.0266	20.7123	21.4780	**16.39**
**C8`**	36.7170	37.4689	37.6835	37.7337	38.0702	37.7257	38.3145	37.7173	38.2603	37.7475	38.7194	32.1504	32.3460	32.6128	32.5305	32.7822	32.5259	32.9103	32.5202	32.9431	32.5401	32.9962	**32**
**H2**	8.6629	8.6020	8.6311	8.5654	8.5437	8.5681	8.6026	8.5708	8.5678	8.5633	8.5647	8.6781	8.5197	8.5677	8.4422	8.4548	8.4475	8.4418	8.4527	8.4583	8.4370	8.4509	**7.971**
**H3**	7.3215	7.4590	7.5493	7.5044	7.6225	7.5021	7.6392	7.4997	7.6299	7.5070	7.6527	7.3076	7.4456	7.5341	7.4931	7.6131	7.4905	7.6286	7.4879	7.6208	7.4959	7.6437	**7.22**
**H5**	7.1696	7.3613	7.4527	7.4481	7.5728	7.4430	7.5957	7.4379	7.5815	7.4533	7.6142	7.1650	7.3449	7.4351	7.4237	7.5495	7.4192	7.5594	7.4147	7.5489	7.4283	7.5805	**7.22**
**H6**	8.0974	8.2998	8.3897	8.4087	8.5283	8.4021	8.6053	8.3956	8.5653	8.4153	8.6195	7.8972	8.0707	8.1892	8.1631	8.3306	8.1581	8.3421	8.1530	8.3320	8.1679	8.3885	**7.971**
**H8**	4.3445	4.4318	4.4558	4.5292	4.5852	4.5223	4.6544	4.5157	4.6229	4.5361	4.6585	4.4978	4.6489	4.7036	4.7168	4.8297	4.7133	4.8452	4.7097	4.8315	4.7203	4.8758	**5.2**
**H(9A-9C)**	1.3191	1.3280	1.3197	1.3196	1.2780	1.3205	1.2937	1.3214	1.2832	1.3191	1.3053	1.3960	1.4276	1.4283	1.4433	1.4195	1.4425	1.4331	1.4418	1.4326	1.4442	1.4560	**1.611**
**H(8`A-8`C)**	2.4778	2.4208	2.3902	2.4035	2.3793	2.4041	2.3533	2.4048	2.3662	2.4033	2.3481	2.9110	2.7452	2.7096	2.6744	2.6284	2.6794	2.5438	2.6843	2.5610	2.6698	2.5128	**2.82**
Nuclei	B972																						
**C1**	134.4302	134.9580	135.9573	135.2517	137.1882	135.2339	136.9092	135.2172	137.2291	135.2642	136.9986	133.0960	134.5569	135.0556	135.2214	136.6788	135.1632	136.7562	135.1090	136.5422	135.2805	136.8545	**130.99**
**C2**	136.2385	136.1466	136.7094	135.9825	136.5017	135.9974	136.8781	136.0094	136.7762	135.9648	136.9004	135.6839	135.8309	136.3876	135.8469	136.4661	135.8466	136.9148	135.8443	136.8312	135.8431	136.9435	**133.34**
**C3**	118.6420	119.3619	119.9304	119.6913	120.3271	119.6734	120.6091	119.6533	120.5621	119.7042	120.6885	118.7138	119.3622	119.8746	119.6667	120.3028	119.6505	120.5119	119.6318	120.4478	119.6777	120.5982	**117.55**
**C4**	170.4080	171.2888	171.9012	171.8240	172.3935	171.7908	172.9800	171.7559	172.8201	171.8517	173.1417	170.6771	171.2152	171.8947	171.5417	172.2306	171.5278	172.5783	171.5099	172.5046	171.5489	172.7454	**168.13**
**C5**	116.5455	117.8870	118.4963	118.6438	119.4131	118.5990	119.6446	118.5521	119.5386	118.6819	119.8150	116.6811	117.8807	118.4867	118.5356	119.3203	118.4989	119.4656	118.4592	119.3807	118.5657	119.6278	**117.55**
**C6**	132.2984	134.1683	135.1909	135.2810	136.6319	135.2152	137.0709	135.1451	136.7199	135.3380	137.5017	132.7192	134.6132	135.8785	135.6223	137.1863	135.5695	137.5344	135.5120	137.4030	135.6656	137.9182	**133.34**
**C7**	197.1472	202.1820	203.6394	205.0747	205.9500	204.8855	211.4809	204.7016	210.7109	205.2515	212.1626	201.5167	205.7889	206.8296	208.0334	208.7686	207.8942	213.5796	207.7543	212.4081	208.1644	214.0847	**195.87**
**C8**	62.2129	63.1640	63.7653	63.5253	64.4519	63.5132	65.0374	63.4956	64.9409	63.5298	65.7131	56.9800	58.9172	59.3838	59.8622	60.6095	59.7928	61.4484	59.7250	61.1550	59.9290	61.7202	**60.75**
**C9**	22.2017	22.5014	22.6308	22.6974	22.9802	22.6894	22.9728	22.6772	22.8720	22.7010	23.1432	18.9615	19.3718	19.4861	19.6193	19.7902	19.6032	19.9459	19.5854	19.8717	19.6315	20.3049	**16.39**
**C8`**	35.6349	36.3454	36.5312	36.8429	37.0375	36.8098	37.2438	36.7749	37.2215	36.8693	37.6484	31.3495	31.4924	31.7924	31.7285	31.9630	31.7188	31.9659	31.7053	32.0216	31.7314	32.0500	**32**
**H2**	8.6537	8.5910	8.6014	8.5457	8.5058	8.5490	8.4992	8.5518	8.4749	8.5424	8.5039	8.6825	8.4992	8.5620	8.4234	8.4354	8.4301	8.3959	8.4360	8.4226	8.4161	8.4046	**7.971**
**H3**	7.3227	7.4597	7.5484	7.5068	7.6209	7.5045	7.6375	7.5020	7.6284	7.5088	7.6533	7.3120	7.4483	7.5346	7.4976	7.6140	7.4951	7.6296	7.4923	7.6214	7.4999	7.6455	**7.22**
**H5**	7.1739	7.3666	7.4552	7.4573	7.5754	7.4520	7.5903	7.4465	7.5774	7.4620	7.6138	7.1665	7.3420	7.4301	7.4225	7.5446	7.4180	7.5559	7.4132	7.5449	7.4267	7.5779	**7.22**
**H6**	8.1158	8.3161	8.3945	8.4358	8.5373	8.4282	8.5663	8.4204	8.5382	8.4429	8.6045	7.9070	8.0727	8.1924	8.1605	8.3194	8.1565	8.3186	8.1518	8.3151	8.1637	8.3642	**7.971**
**H8**	4.3399	4.4508	4.4730	4.5472	4.5919	4.5405	4.6226	4.5339	4.6079	4.5536	4.6488	4.4883	4.6298	4.6848	4.6954	4.8083	4.6924	4.8202	4.6889	4.8127	4.6977	4.8518	**5.2**
**H(9A-9C)**	1.2779	1.2737	1.2581	1.2603	1.2187	1.2618	1.2249	1.2628	1.2101	1.2586	1.2452	1.3585	1.3871	1.3915	1.4080	1.3784	1.4071	1.3926	1.4058	1.3948	1.4087	1.4173	**1.611**
**H(8`A-8`C)**	2.4444	2.3879	2.3637	2.3740	2.3543	2.3748	2.3536	2.3753	2.3559	2.3732	2.3366	2.8829	2.7146	2.6793	2.6431	2.5976	2.6486	2.5129	2.6536	2.5335	2.6373	2.4818	**2.82**
Nuclei	BHandH																						
**C1**	138.2231	138.5552	139.4722	138.6613	140.1957	138.6447	139.2014	138.6466	139.2910	138.6472	139.3414	137.3260	138.1227	139.0514	138.6686	139.4127	138.6143	139.9113	138.5817	139.6959	138.6934	140.0677	**130.99**
**C2**	143.5201	143.3955	144.0049	143.2268	143.8191	143.2277	144.3374	143.2468	144.2139	143.1959	144.3719	143.1561	143.1628	143.7749	143.0888	143.8329	143.0819	144.2258	143.0940	144.0867	143.0659	144.2500	**133.34**
**C3**	123.7766	124.4896	125.0912	124.7508	125.4195	124.7262	125.6101	124.7198	125.5593	124.7437	125.6833	123.8317	124.5005	125.1213	124.7551	125.4992	124.7302	125.6175	124.7238	125.5792	124.7487	125.7026	**117.55**
**C4**	174.6910	175.5022	176.1372	175.9474	176.5413	175.9096	177.2138	175.8903	177.0668	175.9534	177.3817	174.9086	175.5511	176.2159	175.8630	176.6071	175.8357	176.9768	175.8261	176.9107	175.8585	177.1498	**168.13**
**C5**	121.3254	122.7876	123.4824	123.5575	124.4251	123.5004	124.6212	123.4617	124.5293	123.5816	124.7930	121.4351	122.8143	123.5066	123.5206	124.2647	123.4688	124.5249	123.4348	124.4609	123.5397	124.7001	**117.55**
**C6**	139.9383	142.0591	143.3061	143.2147	144.9304	143.1340	145.6585	143.0715	145.4144	143.2611	146.1074	140.2023	142.4966	143.7026	143.6179	144.3620	143.5430	145.9734	143.4853	145.7743	143.6576	146.3435	**133.34**
**C7**	202.9300	207.7859	209.1882	210.2924	210.9441	210.1306	215.8168	209.9874	214.6983	210.4156	216.6335	207.3621	211.4788	213.0278	213.6769	214.4210	213.5328	219.1023	213.4077	218.0494	213.7841	219.7557	**195.87**
**C8**	59.9494	61.2143	61.9067	61.6436	62.6312	61.6098	63.0978	61.5942	62.9356	61.6453	63.8442	54.0631	55.4214	55.9382	56.1974	56.9415	56.1327	57.6583	56.0882	57.3239	56.2306	57.9608	**60.75**
**C9**	22.4684	22.7173	23.0362	22.9961	23.4920	22.9658	23.4131	22.9537	23.4079	22.9951	23.6098	19.0747	19.5603	19.6330	19.8271	20.5712	19.7976	20.2453	19.7870	20.1575	19.8256	20.6529	**16.39**
**C8`**	33.7500	34.5612	34.8118	34.8998	35.2711	34.8687	35.5037	34.8562	35.4510	34.8996	35.9918	28.6454	29.0946	29.4036	29.3316	30.0757	29.3102	29.7782	29.3056	29.7931	29.3204	29.9429	**32**
**H2**	8.9875	8.9200	8.9348	8.8802	8.8770	8.8834	8.9222	8.8858	8.9249	8.8781	8.9222	8.9840	8.8580	8.8706	8.7732	8.6920	8.7803	8.7507	8.7862	8.7811	8.7672	8.7533	**7.971**
**H3**	7.4553	7.5997	7.6892	7.6466	7.7674	7.6451	7.7842	7.6427	7.7763	7.6491	7.8000	7.4516	7.5922	7.6830	7.6387	7.5575	7.6372	7.7745	7.6347	7.7677	7.6412	7.7910	**7.22**
**H5**	7.3003	7.5022	7.5933	7.5917	7.7206	7.5873	7.7378	7.5822	7.7268	7.5967	7.7629	7.2950	7.4886	7.5794	7.5727	7.4915	7.5687	7.7122	7.5639	7.7021	7.5774	7.7361	**7.22**
**H6**	8.3317	8.5445	8.6361	8.6585	8.7941	8.6524	8.8645	8.6454	8.8495	8.6654	8.9071	8.1293	8.3337	8.4417	8.4287	8.3475	8.4246	8.6263	8.4194	8.6221	8.4334	8.6718	**7.971**
**H8**	4.1505	4.2528	4.2749	4.3463	4.3866	4.3407	4.4438	4.3343	4.4339	4.3528	4.4693	4.4049	4.5696	4.6414	4.6481	4.5669	4.6447	4.7936	4.6404	4.7922	4.6522	4.8291	**5.2**
**H(9A-9C)**	1.3947	1.4083	1.3944	1.4043	1.3835	1.4056	1.4087	1.4060	1.4066	1.4041	1.4308	1.4777	1.5048	1.4783	1.5114	1.4301	1.5120	1.4963	1.5117	1.4978	1.5117	1.5179	**1.611**
**H(8`A-8`C)**	2.5482	2.4741	2.4402	2.4443	2.4243	2.4467	2.3801	2.4483	2.3790	2.4430	2.3667	2.9170	2.7545	2.7130	2.6709	2.5897	2.6772	2.5211	2.6825	2.5633	2.6659	2.4973	**2.82**
Nuclei	BHandHLYP																						
**C1**	138.8640	139.2644	139.9330	139.4014	141.4115	139.3890	140.3239	139.3799	140.8073	139.4140	140.7808	137.7359	138.7863	139.5663	139.4872	141.0279	139.4436	140.6370	139.4057	140.4914	139.5319	140.6845	**130.99**
**C2**	142.1451	142.0033	142.5518	141.8479	142.3133	141.8593	142.8334	141.8728	142.6946	141.8367	142.8238	141.6846	141.6776	142.2130	141.6154	142.1662	141.6189	142.6674	141.6254	142.5678	141.6119	142.7010	**133.34**
**C3**	122.5311	123.1246	123.6134	123.3344	123.9644	123.3231	124.1502	123.3143	124.1344	123.3446	124.2197	122.6170	123.1643	123.6329	123.3811	123.9687	123.3691	124.1171	123.3594	124.0780	123.3922	124.1810	**117.55**
**C4**	174.4859	175.2386	175.8387	175.6759	176.2231	175.6490	176.9757	175.6248	176.7716	175.7009	177.0601	174.7783	175.2638	175.8588	175.5025	176.1211	175.4893	176.5698	175.4775	176.5010	175.5141	176.7410	**168.13**
**C5**	120.1712	121.4535	122.0189	122.1435	122.9398	122.1013	123.1049	122.0616	123.0479	122.1835	123.2751	120.2936	121.5123	122.0694	122.1337	122.8970	122.0972	123.0006	122.0628	122.9395	122.1680	123.1441	**117.55**
**C6**	138.1510	140.1493	141.3422	141.2896	142.5504	141.2223	143.2999	141.1564	142.9195	141.3529	143.5841	138.4674	140.5298	141.7475	141.5098	143.1000	141.4526	143.6578	141.3968	143.5207	141.5620	144.0715	**133.34**
**C7**	201.2543	206.5546	207.7752	209.3182	210.2498	209.1454	216.0604	208.9760	215.0849	209.4828	217.0651	206.1611	210.4578	211.6283	212.8433	213.4066	212.7018	218.6263	212.5625	217.3826	212.9773	219.2046	**195.87**
**C8**	60.7965	61.8811	62.5000	62.2233	63.0588	62.2069	63.2656	62.1924	63.2636	62.2383	64.1041	55.4804	57.1821	57.8021	58.1054	58.8956	58.0473	59.5516	57.9937	59.2868	58.1628	59.8130	**60.75**
**C9**	22.2333	22.3886	22.6124	22.6282	22.8233	22.6139	22.7047	22.6016	22.6825	22.6409	22.8547	18.9648	19.2708	19.4004	19.4144	19.6557	19.4060	19.6995	19.4005	19.6674	19.4225	20.0180	**16.39**
**C8`**	34.7377	35.5130	35.7819	35.8289	36.1801	35.8108	36.3989	35.7951	36.3597	35.8459	36.8091	30.5960	30.8142	31.0655	30.9812	31.2247	30.9680	31.3407	30.9569	31.3887	30.9927	31.4699	**32**
**H2**	8.8761	8.8039	8.8520	8.7659	8.7241	8.7690	8.7906	8.7718	8.7505	8.7629	8.7519	8.8981	8.7359	8.7665	8.6405	8.6466	8.6464	8.6344	8.6519	8.6571	8.6345	8.6458	**7.971**
**H3**	7.4433	7.5783	7.6654	7.6233	7.7361	7.6213	7.7566	7.6190	7.7465	7.6252	7.7698	7.4323	7.5668	7.6511	7.6133	7.7280	7.6111	7.7441	7.6087	7.7353	7.6153	7.7592	**7.22**
**H5**	7.2878	7.4785	7.5663	7.5650	7.6852	7.5602	7.7061	7.5552	7.6928	7.5695	7.7273	7.2798	7.4603	7.5446	7.5400	7.6603	7.5356	7.6720	7.5311	7.6611	7.5440	7.6938	**7.22**
**H6**	8.1765	8.3928	8.4992	8.5149	8.6356	8.5078	8.7226	8.5005	8.6822	8.5217	8.7376	8.0113	8.2014	8.3159	8.2915	8.4587	8.2866	8.4826	8.2813	8.4746	8.2959	8.5310	**7.971**
**H8**	4.0776	4.1885	4.2247	4.3024	4.3719	4.2945	4.4593	4.2868	4.4300	4.3099	4.4624	4.2587	4.4280	4.4860	4.5022	4.6169	4.4983	4.6464	4.4943	4.6344	4.5056	4.6772	**5.2**
**H(9A-9C)**	1.2993	1.3062	1.3063	1.3002	1.2417	1.3014	1.2609	1.3023	1.2448	1.2990	1.2689	1.3682	1.3960	1.3920	1.4076	1.3798	1.4070	1.4019	1.4061	1.4026	1.4081	1.4244	**1.611**
**H(8`A-8`C)**	2.4396	2.3870	2.3452	2.3723	2.3466	2.3731	2.3248	2.3737	2.3360	2.3717	2.3239	2.8786	2.7124	2.6679	2.6359	2.5874	2.6411	2.5108	2.6458	2.5345	2.6307	2.4845	**2.82**

[a] methyl protons shifts are averaged

[b] according to [10]

Table S10. Cont. II

Nuclei	R											S											Exp. [b]
	BLYP																						
	Gas	$CHCl_3$ (PCM)	$CHCl_3$ (SMD)	DMSO (PCM)	DMSO (SMD)	CH_3OH (PCM)	CH_3OH (SMD)	C_2H_5OH (PCM)	C_2H_5OH (SMD)	H_2O (PCM)	H_2O (SMD)	Gas	$CHCl_3$ (PCM)	$CHCl_3$ (SMD)	DMSO (PCM)	DMSO (SMD)	CH_3OH (PCM)	CH_3OH (SMD)	C_2H_5OH (PCM)	C_2H_5OH (SMD)	H_2O (PCM)	H_2O (SMD)	
**C1**	139.2740	140.0627	140.7302	140.3557	142.0482	140.6261	141.6136	140.4250	141.4809	140.7208	141.8598	138.3037	138.8878	139.5939	139.5904	141.4131	139.8236	141.0985	139.5956	141.3416	139.9982	141.2014	**130.99**
**C2**	137.3888	137.3557	137.9052	137.1077	137.7439	137.4006	137.9857	137.2334	138.0955	137.4576	138.0801	136.8040	136.8446	137.3898	136.8417	137.6112	137.1207	137.9926	136.9376	138.0835	137.2041	138.0703	**133.34**
**C3**	120.6544	121.3842	121.8707	121.6716	122.2855	121.9330	122.4590	121.7316	122.5866	122.0499	122.6336	119.6392	121.4514	121.9240	121.6569	122.3097	121.9238	122.3772	121.7284	122.4989	122.0306	122.5353	**117.55**
**C4**	177.6519	178.7185	179.3932	179.3068	180.0988	179.5462	180.5234	179.3230	180.6223	179.7057	180.7915	178.1086	178.8813	179.6485	179.1527	180.0400	179.4160	180.2300	179.2172	180.2861	179.5290	180.4844	**168.13**
**C5**	118.5470	119.8601	120.3998	120.5456	121.3222	120.7815	121.4404	120.5550	121.5434	120.9483	121.6902	118.6599	120.0133	120.5942	120.5540	121.3508	120.8003	121.3513	120.5845	121.4329	120.9468	121.5891	**117.55**
**C6**	133.7911	135.6052	136.7931	136.6678	138.3637	136.8753	138.6685	136.6243	138.6992	137.1000	139.1691	138.8182	136.2738	137.4058	137.2526	138.9036	137.4733	139.2393	137.2316	139.1572	137.6692	139.7543	**133.34**
**C7**	203.2767	208.6048	209.8305	211.6985	212.3595	211.7964	217.3300	211.4147	216.3806	212.2229	218.3121	205.2971	212.7975	214.3032	214.9277	216.0897	215.0775	220.0988	214.7652	219.4181	215.4121	220.5992	**195.87**
**C8**	69.1241	70.1253	70.7992	70.3573	71.5764	70.6395	71.9231	70.4601	71.8008	70.7169	72.6971	68.1488	65.4631	65.8331	66.4810	67.5528	66.6957	68.0924	66.4492	68.1157	66.9054	68.4506	**60.75**
**C9**	23.7215	24.0520	24.2062	24.1836	24.6500	24.4455	24.6108	24.2503	24.7226	24.5640	24.8492	16.4537	20.8061	20.7678	21.0333	21.2955	21.2977	21.4360	21.1003	21.5017	21.4093	21.9663	**16.39**
**C8`**	38.2242	38.9477	39.0730	39.4073	39.6721	39.6658	39.7283	39.4601	39.9360	39.7878	40.2492	32.8912	33.8280	34.1359	33.9651	34.2232	34.2348	34.1613	34.0423	34.3099	34.3359	34.3012	**32**
**H2**	8.3954	8.3311	8.3657	8.2850	8.2783	8.3345	8.4554	8.2924	8.3194	8.3386	8.4701	8.3743	8.3060	8.3480	8.2230	8.2163	8.2757	8.3816	8.2357	8.2114	8.2732	8.4083	**7.971**
**H3**	7.1679	7.2953	7.3831	7.3318	7.4491	7.3773	7.6306	7.3305	7.4581	7.3888	7.6578	7.0966	7.2855	7.3681	7.3259	7.4422	7.3712	7.6247	7.3239	7.4500	7.3832	7.6520	**7.22**
**H5**	7.0230	7.2052	7.2897	7.2883	7.4065	7.3310	7.5902	7.2811	7.4161	7.3480	7.6256	6.9899	7.1903	7.2755	7.2597	7.3808	7.3031	7.5580	7.2540	7.3806	7.3187	7.5918	**7.22**
**H6**	7.9452	8.1295	8.2114	8.2406	8.3476	8.2808	8.5506	8.2286	8.3909	8.3029	8.6025	7.8754	7.8764	7.9806	7.9535	8.0977	7.9966	8.2837	7.9470	8.0952	8.0128	8.3454	**7.971**
**H8**	4.5730	4.6582	4.6658	4.7406	4.7734	4.7808	4.9699	4.7284	4.7971	4.8027	5.0172	4.4807	4.8131	4.8614	4.8724	4.9776	4.9165	5.1608	4.8680	4.9755	4.9306	5.2060	**5.2**
**H(9A-9C)**	1.3344	1.3224	1.3240	1.2985	1.2925	1.3477	1.4693	1.3049	1.3035	1.3524	1.4944	1.2856	1.4612	1.4487	1.4808	1.4580	1.5270	1.6506	1.4807	1.4754	1.5371	1.6944	**1.611**
**H(8`A-8`C)**	2.5702	2.5055	2.4761	2.4845	2.4652	2.5330	2.6299	2.4890	2.4571	2.5387	2.6207	2.5654	2.8839	2.8478	2.7998	2.7482	2.8522	2.8332	2.8121	2.6782	2.8501	2.8119	**2.82**
Nuclei	BMK																						
**C1**	154.5489	154.7956	155.8147	155.0346	156.7869	155.0048	155.7582	155.1850	155.9437	155.2232	155.7278	153.3708	154.7062	155.5737	155.3493	157.1804	155.2822	157.0114	155.4300	156.7717	155.5792	157.0888	**130.99**
**C2**	155.5489	155.3349	156.1444	155.0820	155.8798	155.0811	156.4462	155.2910	156.2529	155.2438	156.4023	154.7572	154.6732	155.3927	154.5734	155.4851	154.5591	155.9928	154.7574	155.7913	154.7494	155.9653	**133.34**
**C3**	135.9186	136.5742	137.2866	136.7717	137.6790	136.7457	137.8453	136.9307	137.6978	136.9577	137.8467	136.0540	136.5812	137.2516	136.7609	137.6638	136.7339	137.8342	136.9186	137.6908	136.9479	137.8865	**117.55**
**C4**	189.4005	190.1835	191.0076	190.6219	191.5290	190.5810	192.1342	190.7513	191.8731	190.8217	192.2418	189.7985	190.2293	191.0560	190.4438	191.4104	190.4180	191.7002	190.6026	191.5626	190.6291	191.8308	**168.13**
**C5**	133.4295	134.7613	135.4788	135.4278	136.5512	135.3675	136.6886	135.5196	136.4925	135.6472	136.8148	133.4535	134.7683	135.5390	135.3777	136.4835	135.3275	136.5822	135.4877	136.3606	135.5868	136.7328	**117.55**
**C6**	151.4094	153.6757	155.2637	155.0157	156.8896	154.9195	157.7543	155.0316	157.2746	155.2662	158.1427	152.1159	153.9639	155.4209	155.0094	157.0550	154.9327	157.4837	155.0660	157.2182	155.2429	157.8187	**133.34**
**C7**	216.9478	222.5474	224.1482	225.4916	226.2743	225.2972	232.0453	225.3132	230.7791	225.8361	232.8163	221.5389	226.5692	228.0915	229.2000	230.0942	229.0219	235.6763	229.0559	234.3215	229.5316	236.4133	**195.87**
**C8**	70.5725	71.6400	72.4428	72.1543	73.3210	72.1213	73.7576	72.2984	73.5299	72.3466	74.4771	64.4902	66.3795	67.0821	67.2150	68.2924	67.1424	69.1388	67.2827	68.7153	67.4478	69.3740	**60.75**
**C9**	25.4816	25.8566	26.3477	26.2252	26.6325	26.1938	26.7756	26.3697	26.5780	26.4143	26.8310	22.1260	22.5585	22.7087	22.6468	23.0607	22.6251	23.2222	22.8150	23.0572	22.8297	23.5137	**16.39**
**C8`**	38.7886	39.4905	39.7965	39.7916	40.2856	39.7653	40.4650	39.9500	40.3114	39.9781	40.8997	33.9443	34.1818	34.5674	34.3474	34.7515	34.3245	34.7335	34.5118	34.8069	34.5294	34.8356	**32**
**H2**	9.0522	9.0233	9.0737	8.9738	8.9666	8.9734	9.0551	8.9730	9.0302	8.9645	9.0669	9.0430	8.8827	8.9188	8.8117	8.8174	8.8133	8.8099	8.8146	8.8396	8.8005	8.8138	**7.971**
**H3**	7.6503	7.8088	7.9094	7.8680	7.9716	7.8622	8.0008	7.8561	7.9908	7.8636	8.0111	7.6582	7.8152	7.8999	7.8689	7.9851	7.8629	7.9933	7.8568	7.9965	7.8650	8.0096	**7.22**
**H5**	7.5098	7.7043	7.7841	7.7896	7.9181	7.7809	7.9299	7.7723	7.9192	7.7883	7.9561	7.4861	7.6973	7.7852	7.7793	7.9021	7.7717	7.9135	7.7640	7.8990	7.7768	7.9393	**7.22**
**H6**	8.4072	8.6270	8.7828	8.7961	8.9052	8.7837	9.0195	8.7706	8.9763	8.7979	9.0628	8.3050	8.4960	8.6092	8.6017	8.7715	8.5925	8.7332	8.5831	8.7698	8.6007	8.7910	**7.971**
**H8**	4.2317	4.3488	4.4104	4.4856	4.5292	4.4731	4.5903	4.4604	4.5756	4.4876	4.6005	4.4400	4.6482	4.6995	4.7284	4.8382	4.7206	4.8188	4.7127	4.8373	4.7262	4.8615	**5.2**
**H(9A-9C)**	1.3048	1.3335	1.3264	1.3372	1.2998	1.3347	1.3417	1.3320	1.3281	1.3300	1.3703	1.3776	1.3895	1.3774	1.4034	1.3701	1.3999	1.3895	1.3963	1.3784	1.3971	1.4125	**1.611**
**H(8`A-8`C)**	2.4334	2.3750	2.3238	2.3567	2.3235	2.3539	2.2956	2.3513	2.3080	2.3498	2.2771	2.8035	2.6688	2.6299	2.6096	2.5602	2.6105	2.4481	2.6113	2.4916	2.5990	2.4288	**2.82**
Nuclei	BP86																						
**C1**	135.9939	136.6320	137.6362	136.9544	138.7298	136.9390	138.2948	136.9186	138.3141	136.9642	138.7051	134.4352	135.9816	136.4890	136.5815	138.0085	136.5423	138.0378	136.5022	137.9223	136.6187	138.2985	**130.99**
**C2**	136.0461	136.1412	136.6915	135.9389	136.4876	135.9629	136.9072	135.9812	136.7985	135.9104	137.0603	135.2799	135.6510	136.1578	135.6672	136.3174	135.6758	136.8585	135.6802	136.7480	135.6552	137.0159	**133.34**
**C3**	119.5556	120.4857	121.0080	120.7308	121.3490	120.7247	121.6928	120.7132	121.6404	120.7314	121.9360	119.6977	120.5178	120.9979	120.7317	121.3594	120.7284	121.6195	120.7195	121.5439	120.7299	121.8637	**117.55**
**C4**	174.3602	175.5049	176.1304	175.8536	176.5630	175.8334	177.1710	175.8083	177.0813	175.8678	177.4848	174.7072	175.5024	176.1986	175.7445	176.5291	175.7420	176.8841	175.7330	176.7851	175.7408	177.2044	**168.13**
**C5**	117.4531	118.9871	119.5529	119.6506	120.4030	119.6163	120.7206	119.5768	120.6236	119.6782	121.0496	117.6959	119.0396	119.6143	119.6262	120.4183	119.6030	120.5906	119.5742	120.4936	119.6424	120.9111	**117.55**
**C6**	132.4823	134.5222	135.5397	135.4991	136.9975	135.4473	137.5315	135.3905	137.2521	135.5440	138.0880	132.8422	134.9807	136.2191	135.9532	137.6251	135.9065	138.0996	135.8536	137.8931	135.9910	138.6398	**133.34**
**C7**	200.3568	205.1844	206.6915	207.6362	208.7456	207.4854	213.3518	207.3301	212.6663	207.7757	214.3906	205.2587	209.2590	210.3906	211.2385	212.1476	211.1283	216.2414	211.0136	215.3050	211.3390	216.8705	**195.87**
**C8**	67.0914	68.3350	69.0224	68.6336	69.6697	68.6283	70.1232	68.6177	69.8169	68.6343	71.1003	61.2199	63.3467	63.8475	64.2565	65.1036	64.1999	65.8529	64.1410	65.6179	64.3090	66.2851	**60.75**
**C9**	23.4788	23.9387	24.0159	24.1770	24.4194	24.1716	24.5594	24.1605	24.4086	24.1770	24.8922	20.0447	20.8111	20.8964	21.0566	21.2467	21.0482	21.5506	21.0352	21.4325	21.0604	22.1852	**16.39**
**C8`**	37.5056	38.3780	38.4809	38.5899	38.8300	38.5853	39.0439	38.5763	39.0514	38.5903	39.6736	32.7385	32.9732	33.1909	33.0751	33.2543	33.0771	33.3634	33.0731	33.3752	33.0673	33.5888	**32**
**H2**	8.4690	8.4251	8.4168	8.3721	8.3303	8.3765	8.3610	8.3805	8.3653	8.3675	8.3657	8.5023	8.3508	8.3888	8.2771	8.2780	8.2836	8.2597	8.2894	8.2717	8.2701	8.2813	**7.971**
**H3**	7.1833	7.3254	7.4023	7.3644	7.4695	7.3630	7.4872	7.3613	7.4789	7.3653	7.5157	7.1759	7.3137	7.3896	7.3551	7.4646	7.3535	7.4797	7.3516	7.4705	7.3561	7.5088	**7.22**
**H5**	7.0316	7.2248	7.3017	7.3045	7.4170	7.3004	7.4360	7.2960	7.4267	7.3081	7.4730	7.0289	7.2047	7.2838	7.2819	7.3979	7.2785	7.4064	7.2747	7.3955	7.2847	7.4416	**7.22**
**H6**	8.0317	8.2270	8.2775	8.3141	8.4038	8.3094	8.4531	8.3044	8.4474	8.3183	8.4976	7.7817	7.9419	8.0474	8.0258	8.1760	8.0224	8.1879	8.0185	8.1750	8.0284	8.2496	**7.971**
**H8**	4.6085	4.6845	4.6748	4.7603	4.7803	4.7553	4.8233	4.7502	4.8256	4.7646	4.8587	4.7327	4.8659	4.9018	4.9243	5.0225	4.9224	5.0356	4.9199	5.0205	4.9256	5.0853	**5.2**
**H(9A-9C)**	1.3656	1.3684	1.3441	1.3548	1.3143	1.3570	1.3284	1.3589	1.3132	1.3524	1.3589	1.4453	1.4875	1.4805	1.5086	1.4790	1.5084	1.4981	1.5079	1.4947	1.5083	1.5410	**1.611**
**H(8`A-8`C)**	2.5546	2.4795	2.4461	2.4610	2.4324	2.4627	2.4144	2.4642	2.4111	2.4590	2.4105	3.0093	2.8351	2.7840	2.7540	2.6945	2.7609	2.6012	2.7671	2.6178	2.7468	2.5828	**2.82**
Nuclei	CAM-B3LYP																						
**C1**	140.5940	141.0501	141.9502	141.2482	143.3376	141.2328	142.6219	141.2195	142.7966	141.2664	142.8916	139.3811	140.7764	141.3414	141.3955	142.9372	141.3447	142.8551	141.3003	142.5657	141.4530	142.9212	**130.99**
**C2**	142.4322	142.3676	142.8920	142.2031	142.7302	142.2185	143.2393	142.2342	143.1183	142.1902	143.2313	141.9632	142.0722	142.5741	142.0137	142.6143	142.0196	143.1220	142.0264	143.0134	142.0107	143.1501	**133.34**
**C3**	123.8770	124.5297	125.0152	124.7351	125.3944	124.7281	125.5969	124.7212	125.5656	124.7437	125.6728	123.9590	124.5672	125.0168	124.7695	125.4058	124.7619	125.5862	124.7548	125.5220	124.7791	125.6510	**117.55**
**C4**	177.6432	178.4616	179.0236	178.8866	179.5004	178.8652	180.0958	178.8438	179.9671	178.9091	180.2452	177.9270	178.4619	179.0815	178.7166	179.4216	178.7091	179.7910	178.7009	179.7287	178.7245	179.9540	**168.13**
**C5**	121.6531	122.9882	123.5423	123.6761	124.5002	123.6378	124.6833	123.5998	124.6086	123.7146	124.8524	121.7948	123.0474	123.5984	123.6473	124.6484	123.6163	124.5780	123.5853	124.4990	123.6786	124.7226	**117.55**
**C6**	138.6325	140.6802	141.7768	141.7799	143.1835	141.7181	143.7450	141.6562	143.4517	141.8399	144.1171	138.9686	141.0487	142.3055	142.0534	143.7538	142.0017	144.1534	141.9484	144.0548	142.1023	144.5739	**133.34**
**C7**	205.1841	210.4669	211.8770	213.2659	214.3729	213.0946	219.8334	212.9249	218.8841	213.4315	220.8758	210.1044	214.5528	215.7343	216.9360	217.6675	216.7972	222.7736	216.6596	221.5003	217.0720	223.3268	**195.87**
**C8**	63.5728	64.7701	65.4574	65.1172	66.1087	65.1025	66.5638	65.0883	66.3449	65.1333	67.2834	57.9234	59.8230	60.3104	60.7065	61.5242	60.6433	62.2922	60.5845	61.9726	60.7729	62.5903	**60.75**
**C9**	22.4381	22.6575	22.8386	22.9008	23.1565	22.8881	23.1031	22.8752	23.0434	22.9144	23.2395	19.2771	19.6649	19.7105	19.7757	20.0125	19.7699	20.0783	19.7654	20.0320	19.7840	20.4496	**16.39**
**C8`**	35.5076	36.3386	36.5579	36.6501	37.0360	36.6375	37.2208	36.6255	37.1657	36.6643	37.6889	31.0486	31.2767	31.5537	31.4840	31.7595	31.4764	31.8336	31.4681	31.8936	31.4915	31.9753	**32**
**H2**	8.7689	8.7049	8.7244	8.6617	8.6168	8.6654	8.6438	8.6690	8.6389	8.6580	8.6285	8.7980	8.6167	8.6695	8.5340	8.5473	8.5409	8.5118	8.5472	8.5462	8.5267	8.5219	**7.971**
**H3**	7.4063	7.5412	7.6281	7.5846	7.6999	7.5826	7.7160	7.5807	7.7091	7.5865	7.7301	7.3996	7.5311	7.6171	7.5747	7.6929	7.5726	7.7060	7.5705	7.6986	7.5766	7.7206	**7.22**
**H5**	7.2620	7.4507	7.5382	7.5345	7.6567	7.5299	7.6715	7.5253	7.6620	7.5389	7.6941	7.2579	7.4349	7.5221	7.5119	7.6365	7.5077	7.6445	7.5036	7.6350	7.5158	7.6658	**7.22**
**H6**	8.1470	8.3590	8.4476	8.4737	8.5894	8.4673	8.6366	8.4608	8.6223	8.4796	8.6702	7.9705	8.1468	8.2705	8.2373	8.4092	8.2331	8.4121	8.2286	8.4132	8.2409	8.4600	**7.971**
**H8**	4.2201	4.3249	4.3484	4.4341	4.4931	4.4267	4.5388	4.4195	4.5400	4.4412	4.5650	4.3966	4.5587	4.6191	4.6315	4.7523	4.6282	4.7687	4.6248	4.7611	4.6343	4.8001	**5.2**
**H(9A-9C)**	1.3297	1.3390	1.3314	1.3291	1.2821	1.3304	1.2971	1.3316	1.2847	1.3277	1.3077	1.4016	1.4258	1.4289	1.4408	1.4198	1.4402	1.4318	1.4396	1.4375	1.4411	1.4563	**1.611**
**H(8`A-8`C)**	2.5132	2.4524	2.4238	2.4345	2.4170	2.4350	2.4044	2.4357	2.4044	2.4340	2.3914	2.9425	2.7653	2.7314	2.6913	2.6489	2.6974	2.5595	2.7032	2.5861	2.6851	2.5306	**2.82**

[a] methyl protons shifts are averaged

[b] according to [10]

Table S10. Cont. III

Nuclei	R											S											Exp.[b]
	HF																						
	Gas	$CHCl_3$ (PCM)	$CHCl_3$ (SMD)	DMSO (PCM)	DMSO (SMD)	CH_3OH (PCM)	CH_3OH (SMD)	C_2H_5OH (PCM)	C_2H_5OH (SMD)	H_2O (PCM)	H_2O (SMD)	Gas	$CHCl_3$ (PCM)	$CHCl_3$ (SMD)	DMSO (PCM)	DMSO (SMD)	CH_3OH (PCM)	CH_3OH (SMD)	C_2H_5OH (PCM)	C_2H_5OH (SMD)	H_2O (PCM)	H_2O (SMD)	
**C1**	136.0859	136.4286	136.9983	136.5642	138.6394	136.5601	136.8559	136.5607	137.5918	136.5707	137.8340	135.1106	136.0099	136.9165	136.8715	138.6196	136.8298	138.1526	136.7906	138.0357	136.9136	138.1693	**130.99**
**C2**	144.2959	144.1109	144.6184	143.9366	144.2969	143.9493	145.0625	143.9624	144.8336	143.9247	144.9263	144.1862	143.9606	144.4423	143.6963	144.1843	143.7105	144.6238	143.7251	144.5752	143.6812	144.6859	**133.34**
**C3**	120.5425	120.9815	121.3393	121.0997	121.6987	121.0951	121.5914	121.0919	121.6723	121.1042	121.7618	120.5755	121.0153	121.3955	121.1911	121.7425	121.1823	121.7739	121.1741	121.7432	121.1992	121.8101	**117.55**
**C4**	171.0685	171.6891	172.2443	172.0947	172.4686	172.0696	173.4111	172.0452	173.0954	172.1179	173.3624	171.3313	171.6695	172.1524	171.7723	172.2704	171.7643	172.7640	171.7564	172.6801	171.7787	172.9382	**168.13**
**C5**	117.7778	118.9699	119.4399	119.5840	120.3789	119.5486	120.2895	119.5143	120.3140	119.6174	120.5553	117.8113	119.0523	119.5559	119.6851	120.4237	119.6494	120.4603	119.6142	120.3849	119.7186	120.5859	**117.55**
**C6**	139.8710	141.8434	143.0414	142.9329	144.0689	142.8614	145.3610	142.7878	144.7144	142.9974	145.2266	140.2153	142.1535	143.2403	142.9363	144.2865	142.8868	144.8278	142.8360	144.6896	142.9813	145.2440	**133.34**
**C7**	195.4655	201.2748	202.3678	204.5817	204.9342	204.3815	211.4166	204.1898	210.2401	204.7750	212.8652	200.3146	204.7891	205.8595	207.3684	207.7885	207.2162	214.0876	207.0655	212.5722	207.5129	214.7501	**195.87**
**C8**	54.6914	55.6162	56.0452	55.7978	56.6132	55.7884	56.8235	55.7770	56.8761	55.8047	57.6680	49.9453	51.5670	52.1910	52.5059	53.2429	52.4542	53.9705	52.4041	53.6974	52.5554	54.1978	**60.75**
**C9**	20.7069	20.7584	20.8662	20.7421	21.0481	20.7452	20.9072	20.7468	20.8745	20.7388	20.9507	17.7976	17.8056	17.9115	17.8577	18.0665	17.8560	17.9858	17.8550	17.9557	17.8592	18.1883	**16.39**
**C8`**	32.6429	33.3486	33.5660	33.7469	34.0103	33.7215	34.1987	33.6970	34.1551	33.7689	34.5312	29.0930	29.3330	29.5248	29.4519	29.7044	29.4395	29.7559	29.4273	29.7928	29.4622	29.8640	**32**
**H2**	9.0358	8.9576	9.0240	8.9122	8.8795	8.9150	8.9903	8.9172	8.9268	8.9093	8.8935	9.0687	8.8942	8.9168	8.7657	8.7690	8.7719	8.7430	8.7777	8.7685	8.7596	8.7567	**7.971**
**H3**	7.4286	7.5694	7.6559	7.6163	7.7372	7.6140	7.7469	7.6115	7.7419	7.6185	7.7658	7.4270	7.5652	7.6521	7.6122	7.7319	7.6098	7.7417	7.6072	7.7343	7.6145	7.7576	**7.22**
**H5**	7.2620	7.4585	7.5485	7.5471	7.6720	7.5420	7.6831	7.5367	7.6720	7.5520	7.7063	7.2588	7.4515	7.5381	7.5363	7.6607	7.5316	7.6678	7.5266	7.6572	7.5408	7.6908	**7.22**
**H6**	8.1772	8.4177	8.5445	8.5717	8.7053	8.5623	8.8158	8.5526	8.7578	8.5806	8.8070	8.0620	8.2816	8.3955	8.3692	8.5389	8.3639	8.5572	8.3581	8.5485	8.3743	8.6055	**7.971**
**H8**	3.6262	3.7734	3.8337	3.9160	4.0002	3.9073	4.0866	3.8987	4.0448	3.9245	4.0816	3.7998	3.9985	4.0614	4.0795	4.2064	4.0749	4.2416	4.0700	4.2283	4.0838	4.2685	**5.2**
**H(9A-9C)**	1.2094	1.2101	1.2114	1.1856	1.1335	1.1867	1.1817	1.1873	1.1548	1.1845	1.1607	1.2797	1.2815	1.2708	1.2849	1.2453	1.2842	1.2618	1.2832	1.2631	1.2857	1.2803	**1.611**
**H(8`A-8`C)**	2.2583	2.2315	2.1858	2.2178	2.1970	2.2185	2.1768	2.2190	2.1940	2.2172	2.1932	2.6962	2.5446	2.4939	2.4664	2.4243	2.4706	2.3592	2.4746	2.3767	2.4625	2.3361	**2.82**
Nuclei	LC-BLYP																						
**C1**	143.9454	144.3001	145.4097	144.5079	146.3392	144.4682	145.1219	144.4578	146.1915	144.5303	146.2836	142.9429	144.2114	144.8868	144.9354	146.3990	144.8458	146.3781	144.7913	145.9849	145.0111	146.5188	**130.99**
**C2**	148.5022	148.3123	148.9120	148.1799	148.7307	148.1681	149.3075	148.1834	149.1118	148.1736	149.2571	148.2367	148.1595	148.7302	148.0898	148.6521	148.0728	149.1473	148.0835	149.0482	148.0884	149.1787	**133.34**
**C3**	128.3981	128.9432	129.5272	129.1700	129.8249	129.1365	129.9435	129.1302	129.9864	129.1845	130.1049	128.4577	128.9850	129.5383	129.2181	129.8639	129.1821	130.0193	129.1738	129.9481	129.2354	130.0806	**117.55**
**C4**	181.8964	182.5361	183.1128	182.9647	183.4846	182.9173	184.1724	182.8968	183.9350	182.9925	184.2385	182.1104	182.5115	183.1648	182.7723	183.4027	182.7394	183.7928	182.7322	183.7403	182.7849	183.9455	**168.13**
**C5**	126.0329	127.3491	128.0096	128.0921	128.9347	128.0253	129.0515	127.9857	129.0367	128.1384	129.2952	126.1441	127.4212	128.0695	128.1002	128.9526	128.0385	129.0416	128.0038	128.9555	128.1414	129.1882	**117.55**
**C6**	144.4457	146.5586	147.7101	147.7244	149.3132	147.6325	150.0897	147.5676	149.3684	147.7936	150.0269	144.7647	146.8890	148.2216	147.9599	149.7654	147.8834	150.1624	147.8313	150.0717	148.0120	150.5715	**133.34**
**C7**	210.7961	216.2518	217.8303	219.2261	219.8730	219.0191	225.3038	218.8395	225.0653	219.4065	227.1999	215.4470	220.1457	221.5590	222.7455	223.3196	222.5572	228.8329	222.3988	227.4675	222.9103	229.4875	**195.87**
**C8**	61.3984	62.6319	63.4214	63.1097	64.1261	63.0645	64.5480	63.0462	64.4091	63.1347	65.2976	55.6431	57.3143	57.8214	58.2576	59.0058	58.1666	59.8648	58.1066	59.4794	58.3300	60.2244	**60.75**
**C9**	21.5377	21.6825	21.9377	21.8979	22.3180	21.8578	22.1872	21.8462	22.0012	21.9197	22.1735	18.6324	18.8728	18.9378	19.0093	19.2711	18.9777	19.3164	18.9740	19.2407	19.0228	19.6888	**16.39**
**C8`**	33.6021	34.3992	34.7145	34.8359	35.1835	34.7918	35.4065	34.7739	35.3393	34.8597	35.8933	29.0895	29.3897	29.7641	29.6827	29.9584	29.6509	30.0425	29.6443	30.1159	29.6934	30.2365	**32**
**H2**	8.9711	8.8976	8.9038	8.8550	8.8357	8.8575	8.9003	8.8613	8.8099	8.8515	8.8027	8.9912	8.8085	8.8563	8.7154	8.7346	8.7227	8.6856	8.7307	8.7297	8.7068	8.6896	**7.971**
**H3**	7.5427	7.6754	7.7638	7.7201	7.8369	7.7170	7.8517	7.7154	7.8451	7.7221	7.8687	7.5437	7.6708	7.7599	7.7138	7.8323	7.7108	7.8425	7.7093	7.8366	7.7157	7.8586	**7.22**
**H5**	7.4058	7.5954	7.6843	7.6812	7.8049	7.6755	7.8189	7.6712	7.8049	7.6857	7.8395	7.4060	7.5871	7.6767	7.6670	7.7928	7.6617	7.7982	7.6579	7.7897	7.6710	7.8216	**7.22**
**H6**	8.3027	8.5213	8.6081	8.6422	8.7669	8.6342	8.8393	8.6277	8.7770	8.6487	8.8307	8.1441	8.3320	8.4605	8.4236	8.6087	8.4188	8.6040	8.4150	8.6111	8.4268	8.6501	**7.971**
**H8**	4.0994	4.2241	4.2473	4.3375	4.3854	4.3282	4.4499	4.3208	4.4440	4.3453	4.4811	4.3161	4.4914	4.5661	4.5712	4.6984	4.5663	4.7098	4.5628	4.7083	4.5747	4.7424	**5.2**
**H(9A-9C)**	1.3477	1.3582	1.3449	1.3500	1.3227	1.3501	1.3530	1.3517	1.2974	1.3487	1.3187	1.4131	1.4278	1.4251	1.4407	1.4202	1.4395	1.4310	1.4398	1.4374	1.4406	1.4542	**1.611**
**H(8`A-8`C)**	2.5010	2.4391	2.4146	2.4167	2.4026	2.4164	2.3702	2.4179	2.3908	2.4162	2.3818	2.8887	2.7145	2.6860	2.6365	2.5978	2.6411	2.5031	2.6470	2.5362	2.6309	2.4788	**2.82**
Nuclei	M06																						
**C1**	135.4072	135.7741	137.0115	135.8816	138.0210	135.8649	136.8091	135.8563	137.3380	135.9123	137.0556	134.1674	135.5202	136.1558	136.2300	137.8135	136.1538	137.9154	136.0917	137.5984	136.3238	137.9805	**130.99**
**C2**	140.6153	140.3648	140.9748	140.1830	140.7472	140.1863	141.1872	140.1968	141.0177	140.1945	141.1811	139.8441	139.8784	140.4612	139.8960	140.5961	139.8814	141.2124	139.8755	141.0600	139.9255	141.2192	**133.34**
**C3**	121.6176	122.2938	122.9735	122.5729	123.4101	122.5477	123.6473	122.5304	123.5932	122.6111	123.7286	121.6738	122.2553	122.8671	122.5213	123.3123	122.4950	123.5158	122.4767	123.4472	122.5609	123.5784	**117.55**
**C4**	173.1165	173.9963	174.7342	174.4927	175.3210	174.4521	176.0927	174.4196	175.9087	174.5457	176.2448	173.8447	174.2720	175.0646	174.5038	175.3524	174.4860	175.6260	174.4744	175.6126	174.5338	175.7886	**168.13**
**C5**	119.7718	121.0559	121.7752	121.7509	122.7315	121.6989	122.7996	121.6545	122.7382	121.8148	122.9828	119.7041	120.9774	121.6509	121.6232	122.5692	121.5755	122.6711	121.5353	122.6020	121.6826	122.8037	**117.55**
**C6**	136.8085	138.7038	139.7562	139.8590	141.2826	139.7763	142.3109	139.7016	141.8293	139.9522	142.5930	137.1469	139.1678	140.5409	140.2006	141.9881	140.1354	142.4239	140.0763	142.2946	140.2746	142.8394	**133.34**
**C7**	201.8270	206.8005	208.5395	209.3151	210.6085	209.1531	215.3851	208.9988	214.6233	209.4836	216.2344	206.3715	210.6515	211.8254	212.8411	213.6353	212.6921	218.3336	212.5525	217.1976	212.9995	218.7520	**195.87**
**C8**	60.7304	61.8816	62.6495	62.3326	63.4023	62.2980	63.5929	62.2707	63.6589	62.3799	64.4145	54.1844	55.7499	56.3619	56.6790	57.5433	56.6009	58.4113	56.5332	58.0797	56.7709	58.7141	**60.75**
**C9**	22.1450	22.4238	22.6903	22.7400	23.1676	22.7095	23.3573	22.6872	23.2995	22.7835	23.4614	19.4572	19.6907	19.8485	19.8638	20.2293	19.8402	20.3336	19.8248	20.2582	19.9018	20.8431	**16.39**
**C8`**	36.9348	37.6867	37.9199	37.9636	38.3405	37.9372	38.4473	37.9185	38.4095	38.0033	38.9535	31.7679	31.9520	32.3660	32.1758	32.5544	32.1571	32.7194	32.1443	32.7693	32.2063	32.7972	**32**
**H2**	8.7387	8.6519	8.6561	8.6165	8.5956	8.6186	8.6674	8.6211	8.6218	8.6152	8.6525	8.7596	8.5499	8.5974	8.4553	8.4747	8.4624	8.4395	8.4698	8.4663	8.4487	8.4486	**7.971**
**H3**	7.3369	7.4633	7.5604	7.5046	7.6321	7.5024	7.6528	7.5006	7.6377	7.5073	7.6581	7.2874	7.4302	7.5150	7.4793	7.6060	7.4760	7.6356	7.4734	7.6197	7.4830	7.6504	**7.22**
**H5**	7.1579	7.3516	7.4440	7.4353	7.5710	7.4303	7.5755	7.4258	7.5657	7.4405	7.6026	7.1555	7.3358	7.4320	7.4048	7.5377	7.4011	7.5349	7.3979	7.5315	7.4086	7.5553	**7.22**
**H6**	8.2198	8.4571	8.5350	8.5706	8.6748	8.5635	8.7980	8.5570	8.7357	8.5779	8.8111	7.9934	8.1490	8.2703	8.2290	8.4027	8.2251	8.4051	8.2215	8.4052	8.2330	8.4572	**7.971**
**H8**	4.3442	4.4692	4.4989	4.4507	4.6243	4.4542	4.7164	4.4585	4.6836	4.5675	4.7345	4.4477	4.6028	4.6519	4.6647	4.7811	4.6617	4.7907	4.6590	4.7893	4.6679	4.8239	**5.2**
**H(9A-9C)**	1.3018	1.3045	1.2808	1.2978	1.2531	1.2981	1.2815	1.2991	1.2659	1.2981	1.2924	1.3858	1.4182	1.4256	1.4306	1.4133	1.4304	1.4165	1.4305	1.4256	1.4312	1.4437	**1.611**
**H(8`A-8`C)**	2.4763	2.4121	2.3914	2.3956	2.3829	2.3959	2.3669	2.3969	2.3791	2.3959	2.3540	2.9033	2.7432	2.7104	2.6626	2.6248	2.6682	2.5329	2.6741	2.5623	2.6576	2.5047	**2.82**
Nuclei	M06-2X																						
**C1**	153.0917	153.5294	154.5657	153.6798	155.3824	153.6910	154.3824	153.7038	154.6410	153.6636	154.5610	152.3025	153.6253	154.2248	154.4172	155.8548	154.3561	155.9192	154.3050	155.6636	154.4801	156.1304	**130.99**
**C2**	155.1746	155.0543	155.6859	154.8589	155.5556	154.8934	156.0475	154.9292	155.9461	154.8195	156.0984	154.7655	154.7734	155.3565	154.6837	155.4125	154.7088	155.8874	154.7355	155.7791	154.6544	155.9277	**133.34**
**C3**	136.1590	136.8604	137.4721	137.0980	137.8395	137.1065	138.0767	137.1160	138.0006	137.0834	138.1754	136.1934	136.8293	137.4400	137.0599	137.8148	137.0677	137.9965	137.0767	137.9426	137.0459	138.0999	**117.55**
**C4**	186.9866	187.8245	188.4710	188.2660	188.9823	188.2597	189.6225	188.2546	189.4454	188.2657	189.8145	187.4071	187.9865	188.7105	188.2373	189.0085	188.2488	189.3855	188.2602	189.3135	188.2178	189.5659	**168.13**
**C5**	133.5146	135.0251	135.7169	135.8167	136.7899	135.7892	136.9947	135.7628	136.8748	135.8366	137.2061	133.4360	134.8666	135.5636	135.5696	136.5427	135.5491	136.6670	135.5297	136.5791	135.5821	136.8705	**117.55**
**C6**	151.2911	153.5510	154.7995	154.7508	156.6302	154.6981	157.2971	154.6466	156.9660	154.7945	157.7502	151.6315	153.9149	155.3078	154.9928	157.0373	154.9595	157.4443	154.9245	157.2709	155.0146	157.8386	**133.34**
**C7**	218.4206	224.0871	225.6863	227.0228	227.7392	226.8634	233.4654	226.7049	232.2929	227.1679	234.4902	222.3731	227.5635	229.0579	230.3177	230.9202	230.1563	236.7866	230.0003	235.3937	230.4701	237.6501	**195.87**
**C8**	66.1021	67.3894	68.1145	67.8657	68.9271	67.8608	69.3536	67.8567	69.2455	67.8636	70.1061	60.3620	62.0622	62.4883	63.0156	63.8536	62.9637	64.7319	62.9168	64.3967	63.0637	65.0835	**60.75**
**C9**	23.2058	23.4985	23.7070	23.7340	24.1504	23.7389	24.1959	23.7459	24.1017	23.7236	24.3456	20.3942	20.6118	20.6433	20.7319	21.0349	20.7420	21.1263	20.7541	21.0549	20.7172	21.5210	**16.39**
**C8`**	36.0510	36.7555	36.9669	37.1150	37.4247	37.1125	37.6459	37.1106	37.5970	37.1109	38.1632	30.7152	31.1896	31.5368	31.3585	31.6695	31.3831	31.7058	31.4062	31.7955	31.3257	31.8659	**32**
**H2**	9.2412	9.1940	9.2146	9.1667	9.1653	9.1672	9.2317	9.1673	9.2138	9.1663	9.2327	9.2302	9.0921	9.1468	9.0068	9.0354	9.0138	9.0044	9.0198	9.0317	8.9993	9.0042	**7.971**
**H3**	7.8930	8.0315	8.1240	8.0797	8.2018	8.0760	8.2142	8.0720	8.2077	8.0833	8.2275	7.8444	7.9817	8.0724	8.0317	8.1517	8.0277	8.1674	8.0235	8.1599	8.0355	8.1819	**7.22**
**H5**	7.7188	7.9115	8.0040	8.0015	8.1305	7.9950	8.1471	7.9882	8.1342	8.0078	8.1715	7.7205	7.8974	7.9934	7.9760	8.1058	7.9705	8.1095	7.9646	8.1016	7.9813	8.1306	**7.22**
**H6**	8.7195	8.9331	9.0357	9.0522	9.2020	9.0436	9.2622	9.0346	9.2341	9.0605	9.2978	8.5491	8.7357	8.8647	8.8195	9.0069	8.8151	9.0001	8.8099	9.0002	8.8232	9.0400	**7.971**
**H8**	4.2292	4.3451	4.3884	4.4506	4.5168	4.4418	4.5788	4.4328	4.5575	4.4593	4.6093	4.4880	4.6312	4.7027	4.6959	4.8114	4.6915	4.8114	4.6865	4.8097	4.7000	4.8405	**5.2**
**H(9A-9C)**	1.3567	1.3681	1.3531	1.3672	1.3372	1.3663	1.3712	1.3650	1.3600	1.3682	1.3871	1.4261	1.4313	1.4276	1.4367	1.4173	1.4356	1.4268	1.4342	1.4284	1.4376	1.4441	**1.611**
**H(8`A-8`C)**	2.4619	2.4039	2.3762	2.3853	2.3641	2.3849	2.3357	2.3842	2.3446	2.3860	2.3207	2.7873	2.6416	2.6191	2.5775	2.5343	2.5805	2.4563	2.5830	2.4800	2.5745	2.4273	**2.82**

[a] methyl protons shifts are averaged

[b] according to [10]

Table S10. Cont. IV

Nuclei	R											S											Exp. [b]
	mpW1LYP																						
	Gas	$CHCl_3$ (PCM)	$CHCl_3$ (SMD)	DMSO (PCM)	DMSO (SMD)	CH_3OH (PCM)	CH_3OH (SMD)	C_2H_5OH (PCM)	C_2H_5OH (SMD)	H_2O (PCM)	H_2O (SMD)	Gas	$CHCl_3$ (PCM)	$CHCl_3$ (SMD)	DMSO (PCM)	DMSO (SMD)	CH_3OH (PCM)	CH_3OH (SMD)	C_2H_5OH (PCM)	C_2H_5OH (SMD)	H_2O (PCM)	H_2O (SMD)	
**C1**	139.4828	139.9525	140.8951	140.2162	142.1269	140.1880	141.3847	140.1639	141.5911	140.2438	141.6737	138.0239	139.3201	140.0314	140.0519	141.6015	140.0019	141.3526	139.9557	141.2617	140.1006	141.3699	**130.99**
**C2**	140.0654	139.9633	140.5214	139.8120	140.2594	139.8221	140.7067	139.8327	140.5833	139.8000	140.6754	139.3485	139.4849	140.0212	139.4970	140.0614	139.4929	140.6129	139.4906	140.4986	139.4995	140.6006	**133.34**
**C3**	121.9628	122.6121	123.1375	122.8712	123.4386	122.8537	123.7099	122.8374	123.6709	122.8862	123.7449	122.0772	122.6487	123.1165	122.8876	123.4426	122.8714	123.6473	122.8567	123.5970	122.9013	123.6861	**117.55**
**C4**	176.1473	177.0246	177.6646	177.5228	178.1467	177.4890	178.8123	177.4567	178.6743	177.5533	178.9071	176.5361	177.1020	177.7627	177.3919	178.0767	177.3729	178.4887	177.3548	178.3978	177.4074	178.6295	**168.13**
**C5**	119.7588	121.0521	121.6454	121.7767	122.5021	121.7290	122.7447	121.6831	122.6591	121.8202	122.8719	119.9121	121.1021	121.6630	121.7119	122.4355	121.6731	122.5793	121.6353	122.5049	121.7464	122.6956	**117.55**
**C6**	136.3411	138.3279	139.4122	139.4387	140.8254	139.3726	141.4341	139.3066	141.1391	139.4983	141.7648	136.6810	138.7304	140.0172	139.7711	141.3801	139.7081	141.9011	139.6456	141.7184	139.8277	142.2839	**133.34**
**C7**	202.6211	207.7782	209.3145	210.5766	211.6308	210.3901	217.0461	210.2089	216.1478	210.7558	217.9713	207.9684	212.1537	213.3072	214.4009	215.1623	214.2635	219.8843	214.1282	218.8057	214.5296	220.3958	**195.87**
**C8**	64.8087	65.9413	66.6307	66.2710	67.2010	66.2555	67.4970	66.2398	67.3414	66.2823	68.2675	59.1606	61.1502	61.8125	62.1638	63.0102	62.0972	63.6763	62.0334	63.4471	62.2269	63.9055	**60.75**
**C9**	23.0194	23.2782	23.4066	23.5626	23.7640	23.5450	23.7639	23.5280	23.6835	23.5763	23.8872	19.5716	19.9981	20.1757	20.2377	20.4306	20.2213	20.5847	20.2064	20.5225	20.2514	20.9210	**16.39**
**C8`**	36.3159	37.0893	37.3474	37.4110	37.7421	37.3893	37.9553	37.3691	37.9323	37.4299	38.3577	31.9503	32.0958	32.3503	32.2714	32.4808	32.2555	32.5948	32.2408	32.6120	32.2842	32.6749	**32**
**H2**	8.6760	8.6134	8.6345	8.5731	8.5352	8.5769	8.5767	8.5801	8.5650	8.5692	8.5537	8.7155	8.5439	8.5843	8.4575	8.4611	8.4631	8.4509	8.4681	8.4656	8.4518	8.4608	**7.971**
**H3**	7.3397	7.4720	7.5610	7.5164	7.6269	7.5145	7.6429	7.5123	7.6350	7.5181	7.6539	7.3222	7.4540	7.5397	7.5009	7.6151	7.4987	7.6299	7.4961	7.6226	7.5029	7.6422	**7.22**
**H5**	7.1905	7.3768	7.4660	7.4635	7.5825	7.4586	7.6016	7.4535	7.5914	7.4679	7.6207	7.1791	7.3532	7.4411	7.4319	7.5518	7.4276	7.5619	7.4231	7.5524	7.4357	7.5804	**7.22**
**H6**	8.0834	8.2895	8.3770	8.4032	8.5186	8.3966	8.5837	8.3898	8.5634	8.4092	8.6049	7.8914	8.0614	8.1768	8.1478	8.3090	8.1432	8.3269	8.1382	8.3161	8.1519	8.3729	**7.971**
**H8**	4.3063	4.3981	4.4252	4.5055	4.5605	4.4978	4.6229	4.4902	4.6105	4.5128	4.6327	4.4564	4.6192	4.6720	4.6856	4.7973	4.6823	4.8170	4.6786	4.8046	4.6884	4.8463	**5.2**
**H(9A-9C)**	1.3216	1.3308	1.3202	1.3215	1.2774	1.3232	1.2903	1.3245	1.2801	1.3196	1.3009	1.4008	1.4361	1.4422	1.4560	1.4325	1.4551	1.4529	1.4538	1.4517	1.4567	1.4756	**1.611**
**H(8`A-8`C)**	2.5203	2.4606	2.4330	2.4451	2.4238	2.4459	2.4070	2.4465	2.4101	2.4443	2.3934	2.9829	2.8104	2.7656	2.7300	2.6802	2.7354	2.5986	2.7403	2.6192	2.7247	2.5674	**2.82**
Nuclei	**mpW1PW91**																						
**C1**	136.7091	137.1261	138.2904	137.3527	139.3925	137.3342	138.8484	137.3222	138.8794	137.3708	139.0868	135.4965	136.8443	137.3776	137.4319	138.8932	137.3747	138.9047	137.3297	138.6184	137.4927	138.9966	**130.99**
**C2**	139.0510	138.9813	139.5567	138.8023	139.3841	138.8154	139.7972	138.8338	139.6690	138.7889	139.7849	138.4920	138.6376	139.1844	138.5926	139.2587	138.5931	139.7425	138.5998	139.6330	138.5920	139.7648	**133.34**
**C3**	121.1434	121.8839	122.4572	122.1398	122.8577	122.1259	123.1111	122.1175	123.0669	122.1523	123.1972	121.1969	121.8835	122.4042	122.1282	122.8348	122.1142	123.0503	122.1059	122.9791	122.1411	123.1290	**117.55**
**C4**	173.1450	174.0361	174.6361	174.4798	175.1382	174.4530	175.7414	174.4320	175.6481	174.5044	175.9056	173.4356	174.0253	174.6981	174.2966	175.0454	174.2852	175.4189	174.2781	175.3524	174.3057	175.5825	**168.13**
**C5**	118.9666	120.3546	120.9855	121.0745	121.9413	121.0305	122.1805	120.9922	122.0946	121.1155	122.3576	119.0635	120.3480	120.9568	120.9724	121.8462	120.9359	121.9978	120.9051	121.9093	121.0057	122.1526	**117.55**
**C6**	135.1188	137.1393	138.1020	138.2106	139.6150	138.1465	140.1616	138.0877	139.9062	138.2707	140.5379	135.4925	137.5198	138.7879	138.5089	140.2202	138.4556	140.6265	138.4058	140.5120	138.5558	141.0214	**133.34**
**C7**	200.5499	205.4732	207.1087	208.0696	209.2352	207.9079	214.4321	207.7523	213.5361	208.2238	215.3863	204.9687	209.2878	210.4288	211.5065	212.2688	211.3693	217.0803	211.2400	215.8598	211.6406	217.5855	**195.87**
**C8**	62.5871	63.7277	64.3921	64.0779	65.0594	64.0579	65.5692	64.0437	65.2428	64.0959	66.2934	57.3088	59.1083	59.5712	59.9715	60.7733	59.9036	61.5602	59.8450	61.2368	60.0391	61.8431	**60.75**
**C9**	22.4303	22.7074	22.8165	22.9714	23.2346	22.9561	23.2515	22.9463	23.1372	22.9849	23.3928	19.1269	19.5971	19.6794	19.7761	20.0297	19.7623	20.1776	19.7543	20.1086	19.7888	20.5773	**16.39**
**C8`**	35.5389	36.3291	36.5286	36.6000	36.9580	36.5839	37.1500	36.5735	37.1332	36.6149	37.6110	31.0253	31.2540	31.5589	31.4502	31.7343	31.4412	31.7914	31.4363	31.8597	31.4556	31.8914	**32**
**H2**	8.7270	8.6729	8.6666	8.6268	8.5829	8.6306	8.6029	8.6343	8.6110	8.6233	8.5930	8.7399	8.5718	8.6280	8.4933	8.5106	8.5006	8.4771	8.5073	8.5099	8.4860	8.4856	**7.971**
**H3**	7.3666	7.5071	7.5941	7.5506	7.6672	7.5488	7.6842	7.5469	7.6761	7.5526	7.6994	7.3521	7.4918	7.5782	7.5379	7.6587	7.5357	7.6748	7.5336	7.6662	7.5401	7.6902	**7.22**
**H5**	7.2156	7.4099	7.4980	7.4952	7.6199	7.4906	7.6379	7.4860	7.6293	7.4999	7.6618	7.2035	7.3869	7.4755	7.4658	7.5930	7.4616	7.6047	7.4575	7.5936	7.4700	7.6265	**7.22**
**H6**	8.1720	8.3810	8.4466	8.4858	8.5927	8.4800	8.6356	8.4742	8.6335	8.4916	8.6714	7.9622	8.1369	8.2584	8.2244	8.3946	8.2208	8.3985	8.2168	8.3988	8.2277	8.4460	**7.971**
**H8**	4.3244	4.4232	4.4417	4.5212	4.5686	4.5149	4.6073	4.5088	4.6209	4.5275	4.6357	4.4958	4.6417	4.6998	4.7058	4.8233	4.7033	4.8370	4.7007	4.8311	4.7081	4.8693	**5.2**
**H(9A-9C)**	1.3031	1.3123	1.2861	1.3005	1.2512	1.3018	1.2673	1.3032	1.2532	1.2994	1.2798	1.3839	1.4107	1.4119	1.4245	1.4007	1.4244	1.4147	1.4241	1.4196	1.4248	1.4408	**1.611**
**H(8`A-8`C)**	2.4582	2.3926	2.3693	2.3752	2.3559	2.3760	2.3459	2.3770	2.3405	2.3747	2.3305	2.8765	2.7109	2.6756	2.6345	2.5913	2.6410	2.5022	2.6472	2.5276	2.6285	2.4719	**2.82**
Nuclei	**O3LYP**																						
**C1**	133.9706	135.2869	135.1280	135.8992	137.5413	135.8935	136.8634	135.8840	136.9936	135.9000	136.9568	131.9841	134.0269	134.9667	134.8776	136.4625	134.8098	136.5165	134.7451	136.4801	134.9454	136.6774	**130.99**
**C2**	134.2294	134.0386	134.6059	134.0020	134.6215	134.0143	135.0928	134.0931	135.0297	133.9844	135.0347	133.6011	133.8315	134.4861	133.8598	134.5658	133.8686	134.9908	133.8725	134.9155	133.8449	134.9967	**133.34**
**C3**	117.1429	117.8472	118.4244	118.1586	118.8498	118.1512	119.1048	118.1420	119.0383	118.1592	119.1705	117.2423	117.8329	118.4008	118.1462	118.7940	118.1374	118.9776	118.1236	118.9199	118.1480	119.0503	**117.55**
**C4**	169.3331	170.2178	170.9930	170.6800	171.4504	170.6575	171.9898	170.6246	171.8402	170.6953	172.1262	169.6935	170.1374	170.8394	170.4612	171.2113	170.4568	171.5048	170.4458	171.4066	170.4567	171.6400	**168.13**
**C5**	115.1244	116.3261	117.0316	116.9791	117.7550	116.9488	117.9702	116.9062	117.8594	117.0010	118.1340	115.2829	116.3481	116.9834	116.9736	117.7506	116.9474	117.8633	116.9154	117.7787	116.9914	118.0028	**117.55**
**C6**	130.1343	131.8403	133.2468	132.9143	134.4244	132.8471	134.9826	132.7768	134.7083	132.9717	135.3818	130.8195	132.4740	133.5676	133.4119	134.9398	133.3757	135.3121	133.3309	135.1128	133.4364	135.6140	**133.34**
**C7**	196.4324	202.4039	202.9208	205.2799	206.3184	205.1284	211.0569	204.9884	210.1352	205.4208	211.6531	200.9322	205.3707	206.9113	207.7346	208.6887	207.5898	212.9882	207.4429	212.0350	207.8698	213.5094	**195.87**
**C8**	62.7269	63.8638	64.1933	64.6392	65.2111	64.6247	65.7186	64.6144	65.5898	64.6448	66.4308	57.3727	59.9629	60.5156	61.0054	61.8178	60.9369	62.7983	60.8688	62.5812	61.0698	63.0624	**60.75**
**C9**	21.9077	22.1497	22.4381	22.4448	22.6922	22.4333	22.8050	22.4257	22.7348	22.4497	22.9545	18.3907	18.7982	18.9135	19.0083	19.2082	19.0039	19.4486	18.9953	19.3637	19.0069	19.7532	**16.39**
**C8`**	35.8615	36.6415	36.9984	37.0897	37.4384	37.0783	37.5559	37.0769	37.5415	37.0931	37.8889	31.6791	31.7885	32.1081	32.0205	32.3175	32.0201	32.2296	32.0132	32.2550	32.0126	32.2365	**32**
**H2**	8.5171	8.3815	8.5110	8.3059	8.3131	8.3086	8.3569	8.3167	8.3480	8.3030	8.3618	8.6115	8.3742	8.4056	8.2874	8.3033	8.2956	8.2770	8.3028	8.2844	8.2784	8.2790	**7.971**
**H3**	7.2896	7.4098	7.5061	7.4543	7.5768	7.4521	7.6030	7.4468	7.5953	7.4562	7.6133	7.2766	7.4071	7.4943	7.4552	7.5707	7.4530	7.5845	7.4505	7.5766	7.4572	7.5999	**7.22**
**H5**	7.1552	7.3290	7.4298	7.3986	7.5094	7.3950	7.5202	7.3889	7.5085	7.4017	7.5463	7.1395	7.3009	7.3898	7.3774	7.4980	7.3735	7.5074	7.3692	7.4968	7.3810	7.5278	**7.22**
**H6**	8.0527	8.2175	8.3672	8.3017	8.4640	8.2946	8.5029	8.2853	8.4809	8.3083	8.5390	7.8574	7.9837	8.0812	8.0642	8.2140	8.0612	8.2127	8.0575	8.2001	8.0664	8.2508	**7.971**
**H8**	4.4166	4.5098	4.5410	4.5642	4.6714	4.5583	4.6852	4.5532	4.6658	4.5699	4.7170	4.5458	4.6793	4.7411	4.7470	4.8642	4.7444	4.8665	4.7410	4.8556	4.7490	4.8955	**5.2**
**H(9A-9C)**	1.2321	1.2056	1.2312	1.2013	1.1647	1.2018	1.1980	1.2027	1.1895	1.2008	1.2166	1.3321	1.3605	1.3485	1.3797	1.3521	1.3795	1.3771	1.3787	1.3725	1.3796	1.3957	**1.611**
**H(8`A-8`C)**	2.4772	2.4452	2.4019	2.4401	2.4019	2.4414	2.3990	2.4451	2.4021	2.4388	2.3752	2.9410	2.7694	2.7347	2.6983	2.6563	2.7041	2.5681	2.7092	2.5820	2.6922	2.5313	**2.82**
Nuclei	**OLYP**																						
**C1**	132.8188	134.1516	135.6656	135.1449	137.5235	135.1515	135.6652	135.1532	135.8434	135.1337	135.7556	130.8981	132.7261	133.7888	133.6768	135.1782	133.6081	135.1883	133.5382	135.1931	133.7379	135.3980	**130.99**
**C2**	131.6623	131.6975	132.2794	131.7465	132.3472	131.7531	132.6913	131.7546	132.6220	131.7302	132.7039	131.2536	131.3661	132.0518	131.4162	132.0990	131.4235	132.5894	131.4250	132.5059	131.4005	132.5752	**133.34**
**C3**	115.3417	116.0422	116.6555	116.3161	116.9608	116.3056	117.2576	116.2899	117.1915	116.3169	117.3369	114.4114	116.0541	116.6230	116.3750	116.9979	116.3650	117.1604	116.3492	117.1047	116.3752	117.2329	**117.55**
**C4**	168.2126	169.2294	169.9205	169.5710	170.1814	169.5487	170.9191	169.5215	170.7744	169.5840	171.0771	168.4577	169.1151	169.8267	169.4427	170.2413	169.4360	170.4899	169.4224	170.3927	169.4386	170.6134	**168.13**
**C5**	113.3427	114.4633	115.0535	115.0655	115.7600	115.0339	116.1215	114.9960	116.0017	115.0864	116.2699	113.4100	114.5734	115.1849	115.1704	115.9016	115.1450	116.0297	115.1131	115.9473	115.1848	116.1602	**117.55**
**C6**	127.6886	129.4580	130.3992	130.3959	131.5991	130.3259	132.5021	130.2522	132.2325	130.4552	132.8719	132.4882	129.9940	131.1063	130.8821	132.4316	130.8446	132.8458	130.8002	132.6433	130.9066	133.1053	**133.34**
**C7**	195.3251	201.0301	202.9899	204.5243	206.1991	204.3850	209.0998	204.2443	208.2692	204.6535	209.6663	194.9333	204.0205	205.4470	206.3531	207.3591	206.2114	211.0678	206.0639	210.2799	206.4776	211.6073	**195.87**
**C8**	63.8385	65.2339	66.0677	65.6811	66.8369	65.6883	66.9203	65.6854	66.8721	65.6665	67.5606	62.8816	61.2250	62.0414	62.3546	63.1411	62.2822	64.2114	62.2081	64.0080	62.4201	64.4616	**60.75**
**C9**	22.0417	22.3211	22.5251	22.7061	22.8886	22.6962	23.1868	22.6811	23.0909	22.7070	23.3016	15.7972	18.8615	19.1193	19.1190	19.3070	19.1099	19.6432	19.0962	19.5371	19.1202	19.9486	**16.39**
**C8`**	36.5297	37.3375	37.5614	37.8176	38.0745	37.8036	38.1673	37.7827	38.1190	37.8211	38.5013	31.4966	32.4077	32.6511	32.6378	32.9272	32.6373	32.7834	32.6299	32.8079	32.6276	32.7561	**32**
**H2**	8.3522	8.2235	8.1969	8.1272	8.0714	8.1268	8.2234	8.1264	8.2066	8.1267	8.2299	8.4369	8.2352	8.2566	8.1468	8.1702	8.1543	8.1611	8.1613	8.1618	8.1388	8.1574	**7.971**
**H3**	7.2072	7.3275	7.4178	7.4008	7.5127	7.3974	7.5330	7.3939	7.5211	7.4035	7.5445	7.1593	7.3325	7.4196	7.3792	7.4934	7.3770	7.5033	7.3746	7.4956	7.3809	7.5175	**7.22**
**H5**	7.0851	7.2353	7.3143	7.2988	7.4157	7.2941	7.4375	7.2893	7.4251	7.3029	7.4576	7.0403	7.2266	7.3145	7.2993	7.4188	7.2955	7.4288	7.2915	7.4188	7.3025	7.4474	**7.22**
**H6**	7.9775	8.1327	8.1956	8.2132	8.2977	8.2041	8.4009	8.1954	8.3698	8.2217	8.4374	8.7484	7.8865	7.9743	7.9576	8.1089	7.9547	8.1075	7.9514	8.0939	7.9595	8.1417	**7.971**
**H8**	4.5323	4.5911	4.6096	4.6267	4.6824	4.6200	4.7353	4.6144	4.7106	4.6332	4.7707	4.4883	4.7554	4.8166	4.8221	4.9436	4.8190	4.9419	4.8155	4.9305	4.8243	4.9712	**5.2**
**H(9A-9C)**	1.2237	1.2082	1.1959	1.1823	1.1420	1.1826	1.2055	1.1826	1.1992	1.1815	1.2218	1.2466	1.3653	1.3601	1.3854	1.3618	1.3848	1.3978	1.3840	1.3909	1.3856	1.4134	**1.611**
**H(8`A-8`C)**	2.5179	2.4873	2.4697	2.4886	2.4649	2.4895	2.4510	2.4902	2.4526	2.4873	2.4211	2.3523	2.8150	2.7687	2.7435	2.7024	2.7489	2.6101	2.7538	2.6211	2.7376	2.5695	**2.82**

[a] methyl protons shifts are averaged

[b] according to [10]

Table S10. Cont. V

Nuclei	R												S												Exp. [b]
	OPBE																								
	Gas	$CHCl_3$ (PCM)	$CHCl_3$ (SMD)	DMSO (PCM)	DMSO (SMD)	CH_3OH (PCM)	CH_3OH (SMD)	C_2H_5OH (PCM)	C_2H_5OH (SMD)	H_2O (PCM)	H_2O (SMD)	Gas	$CHCl_3$ (PCM)	$CHCl_3$ (SMD)	DMSO (PCM)	DMSO (SMD)	CH_3OH (PCM)	CH_3OH (SMD)	C_2H_5OH (PCM)	C_2H_5OH (SMD)	H_2O (PCM)	H_2O (SMD)	
**C1**	129.7640	131.1387	132.6552	132.1246	134.4950	132.1378	132.7494	132.1341	132.8307	132.0968	132.7529	127.9531	129.7572	131.3960	130.9073	132.9633	130.8138	132.2249	130.6965	132.6314	130.9636	132.5011	**130.99**
**C2**	130.3586	130.4886	130.9968	130.4429	131.0543	130.4635	131.3872	130.4661	131.3319	130.3820	131.3838	129.9865	130.2359	130.9157	130.1823	130.9380	130.1990	131.3075	130.1994	131.2030	130.1628	131.3207	**133.34**
**C3**	114.2213	115.0550	115.6259	115.3016	116.0144	115.3021	116.3293	115.2846	116.2561	115.3063	116.4542	114.3041	115.0698	115.5900	115.3365	116.0072	115.3394	116.2309	115.3240	116.1537	115.3295	116.3409	**117.55**
**C4**	164.6226	165.6789	166.2199	165.9412	166.5402	165.9340	167.2498	165.9090	167.1104	166.0101	167.4931	164.9027	165.5555	166.0292	165.7368	166.3569	165.7530	166.8192	165.7519	166.6262	165.7218	166.9648	**168.13**
**C5**	112.2687	113.5344	114.0760	114.0744	114.8381	114.0527	115.1922	114.0131	115.0739	114.1130	115.4008	112.4202	113.6143	114.1128	114.1369	114.8497	114.1271	115.0869	114.0994	114.9493	114.1433	115.2524	**117.55**
**C6**	126.1608	127.9997	128.8093	128.8180	129.9411	128.7696	130.8401	128.7038	130.6005	128.8489	131.2575	126.8203	128.5704	129.4079	129.2674	130.6027	129.2570	131.2141	129.2292	130.8690	129.2803	131.4889	**133.34**
**C7**	192.8626	198.3520	200.1588	201.6561	203.2107	201.5462	206.1754	201.4188	205.3233	201.7358	206.5747	196.3497	200.6868	202.1872	203.0429	204.1492	202.9127	207.7285	202.7568	207.1557	203.1474	208.2542	**195.87**
**C8**	61.2284	62.7133	63.5636	63.1881	64.3519	63.1850	64.4468	63.1637	64.3074	63.1720	65.1108	56.6311	58.7512	59.9248	59.9517	61.0884	59.8539	61.5973	59.7391	61.6414	60.0230	61.9029	**60.75**
**C9**	21.2805	21.6678	21.7922	21.9610	22.1440	21.9624	22.3933	21.9464	22.3075	21.9463	22.5303	17.8291	18.2838	18.5005	18.5007	18.7647	18.4933	18.9349	18.4692	18.8763	18.4996	19.2950	**16.39**
**C8`**	35.4845	36.3569	36.4549	36.7323	36.9249	36.7278	37.0000	36.7052	36.9687	36.7163	37.3820	31.0988	31.3654	31.3318	31.4174	31.5251	31.4408	31.6115	31.4469	31.5375	31.3965	31.5941	**32**
**H2**	8.3870	8.2642	8.2215	8.1564	8.0952	8.1575	8.2408	8.1577	8.2328	8.1562	8.2594	8.4575	8.2769	8.2227	8.1564	8.1258	8.1678	8.1855	8.1792	8.1490	8.1477	8.1810	**7.971**
**H3**	7.2282	7.3602	7.4433	7.4277	7.5438	7.4252	7.5609	7.4218	7.5508	7.4327	7.5779	7.2304	7.3678	7.4497	7.4149	7.5264	7.4130	7.5438	7.4104	7.5341	7.4158	7.5608	**7.22**
**H5**	7.1021	7.2653	7.3426	7.3260	7.4471	7.3219	7.4668	7.3170	7.4549	7.3330	7.4935	7.0869	7.2574	7.3371	7.3302	7.4448	7.3268	7.4602	7.3227	7.4473	7.3327	7.4817	**7.22**
**H6**	8.0380	8.1929	8.2297	8.2560	8.3210	8.2498	8.4322	8.2430	8.4104	8.2608	8.4821	7.8021	7.9511	7.9981	8.0067	8.1209	8.0068	8.1616	8.0062	8.1245	8.0072	8.1968	**7.971**
**H8**	4.5419	4.6115	4.6071	4.6376	4.6660	4.6345	4.7386	4.6308	4.7174	4.6371	4.7816	4.6657	4.7892	4.8168	4.8395	4.9337	4.8401	4.9559	4.8394	4.9298	4.8389	4.9886	**5.2**
**H(9A-9C)**	1.1827	1.1710	1.1506	1.1424	1.0922	1.1427	1.1573	1.1423	1.1499	1.1403	1.1786	1.2938	1.3294	1.3061	1.3393	1.2986	1.3395	1.3428	1.3392	1.3289	1.3389	1.3621	**1.611**
**H(8`A-8`C)**	2.4371	2.4087	2.3861	2.4045	2.3742	2.4058	2.3612	2.4063	2.3625	2.4000	2.3311	2.8529	2.7064	2.6163	2.6177	2.5447	2.6266	2.4933	2.6347	2.4903	2.6099	2.4549	**2.82**
Nuclei	OPW91																								
**C1**	130.0734	131.4711	132.9769	132.4406	134.8087	132.4451	133.0129	132.4321	133.1402	132.4191	133.0519	128.2394	130.0724	131.7005	131.1724	133.2463	131.0761	132.5216	130.9590	132.9078	131.2337	132.7953	**130.99**
**C2**	130.5806	130.7074	131.2089	130.6288	131.2637	130.6390	131.5719	130.6356	131.5478	130.6007	131.6048	130.1962	130.4533	131.1259	130.4042	131.1441	130.4116	131.5201	130.4034	131.4168	130.3802	131.5359	**133.34**
**C3**	114.4183	115.2508	115.8151	115.5092	116.1968	115.4994	116.5211	115.4732	116.4393	115.4999	116.6391	114.5029	115.2650	115.7792	115.5361	116.1909	115.5285	116.4136	115.5035	116.3378	115.5247	116.5251	**117.55**
**C4**	165.0246	166.0821	166.6217	166.4195	166.9443	166.4003	167.7080	166.3664	167.5188	166.4192	167.9027	165.3169	165.9654	166.4385	166.1639	166.7698	166.1681	167.2288	166.1553	167.0408	166.1434	167.3773	**168.13**
**C5**	112.4634	113.7286	114.2665	114.2944	115.0217	114.2618	115.3914	114.2128	115.2585	114.3067	115.5871	112.6210	113.8117	114.3056	114.3432	115.0398	114.3225	115.2744	114.2843	115.1397	114.3450	115.4419	**117.55**
**C6**	126.3826	128.2204	129.0301	129.0438	130.1652	128.9844	131.0735	128.9103	130.8310	129.0819	131.4938	127.0499	128.7969	129.6351	129.5203	130.8358	129.4965	131.4451	129.4563	131.1082	129.5270	131.7238	**133.34**
**C7**	193.2195	198.7503	200.5473	202.0469	203.6032	201.9273	206.5400	201.7965	205.7249	202.1429	206.9853	196.7445	201.1005	202.5918	203.4416	204.5479	203.3022	208.1416	203.1393	207.5501	203.5465	208.6706	**195.87**
**C8**	61.4164	62.9130	63.7519	63.3730	64.5345	63.3605	64.5943	63.3262	64.4929	63.3665	65.2823	56.7828	58.9381	60.1067	60.1087	61.2534	60.0077	61.7812	59.8918	61.8124	60.1818	62.0859	**60.75**
**C9**	21.3412	21.7318	21.8564	22.0225	22.2056	22.0139	22.4453	21.9905	22.3695	22.0127	22.5934	17.9020	18.3623	18.5810	18.5780	18.8386	18.5624	19.0151	18.5310	18.9558	18.5743	19.3737	**16.39**
**C8`**	35.5474	36.4260	36.5197	36.7969	36.9960	36.7816	37.0617	36.7522	37.0400	36.7928	37.4582	31.1915	31.4588	31.4241	31.5288	31.6223	31.5399	31.7046	31.5341	31.6351	31.5020	31.6928	**32**
**H2**	8.3887	8.2639	8.2222	8.1590	8.0961	8.1592	8.2467	8.1596	8.2338	8.1570	8.2615	8.4616	8.2780	8.2245	8.1636	8.1292	8.1736	8.1874	8.1840	8.1527	8.1537	8.1831	**7.971**
**H3**	7.2300	7.3612	7.4442	7.4326	7.5443	7.4293	7.5651	7.4261	7.5509	7.4339	7.5785	7.2318	7.3686	7.4505	7.4163	7.5267	7.4137	7.5442	7.4109	7.5345	7.4169	7.5610	**7.22**
**H5**	7.1042	7.2672	7.3446	7.3319	7.4481	7.3269	7.4705	7.3219	7.4560	7.3348	7.4945	7.0894	7.2592	7.3388	7.3327	7.4461	7.3286	7.4614	7.3242	7.4485	7.3348	7.4827	**7.22**
**H6**	8.0365	8.1901	8.2278	8.2553	8.3202	8.2483	8.4351	8.2419	8.4096	8.2601	8.4824	7.8035	7.9505	7.9980	8.0099	8.1218	8.0088	8.1616	8.0074	8.1258	8.0096	8.1969	**7.971**
**H8**	4.5413	4.6099	4.6067	4.6350	4.6660	4.6313	4.7388	4.6276	4.7179	4.6370	4.7820	4.6622	4.7853	4.8138	4.8390	4.9316	4.8382	4.9531	4.8368	4.9279	4.8379	4.9856	**5.2**
**H(9A-9C)**	1.1844	1.1724	1.1522	1.1437	1.0939	1.1434	1.1586	1.1424	1.1520	1.1425	1.1806	1.2943	1.3301	1.3074	1.3418	1.3002	1.3413	1.3445	1.3406	1.3308	1.3411	1.3636	**1.611**
**H(8`A-8`C)**	2.4383	2.4106	2.3882	2.4045	2.3762	2.4051	2.3601	2.4055	2.3644	2.4024	2.3329	2.8575	2.7104	2.6209	2.6250	2.5505	2.6328	2.4979	2.6400	2.4956	2.6165	2.4597	**2.82**
Nuclei	PBE																								
**C1**	135.5369	136.0918	137.1653	136.3677	138.3715	136.3432	137.9723	136.3302	138.0000	136.4097	138.2666	133.9549	135.5103	136.0530	136.0986	137.6401	136.0426	137.7558	136.0033	137.5912	136.1760	137.8979	**130.99**
**C2**	135.9657	136.0179	136.6216	135.7847	136.4967	135.7983	136.9066	135.8222	136.8094	135.7890	136.9073	135.2241	135.5670	136.1193	135.5057	136.3042	135.5028	136.8654	135.5121	136.7580	135.5269	136.8854	**133.34**
**C3**	119.4465	120.3299	120.8882	120.5608	121.3376	120.5455	121.6570	120.5407	121.5966	120.5927	121.7624	119.5345	120.3283	120.8574	120.5295	121.3174	120.5157	121.5667	120.5123	121.4938	120.5600	121.6702	**117.55**
**C4**	173.9823	175.0866	175.7504	175.5136	176.3815	175.4853	176.9469	175.4679	176.8322	175.5580	177.1272	174.3115	175.1027	175.8451	175.3569	176.3062	175.3425	176.6457	175.3376	176.5574	175.3868	176.8245	**168.13**
**C5**	117.3377	118.8348	119.4453	119.5117	120.4263	119.4676	120.7320	119.4345	120.6335	119.5712	120.9231	117.5420	118.8521	119.4821	119.4030	120.3643	119.3686	120.5554	119.3443	120.4643	119.4527	120.7344	**117.55**
**C6**	132.4775	134.5082	135.5902	135.4775	137.1352	135.4145	137.6377	135.3620	137.3771	135.5552	138.0465	132.8577	134.9572	136.2454	135.8915	137.7657	135.8360	138.2041	135.7900	138.0185	135.9600	138.5976	**133.34**
**C7**	200.2846	204.9361	206.5139	207.3187	208.7358	207.1695	213.0970	207.0319	212.3370	207.4784	214.0538	205.2335	209.1883	210.4404	211.1454	212.2526	211.0237	216.2129	210.9132	215.2932	211.2804	216.6930	**195.87**
**C8**	66.5088	67.7352	68.5365	68.0186	69.2245	67.9992	69.7030	67.9905	69.4643	68.0544	70.5121	60.8379	62.8084	63.3380	63.6487	64.6563	63.5816	65.3895	63.5289	65.1438	63.7343	65.6928	**60.75**
**C9**	23.3476	23.7914	23.9606	24.0315	24.4395	24.0128	24.5728	24.0048	24.4653	24.0668	24.7581	19.9739	20.6830	20.7908	20.8644	21.2357	20.8478	21.5194	20.8429	21.4105	20.8983	22.0261	**16.39**
**C8`**	37.1172	37.9328	38.1214	38.1135	38.5395	38.1010	38.7049	38.0990	38.6815	38.1431	39.1975	32.1770	32.4188	32.7113	32.5246	32.8859	32.5166	32.9628	32.5184	32.9920	32.5484	33.0440	**32**
**H2**	8.4940	8.4541	8.4552	8.4051	8.3713	8.4089	8.3990	8.4131	8.4018	8.4020	8.3885	8.5295	8.3765	8.4273	8.2992	8.3159	8.3056	8.2934	8.3120	8.3107	8.2932	8.2994	**7.971**
**H3**	7.2033	7.3447	7.4313	7.3857	7.5032	7.3838	7.5208	7.3823	7.5132	7.3881	7.5355	7.1915	7.3278	7.4143	7.3701	7.4921	7.3680	7.5036	7.3664	7.4955	7.3727	7.5186	**7.22**
**H5**	7.0554	7.2476	7.3344	7.3285	7.4528	7.3239	7.4719	7.3198	7.4630	7.3335	7.4950	7.0514	7.2254	7.3152	7.2983	7.4275	7.2942	7.4386	7.2906	7.4284	7.3027	7.4597	**7.22**
**H6**	8.0650	8.2613	8.3213	8.3484	8.4499	8.3435	8.4938	8.3391	8.4841	8.3536	8.5258	7.8075	7.9693	8.0875	8.0493	8.2174	8.0456	8.2265	8.0420	8.2172	8.0532	8.2729	**7.971**
**H8**	4.5942	4.6682	4.6640	4.7434	4.7783	4.7384	4.8154	4.7339	4.8140	4.7488	4.8411	4.7365	4.8721	4.9225	4.9285	5.0427	4.9260	5.0540	4.9238	5.0416	4.9312	5.0891	**5.2**
**H(9A-9C)**	1.3652	1.3737	1.3620	1.3582	1.3268	1.3594	1.3445	1.3610	1.3344	1.3577	1.3585	1.4620	1.4973	1.5000	1.5101	1.4967	1.5097	1.5158	1.5096	1.5145	1.5110	1.5442	**1.611**
**H(8`A-8`C)**	2.5658	2.4898	2.4677	2.4656	2.4518	2.4665	2.4330	2.4680	2.4322	2.4654	2.4135	3.0023	2.8324	2.7952	2.7516	2.7069	2.7580	2.6137	2.7646	2.6329	2.7457	2.5792	**2.82**
Nuclei	PBE0																								
**C1**	136.2247	136.6526	137.6494	136.8626	138.8179	136.8401	138.3389	136.8206	138.4202	136.8878	138.5165	135.0705	136.3670	136.9426	136.9688	138.3856	136.9063	138.4604	136.8512	138.1253	137.0380	138.5078	**130.99**
**C2**	138.7455	138.6972	139.2899	138.5224	139.1126	138.5294	139.6279	138.5381	139.4524	138.5187	139.5604	138.2240	138.3806	138.9274	138.3295	138.9750	138.3256	139.5396	138.3238	139.3720	138.3367	139.5045	**133.34**
**C3**	120.8925	121.6540	122.2093	121.9097	122.6105	121.8907	122.9447	121.8736	122.8377	121.9304	122.9696	120.9444	121.6525	122.1782	121.8956	122.5853	121.8764	122.8788	121.8592	122.7522	121.9168	122.9010	**117.55**
**C4**	172.7695	173.6677	174.2592	174.1060	174.7504	174.0748	175.4368	174.0455	175.2494	174.1384	175.5356	173.0167	173.6449	174.3079	173.9164	174.6482	173.8991	175.1081	173.8827	174.9805	173.9346	175.2141	**168.13**
**C5**	118.6951	120.1057	120.7177	120.8251	121.6797	120.7761	122.0034	120.7290	121.8560	120.8746	122.1212	118.8136	120.1112	120.7251	120.7385	121.6008	120.6967	121.8294	120.6564	121.6863	120.7809	121.9289	**117.55**
**C6**	134.9467	136.9717	138.0243	138.0376	139.4645	137.9675	140.0789	137.8990	139.7408	138.1064	140.4199	135.3034	137.3666	138.6127	138.3429	140.0727	138.2853	140.5728	138.2274	140.3897	138.3981	140.9095	**133.34**
**C7**	200.1830	205.0445	206.4951	207.5876	208.7013	207.4269	213.8365	207.2685	212.8952	207.7437	214.6933	204.6033	208.8622	210.0809	211.0822	211.8366	210.9380	216.6033	210.7982	215.3664	211.2259	217.0626	**195.87**
**C8**	62.4961	63.6480	64.3663	63.9871	64.9554	63.9622	65.4882	63.9393	65.2096	64.0139	66.1783	57.1221	58.8511	59.3074	59.6939	60.4822	59.6234	61.3132	59.5575	60.9413	59.7669	61.5421	**60.75**
**C9**	22.4232	22.7099	22.8836	22.9713	23.2313	22.9485	23.3154	22.9273	23.1815	22.9957	23.4231	19.1174	19.6122	19.6632	19.7712	20.0251	19.7540	20.2525	19.7389	20.1245	19.7907	20.6140	**16.39**
**C8`**	35.4509	36.2307	36.4509	36.4979	36.8360	36.4785	37.0927	36.4609	36.9944	36.5195	37.4940	30.7624	31.0177	31.3187	31.2114	31.4738	31.1980	31.6175	31.1846	31.6291	31.2246	31.6596	**32**
**H2**	8.7291	8.6745	8.6839	8.6307	8.5912	8.6340	8.6207	8.6372	8.6136	8.6271	8.6069	8.7394	8.5811	8.6311	8.4997	8.5161	8.5067	8.4889	8.5132	8.5182	8.4920	8.4934	**7.971**
**H3**	7.3570	7.4984	7.5860	7.5429	7.6585	7.5407	7.6805	7.5383	7.6697	7.5447	7.6928	7.3444	7.4827	7.5699	7.5283	7.6473	7.5259	7.6672	7.5234	7.6563	7.5302	7.6796	**7.22**
**H5**	7.2043	7.3984	7.4865	7.4833	7.6061	7.4784	7.6287	7.4735	7.6162	7.4877	7.6494	7.1957	7.3777	7.4663	7.4563	7.5822	7.4518	7.5978	7.4472	7.5841	7.4603	7.6170	**7.22**
**H6**	8.1786	8.3833	8.4552	8.4878	8.5945	8.4818	8.6451	8.4757	8.6289	8.4931	8.6774	7.9635	8.1395	8.2598	8.2257	8.3971	8.2217	8.4076	8.2174	8.4040	8.2288	8.4520	**7.971**
**H8**	4.3167	4.4108	4.4199	4.5072	4.5534	4.5008	4.6039	4.4943	4.6020	4.5131	4.6265	4.4991	4.6448	4.7045	4.7095	4.8253	4.7065	4.8443	4.7033	4.8349	4.7119	4.8737	**5.2**
**H(9A-9C)**	1.3125	1.3212	1.3067	1.3099	1.2608	1.3109	1.2798	1.3118	1.2649	1.3088	1.2919	1.3963	1.4224	1.4195	1.4330	1.4094	1.4328	1.4284	1.4324	1.4298	1.4329	1.4515	**1.611**
**H(8`A-8`C)**	2.4651	2.3989	2.3727	2.3791	2.3574	2.3796	2.3472	2.3802	2.3450	2.3785	2.3301	2.8767	2.7128	2.6773	2.6349	2.5897	2.6409	2.5049	2.6468	2.5284	2.6289	2.4711	**2.82**

[a] methyl protons shifts are averaged
[b] according to [10]

Table S10. Cont. VI

Nuclei	R											S											Exp. [b]
	TPSS																						
	Gas	$CHCl_3$ (PCM)	$CHCl_3$ (SMD)	DMSO (PCM)	DMSO (SMD)	CH_3OH (PCM)	CH_3OH (SMD)	C_2H_5OH (PCM)	C_2H_5OH (SMD)	H_2O (PCM)	H_2O (SMD)	Gas	$CHCl_3$ (PCM)	$CHCl_3$ (SMD)	DMSO (PCM)	DMSO (SMD)	CH_3OH (PCM)	CH_3OH (SMD)	C_2H_5OH (PCM)	C_2H_5OH (SMD)	H_2O (PCM)	H_2O (SMD)	
**C1**	132.3080	132.7323	133.5947	132.9917	134.6209	132.9794	133.9174	132.9795	133.4835	133.0145	134.1627	130.9473	132.0464	132.8321	132.7165	134.1527	132.6760	133.8514	132.6492	133.7648	132.7685	133.9904	**130.99**
**C2**	133.0997	133.0157	133.6822	132.9050	133.5045	132.9199	133.9437	132.9461	133.4500	132.9010	133.9167	132.3746	132.5433	133.1843	132.5871	133.2524	132.5896	133.7520	132.6038	133.7197	132.5960	133.7438	**133.34**
**C3**	116.6148	117.2984	117.9237	117.5959	118.2555	117.5862	118.5980	117.5876	118.0301	117.6152	118.6571	116.6745	117.2824	117.8745	117.5564	118.2099	117.5476	118.4658	117.5497	118.7785	117.5754	118.5352	**117.55**
**C4**	170.6841	171.5811	172.3374	172.0858	172.8432	172.0623	173.5294	172.0498	172.7101	172.1186	173.6596	170.9980	171.6247	172.4068	171.9513	172.7573	171.9401	173.2076	171.9397	172.7606	171.9719	173.3439	**168.13**
**C5**	114.5838	115.8370	116.5194	116.5569	117.3608	116.5197	117.6964	116.4938	116.9319	116.6029	117.8309	114.7542	115.8493	116.5202	116.4562	117.2683	116.4268	117.4654	116.4086	117.6460	116.4943	117.6013	**117.55**
**C6**	129.6014	131.4435	132.6218	132.4972	133.9868	132.4389	134.5452	132.3920	132.8937	132.5630	134.9193	129.9037	131.8399	133.1059	132.8246	134.4809	132.7742	135.0675	132.7346	133.8495	132.8818	135.3960	**133.34**
**C7**	196.4615	200.8812	202.3135	203.2587	204.2943	203.1209	208.8163	202.9947	203.7788	203.4005	209.6447	201.0290	204.6160	205.9348	206.6362	207.5175	206.5206	211.4415	206.4167	206.9790	206.7570	211.9588	**195.87**
**C8**	65.1158	66.2120	66.9967	66.5823	67.5160	66.5694	67.7566	66.5676	66.8060	66.6049	68.5367	60.1278	61.8831	62.5632	62.8019	63.6923	62.7474	64.2607	62.7059	64.3460	62.8662	64.4976	**60.75**
**C9**	23.9382	24.2126	24.5053	24.5049	24.8006	24.4912	24.8817	24.4888	24.5589	24.5284	25.0320	20.6506	21.2665	21.4915	21.5390	21.8543	21.5290	22.1455	21.5306	23.4796	21.5593	22.5571	**16.39**
**C8`**	37.5256	38.2274	38.5539	38.5318	38.8578	38.5228	39.1217	38.5251	38.6513	38.5508	39.5382	33.2385	33.3352	33.6679	33.4955	33.7511	33.4908	33.9306	33.4973	35.3565	33.5099	33.9584	**32**
**H2**	8.5053	8.4506	8.4858	8.4146	8.4078	8.4174	8.4634	8.4207	8.2608	8.4123	8.4454	8.5333	8.3896	8.4302	8.3110	8.3267	8.3165	8.3314	8.3222	8.4330	8.3060	8.3282	**7.971**
**H3**	7.1662	7.3060	7.4014	7.3539	7.4751	7.3516	7.4934	7.3498	7.1856	7.3564	7.5050	7.1476	7.2856	7.3782	7.3357	7.4586	7.3331	7.4748	7.3311	7.4493	7.3384	7.4865	**7.22**
**H5**	7.0222	7.2106	7.3064	7.2978	7.4257	7.2928	7.4508	7.2885	7.1240	7.3028	7.4683	7.0178	7.1891	7.2832	7.2681	7.3973	7.2637	7.4087	7.2598	7.3786	7.2727	7.4263	**7.22**
**H6**	7.9512	8.1457	8.2359	8.2495	8.3724	8.2435	8.4400	8.2381	8.0774	8.2555	8.4692	7.7288	7.9081	8.0237	7.9982	8.1671	7.9933	8.1988	7.9889	8.1022	8.0030	8.2393	**7.971**
**H8**	4.4709	4.5419	4.5602	4.6283	4.6855	4.6222	4.7477	4.6168	4.4417	4.6345	4.7669	4.5859	4.7329	4.7881	4.8006	4.9128	4.7971	4.9372	4.7941	4.9314	4.8041	4.9650	**5.2**
**H(9A-9C)**	1.3567	1.3679	1.3676	1.3629	1.3311	1.3636	1.3485	1.3649	1.1767	1.3626	1.3622	1.4392	1.4842	1.4875	1.5034	1.4900	1.5025	1.5193	1.5021	1.6640	1.5046	1.5408	**1.611**
**H(8`A-8`C)**	2.5215	2.4527	2.4256	2.4341	2.4118	2.4348	2.3810	2.4361	2.2522	2.4338	2.3643	2.9653	2.7995	2.7626	2.7257	2.6817	2.7310	2.6015	2.7368	2.8894	2.7208	2.5649	**2.82**
Nuclei	VSXC																						
**C1**	130.0829	130.4045	131.1400	130.4934	131.6615	130.4929	131.1365	130.4902	131.2057	130.4951	131.2579	129.9454	130.6445	131.8488	131.2031	132.5520	131.1505	132.4267	131.1004	132.2788	131.2589	132.5715	**130.99**
**C2**	131.4719	131.5765	132.0332	131.5476	131.9755	131.5536	132.4149	131.5574	132.3103	131.5434	132.5007	131.5035	131.5629	132.0835	131.4748	131.9974	131.4854	132.4275	131.4940	132.3584	131.4659	132.4408	**133.34**
**C3**	115.0253	115.9281	116.4781	116.3182	116.9617	116.2986	117.2532	116.2767	117.1666	116.3381	117.3602	115.1018	115.8625	116.3437	116.1552	116.7869	116.1441	116.9700	116.1304	116.9294	116.1665	117.0707	**117.55**
**C4**	168.4085	169.4140	170.1094	169.9432	170.7388	169.9139	171.2602	169.8824	171.1101	169.9727	171.4432	168.2644	169.1345	169.7602	169.5550	170.3438	169.5352	170.7332	169.5129	170.6261	169.5745	170.8988	**168.13**
**C5**	112.9381	114.3706	114.9696	115.1434	115.9400	115.0989	116.2089	115.0522	116.0882	115.1874	116.3893	112.8384	114.1341	114.6817	114.8040	115.5837	114.7687	115.7340	114.7306	115.6555	114.8386	115.8930	**117.55**
**C6**	129.7735	131.5896	132.6289	132.6050	134.1264	132.5458	134.6888	132.4845	134.4417	132.6624	135.0466	129.7921	131.8114	132.8618	132.8875	134.4094	132.8259	134.9250	132.7615	134.6909	132.9470	135.2245	**133.34**
**C7**	190.4964	194.5948	195.8890	196.6716	197.5040	196.5484	201.3041	196.4236	200.4899	196.7897	202.0685	192.4201	196.4292	197.9570	198.6054	199.5513	198.4654	203.3086	198.3255	202.3644	198.7417	203.9258	**195.87**
**C8**	71.0293	71.8644	72.4464	72.1610	72.9614	72.1470	73.3160	72.1307	73.2068	72.1762	73.9137	63.2954	64.4627	65.0975	65.1337	65.9999	65.0894	66.5041	65.0447	66.3060	65.1788	66.9366	**60.75**
**C9**	27.6811	28.6013	28.9238	29.2582	29.7109	29.2182	30.0806	29.1763	29.9393	29.2980	30.1347	28.6031	28.7198	28.7057	28.8776	29.0104	28.8658	28.9877	28.8527	28.9354	28.8913	29.2920	**16.39**
**C8`**	40.4536	40.9423	41.0239	41.1700	41.3482	41.1601	41.3711	41.1480	41.3482	41.1808	41.7912	35.6942	36.3333	36.3426	36.5410	36.8365	36.5434	36.8289	36.5411	36.8765	36.5384	37.0830	**32**
**H2**	8.7013	8.6318	8.6383	8.5965	8.5956	8.5987	8.6008	8.6009	8.6015	8.5945	8.6002	8.6698	8.5714	8.5462	8.4980	8.4914	8.5044	8.4746	8.5105	8.4951	8.4915	8.4705	**7.971**
**H3**	7.2076	7.3421	7.4286	7.3891	7.5038	7.3867	7.5214	7.3843	7.5128	7.3914	7.5352	7.2377	7.3761	7.4621	7.4257	7.5415	7.4230	7.5574	7.4205	7.5480	7.4281	7.5730	**7.22**
**H5**	7.1085	7.2957	7.3836	7.3798	7.5056	7.3750	7.5203	7.3702	7.5095	7.3844	7.5443	7.1230	7.2984	7.3840	7.3775	7.4993	7.3729	7.5080	7.3684	7.4968	7.3817	7.5256	**7.22**
**H6**	8.1799	8.3473	8.4451	8.4415	8.5921	8.4354	8.6432	8.4296	8.6230	8.4472	8.6738	7.8041	7.9933	8.0857	8.0898	8.2480	8.0846	8.2771	8.0794	8.2615	8.0945	8.3072	**7.971**
**H8**	4.3876	4.4583	4.4912	4.5227	4.5993	4.5183	4.6338	4.5141	4.6138	4.5269	4.6595	4.6175	4.7683	4.8270	4.8563	4.9599	4.8507	5.0035	4.8453	4.9843	4.8616	5.0218	**5.2**
**H(9A-9C)**	1.5961	1.6138	1.6048	1.6171	1.6025	1.6170	1.6242	1.6171	1.6201	1.6172	1.6378	1.7731	1.7717	1.7506	1.7772	1.7569	1.7769	1.7723	1.7766	1.7683	1.7776	1.7889	**1.611**
**H(8`A-8`C)**	2.6858	2.6354	2.6002	2.6143	2.5757	2.6156	2.5544	2.6170	2.5572	2.6129	2.5289	2.8671	2.7641	2.7059	2.7132	2.6678	2.7172	2.6228	2.7210	2.6423	2.7093	2.5927	**2.82**
Nuclei	wB97																						
**C1**	136.3743	136.5643	137.5508	136.6805	138.3186	136.6682	137.1758	136.6563	137.6400	136.6935	137.3306	135.5496	136.5810	137.2430	137.2393	138.6376	137.1701	138.5360	137.1091	138.2897	137.3170	138.7211	**130.99**
**C2**	140.2891	140.1704	140.7301	140.0418	140.6111	140.0495	141.1373	140.0566	141.0098	140.0348	141.1780	140.0931	140.0448	140.6006	139.9574	140.5644	139.9621	140.9964	139.9662	140.9089	139.9528	141.0199	**133.34**
**C3**	121.6439	122.2148	122.7714	122.4318	123.1041	122.4188	123.2000	122.4051	123.1728	122.4445	123.2668	121.6716	122.2096	122.7417	122.4350	123.1020	122.4187	123.2279	122.4023	123.1663	122.4515	123.2949	**117.55**
**C4**	174.2577	174.9242	175.5185	175.3214	175.9512	175.2956	176.5447	175.2691	176.3511	175.3461	176.6908	174.3838	174.8494	175.5012	175.0939	175.7994	175.0823	176.1349	175.0683	176.0692	175.1036	176.2686	**168.13**
**C5**	119.4831	120.7345	121.3443	121.4210	122.2577	121.3777	122.3482	121.3337	122.2709	121.4624	122.5094	119.5892	120.7784	121.3962	121.3974	122.2486	121.3581	122.3057	121.3179	122.2298	121.4354	122.4485	**117.55**
**C6**	136.8221	138.8055	139.9029	139.8974	141.4938	139.8285	142.2089	139.7589	141.7730	139.9631	142.5634	137.0806	139.0577	140.2748	140.0174	141.7669	139.9692	142.1277	139.9168	141.9973	140.0596	142.4749	**133.34**
**C7**	200.5554	205.5196	206.9736	208.1930	208.8203	208.0253	213.7590	207.8570	212.9387	208.3529	214.6659	204.2854	208.7429	210.1036	211.2165	211.7853	211.0496	216.9049	210.8871	215.6715	211.3809	217.6015	**195.87**
**C8**	61.2286	62.1722	62.8269	62.5542	63.4515	62.5338	63.8040	62.5119	63.7336	62.5735	64.4107	55.7397	57.1823	57.6139	58.0157	58.7514	57.9530	59.4666	57.8934	59.1612	58.0807	59.7826	**60.75**
**C9**	22.0700	22.2380	22.4228	22.4373	22.8248	22.4194	22.7743	22.4016	22.6602	22.4557	22.8715	19.5465	19.7727	19.7821	19.8738	20.1420	19.8648	20.1501	19.8559	20.0913	19.8837	20.5181	**16.39**
**C8`**	35.1652	35.7649	35.9908	36.0743	36.3679	36.0567	36.5472	36.0377	36.5002	36.0907	36.9598	30.5263	30.8779	31.2072	31.0800	31.3808	31.0803	31.3789	31.0759	31.4642	31.0751	31.4895	**32**
**H2**	8.8384	8.7915	8.8061	8.7534	8.7565	8.7561	8.8198	8.7586	8.7788	8.7508	8.8177	8.8444	8.6993	8.7460	8.6049	8.6340	8.6136	8.5895	8.6213	8.6222	8.5954	8.5916	**7.971**
**H3**	7.4415	7.5770	7.6646	7.6196	7.7405	7.6173	7.7555	7.6149	7.7454	7.6217	7.7699	7.4525	7.5863	7.6739	7.6277	7.7500	7.6258	7.7576	7.6236	7.7514	7.6295	7.7730	**7.22**
**H5**	7.3028	7.4922	7.5805	7.5754	7.7036	7.5705	7.7161	7.5655	7.7041	7.5800	7.7398	7.3123	7.4932	7.5821	7.5675	7.6967	7.5632	7.7017	7.5588	7.6926	7.5714	7.7244	**7.22**
**H6**	8.2363	8.4473	8.5401	8.5626	8.6990	8.5558	8.7730	8.5487	8.7240	8.5691	8.8069	8.0771	8.2684	8.3886	8.3453	8.5299	8.3432	8.5210	8.3402	8.5241	8.3465	8.5617	**7.971**
**H8**	4.0794	4.2013	4.2289	4.3015	4.3612	4.2941	4.4167	4.2867	4.3964	4.3088	4.4426	4.2843	4.4417	4.5098	4.5068	4.6254	4.5039	4.6339	4.5005	4.6294	4.5091	4.6610	**5.2**
**H(9A-9C)**	1.3373	1.3502	1.3355	1.3434	1.3206	1.3441	1.3538	1.3446	1.3316	1.3428	1.3678	1.4052	1.4218	1.4124	1.4283	1.4126	1.4285	1.4264	1.4284	1.4276	1.4279	1.4449	**1.611**
**H(8`A-8`C)**	2.4600	2.4029	2.3721	2.3774	2.3594	2.3781	2.3295	2.3790	2.3441	2.3770	2.3180	2.8246	2.6620	2.6344	2.5859	2.5485	2.5907	2.4603	2.5952	2.4869	2.5812	2.4374	**2.82**
Nuclei	wB97Z																						
**C1**	137.8029	138.0540	139.0211	138.1992	139.8961	138.1870	138.7316	138.1755	139.5920	138.2141	139.6814	136.9016	138.0110	138.6568	138.6712	140.0912	138.6060	140.0704	138.5482	139.7173	138.7443	140.1871	**130.99**
**C2**	140.7055	140.6032	141.1665	140.4847	141.0596	140.4945	141.6070	140.5037	141.4061	140.4762	141.5856	140.5526	140.5103	141.0764	140.4186	141.0232	140.4259	141.4852	140.4318	141.3445	140.4125	141.4959	**133.34**
**C3**	122.3475	122.9417	123.4842	123.1709	123.8319	123.1596	123.9911	123.1475	123.9336	123.1825	124.0993	122.3753	122.9427	123.4616	123.1718	123.8332	123.1584	124.0179	123.1444	123.8893	123.1862	124.0701	**117.55**
**C4**	174.9165	175.6237	176.2197	176.0405	176.6624	176.0165	177.3046	175.9916	177.0258	176.0640	177.3657	175.0408	175.5562	176.2009	175.8192	176.5285	175.8083	176.9078	175.7954	176.7811	175.8288	177.0410	**168.13**
**C5**	120.1726	121.4443	122.0434	122.1523	122.9687	122.1105	123.1411	122.0679	123.0163	122.1931	123.3151	120.2954	121.5031	122.1147	122.1275	122.9742	122.0916	123.0943	122.0548	122.9461	122.1629	123.2277	**117.55**
**C6**	137.1768	139.1619	140.2691	140.2457	141.8067	140.1813	142.5581	140.1156	141.8911	140.3073	142.5689	137.4753	139.4432	140.6731	140.4268	142.1500	140.3797	142.5033	140.3282	142.3574	140.4684	142.8687	**133.34**
**C7**	202.1768	207.1982	208.6196	209.9149	210.5679	209.7483	215.5392	209.5818	215.1279	210.0748	217.1331	206.1849	210.6989	211.9868	213.1284	213.7439	212.9665	218.9548	212.8080	217.5873	213.2885	219.5702	**195.87**
**C8**	62.2638	63.2785	63.9563	63.6403	64.5673	63.6254	64.9558	63.6092	64.7914	63.6554	65.6105	56.7269	58.2485	58.7010	59.1431	59.8693	59.0782	60.6326	59.0159	60.2657	59.2102	60.9139	**60.75**
**C9**	22.2545	22.4780	22.6788	22.6724	23.0493	22.6560	23.0235	22.6394	22.7994	22.6898	23.0038	19.5952	19.8548	19.8730	19.9815	20.2271	19.9737	20.2590	19.9655	20.1660	19.9902	20.6043	**16.39**
**C8`**	35.4261	36.0803	36.3153	36.4017	36.7065	36.3882	36.9314	36.3733	36.7947	36.4152	37.3166	30.7265	31.0292	31.3557	31.2646	31.5524	31.2617	31.6100	31.2549	31.6389	31.2646	31.7241	**32**
**H2**	8.8045	8.7552	8.7713	8.7178	8.7132	8.7209	8.7796	8.7237	8.6982	8.7149	8.6978	8.8202	8.6677	8.7159	8.5736	8.5991	8.5821	8.5540	8.5897	8.5893	8.5645	8.5574	**7.971**
**H3**	7.4131	7.5511	7.6385	7.5956	7.7147	7.5935	7.7298	7.5914	7.7207	7.5978	7.7443	7.4226	7.5575	7.6444	7.6018	7.7223	7.5997	7.7328	7.5974	7.7240	7.6039	7.7460	**7.22**
**H5**	7.2774	7.4679	7.5557	7.5537	7.6786	7.5490	7.6961	7.5442	7.6777	7.5583	7.7129	7.2894	7.4695	7.5577	7.5457	7.6732	7.5418	7.6791	7.5378	7.6674	7.5496	7.7001	**7.22**
**H6**	8.2021	8.4135	8.5069	8.5295	8.6573	8.5233	8.7307	8.5170	8.6684	8.5355	8.7254	8.0367	8.2260	8.3460	8.3104	8.4911	8.3074	8.4771	8.3036	8.4843	8.3126	8.5189	**7.971**
**H8**	4.1390	4.2501	4.2742	4.3513	4.4040	4.3440	4.4602	4.3368	4.4505	4.3585	4.4898	4.3255	4.4860	4.5491	4.5527	4.6724	4.5495	4.6828	4.5459	4.6757	4.5555	4.7093	**5.2**
**H(9A-9C)**	1.3232	1.3360	1.3236	1.3281	1.3024	1.3291	1.3388	1.3299	1.2880	1.3271	1.3094	1.3934	1.4128	1.4075	1.4242	1.4050	1.4244	1.4147	1.4244	1.4169	1.4237	1.4340	**1.611**
**H(8`A-8`C)**	2.4671	2.4104	2.3794	2.3869	2.3692	2.3875	2.3398	2.3882	2.3568	2.3865	2.3429	2.8470	2.6868	2.6544	2.6087	2.5717	2.6137	2.4834	2.6186	2.5074	2.6037	2.4553	**2.82**

[a] methyl protons shifts are averaged

[b] according to [10]

Table S10. Cont. VII

Nuclei	R										
	X3LYP										
	Gas	$CHCl_3$ (PCM)	$CHCl_3$ (SMD)	DMSO (PCM)	DMSO (SMD)	CH_3OH (PCM)	CH_3OH (SMD)	C_2H_5OH (PCM)	C_2H_5OH (SMD)	H_2O (PCM)	H_2O (SMD)
**C1**	139.3564	139.8444	140.8482	140.1134	142.0566	140.0909	141.3658	140.0679	141.5227	140.1411	141.6467
**C2**	139.9182	139.8474	140.4108	139.6953	140.1921	139.7116	140.6326	139.7238	140.5055	139.6831	140.6144
**C3**	122.0092	122.6988	123.2345	122.9633	123.5748	122.9518	123.8529	122.9370	123.8097	122.9783	123.9078
**C4**	176.1825	177.0882	177.7250	177.5818	178.2391	177.5544	178.8901	177.5242	178.7610	177.6114	179.0087
**C5**	119.8141	121.1439	121.7439	121.8710	122.6356	121.8294	122.8875	121.7849	122.7972	121.9143	123.0321
**C6**	136.1581	138.1629	139.2158	139.2637	140.6897	139.2042	141.2883	139.1404	140.9981	139.3229	141.6419
**C7**	202.8571	207.9768	209.5518	210.7426	211.8371	210.5640	217.1792	210.3863	216.3022	210.9197	218.1127
**C8**	65.0592	66.2153	66.9018	66.5407	67.5099	66.5316	67.8451	66.5179	67.6413	66.5516	68.6175
**C9**	23.0316	23.3183	23.4327	23.5997	23.8343	23.5889	23.8506	23.5740	23.7548	23.6127	23.9870
**C8`**	36.3944	37.1898	37.4326	37.5008	37.8523	37.4859	38.0604	37.4681	38.0401	37.5189	38.4847
**H2**	8.6524	8.5918	8.6063	8.5511	8.5135	8.5549	8.5499	8.5579	8.5426	8.5473	8.5316
**H3**	7.3213	7.4551	7.5439	7.5005	7.6121	7.4985	7.6290	7.4960	7.6209	7.5023	7.6416
**H5**	7.1716	7.3592	7.4485	7.4464	7.5662	7.4416	7.5856	7.4362	7.5755	7.4510	7.6064
**H6**	8.0809	8.2845	8.3663	8.3962	8.5098	8.3898	8.5716	8.3828	8.5557	8.4024	8.5971
**H8**	4.3443	4.4331	4.4575	4.5383	4.5907	4.5308	4.6494	4.5230	4.6418	4.5456	4.6641
**H(9A-9C)**	1.3227	1.3314	1.3179	1.3217	1.2790	1.3235	1.2915	1.3245	1.2806	1.3199	1.3039
**H(8`A-8`C)**	2.5170	2.4557	2.4302	2.4400	2.4194	2.4409	2.4037	2.4412	2.4048	2.4393	2.3899

Nuclei	S											Exp. [b]
	X3LYP											
	Gas	$CHCl_3$ (PCM)	$CHCl_3$ (SMD)	DMSO (PCM)	DMSO (SMD)	CH_3OH (PCM)	CH_3OH (SMD)	C_2H_5OH (PCM)	C_2H_5OH (SMD)	H_2O (PCM)	H_2O (SMD)	
**C1**	137.9117	139.2562	139.9366	139.9579	141.5273	139.9149	141.3110	139.8710	141.2013	140.0058	141.3487	**130.99**
**C2**	139.2169	139.3848	139.9238	139.3913	140.0004	139.3937	140.5425	139.3932	140.4245	139.3932	140.5430	**133.34**
**C3**	122.1353	122.7433	123.2197	122.9818	123.5816	122.9719	123.7914	122.9587	123.7342	122.9949	123.8481	**117.55**
**C4**	176.5499	177.1393	177.8065	177.4289	178.1522	177.4164	178.5567	177.4002	178.4628	177.4439	178.7115	**168.13**
**C5**	119.9992	121.2093	121.7793	121.8153	122.5836	121.7831	122.7292	121.7474	122.6490	121.8494	122.8641	**117.55**
**C6**	136.5146	138.5767	139.8645	139.6151	141.2627	139.5588	141.7748	139.4982	141.5895	139.6709	142.1688	**133.34**
**C7**	208.1208	212.3136	213.4589	214.5395	215.3412	214.4092	220.0232	214.2763	218.9420	214.6672	220.5368	**195.87**
**C8**	59.3212	61.3577	61.9949	62.3629	63.2348	62.3022	63.9246	62.2400	63.6812	62.4263	64.1714	**60.75**
**C9**	19.5775	20.0461	20.2181	20.2904	20.5151	20.2799	20.6827	20.2663	20.6108	20.3042	21.0438	**16.39**
**C8`**	31.9991	32.1508	32.4082	32.3228	32.5612	32.3139	32.6685	32.3014	32.6855	32.3346	32.7610	**32**
**H2**	8.6901	8.5204	8.5632	8.4372	8.4423	8.4428	8.4297	8.4475	8.4451	8.4317	8.4405	**7.971**
**H3**	7.3067	7.4391	7.5255	7.4863	7.6016	7.4841	7.6164	7.4812	7.6087	7.4884	7.6303	**7.22**
**H5**	7.1634	7.3365	7.4251	7.4152	7.5364	7.4110	7.5463	7.4062	7.5365	7.4192	7.5665	**7.22**
**H6**	7.8813	8.0490	8.1656	8.1364	8.2978	8.1318	8.3147	8.1265	8.3041	8.1406	8.3622	**7.971**
**H8**	4.4903	4.6483	4.7010	4.7147	4.8269	4.7114	4.8459	4.7075	4.8333	4.7177	4.8776	**5.2**
**H(9A-9C)**	1.3991	1.4351	1.4417	1.4560	1.4332	1.4550	1.4533	1.4534	1.4521	1.4569	1.4778	**1.611**
**H(8`A-8`C)**	2.9813	2.8071	2.7639	2.7278	2.6788	2.7332	2.5951	2.7379	2.6158	2.7225	2.5651	**2.82**

[a] methyl protons shifts are averaged

[b] according to [10]

Table S11. Theoretical chemical shifts (in ppm) of *(R,S)*-flephedrone calculated with HF and selected functionals combined with 6-311++G** basis set[a]

Nuclei	R											S											Exp. [b]
	B3LYP																						
	Gas	$CHCl_3$ (PCM)	$CHCl_3$ (SMD)	DMSO (PCM)	DMSO (SMD)	CH_3OH (PCM)	CH_3OH (SMD)	C_2H_5OH (PCM)	C_2H_5OH (SMD)	H_2O (PCM)	H_2O (SMD)	Gas	$CHCl_3$ (PCM)	$CHCl_3$ (SMD)	DMSO (PCM)	DMSO (SMD)	CH_3OH (PCM)	CH_3OH (SMD)	C_2H_5OH (PCM)	C_2H_5OH (SMD)	H_2O (PCM)	H_2O (SMD)	
**C1**	139.3516	139.8992	140.9781	140.1863	142.0735	140.1611	141.5120	140.1358	141.5012	140.2082	141.7819	137.8738	139.3186	139.9816	139.9577	141.5554	139.9093	141.4183	139.8637	141.2810	140.0057	141.4292	**129.02**
**C2**	139.7181	139.6929	140.2565	139.4633	139.9708	139.4799	140.4199	139.4943	140.2834	139.4440	140.3925	139.0099	139.2247	139.7699	139.1806	139.8028	139.1780	140.3740	139.1755	140.2362	139.1821	140.3704	**132.19**
**C3**	121.9147	122.6511	123.2036	122.8744	123.4797	122.8576	123.7880	122.8405	123.7263	122.8887	123.8478	122.0546	122.6999	123.1853	122.8846	123.4913	122.8701	123.7328	122.8554	123.6526	122.8966	123.7867	**116.16**
**C4**	176.1996	177.1548	177.8016	177.6113	178.2556	177.5774	178.9142	177.5440	178.7922	177.6426	179.0429	176.5723	177.1853	177.8667	177.4190	178.1469	177.4023	178.5696	177.3850	178.4546	177.4322	178.7245	**166.89**
**C5**	119.7381	121.1100	121.7159	121.7876	122.5392	121.7417	122.8148	121.6959	122.7064	121.8299	122.9605	119.9339	121.1749	121.7562	121.7216	122.4902	121.6855	122.6663	121.6489	122.5622	121.7540	122.7986	**116.16**
**C6**	135.8982	137.9272	138.9534	138.9372	140.4067	138.8712	141.0202	138.8048	140.7434	138.9983	141.3636	136.2778	138.3528	139.6434	139.3323	140.9568	139.2716	141.4709	139.2095	141.2669	139.3872	141.8771	**132.19**
**C7**	202.8768	208.0691	209.7045	210.9565	211.8939	210.7561	217.3250	210.5621	216.3636	211.1520	218.3042	208.1087	212.3579	213.4837	214.5278	215.3474	214.3946	220.1153	214.2618	218.9963	214.6532	220.5944	**196.22**
**C8**	65.2601	66.4393	67.0844	66.6651	67.6873	66.6448	68.1168	66.6260	67.8105	66.6832	68.8556	59.4742	61.6544	62.2820	62.6180	63.4923	62.5505	64.2487	62.4848	63.9791	62.6825	64.4890	**59.75**
**C9**	22.9937	23.3369	23.4371	23.5103	23.7870	23.5040	23.8454	23.4947	23.7278	23.5117	23.9610	19.5432	20.0549	20.2357	20.2537	20.4684	20.2366	20.6702	20.2197	20.5736	20.2683	21.0311	**15.44**
**C8`**	36.5076	37.3387	37.5483	37.6307	37.9535	37.5944	38.1848	37.5637	38.1616	37.6692	38.6153	32.1915	32.3577	32.6229	32.4742	32.7092	32.4611	32.8316	32.4474	32.8288	32.4845	32.9175	**31.11**
**H2**	8.6288	8.5708	8.5739	8.5193	8.4862	8.5229	8.5099	8.5264	8.5151	8.5156	8.4958	8.6701	8.4976	8.5384	8.4115	8.4156	8.4168	8.3988	8.4218	8.4120	8.4060	8.4143	**7.97**
**H3**	7.3119	7.4474	7.5313	7.4874	7.6000	7.4852	7.6152	7.4829	7.6051	7.4891	7.6305	7.2978	7.4323	7.5140	7.4748	7.5907	7.4723	7.6048	7.4697	7.5943	7.4770	7.6211	**7.22**
**H5**	7.1614	7.3510	7.4352	7.4344	7.5536	7.4291	7.5704	7.4238	7.5589	7.4393	7.5939	7.1540	7.3281	7.4120	7.4019	7.5234	7.3974	7.5322	7.3928	7.5197	7.4059	7.5549	**7.22**
**H6**	8.0695	8.2736	8.3471	8.3829	8.4942	8.3751	8.5470	8.3677	8.5388	8.3904	8.5777	7.8720	8.0370	8.1480	8.1191	8.2781	8.1143	8.2908	8.1093	8.2781	8.1233	8.3419	**7.97**
**H8**	4.3623	4.4552	4.4783	4.5600	4.6044	4.5533	4.6526	4.5464	4.6504	4.5660	4.6741	4.4999	4.6578	4.7050	4.7188	4.8309	4.7153	4.8477	4.7118	4.8326	4.7217	4.8820	**4.99**
**H(9A-9C)**	1.3154	1.3242	1.3019	1.3025	1.2691	1.3041	1.2806	1.3055	1.2685	1.3007	1.2933	1.3888	1.4276	1.4302	1.4455	1.4215	1.4441	1.4397	1.4427	1.4360	1.4466	1.4683	**1.51**
**H(8`A-8`C)**	2.5071	2.4491	2.4216	2.4281	2.4102	2.4290	2.3990	2.4298	2.3931	2.4269	2.3861	2.9770	2.8044	2.7564	2.7204	2.6721	2.7257	2.5872	2.7307	2.6042	2.7151	2.5595	**2.71**
Nuclei	B3P86																						
**C1**	136.8453	137.2808	138.3582	137.5043	139.4519	137.5074	138.8537	137.4898	139.1351	137.5460	139.1283	135.6233	136.9063	137.5597	137.4853	138.9540	137.4549	138.8749	137.4076	138.7415	137.5634	138.9677	**129.02**
**C2**	138.7919	138.7416	139.3998	138.5747	139.1636	138.6075	139.5971	138.6182	139.5645	138.5873	139.5816	138.1598	138.3322	138.9376	138.3113	138.9809	138.3306	139.5077	138.3287	139.4734	138.3375	139.5260	**132.19**
**C3**	121.1158	121.8832	122.5099	122.1552	122.8745	122.1613	123.1693	122.1453	123.2080	122.1932	123.2475	121.2077	121.9036	122.4956	122.1573	122.8618	122.1638	123.0956	122.1483	123.1217	122.1950	123.1750	**116.16**
**C4**	173.6985	174.6092	175.2829	175.0662	175.7535	175.0598	176.4063	175.0315	176.3576	175.1159	176.5491	173.9907	174.6001	175.3439	174.8861	175.6462	174.8935	176.0480	174.8781	176.0672	174.9218	176.2106	**166.89**
**C5**	118.9511	120.3570	121.0389	121.0865	121.9545	121.0629	122.2291	121.0175	122.2236	121.1526	122.3983	119.0968	120.3750	121.0516	121.0069	121.8780	120.9911	122.0417	120.9530	122.0531	121.0653	122.1965	**116.16**
**C6**	134.9391	136.9571	138.0470	138.0317	139.4694	137.9871	140.0383	137.9202	139.8454	138.1179	140.4254	135.2870	137.3568	138.6630	138.3561	140.0839	138.3212	140.5742	138.2627	140.5163	138.4312	140.9668	**132.19**
**C7**	200.9937	205.8309	207.3690	208.3757	209.4937	208.2408	214.5962	208.0849	213.8337	208.5478	215.4998	205.5040	209.6504	210.9178	211.8502	212.6202	211.7370	217.2310	211.6033	216.1910	212.0035	217.7281	**196.22**
**C8**	63.6796	64.8464	65.6285	65.2068	66.1771	65.2071	66.5812	65.1854	66.4957	65.2505	67.3827	58.1084	59.9428	60.4781	60.8007	61.6333	60.7568	62.3872	60.6942	62.1777	60.8896	62.6693	**59.75**
**C9**	22.8065	23.0897	23.3077	23.3623	23.6359	23.3647	23.6593	23.3452	23.6707	23.4035	23.8216	19.4663	20.0187	20.1559	20.2089	20.4941	20.2153	20.6857	20.2003	20.7001	20.2472	21.1119	**15.44**
**C8`**	35.9286	36.7158	37.0084	37.0027	37.3475	37.0082	37.5596	36.9918	37.6189	37.0414	38.0311	31.3622	31.5731	31.9325	31.7646	32.0364	31.7748	32.1533	31.7622	32.2999	31.7969	32.2578	**31.11**
**H2**	8.6937	8.6392	8.6481	8.5947	8.5586	8.5992	8.5883	8.6018	8.5814	8.5915	8.5744	8.7028	8.5480	8.5959	8.4713	8.4868	8.4787	8.4611	8.4838	8.4913	8.4649	8.4716	**7.97**
**H3**	7.3323	7.4749	7.5633	7.5186	7.6361	7.5176	7.6523	7.5147	7.6476	7.5207	7.6690	7.3188	7.4600	7.5477	7.5057	7.6274	7.5044	7.6414	7.5013	7.6367	7.5080	7.6590	**7.22**
**H5**	7.1767	7.3724	7.4616	7.4573	7.5836	7.4536	7.6013	7.4480	7.5944	7.4620	7.6265	7.1655	7.3489	7.4382	7.4270	7.5553	7.4237	7.5640	7.4186	7.5573	7.4312	7.5880	**7.22**
**H6**	8.1523	8.3571	8.4270	8.4597	8.5681	8.4550	8.6211	8.4484	8.6073	8.4654	8.6536	7.9340	8.1076	8.2276	8.1948	8.3652	8.1917	8.3755	8.1865	8.3750	8.1986	8.4257	**7.97**
**H8**	4.3930	4.4836	4.4927	4.5762	4.6265	4.5711	4.6819	4.5642	4.6796	4.5824	4.7038	4.5540	4.6976	4.7569	4.7623	4.8783	4.7605	4.8928	4.7566	4.8883	4.7650	4.9288	**4.99**
**H(9A-9C)**	1.3335	1.3438	1.3286	1.3321	1.2867	1.3343	1.2965	1.3346	1.2886	1.3313	1.3143	1.4092	1.4428	1.4421	1.4554	1.4353	1.4561	1.4505	1.4549	1.4566	1.4559	1.4796	**1.51**
**H(8`A-8`C)**	2.4876	2.4212	2.3951	2.4005	2.3811	2.4023	2.3602	2.4024	2.3671	2.4001	2.3503	2.9176	2.7485	2.7146	2.6728	2.6287	2.6800	2.5349	2.6851	2.5651	2.6670	2.5072	**2.71**
Nuclei	B3PW91																						
**C1**	137.0994	137.5782	138.7234	137.8703	140.2084	137.8485	139.3905	137.8187	139.3095	137.8939	139.6388	135.8270	137.2800	137.7781	137.8580	139.3488	137.8089	139.3951	137.7573	139.1374	137.9141	139.4828	**129.02**
**C2**	138.8604	138.8385	139.3967	138.6503	139.5459	138.6706	139.6266	138.6815	139.5161	138.6320	139.6248	138.2843	138.4871	139.0226	138.4452	139.1209	138.4505	139.6147	138.4477	139.5068	138.4432	139.6376	**132.19**
**C3**	121.2185	122.0069	122.5742	122.2708	123.2963	122.2610	123.2495	122.2421	123.2072	122.2821	123.3409	121.3071	122.0247	122.5329	122.2639	122.9651	122.2553	123.1906	122.2380	123.1214	122.2739	123.2752	**116.16**
**C4**	173.7409	174.6858	175.2871	175.1369	176.1105	175.1138	176.3924	175.0819	176.3209	175.1612	176.5562	174.0199	174.6397	175.3121	174.9084	175.6627	174.9018	176.0329	174.8849	175.9623	174.9153	176.2007	**166.89**
**C5**	119.0599	120.4861	121.1030	121.2065	122.3683	121.1668	122.2995	121.1183	122.2138	121.2461	122.4777	119.2098	120.5084	121.1087	121.1148	121.9791	121.0849	122.1342	121.0458	122.0478	121.1449	122.2957	**116.16**
**C6**	134.8881	136.9286	137.8816	137.9622	139.7104	137.9061	139.9572	137.8415	139.7281	138.0174	140.3326	135.2770	137.3211	138.5843	138.3076	139.9907	138.2583	140.3962	138.1982	140.2701	138.3538	140.7941	**132.19**
**C7**	201.1247	206.1066	207.7353	208.8082	210.2420	208.6327	215.1374	208.4510	214.2009	208.9795	216.0833	205.6165	209.9651	211.0502	212.1644	212.9527	212.0348	217.7726	211.8977	216.5723	212.2928	218.2699	**196.22**
**C8**	63.4567	64.6178	65.2257	64.9342	66.2583	64.9202	66.4879	64.8987	66.1020	64.9510	67.2318	58.0320	59.9926	60.4452	60.8767	61.6836	60.8128	62.5182	60.7445	62.2018	60.9444	62.8000	**59.75**
**C9**	22.5160	22.8553	22.9353	23.0789	23.6687	23.0753	23.3953	23.0617	23.2786	23.0832	23.5418	19.1832	19.7035	19.7883	19.8973	20.1279	19.8867	20.3038	19.8683	20.2280	19.9102	20.7016	**15.44**
**C8`**	35.8834	36.7085	36.8598	36.9828	37.6339	36.9657	37.5129	36.9420	37.5231	37.0038	37.9753	31.4292	31.6510	31.9335	31.8297	32.1026	31.8260	32.1558	31.8123	32.2185	31.8333	32.2536	**31.11**
**H2**	8.6633	8.6108	8.6056	8.5605	8.5329	8.5652	8.5339	8.5693	8.5526	8.5562	8.5242	8.6835	8.5130	8.5718	8.4375	8.4524	8.4445	8.4188	8.4505	8.4482	8.4303	8.4283	**7.97**
**H3**	7.3301	7.4703	7.5577	7.5143	7.6458	7.5126	7.6483	7.5105	7.6406	7.5160	7.6634	7.3189	7.4577	7.5443	7.5040	7.6238	7.5020	7.6404	7.4996	7.6317	7.5060	7.6558	**7.22**
**H5**	7.1771	7.3713	7.4596	7.4576	7.5958	7.4530	7.5980	7.4480	7.5902	7.4622	7.6214	7.1690	7.3494	7.4382	7.4277	7.5538	7.4237	7.5651	7.4194	7.5542	7.4316	7.5870	**7.22**
**H6**	8.1341	8.3392	8.4057	8.4426	8.5635	8.4366	8.5897	8.4305	8.5951	8.4485	8.6240	7.9194	8.0892	8.2103	8.1769	8.3421	8.1732	8.3457	8.1688	8.3441	8.1801	8.3934	**7.97**
**H8**	4.3741	4.4705	4.4922	4.5694	4.6265	4.5633	4.6458	4.5568	4.6625	4.5752	4.6720	4.5270	4.6702	4.7258	4.7346	4.8501	4.7322	4.8642	4.7292	4.8566	4.7367	4.8976	**4.99**
**H(9A-9C)**	1.2980	1.3052	1.2789	1.2881	1.2601	1.2903	1.2615	1.2922	1.2492	1.2862	1.2747	1.3734	1.4034	1.4070	1.4206	1.3953	1.4203	1.4103	1.4196	1.4138	1.4210	1.4367	**1.51**
**H(8`A-8`C)**	2.4555	2.3914	2.3685	2.3743	2.3708	2.3754	2.3485	2.3764	2.3407	2.3732	2.3328	2.8889	2.7195	2.6844	2.6446	2.6004	2.6510	2.5113	2.6569	2.5341	2.6383	2.4811	**2.71**
Nuclei	B98																						
**C1**	137.8984	138.3641	139.2698	138.5615	140.4825	138.5589	139.6880	138.5551	139.9714	138.5675	140.0693	136.6672	137.9623	138.5980	138.5797	140.1392	138.5482	140.0261	138.5178	139.8988	138.6168	140.0992	**129.02**
**C2**	138.5254	138.4721	139.0519	138.3240	138.8948	138.3503	139.3548	138.3736	139.2402	138.3008	139.3473	137.9447	138.0861	138.6429	138.0628	138.7300	138.0773	139.2568	138.0902	139.1509	138.0518	139.2683	**132.19**
**C3**	120.9751	121.6883	122.2401	121.9412	122.6168	121.9423	122.8875	121.9406	122.8295	121.9422	122.9429	121.0500	121.6961	122.2005	121.9352	122.6051	121.9366	122.8272	121.9354	122.7659	121.9361	122.8940	**116.16**
**C4**	173.5548	174.4326	175.0664	174.8850	175.5697	174.8724	176.2406	174.8571	176.0696	174.8994	176.3345	173.8262	174.4272	175.1004	174.7024	175.4540	174.7034	175.8475	174.7008	175.7561	174.7028	176.0007	**166.89**
**C5**	118.7946	120.1408	120.7556	120.8501	121.6768	120.8218	121.9460	120.7906	121.8434	120.8797	122.0849	118.9548	120.1808	120.7785	120.7832	121.6150	120.7632	121.7668	120.7403	121.6817	120.8040	121.9090	**116.16**
**C6**	134.9458	136.9033	137.9770	137.9779	139.4196	137.9297	140.0378	137.8779	139.7250	138.0256	140.3768	135.2851	137.2480	138.5012	138.2327	139.8890	138.1917	140.3423	138.1470	140.1749	138.2722	140.7184	**132.19**
**C7**	199.5271	204.5615	206.0295	207.1995	208.2386	207.0479	213.5136	206.8943	212.6014	207.3482	214.4291	204.1599	208.4637	209.6427	210.7144	211.5176	210.5930	216.3722	210.4697	215.2290	210.8323	216.8957	**196.22**
**C8**	65.0962	66.2575	66.8997	66.5776	67.5549	66.5763	67.8574	66.5723	67.7776	66.5806	68.7079	59.7427	61.6256	62.1747	62.5454	63.3980	62.4979	64.1348	62.4503	63.8905	62.5954	64.3856	**59.75**
**C9**	23.2897	23.5779	23.6929	23.8388	24.0941	23.8397	24.1215	23.8371	24.0489	23.8399	24.2881	20.0303	20.4587	20.5633	20.6605	20.8945	20.6623	21.0556	20.6621	20.9805	20.6615	21.4185	**15.44**
**C8`**	36.7092	37.4629	37.6740	37.7309	38.1053	37.7287	38.3351	37.7243	38.2965	37.7357	38.7417	32.2204	32.4032	32.6737	32.5780	32.8420	32.5807	32.9732	32.5806	33.0077	32.5770	33.0565	**31.11**
**H2**	8.6652	8.6018	8.6259	8.5647	8.5366	8.5677	8.5885	8.5705	8.5669	8.5621	8.5601	8.6821	8.5181	8.5678	8.4391	8.4515	8.4446	8.4365	8.4498	8.4543	8.4338	8.4469	**7.97**
**H3**	7.3240	7.4617	7.5525	7.5069	7.6251	7.5048	7.6424	7.5026	7.6335	7.5092	7.6562	7.3088	7.4478	7.5364	7.4945	7.6157	7.4921	7.6318	7.4897	7.6238	7.4970	7.6471	**7.22**
**H5**	7.1716	7.3643	7.4556	7.4510	7.5764	7.4460	7.5983	7.4411	7.5859	7.4560	7.6179	7.1649	7.3464	7.4371	7.4256	7.5523	7.4212	7.5629	7.4169	7.5523	7.4299	7.5837	**7.22**
**H6**	8.0929	8.2967	8.3821	8.4075	8.5267	8.4008	8.6016	8.3943	8.5719	8.4141	8.6200	7.8961	8.0681	8.1863	8.1586	8.3256	8.1538	8.3364	8.1488	8.3271	8.1631	8.3835	**7.97**
**H8**	4.3470	4.4366	4.4606	4.5354	4.5891	4.5287	4.6555	4.5220	4.6334	4.5423	4.6625	4.4951	4.6493	4.7037	4.7151	4.8293	4.7118	4.8454	4.7085	4.8323	4.7182	4.8756	**4.99**
**H(9A-9C)**	1.3189	1.3268	1.3143	1.3162	1.2740	1.3175	1.2894	1.3187	1.2788	1.3151	1.3029	1.3937	1.4261	1.4275	1.4419	1.4166	1.4413	1.4301	1.4407	1.4303	1.4425	1.4532	**1.51**
**H(8`A-8`C)**	2.4802	2.4260	2.3972	2.4073	2.3842	2.4082	2.3651	2.4093	2.3720	2.4067	2.3565	2.9208	2.7533	2.7171	2.6803	2.6349	2.6856	2.5498	2.6907	2.5670	2.6754	2.5194	**2.71**

[a] methyl protons shifts are averaged

[b] according to [3]

Table S11. Cont. I

Nuclei	R											S											Exp. [b]
	B971																						
	Gas	$CHCl_3$ (PCM)	$CHCl_3$ (SMD)	DMSO (PCM)	DMSO (SMD)	CH_3OH (PCM)	CH_3OH (SMD)	C_2H_5OH (PCM)	C_2H_5OH (SMD)	H_2O (PCM)	H_2O (SMD)	Gas	$CHCl_3$ (PCM)	$CHCl_3$ (SMD)	DMSO (PCM)	DMSO (SMD)	CH_3OH (PCM)	CH_3OH (SMD)	C_2H_5OH (PCM)	C_2H_5OH (SMD)	H_2O (PCM)	H_2O (SMD)	
**C1**	137.5124	137.9668	138.8036	138.1617	140.0037	138.1528	139.1730	138.1442	139.5653	138.1768	139.6476	136.2962	137.5659	138.2070	138.1911	139.7146	138.1505	139.5849	138.1134	139.4699	138.2405	139.6733	**129.02**
**C2**	138.2273	138.1790	138.7520	138.0411	138.6151	138.0588	139.0874	138.0759	138.9703	138.0298	139.0743	137.6507	137.7943	138.3494	137.7788	138.4352	137.7852	138.9549	137.7921	138.8493	137.7793	138.9688	**132.19**
**C3**	120.7723	121.4948	122.0366	121.7575	122.4239	121.7508	122.6953	121.7432	122.6290	121.7695	122.7532	120.8439	121.4989	122.0013	121.7481	122.4060	121.7414	122.6248	121.7343	122.5637	121.7602	122.6979	**116.16**
**C4**	173.4520	174.3378	174.9730	174.7959	175.4831	174.7758	176.1541	174.7552	175.9535	174.8209	176.2409	173.7091	174.3304	175.0011	174.6204	175.3683	174.6132	175.7597	174.6046	175.6659	174.6323	175.9170	**166.89**
**C5**	118.5912	119.9438	120.5513	120.6619	121.4796	120.6255	121.7575	120.5889	121.6419	120.7023	121.8945	118.7577	119.9871	120.5816	120.5974	121.4204	120.5694	121.5676	120.5405	121.4822	120.6294	121.7173	**116.16**
**C6**	134.7050	136.6708	137.7813	137.7564	139.2253	137.6992	139.8111	137.6410	139.4847	137.8168	140.1424	135.0285	137.0109	138.2571	138.0065	139.6769	137.9579	140.1333	137.9074	139.9591	138.0571	140.5087	**132.19**
**C7**	199.4123	204.3866	205.8037	206.9774	207.9980	206.8235	213.1394	206.6697	212.2541	207.1300	214.1117	204.0187	208.2806	209.4897	210.5322	211.3305	210.4028	216.0716	210.2738	214.9613	210.6620	216.6144	**196.22**
**C8**	65.0628	66.2181	66.8745	66.5493	67.4912	66.5398	67.7569	66.5294	67.7680	66.5644	68.6439	59.7053	61.5405	62.0853	62.4460	63.2954	62.3913	63.9952	62.3385	63.7609	62.5067	64.2505	**59.75**
**C9**	23.2952	23.5805	23.7317	23.8547	24.1127	23.8461	24.1179	23.8365	24.0691	23.8689	24.2944	20.0444	20.4947	20.5876	20.7006	20.9390	20.6951	21.1020	20.6896	21.0266	20.7123	21.4780	**15.44**
**C8`**	36.7170	37.4689	37.6835	37.7337	38.0702	37.7257	38.3145	37.7173	38.2603	37.7475	38.7194	32.1504	32.3460	32.6128	32.5305	32.7822	32.5259	32.9103	32.5202	32.9431	32.5401	32.9962	**31.11**
**H2**	8.6629	8.6020	8.6311	8.5654	8.5437	8.5681	8.6026	8.5708	8.5678	8.5633	8.5647	8.6781	8.5197	8.5677	8.4422	8.4548	8.4475	8.4418	8.4527	8.4583	8.4370	8.4509	**7.97**
**H3**	7.3215	7.4590	7.5493	7.5044	7.6225	7.5021	7.6392	7.4997	7.6299	7.5070	7.6527	7.3076	7.4456	7.5341	7.4931	7.6131	7.4905	7.6286	7.4879	7.6208	7.4959	7.6437	**7.22**
**H5**	7.1696	7.3613	7.4527	7.4481	7.5728	7.4430	7.5957	7.4379	7.5815	7.4533	7.6142	7.1650	7.3449	7.4351	7.4237	7.5495	7.4192	7.5594	7.4147	7.5489	7.4283	7.5805	**7.22**
**H6**	8.0974	8.2998	8.3897	8.4087	8.5283	8.4021	8.6053	8.3956	8.5653	8.4153	8.6195	7.8972	8.0707	8.1892	8.1631	8.3306	8.1581	8.3421	8.1530	8.3320	8.1679	8.3885	**7.97**
**H8**	4.3445	4.4318	4.4558	4.5292	4.5852	4.5223	4.6544	4.5157	4.6229	4.5361	4.6585	4.4978	4.6489	4.7036	4.7168	4.8297	4.7133	4.8452	4.7097	4.8315	4.7203	4.8758	**4.99**
**H(9A-9C)**	1.3191	1.3280	1.3197	1.3196	1.2780	1.3205	1.2937	1.3214	1.2832	1.3191	1.3053	1.3960	1.4276	1.4283	1.4433	1.4195	1.4425	1.4331	1.4418	1.4326	1.4442	1.4560	**1.51**
**H(8`A-8`C)**	2.4778	2.4208	2.3902	2.4035	2.3793	2.4041	2.3533	2.4048	2.3662	2.4033	2.3481	2.9110	2.7452	2.7096	2.6744	2.6284	2.6794	2.5438	2.6843	2.5610	2.6698	2.5128	**2.71**
Nuclei	B972																						
**C1**	134.4302	134.9580	135.9573	135.2517	137.1882	135.2339	136.9092	135.2172	137.2291	135.2642	136.9986	133.0960	134.5569	135.0556	135.2214	136.6788	135.1632	136.7562	135.1090	136.5422	135.2805	136.8545	**129.02**
**C2**	136.2385	136.1466	136.7094	135.9825	136.5017	135.9974	136.8781	136.0094	136.7762	135.9648	136.9004	135.6839	135.8309	136.3876	135.8469	136.4661	135.8466	136.9148	135.8443	136.8312	135.8431	136.9435	**132.19**
**C3**	118.6420	119.3619	119.9304	119.6913	120.3271	119.6734	120.6091	119.6533	120.5621	119.7042	120.6885	118.7138	119.3622	119.8746	119.6667	120.3028	119.6505	120.5119	119.6318	120.4478	119.6777	120.5982	**116.16**
**C4**	170.4080	171.2888	171.9012	171.8240	172.3935	171.7908	172.9800	171.7559	172.8201	171.8517	173.1417	170.6771	171.2152	171.8947	171.5417	172.2306	171.5278	172.5783	171.5099	172.5046	171.5489	172.7454	**166.89**
**C5**	116.5455	117.8870	118.4963	118.6438	119.4131	118.5990	119.6446	118.5521	119.5386	118.6819	119.8150	116.6811	117.8807	118.4867	118.5356	119.3203	118.4989	119.4656	118.4592	119.3807	118.5657	119.6278	**116.16**
**C6**	132.2984	134.1683	135.1909	135.2810	136.6319	135.2152	137.0709	135.1451	136.7199	135.3380	137.5017	132.7192	134.6132	135.8785	135.6223	137.1863	135.5695	137.5344	135.5120	137.4030	135.6656	137.9182	**132.19**
**C7**	197.1472	202.1820	203.6394	205.0747	205.9500	204.8855	211.4809	204.7016	210.7109	205.2515	212.1626	201.5167	205.7889	206.8296	208.0334	208.7686	207.8942	213.5796	207.7543	212.4081	208.1644	214.0847	**196.22**
**C8**	62.2129	63.1640	63.7653	63.5253	64.4519	63.5132	65.0374	63.4956	64.9409	63.5298	65.7131	56.9800	58.9172	59.3838	59.8622	60.6095	59.7928	61.6484	59.7250	61.1550	59.9290	61.7202	**59.75**
**C9**	22.2017	22.5014	22.6308	22.6974	22.9802	22.6894	22.9728	22.6772	22.8720	22.7010	23.1432	18.9615	19.3718	19.4861	19.6193	19.7902	19.6032	19.9459	19.5854	19.8717	19.6315	20.3049	**15.44**
**C8`**	35.6349	36.3454	36.5312	36.8429	37.0375	36.8098	37.2438	36.7749	37.2215	36.8693	37.6484	31.3495	31.4924	31.7924	31.7285	31.9630	31.7188	31.9659	31.7053	32.0216	31.7314	32.0500	**31.11**
**H2**	8.6537	8.5910	8.6014	8.5457	8.5058	8.5490	8.4992	8.5518	8.4749	8.5424	8.5039	8.6825	8.4992	8.5620	8.4234	8.4354	8.4301	8.3959	8.4360	8.4226	8.4161	8.4046	**7.97**
**H3**	7.3227	7.4597	7.5484	7.5068	7.6209	7.5045	7.6375	7.5020	7.6284	7.5088	7.6533	7.3120	7.4483	7.5346	7.4976	7.6140	7.4951	7.6296	7.4923	7.6214	7.4999	7.6455	**7.22**
**H5**	7.1739	7.3666	7.4552	7.4573	7.5754	7.4520	7.5903	7.4465	7.5774	7.4620	7.6138	7.1665	7.3420	7.4301	7.4225	7.5446	7.4180	7.5559	7.4132	7.5449	7.4267	7.5779	**7.22**
**H6**	8.1158	8.3161	8.3945	8.4358	8.5373	8.4282	8.5663	8.4204	8.5382	8.4429	8.6045	7.9070	8.0727	8.1924	8.1605	8.3194	8.1565	8.3186	8.1518	8.3151	8.1637	8.3642	**7.97**
**H8**	4.3399	4.4508	4.4730	4.5472	4.5919	4.5405	4.6226	4.5339	4.6079	4.5536	4.6488	4.4883	4.6298	4.6848	4.6954	4.8083	4.6924	4.8202	4.6889	4.8127	4.6977	4.8518	**4.99**
**H(9A-9C)**	1.2779	1.2737	1.2581	1.2603	1.2187	1.2618	1.2249	1.2628	1.2101	1.2586	1.2452	1.3585	1.3871	1.3915	1.4080	1.3784	1.4071	1.3926	1.4058	1.3948	1.4087	1.4173	**1.51**
**H(8`A-8`C)**	2.4444	2.3879	2.3637	2.3740	2.3543	2.3748	2.3536	2.3753	2.3559	2.3732	2.3366	2.8829	2.7146	2.6793	2.6431	2.5976	2.6486	2.5129	2.6536	2.5335	2.6373	2.4818	**2.71**
Nuclei	BHandH																						
**C1**	138.2231	138.5552	139.4722	138.6613	140.1957	138.6447	139.2014	138.6466	139.2910	138.6472	139.3414	137.3260	138.1227	139.0514	138.6686	139.4127	138.6143	139.9113	138.5817	139.6959	138.6934	140.0677	**129.02**
**C2**	143.5201	143.3955	144.0049	143.2268	143.8191	143.2277	144.3374	143.2468	144.2139	143.1959	144.3719	143.1561	143.1628	143.7749	143.0888	143.8329	143.0819	144.2258	143.0940	144.0867	143.0659	144.2500	**132.19**
**C3**	123.7766	124.4896	125.0912	124.7508	125.4195	124.7262	125.6101	124.7198	125.5593	124.7437	125.6833	123.8317	124.5005	125.1213	124.7551	125.4992	124.7302	125.6175	124.7238	125.5792	124.7487	125.7026	**116.16**
**C4**	174.6910	175.5022	176.1372	175.9474	176.5413	175.9096	177.2138	175.8903	177.0668	175.9534	177.3817	174.9086	175.5511	176.2159	175.8630	176.6071	175.8357	176.9768	175.8261	176.9107	175.8585	177.1498	**166.89**
**C5**	121.3254	122.7876	123.4824	123.5575	124.4251	123.5004	124.6212	123.4617	124.5293	123.5816	124.7930	121.4351	122.8143	123.5066	123.5206	124.2647	123.4688	124.5249	123.4348	124.4609	123.5397	124.7001	**116.16**
**C6**	139.9383	142.0591	143.3061	143.2147	144.9304	143.1340	145.6585	143.0715	145.4144	143.2611	146.1074	140.2023	142.4966	143.7026	143.6179	144.3620	143.5430	145.9734	143.4853	145.7743	143.6576	146.3435	**132.19**
**C7**	202.9300	207.7859	209.1882	210.2924	210.9441	210.1306	215.8168	209.9874	214.6983	210.4156	216.6335	207.3621	211.4788	213.0278	213.6769	214.4210	213.5328	219.1023	213.4077	218.0494	213.7841	219.7557	**196.22**
**C8**	59.9494	61.2143	61.9067	61.6436	62.6312	61.6098	63.0978	61.5942	62.9356	61.6453	63.8442	54.0631	55.4214	55.9382	56.1974	56.9415	56.1327	57.6583	56.0882	57.3239	56.2306	57.9608	**59.75**
**C9**	22.4684	22.7173	23.0362	22.9961	23.4920	22.9658	23.4131	22.9537	23.4079	22.9951	23.6098	19.0747	19.5603	19.6330	19.8271	20.5712	19.7976	20.2453	19.7870	20.1575	19.8256	20.6529	**15.44**
**C8`**	33.7500	34.5612	34.8118	34.8998	35.2711	34.8687	35.5037	34.8562	35.4510	34.8996	35.9918	28.6454	29.0946	29.4036	29.3316	30.0757	29.3102	29.7782	29.3056	29.7931	29.3204	29.9429	**31.11**
**H2**	8.9875	8.9200	8.9348	8.8802	8.8770	8.8834	8.9222	8.8858	8.9249	8.8781	8.9222	8.9840	8.8580	8.8706	8.7732	8.6920	8.7803	8.7507	8.7862	8.7811	8.7672	8.7533	**7.97**
**H3**	7.4553	7.5997	7.6892	7.6466	7.7674	7.6451	7.7842	7.6427	7.7763	7.6491	7.8000	7.4516	7.5922	7.6830	7.6387	7.5575	7.6372	7.7745	7.6347	7.7677	7.6412	7.7910	**7.22**
**H5**	7.3003	7.5022	7.5933	7.5917	7.7206	7.5873	7.7378	7.5822	7.7268	7.5967	7.7629	7.2950	7.4886	7.5794	7.5727	7.4915	7.5687	7.7122	7.5639	7.7021	7.5774	7.7361	**7.22**
**H6**	8.3317	8.5445	8.6361	8.6585	8.7941	8.6524	8.8645	8.6454	8.8495	8.6654	8.9071	8.1293	8.3337	8.4417	8.4287	8.3475	8.4246	8.6263	8.4194	8.6221	8.4334	8.6718	**7.97**
**H8**	4.1505	4.2528	4.2749	4.3463	4.3866	4.3407	4.4438	4.3343	4.4339	4.3528	4.4693	4.4049	4.5696	4.6414	4.6481	4.5669	4.6447	4.7936	4.6404	4.7922	4.6522	4.8291	**4.99**
**H(9A-9C)**	1.3947	1.4083	1.3944	1.4043	1.3835	1.4056	1.4087	1.4060	1.4066	1.4041	1.4308	1.4777	1.5048	1.4783	1.5114	1.4301	1.5120	1.4963	1.5117	1.4978	1.5117	1.5179	**1.51**
**H(8`A-8`C)**	2.5482	2.4741	2.4402	2.4443	2.4243	2.4467	2.3801	2.4483	2.3790	2.4430	2.3667	2.9170	2.7545	2.7130	2.6709	2.5897	2.6772	2.5211	2.6825	2.5633	2.6659	2.4973	**2.71**
Nuclei	BHandHLYP																						
**C1**	138.8640	139.2644	139.9330	139.4014	141.4115	139.3890	140.3239	139.3799	140.8073	139.4140	140.7808	137.7359	138.7863	139.5663	139.4872	141.0279	139.4436	140.6370	139.4057	140.4914	139.5319	140.6845	**129.02**
**C2**	142.1451	142.0033	142.5518	141.8479	142.3133	141.8593	142.8334	141.8728	142.6946	141.8367	142.8238	141.6846	141.6776	142.2130	141.6154	142.1662	141.6189	142.6674	141.6254	142.5678	141.6119	142.7010	**132.19**
**C3**	122.5311	123.1246	123.6134	123.3344	123.9644	123.3231	124.1502	123.3143	124.1344	123.3446	124.2197	122.6170	123.1643	123.6329	123.3811	123.9687	123.3691	124.1171	123.3594	124.0780	123.3922	124.1810	**116.16**
**C4**	174.4859	175.2386	175.8387	175.6759	176.2231	175.6490	176.9757	175.6248	176.7716	175.7009	177.0601	174.7783	175.2638	175.8588	175.5025	176.1211	175.4893	176.5698	175.4775	176.5010	175.5141	176.7410	**166.89**
**C5**	120.1712	121.4535	122.0189	122.1435	122.9398	122.1013	123.1049	122.0616	123.0479	122.1835	123.2751	120.2936	121.5123	122.0694	122.1337	122.8970	122.0972	123.0006	122.0628	122.9395	122.1680	123.1441	**116.16**
**C6**	138.1510	140.1493	141.3422	141.2896	142.5504	141.2223	143.2999	141.1564	142.9195	141.3529	143.5841	138.4674	140.5298	141.7475	141.5098	143.1000	141.4526	143.6578	141.3968	143.5207	141.5620	144.0715	**132.19**
**C7**	201.2543	206.5546	207.7752	209.3182	210.2498	209.1454	216.0604	208.9760	215.0849	209.4828	217.0651	206.1611	210.4578	211.6283	212.8433	213.4066	212.7018	218.6263	212.5625	217.3826	212.9773	219.2046	**196.22**
**C8**	60.7965	61.8811	62.5000	62.2233	63.0588	62.2069	63.2656	62.1924	63.2636	62.2383	64.1041	55.4804	57.1821	57.8021	58.1054	58.8956	58.0473	59.5516	57.9937	59.2868	58.1628	59.8130	**59.75**
**C9**	22.2333	22.3886	22.6124	22.6282	22.8233	22.6139	22.7047	22.6016	22.6825	22.6409	22.8547	18.9648	19.2708	19.4004	19.4144	19.6557	19.4060	19.6995	19.4005	19.6674	19.4225	20.0180	**15.44**
**C8`**	34.7377	35.5130	35.7819	35.8289	36.1801	35.8108	36.3989	35.7951	36.3597	35.8459	36.8091	30.5960	30.8142	31.0655	30.9812	31.2247	30.9680	31.3407	30.9569	31.3887	30.9927	31.4699	**31.11**
**H2**	8.8761	8.8039	8.8520	8.7659	8.7241	8.7690	8.7906	8.7718	8.7505	8.7629	8.7519	8.8981	8.7359	8.7665	8.6405	8.6466	8.6464	8.6344	8.6519	8.6571	8.6345	8.6458	**7.97**
**H3**	7.4433	7.5783	7.6654	7.6233	7.7361	7.6213	7.7566	7.6190	7.7465	7.6252	7.7698	7.4323	7.5668	7.6511	7.6133	7.7280	7.6111	7.7441	7.6087	7.7353	7.6153	7.7592	**7.22**
**H5**	7.2878	7.4785	7.5663	7.5650	7.6852	7.5602	7.7061	7.5552	7.6928	7.5695	7.7273	7.2798	7.4603	7.5446	7.5400	7.6603	7.5356	7.6720	7.5311	7.6611	7.5440	7.6938	**7.22**
**H6**	8.1765	8.3928	8.4992	8.5149	8.6356	8.5078	8.7226	8.5005	8.6822	8.5217	8.7376	8.0113	8.2014	8.3159	8.2915	8.4587	8.2866	8.4826	8.2813	8.4746	8.2959	8.5310	**7.97**
**H8**	4.0776	4.1885	4.2247	4.3024	4.3719	4.2945	4.4593	4.2868	4.4300	4.3099	4.4624	4.2587	4.4280	4.4860	4.5022	4.6169	4.4983	4.6464	4.4943	4.6344	4.5056	4.6772	**4.99**
**H(9A-9C)**	1.2993	1.3062	1.3063	1.3002	1.2417	1.3014	1.2609	1.3023	1.2448	1.2990	1.2689	1.3682	1.3960	1.3920	1.4076	1.3798	1.4070	1.4019	1.4061	1.4026	1.4081	1.4244	**1.51**
**H(8`A-8`C)**	2.4396	2.3870	2.3452	2.3723	2.3466	2.3731	2.3248	2.3737	2.3360	2.3717	2.3239	2.8786	2.7124	2.6679	2.6359	2.5874	2.6411	2.5108	2.6458	2.5345	2.6307	2.4845	**2.71**

[a] methyl protons shifts are averaged

[b] according to [3]

Table S11. Cont. II

Nuclei	R											S											Exp. [b]
	BLYP																						
	Gas	$CHCl_3$ (PCM)	$CHCl_3$ (SMD)	DMSO (PCM)	DMSO (SMD)	CH_3OH (PCM)	CH_3OH (SMD)	C_2H_5OH (PCM)	C_2H_5OH (SMD)	H_2O (PCM)	H_2O (SMD)	Gas	$CHCl_3$ (PCM)	$CHCl_3$ (SMD)	DMSO (PCM)	DMSO (SMD)	CH_3OH (PCM)	CH_3OH (SMD)	C_2H_5OH (PCM)	C_2H_5OH (SMD)	H_2O (PCM)	H_2O (SMD)	
**C1**	139.2740	140.0627	140.7302	140.3557	142.0482	140.6261	141.6136	140.4250	141.4809	140.7208	141.8598	138.3037	138.8878	139.5939	139.5904	141.4131	139.8236	141.0985	139.5956	141.3416	139.9982	141.2014	**129.02**
**C2**	137.3888	137.3557	137.9052	137.1077	137.7439	137.4006	137.9857	137.2334	138.0955	137.4576	138.0801	136.8040	136.8446	137.3898	136.8417	137.6112	137.1207	137.9926	136.9376	138.0835	137.2041	138.0703	**132.19**
**C3**	120.6544	121.3842	121.8707	121.6716	122.2855	121.9330	122.4590	121.7316	122.5866	122.0499	122.6336	119.6392	121.4514	121.9240	121.6569	122.3097	121.9238	122.3772	121.7284	122.4989	122.0306	122.5353	**116.16**
**C4**	177.6519	178.7185	179.3932	179.3068	180.0988	179.5462	180.5234	179.3230	180.6223	179.7057	180.7915	178.1086	178.8813	179.6485	179.1527	180.0400	179.4160	180.2300	179.2172	180.2861	179.5290	180.4844	**166.89**
**C5**	118.5470	119.8601	120.3998	120.5456	121.3222	120.7815	121.4404	120.5550	121.5434	120.9483	121.6902	118.6599	120.0133	120.5942	120.5540	121.3508	120.8003	121.3513	120.5845	121.4329	120.9468	121.5891	**116.16**
**C6**	133.7911	135.6052	136.7931	136.6678	138.3637	136.8753	138.6685	136.6243	138.6992	137.1000	139.1691	138.8182	136.2738	137.4058	137.2526	138.9036	137.4733	139.2393	137.2316	139.1572	137.6692	139.7543	**132.19**
**C7**	203.2767	208.6048	209.8305	211.6985	212.3595	211.7964	217.3300	211.4147	216.3806	212.2229	218.3121	205.2971	212.7975	214.3032	214.9277	216.0897	215.0775	220.0988	214.7652	219.4181	215.4121	220.5992	**196.22**
**C8**	69.1241	70.1253	70.7992	70.3573	71.5764	70.6395	71.9231	70.4601	71.8008	70.7169	72.6971	68.1488	65.4631	65.8331	66.4810	67.5528	66.6957	68.0924	66.4492	68.1157	66.9054	68.4506	**59.75**
**C9**	23.7215	24.0520	24.2062	24.1836	24.6500	24.4455	24.6108	24.2503	24.7226	24.5640	24.8492	16.4537	20.8061	20.7678	21.0333	21.2955	21.2977	21.4360	21.1003	21.5017	21.4093	21.9663	**15.44**
**C8`**	38.2242	38.9477	39.0730	39.4073	39.6721	39.6658	39.7283	39.4601	39.9360	39.7878	40.2492	32.8912	33.8280	34.1359	33.9651	34.2232	34.2348	34.1613	34.0423	34.3099	34.3359	34.3012	**31.11**
**H2**	8.3954	8.3311	8.3657	8.2850	8.2783	8.3345	8.4554	8.2924	8.3194	8.3386	8.4701	8.3743	8.3060	8.3480	8.2230	8.2163	8.2757	8.3816	8.2357	8.2114	8.2732	8.4083	**7.97**
**H3**	7.1679	7.2953	7.3831	7.3318	7.4491	7.3773	7.6306	7.3305	7.4581	7.3888	7.6578	7.0966	7.2855	7.3681	7.3259	7.4422	7.3712	7.6247	7.3239	7.4500	7.3832	7.6520	**7.22**
**H5**	7.0230	7.2052	7.2897	7.2883	7.4065	7.3310	7.5902	7.2811	7.4161	7.3480	7.6256	6.9899	7.1903	7.2755	7.2597	7.3808	7.3031	7.5580	7.2540	7.3806	7.3187	7.5918	**7.22**
**H6**	7.9452	8.1295	8.2114	8.2406	8.3476	8.2808	8.5506	8.2286	8.3909	8.3029	8.6025	8.9754	7.8764	7.9806	7.9535	8.0977	7.9966	8.2837	7.9470	8.0952	8.0128	8.3454	**7.97**
**H8**	4.5730	4.6582	4.6658	4.7406	4.7734	4.7808	4.9699	4.7284	4.7971	4.8027	5.0172	4.4807	4.8131	4.8614	4.8724	4.9776	4.9165	5.1608	4.8680	4.9755	4.9306	5.2060	**4.99**
**H(9A-9C)**	1.3344	1.3224	1.3240	1.2985	1.2925	1.3477	1.4693	1.3049	1.3035	1.3524	1.4944	1.2856	1.4612	1.4487	1.4808	1.4580	1.5270	1.6506	1.4807	1.4754	1.5371	1.6944	**1.51**
**H(8`A-8`C)**	2.5702	2.5055	2.4761	2.4845	2.4652	2.5330	2.6299	2.4890	2.4571	2.5387	2.6207	2.5654	2.8839	2.8478	2.7998	2.7482	2.8522	2.8332	2.8121	2.6782	2.8501	2.8119	**2.71**
Nuclei	BMK																						
**C1**	154.5489	154.7956	155.8147	155.0346	156.7869	155.0048	155.7582	155.1850	155.9437	155.2232	155.7278	153.3708	154.7062	155.5737	155.3493	157.1804	155.2822	157.0114	155.4300	156.7717	155.5792	157.0888	**129.02**
**C2**	155.5489	155.3349	156.1444	155.0820	155.8798	155.0811	156.4462	155.2910	156.2529	155.2438	156.4023	154.7572	154.6732	155.3927	154.5734	155.4851	154.5591	155.9928	154.7574	155.7913	154.7494	155.9653	**132.19**
**C3**	135.9186	136.5742	137.2866	136.7717	137.6790	136.7457	137.8453	136.9307	137.6978	136.9577	137.8467	136.0540	136.5812	137.2516	136.7609	137.6638	136.7339	137.8342	136.9186	137.6908	136.9479	137.8865	**116.16**
**C4**	189.4005	190.1835	191.0076	190.6219	191.5290	190.5810	192.1342	190.7513	191.8731	190.8217	192.2418	189.7985	190.2293	191.0560	190.4438	191.4104	190.4180	191.7002	190.6026	191.5626	190.6291	191.8308	**166.89**
**C5**	133.4295	134.7613	135.4788	135.4278	136.5512	135.3675	136.6886	135.5196	136.4925	135.6472	136.8148	133.4535	134.7683	135.5390	135.3777	136.4835	135.3275	136.5822	135.4877	136.3606	135.5868	136.7328	**116.16**
**C6**	151.4094	153.6757	155.2637	155.0157	156.8896	154.9195	157.7543	155.0316	157.2746	155.2662	158.1427	152.1159	153.9639	155.4209	155.0094	157.0550	154.9327	157.4837	155.0660	157.2182	155.2429	157.8187	**132.19**
**C7**	216.9478	222.5474	224.1482	225.4916	226.2743	225.2972	232.0453	225.3132	230.7791	225.8361	232.8163	221.5389	226.5692	228.0915	229.2000	230.0942	229.0219	235.6763	229.0559	234.3215	229.5316	236.4133	**196.22**
**C8**	70.5725	71.6400	72.4428	72.1543	73.3210	72.1213	73.7576	72.2984	73.5299	72.3466	74.4771	64.4902	66.3795	67.0821	67.2150	68.2924	67.1424	69.1388	67.2827	68.7153	67.4478	69.3740	**59.75**
**C9**	25.4816	25.8566	26.3477	26.2252	26.6325	26.1938	26.7756	26.3697	26.5780	26.4143	26.8310	22.1260	22.5585	22.7087	22.6468	23.0607	22.6251	23.2222	22.8150	23.0572	22.8297	23.5137	**15.44**
**C8`**	38.7886	39.4905	39.7965	39.7916	40.2856	39.7653	40.4650	39.9500	40.3114	39.9781	40.8997	33.9443	34.1818	34.5674	34.3474	34.7515	34.3245	34.7335	34.5118	34.8069	34.5294	34.8356	**31.11**
**H2**	9.0522	9.0233	9.0737	8.9738	8.9666	8.9734	9.0551	8.9730	9.0302	8.9645	9.0669	9.0430	8.8827	8.9188	8.8117	8.8174	8.8133	8.8099	8.8146	8.8396	8.8005	8.8138	**7.97**
**H3**	7.6503	7.8088	7.9094	7.8680	7.9716	7.8622	8.0008	7.8561	7.9908	7.8636	8.0111	7.6582	7.8152	7.8999	7.8689	7.9851	7.8629	7.9933	7.8568	7.9965	7.8650	8.0096	**7.22**
**H5**	7.5098	7.7043	7.7841	7.7896	7.9181	7.7809	7.9299	7.7723	7.9192	7.7883	7.9561	7.4861	7.6973	7.7852	7.7793	7.9021	7.7717	7.9135	7.7640	7.8990	7.7768	7.9393	**7.22**
**H6**	8.4072	8.6270	8.7828	8.7961	8.9052	8.7837	9.0195	8.7706	8.9763	8.7979	9.0628	8.3050	8.4960	8.6092	8.6017	8.7715	8.5925	8.7332	8.5831	8.7698	8.6007	8.7910	**7.97**
**H8**	4.2317	4.3488	4.4104	4.4856	4.5292	4.4731	4.5903	4.4604	4.5756	4.4876	4.6005	4.4400	4.6482	4.6995	4.7284	4.8382	4.7206	4.8188	4.7127	4.8373	4.7262	4.8615	**4.99**
**H(9A-9C)**	1.3048	1.3335	1.3264	1.3372	1.2998	1.3347	1.3417	1.3320	1.3281	1.3300	1.3703	1.3776	1.3895	1.3774	1.4034	1.3701	1.3999	1.3895	1.3963	1.3784	1.3971	1.4125	**1.51**
**H(8`A-8`C)**	2.4334	2.3750	2.3238	2.3567	2.3235	2.3539	2.2956	2.3513	2.3080	2.3498	2.2771	2.8035	2.6688	2.6299	2.6096	2.5602	2.6105	2.4481	2.6113	2.4916	2.5990	2.4288	**2.71**
Nuclei	BP86																						
**C1**	135.9939	136.6320	137.6362	136.9544	138.7298	136.9390	138.2948	136.9186	138.3141	136.9642	138.7051	134.4352	135.9816	136.4890	136.5815	138.0085	136.5423	138.0378	136.5022	137.9223	136.6187	138.2985	**129.02**
**C2**	136.0461	136.1412	136.6915	135.9389	136.4876	135.9629	136.9072	135.9812	136.7985	135.9104	137.0603	135.2799	135.6510	136.1578	135.6672	136.3174	135.6758	136.8585	135.6802	136.7480	135.6552	137.0159	**132.19**
**C3**	119.5556	120.4857	121.0080	120.7308	121.3490	120.7247	121.6928	120.7132	121.6404	120.7314	121.9360	119.6977	120.5178	120.9979	120.7317	121.3594	120.7284	121.6195	120.7195	121.5439	120.7299	121.8637	**116.16**
**C4**	174.3602	175.5049	176.1304	175.8536	176.5630	175.8334	177.1710	175.8083	177.0813	175.8678	177.4848	174.7072	175.5024	176.1986	175.7445	176.5291	175.7420	176.8841	175.7330	176.7851	175.7408	177.2044	**166.89**
**C5**	117.4531	118.9871	119.5529	119.6506	120.4030	119.6163	120.7206	119.5768	120.6236	119.6782	121.0496	117.6959	119.0396	119.6143	119.6262	120.4183	119.6030	120.5906	119.5742	120.4936	119.6424	120.9111	**116.16**
**C6**	132.4823	134.5222	135.5397	135.4991	136.9975	135.4473	137.5315	135.3905	137.2521	135.5440	138.0880	132.8422	134.9807	136.2191	135.9532	137.6251	135.9065	138.0996	135.8536	137.8931	135.9910	138.6398	**132.19**
**C7**	200.3568	205.1844	206.6915	207.6362	208.7456	207.4854	213.3518	207.3301	212.6663	207.7757	214.3906	205.2587	209.2590	210.3906	211.2385	212.1476	211.1283	216.2414	211.0136	215.3050	211.3390	216.8705	**196.22**
**C8**	67.0914	68.3350	69.0224	68.6336	69.6697	68.6283	70.1232	68.6177	69.8169	68.6343	71.1003	61.2199	63.3467	63.8475	64.2565	65.1036	64.1999	65.8529	64.1410	65.6179	64.3090	66.2851	**59.75**
**C9**	23.4788	23.9387	24.0159	24.1770	24.4194	24.1716	24.5594	24.1605	24.4086	24.1770	24.8922	20.0447	20.8111	20.8964	21.0566	21.2467	21.0482	21.5506	21.0352	21.4325	21.0604	22.1852	**15.44**
**C8`**	37.5056	38.3780	38.4809	38.5899	38.8300	38.5853	39.0439	38.5763	39.0514	38.5903	39.6736	32.7385	32.9732	33.1909	33.0751	33.2543	33.0771	33.3634	33.0731	33.3752	33.0673	33.5888	**31.11**
**H2**	8.4690	8.4251	8.4168	8.3721	8.3303	8.3765	8.3610	8.3805	8.3653	8.3675	8.3657	8.5023	8.3508	8.3888	8.2771	8.2780	8.2836	8.2597	8.2894	8.2717	8.2701	8.2813	**7.97**
**H3**	7.1833	7.3254	7.4023	7.3644	7.4695	7.3630	7.4872	7.3613	7.4789	7.3653	7.5157	7.1759	7.3137	7.3896	7.3551	7.4646	7.3535	7.4797	7.3516	7.4705	7.3561	7.5088	**7.22**
**H5**	7.0316	7.2248	7.3017	7.3045	7.4170	7.3004	7.4360	7.2960	7.4267	7.3081	7.4730	7.0289	7.2047	7.2838	7.2819	7.3979	7.2785	7.4064	7.2747	7.3955	7.2847	7.4416	**7.22**
**H6**	8.0317	8.2270	8.2775	8.3141	8.4038	8.3094	8.4531	8.3044	8.4474	8.3183	8.4976	7.7817	7.9419	8.0474	8.0258	8.1760	8.0224	8.1879	8.0185	8.1750	8.0284	8.2496	**7.97**
**H8**	4.6085	4.6845	4.6748	4.7603	4.7803	4.7553	4.8233	4.7502	4.8256	4.7646	4.8587	4.7327	4.8659	4.9018	4.9243	5.0225	4.9224	5.0356	4.9199	5.0205	4.9256	5.0853	**4.99**
**H(9A-9C)**	1.3656	1.3684	1.3441	1.3548	1.3143	1.3570	1.3284	1.3589	1.3132	1.3524	1.3589	1.4453	1.4875	1.4805	1.5086	1.4790	1.5084	1.4981	1.5079	1.4947	1.5083	1.5410	**1.51**
**H(8`A-8`C)**	2.5546	2.4795	2.4461	2.4610	2.4324	2.4627	2.4144	2.4642	2.4111	2.4590	2.4105	3.0093	2.8351	2.7840	2.7540	2.6945	2.7609	2.6012	2.7671	2.6178	2.7468	2.5828	**2.71**
Nuclei	CAM-B3LYP																						
**C1**	140.5940	141.0501	141.9502	141.2482	143.3376	141.2328	142.6219	141.2195	142.7966	141.2664	142.8916	139.3811	140.7764	141.3414	141.3955	142.9372	141.3447	142.8551	141.3003	142.5657	141.4530	142.9212	**129.02**
**C2**	142.4322	142.3676	142.8920	142.2031	142.7302	142.2185	143.2393	142.2342	143.1183	142.1902	143.2313	141.9632	142.0722	142.5741	142.0137	142.6143	142.0196	143.1220	142.0264	143.0134	142.0107	143.1501	**132.19**
**C3**	123.8770	124.5297	125.0152	124.7351	125.3944	124.7281	125.5969	124.7212	125.5656	124.7437	125.6728	123.9590	124.5672	125.0168	124.7695	125.4058	124.7619	125.5862	124.7548	125.5220	124.7791	125.6510	**116.16**
**C4**	177.6432	178.4616	179.0236	178.8866	179.5004	178.8652	180.0958	178.8438	179.9671	178.9091	180.2452	177.9270	178.4619	179.0815	178.7166	179.4216	178.7091	179.7910	178.7009	179.7287	178.7245	179.9540	**166.89**
**C5**	121.6531	122.9882	123.5423	123.6761	124.5002	123.6378	124.6833	123.5998	124.6086	123.7146	124.8524	121.7948	123.0474	123.5984	123.6473	124.4684	123.6163	124.5780	123.5853	124.4990	123.6786	124.7226	**116.16**
**C6**	138.6325	140.6802	141.7768	141.7799	143.1835	141.7181	143.7450	141.6562	143.4517	141.8399	144.1171	138.9686	141.0487	142.3055	142.0534	143.7538	142.0017	144.1534	141.9484	144.0548	142.1023	144.5739	**132.19**
**C7**	205.1841	210.4669	211.8770	213.2659	214.3729	213.0946	219.8334	212.9249	218.8841	213.4315	220.8758	210.1044	214.5528	215.7343	216.9360	217.6675	216.7972	222.7736	216.6596	221.5003	217.0720	223.3268	**196.22**
**C8**	63.5728	64.7701	65.4574	65.1172	66.1087	65.1025	66.5638	65.0883	66.3449	65.1333	67.2834	57.9234	59.8230	60.3104	60.7065	61.5242	60.6433	62.2922	60.5845	61.9726	60.7729	62.5903	**59.75**
**C9**	22.4381	22.6575	22.8386	22.9008	23.1565	22.8881	23.1031	22.8752	23.0434	22.9144	23.2395	19.2771	19.6649	19.7105	19.7757	20.0125	19.7699	20.0783	19.7654	20.0320	19.7840	20.4496	**15.44**
**C8`**	35.5076	36.3386	36.5579	36.6501	37.0360	36.6375	37.2208	36.6255	37.1657	36.6643	37.6889	31.0486	31.2767	31.5537	31.4840	31.7595	31.4764	31.8336	31.4681	31.8936	31.4915	31.9753	**31.11**
**H2**	8.7689	8.7049	8.7244	8.6617	8.6168	8.6654	8.6438	8.6690	8.6389	8.6580	8.6285	8.7980	8.6167	8.6695	8.5340	8.5473	8.5409	8.5118	8.5472	8.5462	8.5267	8.5219	**7.97**
**H3**	7.4063	7.5412	7.6281	7.5846	7.6999	7.5826	7.7160	7.5807	7.7091	7.5865	7.7301	7.3996	7.5311	7.6171	7.5747	7.6929	7.5726	7.7060	7.5705	7.6986	7.5766	7.7206	**7.22**
**H5**	7.2620	7.4507	7.5382	7.5345	7.6567	7.5299	7.6715	7.5253	7.6620	7.5389	7.6941	7.2579	7.4349	7.5221	7.5119	7.6365	7.5077	7.6445	7.5036	7.6350	7.5158	7.6658	**7.22**
**H6**	8.1470	8.3590	8.4476	8.4737	8.5894	8.4673	8.6366	8.4608	8.6223	8.4796	8.6702	7.9705	8.1468	8.2705	8.2373	8.4092	8.2331	8.4121	8.2286	8.4132	8.2409	8.4600	**7.97**
**H8**	4.2201	4.3249	4.3484	4.4341	4.4931	4.4267	4.5388	4.4195	4.5400	4.4412	4.5650	4.3966	4.5587	4.6191	4.6315	4.7523	4.6282	4.7687	4.6248	4.7611	4.6343	4.8001	**4.99**
**H(9A-9C)**	1.3297	1.3390	1.3314	1.3291	1.2821	1.3304	1.2971	1.3316	1.2847	1.3277	1.3077	1.4016	1.4258	1.4289	1.4408	1.4198	1.4402	1.4318	1.4396	1.4375	1.4411	1.4563	**1.51**
**H(8`A-8`C)**	2.5132	2.4524	2.4238	2.4345	2.4170	2.4350	2.4044	2.4357	2.4044	2.4340	2.3914	2.9425	2.7653	2.7314	2.6913	2.6489	2.6974	2.5595	2.7032	2.5861	2.6851	2.5306	**2.71**

[a] methyl protons shifts are averaged

[b] according to [3]

Table S11. Cont. III

Nuclei	R											S											Exp. [b]
	HF																						
	Gas	$CHCl_3$ (PCM)	$CHCl_3$ (SMD)	DMSO (PCM)	DMSO (SMD)	CH_3OH (PCM)	CH_3OH (SMD)	C_2H_5OH (PCM)	C_2H_5OH (SMD)	H_2O (PCM)	H_2O (SMD)	Gas	$CHCl_3$ (PCM)	$CHCl_3$ (SMD)	DMSO (PCM)	DMSO (SMD)	CH_3OH (PCM)	CH_3OH (SMD)	C_2H_5OH (PCM)	C_2H_5OH (SMD)	H_2O (PCM)	H_2O (SMD)	
**C1**	136.0859	136.4286	136.9983	136.5642	138.6394	136.5601	136.8559	136.5607	137.5918	136.5707	137.8340	135.1106	136.0099	136.9165	136.8715	138.6196	136.8298	138.1526	136.7906	138.0357	136.9136	138.1693	**129.02**
**C2**	144.2959	144.1109	144.6184	143.9366	144.2969	143.9493	145.0625	143.9624	144.8336	143.9247	144.9263	144.1862	143.9606	144.4423	143.6963	144.1843	143.7105	144.6238	143.7251	144.5752	143.6812	144.6859	**132.19**
**C3**	120.5425	120.9815	121.3393	121.0997	121.6987	121.0951	121.5914	121.0919	121.6723	121.1042	121.7618	120.5755	121.0153	121.3955	121.1911	121.7425	121.1823	121.7739	121.1741	121.7432	121.1992	121.8101	**116.16**
**C4**	171.0685	171.6891	172.2443	172.0947	172.4686	172.0696	173.4111	172.0452	173.0954	172.1179	173.3624	171.3313	171.6695	172.1524	171.7723	172.2704	171.7643	172.7640	171.7564	172.6801	171.7787	172.9382	**166.89**
**C5**	117.7778	118.9699	119.4399	119.5840	120.3789	119.5486	120.2895	119.5143	120.3140	119.6174	120.5553	117.8113	119.0523	119.5559	119.6851	120.4237	119.6494	120.4603	119.6142	120.3849	119.7186	120.5859	**116.16**
**C6**	139.8710	141.8434	143.0414	142.9329	144.0689	142.8614	145.3610	142.7878	144.7144	142.9974	145.2266	140.2153	142.1535	143.2403	142.9363	144.2865	142.8868	144.8278	142.8360	144.6896	142.9813	145.2440	**132.19**
**C7**	195.4655	201.2748	202.3678	204.5817	204.9342	204.3815	211.4166	204.1898	210.2401	204.7750	212.8652	200.3146	204.7891	205.8595	207.3684	207.7885	207.2162	214.0876	207.0655	212.5722	207.5129	214.7501	**196.22**
**C8**	54.6914	55.6162	56.0452	55.7978	56.6132	55.7884	56.8235	55.7770	56.8761	55.8047	57.6680	49.9453	51.5670	52.1910	52.5059	53.2429	52.4542	53.9705	52.4041	53.6974	52.5554	54.1978	**59.75**
**C9**	20.7069	20.7584	20.8662	20.7421	21.0481	20.7452	20.9072	20.7468	20.8745	20.7388	20.9507	17.7976	17.8056	17.9115	17.8577	18.0665	17.8560	17.9858	17.8550	17.9557	17.8592	18.1883	**15.44**
**C8`**	32.6429	33.3486	33.5660	33.7469	34.0103	33.7215	34.1987	33.6970	34.1551	33.7689	34.5312	29.0930	29.3330	29.5248	29.4519	29.7044	29.4395	29.7559	29.4273	29.7928	29.4622	29.8640	**31.11**
**H2**	9.0358	8.9576	9.0240	8.9122	8.8795	8.9150	8.9903	8.9172	8.9268	8.9093	8.8935	9.0687	8.8942	8.9168	8.7657	8.7690	8.7719	8.7430	8.7777	8.7685	8.7596	8.7567	**7.97**
**H3**	7.4286	7.5694	7.6559	7.6163	7.7372	7.6140	7.7469	7.6115	7.7419	7.6185	7.7658	7.4270	7.5652	7.6521	7.6122	7.7319	7.6098	7.7417	7.6072	7.7343	7.6145	7.7576	**7.22**
**H5**	7.2620	7.4585	7.5485	7.5471	7.6720	7.5420	7.6831	7.5367	7.6720	7.5520	7.7063	7.2588	7.4515	7.5381	7.5363	7.6607	7.5316	7.6678	7.5266	7.6572	7.5408	7.6908	**7.22**
**H6**	8.1772	8.4177	8.5445	8.5717	8.7053	8.5623	8.8158	8.5526	8.7578	8.5806	8.8070	8.0620	8.2816	8.3955	8.3692	8.5389	8.3639	8.5572	8.3581	8.5485	8.3743	8.6055	**7.97**
**H8**	3.6262	3.7734	3.8337	3.9160	4.0002	3.9073	4.0866	3.8987	4.0448	3.9245	4.0816	3.7998	3.9985	4.0614	4.0795	4.2064	4.0749	4.2416	4.0700	4.2283	4.0838	4.2685	**4.99**
**H(9A-9C)**	1.2094	1.2101	1.2114	1.1856	1.1335	1.1867	1.1817	1.1873	1.1548	1.1845	1.1607	1.2797	1.2815	1.2708	1.2849	1.2453	1.2842	1.2618	1.2832	1.2631	1.2857	1.2803	**1.51**
**H(8`A-8`C)**	2.2583	2.2315	2.1858	2.2178	2.1970	2.2185	2.1768	2.2190	2.1940	2.2172	2.1932	2.6962	2.5446	2.4939	2.4664	2.4243	2.4706	2.3592	2.4746	2.3767	2.4625	2.3361	**2.71**
Nuclei	**LC-BLYP**																						
**C1**	143.9454	144.3001	145.4097	144.5079	146.3392	144.4682	145.1219	144.4578	146.1915	144.5303	146.2836	142.9429	144.2114	144.8868	144.9354	146.3990	144.8458	146.3781	144.7913	145.9849	145.0111	146.5188	**129.02**
**C2**	148.5022	148.3123	148.9120	148.1799	148.7307	148.1681	149.3075	148.1834	149.1118	148.1736	149.2571	148.2367	148.1595	148.7302	148.0898	148.6521	148.0728	149.1473	148.0835	149.0482	148.0884	149.1787	**132.19**
**C3**	128.3981	128.9432	129.5272	129.1700	129.8249	129.1365	129.9435	129.1302	129.9864	129.1845	130.1049	128.4577	128.9850	129.5383	129.2181	129.8639	129.1821	130.0193	129.1738	129.9481	129.2354	130.0806	**116.16**
**C4**	181.8964	182.5361	183.1128	182.9647	183.4846	182.9173	184.1724	182.8968	183.9350	182.9925	184.2385	182.1104	182.5115	183.1648	182.7723	183.4027	182.7394	183.7928	182.7322	183.7403	182.7849	183.9455	**166.89**
**C5**	126.0329	127.3491	128.0096	128.0921	128.9347	128.0253	129.0515	127.9857	129.0367	128.1384	129.2952	126.1441	127.4212	128.0695	128.1002	128.9526	128.0385	129.0416	128.0038	128.9555	128.1414	129.1882	**116.16**
**C6**	144.4457	146.5586	147.7101	147.7244	149.3132	147.6325	150.0897	147.5676	149.3684	147.7936	150.0269	144.7647	146.8890	148.2216	147.9599	149.7654	147.8834	150.1624	147.8313	150.0717	148.0120	150.5715	**132.19**
**C7**	210.7961	216.2518	217.8303	219.2261	219.8730	219.0191	225.3038	218.8395	225.0653	219.4065	227.1999	215.4470	220.1457	221.5590	222.7455	223.3196	222.5572	228.8329	222.3988	227.4675	222.9103	229.4875	**196.22**
**C8**	61.3984	62.6319	63.4214	63.1097	64.1261	63.0645	64.5480	63.0462	64.4091	63.1347	65.2976	55.6431	57.3143	57.8214	58.2576	59.0058	58.1666	59.8648	58.1066	59.4794	58.3300	60.2244	**59.75**
**C9**	21.5377	21.6825	21.9377	21.8979	22.3180	21.8578	22.1872	21.8462	22.0012	21.9197	22.1735	18.6324	18.8728	18.9378	19.0093	19.2711	18.9777	19.3164	18.9740	19.2407	19.0228	19.6888	**15.44**
**C8`**	33.6021	34.3992	34.7145	34.8359	35.1835	34.7918	35.4065	34.7739	35.3393	34.8597	35.8933	29.0895	29.3897	29.7641	29.6827	29.9584	29.6509	30.0425	29.6443	30.1159	29.6934	30.2365	**31.11**
**H2**	8.9711	8.8976	8.9038	8.8550	8.8357	8.8575	8.9003	8.8613	8.8099	8.8515	8.8027	8.9912	8.8085	8.8563	8.7154	8.7346	8.7227	8.6856	8.7307	8.7297	8.7068	8.6896	**7.97**
**H3**	7.5427	7.6754	7.7638	7.7201	7.8369	7.7170	7.8517	7.7154	7.8451	7.7221	7.8687	7.5437	7.6708	7.7599	7.7138	7.8323	7.7108	7.8425	7.7093	7.8366	7.7157	7.8586	**7.22**
**H5**	7.4058	7.5954	7.6843	7.6812	7.8049	7.6755	7.8189	7.6712	7.8049	7.6857	7.8395	7.4060	7.5871	7.6767	7.6670	7.7928	7.6617	7.7982	7.6579	7.7897	7.6710	7.8216	**7.22**
**H6**	8.3027	8.5213	8.6081	8.6422	8.7669	8.6342	8.8393	8.6277	8.7770	8.6487	8.8307	8.1441	8.3320	8.4605	8.4236	8.6087	8.4188	8.6040	8.4150	8.6111	8.4268	8.6501	**7.97**
**H8**	4.0994	4.2241	4.2473	4.3375	4.3854	4.3282	4.4499	4.3208	4.4440	4.3453	4.4811	4.3161	4.4914	4.5661	4.5712	4.6984	4.5663	4.7098	4.5628	4.7083	4.5747	4.7424	**4.99**
**H(9A-9C)**	1.3477	1.3582	1.3449	1.3500	1.3227	1.3501	1.3530	1.3517	1.2974	1.3487	1.3187	1.4131	1.4278	1.4251	1.4407	1.4202	1.4395	1.4310	1.4398	1.4374	1.4406	1.4542	**1.51**
**H(8`A-8`C)**	2.5010	2.4391	2.4146	2.4167	2.4026	2.4164	2.3702	2.4179	2.3908	2.4162	2.3818	2.8887	2.7145	2.6860	2.6365	2.5978	2.6411	2.5031	2.6470	2.5362	2.6309	2.4788	**2.71**
Nuclei	**M06**																						
**C1**	135.4072	135.7741	137.0115	135.8816	138.0210	135.8649	136.8091	135.8563	137.3380	135.9123	137.0556	134.1674	135.5202	136.1558	136.2300	137.8135	136.1538	137.9154	136.0917	137.5984	136.3238	137.9805	**129.02**
**C2**	140.6153	140.3648	140.9748	140.1830	140.7472	140.1863	141.1872	140.1968	141.0177	140.1945	141.1811	139.8441	139.8784	140.4612	139.8960	140.5961	139.8814	141.2124	139.8755	141.0600	139.9255	141.2192	**132.19**
**C3**	121.6176	122.2938	122.9735	122.5729	123.4101	122.5477	123.6473	122.5304	123.5932	122.6111	123.7286	121.6738	122.2553	122.8671	122.5213	123.3123	122.4950	123.5158	122.4767	123.4472	122.5609	123.5784	**116.16**
**C4**	173.1165	173.9963	174.7342	174.4927	175.3210	174.4521	176.0927	174.4196	175.9087	174.5457	176.2448	173.8447	174.2720	175.0646	174.5038	175.3524	174.4860	175.6260	174.4744	175.6126	174.5338	175.7886	**166.89**
**C5**	119.7718	121.0559	121.7752	121.7509	122.7315	121.6989	122.7996	121.6545	122.7382	121.8148	122.9828	119.7041	120.9774	121.6509	121.6232	122.5692	121.5755	122.6711	121.5353	122.6020	121.6826	122.8037	**116.16**
**C6**	136.8085	138.7038	139.7562	139.8590	141.2826	139.7763	142.3109	139.7016	141.8293	139.9522	142.5930	137.1469	139.1678	140.5409	140.2006	141.9881	140.1354	142.4239	140.0763	142.2946	140.2746	142.8394	**132.19**
**C7**	201.8270	206.8005	208.5395	209.3151	210.6085	209.1531	215.3851	208.9988	214.6233	209.4836	216.2344	206.3715	210.6515	211.8254	212.8411	213.6353	212.6921	218.3336	212.5525	217.1976	212.9995	218.7520	**196.22**
**C8**	60.7304	61.8816	62.6495	62.3326	63.4023	62.2980	63.5929	62.2707	63.6589	62.3799	64.4145	54.1844	55.7499	56.3619	56.6790	57.5433	56.6009	58.4113	56.5332	58.0797	56.7709	58.7141	**59.75**
**C9**	22.1450	22.4238	22.6903	22.7400	23.1676	22.7095	23.3573	22.6872	23.2995	22.7835	23.4614	19.4572	19.6907	19.8485	19.8638	20.2293	19.8402	20.3336	19.8248	20.2582	19.9018	20.8431	**15.44**
**C8`**	36.9348	37.6867	37.9199	37.9636	38.3405	37.9372	38.4473	37.9185	38.4095	38.0033	38.9535	31.7679	31.9520	32.3660	32.1758	32.5544	32.1571	32.7194	32.1443	32.7693	32.2063	32.7972	**31.11**
**H2**	8.7387	8.6519	8.6561	8.6165	8.5956	8.6186	8.6674	8.6211	8.6218	8.6152	8.6525	8.7596	8.5499	8.5974	8.4553	8.4747	8.4624	8.4395	8.4698	8.4663	8.4487	8.4486	**7.97**
**H3**	7.3369	7.4633	7.5604	7.5046	7.6321	7.5024	7.6528	7.5006	7.6377	7.5073	7.6581	7.2874	7.4302	7.5150	7.4793	7.6060	7.4760	7.6356	7.4734	7.6197	7.4830	7.6504	**7.22**
**H5**	7.1579	7.3516	7.4440	7.4353	7.5710	7.4303	7.5755	7.4258	7.5657	7.4405	7.6026	7.1555	7.3358	7.4320	7.4048	7.5377	7.4011	7.5349	7.3979	7.5315	7.4086	7.5553	**7.22**
**H6**	8.2198	8.4571	8.5350	8.5706	8.6748	8.5635	8.7980	8.5570	8.7357	8.5779	8.8111	7.9934	8.1490	8.2703	8.2290	8.4027	8.2251	8.4051	8.2215	8.4052	8.2330	8.4572	**7.97**
**H8**	4.3442	4.4692	4.4989	4.5607	4.6243	4.5542	4.7164	4.5485	4.6836	4.5675	4.7345	4.4477	4.6028	4.6519	4.6647	4.7811	4.6617	4.7907	4.6590	4.7893	4.6679	4.8239	**4.99**
**H(9A-9C)**	1.3018	1.3045	1.2808	1.2978	1.2531	1.2981	1.2815	1.2991	1.2659	1.2981	1.2924	1.3858	1.4182	1.4256	1.4306	1.4133	1.4304	1.4165	1.4305	1.4256	1.4312	1.4437	**1.51**
**H(8`A-8`C)**	2.4763	2.4121	2.3914	2.3956	2.3829	2.3959	2.3669	2.3969	2.3791	2.3959	2.3540	2.9033	2.7432	2.7104	2.6626	2.6248	2.6682	2.5329	2.6741	2.5623	2.6576	2.5047	**2.71**
Nuclei	**M06-2X**																						
**C1**	153.0917	153.5294	154.5657	153.6798	155.3824	153.6910	154.3824	153.7038	154.6410	153.6636	154.5610	152.3025	153.6253	154.2248	154.4172	155.8548	154.3561	155.9192	154.3050	155.6636	154.4801	156.1304	**129.02**
**C2**	155.1746	155.0543	155.6859	154.8589	155.5556	154.8934	156.0475	154.9292	155.9461	154.8195	156.0984	154.7655	154.7734	155.3565	154.6837	155.4125	154.7088	155.8874	154.7355	155.7791	154.6544	155.9277	**132.19**
**C3**	136.1590	136.8604	137.4721	137.0980	137.8395	137.1065	138.0767	137.1160	138.0006	137.0834	138.1754	136.1934	136.8293	137.4400	137.0599	137.8148	137.0677	137.9965	137.0767	137.9426	137.0459	138.0999	**116.16**
**C4**	186.9866	187.8245	188.4710	188.2660	188.9823	188.2597	189.6225	188.2546	189.4454	188.2657	189.8145	187.4071	187.9865	188.7105	188.2373	189.0085	188.2488	189.3855	188.2602	189.3135	188.2178	189.5659	**166.89**
**C5**	133.5146	135.0251	135.7169	135.8167	136.7899	135.7892	136.9947	135.7628	136.8748	135.8366	137.2061	133.4360	134.8666	135.5636	135.5696	136.5427	135.5491	136.6670	135.5297	136.5791	135.5821	136.8705	**116.16**
**C6**	151.2911	153.5510	154.7995	154.7508	156.6302	154.6981	157.2971	154.6466	156.9660	154.7945	157.7502	151.6315	153.9149	155.3078	154.9928	157.0373	154.9595	157.4443	154.9245	157.2709	155.0146	157.8386	**132.19**
**C7**	218.4206	224.0871	225.6863	227.0228	227.7392	226.8634	233.4654	226.7049	232.2929	227.1679	234.4902	222.3731	227.5635	229.0579	230.3177	230.9202	230.1563	236.7866	230.0003	235.3937	230.4701	237.6501	**196.22**
**C8**	66.1021	67.3894	68.1145	67.8657	68.9271	67.8608	69.3536	67.8567	69.2455	67.8636	70.1061	60.3620	62.0622	62.4883	63.0156	63.8536	62.9637	64.7319	62.9168	64.3967	63.0637	65.0835	**59.75**
**C9**	23.2058	23.4985	23.7070	23.7340	24.1504	23.7389	24.1959	23.7459	24.1017	23.7236	24.3456	20.3942	20.6118	20.6433	20.7319	21.0349	20.7420	21.1263	20.7541	21.0549	20.7172	21.5210	**15.44**
**C8`**	36.0510	36.7555	36.9669	37.1150	37.4247	37.1125	37.6459	37.1106	37.5970	37.1109	38.1632	30.7152	31.1896	31.5368	31.3585	31.6695	31.3831	31.7058	31.4062	31.7955	31.3257	31.8659	**31.11**
**H2**	9.2412	9.1940	9.2146	9.1667	9.1653	9.1672	9.2317	9.1673	9.2138	9.1663	9.2327	9.2302	9.0921	9.1468	9.0068	9.0354	9.0138	9.0044	9.0198	9.0317	8.9993	9.0042	**7.97**
**H3**	7.8930	8.0315	8.1240	8.0797	8.2018	8.0760	8.2142	8.0720	8.2077	8.0833	8.2275	7.8444	7.9817	8.0724	8.0317	8.1517	8.0277	8.1674	8.0235	8.1599	8.0355	8.1819	**7.22**
**H5**	7.7188	7.9115	8.0040	8.0015	8.1305	7.9950	8.1471	7.9882	8.1342	8.0078	8.1715	7.7205	7.8974	7.9934	7.9760	8.1058	7.9705	8.1095	7.9646	8.1016	7.9813	8.1306	**7.22**
**H6**	8.7195	8.9331	9.0357	9.0522	9.2020	9.0436	9.2622	9.0346	9.2341	9.0605	9.2978	8.5491	8.7357	8.8647	8.8195	9.0069	8.8151	9.0001	8.8099	9.0002	8.8232	9.0400	**7.97**
**H8**	4.2292	4.3451	4.3884	4.4506	4.5168	4.4418	4.5788	4.4328	4.5575	4.4593	4.6093	4.4880	4.6312	4.7027	4.6959	4.8114	4.6915	4.8114	4.6865	4.8097	4.7000	4.8405	**4.99**
**H(9A-9C)**	1.3567	1.3681	1.3531	1.3672	1.3372	1.3663	1.3712	1.3650	1.3600	1.3682	1.3871	1.4261	1.4313	1.4276	1.4367	1.4173	1.4356	1.4268	1.4342	1.4284	1.4376	1.4441	**1.51**
**H(8`A-8`C)**	2.4619	2.4039	2.3762	2.3853	2.3641	2.3849	2.3357	2.3842	2.3446	2.3860	2.3207	2.7873	2.6416	2.6191	2.5775	2.5343	2.5805	2.4563	2.5830	2.4800	2.5745	2.4273	**2.71**

[a] methyl protons shifts are averaged

[b] according to [3]

Table S11. Cont. IV

Nuclei	R											S											Exp. [b]
	mpW1LYP																						
	Gas	$CHCl_3$ (PCM)	$CHCl_3$ (SMD)	DMSO (PCM)	DMSO (SMD)	CH_3OH (PCM)	CH_3OH (SMD)	C_2H_5OH (PCM)	C_2H_5OH (SMD)	H_2O (PCM)	H_2O (SMD)	Gas	$CHCl_3$ (PCM)	$CHCl_3$ (SMD)	DMSO (PCM)	DMSO (SMD)	CH_3OH (PCM)	CH_3OH (SMD)	C_2H_5OH (PCM)	C_2H_5OH (SMD)	H_2O (PCM)	H_2O (SMD)	
**C1**	139.4828	139.9525	140.8951	140.2162	142.1269	140.1880	141.3847	140.1639	141.5911	140.2438	141.6737	138.0239	139.3201	140.0314	140.0519	141.6015	140.0019	141.3526	139.9557	141.2617	140.1006	141.3699	**129.02**
**C2**	140.0654	139.9633	140.5214	139.8120	140.2594	139.8221	140.7067	139.8327	140.5833	139.8000	140.6754	139.3485	139.4849	140.0212	139.4970	140.0614	139.4929	140.6129	139.4906	140.4986	139.4995	140.6006	**132.19**
**C3**	121.9628	122.6121	123.1375	122.8712	123.4386	122.8537	123.7099	122.8374	123.6709	122.8862	123.7449	122.0772	122.6487	123.1165	122.8876	123.4426	122.8714	123.6473	122.8567	123.5970	122.9013	123.6861	**116.16**
**C4**	176.1473	177.0246	177.6646	177.5228	178.1467	177.4890	178.8123	177.4567	178.6743	177.5533	178.9071	176.5361	177.1020	177.7627	177.3919	178.0767	177.3729	178.4887	177.3548	178.3978	177.4074	178.6295	**166.89**
**C5**	119.7588	121.0521	121.6454	121.7767	122.5021	121.7290	122.7447	121.6831	122.6591	121.8202	122.8719	119.9121	121.1021	121.6630	121.7119	122.4355	121.6731	122.5793	121.6353	122.5049	121.7464	122.6956	**116.16**
**C6**	136.3411	138.3279	139.4122	139.4387	140.8254	139.3726	141.4341	139.3066	141.1391	139.4983	141.7648	136.6810	138.7304	140.0172	139.7711	141.3801	139.7081	141.9011	139.6456	141.7184	139.8277	142.2839	**132.19**
**C7**	202.6211	207.7782	209.3145	210.5766	211.6308	210.3901	217.0461	210.2089	216.1478	210.7558	217.9713	207.9684	212.1537	213.3072	214.4009	215.1623	214.2635	219.8843	214.1282	218.8057	214.5296	220.3958	**196.22**
**C8**	64.8087	65.9413	66.6307	66.2710	67.2010	66.2555	67.4970	66.2398	67.3414	66.2823	68.2675	59.1606	61.1502	61.8125	62.1638	63.0102	62.0972	63.6763	62.0334	63.4471	62.2269	63.9055	**59.75**
**C9**	23.0194	23.2782	23.4066	23.5626	23.7640	23.5450	23.7639	23.5280	23.6835	23.5763	23.8872	19.5716	19.9981	20.1757	20.2377	20.4306	20.2213	20.5847	20.2064	20.5225	20.2514	20.9210	**15.44**
**C8`**	36.3159	37.0893	37.3474	37.4110	37.7421	37.3893	37.9553	37.3691	37.9323	37.4299	38.3577	31.9503	32.0958	32.3503	32.2714	32.4808	32.2555	32.5948	32.2408	32.6120	32.2842	32.6749	**31.11**
**H2**	8.6760	8.6134	8.6345	8.5731	8.5352	8.5769	8.5767	8.5801	8.5650	8.5692	8.5537	8.7155	8.5439	8.5843	8.4575	8.4611	8.4631	8.4509	8.4681	8.4656	8.4518	8.4608	**7.97**
**H3**	7.3397	7.4720	7.5610	7.5164	7.6269	7.5145	7.6429	7.5123	7.6350	7.5181	7.6539	7.3222	7.4540	7.5397	7.5009	7.6151	7.4987	7.6299	7.4961	7.6226	7.5029	7.6422	**7.22**
**H5**	7.1905	7.3768	7.4660	7.4635	7.5825	7.4586	7.6016	7.4535	7.5914	7.4679	7.6207	7.1791	7.3532	7.4411	7.4319	7.5518	7.4276	7.5619	7.4231	7.5524	7.4357	7.5804	**7.22**
**H6**	8.0834	8.2895	8.3770	8.4032	8.5186	8.3966	8.5837	8.3898	8.5634	8.4092	8.6049	7.8914	8.0614	8.1768	8.1478	8.3090	8.1432	8.3269	8.1382	8.3161	8.1519	8.3729	**7.97**
**H8**	4.3063	4.3981	4.4252	4.5055	4.5605	4.4978	4.6229	4.4902	4.6105	4.5128	4.6327	4.4564	4.6192	4.6720	4.6856	4.7973	4.6823	4.8170	4.6786	4.8046	4.6884	4.8463	**4.99**
**H(9A-9C)**	1.3216	1.3308	1.3202	1.3215	1.2774	1.3232	1.2903	1.3245	1.2801	1.3196	1.3009	1.4008	1.4361	1.4422	1.4560	1.4325	1.4551	1.4529	1.4538	1.4517	1.4567	1.4756	**1.51**
**H(8`A-8`C)**	2.5203	2.4606	2.4330	2.4451	2.4238	2.4459	2.4070	2.4465	2.4101	2.4443	2.3934	2.9829	2.8104	2.7656	2.7300	2.6802	2.7354	2.5986	2.7403	2.6192	2.7247	2.5674	**2.71**
Nuclei	mpW1PW91																						
**C1**	136.7091	137.1261	138.2904	137.3527	139.3925	137.3342	138.8484	137.3222	138.8794	137.3708	139.0868	135.4965	136.8443	137.3776	137.4319	138.8932	137.3747	138.9047	137.3297	138.6184	137.4927	138.9966	**129.02**
**C2**	139.0510	138.9813	139.5567	138.8023	139.3841	138.8154	139.7972	138.8338	139.6690	138.7889	139.7849	138.4920	138.6376	139.1844	138.5926	139.2587	138.5931	139.7425	138.5998	139.6330	138.5920	139.7648	**132.19**
**C3**	121.1434	121.8839	122.4572	122.1398	122.8577	122.1259	123.1111	122.1175	123.0669	122.1523	123.1972	121.1969	121.8835	122.4042	122.1282	122.8348	122.1142	123.0503	122.1059	122.9791	122.1411	123.1290	**116.16**
**C4**	173.1450	174.0361	174.6361	174.4798	175.1382	174.4530	175.7414	174.4320	175.6481	174.5044	175.9056	173.4356	174.0253	174.6981	174.2966	175.0454	174.2852	175.4189	174.2781	175.3524	174.3057	175.5825	**166.89**
**C5**	118.9666	120.3546	120.9855	121.0745	121.9413	121.0305	122.1805	120.9922	122.0946	121.1155	122.3576	119.0635	120.3480	120.9568	120.9724	121.8462	120.9359	121.9978	120.9051	121.9093	121.0057	122.1526	**116.16**
**C6**	135.1188	137.1393	138.1020	138.2106	139.6150	138.1465	140.1616	138.0877	139.9062	138.2707	140.5379	135.4925	137.5198	138.7879	138.5089	140.2202	138.4556	140.6265	138.4058	140.5120	138.5558	141.0214	**132.19**
**C7**	200.5499	205.4732	207.1087	208.0696	209.2352	207.9079	214.4321	207.7523	213.5361	208.2238	215.3863	204.9687	209.2878	210.4288	211.5065	212.2688	211.3693	217.0803	211.2400	215.8598	211.6406	217.5855	**196.22**
**C8**	62.5871	63.7277	64.3921	64.0779	65.0594	64.0579	65.5692	64.0437	65.2428	64.0959	66.2934	57.3088	59.1083	59.5712	59.9715	60.7733	59.9036	61.5602	59.8450	61.2368	60.0391	61.8431	**59.75**
**C9**	22.4303	22.7074	22.8165	22.9714	23.2346	22.9561	23.2515	22.9463	23.1372	22.9849	23.3928	19.1269	19.5971	19.6794	19.7761	20.0297	19.7623	20.1776	19.7543	20.1086	19.7888	20.5773	**15.44**
**C8`**	35.5389	36.3291	36.5286	36.6000	36.9580	36.5839	37.1500	36.5735	37.1332	36.6149	37.6110	31.0253	31.2540	31.5589	31.4502	31.7343	31.4412	31.7914	31.4363	31.8597	31.4556	31.8914	**31.11**
**H2**	8.7270	8.6729	8.6666	8.6268	8.5829	8.6306	8.6029	8.6343	8.6110	8.6233	8.5930	8.7399	8.5718	8.6280	8.4933	8.5106	8.5006	8.4771	8.5073	8.5099	8.4860	8.4856	**7.97**
**H3**	7.3666	7.5071	7.5941	7.5506	7.6672	7.5488	7.6842	7.5469	7.6761	7.5526	7.6994	7.3521	7.4918	7.5782	7.5379	7.6587	7.5357	7.6748	7.5336	7.6662	7.5401	7.6902	**7.22**
**H5**	7.2156	7.4099	7.4980	7.4952	7.6199	7.4906	7.6379	7.4860	7.6293	7.4999	7.6618	7.2035	7.3869	7.4755	7.4658	7.5930	7.4616	7.6047	7.4575	7.5936	7.4700	7.6265	**7.22**
**H6**	8.1720	8.3810	8.4466	8.4858	8.5927	8.4800	8.6356	8.4742	8.6335	8.4916	8.6714	7.9622	8.1369	8.2584	8.2244	8.3946	8.2208	8.3985	8.2168	8.3988	8.2277	8.4460	**7.97**
**H8**	4.3244	4.4232	4.4417	4.5212	4.5686	4.5149	4.6073	4.5088	4.6209	4.5275	4.6357	4.4958	4.6417	4.6998	4.7058	4.8233	4.7033	4.8370	4.7007	4.8311	4.7081	4.8693	**4.99**
**H(9A-9C)**	1.3031	1.3123	1.2861	1.3005	1.2512	1.3018	1.2673	1.3032	1.2532	1.2994	1.2798	1.3839	1.4107	1.4119	1.4245	1.4007	1.4244	1.4147	1.4241	1.4196	1.4248	1.4408	**1.51**
**H(8`A-8`C)**	2.4582	2.3926	2.3693	2.3752	2.3559	2.3760	2.3459	2.3770	2.3405	2.3747	2.3305	2.8765	2.7109	2.6756	2.6345	2.5913	2.6410	2.5022	2.6472	2.5276	2.6285	2.4719	**2.71**
Nuclei	O3LYP																						
**C1**	133.9706	135.2869	135.1280	135.8992	137.5413	135.8935	136.8634	135.8840	136.9936	135.9000	136.9568	131.9841	134.0269	134.9667	134.8776	136.4625	134.8098	136.5165	134.7451	136.4801	134.9454	136.6774	**129.02**
**C2**	134.2294	134.0386	134.6059	134.0020	134.6215	134.0143	135.0928	134.0931	135.0297	133.9844	135.0347	133.6011	133.8315	134.4861	133.8598	134.5658	133.8686	134.9908	133.8725	134.9155	133.8449	134.9967	**132.19**
**C3**	117.1429	117.8472	118.4244	118.1586	118.8498	118.1512	119.1048	118.1420	119.0383	118.1592	119.1705	117.2423	117.8329	118.4008	118.1462	118.7940	118.1374	118.9776	118.1236	118.9199	118.1480	119.0503	**116.16**
**C4**	169.3331	170.2178	170.9930	170.6800	171.4504	170.6575	171.9898	170.6246	171.8402	170.6953	172.1262	169.6935	170.1374	170.8394	170.4612	171.2113	170.4568	171.5048	170.4458	171.4066	170.4567	171.6400	**166.89**
**C5**	115.1244	116.3261	117.0316	116.9791	117.7550	116.9488	117.9702	116.9062	117.8594	117.0010	118.1340	115.2829	116.3481	116.9834	116.9736	117.7506	116.9474	117.8633	116.9154	117.7787	116.9914	118.0028	**116.16**
**C6**	130.1343	131.8403	133.2468	132.9143	134.4244	132.8471	134.9826	132.7768	134.7083	132.9717	135.3818	130.8195	132.4740	133.5676	133.4119	134.9398	133.3757	135.3121	133.3309	135.1128	133.4364	135.6140	**132.19**
**C7**	196.4324	202.4039	202.9208	205.2799	206.3184	205.1284	211.0569	204.9884	210.1352	205.4208	211.6531	200.9322	205.3707	206.9113	207.7346	208.6887	207.5898	212.9882	207.4429	212.0350	207.8698	213.5094	**196.22**
**C8**	62.7269	63.8638	64.1933	64.6392	65.2111	64.6247	65.7186	64.6144	65.5898	64.6448	66.4308	57.3727	59.9629	60.5156	61.0054	61.8178	60.9369	62.7983	60.8688	62.5812	61.0698	63.0624	**59.75**
**C9**	21.9077	22.1497	22.4381	22.4448	22.6922	22.4333	22.8050	22.4257	22.7348	22.4497	22.9545	18.3907	18.7982	18.9135	19.0083	19.2082	19.0039	19.4486	18.9953	19.3637	19.0069	19.7532	**15.44**
**C8`**	35.8615	36.6415	36.9984	37.0897	37.4384	37.0783	37.5559	37.0769	37.5415	37.0931	37.8889	31.6791	31.7885	32.1081	32.0205	32.3175	32.0201	32.2296	32.0132	32.2550	32.0126	32.2365	**31.11**
**H2**	8.5171	8.3815	8.5110	8.3059	8.3131	8.3086	8.3569	8.3167	8.3480	8.3030	8.3618	8.6115	8.3742	8.4056	8.2874	8.3033	8.2956	8.2770	8.3028	8.2844	8.2784	8.2790	**7.97**
**H3**	7.2896	7.4098	7.5061	7.4543	7.5768	7.4521	7.6030	7.4468	7.5953	7.4562	7.6133	7.2766	7.4071	7.4943	7.4552	7.5707	7.4530	7.5845	7.4505	7.5766	7.4572	7.5999	**7.22**
**H5**	7.1552	7.3290	7.4298	7.3986	7.5094	7.3950	7.5202	7.3889	7.5085	7.4017	7.5463	7.1395	7.3009	7.3898	7.3774	7.4980	7.3735	7.5074	7.3692	7.4968	7.3810	7.5278	**7.22**
**H6**	8.0527	8.2175	8.3672	8.3017	8.4640	8.2946	8.5029	8.2853	8.4809	8.3083	8.5390	7.8574	7.9837	8.0812	8.0642	8.2140	8.0612	8.2127	8.0575	8.2001	8.0664	8.2508	**7.97**
**H8**	4.4166	4.5098	4.5410	4.5642	4.6714	4.5583	4.6852	4.5532	4.6658	4.5699	4.7170	4.5458	4.6793	4.7411	4.7470	4.8642	4.7444	4.8665	4.7410	4.8556	4.7490	4.8955	**4.99**
**H(9A-9C)**	1.2321	1.2056	1.2312	1.2013	1.1647	1.2018	1.1980	1.2027	1.1895	1.2008	1.2166	1.3321	1.3605	1.3485	1.3797	1.3521	1.3795	1.3771	1.3787	1.3725	1.3796	1.3957	**1.51**
**H(8`A-8`C)**	2.4772	2.4452	2.4019	2.4401	2.4019	2.4414	2.3990	2.4451	2.4021	2.4388	2.3752	2.9410	2.7694	2.7347	2.6983	2.6563	2.7041	2.5681	2.7092	2.5820	2.6922	2.5313	**2.71**
Nuclei	OLYP																						
**C1**	132.8188	134.1516	135.6656	135.1449	137.5235	135.1515	135.6652	135.1532	135.8434	135.1337	135.7556	130.8981	132.7261	133.7888	133.6768	135.1782	133.6081	135.1883	133.5382	135.1931	133.7379	135.3980	**129.02**
**C2**	131.6623	131.6975	132.2794	131.7465	132.3472	131.7531	132.6913	131.7546	132.6220	131.7302	132.7039	131.2536	131.3661	132.0518	131.4162	132.0990	131.4235	132.5894	131.4250	132.5059	131.4005	132.5752	**132.19**
**C3**	115.3417	116.0422	116.6555	116.3161	116.9608	116.3056	117.2576	116.2899	117.1915	116.3169	117.3369	114.4114	116.0541	116.6230	116.3750	116.9979	116.3650	117.1604	116.3492	117.1047	116.3752	117.2329	**116.16**
**C4**	168.2126	169.2294	169.9205	169.5710	170.1814	169.5487	170.9191	169.5215	170.7744	169.5840	171.0771	168.4577	169.1151	169.8267	169.4427	170.2413	169.4360	170.4899	169.4224	170.3927	169.4386	170.6134	**166.89**
**C5**	113.3427	114.4633	115.0535	115.0655	115.7600	115.0339	116.1215	114.9960	116.0017	115.0864	116.2699	113.4100	114.5734	115.1849	115.1704	115.9016	115.1450	116.0297	115.1131	115.9473	115.1848	116.1602	**116.16**
**C6**	127.6886	129.4580	130.3992	130.3959	131.5991	130.3259	132.5021	130.2522	132.2325	130.4552	132.8719	132.4882	129.9940	131.1063	130.8821	132.4316	130.8446	132.8458	130.8002	132.6433	130.9066	133.1053	**132.19**
**C7**	195.3251	201.0301	202.9899	204.5243	206.1991	204.3850	209.0998	204.2443	208.2692	204.6535	209.6663	194.9333	204.0205	205.4470	206.3531	207.3591	206.2114	211.0678	206.0639	210.2799	206.4776	211.6073	**196.22**
**C8**	63.8385	65.2339	66.0677	65.6811	66.8369	65.6883	66.9203	65.6854	66.8721	65.6665	67.5606	62.8816	61.2250	62.0414	62.3546	63.1411	62.2822	64.2114	62.2081	64.0080	62.4201	64.4616	**59.75**
**C9**	22.0417	22.3211	22.5251	22.7061	22.8886	22.6962	23.1868	22.6811	23.0909	22.7070	23.3016	15.7972	18.8615	19.1193	19.1190	19.3070	19.1099	19.6432	19.0962	19.5371	19.1202	19.9486	**15.44**
**C8`**	36.5297	37.3375	37.5614	37.8176	38.0745	37.8036	38.1673	37.7827	38.1190	37.8211	38.5013	31.4966	32.4077	32.6511	32.6378	32.9272	32.6373	32.7834	32.6299	32.8079	32.6276	32.7561	**31.11**
**H2**	8.3522	8.2235	8.1969	8.1272	8.0714	8.1268	8.2234	8.1264	8.2066	8.1267	8.2299	8.4369	8.2352	8.2566	8.1468	8.1702	8.1543	8.1611	8.1613	8.1618	8.1388	8.1574	**7.97**
**H3**	7.2072	7.3275	7.4178	7.4008	7.5127	7.3974	7.5330	7.3939	7.5211	7.4035	7.5445	7.1593	7.3325	7.4196	7.3792	7.4934	7.3770	7.5033	7.3746	7.4956	7.3809	7.5175	**7.22**
**H5**	7.0851	7.2353	7.3143	7.2988	7.4157	7.2941	7.4375	7.2893	7.4251	7.3029	7.4576	7.0403	7.2266	7.3145	7.2993	7.4188	7.2955	7.4288	7.2915	7.4188	7.3025	7.4474	**7.22**
**H6**	7.9775	8.1327	8.1956	8.2132	8.2977	8.2041	8.4009	8.1954	8.3698	8.2217	8.4374	8.7484	7.8865	7.9743	7.9576	8.1089	7.9547	8.1075	7.9514	8.0939	7.9595	8.1417	**7.97**
**H8**	4.5323	4.5911	4.6096	4.6267	4.6824	4.6200	4.7353	4.6144	4.7106	4.6332	4.7707	4.4883	4.7554	4.8166	4.8221	4.9436	4.8190	4.9419	4.8155	4.9305	4.8243	4.9712	**4.99**
**H(9A-9C)**	1.2237	1.2082	1.1959	1.1823	1.1420	1.1826	1.2055	1.1826	1.1992	1.1815	1.2218	1.2466	1.3653	1.3601	1.3854	1.3618	1.3848	1.3978	1.3840	1.3909	1.3856	1.4134	**1.51**
**H(8`A-8`C)**	2.5179	2.4873	2.4697	2.4886	2.4649	2.4895	2.4510	2.4902	2.4526	2.4873	2.4211	2.3523	2.8150	2.7687	2.7435	2.7024	2.7489	2.6101	2.7538	2.6211	2.7376	2.5695	**2.71**

[a] methyl protons shifts are averaged

[b] according to [3]

Table S11. Cont. V

Nuclei	R											S											**Exp.** [b]
	**OPBE**																						
	Gas	$CHCl_3$ (PCM)	$CHCl_3$ (SMD)	DMSO (PCM)	DMSO (SMD)	CH_3OH (PCM)	CH_3OH (SMD)	C_2H_5OH (PCM)	C_2H_5OH (SMD)	H_2O (PCM)	H_2O (SMD)	Gas	$CHCl_3$ (PCM)	$CHCl_3$ (SMD)	DMSO (PCM)	DMSO (SMD)	CH_3OH (PCM)	CH_3OH (SMD)	C_2H_5OH (PCM)	C_2H_5OH (SMD)	H_2O (PCM)	H_2O (SMD)	
**C1**	129.7640	131.1387	132.6552	132.1246	134.4950	132.1378	132.7494	132.1341	132.8307	132.0968	132.7529	127.9531	129.7572	131.3960	130.9073	132.9633	130.8138	132.2249	130.6965	132.6314	130.9636	132.5011	**129.02**
**C2**	130.3586	130.4886	130.9968	130.4429	131.0543	130.4635	131.3872	130.4661	131.3319	130.3820	131.3838	129.9865	130.2359	130.9157	130.1823	130.9380	130.1990	131.3075	130.1994	131.2030	130.1628	131.3207	**132.19**
**C3**	114.2213	115.0550	115.6259	115.3016	116.0144	115.3021	116.3293	115.2846	116.2561	115.3063	116.4542	114.3041	115.0698	115.5900	115.3365	116.0072	115.3394	116.2309	115.3240	116.1537	115.3295	116.3409	**116.16**
**C4**	164.6226	165.6789	166.2199	165.9412	166.5402	165.9340	167.2498	165.9090	167.1104	166.0101	167.4931	164.9027	165.5555	166.0292	165.7368	166.3569	165.7530	166.8192	165.7519	166.6262	165.7218	166.9648	**166.89**
**C5**	112.2687	113.5344	114.0760	114.0744	114.8381	114.0527	115.1922	114.0131	115.0739	114.1130	115.4008	112.4202	113.6143	114.1128	114.1369	114.8497	114.1271	115.0869	114.0994	114.9493	114.1433	115.2524	**116.16**
**C6**	126.1608	127.9997	128.8093	128.8180	129.9411	128.7696	130.8401	128.7038	130.6005	128.8489	131.2575	126.8203	128.5704	129.4079	129.2674	130.6027	129.2570	131.2141	129.2292	130.8690	129.2803	131.4889	**132.19**
**C7**	192.8626	198.3520	200.1588	201.6561	203.2107	201.5462	206.1754	201.4188	205.3233	201.7358	206.5747	196.3497	200.6868	202.1872	203.0429	204.1492	202.9127	207.7285	202.7568	207.1557	203.1474	208.2542	**196.22**
**C8**	61.2284	62.7133	63.5636	63.1881	64.3519	63.1850	64.4468	63.1637	64.3074	63.1720	65.1108	56.6311	58.7512	59.9248	59.9517	61.0884	59.8539	61.5973	59.7391	61.6414	60.0230	61.9029	**59.75**
**C9**	21.2805	21.6678	21.7922	21.9610	22.1440	21.9624	22.3933	21.9464	22.3075	21.9463	22.5303	17.8291	18.2838	18.5005	18.5007	18.7647	18.4933	18.9349	18.4692	18.8763	18.4996	19.2950	**15.44**
**C8`**	35.4845	36.3569	36.4549	36.7323	36.9249	36.7278	37.0000	36.7052	36.9687	36.7163	37.3820	31.0988	31.3654	31.3318	31.4174	31.5251	31.4408	31.6115	31.4469	31.5375	31.3965	31.5941	**31.11**
**H2**	8.3870	8.2642	8.2215	8.1564	8.0952	8.1575	8.2408	8.1577	8.2328	8.1562	8.2594	8.4575	8.2769	8.2227	8.1564	8.1258	8.1678	8.1855	8.1792	8.1490	8.1477	8.1810	**7.97**
**H3**	7.2282	7.3602	7.4433	7.4277	7.5438	7.4252	7.5609	7.4218	7.5508	7.4327	7.5779	7.2304	7.3678	7.4497	7.4149	7.5264	7.4130	7.5438	7.4104	7.5341	7.4158	7.5608	**7.22**
**H5**	7.1021	7.2653	7.3426	7.3260	7.4471	7.3219	7.4668	7.3170	7.4549	7.3330	7.4935	7.0869	7.2574	7.3371	7.3302	7.4448	7.3268	7.4602	7.3227	7.4473	7.3327	7.4817	**7.22**
**H6**	8.0380	8.1929	8.2297	8.2560	8.3210	8.2498	8.4322	8.2430	8.4104	8.2608	8.4821	7.8021	7.9511	7.9981	8.0067	8.1209	8.0068	8.1616	8.0062	8.1245	8.0072	8.1968	**7.97**
**H8**	4.5419	4.6115	4.6071	4.6376	4.6660	4.6345	4.7386	4.6308	4.7174	4.6371	4.7816	4.6657	4.7892	4.8168	4.8395	4.9337	4.8401	4.9559	4.8394	4.9298	4.8389	4.9886	**4.99**
**H(9A-9C)**	1.1827	1.1710	1.1506	1.1424	1.0922	1.1427	1.1573	1.1423	1.1499	1.1403	1.1786	1.2938	1.3294	1.3061	1.3393	1.2986	1.3395	1.3428	1.3392	1.3289	1.3389	1.3621	**1.51**
**H(8`A-8`C)**	2.4371	2.4087	2.3861	2.4045	2.3742	2.4058	2.3612	2.4063	2.3625	2.4000	2.3311	2.8529	2.7064	2.6163	2.6177	2.5447	2.6266	2.4933	2.6347	2.4903	2.6099	2.4549	**2.71**
Nuclei	**OPW91**																						
**C1**	130.0734	131.4711	132.9769	132.4406	134.8087	132.4451	133.0129	132.4321	133.1402	132.4191	133.0519	128.2394	130.0724	131.7005	131.1724	133.2463	131.0761	132.5216	130.9590	132.9078	131.2337	132.7953	**129.02**
**C2**	130.5806	130.7074	131.2089	130.6288	131.2637	130.6390	131.5719	130.6356	131.5478	130.6007	131.6048	130.1962	130.4533	131.1259	130.4042	131.1441	130.4116	131.5201	130.4034	131.4168	130.3802	131.5359	**132.19**
**C3**	114.4183	115.2508	115.8151	115.5092	116.1968	115.4994	116.5211	115.4732	116.4393	115.4999	116.6391	114.5029	115.2650	115.7792	115.5361	116.1909	115.5285	116.4136	115.5035	116.3378	115.5247	116.5251	**116.16**
**C4**	165.0246	166.0821	166.6217	166.4195	166.9443	166.4003	167.7080	166.3664	167.5188	166.4192	167.9027	165.3169	165.9654	166.4385	166.1639	166.7698	166.1681	167.2288	166.1553	167.0408	166.1434	167.3773	**166.89**
**C5**	112.4634	113.7286	114.2665	114.2944	115.0217	114.2618	115.3914	114.2128	115.2585	114.3067	115.5871	112.6210	113.8117	114.3056	114.3432	115.0398	114.3225	115.2744	114.2843	115.1397	114.3450	115.4419	**116.16**
**C6**	126.3826	128.2204	129.0301	129.0438	130.1652	128.9844	131.0735	128.9103	130.8310	129.0819	131.4938	127.0499	128.7969	129.6351	129.5203	130.8358	129.4965	131.4451	129.4563	131.1082	129.5270	131.7238	**132.19**
**C7**	193.2195	198.7503	200.5473	202.0469	203.6032	201.9273	206.5400	201.7965	205.7249	202.1429	206.9853	196.7445	201.1005	202.5918	203.4416	204.5479	203.3022	208.1416	203.1393	207.5501	203.5465	208.6706	**196.22**
**C8**	61.4164	62.9130	63.7519	63.3730	64.5345	63.3605	64.5943	63.3262	64.4929	63.3665	65.2823	56.7828	58.9381	60.1067	60.1087	61.2534	60.0077	61.7812	59.8918	61.8124	60.1818	62.0859	**59.75**
**C9**	21.3412	21.7318	21.8564	22.0225	22.2056	22.0139	22.4453	21.9905	22.3695	22.0127	22.5934	17.9020	18.3623	18.5810	18.5780	18.8386	18.5624	19.0151	18.5310	18.9558	18.5743	19.3737	**15.44**
**C8`**	35.5474	36.4260	36.5197	36.7969	36.9960	36.7816	37.0617	36.7522	37.0400	36.7928	37.4582	31.1915	31.4588	31.4241	31.5288	31.6223	31.5399	31.7046	31.5341	31.6351	31.5020	31.6928	**31.11**
**H2**	8.3887	8.2639	8.2222	8.1590	8.0961	8.1592	8.2467	8.1596	8.2338	8.1570	8.2615	8.4616	8.2780	8.2245	8.1636	8.1292	8.1736	8.1874	8.1840	8.1527	8.1537	8.1831	**7.97**
**H3**	7.2300	7.3612	7.4442	7.4326	7.5443	7.4293	7.5651	7.4261	7.5509	7.4339	7.5785	7.2318	7.3686	7.4505	7.4163	7.5267	7.4137	7.5442	7.4109	7.5345	7.4169	7.5610	**7.22**
**H5**	7.1042	7.2672	7.3446	7.3319	7.4481	7.3269	7.4705	7.3219	7.4560	7.3348	7.4945	7.0894	7.2592	7.3388	7.3327	7.4461	7.3286	7.4614	7.3242	7.4485	7.3348	7.4827	**7.22**
**H6**	8.0365	8.1901	8.2278	8.2553	8.3202	8.2483	8.4351	8.2419	8.4096	8.2601	8.4824	7.8035	7.9505	7.9980	8.0099	8.1218	8.0088	8.1616	8.0074	8.1258	8.0096	8.1969	**7.97**
**H8**	4.5413	4.6099	4.6067	4.6350	4.6660	4.6313	4.7388	4.6276	4.7179	4.6370	4.7820	4.6622	4.7853	4.8138	4.8390	4.9316	4.8382	4.9531	4.8368	4.9279	4.8379	4.9856	**4.99**
**H(9A-9C)**	1.1844	1.1724	1.1522	1.1437	1.0939	1.1434	1.1586	1.1424	1.1520	1.1425	1.1806	1.2943	1.3301	1.3074	1.3418	1.3002	1.3413	1.3445	1.3406	1.3308	1.3411	1.3636	**1.51**
**H(8`A-8`C)**	2.4383	2.4106	2.3882	2.4045	2.3762	2.4051	2.3601	2.4055	2.3644	2.4024	2.3329	2.8575	2.7104	2.6209	2.6250	2.5505	2.6328	2.4979	2.6400	2.4956	2.6165	2.4597	**2.71**
Nuclei	**PBE**																						
**C1**	135.5369	136.0918	137.1653	136.3677	138.3715	136.3432	137.9723	136.3302	138.0000	136.4097	138.2666	133.9549	135.5103	136.0530	136.0986	137.6401	136.0426	137.7558	136.0033	137.5912	136.1760	137.8979	**129.02**
**C2**	135.9657	136.0179	136.6216	135.7847	136.4967	135.7983	136.9066	135.8222	136.8094	135.7890	136.9073	135.2241	135.5670	136.1193	135.5057	136.3042	135.5028	136.8654	135.5121	136.7580	135.5269	136.8854	**132.19**
**C3**	119.4465	120.3299	120.8882	120.5608	121.3376	120.5455	121.6570	120.5407	121.5966	120.5927	121.7624	119.5345	120.3283	120.8574	120.5295	121.3174	120.5157	121.5667	120.5123	121.4938	120.5600	121.6702	**116.16**
**C4**	173.9823	175.0866	175.7504	175.5136	176.3815	175.4853	176.9469	175.4679	176.8322	175.5580	177.1272	174.3115	175.1027	175.8451	175.3569	176.3062	175.3425	176.6457	175.3376	176.5574	175.3868	176.8245	**166.89**
**C5**	117.3377	118.8348	119.4453	119.5117	120.4263	119.4676	120.7320	119.4345	120.6335	119.5712	120.9231	117.5420	118.8521	119.4821	119.4030	120.3643	119.3686	120.5554	119.3443	120.4643	119.4527	120.7344	**116.16**
**C6**	132.4775	134.5082	135.5902	135.4775	137.1352	135.4145	137.6377	135.3620	137.3771	135.5552	138.0465	132.8577	134.9572	136.2454	135.8915	137.7657	135.8360	138.2041	135.7900	138.0185	135.9600	138.5976	**132.19**
**C7**	200.2846	204.9361	206.5139	207.3187	208.7358	207.1695	213.0970	207.0319	212.3370	207.4784	214.0538	205.2335	209.1883	210.4404	211.1454	212.2526	211.0237	216.2129	210.9132	215.2932	211.2804	216.6930	**196.22**
**C8**	66.5088	67.7352	68.5365	68.0186	69.2245	67.9992	69.7030	67.9905	69.4643	68.0544	70.5121	60.8379	62.8084	63.3380	63.6487	64.6563	63.5816	65.3895	63.5289	65.1438	63.7343	65.6928	**59.75**
**C9**	23.3476	23.7914	23.9606	24.0315	24.4395	24.0128	24.5728	24.0048	24.4653	24.0668	24.7581	19.9739	20.6830	20.7908	20.8644	21.2357	20.8478	21.5194	20.8429	21.4105	20.8983	22.0261	**15.44**
**C8`**	37.1172	37.9328	38.1214	38.1135	38.5395	38.1010	38.7049	38.0990	38.6815	38.1431	39.1975	32.1770	32.4188	32.7113	32.5246	32.8859	32.5166	32.9628	32.5184	32.9920	32.5484	33.0440	**31.11**
**H2**	8.4940	8.4541	8.4552	8.4051	8.3713	8.4089	8.3990	8.4131	8.4018	8.4020	8.3885	8.5295	8.3765	8.4273	8.2992	8.3159	8.3056	8.2934	8.3120	8.3107	8.2932	8.2994	**7.97**
**H3**	7.2033	7.3447	7.4313	7.3857	7.5032	7.3838	7.5208	7.3823	7.5132	7.3881	7.5355	7.1915	7.3278	7.4143	7.3701	7.4921	7.3680	7.5036	7.3664	7.4955	7.3727	7.5186	**7.22**
**H5**	7.0554	7.2476	7.3344	7.3285	7.4528	7.3239	7.4719	7.3198	7.4630	7.3335	7.4950	7.0514	7.2254	7.3152	7.2983	7.4275	7.2942	7.4386	7.2906	7.4284	7.3027	7.4597	**7.22**
**H6**	8.0650	8.2613	8.3213	8.3484	8.4499	8.3435	8.4938	8.3391	8.4841	8.3536	8.5258	7.8075	7.9693	8.0875	8.0493	8.2174	8.0456	8.2265	8.0420	8.2172	8.0532	8.2729	**7.97**
**H8**	4.5942	4.6682	4.6640	4.7434	4.7783	4.7384	4.8154	4.7339	4.8140	4.7488	4.8411	4.7365	4.8721	4.9225	4.9285	5.0427	4.9260	5.0540	4.9238	5.0416	4.9312	5.0891	**4.99**
**H(9A-9C)**	1.3652	1.3737	1.3620	1.3582	1.3268	1.3594	1.3445	1.3610	1.3344	1.3577	1.3585	1.4620	1.4973	1.5000	1.5101	1.4967	1.5097	1.5158	1.5096	1.5145	1.5110	1.5442	**1.51**
**H(8`A-8`C)**	2.5658	2.4898	2.4677	2.4656	2.4518	2.4665	2.4330	2.4680	2.4322	2.4654	2.4135	3.0023	2.8324	2.7952	2.7516	2.7069	2.7580	2.6137	2.7646	2.6329	2.7457	2.5792	**2.71**
Nuclei	**PBE0**																						
**C1**	136.2247	136.6526	137.6494	136.8626	138.8179	136.8401	138.3389	136.8206	138.4202	136.8878	138.5165	135.0705	136.3670	136.9426	136.9688	138.3856	136.9063	138.4604	136.8512	138.1253	137.0380	138.5078	**129.02**
**C2**	138.7455	138.6972	139.2899	138.5224	139.1126	138.5294	139.6279	138.5381	139.4524	138.5187	139.5604	138.2240	138.3806	138.9274	138.3295	138.9750	138.3256	139.5396	138.3238	139.3720	138.3367	139.5045	**132.19**
**C3**	120.8925	121.6540	122.2093	121.9097	122.6105	121.8907	122.9447	121.8736	122.8377	121.9304	122.9696	120.9444	121.6525	122.1782	121.8956	122.5853	121.8764	122.8788	121.8592	122.7522	121.9168	122.9010	**116.16**
**C4**	172.7695	173.6677	174.2592	174.1060	174.7504	174.0748	175.4368	174.0455	175.2494	174.1384	175.5356	173.0167	173.6449	174.3079	173.9164	174.6482	173.8991	175.1081	173.8827	174.9805	173.9346	175.2141	**166.89**
**C5**	118.6951	120.1057	120.7177	120.8251	121.6797	120.7761	122.0034	120.7290	121.8560	120.8746	122.1212	118.8136	120.1112	120.7251	120.7385	121.6008	120.6967	121.8294	120.6564	121.6863	120.7809	121.9289	**116.16**
**C6**	134.9467	136.9717	138.0243	138.0376	139.4645	137.9675	140.0789	137.8990	139.7408	138.1064	140.4199	135.3034	137.3666	138.6127	138.3429	140.0727	138.2853	140.5728	138.2274	140.3897	138.3981	140.9095	**132.19**
**C7**	200.1830	205.0445	206.4951	207.5876	208.7013	207.4269	213.8365	207.2685	212.8952	207.7437	214.6933	204.6033	208.8622	210.0809	211.0822	211.8366	210.9380	216.6033	210.7982	215.3664	211.2259	217.0626	**196.22**
**C8**	62.4961	63.6480	64.3663	63.9871	64.9554	63.9622	65.4882	63.9393	65.2096	64.0139	66.1783	57.1221	58.8511	59.3074	59.6939	60.4822	59.6234	61.3132	59.5575	60.9413	59.7669	61.5421	**59.75**
**C9**	22.4232	22.7099	22.8836	22.9713	23.2313	22.9485	23.3154	22.9273	23.1815	22.9957	23.4231	19.1174	19.6122	19.6632	19.7712	20.0251	19.7540	20.2525	19.7389	20.1245	19.7907	20.6140	**15.44**
**C8`**	35.4509	36.2307	36.4509	36.4979	36.8360	36.4785	37.0927	36.4609	36.9944	36.5195	37.4940	30.7624	31.0177	31.3187	31.2114	31.4738	31.1980	31.6175	31.1846	31.6291	31.2246	31.6596	**31.11**
**H2**	8.7291	8.6745	8.6839	8.6307	8.5912	8.6340	8.6207	8.6372	8.6136	8.6271	8.6069	8.7394	8.5811	8.6311	8.4997	8.5161	8.5067	8.4889	8.5132	8.5182	8.4920	8.4934	**7.97**
**H3**	7.3570	7.4984	7.5860	7.5429	7.6585	7.5407	7.6805	7.5383	7.6697	7.5447	7.6928	7.3444	7.4827	7.5699	7.5283	7.6473	7.5259	7.6672	7.5234	7.6563	7.5302	7.6796	**7.22**
**H5**	7.2043	7.3984	7.4865	7.4833	7.6061	7.4784	7.6287	7.4735	7.6162	7.4877	7.6494	7.1957	7.3777	7.4663	7.4563	7.5822	7.4518	7.5978	7.4472	7.5841	7.4603	7.6170	**7.22**
**H6**	8.1786	8.3833	8.4552	8.4878	8.5945	8.4818	8.6451	8.4757	8.6289	8.4931	8.6774	7.9635	8.1395	8.2598	8.2257	8.3971	8.2217	8.4076	8.2174	8.4040	8.2288	8.4520	**7.97**
**H8**	4.3167	4.4108	4.4199	4.5072	4.5534	4.5008	4.6039	4.4943	4.6020	4.5131	4.6265	4.4991	4.6448	4.7045	4.7095	4.8253	4.7065	4.8443	4.7033	4.8349	4.7119	4.8737	**4.99**
**H(9A-9C)**	1.3125	1.3212	1.3067	1.3099	1.2608	1.3109	1.2798	1.3118	1.2649	1.3088	1.2919	1.3963	1.4224	1.4195	1.4330	1.4094	1.4328	1.4284	1.4324	1.4298	1.4329	1.4515	**1.51**
**H(8`A-8`C)**	2.4651	2.3989	2.3727	2.3791	2.3574	2.3796	2.3472	2.3802	2.3450	2.3785	2.3301	2.8767	2.7128	2.6773	2.6349	2.5897	2.6409	2.5049	2.6468	2.5284	2.6289	2.4711	**2.71**

[a] methyl protons shifts are averaged

[b] according to [3]

Table S11. Cont. VI

Nuclei	R											S											Exp. [b]
	TPSS																						
	Gas	$CHCl_3$ (PCM)	$CHCl_3$ (SMD)	DMSO (PCM)	DMSO (SMD)	CH_3OH (PCM)	CH_3OH (SMD)	C_2H_5OH (PCM)	C_2H_5OH (SMD)	H_2O (PCM)	H_2O (SMD)	Gas	$CHCl_3$ (PCM)	$CHCl_3$ (SMD)	DMSO (PCM)	DMSO (SMD)	CH_3OH (PCM)	CH_3OH (SMD)	C_2H_5OH (PCM)	C_2H_5OH (SMD)	H_2O (PCM)	H_2O (SMD)	
**C1**	132.3080	132.7323	133.5947	132.9917	134.6209	132.9794	133.9174	132.9795	133.4835	133.0145	134.1627	130.9473	132.0464	132.8321	132.7165	134.1527	132.6760	133.8514	132.6492	133.7648	132.7685	133.9904	**129.02**
**C2**	133.0997	133.0157	133.6822	132.9050	133.5045	132.9199	133.9437	132.9461	133.4500	132.9010	133.9167	132.3746	132.5433	133.1843	132.5871	133.2524	132.5896	133.7520	132.6038	133.7197	132.5960	133.7438	**132.19**
**C3**	116.6148	117.2984	117.9237	117.5959	118.2555	117.5862	118.5980	117.5876	118.0301	117.6152	118.6571	116.6745	117.2824	117.8745	117.5564	118.2099	117.5476	118.4658	117.5497	118.7785	117.5754	118.5352	**116.16**
**C4**	170.6841	171.5811	172.3374	172.0858	172.8432	172.0623	173.5294	172.0498	172.7101	172.1186	173.6596	170.9980	171.6247	172.4068	171.9513	172.7573	171.9401	173.2076	171.9397	172.7606	171.9719	173.3439	**166.89**
**C5**	114.5838	115.8370	116.5194	116.5569	117.3608	116.5197	117.6964	116.4938	116.9319	116.6029	117.8309	114.7542	115.8493	116.5202	116.4562	117.2683	116.4268	117.4654	116.4086	117.6460	116.4943	117.6013	**116.16**
**C6**	129.6014	131.4435	132.6218	132.4972	133.9868	132.4389	134.5452	132.3920	132.8937	132.5630	134.9193	129.9037	131.8399	133.1059	132.8246	134.4809	132.7742	135.0675	132.7346	133.8495	132.8818	135.3960	**132.19**
**C7**	196.4615	200.8812	202.3135	203.2587	204.2943	203.1209	208.8163	202.9947	203.7788	203.4005	209.6447	201.0290	204.6160	205.9348	206.6362	207.5175	206.5206	211.4415	206.4167	206.9790	206.7570	211.9588	**196.22**
**C8**	65.1158	66.2120	66.9967	66.5823	67.5160	66.5694	67.7566	66.5676	66.8060	66.6049	68.5367	60.1278	61.8831	62.5632	62.8019	63.6923	62.7474	64.2607	62.7059	64.3460	62.8662	64.4976	**59.75**
**C9**	23.9382	24.2126	24.5053	24.5049	24.8006	24.4912	24.8817	24.4888	24.5589	24.5284	25.0320	20.6506	21.2665	21.4915	21.5390	21.8543	21.5290	22.1455	21.5306	23.4796	21.5593	22.5571	**15.44**
**C8`**	37.5256	38.2274	38.5539	38.5318	38.8578	38.5228	39.1217	38.5251	38.6513	38.5508	39.5382	33.2385	33.3352	33.6679	33.4955	33.7511	33.4908	33.9306	33.4973	35.3565	33.5099	33.9584	**31.11**
**H2**	8.5053	8.4506	8.4858	8.4146	8.4078	8.4174	8.4634	8.4207	8.2608	8.4123	8.4454	8.5333	8.3896	8.4302	8.3110	8.3267	8.3165	8.3314	8.3222	8.4330	8.3060	8.3282	**7.97**
**H3**	7.1662	7.3060	7.4014	7.3539	7.4751	7.3516	7.4934	7.3498	7.1856	7.3564	7.5050	7.1476	7.2856	7.3782	7.3357	7.4586	7.3331	7.4748	7.3311	7.4493	7.3384	7.4865	**7.22**
**H5**	7.0222	7.2106	7.3064	7.2978	7.4257	7.2928	7.4508	7.2885	7.1240	7.3028	7.4683	7.0178	7.1891	7.2832	7.2681	7.3973	7.2637	7.4087	7.2598	7.3786	7.2727	7.4263	**7.22**
**H6**	7.9512	8.1457	8.2359	8.2495	8.3724	8.2435	8.4400	8.2381	8.0774	8.2555	8.4692	7.7288	7.9081	8.0237	7.9982	8.1671	7.9933	8.1988	7.9889	8.1022	8.0030	8.2393	**7.97**
**H8**	4.4709	4.5419	4.5602	4.6283	4.6855	4.6222	4.7477	4.6168	4.4417	4.6345	4.7669	4.5859	4.7329	4.7881	4.8006	4.9128	4.7971	4.9372	4.7941	4.9314	4.8041	4.9650	**4.99**
**H(9A-9C)**	1.3567	1.3679	1.3676	1.3629	1.3311	1.3636	1.3485	1.3649	1.1767	1.3626	1.3622	1.4392	1.4842	1.4875	1.5034	1.4900	1.5025	1.5193	1.5021	1.6640	1.5046	1.5408	**1.51**
**H(8`A-8`C)**	2.5215	2.4527	2.4256	2.4341	2.4118	2.4348	2.3810	2.4361	2.2522	2.4338	2.3643	2.9653	2.7995	2.7626	2.7257	2.6817	2.7310	2.6015	2.7368	2.8894	2.7208	2.5649	**2.71**
Nuclei	VSXC																						
**C1**	130.0829	130.4045	131.1400	130.4934	131.6615	130.4929	131.1365	130.4902	131.2057	130.4951	131.2579	129.9454	130.6445	131.8488	131.2031	132.5520	131.1505	132.4267	131.1004	132.2788	131.2589	132.5715	**129.02**
**C2**	131.4719	131.5765	132.0332	131.5476	131.9755	131.5536	132.4149	131.5574	132.3103	131.5434	132.5007	131.5035	131.5629	132.0835	131.4748	131.9974	131.4854	132.4275	131.4940	132.3584	131.4659	132.4408	**132.19**
**C3**	115.0253	115.9281	116.4781	116.3182	116.9617	116.2986	117.2532	116.2767	117.1666	116.3381	117.3602	115.1018	115.8625	116.3437	116.1552	116.7869	116.1441	116.9700	116.1304	116.9294	116.1665	117.0707	**116.16**
**C4**	168.4085	169.4140	170.1094	169.9432	170.7388	169.9139	171.2602	169.8824	171.1101	169.9727	171.4432	168.2644	169.1345	169.7602	169.5550	170.3438	169.5352	170.7332	169.5129	170.6261	169.5745	170.8988	**166.89**
**C5**	112.9381	114.3706	114.9696	115.1434	115.9400	115.0989	116.2089	115.0522	116.0882	115.1874	116.3893	112.8384	114.1341	114.6817	114.8040	115.5837	114.7687	115.7340	114.7306	115.6555	114.8386	115.8930	**116.16**
**C6**	129.7735	131.5896	132.6289	132.6050	134.1264	132.5458	134.6888	132.4845	134.4417	132.6624	135.0466	129.7921	131.8114	132.8618	132.8875	134.4094	132.8259	134.9250	132.7615	134.6909	132.9470	135.2245	**132.19**
**C7**	190.4964	194.5948	195.8890	196.6716	197.5040	196.5484	201.3041	196.4236	200.4899	196.7897	202.0685	192.4201	196.4292	197.9570	198.6054	199.5513	198.4654	203.3086	198.3255	202.3644	198.7417	203.9258	**196.22**
**C8**	71.0293	71.8644	72.4464	72.1610	72.9614	72.1470	73.3160	72.1307	73.2068	72.1762	73.9137	63.2954	64.4627	65.0975	65.1337	65.9999	65.0894	66.5041	65.0447	66.3060	65.1788	66.9366	**59.75**
**C9**	27.6811	28.6013	28.9238	29.2582	29.7109	29.2182	30.0806	29.1763	29.9393	29.2980	30.1347	28.6031	28.7198	28.7057	28.8776	29.0104	28.8658	28.9877	28.8527	28.9354	28.8913	29.2920	**15.44**
**C8`**	40.4536	40.9423	41.0239	41.1700	41.3482	41.1601	41.3711	41.1480	41.3482	41.1808	41.7912	35.6942	36.3333	36.3426	36.5410	36.8365	36.5434	36.8289	36.5411	36.8765	36.5384	37.0830	**31.11**
**H2**	8.7013	8.6318	8.6383	8.5965	8.5956	8.5987	8.6008	8.6009	8.6015	8.5945	8.6002	8.6698	8.5714	8.5462	8.4980	8.4914	8.5044	8.4746	8.5105	8.4951	8.4915	8.4705	**7.97**
**H3**	7.2076	7.3421	7.4286	7.3891	7.5038	7.3867	7.5214	7.3843	7.5128	7.3914	7.5352	7.2377	7.3761	7.4621	7.4257	7.5415	7.4230	7.5574	7.4205	7.5480	7.4281	7.5730	**7.22**
**H5**	7.1085	7.2957	7.3836	7.3798	7.5056	7.3750	7.5203	7.3702	7.5095	7.3844	7.5443	7.1230	7.2984	7.3840	7.3775	7.4993	7.3729	7.5080	7.3684	7.4968	7.3817	7.5256	**7.22**
**H6**	8.1799	8.3473	8.4451	8.4415	8.5921	8.4354	8.6432	8.4296	8.6230	8.4472	8.6738	7.8041	7.9933	8.0857	8.0898	8.2480	8.0846	8.2771	8.0794	8.2615	8.0945	8.3072	**7.97**
**H8**	4.3876	4.4583	4.4912	4.5227	4.5993	4.5183	4.6338	4.5141	4.6138	4.5269	4.6595	4.6175	4.7683	4.8270	4.8563	4.9599	4.8507	5.0035	4.8453	4.9843	4.8616	5.0218	**4.99**
**H(9A-9C)**	1.5961	1.6138	1.6048	1.6171	1.6025	1.6170	1.6242	1.6171	1.6201	1.6172	1.6378	1.7731	1.7717	1.7506	1.7772	1.7569	1.7769	1.7723	1.7766	1.7683	1.7776	1.7889	**1.51**
**H(8`A-8`C)**	2.6858	2.6354	2.6002	2.6143	2.5757	2.6156	2.5544	2.6170	2.5572	2.6129	2.5289	2.8671	2.7641	2.7059	2.7132	2.6678	2.7172	2.6228	2.7210	2.6423	2.7093	2.5927	**2.71**
Nuclei	wB97																						
**C1**	136.3743	136.5643	137.5508	136.6805	138.3186	136.6682	137.1758	136.6563	137.6400	136.6935	137.3306	135.5496	136.5810	137.2430	137.2393	138.6376	137.1701	138.5360	137.1091	138.2897	137.3170	138.7211	**129.02**
**C2**	140.2891	140.1704	140.7301	140.0418	140.6111	140.0495	141.1373	140.0566	141.0098	140.0348	141.1780	140.0931	140.0448	140.6006	139.9574	140.5644	139.9621	140.9964	139.9662	140.9089	139.9528	141.0199	**132.19**
**C3**	121.6439	122.2148	122.7714	122.4318	123.1041	122.4188	123.2000	122.4051	123.1728	122.4445	123.2668	121.6716	122.2096	122.7417	122.4350	123.1020	122.4187	123.2279	122.4023	123.1663	122.4515	123.2949	**116.16**
**C4**	174.2577	174.9242	175.5185	175.3214	175.9512	175.2956	176.5447	175.2691	176.3511	175.3461	176.6908	174.3838	174.8494	175.5012	175.0939	175.7994	175.0823	176.1349	175.0683	176.0692	175.1036	176.2686	**166.89**
**C5**	119.4831	120.7345	121.3443	121.4210	122.2577	121.3777	122.3482	121.3337	122.2709	121.4624	122.5094	119.5892	120.7784	121.3962	121.3974	122.2486	121.3581	122.3057	121.3179	122.2298	121.4354	122.4485	**116.16**
**C6**	136.8221	138.8055	139.9029	139.8974	141.4938	139.8285	142.2089	139.7589	141.7730	139.9631	142.5634	137.0806	139.0577	140.2748	140.0174	141.7669	139.9692	142.1277	139.9168	141.9973	140.0596	142.4749	**132.19**
**C7**	200.5554	205.5196	206.9736	208.1930	208.8203	208.0253	213.7590	207.8570	212.9387	208.3529	214.6659	204.2854	208.7429	210.1036	211.2165	211.7853	211.0496	216.9049	210.8871	215.6715	211.3809	217.6015	**196.22**
**C8**	61.2286	62.1722	62.8269	62.5542	63.4515	62.5338	63.8040	62.5119	63.7336	62.5735	64.4107	55.7397	57.1823	57.6139	58.0157	58.7514	57.9530	59.4666	57.8934	59.1612	58.0807	59.7826	**59.75**
**C9**	22.0700	22.2380	22.4228	22.4373	22.8248	22.4194	22.7743	22.4016	22.6602	22.4557	22.8715	19.5465	19.7727	19.7821	19.8738	20.1420	19.8648	20.1501	19.8559	20.0913	19.8837	20.5181	**15.44**
**C8`**	35.1652	35.7649	35.9908	36.0743	36.3679	36.0567	36.5472	36.0377	36.5002	36.0907	36.9598	30.5263	30.8779	31.2072	31.0800	31.3808	31.0803	31.3789	31.0759	31.4642	31.0751	31.4895	**31.11**
**H2**	8.8384	8.7915	8.8061	8.7534	8.7565	8.7561	8.8198	8.7586	8.7788	8.7508	8.8177	8.8444	8.6993	8.7460	8.6049	8.6340	8.6136	8.5895	8.6213	8.6222	8.5954	8.5916	**7.97**
**H3**	7.4415	7.5770	7.6646	7.6196	7.7405	7.6173	7.7555	7.6149	7.7454	7.6217	7.7699	7.4525	7.5863	7.6739	7.6277	7.7500	7.6258	7.7576	7.6236	7.7514	7.6295	7.7730	**7.22**
**H5**	7.3028	7.4922	7.5805	7.5754	7.7036	7.5705	7.7161	7.5655	7.7041	7.5800	7.7398	7.3123	7.4932	7.5821	7.5675	7.6967	7.5632	7.7017	7.5588	7.6926	7.5714	7.7244	**7.22**
**H6**	8.2363	8.4473	8.5401	8.5626	8.6990	8.5558	8.7730	8.5487	8.7240	8.5691	8.8069	8.0771	8.2684	8.3886	8.3453	8.5299	8.3432	8.5210	8.3402	8.5241	8.3465	8.5617	**7.97**
**H8**	4.0794	4.2013	4.2289	4.3015	4.3612	4.2941	4.4167	4.2867	4.3964	4.3088	4.4426	4.2843	4.4417	4.5098	4.5068	4.6254	4.5039	4.6339	4.5005	4.6294	4.5091	4.6610	**4.99**
**H(9A-9C)**	1.3373	1.3502	1.3355	1.3434	1.3206	1.3441	1.3538	1.3446	1.3316	1.3428	1.3678	1.4052	1.4218	1.4124	1.4283	1.4126	1.4285	1.4264	1.4284	1.4276	1.4279	1.4449	**1.51**
**H(8`A-8`C)**	2.4600	2.4029	2.3721	2.3774	2.3594	2.3781	2.3295	2.3790	2.3441	2.3770	2.3180	2.8246	2.6620	2.6344	2.5859	2.5485	2.5907	2.4603	2.5952	2.4869	2.5812	2.4374	**2.71**
Nuclei	wB97X																						
**C1**	137.8029	138.0540	139.0211	138.1992	139.8961	138.1870	138.7316	138.1755	139.5920	138.2141	139.6814	136.9016	138.0110	138.6568	138.6712	140.0912	138.6060	140.0704	138.5482	139.7173	138.7443	140.1871	**129.02**
**C2**	140.7055	140.6032	141.1665	140.4847	141.0596	140.4945	141.6070	140.5037	141.4061	140.4762	141.5856	140.5526	140.5103	141.0764	140.4186	141.0232	140.4259	141.4852	140.4318	141.3445	140.4125	141.4959	**132.19**
**C3**	122.3475	122.9417	123.4842	123.1709	123.8319	123.1596	123.9911	123.1475	123.9336	123.1825	124.0993	122.3753	122.9427	123.4616	123.1718	123.8332	123.1584	124.0179	123.1444	123.8893	123.1862	124.0701	**116.16**
**C4**	174.9165	175.6237	176.2197	176.0405	176.6624	176.0165	177.3046	175.9916	177.0258	176.0640	177.3657	175.0408	175.5562	176.2009	175.8192	176.5285	175.8083	176.9078	175.7954	176.7811	175.8288	177.0410	**166.89**
**C5**	120.1726	121.4443	122.0434	122.1523	122.9687	122.1105	123.1411	122.0679	123.0163	122.1931	123.3151	120.2954	121.5031	122.1147	122.1275	122.9742	122.0916	123.0943	122.0548	122.9461	122.1629	123.2277	**116.16**
**C6**	137.1768	139.1619	140.2691	140.2457	141.8067	140.1813	142.5581	140.1156	141.8911	140.3073	142.5689	137.4753	139.4432	140.6731	140.4268	142.1500	140.3797	142.5033	140.3282	142.3574	140.4684	142.8687	**132.19**
**C7**	202.1768	207.1982	208.6196	209.9149	210.5679	209.7483	215.5392	209.5818	215.1279	210.0748	217.1331	206.1849	210.6989	211.9868	213.1284	213.7439	212.9665	218.9548	212.8080	217.5873	213.2885	219.5702	**196.22**
**C8**	62.2638	63.2785	63.9563	63.6403	64.5673	63.6254	64.9558	63.6092	64.7914	63.6554	65.6105	56.7269	58.2485	58.7010	59.1431	59.8693	59.0782	60.6326	59.0159	60.2657	59.2102	60.9139	**59.75**
**C9**	22.2545	22.4780	22.6788	22.6724	23.0493	22.6560	23.0235	22.6394	22.7994	22.6898	23.0038	19.5952	19.8548	19.8730	19.9815	20.2271	19.9737	20.2590	19.9655	20.1660	19.9902	20.6043	**15.44**
**C8`**	35.4261	36.0803	36.3153	36.4017	36.7065	36.3882	36.9314	36.3733	36.7947	36.4152	37.3166	30.7265	31.0292	31.3557	31.2646	31.5524	31.2617	31.6100	31.2549	31.6389	31.2646	31.7241	**31.11**
**H2**	8.8045	8.7552	8.7713	8.7178	8.7132	8.7209	8.7796	8.7237	8.6982	8.7149	8.6978	8.8202	8.6677	8.7159	8.5736	8.5991	8.5821	8.5540	8.5897	8.5893	8.5645	8.5574	**7.97**
**H3**	7.4131	7.5511	7.6385	7.5956	7.7147	7.5935	7.7298	7.5914	7.7207	7.5978	7.7443	7.4226	7.5575	7.6444	7.6018	7.7223	7.5997	7.7328	7.5974	7.7240	7.6039	7.7460	**7.22**
**H5**	7.2774	7.4679	7.5557	7.5537	7.6786	7.5490	7.6961	7.5442	7.6777	7.5583	7.7129	7.2894	7.4695	7.5577	7.5457	7.6732	7.5418	7.6791	7.5378	7.6674	7.5496	7.7001	**7.22**
**H6**	8.2021	8.4135	8.5069	8.5295	8.6573	8.5233	8.7307	8.5170	8.6684	8.5355	8.7254	8.0367	8.2260	8.3460	8.3104	8.4911	8.3074	8.4771	8.3036	8.4843	8.3126	8.5189	**7.97**
**H8**	4.1390	4.2501	4.2742	4.3513	4.4040	4.3440	4.4602	4.3368	4.4505	4.3585	4.4898	4.3255	4.4860	4.5491	4.5527	4.6724	4.5495	4.6828	4.5459	4.6757	4.5555	4.7093	**4.99**
**H(9A-9C)**	1.3232	1.3360	1.3236	1.3281	1.3024	1.3291	1.3388	1.3299	1.2880	1.3271	1.3094	1.3934	1.4128	1.4075	1.4242	1.4050	1.4244	1.4147	1.4244	1.4169	1.4237	1.4340	**1.51**
**H(8`A-8`C)**	2.4671	2.4104	2.3794	2.3869	2.3692	2.3875	2.3398	2.3882	2.3568	2.3865	2.3429	2.8470	2.6868	2.6544	2.6087	2.5717	2.6137	2.4834	2.6186	2.5074	2.6037	2.4553	**2.71**

[a] methyl protons shifts are averaged

[b] according to [3]

Table S11. Cont. VII

Nuclei	R										
	X3LYP										
	Gas	$CHCl_3$ (PCM)	$CHCl_3$ (SMD)	DMSO (PCM)	DMSO (SMD)	CH_3OH (PCM)	CH_3OH (SMD)	C_2H_5OH (PCM)	C_2H_5OH (SMD)	H_2O (PCM)	H_2O (SMD)
**C1**	139.3564	139.8444	140.8482	140.1134	142.0566	140.0909	141.3658	140.0679	141.5227	140.1411	141.6467
**C2**	139.9182	139.8474	140.4108	139.6953	140.1921	139.7116	140.6326	139.7238	140.5055	139.6831	140.6144
**C3**	122.0092	122.6988	123.2345	122.9633	123.5748	122.9518	123.8529	122.9370	123.8097	122.9783	123.9078
**C4**	176.1825	177.0882	177.7250	177.5818	178.2391	177.5544	178.8901	177.5242	178.7610	177.6114	179.0087
**C5**	119.8141	121.1439	121.7439	121.8710	122.6356	121.8294	122.8875	121.7849	122.7972	121.9143	123.0321
**C6**	136.1581	138.1629	139.2158	139.2637	140.6897	139.2042	141.2883	139.1404	140.9981	139.3229	141.6419
**C7**	202.8571	207.9768	209.5518	210.7426	211.8371	210.5640	217.1792	210.3863	216.3022	210.9197	218.1127
**C8**	65.0592	66.2153	66.9018	66.5407	67.5099	66.5316	67.8451	66.5179	67.6413	66.5516	68.6175
**C9**	23.0316	23.3183	23.4327	23.5997	23.8343	23.5889	23.8506	23.5740	23.7548	23.6127	23.9870
**C8`**	36.3944	37.1898	37.4326	37.5008	37.8523	37.4859	38.0604	37.4681	38.0401	37.5189	38.4847
**H2**	8.6524	8.5918	8.6063	8.5511	8.5135	8.5549	8.5499	8.5579	8.5426	8.5473	8.5316
**H3**	7.3213	7.4551	7.5439	7.5005	7.6121	7.4985	7.6290	7.4960	7.6209	7.5023	7.6416
**H5**	7.1716	7.3592	7.4485	7.4464	7.5662	7.4416	7.5856	7.4362	7.5755	7.4510	7.6064
**H6**	8.0809	8.2845	8.3663	8.3962	8.5098	8.3898	8.5716	8.3828	8.5557	8.4024	8.5971
**H8**	4.3443	4.4331	4.4575	4.5383	4.5907	4.5308	4.6494	4.5230	4.6418	4.5456	4.6641
**H(9A-9C)**	1.3227	1.3314	1.3179	1.3217	1.2790	1.3235	1.2915	1.3245	1.2806	1.3199	1.3039
**H(8`A-8`C)**	2.5170	2.4557	2.4302	2.4400	2.4194	2.4409	2.4037	2.4412	2.4048	2.4393	2.3899

Nuclei	S											Exp. [b]
	X3LYP											
	Gas	$CHCl_3$ (PCM)	$CHCl_3$ (SMD)	DMSO (PCM)	DMSO (SMD)	CH_3OH (PCM)	CH_3OH (SMD)	C_2H_5OH (PCM)	C_2H_5OH (SMD)	H_2O (PCM)	H_2O (SMD)	
**C1**	137.9117	139.2562	139.9366	139.9579	141.5273	139.9149	141.3110	139.8710	141.2013	140.0058	141.3487	**129.02**
**C2**	139.2169	139.3848	139.9238	139.3913	140.0004	139.3937	140.5425	139.3932	140.4245	139.3932	140.5430	**132.19**
**C3**	122.1353	122.7433	123.2197	122.9818	123.5816	122.9719	123.7914	122.9587	123.7342	122.9949	123.8481	**116.16**
**C4**	176.5499	177.1393	177.8065	177.4289	178.1522	177.4164	178.5567	177.4002	178.4628	177.4439	178.7115	**166.89**
**C5**	119.9992	121.2093	121.7793	121.8153	122.5836	121.7831	122.7292	121.7474	122.6490	121.8494	122.8641	**116.16**
**C6**	136.5146	138.5767	139.8645	139.6151	141.2627	139.5588	141.7748	139.4982	141.5895	139.6709	142.1688	**132.19**
**C7**	208.1208	212.3136	213.4589	214.5395	215.3412	214.4092	220.0232	214.2763	218.9420	214.6672	220.5368	**196.22**
**C8**	59.3212	61.3577	61.9949	62.3629	63.2348	62.3022	63.9246	62.2400	63.6812	62.4263	64.1714	**59.75**
**C9**	19.5775	20.0461	20.2181	20.2904	20.5151	20.2799	20.6827	20.2663	20.6108	20.3042	21.0438	**15.44**
**C8`**	31.9991	32.1508	32.4082	32.3228	32.5612	32.3139	32.6685	32.3014	32.6855	32.3346	32.7610	**31.11**
**H2**	8.6901	8.5204	8.5632	8.4372	8.4423	8.4428	8.4297	8.4475	8.4451	8.4317	8.4405	**7.97**
**H3**	7.3067	7.4391	7.5255	7.4863	7.6016	7.4841	7.6164	7.4812	7.6087	7.4884	7.6303	**7.22**
**H5**	7.1634	7.3365	7.4251	7.4152	7.5364	7.4110	7.5463	7.4062	7.5365	7.4192	7.5665	**7.22**
**H6**	7.8813	8.0490	8.1656	8.1364	8.2978	8.1318	8.3147	8.1265	8.3041	8.1406	8.3622	**7.97**
**H8**	4.4903	4.6483	4.7010	4.7147	4.8269	4.7114	4.8459	4.7075	4.8333	4.7177	4.8776	**4.99**
**H(9A-9C)**	1.3991	1.4351	1.4417	1.4560	1.4332	1.4550	1.4533	1.4534	1.4521	1.4569	1.4778	**1.51**
**H(8`A-8`C)**	2.9813	2.8071	2.7639	2.7278	2.6788	2.7332	2.5951	2.7379	2.6158	2.7225	2.5651	**2.71**

[a] methyl protons shifts are averaged

[b] according to [3]

Table S12. Theoretical chemical shifts (in ppm) of *(R,S)*-4-fluoro-3-methyl-α-pyrrolidinopentiophenone calculated with HF and selected functionals combined with 6-311++G** basis set[a]

Nuclei	R											S											Exp. [b]
	B3LYP																						
	Gas	$CHCl_3$ (PCM)	$CHCl_3$ (SMD)	DMSO (PCM)	DMSO (SMD)	CH_3OH (PCM)	CH_3OH (SMD)	C_2H_5OH (PCM)	C_2H_5OH (SMD)	H_2O (PCM)	H_2O (SMD)	Gas	$CHCl_3$ (PCM)	$CHCl_3$ (SMD)	DMSO (PCM)	DMSO (SMD)	CH_3OH (PCM)	CH_3OH (SMD)	C_2H_5OH (PCM)	C_2H_5OH (SMD)	H_2O (PCM)	H_2O (SMD)	
**C1**	139.3755	140.0427	142.7648	140.5668	143.1256	140.4942	142.1439	140.4324	142.3391	140.6510	142.5183	139.3757	140.0427	142.7649	140.5666	143.1255	140.4942	142.1439	140.4324	142.3392	140.6510	142.5183	**131.615**
**C2**	140.4036	140.2933	140.1983	140.0339	140.5590	140.0477	140.9647	140.0620	140.8421	140.0201	140.9432	140.4037	140.2932	140.1983	140.0341	140.5590	140.0477	140.9647	140.0620	140.8422	140.0202	140.9432	**133.405**
**C3**	133.9440	135.1377	136.1303	135.4566	136.4910	135.4358	136.8612	135.4144	136.7649	135.4750	137.0063	133.9439	135.1376	136.1302	135.4566	136.4910	135.4358	136.8612	135.4144	136.7650	135.4748	137.0063	**126.19**
**C4**	175.0792	176.1018	176.8933	176.6232	177.2539	176.5809	177.9822	176.5396	177.7833	176.6634	178.0779	175.0792	176.1018	176.8932	176.6231	177.2540	176.5809	177.9822	176.5396	177.7832	176.6633	178.0779	**165.04**
**C5**	119.1645	120.4066	121.4952	121.0395	121.8558	120.9904	122.1814	120.9432	122.0557	121.0871	122.3008	119.1644	120.4066	121.4953	121.0394	121.8560	120.9904	122.1814	120.9433	122.0557	121.0871	122.3008	**116.425**
**C6**	133.2854	134.9765	136.3836	135.8189	136.7443	135.7750	137.5550	135.7275	137.2382	135.8526	137.8211	133.2853	134.9766	136.3837	135.8189	136.7443	135.7750	137.5550	135.7275	137.2381	135.8527	137.8211	**129.92**
**C7**	204.0478	208.6264	212.4871	211.3148	212.8480	211.1090	217.7068	210.9135	216.7338	211.5241	218.9014	204.0478	208.6263	212.4873	211.3149	212.8477	211.1090	217.7068	210.9135	216.7338	211.5244	218.9014	**195.9**
**C8**	66.3870	67.4898	68.8273	68.0355	69.1880	67.9921	69.6095	67.9500	69.5372	68.0774	70.0273	66.3870	67.4897	68.8273	68.0357	69.1880	67.9921	69.6095	67.9500	69.5372	68.0774	70.0273	**67.6**
**C9**	38.8637	38.5069	38.1349	38.2985	38.4957	38.3121	38.6517	38.3239	38.5708	38.2809	38.7252	38.8637	38.5069	38.1349	38.2985	38.4955	38.3121	38.6517	38.3239	38.5708	38.2809	38.7252	**32.2**
**C10**	19.9220	20.4111	20.7730	20.6425	21.1338	20.6258	21.0400	20.6084	21.0077	20.6555	21.1435	19.9221	20.4111	20.7731	20.6426	21.1338	20.6258	21.0400	20.6083	21.0077	20.6555	21.1435	**23.4**
**C11**	16.1211	16.2907	16.1611	16.3120	16.5218	16.3082	16.4855	16.3038	16.4696	16.3129	16.6485	16.1210	16.2907	16.1610	16.3119	16.5218	16.3082	16.4855	16.3038	16.4696	16.3128	16.6486	**17.9**
**C1`**	52.3772	53.2656	53.9433	53.7458	54.3041	53.7148	54.2632	53.6829	54.2101	53.7723	54.6892	56.8929	58.0272	58.6062	58.4745	58.9669	58.4411	59.1773	58.4080	59.0987	58.5051	59.4059	**52.4**
**C2`**	26.1369	26.6323	26.8080	26.7823	27.1686	26.7692	27.2343	26.7556	27.2235	26.7928	27.1286	25.8015	25.9684	26.3079	26.0974	26.6686	26.0796	26.6164	26.0632	26.5864	26.1146	26.3895	**14.5**
**C3`**	25.8015	25.9683	26.3079	26.0972	26.6686	26.0796	26.6164	26.0632	26.5864	26.1146	26.3895	26.1368	26.6323	26.8080	26.7823	27.1686	26.7692	27.2343	26.7556	27.2235	26.7929	27.1286	**14.2**
**C4`**	56.8929	58.0273	58.6064	58.4745	58.9669	58.4411	59.1773	58.4080	59.0986	58.5051	59.4058	52.3772	53.2656	53.9433	53.7457	54.3041	53.7148	54.2632	53.6828	54.2101	53.7722	54.6892	**53.9**
**H2**	8.5043	8.4250	8.3022	8.3514	8.2863	8.3583	8.3299	8.3645	8.3140	8.3433	8.3138	8.5043	8.4250	8.3022	8.3514	8.2863	8.3583	8.3299	8.3645	8.3140	8.3433	8.3138	**8.13**
**H5**	7.0921	7.2500	7.4512	7.3157	7.4353	7.3115	7.4552	7.3074	7.4420	7.3196	7.4769	7.0921	7.2501	7.4512	7.3157	7.4353	7.3115	7.4552	7.3074	7.4420	7.3196	7.4769	**8.03**
**H6**	7.7808	7.9632	8.1461	8.0508	8.1302	8.0461	8.1971	8.0410	8.1685	8.0546	8.2240	7.7808	7.9632	8.1461	8.0509	8.1302	8.0461	8.1971	8.0410	8.1685	8.0546	8.2240	**7.4**
**H(3``A-3``C)**	2.3644	2.4096	2.4611	2.4225	2.4452	2.4212	2.4528	2.4200	2.4474	2.4236	2.4601	2.3644	2.4096	2.4611	2.4225	2.4452	2.4212	2.4528	2.4200	2.4474	2.4236	2.4601	**2.32**
**H8**	4.6160	4.6788	4.7835	4.7191	4.7676	4.7163	4.8213	4.7133	4.7986	4.7215	4.8118	4.6160	4.6788	4.7835	4.7191	4.7676	4.7163	4.8213	4.7133	4.7986	4.7215	4.8118	**5.64**
**H(9A-9B)**	1.6648	1.7163	1.7262	1.7361	1.7103	1.7367	1.7043	1.7364	1.6942	1.7345	1.7317	1.6648	1.7163	1.7262	1.7362	1.7103	1.7367	1.7043	1.7364	1.6942	1.7345	1.7317	**1.92**
**H(10A)**	1.0804	0.9756	0.8678	0.9260	0.8519	0.9290	0.9026	0.9321	0.8926	0.9229	0.9126	1.0804	0.9756	0.8678	0.9260	0.8519	0.9290	0.9026	0.9321	0.8926	0.9229	0.9126	**1.26**
**H(10B)**	1.7462	1.6391	1.5015	1.5641	1.4856	1.5682	1.4844	1.5724	1.4863	1.5600	1.4513	1.7462	1.6391	1.5015	1.5641	1.4856	1.5682	1.4844	1.5724	1.4863	1.5600	1.4513	**1.07**
**H(11A-11C)**	0.7741	0.7878	0.7686	0.7923	0.7528	0.7917	0.7585	0.7911	0.7541	0.7926	0.7490	0.7741	0.7878	0.7686	0.7923	0.7527	0.7917	0.7585	0.7911	0.7541	0.7926	0.7490	**0.78**
**H(1`A)**	2.7322	2.7249	2.6772	2.7097	2.6613	2.7105	2.6677	2.7112	2.6676	2.7087	2.6527	2.8527	2.8515	2.8315	2.8353	2.8156	2.8358	2.8203	2.8364	2.8237	2.8348	2.7952	**3.47**
**H(1`B)**	3.1916	3.2330	3.3417	3.2639	3.3258	3.2618	3.3041	3.2595	3.2957	3.2655	3.3097	2.8692	2.7005	2.7317	2.6722	2.7158	2.6720	2.6616	2.6722	2.6626	2.6726	2.6622	**3.63**
**H(4`A)**	2.8692	2.7005	2.7317	2.6722	2.7158	2.6720	2.6616	2.6722	2.6626	2.6726	2.6622	3.1916	3.2330	3.3417	3.2639	3.3258	3.2618	3.3041	3.2595	3.2957	3.2655	3.3097	**3.07**
**H(4`B)**	2.8526	2.8515	2.8315	2.8353	2.8156	2.8358	2.8203	2.8364	2.8237	2.8348	2.7952	2.7322	2.7249	2.6772	2.7097	2.6613	2.7105	2.6677	2.7112	2.6676	2.7087	2.6527	**3.25**
Nuclei	**B3P86**																						
**C1**	136.8295	137.4209	138.1401	137.8085	139.4870	137.7905	138.6374	137.7537	138.7146	137.8735	138.9687	136.8295	137.4209	138.1401	137.8085	139.4870	137.7903	138.6373	137.7538	138.7146	137.8734	138.9687	**131.615**
**C2**	139.6013	139.4796	139.9370	139.2408	139.8134	139.2819	140.0902	139.3003	140.1047	139.2447	140.0817	139.6012	139.4796	139.9370	139.2408	139.8134	139.2819	140.0904	139.3004	140.1047	139.2445	140.0817	**133.405**
**C3**	132.0870	133.3567	134.3510	133.7464	134.8443	133.7466	135.1908	133.7244	135.1953	133.7900	135.3475	132.0869	133.3568	134.3511	133.7464	134.8443	133.7465	135.1909	133.7244	135.1953	133.7900	135.3475	**126.19**
**C4**	172.5187	173.4314	174.1336	173.9211	174.5502	173.9074	175.3563	173.8729	175.2591	173.9788	175.5104	172.5188	173.4315	174.1336	173.9211	174.5501	173.9074	175.3563	173.8730	175.2590	173.9789	175.5104	**165.04**
**C5**	118.3657	119.6147	120.2815	120.2784	121.1201	120.2564	121.4226	120.2131	121.3943	120.3438	121.5872	118.3656	119.6147	120.2814	120.2784	121.1201	120.2565	121.4227	120.2131	121.3943	120.3438	121.5872	**116.425**
**C6**	132.3825	134.0649	135.1198	135.0139	136.3572	134.9801	137.0877	134.9234	136.9696	135.0884	137.4042	132.3824	134.0649	135.1197	135.0139	136.3572	134.9801	137.0878	134.9233	136.9696	135.0884	137.4042	**129.92**
**C7**	202.1737	206.4036	207.4086	208.8194	209.5878	208.6743	214.2270	208.5108	213.1729	209.0057	215.3496	202.1736	206.4036	207.4086	208.8194	209.5878	208.6745	214.2273	208.5110	213.1729	209.0055	215.3496	**195.9**
**C8**	64.6997	65.8115	66.2596	66.3673	67.3068	66.3501	67.7370	66.3121	67.6435	66.4284	68.1601	64.6995	65.8115	66.2596	66.3673	67.3068	66.3502	67.7370	66.3121	67.6435	66.4284	68.1601	**67.6**
**C9**	37.7290	37.4811	37.7349	37.3766	37.7863	37.4041	37.8947	37.4097	37.9104	37.3942	38.0409	37.7290	37.4811	37.7348	37.3766	37.7864	37.4041	37.8947	37.4098	37.9104	37.3942	38.0409	**32.2**
**C10**	18.6596	19.1347	19.4324	19.4345	19.9540	19.4372	19.8043	19.4179	19.8809	19.4755	19.9523	18.6596	19.1347	19.4324	19.4345	19.9539	19.4371	19.8043	19.4178	19.8809	19.4755	19.9523	**23.4**
**C11**	15.7178	15.8557	16.0180	15.9133	16.1369	15.9305	16.0762	15.9255	16.1701	15.9408	16.2599	15.7178	15.8557	16.0180	15.9133	16.1368	15.9304	16.0762	15.9255	16.1701	15.9408	16.2599	**17.9**
**C1`**	51.2961	52.1242	52.3788	52.6108	53.1461	52.6025	53.0963	52.5718	53.1618	52.6620	53.5407	55.5635	56.6358	57.0546	57.0874	57.5827	57.0806	57.8202	57.0522	57.8535	57.1376	58.0713	**52.4**
**C2`**	24.9356	25.3534	25.6763	25.5182	25.9499	25.5290	25.9593	25.5180	26.0584	25.5516	25.8309	24.4380	24.5916	25.0348	24.7215	25.2332	24.7318	25.1479	24.7206	25.2377	24.7560	24.9572	**14.5**
**C3`**	24.4379	24.5916	25.0349	24.7215	25.2332	24.7319	25.1479	24.7206	25.2377	24.7561	24.9571	24.9356	25.3534	25.6764	25.5182	25.9499	25.5291	25.9593	25.5179	26.0584	25.5516	25.8309	**14.2**
**C4`**	55.5634	56.6358	57.0546	57.0874	57.5828	57.0807	57.8202	57.0522	57.8535	57.1376	58.0713	51.2960	52.1242	52.3787	52.6108	53.1461	52.6024	53.0962	52.5719	53.1618	52.6620	53.5407	**53.9**
**H2**	8.5589	8.4844	8.5153	8.4223	8.4239	8.4288	8.4540	8.4331	8.4632	8.4171	8.4418	8.5589	8.4844	8.5153	8.4223	8.4239	8.4288	8.4540	8.4331	8.4632	8.4171	8.4418	**8.13**
**H5**	7.1119	7.2807	7.3752	7.3491	7.4737	7.3466	7.4871	7.3421	7.4823	7.3527	7.5085	7.1119	7.2807	7.3752	7.3491	7.4738	7.3466	7.4871	7.3421	7.4823	7.3527	7.5085	**8.03**
**H6**	7.8606	8.0344	8.1337	8.1253	8.2514	8.1211	8.3216	8.1150	8.3099	8.1305	8.3473	7.8606	8.0344	8.1337	8.1253	8.2514	8.1211	8.3216	8.1149	8.3099	8.1305	8.3473	**7.4**
**H(3``A-3``C)**	2.3528	2.3991	2.4152	2.4110	2.4349	2.4115	2.4414	2.4102	2.4420	2.4118	2.4506	2.3528	2.3991	2.4152	2.4110	2.4349	2.4115	2.4415	2.4102	2.4420	2.4118	2.4506	**2.32**
**H8**	4.6145	4.6807	4.7497	4.7251	4.7937	4.7231	4.8542	4.7194	4.8422	4.7282	4.8541	4.6145	4.6807	4.7497	4.7251	4.7937	4.7231	4.8543	4.7194	4.8422	4.7282	4.8541	**5.64**
**H(9A-9B)**	1.7347	1.7800	1.7641	1.8098	1.8250	1.8097	1.8119	1.8075	1.8121	1.8112	1.8427	1.7347	1.7799	1.7641	1.8098	1.8250	1.8097	1.8119	1.8075	1.8121	1.8112	1.8427	**1.92**
**H(10A)**	1.0371	0.9309	0.9356	0.8799	0.8098	0.8846	0.8521	0.8874	0.8556	0.8765	0.8620	1.0371	0.9308	0.9356	0.8799	0.8098	0.8846	0.8521	0.8874	0.8556	0.8765	0.8620	**1.26**
**H(10B)**	1.7619	1.6513	1.5951	1.5752	1.4998	1.5809	1.4980	1.5848	1.5093	1.5711	1.4652	1.7619	1.6513	1.5951	1.5752	1.4998	1.5809	1.4980	1.5848	1.5093	1.5711	1.4652	**1.07**
**H(11A-11C)**	0.7378	0.7516	0.7360	0.7562	0.7247	0.7571	0.7305	0.7562	0.7358	0.7566	0.7228	0.7378	0.7517	0.7360	0.7562	0.7247	0.7570	0.7305	0.7562	0.7358	0.7566	0.7228	**0.78**
**H(1`A)**	2.6871	2.6842	2.6690	2.6754	2.6359	2.6771	2.6413	2.6769	2.6461	2.6750	2.6265	2.8044	2.8092	2.7946	2.7927	2.7700	2.7948	2.7756	2.7951	2.7802	2.7920	2.7503	**3.47**
**H(1`B)**	3.1964	3.2353	3.2638	3.2658	3.3315	3.2649	3.3071	3.2621	3.3065	3.2680	3.3120	2.9088	2.7204	2.6825	2.6841	2.6992	2.6860	2.6352	2.6862	2.6315	2.6838	2.6379	**3.63**
**H(4`A)**	2.9088	2.7204	2.6825	2.6841	2.6992	2.6860	2.6352	2.6862	2.6315	2.6838	2.6379	3.1964	3.2353	3.2638	3.2658	3.3316	3.2649	3.3071	3.2621	3.3065	3.2680	3.3120	**3.07**
**H(4`B)**	2.8044	2.8092	2.7946	2.7927	2.7700	2.7948	2.7756	2.7951	2.7802	2.7920	2.7503	2.6871	2.6842	2.6690	2.6754	2.6359	2.6770	2.6413	2.6769	2.6461	2.6750	2.6265	**3.25**

[a] methyl protons shifts are averaged

[b] according to [11]

Table S12. Cont. I

Nuclei	R											S											Exp. [b]
	B3PW91																						
	Gas	$CHCl_3$ (PCM)	$CHCl_3$ (SMD)	DMSO (PCM)	DMSO (SMD)	CH_3OH (PCM)	CH_3OH (SMD)	C_2H_5OH (PCM)	C_2H_5OH (SMD)	H_2O (PCM)	H_2O (SMD)	Gas	$CHCl_3$ (PCM)	$CHCl_3$ (SMD)	DMSO (PCM)	DMSO (SMD)	CH_3OH (PCM)	CH_3OH (SMD)	C_2H_5OH (PCM)	C_2H_5OH (SMD)	H_2O (PCM)	H_2O (SMD)	
**C1**	137.0403	137.7221	138.3072	138.2788	140.9199	138.2202	139.8997	138.1604	140.0100	138.3475	140.3049	137.0402	137.7220	138.3071	138.2786	140.6011	138.2202	139.8996	138.1604	140.0101	138.3475	140.3050	**131.615**
**C2**	139.7092	139.6170	140.0050	139.3682	140.2496	139.3928	140.2496	139.4080	140.1605	139.3461	140.2213	139.7090	139.6170	140.0049	139.3681	139.9306	139.3928	140.2497	139.4080	140.1606	139.3461	140.2214	**133.405**
**C3**	132.1402	133.4300	134.3487	133.8139	135.2383	133.7985	135.2880	133.7742	135.2004	133.8304	135.4588	132.1401	133.4300	134.3486	133.8140	134.9193	133.7985	135.2878	133.7742	135.2003	133.8304	135.4587	**126.19**
**C4**	172.6321	173.5898	174.2255	174.0868	175.0779	174.0584	175.4870	174.0212	175.3174	174.1168	175.5947	172.6321	173.5898	174.2255	174.0869	174.7589	174.0584	175.4870	174.0212	175.3174	174.1168	175.5946	**165.04**
**C5**	118.5053	119.7733	120.3612	120.4279	121.6393	120.3901	121.6485	120.3440	121.5329	120.4669	121.7955	118.5053	119.7734	120.3612	120.4278	121.3203	120.3901	121.6487	120.3440	121.5330	120.4669	121.7954	**116.425**
**C6**	132.3426	134.0470	135.0527	134.9248	136.2711	134.8833	136.5931	134.8310	136.3313	134.9622	136.8953	132.3425	134.0471	135.0527	134.9248	135.9521	134.8833	136.5932	134.8310	136.3313	134.9622	136.8954	**129.92**
**C7**	202.1763	206.5424	207.4324	209.1500	210.7982	208.9626	215.3942	208.7747	214.3674	209.3414	216.6349	202.1763	206.5427	207.4323	209.1498	210.4790	208.9626	215.3942	208.7747	214.3675	209.3414	216.6349	**195.9**
**C8**	64.6471	65.8094	66.1215	66.4144	67.8268	66.3722	67.9977	66.3240	67.8865	66.4604	68.5238	64.6471	65.8094	66.1214	66.4144	67.5078	66.3722	67.9978	66.3240	67.8864	66.4604	68.5238	**67.6**
**C9**	37.2320	37.0096	37.2074	36.8817	37.4464	36.8940	37.1930	36.8976	37.1256	36.8715	37.3336	37.2320	37.0096	37.2074	36.8818	37.1274	36.8940	37.1930	36.8976	37.1255	36.8715	37.3336	**32.2**
**C10**	18.6232	19.1379	19.3618	19.4063	20.1898	19.3958	19.7341	19.3763	19.7148	19.4177	19.9053	18.6232	19.1379	19.3618	19.4063	19.8708	19.3958	19.7341	19.3763	19.7147	19.4177	19.9053	**23.4**
**C11**	15.7464	15.9094	15.9924	15.9450	16.4560	15.9486	16.0764	15.9430	16.0768	15.9432	16.2582	15.7464	15.9095	15.9924	15.9450	16.1371	15.9486	16.0764	15.9430	16.0768	15.9432	16.2582	**17.9**
**C1`**	51.2166	52.0914	52.2518	52.5475	53.4078	52.5272	53.0134	52.4976	52.9857	52.5681	53.4769	55.5120	56.6077	56.9334	57.0536	57.5727	57.0315	57.7811	57.0008	57.7207	57.0768	58.0230	**52.4**
**C2`**	25.1812	25.6166	25.8406	25.7624	26.4873	25.7589	26.1784	25.7463	26.1896	25.7675	26.0594	24.7226	24.8846	25.2413	25.0099	25.5695	25.0044	25.5095	24.9903	25.5050	25.0177	25.2750	**14.5**
**C3`**	24.7226	24.8847	25.2412	25.0100	25.8886	25.0044	25.5095	24.9903	25.5050	25.0177	25.2750	25.1813	25.6166	25.8406	25.7625	26.1683	25.7589	26.1784	25.7463	26.1898	25.7675	26.0594	**14.2**
**C4`**	55.5120	56.6077	56.9334	57.0535	57.8918	57.0315	57.7811	57.0008	57.7207	57.0768	58.0229	51.2165	52.0915	52.2518	52.5475	53.0889	52.5272	53.0134	52.4976	52.9856	52.5681	53.4769	**53.9**
**H2**	8.5362	8.4551	8.4943	8.3800	8.3471	8.3877	8.3598	8.3944	8.3538	8.3719	8.3397	8.5362	8.4551	8.4943	8.3800	8.3316	8.3877	8.3598	8.3944	8.3538	8.3719	8.3397	**8.13**
**H5**	7.1068	7.2706	7.3648	7.3399	7.4780	7.3363	7.4860	7.3324	7.4744	7.3434	7.5067	7.1068	7.2707	7.3649	7.3399	7.4625	7.3363	7.4860	7.3324	7.4744	7.3434	7.5067	**8.03**
**H6**	7.8438	8.0201	8.1233	8.1067	8.2119	8.1028	8.2536	8.0982	8.2316	8.1102	8.2766	7.8438	8.0201	8.1233	8.1067	8.1964	8.1028	8.2536	8.0982	8.2316	8.1102	8.2766	**7.4**
**H(3``A-3``C)**	2.3434	2.3898	2.4059	2.4044	2.4441	2.4039	2.4399	2.4033	2.4369	2.4051	2.4461	2.3434	2.3899	2.4058	2.4044	2.4286	2.4039	2.4399	2.4033	2.4369	2.4051	2.4461	**2.32**
**H8**	4.6079	4.6693	4.7385	4.7110	4.7783	4.7091	4.8234	4.7066	4.8017	4.7128	4.8142	4.6079	4.6693	4.7385	4.7110	4.7628	4.7091	4.8234	4.7066	4.8017	4.7128	4.8142	**5.64**
**H(9A-9B)**	1.6892	1.7347	1.7214	1.7568	1.7451	1.7568	1.7107	1.7563	1.7050	1.7565	1.7395	1.6892	1.7347	1.7214	1.7568	1.7296	1.7568	1.7106	1.7563	1.7050	1.7565	1.7395	**1.92**
**H(10A)**	1.0241	0.9190	0.9195	0.8653	0.8106	0.8700	0.8492	0.8742	0.8418	0.8607	0.8539	1.0241	0.9190	0.9195	0.8653	0.7951	0.8700	0.8492	0.8742	0.8418	0.8607	0.8539	**1.26**
**H(10B)**	1.7216	1.6103	1.5615	1.5381	1.4733	1.5427	1.4533	1.5471	1.4588	1.5337	1.4193	1.7216	1.6104	1.5615	1.5381	1.4578	1.5427	1.4533	1.5471	1.4588	1.5337	1.4192	**1.07**
**H(11A-11C)**	0.7285	0.7397	0.7250	0.7442	0.7211	0.7444	0.7124	0.7444	0.7117	0.7441	0.6997	0.7285	0.7398	0.7250	0.7442	0.7057	0.7444	0.7124	0.7444	0.7117	0.7441	0.6997	**0.78**
**H(1`A)**	2.6823	2.6742	2.6584	2.6636	2.6340	2.6648	2.6258	2.6656	2.6272	2.6626	2.6099	2.8004	2.8017	2.7829	2.7872	2.7675	2.7883	2.7780	2.7892	2.7820	2.7864	2.7514	**3.47**
**H(1`B)**	3.1722	3.2108	3.2404	3.2414	3.3115	3.2398	3.2744	3.2379	3.2690	3.2430	3.2771	2.8663	2.6883	2.6542	2.6604	2.6903	2.6606	2.6394	2.6608	2.6378	2.6609	2.6369	**3.63**
**H(4`A)**	2.8663	2.6883	2.6542	2.6604	2.7058	2.6606	2.6394	2.6608	2.6378	2.6609	2.6369	3.1722	3.2108	3.2404	3.2414	3.2960	3.2398	3.2744	3.2379	3.2690	3.2430	3.2771	**3.07**
**H(4`B)**	2.8004	2.8017	2.7829	2.7872	2.7830	2.7883	2.7780	2.7892	2.7820	2.7864	2.7514	2.6823	2.6743	2.6584	2.6636	2.6185	2.6648	2.6258	2.6656	2.6272	2.6626	2.6099	**3.25**
Nuclei	B98																						
**C1**	137.9211	138.5798	139.2734	139.0736	141.4106	139.0383	140.6735	139.0051	140.8533	139.1162	141.0483	137.9211	138.5798	139.2734	139.0737	141.4108	139.0385	140.6735	139.0050	140.8533	139.1163	141.0483	**131.615**
**C2**	139.2758	139.1442	139.5291	138.9343	139.4902	138.9655	139.8079	138.9935	139.7159	138.9066	139.7756	139.2757	139.1443	139.5292	138.9344	139.4900	138.9655	139.8081	138.9935	139.7159	138.9066	139.7756	**133.405**
**C3**	132.6234	133.8277	134.7505	134.1920	135.2659	134.1883	135.6179	134.1818	135.5434	134.1975	135.7735	132.6234	133.8278	134.7505	134.1920	135.2657	134.1882	135.6180	134.1817	135.5435	134.1976	135.7735	**126.19**
**C4**	172.4828	173.3581	174.0426	173.8295	174.4933	173.8134	175.2262	173.7952	175.0530	173.8476	175.3221	172.4828	173.3579	174.0425	173.8295	174.4931	173.8133	175.2260	173.7951	175.0529	173.8475	175.3221	**165.04**
**C5**	118.2957	119.4783	120.0595	120.1013	120.9583	120.0766	121.2853	120.0500	121.1785	120.1276	121.4198	118.2957	119.4782	120.0595	120.1012	120.9582	120.0766	121.2852	120.0499	121.1785	120.1275	121.4198	**116.425**
**C6**	132.3682	134.0113	134.9610	134.9078	135.8816	134.8731	136.5314	134.8346	136.2545	134.9403	136.8059	132.3682	134.0113	134.9610	134.9078	135.8817	134.8731	136.5314	134.8345	136.2547	134.9403	136.8059	**129.92**
**C7**	200.6081	205.1118	206.1644	207.7224	209.0355	207.5537	214.1409	207.3876	213.1374	207.8924	215.3578	200.6081	205.1118	206.1644	207.7224	209.0354	207.5537	214.1409	207.3873	213.1373	207.8924	215.3578	**195.9**
**C8**	66.2711	67.3951	67.7401	67.9768	69.0758	67.9487	69.5517	67.9200	69.4808	68.0081	70.0175	66.2711	67.3952	67.7401	67.9768	69.0758	67.9488	69.5517	67.9200	69.4808	68.0081	70.0175	**67.6**
**C9**	38.5312	38.2384	38.4596	38.0938	38.3588	38.1174	38.4289	38.1386	38.3707	38.0734	38.5097	38.5312	38.2383	38.4596	38.0937	38.3587	38.1173	38.4288	38.1387	38.3706	38.0734	38.5097	**32.2**
**C10**	20.0430	20.4824	20.7437	20.7413	21.2037	20.7418	21.0840	20.7396	21.0723	20.7427	21.1981	20.0429	20.4824	20.7437	20.7413	21.2037	20.7418	21.0840	20.7396	21.0723	20.7428	21.1981	**23.4**
**C11**	16.3459	16.4532	16.5836	16.5049	16.7119	16.5173	16.6578	16.5269	16.6626	16.4951	16.8257	16.3459	16.4531	16.5836	16.5048	16.7119	16.5173	16.6578	16.5269	16.6627	16.4951	16.8257	**17.9**
**C1`**	52.4759	53.3041	53.5114	53.7966	54.3257	53.7837	54.2702	53.7677	54.2452	53.8101	54.7016	56.9251	57.9074	58.3002	58.3531	58.8738	58.3399	59.0782	58.3243	59.0187	58.3682	59.2848	**52.4**
**C2`**	26.3508	26.7353	27.0204	26.9044	27.3140	26.9079	27.3476	26.9089	27.3567	26.9035	27.2354	25.8887	26.0193	26.3702	26.1745	26.7324	26.1785	26.6952	26.1802	26.6902	26.1735	26.4841	**14.5**
**C3`**	25.8887	26.0193	26.3702	26.1745	26.7324	26.1785	26.6952	26.1802	26.6902	26.1734	26.4841	26.3510	26.7354	27.0204	26.9045	27.3140	26.9079	27.3475	26.9089	27.3567	26.9035	27.2354	**14.2**
**C4`**	56.9250	57.9073	58.3002	58.3531	58.8739	58.3399	59.0782	58.3242	59.0187	58.3682	59.2848	52.4759	53.3041	53.5114	53.7966	54.3257	53.7837	54.2702	53.7676	54.2452	53.8102	54.7016	**53.9**
**H2**	8.5336	8.4544	8.4877	8.3889	8.3455	8.3947	8.3689	8.4003	8.3585	8.3830	8.3487	8.5336	8.4544	8.4877	8.3889	8.3455	8.3947	8.3689	8.4003	8.3585	8.3830	8.3487	**8.13**
**H5**	7.1008	7.2669	7.3604	7.3367	7.4582	7.3328	7.4812	7.3290	7.4700	7.3407	7.5015	7.1008	7.2669	7.3604	7.3367	7.4582	7.3328	7.4812	7.3290	7.4700	7.3406	7.5015	**8.03**
**H6**	7.8185	7.9981	8.1054	8.0877	8.1781	8.0828	8.2390	8.0779	8.2142	8.0923	8.2641	7.8185	7.9981	8.1054	8.0877	8.1781	8.0829	8.2390	8.0779	8.2142	8.0923	8.2641	**7.4**
**H(3``A-3``C)**	2.3614	2.4093	2.4266	2.4231	2.4481	2.4224	2.4577	2.4218	2.4550	2.4242	2.4625	2.3614	2.4093	2.4266	2.4231	2.4481	2.4224	2.4577	2.4218	2.4550	2.4242	2.4625	**2.32**
**H8**	4.5750	4.6359	4.7129	4.6777	4.7297	4.6751	4.7972	4.6725	4.7740	4.6803	4.7838	4.5750	4.6359	4.7129	4.6777	4.7297	4.6751	4.7973	4.6725	4.7740	4.6803	4.7838	**5.64**
**H(9A-9B)**	1.6832	1.7296	1.7179	1.7516	1.7261	1.7509	1.7072	1.7501	1.7003	1.7522	1.7302	1.6832	1.7296	1.7179	1.7515	1.7261	1.7508	1.7072	1.7501	1.7003	1.7522	1.7302	**1.92**
**H(10A)**	1.0852	0.9633	0.9711	0.9093	0.8364	0.9135	0.8883	0.9176	0.8808	0.9054	0.8920	1.0852	0.9633	0.9711	0.9093	0.8364	0.9135	0.8883	0.9176	0.8808	0.9054	0.8920	**1.26**
**H(10B)**	1.7310	1.6250	1.5780	1.5509	1.4743	1.5554	1.4716	1.5599	1.4769	1.5471	1.4367	1.7310	1.6250	1.5780	1.5509	1.4743	1.5554	1.4716	1.5599	1.4769	1.5471	1.4367	**1.07**
**H(11A-11C)**	0.7552	0.7650	0.7507	0.7698	0.7308	0.7695	0.7370	0.7693	0.7356	0.7702	0.7241	0.7552	0.7650	0.7507	0.7697	0.7308	0.7695	0.7369	0.7693	0.7356	0.7702	0.7241	**0.78**
**H(1`A)**	2.7001	2.6901	2.6759	2.6808	2.6348	2.6814	2.6447	2.6822	2.6467	2.6803	2.6314	2.8208	2.8186	2.8016	2.8037	2.7834	2.8045	2.7945	2.8055	2.7997	2.8032	2.7681	**3.47**
**H(1`B)**	3.1860	3.2218	3.2579	3.2526	3.3082	3.2505	3.2906	3.2485	3.2844	3.2547	3.2913	2.8627	2.7059	2.6708	2.6765	2.7080	2.6772	2.6656	2.6781	2.6671	2.6764	2.6609	**3.63**
**H(4`A)**	2.8627	2.7059	2.6708	2.6765	2.7080	2.6772	2.6656	2.6781	2.6671	2.6764	2.6609	3.1860	3.2218	3.2579	3.2526	3.3082	3.2505	3.2906	3.2485	3.2844	3.2547	3.2913	**3.07**
**H(4`B)**	2.8208	2.8185	2.8016	2.8037	2.7834	2.8045	2.7945	2.8055	2.7997	2.8031	2.7681	2.7001	2.6901	2.6760	2.6808	2.6348	2.6814	2.6447	2.6822	2.6467	2.6803	2.6314	**3.25**

[a] methyl protons shifts are averaged

[b] according to [11]

Table S12. Cont. II

Nuclei	R											S											Exp. [b]
	B971																						
	Gas	$CHCl_3$ (PCM)	$CHCl_3$ (SMD)	DMSO (PCM)	DMSO (SMD)	CH_3OH (PCM)	CH_3OH (SMD)	C_2H_5OH (PCM)	C_2H_5OH (SMD)	H_2O (PCM)	H_2O (SMD)	Gas	$CHCl_3$ (PCM)	$CHCl_3$ (SMD)	DMSO (PCM)	DMSO (SMD)	CH_3OH (PCM)	CH_3OH (SMD)	C_2H_5OH (PCM)	C_2H_5OH (SMD)	H_2O (PCM)	H_2O (SMD)	
**C1**	137.5023	138.1554	138.8248	138.6517	140.5655	138.6077	140.2965	138.5669	140.4574	138.7038	140.6705	137.5023	138.1554	138.8249	138.6515	140.5654	138.6077	140.2965	138.5670	140.4576	138.7038	140.6704	**131.615**
**C2**	138.9808	138.8679	139.2516	138.6718	139.2349	138.6951	139.5148	138.7171	139.4247	138.6555	139.4835	138.9808	138.8679	139.2516	138.6718	139.2348	138.6951	139.5148	138.7171	139.4247	138.6555	139.4835	**133.405**
**C3**	132.3901	133.6047	134.5247	133.9773	135.0276	133.9658	135.3912	133.9534	135.3152	133.9941	135.5527	132.3901	133.6047	134.5247	133.9773	135.0276	133.9658	135.3912	133.9534	135.3151	133.9939	135.5527	**126.19**
**C4**	172.3979	173.2721	173.9443	173.7416	174.3753	173.7181	175.1405	173.6944	174.9699	173.7699	175.2418	172.3979	173.2721	173.9442	173.7416	174.3753	173.7180	175.1405	173.6944	174.9698	173.7698	175.2418	**165.04**
**C5**	118.0738	119.2602	119.8390	119.8882	120.6908	119.8562	121.0571	119.8240	120.9493	119.9247	121.1962	118.0738	119.2602	119.8389	119.8881	120.6908	119.8562	121.0571	119.8240	120.9493	119.9248	121.1962	**116.425**
**C6**	132.1219	133.7655	134.7170	134.6759	135.8521	134.6335	136.2608	134.5895	135.9936	134.7208	136.5477	132.1219	133.7655	134.7170	134.6759	135.8522	134.6336	136.2608	134.5895	135.9936	134.7210	136.5478	**129.92**
**C7**	200.5079	204.9371	205.9614	207.5218	208.4653	207.3457	213.9130	207.1744	212.9147	207.6995	215.1477	200.5079	204.9371	205.9615	207.5217	208.4653	207.3461	213.9130	207.1744	212.9147	207.6995	215.1476	**195.9**
**C8**	66.1003	67.2120	67.5690	67.7979	68.7688	67.7621	69.3420	67.7277	69.2645	67.8399	69.8154	66.1003	67.2120	67.5691	67.7979	68.7688	67.7621	69.3420	67.7277	69.2644	67.8400	69.8153	**67.6**
**C9**	38.5329	38.2732	38.5047	38.1445	38.4938	38.1598	38.4546	38.1749	38.3961	38.1364	38.5557	38.5328	38.2733	38.5048	38.1445	38.4939	38.1597	38.4546	38.1749	38.3961	38.1365	38.5558	**32.2**
**C10**	20.0511	20.4991	20.7580	20.7694	21.2305	20.7620	21.0941	20.7539	21.0801	20.7823	21.2157	20.0511	20.4990	20.7580	20.7694	21.2305	20.7620	21.0941	20.7539	21.0802	20.7823	21.2157	**23.4**
**C11**	16.2983	16.4105	16.5318	16.4740	16.6754	16.4782	16.6076	16.4817	16.6117	16.4758	16.7834	16.2983	16.4105	16.5318	16.4740	16.6755	16.4782	16.6076	16.4817	16.6116	16.4758	16.7834	**17.9**
**C1`**	52.4017	53.2148	53.4250	53.7169	54.2285	53.6959	54.1637	53.6737	54.1399	53.7420	54.6001	56.8263	57.7875	58.1812	58.2424	58.7303	58.2211	58.9632	58.1995	58.9039	58.2685	59.1712	**52.4**
**C2`**	26.1737	26.5501	26.8443	26.7385	27.1369	26.7335	27.1666	26.7281	27.1757	26.7492	27.0687	25.6913	25.8433	26.1892	26.0034	26.5164	25.9995	26.5178	25.9954	26.5127	26.0129	26.3215	**14.5**
**C3`**	25.6914	25.8433	26.1893	26.0034	26.5164	25.9996	26.5178	25.9954	26.5127	26.0128	26.3215	26.1737	26.5501	26.8443	26.7386	27.1370	26.7336	27.1667	26.7281	27.1757	26.7493	27.0687	**14.2**
**C4`**	56.8263	57.7875	58.1812	58.2423	58.7302	58.2211	58.9632	58.1996	58.9040	58.2683	59.1711	52.4017	53.2148	53.4251	53.7169	54.2285	53.6959	54.1637	53.6737	54.1399	53.7420	54.6001	**53.9**
**H2**	8.5316	8.4545	8.4879	8.3916	8.3798	8.3972	8.3696	8.4025	8.3605	8.3863	8.3500	8.5316	8.4545	8.4879	8.3916	8.3798	8.3972	8.3696	8.4025	8.3605	8.3863	8.3500	**8.13**
**H5**	7.1010	7.2667	7.3608	7.3363	7.4552	7.3324	7.4770	7.3285	7.4659	7.3404	7.4972	7.1010	7.2667	7.3608	7.3363	7.4552	7.3324	7.4770	7.3285	7.4659	7.3405	7.4972	**8.03**
**H6**	7.8264	8.0008	8.1059	8.0899	8.1973	8.0850	8.2380	8.0800	8.2146	8.0947	8.2627	7.8264	8.0008	8.1059	8.0899	8.1973	8.0850	8.2380	8.0800	8.2146	8.0948	8.2627	**7.4**
**H(3``A-3``C)**	2.3544	2.4012	2.4186	2.4152	2.4382	2.4143	2.4507	2.4135	2.4482	2.4165	2.4557	2.3544	2.4012	2.4186	2.4152	2.4382	2.4143	2.4507	2.4135	2.4481	2.4165	2.4557	**2.32**
**H8**	4.5787	4.6379	4.7146	4.6802	4.7383	4.6775	4.7988	4.6748	4.7764	4.6831	4.7847	4.5787	4.6379	4.7146	4.6802	4.7383	4.6775	4.7988	4.6748	4.7765	4.6831	4.7847	**5.64**
**H(9A-9B)**	1.6909	1.7338	1.7235	1.7575	1.7577	1.7566	1.7090	1.7555	1.7025	1.7588	1.7330	1.6909	1.7338	1.7235	1.7575	1.7578	1.7566	1.7090	1.7555	1.7025	1.7588	1.7330	**1.92**
**H(10A)**	1.0781	0.9592	0.9693	0.9078	0.8335	0.9117	0.8879	0.9156	0.8806	0.9042	0.8908	1.0781	0.9592	0.9693	0.9078	0.8335	0.9117	0.8879	0.9156	0.8806	0.9042	0.8909	**1.26**
**H(10B)**	1.7292	1.6230	1.5745	1.5490	1.4774	1.5533	1.4692	1.5576	1.4747	1.5455	1.4352	1.7292	1.6230	1.5745	1.5491	1.4774	1.5533	1.4692	1.5576	1.4748	1.5455	1.4353	**1.07**
**H(11A-11C)**	0.7494	0.7582	0.7437	0.7634	0.7279	0.7630	0.7298	0.7627	0.7288	0.7642	0.7171	0.7493	0.7582	0.7437	0.7634	0.7279	0.7630	0.7298	0.7627	0.7288	0.7642	0.7171	**0.78**
**H(1`A)**	2.6897	2.6816	2.6674	2.6745	2.6310	2.6748	2.6369	2.6753	2.6388	2.6745	2.6229	2.8111	2.8097	2.7940	2.7964	2.7732	2.7970	2.7869	2.7977	2.7919	2.7962	2.7601	**3.47**
**H(1`B)**	3.1902	3.2235	3.2584	3.2548	3.3141	3.2526	3.2902	3.2504	3.2844	3.2574	3.2904	2.8604	2.7056	2.6677	2.6758	2.6986	2.6761	2.6646	2.6768	2.6656	2.6761	2.6602	**3.63**
**H(4`A)**	2.8604	2.7056	2.6677	2.6758	2.6986	2.6761	2.6646	2.6768	2.6656	2.6761	2.6602	3.1902	3.2235	3.2584	3.2548	3.3141	3.2526	3.2903	3.2504	3.2844	3.2574	3.2904	**3.07**
**H(4`B)**	2.8111	2.8097	2.7940	2.7964	2.7733	2.7969	2.7869	2.7977	2.7919	2.7962	2.7601	2.6897	2.6816	2.6674	2.6745	2.6310	2.6749	2.6369	2.6753	2.6388	2.6745	2.6229	**3.25**
Nuclei	B972																						
**C1**	134.4281	135.1489	136.2116	136.2220	138.2141	136.1461	137.2162	136.0737	137.3801	136.2992	137.6722	134.4282	135.1488	136.2117	136.2220	138.2141	136.1461	137.2156	136.0736	137.3801	136.2993	137.6721	**131.615**
**C2**	137.0114	136.8759	137.3370	136.7029	137.2107	136.7149	137.5467	136.7241	137.4567	136.6860	137.5214	137.0114	136.8759	137.3372	136.7027	137.2107	136.7149	137.5468	136.7240	137.4567	136.6860	137.5214	**133.405**
**C3**	129.4761	130.6751	131.5774	131.1349	132.1706	131.1126	132.4963	131.0875	132.4150	131.1517	132.6733	129.4761	130.6751	131.5774	131.1350	132.1706	131.1126	132.4964	131.0874	132.4150	131.1518	132.6733	**126.19**
**C4**	169.3810	170.2839	170.9947	170.8307	171.3548	170.8062	172.0589	170.7779	171.8857	170.8484	172.1553	169.3810	170.2839	170.9946	170.8307	171.3548	170.8061	172.0590	170.7779	171.8857	170.8483	172.1552	**165.04**
**C5**	116.0208	117.1948	117.8247	117.9435	118.7106	117.8975	119.0111	117.8494	118.9013	117.9834	119.1617	116.0208	117.1948	117.8246	117.9434	118.7106	117.8975	119.0111	117.8494	118.9013	117.9834	119.1616	**116.425**
**C6**	129.7820	131.3511	132.1260	132.1091	133.1708	132.0629	133.9092	132.0131	133.6344	132.1456	134.1640	129.7819	131.3510	132.1261	132.1089	133.1708	132.0630	133.9095	132.0132	133.6344	132.1457	134.1639	**129.92**
**C7**	198.0969	202.4565	203.7760	205.5634	206.5521	205.3566	211.1615	205.1545	210.1947	205.7663	212.3969	198.0969	202.4566	203.7761	205.5632	206.5521	205.3566	211.1613	205.1545	210.1947	205.7664	212.3969	**195.9**
**C8**	63.3683	64.4415	64.9688	65.2435	66.2201	65.1775	66.6094	65.1144	66.5334	65.3088	67.0683	63.3683	64.4415	64.9688	65.2434	66.2201	65.1775	66.6089	65.1143	66.5334	65.3089	67.0683	**67.6**
**C9**	36.5430	36.2532	36.3741	36.1334	36.3972	36.1377	36.5148	36.1404	36.4525	36.1251	36.6258	36.5431	36.2532	36.3743	36.1333	36.3972	36.1378	36.5150	36.1405	36.4526	36.1252	36.6259	**32.2**
**C10**	18.7723	19.2439	19.4839	19.5650	20.0350	19.5449	19.9146	19.5224	19.8995	19.5801	20.0596	18.7724	19.2440	19.4840	19.5651	20.0350	19.5448	19.9146	19.5224	19.8995	19.5801	20.0597	**23.4**
**C11**	15.9213	16.0316	16.1329	16.1175	16.3056	16.1137	16.2484	16.1075	16.2495	16.1171	16.4222	15.9213	16.0316	16.1330	16.1176	16.3056	16.1137	16.2483	16.1075	16.2495	16.1170	16.4222	**17.9**
**C1`**	50.3924	51.2113	51.4065	51.7029	52.2259	51.6755	52.1413	51.6456	52.1125	51.7240	52.5743	54.6976	55.6987	56.0698	56.2264	56.6664	56.1964	56.8386	56.1643	56.7820	56.2507	57.0758	**52.4**
**C2`**	25.1067	25.4933	25.7434	25.7066	26.0791	25.6942	26.0912	25.6794	26.1035	25.7142	25.9753	24.6934	24.8150	25.2349	25.0281	25.5090	25.0192	25.4248	25.0071	25.4154	25.0316	25.2151	**14.5**
**C3`**	24.6934	24.8149	25.2350	25.0281	25.5090	25.0191	25.4253	25.0071	25.4154	25.0316	25.2150	25.1067	25.4933	25.7435	25.7066	26.0791	25.6942	26.0912	25.6794	26.1035	25.7142	25.9753	**14.2**
**C4`**	54.6976	55.6988	56.0698	56.2264	56.6664	56.1965	56.8384	56.1644	56.7820	56.2506	57.0758	50.3924	51.2113	51.4066	51.7028	52.2259	51.6755	52.1407	51.6457	52.1125	51.7239	52.5743	**53.9**
**H2**	8.5259	8.4359	8.4464	8.3267	8.2997	8.3351	8.3490	8.3425	8.3390	8.3176	8.3254	8.5259	8.4359	8.4464	8.3267	8.2997	8.3351	8.3490	8.3425	8.3390	8.3176	8.3253	**8.13**
**H5**	7.0970	7.2557	7.3422	7.3329	7.4529	7.3280	7.4745	7.3230	7.4633	7.3374	7.4960	7.0970	7.2557	7.3422	7.3329	7.4529	7.3280	7.4745	7.3230	7.4633	7.3374	7.4960	**8.03**
**H6**	7.8288	7.9988	8.0753	8.0672	8.1633	8.0638	8.2300	8.0599	8.2054	8.0700	8.2511	7.8288	7.9988	8.0753	8.0672	8.1633	8.0638	8.2300	8.0599	8.2054	8.0700	8.2511	**7.4**
**H(3``A-3``C)**	2.3521	2.3995	2.4188	2.4205	2.4384	2.4196	2.4486	2.4184	2.4454	2.4212	2.4549	2.3521	2.3995	2.4188	2.4205	2.4384	2.4196	2.4486	2.4184	2.4454	2.4212	2.4549	**2.32**
**H8**	4.5842	4.6361	4.6887	4.6705	4.7264	4.6688	4.7847	4.6667	4.7629	4.6718	4.7760	4.5842	4.6361	4.6887	4.6705	4.7264	4.6688	4.7847	4.6667	4.7629	4.6718	4.7760	**5.64**
**H(9A-9B)**	1.6515	1.6921	1.6557	1.6940	1.6871	1.6931	1.6861	1.6920	1.6797	1.6946	1.7088	1.6515	1.6921	1.6557	1.6940	1.6871	1.6931	1.6861	1.6921	1.6797	1.6946	1.7088	**1.92**
**H(10A)**	1.0437	0.9397	0.9161	0.8857	0.8210	0.8903	0.8712	0.8943	0.8635	0.8810	0.8808	1.0437	0.9397	0.9161	0.8857	0.8210	0.8903	0.8712	0.8943	0.8635	0.8810	0.8808	**1.26**
**H(10B)**	1.7027	1.5952	1.5541	1.5289	1.4487	1.5333	1.4496	1.5372	1.4540	1.5245	1.4176	1.7027	1.5952	1.5541	1.5289	1.4487	1.5332	1.4496	1.5372	1.4540	1.5245	1.4176	**1.07**
**H(11A-11C)**	0.7425	0.7519	0.7330	0.7583	0.7190	0.7580	0.7275	0.7574	0.7261	0.7584	0.7156	0.7425	0.7519	0.7330	0.7583	0.7190	0.7580	0.7275	0.7575	0.7261	0.7584	0.7156	**0.78**
**H(1`A)**	2.6841	2.6726	2.6522	2.6602	2.6150	2.6610	2.6233	2.6616	2.6251	2.6593	2.6057	2.8082	2.8081	2.7978	2.8011	2.7744	2.8016	2.7817	2.8019	2.7866	2.8006	2.7566	**3.47**
**H(1`B)**	3.1705	3.2089	3.2370	3.2399	3.2976	3.2376	3.2792	3.2351	3.2739	3.2421	3.2814	2.8427	2.6855	2.6639	2.6745	2.7047	2.6736	2.6513	2.6730	2.6523	2.6758	2.6524	**3.63**
**H(4`A)**	2.8427	2.6855	2.6639	2.6745	2.7047	2.6736	2.6513	2.6730	2.6523	2.6758	2.6524	3.1705	3.2089	3.2370	3.2400	3.2976	3.2376	3.2792	3.2351	3.2739	3.2421	3.2814	**3.07**
**H(4`B)**	2.8082	2.8081	2.7978	2.8011	2.7744	2.8016	2.7818	2.8019	2.7866	2.8005	2.7566	2.6841	2.6726	2.6522	2.6602	2.6150	2.6610	2.6233	2.6616	2.6251	2.6593	2.6057	**3.25**

[a] methyl protons shifts are averaged

[b] according to [11]

Table S12. Cont. III

Nuclei	R											S											Exp. [b]
	BHandH																						
	Gas	$CHCl_3$ (PCM)	$CHCl_3$ (SMD)	DMSO (PCM)	DMSO (SMD)	CH_3OH (PCM)	CH_3OH (SMD)	C_2H_5OH (PCM)	C_2H_5OH (SMD)	H_2O (PCM)	H_2O (SMD)	Gas	$CHCl_3$ (PCM)	$CHCl_3$ (SMD)	DMSO (PCM)	DMSO (SMD)	CH_3OH (PCM)	CH_3OH (SMD)	C_2H_5OH (PCM)	C_2H_5OH (SMD)	H_2O (PCM)	H_2O (SMD)	
**C1**	138.5231	139.0089	140.0484	139.2169	140.8206	139.2009	140.0353	139.2020	139.8269	139.2012	140.3005	138.5231	139.0089	140.0483	139.2169	139.9610	139.2009	140.0352	139.2020	139.8269	139.2013	140.3005	**131.615**
**C2**	143.6560	143.4520	144.0173	143.2363	143.7038	143.2417	144.0998	143.2650	144.0438	143.2009	144.0901	143.6561	143.4520	144.0173	143.2363	143.9804	143.2416	144.0998	143.2650	144.0437	143.2008	144.0902	**133.405**
**C3**	133.9273	135.1849	136.1533	135.5744	136.6556	135.5459	136.9059	135.5348	136.8238	135.5710	137.0520	133.9274	135.1849	136.1534	135.5744	136.3185	135.5457	136.9059	135.5348	136.8240	135.5709	137.0520	**126.19**
**C4**	172.9977	173.8020	174.3871	174.2354	174.7534	174.1977	175.4712	174.1786	175.3400	174.2414	175.5908	172.9977	173.8020	174.3872	174.2354	174.9795	174.1976	175.4712	174.1786	175.3401	174.2414	175.5907	**165.04**
**C5**	120.8195	122.1800	122.8532	122.8946	123.7688	122.8410	123.9883	122.8058	123.8658	122.9153	124.1428	120.8194	122.1800	122.8534	122.8946	123.6387	122.8411	123.9885	122.8058	123.8658	122.9153	124.1427	**116.425**
**C6**	136.8177	138.5905	139.5296	139.6440	141.0709	139.5616	141.8010	139.4990	141.6438	139.6930	142.2005	136.8176	138.5905	139.5296	139.6440	140.3881	139.5618	141.8010	139.4990	141.6438	139.6931	142.2006	**129.92**
**C7**	204.3560	208.8294	210.2410	211.2214	211.8276	211.0726	217.1203	210.9402	215.5238	211.3317	218.2226	204.3564	208.8294	210.2410	211.2214	211.9655	211.0722	217.1203	210.9402	215.5237	211.3320	218.2225	**195.9**
**C8**	59.9710	61.1513	61.9676	61.6176	62.4581	61.5856	63.0398	61.5711	62.7043	61.6170	63.4925	59.9711	61.1513	61.9677	61.6176	62.3617	61.5857	63.0397	61.5711	62.7043	61.6169	63.4926	**67.6**
**C9**	36.1990	35.9426	36.1735	35.7678	36.1538	35.7674	36.1778	35.7852	36.0766	35.7381	36.4154	36.1990	35.9426	36.1735	35.7678	36.5119	35.7674	36.1778	35.7852	36.0766	35.7381	36.4154	**32.2**
**C10**	16.4266	16.8265	17.2160	17.1627	17.6889	17.1304	17.5426	17.1165	17.5312	17.1634	17.7174	16.4266	16.8265	17.2160	17.1627	17.9068	17.1305	17.5426	17.1165	17.5311	17.1634	17.7174	**23.4**
**C11**	15.0818	15.1659	15.3458	15.2140	15.4221	15.2011	15.3850	15.2065	15.3937	15.1963	15.5738	15.0817	15.1659	15.3459	15.2140	15.9581	15.2012	15.3851	15.2065	15.3936	15.1963	15.5738	**17.9**
**C1`**	48.3052	49.0773	49.4876	49.5717	50.1422	49.5314	50.1232	49.5097	50.0703	49.5798	50.5682	52.1378	53.1666	53.5836	53.6420	54.3861	53.6044	54.3440	53.5850	54.2807	53.6476	54.6197	**52.4**
**C2`**	21.6445	22.0099	22.4106	22.1936	22.6486	22.1737	22.6993	22.1721	22.6448	22.1821	22.7065	21.0580	21.2399	21.5903	21.4125	22.1566	21.3904	21.8122	21.3868	21.8273	21.4037	21.7538	**14.5**
**C3`**	21.0579	21.2399	21.5903	21.4125	21.8971	21.3904	21.8122	21.3868	21.8273	21.4038	21.7539	21.6445	22.0099	22.4107	22.1936	22.9377	22.1737	22.6993	22.1721	22.6447	22.1821	22.7065	**14.2**
**C4`**	52.1378	53.1666	53.5836	53.6420	54.1928	53.6044	54.3439	53.5850	54.2807	53.6475	54.6198	48.3052	49.0773	49.4875	49.5717	50.3158	49.5315	50.1232	49.5097	50.0703	49.5798	50.5682	**53.9**
**H2**	8.8186	8.7294	8.7294	8.6790	8.6755	8.6823	8.6918	8.6850	8.7274	8.6770	8.6792	8.8186	8.7294	8.7294	8.6790	8.5978	8.6823	8.6918	8.6850	8.7274	8.6770	8.6792	**8.13**
**H5**	7.2358	7.4200	7.5085	7.5012	7.6284	7.4972	7.6456	7.4925	7.6367	7.5059	7.6679	7.2358	7.4200	7.5085	7.5012	7.4200	7.4972	7.6456	7.4925	7.6367	7.5059	7.6679	**8.03**
**H6**	8.0025	8.1826	8.2567	8.2840	8.4233	8.2779	8.4869	8.2710	8.4820	8.2910	8.5171	8.0025	8.1826	8.2567	8.2840	8.2028	8.2779	8.4869	8.2710	8.4820	8.2910	8.5171	**7.4**
**H(3``A-3``C)**	2.4320	2.4746	2.4883	2.4853	2.5047	2.4856	2.5112	2.4851	2.5092	2.4859	2.5191	2.4321	2.4746	2.4884	2.4853	2.4040	2.4856	2.5112	2.4851	2.5092	2.4859	2.5191	**2.32**
**H8**	4.2562	4.3350	4.3421	4.4012	4.4805	4.3969	4.5478	4.3919	4.5369	4.4065	4.5641	4.2562	4.3350	4.3421	4.4012	4.3200	4.3969	4.5478	4.3919	4.5370	4.4065	4.5641	**5.64**
**H(9A-9B)**	1.7454	1.7813	1.7762	1.8032	1.8093	1.8023	1.7945	1.8006	1.7948	1.8051	1.8317	1.7454	1.7813	1.7762	1.8032	1.7220	1.8023	1.7945	1.8006	1.7948	1.8051	1.8317	**1.92**
**H(10A)**	1.0219	0.8509	0.8037	0.7929	0.7154	0.7965	0.7431	0.7994	0.7742	0.7906	0.7455	1.0219	0.8510	0.8037	0.7929	0.7154	0.7965	0.7430	0.7994	0.7742	0.7906	0.7455	**1.26**
**H(10B)**	1.7102	1.6304	1.5538	1.5563	1.4590	1.5615	1.4536	1.5659	1.4657	1.5525	1.4241	1.7102	1.6304	1.5538	1.5563	1.4590	1.5615	1.4536	1.5659	1.4656	1.5525	1.4240	**1.07**
**H(11A-11C)**	0.7561	0.7663	0.7315	0.7725	0.7301	0.7727	0.7313	0.7721	0.7436	0.7735	0.7260	0.7561	0.7663	0.7315	0.7725	0.6913	0.7727	0.7314	0.7721	0.7436	0.7735	0.7260	**0.78**
**H(1`A)**	2.5684	2.5623	2.5390	2.5523	2.5016	2.5538	2.5187	2.5545	2.5185	2.5519	2.5002	2.7069	2.7131	2.7033	2.6971	2.6610	2.6993	2.6771	2.7006	2.6693	2.6960	2.6545	**3.47**
**H(1`B)**	3.0770	3.1058	3.1306	3.1328	3.1871	3.1315	3.1710	3.1295	3.1713	3.1351	3.1756	2.8942	2.6788	2.6485	2.6088	2.5995	2.6132	2.5398	2.6167	2.5165	2.6058	2.5311	**3.63**
**H(4`A)**	2.8942	2.6788	2.6485	2.6088	2.5995	2.6132	2.5398	2.6167	2.5165	2.6058	2.5311	3.0770	3.1058	3.1306	3.1328	3.1871	3.1315	3.1710	3.1295	3.1713	3.1351	3.1756	**3.07**
**H(4`B)**	2.7069	2.7131	2.7033	2.6971	2.6610	2.6993	2.6771	2.7006	2.6693	2.6960	2.6545	2.5684	2.5623	2.5390	2.5523	2.5016	2.5538	2.5187	2.5545	2.5185	2.5518	2.5002	**3.25**
Nuclei	BHandHLYP																						
**C1**	139.0935	139.5781	140.2537	139.8902	141.6619	139.8596	140.5194	139.8339	140.8006	139.9233	141.8788	139.0935	139.5781	140.2537	139.8902	141.6619	139.8595	140.5194	139.8338	140.8006	139.9233	141.8788	**131.615**
**C2**	142.3563	142.1689	142.5366	141.9714	142.4129	141.9866	142.7982	142.0039	142.7184	141.9560	142.7515	142.3564	142.1690	142.5366	141.9714	142.4129	141.9866	142.7982	142.0039	142.7184	141.9560	142.7517	**133.405**
**C3**	133.7017	134.7422	135.6058	135.0545	136.0378	135.0384	136.2323	135.0246	136.1984	135.0696	136.4859	133.7017	134.7423	135.6058	135.0546	136.0378	135.0386	136.2323	135.0246	136.1984	135.0696	136.4860	**126.19**
**C4**	173.0336	173.8142	174.4035	174.2646	174.7558	174.2348	175.5620	174.2081	175.3775	174.2931	175.6506	173.0337	173.8141	174.4035	174.2646	174.7558	174.2350	175.5620	174.2081	175.3775	174.2931	175.6505	**165.04**
**C5**	119.8627	121.0296	121.6010	121.6710	122.4508	121.6300	122.6183	121.5918	122.5543	121.7103	122.9242	119.8627	121.0295	121.6010	121.6708	122.4508	121.6299	122.6183	121.5918	122.5543	121.7103	122.9240	**116.425**
**C6**	135.3208	137.0210	138.0159	138.0138	139.1592	137.9580	140.0664	137.9030	139.7228	138.0642	139.7503	135.3208	137.0209	138.0159	138.0137	139.1592	137.9579	140.0664	137.9030	139.7228	138.0642	139.7503	**129.92**
**C7**	202.5936	207.3125	208.3313	209.9802	210.5430	209.8023	216.0223	209.6303	214.9167	210.1534	218.2154	202.5939	207.3124	208.3313	209.9806	210.5430	209.8024	216.0223	209.6303	214.9168	210.1534	218.2153	**195.9**
**C8**	61.7523	62.7483	63.0651	63.2236	64.0215	63.1928	64.3600	63.1649	64.3666	63.2529	65.0687	61.7523	62.7483	63.0651	63.2235	64.0215	63.1927	64.3600	63.1649	64.3665	63.2529	65.0687	**67.6**
**C9**	35.6300	35.2316	35.4893	35.0833	35.4232	35.0938	35.4144	35.1066	35.4013	35.0723	35.2899	35.6300	35.2317	35.4893	35.0833	35.4233	35.0939	35.4144	35.1066	35.4013	35.0723	35.2900	**32.2**
**C10**	18.2448	18.6433	18.9476	18.9323	19.4227	18.9152	19.2756	18.9001	19.2814	18.9479	19.3552	18.2448	18.6433	18.9476	18.9324	19.4228	18.9151	19.2756	18.9001	19.2814	18.9479	19.3552	**23.4**
**C11**	15.9367	16.0201	16.1876	16.0982	16.3194	16.0935	16.2590	16.0911	16.2725	16.1022	16.3945	15.9366	16.0201	16.1876	16.0982	16.3194	16.0935	16.2590	16.0911	16.2725	16.1022	16.3945	**17.9**
**C1`**	49.1257	49.9121	50.1571	50.4178	50.9480	50.3871	50.8730	50.3583	50.8725	50.4463	51.3085	53.3300	54.2956	54.6152	54.7513	55.2096	54.7231	55.3704	54.6972	55.3230	54.7782	55.6537	**52.4**
**C2`**	24.4387	24.7726	25.0457	24.9270	25.3437	24.9175	25.3375	24.9105	25.3733	24.9356	25.2057	23.9114	23.9971	24.3946	24.1330	24.6541	24.1226	24.5293	24.1152	24.5516	24.1429	24.4128	**14.5**
**C3`**	23.9114	23.9971	24.3946	24.1330	24.6541	24.1226	24.5293	24.1152	24.5516	24.1429	24.4128	24.4386	24.7726	25.0457	24.9271	25.3437	24.9175	25.3375	24.9105	25.3732	24.9356	25.2058	**14.2**
**C4`**	53.3300	54.2956	54.6152	54.7513	55.2096	54.7231	55.3704	54.6972	55.3230	54.7782	55.6536	49.1256	49.9120	50.1571	50.4177	50.9480	50.3871	50.8730	50.3583	50.8726	50.4463	51.3085	**53.9**
**H2**	8.7296	8.6465	8.6812	8.5873	8.5827	8.5924	8.6264	8.5972	8.6101	8.5820	8.5233	8.7296	8.6465	8.6812	8.5873	8.5827	8.5924	8.6264	8.5972	8.6101	8.5820	8.5233	**8.13**
**H5**	7.2306	7.3990	7.4904	7.4721	7.5889	7.4682	7.6079	7.4642	7.5955	7.4757	7.6302	7.2306	7.3990	7.4904	7.4721	7.5889	7.4682	7.6079	7.4642	7.5955	7.4757	7.6302	**8.03**
**H6**	7.8857	8.0768	8.1789	8.1841	8.3094	8.1779	8.3948	8.1715	8.3600	8.1899	8.3735	7.8857	8.0768	8.1789	8.1841	8.3094	8.1779	8.3948	8.1715	8.3600	8.1899	8.3735	**7.4**
**H(3``A-3``C)**	2.4202	2.4603	2.4730	2.4732	2.4898	2.4727	2.4996	2.4721	2.4965	2.4736	2.5089	2.4202	2.4602	2.4730	2.4732	2.4898	2.4727	2.4996	2.4721	2.4964	2.4736	2.5089	**2.32**
**H8**	4.1992	4.2886	4.3623	4.3556	4.4381	4.3512	4.5126	4.3468	4.4802	4.3597	4.4840	4.1992	4.2886	4.3623	4.3556	4.4381	4.3512	4.5126	4.3468	4.4802	4.3597	4.4840	**5.64**
**H(9A-9B)**	1.5520	1.5987	1.5909	1.6324	1.6380	1.6311	1.6370	1.6294	1.6279	1.6334	1.5981	1.5520	1.5987	1.5909	1.6324	1.6380	1.6311	1.6370	1.6294	1.6279	1.6334	1.5982	**1.92**
**H(10A)**	1.0619	0.9158	0.9261	0.8642	0.7859	0.8678	0.8336	0.8711	0.8214	0.8608	0.8369	1.0619	0.9158	0.9261	0.8642	0.7859	0.8677	0.8336	0.8711	0.8214	0.8608	0.8369	**1.26**
**H(10B)**	1.6379	1.5445	1.4942	1.4735	1.3984	1.4781	1.4094	1.4826	1.4104	1.4692	1.3665	1.6379	1.5446	1.4942	1.4735	1.3984	1.4781	1.4094	1.4826	1.4104	1.4692	1.3665	**1.07**
**H(11A-11C)**	0.7728	0.7821	0.7651	0.7906	0.7526	0.7902	0.7666	0.7897	0.7611	0.7909	0.7450	0.7728	0.7821	0.7651	0.7906	0.7526	0.7902	0.7666	0.7897	0.7611	0.7909	0.7450	**0.78**
**H(1`A)**	2.5918	2.5834	2.5679	2.5764	2.5262	2.5770	2.5437	2.5775	2.5431	2.5758	2.5237	2.7263	2.7196	2.6936	2.6997	2.6675	2.7012	2.6769	2.7025	2.6838	2.6984	2.6637	**3.47**
**H(1`B)**	3.0055	3.0567	3.0846	3.0964	3.1566	3.0940	3.1482	3.0914	3.1395	3.0987	3.1421	2.7070	2.5447	2.5125	2.5163	2.5371	2.5171	2.4767	2.5179	2.4831	2.5158	2.5100	**3.63**
**H(4`A)**	2.7070	2.5447	2.5125	2.5163	2.5371	2.5171	2.4767	2.5179	2.4831	2.5158	2.5100	3.0055	3.0567	3.0846	3.0964	3.1566	3.0940	3.1482	3.0914	3.1395	3.0987	3.1421	**3.07**
**H(4`B)**	2.7263	2.7196	2.6936	2.6998	2.6675	2.7012	2.6769	2.7025	2.6838	2.6984	2.6637	2.5918	2.5834	2.5679	2.5764	2.5262	2.5770	2.5437	2.5775	2.5431	2.5758	2.5237	**3.25**

[a] methyl protons shifts are averaged

[b] according to [11]

Table S12. Cont. IV

Nuclei	R											S											Exp. [b]
	BLYP																						
	Gas	$CHCl_3$ (PCM)	$CHCl_3$ (SMD)	DMSO (PCM)	DMSO (SMD)	CH_3OH (PCM)	CH_3OH (SMD)	C_2H_5OH (PCM)	C_2H_5OH (SMD)	H_2O (PCM)	H_2O (SMD)	Gas	$CHCl_3$ (PCM)	$CHCl_3$ (SMD)	DMSO (PCM)	DMSO (SMD)	CH_3OH (PCM)	CH_3OH (SMD)	C_2H_5OH (PCM)	C_2H_5OH (SMD)	H_2O (PCM)	H_2O (SMD)	
**C1**	139.1023	139.8307	140.7373	140.7483	142.9806	140.9580	141.8737	140.7116	142.2899	141.1834	142.3558	139.1023	139.8307	140.7373	140.7483	142.9805	140.9580	141.8737	140.7116	142.2899	141.1834	142.3558	**131.615**
**C2**	138.3075	138.1046	138.6118	137.9924	138.6335	138.2746	138.8389	138.0957	138.9199	138.3526	138.9335	138.3075	138.1046	138.6118	137.9924	138.6336	138.2746	138.8389	138.0957	138.9198	138.3526	138.9335	**133.405**
**C3**	133.6207	134.8133	135.6698	135.1959	136.2533	135.4544	136.4909	135.2509	136.5884	135.5774	136.7451	133.6207	134.8133	135.6698	135.1959	136.2533	135.4544	136.4909	135.2509	136.5885	135.5774	136.7451	**126.19**
**C4**	177.0599	178.2527	178.9276	178.7632	179.5034	179.0167	179.9651	178.8071	179.9764	179.1485	180.1703	177.0599	178.2527	178.9276	178.7632	179.5034	179.0167	179.9651	178.8071	179.9765	179.1485	180.1703	**165.04**
**C5**	117.8710	119.0826	119.6048	119.7418	120.4829	119.9793	120.6509	119.7551	120.7215	120.1438	120.8583	117.8710	119.0826	119.6048	119.7418	120.4829	119.9793	120.6510	119.7551	120.7215	120.1438	120.8583	**116.425**
**C6**	131.3114	132.8151	133.6851	133.6401	134.8688	133.8647	135.4234	133.6268	135.3290	134.0528	135.8245	131.3114	132.8151	133.6851	133.6401	134.8688	133.8647	135.4234	133.6268	135.3290	134.0528	135.8245	**129.92**
**C7**	204.2394	208.5677	209.9137	211.3715	212.8671	211.4664	216.9166	211.1043	216.3391	211.9138	218.1791	204.2394	208.5677	209.9138	211.3715	212.8672	211.4664	216.9167	211.1043	216.3392	211.9138	218.1791	**195.9**
**C8**	70.2995	71.2192	71.9656	71.9524	73.3942	72.1812	73.6457	71.9488	73.7815	72.3627	74.1224	70.2995	71.2192	71.9655	71.9524	73.3941	72.1812	73.6457	71.9488	73.7815	72.3627	74.1224	**67.6**
**C9**	41.8145	41.2997	41.5414	41.0759	41.4499	41.3675	41.5417	41.1963	41.6358	41.4252	41.7008	41.8145	41.2997	41.5414	41.0759	41.4499	41.3675	41.5417	41.1963	41.6358	41.4252	41.7008	**32.2**
**C10**	21.7311	22.1582	22.4351	22.3999	23.0339	22.6624	22.7506	22.4627	22.9244	22.7768	22.9351	21.7311	22.1582	22.4351	22.3999	23.0339	22.6624	22.7506	22.4627	22.9244	22.7768	22.9351	**23.4**
**C11**	16.4195	16.5063	16.6282	16.5371	16.8576	16.8136	16.6301	16.6280	16.8180	16.9014	16.8962	16.4195	16.5063	16.6282	16.5371	16.8575	16.8136	16.6301	16.6280	16.8180	16.9014	16.8962	**17.9**
**C1`**	55.3279	56.0908	56.3397	56.5755	57.2738	56.8226	57.0693	56.6074	57.2059	56.9677	57.5947	60.0410	61.2268	61.5342	61.7120	62.2350	61.9623	62.2824	61.7507	62.3998	62.1014	62.6294	**52.4**
**C2`**	27.6834	28.1709	28.3699	28.3979	28.8588	28.6638	28.7555	28.4673	28.9424	28.7714	28.7790	27.5948	27.8034	28.0882	27.9527	28.5247	28.2184	28.2541	28.0231	28.4320	28.3288	28.1366	**14.5**
**C3`**	27.5948	27.8034	28.0882	27.9527	28.5247	28.2184	28.2542	28.0231	28.4322	28.3288	28.1366	27.6834	28.1709	28.3699	28.3978	28.8587	28.6638	28.7554	28.4673	28.9424	28.7714	28.7790	**14.2**
**C4`**	60.0410	61.2268	61.5342	61.7120	62.2350	61.9623	62.2824	61.7507	62.3997	62.1014	62.6294	55.3279	56.0908	56.3397	56.5755	57.2738	56.8226	57.0694	56.6074	57.2060	56.9677	57.5947	**53.9**
**H2**	8.3058	8.2281	8.2401	8.1366	8.1018	8.1909	8.3154	8.1521	8.1326	8.1848	8.3136	8.3058	8.2281	8.2401	8.1366	8.1018	8.1909	8.3154	8.1521	8.1326	8.1848	8.3136	**8.13**
**H5**	6.9499	7.0955	7.1758	7.1650	7.2815	7.2079	7.4693	7.1584	7.2922	7.2246	7.5014	6.9499	7.0955	7.1758	7.1650	7.2815	7.2079	7.4693	7.1584	7.2922	7.2246	7.5014	**8.03**
**H6**	7.6665	7.8440	7.9011	7.9016	7.9802	7.9460	8.2096	7.8977	8.0180	7.9595	8.2500	7.6665	7.8440	7.9011	7.9016	7.9802	7.9460	8.2096	7.8977	8.0180	7.9595	8.2500	**7.4**
**H(3``A-3``C)**	2.3207	2.3668	2.3823	2.3807	2.4030	2.4276	2.5792	2.3819	2.4090	2.4364	2.5972	2.3207	2.3668	2.3823	2.3807	2.4030	2.4276	2.5792	2.3819	2.4090	2.4364	2.5972	**2.32**
**H8**	4.9473	4.9949	5.0049	5.0048	5.0360	5.0513	5.2383	5.0054	5.0590	5.0611	5.2389	4.9473	4.9949	5.0049	5.0048	5.0360	5.0513	5.2383	5.0054	5.0590	5.0611	5.2389	**5.64**
**H(9A-9B)**	1.7415	1.7722	1.7758	1.7885	1.8089	1.8350	1.9786	1.7887	1.8016	1.8442	2.0160	1.7415	1.7722	1.7758	1.7885	1.8089	1.8350	1.9786	1.7887	1.8016	1.8442	2.0160	**1.92**
**H(10A)**	1.1227	1.0569	1.0061	1.0073	0.9252	1.0574	1.1442	1.0151	0.9716	1.0602	1.1702	1.1227	1.0569	1.0061	1.0073	0.9252	1.0574	1.1442	1.0151	0.9715	1.0602	1.1702	**1.26**
**H(10B)**	1.8379	1.7168	1.6704	1.6399	1.5669	1.6918	1.7280	1.6512	1.5671	1.6908	1.7041	1.8379	1.7168	1.6704	1.6399	1.5669	1.6918	1.7280	1.6512	1.5671	1.6908	1.7041	**1.07**
**H(11A-11C)**	0.7854	0.7956	0.7708	0.7998	0.7647	0.8468	0.9361	0.8013	0.7678	0.8555	0.9392	0.7854	0.7956	0.7708	0.7998	0.7647	0.8468	0.9361	0.8013	0.7678	0.8555	0.9392	**0.78**
**H(1`A)**	2.8373	2.8287	2.8039	2.8107	2.7636	2.8595	2.9351	2.8157	2.7713	2.8646	2.9267	2.9507	2.9504	2.9346	2.9383	2.9253	2.9865	3.0934	2.9421	2.9337	2.9929	3.0779	**3.47**
**H(1`B)**	3.3462	3.3752	3.4165	3.3990	3.4767	3.4445	3.6175	3.3975	3.4452	3.4561	3.6333	3.0201	2.8340	2.8312	2.8107	2.8476	2.8583	2.9695	2.8138	2.8063	2.8661	2.9770	**3.63**
**H(4`A)**	3.0201	2.8340	2.8312	2.8107	2.8476	2.8583	2.9695	2.8138	2.8063	2.8661	2.9770	3.3462	3.3752	3.4165	3.3990	3.4767	3.4445	3.6175	3.3975	3.4452	3.4561	3.6333	**3.07**
**H(4`B)**	2.9507	2.9504	2.9346	2.9383	2.9253	2.9865	3.0934	2.9421	2.9337	2.9929	3.0779	2.8373	2.8287	2.8039	2.8107	2.7636	2.8595	2.9351	2.8157	2.7713	2.8646	2.9267	**3.25**
Nuclei	BMK																						
**C1**	154.5953	155.0700	155.8393	155.2298	157.2579	155.2045	156.2680	155.3907	156.1737	155.4160	156.4508	154.5954	155.0700	155.8393	155.2298	157.2579	155.2045	156.2681	155.3907	156.1735	155.4158	156.4507	**131.615**
**C2**	156.2832	155.7767	156.4349	155.4521	156.1769	155.4582	156.4567	155.6756	156.3051	155.6074	156.4481	156.2832	155.7768	156.4349	155.4521	156.1769	155.4582	156.4566	155.6754	156.3051	155.6075	156.4481	**133.405**
**C3**	148.5728	149.6892	150.7410	149.9666	151.2428	149.9389	151.4493	150.1218	151.2931	150.1539	151.5252	148.5729	149.6892	150.7410	149.9666	151.2428	149.9389	151.4492	150.1218	151.2931	150.1539	151.5254	**126.19**
**C4**	188.0443	188.7213	189.5032	189.1026	189.9730	189.0631	190.6451	189.2349	190.3564	189.3020	190.7229	188.0442	188.7214	189.5032	189.1026	189.9730	189.0631	190.6451	189.2350	190.3564	189.3021	190.7229	**165.04**
**C5**	132.7638	134.0032	134.7389	134.6531	135.7470	134.5975	135.9333	134.7532	135.7072	134.8674	136.0266	132.7638	134.0032	134.7389	134.6530	135.7470	134.5975	135.9333	134.7534	135.7072	134.8674	136.0266	**116.425**
**C6**	148.7418	150.5055	151.8640	151.5792	153.1718	151.4945	153.8956	151.6212	153.6229	151.8216	154.2581	148.7418	150.5055	151.8640	151.5792	153.1718	151.4945	153.8954	151.6211	153.6231	151.8215	154.2580	**129.92**
**C7**	218.4913	223.2471	224.4007	226.0069	226.9150	225.8185	232.9452	225.8419	231.3494	226.3487	234.0987	218.4914	223.2472	224.4007	226.0069	226.9150	225.8185	232.9453	225.8419	231.3490	226.3484	234.0986	**195.9**
**C8**	71.7640	72.8963	73.4172	73.4380	74.5797	73.3922	75.0619	73.5574	74.7021	73.6429	75.4106	71.7641	72.8963	73.4172	73.4380	74.5797	73.3922	75.0620	73.5575	74.7020	73.6430	75.4106	**67.6**
**C9**	40.3663	40.4399	40.6858	40.4164	41.1542	40.4043	41.1181	40.6033	40.9415	40.5893	41.3374	40.3664	40.4399	40.6857	40.4163	41.1542	40.4043	41.1181	40.6033	40.9416	40.5892	41.3374	**32.2**
**C10**	21.4559	21.8392	22.1200	22.0395	22.7228	22.0118	22.5067	22.1955	22.4010	22.2274	22.5108	21.4558	21.8393	22.1199	22.0395	22.7228	22.0118	22.5067	22.1954	22.4010	22.2275	22.5108	**23.4**
**C11**	17.3136	17.2802	17.5523	17.3092	17.6787	17.2897	17.5890	17.4818	17.5016	17.4897	17.7787	17.3136	17.2802	17.5523	17.3092	17.6787	17.2897	17.5890	17.4818	17.5018	17.4897	17.7787	**17.9**
**C1`**	56.3986	56.7890	57.3295	57.1702	57.7680	57.1275	57.7895	57.2969	57.6895	57.3725	58.0585	60.5768	61.0850	61.4703	61.4511	62.1299	61.4130	62.2472	61.5861	62.0826	61.6490	62.4507	**52.4**
**C2`**	27.8518	27.9355	28.2439	28.1074	28.7043	28.0813	28.6897	28.2665	28.5560	28.2938	28.6605	27.3065	27.4390	27.9737	27.4414	28.0369	27.4217	27.9671	27.6137	27.8963	27.6218	27.7496	**14.5**
**C3`**	27.3064	27.4391	27.9737	27.4414	28.0369	27.4217	27.9671	27.6138	27.8962	27.6218	27.7495	27.8516	27.9354	28.2439	28.1074	28.7043	28.0813	28.6897	28.2666	28.5559	28.2938	28.6605	**14.2**
**C4`**	60.5769	61.0850	61.4702	61.4511	62.1299	61.4130	62.2472	61.5860	62.0826	61.6489	62.4508	56.3986	56.7891	57.3294	57.1702	57.7680	57.1275	57.7895	57.2967	57.6896	57.3725	58.0586	**53.9**
**H2**	8.9131	8.8096	8.8517	8.7629	8.7532	8.7626	8.7834	8.7623	8.7889	8.7536	8.7896	8.9131	8.8096	8.8517	8.7629	8.7532	8.7626	8.7834	8.7622	8.7889	8.7536	8.7896	**8.13**
**H5**	7.4079	7.5890	7.6719	7.6719	7.7898	7.6640	7.8055	7.6560	7.7981	7.6698	7.8294	7.4079	7.5890	7.6718	7.6719	7.7898	7.6640	7.8055	7.6560	7.7981	7.6698	7.8294	**8.03**
**H6**	8.1831	8.3821	8.4746	8.5002	8.6259	8.4896	8.6994	8.4791	8.6792	8.5007	8.7274	8.1831	8.3821	8.4746	8.5002	8.6259	8.4896	8.6994	8.4791	8.6792	8.5007	8.7274	**7.4**
**H(3``A-3``C)**	2.3407	2.3986	2.4141	2.4216	2.4430	2.4172	2.4541	2.4128	2.4498	2.4163	2.4685	2.3407	2.3986	2.4141	2.4216	2.4430	2.4172	2.4541	2.4128	2.4498	2.4163	2.4685	**2.32**
**H8**	4.3983	4.4643	4.4754	4.5266	4.5805	4.5191	4.6391	4.5116	4.6226	4.5243	4.6404	4.3983	4.4643	4.4754	4.5266	4.5805	4.5191	4.6391	4.5116	4.6226	4.5243	4.6404	**5.64**
**H(9A-9B)**	1.5996	1.6604	1.6389	1.7168	1.7305	1.7102	1.7139	1.7036	1.7088	1.7136	1.7736	1.5996	1.6604	1.6389	1.7168	1.7305	1.7102	1.7139	1.7036	1.7088	1.7136	1.7735	**1.92**
**H(10A)**	1.0656	0.8935	0.9246	0.8365	0.7703	0.8363	0.8082	0.8361	0.8162	0.8272	0.8214	1.0656	0.8935	0.9246	0.8365	0.7703	0.8363	0.8082	0.8362	0.8162	0.8272	0.8215	**1.26**
**H(10B)**	1.6499	1.5451	1.4844	1.4770	1.3963	1.4783	1.3907	1.4796	1.3998	1.4661	1.3625	1.6499	1.5451	1.4844	1.4770	1.3963	1.4783	1.3907	1.4796	1.3998	1.4661	1.3624	**1.07**
**H(11A-11C)**	0.6958	0.6946	0.6838	0.7097	0.6666	0.7054	0.6721	0.7012	0.6762	0.7043	0.6737	0.6957	0.6946	0.6838	0.7097	0.6666	0.7054	0.6722	0.7012	0.6762	0.7043	0.6737	**0.78**
**H(1`A)**	2.6172	2.6232	2.6134	2.6228	2.5732	2.6197	2.5923	2.6167	2.5919	2.6162	2.5730	2.7391	2.7609	2.7194	2.7526	2.7093	2.7504	2.7260	2.7482	2.7287	2.7451	2.6960	**3.47**
**H(1`B)**	3.0581	3.0696	3.0873	3.1209	3.1634	3.1143	3.1589	3.1077	3.1538	3.1178	3.1781	2.7381	2.6001	2.5522	2.5588	2.5539	2.5574	2.5085	2.5561	2.5046	2.5506	2.4950	**3.63**
**H(4`A)**	2.7381	2.6001	2.5522	2.5588	2.5539	2.5574	2.5085	2.5561	2.5046	2.5506	2.4950	3.0582	3.0696	3.0873	3.1209	3.1634	3.1143	3.1589	3.1077	3.1538	3.1178	3.1781	**3.07**
**H(4`B)**	2.7391	2.7609	2.7194	2.7526	2.7093	2.7504	2.7259	2.7482	2.7287	2.7451	2.6960	2.6172	2.6232	2.6134	2.6228	2.5732	2.6197	2.5923	2.6166	2.5919	2.6162	2.5730	**3.25**

[a] methyl protons shifts are averaged

[b] according to [11]

Table S12. Cont. V

Nuclei	R											S											Exp. [b]
	BP86																						
	Gas	$CHCl_3$ (PCM)	$CHCl_3$ (SMD)	DMSO (PCM)	DMSO (SMD)	CH_3OH (PCM)	CH_3OH (SMD)	C_2H_5OH (PCM)	C_2H_5OH (SMD)	H_2O (PCM)	H_2O (SMD)	Gas	$CHCl_3$ (PCM)	$CHCl_3$ (SMD)	DMSO (PCM)	DMSO (SMD)	CH_3OH (PCM)	CH_3OH (SMD)	C_2H_5OH (PCM)	C_2H_5OH (SMD)	H_2O (PCM)	H_2O (SMD)	
**C1**	135.8495	136.6689	137.3066	137.1648	138.8647	137.1188	138.8268	137.0731	138.9655	137.2099	139.3922	135.8495	136.6688	137.3066	137.1648	138.8647	137.1188	138.8267	137.0731	138.9655	137.2099	139.3922	**131.615**
**C2**	137.1580	137.1273	137.4634	136.8237	137.3944	136.8545	137.6705	136.8798	137.5720	136.7892	137.8095	137.1580	137.1274	137.4634	136.8237	137.3944	136.8545	137.6706	136.8798	137.5720	136.7892	137.8095	**133.405**
**C3**	131.2546	132.7135	133.6445	133.1012	134.1399	133.0900	134.5922	133.0730	134.4864	133.1064	134.9261	131.2546	132.7136	133.6445	133.1012	134.1399	133.0900	134.5921	133.0730	134.4864	133.1064	134.9261	**126.19**
**C4**	173.5094	174.6598	175.4049	175.1708	175.8332	175.1449	176.6466	175.1141	176.4759	175.1906	176.9237	173.5094	174.6599	175.4049	175.1708	175.8332	175.1449	176.6466	175.1141	176.4760	175.1906	176.9237	**165.04**
**C5**	116.7347	118.0887	118.6556	118.7192	119.4601	118.6869	119.9173	118.6498	119.7898	118.7453	120.2057	116.7347	118.0886	118.6556	118.7192	119.4601	118.6869	119.9174	118.6498	119.7899	118.7453	120.2057	**116.425**
**C6**	130.0241	131.6968	132.6228	132.5377	133.8109	132.4973	134.1749	132.4511	133.9164	132.5700	134.6480	130.0241	131.6967	132.6228	132.5377	133.8109	132.4973	134.1752	132.4511	133.9164	132.5700	134.6480	**129.92**
**C7**	201.3531	205.4954	206.4738	207.8151	208.8001	207.6547	213.4912	207.4954	212.6871	207.9694	214.8552	201.3531	205.4954	206.4738	207.8151	208.8001	207.6547	213.4915	207.4954	212.6872	207.9694	214.8552	**195.9**
**C8**	67.9572	69.2530	69.5507	69.8540	70.8565	69.8146	71.4310	69.7729	71.3366	69.8900	72.1225	67.9572	69.2531	69.5508	69.8540	70.8565	69.8146	71.4310	69.7729	71.3366	69.8900	72.1225	**67.6**
**C9**	40.4637	40.2504	40.3612	40.0966	40.4368	40.1125	40.3996	40.1237	40.3132	40.0774	40.7261	40.4637	40.2504	40.3612	40.0966	40.4368	40.1125	40.3994	40.1237	40.3132	40.0774	40.7261	**32.2**
**C10**	20.2223	20.8053	20.9872	21.0456	21.4920	21.0383	21.3175	21.0264	21.2974	21.0476	21.6503	20.2223	20.8052	20.9872	21.0456	21.4920	21.0383	21.3175	21.0264	21.2975	21.0476	21.6503	**23.4**
**C11**	15.9623	16.2096	16.2346	16.2095	16.3481	16.2179	16.2556	16.2213	16.2579	16.1965	16.6174	15.9623	16.2096	16.2346	16.2095	16.3481	16.2179	16.2555	16.2213	16.2579	16.1965	16.6174	**17.9**
**C1`**	53.7829	54.7406	54.8451	55.1919	55.6558	55.1740	55.5814	55.1504	55.5503	55.2035	56.2153	58.2864	59.5681	59.9372	60.0034	60.3997	59.9861	60.7371	59.9633	60.6626	60.0145	61.1344	**52.4**
**C2`**	26.1951	26.8168	26.9812	26.9805	27.3139	26.9785	27.3480	26.9714	27.3473	26.9775	27.4367	25.9009	26.1838	26.5226	26.2878	26.7346	26.2875	26.7740	26.2823	26.7608	26.2833	26.6926	**14.5**
**C3`**	25.9009	26.1839	26.5227	26.2878	26.7346	26.2875	26.7741	26.2823	26.7609	26.2833	26.6926	26.1951	26.8168	26.9812	26.9805	27.3139	26.9785	27.3480	26.9714	27.3473	26.9775	27.4367	**14.2**
**C4`**	58.2864	59.5681	59.9371	60.0034	60.3997	59.9861	60.7371	59.9633	60.6626	60.0145	61.1344	53.7829	54.7406	54.8451	55.1919	55.6558	55.1740	55.5815	55.1504	55.5504	55.2035	56.2153	**53.9**
**H2**	8.3612	8.2860	8.3027	8.2214	8.2039	8.2281	8.1941	8.2340	8.1837	8.2142	8.1912	8.3612	8.2860	8.3027	8.2214	8.2039	8.2281	8.1941	8.2340	8.1837	8.2142	8.1912	**8.13**
**H5**	6.9613	7.1202	7.1998	7.1871	7.2969	7.1841	7.3205	7.1807	7.3088	7.1896	7.3540	6.9613	7.1202	7.1998	7.1871	7.2969	7.1841	7.3205	7.1807	7.3088	7.1896	7.3540	**8.03**
**H6**	7.7401	7.9051	7.9937	7.9859	8.0781	7.9824	8.1108	7.9784	8.0894	7.9886	8.1475	7.7401	7.9051	7.9937	7.9859	8.0781	7.9824	8.1108	7.9784	8.0893	7.9886	8.1475	**7.4**
**H(3``A-3``C)**	2.3125	2.3615	2.3688	2.3760	2.3904	2.3760	2.4024	2.3756	2.3987	2.3757	2.4220	2.3125	2.3615	2.3688	2.3760	2.3904	2.3760	2.4024	2.3756	2.3987	2.3757	2.4220	**2.32**
**H8**	4.9453	4.9831	5.0348	5.0075	5.0434	5.0070	5.0842	5.0061	5.0681	5.0074	5.0885	4.9453	4.9831	5.0348	5.0075	5.0434	5.0070	5.0842	5.0061	5.0681	5.0074	5.0885	**5.64**
**H(9A-9B)**	1.8372	1.8848	1.8480	1.9112	1.9166	1.9107	1.8542	1.9097	1.8466	1.9112	1.8997	1.8372	1.8848	1.8480	1.9112	1.9166	1.9107	1.8541	1.9097	1.8466	1.9112	1.8997	**1.92**
**H(10A)**	1.0845	1.0051	0.9907	0.9589	0.8776	0.9636	0.9342	0.9678	0.9268	0.9539	0.9592	1.0845	1.0051	0.9907	0.9589	0.8776	0.9636	0.9342	0.9678	0.9268	0.9539	0.9592	**1.26**
**H(10B)**	1.8693	1.7419	1.6809	1.6674	1.5836	1.6725	1.5678	1.6773	1.5735	1.6621	1.5408	1.8693	1.7419	1.6809	1.6674	1.5836	1.6725	1.5678	1.6773	1.5735	1.6621	1.5408	**1.07**
**H(11A-11C)**	0.7551	0.7684	0.7423	0.7735	0.7313	0.7740	0.7311	0.7743	0.7294	0.7727	0.7325	0.7551	0.7684	0.7423	0.7735	0.7313	0.7740	0.7311	0.7743	0.7294	0.7727	0.7325	**0.78**
**H(1`A)**	2.7902	2.7841	2.7553	2.7729	2.7233	2.7744	2.7188	2.7756	2.7209	2.7711	2.7166	2.8954	2.9111	2.8842	2.8996	2.8701	2.9010	2.8800	2.9021	2.8845	2.8980	2.8623	**3.47**
**H(1`B)**	3.3691	3.3961	3.4194	3.4227	3.4818	3.4216	3.4434	3.4202	3.4380	3.4234	3.4565	3.0749	2.8672	2.8241	2.8261	2.8371	2.8276	2.7866	2.8292	2.7876	2.8247	2.7945	**3.63**
**H(4`A)**	3.0749	2.8672	2.8241	2.8261	2.8371	2.8276	2.7866	2.8292	2.7876	2.8247	2.7945	3.3691	3.3961	3.4194	3.4227	3.4818	3.4216	3.4434	3.4202	3.4380	3.4234	3.4565	**3.07**
**H(4`B)**	2.8954	2.9111	2.8842	2.8996	2.8701	2.9010	2.8800	2.9021	2.8845	2.8980	2.8623	2.7902	2.7841	2.7553	2.7729	2.7233	2.7744	2.7188	2.7756	2.7209	2.7711	2.7166	**3.25**
Nuclei	**CAM-B3LYP**																						
**C1**	140.7673	141.4022	142.0344	141.9590	143.7829	141.8996	143.7179	141.8445	143.7932	142.0224	144.0912	140.7674	141.4023	142.0344	141.9591	143.7829	141.8996	143.7178	141.8445	143.7932	142.0223	144.0913	**131.615**
**C2**	142.8778	142.7805	143.1265	142.5784	143.0953	142.5986	143.4370	142.6183	143.3496	142.5609	143.4037	142.8778	142.7805	143.1265	142.5783	143.0953	142.5986	143.4371	142.6184	143.3496	142.5609	143.4036	**133.405**
**C3**	135.1922	136.3104	137.1668	136.6368	137.6759	136.6225	137.9967	136.6084	137.9332	136.6522	138.1491	135.1922	136.3103	137.1669	136.6367	137.6759	136.6225	137.9966	136.6085	137.9332	136.6522	138.1491	**126.19**
**C4**	176.1497	176.9796	177.5684	177.4121	177.9814	177.3875	178.6692	177.3634	178.5164	177.4375	178.7643	176.1496	176.9797	177.5685	177.4121	177.9815	177.3875	178.6692	177.3635	178.5164	177.4373	178.7645	**165.04**
**C5**	121.2550	122.4661	123.0200	123.1070	123.9183	123.0684	124.2249	123.0309	124.1273	123.1459	124.3710	121.2549	122.4660	123.0198	123.1070	123.9182	123.0684	124.2250	123.0308	124.1273	123.1459	124.3710	**116.425**
**C6**	135.7553	137.4800	138.4589	138.3653	139.6274	138.3258	139.8853	138.2856	139.6373	138.4039	140.1915	135.7552	137.4800	138.4589	138.3654	139.6275	138.3258	139.8854	138.2856	139.6374	138.4040	140.1916	**129.92**
**C7**	206.5296	211.2980	212.2868	214.1749	214.8891	213.9691	221.0968	213.7692	219.8941	214.3769	222.3800	206.5296	211.2982	212.2872	214.1747	214.8887	213.9691	221.0969	213.7691	219.8940	214.3768	222.3801	**195.9**
**C8**	64.4903	65.5933	65.9290	66.1598	67.0446	66.1216	67.7350	66.0854	67.5907	66.1999	68.2492	64.4903	65.5934	65.9290	66.1599	67.0447	66.1216	67.7350	66.0853	67.5907	66.1999	68.2491	**67.6**
**C9**	37.0107	36.6612	36.8601	36.4685	36.8137	36.4856	36.6654	36.5036	36.6098	36.4544	36.7796	37.0107	36.6613	36.8602	36.4684	36.8136	36.4856	36.6655	36.5034	36.6098	36.4544	36.7796	**32.2**
**C10**	18.6342	19.0887	19.3201	19.3569	19.8512	19.3462	19.6814	19.3357	19.6574	19.3687	19.8232	18.6342	19.0887	19.3202	19.3569	19.8512	19.3462	19.6814	19.3357	19.6575	19.3687	19.8231	**23.4**
**C11**	15.7228	15.8573	15.9562	15.9062	16.1315	15.9080	16.0611	15.9099	16.0546	15.9063	16.2365	15.7228	15.8573	15.9562	15.9063	16.1316	15.9080	16.0611	15.9098	16.0547	15.9063	16.2366	**17.9**
**C1`**	50.9019	51.7724	51.9853	52.2605	52.8135	52.2380	52.7664	52.2151	52.7311	52.2831	53.2215	55.1542	56.1982	56.4964	56.6552	57.1631	56.6311	57.3428	56.6073	57.2882	56.6802	57.6062	**52.4**
**C2`**	25.0134	25.3949	25.6401	25.5375	25.9761	25.5336	25.9790	25.5299	25.9871	25.5431	25.8650	24.4930	24.6381	24.9909	24.7688	25.3057	24.7623	25.2718	24.7562	25.2731	24.7772	25.0593	**14.5**
**C3`**	24.4930	24.6382	24.9909	24.7688	25.3058	24.7623	25.2717	24.7562	25.2731	24.7773	25.0592	25.0134	25.3948	25.6401	25.5376	25.9760	25.5336	25.9789	25.5300	25.9870	25.5432	25.8650	**14.2**
**C4`**	55.1542	56.1982	56.4963	56.6552	57.1631	56.6311	57.3430	56.6074	57.2881	56.6802	57.6063	50.9020	51.7725	51.9853	52.2605	52.8135	52.2380	52.7664	52.2150	52.7311	52.2830	53.2215	**53.9**
**H2**	8.6290	8.5436	8.5812	8.4642	8.4628	8.4720	8.4221	8.4795	8.4223	8.4563	8.4006	8.6289	8.5436	8.5812	8.4642	8.4628	8.4720	8.4221	8.4795	8.4223	8.4563	8.4006	**8.13**
**H5**	7.2013	7.3670	7.4587	7.4362	7.5563	7.4325	7.5731	7.4289	7.5625	7.4397	7.5936	7.2013	7.3670	7.4587	7.4362	7.5563	7.4325	7.5731	7.4289	7.5625	7.4397	7.5936	**8.03**
**H6**	7.8575	8.0381	8.1443	8.1298	8.2551	8.1251	8.2805	8.1205	8.2606	8.1341	8.3042	7.8575	8.0381	8.1443	8.1298	8.2551	8.1251	8.2805	8.1205	8.2606	8.1342	8.3042	**7.4**
**H(3``A-3``C)**	2.3848	2.4269	2.4415	2.4403	2.4617	2.4396	2.4708	2.4390	2.4686	2.4410	2.4765	2.3848	2.4269	2.4415	2.4404	2.4617	2.4396	2.4708	2.4390	2.4686	2.4409	2.4765	**2.32**
**H8**	4.3943	4.4695	4.5467	4.5250	4.6080	4.5214	4.6578	4.5180	4.6362	4.5284	4.6464	4.3943	4.4695	4.5467	4.5250	4.6079	4.5214	4.6578	4.5179	4.6362	4.5284	4.6464	**5.64**
**H(9A-9B)**	1.6413	1.6850	1.6756	1.7081	1.7169	1.7079	1.6565	1.7079	1.6509	1.7079	1.6853	1.6413	1.6850	1.6756	1.7081	1.7169	1.7079	1.6565	1.7078	1.6509	1.7079	1.6853	**1.92**
**H(10A)**	1.0475	0.9231	0.9283	0.8699	0.8001	0.8740	0.8544	0.8780	0.8489	0.8657	0.8533	1.0475	0.9231	0.9283	0.9814	0.8001	0.8740	0.8544	0.8781	0.8490	0.8657	0.8533	**1.26**
**H(10B)**	1.6969	1.5965	1.5475	1.5235	1.4519	1.5279	1.4416	1.5325	1.4479	1.5193	1.4118	1.6969	1.5965	1.5475	1.6006	1.4519	1.5279	1.4416	1.5325	1.4479	1.5193	1.4118	**1.07**
**H(11A-11C)**	0.7816	0.7916	0.7753	0.7965	0.7637	0.7964	0.7619	0.7964	0.7624	0.7966	0.7501	0.7816	0.7916	0.7753	0.7965	0.7637	0.7964	0.7619	0.7964	0.7624	0.7966	0.7501	**0.78**
**H(1`A)**	2.6925	2.6852	2.6697	2.6786	2.6330	2.6792	2.6420	2.6798	2.6428	2.6780	2.6235	2.8184	2.8149	2.7940	2.8471	2.7730	2.7999	2.7883	2.8009	2.7915	2.7980	2.6235	**3.47**
**H(1`B)**	3.1130	3.1575	3.1861	3.1924	3.2560	3.1902	3.2309	3.1881	3.2249	3.1944	3.2361	2.8046	2.6373	2.5959	2.6490	2.6281	2.6158	2.6015	2.6156	2.5956	2.6169	3.2361	**3.63**
**H(4`A)**	2.8046	2.6373	2.5959	2.6163	2.6281	2.6158	2.6015	2.6156	2.5956	2.6170	2.6006	3.1130	3.1576	3.1860	3.2391	3.2560	3.1902	3.2309	3.1881	3.2249	3.1944	2.6006	**3.07**
**H(4`B)**	2.8183	2.8149	2.7940	2.7989	2.7730	2.7999	2.7883	2.8009	2.7915	2.7980	2.7605	2.6925	2.6852	2.6697	2.7228	2.6330	2.6792	2.6419	2.6798	2.6428	2.6780	2.7605	**3.25**

[a] methyl protons shifts are averaged
[b] according to [11]

Table S12. Cont. VI

Nuclei	R											S											Exp. [b]
	HF																						
	Gas	$CHCl_3$ (PCM)	$CHCl_3$ (SMD)	DMSO (PCM)	DMSO (SMD)	CH_3OH (PCM)	CH_3OH (SMD)	C_2H_5OH (PCM)	C_2H_5OH (SMD)	H_2O (PCM)	H_2O (SMD)	Gas	$CHCl_3$ (PCM)	$CHCl_3$ (SMD)	DMSO (PCM)	DMSO (SMD)	CH_3OH (PCM)	CH_3OH (SMD)	C_2H_5OH (PCM)	C_2H_5OH (SMD)	H_2O (PCM)	H_2O (SMD)	
**C1**	136.7700	137.4506	138.5121	138.3885	140.2679	138.3305	138.9667	138.2848	139.3491	138.4603	139.4218	136.7699	137.4506	138.5121	138.3877	140.2679	138.3303	138.9666	138.2848	139.3492	138.4603	139.4218	**131.615**
**C2**	143.5411	143.2428	143.6356	142.9366	143.2603	142.9589	143.7604	142.9792	143.6162	142.9118	143.7036	143.5409	143.2428	143.6356	142.9368	143.2603	142.9589	143.7601	142.9792	143.6164	142.9115	143.7036	**133.405**
**C3**	131.0776	132.0180	132.8003	132.3447	133.3025	132.3299	133.2728	132.3173	133.3095	132.3610	133.4360	131.0776	132.0180	132.8003	132.3446	133.3025	132.3299	133.2727	132.3173	133.3094	132.3610	133.4360	**126.19**
**C4**	169.3417	169.9108	170.3885	170.1367	170.4612	170.1240	171.2854	170.1102	171.0491	170.1454	171.3430	169.3418	169.9108	170.3885	170.1367	170.4612	170.1239	171.2854	170.1102	171.0490	170.1458	171.3430	**165.04**
**C5**	118.0916	119.2727	119.8232	119.9283	120.7251	119.8894	120.7243	119.8523	120.7011	119.9662	120.8996	118.0915	119.2727	119.8232	119.9282	120.7251	119.8894	120.7243	119.8522	120.7010	119.9664	120.8996	**116.425**
**C6**	136.5481	138.0556	138.7096	138.6500	139.6108	138.6082	140.6638	138.5609	140.2241	138.6798	140.7970	136.5481	138.0555	138.7096	138.6504	139.6108	138.6082	140.6638	138.5609	140.2242	138.6797	140.7970	**129.92**
**C7**	196.8686	202.2947	203.6023	205.8404	206.0147	205.6200	212.7906	205.4118	211.4247	206.0655	214.1018	196.8685	202.2947	203.6023	205.8398	206.0147	205.6197	212.7908	205.4118	211.4242	206.0652	214.1018	**195.9**
**C8**	55.6369	56.6278	56.9786	57.2050	57.9480	57.1651	58.5084	57.1281	58.4969	57.2477	58.8852	55.6369	56.6278	56.9786	57.2053	57.9480	57.1650	58.5085	57.1281	58.4969	57.2476	58.8852	**67.6**
**C9**	31.1525	30.6520	30.7940	30.3781	30.6671	30.3933	30.6402	30.4071	30.6304	30.3613	30.6525	31.1525	30.6520	30.7940	30.3784	30.6671	30.3933	30.6401	30.4072	30.6304	30.3614	30.6525	**32.2**
**C10**	16.6895	17.0661	17.3193	17.2668	17.7688	17.2555	17.6589	17.2435	17.6444	17.2758	17.7397	16.6895	17.0662	17.3193	17.2670	17.7688	17.2554	17.6588	17.2435	17.6444	17.2759	17.7397	**23.4**
**C11**	15.7952	15.8252	15.9804	15.8538	16.0978	15.8522	16.0508	15.8509	16.0503	15.8545	16.1727	15.7951	15.8252	15.9804	15.8538	16.0978	15.8522	16.0508	15.8510	16.0503	15.8544	16.1727	**17.9**
**C1`**	44.7153	45.4285	45.6742	45.8487	46.3788	45.8246	46.3455	45.8008	46.3209	45.8705	46.7024	48.5690	49.4218	49.7058	49.8232	50.2869	49.7992	50.3683	49.7759	50.3294	49.8459	50.6024	**52.4**
**C2`**	22.8507	23.0851	23.3369	23.1460	23.5864	23.1416	23.5884	23.1375	23.6079	23.1492	23.4291	22.2077	22.2518	22.6057	22.3581	22.8846	22.3510	22.7464	22.3449	22.7511	22.3652	22.5856	**14.5**
**C3`**	22.2078	22.2518	22.6057	22.3583	22.8846	22.3510	22.7464	22.3450	22.7511	22.3652	22.5856	22.8507	23.0851	23.3369	23.1461	23.5864	23.1416	23.5885	23.1375	23.6079	23.1492	23.4291	**14.2**
**C4`**	48.5690	49.4218	49.7058	49.8232	50.2869	49.7992	50.3682	49.7759	50.3294	49.8458	50.6024	44.7153	45.4285	45.6742	45.8486	46.3788	45.8245	46.3456	45.8007	46.3209	45.8704	46.7024	**53.9**
**H2**	8.8665	8.7438	8.7622	8.6084	8.6166	8.6170	8.6556	8.6241	8.6319	8.5985	8.6261	8.8665	8.7438	8.7622	8.6085	8.6166	8.6170	8.6556	8.6241	8.6319	8.5985	8.6261	**8.13**
**H5**	7.2274	7.4088	7.4982	7.4886	7.6148	7.4840	7.6279	7.4792	7.6176	7.4930	7.6509	7.2274	7.4088	7.4982	7.4886	7.6148	7.4840	7.6279	7.4792	7.6176	7.4931	7.6509	**8.03**
**H6**	7.8778	8.0900	8.1831	8.1892	8.3218	8.1830	8.4016	8.1763	8.3605	8.1946	8.4236	7.8778	8.0900	8.1831	8.1892	8.3218	8.1830	8.4016	8.1763	8.3605	8.1946	8.4236	**7.4**
**H(3``A-3``C)**	2.4305	2.4725	2.4854	2.4847	2.4997	2.4841	2.5061	2.4834	2.5036	2.4852	2.5132	2.4305	2.4725	2.4854	2.4847	2.4997	2.4841	2.5061	2.4834	2.5036	2.4852	2.5132	**2.32**
**H8**	3.6213	3.7331	3.8135	3.8152	3.9205	3.8098	3.9903	3.8042	3.9529	3.8202	3.9867	3.6212	3.7331	3.8135	3.8152	3.9205	3.8098	3.9903	3.8042	3.9529	3.8202	3.9867	**5.64**
**H(9A-9B)**	1.2882	1.3245	1.2983	1.3195	1.3168	1.3196	1.3306	1.3186	1.3195	1.3186	1.3429	1.2882	1.3245	1.2983	1.3196	1.3168	1.3195	1.3306	1.3186	1.3195	1.3186	1.3429	**1.92**
**H(10A)**	1.0531	0.8619	0.8575	0.8056	0.7347	0.8089	0.7622	0.8123	0.7522	0.8027	0.7649	1.0531	0.8619	0.8575	0.8055	1.2668	0.8089	0.7622	0.8123	0.7522	0.8027	0.7649	**1.26**
**H(10B)**	1.4564	1.3727	1.3350	1.3045	1.2270	1.3085	1.2390	1.3123	1.2393	1.3007	1.2161	1.4564	1.3727	1.3350	1.3045	1.9186	1.3085	1.2389	1.3122	1.2393	1.3007	1.2161	**1.07**
**H(11A-11C)**	0.7568	0.7585	0.7391	0.7662	0.7252	0.7653	0.7329	0.7643	0.7291	0.7671	0.7249	0.7567	0.7585	0.7391	0.7661	0.7252	0.7653	0.7329	0.7644	0.7291	0.7671	0.7249	**0.78**
**H(1`A)**	2.3616	2.3539	2.3340	2.3444	2.2877	2.3452	2.3152	2.3457	2.3144	2.3438	2.2992	2.5162	2.5090	2.4837	2.4846	3.2397	2.4863	2.4642	2.4879	2.4710	2.4830	2.4445	**3.47**
**H(1`B)**	2.7076	2.7712	2.8004	2.8146	2.8730	2.8118	2.8748	2.8087	2.8650	2.8173	2.8833	2.3779	2.2663	2.2499	2.2622	3.0948	2.2620	2.2553	2.2620	2.2619	2.2629	2.2660	**3.63**
**H(4`A)**	2.3779	2.2664	2.2499	2.2621	2.2901	2.2620	2.2553	2.2620	2.2619	2.2629	2.2660	2.7076	2.7712	2.8004	2.8146	3.7227	2.8118	2.8748	2.8086	2.8650	2.8173	2.8833	**3.07**
**H(4`B)**	2.5162	2.5090	2.4837	2.4846	2.4435	2.4864	2.4642	2.4880	2.4709	2.4830	2.4445	2.3616	2.3539	2.3340	2.3444	3.0997	2.3452	2.3153	2.3457	2.3144	2.3438	2.2992	**3.25**
Nuclei	**LC-BLYP**																						
**C1**	144.3471	145.1746	146.1271	145.5455	147.4054	145.4985	147.8195	145.4808	146.3253	145.5740	148.0885	144.3471	145.1746	146.1271	145.5454	147.4054	145.4987	147.8195	145.4806	146.3253	145.5741	148.0885	**131.615**
**C2**	148.4754	148.2648	148.7467	148.0884	148.5602	148.0797	148.8570	148.0984	148.8914	148.0786	148.8342	148.4754	148.2648	148.7467	148.0883	148.5603	148.0798	148.8570	148.0985	148.8911	148.0786	148.8343	**133.405**
**C3**	138.7252	139.7530	140.6841	140.0785	141.1387	140.0411	141.4830	140.0306	141.2436	140.0964	141.6279	138.7252	139.7530	140.6843	140.0785	141.1388	140.0410	141.4830	140.0306	141.2435	140.0965	141.6281	**126.19**
**C4**	179.9222	180.5387	181.1389	180.9357	181.4004	180.8895	181.9408	180.8705	181.9657	180.9623	182.0533	179.9221	180.5386	181.1390	180.9356	181.4002	180.8894	181.9409	180.8706	181.9659	180.9623	182.0532	**165.04**
**C5**	125.8235	127.0707	127.7455	127.7575	128.6337	127.6939	128.9163	127.6577	128.6770	127.8007	129.0752	125.8235	127.0706	127.7455	127.7575	128.6337	127.6938	128.9164	127.6577	128.6771	127.8006	129.0751	**116.425**
**C6**	141.1115	142.7647	143.7145	143.8647	145.1527	143.7761	145.1332	143.7143	145.7000	143.9307	145.4752	141.1115	142.7647	143.7146	143.8647	145.1528	143.7762	145.1332	143.7143	145.6999	143.9306	145.4753	**129.92**
**C7**	212.2284	217.5525	218.8102	220.4161	221.0428	220.2164	228.4143	220.0453	225.3084	220.5896	229.5681	212.2284	217.5525	218.8102	220.4161	221.0427	220.2163	228.4143	220.0453	225.3083	220.5899	229.5681	**195.9**
**C8**	62.4068	63.5784	64.2070	64.1330	65.0092	64.0796	66.0646	64.0535	65.2574	64.1666	66.4450	62.4068	63.5784	64.2070	64.1329	65.0092	64.0796	66.0645	64.0535	65.2575	64.1666	66.4450	**67.6**
**C9**	34.8248	34.4400	34.6162	34.2810	34.5810	34.2731	34.3774	34.2918	34.5123	34.2704	34.4894	34.8248	34.4401	34.6163	34.2810	34.5810	34.2731	34.3775	34.2917	34.5123	34.2703	34.4893	**32.2**
**C10**	16.5280	16.8981	17.1967	17.2082	17.6860	17.1681	17.5187	17.1551	17.5105	17.2288	17.6525	16.5280	16.8981	17.1967	17.2082	17.6861	17.1681	17.5186	17.1550	17.5104	17.2288	17.6526	**23.4**
**C11**	14.7564	14.8096	14.9731	14.8900	15.0938	14.8638	15.0275	14.8647	15.0419	14.8976	15.2119	14.7564	14.8096	14.9731	14.8900	15.0938	14.8638	15.0275	14.8647	15.0420	14.8977	15.2118	**17.9**
**C1`**	48.8671	49.6225	49.9314	50.1222	50.6946	50.0688	50.7020	50.0428	50.6232	50.1557	51.1340	52.7212	53.6567	54.0446	54.1546	54.7052	54.1021	54.8613	54.0773	54.7563	54.1872	55.1516	**52.4**
**C2`**	23.0397	23.2914	23.6281	23.4380	23.8793	23.4085	23.9010	23.4061	23.8641	23.4488	23.7717	22.3704	22.4556	22.8366	22.5886	23.0727	22.5583	23.0331	22.5554	22.9648	22.6002	22.8703	**14.5**
**C3`**	22.3704	22.4557	22.8366	22.5886	23.0728	22.5583	23.0331	22.5555	22.9647	22.6002	22.8704	23.0397	23.2915	23.6282	23.4380	23.8794	23.4084	23.9009	23.4060	23.8640	23.4487	23.7717	**14.2**
**C4`**	52.7212	53.6566	54.0446	54.1546	54.7052	54.1021	54.8615	54.0774	54.7564	54.1873	55.1517	48.8671	49.6225	49.9314	50.1223	50.6945	50.0688	50.7021	50.0428	50.6233	50.1558	51.1340	**53.9**
**H2**	8.8092	8.6826	8.7074	8.6206	8.6143	8.6238	8.5161	8.6285	8.6608	8.6164	8.5091	8.8092	8.6826	8.7074	8.6206	8.6144	8.6238	8.5161	8.6285	8.6608	8.6164	8.5091	**8.13**
**H5**	7.3575	7.5268	7.6168	7.6041	7.7270	7.5988	7.7339	7.5950	7.7325	7.6081	7.7567	7.3575	7.5268	7.6168	7.6041	7.7270	7.5988	7.7339	7.5950	7.7325	7.6081	7.7567	**8.03**
**H6**	7.9955	8.1707	8.2717	8.2788	8.4132	8.2711	8.4015	8.2651	8.4670	8.2851	8.4323	7.9955	8.1707	8.2717	8.2788	8.4132	8.2711	8.4015	8.2651	8.4670	8.2851	8.4323	**7.4**
**H(3``A-3``C)**	2.4131	2.4510	2.4662	2.4646	2.4843	2.4630	2.4929	2.4630	2.4900	2.4651	2.5009	2.4131	2.4510	2.4662	2.4646	2.4843	2.4630	2.4929	2.4629	2.4900	2.4651	2.5009	**2.32**
**H8**	4.1886	4.2740	4.3471	4.3522	4.4413	4.3456	4.4823	4.3408	4.4969	4.3576	4.4948	4.1886	4.2740	4.3471	4.3522	4.4413	4.3456	4.4823	4.3408	4.4969	4.3576	4.4948	**5.64**
**H(9A-9B)**	1.6303	1.6632	1.6493	1.6916	1.6941	1.6894	1.6166	1.6887	1.6858	1.6926	1.6481	1.6303	1.6632	1.6493	1.6917	1.6941	1.6894	1.6166	1.6887	1.6858	1.6926	1.6480	**1.92**
**H(10A)**	0.9991	0.8346	0.8200	0.7845	0.7143	0.7862	0.7437	0.7896	0.7638	0.7819	0.7575	0.9991	0.8346	0.8200	0.7845	0.7143	0.7863	0.7437	0.7896	0.7638	0.7819	0.7575	**1.26**
**H(10B)**	1.6465	1.5484	1.4942	1.4756	1.3942	1.4794	1.3841	1.4847	1.4039	1.4711	1.3599	1.6465	1.5484	1.4942	1.4756	1.3942	1.4794	1.3841	1.4847	1.4039	1.4711	1.3599	**1.07**
**H(11A-11C)**	0.7782	0.7796	0.7577	0.7883	0.7502	0.7868	0.7401	0.7869	0.7596	0.7888	0.7348	0.7782	0.7796	0.7577	0.7884	0.7502	0.7868	0.7402	0.7869	0.7596	0.7888	0.7348	**0.78**
**H(1`A)**	2.6544	2.6487	2.6305	2.6418	2.5896	2.6417	2.6032	2.6431	2.6064	2.6410	2.5862	2.7818	2.7823	2.7668	2.7622	2.7279	2.7629	2.7539	2.7652	2.7342	2.7605	2.7249	**3.47**
**H(1`B)**	3.0063	3.0474	3.0782	3.0838	3.1401	3.0804	3.1156	3.0786	3.1249	3.0860	3.1249	2.7260	2.5820	2.5307	2.5386	2.5326	2.5402	2.5235	2.5434	2.4737	2.5362	2.5222	**3.63**
**H(4`A)**	2.7260	2.5820	2.5307	2.5386	2.5326	2.5402	2.5235	2.5434	2.4736	2.5362	2.5222	3.0063	3.0474	3.0782	3.0838	3.1401	3.0804	3.1157	3.0786	3.1250	3.0860	3.1249	**3.07**
**H(4`B)**	2.7818	2.7823	2.7668	2.7622	2.7279	2.7629	2.7539	2.7652	2.7342	2.7606	2.7249	2.6543	2.6487	2.6305	2.6419	2.5896	2.6417	2.6032	2.6431	2.6064	2.6410	2.5862	**3.25**

[a] methyl protons shifts are averaged

[b] according to [11]

Table S12. Cont. VII

Nuclei	R											S											Exp. [b]
	M06																						
	Gas	$CHCl_3$ (PCM)	$CHCl_3$ (SMD)	DMSO (PCM)	DMSO (SMD)	CH_3OH (PCM)	CH_3OH (SMD)	C_2H_5OH (PCM)	C_2H_5OH (SMD)	H_2O (PCM)	H_2O (SMD)	Gas	$CHCl_3$ (PCM)	$CHCl_3$ (SMD)	DMSO (PCM)	DMSO (SMD)	CH_3OH (PCM)	CH_3OH (SMD)	C_2H_5OH (PCM)	C_2H_5OH (SMD)	H_2O (PCM)	H_2O (SMD)	
**C1**	137.0654	138.4516	140.2395	139.3236	141.3503	139.2467	140.1123	139.1720	140.4485	139.4069	140.3634	137.0654	138.4516	140.2398	139.3235	141.3504	139.2467	140.1121	139.1721	140.4484	139.4069	140.3634	**131.615**
**C2**	141.0015	140.3104	140.8587	140.0820	140.7204	140.0833	141.1742	140.0927	141.0845	140.0951	141.1353	141.0016	140.3104	140.8587	140.0820	140.7204	140.0832	141.1741	140.0926	141.0844	140.0952	141.1353	**133.405**
**C3**	130.9031	132.0791	133.0375	132.4179	133.6008	132.3928	133.8570	132.3762	133.7813	132.4567	134.0117	130.9030	132.0792	133.0375	132.4179	133.6008	132.3928	133.8570	132.3761	133.7814	132.4567	134.0117	**126.19**
**C4**	171.8317	173.1475	173.7103	173.3977	174.0439	173.3927	174.6046	173.3944	174.4353	173.4163	174.6999	171.8316	173.1473	173.7102	173.3977	174.0439	173.3927	174.6047	173.3946	174.4353	173.4162	174.6999	**165.04**
**C5**	119.3016	120.8022	121.4723	121.4782	122.3461	121.4305	122.5495	121.3899	122.4467	121.5376	122.6841	119.3016	120.8022	121.4723	121.4782	122.3461	121.4305	122.5494	121.3900	122.4466	121.5376	122.6841	**116.425**
**C6**	133.8836	134.9666	135.5843	135.7167	136.9736	135.6628	137.7766	135.6201	137.3978	135.7850	138.0348	133.8836	134.9667	135.5844	135.7167	136.9736	135.6628	137.7764	135.6201	137.3978	135.7851	138.0348	**129.92**
**C7**	204.0628	209.7572	211.7199	212.7334	213.5690	212.5343	218.3399	212.3391	217.4244	212.9344	219.4025	204.0628	209.7574	211.7199	212.7333	213.5689	212.5343	218.3399	212.3391	217.4244	212.9344	219.4025	**195.9**
**C8**	61.9162	63.3412	64.4897	64.1687	65.1960	64.1061	65.7786	64.0496	65.7619	64.2417	66.3624	61.9162	63.3412	64.4897	64.1686	65.1960	64.1061	65.7787	64.0496	65.7618	64.2418	66.3624	**67.6**
**C9**	35.6222	35.2871	35.5108	35.0907	35.4757	35.1023	35.6010	35.1220	35.5344	35.0941	35.6326	35.6222	35.2872	35.5107	35.0907	35.4756	35.1023	35.6010	35.1220	35.5343	35.0942	35.6326	**32.2**
**C10**	16.7083	17.5813	18.1514	18.0859	18.8059	18.0490	18.6582	18.0189	18.6456	18.1346	18.8069	16.7083	17.5813	18.1514	18.0859	18.8057	18.0491	18.6582	18.0190	18.6456	18.1347	18.8069	**23.4**
**C11**	15.7148	15.7061	15.9629	15.7600	16.1540	15.7418	16.0965	15.7320	16.1293	15.7928	16.4520	15.7148	15.7062	15.9629	15.7600	16.1539	15.7418	16.0965	15.7321	16.1294	15.7928	16.4520	**17.9**
**C1`**	50.9543	51.6493	52.0331	52.0880	52.7707	52.0474	52.7164	52.0150	52.7142	52.1411	53.1882	55.2346	56.1683	56.6054	56.7013	57.3066	56.6585	57.4892	56.6233	57.4017	56.7561	57.8339	**52.4**
**C2`**	23.7434	24.1299	24.4939	24.2390	24.8309	24.2235	24.8726	24.2161	24.8491	24.2685	24.8089	22.3749	22.6194	23.0874	22.7231	23.4548	22.7089	23.3382	22.7025	23.3656	22.7512	22.9773	**14.5**
**C3`**	22.3749	22.6195	23.0875	22.7231	23.4548	22.7090	23.3382	22.7026	23.3655	22.7512	22.9773	23.7432	24.1299	24.4938	24.2390	24.8309	24.2236	24.8725	24.2161	24.8491	24.2684	24.8089	**14.2**
**C4`**	55.2346	56.1682	56.6053	56.7013	57.3067	56.6585	57.4893	56.6234	57.4017	56.7562	57.8339	50.9543	51.6491	52.0331	52.0880	52.7707	52.0474	52.7165	52.0149	52.7142	52.1412	53.1882	**53.9**
**H2**	8.4710	8.3003	8.2646	8.2010	8.2098	8.2085	8.2547	8.2168	8.2356	8.1948	8.2447	8.4710	8.3004	8.2646	8.2011	8.2098	8.2085	8.2547	8.2168	8.2356	8.1948	8.2447	**8.13**
**H5**	7.0190	7.2548	7.3533	7.3406	7.4661	7.3364	7.4790	7.3327	7.4666	7.3450	7.5012	7.0190	7.2548	7.3533	7.3406	7.4661	7.3364	7.4790	7.3327	7.4666	7.3449	7.5012	**8.03**
**H6**	7.8965	8.0718	8.0862	8.1049	8.2127	8.1041	8.2688	8.1044	8.2319	8.1066	8.3138	7.8965	8.0718	8.0862	8.1049	8.2127	8.1041	8.2687	8.1044	8.2319	8.1066	8.3138	**7.4**
**H(3``A-3``C)**	2.4118	2.5040	2.5244	2.5148	2.5391	2.5145	2.5449	2.5147	2.5432	2.5156	2.5449	2.4118	2.5040	2.5244	2.5148	2.5391	2.5145	2.5449	2.5147	2.5432	2.5156	2.5449	**2.32**
**H8**	4.3790	4.4188	4.4309	4.4650	4.5350	4.4607	4.5910	4.4573	4.5556	4.4698	4.6395	4.3790	4.4188	4.4309	4.4650	4.5350	4.4607	4.5910	4.4573	4.5556	4.4698	4.6395	**5.64**
**H(9A-9B)**	1.7493	1.7094	1.6776	1.7022	1.7023	1.7034	1.7160	1.7054	1.7097	1.7018	1.7519	1.7493	1.7094	1.6776	1.7022	1.7023	1.7034	1.7160	1.7054	1.7097	1.7018	1.7519	**1.92**
**H(10A)**	0.8741	0.7912	0.7792	0.7652	0.7380	0.7659	0.7503	0.7671	0.7494	0.7652	0.7732	0.8741	0.7912	0.7792	0.7653	0.7380	0.7659	0.7503	0.7671	0.7494	0.7652	0.7732	**1.26**
**H(10B)**	1.7340	1.5759	1.5334	1.4945	1.4180	1.4990	1.4175	1.5041	1.4261	1.4907	1.3680	1.7340	1.5759	1.5334	1.4945	1.4180	1.4990	1.4175	1.5041	1.4261	1.4908	1.3680	**1.07**
**H(11A-11C)**	0.7419	0.7472	0.7182	0.7425	0.7135	0.7428	0.7109	0.7437	0.7109	0.7428	0.6819	0.7419	0.7472	0.7182	0.7425	0.7135	0.7428	0.7109	0.7437	0.7109	0.7427	0.6819	**0.78**
**H(1`A)**	2.6595	2.6310	2.6033	2.6005	2.5642	2.6018	2.5733	2.6038	2.5724	2.6000	2.5569	2.8983	2.8520	2.8409	2.8186	2.7856	2.8201	2.8149	2.8222	2.8185	2.8178	2.7893	**3.47**
**H(1`B)**	3.1047	3.0988	3.1243	3.1274	3.1824	3.1247	3.1714	3.1227	3.1622	3.1307	3.1733	2.8031	2.6561	2.6455	2.6169	2.6287	2.6187	2.5969	2.6211	2.6037	2.6161	2.5760	**3.63**
**H(4`A)**	2.8031	2.6561	2.6455	2.6169	2.6287	2.6187	2.5969	2.6211	2.6037	2.6161	2.5760	3.1047	3.0988	3.1243	3.1274	3.1824	3.1247	3.1714	3.1227	3.1622	3.1307	3.1733	**3.07**
**H(4`B)**	2.8983	2.8520	2.8409	2.8186	2.7856	2.8201	2.8149	2.8223	2.8185	2.8177	2.7893	2.6595	2.6310	2.6034	2.6005	2.5642	2.6018	2.5734	2.6038	2.5724	2.6000	2.5569	**3.25**
Nuclei	**M06-2X**																						
**C1**	153.2701	154.2918	155.2203	154.6145	156.2513	154.6227	155.5608	154.6307	156.0977	154.5977	155.9505	153.2701	154.2918	155.2203	154.6145	156.2513	154.6227	155.5608	154.6306	156.0977	154.5977	155.9504	**131.615**
**C2**	155.6852	155.6175	156.2016	155.3655	155.9885	155.4052	156.2748	155.4457	156.2335	155.3216	156.2383	155.6852	155.6175	156.2016	155.3655	155.9886	155.4052	156.2748	155.4455	156.2335	155.3216	156.2383	**133.405**
**C3**	147.8397	149.1050	150.0569	149.4466	150.5836	149.4522	150.8370	149.4581	150.7565	149.4347	150.9812	147.8397	149.1049	150.0569	149.4466	150.5836	149.4521	150.8369	149.4580	150.7565	149.4347	150.9812	**126.19**
**C4**	185.6334	186.4291	187.0524	186.8793	187.5227	186.8717	188.3306	186.8654	188.1116	186.8804	188.5326	185.6334	186.4290	187.0524	186.8793	187.5226	186.8717	188.3306	186.8653	188.1116	186.8805	188.5326	**165.04**
**C5**	132.4865	133.9357	134.6020	134.6717	135.6116	134.6477	135.8981	134.6250	135.7901	134.6877	136.1072	132.4864	133.9358	134.6020	134.6717	135.6117	134.6478	135.8981	134.6251	135.7901	134.6877	136.1071	**116.425**
**C6**	148.6156	150.3509	151.3580	151.4476	153.0243	151.3974	153.5483	151.3494	153.0819	151.4894	153.8860	148.6156	150.3508	151.3580	151.4476	153.0243	151.3975	153.5483	151.3493	153.0819	151.4894	153.8862	**129.92**
**C7**	219.8759	225.6379	226.9337	228.5720	228.9333	228.4221	235.4422	228.2707	234.5748	228.7048	236.8006	219.8759	225.6379	226.9337	228.5720	228.9333	228.4221	235.4422	228.2707	234.5748	228.7048	236.8005	**195.9**
**C8**	67.2387	68.8031	69.4851	69.4405	70.2220	69.4312	70.9265	69.4210	71.1182	69.4408	71.3988	67.2387	68.8031	69.4851	69.4405	70.2220	69.4313	70.9265	69.4210	71.1183	69.4408	71.3988	**67.6**
**C9**	36.9696	36.9458	37.1478	36.9032	37.2728	36.9277	37.3983	36.9530	37.3539	36.8736	37.5866	36.9696	36.9457	37.1478	36.9032	37.2728	36.9277	37.3983	36.9531	37.3539	36.8736	37.5866	**32.2**
**C10**	18.1477	18.5362	18.8172	18.7811	19.2816	18.7856	19.1087	18.7916	19.0516	18.7711	19.0875	18.1476	18.5362	18.8172	18.7811	19.2816	18.7856	19.1087	18.7915	19.0516	18.7711	19.0875	**23.4**
**C11**	15.1800	15.2020	15.3180	15.1823	15.3831	15.2037	15.2840	15.2266	15.3231	15.1556	15.5654	15.1800	15.2020	15.3180	15.1823	15.3831	15.2038	15.2840	15.2266	15.3231	15.1556	15.5654	**17.9**
**C1`**	52.8402	53.4905	53.7693	53.9360	54.5072	53.9285	54.4838	53.9223	54.4927	53.9368	54.9763	56.9817	57.9829	58.3494	58.4105	58.9562	58.4068	59.0951	58.4043	59.0618	58.4073	59.3981	**52.4**
**C2`**	24.7714	25.1364	25.3996	25.3126	25.7001	25.3263	25.7565	25.3403	25.8918	25.2925	25.6859	23.8439	24.0932	24.3628	24.2469	24.7248	24.2570	24.6742	24.2688	24.6511	24.2309	24.6514	**14.5**
**C3`**	23.8439	24.0932	24.3628	24.2468	24.7246	24.2570	24.6742	24.2687	24.6511	24.2311	24.6514	24.7714	25.1365	25.3996	25.3125	25.7002	25.3263	25.7565	25.3403	25.8918	25.2925	25.6859	**14.2**
**C4`**	56.9817	57.9830	58.3494	58.4105	58.9562	58.4069	59.0950	58.4043	59.0618	58.4073	59.3982	52.8402	53.4904	53.7693	53.9360	54.5072	53.9285	54.4838	53.9222	54.4927	53.9369	54.9763	**53.9**
**H2**	9.0581	8.9654	8.9916	8.9196	8.9408	8.9206	8.9529	8.9215	8.9111	8.9190	8.9394	9.0581	8.9654	8.9916	8.9196	8.9408	8.9206	8.9529	8.9215	8.9111	8.9190	8.9394	**8.13**
**H5**	7.6107	7.7856	7.8778	7.8682	7.9943	7.8622	8.0101	7.8558	7.9976	7.8740	8.0314	7.6107	7.7856	7.8778	7.8682	7.9943	7.8622	8.0101	7.8558	7.9976	7.8740	8.0314	**8.03**
**H6**	8.3971	8.5484	8.6390	8.6544	8.7977	8.6460	8.8607	8.6375	8.8110	8.6625	8.8901	8.3971	8.5484	8.6390	8.6544	8.7977	8.6460	8.8607	8.6375	8.8110	8.6625	8.8901	**7.4**
**H(3``A-3``C)**	2.4798	2.5017	2.5211	2.5122	2.5364	2.5104	2.5411	2.5083	2.5389	2.5141	2.5505	2.4798	2.5017	2.5211	2.5122	2.5365	2.5104	2.5410	2.5082	2.5389	2.5141	2.5505	**2.32**
**H8**	4.3193	4.3616	4.4037	4.4282	4.5269	4.4214	4.5863	4.4145	4.5260	4.4352	4.6028	4.3193	4.3616	4.4037	4.4282	4.5269	4.4214	4.5863	4.4145	4.5260	4.4352	4.6028	**5.64**
**H(9A-9B)**	1.6803	1.6995	1.6943	1.7378	1.7405	1.7341	1.7320	1.7300	1.7301	1.7416	1.7687	1.6803	1.6995	1.6943	1.7378	1.7405	1.7341	1.7320	1.7300	1.7301	1.7416	1.7687	**1.92**
**H(10A)**	0.9398	0.7567	0.7382	0.7032	0.6622	0.7068	0.6864	0.7054	0.6630	0.7024	0.7059	0.9398	0.7567	0.6866	0.7032	0.6888	0.7068	0.6864	0.7054	0.6630	0.7024	0.7059	**1.26**
**H(10B)**	1.6488	1.5352	1.4812	1.4564	1.3725	1.4644	1.3706	1.4630	1.3623	1.4534	1.3464	1.6488	1.5352	1.4408	1.4564	1.3730	1.4644	1.3706	1.4630	1.3623	1.4534	1.3464	**1.07**
**H(11A-11C)**	0.7277	0.7091	0.6856	0.7150	0.6795	0.7131	0.6779	0.7109	0.6680	0.7171	0.6711	0.7277	0.7091	0.6856	0.7150	0.6795	0.7131	0.6779	0.7110	0.6680	0.7171	0.6711	**0.78**
**H(1`A)**	2.5821	2.5881	2.5775	2.5884	2.5466	2.5871	2.5703	2.5857	2.5747	2.5896	2.5519	2.7647	2.8026	2.7483	2.7947	2.7758	2.7958	2.7734	2.7944	2.7988	2.7946	2.7450	**3.47**
**H(1`B)**	3.0495	3.0681	3.1025	3.1065	3.1673	3.0992	3.1581	3.0978	3.1500	3.1107	3.1804	2.7129	2.5976	2.5529	2.5617	2.5202	2.5641	2.5178	2.5627	2.5406	2.5613	2.5144	**3.63**
**H(4`A)**	2.7129	2.5976	2.5701	2.5617	2.5561	2.5641	2.5178	2.5627	2.5406	2.5613	2.5144	3.0495	3.0681	3.0317	3.1065	3.1605	3.0992	3.1581	3.0978	3.1500	3.1107	3.1804	**3.07**
**H(4`B)**	2.7647	2.8026	2.7881	2.7947	2.7514	2.7958	2.7734	2.7944	2.7989	2.7946	2.7450	2.5821	2.5881	2.5108	2.5884	2.5727	2.5871	2.5703	2.5857	2.5747	2.5896	2.5519	**3.25**

[a] methyl protons shifts are averaged

[b] according to [11]

Table S12. Cont. VIII

Nuclei	R											S											Exp. [b]
	mpW1LYP																						
	Gas	$CHCl_3$ (PCM)	$CHCl_3$ (SMD)	DMSO (PCM)	DMSO (SMD)	CH_3OH (PCM)	CH_3OH (SMD)	C_2H_5OH (PCM)	C_2H_5OH (SMD)	H_2O (PCM)	H_2O (SMD)	Gas	$CHCl_3$ (PCM)	$CHCl_3$ (SMD)	DMSO (PCM)	DMSO (SMD)	CH_3OH (PCM)	CH_3OH (SMD)	C_2H_5OH (PCM)	C_2H_5OH (SMD)	H_2O (PCM)	H_2O (SMD)	
**C1**	139.5814	140.1751	140.9777	140.6894	143.2752	140.6339	142.2699	140.5868	142.4938	140.7481	142.6277	139.5814	140.1751	140.9777	140.6894	143.2752	140.6339	142.2698	140.5868	142.4939	140.7481	142.6277	**131.615**
**C2**	140.6100	140.4399	140.7913	140.2286	140.6777	140.2422	141.0622	140.2566	140.9689	140.2140	141.0227	140.6098	140.4399	140.7913	140.2286	140.6779	140.2422	141.0622	140.2566	140.9689	140.2139	141.0227	**133.405**
**C3**	134.1464	135.2290	136.1264	135.5715	136.5570	135.5515	136.8796	135.5326	136.8156	135.5884	137.0117	134.1461	135.2290	136.1264	135.5715	136.5569	135.5515	136.8796	135.5326	136.8156	135.5884	137.0118	**126.19**
**C4**	174.9125	175.8657	176.6014	176.4549	177.0773	176.4119	177.7718	176.3716	177.6013	176.4949	177.8533	174.9125	175.8657	176.6014	176.4549	177.0775	176.4119	177.7718	176.3716	177.6012	176.4949	177.8532	**165.04**
**C5**	119.2010	120.3495	120.9195	121.0114	121.7973	120.9653	122.0837	120.9217	121.9890	121.0543	122.1934	119.2010	120.3495	120.9195	121.0114	121.7973	120.9653	122.0837	120.9217	121.9889	121.0542	122.1935	**116.425**
**C6**	133.6017	135.2712	136.2078	136.2118	137.0592	136.1573	137.8393	136.1018	137.5482	136.2586	138.0932	133.6016	135.2712	136.2078	136.2118	137.0591	136.1573	137.8392	136.1018	137.5482	136.2585	138.0934	**129.92**
**C7**	203.8368	208.4344	209.6616	211.1899	212.7198	210.9948	217.6963	210.8083	216.7168	211.3808	218.8838	203.8365	208.4344	209.6616	211.1899	212.7194	210.9948	217.6963	210.8083	216.7168	211.3810	218.8840	**195.9**
**C8**	65.7144	66.7517	67.0420	67.3286	68.4046	67.2862	68.8000	67.2464	68.7584	67.3685	69.1910	65.7143	66.7517	67.0420	67.3286	68.4046	67.2862	68.8000	67.2464	68.7584	67.3684	69.1910	**67.6**
**C9**	38.6903	38.2641	38.4588	38.1116	38.2426	38.1202	38.3578	38.1294	38.3106	38.1003	38.4110	38.6902	38.2641	38.4588	38.1116	38.2425	38.1202	38.3578	38.1294	38.3107	38.1004	38.4110	**32.2**
**C10**	19.9646	20.3826	20.6745	20.6752	21.1266	20.6555	21.0109	20.6369	21.0055	20.6915	21.0948	19.9646	20.3826	20.6745	20.6752	21.1266	20.6555	21.0109	20.6369	21.0055	20.6914	21.0948	**23.4**
**C11**	16.2356	16.3458	16.5064	16.4356	16.6122	16.4286	16.5569	16.4228	16.5670	16.4400	16.7073	16.2356	16.3458	16.5063	16.4356	16.6123	16.4286	16.5569	16.4228	16.5670	16.4400	16.7074	**17.9**
**C1`**	52.0088	52.8564	53.0822	53.4081	53.9346	53.3740	53.8687	53.3408	53.8452	53.4378	54.2795	56.5396	57.5874	57.9602	58.0930	58.5770	58.0584	58.7417	58.0257	58.6925	58.1243	58.9582	**52.4**
**C2`**	25.8957	26.3447	26.5842	26.5645	26.9311	26.5482	26.9756	26.5335	26.9902	26.5778	26.8635	25.5661	25.6938	26.1283	25.8900	26.4535	25.8720	26.3801	25.8561	26.3761	25.9062	26.1494	**14.5**
**C3`**	25.5661	25.6938	26.1283	25.8900	26.4535	25.8720	26.3800	25.8561	26.3761	25.9061	26.1494	25.8957	26.3447	26.5841	26.5645	26.9311	26.5482	26.9756	26.5335	26.9902	26.5778	26.8635	**14.2**
**C4`**	56.5395	57.5874	57.9603	58.0930	58.5769	58.0584	58.7418	58.0257	58.6926	58.1243	58.9584	52.0087	52.8564	53.0821	53.4081	53.9347	53.3740	53.8687	53.3408	53.8452	53.4378	54.2795	**53.9**
**H2**	8.5453	8.4648	8.4937	8.4003	8.3306	8.4062	8.3740	8.4113	8.3614	8.3939	8.3539	8.5453	8.4648	8.4937	8.4003	8.3306	8.4062	8.3740	8.4113	8.3614	8.3939	8.3539	**8.13**
**H5**	7.1145	7.2719	7.3612	7.3423	7.4603	7.3384	7.4815	7.3342	7.4711	7.3459	7.4995	7.1145	7.2719	7.3612	7.3423	7.4603	7.3384	7.4815	7.3342	7.4711	7.3459	7.4995	**8.03**
**H6**	7.7941	7.9721	8.0730	8.0664	8.1444	8.0614	8.2114	8.0559	8.1854	8.0707	8.2343	7.7941	7.9721	8.0730	8.0664	8.1444	8.0614	8.2114	8.0559	8.1854	8.0707	8.2343	**7.4**
**H(3``A-3``C)**	2.3861	2.4282	2.4451	2.4435	2.4632	2.4428	2.4717	2.4419	2.4693	2.4441	2.4747	2.3861	2.4282	2.4451	2.4435	2.4631	2.4428	2.4717	2.4419	2.4693	2.4441	2.4747	**2.32**
**H8**	4.5405	4.6030	4.6753	4.6513	4.7031	4.6484	4.7590	4.6450	4.7379	4.6538	4.7451	4.5404	4.6030	4.6753	4.6513	4.7031	4.6484	4.7590	4.6450	4.7379	4.6538	4.7451	**5.64**
**H(9A-9B)**	1.6505	1.6989	1.6808	1.7247	1.6890	1.7245	1.6835	1.7235	1.6770	1.7245	1.7065	1.6505	1.6989	1.6808	1.7247	1.6890	1.7245	1.6835	1.7235	1.6770	1.7245	1.7065	**1.92**
**H(10A)**	1.0855	0.9687	0.9729	0.9227	0.8473	0.9263	0.8999	0.9295	0.8925	0.9192	0.9047	1.0855	0.9687	0.9729	0.9227	0.8473	0.9263	0.8999	0.9295	0.8925	0.9192	0.9047	**1.26**
**H(10B)**	1.7318	1.6280	1.5872	1.5585	1.4795	1.5628	1.4809	1.5668	1.4854	1.5543	1.4454	1.7318	1.6280	1.5872	1.5585	1.4795	1.5628	1.4809	1.5668	1.4854	1.5543	1.4454	**1.07**
**H(11A-11C)**	0.7863	0.7963	0.7825	0.8052	0.7636	0.8049	0.7711	0.8042	0.7695	0.8055	0.7578	0.7863	0.7963	0.7825	0.8052	0.7636	0.8049	0.7711	0.8042	0.7695	0.8055	0.7578	**0.78**
**H(1`A)**	2.7162	2.7062	2.6903	2.6973	2.6464	2.6982	2.6556	2.6987	2.6583	2.6964	2.6372	2.8399	2.8370	2.8159	2.8246	2.8038	2.8255	2.8101	2.8262	2.8166	2.8236	2.7809	**3.47**
**H(1`B)**	3.1751	3.2169	3.2545	3.2533	3.3124	3.2512	3.2939	3.2488	3.2882	3.2551	3.2961	2.8533	2.6856	2.6641	2.6621	2.7031	2.6625	2.6526	2.6629	2.6567	2.6620	2.6500	**3.63**
**H(4`A)**	2.8533	2.6856	2.6641	2.6621	2.7031	2.6625	2.6526	2.6629	2.6567	2.6619	2.6500	3.1751	3.2169	3.2545	3.2533	3.3124	3.2512	3.2939	3.2488	3.2882	3.2550	3.2962	**3.07**
**H(4`B)**	2.8399	2.8370	2.8159	2.8246	2.8038	2.8255	2.8101	2.8262	2.8166	2.8236	2.7809	2.7162	2.7062	2.6903	2.6973	2.6464	2.6982	2.6556	2.6987	2.6583	2.6964	2.6372	**3.25**
Nuclei	mpW1PW91																						
**C1**	136.6939	137.3276	137.9762	137.8634	139.5017	137.8071	139.5506	137.7608	138.6614	137.9208	139.9257	136.6939	137.3276	137.9762	137.8634	139.5017	137.8071	139.5508	137.7608	138.6614	137.9208	139.9256	**131.615**
**C2**	139.7884	139.6733	140.0697	139.4195	140.0010	139.4408	140.2455	139.4672	140.2182	139.3977	140.2078	139.7884	139.6734	140.0697	139.4195	140.0010	139.4408	140.2455	139.4673	140.2182	139.3977	140.2078	**133.405**
**C3**	131.9261	133.1562	134.0791	133.5280	134.6189	133.5084	134.9850	133.4943	134.8363	133.5456	135.1528	131.9261	133.1562	134.0791	133.5280	134.6189	133.5084	134.9849	133.4943	134.8365	133.5456	135.1528	**126.19**
**C4**	171.9409	172.8370	173.4594	173.3402	173.9484	173.3046	174.7576	173.2751	174.5278	173.3737	174.8649	171.9409	172.8371	173.4594	173.3402	173.9484	173.3046	174.7575	173.2751	174.5279	173.3737	174.8650	**165.04**
**C5**	118.4212	119.6411	120.2487	120.2781	121.1201	120.2373	121.4977	120.2027	121.2636	120.3167	121.6446	118.4211	119.6411	120.2488	120.2781	121.1200	120.2373	121.4976	120.2027	121.2635	120.3167	121.6447	**116.425**
**C6**	132.4768	134.1867	135.1767	135.1048	136.4472	135.0544	136.7242	135.0087	136.9411	135.1509	137.0395	132.4768	134.1868	135.1767	135.1048	136.4472	135.0544	136.7243	135.0087	136.9409	135.1509	137.0394	**129.92**
**C7**	201.6360	205.9828	206.9194	208.5946	209.2684	208.4081	214.9780	208.2323	212.9177	208.7757	216.2004	201.6360	205.9825	206.9195	208.5946	209.2683	208.4081	214.9778	208.2322	212.9177	208.7757	216.2007	**195.9**
**C8**	63.6022	64.7282	65.1276	65.3172	66.2170	65.2750	66.9036	65.2400	66.5231	65.3582	67.4149	63.6022	64.7283	65.1276	65.3172	66.2170	65.2750	66.9035	65.2399	66.5232	65.3582	67.4149	**67.6**
**C9**	36.6617	36.4371	36.6363	36.3113	36.6987	36.3199	36.5807	36.3343	36.7354	36.3024	36.7199	36.6617	36.4372	36.6363	36.3113	36.6987	36.3198	36.5807	36.3343	36.7354	36.3024	36.7200	**32.2**
**C10**	18.3302	18.8139	19.0513	19.0869	19.5919	19.0721	19.4035	19.0627	19.4369	19.1000	19.5724	18.3302	18.8139	19.0512	19.0869	19.5918	19.0721	19.4035	19.0626	19.4368	19.1000	19.5724	**23.4**
**C11**	15.7468	15.8768	15.9750	15.9232	16.1381	15.9211	16.0623	15.9245	16.0769	15.9241	16.2395	15.7468	15.8768	15.9750	15.9232	16.1381	15.9211	16.0622	15.9245	16.0769	15.9241	16.2394	**17.9**
**C1`**	50.6020	51.4331	51.6436	51.8910	52.4300	51.8659	52.3758	51.8458	52.3430	51.9139	52.8324	54.8332	55.8629	56.2050	56.3078	56.8174	56.2802	57.0452	56.2586	56.9406	56.3333	57.2896	**52.4**
**C2`**	24.7040	25.0991	25.3658	25.2486	25.6805	25.2403	25.6737	25.2375	25.6946	25.2555	25.5575	24.2157	24.3640	24.7322	24.5023	25.0103	24.4911	25.0153	24.4858	24.9091	24.5124	24.7926	**14.5**
**C3`**	24.2156	24.3640	24.7323	24.5023	25.0103	24.4911	25.0152	24.4858	24.9091	24.5124	24.7926	24.7040	25.0991	25.3658	25.2486	25.6805	25.2403	25.6737	25.2376	25.6946	25.2555	25.5575	**14.2**
**C4`**	54.8331	55.8629	56.2050	56.3078	56.8174	56.2802	57.0453	56.2587	56.9406	56.3333	57.2897	50.6021	51.4331	51.6435	51.8910	52.4299	51.8659	52.3758	51.8458	52.3429	51.9139	52.8324	**53.9**
**H2**	8.5913	8.5113	8.5471	8.4368	8.4432	8.4438	8.4110	8.4505	8.4815	8.4300	8.3923	8.5913	8.5113	8.5471	8.4368	8.4432	8.4438	8.4109	8.4505	8.4815	8.4300	8.3923	**8.13**
**H5**	7.1374	7.3037	7.3973	7.3727	7.4968	7.3691	7.5201	7.3655	7.5046	7.3765	7.5417	7.1374	7.3037	7.3973	7.3727	7.4968	7.3691	7.5201	7.3655	7.5046	7.3765	7.5417	**8.03**
**H6**	7.8828	8.0560	8.1592	8.1417	8.2688	8.1374	8.2840	8.1330	8.3175	8.1459	8.3083	7.8828	8.0560	8.1592	8.1417	8.2688	8.1374	8.2840	8.1331	8.3175	8.1459	8.3083	**7.4**
**H(3``A-3``C)**	2.3608	2.4068	2.4222	2.4197	2.4418	2.4192	2.4532	2.4188	2.4477	2.4203	2.4590	2.3608	2.4068	2.4222	2.4197	2.4418	2.4192	2.4532	2.4188	2.4477	2.4203	2.4590	**2.32**
**H8**	4.5358	4.6003	4.6682	4.6448	4.7161	4.6421	4.7598	4.6396	4.7558	4.6474	4.7517	4.5358	4.6003	4.6682	4.6448	4.7161	4.6421	4.7598	4.6396	4.7558	4.6474	4.7517	**5.64**
**H(9A-9B)**	1.6837	1.7267	1.7130	1.7470	1.7593	1.7471	1.6905	1.7470	1.7486	1.7469	1.7202	1.6837	1.7267	1.7130	1.7470	1.7594	1.7471	1.6906	1.7470	1.7485	1.7469	1.7202	**1.92**
**H(10A)**	1.0162	0.9022	0.8993	0.8458	0.7767	0.8502	0.8292	0.8546	0.8167	0.8417	0.8316	1.0162	0.9022	0.8993	0.8458	0.7766	0.8502	0.8292	0.8546	0.8167	0.8417	0.8316	**1.26**
**H(10B)**	1.7063	1.5987	1.5480	1.5258	1.4525	1.5304	1.4408	1.5350	1.4574	1.5217	1.4087	1.7063	1.5987	1.5480	1.5258	1.4525	1.5304	1.4408	1.5350	1.4574	1.5217	1.4087	**1.07**
**H(11A-11C)**	0.7308	0.7402	0.7238	0.7432	0.7107	0.7433	0.7101	0.7435	0.7175	0.7433	0.6979	0.7308	0.7402	0.7238	0.7432	0.7107	0.7433	0.7101	0.7435	0.7175	0.7433	0.6979	**0.78**
**H(1`A)**	2.6561	2.6496	2.6336	2.6398	2.5970	2.6408	2.6030	2.6418	2.6086	2.6391	2.5871	2.7779	2.7789	2.7625	2.7647	2.7386	2.7657	2.7581	2.7668	2.7498	2.7640	2.7318	**3.47**
**H(1`B)**	3.1516	3.1916	3.2180	3.2216	3.2831	3.2199	3.2523	3.2183	3.2602	3.2235	3.2571	2.8531	2.6777	2.6350	2.6494	2.6574	2.6497	2.6262	2.6505	2.5969	2.6495	2.6225	**3.63**
**H(4`A)**	2.8531	2.6777	2.6350	2.6494	2.6574	2.6497	2.6262	2.6505	2.5969	2.6495	2.6225	3.1516	3.1916	3.2180	3.2216	3.2831	3.2199	3.2524	3.2183	3.2602	3.2235	3.2571	**3.07**
**H(4`B)**	2.7779	2.7789	2.7625	2.7647	2.7386	2.7657	2.7581	2.7668	2.7498	2.7640	2.7318	2.6561	2.6496	2.6336	2.6398	2.5970	2.6408	2.6030	2.6418	2.6086	2.6391	2.5870	**3.25**

[a] methyl protons shifts are averaged

[b] according to [11]

Table S12. Cont. IX

Nuclei	R											S											Exp. [b]
	O3LYP																						
	Gas	$CHCl_3$ (PCM)	$CHCl_3$ (SMD)	DMSO (PCM)	DMSO (SMD)	CH_3OH (PCM)	CH_3OH (SMD)	C_2H_5OH (PCM)	C_2H_5OH (SMD)	H_2O (PCM)	H_2O (SMD)	Gas	$CHCl_3$ (PCM)	$CHCl_3$ (SMD)	DMSO (PCM)	DMSO (SMD)	CH_3OH (PCM)	CH_3OH (SMD)	C_2H_5OH (PCM)	C_2H_5OH (SMD)	H_2O (PCM)	H_2O (SMD)	
**C1**	134.4305	136.0139	137.9574	137.1814	139.5771	137.1179	138.4207	137.0526	138.7558	137.2392	138.7828	134.4307	136.0139	137.9574	137.1814	139.5771	137.1179	138.4208	137.0526	138.7558	137.2392	138.7828	**131.615**
**C2**	135.1040	135.0620	135.7541	135.0220	135.7085	135.0380	136.0387	135.0487	135.8986	134.9998	135.9981	135.1041	135.0620	135.7541	135.0220	135.7085	135.0380	136.0387	135.0487	135.8985	134.9998	135.9981	**133.405**
**C3**	128.1027	129.4127	130.3350	129.8526	130.8538	129.8424	131.1689	129.8261	131.0709	129.8552	131.3013	128.1026	129.4127	130.3349	129.8526	130.8538	129.8424	131.1687	129.8261	131.0708	129.8552	131.3013	**126.19**
**C4**	168.6663	169.3575	169.7684	169.5991	170.0928	169.5950	170.7061	169.5852	170.5278	169.5957	170.7645	168.6663	169.3575	169.7683	169.5991	170.0928	169.5950	170.7061	169.5852	170.5278	169.5957	170.7645	**165.04**
**C5**	114.5502	115.7242	116.2170	116.2527	116.8387	116.2344	117.1374	116.2103	117.0089	116.2626	117.2369	114.5502	115.7242	116.2170	116.2527	116.8387	116.2344	117.1374	116.2103	117.0087	116.2626	117.2369	**116.425**
**C6**	127.5968	128.8400	129.5626	129.8281	130.8978	129.7743	131.5685	129.7154	131.3073	129.8721	131.7886	127.5969	128.8400	129.5627	129.8281	130.8978	129.7743	131.5686	129.7154	131.3074	129.8721	131.7886	**129.92**
**C7**	197.3149	202.3848	204.5863	205.5630	206.9613	205.3692	211.2504	205.1748	210.5828	205.7455	212.3456	197.3149	202.3848	204.5862	205.5630	206.9613	205.3692	211.2505	205.1748	210.5828	205.7455	212.3456	**195.9**
**C8**	64.3639	65.7214	66.8563	66.7463	67.8531	66.6788	68.2920	66.6103	68.3035	66.8083	68.6082	64.3639	65.7214	66.8563	66.7463	67.8531	66.6788	68.2920	66.6103	68.3034	66.8083	68.6082	**67.6**
**C9**	36.9425	36.6165	36.7516	36.5067	36.6592	36.5226	36.9282	36.5337	36.8474	36.4852	36.9229	36.9425	36.6165	36.7517	36.5067	36.6592	36.5228	36.9282	36.5337	36.8475	36.4852	36.9229	**32.2**
**C10**	19.4987	19.9738	20.3140	20.3197	20.8098	20.3079	20.7221	20.2909	20.6949	20.3242	20.7827	19.4988	19.9738	20.3139	20.3197	20.8097	20.3079	20.7222	20.2909	20.6950	20.3242	20.7827	**23.4**
**C11**	16.0561	16.1417	16.3097	16.2617	16.4860	16.2635	16.4415	16.2606	16.4421	16.2538	16.5680	16.0561	16.1417	16.3097	16.2617	16.4860	16.2635	16.4417	16.2606	16.4421	16.2538	16.5680	**17.9**
**C1`**	50.8183	51.5522	51.9154	52.1010	52.6666	52.0772	52.6347	52.0482	52.5901	52.1169	53.0371	55.3148	56.2422	56.7045	56.8054	57.3623	56.7815	57.4538	56.7522	57.3887	56.8214	57.6664	**52.4**
**C2`**	25.9067	26.2739	26.5823	26.5115	26.8869	26.5055	26.9100	26.4945	26.9113	26.5109	26.7430	25.7740	25.9669	26.3359	26.0920	26.6513	26.0951	26.4600	26.0938	26.4697	26.0827	26.2127	**14.5**
**C3`**	25.7740	25.9669	26.3358	26.0920	26.6513	26.0951	26.4599	26.0938	26.4696	26.0827	26.2127	25.9067	26.2739	26.5823	26.5115	26.8869	26.5056	26.9100	26.4945	26.9113	26.5109	26.7430	**14.2**
**C4`**	55.3148	56.2422	56.7045	56.8054	57.3623	56.7815	57.4539	56.7522	57.3887	56.8214	57.6664	50.8183	51.5522	51.9154	52.1010	52.6666	52.0772	52.6348	52.0482	52.5902	52.1169	53.0371	**53.9**
**H2**	8.3569	8.1900	8.1234	8.0662	8.0208	8.0749	8.0938	8.0830	8.0613	8.0573	8.0811	8.3569	8.1900	8.1234	8.0662	8.0208	8.0749	8.0938	8.0830	8.0613	8.0573	8.0811	**8.13**
**H5**	7.0545	7.2194	7.2975	7.2865	7.3946	7.2832	7.4164	7.2794	7.4046	7.2895	7.4330	7.0545	7.2194	7.2975	7.2865	7.3946	7.2832	7.4165	7.2794	7.4045	7.2895	7.4330	**8.03**
**H6**	7.7520	7.8578	7.8909	7.9209	8.0020	7.9176	8.0547	7.9139	8.0266	7.9236	8.0727	7.7520	7.8578	7.8909	7.9209	8.0020	7.9176	8.0547	7.9139	8.0266	7.9236	8.0727	**7.4**
**H(3``A-3``C)**	2.3520	2.3915	2.4027	2.4013	2.4183	2.4012	2.4275	2.4007	2.4234	2.4012	2.4319	2.3521	2.3915	2.4027	2.4013	2.4183	2.4012	2.4275	2.4007	2.4234	2.4012	2.4319	**2.32**
**H8**	4.7384	4.7673	4.7634	4.7784	4.8071	4.7783	4.8337	4.7779	4.8158	4.7783	4.8145	4.7384	4.7673	4.7634	4.7784	4.8071	4.7784	4.8337	4.7779	4.8158	4.7783	4.8145	**5.64**
**H(9A-9B)**	1.5682	1.5951	1.5689	1.6249	1.6058	1.6230	1.6378	1.6207	1.6226	1.6266	1.6569	1.5681	1.5951	1.5689	1.6249	1.6058	1.6230	1.6378	1.6207	1.6227	1.6266	1.6569	**1.92**
**H(10A)**	1.0566	0.9964	0.9826	0.9640	0.9298	0.9674	0.9602	0.9705	0.9581	0.9605	0.9652	1.0566	0.9964	0.9825	0.9640	0.9298	0.9674	0.9602	0.9705	0.9581	0.9605	0.9652	**1.26**
**H(10B)**	1.6642	1.5683	1.5303	1.5138	1.4464	1.5180	1.4472	1.5218	1.4534	1.5096	1.4200	1.6642	1.5683	1.5303	1.5138	1.4464	1.5180	1.4472	1.5218	1.4534	1.5096	1.4200	**1.07**
**H(11A-11C)**	0.7585	0.7698	0.7538	0.7850	0.7581	0.7846	0.7601	0.7838	0.7600	0.7853	0.7493	0.7585	0.7698	0.7538	0.7850	0.7581	0.7846	0.7601	0.7838	0.7600	0.7853	0.7493	**0.78**
**H(1`A)**	2.7600	2.7462	2.7246	2.7326	2.6846	2.7339	2.6932	2.7349	2.6962	2.7312	2.6672	2.8987	2.8955	2.8865	2.8784	2.8448	2.8798	2.8458	2.8810	2.8530	2.8770	2.8209	**3.47**
**H(1`B)**	3.2315	3.2637	3.2930	3.2992	3.3560	3.2968	3.3425	3.2941	3.3370	3.3014	3.3398	2.8789	2.7804	2.7781	2.7692	2.7903	2.7691	2.7649	2.7690	2.7713	2.7695	2.7604	**3.63**
**H(4`A)**	2.8789	2.7804	2.7781	2.7692	2.7903	2.7691	2.7649	2.7690	2.7713	2.7695	2.7604	3.2314	3.2637	3.2929	3.2992	3.3560	3.2968	3.3425	3.2941	3.3370	3.3014	3.3398	**3.07**
**H(4`B)**	2.8987	2.8955	2.8865	2.8784	2.8448	2.8798	2.8458	2.8810	2.8530	2.8770	2.8209	2.7601	2.7462	2.7246	2.7326	2.6846	2.7339	2.6932	2.7349	2.6963	2.7312	2.6672	**3.25**
Nuclei	OLYP																						
**C1**	133.4973	134.9967	136.7423	135.9869	138.1355	135.9105	137.0523	135.8279	137.3582	136.0483	137.3957	133.4972	134.9967	136.7423	135.9869	138.1355	135.9105	137.0523	135.8279	137.3582	136.0483	137.3958	**131.615**
**C2**	132.8148	132.8487	133.5496	132.8076	133.4430	132.8239	133.7915	132.8337	133.6470	132.7821	133.7443	132.8148	132.8487	133.5496	132.8076	133.4430	132.8239	133.7915	132.8337	133.6470	132.7821	133.7444	**133.405**
**C3**	126.5599	127.8006	128.6789	128.1954	129.1589	128.1879	129.5213	128.1742	129.4129	128.1937	129.6445	126.5599	127.8006	128.6789	128.1954	129.1589	128.1879	129.5213	128.1742	129.4129	128.1936	129.6445	**126.19**
**C4**	167.7537	168.3736	168.9136	168.7044	169.3090	168.6971	169.8721	168.6845	169.7070	168.7031	169.9347	167.7538	168.3736	168.9136	168.7044	169.3090	168.6971	169.8721	168.6845	169.7070	168.7031	169.9348	**165.04**
**C5**	112.8194	113.7678	114.2270	114.2360	114.8109	114.2212	115.1488	114.2014	115.0169	114.2416	115.2399	112.8194	113.7678	114.2270	114.2360	114.8109	114.2212	115.1488	114.2014	115.0169	114.2417	115.2399	**116.425**
**C6**	125.1417	126.6345	127.4010	127.6455	128.7254	127.5959	129.3246	127.5396	129.0740	127.6825	129.5520	125.1417	126.6345	127.4011	127.6455	128.7254	127.5959	129.3246	127.5396	129.0740	127.6826	129.5520	**129.92**
**C7**	195.8448	200.6564	202.7457	203.5979	204.9396	203.3928	208.7989	203.1824	208.2210	203.7825	209.8532	195.8448	200.6564	202.7457	203.5979	204.9396	203.3928	208.7989	203.1824	208.2210	203.7826	209.8532	**195.9**
**C8**	65.8275	67.1728	68.3605	68.2452	69.2981	68.1620	69.7262	68.0754	69.7499	68.3160	69.9831	65.8275	67.1728	68.3605	68.2452	69.2981	68.1620	69.7262	68.0754	69.7499	68.3161	69.9831	**67.6**
**C9**	37.9207	37.6469	37.8141	37.5531	37.6981	37.5696	38.0419	37.5808	37.9519	37.5290	38.0099	37.9207	37.6469	37.8141	37.5531	37.6981	37.5696	38.0419	37.5808	37.9519	37.5291	38.0098	**32.2**
**C10**	20.3218	20.8147	21.1874	21.1758	21.6556	21.1624	21.5838	21.1436	21.5598	21.1798	21.6288	20.3217	20.8147	21.1874	21.1758	21.6556	21.1624	21.5838	21.1436	21.5598	21.1797	21.6288	**23.4**
**C11**	16.1889	16.2840	16.4723	16.4168	16.6185	16.4168	16.5822	16.4115	16.5846	16.4083	16.6990	16.1890	16.2840	16.4723	16.4168	16.6185	16.4168	16.5822	16.4115	16.5846	16.4083	16.6990	**17.9**
**C1`**	51.7583	52.4747	52.8734	53.0306	53.5856	53.0041	53.5789	52.9722	53.5340	53.0470	53.9720	56.3513	57.2514	57.6980	57.8094	58.3407	57.7827	58.4390	57.7503	58.3706	57.8255	58.6724	**52.4**
**C2`**	26.4821	26.8755	27.2199	27.1791	27.5358	27.1676	27.5686	27.1510	27.5718	27.1812	27.3888	26.5391	26.7441	27.1023	26.8670	27.4076	26.8703	27.1995	26.8677	27.2099	26.8549	26.9266	**14.5**
**C3`**	26.5391	26.7441	27.1023	26.8670	27.4076	26.8703	27.1995	26.8677	27.2099	26.8549	26.9266	26.4820	26.8755	27.2199	27.1791	27.5358	27.1676	27.5686	27.1510	27.5718	27.1812	27.3888	**14.2**
**C4`**	56.3514	57.2514	57.6980	57.8094	58.3407	57.7827	58.4390	57.7503	58.3706	57.8255	58.6723	51.7583	52.4747	52.8734	53.0306	53.5856	53.0041	53.5789	52.9722	53.5340	53.0469	53.9720	**53.9**
**H2**	8.1736	8.0277	7.9809	7.9241	7.8976	7.9339	7.9768	7.9436	7.9446	7.9146	7.9655	8.1736	8.0277	7.9809	7.9241	7.8976	7.9339	7.9768	7.9436	7.9446	7.9146	7.9655	**8.13**
**H5**	6.9912	7.1352	7.2110	7.1979	7.3065	7.1947	7.3280	7.1915	7.3164	7.2005	7.3432	6.9911	7.1352	7.2110	7.1979	7.3065	7.1947	7.3280	7.1915	7.3164	7.2005	7.3432	**8.03**
**H6**	7.6484	7.7595	7.7955	7.8198	7.9092	7.8178	7.9577	7.8157	7.9300	7.8213	7.9755	7.6484	7.7595	7.7955	7.8198	7.9092	7.8178	7.9577	7.8157	7.9300	7.8213	7.9755	**7.4**
**H(3``A-3``C)**	2.3288	2.3659	2.3819	2.3779	2.3962	2.3778	2.4072	2.3774	2.4030	2.3776	2.4093	2.3288	2.3659	2.3819	2.3779	2.3962	2.3778	2.4072	2.3774	2.4030	2.3776	2.4094	**2.32**
**H8**	4.9202	4.9332	4.9108	4.9278	4.9522	4.9297	4.9716	4.9315	4.9568	4.9257	4.9445	4.9202	4.9332	4.9108	4.9278	4.9522	4.9297	4.9716	4.9315	4.9568	4.9257	4.9445	**5.64**
**H(9A-9B)**	1.5841	1.6326	1.6229	1.6739	1.6596	1.6719	1.6974	1.6698	1.6832	1.6756	1.7146	1.5841	1.6326	1.6229	1.6739	1.6596	1.6719	1.6974	1.6698	1.6832	1.6756	1.7146	**1.92**
**H(10A)**	1.1064	1.0590	1.0269	1.0190	0.9745	1.0224	1.0070	1.0255	1.0042	1.0151	1.0107	1.1063	1.0590	1.0269	1.0190	0.9745	1.0224	1.0070	1.0255	1.0042	1.0151	1.0107	**1.26**
**H(10B)**	1.6864	1.6035	1.5696	1.5493	1.4876	1.5534	1.4877	1.5574	1.4940	1.5449	1.4596	1.6864	1.6035	1.5696	1.5493	1.4876	1.5534	1.4877	1.5574	1.4940	1.5449	1.4596	**1.07**
**H(11A-11C)**	0.7708	0.7853	0.7679	0.7981	0.7704	0.7976	0.7704	0.7970	0.7701	0.7980	0.7588	0.7707	0.7853	0.7679	0.7981	0.7704	0.7976	0.7704	0.7970	0.7701	0.7980	0.7588	**0.78**
**H(1`A)**	2.8205	2.8088	2.7881	2.7946	2.7464	2.7958	2.7504	2.7969	2.7540	2.7930	2.7192	2.9653	2.9586	2.9525	2.9425	2.9109	2.9439	2.9077	2.9453	2.9158	2.9407	2.7192	**3.47**
**H(1`B)**	3.3214	3.3554	3.3871	3.3900	3.4499	3.3877	3.4337	3.3854	3.4292	3.3919	3.4276	2.9696	2.8737	2.8750	2.8601	2.8805	2.8595	2.8590	2.8590	2.8647	2.8604	3.4276	**3.63**
**H(4`A)**	2.9696	2.8737	2.8750	2.8601	2.8805	2.8595	2.8590	2.8590	2.8647	2.8604	2.8510	3.3214	3.3554	3.3871	3.3900	3.4499	3.3877	3.4337	3.3854	3.4292	3.3919	2.8510	**3.07**
**H(4`B)**	2.9653	2.9586	2.9525	2.9425	2.9109	2.9439	2.9077	2.9453	2.9158	2.9407	2.8784	2.8205	2.8088	2.7881	2.7946	2.7464	2.7958	2.7504	2.7969	2.7540	2.7930	2.8784	**3.25**

[a] methyl protons shifts are averaged

[b] according to [11]

Table S12. Cont. X

Nuclei	R											S											Exp. [b]
	OPBE																						
	Gas	$CHCl_3$ (PCM)	$CHCl_3$ (SMD)	DMSO (PCM)	DMSO (SMD)	CH_3OH (PCM)	CH_3OH (SMD)	C_2H_5OH (PCM)	C_2H_5OH (SMD)	H_2O (PCM)	H_2O (SMD)	Gas	$CHCl_3$ (PCM)	$CHCl_3$ (SMD)	DMSO (PCM)	DMSO (SMD)	CH_3OH (PCM)	CH_3OH (SMD)	C_2H_5OH (PCM)	C_2H_5OH (SMD)	H_2O (PCM)	H_2O (SMD)	
**C1**	130.1638	131.8468	133.4651	132.7350	135.1447	132.6682	133.8744	132.5918	134.1630	132.8029	134.3068	130.1638	131.8468	133.4651	132.7350	135.1447	132.6682	133.8744	132.5918	134.1630	132.8029	134.3068	**131.615**
**C2**	131.6232	131.6706	132.3579	131.5537	132.2232	131.5803	132.5470	131.5885	132.4160	131.5220	132.5190	131.6232	131.6705	132.3579	131.5537	132.2232	131.5803	132.5470	131.5885	132.4160	131.5220	132.5190	**133.405**
**C3**	123.9490	125.4052	126.2792	125.8080	126.8467	125.8063	127.2134	125.7855	127.0967	125.8029	127.3817	123.9490	125.4053	126.2792	125.8080	126.8467	125.8063	127.2133	125.7855	127.0967	125.8029	127.3817	**126.19**
**C4**	164.2404	164.9405	165.3607	165.1529	165.5946	165.1622	166.2741	165.1523	166.1024	165.1374	166.3392	164.2404	164.9406	165.3607	165.1529	165.5946	165.1622	166.2741	165.1523	166.1024	165.1374	166.3392	**165.04**
**C5**	111.7064	112.8862	113.3260	113.3367	113.9238	113.3332	114.2926	113.3095	114.1480	113.3328	114.4223	111.7065	112.8861	113.3260	113.3367	113.9237	113.3332	114.2927	113.3095	114.1480	113.3328	114.4223	**116.425**
**C6**	123.7638	125.1525	125.8779	126.0227	126.9664	125.9846	127.7181	125.9280	127.4830	126.0529	127.9046	123.7639	125.1525	125.8779	126.0227	126.9664	125.9846	127.7181	125.9280	127.4830	126.0529	127.9046	**129.92**
**C7**	192.9939	197.7806	199.6398	200.4668	202.0434	200.2841	205.5210	200.0936	204.9348	200.6466	206.6364	192.9936	197.7807	199.6398	200.4668	202.0434	200.2841	205.5208	200.0936	204.9348	200.6466	206.6364	**195.9**
**C8**	63.1487	64.6985	65.6901	65.6289	66.8838	65.5642	67.1265	65.4883	67.1166	65.6927	67.5609	63.1486	64.6985	65.6901	65.6289	66.8838	65.5642	67.1265	65.4883	67.1166	65.6927	67.5609	**67.6**
**C9**	35.5355	35.4545	35.5555	35.3420	35.5403	35.3644	35.8125	35.3687	35.7183	35.3154	35.8714	35.5355	35.4545	35.5554	35.3420	35.5403	35.3644	35.8125	35.3687	35.7183	35.3155	35.8714	**32.2**
**C10**	18.3181	18.9088	19.1661	19.1859	19.6018	19.1867	19.5290	19.1692	19.4986	19.1796	19.6633	18.3183	18.9088	19.1661	19.1859	19.6018	19.1867	19.5290	19.1692	19.4986	19.1796	19.6633	**23.4**
**C11**	15.5201	15.6876	15.7560	15.7279	15.8915	15.7405	15.8367	15.7354	15.8377	15.7113	15.9874	15.5201	15.6876	15.7560	15.7279	15.8915	15.7405	15.8367	15.7354	15.8377	15.7113	15.9874	**17.9**
**C1`**	49.8449	50.6489	50.9230	51.1126	51.6517	51.1022	51.5608	51.0735	51.5300	51.1173	51.9938	54.2100	55.1987	55.5796	55.6591	56.1007	55.6493	56.2824	55.6210	56.2091	55.6631	56.4947	**52.4**
**C2`**	25.1051	25.5210	25.7450	25.6658	25.9852	25.6736	25.9823	25.6634	25.9874	25.6536	25.8054	24.9998	25.2327	25.5035	25.2396	25.6627	25.2517	25.5295	25.2468	25.5452	25.2242	25.2410	**14.5**
**C3`**	24.9998	25.2328	25.5035	25.2396	25.6627	25.2517	25.5295	25.2468	25.5452	25.2242	25.2410	25.1051	25.5210	25.7450	25.6658	25.9853	25.6736	25.9824	25.6634	25.9874	25.6536	25.8054	**14.2**
**C4`**	54.2099	55.1986	55.5796	55.6591	56.1007	55.6493	56.2823	55.6210	56.2091	55.6631	56.4947	49.8449	50.6489	50.9230	51.1126	51.6517	51.1022	51.5607	51.0735	51.5300	51.1173	51.9938	**53.9**
**H2**	8.2226	8.0701	8.0263	7.9709	7.9185	7.9810	8.0136	7.9894	7.9826	7.9594	7.9992	8.2226	8.0701	8.0263	7.9709	7.9185	7.9810	8.0136	7.9894	7.9826	7.9594	7.9992	**8.13**
**H5**	7.0052	7.1674	7.2404	7.2314	7.3342	7.2287	7.3618	7.2251	7.3489	7.2331	7.3776	7.0052	7.1674	7.2404	7.2314	7.3342	7.2287	7.3618	7.2251	7.3489	7.2331	7.3776	**8.03**
**H6**	7.7298	7.8332	7.8662	7.8914	7.9483	7.8899	8.0196	7.8872	7.9933	7.8914	8.0281	7.7298	7.8332	7.8662	7.8914	7.9483	7.8899	8.0196	7.8872	7.9933	7.8914	8.0281	**7.4**
**H(3``A-3``C)**	2.3006	2.3428	2.3510	2.3518	2.3659	2.3522	2.3764	2.3519	2.3715	2.3505	2.3813	2.3006	2.3428	2.3510	2.3518	2.3659	2.3522	2.3764	2.3519	2.3715	2.3505	2.3813	**2.32**
**H8**	4.9106	4.9232	4.9071	4.9234	4.9287	4.9245	4.9647	4.9246	4.9490	4.9212	4.9474	4.9106	4.9232	4.9071	4.9234	4.9287	4.9245	4.9647	4.9246	4.9490	4.9212	4.9474	**5.64**
**H(9A-9B)**	1.6061	1.6482	1.6221	1.6801	1.6675	1.6794	1.6867	1.6778	1.6719	1.6798	1.7115	1.6061	1.6482	1.6221	1.6801	1.6675	1.6794	1.6867	1.6778	1.6719	1.6798	1.7115	**1.92**
**H(10A)**	1.0122	0.9629	0.9456	0.9275	0.8788	0.9302	0.9235	0.9322	0.9209	0.9244	0.9279	1.0122	0.9628	0.9456	0.9275	0.8788	0.9302	0.9235	0.9322	0.9209	0.9244	0.9279	**1.26**
**H(10B)**	1.6375	1.5546	1.5068	1.4977	1.4315	1.5023	1.4226	1.5061	1.4293	1.4924	1.3981	1.6375	1.5546	1.5068	1.4977	1.4315	1.5023	1.4226	1.5061	1.4293	1.4924	1.3981	**1.07**
**H(11A-11C)**	0.7026	0.7182	0.6974	0.7270	0.6917	0.7270	0.6980	0.7262	0.6979	0.7263	0.6842	0.7026	0.7182	0.6974	0.7270	0.6917	0.7270	0.6981	0.7262	0.6979	0.7263	0.6842	**0.78**
**H(1`A)**	2.7472	2.7394	2.7126	2.7224	2.6764	2.7242	2.6780	2.7252	2.6813	2.7200	2.6524	2.8879	2.8909	2.8763	2.8749	2.8402	2.8764	2.8367	2.8772	2.8430	2.8728	2.8136	**3.47**
**H(1`B)**	3.2883	3.3208	3.3402	3.3493	3.4003	3.3476	3.3780	3.3453	3.3735	3.3501	3.3732	2.9521	2.8547	2.8367	2.8284	2.8570	2.8290	2.8114	2.8291	2.8174	2.8273	2.8045	**3.63**
**H(4`A)**	2.9521	2.8547	2.8367	2.8284	2.8570	2.8290	2.8114	2.8291	2.8174	2.8273	2.8045	3.2883	3.3208	3.3402	3.3493	3.4003	3.3476	3.3780	3.3453	3.3735	3.3501	3.3732	**3.07**
**H(4`B)**	2.8879	2.8909	2.8763	2.8749	2.8402	2.8764	2.8367	2.8772	2.8430	2.8728	2.8136	2.7472	2.7394	2.7126	2.7224	2.6764	2.7242	2.6780	2.7252	2.6813	2.7200	2.6524	**3.25**
Nuclei	OPW91																						
**C1**	130.4872	132.1627	133.7830	133.0487	135.4642	132.9727	134.1820	132.8880	134.4745	133.1116	134.6156	130.4872	132.1627	133.7830	133.0487	135.4641	132.9727	134.1820	132.8880	134.4745	133.1116	134.6156	**131.615**
**C2**	131.8399	131.8933	132.5753	131.7821	132.4372	131.7986	132.7664	131.7973	132.6340	131.7469	132.7412	131.8400	131.8933	132.5753	131.7821	132.4373	131.7986	132.7664	131.7973	132.6340	131.7469	132.7411	**133.405**
**C3**	124.2485	125.6989	126.5650	126.1025	127.1236	126.0910	127.4911	126.0613	127.3754	126.0935	127.6604	124.2486	125.6989	126.5650	126.1025	127.1236	126.0911	127.4911	126.0613	127.3754	126.0935	127.6605	**126.19**
**C4**	164.6458	165.3503	165.7686	165.5709	166.0012	165.5701	166.6838	165.5507	166.5122	165.5519	166.7513	164.6457	165.3503	165.7686	165.5709	166.0012	165.5700	166.6838	165.5507	166.5122	165.5518	166.7512	**165.04**
**C5**	111.9064	113.0842	113.5160	113.5387	114.1088	113.5251	114.4785	113.4923	114.3344	113.5311	114.6092	111.9064	113.0842	113.5160	113.5387	114.1089	113.5250	114.4785	113.4923	114.3344	113.5311	114.6091	**116.425**
**C6**	123.9800	125.3816	126.1047	126.2632	127.1914	126.2144	127.9521	126.1484	127.7162	126.2901	128.1430	123.9800	125.3816	126.1047	126.2632	127.1914	126.2145	127.9521	126.1484	127.7162	126.2900	128.1431	**129.92**
**C7**	193.3888	198.1862	200.0521	200.8824	202.4617	200.6902	205.9534	200.4907	205.3681	201.0577	207.0692	193.3888	198.1862	200.0521	200.8824	202.4619	200.6902	205.9534	200.4907	205.3681	201.0577	207.0694	**195.9**
**C8**	63.3801	64.9188	65.9103	65.8514	67.1072	65.7773	67.3391	65.6927	67.3318	65.9107	67.7714	63.3801	64.9188	65.9103	65.8514	67.1072	65.7773	67.3391	65.6927	67.3318	65.9107	67.7714	**67.6**
**C9**	35.6884	35.6061	35.7020	35.4965	35.6838	35.5092	35.9565	35.5047	35.8623	35.4660	36.0144	35.6884	35.6061	35.7020	35.4965	35.6839	35.5092	35.9565	35.5047	35.8623	35.4661	36.0144	**32.2**
**C10**	18.4459	19.0380	19.2952	19.3222	19.7305	19.3128	19.6580	19.2859	19.6278	19.3122	19.7884	18.4459	19.0380	19.2952	19.3222	19.7304	19.3128	19.6580	19.2859	19.6278	19.3122	19.7884	**23.4**
**C11**	15.5572	15.7287	15.7985	15.7763	15.9356	15.7785	15.8798	15.7642	15.8814	15.7559	16.0329	15.5572	15.7287	15.7985	15.7763	15.9355	15.7785	15.8798	15.7642	15.8814	15.7559	16.0329	**17.9**
**C1`**	49.9864	50.7893	51.0654	51.2601	51.7938	51.2391	51.7049	51.2009	51.6736	51.2612	52.1407	54.3601	55.3527	55.7326	55.8216	56.2547	55.8013	56.4372	55.7636	56.3648	55.8221	56.6561	**52.4**
**C2`**	25.2222	25.6425	25.8657	25.7984	26.1100	25.7957	26.1072	25.7760	26.1126	25.7823	25.9350	25.1193	25.3596	25.6290	25.3729	25.7903	25.3750	25.6570	25.3613	25.6731	25.3535	25.3721	**14.5**
**C3`**	25.1195	25.3596	25.6290	25.3729	25.7904	25.3751	25.6570	25.3613	25.6731	25.3535	25.3721	25.2221	25.6425	25.8657	25.7984	26.1099	25.7957	26.1072	25.7760	26.1126	25.7824	25.9350	**14.2**
**C4`**	54.3602	55.3527	55.7326	55.8216	56.2547	55.8013	56.4372	55.7636	56.3648	55.8221	56.6560	49.9865	50.7893	51.0654	51.2601	51.7938	51.2392	51.7049	51.2009	51.6736	51.2613	52.1407	**53.9**
**H2**	8.2239	8.0723	8.0276	7.9744	7.9196	7.9837	8.0152	7.9919	7.9838	7.9627	8.0010	8.2239	8.0723	8.0276	7.9744	7.9196	7.9837	8.0152	7.9919	7.9838	7.9627	8.0010	**8.13**
**H5**	7.0076	7.1691	7.2419	7.2337	7.3353	7.2303	7.3630	7.2265	7.3501	7.2351	7.3787	7.0076	7.1691	7.2419	7.2337	7.3353	7.2303	7.3630	7.2265	7.3501	7.2351	7.3787	**8.03**
**H6**	7.7289	7.8330	7.8657	7.8924	7.9477	7.8902	8.0198	7.8872	7.9932	7.8922	8.0288	7.7289	7.8330	7.8657	7.8924	7.9477	7.8902	8.0198	7.8872	7.9932	7.8922	8.0288	**7.4**
**H(3``A-3``C)**	2.3019	2.3440	2.3523	2.3538	2.3670	2.3535	2.3776	2.3529	2.3727	2.3523	2.3825	2.3019	2.3440	2.3523	2.3538	2.3670	2.3535	2.3776	2.3529	2.3727	2.3523	2.3825	**2.32**
**H8**	4.9094	4.9232	4.9062	4.9241	4.9280	4.9245	4.9641	4.9244	4.9483	4.9216	4.9466	4.9094	4.9232	4.9062	4.9241	4.9280	4.9245	4.9641	4.9244	4.9483	4.9216	4.9466	**5.64**
**H(9A-9B)**	1.6027	1.6455	1.6198	1.6791	1.6660	1.6777	1.6855	1.6758	1.6704	1.6786	1.7100	1.6027	1.6455	1.6198	1.6791	1.6659	1.6777	1.6855	1.6758	1.6704	1.6786	1.7100	**1.92**
**H(10A)**	1.0160	0.9672	0.9496	0.9321	0.8824	0.9341	0.9274	0.9359	0.9248	0.9286	0.9318	1.0160	0.9672	0.9496	0.9321	0.8824	0.9341	0.9274	0.9359	0.9248	0.9286	0.9318	**1.26**
**H(10B)**	1.6388	1.5556	1.5086	1.4994	1.4326	1.5033	1.4245	1.5069	1.4311	1.4938	1.3996	1.6388	1.5556	1.5086	1.4994	1.4326	1.5033	1.4245	1.5069	1.4311	1.4938	1.3996	**1.07**
**H(11A-11C)**	0.7047	0.7207	0.7001	0.7303	0.6945	0.7297	0.7010	0.7287	0.7009	0.7294	0.6874	0.7047	0.7207	0.7001	0.7303	0.6945	0.7297	0.7010	0.7287	0.7009	0.7294	0.6874	**0.78**
**H(1`A)**	2.7511	2.7432	2.7164	2.7269	2.6799	2.7279	2.6815	2.7287	2.6847	2.7241	2.6556	2.8918	2.8938	2.8795	2.8782	2.8432	2.8790	2.8395	2.8797	2.8459	2.8758	2.8164	**3.47**
**H(1`B)**	3.2846	3.3175	3.3376	3.3472	3.3979	3.3449	3.3761	3.3424	3.3713	3.3478	3.3710	2.9471	2.8504	2.8333	2.8254	2.8543	2.8253	2.8089	2.8252	2.8147	2.8241	2.8024	**3.63**
**H(4`A)**	2.9471	2.8504	2.8333	2.8254	2.8543	2.8253	2.8089	2.8252	2.8147	2.8241	2.8024	3.2846	3.3175	3.3376	3.3472	3.3979	3.3449	3.3761	3.3424	3.3713	3.3478	3.3709	**3.07**
**H(4`B)**	2.8918	2.8938	2.8795	2.8782	2.8432	2.8791	2.8395	2.8797	2.8459	2.8758	2.8164	2.7511	2.7432	2.7164	2.7269	2.6799	2.7279	2.6815	2.7287	2.6847	2.7241	2.6556	**3.25**

[a] methyl protons shifts are averaged

[b] according to [11]

Table S12. Cont. XI

Nuclei	R											S											Exp. [b]
	PBE																						
	Gas	$CHCl_3$ (PCM)	$CHCl_3$ (SMD)	DMSO (PCM)	DMSO (SMD)	CH_3OH (PCM)	CH_3OH (SMD)	C_2H_5OH (PCM)	C_2H_5OH (SMD)	H_2O (PCM)	H_2O (SMD)	Gas	$CHCl_3$ (PCM)	$CHCl_3$ (SMD)	DMSO (PCM)	DMSO (SMD)	CH_3OH (PCM)	CH_3OH (SMD)	C_2H_5OH (PCM)	C_2H_5OH (SMD)	H_2O (PCM)	H_2O (SMD)	
**C1**	135.4010	136.2845	136.8305	136.8835	138.7209	136.8139	138.7494	136.7616	138.8051	136.9772	139.1750	135.4010	136.2844	136.8304	136.8835	138.7211	136.8139	138.7493	136.7616	138.8051	136.9772	139.1750	**131.615**
**C2**	137.0261	137.0017	137.4032	136.6842	137.4130	136.7058	137.6474	136.7372	137.5601	136.6803	137.6257	137.0261	137.0017	137.4031	136.6842	137.4129	136.7058	137.6475	136.7372	137.5602	136.6803	137.6255	**133.405**
**C3**	130.9970	132.4079	133.3882	132.7609	133.9682	132.7406	134.4025	132.7307	134.3005	132.7969	134.5973	130.9970	132.4078	133.3881	132.7609	133.9682	132.7406	134.4026	132.7307	134.3005	132.7969	134.5971	**126.19**
**C4**	173.1277	174.2088	174.9501	174.6799	175.5039	174.6454	176.2899	174.6222	176.1339	174.7308	176.4161	173.1277	174.2089	174.9502	174.6799	175.5038	174.6454	176.2899	174.6222	176.1339	174.7308	176.4160	**165.04**
**C5**	116.5915	117.8697	118.4805	118.4425	119.3445	118.4031	119.7919	118.3746	119.6678	118.4985	119.9365	116.5915	117.8696	118.4806	118.4425	119.3444	118.4031	119.7919	118.3746	119.6680	118.4985	119.9366	**116.425**
**C6**	129.8918	131.5631	132.5847	132.3833	133.8149	132.3343	134.0958	132.2944	133.8658	132.4455	134.4467	129.8918	131.5631	132.5847	132.3833	133.8148	132.3343	134.0958	132.2944	133.8658	132.4455	134.4466	**129.92**
**C7**	201.2817	205.3783	206.2869	207.7408	208.9029	207.5603	213.5375	207.3982	212.6731	207.9397	214.7818	201.2817	205.3782	206.2868	207.7408	208.9031	207.5603	213.5375	207.3982	212.6733	207.9397	214.7818	**195.9**
**C8**	67.1250	68.3943	68.7255	69.0038	70.1404	68.9496	70.7526	68.9098	70.6063	69.0772	71.3150	67.1250	68.3944	68.7257	69.0038	70.1404	68.9496	70.7527	68.9098	70.6062	69.0772	71.3150	**67.6**
**C9**	40.2194	40.0225	40.2384	39.8491	40.3239	39.8548	40.3308	39.8715	40.2350	39.8612	40.5180	40.2194	40.0226	40.2383	39.8491	40.3239	39.8548	40.3308	39.8715	40.2349	39.8612	40.5180	**32.2**
**C10**	19.9934	20.5540	20.7813	20.7722	21.3631	20.7558	21.2111	20.7500	21.1824	20.8051	21.4177	19.9934	20.5540	20.7813	20.7722	21.3631	20.7558	21.2111	20.7500	21.1825	20.8051	21.4176	**23.4**
**C11**	15.9004	16.1139	16.1849	16.0886	16.3844	16.0874	16.3096	16.0969	16.3049	16.1070	16.5319	15.9004	16.1139	16.1849	16.0886	16.3844	16.0874	16.3096	16.0969	16.3049	16.1070	16.5319	**17.9**
**C1`**	53.2603	54.2097	54.3587	54.6259	55.2663	54.5999	55.1848	54.5843	55.1514	54.6674	55.6879	57.7398	58.9407	59.3490	59.3547	59.9501	59.3273	60.2398	59.3107	60.1763	59.3987	60.4866	**52.4**
**C2`**	25.6456	26.2390	26.4721	26.3909	26.9067	26.3788	26.9324	26.3772	26.9298	26.4201	26.8990	25.3706	25.6351	26.0296	25.7333	26.3613	25.7222	26.3935	25.7221	26.3814	25.7617	26.2009	**14.5**
**C3`**	25.3706	25.6351	26.0296	25.7333	26.3612	25.7222	26.3935	25.7221	26.3814	25.7617	26.2008	25.6456	26.2389	26.4721	26.3909	26.9068	26.3788	26.9323	26.3772	26.9299	26.4201	26.8991	**14.2**
**C4`**	57.7398	58.9407	59.3491	59.3547	59.9501	59.3273	60.2398	59.3107	60.1762	59.3987	60.4867	53.2603	54.2097	54.3587	54.6259	55.2663	54.5999	55.1849	54.5843	55.1514	54.6674	55.6878	**53.9**
**H2**	8.3861	8.3075	8.3460	8.2342	8.2315	8.2413	8.2211	8.2483	8.2176	8.2274	8.2032	8.3861	8.3075	8.3460	8.2342	8.2314	8.2413	8.2211	8.2483	8.2176	8.2274	8.2032	**8.13**
**H5**	6.9781	7.1346	7.2290	7.1971	7.3190	7.1936	7.3430	7.1906	7.3320	7.2010	7.3626	6.9781	7.1346	7.2290	7.1971	7.3190	7.1936	7.3431	7.1906	7.3321	7.2010	7.3626	**8.03**
**H6**	7.7722	7.9285	8.0311	7.9996	8.1059	7.9963	8.1306	7.9933	8.1144	8.0031	8.1521	7.7722	7.9285	8.0311	7.9996	8.1059	7.9963	8.1306	7.9933	8.1144	8.0031	8.1521	**7.4**
**H(3``A-3``C)**	2.3186	2.3670	2.3850	2.3782	2.4062	2.3776	2.4178	2.3776	2.4150	2.3794	2.4234	2.3186	2.3670	2.3850	2.3782	2.4062	2.3776	2.4178	2.3776	2.4150	2.3794	2.4234	**2.32**
**H8**	4.9540	4.9847	5.0510	5.0028	5.0559	5.0023	5.0958	5.0020	5.0823	5.0038	5.0839	4.9540	4.9848	5.0510	5.0028	5.0559	5.0023	5.0958	5.0020	5.0823	5.0038	5.0839	**5.64**
**H(9A-9B)**	1.8410	1.8833	1.8635	1.9019	1.9155	1.9013	1.8587	1.9010	1.8520	1.9028	1.8902	1.8410	1.8833	1.8635	1.9019	1.9155	1.9013	1.8587	1.9010	1.8520	1.9028	1.8902	**1.92**
**H(10A)**	1.0625	0.9818	0.9807	0.9307	0.8674	0.9351	0.9267	0.9398	0.9205	0.9269	0.9362	1.0625	0.9818	0.9807	0.9307	0.8674	0.9351	0.9267	0.9398	0.9205	0.9269	0.9362	**1.26**
**H(10B)**	1.8358	1.7187	1.6709	1.6473	1.5798	1.6516	1.5635	1.6564	1.5706	1.6438	1.5277	1.8358	1.7187	1.6710	1.6473	1.5798	1.6516	1.5635	1.6564	1.5706	1.6438	1.5277	**1.07**
**H(11A-11C)**	0.7527	0.7636	0.7501	0.7651	0.7364	0.7653	0.7362	0.7658	0.7366	0.7656	0.7231	0.7527	0.7636	0.7501	0.7651	0.7364	0.7653	0.7362	0.7658	0.7366	0.7656	0.7231	**0.78**
**H(1`A)**	2.7877	2.7819	2.7641	2.7696	2.7330	2.7704	2.7317	2.7716	2.7338	2.7695	2.7135	2.8910	2.9062	2.8891	2.8946	2.8785	2.8951	2.8892	2.8962	2.8933	2.8948	2.8583	**3.47**
**H(1`B)**	3.3732	3.3999	3.4309	3.4219	3.4901	3.4203	3.4539	3.4193	3.4497	3.4240	3.4531	3.0558	2.8645	2.8252	2.8285	2.8430	2.8286	2.8064	2.8296	2.8036	2.8295	2.7991	**3.63**
**H(4`A)**	3.0558	2.8645	2.8252	2.8285	2.8430	2.8286	2.8064	2.8296	2.8036	2.8295	2.7991	3.3732	3.3999	3.4309	3.4219	3.4901	3.4203	3.4539	3.4193	3.4497	3.4240	3.4530	**3.07**
**H(4`B)**	2.8910	2.9062	2.8891	2.8946	2.8785	2.8951	2.8891	2.8962	2.8933	2.8948	2.8583	2.7877	2.7819	2.7641	2.7696	2.7331	2.7704	2.7317	2.7716	2.7338	2.7695	2.7134	**3.25**
Nuclei	PBE0																						
**C1**	136.2007	136.9147	137.5615	137.3512	138.9409	137.3039	139.2539	137.2630	138.1230	137.4045	139.5381	136.2008	136.9149	137.5615	137.3512	138.9409	137.3041	139.2540	137.2630	138.1230	137.4045	139.5381	**131.615**
**C2**	139.4557	139.3832	139.7713	139.1498	139.6996	139.1641	140.0174	139.1793	139.9414	139.1388	139.9259	139.4558	139.3832	139.7713	139.1499	139.6996	139.1642	140.0177	139.1793	139.9414	139.1388	139.9260	**133.405**
**C3**	131.5821	132.8494	133.7706	133.2264	134.3125	133.2015	134.7612	133.1780	134.5509	133.2529	134.8747	131.5821	132.8495	133.7705	133.2264	134.3126	133.2014	134.7612	133.1780	134.5509	133.2529	134.8748	**126.19**
**C4**	171.5776	172.4460	173.0738	172.8931	173.4817	172.8571	174.3616	172.8237	174.0700	172.9307	174.4196	171.5776	172.4460	173.0739	172.8931	173.4817	172.8571	174.3615	172.8237	174.0700	172.9307	174.4197	**165.04**
**C5**	118.1438	119.3874	119.9921	120.0197	120.8467	119.9746	121.3084	119.9316	121.0125	120.0661	121.4028	118.1437	119.3874	119.9922	120.0197	120.8467	119.9744	121.3085	119.9316	121.0125	120.0661	121.4028	**116.425**
**C6**	132.2745	133.9637	134.9490	134.9149	136.2680	134.8546	136.4989	134.7949	136.7876	134.9732	136.7704	132.2746	133.9636	134.9491	134.9149	136.2680	134.8545	136.4988	134.7949	136.7876	134.9732	136.7705	**129.92**
**C7**	201.3075	205.6698	206.5945	208.1217	208.7899	207.9497	214.6600	207.7847	212.3530	208.2937	215.7870	201.3076	205.6698	206.5945	208.1216	208.7899	207.9497	214.6602	207.7847	212.3530	208.2937	215.7870	**195.9**
**C8**	63.2461	64.4151	64.8221	64.9469	65.8253	64.9073	66.6518	64.8710	66.1021	64.9892	67.0631	63.2461	64.4152	64.8222	64.9469	65.8253	64.9073	66.6518	64.8710	66.1021	64.9891	67.0631	**67.6**
**C9**	36.7732	36.5863	36.7496	36.4519	36.8216	36.4569	36.7620	36.4636	36.8731	36.4500	36.8488	36.7734	36.5862	36.7498	36.4519	36.8216	36.4569	36.7620	36.4636	36.8731	36.4500	36.8488	**32.2**
**C10**	18.2181	18.7218	18.9479	18.9937	19.4770	18.9735	19.3649	18.9547	19.3386	19.0156	19.4816	18.2181	18.7218	18.9478	18.9937	19.4770	18.9735	19.3650	18.9547	19.3386	19.0156	19.4816	**23.4**
**C11**	15.6450	15.7854	15.8700	15.8237	16.0155	15.8164	16.0182	15.8107	15.9753	15.8334	16.1412	15.6450	15.7853	15.8700	15.8237	16.0155	15.8163	16.0182	15.8107	15.9753	15.8334	16.1412	**17.9**
**C1`**	50.4130	51.2513	51.4635	51.6937	52.2179	51.6631	52.2495	51.6338	52.1524	51.7256	52.6385	54.6200	55.6382	55.9981	56.0835	56.5847	56.0504	56.8847	56.0193	56.7286	56.1179	57.0767	**52.4**
**C2`**	24.3359	24.7427	25.0099	24.8926	25.3084	24.8789	25.3847	24.8668	25.3331	24.9084	25.2256	23.8225	23.9894	24.3470	24.1202	24.6026	24.1044	24.7082	24.0909	24.5231	24.1387	24.4528	**14.5**
**C3`**	23.8225	23.9894	24.3470	24.1202	24.6026	24.1044	24.7081	24.0909	24.5231	24.1386	24.4527	24.3359	24.7427	25.0099	24.8926	25.3084	24.8788	25.3847	24.8668	25.3331	24.9084	25.2256	**14.2**
**C4`**	54.6201	55.6382	55.9983	56.0835	56.5847	56.0504	56.8846	56.0193	56.7286	56.1179	57.0766	50.4130	51.2513	51.4635	51.6937	52.2179	51.6630	52.2494	51.6338	52.1524	51.7256	52.6384	**53.9**
**H2**	8.5916	8.5070	8.5428	8.4438	8.4492	8.4490	8.4073	8.4538	8.4910	8.4381	8.3888	8.5916	8.5070	8.5428	8.4438	8.4492	8.4490	8.4074	8.4538	8.4910	8.4381	8.3888	**8.13**
**H5**	7.1345	7.3011	7.3938	7.3709	7.4938	7.3670	7.5145	7.3630	7.5028	7.3742	7.5334	7.1345	7.3011	7.3938	7.3709	7.4938	7.3669	7.5146	7.3630	7.5028	7.3742	7.5334	**8.03**
**H6**	7.8890	8.0526	8.1579	8.1427	8.2720	8.1372	8.2843	8.1317	8.3261	8.1473	8.3064	7.8890	8.0526	8.1579	8.1427	8.2720	8.1372	8.2843	8.1317	8.3261	8.1473	8.3064	**7.4**
**H(3``A-3``C)**	2.3570	2.4017	2.4169	2.4136	2.4343	2.4129	2.4512	2.4123	2.4416	2.4140	2.4546	2.3569	2.4017	2.4169	2.4136	2.4343	2.4129	2.4512	2.4123	2.4416	2.4140	2.4546	**2.32**
**H8**	4.5366	4.5941	4.6639	4.6450	4.7167	4.6413	4.7588	4.6375	4.7612	4.6482	4.7512	4.5366	4.5940	4.6639	4.6450	4.7167	4.6413	4.7588	4.6375	4.7612	4.6482	4.7512	**5.64**
**H(9A-9B)**	1.7007	1.7415	1.7236	1.7637	1.7742	1.7630	1.7035	1.7622	1.7646	1.7637	1.7305	1.7007	1.7415	1.7236	1.7637	1.7742	1.7631	1.7035	1.7622	1.7646	1.7637	1.7305	**1.92**
**H(10A)**	1.0141	0.8933	0.8929	0.8433	0.7729	0.8466	0.8278	0.8497	0.8187	0.8397	0.8291	1.0141	0.8932	0.8928	0.8433	0.7729	0.8466	0.8278	0.8497	0.8187	0.8397	0.8292	**1.26**
**H(10B)**	1.7109	1.6058	1.5536	1.5331	1.4578	1.5374	1.4496	1.5417	1.4660	1.5287	1.4149	1.7108	1.6058	1.5536	1.5331	1.4578	1.5374	1.4496	1.5417	1.4660	1.5287	1.4149	**1.07**
**H(11A-11C)**	0.7296	0.7369	0.7204	0.7415	0.7072	0.7411	0.7083	0.7407	0.7173	0.7415	0.6945	0.7296	0.7369	0.7204	0.7415	0.7072	0.7411	0.7083	0.7407	0.7173	0.7415	0.6945	**0.78**
**H(1`A)**	2.6474	2.6430	2.6269	2.6349	2.5901	2.6354	2.6008	2.6359	2.6033	2.6340	2.5809	2.7682	2.7730	2.7551	2.7579	2.7292	2.7587	2.7564	2.7596	2.7392	2.7568	2.7255	**3.47**
**H(1`B)**	3.1638	3.2018	3.2264	3.2308	3.2894	3.2288	3.2616	3.2268	3.2681	3.2325	3.2635	2.8600	2.6934	2.6410	2.6557	2.6565	2.6568	2.6363	2.6582	2.5968	2.6546	2.6273	**3.63**
**H(4`A)**	2.8601	2.6934	2.6410	2.6557	2.6565	2.6568	2.6363	2.6582	2.5968	2.6546	2.6273	3.1638	3.2018	3.2264	3.2308	3.2894	3.2288	3.2615	3.2268	3.2681	3.2325	3.2634	**3.07**
**H(4`B)**	2.7681	2.7729	2.7551	2.7579	2.7292	2.7587	2.7564	2.7596	2.7392	2.7568	2.7255	2.6473	2.6430	2.6269	2.6349	2.5901	2.6354	2.6007	2.6359	2.6033	2.6340	2.5809	**3.25**

[a] methyl protons shifts are averaged

[b] according to [11]

Table S12. Cont. XII

Nuclei	R											S											Exp. [b]
	TPSS																						
	Gas	$CHCl_3$ (PCM)	$CHCl_3$ (SMD)	DMSO (PCM)	DMSO (SMD)	CH_3OH (PCM)	CH_3OH (SMD)	C_2H_5OH (PCM)	C_2H_5OH (SMD)	H_2O (PCM)	H_2O (SMD)	Gas	$CHCl_3$ (PCM)	$CHCl_3$ (SMD)	DMSO (PCM)	DMSO (SMD)	CH_3OH (PCM)	CH_3OH (SMD)	C_2H_5OH (PCM)	C_2H_5OH (SMD)	H_2O (PCM)	H_2O (SMD)	
**C1**	132.1154	132.6981	133.4308	133.0960	134.7326	133.0650	133.9149	133.0485	133.5528	133.1391	134.1959	132.1154	132.6981	133.4308	133.0960	134.7326	133.0650	133.9149	133.0485	134.1611	133.1391	134.1959	**131.615**
**C2**	134.1276	134.0239	134.4897	133.8755	134.4383	133.8954	134.6701	133.9258	134.4336	133.8666	134.6754	134.1276	134.0239	134.4899	133.8755	134.4383	133.8954	134.6701	133.9258	135.0318	133.8666	134.6754	**133.405**
**C3**	128.4529	129.6100	130.6164	130.0088	131.0505	129.9952	131.4360	129.9925	130.4846	130.0321	131.5560	128.4529	129.6100	130.6165	130.0088	131.0505	129.9952	131.4360	129.9925	131.1280	130.0321	131.5560	**126.19**
**C4**	170.0230	170.8869	171.6752	171.3885	172.0710	171.3619	172.8875	171.3473	172.0048	171.4248	172.9799	170.0230	170.8869	171.6752	171.3885	172.0710	171.3619	172.8875	171.3473	172.1726	171.4248	172.9799	**165.04**
**C5**	113.9299	115.0019	115.6710	115.6200	116.3826	115.5886	116.7059	115.5687	116.0031	115.6603	116.8219	113.9299	115.0019	115.6709	115.6200	116.3826	115.5886	116.7059	115.5687	116.8124	115.6603	116.8219	**116.425**
**C6**	127.3100	128.8826	129.9133	129.8794	131.1746	129.8254	131.9183	129.7821	130.2733	129.9406	132.2412	127.3100	128.8826	129.9131	129.8794	131.1746	129.8254	131.9183	129.7821	130.9192	129.9406	132.2412	**129.92**
**C7**	197.4144	201.1916	202.2671	203.3678	204.2892	203.2251	208.3038	203.0973	203.8818	203.5173	209.3393	197.4144	201.1916	202.2672	203.3678	204.2892	203.2251	208.3038	203.0973	203.6845	203.5174	209.3393	**195.9**
**C8**	65.9602	67.0260	67.5026	67.6065	68.5603	67.5741	68.8519	67.5539	67.7962	67.6488	69.3297	65.9602	67.0260	67.5026	67.6065	68.5603	67.5741	68.8519	67.5539	69.1577	67.6488	69.3297	**67.6**
**C9**	39.0325	38.7522	39.0883	38.7323	39.1760	38.7395	39.3575	38.7582	38.8853	38.7364	39.5264	39.0325	38.7522	39.0883	38.7323	39.1760	38.7395	39.3575	38.7582	40.5780	38.7365	39.5264	**32.2**
**C10**	20.8238	21.1744	21.5479	21.4566	21.9344	21.4466	21.8461	21.4478	21.5057	21.4765	21.9460	20.8239	21.1744	21.5479	21.4566	21.9344	21.4466	21.8461	21.4478	23.3974	21.4765	21.9460	**23.4**
**C11**	16.7586	16.8356	17.0577	16.9276	17.1739	16.9286	17.1255	16.9409	16.9808	16.9372	17.2738	16.7586	16.8356	17.0577	16.9276	17.1739	16.9286	17.1255	16.9409	18.9243	16.9372	17.2738	**17.9**
**C1`**	53.1126	53.8236	54.1272	54.3471	54.8653	54.3207	54.8088	54.3057	54.4950	54.3824	55.2386	57.2363	58.2473	58.7417	58.7163	59.1883	58.6948	59.5114	58.6847	60.3550	58.7473	59.7122	**52.4**
**C2`**	26.3285	26.7518	27.1166	26.9700	27.3956	26.9629	27.4188	26.9674	27.0474	26.9873	27.2584	26.0732	26.1679	26.6933	26.3390	26.8670	26.3320	26.8299	26.3370	28.2499	26.3566	26.6387	**14.5**
**C3`**	26.0732	26.1679	26.6932	26.3390	26.8670	26.3320	26.8299	26.3370	26.4145	26.3566	26.6387	26.3285	26.7518	27.1166	26.9700	27.3956	26.9629	27.4188	26.9674	28.8756	26.9873	27.2584	**14.2**
**C4`**	57.2362	58.2473	58.7417	58.7163	59.1883	58.6948	59.5114	58.6847	58.8915	58.7473	59.7122	53.1126	53.8236	54.1272	54.3471	54.8653	54.3207	54.8088	54.3057	56.0088	54.3824	55.2386	**53.9**
**H2**	8.4087	8.3305	8.3664	8.2797	8.2797	8.2841	8.3180	8.2889	8.1285	8.2757	8.3031	8.4087	8.3305	8.3664	8.2797	8.2797	8.2841	8.3180	8.2889	8.3999	8.2757	8.3031	**8.13**
**H5**	6.9558	7.1161	7.2149	7.1870	7.3101	7.1833	7.3281	7.1801	7.0152	7.1909	7.3417	6.9558	7.1161	7.2149	7.1870	7.3101	7.1833	7.3281	7.1801	7.2995	7.1909	7.3417	**8.03**
**H6**	7.6857	7.8508	7.9491	7.9440	8.0661	7.9386	8.1409	7.9338	7.7719	7.9493	8.1611	7.6857	7.8508	7.9491	7.9440	8.0661	7.9386	8.1409	7.9338	8.0475	7.9493	8.1611	**7.4**
**H(3``A-3``C)**	2.3213	2.3642	2.3852	2.3791	2.4039	2.3785	2.4113	2.3785	2.1943	2.3801	2.4124	2.3213	2.3642	2.3852	2.3791	2.4039	2.3785	2.4113	2.3785	2.5338	2.3801	2.4124	**2.32**
**H8**	4.7279	4.7860	4.8488	4.8333	4.9046	4.8303	4.9639	4.8279	4.6536	4.8365	4.9601	4.7279	4.7860	4.8488	4.8333	4.9046	4.8303	4.9639	4.8279	4.9649	4.8365	4.9601	**5.64**
**H(9A-9B)**	1.7641	1.8094	1.7987	1.8472	1.8696	1.8450	1.8551	1.8434	1.6572	1.8496	1.8860	1.7641	1.8094	1.7987	1.8472	1.8696	1.8450	1.8551	1.8434	2.0028	1.8496	1.8860	**1.92**
**H(10A)**	1.0680	0.9690	0.9904	0.9298	0.8577	0.9329	0.9208	0.9365	1.1106	0.9270	0.9163	1.0681	0.9690	0.9905	0.9298	0.8577	0.9329	0.9208	0.9365	1.1106	0.9270	0.9163	**1.26**
**H(10B)**	1.8095	1.6801	1.6277	1.6073	1.5442	1.6113	1.5335	1.6161	1.9876	1.6040	1.4944	1.8095	1.6801	1.6277	1.6073	1.5442	1.6113	1.5335	1.6161	1.9876	1.6041	1.4944	**1.07**
**H(11A-11C)**	0.7555	0.7642	0.7535	0.7741	0.7461	0.7735	0.7561	0.7735	0.5830	0.7750	0.7414	0.7555	0.7642	0.7535	0.7741	0.7461	0.7735	0.7561	0.7735	0.9409	0.7750	0.7414	**0.78**
**H(1`A)**	2.7026	2.7050	2.6970	2.7012	2.6694	2.7016	2.6755	2.7025	2.6525	2.7012	2.6502	2.7981	2.8151	2.8087	2.8088	2.7947	2.8091	2.7956	2.8100	3.0026	2.8089	2.6502	**3.47**
**H(1`B)**	3.2696	3.3049	3.3388	3.3390	3.4081	3.3368	3.3898	3.3352	3.3248	3.3415	3.3849	3.0280	2.8216	2.7948	2.7819	2.8053	2.7830	2.7365	2.7849	2.8644	2.7816	3.3849	**3.63**
**H(4`A)**	3.0280	2.8216	2.7948	2.7819	2.8053	2.7830	2.7365	2.7849	2.8644	2.7816	2.7356	3.2696	3.3049	3.3388	3.3390	3.4081	3.3368	3.3898	3.3352	3.3248	3.3415	2.7356	**3.07**
**H(4`B)**	2.7981	2.8151	2.8087	2.8088	2.7947	2.8091	2.7956	2.8100	3.0026	2.8089	2.7709	2.7026	2.7050	2.6970	2.7012	2.6694	2.7016	2.6755	2.7025	2.6525	2.7012	2.7709	**3.25**
Nuclei	VSXC																						
**C1**	133.7089	134.0623	134.3904	134.2358	135.9678	134.2244	134.9923	134.2120	135.1654	134.2490	135.3549	133.7090	134.0623	134.3904	134.2358	135.9677	134.2245	134.9923	134.2121	135.1654	134.2490	135.3547	**131.615**
**C2**	131.0275	130.5956	131.3267	130.3155	130.7174	130.3370	131.1024	130.3562	131.0410	130.2964	131.1471	131.0274	130.5957	131.3267	130.3154	130.7174	130.3370	131.1024	130.3562	131.0410	130.2963	131.1472	**133.405**
**C3**	128.2955	129.8523	130.6098	130.3512	131.2930	130.3289	131.5435	130.3036	131.4760	130.3735	131.5973	128.2956	129.8523	130.6098	130.3512	131.2929	130.3289	131.5435	130.3037	131.4760	130.3734	131.5974	**126.19**
**C4**	166.2958	167.2811	167.9398	167.7958	168.4898	167.7669	169.1783	167.7358	169.0055	167.8252	169.3071	166.2958	167.2810	167.9398	167.7958	168.4898	167.7668	169.1783	167.7357	169.0055	167.8250	169.3071	**165.04**
**C5**	111.1342	112.3892	113.0735	113.1249	113.9026	113.0816	114.3287	113.0363	114.1744	113.1678	114.5223	111.1342	112.3892	113.0735	113.1249	113.9026	113.0816	114.3287	113.0362	114.1744	113.1678	114.5224	**116.425**
**C6**	126.0866	127.9182	128.9767	128.9941	130.1063	128.9310	130.7976	128.8655	130.5125	129.0555	131.0866	126.0865	127.9181	128.9767	128.9941	130.1063	128.9310	130.7976	128.8655	130.5125	129.0555	131.0864	**129.92**
**C7**	193.2388	197.7924	198.7855	200.1230	201.1223	199.9824	205.3983	199.8403	204.5527	200.2583	206.5185	193.2389	197.7924	198.7855	200.1229	201.1223	199.9824	205.3983	199.8403	204.5527	200.2584	206.5187	**195.9**
**C8**	71.8578	72.4767	73.0341	72.8352	73.6621	72.8137	73.9188	72.7904	73.8728	72.8577	74.4210	71.8577	72.4767	73.0341	72.8352	73.6621	72.8137	73.9188	72.7904	73.8728	72.8577	74.4210	**67.6**
**C9**	44.2072	44.7806	45.2319	45.0299	45.3275	45.0213	45.9109	45.0098	45.7706	45.0390	46.0933	44.2073	44.7806	45.2319	45.0299	45.3275	45.0213	45.9109	45.0098	45.7706	45.0391	46.0933	**32.2**
**C10**	24.9891	25.8370	26.0231	26.3259	26.7484	26.2987	26.8859	26.2694	26.7937	26.3533	27.0762	24.9890	25.8370	26.0231	26.3259	26.7485	26.2987	26.8859	26.2694	26.7937	26.3533	27.0763	**23.4**
**C11**	20.9345	20.9697	21.0762	20.9972	21.1076	20.9991	21.0676	20.9988	21.0796	20.9968	21.1670	20.9344	20.9696	21.0762	20.9972	21.1076	20.9991	21.0676	20.9988	21.0796	20.9967	21.1670	**17.9**
**C1`**	58.4455	58.9814	59.2906	59.3585	59.8879	59.3368	59.7529	59.3133	59.7237	59.3809	59.9371	61.7124	62.2969	62.6234	62.6433	63.1140	62.6232	62.9800	62.6013	62.9682	62.6642	63.3059	**52.4**
**C2`**	30.5271	31.0155	31.2698	31.2828	31.5322	31.2687	31.5100	31.2525	31.4957	31.2977	31.5937	28.6930	29.1390	29.4794	29.3495	29.7933	29.3402	29.8375	29.3287	29.7984	29.3598	29.6730	**14.5**
**C3`**	28.6930	29.1391	29.4794	29.3494	29.7932	29.3401	29.8375	29.3288	29.7984	29.3598	29.6730	30.5270	31.0155	31.2698	31.2827	31.5320	31.2686	31.5100	31.2526	31.4957	31.2976	31.5935	**14.2**
**C4`**	61.7125	62.2968	62.6234	62.6433	63.1140	62.6233	62.9800	62.6013	62.9682	62.6642	63.3060	58.4456	58.9814	59.2906	59.3585	59.8879	59.3369	59.7529	59.3133	59.7237	59.3809	59.9372	**53.9**
**H2**	8.3247	8.2475	8.3393	8.1901	8.1459	8.1945	8.2457	8.1988	8.2278	8.1858	8.2208	8.3247	8.2475	8.3393	8.1901	8.1460	8.1945	8.2457	8.1988	8.2278	8.1858	8.2208	**8.13**
**H5**	6.9926	7.1606	7.2527	7.2436	7.3628	7.2388	7.3899	7.2341	7.3764	7.2481	7.4107	6.9926	7.1606	7.2527	7.2436	7.3628	7.2388	7.3899	7.2341	7.3764	7.2481	7.4107	**8.03**
**H6**	7.6939	7.8705	7.9640	7.9698	8.0814	7.9637	8.1480	7.9577	8.1203	7.9756	8.1829	7.6939	7.8705	7.9640	7.9698	8.0814	7.9637	8.1480	7.9577	8.1203	7.9756	8.1829	**7.4**
**H(3``A-3``C)**	2.3252	2.4885	2.4814	2.5126	2.5143	2.5122	2.4891	2.5118	2.4963	2.5128	2.4515	2.3252	2.4885	2.4814	2.5126	2.5143	2.5122	2.4891	2.5118	2.4963	2.5128	2.4515	**2.32**
**H8**	4.3190	4.4318	4.4967	4.4928	4.5777	4.4891	4.6335	4.4856	4.6138	4.4963	4.6517	4.3189	4.4318	4.4967	4.4928	4.5777	4.4892	4.6335	4.4856	4.6138	4.4963	4.6517	**5.64**
**H(9A-9B)**	1.8546	1.8821	1.9136	1.9048	1.8818	1.9035	1.8943	1.9024	1.8903	1.9058	1.8918	1.8546	1.8821	1.9136	1.9048	1.8818	1.9035	1.8943	1.9024	1.8903	1.9059	1.8918	**1.92**
**H(10A)**	1.0056	1.0053	0.9968	1.0050	0.9784	1.0053	0.9766	1.0056	0.9872	1.0046	0.9712	1.0056	1.0054	0.9968	1.0049	0.9784	1.0052	0.9766	1.0056	0.9872	1.0046	0.9712	**1.26**
**H(10B)**	2.2840	2.1190	2.0705	1.9916	1.8793	2.0006	1.8503	2.0096	1.8642	1.9829	1.7764	2.2839	2.1190	2.0705	1.9916	1.8793	2.0006	1.8503	2.0096	1.8642	1.9829	1.7764	**1.07**
**H(11A-11C)**	0.9960	1.0168	1.0128	1.0267	0.9955	1.0263	0.9993	1.0259	0.9998	1.0271	0.9922	0.9960	1.0168	1.0128	1.0267	0.9955	1.0263	0.9993	1.0259	0.9998	1.0271	0.9922	**0.78**
**H(1`A)**	2.4656	2.5020	2.5219	2.5184	2.5119	2.5176	2.5318	2.5168	2.5291	2.5192	2.5484	2.9226	2.9016	2.8944	2.8967	2.8818	2.8969	2.8747	2.8972	2.8792	2.8965	2.8890	**3.47**
**H(1`B)**	3.1407	3.1350	3.1797	3.1401	3.2131	3.1396	3.2092	3.1393	3.2014	3.1405	3.2193	2.8613	2.7780	2.8159	2.7513	2.7751	2.7528	2.7295	2.7544	2.7410	2.7499	2.7383	**3.63**
**H(4`A)**	2.8613	2.7780	2.8159	2.7513	2.7751	2.7528	2.7295	2.7544	2.7410	2.7499	2.7384	3.1407	3.1350	3.1797	3.1401	3.2131	3.1396	3.2092	3.1393	3.2014	3.1405	3.2193	**3.07**
**H(4`B)**	2.9226	2.9016	2.8944	2.8967	2.8818	2.8969	2.8747	2.8972	2.8792	2.8965	2.8890	2.4656	2.5020	2.5219	2.5184	2.5119	2.5176	2.5318	2.5168	2.5291	2.5192	2.5484	**3.25**

[a] methyl protons shifts are averaged

[b] according to [11]

Table S12. Cont. XIII

Nuclei	R											S											Exp. [b]
	wB97																						
	Gas	$CHCl_3$ (PCM)	$CHCl_3$ (SMD)	DMSO (PCM)	DMSO (SMD)	CH_3OH (PCM)	CH_3OH (SMD)	C_2H_5OH (PCM)	C_2H_5OH (SMD)	H_2O (PCM)	H_2O (SMD)	Gas	$CHCl_3$ (PCM)	$CHCl_3$ (SMD)	DMSO (PCM)	DMSO (SMD)	CH_3OH (PCM)	CH_3OH (SMD)	C_2H_5OH (PCM)	C_2H_5OH (SMD)	H_2O (PCM)	H_2O (SMD)	
**C1**	136.5685	137.2998	138.0458	137.5481	139.3513	137.5342	139.5815	137.5198	138.2796	137.5620	139.8130	136.5685	137.2999	138.0458	137.5481	139.3513	137.5344	139.5814	137.5197	138.2798	137.5619	139.8131	**131.615**
**C2**	140.4597	140.2803	140.7341	140.1078	140.6099	140.1179	140.8866	140.1271	140.9111	140.0988	140.8928	140.4597	140.2804	140.7341	140.1080	140.6099	140.1179	140.8868	140.1271	140.9110	140.0988	140.8929	**133.405**
**C3**	131.8491	132.8916	133.7579	133.2076	134.2702	133.1912	134.5709	133.1736	134.3426	133.2232	134.7015	131.8491	132.8916	133.7580	133.2076	134.2702	133.1911	134.5710	133.1736	134.3428	133.2231	134.7015	**126.19**
**C4**	172.6725	173.3200	173.9430	173.7031	174.2581	173.6773	174.6970	173.6509	174.7453	173.7281	174.8004	172.6725	173.3199	173.9431	173.7031	174.2580	173.6773	174.6971	173.6509	174.7452	173.7280	174.8004	**165.04**
**C5**	119.2416	120.4446	121.0584	121.0792	121.9447	121.0392	122.1559	120.9986	121.9530	121.1175	122.2990	119.2416	120.4446	121.0583	121.0792	121.9447	121.0392	122.1559	120.9986	121.9529	121.1175	122.2989	**116.425**
**C6**	133.7353	135.2434	136.1565	136.2387	137.5154	136.1726	137.6767	136.1065	137.9970	136.3022	138.0135	133.7353	135.2434	136.1565	136.2388	137.5153	136.1727	137.6765	136.1065	137.9968	136.3022	138.0133	**129.92**
**C7**	201.8010	206.7178	207.7402	209.2842	209.8800	209.1261	216.5903	208.9682	213.8414	209.4353	217.6960	201.8010	206.7175	207.7402	209.2842	209.8800	209.1262	216.5903	208.9677	213.8416	209.4353	217.6961	**195.9**
**C8**	61.8342	62.9820	63.4739	63.4435	64.2461	63.4181	65.1654	63.3917	64.4408	63.4676	65.4934	61.8342	62.9819	63.4739	63.4435	64.2461	63.4181	65.1654	63.3916	64.4408	63.4677	65.4934	**67.6**
**C9**	35.0243	34.8332	34.9958	34.6955	35.0458	34.7049	34.7826	34.7132	34.9627	34.6866	34.9646	35.0243	34.8332	34.9958	34.6955	35.0458	34.7049	34.7825	34.7132	34.9626	34.6865	34.9646	**32.2**
**C10**	18.2027	18.6531	18.8496	18.9099	19.3911	18.8932	19.2054	18.8758	19.1873	18.9260	19.3370	18.2028	18.6532	18.8496	18.9099	19.3910	18.8932	19.2054	18.8758	19.1872	18.9260	19.3371	**23.4**
**C11**	15.6995	15.7560	15.8801	15.8139	16.0215	15.8087	15.9265	15.8027	15.9471	15.8194	16.0957	15.6995	15.7560	15.8800	15.8140	16.0215	15.8087	15.9264	15.8027	15.9470	15.8194	16.0956	**17.9**
**C1`**	49.2258	49.8232	50.0620	50.2647	50.7891	50.2356	50.7958	50.2058	50.7027	50.2928	51.1399	52.8945	53.7467	54.0785	54.1592	54.6644	54.1319	54.8314	54.1044	54.6995	54.1857	55.1043	**52.4**
**C2`**	23.7756	24.1041	24.3533	24.2614	24.6818	24.2513	24.7299	24.2406	24.6529	24.2714	24.6392	23.1903	23.2947	23.6379	23.4229	23.9010	23.4127	23.8543	23.4017	23.8006	23.4334	23.7318	**14.5**
**C3`**	23.1903	23.2948	23.6380	23.4230	23.9011	23.4126	23.8544	23.4018	23.8006	23.4333	23.7318	23.7755	24.1041	24.3533	24.2613	24.6818	24.2513	24.7299	24.2406	24.6529	24.2714	24.6391	**14.2**
**C4`**	52.8945	53.7466	54.0785	54.1592	54.6643	54.1320	54.8313	54.1043	54.6996	54.1857	55.1043	49.2259	49.8232	50.0619	50.2647	50.7891	50.2355	50.7957	50.2057	50.7028	50.2929	51.1399	**53.9**
**H2**	8.6809	8.5744	8.6075	8.5183	8.5204	8.5212	8.4545	8.5240	8.5726	8.5156	8.4553	8.6809	8.5744	8.6075	8.5183	8.5204	8.5212	8.4545	8.5240	8.5726	8.5157	8.4553	**8.13**
**H5**	7.2542	7.4280	7.5179	7.5041	7.6322	7.4996	7.6357	7.4950	7.6366	7.5084	7.6595	7.2542	7.4280	7.5179	7.5041	7.6322	7.4996	7.6357	7.4950	7.6366	7.5084	7.6595	**8.03**
**H6**	7.9477	8.1022	8.2025	8.2030	8.3341	8.1962	8.3494	8.1894	8.3856	8.2096	8.3807	7.9477	8.1022	8.2025	8.2030	8.3342	8.1962	8.3494	8.1894	8.3856	8.2096	8.3807	**7.4**
**H(3``A-3``C)**	2.3549	2.3976	2.4124	2.4103	2.4336	2.4095	2.4577	2.4087	2.4403	2.4111	2.4663	2.3549	2.3976	2.4124	2.4103	2.4336	2.4096	2.4577	2.4087	2.4403	2.4111	2.4663	**2.32**
**H8**	4.2034	4.2712	4.3375	4.3387	4.4228	4.3334	4.4729	4.3282	4.4740	4.3440	4.4832	4.2034	4.2712	4.3375	4.3387	4.4227	4.3334	4.4729	4.3282	4.4740	4.3440	4.4832	**5.64**
**H(9A-9B)**	1.6611	1.7015	1.6840	1.7245	1.7275	1.7231	1.6535	1.7217	1.7210	1.7258	1.6931	1.6611	1.7015	1.6840	1.7245	1.7275	1.7231	1.6535	1.7217	1.7210	1.7258	1.6931	**1.92**
**H(10A)**	1.0493	0.8934	0.8871	0.8401	0.7823	0.8426	0.8036	0.8451	0.8313	0.8378	0.8170	1.0493	0.8934	0.8871	0.8401	0.7823	0.8426	0.8036	0.8451	0.8313	0.8378	0.8170	**1.26**
**H(10B)**	1.6872	1.5804	1.5359	1.5046	1.4271	1.5090	1.4210	1.5134	1.4409	1.5005	1.3992	1.6872	1.5804	1.5359	1.5046	1.4271	1.5090	1.4210	1.5134	1.4409	1.5005	1.3992	**1.07**
**H(11A-11C)**	0.7650	0.7661	0.7469	0.7725	0.7383	0.7717	0.7282	0.7709	0.7482	0.7734	0.7234	0.7650	0.7661	0.7469	0.7725	0.7383	0.7717	0.7282	0.7710	0.7482	0.7734	0.7234	**0.78**
**H(1`A)**	2.6078	2.6030	2.5890	2.5986	2.5571	2.5991	2.5722	2.5994	2.5745	2.5982	2.5473	2.7476	2.7543	2.7398	2.7341	2.7058	2.7357	2.7330	2.7371	2.7124	2.7326	2.7012	**3.47**
**H(1`B)**	3.0403	3.0692	3.0961	3.1040	3.1621	3.1014	3.1512	3.0988	3.1494	3.1065	3.1559	2.7254	2.5997	2.5452	2.5525	2.5629	2.5554	2.5567	2.5580	2.5054	2.5499	2.5531	**3.63**
**H(4`A)**	2.7253	2.5997	2.5452	2.5525	2.5629	2.5554	2.5567	2.5580	2.5054	2.5499	2.5531	3.0403	3.0692	3.0961	3.1039	3.1621	3.1014	3.1512	3.0988	3.1494	3.1065	3.1559	**3.07**
**H(4`B)**	2.7476	2.7543	2.7398	2.7341	2.7058	2.7357	2.7330	2.7371	2.7124	2.7326	2.7012	2.6078	2.6030	2.5889	2.5986	2.5571	2.5991	2.5722	2.5994	2.5745	2.5982	2.5473	**3.25**
Nuclei	wB97X																						
**C1**	137.9167	138.7291	139.4027	139.1862	140.8400	139.1410	141.1404	139.0995	139.8806	139.2327	141.3741	137.9167	138.7291	139.4025	139.1862	140.8401	139.1410	141.1404	139.0995	139.8809	139.2327	141.3740	**131.615**
**C2**	141.0494	140.9138	141.3464	140.7659	141.2763	140.7779	141.5666	140.7886	141.5609	140.7548	141.5438	141.0494	140.9138	141.3464	140.7660	141.2763	140.7779	141.5667	140.7886	141.5608	140.7550	141.5435	**133.405**
**C3**	132.8983	133.9883	134.8595	134.3501	135.3910	134.3308	135.7372	134.3109	135.4769	134.3691	135.8600	132.8983	133.9883	134.8594	134.3501	135.3908	134.3308	135.7371	134.3109	135.4769	134.3693	135.8600	**126.19**
**C4**	173.4290	174.0925	174.7321	174.4425	175.0116	174.4248	175.4899	174.4055	175.4614	174.4603	175.5807	173.4290	174.0925	174.7321	174.4425	175.0116	174.4248	175.4899	174.4055	175.4614	174.4604	175.5807	**165.04**
**C5**	119.9239	121.1094	121.7143	121.7414	122.5798	121.7041	122.8240	121.6659	122.5887	121.7782	122.9578	119.9239	121.1094	121.7143	121.7415	122.5798	121.7041	122.8240	121.6659	122.5887	121.7781	122.9578	**116.425**
**C6**	134.1177	135.6553	136.6069	136.6152	137.9383	136.5613	138.1354	136.5056	138.3862	136.6677	138.4562	134.1177	135.6553	136.6068	136.6153	137.9383	136.5613	138.1354	136.5056	138.3860	136.6675	138.4561	**129.92**
**C7**	203.4902	208.4617	209.3787	211.1218	211.5950	210.9424	218.2752	210.7679	215.5453	211.2963	219.3749	203.4902	208.4617	209.3790	211.1218	211.5948	210.9424	218.2752	210.7679	215.5457	211.2960	219.3747	**195.9**
**C8**	63.1447	64.2837	64.7138	64.7495	65.5120	64.7219	66.5338	64.6951	65.6891	64.7782	66.8394	63.1447	64.2837	64.7138	64.7495	65.5120	64.7218	66.5338	64.6951	65.6891	64.7781	66.8394	**67.6**
**C9**	35.8867	35.6553	35.7926	35.4652	35.8043	35.4843	35.6574	35.5015	35.6816	35.4482	35.7980	35.8867	35.6553	35.7927	35.4652	35.8045	35.4842	35.6574	35.5015	35.6816	35.4481	35.7980	**32.2**
**C10**	18.3267	18.7758	18.9867	19.0483	19.5211	19.0352	19.3979	19.0208	19.3074	19.0616	19.5186	18.3267	18.7758	18.9866	19.0483	19.5211	19.0352	19.3980	19.0208	19.3074	19.0616	19.5186	**23.4**
**C11**	15.7170	15.7824	15.9009	15.8356	16.0411	15.8344	15.9850	15.8321	15.9413	15.8373	16.1378	15.7170	15.7825	15.9008	15.8355	16.0412	15.8345	15.9851	15.8322	15.9413	15.8372	16.1378	**17.9**
**C1`**	49.9816	50.6681	50.9209	51.1061	51.6482	51.0811	51.6812	51.0548	51.5477	51.1309	52.0378	53.9090	54.7912	55.1324	55.2314	55.7325	55.2038	55.9347	55.1762	55.7634	55.2594	56.1763	**52.4**
**C2`**	24.2890	24.5786	24.8405	24.7246	25.1372	24.7177	25.2136	24.7101	25.0923	24.7322	25.1028	23.6801	23.7770	24.1376	23.9227	24.4125	23.9135	24.4023	23.9035	24.2810	23.9324	24.2369	**14.5**
**C3`**	23.6801	23.7770	24.1375	23.9227	24.4125	23.9135	24.4023	23.9035	24.2810	23.9324	24.2370	24.2890	24.5786	24.8404	24.7246	25.1372	24.7177	25.2137	24.7101	25.0924	24.7322	25.1028	**14.2**
**C4`**	53.9090	54.7912	55.1324	55.2314	55.7324	55.2039	55.9348	55.1762	55.7634	55.2594	56.1763	49.9816	50.6681	50.9208	51.1061	51.6481	51.0810	51.6813	51.0548	51.5477	51.1309	52.0377	**53.9**
**H2**	8.6573	8.5506	8.5924	8.4873	8.5035	8.4927	8.4297	8.4977	8.5448	8.4820	8.4250	8.6573	8.5506	8.5924	8.4873	8.5035	8.4927	8.4297	8.4977	8.5447	8.4820	8.4250	**8.13**
**H5**	7.2317	7.4023	7.4917	7.4772	7.6027	7.4732	7.6054	7.4691	7.6042	7.4810	7.6267	7.2317	7.4023	7.4917	7.4772	7.6027	7.4732	7.6054	7.4691	7.6042	7.4810	7.6267	**8.03**
**H6**	7.9266	8.0749	8.1830	8.1696	8.3042	8.1642	8.3142	8.1586	8.3490	8.1751	8.3438	7.9266	8.0749	8.1830	8.1696	8.3042	8.1642	8.3142	8.1586	8.3490	8.1751	8.3438	**7.4**
**H(3``A-3``C)**	2.3514	2.3974	2.4118	2.4128	2.4345	2.4120	2.4511	2.4111	2.4400	2.4138	2.4563	2.3514	2.3974	2.4118	2.4128	2.4345	2.4120	2.4511	2.4112	2.4400	2.4138	2.4563	**2.32**
**H8**	4.2964	4.3549	4.4302	4.4201	4.5025	4.4155	4.5433	4.4109	4.5481	4.4247	4.5434	4.2964	4.3549	4.4302	4.4201	4.5025	4.4155	4.5433	4.4109	4.5481	4.4247	4.5434	**5.64**
**H(9A-9B)**	1.6561	1.6953	1.6759	1.7062	1.7129	1.7071	1.6447	1.7075	1.6979	1.7055	1.6780	1.6561	1.6953	1.6760	1.7062	1.7129	1.7071	1.6447	1.7075	1.6978	1.7055	1.6780	**1.92**
**H(10A)**	1.0286	0.8865	0.8872	0.8404	0.7833	0.8434	0.8083	0.8463	0.8280	0.8376	0.8194	1.0286	0.8865	0.8872	0.8404	0.7833	0.8434	0.8083	0.8463	0.8280	0.8376	0.8194	**1.26**
**H(10B)**	1.6766	1.5661	1.5187	1.4915	1.4167	1.4961	1.4105	1.5007	1.4224	1.4873	1.3852	1.6766	1.5661	1.5187	1.4915	1.4167	1.4961	1.4105	1.5007	1.4224	1.4873	1.3852	**1.07**
**H(11A-11C)**	0.7565	0.7582	0.7409	0.7657	0.7337	0.7653	0.7245	0.7649	0.7398	0.7662	0.7170	0.7565	0.7582	0.7409	0.7657	0.7337	0.7653	0.7245	0.7649	0.7398	0.7662	0.7170	**0.78**
**H(1`A)**	2.6307	2.6257	2.6109	2.6226	2.5779	2.6232	2.5929	2.6237	2.5918	2.6222	2.5692	2.7666	2.7694	2.7512	2.7519	2.7214	2.7532	2.7532	2.7546	2.7278	2.7508	2.7188	**3.47**
**H(1`B)**	3.0645	3.0957	3.1234	3.1296	3.1868	3.1272	3.1722	3.1249	3.1715	3.1320	3.1753	2.7371	2.6172	2.5571	2.5764	2.5746	2.5784	2.5686	2.5805	2.5209	2.5748	2.5617	**3.63**
**H(4`A)**	2.7371	2.6172	2.5571	2.5764	2.5746	2.5784	2.5687	2.5805	2.5209	2.5748	2.5617	3.0645	3.0957	3.1234	3.1296	3.1868	3.1272	3.1722	3.1249	3.1715	3.1320	3.1753	**3.07**
**H(4`B)**	2.7666	2.7694	2.7513	2.7519	2.7214	2.7532	2.7533	2.7546	2.7278	2.7508	2.7188	2.6307	2.6257	2.6109	2.6226	2.5779	2.6232	2.5929	2.6237	2.5918	2.6222	2.5692	**3.25**

[a] methyl protons shifts are averaged

[b] according to [11]

Table S12. Cont. XIV

Nuclei	R											S											Exp. [b]
	X3LYP																						
	Gas	$CHCl_3$ (PCM)	$CHCl_3$ (SMD)	DMSO (PCM)	DMSO (SMD)	CH_3OH (PCM)	CH_3OH (SMD)	C_2H_5OH (PCM)	C_2H_5OH (SMD)	H_2O (PCM)	H_2O (SMD)	Gas	$CHCl_3$ (PCM)	$CHCl_3$ (SMD)	DMSO (PCM)	DMSO (SMD)	CH_3OH (PCM)	CH_3OH (SMD)	C_2H_5OH (PCM)	C_2H_5OH (SMD)	H_2O (PCM)	H_2O (SMD)	
**C1**	139.4041	140.0223	140.8129	140.5269	143.1312	140.4811	142.1658	140.4364	142.3764	140.5810	142.5466	139.4041	140.0223	140.8128	140.5269	143.1313	140.4811	142.1656	140.4364	142.3764	140.5811	142.5465	**131.615**
**C2**	140.5500	140.4125	140.7742	140.2073	140.7052	140.2265	141.0797	140.2422	140.9813	140.1926	141.0557	140.5502	140.4126	140.7740	140.2075	140.7050	140.2265	141.0797	140.2422	140.9813	140.1927	141.0559	**133.405**
**C3**	134.0323	135.1799	136.0888	135.5425	136.5779	135.5274	136.9165	135.5089	136.8432	135.5602	137.0649	134.0323	135.1799	136.0888	135.5424	136.5778	135.5274	136.9165	135.5089	136.8432	135.5603	137.0650	**126.19**
**C4**	175.0095	175.9667	176.6937	176.5244	177.1874	176.4907	177.8944	176.4543	177.7179	176.5608	177.9908	175.0093	175.9669	176.6938	176.5244	177.1874	176.4907	177.8944	176.4543	177.7179	176.5609	177.9907	**165.04**
**C5**	119.2391	120.4282	121.0066	121.0986	121.9322	121.0585	122.2322	121.0162	122.1290	121.1412	122.3575	119.2391	120.4281	121.0067	121.0986	121.9323	121.0585	122.2323	121.0162	122.1290	121.1413	122.3575	**116.425**
**C6**	133.4957	135.1735	136.1169	136.1069	136.9788	136.0579	137.7358	136.0047	137.4435	136.1542	138.0023	133.4956	135.1735	136.1169	136.1067	136.9787	136.0579	137.7358	136.0047	137.4435	136.1543	138.0022	**129.92**
**C7**	204.0680	208.6018	209.7906	211.2857	212.8613	211.1040	217.7497	210.9238	216.7862	211.4694	218.9551	204.0677	208.6014	209.7908	211.2857	212.8614	211.1040	217.7499	210.9238	216.7862	211.4694	218.9552	**195.9**
**C8**	66.0484	67.1131	67.4097	67.6924	68.8127	67.6555	69.2078	67.6167	69.1583	67.7330	69.6317	66.0484	67.1131	67.4096	67.6925	68.8127	67.6555	69.2079	67.6167	69.1583	67.7330	69.6317	**67.6**
**C9**	38.8515	38.4537	38.6398	38.3061	38.4752	38.3194	38.5849	38.3296	38.5301	38.2961	38.6638	38.8516	38.4537	38.6397	38.3060	38.4753	38.3194	38.5849	38.3296	38.5301	38.2962	38.6638	**32.2**
**C10**	19.8530	20.2980	20.5805	20.5917	21.0678	20.5777	20.9428	20.5605	20.9327	20.6084	21.0515	19.8530	20.2981	20.5805	20.5918	21.0678	20.5777	20.9429	20.5605	20.9327	20.6084	21.0515	**23.4**
**C11**	16.1311	16.2611	16.4069	16.3426	16.5437	16.3421	16.4790	16.3381	16.4858	16.3464	16.6472	16.1310	16.2611	16.4069	16.3427	16.5437	16.3421	16.4792	16.3381	16.4858	16.3464	16.6472	**17.9**
**C1`**	52.1870	53.0482	53.2659	53.5913	54.1404	53.5638	54.0666	53.5327	54.0389	53.6206	54.4972	56.7023	57.7867	58.1582	58.2874	58.7947	58.2596	58.9712	58.2291	58.9152	58.3175	59.2028	**52.4**
**C2`**	25.9147	26.3743	26.6079	26.5848	26.9763	26.5752	27.0117	26.5626	27.0228	26.5976	26.9096	25.5699	25.7049	26.1291	25.8844	26.4747	25.8740	26.4010	25.8609	26.3930	25.8987	26.1762	**14.5**
**C3`**	25.5699	25.7049	26.1291	25.8844	26.4747	25.8740	26.4010	25.8609	26.3930	25.8987	26.1764	25.9147	26.3743	26.6078	26.5849	26.9763	26.5752	27.0118	26.5626	27.0228	26.5976	26.9097	**14.2**
**C4`**	56.7024	57.7867	58.1582	58.2873	58.7947	58.2596	58.9712	58.2291	58.9152	58.3175	59.2028	52.1869	53.0481	53.2660	53.5913	54.1403	53.5638	54.0664	53.5327	54.0389	53.6205	54.4973	**53.9**
**H2**	8.5259	8.4459	8.4741	8.3825	8.3137	8.3882	8.3541	8.3930	8.3416	8.3765	8.3347	8.5259	8.4459	8.4741	8.3825	8.3136	8.3882	8.3541	8.3930	8.3416	8.3765	8.3347	**8.13**
**H5**	7.1012	7.2586	7.3480	7.3284	7.4453	7.3246	7.4665	7.3203	7.4558	7.3319	7.4859	7.1013	7.2586	7.3480	7.3284	7.4453	7.3246	7.4665	7.3203	7.4558	7.3319	7.4859	**8.03**
**H6**	7.7917	7.9695	8.0706	8.0644	8.1439	8.0593	8.2106	8.0535	8.1847	8.0691	8.2342	7.7917	7.9695	8.0706	8.0644	8.1439	8.0593	8.2106	8.0535	8.1847	8.0691	8.2342	**7.4**
**H(3``A-3``C)**	2.3710	2.4141	2.4315	2.4306	2.4536	2.4298	2.4622	2.4285	2.4596	2.4314	2.4669	2.3711	2.4141	2.4316	2.4306	2.4536	2.4298	2.4622	2.4285	2.4596	2.4314	2.4669	**2.32**
**H8**	4.5908	4.6521	4.7228	4.6993	4.7495	4.6964	4.8055	4.6929	4.7844	4.7019	4.7930	4.5908	4.6521	4.7228	4.6993	4.7495	4.6964	4.8055	4.6929	4.7844	4.7019	4.7930	**5.64**
**H(9A-9B)**	1.6700	1.7186	1.7006	1.7470	1.7128	1.7462	1.7045	1.7448	1.6977	1.7474	1.7295	1.6700	1.7186	1.7006	1.7470	1.7128	1.7462	1.7045	1.7448	1.6977	1.7474	1.7295	**1.92**
**H(10A)**	1.0788	0.9678	0.9721	0.9234	0.8482	0.9270	0.9015	0.9298	0.8938	0.9200	0.9082	1.0788	0.9678	0.9721	0.9234	0.8482	0.9270	0.9015	0.9298	0.8938	0.9200	0.9082	**1.26**
**H(10B)**	1.7441	1.6373	1.5950	1.5671	1.4883	1.5715	1.4882	1.5754	1.4926	1.5630	1.4528	1.7441	1.6374	1.5950	1.5672	1.4883	1.5715	1.4882	1.5754	1.4926	1.5630	1.4528	**1.07**
**H(11A-11C)**	0.7775	0.7888	0.7748	0.7980	0.7572	0.7975	0.7642	0.7966	0.7625	0.7983	0.7520	0.7775	0.7888	0.7748	0.7980	0.7572	0.7975	0.7642	0.7966	0.7625	0.7983	0.7520	**0.78**
**H(1`A)**	2.7254	2.7159	2.7001	2.7073	2.6580	2.7081	2.6654	2.7083	2.6679	2.7065	2.6483	2.8459	2.8443	2.8242	2.8320	2.8124	2.8329	2.8185	2.8334	2.8246	2.8312	2.7903	**3.47**
**H(1`B)**	3.1908	3.2308	3.2685	3.2671	3.3265	3.2649	3.3058	3.2623	3.2999	3.2690	3.3086	2.8710	2.6984	2.6765	2.6732	2.7143	2.6736	2.6619	2.6738	2.6654	2.6730	2.6605	**3.63**
**H(4`A)**	2.8709	2.6984	2.6765	2.6732	2.7143	2.6736	2.6619	2.6738	2.6654	2.6730	2.6605	3.1908	3.2308	3.2685	3.2671	3.3264	3.2649	3.3057	3.2623	3.2999	3.2690	3.3086	**3.07**
**H(4`B)**	2.8459	2.8443	2.8242	2.8320	2.8124	2.8329	2.8185	2.8334	2.8246	2.8312	2.7903	2.7254	2.7159	2.7001	2.7073	2.6580	2.7081	2.6654	2.7083	2.6679	2.7066	2.6483	**3.25**

[a] methyl protons shifts are averaged

[b] according to [11]

Table S13. Theoretical chemical shifts (in ppm) of *(R,S)*-4-fluoro-α-pyrrolidinooctanophenone calculated with HF and selected functionals combined with 6-311++G** basis set[a]

Nuclei	R											S											Exp. [b]
	B3LYP																						
	Gas	$CHCl_3$ (PCM)	$CHCl_3$ (SMD)	DMSO (PCM)	DMSO (SMD)	CH_3OH (PCM)	CH_3OH (SMD)	C_2H_5OH (PCM)	C_2H_5OH (SMD)	H_2O (PCM)	H_2O (SMD)	Gas	$CHCl_3$ (PCM)	$CHCl_3$ (SMD)	DMSO (PCM)	DMSO (SMD)	CH_3OH (PCM)	CH_3OH (SMD)	C_2H_5OH (PCM)	C_2H_5OH (SMD)	H_2O (PCM)	H_2O (SMD)	
**C1**	139.7440	140.6555	142.0342	141.4602	143.3098	141.3148	142.4277	141.1275	142.5152	141.5693	142.9951	139.7439	140.6555	142.0341	141.4601	143.3098	141.3148	142.4277	141.1275	142.5152	141.5693	142.9952	**131.79**
**C2**	139.7404	139.8313	140.3450	139.5953	140.0872	139.6159	140.5636	139.6375	140.4349	139.5753	140.4849	139.7404	139.8313	140.3449	139.5953	140.0874	139.6158	140.5635	139.6375	140.4348	139.5751	140.4849	**131.93**
**C3**	122.0847	122.7996	123.3543	122.9484	123.5596	122.9391	123.8685	122.9308	123.7838	122.9561	123.8939	122.0848	122.7996	123.3542	122.9484	123.5597	122.9391	123.8685	122.9307	123.7837	122.9562	123.8939	**116.53**
**C4**	176.3853	177.2381	177.8754	177.5510	178.2024	177.5430	178.8558	177.5369	178.6824	177.5595	178.9299	176.3853	177.2381	177.8753	177.5509	178.2023	177.5430	178.8558	177.5369	178.6825	177.5595	178.9301	**168.1**
**C5**	119.8360	121.1437	121.7586	121.8151	122.5580	121.7557	122.8483	121.6924	122.7230	121.8667	123.0200	119.8360	121.1437	121.7585	121.8152	122.5580	121.7557	122.8483	121.6924	122.7232	121.8666	123.0200	**116.51**
**C6**	135.5687	137.4684	138.3866	138.4288	139.8539	138.3931	140.6351	138.3688	140.3514	138.4701	140.8634	135.5687	137.4684	138.3867	138.4288	139.8539	138.3930	140.6351	138.3688	140.3514	138.4700	140.8633	**131.88**
**C7**	203.6186	208.4436	210.2930	211.5325	212.4799	211.2330	217.4623	210.8895	216.3461	211.7872	218.9247	203.6186	208.4436	210.2931	211.5325	212.4799	211.2330	217.4623	210.8895	216.3462	211.7867	218.9249	**195.2**
**C8**	66.5090	67.7289	68.5733	68.3407	69.3202	68.2691	69.9581	68.1991	69.7954	68.4042	70.3423	66.5090	67.7289	68.5733	68.3407	69.3202	68.2691	69.9581	68.1991	69.7954	68.4042	70.3423	**54.87**
**C9**	37.1546	36.8932	36.9132	36.5849	36.8580	36.6191	37.0537	36.6673	36.9711	36.5582	37.0637	37.1546	36.8932	36.9132	36.5849	36.8580	36.6191	37.0537	36.6673	36.9710	36.5582	37.0638	**31.13**
**C10**	27.6112	28.1809	28.4922	28.3496	28.9997	28.3440	28.8458	28.3444	28.8208	28.3576	28.9233	27.6112	28.1809	28.4922	28.3497	28.9997	28.3440	28.8458	28.3444	28.8208	28.3576	28.9233	**30.82**
**C11**	37.1857	37.3183	37.6583	37.1831	37.7456	37.1935	37.7322	37.2080	37.7006	37.1728	37.8968	37.1857	37.3183	37.6582	37.1830	37.7456	37.1936	37.7322	37.2080	37.7007	37.1729	37.8968	**29.11**
**C12**	38.8215	39.1603	39.4656	39.3341	39.8093	39.3151	39.7210	39.2941	39.6945	39.3497	39.7747	38.8216	39.1603	39.4657	39.3340	39.8094	39.3151	39.7210	39.2941	39.6944	39.3497	39.7748	**25.52**
**C13**	29.8649	30.1122	30.3311	30.1260	30.4232	30.1224	30.4388	30.1191	30.4109	30.1278	30.4278	29.8650	30.1122	30.3312	30.1260	30.4231	30.1225	30.4388	30.1191	30.4109	30.1278	30.4278	**22.36**
**C14**	16.8464	17.0714	17.1281	17.1359	17.2139	17.1263	17.2234	17.1162	17.1938	17.1433	17.2338	16.8464	17.0714	17.1282	17.1359	17.2138	17.1263	17.2234	17.1162	17.1938	17.1432	17.2338	**13.85**
**C1`**	52.4430	53.2501	53.5796	53.7270	54.2978	53.6868	54.2682	53.6444	54.1816	53.7622	54.7742	56.8713	57.8585	58.3021	58.3786	58.8622	58.3368	59.1280	58.2937	59.0180	58.4156	59.4453	**51.23**
**C2`**	26.0864	26.6096	26.9407	26.7529	27.2069	26.7417	27.3157	26.7336	27.2752	26.7638	27.1542	25.8012	26.1795	26.5397	26.2972	26.8405	26.2786	26.7254	26.2545	26.7083	26.3104	26.5119	**23.85**
**C3`**	25.8012	26.1795	26.5398	26.2972	26.8405	26.2785	26.7254	26.2545	26.7084	26.3104	26.5120	26.0864	26.6096	26.9407	26.7529	27.2068	26.7417	27.3157	26.7336	27.2752	26.7638	27.1542	**23.66**
**C4`**	56.8713	57.8585	58.3021	58.3786	58.8622	58.3369	59.1280	58.2937	59.0180	58.4154	59.4454	52.4430	53.2501	53.5795	53.7270	54.2979	53.6869	54.2683	53.6444	54.1816	53.7622	54.7740	**52.72**
**H2**	8.6734	8.5716	8.5291	8.4413	8.4174	8.4598	8.4642	8.4844	8.4585	8.4273	8.4239	8.6734	8.5716	8.5291	8.4413	8.4174	8.4598	8.4642	8.4844	8.4585	8.4273	8.4239	**8.13**
**H3**	7.3212	7.4479	7.5248	7.4715	7.5851	7.4724	7.6015	7.4743	7.5911	7.4711	7.6161	7.3212	7.4479	7.5248	7.4715	7.5851	7.4724	7.6015	7.4742	7.5911	7.4711	7.6161	**7.22**
**H5**	7.1132	7.2968	7.3811	7.3806	7.5011	7.3731	7.5252	7.3659	7.5113	7.3874	7.5560	7.1132	7.2968	7.3811	7.3806	7.5011	7.3731	7.5252	7.3659	7.5113	7.3874	7.5560	**7.22**
**H6**	7.9527	8.1444	8.2339	8.2577	8.3717	8.2512	8.4472	8.2430	8.4253	8.2631	8.4517	7.9527	8.1444	8.2339	8.2577	8.3718	8.2512	8.4472	8.2430	8.4253	8.2631	8.4517	**8.13**
**H8**	4.6213	4.7003	4.7284	4.7485	4.8209	4.7447	4.8619	4.7397	4.8460	4.7514	4.8420	4.6213	4.7003	4.7284	4.7485	4.8209	4.7447	4.8619	4.7397	4.8460	4.7514	4.8420	**5.5**
**H(9A-9B)**	1.6744	1.7230	1.6952	1.7318	1.7394	1.7361	1.7440	1.7433	1.7352	1.7294	1.7633	1.6744	1.7230	1.6952	1.7318	1.7394	1.7361	1.7440	1.7433	1.7352	1.7294	1.7633	**2.08**
**H(10-13A-10-13B)**	1.2019	1.1748	1.1382	1.1621	1.1149	1.1624	1.1177	1.1622	1.1151	1.1615	1.1159	1.2019	1.1748	1.1382	1.1621	1.1149	1.1624	1.1177	1.1622	1.1151	1.1615	1.1159	**1.25**
**H(14A-14C)**	0.9542	0.9509	0.9274	0.9477	0.9189	0.9473	0.9196	0.9467	0.9167	0.9478	0.9209	0.9542	0.9509	0.9275	0.9477	0.9189	0.9473	0.9196	0.9467	0.9167	0.9478	0.9210	**0.79**
**H(1`A-1`B)**	2.9748	2.9963	2.9997	3.0082	3.0152	3.0070	3.0217	3.0057	3.0181	3.0092	3.0059	2.8861	2.8057	2.7806	2.7753	2.7692	2.7754	2.7470	2.7752	2.7466	2.7747	3.0059	**3.69**
**H(2`-3`A-2`-3`B)**	1.6646	1.7246	1.6943	1.7673	1.7228	1.7628	1.7410	1.7574	1.7327	1.7710	1.7734	1.6646	1.7246	1.6943	1.7673	1.7228	1.7628	1.7410	1.7574	1.7327	1.7710	1.7734	**2.18**
**H(4`A)**	2.8937	2.7424	2.7044	2.7040	2.7162	2.7035	2.6638	2.7024	2.6619	2.7039	2.6761	3.1957	3.2373	3.2700	3.2776	3.3397	3.2745	3.3374	3.2714	3.3312	3.2805	2.6761	**3.12**
**H(4`B)**	2.8784	2.8690	2.8569	2.8466	2.8222	2.8473	2.8302	2.8480	2.8313	2.8455	2.7966	2.7538	2.7552	2.7294	2.7387	2.6907	2.7394	2.7059	2.7399	2.7050	2.7378	2.7966	**3.86**
Nuclei	B3P86																						
**C1**	137.0834	137.8838	138.5950	138.1997	140.4007	138.1971	139.7281	138.1738	139.9163	138.2476	140.2401	137.0836	137.8838	138.5950	138.1997	140.4007	138.1971	139.7281	138.1738	139.9164	138.2476	140.2401	**131.79**
**C2**	138.7928	138.8426	139.4558	138.6348	139.1986	138.6691	139.6535	138.6809	139.6182	138.6455	139.6258	138.7927	138.8426	139.4557	138.6348	139.1985	138.6691	139.6535	138.6809	139.6181	138.6454	139.6258	**131.93**
**C3**	121.1615	121.9450	122.6230	122.2596	122.9450	122.2700	123.2610	122.2581	123.2718	122.2929	123.3166	121.1615	121.9450	122.6231	122.2596	122.9449	122.2700	123.2610	122.2581	123.2717	122.2928	123.3166	**116.53**
**C4**	173.9036	174.7161	175.4467	175.0930	175.7719	175.0900	176.4012	175.0650	176.3353	175.1398	176.5148	173.9036	174.7161	175.4468	175.0930	175.7718	175.0900	176.4013	175.0649	176.3352	175.1398	176.5148	**168.1**
**C5**	119.0932	120.4249	121.1457	121.1011	121.9976	121.0809	122.2909	121.0385	122.2654	121.1644	122.4717	119.0933	120.4249	121.1457	121.1011	121.9977	121.0809	122.2910	121.0384	122.2655	121.1644	122.4717	**116.51**
**C6**	134.7635	136.5921	137.7420	137.5435	138.9370	137.4931	139.5843	137.4212	139.4478	137.6355	139.8721	134.7635	136.5921	137.7420	137.5435	138.9370	137.4931	139.5844	137.4212	139.4478	137.6355	139.8721	**131.88**
**C7**	201.7091	206.1582	207.2235	208.6080	209.9808	208.4686	214.8876	208.3097	213.9505	208.7868	216.2023	201.7088	206.1582	207.2235	208.6080	209.9808	208.4686	214.8878	208.3097	213.9506	208.7865	216.2023	**195.2**
**C8**	64.8079	65.9343	66.5679	66.5613	67.5044	66.5388	68.2200	66.4954	68.2101	66.6277	68.6156	64.8079	65.9343	66.5680	66.5613	67.5045	66.5388	68.2201	66.4954	68.2101	66.6277	68.6156	**54.87**
**C9**	36.1125	35.8844	36.1431	35.7858	35.9382	35.8087	36.0235	35.8099	36.0965	35.8083	36.1250	36.1126	35.8844	36.1431	35.7858	35.9383	35.8087	36.0235	35.8099	36.0964	35.8083	36.1250	**31.13**
**C10**	26.4016	26.9937	27.3249	27.2664	27.8758	27.2696	27.6542	27.2513	27.7532	27.3075	27.7928	26.4017	26.9937	27.3250	27.2664	27.8759	27.2696	27.6541	27.2513	27.7533	27.3074	27.7928	**30.82**
**C11**	35.6753	35.7321	36.1663	35.7039	36.1700	35.7285	36.1437	35.7310	36.2176	35.7242	36.3336	35.6754	35.7321	36.1664	35.7039	36.1700	35.7285	36.1438	35.7310	36.2176	35.7242	36.3336	**29.11**
**C12**	37.2531	37.5794	37.8592	37.7441	38.2383	37.7533	38.1084	37.7409	38.2044	37.7796	38.1869	37.2531	37.5794	37.8592	37.7441	38.2383	37.7533	38.1084	37.7409	38.2043	37.7796	38.1869	**25.52**
**C13**	28.2609	28.4559	28.7123	28.5134	28.7908	28.5316	28.7900	28.5276	28.8713	28.5399	28.7979	28.2609	28.4559	28.7124	28.5134	28.7908	28.5316	28.7900	28.5276	28.8713	28.5399	28.7979	**22.36**
**C14**	16.4118	16.5961	16.6704	16.6782	16.7716	16.6932	16.7542	16.6863	16.8392	16.7079	16.7903	16.4118	16.5961	16.6704	16.6782	16.7717	16.6932	16.7541	16.6863	16.8392	16.7079	16.7903	**13.85**
**C1`**	51.4586	52.2270	52.4925	52.5680	53.2231	52.5592	53.1618	52.5291	53.1915	52.6207	53.6755	55.5406	56.5732	57.0357	57.1199	57.5941	57.1046	57.8900	57.0675	57.8765	57.1780	58.2123	**51.23**
**C2`**	24.8445	25.2727	25.6163	25.5167	25.8866	25.5220	25.9858	25.5055	26.0735	25.5557	25.8103	24.5386	24.8450	25.2192	24.8834	25.5159	24.9056	25.5366	24.9053	25.4888	24.9059	25.1849	**23.85**
**C3`**	24.5387	24.8450	25.2192	24.8834	25.5160	24.9056	25.3966	24.9053	25.4888	24.9060	25.1849	24.8445	25.2727	25.6162	25.5167	25.8865	25.5220	25.9859	25.5055	26.0735	25.5557	25.8103	**23.66**
**C4`**	55.5405	56.5732	57.0356	57.1199	57.5941	57.1046	57.8900	57.0675	57.8765	57.1780	58.2123	51.4586	52.2270	52.4925	52.5680	53.2230	52.5592	53.1620	52.5291	53.1916	52.6207	53.6755	**52.72**
**H2**	8.7392	8.6447	8.6970	8.5823	8.5070	8.5883	8.5348	8.5923	8.5317	8.5776	8.5038	8.7392	8.6447	8.6970	8.5823	8.5070	8.5883	8.5348	8.5923	8.5317	8.5776	8.5038	**8.13**
**H3**	7.3337	7.4702	7.5701	7.5138	7.6235	7.5132	7.6370	7.5107	7.6327	7.5155	7.6549	7.3337	7.4702	7.5701	7.5138	7.6235	7.5132	7.6370	7.5107	7.6327	7.5155	7.6549	**7.22**
**H5**	7.1474	7.3333	7.4302	7.4093	7.5333	7.4059	7.5590	7.4005	7.5500	7.4138	7.5840	7.1474	7.3333	7.4302	7.4093	7.5333	7.4059	7.5590	7.4005	7.5500	7.4138	7.5840	**7.22**
**H6**	8.0119	8.1936	8.2856	8.3051	8.4182	8.3001	8.4988	8.2933	8.4772	8.3111	8.5069	8.0119	8.1936	8.2856	8.3051	8.4182	8.3001	8.4988	8.2933	8.4771	8.3111	8.5069	**8.13**
**H8**	4.6254	4.6960	4.7298	4.7363	4.8181	4.7354	4.8719	4.7327	4.8546	4.7385	4.8647	4.6254	4.6960	4.7298	4.7363	4.8181	4.7354	4.8719	4.7327	4.8546	4.7385	4.8647	**5.5**
**H(9A-9B)**	1.7458	1.7919	1.7901	1.8253	1.7930	1.8234	1.7822	1.8197	1.7868	1.8284	1.8093	1.7458	1.7919	1.7901	1.8253	1.7930	1.8234	1.7822	1.8197	1.7868	1.8284	1.8093	**2.08**
**H(10-13A-10-13B)**	1.1889	1.1592	1.1310	1.1399	1.0947	1.1422	1.0973	1.1426	1.0997	1.1390	1.0966	1.1890	1.1592	1.1310	1.1399	1.0947	1.1422	1.0973	1.1426	1.0997	1.1390	1.0966	**1.25**
**H(14A-14C)**	0.9192	0.9158	0.8956	0.9098	0.8844	0.9113	0.8847	0.9111	0.8872	0.9095	0.8877	0.9192	0.9158	0.8956	0.9098	0.8844	0.9113	0.8847	0.9111	0.8872	0.9095	0.8877	**0.79**
**H(1`A-1`B)**	2.9539	2.9694	2.9771	2.9793	2.9882	2.9796	2.9930	2.9781	2.9949	2.9803	2.9820	2.8869	2.8015	2.7624	2.7622	2.7598	2.7653	2.7262	2.7668	2.7369	2.7606	2.7158	**3.69**
**H(2`-3`A-2`-3`B)**	1.6531	1.7111	1.6726	1.7411	1.7071	1.7403	1.7256	1.7378	1.7217	1.7432	1.7606	1.6531	1.7111	1.6726	1.7411	1.7071	1.7403	1.7256	1.7378	1.7217	1.7432	1.7606	**2.18**
**H(4`A)**	2.9440	2.7797	2.7181	2.7120	2.7345	2.7164	2.6580	2.7193	2.6678	2.7092	2.6662	3.1970	3.2311	3.2632	3.2664	3.3279	3.2646	3.3241	3.2610	3.3229	3.2695	3.3304	**3.12**
**H(4`B)**	2.8298	2.8233	2.8068	2.8125	2.7851	2.8142	2.7944	2.8142	2.8061	2.8120	2.7653	2.7109	2.7077	2.6911	2.6922	2.6485	2.6946	2.6619	2.6951	2.6669	2.6911	2.6335	**3.86**

[a] methyl protons shifts are averaged

[b] according to [12]

Table S13. Cont. I

Nuclei	R											S											Exp. [b]
	B3PW91																						
	Gas	$CHCl_3$ (PCM)	$CHCl_3$ (SMD)	DMSO (PCM)	DMSO (SMD)	CH_3OH (PCM)	CH_3OH (SMD)	C_2H_5OH (PCM)	C_2H_5OH (SMD)	H_2O (PCM)	H_2O (SMD)	Gas	$CHCl_3$ (PCM)	$CHCl_3$ (SMD)	DMSO (PCM)	DMSO (SMD)	CH_3OH (PCM)	CH_3OH (SMD)	C_2H_5OH (PCM)	C_2H_5OH (SMD)	H_2O (PCM)	H_2O (SMD)	
C1	137.3645	138.2163	139.5596	138.6203	141.1688	138.5840	140.1980	138.5428	140.2522	138.6624	140.8332	137.3646	138.2164	139.5596	138.6209	140.8499	138.5840	140.1979	138.5429	140.2523	138.6624	140.8332	**131.79**
C2	138.8786	138.9320	139.4523	138.7409	139.6047	138.7629	139.7207	138.7751	139.6234	138.7210	139.7073	138.8786	138.9321	139.4523	138.7408	139.2858	138.7629	139.7206	138.7751	139.6233	138.7210	139.7073	**131.93**
C3	121.3464	122.1193	122.6939	122.3239	123.3407	122.3207	123.3300	122.3080	123.2511	122.3284	123.3858	121.3464	122.1193	122.6939	122.3239	123.0218	122.3207	123.3300	122.3080	123.2512	122.3284	123.3858	**116.53**
C4	173.9175	174.7530	175.3773	175.1270	176.0876	175.1110	176.3736	175.0858	176.2225	175.1436	176.4569	173.9174	174.7530	175.3773	175.1270	175.7686	175.1110	176.3736	175.0858	176.2225	175.1436	176.4568	**168.1**
C5	119.1873	120.5407	121.1755	121.1980	122.4166	121.1623	122.3683	121.1177	122.2464	121.2341	122.5596	119.1873	120.5408	121.1755	121.1979	122.0976	121.1623	122.3683	121.1178	122.2464	121.2341	122.5597	**116.51**
C6	134.5875	136.4784	137.3751	137.5710	139.2512	137.5080	139.5727	137.4362	139.3402	137.6323	139.8227	134.5876	136.4784	137.3751	137.5709	138.9321	137.5080	139.5728	137.4363	139.3404	137.6323	139.8227	**131.88**
C7	201.7750	206.3530	208.1156	208.9132	210.5822	208.7357	215.2564	208.5546	214.1387	209.0906	216.7068	201.7750	206.3531	208.1156	208.9134	210.2635	208.7357	215.2562	208.5546	214.1385	209.0906	216.7068	**195.2**
C8	64.8040	66.0074	66.8207	66.6379	67.9609	66.5916	68.3251	66.5385	68.1722	66.6879	68.7655	64.8040	66.0069	66.8207	66.6385	67.6419	66.5916	68.3251	66.5385	68.1722	66.6879	68.7655	**54.87**
C9	35.6465	35.4775	35.4913	35.3856	35.8745	35.3959	35.6827	35.3974	35.6386	35.3777	35.7426	35.6465	35.4776	35.4913	35.3856	35.5555	35.3959	35.6826	35.3974	35.6386	35.3777	35.7426	**31.13**
C10	26.1614	26.7547	26.9834	27.0064	27.9075	26.9959	27.4018	26.9765	27.3991	27.0185	27.5461	26.1613	26.7546	26.9834	27.0064	27.5885	26.9959	27.4018	26.9765	27.3990	27.0185	27.5461	**30.82**
C11	35.4336	35.5429	35.8310	35.4896	36.3003	35.5001	35.9266	35.5013	35.9047	35.4817	36.1113	35.4336	35.5428	35.8310	35.4898	35.9813	35.5001	35.9265	35.5013	35.9046	35.4817	36.1113	**29.11**
C12	36.9439	37.2879	37.5087	37.4449	38.2312	37.4390	37.7900	37.4244	37.7888	37.4530	37.8748	36.9440	37.2879	37.5087	37.4450	37.9123	37.4390	37.7901	37.4244	37.7887	37.4530	37.8748	**25.52**
C13	28.2326	28.4628	28.6279	28.5058	29.1001	28.5087	28.7676	28.5028	28.7548	28.5047	28.7787	28.2325	28.4628	28.6279	28.5057	28.7812	28.5087	28.7675	28.5027	28.7548	28.5048	28.7787	**22.36**
C14	16.4298	16.6433	16.6448	16.7183	17.1147	16.7179	16.7817	16.7088	16.7699	16.7208	16.8179	16.4299	16.6434	16.6448	16.7183	16.7958	16.7179	16.7816	16.7088	16.7699	16.7208	16.8178	**13.85**
C1`	51.2577	52.0600	52.3040	52.5115	53.3865	52.4856	53.0757	52.4515	53.0021	52.5388	53.6024	55.4709	56.4890	56.8905	57.0331	57.5091	57.0031	57.7631	56.9645	57.6619	57.0635	58.0933	**51.23**
C2`	25.1287	25.5832	25.8665	25.7707	26.5017	25.7620	26.2242	25.7445	26.2122	25.7820	26.0244	24.7778	25.0766	25.4001	25.1100	25.7222	25.1161	25.6045	25.1128	25.6091	25.1061	25.3792	**23.85**
C3`	24.7777	25.0765	25.4001	25.1105	26.0411	25.1160	25.6045	25.1127	25.6092	25.1061	25.3791	25.1288	25.5832	25.8665	25.7710	26.1827	25.7620	26.2242	25.7445	26.2123	25.7820	26.0244	**23.66**
C4`	55.4708	56.4889	56.8905	57.0326	57.8281	57.0031	57.7631	56.9644	57.6619	57.0635	58.0933	51.2577	52.0599	52.3040	52.5115	53.0676	52.4856	53.0756	52.4515	53.0022	52.5387	53.6024	**52.72**
H2	8.7103	8.6070	8.5673	8.5299	8.4699	8.5378	8.4926	8.5448	8.4922	8.5219	8.4533	8.7103	8.6070	8.5673	8.5299	8.4544	8.5378	8.4926	8.5448	8.4922	8.5219	8.4533	**8.13**
H3	7.3394	7.4704	7.5521	7.5077	7.6323	7.5067	7.6349	7.5053	7.6265	7.5087	7.6511	7.3394	7.4704	7.5521	7.5078	7.6168	7.5067	7.6349	7.5053	7.6265	7.5087	7.6511	**7.22**
H5	7.1369	7.3234	7.4096	7.4036	7.5461	7.3995	7.5606	7.3951	7.5472	7.4076	7.5861	7.1369	7.3234	7.4096	7.4036	7.5306	7.3995	7.5606	7.3951	7.5471	7.4076	7.5861	**7.22**
H6	8.0140	8.1968	8.2900	8.2999	8.4377	8.2941	8.4796	8.2881	8.4586	8.3052	8.4788	8.0140	8.1968	8.2900	8.2998	8.4223	8.2941	8.4796	8.2881	8.4587	8.3052	8.4788	**8.13**
H8	4.6225	4.6925	4.7350	4.7304	4.8337	4.7285	4.8595	4.7264	4.8415	4.7321	4.8437	4.6225	4.6925	4.7350	4.7303	4.8182	4.7285	4.8595	4.7264	4.8415	4.7321	4.8437	**5.5**
H(9A-9B)	1.6924	1.7398	1.7075	1.7804	1.7654	1.7782	1.7426	1.7757	1.7410	1.7826	1.7605	1.6924	1.7398	1.7075	1.7804	1.7499	1.7782	1.7426	1.7757	1.7410	1.7826	1.7605	**2.08**
H(10-13A-10-13B)	1.1619	1.1324	1.0979	1.1165	1.0864	1.1180	1.0759	1.1193	1.0754	1.1153	1.0725	1.1619	1.1324	1.0979	1.1165	1.0709	1.1180	1.0759	1.1193	1.0754	1.1153	1.0725	**1.25**
H(14A-14C)	0.9089	0.9041	0.8834	0.8995	0.8883	0.9002	0.8758	0.9007	0.8745	0.8989	0.8760	0.9089	0.9042	0.8834	0.8995	0.8728	0.9002	0.8758	0.9007	0.8745	0.8989	0.8760	**0.79**
H(1`A-1`B)	2.9352	2.9535	2.9613	2.9668	2.9890	2.9663	2.9814	2.9656	2.9796	2.9674	2.9660	2.8628	2.7786	2.7548	2.7425	2.7390	2.7444	2.7144	2.7462	2.7179	2.7411	2.7019	**3.69**
H(2`-3`A-2`-3`B)	1.6419	1.6981	1.6698	1.7314	1.7119	1.7295	1.7143	1.7274	1.7065	1.7334	1.7473	1.6419	1.6981	1.6698	1.7313	1.6964	1.7295	1.7143	1.7274	1.7064	1.7334	1.7473	**2.18**
H(4`A)	2.8969	2.7390	2.6984	2.6833	2.7181	2.6861	2.6443	2.6888	2.6460	2.6811	2.6520	3.1688	3.2062	3.2405	3.2479	3.3045	3.2453	3.3074	3.2424	3.3027	3.2506	3.3091	**3.12**
H(4`B)	2.8287	2.8182	2.8111	2.8018	2.7909	2.8027	2.7845	2.8034	2.7898	2.8012	2.7518	2.7017	2.7010	2.6820	2.6857	2.6424	2.6874	2.6555	2.6887	2.6566	2.6843	2.6229	**3.86**
Nuclei	B98																						
C1	138.3861	139.1649	140.4959	139.4943	141.7469	139.4786	140.9154	139.4636	141.0236	139.5145	141.5464	138.3859	139.1651	140.4960	139.4944	141.7468	139.4786	140.9153	139.4635	141.0237	139.5145	141.5464	**131.79**
C2	138.6697	138.6917	139.1617	138.4909	138.9633	138.5228	139.3950	138.5514	139.2860	138.4619	139.2967	138.6697	138.6918	139.1616	138.4909	138.9632	138.5229	139.3949	138.5516	139.2861	138.4619	139.2967	**131.93**
C3	121.0784	121.8156	122.4177	122.0425	122.7352	122.0473	123.0190	122.0490	122.9505	122.0395	123.0353	121.0785	121.8155	122.4178	122.0424	122.7354	122.0472	123.0190	122.0490	122.9507	122.0395	123.0353	**116.53**
C4	173.7000	174.5104	175.1610	174.9090	175.5572	174.9011	176.1754	174.8904	176.0258	174.9180	176.2418	173.7000	174.5102	175.1610	174.9088	175.5572	174.9011	176.1756	174.8904	176.0257	174.9180	176.2418	**168.1**
C5	118.9046	120.1908	120.8150	120.8450	121.7112	120.8201	121.9746	120.7925	121.8719	120.8711	122.1772	118.9047	120.1908	120.8150	120.8450	121.7112	120.8201	121.9746	120.7925	121.8717	120.8712	122.1772	**116.51**
C6	134.5982	136.4476	137.3328	137.5688	138.8504	137.5145	139.5600	137.4572	139.3090	137.6222	139.7331	134.5981	136.4477	137.3327	137.5689	138.8504	137.5145	139.5600	137.4572	139.3090	137.6223	139.7331	**131.88**
C7	200.2633	204.8889	206.6379	207.3940	208.7742	207.2367	213.7939	207.0820	212.6960	207.5500	215.3167	200.2632	204.8888	206.6385	207.3939	208.7741	207.2367	213.7938	207.0823	212.6960	207.5503	215.3167	**195.2**
C8	66.4691	67.6252	68.3963	68.1743	69.1924	68.1472	69.8314	68.1199	69.7053	68.2045	70.2790	66.4691	67.6251	68.3964	68.1742	69.1924	68.1472	69.8313	68.1199	69.7053	68.2045	70.2790	**54.87**
C9	36.8864	36.6288	36.6738	36.5274	36.7193	36.5483	36.8690	36.5668	36.8166	36.5103	36.9030	36.8864	36.6288	36.6737	36.5276	36.7191	36.5483	36.8691	36.5669	36.8166	36.5103	36.9030	**31.13**
C10	27.6385	28.1528	28.4190	28.3980	28.9752	28.3979	28.8124	28.3952	28.8117	28.4003	28.9096	27.6385	28.1528	28.4191	28.3979	28.9752	28.3979	28.8124	28.3952	28.8118	28.4003	28.9096	**30.82**
C11	36.8550	36.9202	37.2192	36.8523	37.3709	36.8743	37.3328	36.8934	37.3147	36.8331	37.5146	36.8549	36.9203	37.2192	36.8522	37.3709	36.8743	37.3329	36.8934	37.3147	36.8331	37.5146	**29.11**
C12	38.3761	38.6689	38.9421	38.8478	39.3324	38.8502	39.2296	38.8503	39.2294	38.8481	39.2872	38.3761	38.6689	38.9421	38.8478	39.3324	38.8502	39.2296	38.8503	39.2296	38.8480	39.2872	**25.52**
C13	29.5566	29.7397	29.9266	29.7837	30.0731	29.7970	30.0658	29.8076	30.0571	29.7733	30.0614	29.5566	29.7398	29.9266	29.7837	30.0731	29.7970	30.0657	29.8076	30.0572	29.7733	30.0614	**22.36**
C14	17.0197	17.1852	17.2176	17.2688	17.3571	17.2781	17.3471	17.2850	17.3394	17.2624	17.3623	17.0197	17.1853	17.2176	17.2688	17.3572	17.2781	17.3471	17.2850	17.3395	17.2624	17.3623	**13.85**
C1`	52.5177	53.2990	53.5734	53.7328	54.3305	53.7198	54.2589	53.7046	54.1936	53.7479	54.7567	56.8480	57.7803	58.1986	58.3161	58.8176	58.2952	59.0746	58.2721	58.9862	58.3384	59.3919	**51.23**
C2`	26.3044	26.7187	27.0115	26.9275	27.3424	26.9271	27.4333	26.9245	27.4151	26.9305	27.2806	25.9561	26.2650	26.6082	26.2868	26.9021	26.3055	26.7675	26.3213	26.7759	26.2708	26.5401	**23.85**
C3`	25.9562	26.2649	26.6082	26.2868	26.9021	26.3055	26.7676	26.3213	26.7759	26.2707	26.5401	26.3044	26.7186	27.0115	26.9275	27.3424	26.9271	27.4333	26.9245	27.4151	26.9305	27.2806	**23.66**
C4`	56.8479	57.7802	58.1987	58.3160	58.8176	58.2952	59.0746	58.2720	58.9861	58.3383	59.3919	52.5177	53.2991	53.5733	53.7329	54.3306	53.7198	54.2589	53.7046	54.1936	53.7479	54.7567	**52.72**
H2	8.7138	8.6192	8.5816	8.5493	8.4689	8.5557	8.5103	8.5617	8.5070	8.5430	8.4604	8.7138	8.6192	8.5815	8.5493	8.4689	8.5557	8.5103	8.5617	8.5070	8.5430	8.4604	**8.13**
H3	7.3308	7.4666	7.5545	7.5081	7.6197	7.5063	7.6370	7.5046	7.6287	7.5099	7.6479	7.3308	7.4666	7.5545	7.5081	7.6197	7.5063	7.6370	7.5046	7.6287	7.5099	7.6479	**7.22**
H5	7.1292	7.3142	7.4019	7.3959	7.5223	7.3913	7.5462	7.3867	7.5348	7.4004	7.5759	7.1292	7.3142	7.4019	7.3959	7.5223	7.3913	7.5462	7.3868	7.5348	7.4004	7.5759	**7.22**
H6	7.9721	8.1564	8.2591	8.2658	8.3991	8.2591	8.4756	8.2525	8.4559	8.2723	8.4782	7.9721	8.1564	8.2590	8.2658	8.3991	8.2591	8.4756	8.2525	8.4559	8.2723	8.4782	**8.13**
H8	4.5750	4.6530	4.6950	4.6953	4.7825	4.6930	4.8313	4.6907	4.8156	4.6978	4.8120	4.5750	4.6530	4.6950	4.6953	4.7825	4.6930	4.8313	4.6907	4.8156	4.6978	4.8120	**5.5**
H(9A-9B)	1.6864	1.7235	1.6967	1.7636	1.7394	1.7608	1.7401	1.7581	1.7363	1.7666	1.7530	1.6864	1.7235	1.6967	1.7636	1.7394	1.7608	1.7401	1.7581	1.7363	1.7666	1.7530	**2.08**
H(10-13A-10-13B)	1.1900	1.1580	1.1250	1.1423	1.0972	1.1434	1.1025	1.1446	1.1021	1.1416	1.0967	1.1900	1.1580	1.1250	1.1423	1.0972	1.1434	1.1025	1.1446	1.1021	1.1416	1.0967	**1.25**
H(14A-14C)	0.9310	0.9246	0.9047	0.9199	0.8935	0.9203	0.8961	0.9208	0.8954	0.9198	0.8946	0.9310	0.9246	0.9047	0.9199	0.8935	0.9203	0.8961	0.9208	0.8954	0.9198	0.8946	**0.79**
H(1`A-1`B)	2.9516	2.9712	2.9799	2.9851	2.9945	2.9841	2.9995	2.9833	2.9986	2.9863	2.9814	2.8644	2.7894	2.7693	2.7572	2.7536	2.7587	2.7353	2.7605	2.7377	2.7562	2.7249	**3.69**
H(2`-3`A-2`-3`B)	1.6594	1.7154	1.6872	1.7501	1.7151	1.7478	1.7358	1.7456	1.7293	1.7526	1.7695	1.6594	1.7154	1.6872	1.7500	1.7151	1.7478	1.7358	1.7456	1.7293	1.7526	1.7695	**2.18**
H(4`A)	2.8862	2.7447	2.7121	2.6974	2.7152	2.6997	2.6664	2.7022	2.6672	2.6957	2.6773	3.1819	3.2192	3.2560	3.2623	3.3244	3.2591	3.3205	3.2561	3.3176	3.2657	3.3173	**3.12**
H(4`B)	2.8427	2.8341	2.8265	2.8170	2.7920	2.8178	2.8042	2.8188	2.8081	2.8167	2.7724	2.7214	2.7233	2.7037	2.7079	2.6645	2.7092	2.6785	2.7106	2.6795	2.7069	2.6455	**3.86**

[a] methyl protons shifts are averaged
[b] according to [12]

Table S13. Cont. II

Nuclei	R											S											Exp.[b]
	B971																						
	Gas	$CHCl_3$ (PCM)	$CHCl_3$ (SMD)	DMSO (PCM)	DMSO (SMD)	CH_3OH (PCM)	CH_3OH (SMD)	C_2H_5OH (PCM)	C_2H_5OH (SMD)	H_2O (PCM)	H_2O (SMD)	Gas	$CHCl_3$ (PCM)	$CHCl_3$ (SMD)	DMSO (PCM)	DMSO (SMD)	CH_3OH (PCM)	CH_3OH (SMD)	C_2H_5OH (PCM)	C_2H_5OH (SMD)	H_2O (PCM)	H_2O (SMD)	
**C1**	137.9579	138.7472	140.0938	139.0723	141.3254	139.0511	140.5387	139.0325	140.6413	139.1010	141.1680	137.9579	138.7472	140.0938	139.0723	141.3253	139.0512	140.5387	139.0325	140.6414	139.1010	141.1681	**131.79**
**C2**	138.3790	138.4118	138.8725	138.2234	138.6679	138.2469	139.0987	138.2692	138.9878	138.2060	138.9993	138.3790	138.4117	138.8724	138.2234	138.6678	138.2470	139.0987	138.2692	138.9878	138.2059	138.9992	**131.93**
**C3**	120.8861	121.6303	122.2331	121.8686	122.5508	121.8653	122.8358	121.8608	122.7656	121.8770	122.8585	120.8861	121.6303	122.2331	121.8686	122.5508	121.8653	122.8357	121.8609	122.7654	121.8770	122.8584	**116.53**
**C4**	173.5870	174.4058	175.0620	174.8162	175.4697	174.8001	176.0781	174.7831	175.9292	174.8372	176.1579	173.5870	174.4059	175.0620	174.8162	175.4697	174.8002	176.0781	174.7830	175.9293	174.8372	176.1579	**168.1**
**C5**	118.7067	119.9991	120.6268	120.6631	121.5263	120.6301	121.7879	120.5967	121.6853	120.7004	121.9924	118.7067	119.9992	120.6268	120.6630	121.5264	120.6301	121.7879	120.5967	121.6852	120.7004	121.9925	**116.51**
**C6**	134.3132	136.1577	137.0294	137.2873	138.5643	137.2244	139.2420	137.1607	138.9968	137.3527	139.4332	134.3132	136.1576	137.0295	137.2873	138.5642	137.2243	139.2420	137.1608	138.9969	137.3528	139.4331	**131.88**
**C7**	200.1399	204.7116	206.4713	207.1825	208.5987	207.0220	213.5556	206.8653	212.4802	207.3455	215.0840	200.1399	204.7119	206.4711	207.1825	208.5990	207.0220	213.5556	206.8652	212.4801	207.3456	215.0840	**195.2**
**C8**	66.2700	67.4167	68.1726	67.9741	68.9802	67.9396	69.6005	67.9068	69.4827	68.0151	70.0458	66.2700	67.4167	68.1724	67.9741	68.9802	67.9396	69.6005	67.9069	69.4827	68.0152	70.0458	**54.87**
**C9**	36.8619	36.6248	36.6577	36.5434	36.7179	36.5549	36.8570	36.5661	36.8055	36.5388	36.9061	36.8619	36.6247	36.6578	36.5434	36.7179	36.5549	36.8570	36.5661	36.8054	36.5389	36.9061	**31.13**
**C10**	27.6649	28.1911	28.4444	28.4462	29.0102	28.4381	28.8356	28.4296	28.8361	28.4599	28.9396	27.6649	28.1911	28.4444	28.4462	29.0103	28.4381	28.8356	28.4296	28.8361	28.4599	28.9396	**30.82**
**C11**	36.8585	36.9218	37.2181	36.8639	37.3694	36.8775	37.3278	36.8904	37.3088	36.8566	37.5133	36.8585	36.9219	37.2181	36.8639	37.3696	36.8776	37.3278	36.8903	37.3088	36.8565	37.5132	**29.11**
**C12**	38.3909	38.6888	38.9535	38.8745	39.3479	38.8692	39.2394	38.8639	39.2396	38.8856	39.3057	38.3909	38.6887	38.9535	38.8744	39.3479	38.8693	39.2393	38.8638	39.2396	38.8856	39.3057	**25.52**
**C13**	29.5262	29.7114	29.8938	29.7636	30.0410	29.7689	30.0304	29.7736	30.0218	29.7645	30.0308	29.5262	29.7114	29.8938	29.7635	30.0411	29.7689	30.0304	29.7736	30.0218	29.7645	30.0308	**22.36**
**C14**	16.9704	17.1396	17.1657	17.2310	17.3068	17.2326	17.2929	17.2336	17.2853	17.2359	17.3134	16.9704	17.1396	17.1657	17.2311	17.3068	17.2326	17.2930	17.2336	17.2854	17.2358	17.3133	**13.85**
**C1`**	52.4411	53.2195	53.4844	53.6577	54.2418	53.6367	54.1675	53.6156	54.1020	53.6843	54.6682	56.7490	57.6864	58.1043	58.2296	58.7249	58.2010	58.9752	58.1722	58.8884	58.2627	59.2925	**51.23**
**C2`**	26.1067	26.5359	26.8246	26.7648	27.1656	26.7559	27.2507	26.7468	27.2329	26.7795	27.1086	25.7726	26.0821	26.4300	26.1125	26.7222	26.1234	26.5932	26.1329	26.5994	26.1079	26.3818	**23.85**
**C3`**	25.7726	26.0820	26.4300	26.1126	26.7222	26.1234	26.5932	26.1330	26.5994	26.1079	26.3817	26.1067	26.5358	26.8247	26.7649	27.1656	26.7560	27.2506	26.7468	27.2329	26.7796	27.1086	**23.66**
**C4`**	56.7490	57.6864	58.1043	58.2295	58.7248	58.2010	58.9752	58.1721	58.8883	58.2626	59.2925	52.4411	53.2195	53.4846	53.6578	54.2418	53.6367	54.1675	53.6157	54.1019	53.6843	54.6683	**52.72**
**H2**	8.7130	8.6205	8.5818	8.5548	8.4695	8.5605	8.5097	8.5658	8.5060	8.5492	8.4595	8.7130	8.6205	8.5818	8.5547	8.4695	8.5605	8.5097	8.5658	8.5060	8.5492	8.4595	**8.13**
**H3**	7.3267	7.4622	7.5503	7.5048	7.6156	7.5027	7.6328	7.5007	7.6245	7.5069	7.6441	7.3267	7.4621	7.5503	7.5047	7.6156	7.5027	7.6328	7.5007	7.6245	7.5069	7.6441	**7.22**
**H5**	7.1292	7.3125	7.3992	7.3941	7.5183	7.3894	7.5413	7.3846	7.5301	7.3990	7.5702	7.1292	7.3125	7.3992	7.3941	7.5183	7.3893	7.5413	7.3846	7.5301	7.3990	7.5702	**7.22**
**H6**	7.9816	8.1607	8.2609	8.2675	8.3993	8.2608	8.4733	8.2543	8.4537	8.2741	8.4750	7.9816	8.1607	8.2609	8.2675	8.3993	8.2608	8.4733	8.2543	8.4537	8.2741	8.4750	**8.13**
**H8**	4.5809	4.6550	4.7011	4.6960	4.7853	4.6937	4.8333	4.6913	4.8178	4.6985	4.8122	4.5809	4.6550	4.7011	4.6960	4.7853	4.6937	4.8333	4.6913	4.8178	4.6986	4.8122	**5.5**
**H(9A-9B)**	1.6950	1.7314	1.7009	1.7727	1.7457	1.7696	1.7438	1.7665	1.7404	1.7763	1.7578	1.6950	1.7314	1.7009	1.7727	1.7457	1.7696	1.7438	1.7665	1.7403	1.7763	1.7578	**2.08**
**H(10-13A-10-13B)**	1.1898	1.1569	1.1241	1.1418	1.0959	1.1427	1.1015	1.1437	1.1009	1.1414	1.0960	1.1898	1.1569	1.1241	1.1418	1.0959	1.1428	1.1015	1.1437	1.1009	1.1414	1.0960	**1.25**
**H(14A-14C)**	0.9262	0.9195	0.8998	0.9154	0.8883	0.9156	0.8910	0.9158	0.8902	0.9156	0.8896	0.9262	0.9195	0.8998	0.9154	0.8883	0.9156	0.8911	0.9158	0.8903	0.9156	0.8896	**0.79**
**H(1`A-1`B)**	2.9480	2.9666	2.9750	2.9807	2.9895	2.9797	2.9937	2.9786	2.9928	2.9822	2.9751	2.8620	2.7881	2.7681	2.7564	2.7524	2.7578	2.7328	2.7594	2.7356	2.7557	2.7222	**3.69**
**H(2`-3`A-2`-3`B)**	1.6590	1.7145	1.6864	1.7486	1.7137	1.7463	1.7340	1.7440	1.7275	1.7514	1.7675	1.6590	1.7145	1.6865	1.7487	1.7137	1.7463	1.7340	1.7440	1.7275	1.7514	1.7675	**2.18**
**H(4`A)**	2.8916	2.7505	2.7172	2.7005	2.7180	2.7029	2.6679	2.7055	2.6692	2.6990	2.6779	3.1854	3.2210	3.2569	3.2637	3.3248	3.2604	3.3200	3.2572	3.3172	3.2672	3.3168	**3.12**
**H(4`B)**	2.8325	2.8257	2.8192	2.8122	2.7869	2.8127	2.7977	2.8133	2.8020	2.8123	2.7665	2.7106	2.7123	2.6933	2.6978	2.6543	2.6989	2.6673	2.7000	2.6683	2.6971	2.6335	**3.86**
Nuclei	B972																						
**C1**	134.8089	135.7802	136.9805	137.1435	138.4018	137.0860	137.6594	137.0252	137.6561	137.1926	138.4940	134.8089	135.7802	136.9805	137.1435	138.4018	137.0861	137.6594	137.0251	137.6561	137.1925	138.4940	**131.79**
**C2**	136.3116	136.2928	136.8023	136.0499	136.6284	136.0667	136.9839	136.0811	136.9153	136.0297	136.9539	136.3116	136.2927	136.8023	136.0499	136.6284	136.0668	136.9839	136.0810	136.9154	136.0296	136.9539	**131.93**
**C3**	118.7780	119.4634	120.0489	119.7596	120.3977	119.7456	120.6719	119.7285	120.6023	119.7684	120.7324	118.7780	119.4634	120.0488	119.7596	120.3977	119.7457	120.6719	119.7284	120.6023	119.7685	120.7324	**116.53**
**C4**	170.5791	171.3249	171.9325	171.6078	172.2871	171.5891	172.8917	171.5681	172.7550	171.6213	172.9340	170.5791	171.3248	171.9324	171.6078	172.2870	171.5892	172.8917	171.5681	172.7551	171.6214	172.9341	**168.1**
**C5**	116.6218	117.8904	118.5100	118.6924	119.3953	118.6471	119.6764	118.5992	119.5557	118.7306	119.8804	116.6218	117.8903	118.5100	118.6925	119.3953	118.6472	119.6764	118.5992	119.5556	118.7306	119.8804	**116.51**
**C6**	132.0025	133.7516	134.6831	134.5632	136.1143	134.5041	136.7747	134.4435	136.5605	134.6155	136.9243	132.0025	133.7516	134.6832	134.5632	136.1143	134.5041	136.7747	134.4435	136.5605	134.6155	136.9243	**131.88**
**C7**	197.7084	202.3854	203.9953	205.9540	206.2360	205.7561	211.1804	205.5561	209.9691	206.1363	212.8165	197.7084	202.3858	203.9950	205.9540	206.2360	205.7561	211.1804	205.5560	209.9690	206.1363	212.8165	**195.2**
**C8**	63.4988	64.6959	65.5210	65.6629	66.3756	65.6036	67.0354	65.5423	66.8280	65.7148	67.5126	63.4988	64.6959	65.5210	65.6630	66.3756	65.6036	67.0354	65.5423	66.8280	65.7148	67.5126	**54.87**
**C9**	34.9897	34.7638	34.8547	34.5929	34.9074	34.6015	35.0731	34.6079	35.0157	34.5806	35.0839	34.9897	34.7637	34.8547	34.5929	34.9074	34.6016	35.0731	34.6079	35.0158	34.5806	35.0839	**31.13**
**C10**	26.0367	26.5440	26.8472	26.8206	27.4511	26.8013	27.2698	26.7800	27.2627	26.8350	27.4033	26.0367	26.5440	26.8472	26.8206	27.4511	26.8013	27.2698	26.7800	27.2627	26.8348	27.4034	**30.82**
**C11**	35.1774	35.2516	35.5671	35.2137	35.7190	35.2174	35.6745	35.2180	35.6527	35.2053	35.8517	35.1774	35.2516	35.5670	35.2137	35.7190	35.2174	35.6745	35.2180	35.6527	35.2054	35.8517	**29.11**
**C12**	36.6134	36.8984	37.1534	37.1431	37.5424	37.1282	37.4315	37.1115	37.4307	37.1534	37.5223	36.6134	36.8984	37.1535	37.1431	37.5425	37.1282	37.4315	37.1115	37.4308	37.1534	37.5223	**25.52**
**C13**	28.2326	28.4235	28.6155	28.5272	28.7792	28.5230	28.7619	28.5160	28.7482	28.5267	28.7781	28.2326	28.4234	28.6155	28.5271	28.7792	28.5230	28.7619	28.5161	28.7482	28.5266	28.7781	**22.36**
**C14**	16.5580	16.7272	16.7609	16.8870	16.9251	16.8784	16.9045	16.8677	16.8923	16.8908	16.9465	16.5580	16.7272	16.7608	16.8869	16.9250	16.8784	16.9045	16.8677	16.8923	16.8908	16.9465	**13.85**
**C1`**	50.4284	51.1614	51.4450	51.7720	52.2602	51.7409	52.2001	51.7073	52.1241	51.7971	52.7224	54.6531	55.5810	55.9895	56.1942	56.6326	56.1594	56.8078	56.1220	56.7158	56.2225	57.1181	**51.23**
**C2`**	25.0637	25.4848	25.8033	25.6862	26.1205	25.6707	26.1502	25.6530	26.1362	25.6968	25.9596	24.7216	24.9676	25.2925	25.1576	25.6280	25.1509	25.4971	25.1418	25.5048	25.1594	25.3054	**23.85**
**C3`**	24.7216	24.9675	25.2925	25.1576	25.6280	25.1510	25.4971	25.1418	25.5048	25.1594	25.3054	25.0637	25.4847	25.8033	25.6863	26.1205	25.6707	26.1502	25.6528	26.1361	25.6968	25.9596	**23.66**
**C4`**	54.6531	55.5809	55.9896	56.1941	56.6326	56.1594	56.8078	56.1221	56.7158	56.2225	57.1181	50.4285	51.1614	51.4451	51.7720	52.2603	51.7409	52.2001	51.7073	52.1241	51.7971	52.7224	**52.72**
**H2**	8.6878	8.5592	8.5417	8.3954	8.4285	8.4029	8.4611	8.4103	8.4688	8.3883	8.4115	8.6878	8.5592	8.5417	8.3954	8.4285	8.4029	8.4611	8.4103	8.4688	8.3884	8.4115	**8.13**
**H3**	7.3342	7.4591	7.5441	7.4876	7.6089	7.4859	7.6278	7.4839	7.6196	7.4892	7.6480	7.3342	7.4591	7.5441	7.4876	7.6088	7.4859	7.6278	7.4839	7.6196	7.4892	7.6480	**7.22**
**H5**	7.1246	7.3056	7.3964	7.4085	7.5202	7.4028	7.5507	7.3967	7.5365	7.4137	7.5813	7.1246	7.3056	7.3964	7.4085	7.5202	7.4027	7.5507	7.3967	7.5365	7.4137	7.5813	**7.22**
**H6**	8.0091	8.1941	8.2801	8.2570	8.3923	8.2524	8.4559	8.2476	8.4415	8.2613	8.4406	8.0091	8.1941	8.2801	8.2570	8.3923	8.2524	8.4559	8.2476	8.4415	8.2613	8.4406	**8.13**
**H8**	4.5979	4.6662	4.6965	4.6978	4.7701	4.6954	4.8178	4.6928	4.8038	4.6999	4.7959	4.5979	4.6662	4.6965	4.6978	4.7701	4.6954	4.8178	4.6928	4.8038	4.6999	4.7959	**5.5**
**H(9A-9B)**	1.6528	1.6966	1.6781	1.6882	1.7119	1.6868	1.7146	1.6853	1.7123	1.6895	1.7187	1.6528	1.6966	1.6781	1.6882	1.7119	1.6868	1.7146	1.6853	1.7123	1.6894	1.7187	**2.08**
**H(10-13A-10-13B)**	1.1634	1.1355	1.1013	1.1249	1.0761	1.1256	1.0792	1.1262	1.0798	1.1241	1.0761	1.1634	1.1355	1.1012	1.1249	1.0761	1.1256	1.0792	1.1262	1.0798	1.1241	1.0761	**1.25**
**H(14A-14C)**	0.9222	0.9177	0.8963	0.9192	0.8870	0.9193	0.8888	0.9192	0.8881	0.9190	0.8899	0.9222	0.9177	0.8963	0.9191	0.8870	0.9192	0.8888	0.9192	0.8881	0.9190	0.8900	**0.79**
**H(1`A-1`B)**	2.9360	2.9559	2.9627	2.9699	2.9748	2.9690	2.9825	2.9680	2.9811	2.9705	2.9660	2.8519	2.7754	2.7542	2.7578	2.7400	2.7588	2.7238	2.7598	2.7244	2.7568	2.7133	**3.69**
**H(2`-3`A-2`-3`B)**	1.6395	1.6971	1.6670	1.7420	1.6907	1.7395	1.7111	1.7367	1.7037	1.7443	1.7447	1.6395	1.6971	1.6670	1.7420	1.6907	1.7394	1.7111	1.7367	1.7037	1.7443	1.7447	**2.18**
**H(4`A)**	2.8705	2.7253	2.6920	2.7049	2.6985	2.7063	2.6585	2.7076	2.6567	2.7037	2.6715	3.1710	3.2128	3.2463	3.2573	3.3134	3.2546	3.3131	3.2518	3.3087	3.2596	3.3129	**3.12**
**H(4`B)**	2.8333	2.8255	2.8165	2.8106	2.7814	2.8114	2.7890	2.8120	2.7921	2.8098	2.7552	2.7011	2.6990	2.6790	2.6824	2.6363	2.6835	2.6518	2.6843	2.6535	2.6813	2.6192	**3.86**

[a] methyl protons shifts are averaged

[b] according to [12]

Table S13. Cont. III

Nuclei	R											S											Exp. [b]
	BHandH																						
	Gas	$CHCl_3$ (PCM)	$CHCl_3$ (SMD)	DMSO (PCM)	DMSO (SMD)	CH_3OH (PCM)	CH_3OH (SMD)	C_2H_5OH (PCM)	C_2H_5OH (SMD)	H_2O (PCM)	H_2O (SMD)	Gas	$CHCl_3$ (PCM)	$CHCl_3$ (SMD)	DMSO (PCM)	DMSO (SMD)	CH_3OH (PCM)	CH_3OH (SMD)	C_2H_5OH (PCM)	C_2H_5OH (SMD)	H_2O (PCM)	H_2O (SMD)	
**C1**	138.6392	139.2893	140.2741	139.4897	140.9812	139.4671	140.1446	139.4631	140.2254	139.4815	140.2996	138.6392	139.2892	140.2741	139.4897	140.2338	139.4672	140.1447	139.4631	140.2253	139.4815	140.2996	**131.79**
**C2**	143.6180	143.5853	144.1635	143.3967	143.9441	143.3976	144.4462	143.4166	144.3166	143.3660	144.4531	143.6180	143.5853	144.1635	143.3967	144.1408	143.3976	144.4462	143.4168	144.3166	143.3659	144.4531	**131.93**
**C3**	123.6791	124.4243	125.1207	124.6773	125.4049	124.6536	125.6146	124.6479	125.5729	124.6695	125.6917	123.6791	124.4242	125.1207	124.6773	125.4214	124.6537	125.6146	124.6479	125.5729	124.6693	125.6917	**116.53**
**C4**	174.8189	175.5463	176.2178	175.9829	176.5818	175.9460	177.2668	175.9274	177.1168	175.9878	177.4192	174.8190	175.5463	176.2178	175.9829	176.7270	175.9459	177.2667	175.9275	177.1168	175.9878	177.4192	**168.1**
**C5**	121.4880	122.8883	123.6214	123.6417	124.5170	123.5852	124.7004	123.5472	124.6068	123.6651	124.8658	121.4880	122.8883	123.6214	123.6417	124.3858	123.5851	124.7004	123.5472	124.6068	123.6651	124.8658	**116.51**
**C6**	139.7530	141.7171	142.7531	142.8378	144.4941	142.7554	145.1509	142.6914	144.9187	142.8863	145.6139	139.7530	141.7171	142.7531	142.8378	143.5819	142.7552	145.1510	142.6913	144.9187	142.8863	145.6139	**131.88**
**C7**	204.0200	208.5299	209.8118	211.0136	211.4334	210.8507	216.6692	210.7065	215.4841	211.1391	217.6266	204.0201	208.5300	209.8118	211.0136	211.7577	210.8508	216.6695	210.7068	215.4841	211.1388	217.6266	**195.2**
**C8**	60.0629	61.2181	61.7782	61.7237	62.4634	61.6795	63.1245	61.6541	62.9853	61.7361	63.5882	60.0629	61.2181	61.7782	61.7237	62.4678	61.6794	63.1245	61.6542	62.9853	61.7361	63.5882	**54.87**
**C9**	34.0304	33.8239	33.9730	33.6923	34.0343	33.6886	34.1544	33.7035	34.0820	33.6660	34.3061	34.0305	33.8240	33.9730	33.6923	34.4364	33.6886	34.1544	33.7035	34.0820	33.6661	34.3061	**31.13**
**C10**	25.2679	25.8006	26.1146	26.0254	26.7993	25.9980	26.5173	25.9890	26.5188	26.0215	26.7306	25.2679	25.8006	26.1146	26.0254	26.7695	25.9980	26.5173	25.9890	26.5188	26.0215	26.7306	**30.82**
**C11**	35.3003	35.3330	35.7168	35.2551	35.7623	35.2494	35.7712	35.2622	35.7494	35.2309	35.9759	35.3004	35.3329	35.7168	35.2551	35.9992	35.2493	35.7713	35.2622	35.7494	35.2310	35.9759	**29.11**
**C12**	37.3792	37.7038	38.0091	37.9152	38.4186	37.8914	38.2389	37.8858	38.2601	37.9077	38.3532	37.3791	37.7039	38.0091	37.9152	38.6593	37.8915	38.2389	37.8858	38.2601	37.9077	38.3532	**25.52**
**C13**	26.7117	26.9103	27.1369	26.9798	27.2221	26.9655	27.2254	26.9698	27.2194	26.9635	27.2471	26.7117	26.9104	27.1369	26.9798	27.7239	26.9656	27.2254	26.9698	27.2194	26.9634	27.2471	**22.36**
**C14**	15.7965	15.9832	16.0403	16.0871	16.1379	16.0698	16.1099	16.0708	16.1186	16.0737	16.1574	15.7965	15.9832	16.0403	16.0871	16.8312	16.0698	16.1100	16.0708	16.1186	16.0737	16.1574	**13.85**
**C1`**	48.3744	49.1967	49.4608	49.6885	50.1596	49.6499	50.2257	49.6302	50.1525	49.6953	50.6296	52.1935	53.2710	53.6481	53.7873	54.5314	53.7431	54.4348	53.7175	54.3940	53.7991	54.7570	**51.23**
**C2`**	21.5794	22.0519	22.3154	22.2316	22.6173	22.2086	22.7071	22.2043	22.7041	22.2235	22.6995	21.2208	21.4108	21.7851	21.5130	22.2571	21.4962	21.8977	21.4978	21.9033	21.4988	21.8255	**23.85**
**C3`**	21.2208	21.4108	21.7851	21.5130	22.0184	21.4962	21.8978	21.4978	21.9033	21.4987	21.8255	21.5794	22.0520	22.3154	22.2316	22.9757	22.2086	22.7071	22.2042	22.7041	22.2234	22.6995	**23.66**
**C4`**	52.1935	53.2710	53.6481	53.7873	54.2837	53.7431	54.4347	53.7176	54.3940	53.7990	54.7570	48.3744	49.1967	49.4608	49.6885	50.4326	49.6499	50.2257	49.6302	50.1525	49.6953	50.6296	**52.72**
**H2**	9.0235	8.9318	8.9487	8.8693	8.8865	8.8737	8.9047	8.8772	8.9024	8.8662	8.9029	9.0235	8.9318	8.9487	8.8693	8.7881	8.8737	8.9047	8.8772	8.9024	8.8662	8.9029	**8.13**
**H3**	7.4545	7.5964	7.6930	7.6419	7.7659	7.6405	7.7843	7.6383	7.7766	7.6442	7.7991	7.4545	7.5964	7.6930	7.6419	7.5607	7.6405	7.7843	7.6383	7.7766	7.6441	7.7991	**7.22**
**H5**	7.2781	7.4732	7.5675	7.5576	7.6925	7.5534	7.7041	7.5483	7.6934	7.5627	7.7271	7.2781	7.4732	7.5675	7.5576	7.4764	7.5534	7.7041	7.5483	7.6934	7.5627	7.7271	**7.22**
**H6**	8.1852	8.3700	8.4587	8.4675	8.6232	8.4616	8.6715	8.4549	8.6540	8.4741	8.7156	8.1852	8.3700	8.4587	8.4675	8.3863	8.4616	8.6715	8.4549	8.6540	8.4741	8.7156	**8.13**
**H8**	4.2740	4.3303	4.3803	4.3745	4.4715	4.3716	4.5023	4.3678	4.4886	4.3784	4.5298	4.2740	4.3303	4.3803	4.3745	4.2933	4.3716	4.5023	4.3678	4.4886	4.3784	4.5298	**5.5**
**H(9A-9B)**	1.7836	1.8237	1.7948	1.8463	1.8609	1.8451	1.8555	1.8432	1.8496	1.8485	1.8934	1.7836	1.8237	1.7948	1.8463	1.7651	1.8451	1.8555	1.8432	1.8496	1.8485	1.8934	**2.08**
**H(10-13A-10-13B)**	1.1768	1.1397	1.1063	1.1190	1.0697	1.1210	1.0701	1.1221	1.0702	1.1182	1.0666	1.1768	1.1397	1.1063	1.1190	1.0378	1.1210	1.0701	1.1221	1.0702	1.1182	1.0666	**1.25**
**H(14A-14C)**	0.9614	0.9531	0.9292	0.9453	0.9134	0.9466	0.9136	0.9469	0.9135	0.9450	0.9132	0.9614	0.9531	0.9292	0.9453	0.8640	0.9466	0.9136	0.9470	0.9135	0.9451	0.9132	**0.79**
**H(1`A-1`B)**	2.8481	2.8609	2.8597	2.8630	2.7618	2.8635	2.8669	2.8631	2.8659	2.8635	2.8610	2.8239	2.7243	2.6937	2.6794	2.6569	2.6829	2.6256	2.6855	2.6285	2.6772	2.6040	**3.69**
**H(2`-3`A-2`-3`B)**	1.6283	1.6812	1.6442	1.7104	1.6670	1.7091	1.6829	1.7069	1.6761	1.7127	1.7126	1.6283	1.6811	1.6442	1.7104	1.6292	1.7091	1.6829	1.7069	1.6761	1.7128	1.7126	**2.18**
**H(4`A)**	2.9163	2.7062	2.6663	2.6324	2.4855	2.6373	2.5520	2.6414	2.5551	2.6290	2.5375	3.0981	3.1297	3.1526	3.1513	3.2207	3.1501	3.1950	3.1483	3.1935	3.1534	3.2029	**3.12**
**H(4`B)**	2.7315	2.7424	2.7210	2.7264	2.5684	2.7284	2.6992	2.7296	2.7019	2.7255	2.6706	2.5982	2.5920	2.5668	2.5746	2.5276	2.5767	2.5387	2.5779	2.5383	2.5736	2.5191	**3.86**
Nuclei	BHandHLYP																						
**C1**	139.1928	139.9013	140.7112	140.1569	142.5336	140.1385	141.5500	140.1233	141.7502	140.1750	142.0334	139.1929	139.9013	140.7111	140.1569	142.5334	140.1385	141.5501	140.1233	141.7502	140.1750	142.0331	**131.79**
**C2**	142.1655	142.1322	142.6342	141.9776	142.3637	141.9894	142.8794	142.0035	142.7529	141.9658	142.8286	142.1654	142.1322	142.6342	141.9776	142.3638	141.9896	142.8793	142.0035	142.7529	141.9658	142.8286	**131.93**
**C3**	122.5031	123.1334	123.7060	123.3234	124.0129	123.3150	124.2155	123.3087	124.1826	123.3306	124.2942	122.5030	123.1334	123.7061	123.3234	124.0130	123.3151	124.2155	123.3087	124.1826	123.3306	124.2943	**116.53**
**C4**	174.6361	175.2921	175.9223	175.6698	176.1555	175.6474	176.8358	175.6275	176.6732	175.6908	176.9422	174.6361	175.2922	175.9224	175.6698	176.1556	175.6475	176.8359	175.6274	176.6732	175.6907	176.9424	**168.1**
**C5**	120.2659	121.5074	122.1204	122.1537	122.9907	122.1148	123.1594	122.0783	123.0879	122.1906	123.3556	120.2659	121.5074	122.1204	122.1537	122.9907	122.1149	123.1594	122.0782	123.0879	122.1905	123.3556	**116.51**
**C6**	138.0345	139.8676	140.8657	141.0077	142.0466	140.9359	142.8027	140.8664	142.5355	141.0756	143.0370	138.0345	139.8675	140.8657	141.0077	142.0466	140.9358	142.8030	140.8664	142.5355	141.0756	143.0369	**131.88**
**C7**	202.1610	207.0232	208.2075	209.6662	210.9423	209.4976	216.5725	209.3331	215.3852	209.8275	217.9373	202.1614	207.0232	208.2072	209.6662	210.9423	209.4975	216.5725	209.3331	215.3852	209.8275	217.9374	**195.2**
**C8**	61.8693	62.8947	63.3940	63.3931	64.3258	63.3590	64.8884	63.3280	64.8140	63.4260	65.2323	61.8694	62.8947	63.3939	63.3931	64.3258	63.3591	64.8885	63.3281	64.8140	63.4260	65.2323	**54.87**
**C9**	34.0695	33.7412	33.8469	33.6151	33.7048	33.6229	33.6939	33.6332	33.7078	33.6073	33.7290	34.0694	33.7412	33.8469	33.6151	33.7048	33.6229	33.6939	33.6332	33.7078	33.6073	33.7290	**31.13**
**C10**	25.7789	26.2817	26.5863	26.5654	27.1335	26.5479	26.9323	26.5327	26.9407	26.5818	27.0472	25.7789	26.2817	26.5863	26.5654	27.1334	26.5478	26.9324	26.5327	26.9407	26.5818	27.0472	**30.82**
**C11**	35.1028	35.1280	35.5073	35.0932	35.5837	35.0978	35.5415	35.1046	35.5540	35.0881	35.7227	35.1029	35.1280	35.5073	35.0932	35.5838	35.0978	35.5414	35.1046	35.5540	35.0883	35.7227	**29.11**
**C12**	36.6734	36.9553	37.2405	37.1545	37.6318	37.1408	37.5146	37.1298	37.5133	37.1672	37.5952	36.6734	36.9553	37.2406	37.1545	37.6318	37.1409	37.5145	37.1298	37.5133	37.1672	37.5953	**25.52**
**C13**	27.8430	28.0148	28.2417	28.0882	28.3720	28.0846	28.3550	28.0833	28.3650	28.0912	28.3787	27.8430	28.0147	28.2418	28.0883	28.3721	28.0847	28.3550	28.0833	28.3650	28.0912	28.3788	**22.36**
**C14**	16.6334	16.7928	16.8568	16.9023	16.9934	16.8951	16.9712	16.8905	16.9770	16.9088	17.0098	16.6335	16.7928	16.8568	16.9023	16.9934	16.8952	16.9712	16.8905	16.9770	16.9088	17.0097	**13.85**
**C1`**	49.2159	49.9659	50.2127	50.4295	50.9631	50.3993	50.8971	50.3717	50.8545	50.4581	51.3450	53.3066	54.2167	54.6159	54.7419	55.1964	54.7107	55.3844	54.6815	55.3087	54.7711	55.6956	**51.23**
**C2`**	24.3806	24.7355	25.0468	24.9061	25.3240	24.8946	25.3705	24.8857	25.3842	24.9169	25.2014	23.9773	24.1581	24.5203	24.2206	24.8261	24.2173	24.6874	24.2165	24.7090	24.2235	24.4923	**23.85**
**C3`**	23.9774	24.1581	24.5203	24.2206	24.8261	24.2173	24.6874	24.2166	24.7090	24.2236	24.4923	24.3806	24.7355	25.0468	24.9061	25.3240	24.8947	25.3706	24.8856	25.3842	24.9169	25.2015	**23.66**
**C4`**	53.3066	54.2167	54.6158	54.7419	55.1963	54.7106	55.3845	54.6815	55.3087	54.7711	55.6956	49.2157	49.9659	50.2128	50.4295	50.9630	50.3992	50.8971	50.3717	50.8545	50.4582	51.3450	**52.72**
**H2**	8.9169	8.8163	8.8513	8.7600	8.6681	8.7642	8.7158	8.7683	8.7024	8.7557	8.6792	8.9169	8.8163	8.8513	8.7600	8.6681	8.7642	8.7158	8.7683	8.7024	8.7557	8.6792	**8.13**
**H3**	7.4454	7.5769	7.6704	7.6188	7.7282	7.6170	7.7465	7.6150	7.7375	7.6205	7.7606	7.4454	7.5769	7.6704	7.6188	7.7282	7.6170	7.7465	7.6150	7.7375	7.6205	7.7606	**7.22**
**H5**	7.2614	7.4455	7.5353	7.5273	7.6449	7.5229	7.6690	7.5182	7.6563	7.5315	7.6944	7.2614	7.4455	7.5353	7.5273	7.6449	7.5229	7.6690	7.5182	7.6563	7.5315	7.6944	**7.22**
**H6**	8.0527	8.2502	8.3488	8.3665	8.4936	8.3595	8.5791	8.3524	8.5490	8.3730	8.5954	8.0527	8.2502	8.3488	8.3665	8.4936	8.3595	8.5791	8.3524	8.5490	8.3730	8.5953	**8.13**
**H8**	4.2074	4.2966	4.3546	4.3558	4.4558	4.3521	4.5242	4.3483	4.4984	4.3594	4.5145	4.2074	4.2966	4.3546	4.3558	4.4557	4.3521	4.5242	4.3483	4.4984	4.3594	4.5144	**5.5**
**H(9A-9B)**	1.5691	1.6158	1.5988	1.6579	1.6068	1.6553	1.6070	1.6526	1.6026	1.6603	1.6274	1.5690	1.6158	1.5988	1.6579	1.6068	1.6553	1.6070	1.6526	1.6026	1.6603	1.6274	**2.08**
**H(10-13A-10-13B)**	1.1338	1.0990	1.0700	1.0854	1.0310	1.0864	1.0413	1.0873	1.0384	1.0843	1.0380	1.1338	1.0990	1.0700	1.0854	1.0309	1.0864	1.0413	1.0873	1.0384	1.0844	1.0380	**1.25**
**H(14A-14C)**	0.9551	0.9463	0.9245	0.9431	0.9122	0.9435	0.9176	0.9438	0.9158	0.9427	0.9177	0.9551	0.9463	0.9245	0.9431	0.9122	0.9435	0.9176	0.9438	0.9158	0.9427	0.9177	**0.79**
**H(1`A-1`B)**	2.8160	2.8373	2.8425	2.8531	2.8538	2.8523	2.8652	2.8514	2.8617	2.8538	2.8554	2.7349	2.6598	2.6184	2.6252	2.6227	2.6270	2.5982	2.6287	2.6043	2.6236	2.5875	**3.69**
**H(2`-3`A-2`-3`B)**	1.5840	1.6433	1.6048	1.6804	1.6404	1.6781	1.6638	1.6757	1.6557	1.6825	1.6923	1.5840	1.6433	1.6049	1.6804	1.6404	1.6781	1.6638	1.6758	1.6557	1.6825	1.6923	**2.18**
**H(4`A)**	2.7250	2.5834	2.5238	2.5377	2.5616	2.5398	2.5008	2.5420	2.5061	2.5358	2.5122	3.0171	3.0646	3.0971	3.1088	3.1630	3.1060	3.1649	3.1031	3.1581	3.1115	3.1696	**3.12**
**H(4`B)**	2.7449	2.7361	2.7129	2.7127	2.6836	2.7141	2.6956	2.7155	2.7024	2.7113	2.6629	2.6148	2.6099	2.5879	2.5974	2.5446	2.5986	2.5654	2.5997	2.5652	2.5961	2.5411	**3.86**

[a] methyl protons shifts are averaged

[b] according to [12]

Table S13. Cont. IV

Nuclei	R											S											Exp. [b]
	BLYP																						
	Gas	$CHCl_3$ (PCM)	$CHCl_3$ (SMD)	DMSO (PCM)	DMSO (SMD)	CH_3OH (PCM)	CH_3OH (SMD)	C_2H_5OH (PCM)	C_2H_5OH (SMD)	H_2O (PCM)	H_2O (SMD)	Gas	$CHCl_3$ (PCM)	$CHCl_3$ (SMD)	DMSO (PCM)	DMSO (SMD)	CH_3OH (PCM)	CH_3OH (SMD)	C_2H_5OH (PCM)	C_2H_5OH (SMD)	H_2O (PCM)	H_2O (SMD)	
**C1**	139.6091	141.0335	141.7561	141.9121	143.2565	142.1359	142.2988	141.8987	142.5049	142.3276	142.9824	139.6091	141.0335	141.7561	141.9119	143.2565	142.1360	142.2988	141.8987	142.5050	142.3277	142.9824	**131.79**
**C2**	137.4832	137.5151	138.0115	137.2567	137.9429	137.5560	138.1711	137.3924	138.2743	137.5982	138.2031	137.4832	137.5151	138.0116	137.2567	137.9429	137.5561	138.1711	137.3924	138.2743	137.5982	138.2031	**131.93**
**C3**	120.8887	121.5245	122.0251	121.7467	122.3655	122.0116	122.5178	121.8149	122.6425	122.1226	122.6245	120.8887	121.5245	122.0252	121.7468	122.3655	122.0116	122.5178	121.8149	122.6425	122.1227	122.6245	**116.53**
**C4**	178.0063	178.8497	179.5600	179.1766	180.0903	179.4342	180.4946	179.2298	180.5444	179.5594	180.6774	178.0063	178.8497	179.5600	179.1767	180.0903	179.4341	180.4946	179.2298	180.5443	179.5594	180.6774	**168.1**
**C5**	118.7005	119.9642	120.4428	120.6576	121.3347	120.8935	121.4722	120.6674	121.5448	121.0607	121.7331	118.7005	119.9642	120.4429	120.6577	121.3347	120.8936	121.4722	120.6674	121.5448	121.0607	121.7331	**116.51**
**C6**	133.4320	135.1829	136.2363	136.1198	137.8295	136.3391	138.3709	136.0964	138.3047	136.5383	138.7340	133.4320	135.1829	136.2363	136.1198	137.8295	136.3392	138.3709	136.0964	138.3048	136.5384	138.7340	**131.88**
**C7**	203.9340	209.0041	210.2638	212.0040	212.6151	212.0927	216.8718	211.7208	216.0190	212.5486	218.4142	203.9340	209.0041	210.2637	212.0038	212.6151	212.0927	216.8718	211.7208	216.0191	212.5486	218.4142	**195.2**
**C8**	70.4214	71.7823	72.6560	72.5572	73.5816	72.7895	74.0549	72.5589	74.0361	72.9627	74.5550	70.4214	71.7823	72.6560	72.5572	73.5816	72.7895	74.0549	72.5589	74.0361	72.9628	74.5550	**54.87**
**C9**	39.9894	39.6157	39.7225	39.3191	39.7714	39.6231	39.9218	39.4633	39.9995	39.6560	39.9992	39.9894	39.6157	39.7224	39.3192	39.7714	39.6231	39.9218	39.4633	39.9994	39.6561	39.9992	**31.13**
**C10**	29.3739	29.8045	30.2251	29.9824	30.8272	30.2497	30.4966	30.0547	30.6746	30.3554	30.6518	29.3739	29.8045	30.2252	29.9824	30.8272	30.2497	30.4966	30.0547	30.6744	30.3554	30.6518	**30.82**
**C11**	39.0906	39.1884	39.4835	39.0427	39.7098	39.3344	39.4960	39.1632	39.6675	39.3920	39.7470	39.0906	39.1884	39.4836	39.0427	39.7098	39.3344	39.4960	39.1632	39.6675	39.3921	39.7470	**29.11**
**C12**	40.7888	41.0421	41.3481	41.2352	41.8084	41.4982	41.5172	41.3000	41.7029	41.6132	41.6686	40.7888	41.0421	41.3481	41.2352	41.8084	41.4982	41.5172	41.3000	41.7029	41.6131	41.6686	**25.52**
**C13**	31.8876	32.0852	32.2413	32.0972	32.4670	32.3764	32.2796	32.1932	32.4563	32.4590	32.3559	31.8876	32.0852	32.2413	32.0971	32.4670	32.3764	32.2796	32.1932	32.4562	32.4590	32.3559	**22.36**
**C14**	17.1634	17.3215	17.3350	17.3944	17.5443	17.6674	17.3535	17.4786	17.5317	17.7624	17.4539	17.1634	17.3215	17.3350	17.3944	17.5443	17.6674	17.3535	17.4786	17.5317	17.7624	17.4539	**13.85**
**C1`**	55.4135	56.1867	56.4431	56.7162	57.3333	56.9548	57.1063	56.7327	57.2310	57.1181	57.7471	60.0607	61.0163	61.3737	61.5481	62.1017	61.7899	62.1836	61.5703	62.2851	61.9459	62.6051	**51.23**
**C2`**	27.6364	28.1277	28.4947	28.3520	28.9347	28.6180	28.8639	28.4212	29.0152	28.7256	28.8135	27.5355	27.9777	28.2496	28.1832	28.7414	28.4485	28.4046	28.2520	28.5877	28.5588	28.3062	**23.85**
**C3`**	27.5355	27.9777	28.2496	28.1833	28.7414	28.4484	28.4046	28.2520	28.5877	28.5588	28.3062	27.6364	28.1277	28.4947	28.3519	28.9347	28.6180	28.8639	28.4212	29.0151	28.7256	28.8135	**23.66**
**C4`**	60.0607	61.0163	61.3738	61.5481	62.1017	61.7900	62.1836	61.5703	62.2851	61.9459	62.6051	55.4135	56.1867	56.4430	56.7162	57.3333	56.9547	57.1063	56.7327	57.2310	57.1181	57.7471	**52.72**
**H2**	8.4456	8.2914	8.3204	8.1864	8.2206	8.2400	8.4282	8.2011	8.2664	8.2357	8.4076	8.4456	8.2914	8.3204	8.1864	8.2206	8.2400	8.4282	8.2011	8.2664	8.2357	8.4076	**8.13**
**H3**	7.1848	7.2868	7.3787	7.3104	7.4359	7.3565	7.6219	7.3101	7.4480	7.3669	7.6461	7.1848	7.2868	7.3787	7.3104	7.4359	7.3565	7.6219	7.3101	7.4480	7.3669	7.6461	**7.22**
**H5**	6.9698	7.1544	7.2318	7.2558	7.3528	7.2970	7.5455	7.2457	7.3669	7.3170	7.5897	6.9698	7.1544	7.2318	7.2558	7.3528	7.2970	7.5455	7.2457	7.3669	7.3170	7.5897	**7.22**
**H6**	7.8379	8.0181	8.0910	8.1049	8.2229	8.1467	8.4574	8.0960	8.2779	8.1655	8.4752	7.8379	8.0181	8.0910	8.1049	8.2229	8.1467	8.4574	8.0960	8.2780	8.1655	8.4752	**8.13**
**H8**	4.9516	5.0023	5.0038	5.0252	5.0838	5.0711	5.2730	5.0244	5.0984	5.0821	5.2650	4.9516	5.0023	5.0038	5.0252	5.0838	5.0711	5.2730	5.0244	5.0984	5.0821	5.2650	**5.5**
**H(9A-9B)**	1.7580	1.7881	1.8062	1.7951	1.8478	1.8423	2.0274	1.7968	1.8524	1.8505	2.0566	1.7580	1.7881	1.8062	1.7951	1.8478	1.8423	2.0274	1.7968	1.8524	1.8505	2.0566	**2.08**
**H(10-13A-10-13B)**	1.2656	1.2387	1.1992	1.2249	1.1809	1.2730	1.3503	1.2286	1.1843	1.2796	1.3600	1.2656	1.2387	1.1992	1.2249	1.1809	1.2730	1.3503	1.2286	1.1843	1.2796	1.3600	**1.25**
**H(14A-14C)**	0.9625	0.9589	0.9331	0.9551	0.9275	1.0026	1.0965	0.9577	0.9296	1.0102	1.1087	0.9625	0.9589	0.9331	0.9551	0.9275	1.0026	1.0965	0.9577	0.9296	1.0101	1.1087	**0.79**
**H(1`A-1`B)**	3.1104	3.1271	3.1263	2.3128	3.1437	3.1822	3.3157	3.1366	3.1482	3.1907	3.3052	3.0069	2.9281	2.8991	2.0849	2.8873	2.9478	3.0387	2.9045	2.8714	2.9522	3.0365	**3.69**
**H(2`-3`A-2`-3`B)**	1.7292	1.7891	1.7535	1.8305	1.7840	1.8750	1.9690	1.8270	1.7970	1.8887	2.0143	1.7292	1.7891	1.7535	1.8305	1.7840	1.8750	1.9690	1.8270	1.7969	1.8887	2.0143	**2.18**
**H(4`A)**	3.0390	2.8875	2.8420	1.9974	2.8478	2.8981	2.9765	2.8552	2.8077	2.9021	2.9941	3.3579	3.3936	3.4186	2.5685	3.4891	3.4714	3.6522	3.4239	3.4821	3.4840	3.6582	**3.12**
**H(4`B)**	2.9749	2.9687	2.9562	2.1724	2.9267	2.9975	3.1009	2.9538	2.9350	3.0023	3.0790	2.8630	2.8607	2.8340	2.0571	2.7983	2.8930	2.9792	2.8494	2.8143	2.8976	2.9523	**3.86**
Nuclei	BMK																						
**C1**	154.7628	155.2412	156.0996	155.3902	156.9601	155.3645	156.0463	155.5496	156.1381	155.5766	156.6176	154.7626	155.2412	156.0996	155.3902	156.9601	155.3645	156.0463	155.5496	156.1381	155.5766	156.6176	**131.79**
**C2**	155.5428	155.2676	156.0638	154.9460	155.8212	154.9531	156.2002	155.1715	155.9140	155.1005	156.0044	155.5429	155.2675	156.0638	154.9460	155.8211	154.9531	156.2002	155.1715	155.9140	155.1005	156.0044	**131.93**
**C3**	136.1040	136.7626	137.6088	136.9946	138.0368	136.9656	138.3072	137.1477	138.0906	137.1833	137.9967	136.1038	136.7626	137.6088	136.9946	138.0368	136.9656	138.3072	137.1477	138.0906	137.1833	137.9967	**116.53**
**C4**	189.6047	190.2567	191.1020	190.6045	191.5969	190.5666	192.2410	190.7401	191.9495	190.8020	192.1636	189.6047	190.2566	191.1020	190.6045	191.5969	190.5666	192.2410	190.7401	191.9495	190.8020	192.1636	**168.1**
**C5**	133.4992	134.8983	135.6581	135.5731	136.6946	135.5164	136.8485	135.6709	136.6624	135.7886	136.9968	133.4994	134.8984	135.6581	135.5731	136.6946	135.5164	136.8485	135.6709	136.6624	135.7886	136.9968	**116.51**
**C6**	151.2388	153.0000	154.4171	154.2320	156.3033	154.1351	157.1478	154.2499	156.7099	154.4862	157.3021	151.2388	153.0001	154.4171	154.2320	156.3033	154.1351	157.1478	154.2499	156.7099	154.4862	157.3021	**131.88**
**C7**	218.2850	223.3781	224.3719	225.7748	226.1446	225.6024	232.0422	225.6438	230.7884	226.1019	233.8295	218.2853	223.3782	224.3719	225.7748	226.1446	225.6024	232.0422	225.6438	230.7884	226.1019	233.8295	**195.2**
**C8**	71.5254	73.0646	73.7488	73.7882	74.5456	73.7314	75.0997	73.8843	74.9236	74.0031	75.9921	71.5255	73.0647	73.7488	73.7882	74.5456	73.7314	75.0997	73.8843	74.9236	74.0031	75.9921	**54.87**
**C9**	39.0583	38.5435	38.7160	38.4136	38.9774	38.3944	39.1237	38.5878	39.0209	38.5948	39.4503	39.0583	38.5435	38.7160	38.4136	38.9774	38.3944	39.1237	38.5878	39.0209	38.5948	39.4503	**31.13**
**C10**	29.8532	30.1614	30.6113	30.3391	31.1543	30.3102	30.9005	30.4934	30.8267	30.5287	31.1104	29.8531	30.1614	30.6113	30.3391	31.1543	30.3102	30.9005	30.4934	30.8267	30.5288	31.1104	**30.82**
**C11**	39.0401	38.8641	39.3512	38.7731	39.5219	38.7637	39.4356	38.9647	39.3321	38.9428	39.5711	39.0401	38.8641	39.3512	38.7731	39.5219	38.7637	39.4356	38.9647	39.3321	38.9428	39.5711	**29.11**
**C12**	40.5806	40.8146	41.1751	40.9192	41.5867	40.8958	41.4176	41.0844	41.3188	41.1039	41.4364	40.5806	40.8145	41.1751	40.9192	41.5867	40.8957	41.4176	41.0844	41.3188	41.1039	41.4364	**25.52**
**C13**	30.9662	30.9782	31.2725	30.9590	31.4386	30.9450	31.3720	31.1418	31.2660	31.1337	31.3274	30.9663	30.9781	31.2725	30.9590	31.4386	30.9450	31.3720	31.1418	31.2660	31.1337	31.3274	**22.36**
**C14**	18.1325	18.1907	18.3060	18.2125	18.4520	18.1948	18.3887	18.3886	18.2933	18.3910	18.3836	18.1326	18.1907	18.3060	18.2125	18.4520	18.1948	18.3887	18.3886	18.2933	18.3910	18.3836	**13.85**
**C1`**	56.0789	56.7213	57.1191	57.0131	57.7800	56.9681	57.7027	57.1364	57.5327	57.2189	58.1918	60.5395	61.2398	61.7055	61.7555	62.3818	61.7207	62.5565	61.8939	62.3835	61.9472	62.6855	**51.23**
**C2`**	27.6957	28.1224	28.5016	28.4741	28.9656	28.4478	29.0152	28.6303	28.9166	28.6590	28.8898	27.1638	27.4561	27.9129	27.4105	28.0595	27.3771	27.9144	27.5598	27.8149	27.6075	27.8138	**23.85**
**C3`**	27.1639	27.4561	27.9129	27.4105	28.0595	27.3772	27.9144	27.5598	27.8149	27.6075	27.8138	27.6957	28.1223	28.5016	28.4741	28.9656	28.4479	29.0152	28.6303	28.9166	28.6590	28.8898	**23.66**
**C4`**	60.5394	61.2398	61.7055	61.7555	62.3818	61.7208	62.5565	61.8939	62.3835	61.9472	62.6855	56.0789	56.7212	57.1191	57.0131	57.7800	56.9681	57.7027	57.1364	57.5327	57.2189	58.1918	**52.72**
**H2**	9.1102	9.0152	9.0684	8.9549	8.9937	8.9556	9.0335	8.9562	9.0149	8.9448	8.9938	9.1102	9.0152	9.0684	8.9549	8.9937	8.9556	9.0335	8.9562	9.0149	8.9448	8.9938	**8.13**
**H3**	7.6269	7.7553	7.8601	7.8066	7.9398	7.8004	7.9774	7.7942	7.9619	7.8028	7.9586	7.6269	7.7553	7.8601	7.8066	7.9398	7.8004	7.9774	7.7942	7.9619	7.8028	7.9586	**7.22**
**H5**	7.4667	7.6906	7.7673	7.7866	7.9072	7.7775	7.9149	7.7684	7.9114	7.7857	7.9491	7.4667	7.6906	7.7673	7.7866	7.9072	7.7775	7.9149	7.7684	7.9114	7.7857	7.9491	**7.22**
**H6**	8.4055	8.5834	8.6734	8.6816	8.8134	8.6717	8.8515	8.6619	8.8335	8.6816	8.8621	8.4055	8.5834	8.6734	8.6816	8.8134	8.6717	8.8515	8.6619	8.8335	8.6816	8.8621	**8.13**
**H8**	4.4030	4.5004	4.5470	4.5709	4.6335	4.5626	4.6807	4.5544	4.6540	4.5694	4.6530	4.4030	4.5004	4.5470	4.5709	4.6335	4.5626	4.6807	4.5544	4.6540	4.5694	4.6530	**5.5**
**H(9A-9B)**	1.6844	1.6634	1.6400	1.6806	1.6983	1.6742	1.7132	1.6683	1.7132	1.6774	1.7764	1.6844	1.6634	1.6400	1.6806	1.6983	1.6742	1.7132	1.6683	1.7132	1.6774	1.7764	**2.08**
**H(10-13A-10-13B)**	1.1481	1.0926	1.0609	1.0673	1.0235	1.0653	1.0356	1.0635	1.0326	1.0598	1.0172	1.1481	1.0926	1.0609	1.0673	1.0235	1.0653	1.0356	1.0635	1.0326	1.0598	1.0172	**1.25**
**H(14A-14C)**	0.8768	0.8661	0.8384	0.8623	0.8259	0.8593	0.8301	0.8564	0.8306	0.8556	0.8270	0.8768	0.8661	0.8384	0.8623	0.8259	0.8593	0.8301	0.8564	0.8306	0.8556	0.8270	**0.79**
**H(1`A-1`B)**	2.8503	2.8506	2.8407	2.8621	2.8651	2.8586	2.8651	2.8553	2.8656	2.8558	2.8427	2.7257	2.6876	2.6498	2.6763	2.6386	2.6740	2.6140	2.6718	2.6239	2.6688	2.6217	**3.69**
**H(2`-3`A-2`-3`B)**	1.5999	1.6718	1.6311	1.7185	1.6599	1.7123	1.6787	1.7061	1.6729	1.7149	1.7022	1.5999	1.6718	1.6311	1.7185	1.6599	1.7123	1.6787	1.7061	1.6729	1.7149	1.7022	**2.18**
**H(4`A)**	2.7131	2.6174	2.5636	2.5803	2.5590	2.5780	2.4966	2.5760	2.5060	2.5732	2.5201	3.0782	3.0691	3.0810	3.0966	3.1568	3.0921	3.1516	3.0876	3.1519	3.0914	3.1518	**3.12**
**H(4`B)**	2.7382	2.7578	2.7360	2.7722	2.7183	2.7701	2.7315	2.7676	2.7419	2.7644	2.7233	2.6225	2.6323	2.6003	2.6275	2.5734	2.6252	2.5786	2.6229	2.5793	2.6201	2.5336	**3.86**

[a] methyl protons shifts are averaged

[b] according to [12]

Table S13. Cont. V

Nuclei	R											S											Exp. [b]
	BP86																						
	Gas	$CHCl_3$ (PCM)	$CHCl_3$ (SMD)	DMSO (PCM)	DMSO (SMD)	CH_3OH (PCM)	CH_3OH (SMD)	C_2H_5OH (PCM)	C_2H_5OH (SMD)	H_2O (PCM)	H_2O (SMD)	Gas	$CHCl_3$ (PCM)	$CHCl_3$ (SMD)	DMSO (PCM)	DMSO (SMD)	CH_3OH (PCM)	CH_3OH (SMD)	C_2H_5OH (PCM)	C_2H_5OH (SMD)	H_2O (PCM)	H_2O (SMD)	
C1	136.2321	137.2238	138.5261	137.6112	139.7629	137.5839	139.1691	137.5547	139.1902	137.6362	139.9830	136.2321	137.2238	138.5261	137.6112	139.7629	137.5839	139.1691	137.5547	139.1902	137.6361	139.9830	**131.79**
C2	136.0759	136.2532	136.7936	136.0369	136.5963	136.0629	137.0057	136.0828	136.9087	136.0065	137.1473	136.0759	136.2532	136.7936	136.0369	136.5963	136.0629	137.0057	136.0828	136.9087	136.0065	137.1473	**131.93**
C3	119.7505	120.6437	121.1724	120.8387	121.4424	120.8397	121.7912	120.8345	121.7083	120.8321	121.9703	119.7505	120.6437	121.1724	120.8387	121.4424	120.8397	121.7912	120.8345	121.7083	120.8321	121.9703	**116.53**
C4	174.5949	175.6154	176.2724	175.9947	176.6773	175.9818	177.2618	175.9629	177.1174	176.0015	177.5116	174.5949	175.6154	176.2724	175.9947	176.6773	175.9818	177.2618	175.9629	177.1174	176.0016	177.5116	**168.1**
C5	117.6308	119.0620	119.6482	119.6918	120.4940	119.6617	120.8350	119.6265	120.7092	119.7149	121.1578	117.6308	119.0620	119.6482	119.6918	120.4940	119.6617	120.8350	119.6265	120.7092	119.7151	121.1578	**116.51**
C6	132.0306	133.9623	134.8751	135.0256	136.4005	134.9656	137.0480	134.9008	136.8091	135.0775	137.4945	132.0306	133.9623	134.8751	135.0256	136.4006	134.9657	137.0480	134.9008	136.8091	135.0775	137.4945	**131.88**
C7	201.0410	205.3927	207.1230	207.7232	209.1847	207.5692	213.4787	207.4148	212.5240	207.8707	215.0883	201.0410	205.3927	207.1230	207.7232	209.1848	207.5692	213.4787	207.4148	212.5240	207.8707	215.0883	**195.2**
C8	68.1305	69.4688	70.2907	70.0812	71.0895	70.0403	71.8284	69.9974	71.6594	70.1194	72.4573	68.1305	69.4688	70.2907	70.0812	71.0896	70.0403	71.8284	69.9974	71.6594	70.1193	72.4573	**54.87**
C9	38.7037	38.5672	38.5201	38.4359	38.5330	38.4509	38.7492	38.4612	38.6584	38.4175	38.9866	38.7037	38.5672	38.5201	38.4359	38.5330	38.4509	38.7492	38.4612	38.6584	38.4176	38.9865	**31.13**
C10	27.9341	28.6185	28.8128	28.8373	29.3631	28.8311	29.1514	28.8198	29.1502	28.8386	29.4369	27.9341	28.6185	28.8128	28.8373	29.3631	28.8311	29.1514	28.8198	29.1502	28.8386	29.4369	**30.82**
C11	37.4166	37.5974	37.8544	37.4777	37.9244	37.4955	37.8867	37.5078	37.8582	37.4557	38.2358	37.4166	37.5974	37.8544	37.4777	37.9244	37.4955	37.8867	37.5078	37.8582	37.4556	38.2358	**29.11**
C12	39.0440	39.4512	39.6429	39.5778	39.9851	39.5768	39.8589	39.5707	39.8611	39.5739	40.0887	39.0440	39.4512	39.6429	39.5778	39.9851	39.5768	39.8589	39.5707	39.8611	39.5741	40.0887	**25.52**
C13	30.0622	30.3534	30.4831	30.3425	30.5556	30.3523	30.5470	30.3568	30.5325	30.3280	30.6960	30.0622	30.3534	30.4831	30.3425	30.5556	30.3523	30.5470	30.3568	30.5325	30.3280	30.6960	**22.36**
C14	16.6708	16.9480	16.8978	16.9780	16.9739	16.9838	16.9551	16.9846	16.9416	16.9678	17.1358	16.6708	16.9480	16.8978	16.9780	16.9739	16.9838	16.9552	16.9846	16.9416	16.9677	17.1357	**13.85**
C1`	53.8804	54.7724	54.9840	55.1753	55.7000	55.1572	55.6437	55.1342	55.5702	55.1877	56.3637	58.2661	59.4131	59.8086	59.9303	60.3455	59.9034	60.6832	59.8719	60.5898	59.9517	61.1879	**51.23**
C2`	26.1253	26.7421	26.9843	26.9475	27.2797	26.9396	27.3959	26.9275	27.3649	26.9511	27.4087	25.9219	26.4540	26.7334	26.5213	27.0486	26.5309	26.9363	26.5337	26.9285	26.5056	26.8449	**23.85**
C3`	25.9219	26.4540	26.7334	26.5213	27.0486	26.5309	26.9363	26.5337	26.9285	26.5056	26.8449	26.1253	26.7421	26.9843	26.9475	27.2797	26.9396	27.3959	26.9275	27.3649	26.9511	27.4087	**23.66**
C4`	58.2661	59.4131	59.8086	59.9303	60.3455	59.9033	60.6832	59.8719	60.5898	59.9517	61.1879	53.8804	54.7724	54.9840	55.1753	55.7000	55.1572	55.6437	55.1342	55.5702	55.1876	56.3637	**52.72**
H2	8.5234	8.4310	8.3850	8.3614	8.2790	8.3684	8.3139	8.3746	8.3150	8.3538	8.2872	8.5234	8.4310	8.3850	8.3614	8.2790	8.3684	8.3139	8.3746	8.3150	8.3538	8.2872	**8.13**
H3	7.1939	7.3249	7.3954	7.3623	7.4581	7.3616	7.4744	7.3605	7.4658	7.3625	7.5038	7.1939	7.3249	7.3954	7.3623	7.4581	7.3616	7.4744	7.3605	7.4658	7.3625	7.5038	**7.22**
H5	6.9895	7.1742	7.2496	7.2535	7.3697	7.2498	7.3979	7.2458	7.3849	7.2566	7.4369	6.9895	7.1742	7.2496	7.2535	7.3697	7.2498	7.3979	7.2458	7.3849	7.2566	7.4369	**7.22**
H6	7.9040	8.0756	8.1488	8.1731	8.2748	8.1683	8.3473	8.1629	8.3318	8.1771	8.3542	7.9040	8.0756	8.1488	8.1731	8.2748	8.1683	8.3473	8.1629	8.3318	8.1771	8.3542	**8.13**
H8	4.9624	5.0171	5.0267	5.0431	5.0997	5.0428	5.1307	5.0419	5.1199	5.0429	5.1193	4.9624	5.0171	5.0267	5.0431	5.0997	5.0428	5.1307	5.0419	5.1199	5.0429	5.1193	**5.5**
H(9A-9B)	1.8498	1.8952	1.8585	1.9364	1.9050	1.9340	1.9001	1.9315	1.8954	1.9385	1.9359	1.8498	1.8952	1.8585	1.9364	1.9050	1.9340	1.9001	1.9315	1.8954	1.9385	1.9359	**2.08**
H(10-13A-10-13B)	1.2593	1.2328	1.1896	1.2179	1.1644	1.2197	1.1661	1.2211	1.1657	1.2158	1.1754	1.2593	1.2328	1.1896	1.2179	1.1644	1.2197	1.1661	1.2211	1.1657	1.2158	1.1754	**1.25**
H(14A-14C)	0.9338	0.9320	0.9007	0.9273	0.8909	0.9284	0.8922	0.9293	0.8906	0.9258	0.9057	0.9338	0.9320	0.9007	0.9273	0.8909	0.9284	0.8922	0.9293	0.8906	0.9258	0.9057	**0.79**
H(1`A-1`B)	3.0868	3.0996	3.0959	3.1109	3.1092	3.1108	3.1133	3.1104	3.1121	3.1106	3.1074	3.0181	2.9246	2.8881	2.8864	2.8724	2.8890	2.8459	2.8914	2.8467	2.8838	2.8427	**3.69**
H(2`-3`A-2`-3`B)	1.7268	1.7828	1.7426	1.8139	1.7685	1.8126	1.7871	1.8111	1.7803	1.8148	1.8363	1.7268	1.7828	1.7426	1.8139	1.7685	1.8126	1.7871	1.8111	1.7803	1.8148	1.8363	**2.18**
H(4`A)	3.1049	2.9263	2.8699	2.8634	2.8681	2.8674	2.8045	2.8713	2.8037	2.8594	2.8197	3.3627	3.3857	3.4107	3.4248	3.4747	3.4225	3.4707	3.4200	3.4672	3.4267	3.4818	**3.12**
H(4`B)	2.9314	2.9230	2.9064	2.9095	2.8766	2.9106	2.8874	2.9116	2.8896	2.9082	2.8657	2.8110	2.8136	2.7811	2.7970	2.7436	2.7991	2.7560	2.8009	2.7569	2.7945	2.7329	**3.86**
Nuclei	CAM-B3LYP																						
C1	140.9282	141.7944	143.3001	142.1583	144.6701	142.1313	143.8095	142.1077	143.9213	142.1899	144.4637	140.9282	141.7945	143.3003	142.1581	144.6702	142.1313	143.8094	142.1078	143.9212	142.1899	144.4638	**131.79**
C2	142.5117	142.5240	142.9418	142.3130	142.7791	142.3309	143.2584	142.3486	143.1407	142.2974	143.2194	142.5118	142.5240	142.9418	142.3129	142.7791	142.3310	143.2586	142.3486	143.1407	142.2974	143.2196	**131.93**
C3	123.8388	124.5284	125.1568	124.7622	125.4532	124.7589	125.6886	124.7556	125.6266	124.7671	125.7737	123.8389	124.5285	125.1567	124.7622	125.4534	124.7589	125.6886	124.7555	125.6265	124.7671	125.7737	**116.53**
C4	177.7941	178.5081	179.0686	178.8493	179.4281	178.8318	180.0541	178.8147	179.8952	178.8674	180.1392	177.7941	178.5081	179.0686	178.8493	179.4280	178.8319	180.0541	178.8147	179.8952	178.8673	180.1392	**168.1**
C5	121.7724	123.0645	123.6934	123.7054	124.5767	123.6695	124.7831	123.6344	124.6811	123.7417	124.9750	121.7724	123.0645	123.6933	123.7054	124.5767	123.6697	124.7832	123.6344	124.6812	123.7417	124.9749	**116.51**
C6	138.4383	140.3040	141.0010	141.3652	142.5633	141.3000	143.2030	141.2348	142.9652	141.4289	143.4117	138.4382	140.3040	141.0010	141.3652	142.5633	141.3001	143.2029	141.2348	142.9652	141.4289	143.4115	**131.88**
C7	206.1039	211.0612	212.9813	213.7932	215.2696	213.6136	220.6786	213.4387	219.5080	213.9711	222.2280	206.1036	211.0612	212.9810	213.7932	215.2692	213.6135	220.6787	213.4390	219.5080	213.9711	222.2276	**195.2**
C8	64.6013	65.7447	66.5120	66.3294	67.3255	66.2911	67.9295	66.2548	67.8194	66.3699	68.3706	64.6013	65.7446	66.5120	66.3295	67.3255	66.2911	67.9295	66.2548	67.8194	66.3699	68.3705	**54.87**
C9	35.3318	35.0465	34.9613	34.8735	34.9924	34.8858	35.0267	34.8985	35.0119	34.8635	35.0542	35.3318	35.0465	34.9613	34.8734	34.9924	34.8858	35.0267	34.8986	35.0120	34.8635	35.0541	**31.13**
C10	26.5810	27.1352	27.3679	27.3952	27.9953	27.3836	27.7848	27.3724	27.7788	27.4086	27.8989	26.5810	27.1352	27.3679	27.3952	27.9953	27.3836	27.7848	27.3723	27.7787	27.4086	27.8991	**30.82**
C11	36.1354	36.2004	36.5146	36.1549	36.6627	36.1648	36.6332	36.1750	36.6106	36.1473	36.8146	36.1355	36.2005	36.5145	36.1549	36.6627	36.1648	36.6333	36.1749	36.6106	36.1473	36.8145	**29.11**
C12	37.8378	38.1619	38.4092	38.3354	38.8460	38.3268	38.7204	38.3188	38.7131	38.3459	38.8078	37.8378	38.1619	38.4091	38.3354	38.8461	38.3268	38.7205	38.3188	38.7132	38.3459	38.8078	**25.52**
C13	28.4937	28.7030	28.8782	28.7557	29.0518	28.7573	29.0403	28.7592	29.0286	28.7561	29.0559	28.4937	28.7030	28.8781	28.7556	29.0518	28.7573	29.0403	28.7592	29.0286	28.7561	29.0559	**22.36**
C14	16.4442	16.6417	16.6533	16.7294	16.8275	16.7270	16.8087	16.7253	16.7971	16.7339	16.8441	16.4444	16.6417	16.6533	16.7294	16.8276	16.7270	16.8087	16.7252	16.7970	16.7339	16.8441	**13.85**
C1`	51.0019	51.8246	51.9724	52.2129	52.8555	52.1850	52.7762	52.1578	52.7042	52.2423	53.2630	55.1214	56.1291	56.4963	56.6661	57.1489	56.6367	57.3497	56.6075	57.2520	56.6960	57.6735	**51.23**
C2`	24.9465	25.3520	25.6480	25.5480	25.9499	25.5397	25.9956	25.5320	25.9904	25.5581	25.8215	24.5881	24.8130	25.1503	24.8404	25.4828	24.8433	25.3433	24.8466	25.3463	24.8398	25.1418	**23.85**
C3`	24.5882	24.8130	25.1503	24.8405	25.4828	24.8434	25.3433	24.8467	25.3463	24.8398	25.1418	24.9465	25.3519	25.6480	25.5481	25.9498	25.5397	25.9956	25.5320	25.9904	25.5581	25.8214	**23.66**
C4`	55.1213	56.1291	56.4964	56.6661	57.1489	56.6366	57.3499	56.6074	57.2520	56.6960	57.6734	51.0019	51.8245	51.9725	52.2130	52.8555	52.1850	52.7763	52.1578	52.7042	52.2423	53.2631	**52.72**
H2	8.8151	8.7078	8.6586	8.6372	8.5486	8.6433	8.5851	8.6489	8.5804	8.6309	8.5389	8.8151	8.7078	8.6586	8.6372	8.5487	8.6433	8.5851	8.6489	8.5804	8.6309	8.5389	**8.13**
H3	7.4066	7.5369	7.6238	7.5786	7.6900	7.5770	7.7070	7.5754	7.6992	7.5799	7.7229	7.4066	7.5369	7.6238	7.5786	7.6900	7.5770	7.7070	7.5754	7.6992	7.5799	7.7229	**7.22**
H5	7.2382	7.4199	7.5023	7.4959	7.6208	7.4914	7.6424	7.4870	7.6307	7.5001	7.6661	7.2382	7.4199	7.5023	7.4959	7.6208	7.4914	7.6424	7.4870	7.6307	7.5001	7.6661	**7.22**
H6	8.0282	8.2138	8.3196	8.3378	8.4424	8.3316	8.5141	8.3255	8.4898	8.3436	8.5205	8.0282	8.2138	8.3196	8.3378	8.4424	8.3316	8.5141	8.3254	8.4898	8.3436	8.5205	**8.13**
H8	4.4077	4.4833	4.5518	4.5367	4.6353	4.5337	4.6968	4.5309	4.6740	4.5395	4.6855	4.4077	4.4833	4.5518	4.5367	4.6353	4.5337	4.6968	4.5308	4.6740	4.5395	4.6855	**5.5**
H(9A-9B)	1.6636	1.7086	1.6564	1.7438	1.6990	1.7413	1.6927	1.7390	1.6918	1.7459	1.7073	1.6637	1.7086	1.6564	1.7438	1.6990	1.7413	1.6927	1.7390	1.6918	1.7459	1.7073	**2.08**
H(10-13A-10-13B)	1.1817	1.1491	1.1110	1.1331	1.0841	1.1342	1.0904	1.1354	1.0890	1.1319	1.0869	1.1817	1.1491	1.1110	1.1331	1.0841	1.1342	1.0905	1.1354	1.0890	1.1319	1.0869	**1.25**
H(14A-14C)	0.9689	0.9620	0.9403	0.9579	0.9303	0.9583	0.9335	0.9588	0.9324	0.9574	0.9334	0.9689	0.9620	0.9403	0.9579	0.9303	0.9583	0.9335	0.9588	0.9323	0.9574	0.9333	**0.79**
H(1`A-1`B)	2.9184	2.9382	2.9420	2.9510	2.9569	2.9501	2.9627	2.9494	2.9606	2.9517	2.9496	2.8300	2.7545	2.7330	2.7228	2.7192	2.7241	2.6960	2.7258	2.7013	2.7216	2.6849	**3.69**
H(2`-3`A-2`-3`B)	1.6537	1.7126	1.6853	1.7485	1.7123	1.7462	1.7330	1.7440	1.7251	1.7507	1.7633	1.6537	1.7126	1.6853	1.7485	1.7123	1.7462	1.7330	1.7440	1.7251	1.7507	1.7633	**2.18**
H(4`A)	2.8233	2.6801	2.6423	2.6326	2.6514	2.6345	2.5985	2.6367	2.6014	2.6312	2.6094	3.1207	3.1643	3.1936	3.2042	3.2636	3.2013	3.2602	3.1986	3.2549	3.2069	3.2626	**3.12**
H(4`B)	2.8365	2.8289	2.8238	2.8129	2.7870	2.8138	2.7935	2.8150	2.8012	2.8120	2.7603	2.7160	2.7121	2.6904	2.6978	2.6502	2.6990	2.6652	2.7003	2.6663	2.6965	2.6366	**3.86**

[a] methyl protons shifts are averaged

[b] according to [12]

Table S13. Cont. VI

Nuclei	R											S											Exp. [b]
	HF																						
	Gas	$CHCl_3$ (PCM)	$CHCl_3$ (SMD)	DMSO (PCM)	DMSO (SMD)	CH_3OH (PCM)	CH_3OH (SMD)	C_2H_5OH (PCM)	C_2H_5OH (SMD)	H_2O (PCM)	H_2O (SMD)	Gas	$CHCl_3$ (PCM)	$CHCl_3$ (SMD)	DMSO (PCM)	DMSO (SMD)	CH_3OH (PCM)	CH_3OH (SMD)	C_2H_5OH (PCM)	C_2H_5OH (SMD)	H_2O (PCM)	H_2O (SMD)	
**C1**	136.4035	137.1278	138.0461	138.4872	139.9277	138.4031	138.5011	138.3173	138.7464	138.5615	139.0624	136.4034	137.1278	138.0460	138.4871	139.9277	138.4032	138.5011	138.3173	138.7465	138.5615	139.0625	**131.79**
**C2**	144.3799	144.1940	144.6117	143.8110	144.2376	143.8387	144.8999	143.8673	144.7404	143.7843	144.8036	144.3800	144.1940	144.6118	143.8109	144.2376	143.8389	144.8999	143.8673	144.7404	143.7843	144.8035	**131.93**
**C3**	120.4241	120.9461	121.4129	121.2212	121.7448	121.2097	121.7134	121.1984	121.7225	121.2306	121.8327	120.4241	120.9463	121.4129	121.2212	121.7448	121.2096	121.7134	121.1984	121.7225	121.2306	121.8329	**116.53**
**C4**	171.1246	171.6259	172.1570	171.7650	172.2621	171.7599	173.0841	171.7556	172.8834	171.7699	173.1504	171.1246	171.6259	172.1571	171.7651	172.2621	171.7598	173.0841	171.7557	172.8834	171.7699	173.1503	**168.1**
**C5**	117.8580	119.0948	119.6067	119.8387	120.5249	119.7959	120.4836	119.7533	120.4426	119.8779	120.6979	117.8581	119.0948	119.6066	119.8387	120.5249	119.7961	120.4836	119.7532	120.4426	119.8779	120.6980	**116.51**
**C6**	139.8806	141.5815	142.5198	142.1624	143.6282	142.1252	144.6559	142.0906	144.3152	142.2001	144.7706	139.8807	141.5812	142.5197	142.1623	143.6282	142.1252	144.6559	142.0904	144.3152	142.2001	144.7706	**131.88**
**C7**	196.4816	201.8505	203.1192	205.7711	205.6536	205.5218	212.2035	205.2726	210.7097	206.0034	213.6470	196.4817	201.8506	203.1191	205.7712	205.6536	205.5218	212.2035	205.2726	210.7097	206.0034	213.6466	**195.2**
**C8**	55.7809	56.7123	57.1526	57.4317	58.0702	57.3775	58.5676	57.3217	58.4461	57.4800	58.9026	55.7810	56.7123	57.1526	57.4318	58.0702	57.3774	58.5676	57.3217	58.4461	57.4800	58.9025	**54.87**
**C9**	29.8996	29.5113	29.5627	29.1502	29.4549	29.1696	29.4271	29.1890	29.4288	29.1307	29.4061	29.8997	29.5113	29.5627	29.1501	29.4549	29.1696	29.4271	29.1891	29.4288	29.1307	29.4061	**31.13**
**C10**	23.2904	23.7279	24.0237	23.9132	24.5483	23.9023	24.3894	23.8917	24.3757	23.9233	24.5000	23.2903	23.7279	24.0237	23.9132	24.5483	23.9023	24.3894	23.8917	24.3757	23.9233	24.5001	**30.82**
**C11**	31.9658	31.9776	32.2737	31.8838	32.4266	31.8913	32.3529	31.8988	32.3537	31.8754	32.5134	31.9658	31.9775	32.2738	31.8838	32.4266	31.8914	32.3529	31.8988	32.3537	31.8754	32.5133	**29.11**
**C12**	33.2038	33.4266	33.7197	33.6077	34.0592	33.5944	33.9779	33.5822	33.9595	33.6206	34.0594	33.2038	33.4266	33.7196	33.6077	34.0592	33.5943	33.9779	33.5822	33.9595	33.6206	34.0594	**25.52**
**C13**	25.4897	25.6365	25.8280	25.6776	25.9818	25.6765	25.9469	25.6757	25.9433	25.6779	25.9788	25.4897	25.6364	25.8278	25.6776	25.9818	25.6766	25.9469	25.6757	25.9433	25.6779	25.9788	**22.36**
**C14**	16.3904	16.5117	16.5768	16.6005	16.7041	16.5939	16.6921	16.5881	16.6847	16.6061	16.7259	16.3904	16.5117	16.5768	16.6004	16.7041	16.5939	16.6921	16.5882	16.6848	16.6061	16.7259	**13.85**
**C1`**	44.7187	45.4339	45.6746	45.8521	46.3765	45.8201	46.3408	45.7884	46.2817	45.8817	46.6971	48.5589	49.3425	49.6846	49.8323	50.2795	49.8039	50.3316	49.7751	50.2617	49.8579	50.5880	**51.23**
**C2`**	22.8192	23.0499	23.3350	23.1165	23.5978	23.1132	23.5619	23.1098	23.5667	23.1188	23.3700	22.2374	22.3215	22.6524	22.3929	22.9300	22.3879	22.7818	22.3839	22.7996	22.3972	22.5805	**23.85**
**C3`**	22.2374	22.3214	22.6525	22.3929	22.9300	22.3879	22.7818	22.3838	22.7996	22.3972	22.5804	22.8192	23.0498	23.3350	23.1164	23.5978	23.1130	23.5619	23.1098	23.5667	23.1188	23.3700	**23.66**
**C4`**	48.5589	49.3424	49.6846	49.8323	50.2795	49.8038	50.3316	49.7751	50.2617	49.8579	50.5880	44.7187	45.4340	45.6745	45.8522	46.3765	45.8201	46.3408	45.7884	46.2817	45.8817	46.6972	**52.72**
**H2**	9.0701	8.9542	8.9762	8.7692	8.8084	8.7812	8.8743	8.7933	8.8631	8.7584	8.8267	9.0701	8.9542	8.9762	8.7692	8.8084	8.7812	8.8743	8.7933	8.8631	8.7584	8.8267	**8.13**
**H3**	7.4300	7.5696	7.6605	7.6188	7.7384	7.6167	7.7471	7.6143	7.7402	7.6208	7.7645	7.4300	7.5696	7.6605	7.6188	7.7384	7.6167	7.7471	7.6143	7.7402	7.6208	7.7645	**7.22**
**H5**	7.2460	7.4415	7.5333	7.5296	7.6528	7.5244	7.6636	7.5188	7.6531	7.5346	7.6900	7.2460	7.4415	7.5333	7.5296	7.6528	7.5244	7.6636	7.5188	7.6531	7.5346	7.6900	**7.22**
**H6**	8.0736	8.2925	8.4070	8.4017	8.5655	8.3964	8.6403	8.3911	8.6097	8.4070	8.6617	8.0736	8.2925	8.4070	8.4017	8.5655	8.3964	8.6403	8.3910	8.6097	8.4070	8.6617	**8.13**
**H8**	3.6324	3.7491	3.8285	3.8185	3.9415	3.8142	4.0156	3.8099	3.9891	3.8230	4.0086	3.6324	3.7491	3.8285	3.8186	3.9415	3.8142	4.0156	3.8099	3.9891	3.8230	4.0086	**5.5**
**H(9A-9B)**	1.3075	1.3449	1.3263	1.3155	1.3343	1.3164	1.3526	1.3171	1.3440	1.3149	1.3607	1.3075	1.3449	1.3263	1.3155	1.3343	1.3164	1.3526	1.3171	1.3440	1.3149	1.3607	**2.08**
**H(10-13A-10-13B)**	1.0044	0.9654	0.9376	0.9471	0.8968	0.9479	0.9043	0.9488	0.9031	0.9464	0.9008	1.0043	0.9654	0.9376	0.9471	0.8968	0.9479	0.9043	0.9488	0.9031	0.9464	0.9008	**1.25**
**H(14A-14C)**	0.9312	0.9202	0.8994	0.9193	0.8867	0.9193	0.8883	0.9192	0.8880	0.9193	0.8891	0.9312	0.9202	0.8994	0.9193	0.8867	0.9193	0.8883	0.9192	0.8880	0.9193	0.8891	**0.79**
**H(1`A-1`B)**	2.5533	2.5833	2.5875	4.0122	2.5938	2.5907	2.6105	2.5897	2.6069	2.5924	2.6046	2.4515	2.3988	2.3673	3.7878	2.3735	2.3901	2.3554	2.3902	2.3591	2.3894	2.3503	**3.69**
**H(2`-3`A-2`-3`B)**	1.4318	1.4977	1.4621	1.5540	1.4979	1.5504	1.5153	1.5467	1.5086	1.5574	1.5412	1.4318	1.4977	1.4621	1.5540	1.4979	1.5504	1.5153	1.5467	1.5086	1.5574	1.5412	**2.18**
**H(4`A)**	2.3740	2.2775	2.2423	3.7647	2.2922	2.2831	2.2448	2.2815	2.2477	2.2860	2.2620	2.7264	2.7907	2.8219	4.3261	2.8852	2.8224	2.8920	2.8198	2.8852	2.8273	2.8991	**3.12**
**H(4`B)**	2.5289	2.5200	2.4923	3.8108	2.4548	2.4972	2.4661	2.4990	2.4706	2.4928	2.4386	2.3801	2.3758	2.3530	3.6983	2.3024	2.3591	2.3291	2.3597	2.3287	2.3574	2.3100	**3.86**
Nuclei	LC-BLYP																						
**C1**	144.3337	145.2525	146.1785	145.6373	148.5197	145.5838	147.4982	145.5603	147.5544	145.6745	147.9878	144.3338	145.2525	146.1784	145.6371	148.5198	145.5837	147.4981	145.5604	147.5545	145.6747	147.9879	**131.79**
**C2**	148.6496	148.4843	149.0546	148.3427	148.7114	148.3324	149.2174	148.3487	149.1040	148.3349	149.1780	148.6495	148.4842	149.0545	148.3428	148.7113	148.3324	149.2174	148.3486	149.1039	148.3350	149.1780	**131.93**
**C3**	128.2690	128.8816	129.5093	129.1027	129.9143	129.0701	130.1131	129.0648	130.0465	129.1164	130.2165	128.2689	128.8817	129.5093	129.1027	129.9141	129.0702	130.1130	129.0649	130.0462	129.1163	130.2166	**116.53**
**C4**	181.9798	182.4933	183.1107	182.8576	183.2651	182.8151	183.9702	182.7994	183.8042	182.8803	184.0980	181.9798	182.4933	183.1108	182.8578	183.2648	182.8149	183.9704	182.7994	183.8041	182.8802	184.0981	**168.1**
**C5**	126.1569	127.4600	128.1494	128.1739	129.0986	128.1091	129.2707	128.0714	129.1631	128.2181	129.4931	126.1568	127.4600	128.1495	128.1741	129.0987	128.1090	129.2708	128.0716	129.1631	128.2182	129.4930	**116.51**
**C6**	144.2894	146.1433	147.2813	147.3141	148.4182	147.2206	149.0102	147.1539	148.7911	147.3847	149.2672	144.2894	146.1434	147.2812	147.3142	148.4182	147.2207	149.0102	147.1540	148.7910	147.3848	149.2671	**131.88**
**C7**	211.8278	217.1487	218.4570	220.0985	221.7729	219.8831	227.5238	219.6985	226.1955	220.2906	228.9320	211.8278	217.1488	218.4569	220.0982	221.7726	219.8829	227.5238	219.6986	226.1955	220.2905	228.9321	**195.2**
**C8**	62.4909	63.6231	64.2597	64.2397	65.3892	64.1774	65.9514	64.1437	65.7846	64.2835	66.3958	62.4909	63.6233	64.2597	64.2397	65.3891	64.1773	65.9515	64.1438	65.7846	64.2834	66.3959	**54.87**
**C9**	33.0680	32.6961	32.9683	32.5839	32.7305	32.5691	32.6887	32.5814	32.6630	32.5807	32.7201	33.0680	32.6960	32.9684	32.5840	32.7304	32.5690	32.6887	32.5813	32.6630	32.5807	32.7202	**31.13**
**C10**	25.0507	25.5080	25.7804	25.7971	26.3215	25.7569	26.1723	25.7442	26.1362	25.8182	26.3359	25.0508	25.5079	25.7804	25.7972	26.3215	25.7569	26.1723	25.7442	26.1361	25.8182	26.3359	**30.82**
**C11**	34.5220	34.4735	34.9320	34.4506	34.9453	34.4311	34.9186	34.4392	34.8721	34.4519	35.0881	34.5220	34.4734	34.9321	34.4506	34.9454	34.4312	34.9185	34.4391	34.8721	34.4519	35.0881	**29.11**
**C12**	36.2808	36.5507	36.8178	36.7768	37.2355	36.7408	37.1366	36.7323	37.1251	36.7934	37.2658	36.2808	36.5507	36.8178	36.7768	37.2356	36.7409	37.1365	36.7323	37.1252	36.7934	37.2659	**25.52**
**C13**	26.2331	26.3735	26.6274	26.4658	26.7372	26.4395	26.7270	26.4405	26.7013	26.4731	26.7419	26.2331	26.3736	26.6275	26.4656	26.7372	26.4395	26.7270	26.4405	26.7012	26.4731	26.7418	**22.36**
**C14**	15.4616	15.5941	15.6570	15.7249	15.7857	15.6951	15.7837	15.6926	15.7615	15.7359	15.8362	15.4616	15.5940	15.6571	15.7249	15.7858	15.6951	15.7838	15.6927	15.7614	15.7360	15.8362	**13.85**
**C1`**	48.9243	49.7454	50.0229	50.2601	50.8108	50.2054	50.7778	50.1787	50.6826	50.2953	51.1597	52.7027	53.6594	54.0269	54.2002	54.7329	54.1469	54.8610	54.1209	54.7844	54.2335	55.1771	**51.23**
**C2`**	22.9721	23.2680	23.5662	23.4320	23.8331	23.4010	23.8693	23.3975	23.8636	23.4443	23.7302	22.4919	22.5698	22.9001	22.6625	23.1303	22.6356	23.0545	22.6358	23.0430	22.6706	22.9267	**23.85**
**C3`**	22.4919	22.5699	22.9002	22.6624	23.1302	22.6355	23.0545	22.6359	23.0430	22.6706	22.9267	22.9721	23.2680	23.5662	23.4319	23.8329	23.4010	23.8692	23.3975	23.8636	23.4442	23.7302	**23.66**
**C4`**	52.7027	53.6594	54.0269	54.2003	54.7329	54.1469	54.8611	54.1209	54.7844	54.2335	55.1771	48.9243	49.7454	50.0229	50.2601	50.8108	50.2054	50.7778	50.1787	50.6826	50.2954	51.1597	**52.72**
**H2**	9.0123	8.8795	8.9082	8.8108	8.6896	8.8153	8.7346	8.8210	8.7351	8.8051	8.7059	9.0123	8.8795	8.9082	8.8108	8.6896	8.8153	8.7346	8.8210	8.7351	8.8051	8.7059	**8.13**
**H3**	7.5402	7.6698	7.7636	7.7115	7.8303	7.7087	7.8492	7.7074	7.8413	7.7132	7.8687	7.5402	7.6699	7.7636	7.7115	7.8303	7.7087	7.8492	7.7074	7.8413	7.7132	7.8687	**7.22**
**H5**	7.3881	7.5726	7.6648	7.6567	7.7794	7.6510	7.7928	7.6468	7.7828	7.6611	7.8180	7.3881	7.5726	7.6648	7.6567	7.7794	7.6510	7.7928	7.6468	7.7828	7.6611	7.8180	**7.22**
**H6**	8.1915	8.3747	8.4717	8.4829	8.5778	8.4756	8.6442	8.4699	8.6229	8.4886	8.6675	8.1915	8.3747	8.4717	8.4829	8.5778	8.4757	8.6442	8.4699	8.6229	8.4886	8.6675	**8.13**
**H8**	4.2052	4.2789	4.3291	4.3443	4.4160	4.3392	4.5006	4.3357	4.4719	4.3480	4.5224	4.2052	4.2789	4.3291	4.3443	4.4160	4.3392	4.5006	4.3357	4.4719	4.3480	4.5224	**5.5**
**H(9A-9B)**	1.6608	1.6955	1.6938	1.7274	1.6824	1.7244	1.6683	1.7229	1.6685	1.7292	1.6815	1.6608	1.6955	1.6938	1.7274	1.6823	1.7243	1.6683	1.7229	1.6685	1.7292	1.6816	**2.08**
**H(10-13A-10-13B)**	1.1538	1.1143	1.0835	1.0982	1.0377	1.0985	1.0459	1.1003	1.0447	1.0969	1.0437	1.1538	1.1143	1.0835	1.0982	1.0377	1.0985	1.0459	1.1003	1.0447	1.0969	1.0437	**1.25**
**H(14A-14C)**	0.9733	0.9623	0.9406	0.9583	0.9256	0.9578	0.9296	0.9588	0.9286	0.9579	0.9313	0.9733	0.9623	0.9406	0.9583	0.9257	0.9578	0.9296	0.9588	0.9286	0.9579	0.9313	**0.79**
**H(1`A-1`B)**	2.8505	2.8672	2.8690	2.8796	2.8744	2.8780	2.8778	2.8779	2.8757	2.8801	2.8725	2.7669	2.6916	2.6662	2.6607	2.6645	2.6611	2.6373	2.6632	2.6427	2.6595	2.6263	**3.69**
**H(2`-3`A-2`-3`B)**	1.6417	1.7002	1.6646	1.7392	1.6998	1.7357	1.7212	1.7339	1.7125	1.7414	1.7515	1.6417	1.7003	1.6645	1.7392	1.6998	1.7357	1.7212	1.7339	1.7125	1.7415	1.7515	**2.18**
**H(4`A)**	2.7349	2.5890	2.5522	2.5471	2.5772	2.5473	2.5179	2.5493	2.5210	2.5462	2.5245	3.0212	3.0617	3.0878	3.0996	3.1453	3.0961	3.1358	3.0942	3.1312	3.1019	3.1452	**3.12**
**H(4`B)**	2.7989	2.7942	2.7803	2.7743	2.7517	2.7749	2.7567	2.7771	2.7644	2.7728	2.7282	2.6798	2.6728	2.6503	2.6595	2.6035	2.6597	2.6199	2.6615	2.6201	2.6583	2.5998	**3.86**

[a] methyl protons shifts are averaged
[b] according to [12]

Table S13. Cont. VII

Nuclei	R											S											Exp. [b]
	M06																						
	Gas	$CHCl_3$ (PCM)	$CHCl_3$ (SMD)	DMSO (PCM)	DMSO (SMD)	CH_3OH (PCM)	CH_3OH (SMD)	C_2H_5OH (PCM)	C_2H_5OH (SMD)	H_2O (PCM)	H_2O (SMD)	Gas	$CHCl_3$ (PCM)	$CHCl_3$ (SMD)	DMSO (PCM)	DMSO (SMD)	CH_3OH (PCM)	CH_3OH (SMD)	C_2H_5OH (PCM)	C_2H_5OH (SMD)	H_2O (PCM)	H_2O (SMD)	
**C1**	136.7395	137.4383	139.1872	139.7500	142.0626	139.7117	141.0007	139.6815	141.1315	139.8011	141.0203	136.7395	137.4383	139.1873	139.7500	142.0627	139.7119	141.0007	139.6817	141.1315	139.8011	141.0202	**131.79**
**C2**	140.3362	140.1738	140.8382	140.5152	141.1710	140.5096	141.5886	140.5117	141.4805	140.5343	141.5920	140.3362	140.1738	140.8382	140.5152	141.1710	140.5097	141.5887	140.5116	141.4805	140.5343	141.5918	**131.93**
**C3**	121.7301	122.5264	123.1854	122.5950	123.4710	122.5721	123.7068	122.5568	123.6508	122.6311	123.6545	121.7301	122.5264	123.1855	122.5950	123.4710	122.5720	123.7068	122.5565	123.6508	122.6311	123.6544	**116.53**
**C4**	173.6051	174.3515	174.9011	173.7989	174.4242	173.7727	175.0521	173.7545	174.8896	173.8386	175.3265	173.6051	174.3515	174.9010	173.7989	174.4241	173.7727	175.0521	173.7543	174.8896	173.8386	175.3266	**168.1**
**C5**	119.8470	121.1310	121.8720	121.8447	122.7079	121.7943	122.9654	121.7514	122.8696	121.9074	122.8272	119.8470	121.1310	121.8721	121.8447	122.7078	121.7943	122.9653	121.7514	122.8696	121.9074	122.8273	**116.51**
**C6**	136.5055	138.2404	139.2303	138.7073	140.0987	138.6322	140.7600	138.5651	140.5297	138.7932	141.3227	136.5055	138.2404	139.2303	138.7073	140.0987	138.6322	140.7600	138.5651	140.5297	138.7932	141.3226	**131.88**
**C7**	204.4927	209.2057	210.9794	212.9963	213.9539	212.8363	218.6410	212.6850	217.5696	213.1640	219.3307	204.4927	209.2057	210.9794	212.9963	213.9539	212.8362	218.6410	212.6849	217.5696	213.1640	219.3304	**195.2**
**C8**	62.3082	63.3776	64.7624	64.9615	66.0302	64.9170	66.3705	64.8807	66.2609	65.0187	66.5588	62.3082	63.3776	64.7625	64.9615	66.0303	64.9170	66.3706	64.8806	66.2609	65.0187	66.5588	**54.87**
**C9**	33.9746	33.6588	33.7535	33.1060	33.3277	33.1061	33.4541	33.1140	33.4109	33.1206	33.4528	33.9746	33.6588	33.7536	33.1060	33.3276	33.1061	33.4540	33.1140	33.4109	33.1206	33.4527	**31.13**
**C10**	24.2953	24.9543	25.5095	25.6106	26.5097	25.5760	26.4851	25.5488	26.4175	25.6577	26.7669	24.2953	24.9543	25.5095	25.6106	26.5096	25.5759	26.4851	25.5487	26.4175	25.6577	26.7668	**30.82**
**C11**	34.8467	34.7314	35.1637	34.5408	35.2789	34.5317	35.1177	34.5306	35.1156	34.5642	35.3094	34.8467	34.7314	35.1638	34.5408	35.2789	34.5318	35.1177	34.5306	35.1156	34.5642	35.3093	**29.11**
**C12**	36.4097	36.7323	37.0917	36.9663	37.5993	36.9423	37.5640	36.9262	37.5307	37.0036	37.6381	36.4097	36.7323	37.0917	36.9663	37.5993	36.9423	37.5641	36.9263	37.5307	37.0036	37.6382	**25.52**
**C13**	26.8520	26.9758	27.2556	26.9736	27.4054	26.9616	27.3243	26.9574	27.3210	26.9996	27.3574	26.8520	26.9758	27.2557	26.9736	27.4053	26.9616	27.3243	26.9574	27.3210	26.9996	27.3574	**22.36**
**C14**	15.7773	15.9410	16.1122	16.1076	16.2987	16.0880	16.2939	16.0763	16.3185	16.1411	16.3007	15.7773	15.9410	16.1122	16.1076	16.2986	16.0879	16.2939	16.0763	16.3185	16.1411	16.3008	**13.85**
**C1`**	50.6358	51.4709	52.0016	52.1078	52.8262	52.0713	52.6260	52.0428	52.6031	52.1574	52.7996	55.3156	56.3435	56.8201	56.9296	57.5812	56.8855	57.7505	56.8490	57.6713	56.9853	57.8463	**51.23**
**C2`**	23.4130	23.8827	24.3043	23.9912	24.5395	23.9728	24.4447	23.9622	24.4547	24.0233	24.4894	22.3000	22.9204	23.4016	23.1480	23.9340	23.1231	23.6949	23.1060	23.7548	23.1863	22.8523	**23.85**
**C3`**	22.3000	22.9204	23.4015	23.1480	23.9341	23.1231	23.6948	23.1060	23.7547	23.1863	22.8522	23.4130	23.8827	24.3043	23.9912	24.5395	23.9729	24.4446	23.9623	24.4547	24.0233	24.4894	**23.66**
**C4`**	55.3156	56.3435	56.8201	56.9296	57.5812	56.8856	57.7505	56.8491	57.6713	56.9853	57.8463	50.6358	51.4709	52.0015	52.1078	52.8263	52.0713	52.6260	52.0428	52.6030	52.1574	52.7997	**52.72**
**H2**	8.7458	8.6063	8.5389	8.3820	8.3631	8.3864	8.4368	8.3913	8.4245	8.3783	8.4346	8.7458	8.6063	8.5389	8.3820	8.3631	8.3864	8.4368	8.3913	8.4245	8.3783	8.4346	**8.13**
**H3**	7.3056	7.4123	7.4701	7.4308	7.5674	7.4280	7.6021	7.4258	7.5895	7.4339	7.5909	7.3056	7.4123	7.4701	7.4308	7.5674	7.4280	7.6021	7.4258	7.5895	7.4339	7.5909	**7.22**
**H5**	7.1471	7.3475	7.4562	7.4147	7.5406	7.4104	7.5526	7.4067	7.5431	7.4193	7.5372	7.1471	7.3475	7.4562	7.4147	7.5406	7.4104	7.5526	7.4067	7.5431	7.4193	7.5372	**7.22**
**H6**	8.0143	8.2403	8.3301	8.2337	8.3337	8.2291	8.3510	8.2251	8.3316	8.2385	8.4309	8.0143	8.2403	8.3301	8.2337	8.3337	8.2291	8.3510	8.2251	8.3316	8.2385	8.4309	**8.13**
**H8**	4.3873	4.4404	4.5343	4.5061	4.5536	4.5030	4.6173	4.5003	4.5881	4.5097	4.7136	4.3873	4.4404	4.5343	4.5061	4.5536	4.5030	4.6173	4.5003	4.5881	4.5097	4.7136	**5.5**
**H(9A-9B)**	1.8167	1.8196	1.7577	1.7271	1.7001	1.7266	1.6981	1.7266	1.6906	1.7282	1.7743	1.8167	1.8196	1.7577	1.7271	1.7001	1.7266	1.6981	1.7266	1.6906	1.7282	1.7742	**2.08**
**H(10-13A-10-13B)**	1.1913	1.1614	1.1081	1.1263	1.0823	1.1272	1.0890	1.1286	1.0867	1.1261	1.0894	1.1913	1.1614	1.1081	1.1263	1.0823	1.1272	1.0890	1.1286	1.0867	1.1261	1.0894	**1.25**
**H(14A-14C)**	0.8935	0.8845	0.8652	0.8810	0.8587	0.8809	0.8644	0.8814	0.8656	0.8816	0.8702	0.8935	0.8845	0.8652	0.8810	0.8587	0.8809	0.8644	0.8814	0.8656	0.8816	0.8702	**0.79**
**H(1`A-1`B)**	2.8882	2.8996	2.9026	2.8993	2.9034	2.8988	2.9098	2.8988	2.9053	2.9004	2.9533	2.8170	2.7641	2.7480	2.7688	2.7577	2.7691	2.7782	2.7700	2.7759	2.7691	2.7366	**3.69**
**H(2`-3`A-2`-3`B)**	1.6076	1.6738	1.6582	1.7262	1.6878	1.7240	1.7103	1.7223	1.7039	1.7289	1.7412	1.1913	1.1614	1.1081	1.1263	1.0823	1.1272	1.0890	1.1286	1.0867	1.1261	1.0894	**2.18**
**H(4`A)**	2.7136	2.6299	2.6049	2.6448	2.6561	2.6440	2.6651	2.6439	2.6588	2.6463	2.6526	3.0863	3.1159	3.1465	3.1458	3.1906	3.1441	3.1947	3.1429	3.1865	3.1480	3.2379	**3.12**
**H(4`B)**	2.9205	2.8983	2.8911	2.8927	2.8594	2.8941	2.8913	2.8962	2.8930	2.8918	2.8205	2.6901	2.6832	2.6587	2.6528	2.6163	2.6535	2.6250	2.6548	2.6241	2.6528	2.6687	**3.86**
Nuclei	**M06-2X**																						
**C1**	153.4197	154.5722	155.4853	154.9225	157.4968	154.9186	156.4566	154.9168	156.4545	154.9205	156.3853	153.4197	154.5722	155.4853	154.9225	157.4968	154.9186	156.4566	154.9168	156.4546	154.9205	156.3853	**131.79**
**C2**	155.1694	155.0797	155.7659	154.9067	155.5252	154.9406	155.9982	154.9758	155.8808	154.8674	156.0327	155.1694	155.0797	155.7659	154.9066	155.5252	154.9406	155.9982	154.9757	155.8808	154.8674	156.0327	**131.93**
**C3**	136.0211	136.8448	137.4934	137.0851	137.8625	137.0947	138.1302	137.1054	138.0647	137.0689	138.2095	136.0211	136.8448	137.4934	137.0851	137.8625	137.0947	138.1302	137.1052	138.0647	137.0689	138.2095	**116.53**
**C4**	187.1175	187.8325	188.4919	188.2201	188.7848	188.2181	189.4744	188.2173	189.3234	188.2154	189.6913	187.1175	187.8325	188.4919	188.2201	188.7848	188.2181	189.4744	188.2173	189.3234	188.2154	189.6913	**168.1**
**C5**	133.6444	135.1733	135.8765	135.9193	136.8070	135.8956	137.0657	135.8728	136.9525	135.9355	137.2247	133.6444	135.1733	135.8765	135.9193	136.8070	135.8956	137.0657	135.8728	136.9525	135.9355	137.2247	**116.51**
**C6**	151.2125	153.0661	154.2394	154.2054	155.7455	154.1545	156.3093	154.1051	156.0950	154.2477	157.0478	151.2125	153.0661	154.2394	154.2055	155.7455	154.1545	156.3093	154.1050	156.0949	154.2477	157.0478	**131.88**
**C7**	219.4178	225.2157	226.5468	228.1930	229.5552	228.0237	235.7021	227.8570	234.2369	228.3492	236.4504	219.4178	225.2157	226.5468	228.1930	229.5552	228.0237	235.7021	227.8571	234.2374	228.3492	236.4504	**195.2**
**C8**	67.2872	68.8639	69.5712	69.5554	70.9043	69.5317	71.5188	69.5099	71.2851	69.5720	71.7960	67.2872	68.8639	69.5712	69.5553	70.9043	69.5317	71.5188	69.5100	71.2850	69.5720	71.7960	**54.87**
**C9**	35.5238	35.2768	35.4231	35.1682	35.3427	35.1937	35.5447	35.2207	35.4242	35.1379	35.8854	35.5238	35.2768	35.4230	35.1681	35.3427	35.1937	35.5447	35.2207	35.4242	35.1379	35.8854	**31.13**
**C10**	26.3737	26.8258	27.0914	27.0936	27.6873	27.0961	27.5154	27.1001	27.4827	27.0853	27.7535	26.3737	26.8258	27.0913	27.0935	27.6873	27.0961	27.5154	27.1000	27.4828	27.0853	27.7535	**30.82**
**C11**	35.8549	35.8468	36.2837	35.7699	36.3578	35.7950	36.2981	35.8216	36.2898	35.7396	36.3506	35.8549	35.8468	36.2837	35.7699	36.3578	35.7950	36.2981	35.8216	36.2899	35.7396	36.3506	**29.11**
**C12**	37.5717	37.8909	38.1630	38.0950	38.6110	38.1039	38.4767	38.1137	38.4829	38.0803	38.5091	37.5717	37.8909	38.1630	38.0950	38.6110	38.1039	38.4767	38.1138	38.4829	38.0803	38.5091	**25.52**
**C13**	27.4100	27.5697	27.8054	27.6084	27.9427	27.6278	27.9092	27.6482	27.9205	27.5834	27.8205	27.4100	27.5697	27.8054	27.6084	27.9427	27.6278	27.9092	27.6482	27.9205	27.5834	27.8205	**22.36**
**C14**	15.9414	15.9867	15.9035	16.0207	16.0039	16.0384	15.9866	16.0576	15.9631	15.9979	16.2068	15.9414	15.9867	15.9035	16.0208	16.0039	16.0384	15.9866	16.0576	15.9631	15.9979	16.2068	**13.85**
**C1`**	52.9110	53.4208	53.6521	53.8296	54.4744	53.8210	54.4450	53.8141	54.3482	53.8323	54.9206	57.0977	58.1237	58.4890	58.5758	59.1546	58.5706	59.2285	58.5666	59.2046	58.5741	59.4819	**51.23**
**C2`**	24.5745	25.1092	25.3996	25.3261	25.7983	25.3339	25.7856	25.3429	25.7843	25.3126	25.5741	24.0916	24.2808	24.4796	24.3674	24.7211	24.3830	24.7199	24.3998	24.6828	24.3464	24.8957	**23.85**
**C3`**	24.0916	24.2808	24.4796	24.3674	24.7211	24.3830	24.7199	24.3997	24.6828	24.3464	24.8957	24.5745	25.1092	25.3996	25.3262	25.7983	25.3339	25.7856	25.3429	25.7843	25.3126	25.5741	**23.66**
**C4`**	57.0977	58.1237	58.4890	58.5757	59.1546	58.5706	59.2285	58.5666	59.2045	58.5741	59.4819	52.9110	53.4208	53.6521	53.8296	54.4744	53.8210	54.4450	53.8141	54.3483	53.8323	54.9206	**52.72**
**H2**	9.2660	9.1556	9.1885	9.1027	9.0318	9.1052	9.0789	9.1073	9.0814	9.1003	9.1208	9.2660	9.1556	9.1885	9.1027	9.0318	9.1052	9.0789	9.1073	9.0814	9.1003	9.1208	**8.13**
**H3**	7.8380	7.9580	8.0589	7.9934	8.0884	7.9907	8.1092	7.9876	8.1015	7.9961	8.1339	7.8380	7.9580	8.0589	7.9934	8.0884	7.9907	8.1092	7.9876	8.1015	7.9961	8.1339	**7.22**
**H5**	7.7366	7.9260	8.0189	8.0118	8.1296	8.0057	8.1461	7.9993	8.1371	8.0177	8.1506	7.7366	7.9260	8.0189	8.0118	8.1296	8.0057	8.1461	7.9993	8.1371	8.0177	8.1506	**7.22**
**H6**	8.5673	8.7324	8.8270	8.8445	8.9783	8.8363	9.0422	8.8277	9.0272	8.8524	9.0693	8.5673	8.7324	8.8270	8.8445	8.9783	8.8363	9.0422	8.8277	9.0272	8.8524	9.0693	**8.13**
**H8**	4.3040	4.3390	4.3774	4.3995	4.4501	4.3943	4.5274	4.3888	4.5096	4.4046	4.5325	4.3040	4.3390	4.3774	4.3995	4.4501	4.3943	4.5274	4.3888	4.5096	4.4046	4.5325	**5.5**
**H(9A-9B)**	1.7119	1.7083	1.6976	1.7305	1.7062	1.7272	1.7029	1.7236	1.6962	1.7339	1.7581	1.7119	1.7083	1.6976	1.7305	1.7062	1.7272	1.7029	1.7236	1.6962	1.7339	1.7581	**2.08**
**H(10-13A-10-13B)**	1.1135	1.0623	1.0350	1.0449	0.9906	1.0448	0.9954	1.0443	0.9967	1.0452	0.9881	1.1135	1.0623	1.0350	1.0449	0.9906	1.0448	0.9954	1.0443	0.9967	1.0452	0.9881	**1.25**
**H(14A-14C)**	0.9533	0.9288	0.8953	0.9192	0.8718	0.9185	0.8776	0.9174	0.8729	0.9201	0.8998	0.9533	0.9288	0.8953	0.9192	0.8718	0.9185	0.8776	0.9174	0.8729	0.9201	0.8998	**0.79**
**H(1`A-1`B)**	2.8296	2.8403	2.8435	2.8546	2.8541	2.8523	2.8612	2.8498	2.8580	2.8568	2.8528	2.7731	2.7297	2.7053	2.7016	2.6920	2.7020	2.6738	2.7020	2.6736	2.7015	2.6611	**3.69**
**H(2`-3`A-2`-3`B)**	1.6435	1.6988	1.6685	1.7408	1.7065	1.7368	1.7283	1.7324	1.7212	1.7449	1.7398	1.6435	1.6988	1.6685	1.7408	1.7065	1.7368	1.7283	1.7324	1.7212	1.7449	1.7398	**2.18**
**H(4`A)**	2.7447	2.6319	2.5902	2.5916	2.5962	2.5921	2.5511	2.5924	2.5457	2.5914	2.5591	3.0548	3.0671	3.0871	3.0996	3.1423	3.0959	3.1364	3.0920	3.1313	3.1034	3.1615	**3.12**
**H(4`B)**	2.8014	2.8274	2.8205	2.8117	2.7878	2.8119	2.7965	2.8117	2.8015	2.8117	2.7632	2.6044	2.6135	2.6000	2.6094	2.5658	2.6086	2.5859	2.6075	2.5847	2.6103	2.5442	**3.86**

[a] methyl protons shifts are averaged

[b] according to [12]

Table S13. Cont. VIII

Nuclei	R											S											**Exp.** [b]
	**mpW1LYP**																						
	Gas	$CHCl_3$ (PCM)	$CHCl_3$ (SMD)	DMSO (PCM)	DMSO (SMD)	CH_3OH (PCM)	CH_3OH (SMD)	C_2H_5OH (PCM)	C_2H_5OH (SMD)	H_2O (PCM)	H_2O (SMD)	Gas	$CHCl_3$ (PCM)	$CHCl_3$ (SMD)	DMSO (PCM)	DMSO (SMD)	CH_3OH (PCM)	CH_3OH (SMD)	C_2H_5OH (PCM)	C_2H_5OH (SMD)	H_2O (PCM)	H_2O (SMD)	
**C1**	139.9564	140.7835	142.2031	141.1581	143.4884	141.1218	142.5504	141.0900	142.6807	141.1946	143.0877	139.9564	140.7835	142.2031	141.1581	143.4884	141.1218	142.5502	141.0900	142.6808	141.1946	143.0877	**131.79**
**C2**	140.0869	140.1128	140.5989	139.9647	140.3317	139.9757	140.8095	139.9871	140.7017	139.9513	140.6988	140.0869	140.1128	140.5989	139.9647	140.3317	139.9757	140.8098	139.9871	140.7018	139.9513	140.6990	**131.93**
**C3**	122.1020	122.7540	123.3140	122.9669	123.5408	122.9548	123.8126	122.9436	123.7587	122.9758	123.8302	122.1021	122.7540	123.3140	122.9669	123.5408	122.9548	123.8129	122.9436	123.7587	122.9758	123.8302	**116.53**
**C4**	176.3414	177.1242	177.7564	177.5545	178.1089	177.5267	178.7385	177.5003	178.5908	177.5782	178.8000	176.3414	177.1242	177.7564	177.5545	178.1089	177.5267	178.7387	177.5003	178.5908	177.5782	178.8001	**168.1**
**C5**	119.8476	121.0787	121.6784	121.7389	122.5018	121.6958	122.7484	121.6543	122.6555	121.7781	122.9175	119.8476	121.0787	121.6784	121.7389	122.5018	121.6958	122.7481	121.6543	122.6556	121.7781	122.9175	**116.51**
**C6**	135.9743	137.8039	138.6879	138.9772	140.1969	138.9012	140.9518	138.8263	140.6930	139.0473	141.1671	135.9742	137.8039	138.6879	138.9772	140.1969	138.9012	140.9516	138.8263	140.6930	139.0473	141.1669	**131.88**
**C7**	203.3962	208.1939	210.0966	210.8527	212.3329	210.6699	217.3761	210.4927	216.2790	211.0298	218.8398	203.3962	208.1939	210.0966	210.8527	212.3329	210.6699	217.3759	210.4927	216.2790	211.0298	218.8394	**195.2**
**C8**	65.8383	66.9702	67.7779	67.5352	68.5552	67.4905	69.1310	67.4494	69.0094	67.5781	69.4798	65.8383	66.9702	67.7779	67.5352	68.5552	67.4905	69.1311	67.4494	69.0093	67.5781	69.4798	**54.87**
**C9**	36.8987	36.5656	36.5674	36.4418	36.5378	36.4484	36.6783	36.4565	36.6313	36.4332	36.6677	36.8987	36.5656	36.5674	36.4418	36.5377	36.4484	36.6784	36.4565	36.6313	36.4332	36.6677	**31.13**
**C10**	27.7123	28.2105	28.5088	28.4945	29.0481	28.4739	28.8709	28.4550	28.8715	28.5120	28.9354	27.7123	28.2105	28.5088	28.4945	29.0481	28.4739	28.8709	28.4550	28.8715	28.5120	28.9354	**30.82**
**C11**	37.4401	37.5086	37.8603	37.4671	37.9805	37.4706	37.9434	37.4751	37.9393	37.4614	38.1000	37.4401	37.5086	37.8603	37.4671	37.9805	37.4706	37.9432	37.4751	37.9392	37.4614	38.0998	**29.11**
**C12**	39.1710	39.4604	39.7735	39.6831	40.1557	39.6647	40.0461	39.6481	40.0446	39.6990	40.0900	39.1710	39.4604	39.7735	39.6831	40.1557	39.6647	40.0462	39.6481	40.0446	39.6990	40.0900	**25.52**
**C13**	30.0534	30.2462	30.4763	30.3292	30.6024	30.3225	30.5962	30.3173	30.5967	30.3334	30.5745	30.0534	30.2462	30.4763	30.3292	30.6024	30.3225	30.5963	30.3173	30.5967	30.3334	30.5745	**22.36**
**C14**	16.9568	17.1292	17.1924	17.2551	17.3117	17.2441	17.2998	17.2345	17.2979	17.2639	17.2966	16.9568	17.1292	17.1924	17.2551	17.3117	17.2441	17.2999	17.2345	17.2978	17.2639	17.2966	**13.85**
**C1`**	52.0596	52.8264	53.1517	53.3370	53.9059	53.3012	53.8501	53.2670	53.7906	53.3697	54.3351	56.5060	57.4407	57.8845	58.0141	58.4818	57.9734	58.7049	57.9346	58.6243	58.0510	59.0072	**51.23**
**C2`**	25.8538	26.3325	26.6606	26.5701	26.9727	26.5509	27.0562	26.5337	27.0426	26.5866	26.8914	25.5831	25.8965	26.2726	26.0203	26.6055	26.0119	26.4749	26.0047	26.4829	26.0259	26.2585	**23.85**
**C3`**	25.5831	25.8965	26.2726	26.0203	26.6055	26.0119	26.4748	26.0047	26.4828	26.0260	26.2585	25.8537	26.3325	26.6606	26.5701	26.9727	26.5509	27.0562	26.5337	27.0427	26.5866	26.8915	**23.66**
**C4`**	56.5060	57.4407	57.8845	58.0141	58.4818	57.9734	58.7049	57.9346	58.6243	58.0510	59.0071	52.0596	52.8264	53.1517	53.3370	53.9059	53.3012	53.8500	53.2670	53.7906	53.3697	54.3350	**52.72**
**H2**	8.7210	8.6206	8.5765	8.5556	8.4600	8.5618	8.5091	8.5673	8.5048	8.5489	8.4618	8.7210	8.6206	8.5765	8.5556	8.4600	8.5618	8.5091	8.5673	8.5048	8.5489	8.4618	**8.13**
**H3**	7.3462	7.4713	7.5539	7.5112	7.6133	7.5098	7.6306	7.5079	7.6232	7.5124	7.6404	7.3462	7.4713	7.5539	7.5112	7.6133	7.5098	7.6306	7.5079	7.6232	7.5124	7.6404	**7.22**
**H5**	7.1427	7.3226	7.4086	7.4041	7.5265	7.3998	7.5501	7.3950	7.5396	7.4082	7.5782	7.1427	7.3226	7.4086	7.4041	7.5265	7.3998	7.5501	7.3950	7.5396	7.4082	7.5782	**7.22**
**H6**	7.9614	8.1504	8.2514	8.2660	8.3906	8.2590	8.4685	8.2516	8.4484	8.2726	8.4704	7.9614	8.1504	8.2514	8.2660	8.3906	8.2590	8.4685	8.2516	8.4484	8.2726	8.4704	**8.13**
**H8**	4.5415	4.6186	4.6612	4.6667	4.7547	4.6638	4.7998	4.6606	4.7858	4.6693	4.7770	4.5415	4.6186	4.6612	4.6667	4.7547	4.6638	4.7998	4.6606	4.7858	4.6693	4.7770	**5.5**
**H(9A-9B)**	1.6545	1.7015	1.6735	1.7462	1.7185	1.7436	1.7227	1.7407	1.7170	1.7486	1.7376	1.6545	1.7015	1.6735	1.7462	1.7185	1.7436	1.7226	1.7407	1.7170	1.7486	1.7376	**2.08**
**H(10-13A-10-13B)**	1.1983	1.1681	1.1362	1.1578	1.1105	1.1588	1.1160	1.1594	1.1160	1.1569	1.1105	1.1983	1.1681	1.1362	1.1578	1.1105	1.1588	1.1160	1.1594	1.1160	1.1569	1.1105	**1.25**
**H(14A-14C)**	0.9688	0.9627	0.9437	0.9618	0.9332	0.9621	0.9357	0.9622	0.9356	0.9615	0.9331	0.9688	0.9627	0.9437	0.9618	0.9332	0.9621	0.9357	0.9622	0.9356	0.9615	0.9331	**0.79**
**H(1`A-1`B)**	2.9585	2.9773	2.9862	2.9942	3.0008	2.9932	3.0082	2.9920	3.0074	2.9951	2.9905	2.8716	2.7915	2.7698	2.7609	2.7570	2.7625	2.7361	2.7640	2.7391	2.7594	2.7217	**3.69**
**H(2`-3`A-2`-3`B)**	1.6621	1.7194	1.6938	1.7579	1.7212	1.7555	1.7413	1.7528	1.7359	1.7602	1.7702	1.6621	1.7194	1.6938	1.7579	1.7212	1.7555	1.7413	1.7528	1.7359	1.7602	1.7702	**2.18**
**H(4`A)**	2.8781	2.7280	2.6936	2.6866	2.7037	2.6885	2.6534	2.6903	2.6549	2.6849	2.6624	3.1784	3.2186	3.2572	3.2649	3.3267	3.2618	3.3253	3.2585	3.3219	3.2677	3.3221	**3.12**
**H(4`B)**	2.8652	2.8550	2.8460	2.8352	2.8103	2.8365	2.8187	2.8376	2.8233	2.8339	2.7810	2.7387	2.7359	2.7151	2.7236	2.6748	2.7247	2.6912	2.7255	2.6930	2.7225	2.6590	**3.86**
Nuclei	**mpW1PW91**																						
**C1**	137.0159	137.8241	139.1885	138.1931	140.4564	138.1609	139.7679	138.1367	139.8251	138.2259	140.3928	137.0160	137.8241	139.1885	138.1931	140.4565	138.1609	139.7677	138.1367	139.8251	138.2259	140.3928	**131.79**
**C2**	139.1128	139.1284	139.6291	138.8983	139.4168	138.9145	139.8285	138.9354	139.7236	138.8813	139.8026	139.1128	139.1283	139.6291	138.8983	139.4170	138.9145	139.8284	138.9354	139.7236	138.8813	139.8026	**131.93**
**C3**	121.1252	121.8940	122.5551	122.1904	122.9167	122.1808	123.2087	122.1767	123.1263	122.1982	123.2704	121.1252	121.8940	122.5550	122.1904	122.9168	122.1808	123.2086	122.1767	123.1263	122.1982	123.2704	**116.53**
**C4**	173.3567	174.1463	174.7634	174.5083	175.1361	174.4859	175.7471	174.4693	175.5926	174.5285	175.8225	173.3567	174.1463	174.7633	174.5083	175.1361	174.4859	175.7472	174.4693	175.5926	174.5285	175.8225	**168.1**
**C5**	119.0903	120.3998	121.0291	121.0612	121.9529	121.0209	122.2293	120.9863	122.1057	121.0991	122.4359	119.0903	120.3999	121.0291	121.0613	121.9529	121.0209	122.2294	120.9863	122.1057	121.0991	122.4359	**116.51**
**C6**	134.9413	136.7598	137.5390	137.7371	139.0919	137.6681	139.7237	137.6048	139.4973	137.8023	139.9696	134.9414	136.7597	137.5390	137.7371	139.0918	137.6681	139.7236	137.6048	139.4973	137.8023	139.9696	**131.88**
**C7**	201.2044	205.7347	207.5233	208.2837	209.6640	208.1101	214.7111	207.9462	213.5857	208.4517	216.1484	201.2044	205.7349	207.5233	208.2837	209.6640	208.1101	214.7112	207.9462	213.5857	208.4517	216.1484	**195.2**
**C8**	63.7059	64.8475	65.6612	65.4996	66.4682	65.4525	67.1640	65.4128	67.0290	65.5454	67.5846	63.7059	64.8475	65.6612	65.4996	66.4683	65.4525	67.1640	65.4127	67.0290	65.5454	67.5846	**54.87**
**C9**	35.0519	34.8561	34.8689	34.7499	34.9155	34.7537	35.0107	34.7633	34.9822	34.7457	35.0671	35.0519	34.8560	34.8689	34.7499	34.9155	34.7537	35.0107	34.7632	34.9822	34.7457	35.0671	**31.13**
**C10**	25.8847	26.4496	26.6898	26.7010	27.3112	26.6851	27.0929	26.6750	27.0915	26.7157	27.2331	25.8847	26.4496	26.6897	26.7010	27.3112	26.6851	27.0929	26.6750	27.0915	26.7157	27.2331	**30.82**
**C11**	35.2073	35.2765	35.5686	35.2493	35.7128	35.2539	35.6855	35.2639	35.6570	35.2442	35.8710	35.2073	35.2764	35.5685	35.2493	35.7129	35.2539	35.6857	35.2639	35.6570	35.2442	35.8710	**29.11**
**C12**	36.7949	37.1126	37.3511	37.2760	37.7612	37.2645	37.6307	37.2589	37.6294	37.2864	37.7181	36.7949	37.1124	37.3511	37.2760	37.7611	37.2645	37.6308	37.2590	37.6294	37.2865	37.7181	**25.52**
**C13**	27.9737	28.1746	28.3538	28.2299	28.5066	28.2275	28.4980	28.2307	28.4832	28.2314	28.5071	27.9737	28.1747	28.3537	28.2299	28.5066	28.2275	28.4980	28.2306	28.4832	28.2314	28.5071	**22.36**
**C14**	16.4221	16.6061	16.6266	16.6870	16.7802	16.6812	16.7589	16.6813	16.7471	16.6919	16.7931	16.4221	16.6061	16.6267	16.6870	16.7802	16.6812	16.7589	16.6813	16.7471	16.6919	16.7931	**13.85**
**C1`**	50.7171	51.4886	51.7262	51.8494	52.4818	51.8187	52.4073	51.7940	52.3348	51.8785	52.9195	54.8001	55.8037	56.1971	56.3298	56.8211	56.2963	57.0596	56.2685	56.9551	56.3608	57.3685	**51.23**
**C2`**	24.6416	25.0540	25.3471	25.2742	25.6576	25.2602	25.7206	25.2521	25.7135	25.2869	25.5187	24.3091	24.5560	24.9080	24.5878	25.2150	24.5875	25.0876	24.5928	25.0866	24.5874	24.8842	**23.85**
**C3`**	24.3091	24.5560	24.9080	24.5878	25.2150	24.5875	25.0875	24.5927	25.0866	24.5874	24.8842	24.6416	25.0540	25.3471	25.2742	25.6575	25.2602	25.7205	25.2521	25.7135	25.2869	25.5187	**23.66**
**C4`**	54.8001	55.8037	56.1971	56.3298	56.8211	56.2963	57.0596	56.2686	56.9551	56.3608	57.3685	50.7171	51.4885	51.7261	51.8494	52.4818	51.8187	52.4074	51.7940	52.3348	51.8786	52.9195	**52.72**
**H2**	8.7680	8.6702	8.6363	8.5996	8.5209	8.6062	8.5492	8.6125	8.5482	8.5931	8.5077	8.7680	8.6702	8.6363	8.5996	8.5209	8.6062	8.5493	8.6125	8.5482	8.5931	8.5077	**8.13**
**H3**	7.3664	7.5014	7.5904	7.5439	7.6558	7.5426	7.6729	7.5412	7.6646	7.5454	7.6882	7.3664	7.5014	7.5903	7.5439	7.6558	7.5426	7.6729	7.5412	7.6646	7.5454	7.6882	**7.22**
**H5**	7.1841	7.3673	7.4476	7.4421	7.5674	7.4379	7.5953	7.4337	7.5820	7.4464	7.6223	7.1841	7.3673	7.4476	7.4422	7.5674	7.4379	7.5953	7.4337	7.5820	7.4464	7.6223	**7.22**
**H6**	8.0386	8.2194	8.3083	8.3309	8.4422	8.3249	8.5163	8.3190	8.4926	8.3368	8.5162	8.0386	8.2193	8.3083	8.3309	8.4422	8.3249	8.5163	8.3190	8.4926	8.3368	8.5162	**8.13**
**H8**	4.5456	4.6113	4.6567	4.6527	4.7377	4.6506	4.7936	4.6485	4.7718	4.6551	4.7832	4.5456	4.6113	4.6567	4.6527	4.7376	4.6506	4.7936	4.6485	4.7718	4.6551	4.7832	**5.5**
**H(9A-9B)**	1.6871	1.7331	1.6874	1.7651	1.7289	1.7626	1.7220	1.7601	1.7228	1.7678	1.7375	1.6871	1.7330	1.6874	1.7651	1.7289	1.7626	1.7220	1.7601	1.7228	1.7678	1.7375	**2.08**
**H(10-13A-10-13B)**	1.1523	1.1213	1.0840	1.1026	1.0562	1.1041	1.0613	1.1055	1.0601	1.1015	1.0585	1.1523	1.1213	1.0840	1.1026	1.0562	1.1041	1.0613	1.1055	1.0601	1.1015	1.0585	**1.25**
**H(14A-14C)**	0.9138	0.9081	0.8865	0.9022	0.8756	0.9029	0.8784	0.9036	0.8768	0.9018	0.8791	0.9138	0.9081	0.8865	0.9022	0.8756	0.9029	0.8784	0.9036	0.8768	0.9018	0.8791	**0.79**
**H(1`A-1`B)**	2.9172	2.9331	2.9385	2.9418	2.9486	2.9412	2.9551	2.9407	2.9529	2.9426	2.9418	2.8413	2.7600	2.7351	2.7250	2.7185	2.7269	2.6940	2.7290	2.6992	2.7236	2.6830	**3.69**
**H(2`-3`A-2`-3`B)**	1.6368	1.6921	1.6615	1.7232	1.6875	1.7212	1.7087	1.7194	1.7000	1.7253	1.7422	1.6368	1.6921	1.6615	1.7232	1.6875	1.7212	1.7087	1.7194	1.7000	1.7253	1.7422	**2.18**
**H(4`A)**	2.8839	2.7282	2.6851	2.6684	2.6857	2.6713	2.6265	2.6745	2.6297	2.6662	2.6354	3.1547	3.1912	3.2218	3.2249	3.2847	3.2223	3.2824	3.2198	3.2770	3.2277	3.2862	**3.12**
**H(4`B)**	2.7987	2.7917	2.7851	2.7815	2.7513	2.7825	2.7616	2.7835	2.7688	2.7809	2.7306	2.6796	2.6749	2.6552	2.6586	2.6125	2.6601	2.6279	2.6616	2.6288	2.6574	2.5974	**3.86**

[a] methyl protons shifts are averaged
[b] according to [12]

Table S13. Cont. IX

Nuclei	R											S											Exp. [b]
	O3LYP																						
	Gas	$CHCl_3$ (PCM)	$CHCl_3$ (SMD)	DMSO (PCM)	DMSO (SMD)	CH_3OH (PCM)	CH_3OH (SMD)	C_2H_5OH (PCM)	C_2H_5OH (SMD)	H_2O (PCM)	H_2O (SMD)	Gas	$CHCl_3$ (PCM)	$CHCl_3$ (SMD)	DMSO (PCM)	DMSO (SMD)	CH_3OH (PCM)	CH_3OH (SMD)	C_2H_5OH (PCM)	C_2H_5OH (SMD)	H_2O (PCM)	H_2O (SMD)	
**C1**	134.8069	136.8725	138.2604	137.4523	139.9273	137.4307	138.8371	137.4056	139.0108	137.4694	138.9821	134.8069	136.8725	138.2604	137.4523	139.9273	137.4307	138.8371	137.4056	139.0108	137.4694	138.9821	**131.79**
**C2**	134.3566	134.0195	134.5084	133.9055	134.5083	133.9255	134.8682	133.9400	134.7489	133.8797	135.0358	134.3566	134.0195	134.5086	133.9055	134.5083	133.9255	134.8683	133.9400	134.7489	133.8797	135.0358	**131.93**
**C3**	117.2995	117.9515	118.6167	118.2515	118.8991	118.2454	119.1305	118.2337	119.0837	118.2505	119.1191	117.2995	117.9515	118.6167	118.2514	118.8991	118.2454	119.1306	118.2337	119.0837	118.2505	119.1191	**116.53**
**C4**	169.4121	170.0003	170.6616	170.4585	171.0177	170.4384	171.6087	170.4134	171.4691	170.4715	171.6948	169.4121	170.0003	170.6619	170.4584	171.0177	170.4383	171.6086	170.4134	171.4690	170.4715	171.6948	**168.1**
**C5**	115.1868	116.4433	117.0470	117.0789	117.7639	117.0504	118.0270	117.0164	117.9192	117.0989	118.1826	115.1868	116.4433	117.0469	117.0790	117.7639	117.0503	118.0270	117.0164	117.9192	117.0989	118.1826	**116.51**
**C6**	129.8788	131.2176	131.9993	132.2893	133.3694	132.2264	134.0509	132.1592	133.8051	132.3425	134.4585	129.8788	131.2176	131.9992	132.2893	133.3694	132.2264	134.0508	132.1592	133.8051	132.3425	134.4585	**131.88**
**C7**	196.9625	202.8323	204.6364	205.5323	207.0811	205.3807	211.4363	205.2250	210.4879	205.6733	212.2084	196.9625	202.8323	204.6362	205.5324	207.0811	205.3804	211.4360	205.2250	210.4879	205.6733	212.2084	**195.2**
**C8**	64.5932	66.4169	67.2970	67.1314	68.4311	67.1078	68.9181	67.0788	68.7775	67.1476	68.9926	64.5932	66.4169	67.2970	67.1314	68.4311	67.1077	68.9181	67.0788	68.7774	67.1476	68.9926	**54.87**
[truncated: 948,818 more chars]
